# Supplementary material for: A library of ab initio Raman spectra for automated identification of 2D materials
Source: Nat Commun. 2020 Jun 15;11:3011. doi: 10.1038/s41467-020-16529-6 (PMC7296020; doi:10.1038/s41467-020-16529-6)
Supplement: Supplementary file 1 — Supplementary Information [file 41467_2020_16529_MOESM1_ESM.pdf]

Supplementary Information for "A library of *ab initio* Raman spectra for automated identification of 2D materials"

Alireza Taghizadeh[†1,2,3], Ulrik Leffers[3], Thomas G. Pedersen[1,2], and Kristian S. Thygesen[3,4]

[1]Department of Materials and Production, Aalborg University, 9220 Aalborg Øst, Denmark
[2]Center for Nanostructured Graphene (CNG), 9220 Aalborg Øst, Denmark
[3]Computational Atomic-scale Materials Design (CAMD), Department of Physics, Technical University of Denmark (DTU), 2800 Kgs. Lyngby, Denmark
[4]Center for Nanostructured Graphene (CNG), Technical University of Denmark (DTU), 2800 Kgs. Lyngby, Denmark

May 5, 2020

This document provides supplementary information to "A library of *ab initio* Raman spectra for automated identification of 2D materials", in which we provide calculated Raman spectra of 733 monolayers selected as the most stable materials from the computational 2D materials database (C2DB). The spectra are calculated for nine possible configurations of input/output polarization vectors and three excitation frequencies at room temperature. Different 2D materials are uniquely labeled by their chemical formula, space groups and Wyckoff positions. Furthermore, an extended scatter plot of $\langle\omega\rangle$ and $\delta\omega/\langle\omega\rangle$ comparing results for the 733 monolayers to all experimental Raman spectra of Fig. 6 is included.

[†]ata@nano.aau.dk

Supplementary Note 1: Raman tensors for point groups D_{3h} and C_{3v}

For the point groups D_{3h} and C_{3v}, the Raman mode tensors are given by [1]:

$$R_{D_{3h}}^{\mathrm{A}_1'} : \begin{bmatrix} a & 0 & 0 \\ 0 & a & 0 \\ 0 & 0 & b \end{bmatrix}, \quad R_{D_{3h}}^{\mathrm{E}''} : \left\{ \begin{bmatrix} 0 & 0 & -c \\ 0 & 0 & 0 \\ -c & 0 & 0 \end{bmatrix}, \begin{bmatrix} 0 & 0 & 0 \\ 0 & 0 & c \\ 0 & c & 0 \end{bmatrix} \right\}, \quad R_{D_{3h}}^{\mathrm{E}'} : \left\{ \begin{bmatrix} d & 0 & 0 \\ 0 & -d & 0 \\ 0 & 0 & 0 \end{bmatrix}, \begin{bmatrix} 0 & d & 0 \\ d & 0 & 0 \\ 0 & 0 & 0 \end{bmatrix} \right\}, \tag{1}$$

$$R_{C_{3v}}^{\mathrm{A}_1} : \begin{bmatrix} a & 0 & 0 \\ 0 & a & 0 \\ 0 & 0 & b \end{bmatrix}, \quad R_{C_{3v}}^{\mathrm{E}} : \left\{ \begin{bmatrix} c & 0 & 0 \\ 0 & -c & d \\ 0 & d & 0 \end{bmatrix}, \begin{bmatrix} 0 & -c & -d \\ -c & 0 & 0 \\ -d & 0 & 0 \end{bmatrix} \right\}, \tag{2}$$

where a, b, c, d are the mode amplitudes. These equations clearly demonstrate that the E'' modes of MoS_2 or $MoSe_2$ are not active for diagonal polarization configurations and, hence, are not observed in the spectra of Fig. 4.

Supplementary Note 2: Raman fingerprints

Supplementary Figure 1 shows a scatter plot of the first Raman moment and normalized standard deviation for 733 calculated spectra at excitation wavelength of 532 nm and *xx*-polarization setup in comparison with the corresponding experimental results shown in Fig. 6 of the main text. Most calculated Raman fingerprints are close to experimental data, however, with significant disagreement observed for Mo_2Te_4 and Pd_2Se_4.

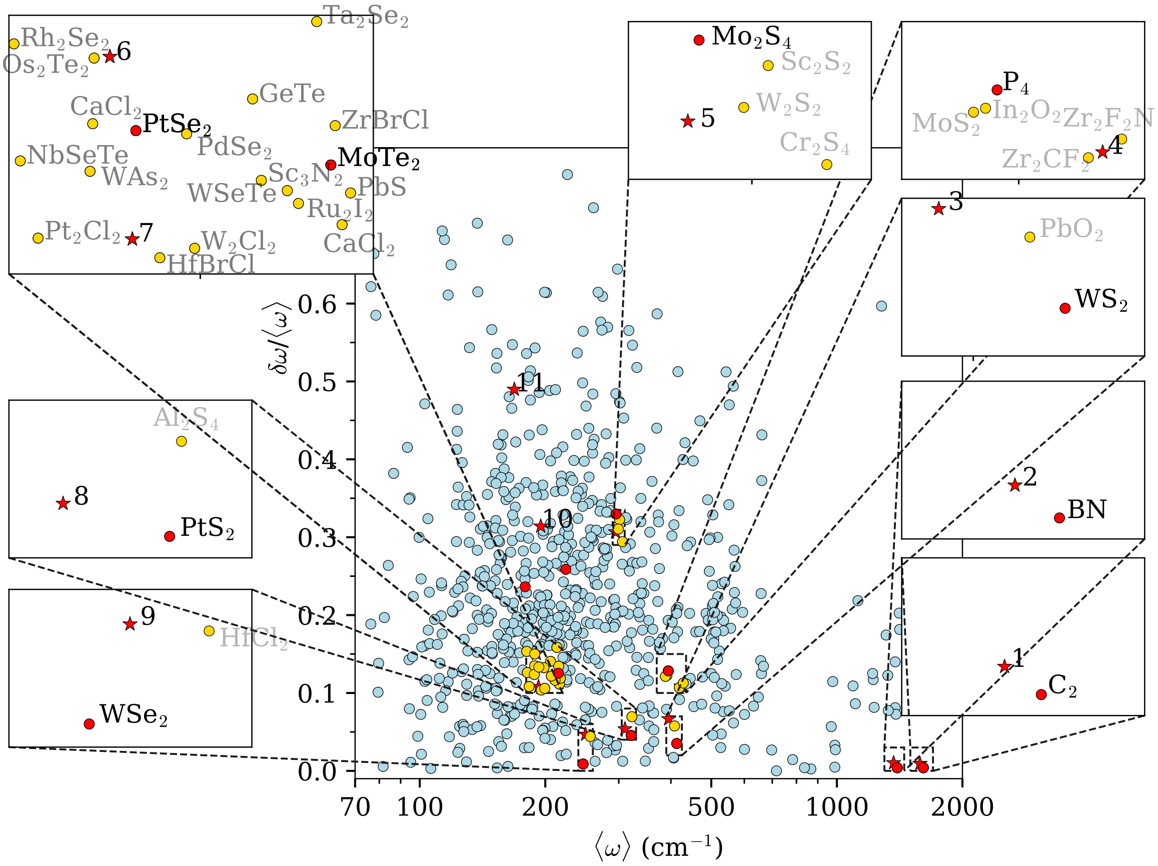

Supplementary Figure 1: Calculated Raman moments. First Raman moment and normalized standard deviation for 733 calculated spectra at excitation wavelength of 532 nm and *xx* polarization setup (circles). For comparison, experimental spectra for all materials in Fig. 6, i.e. Graphene (C_2), BN, WSe_2, WS_2, $MoTe_2$, PtS_2, $PtSe_2$ (all in H-phase), Phosphorene (P_4), Mo_2S_4 (T-phase), Mo_2Te_4 (T′-phase), and Pd_2Se_4, are also shown (red stars). The experimental data are extracted from Refs. [2], [3], [4], [5], [6], [7], [8], [9], [10], [11], and [12] for (1) to (11), respectively. We highlight corresponding theoretical points as red circles. The insets are zooms of the vicinity of the experimental data showing a good match with calculated Raman fingerprints in most cases. Exceptions are Mo_2Te_4 (star 10 and left-most additional red circle) and Pd_2Se_4 (star 11 and right-most red circle), for which significant discrepancy is observed (no zooms shown).

Supplementary Figures

Ag_2Br_2, 2, i-0

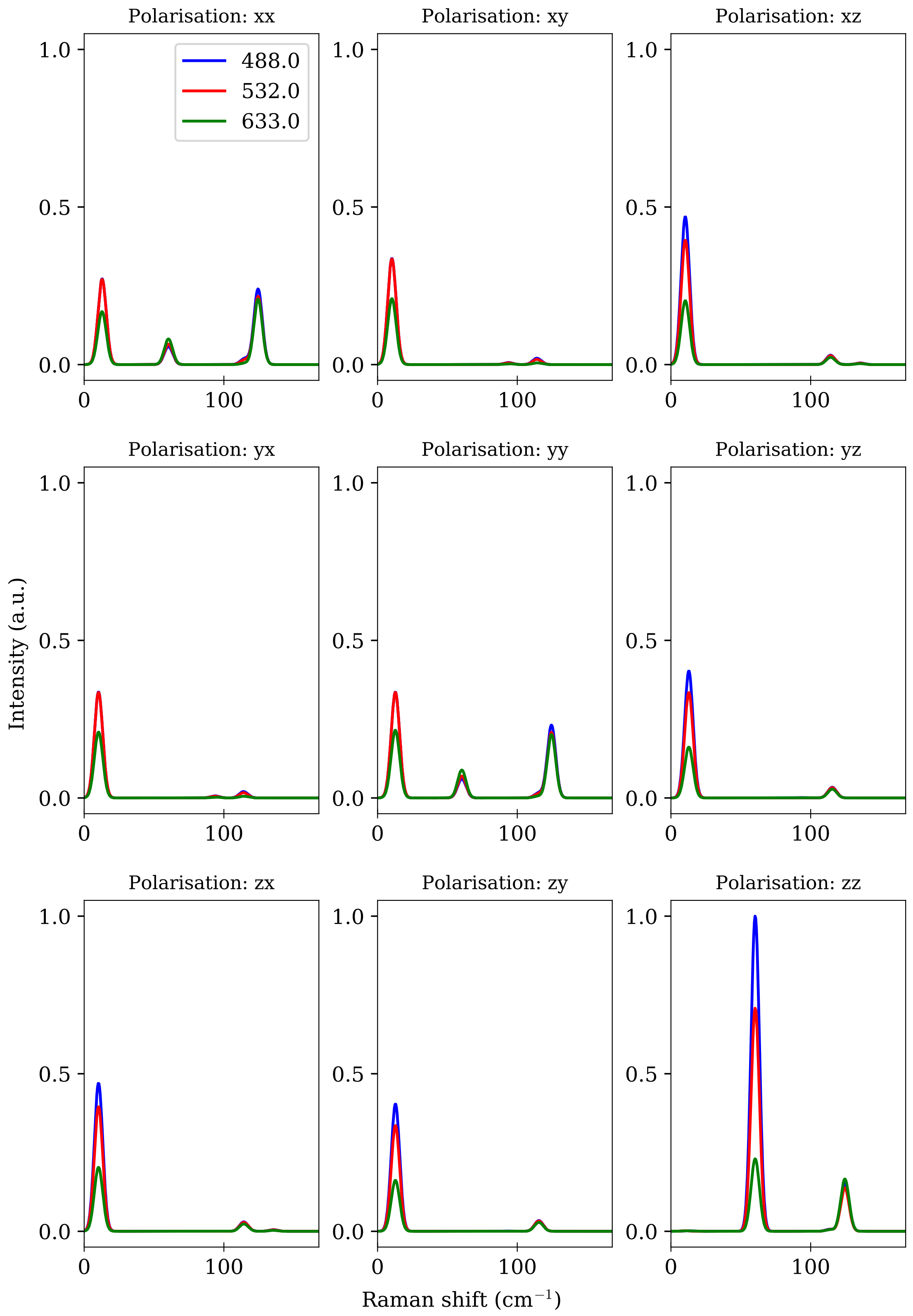

Supplementary Figure 2: Raman spectrum of Ag_2Br_2 with space group 2 and Wyckoff positions i-0.

Ag_2Cl_2, 1, a-0

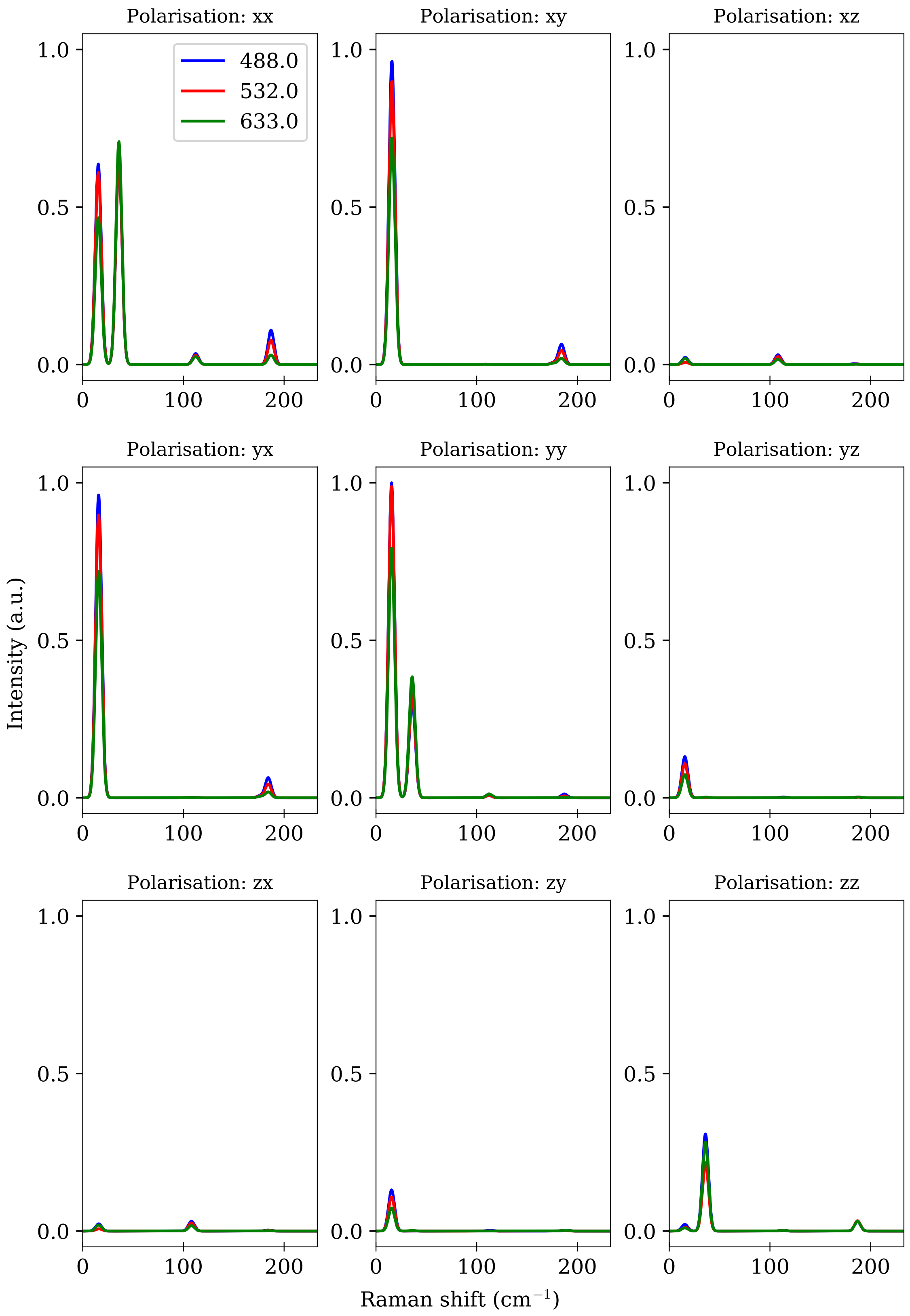

Supplementary Figure 3: Raman spectrum of Ag_2Cl_2 with space group 1 and Wyckoff positions a-0.

Ag_2I_2, 2, i-0

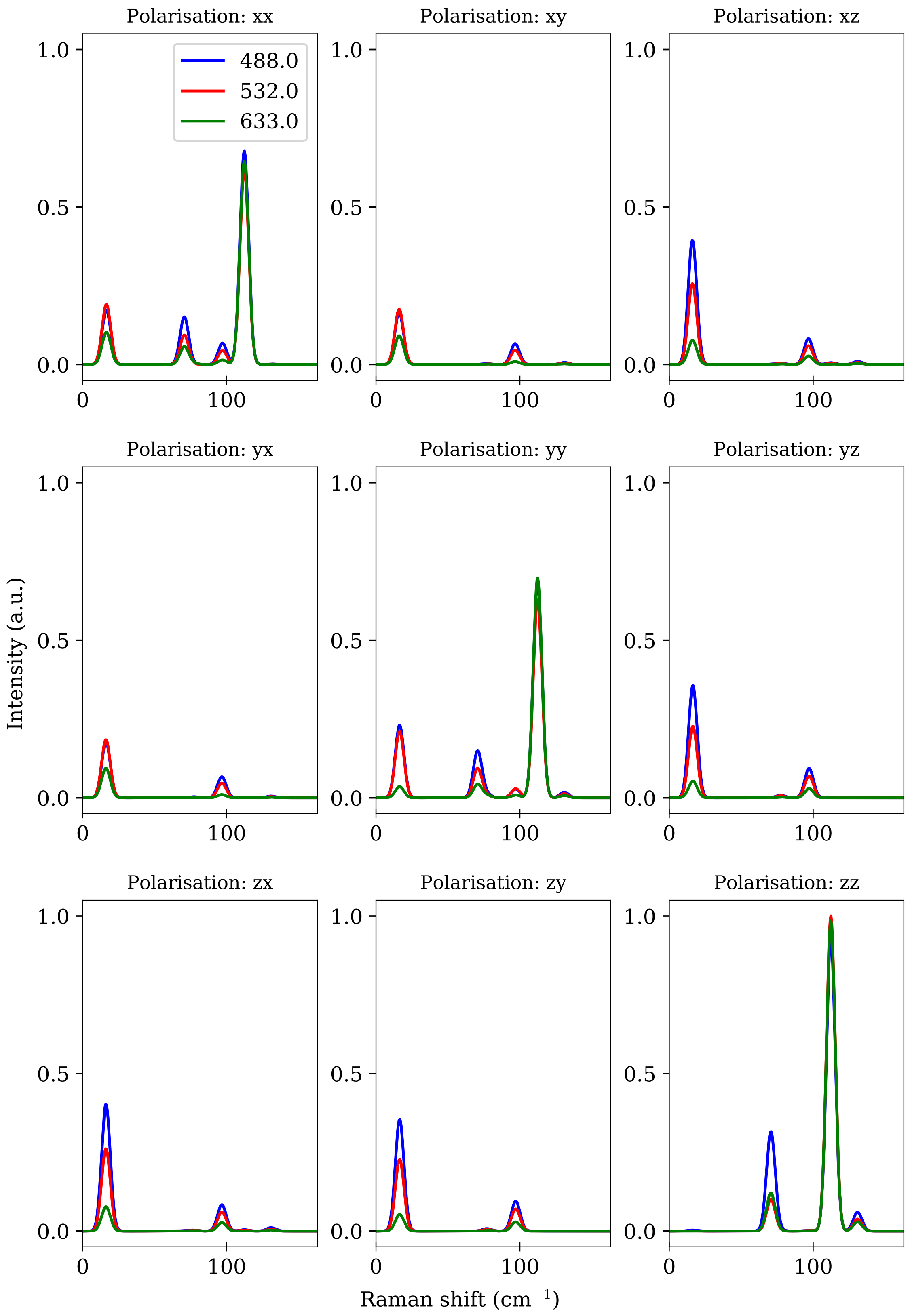

Supplementary Figure 4: Raman spectrum of Ag_2I_2 with space group 2 and Wyckoff positions i-0.

Ag_2O_2, 10, f-g-m-0

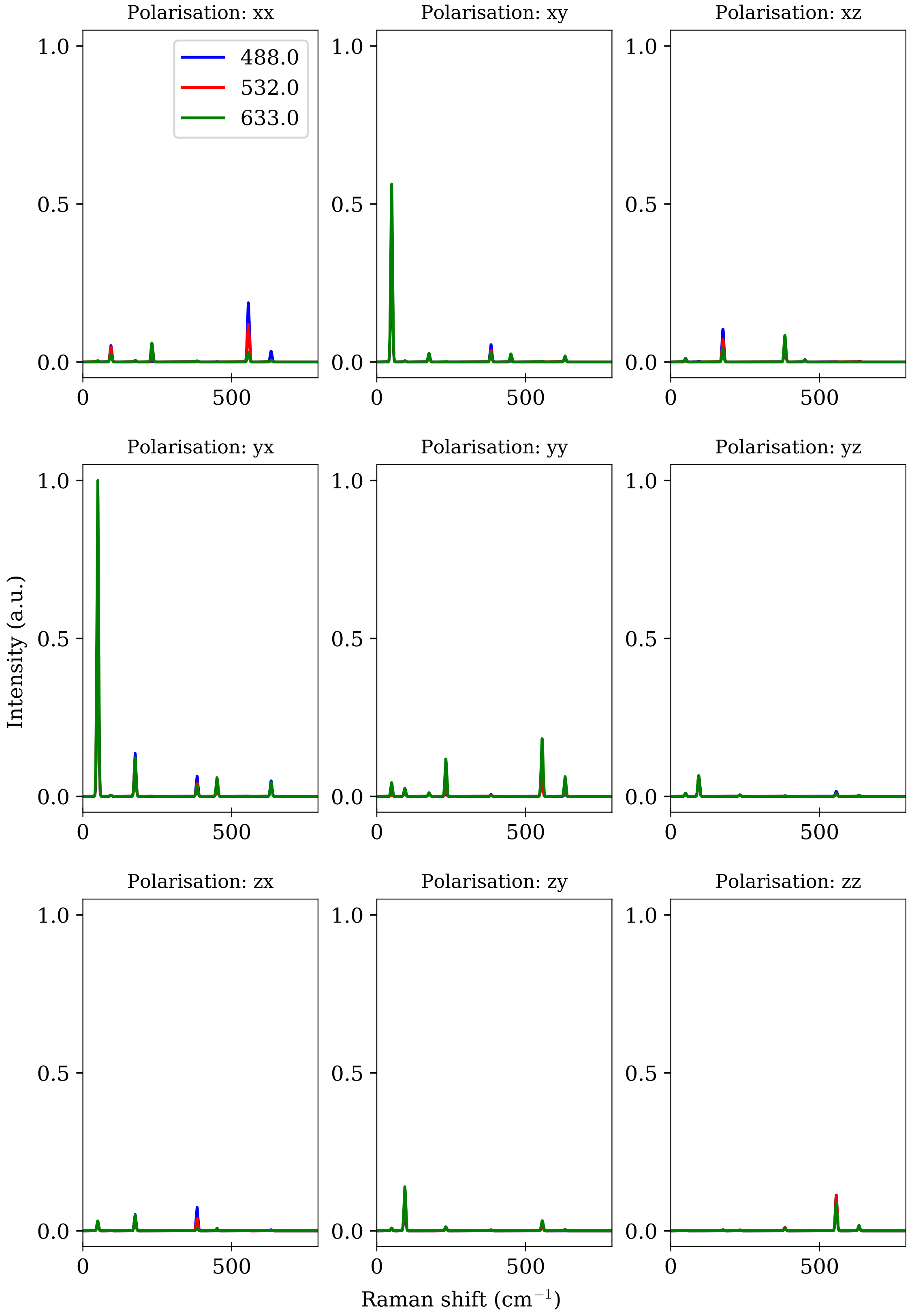

Supplementary Figure 5: Raman spectrum of Ag_2O_2 with space group 10 and Wyckoff positions f-g-m-0.

Ag_2S_2, 10, b-d-m-0

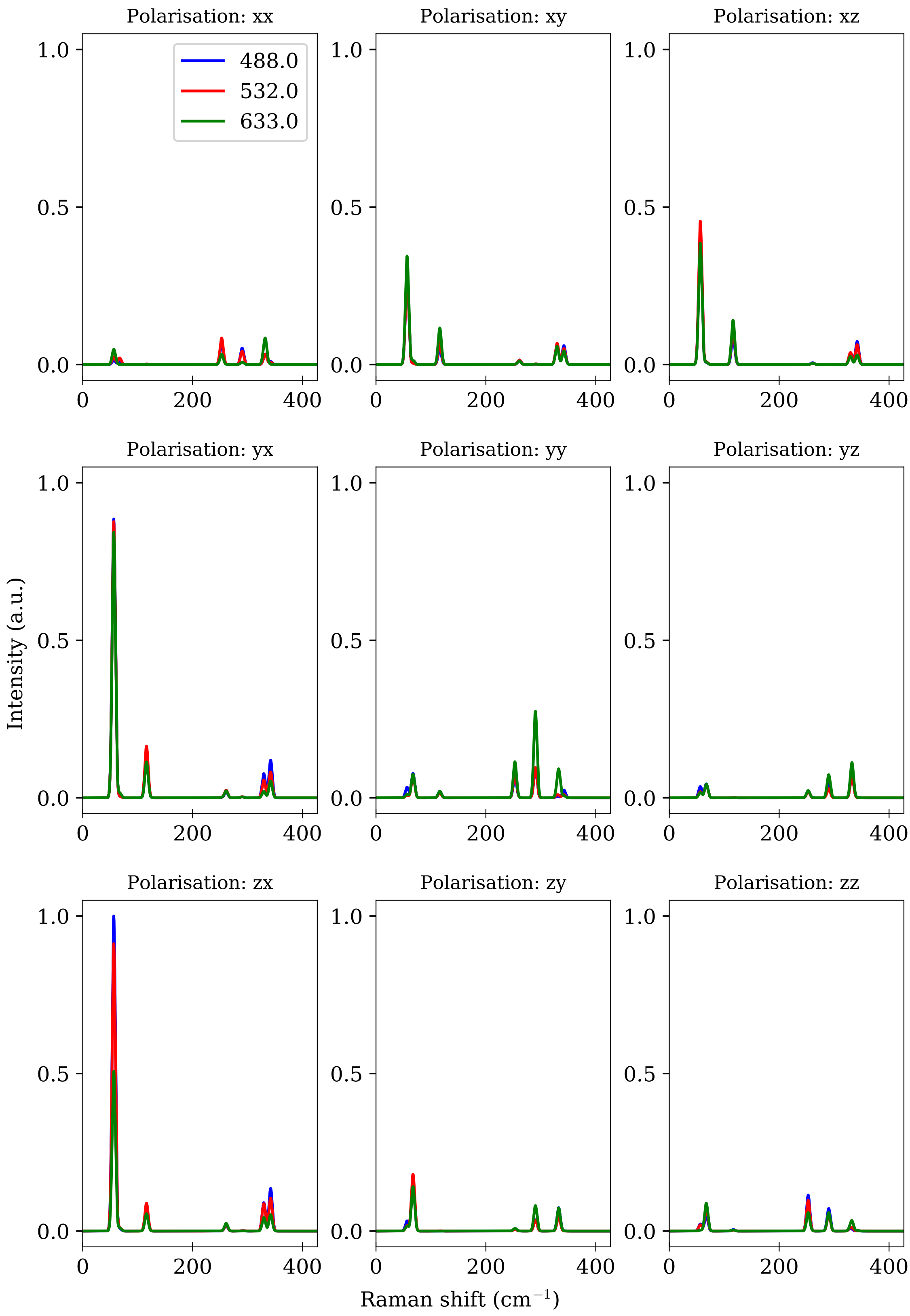

Supplementary Figure 6: Raman spectrum of Ag_2S_2 with space group 10 and Wyckoff positions b-d-m-0.

Ag_2S_2, 2, i-0

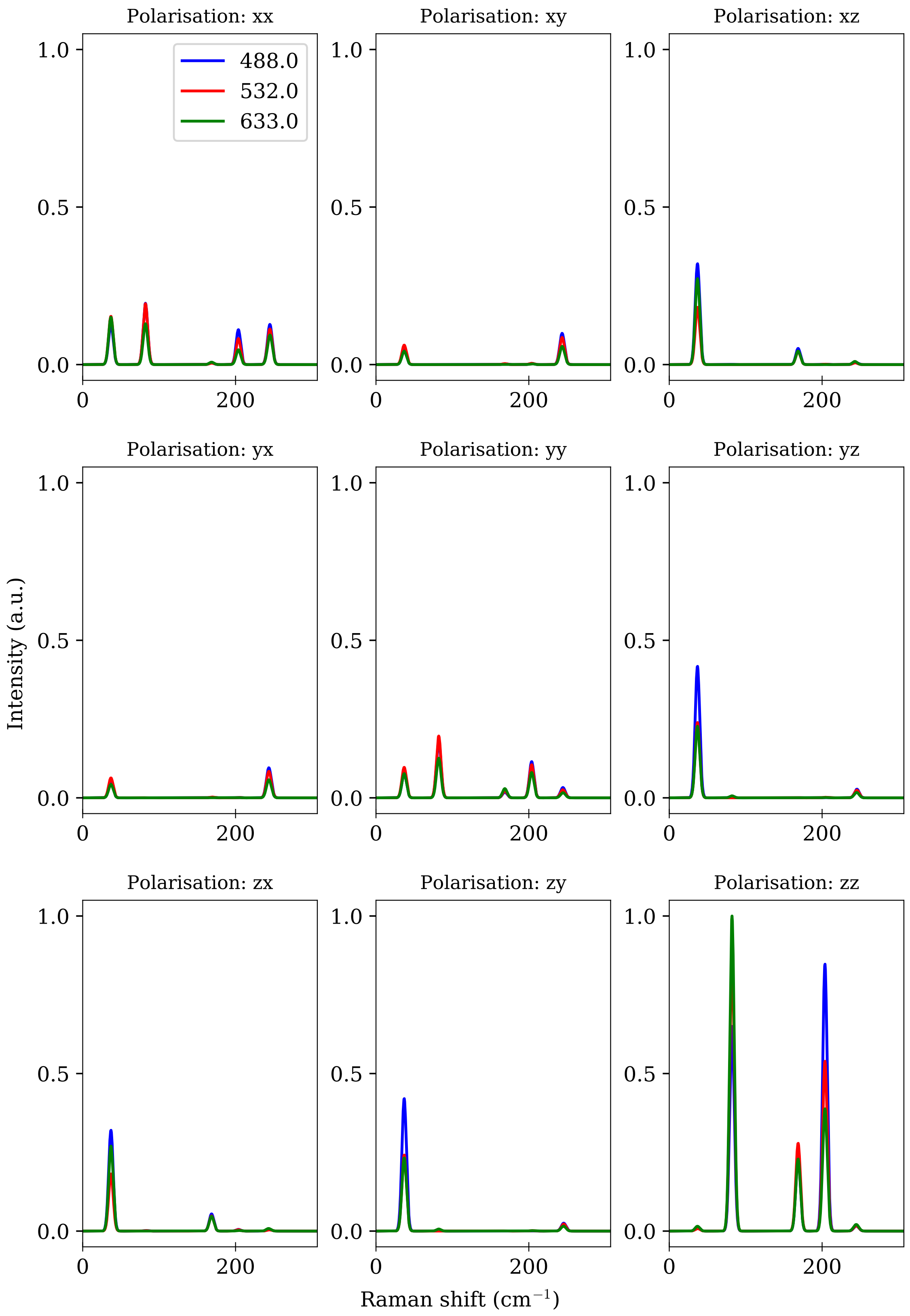

Supplementary Figure 7: Raman spectrum of Ag_2S_2 with space group 2 and Wyckoff positions i-0.

Ag_2Se_2, 1, a-0

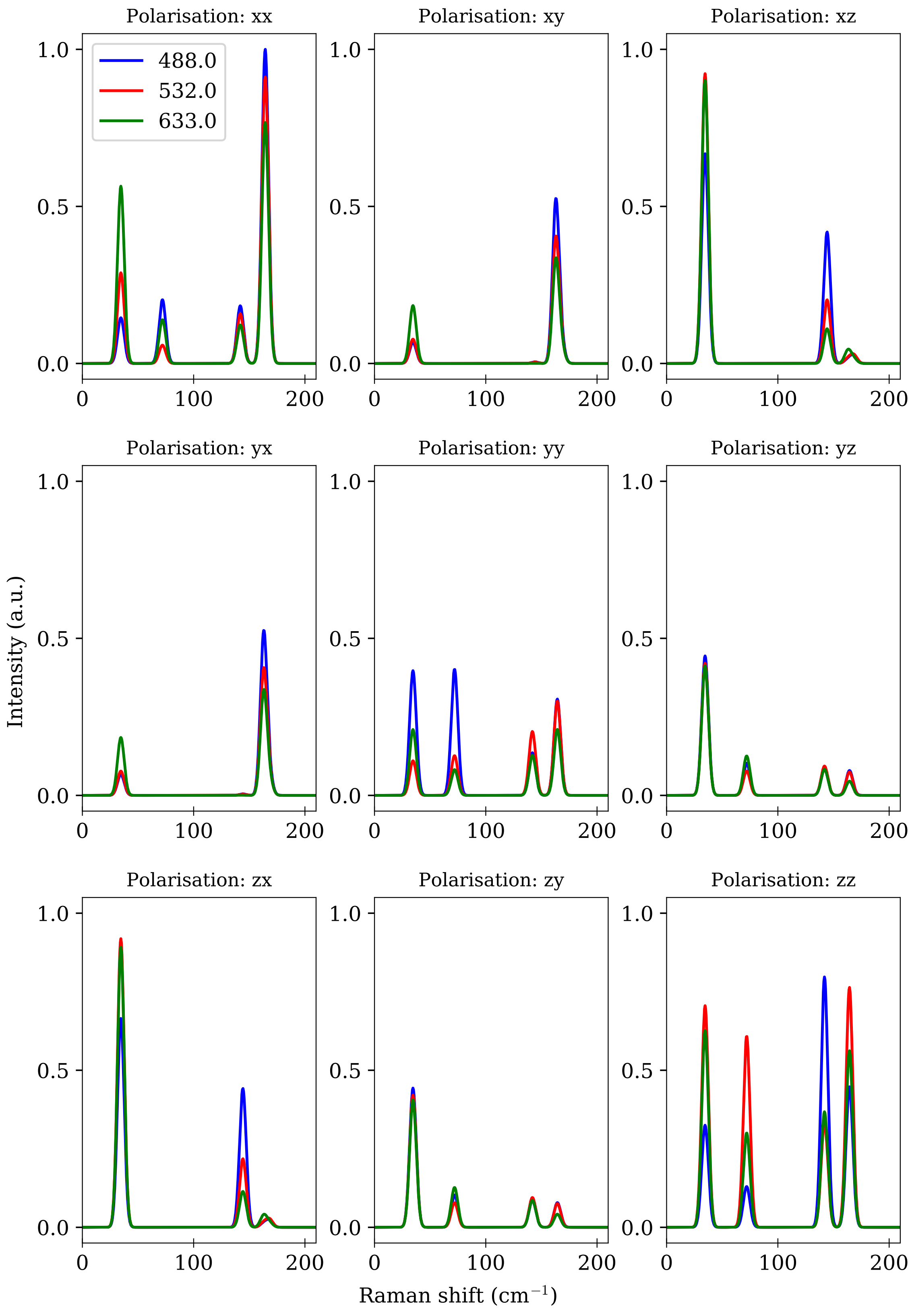

Supplementary Figure 8: Raman spectrum of Ag_2Se_2 with space group 1 and Wyckoff positions a-0.

Ag_2Se_2, 6, a-b-0

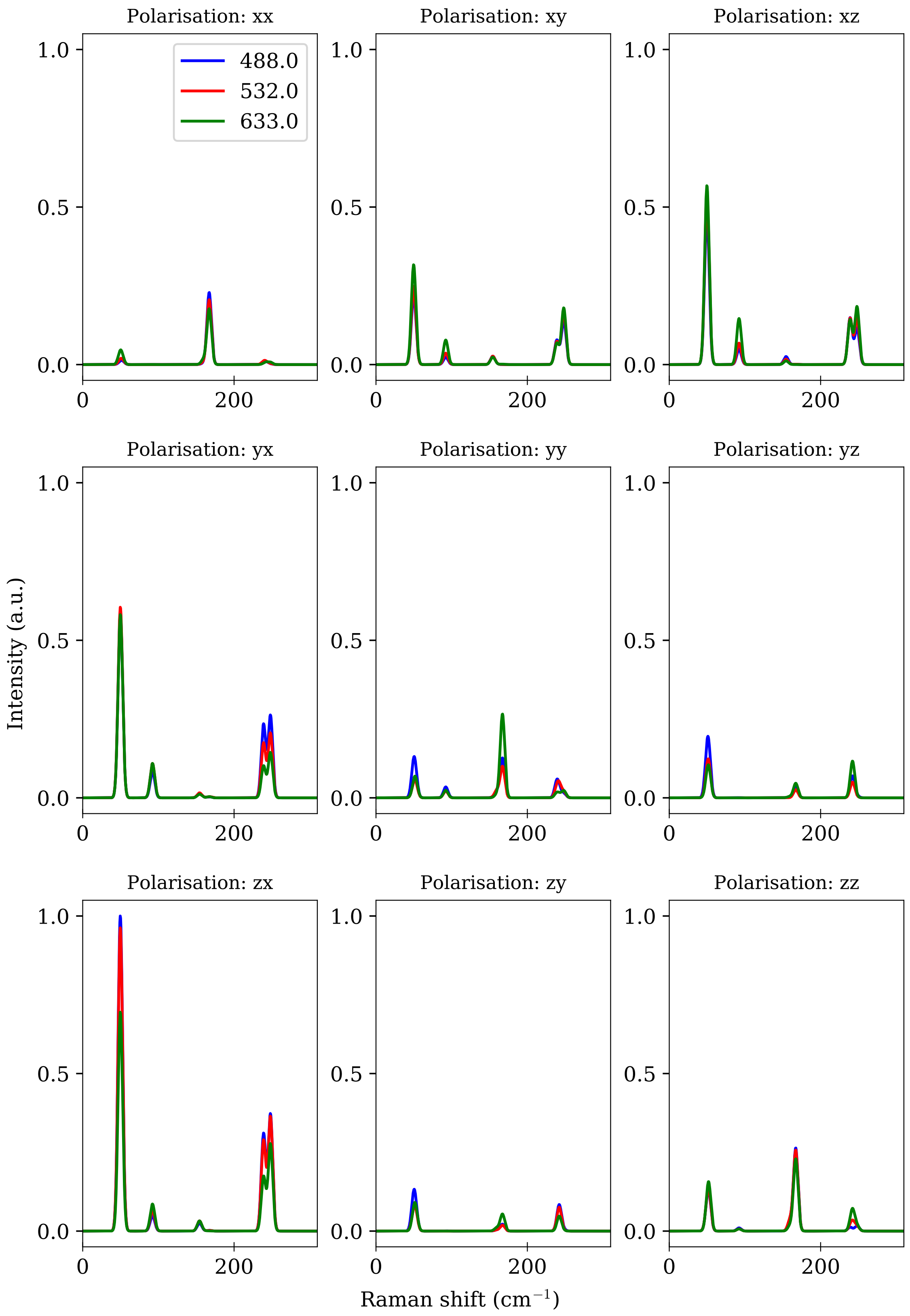

Supplementary Figure 9: Raman spectrum of Ag_2Se_2 with space group 6 and Wyckoff positions a-b-0.

Ag_2Te_2, 2, i-0

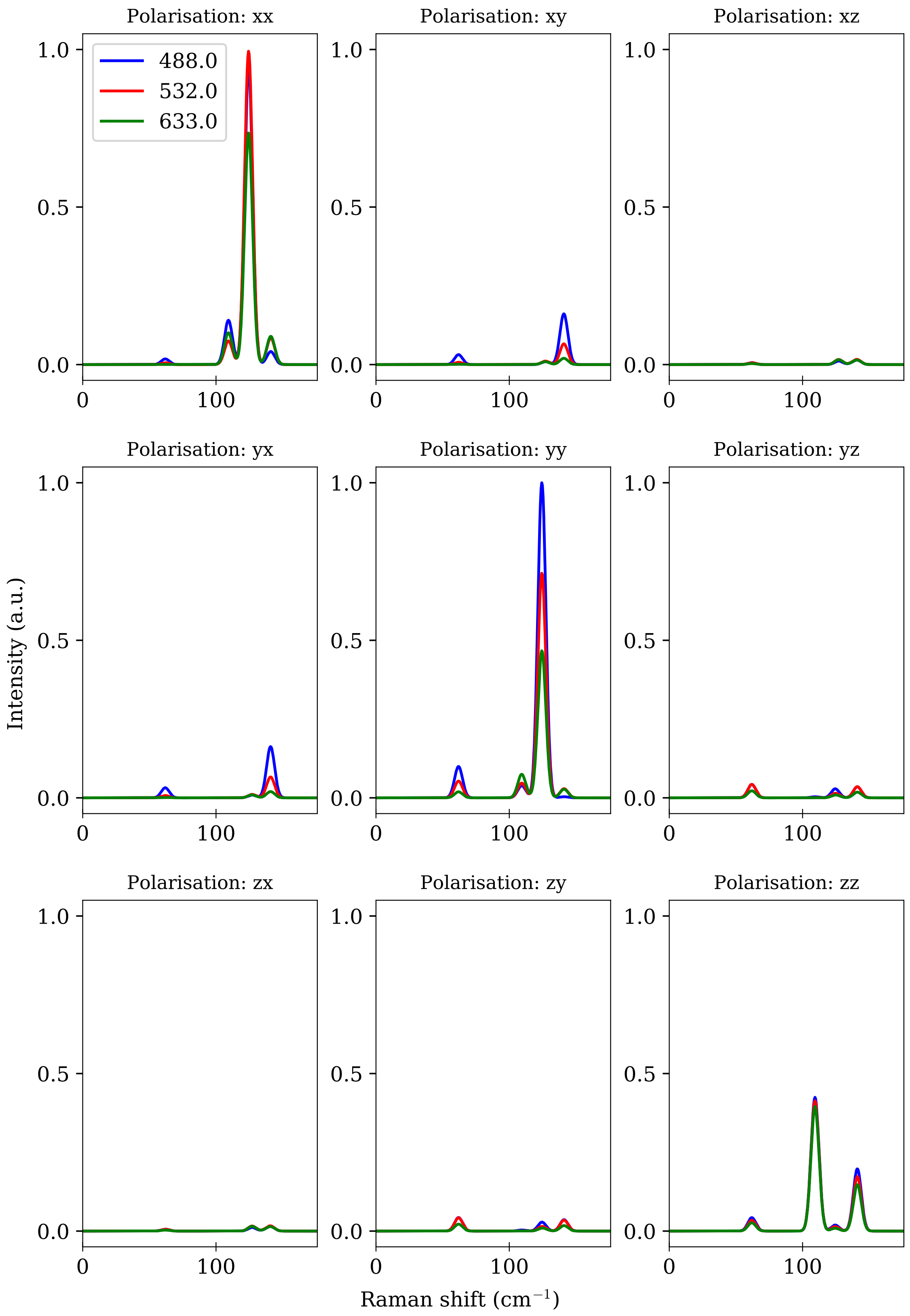

Supplementary Figure 10: Raman spectrum of Ag_2Te_2 with space group 2 and Wyckoff positions i-0.

Ag_2Te_2, 6, a-b-0

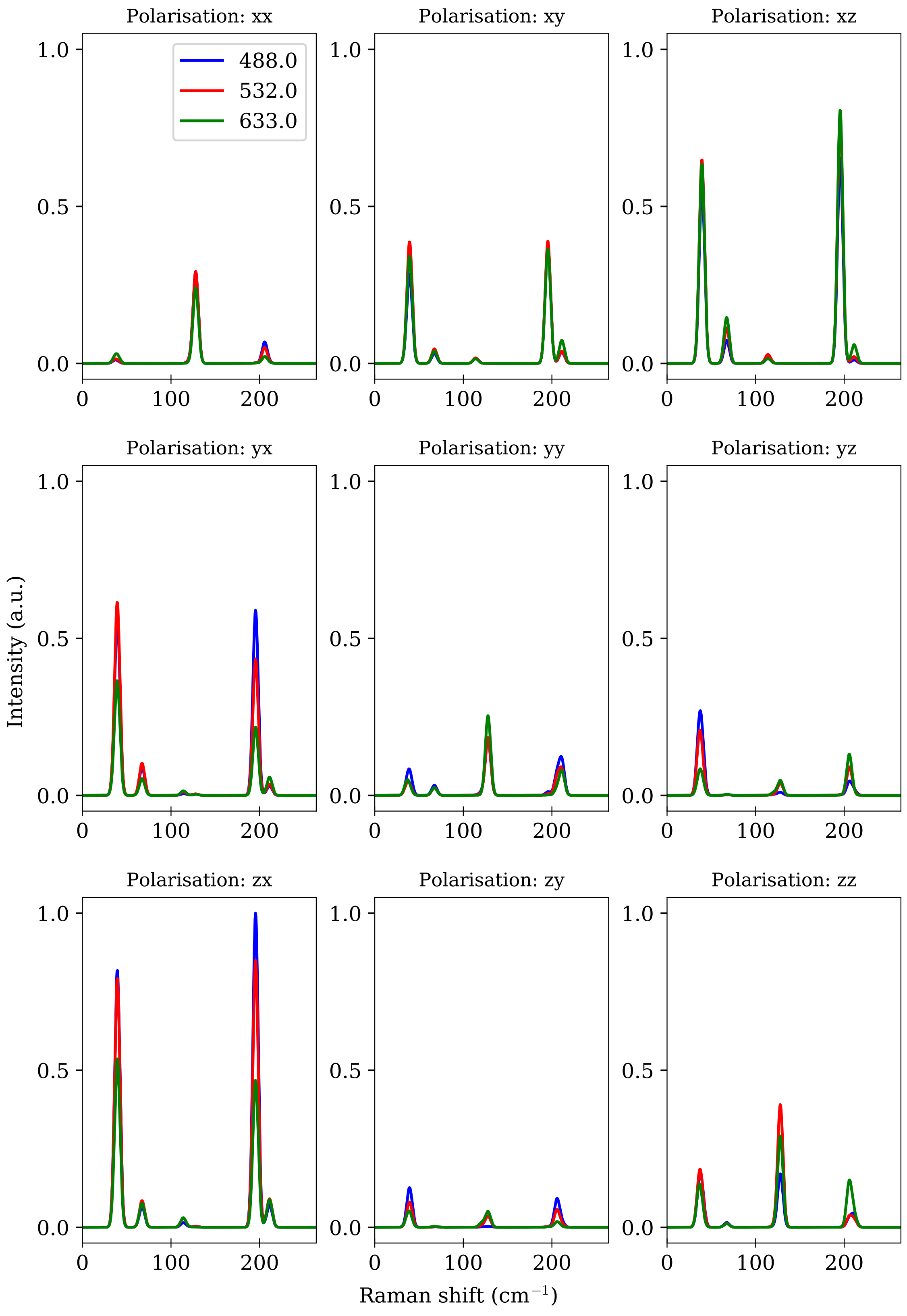

Supplementary Figure 11: Raman spectrum of Ag_2Te_2 with space group 6 and Wyckoff positions a-b-0.

$AgLi_2$, 187, a-i-0

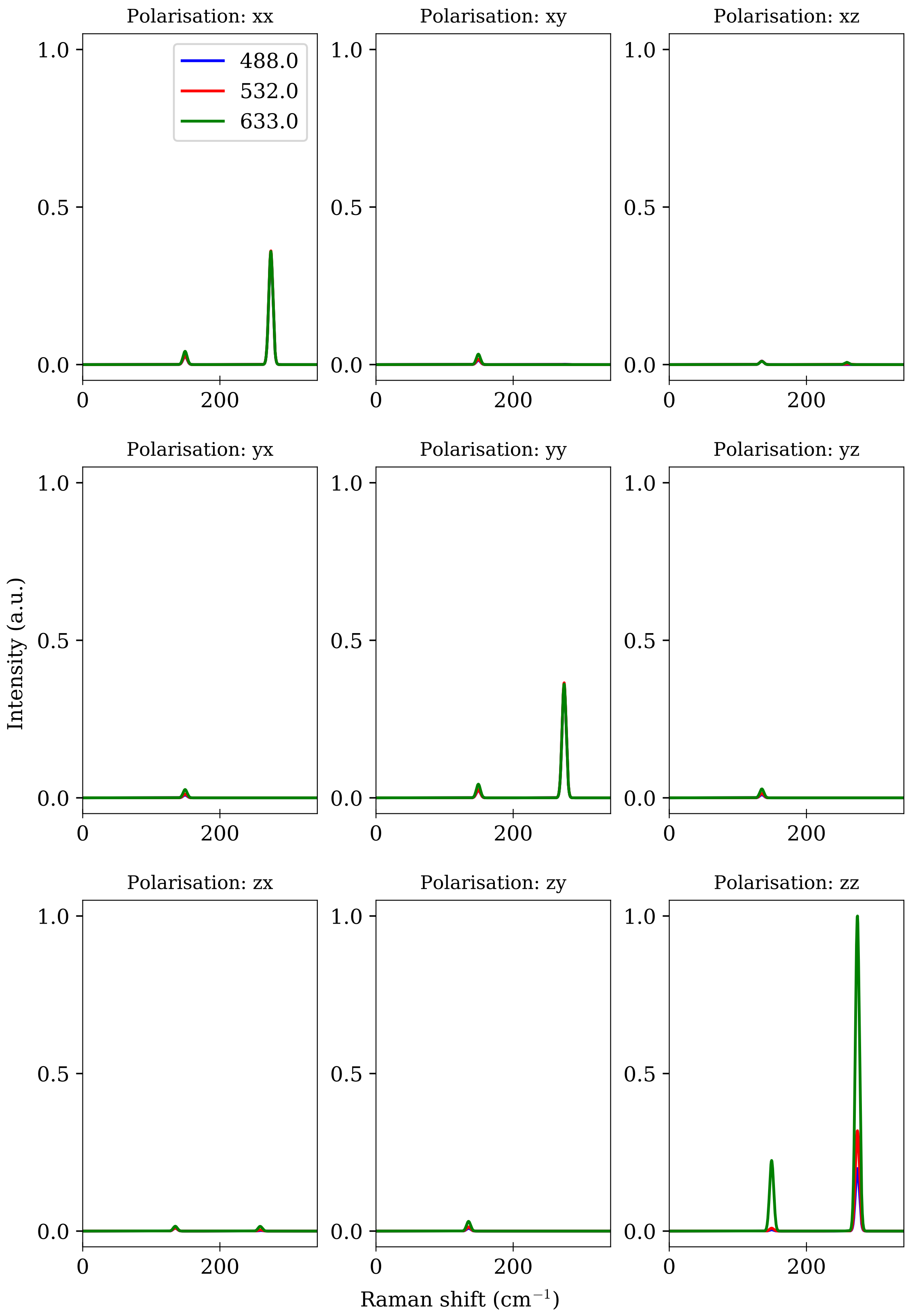

Supplementary Figure 12: Raman spectrum of $AgLi_2$ with space group 187 and Wyckoff positions a-i-0.

$AgSe_2$, 115, d-g-0

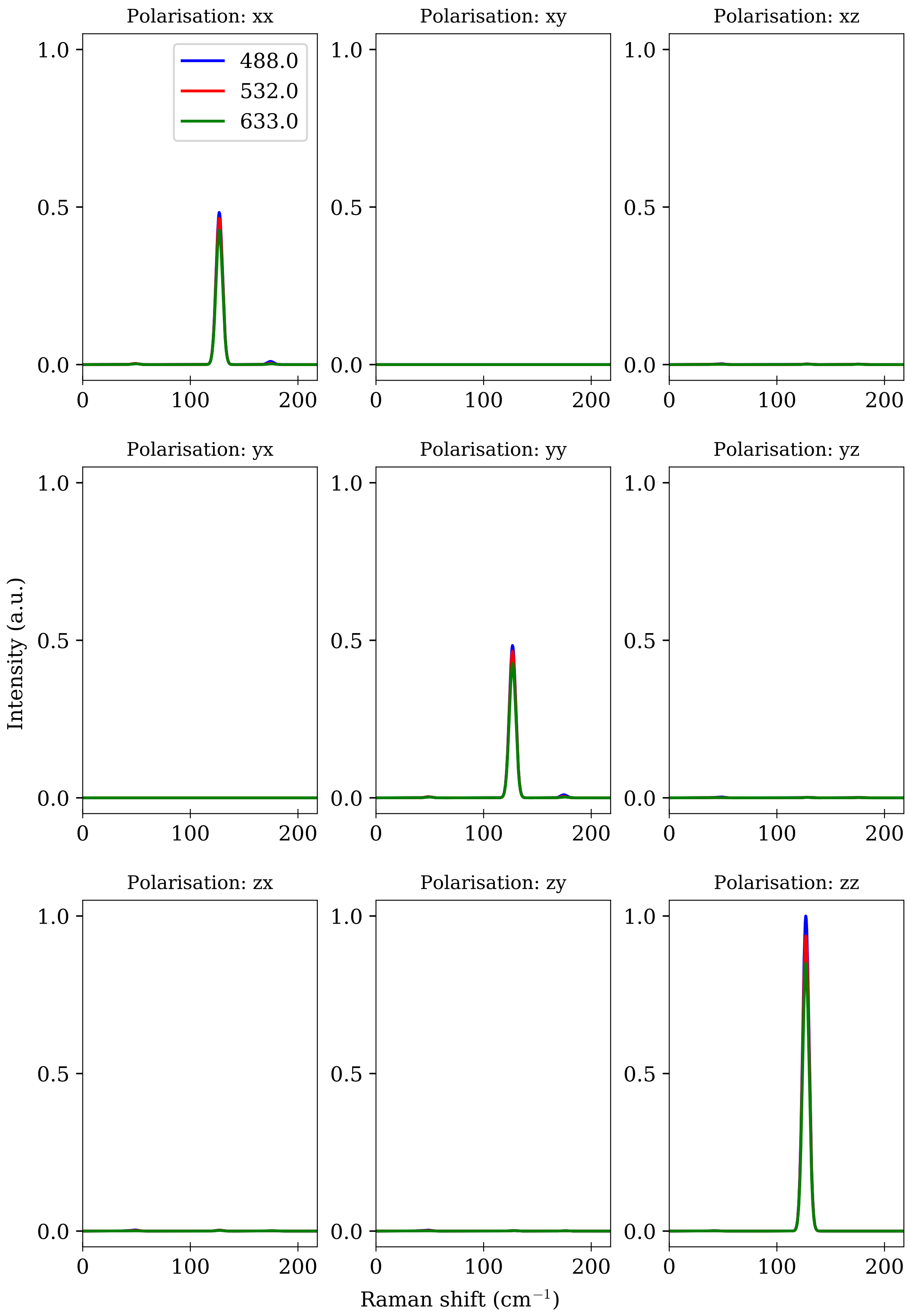

Supplementary Figure 13: Raman spectrum of $AgSe_2$ with space group 115 and Wyckoff positions d-g-0.

$AgTe_2$, 115, a-g-0

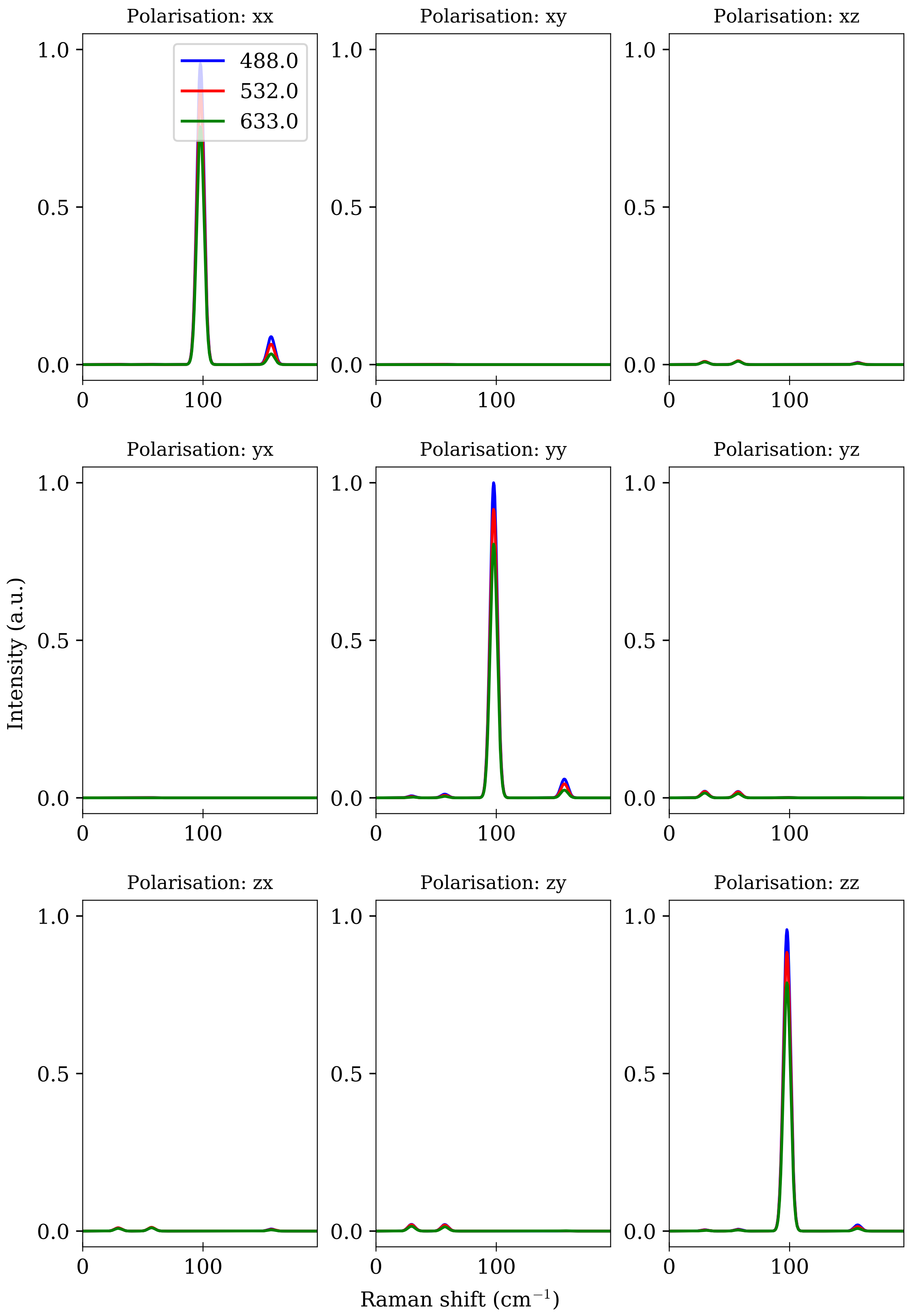

Supplementary Figure 14: Raman spectrum of $AgTe_2$ with space group 115 and Wyckoff positions a-g-0.

$Al_2Br_2O_2$, 59, a-b-0

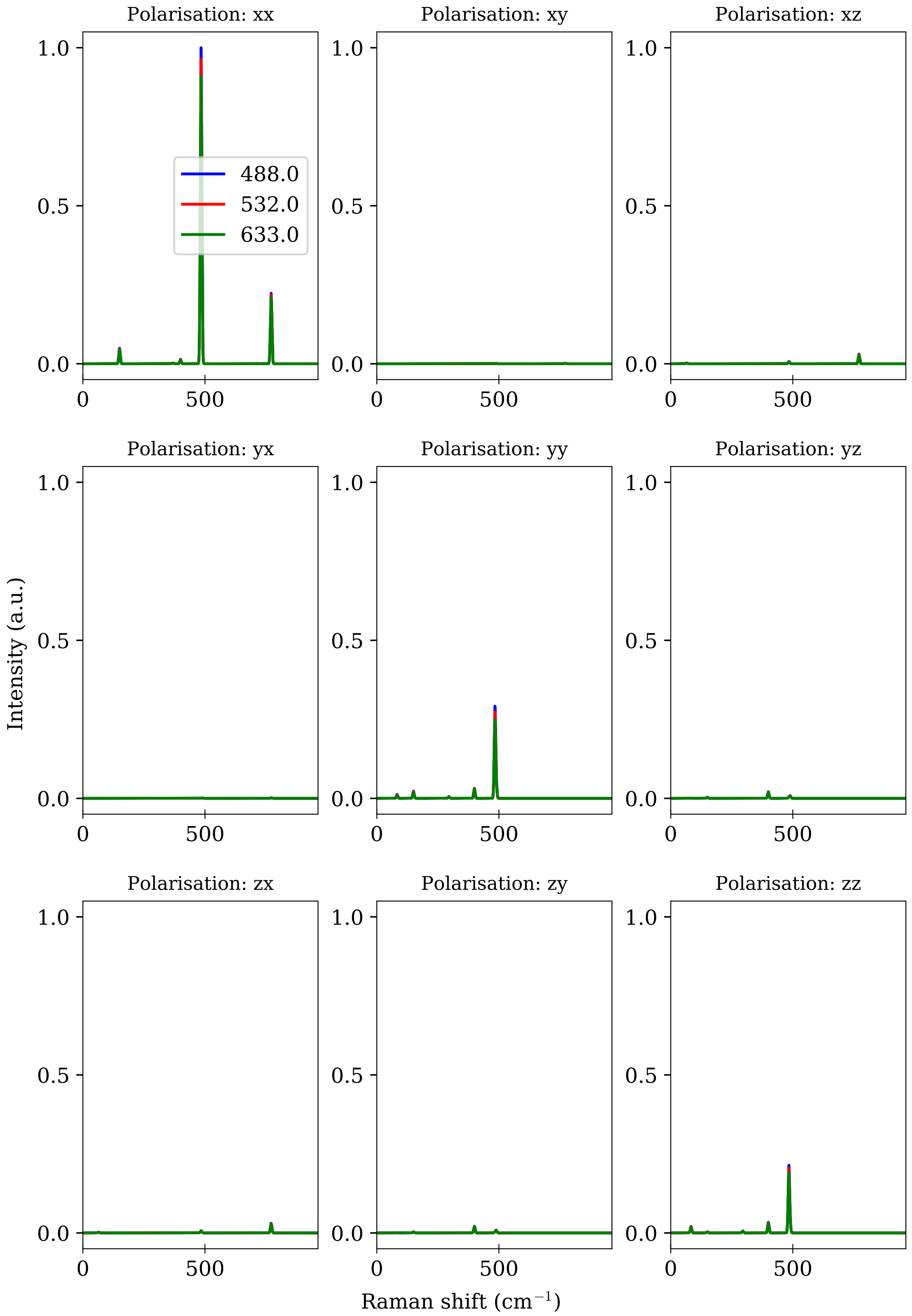

Supplementary Figure 15: Raman spectrum of $Al_2Br_2O_2$ with space group 59 and Wyckoff positions a-b-0.

$Al_2Br_2S_2$, 59, a-b-0

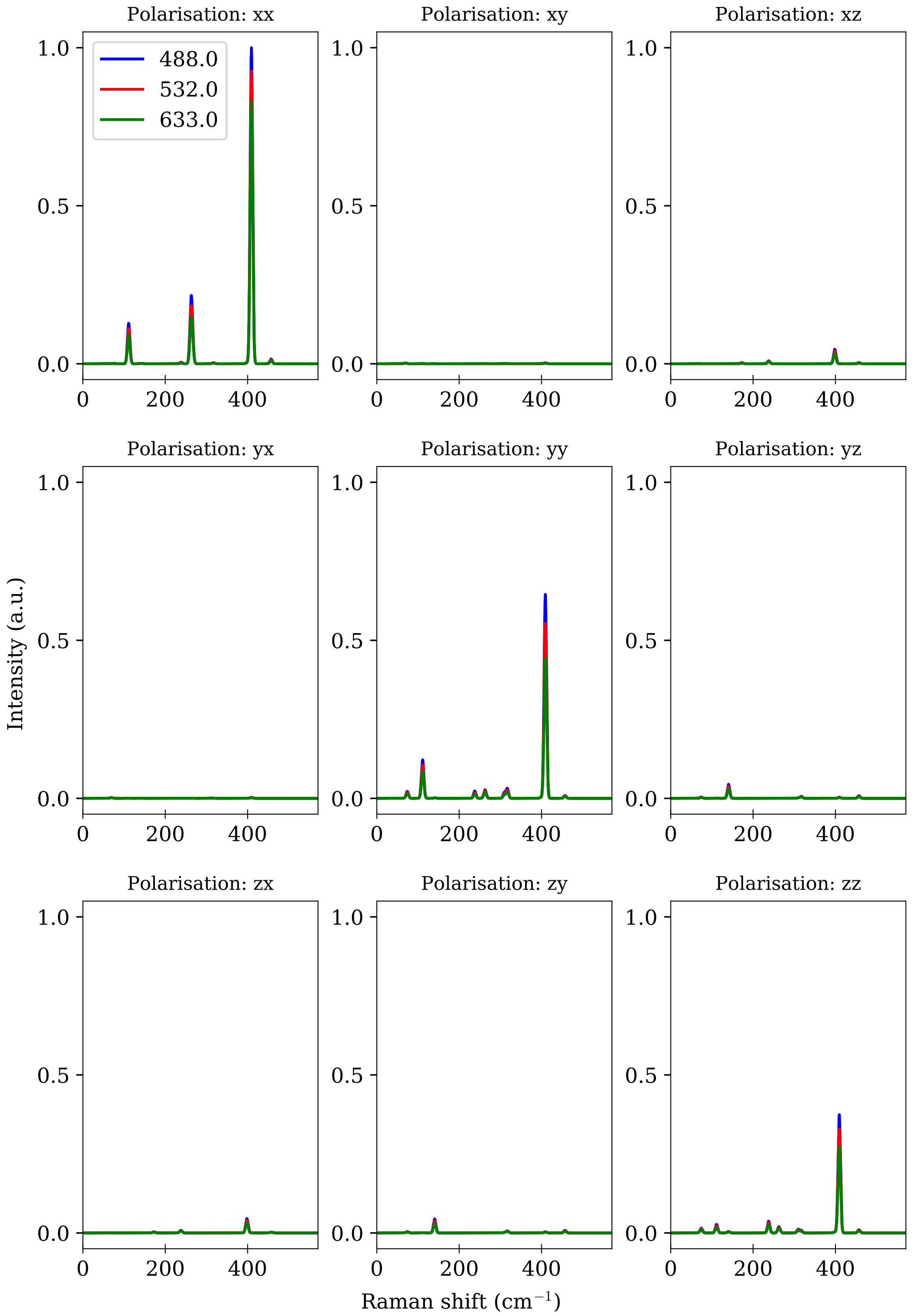

Supplementary Figure 16: Raman spectrum of $Al_2Br_2S_2$ with space group 59 and Wyckoff positions a-b-0.

$Al_2Br_2Se_2$, 59, a-b-0

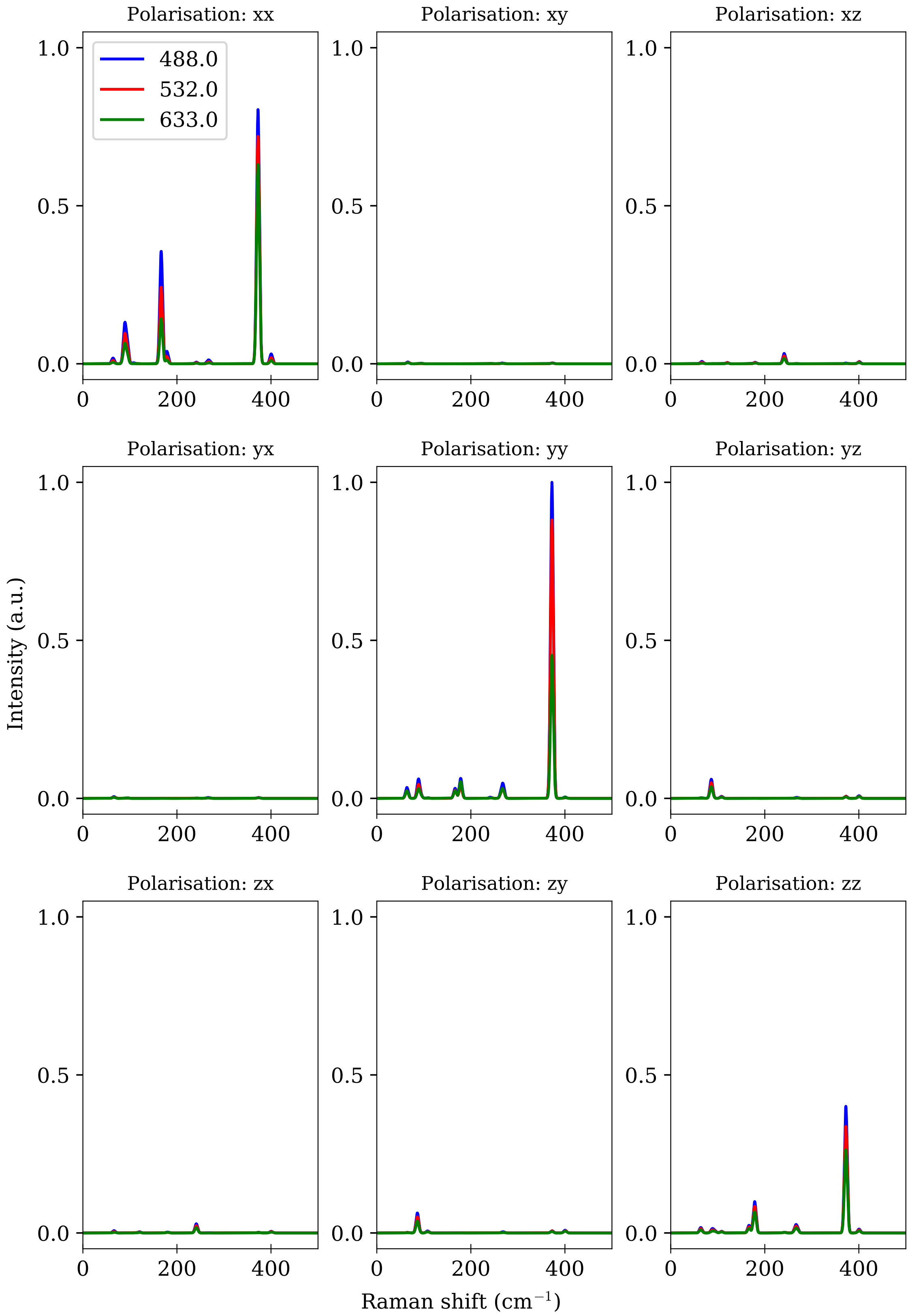

Supplementary Figure 17: Raman spectrum of $Al_2Br_2Se_2$ with space group 59 and Wyckoff positions a-b-0.

$Al_2Cl_2O_2$, 59, a-b-0

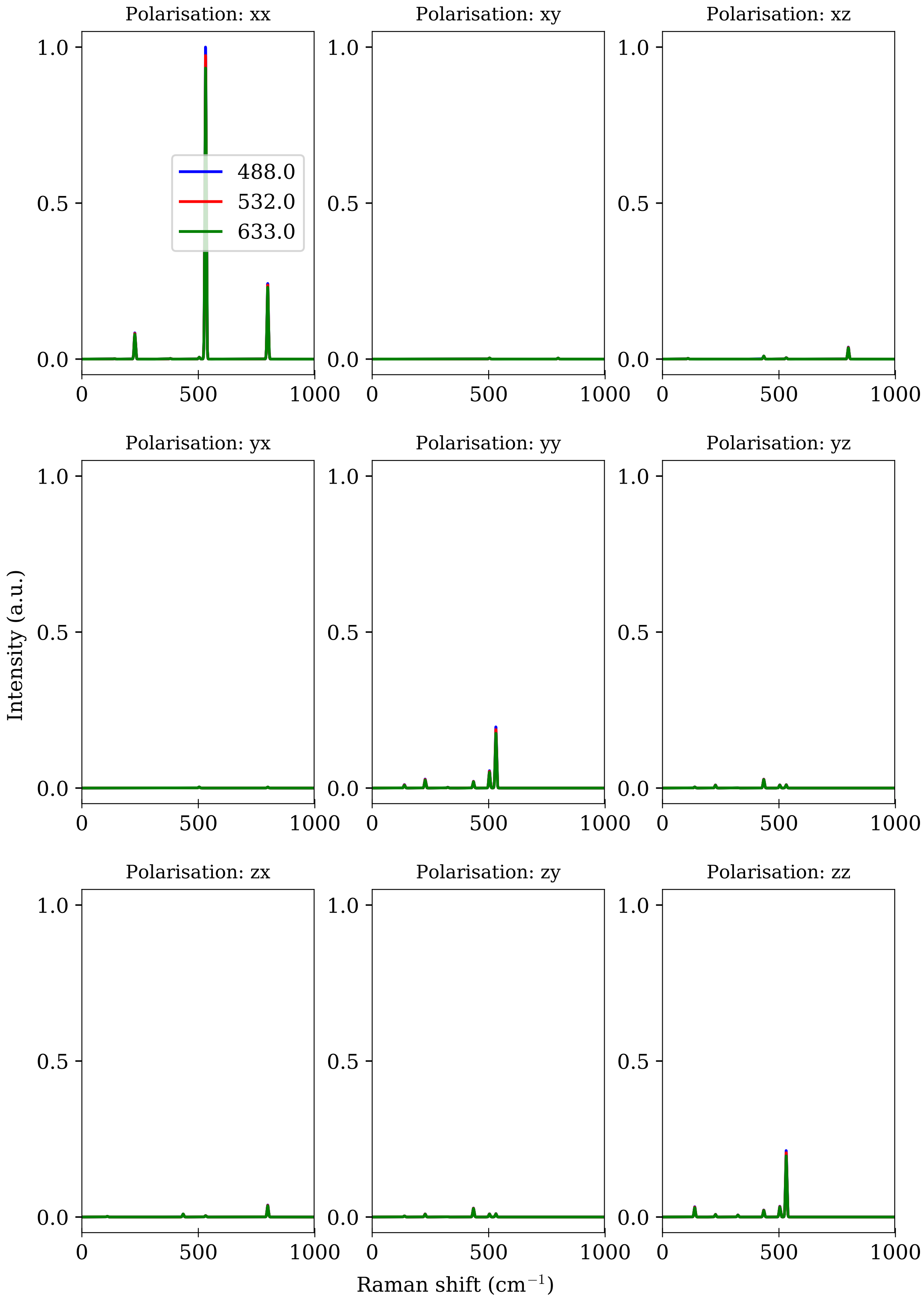

Supplementary Figure 18: Raman spectrum of $Al_2Cl_2O_2$ with space group 59 and Wyckoff positions a-b-0.

$Al_2Cl_2S_2$, 59, a-b-0

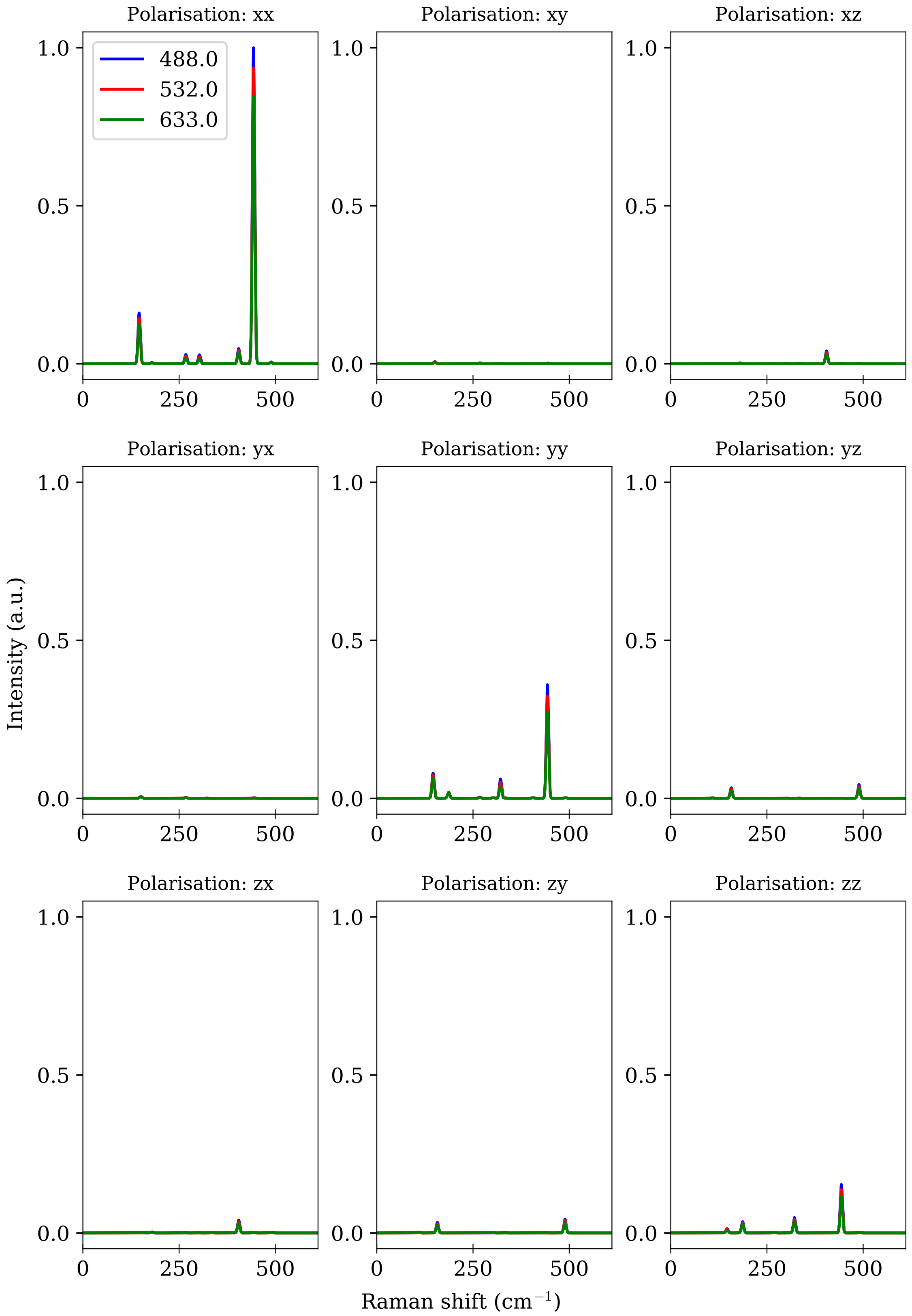

Supplementary Figure 19: Raman spectrum of $Al_2Cl_2S_2$ with space group 59 and Wyckoff positions a-b-0.

$Al_2I_2S_2$, 59, a-b-0

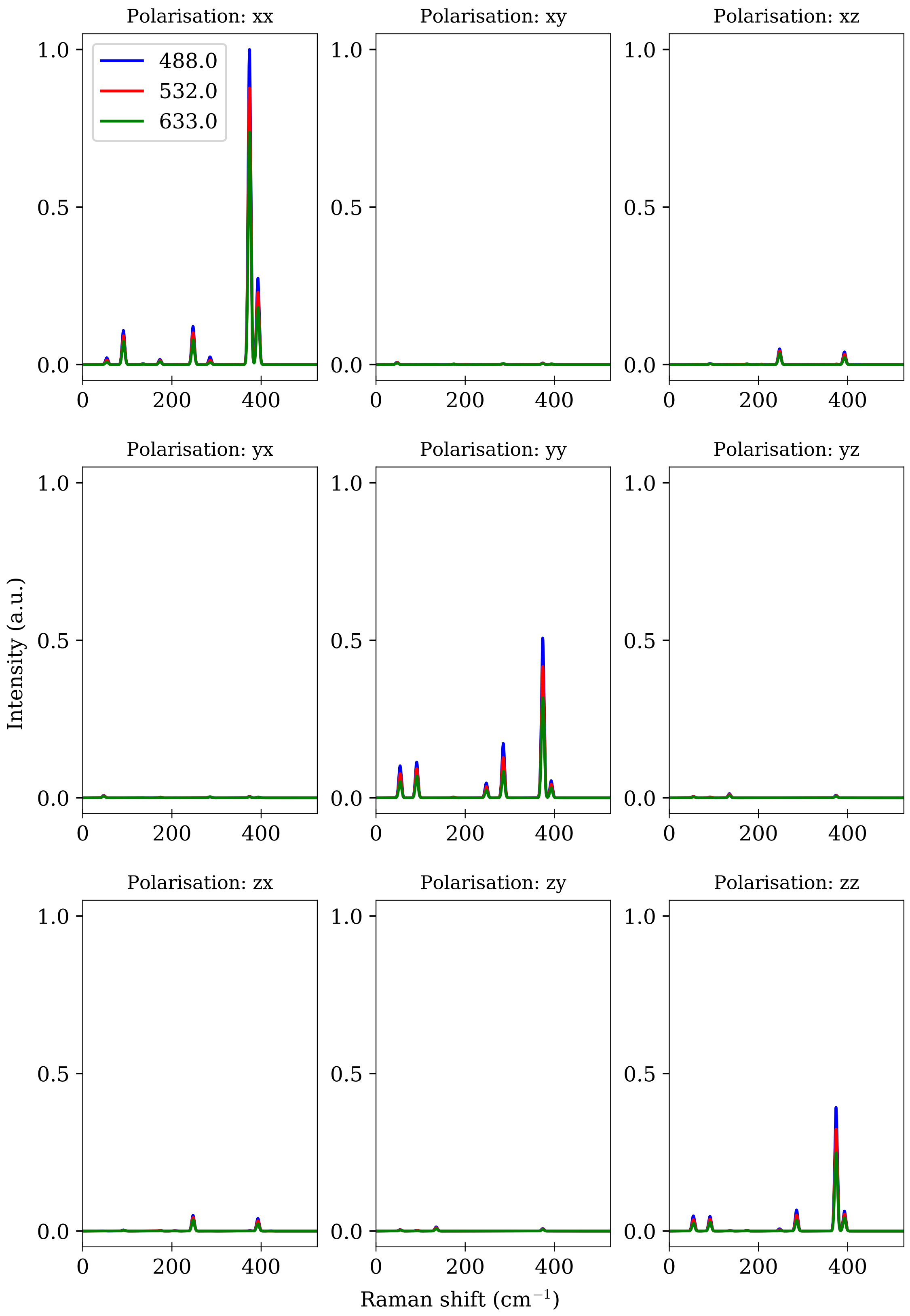

Supplementary Figure 20: Raman spectrum of $Al_2I_2S_2$ with space group 59 and Wyckoff positions a-b-0.

$Al_2I_2Se_2$, 59, a-b-0

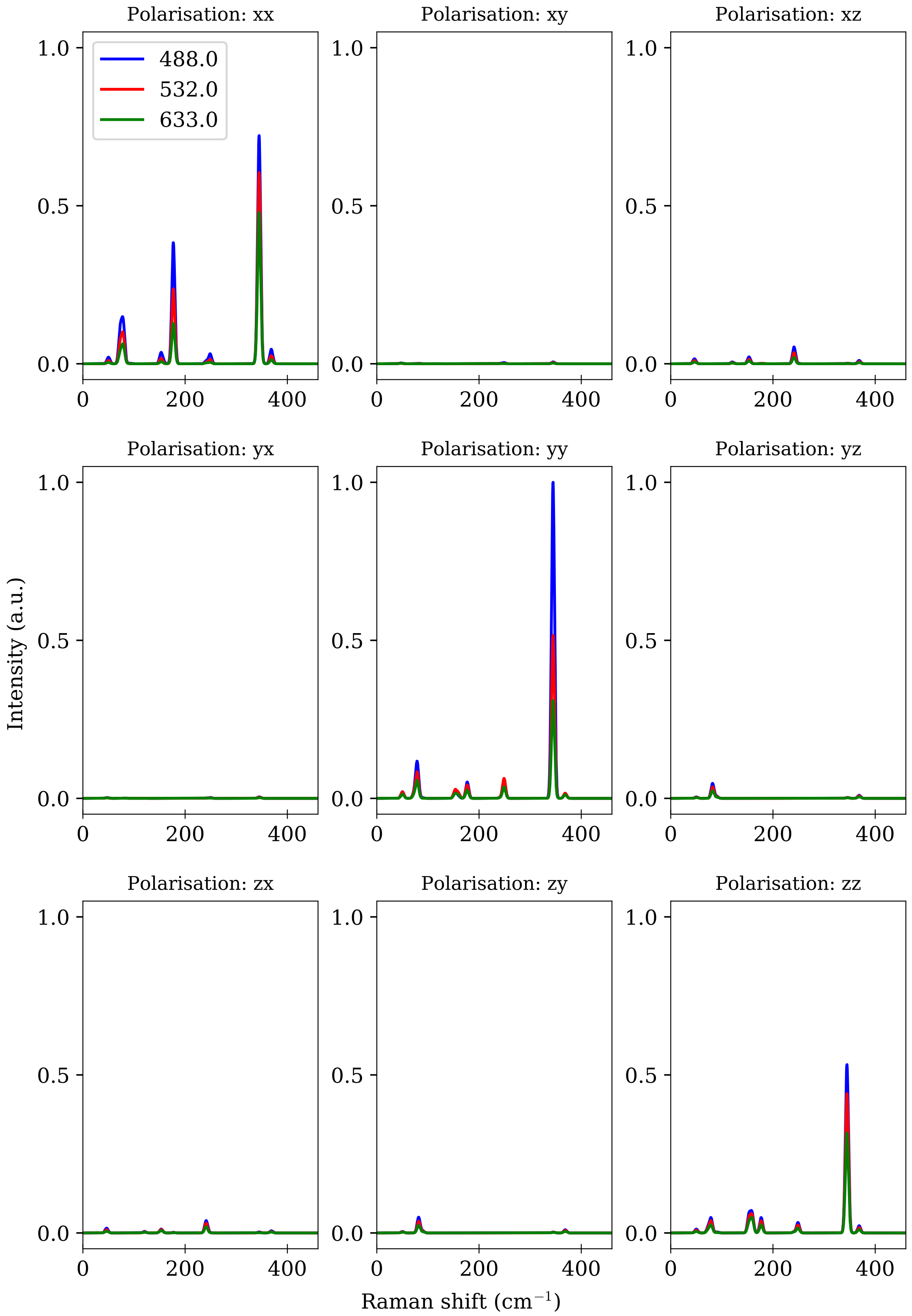

Supplementary Figure 21: Raman spectrum of $Al_2I_2Se_2$ with space group 59 and Wyckoff positions a-b-0.

Al_2O_2, 187, h-i-0

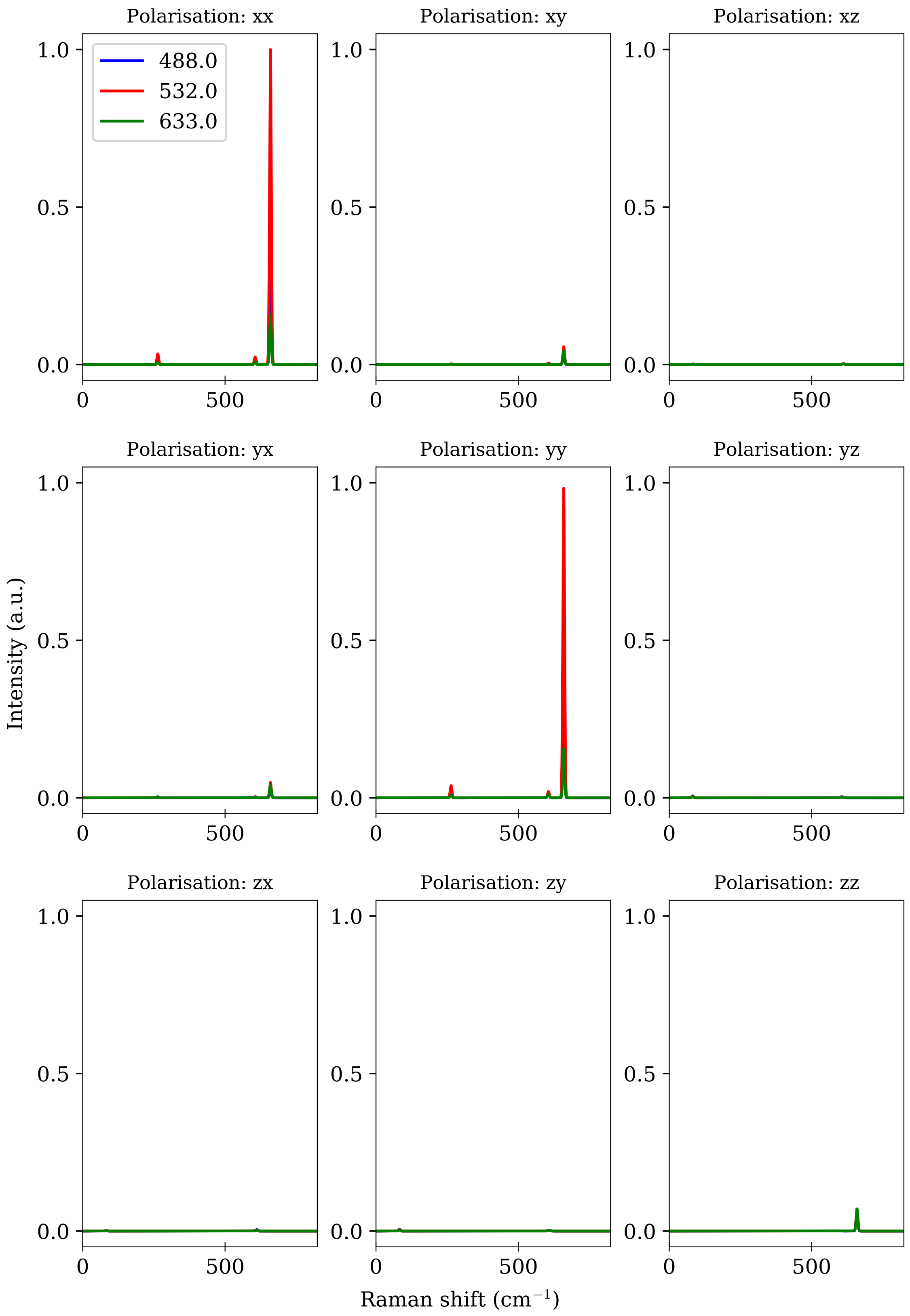

Supplementary Figure 22: Raman spectrum of Al_2O_2 with space group 187 and Wyckoff positions h-i-0.

Al_2S_2, 164, c-d-0

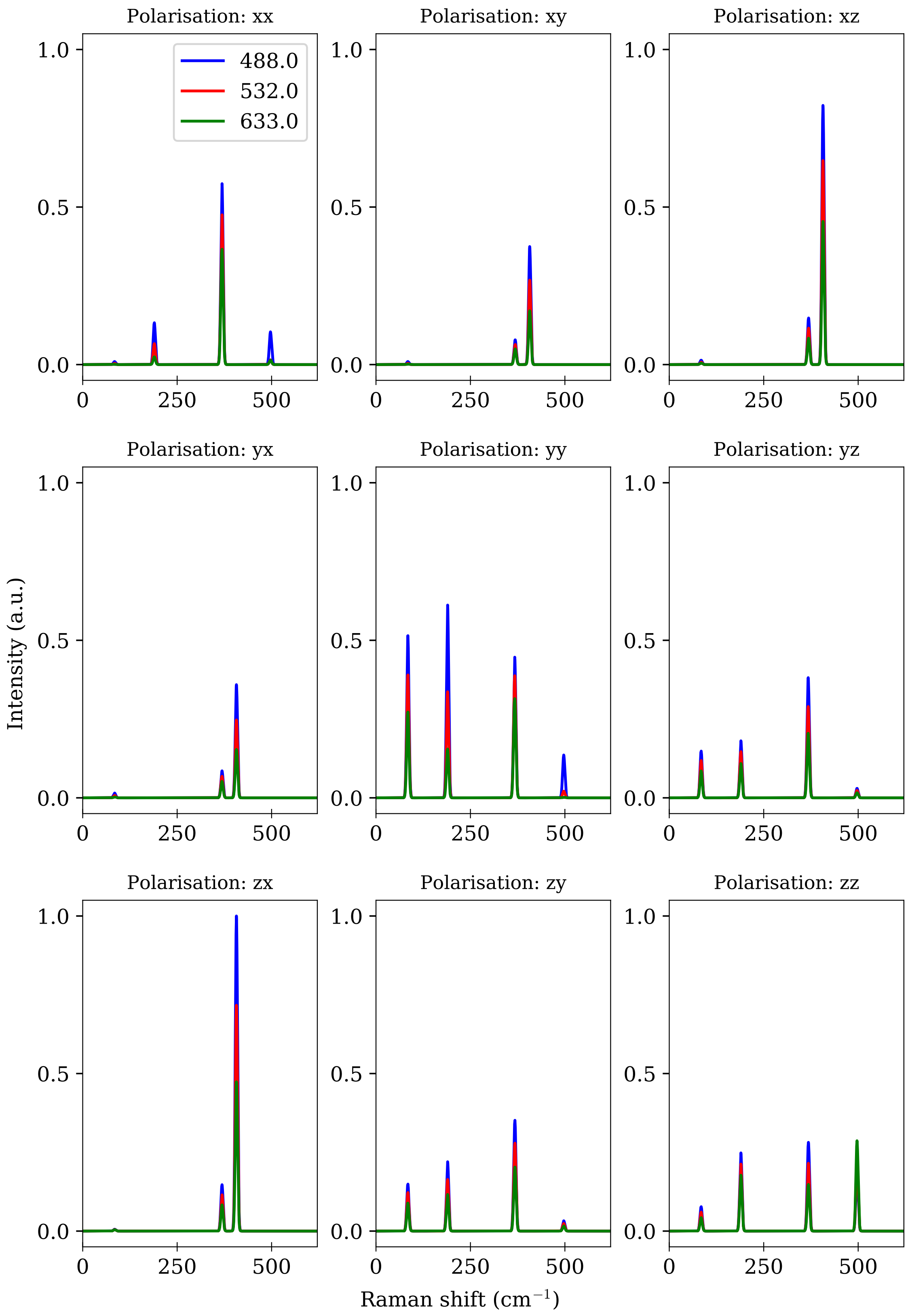

Supplementary Figure 23: Raman spectrum of Al_2S_2 with space group 164 and Wyckoff positions c-d-0.

Al_2S_2, 187, h-i-0

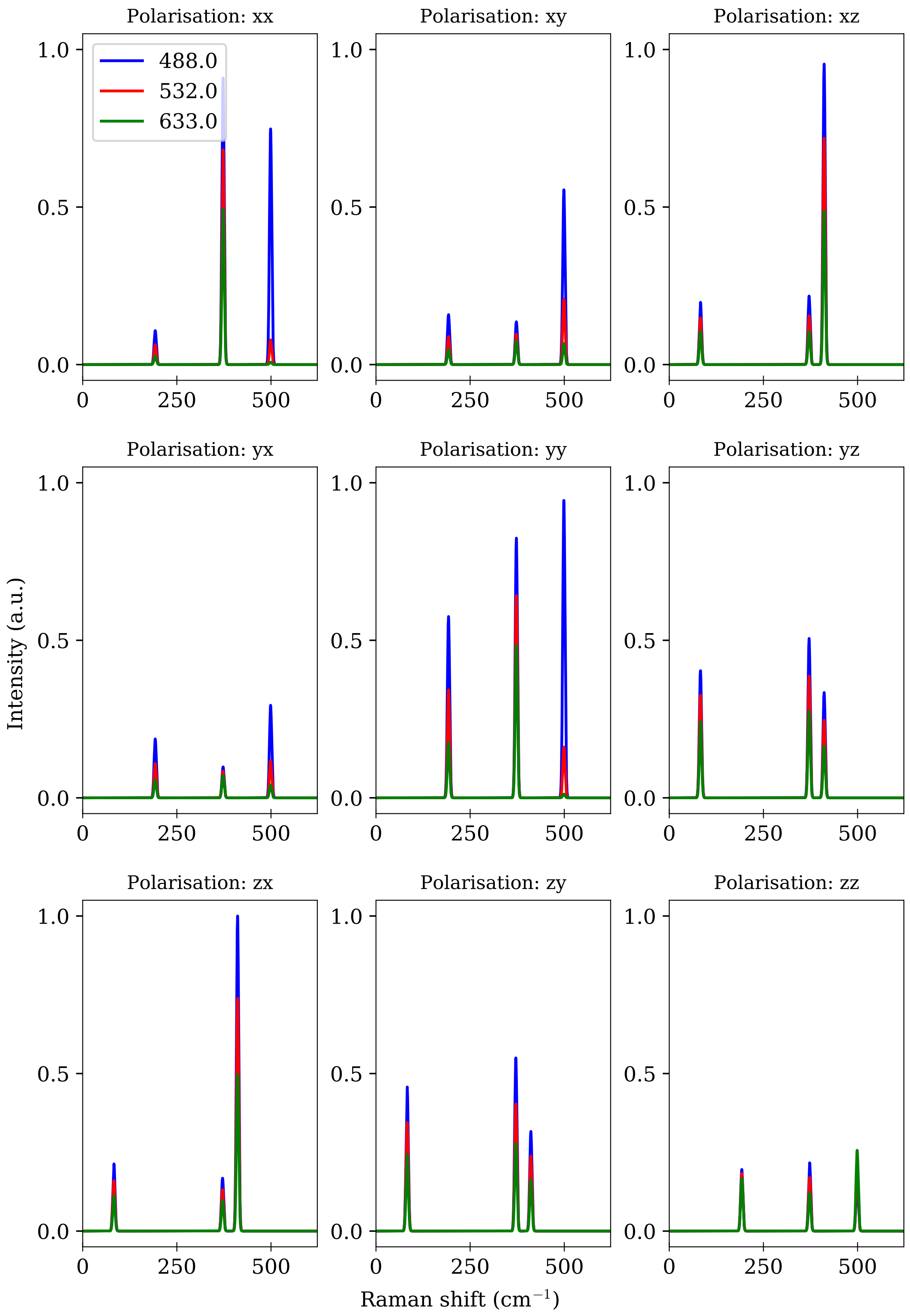

Supplementary Figure 24: Raman spectrum of Al_2S_2 with space group 187 and Wyckoff positions h-i-0.

Al_2S_4, 10, f-g-m-n-0

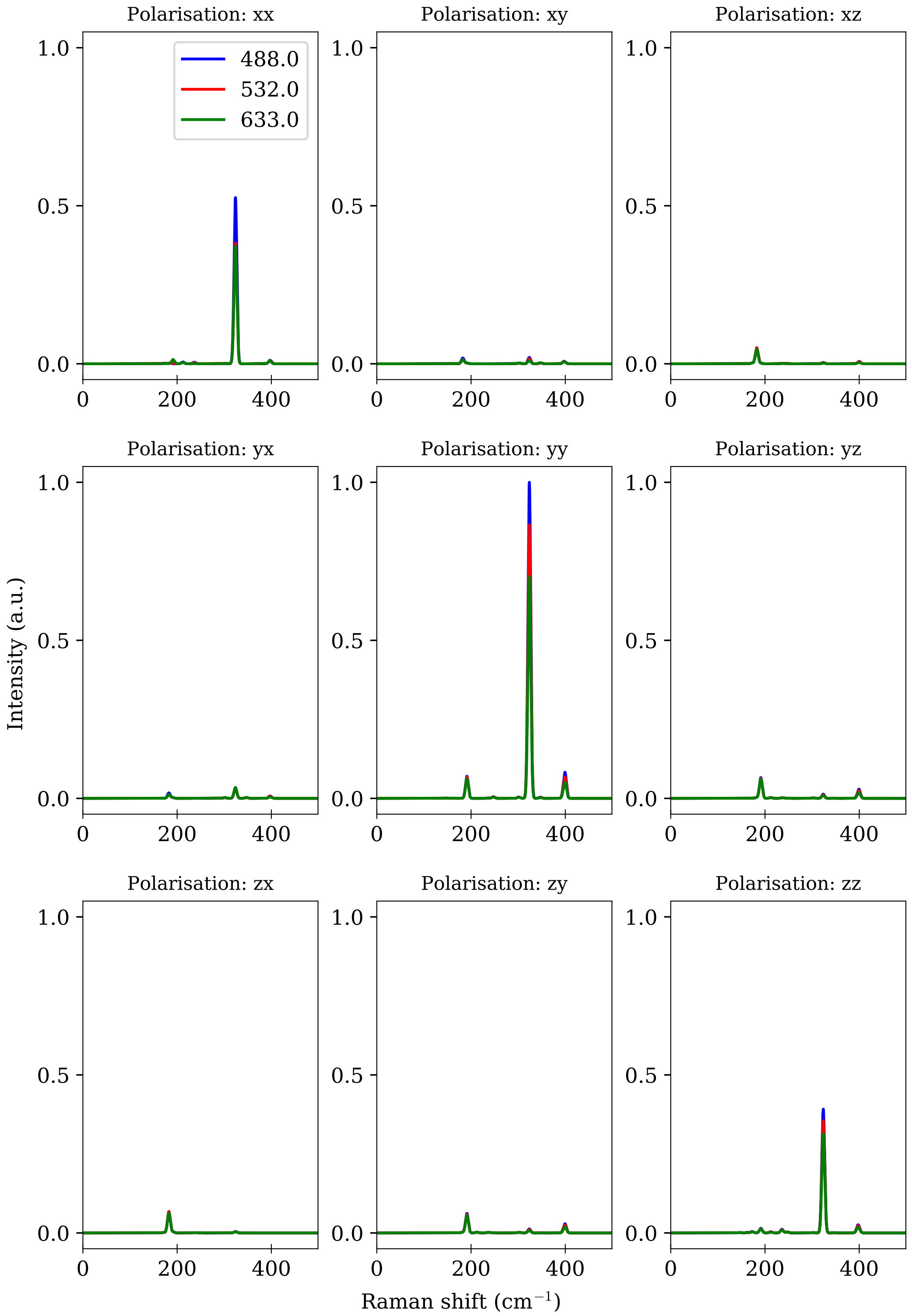

Supplementary Figure 25: Raman spectrum of Al_2S_4 with space group 10 and Wyckoff positions f-g-m-n-0.

Al_2Se_2, 164, c-d-0

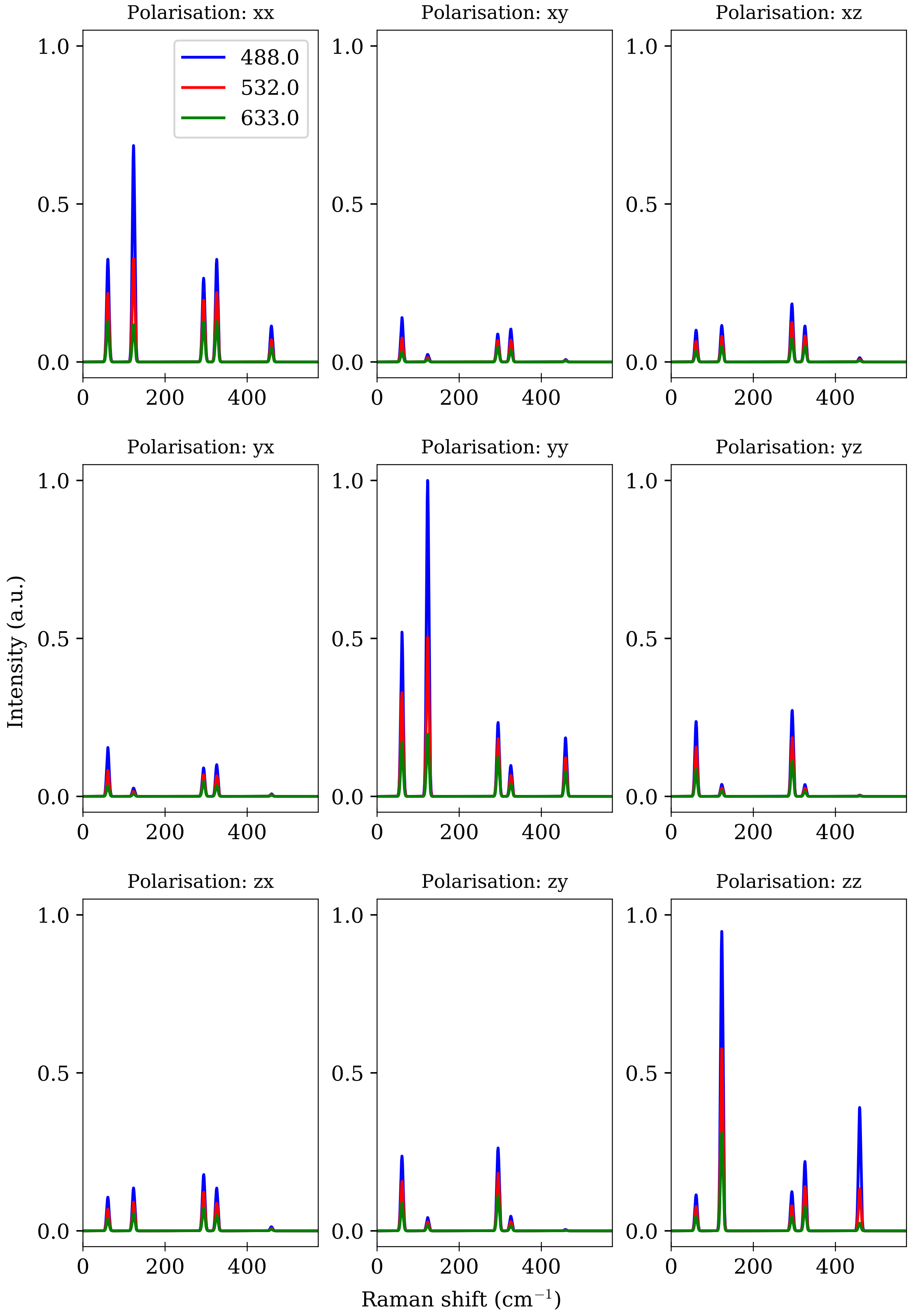

Supplementary Figure 26: Raman spectrum of Al_2Se_2 with space group 164 and Wyckoff positions c-d-0.

Al_2Se_2, 187, h-i-0

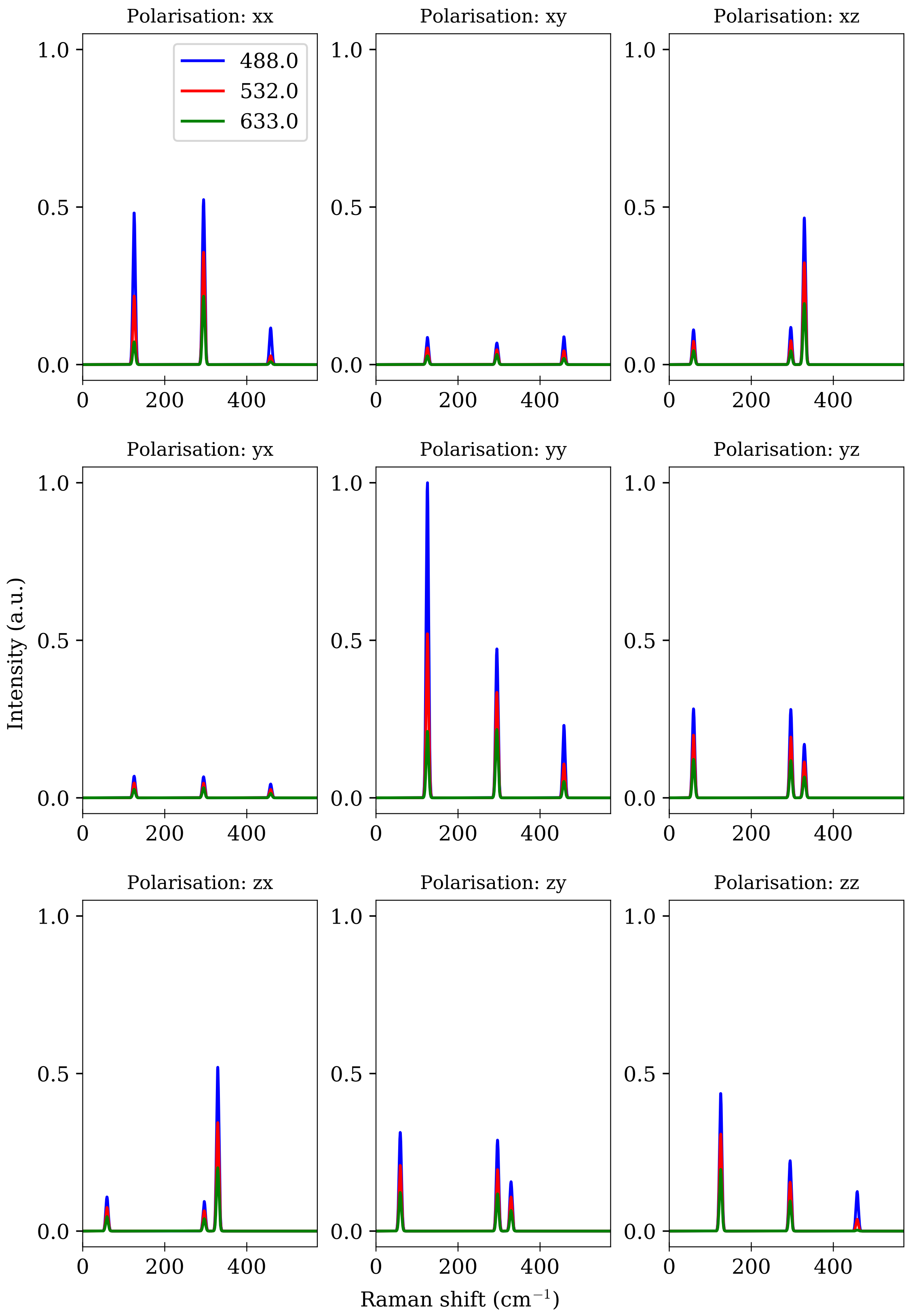

Supplementary Figure 27: Raman spectrum of Al_2Se_2 with space group 187 and Wyckoff positions h-i-0.

Al_2Se_4, 2, f-g-i-0

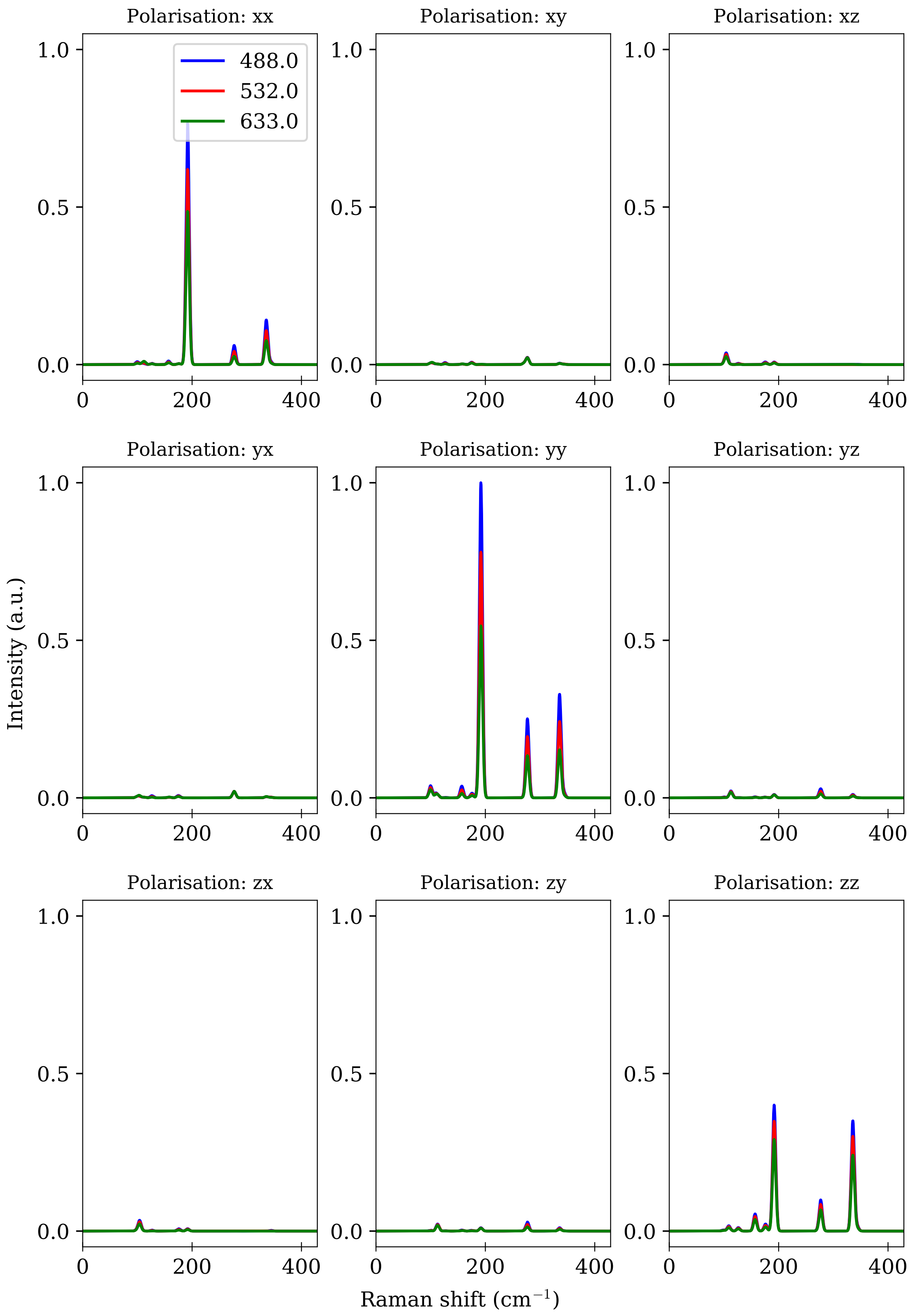

Supplementary Figure 28: Raman spectrum of Al_2Se_4 with space group 2 and Wyckoff positions f-g-i-0.

Al_2Te_2, 187, h-i-0

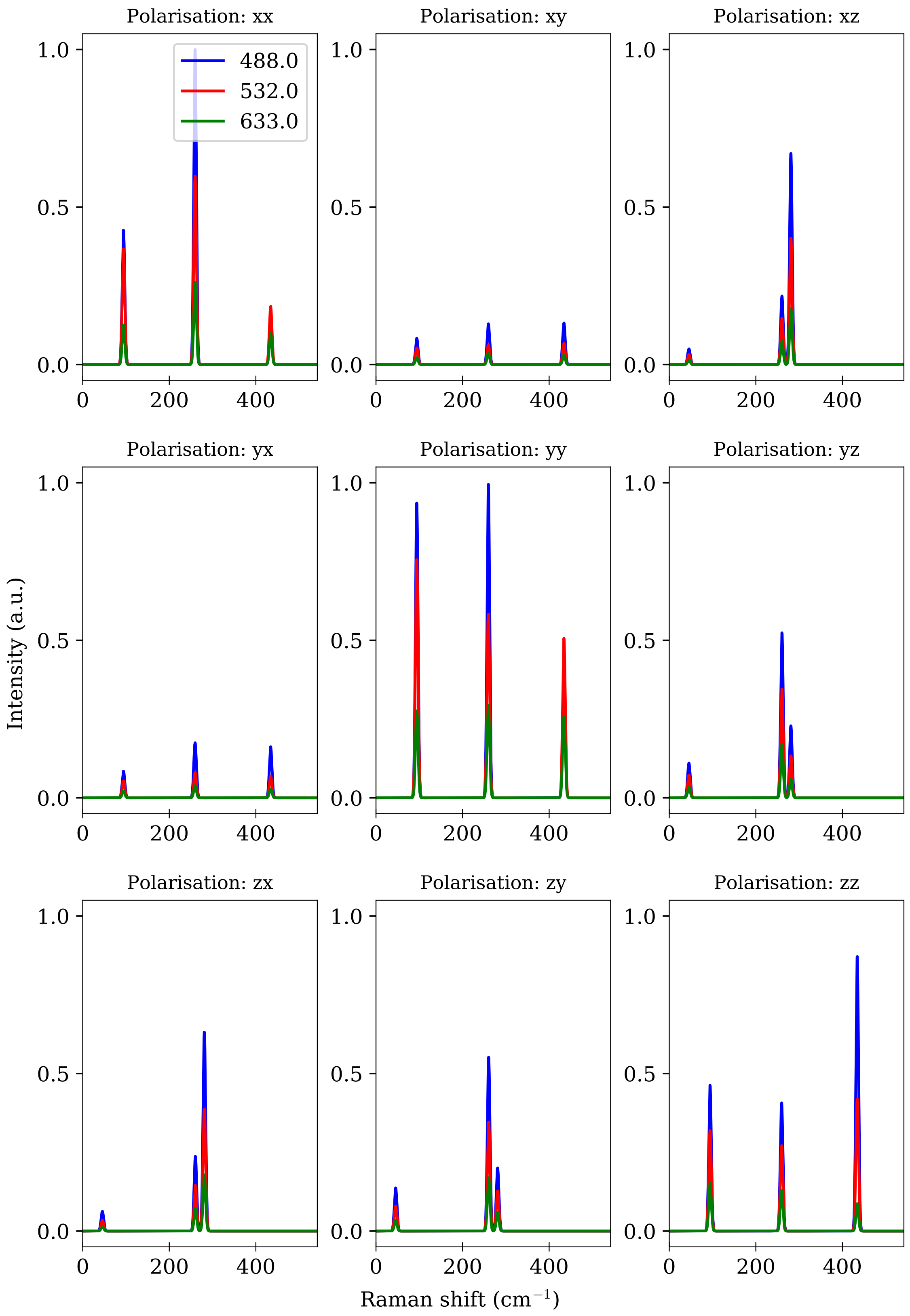

Supplementary Figure 29: Raman spectrum of Al_2Te_2 with space group 187 and Wyckoff positions h-i-0.

Al_2Te_4, 12, d-i-0

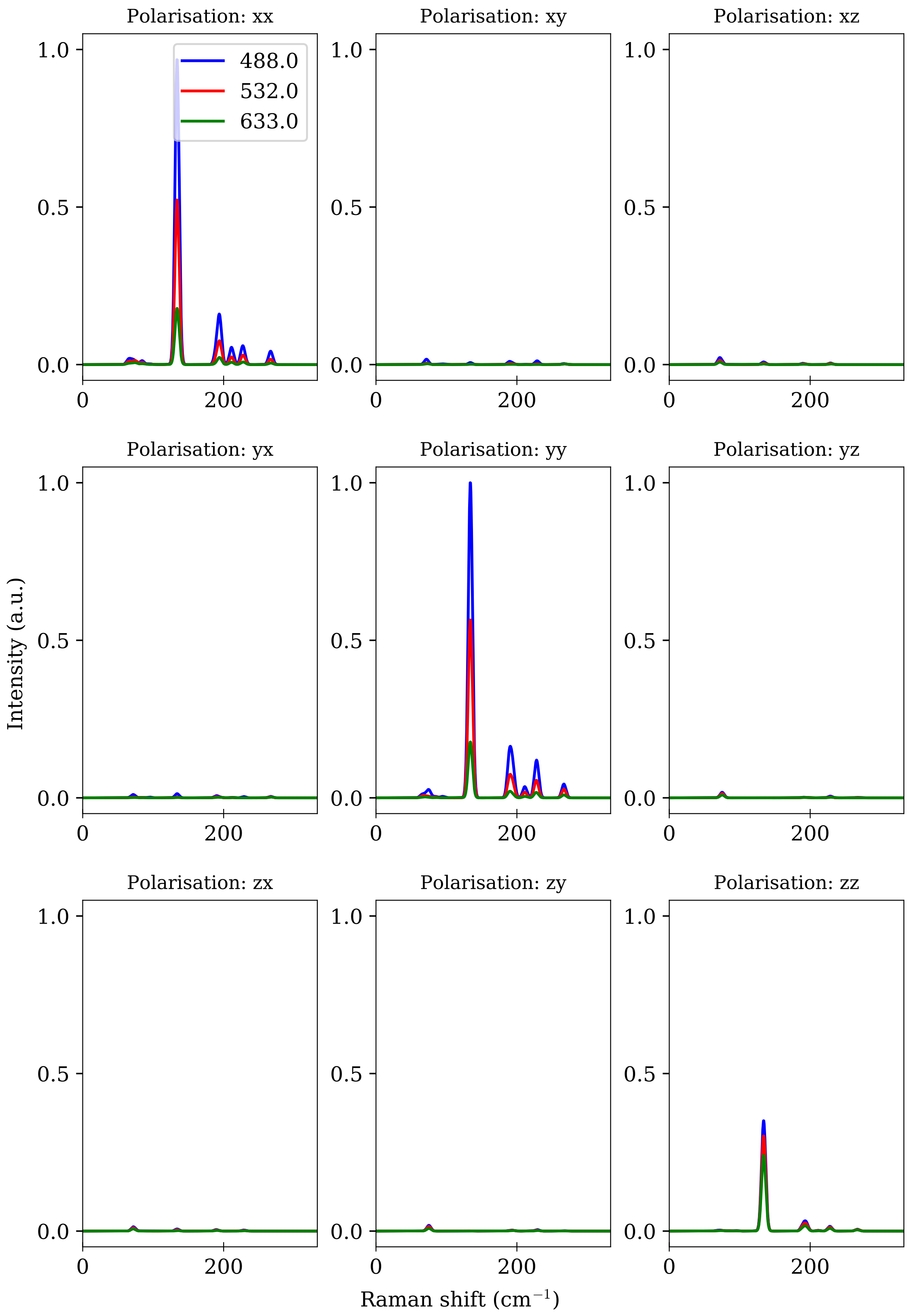

Supplementary Figure 30: Raman spectrum of Al_2Te_4 with space group 12 and Wyckoff positions d-i-0.

AlI_2, 187, b-i-0

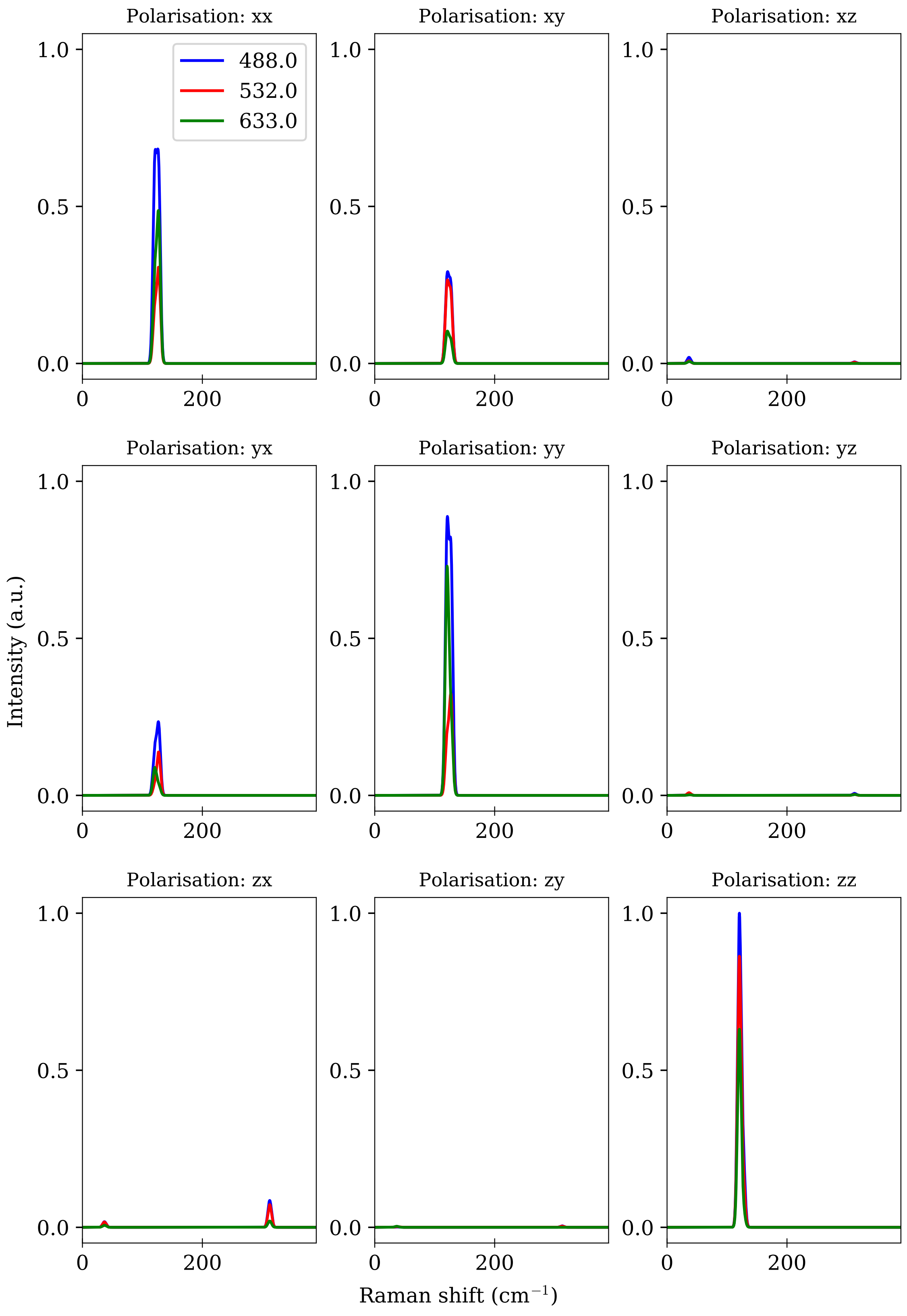

Supplementary Figure 31: Raman spectrum of AlI_2 with space group 187 and Wyckoff positions b-i-0.

AlN, 187, d-f-0

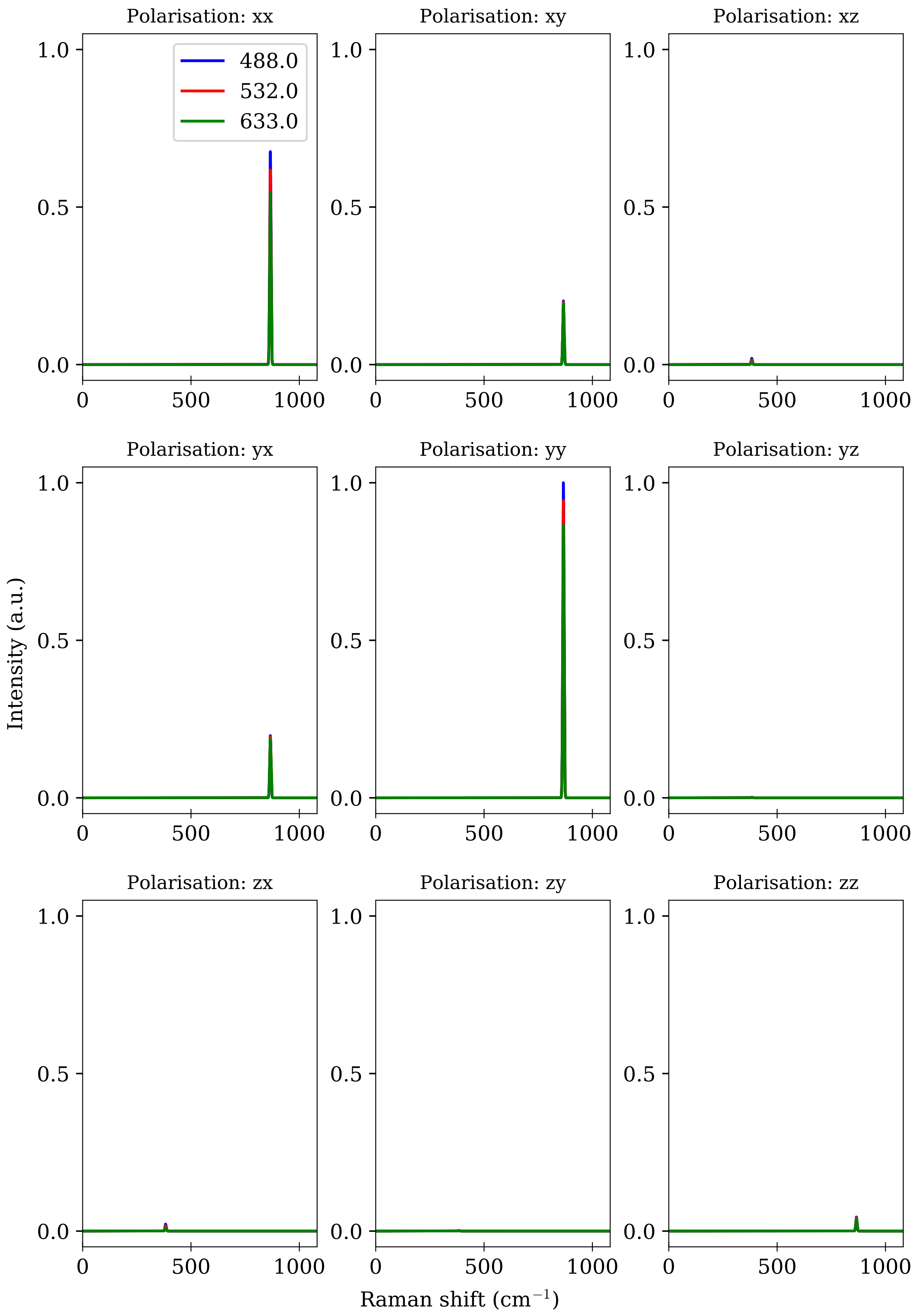

Supplementary Figure 32: Raman spectrum of AlN with space group 187 and Wyckoff positions d-f-0.

As_2, 164, d-0

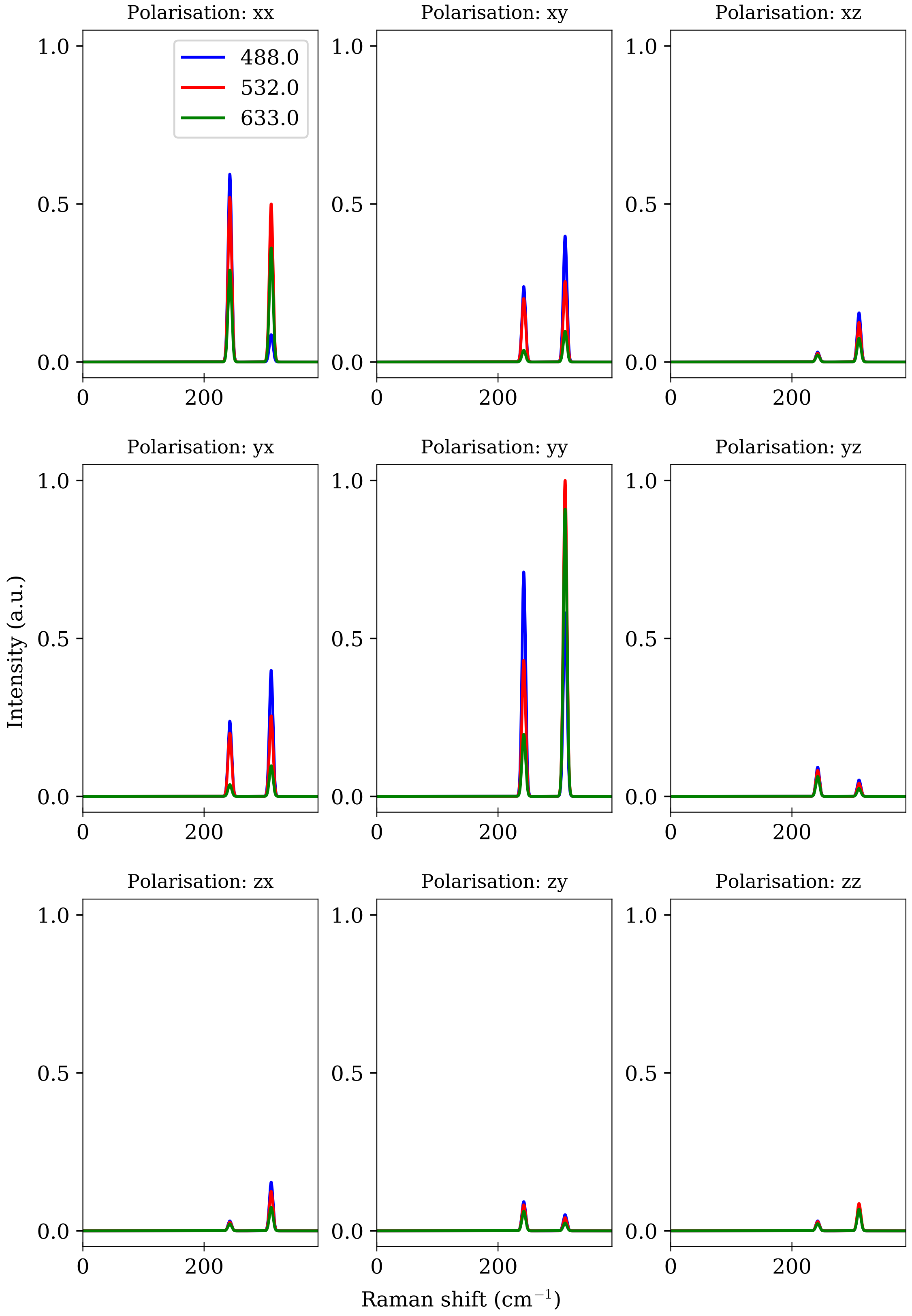

Supplementary Figure 33: Raman spectrum of As_2 with space group 164 and Wyckoff positions d-0.

AsBrSe, 156, a-b-c-0

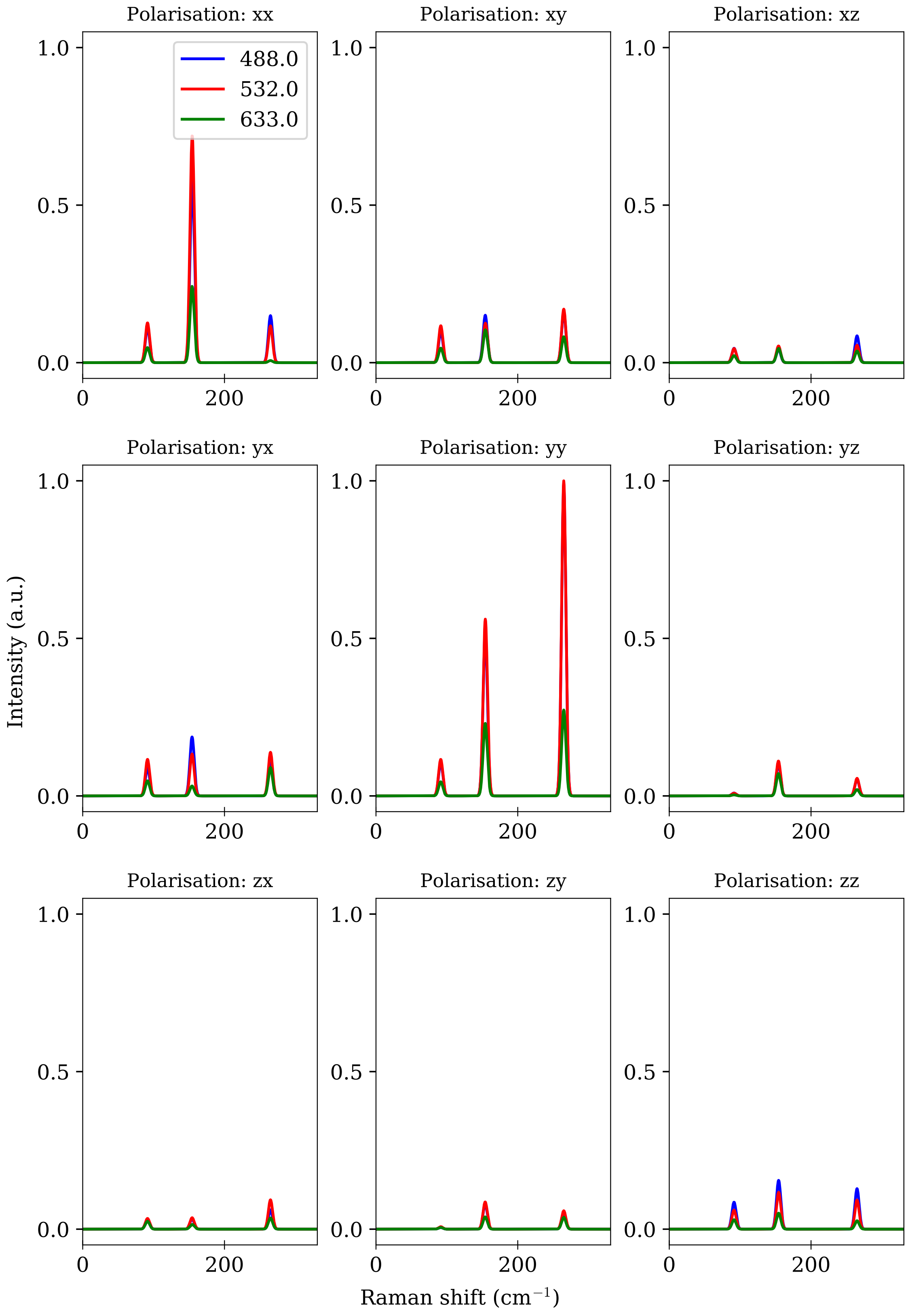

Supplementary Figure 34: Raman spectrum of AsBrSe with space group 156 and Wyckoff positions a-b-c-0.

AsBrS, 156, a-b-c-0

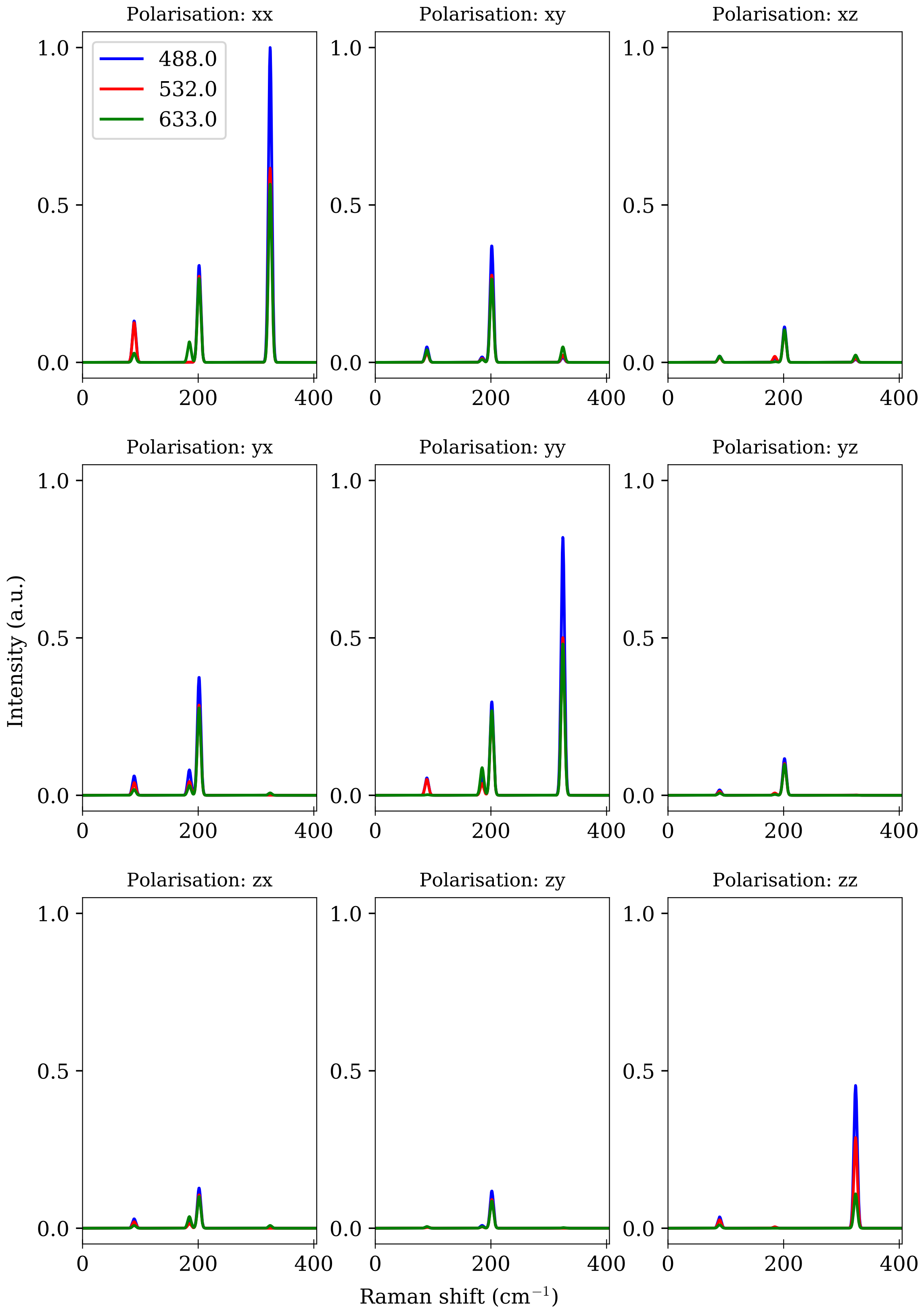

Supplementary Figure 35: Raman spectrum of AsBrS with space group 156 and Wyckoff positions a-b-c-0.

AsBrS, 156, a-c-0

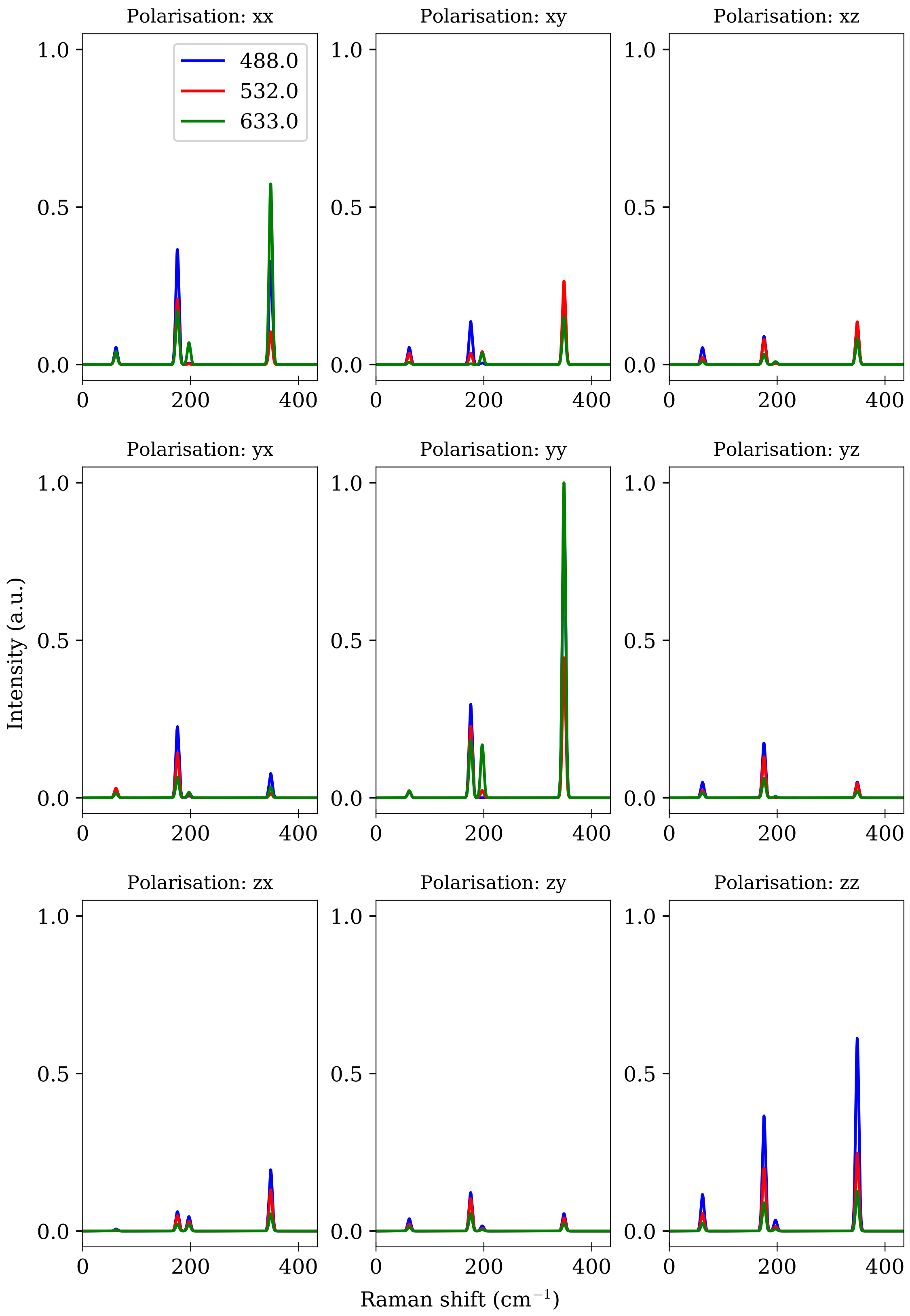

Supplementary Figure 36: Raman spectrum of AsBrS with space group 156 and Wyckoff positions a-c-0.

AsBrTe, 156, a-b-c-0

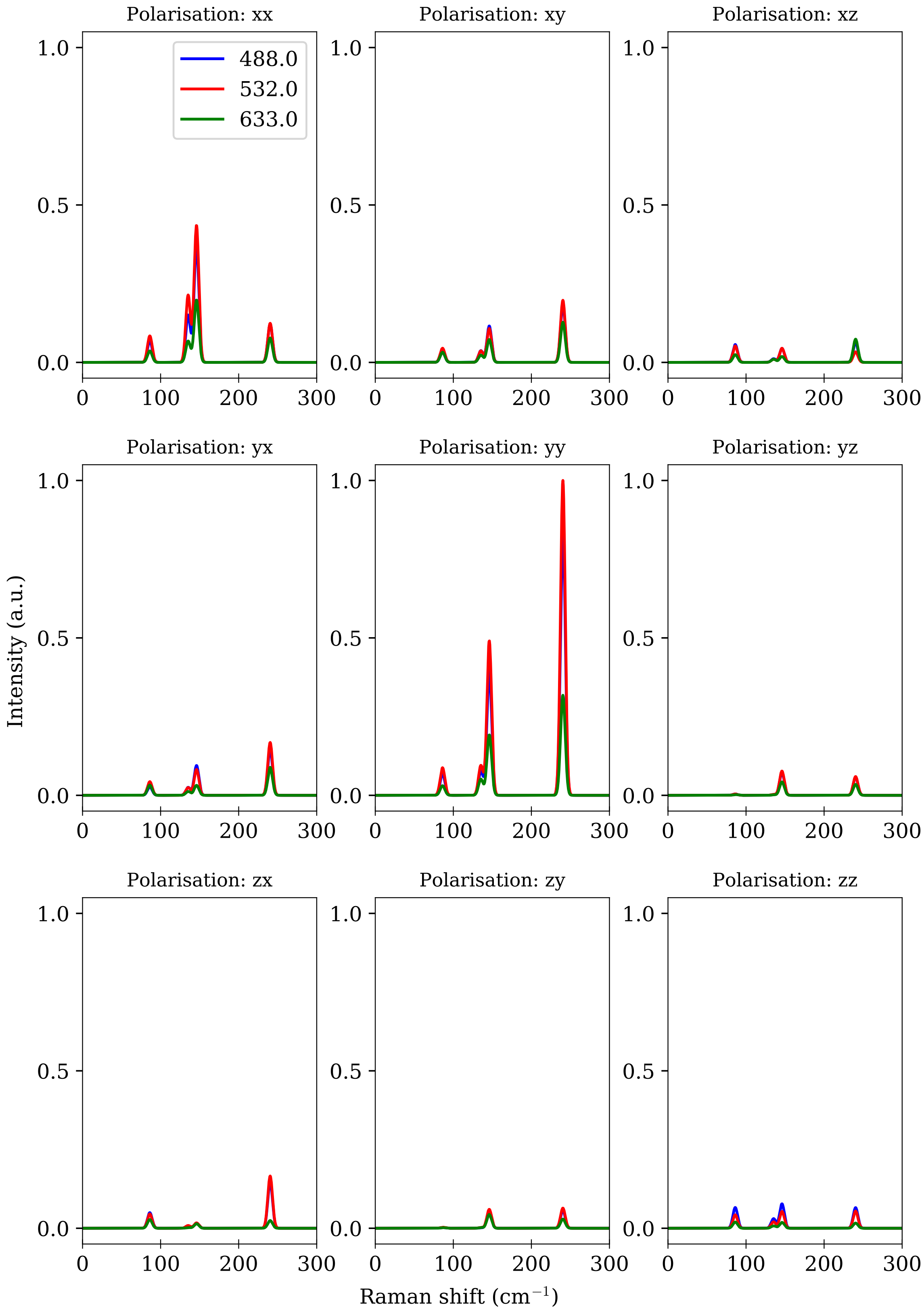

Supplementary Figure 37: Raman spectrum of AsBrTe with space group 156 and Wyckoff positions a-b-c-0.

AsBrTe, 156, a-c-0

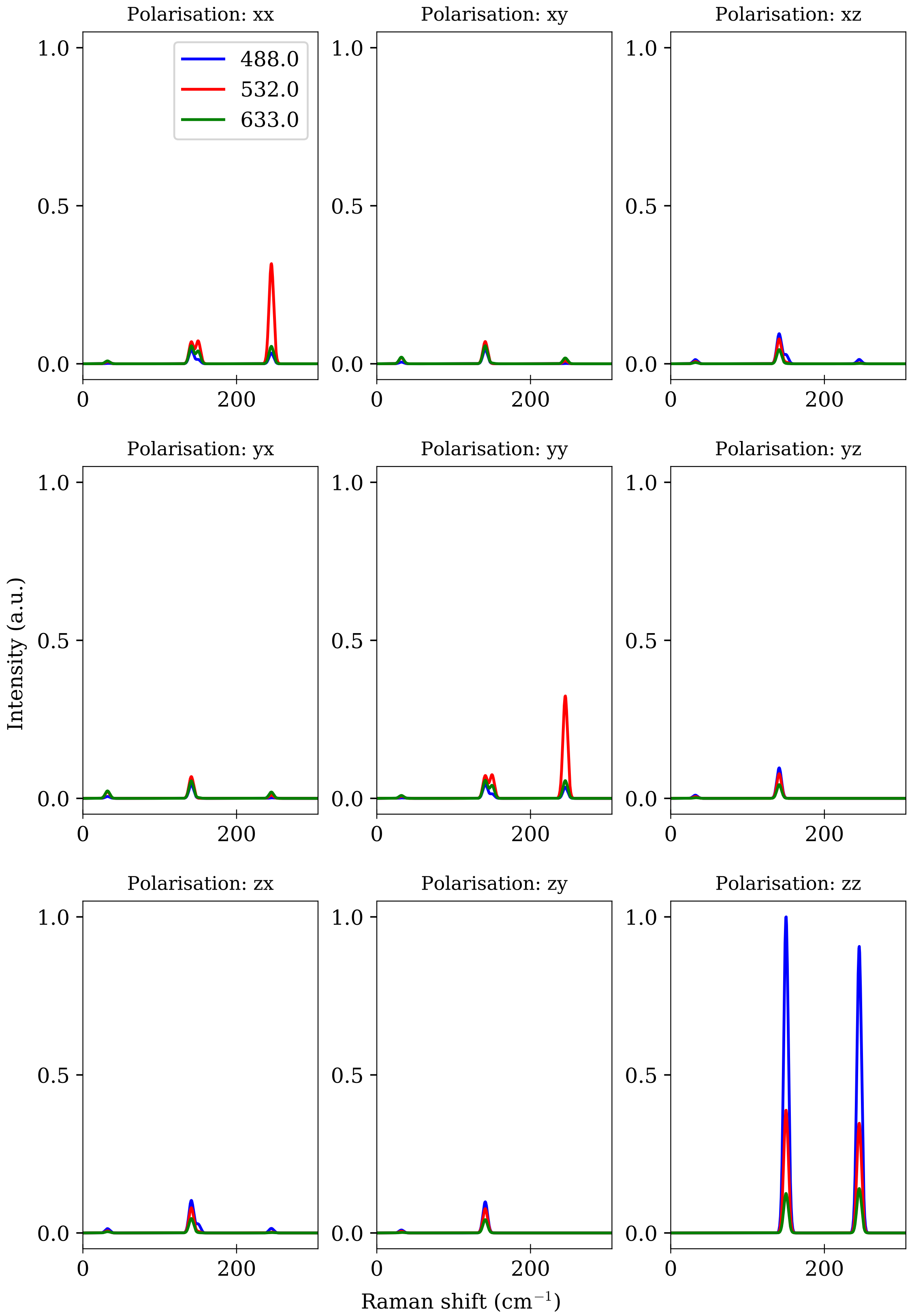

Supplementary Figure 38: Raman spectrum of AsBrTe with space group 156 and Wyckoff positions a-c-0.

AsB, 187, d-f-0

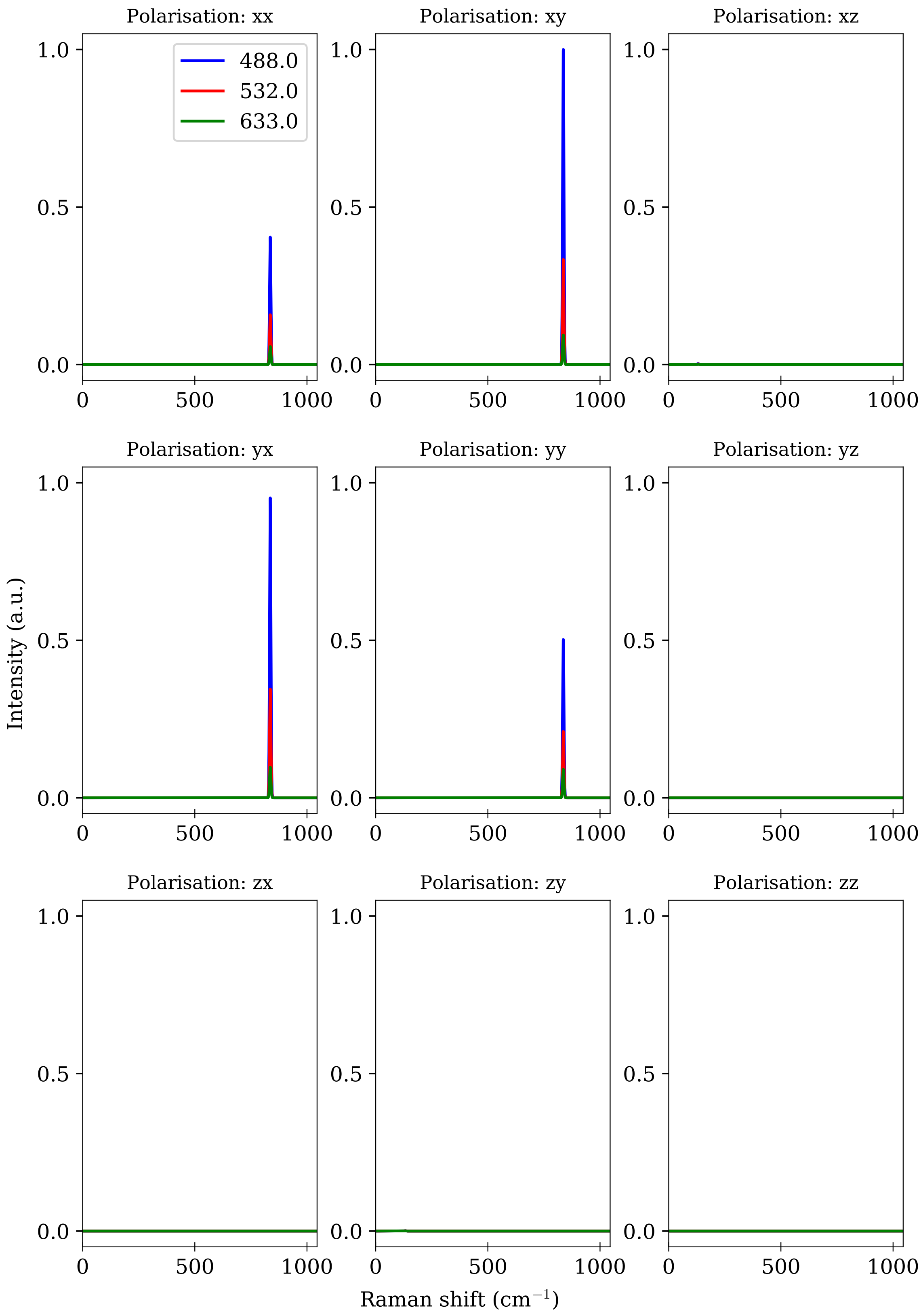

Supplementary Figure 39: Raman spectrum of AsB with space group 187 and Wyckoff positions d-f-0.

AsClSe, 156, a-b-c-0

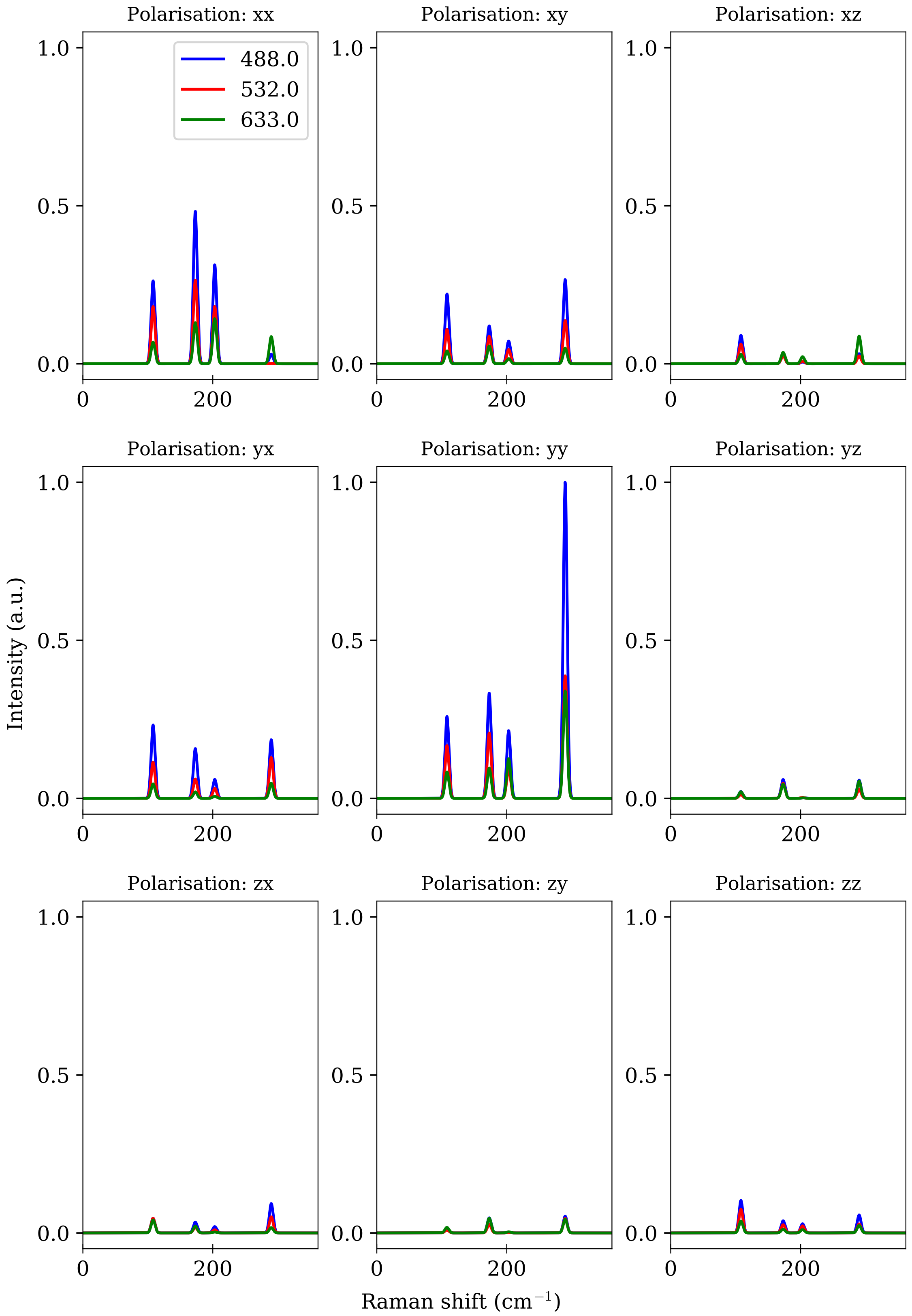

Supplementary Figure 40: Raman spectrum of AsClSe with space group 156 and Wyckoff positions a-b-c-0.

AsClS, 156, a-b-c-0

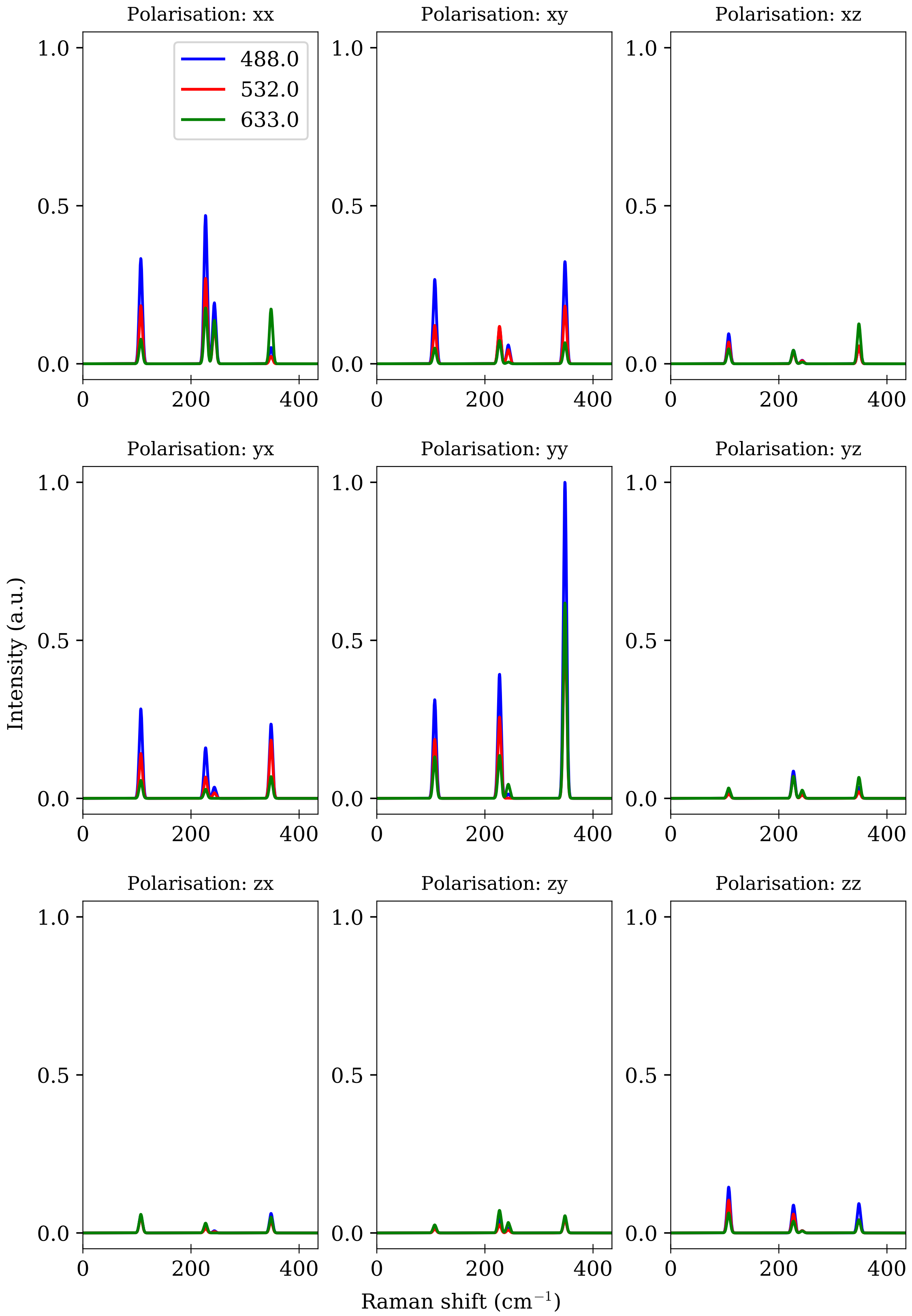

Supplementary Figure 41: Raman spectrum of AsClS with space group 156 and Wyckoff positions a-b-c-0.

AsClTe, 156, a-b-c-0

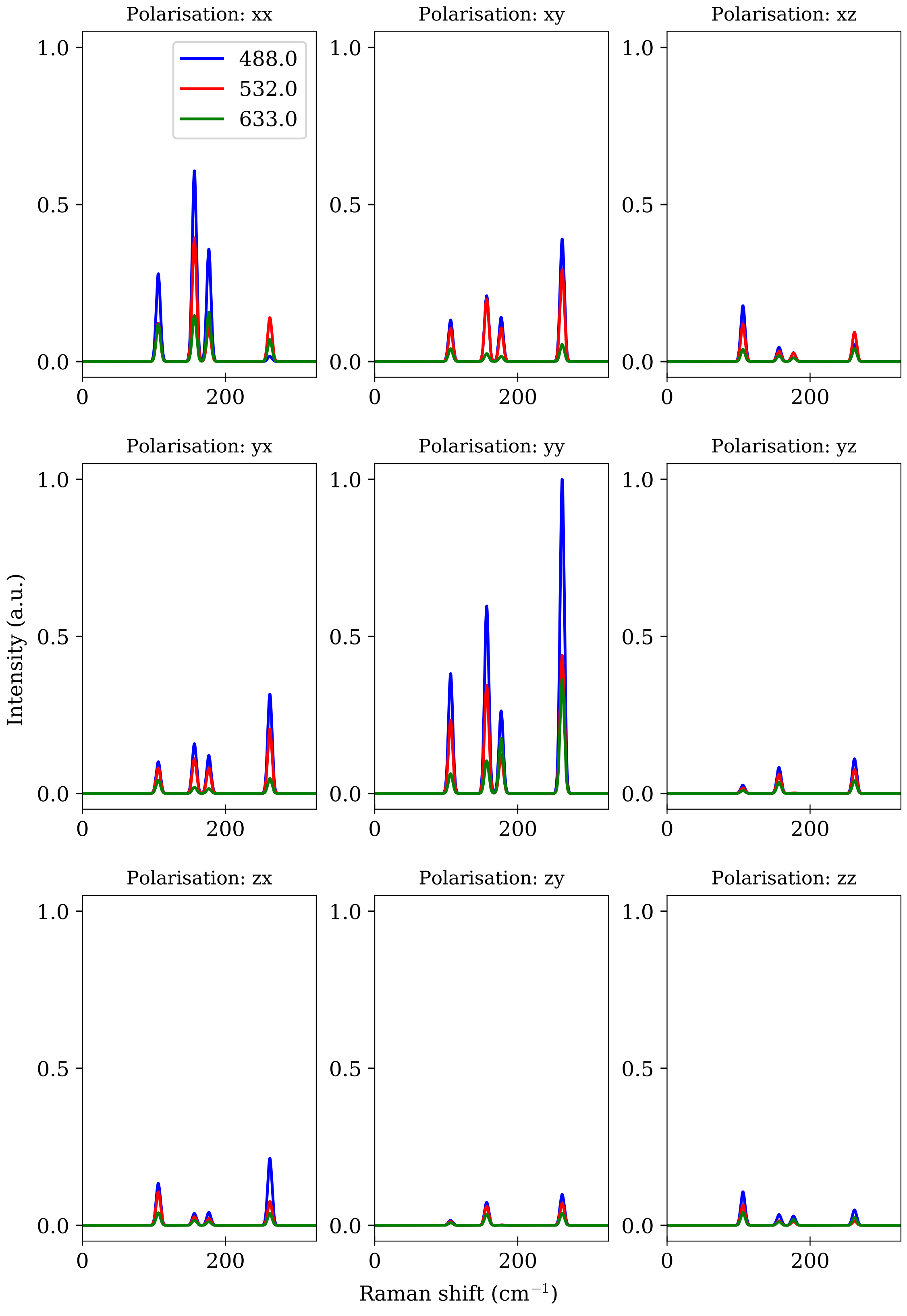

Supplementary Figure 42: Raman spectrum of AsClTe with space group 156 and Wyckoff positions a-b-c-0.

AsISe, 156, a-b-c-0

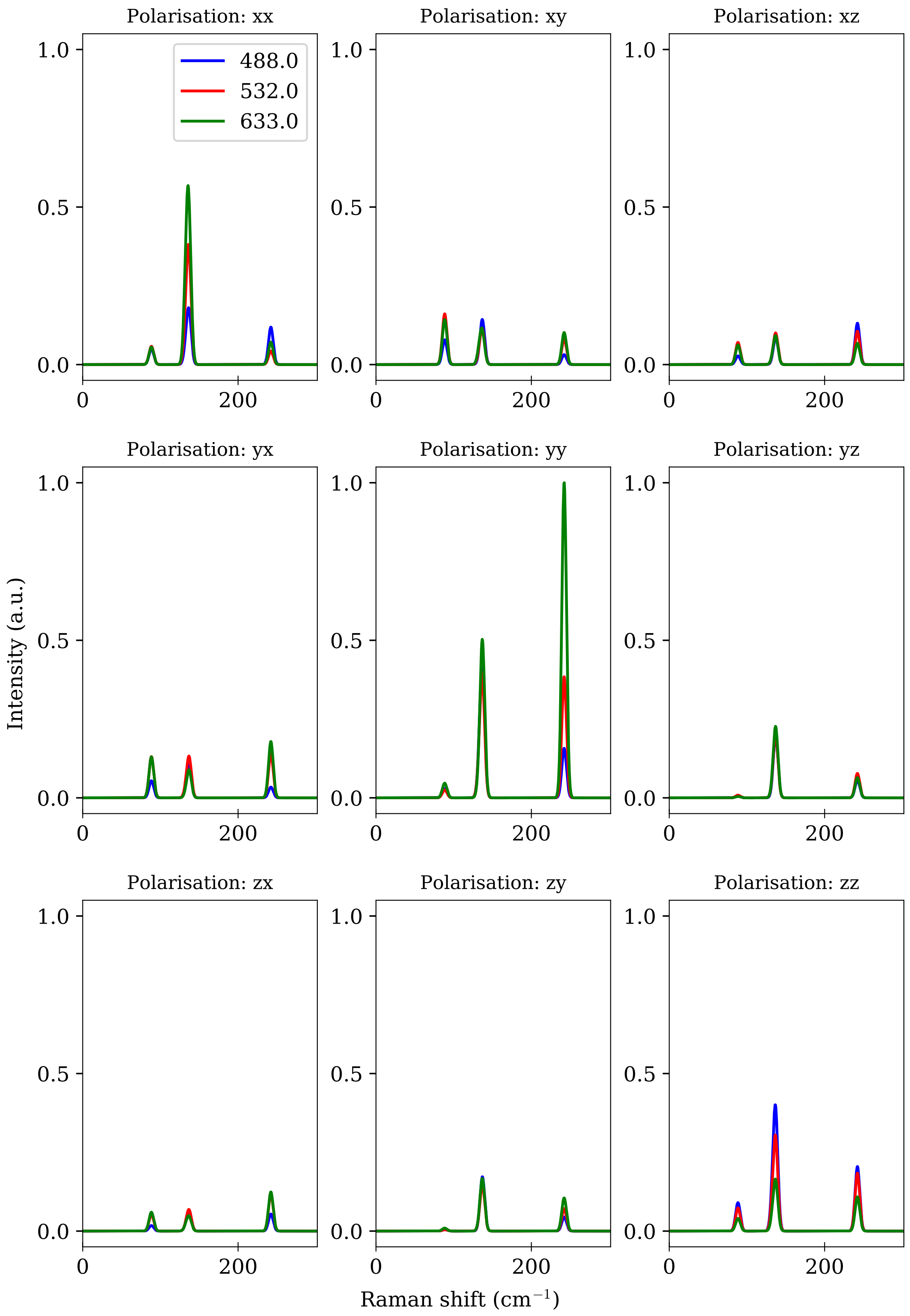

Supplementary Figure 43: Raman spectrum of AsISe with space group 156 and Wyckoff positions a-b-c-0.

AsISe, 156, a-c-0

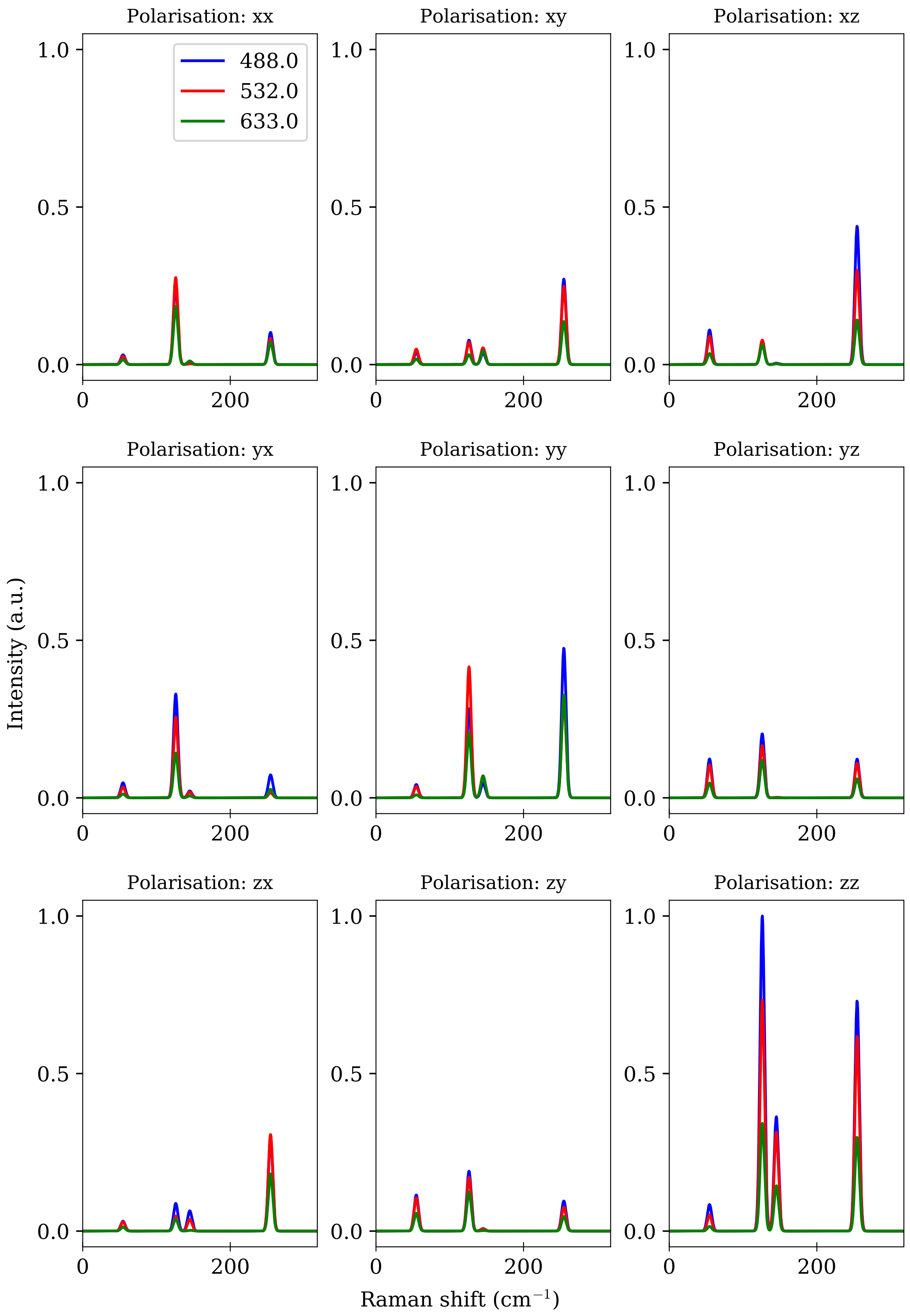

Supplementary Figure 44: Raman spectrum of AsISe with space group 156 and Wyckoff positions a-c-0.

AsIS, 156, a-b-c-0

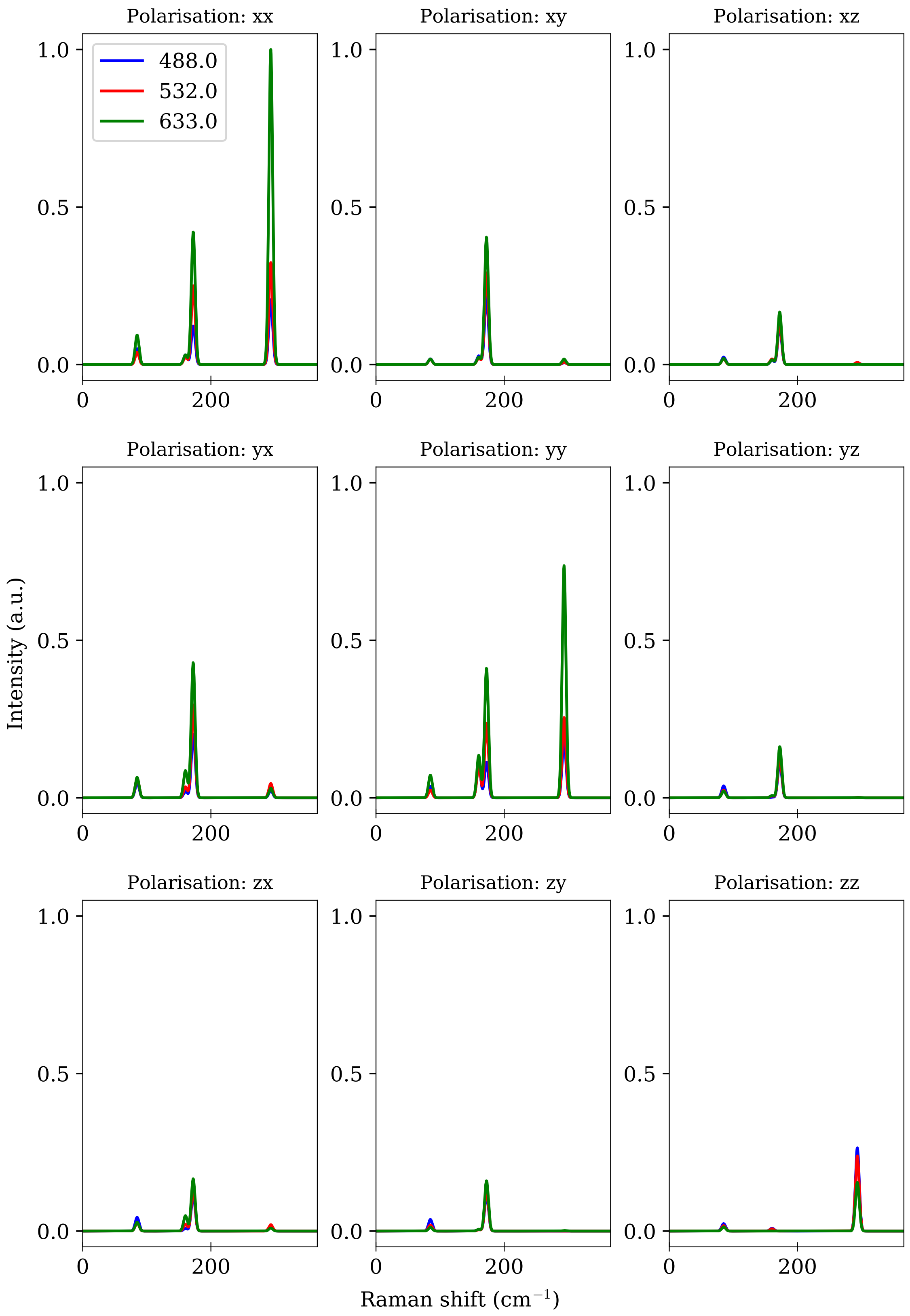

Supplementary Figure 45: Raman spectrum of AsIS with space group 156 and Wyckoff positions a-b-c-0.

AsITe, 156, a-b-c-0

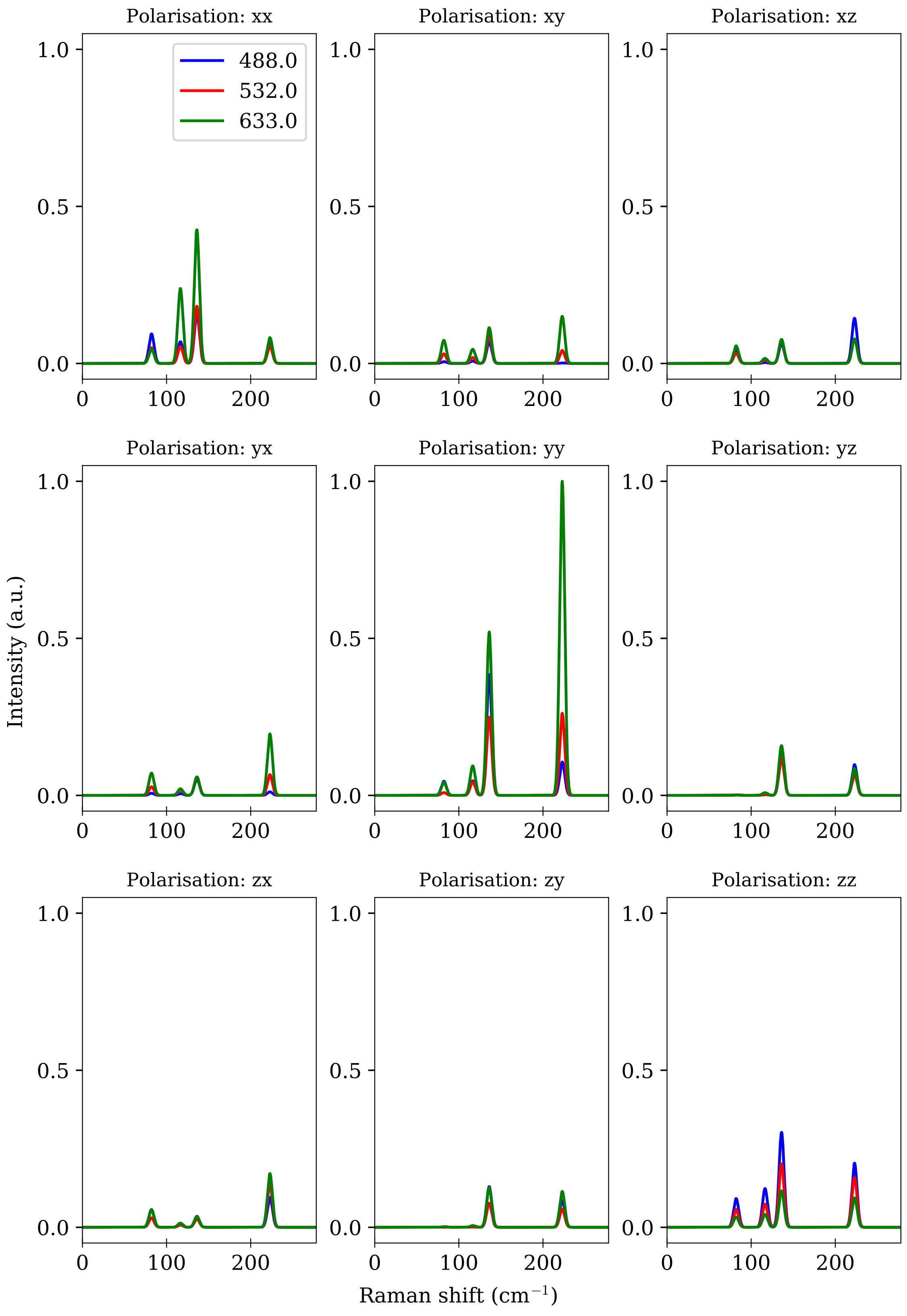

Supplementary Figure 46: Raman spectrum of AsITe with space group 156 and Wyckoff positions a-b-c-0.

AsITe, 156, a-c-0

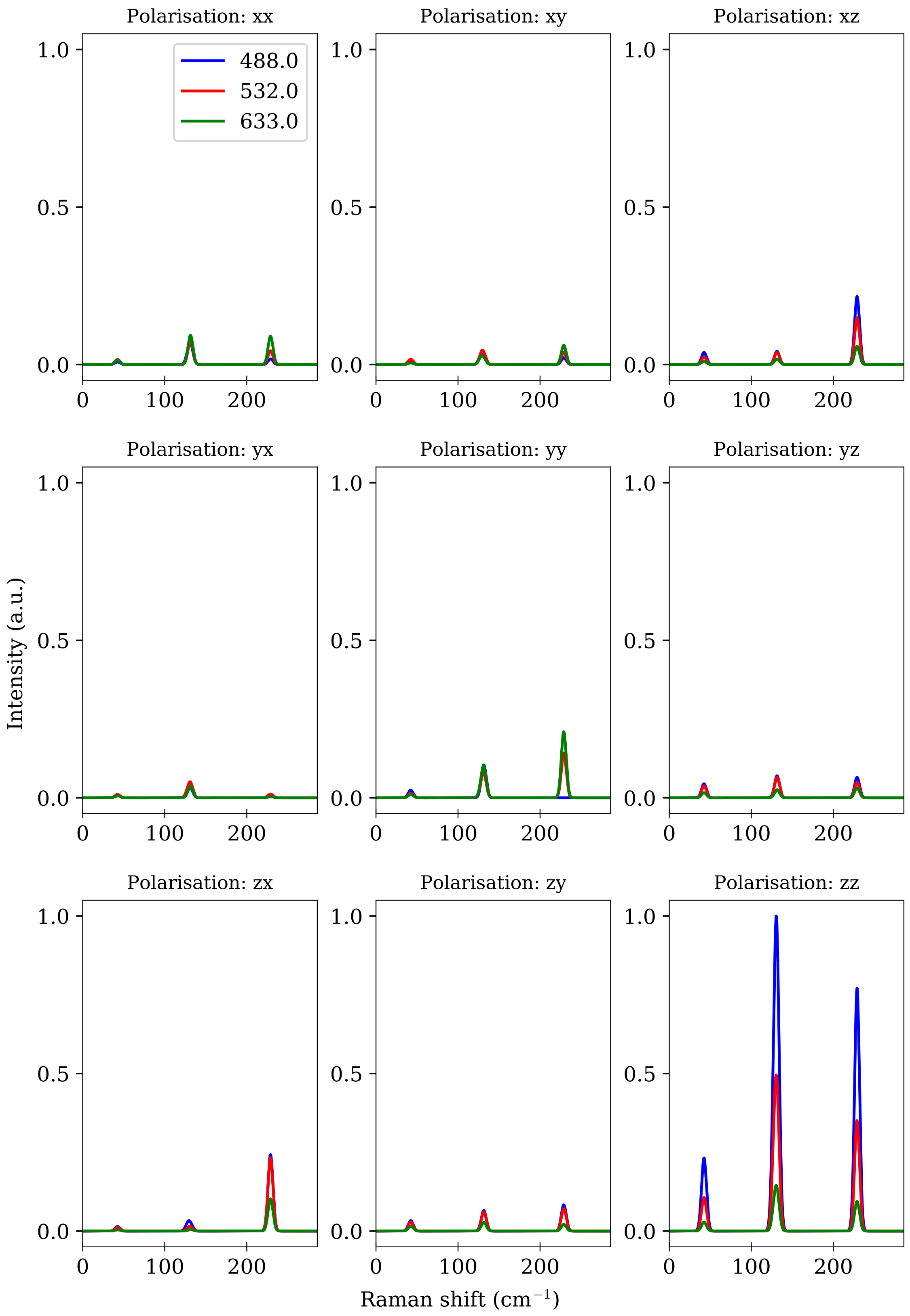

Supplementary Figure 47: Raman spectrum of AsITe with space group 156 and Wyckoff positions a-c-0.

AsS_2, 164, b-d-0

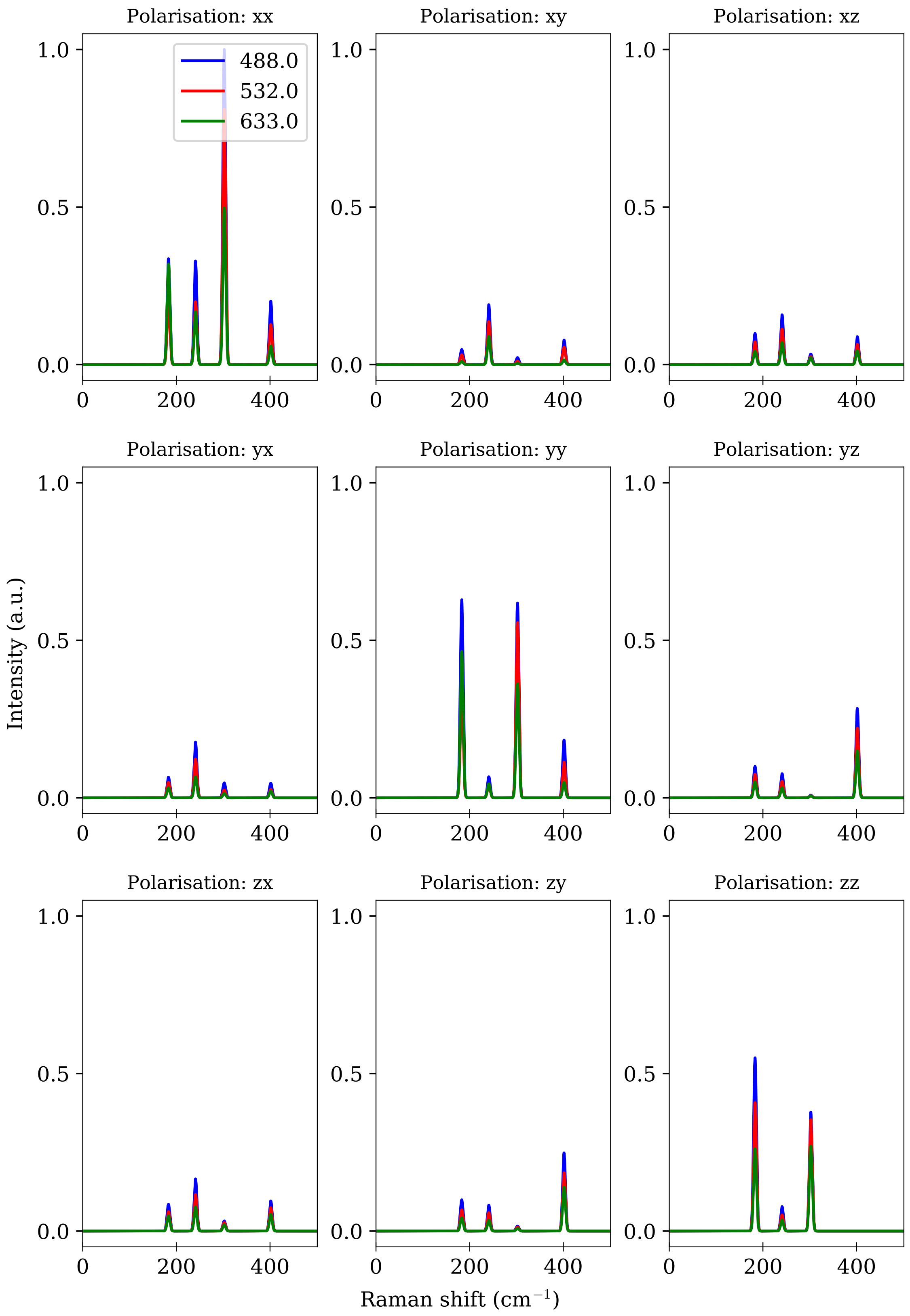

Supplementary Figure 48: Raman spectrum of AsS_2 with space group 164 and Wyckoff positions b-d-0.

$AsSe_2$, 164, b-d-0

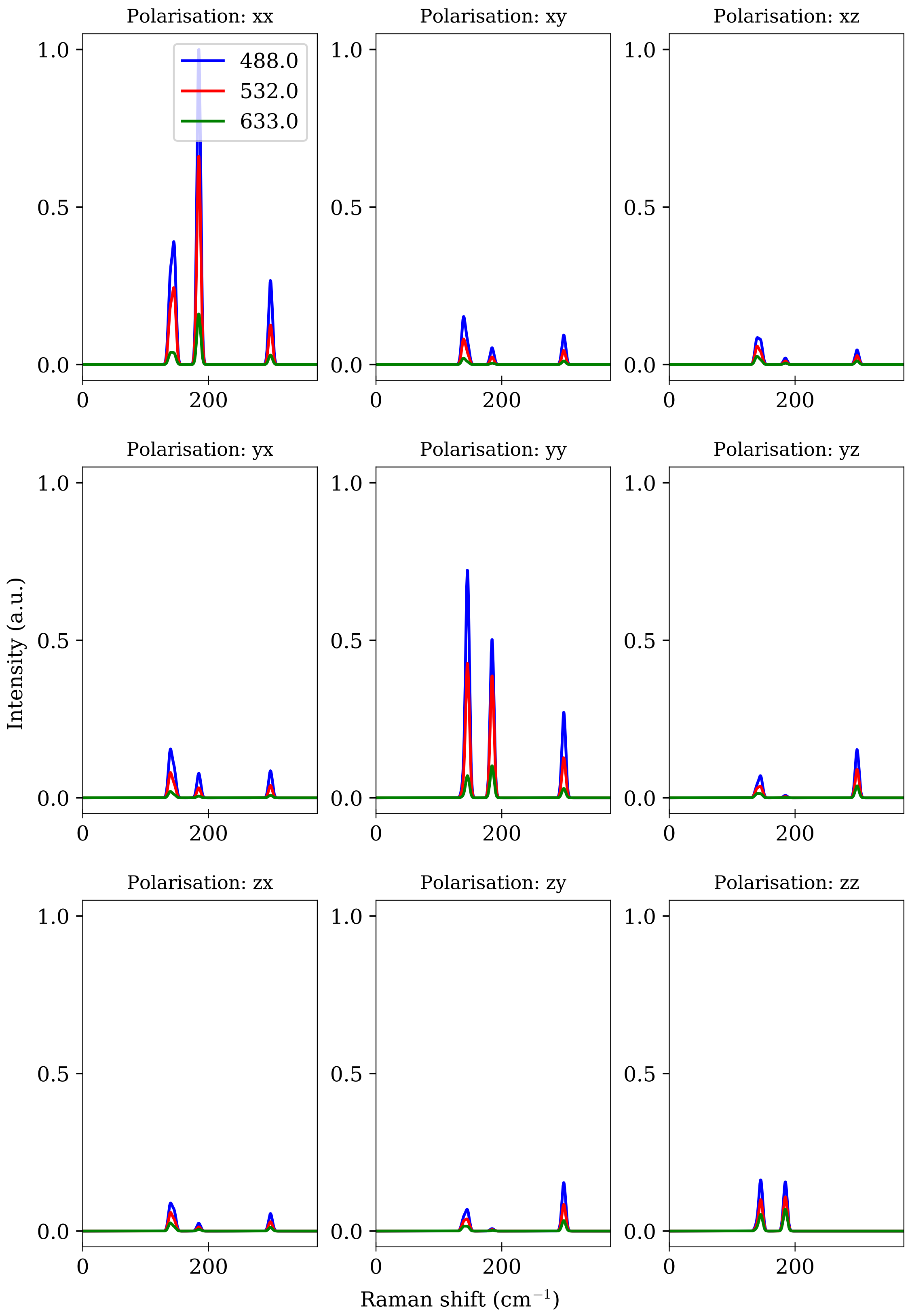

Supplementary Figure 49: Raman spectrum of $AsSe_2$ with space group 164 and Wyckoff positions b-d-0.

$AsTe_2$, 164, b-d-0

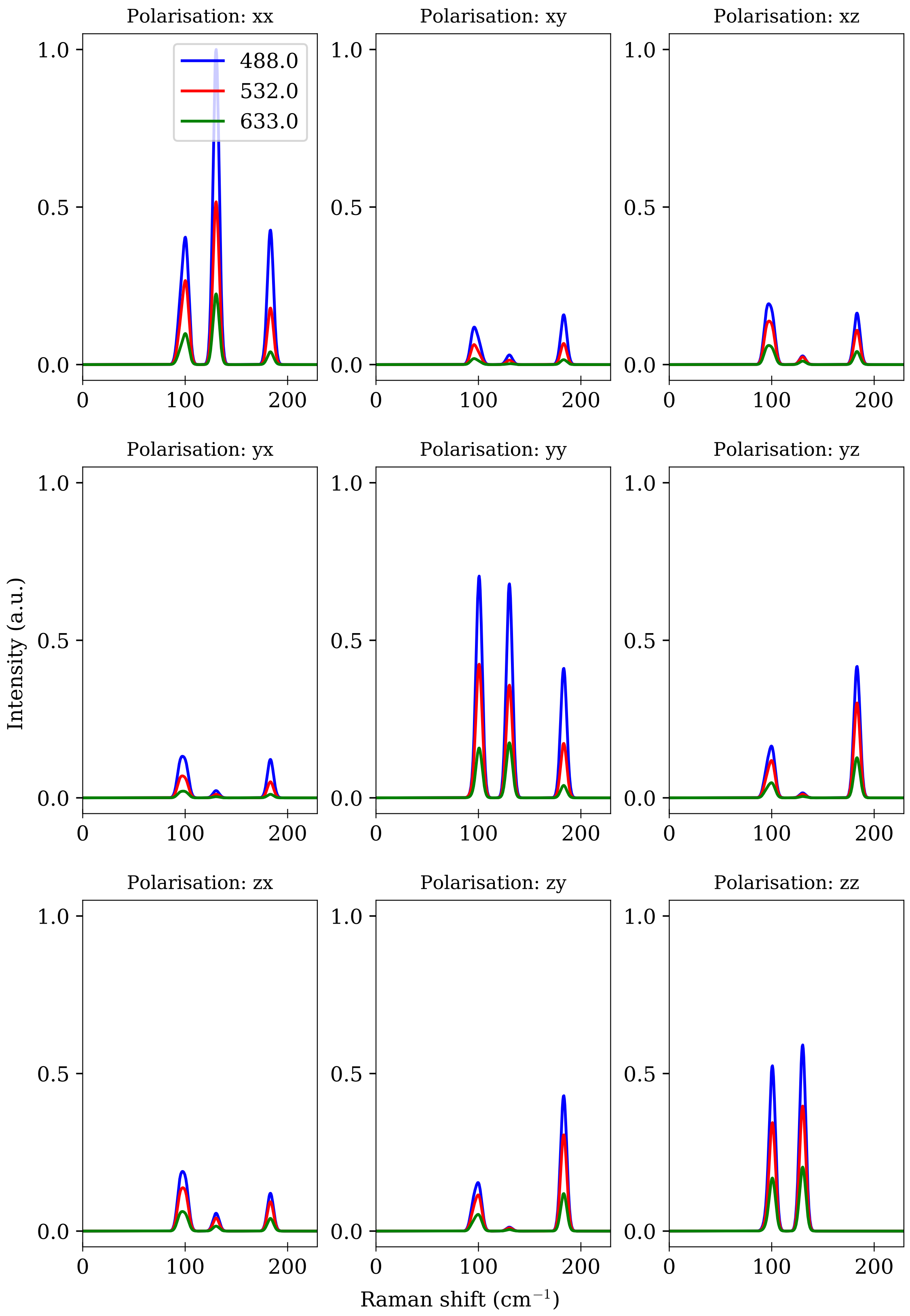

Supplementary Figure 50: Raman spectrum of $AsTe_2$ with space group 164 and Wyckoff positions b-d-0.

$AsTe_2$, 187, b-i-0

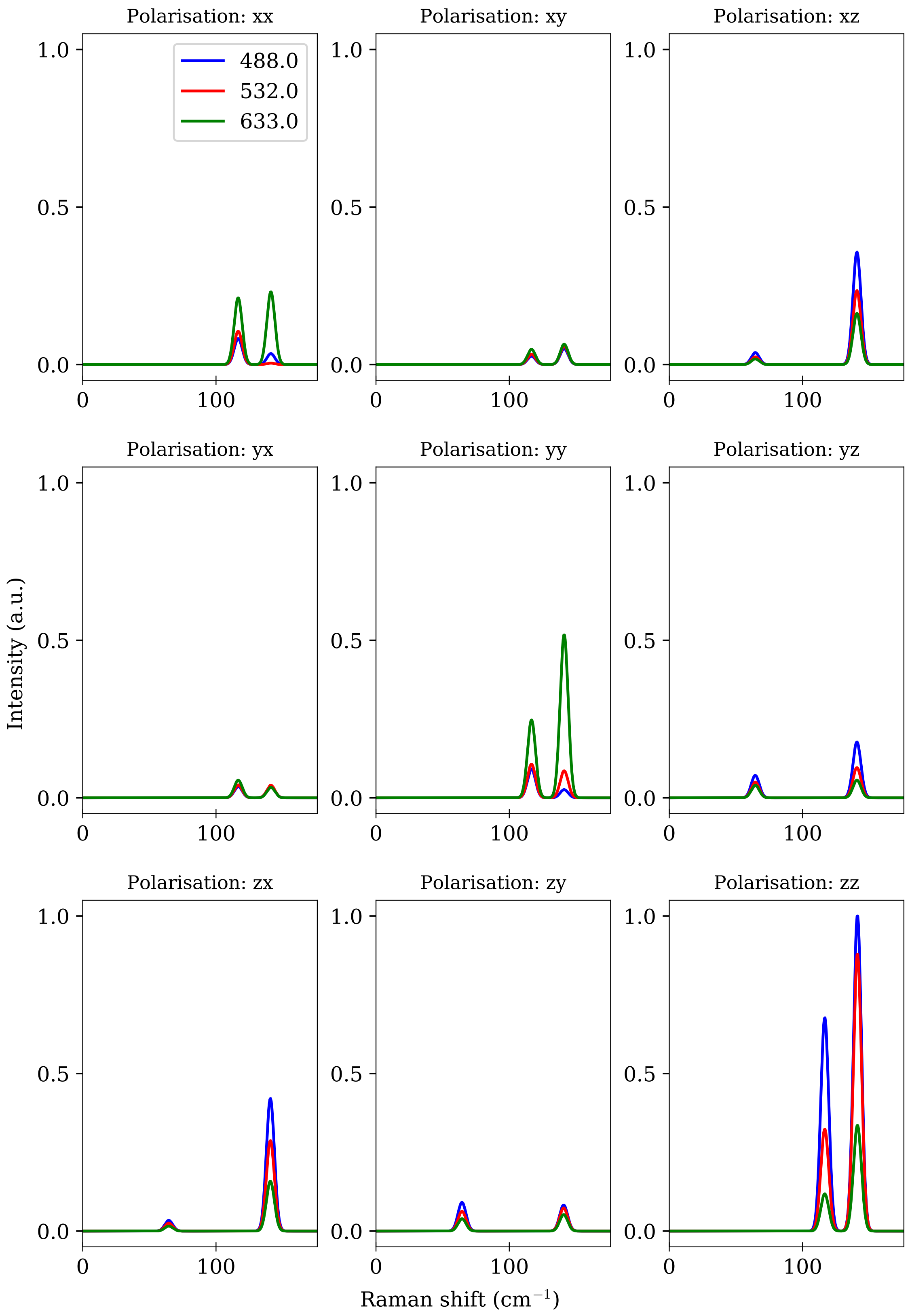

Supplementary Figure 51: Raman spectrum of $AsTe_2$ with space group 187 and Wyckoff positions b-i-0.

Au_2O_2, 10, f-g-m-0

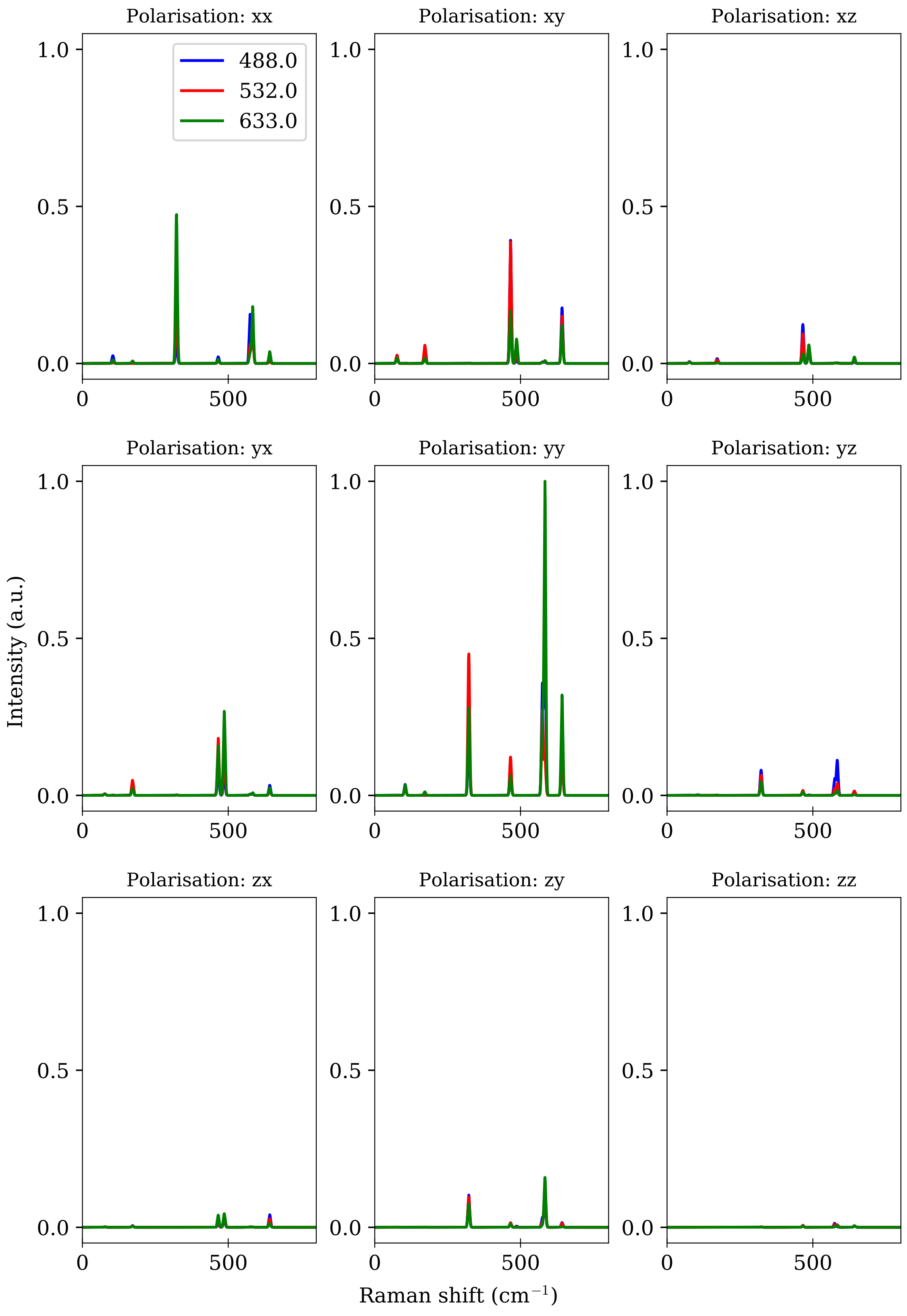

Supplementary Figure 52: Raman spectrum of Au_2O_2 with space group 10 and Wyckoff positions f-g-m-0.

Au_2S_2, 10, f-g-m-0

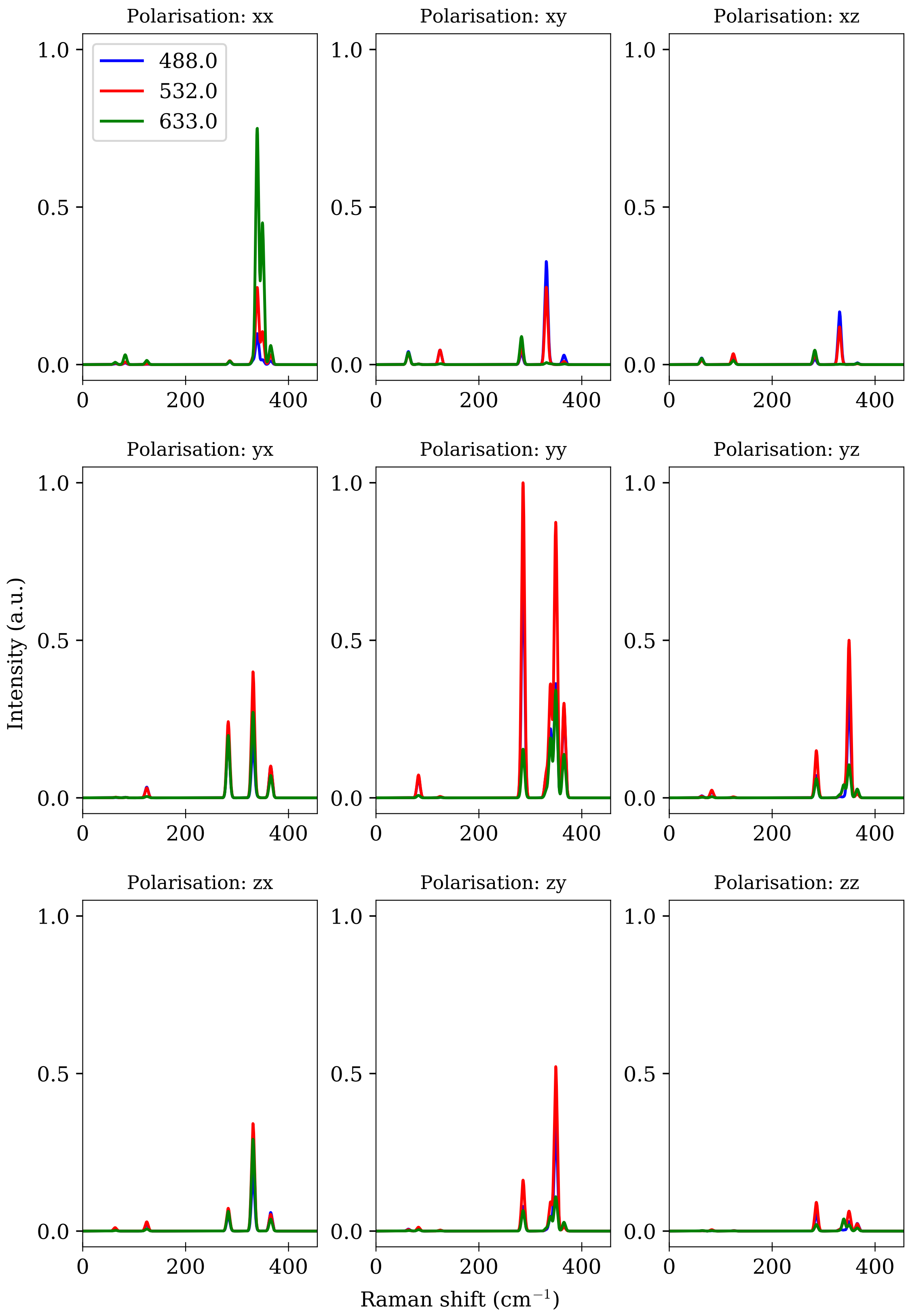

Supplementary Figure 53: Raman spectrum of Au_2S_2 with space group 10 and Wyckoff positions f-g-m-0.

Au_2Se_2, 10, f-g-m-0

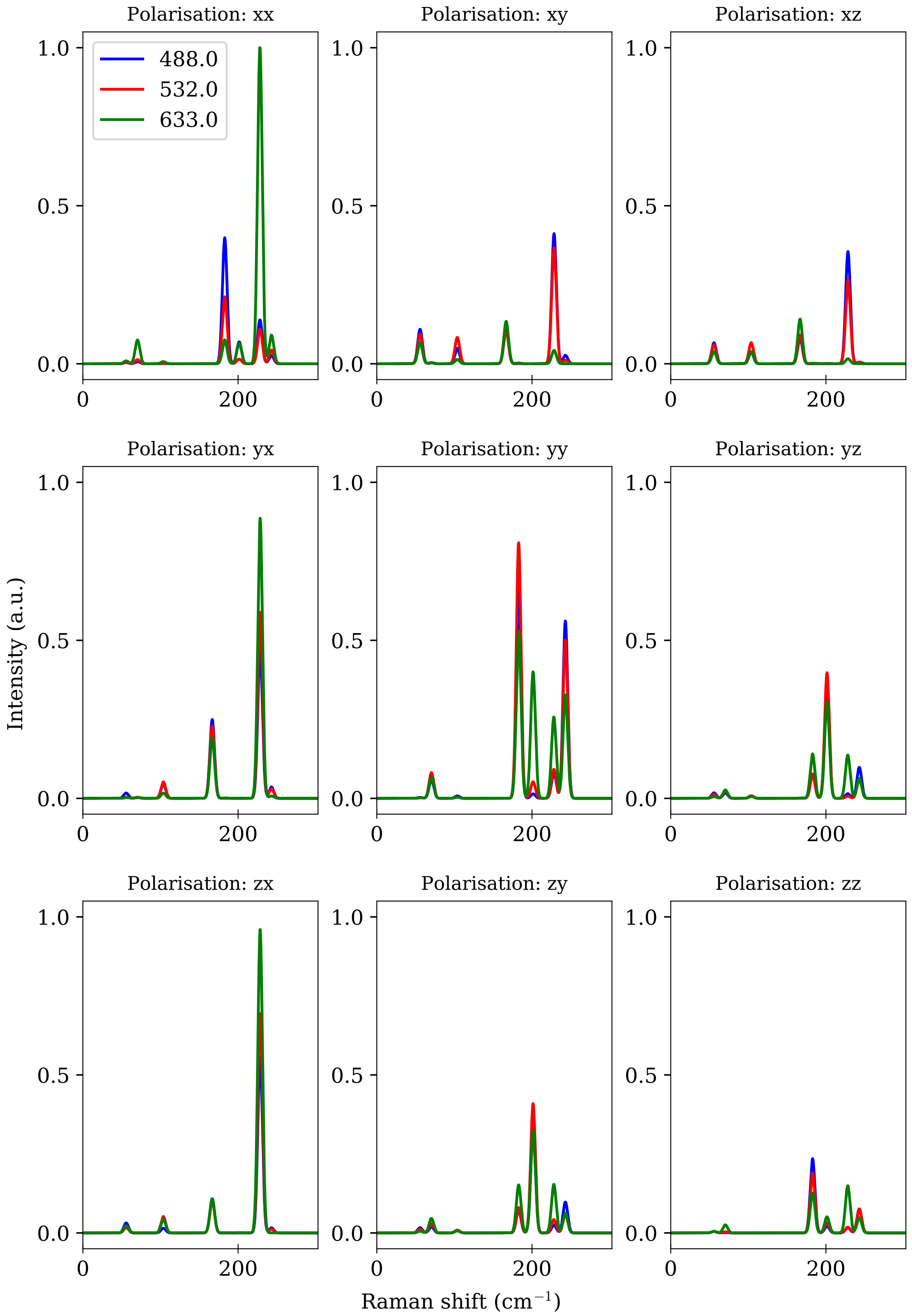

Supplementary Figure 54: Raman spectrum of Au_2Se_2 with space group 10 and Wyckoff positions f-g-m-0.

Au_2Te_2, 6, a-b-0

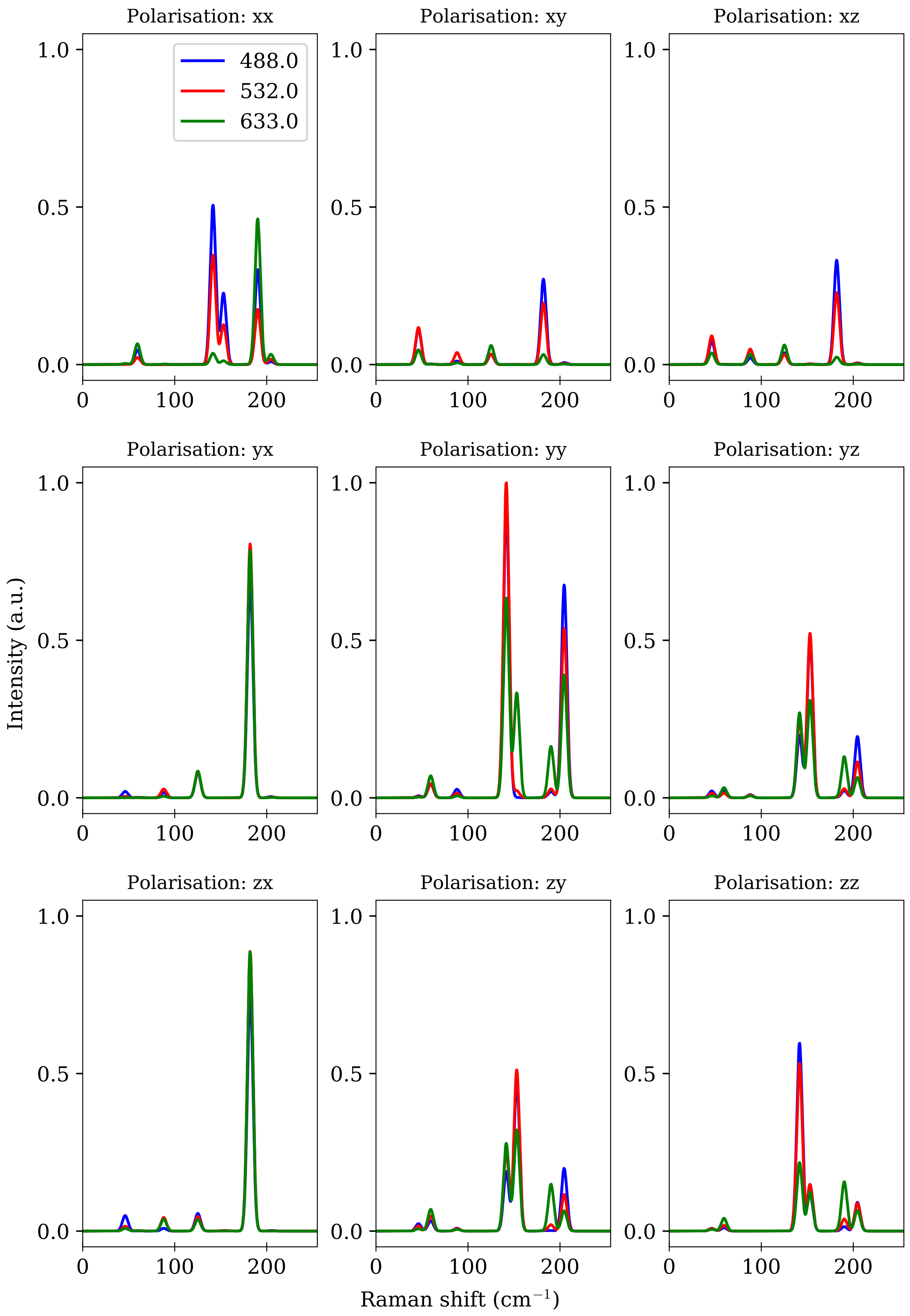

Supplementary Figure 55: Raman spectrum of Au_2Te_2 with space group 6 and Wyckoff positions a-b-0.

AuSe$_2$, 187, b-i-0

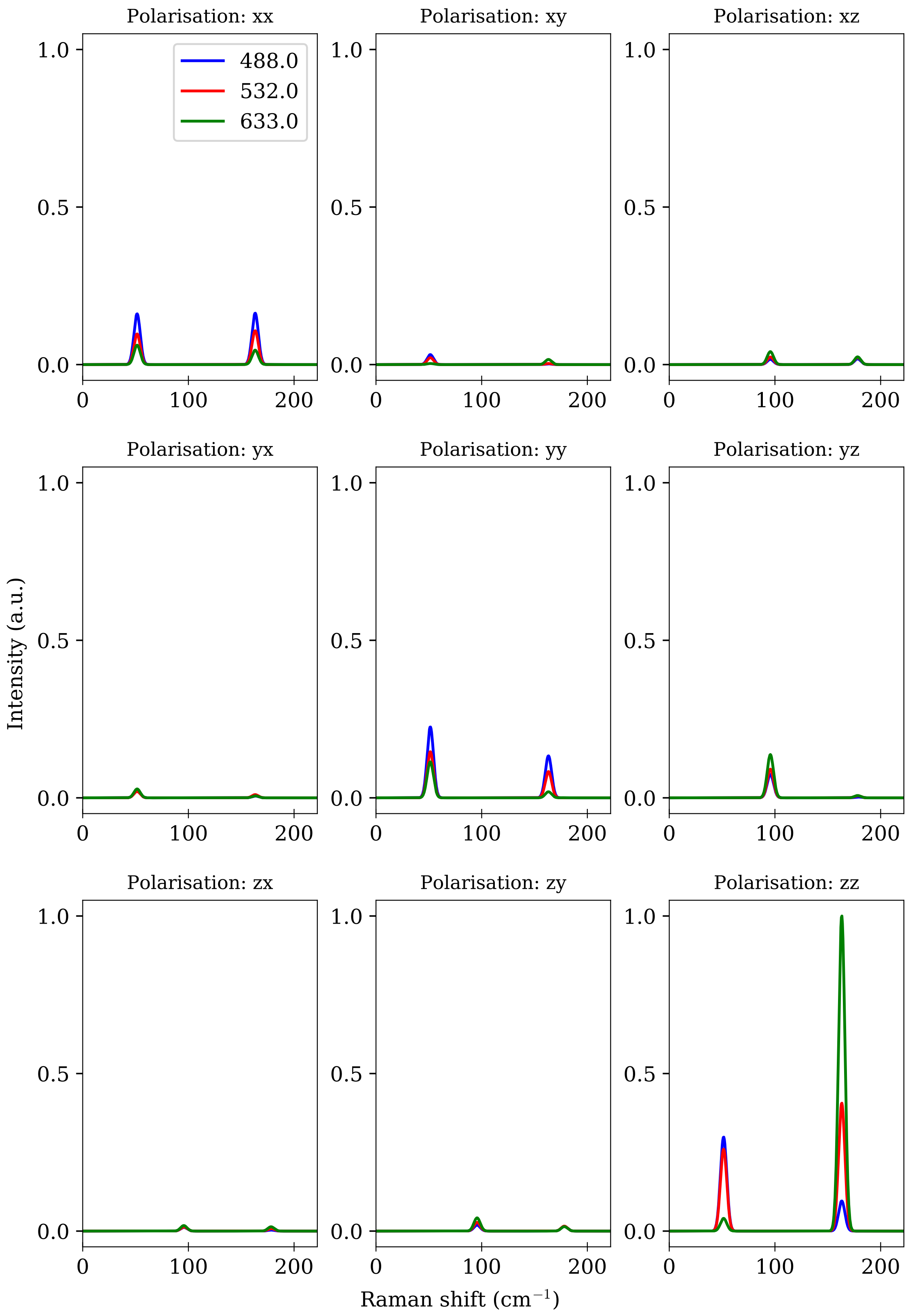

Supplementary Figure 56: Raman spectrum of AuSe$_2$ with space group 187 and Wyckoff positions b-i-0.

$AuTe_2$, 164, b-d-0

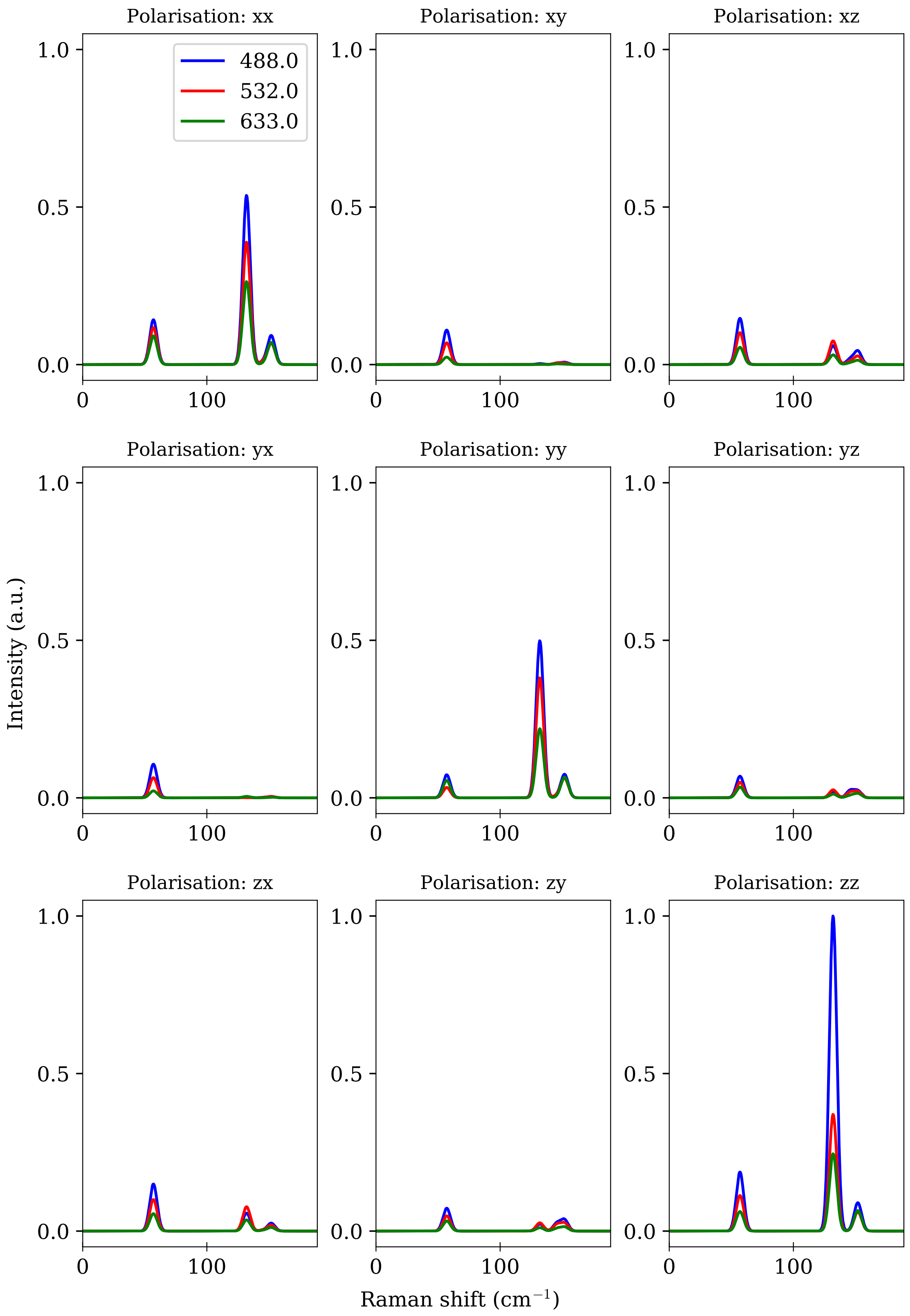

Supplementary Figure 57: Raman spectrum of $AuTe_2$ with space group 164 and Wyckoff positions b-d-0.

$AuTe_2$, 187, b-i-0

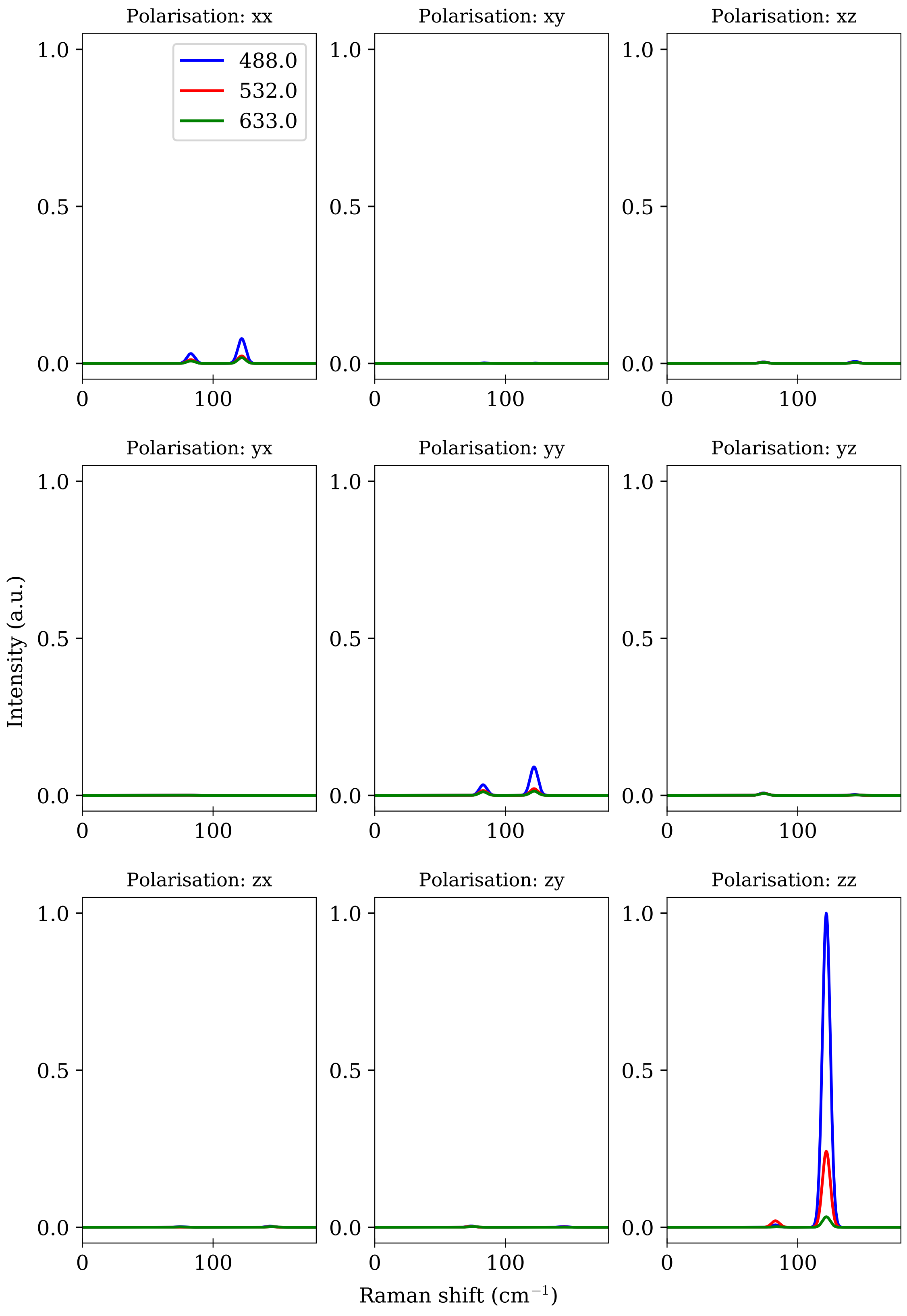

Supplementary Figure 58: Raman spectrum of $AuTe_2$ with space group 187 and Wyckoff positions b-i-0.

$BaBr_2$, 164, b-d-0

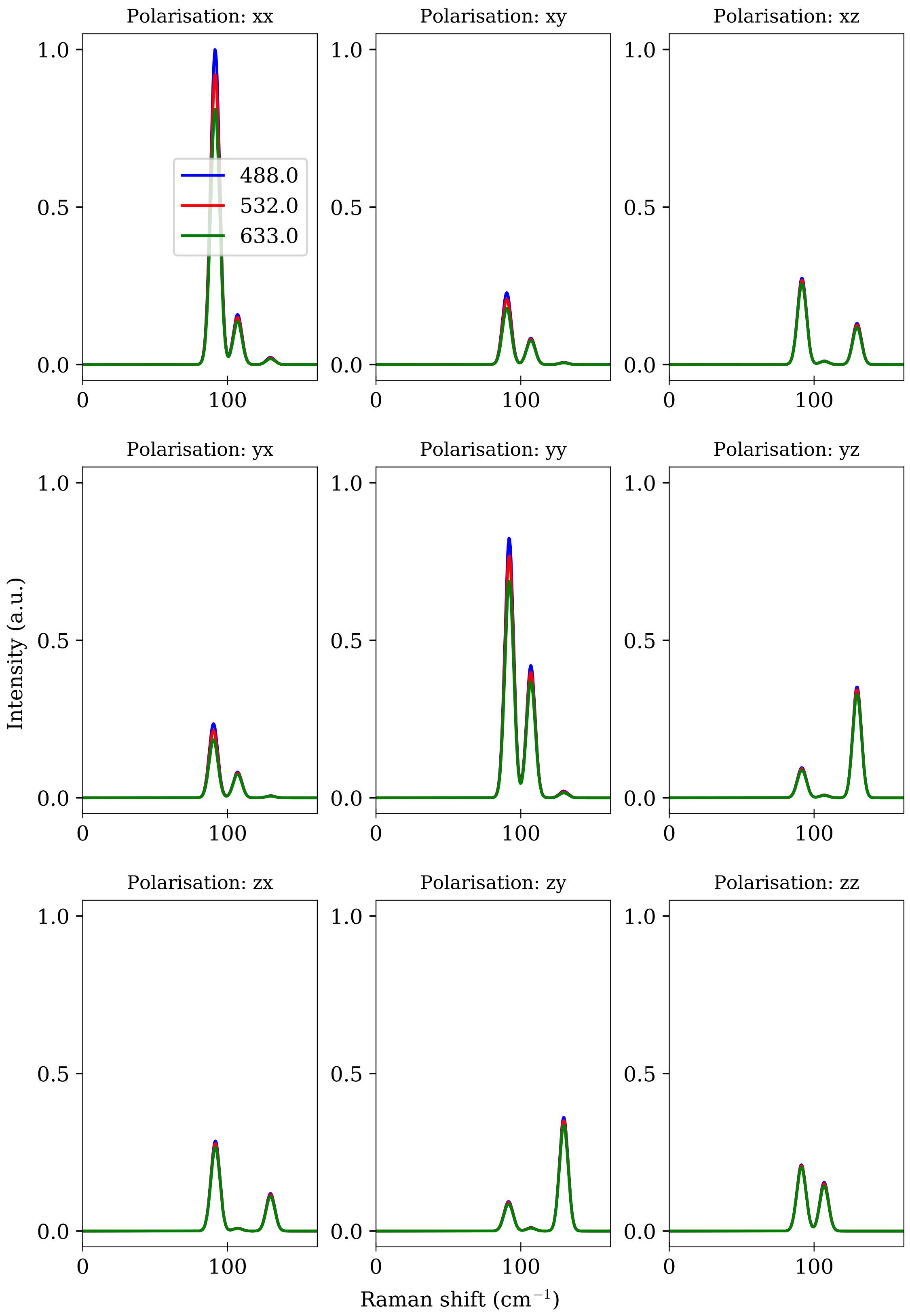

Supplementary Figure 59: Raman spectrum of $BaBr_2$ with space group 164 and Wyckoff positions b-d-0.

$BaCl_2$, 164, b-d-0

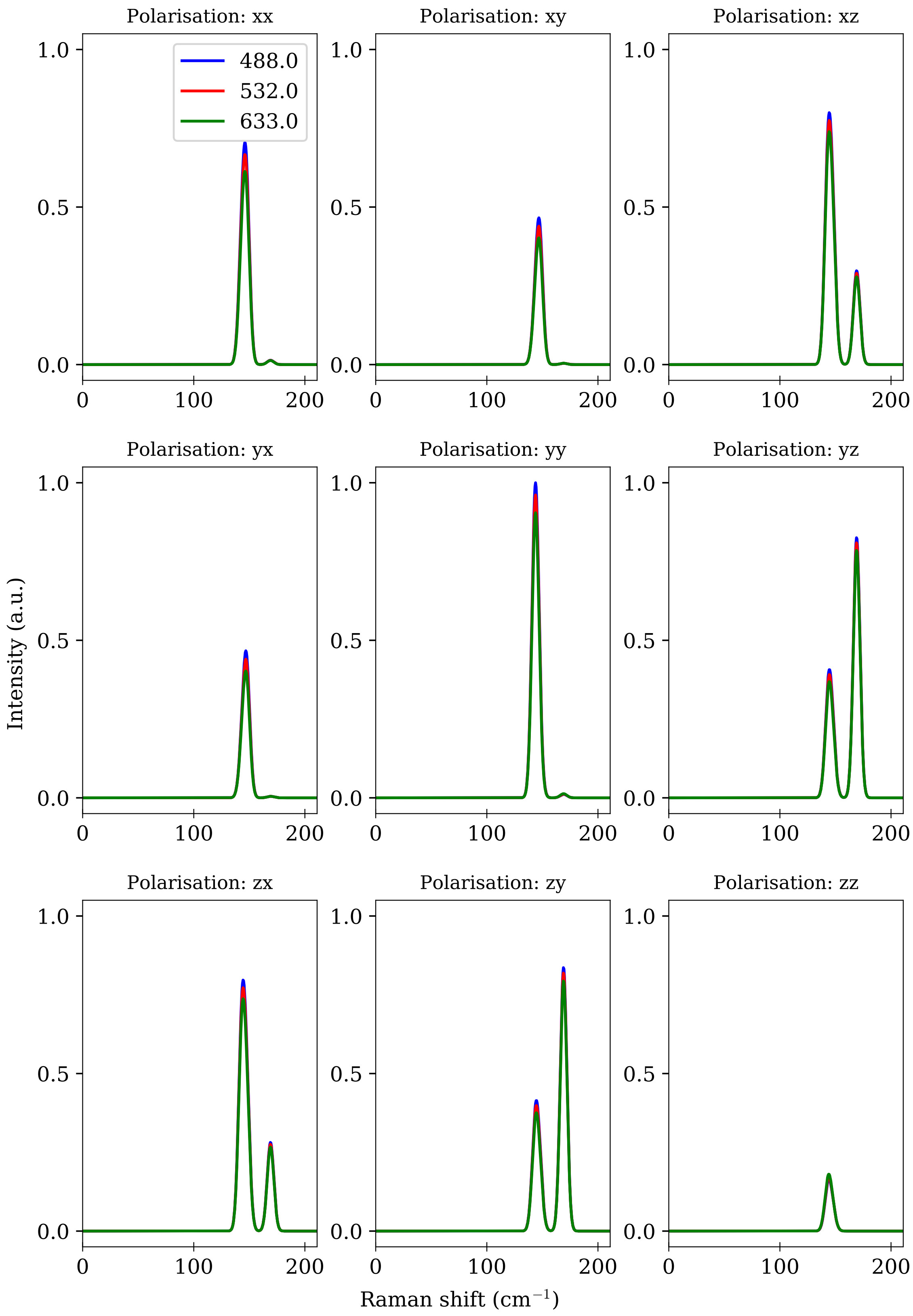

Supplementary Figure 60: Raman spectrum of $BaCl_2$ with space group 164 and Wyckoff positions b-d-0.

Bi_2Hf, 164, b-d-0

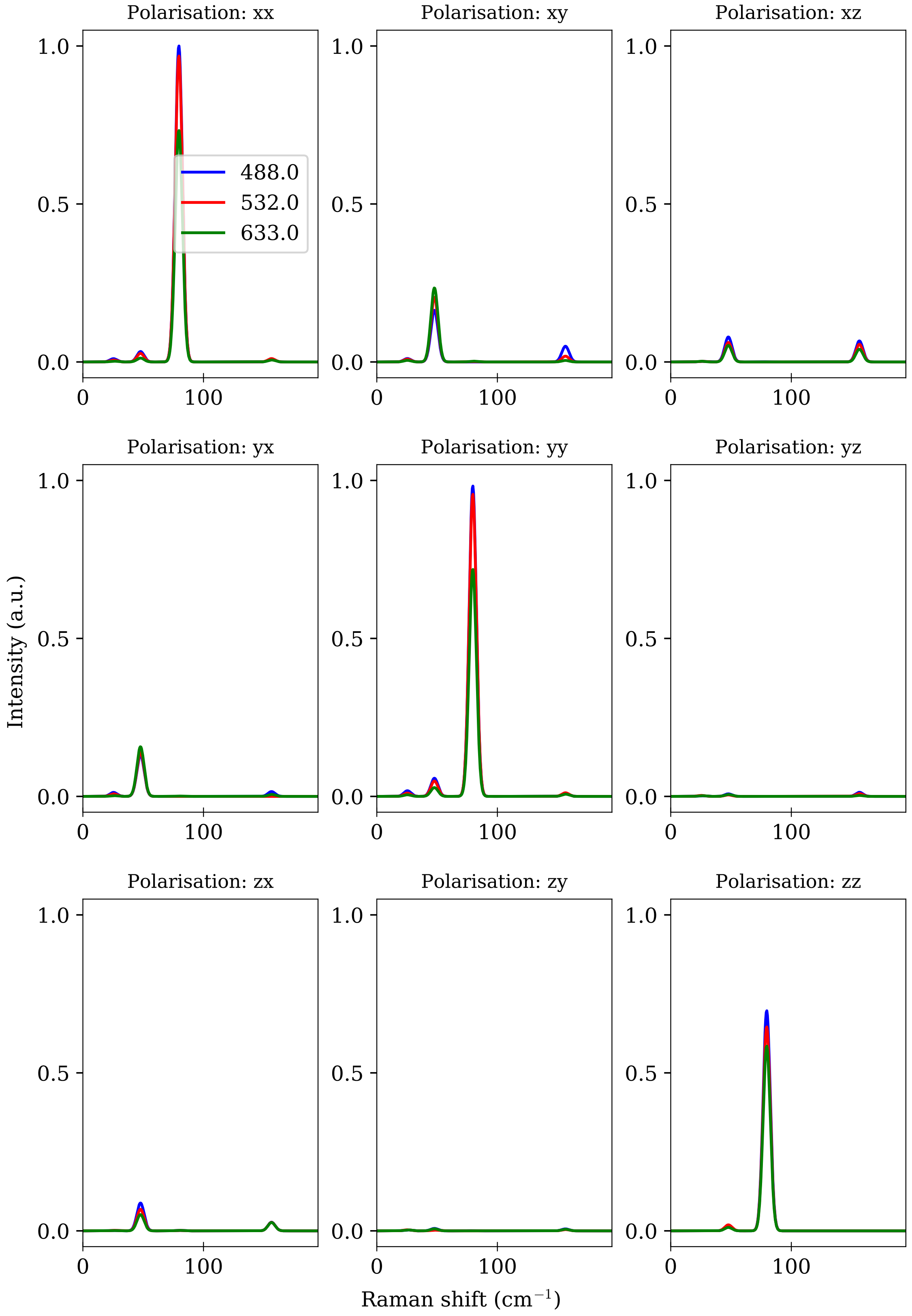

Supplementary Figure 61: Raman spectrum of Bi_2Hf with space group 164 and Wyckoff positions b-d-0.

Bi_2Hf, 187, b-i-0

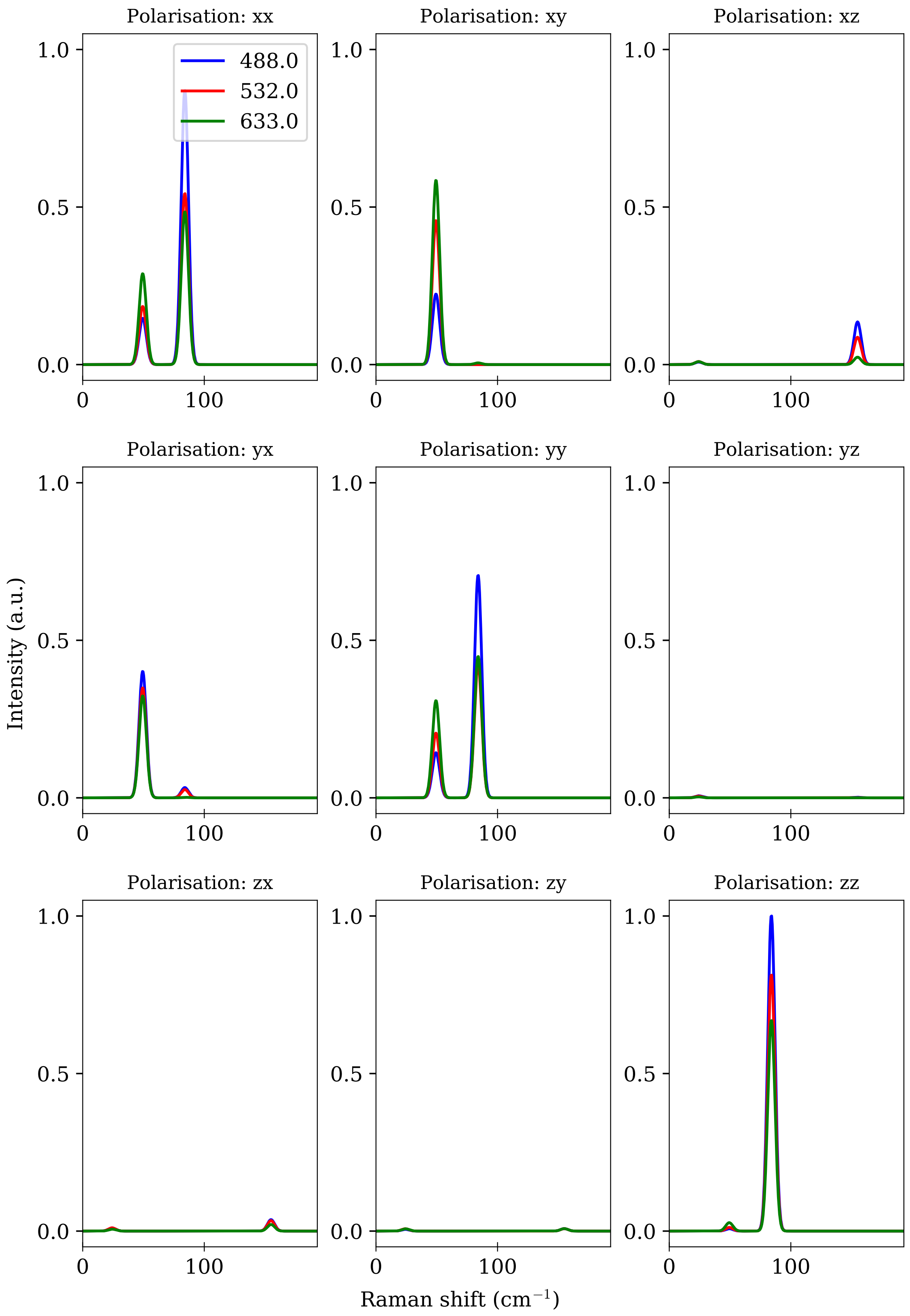

Supplementary Figure 62: Raman spectrum of Bi_2Hf with space group 187 and Wyckoff positions b-i-0.

Bi_2Mo, 164, b-d-0

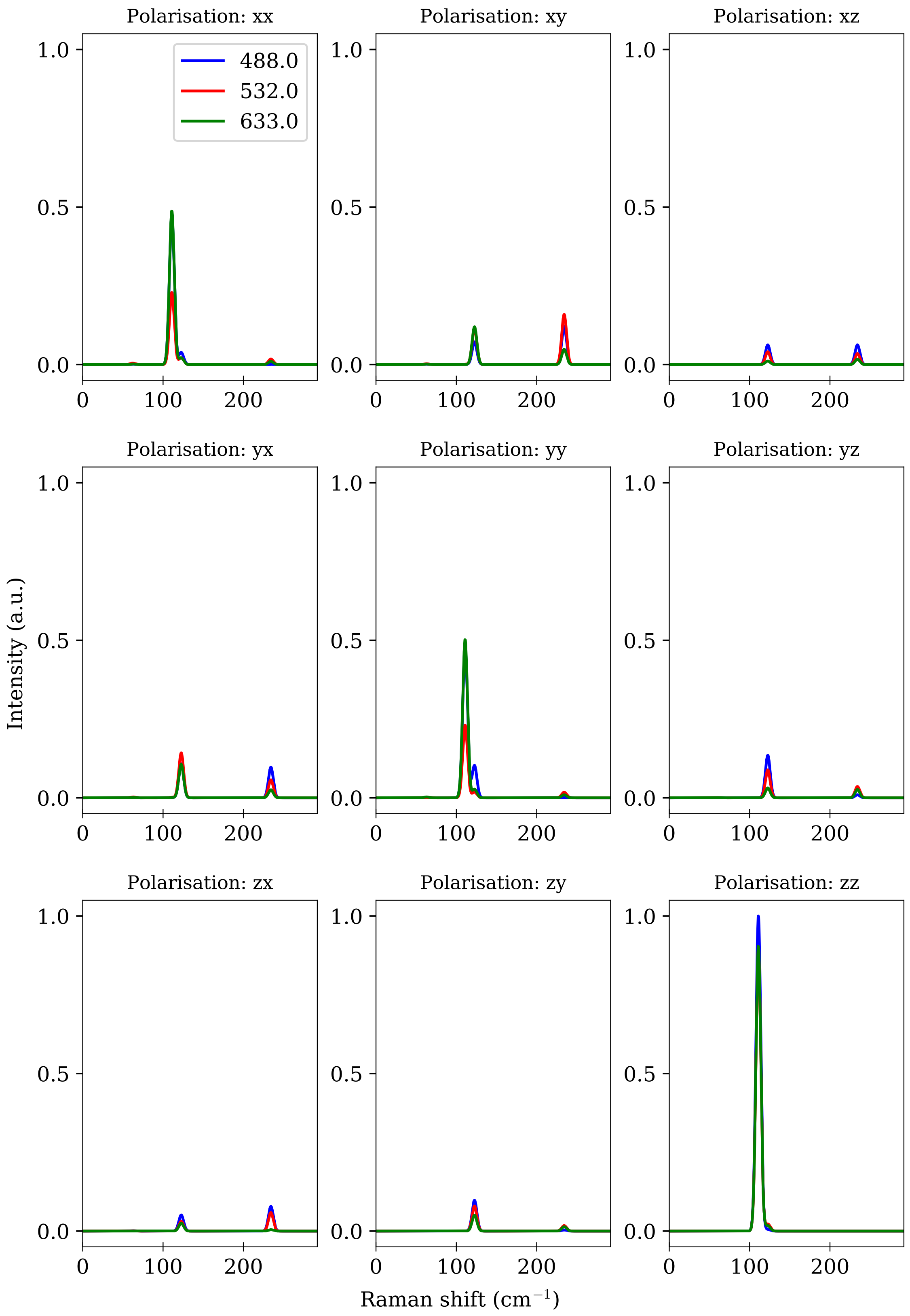

Supplementary Figure 63: Raman spectrum of Bi_2Mo with space group 164 and Wyckoff positions b-d-0.

Bi_2Nb, 164, b-d-0

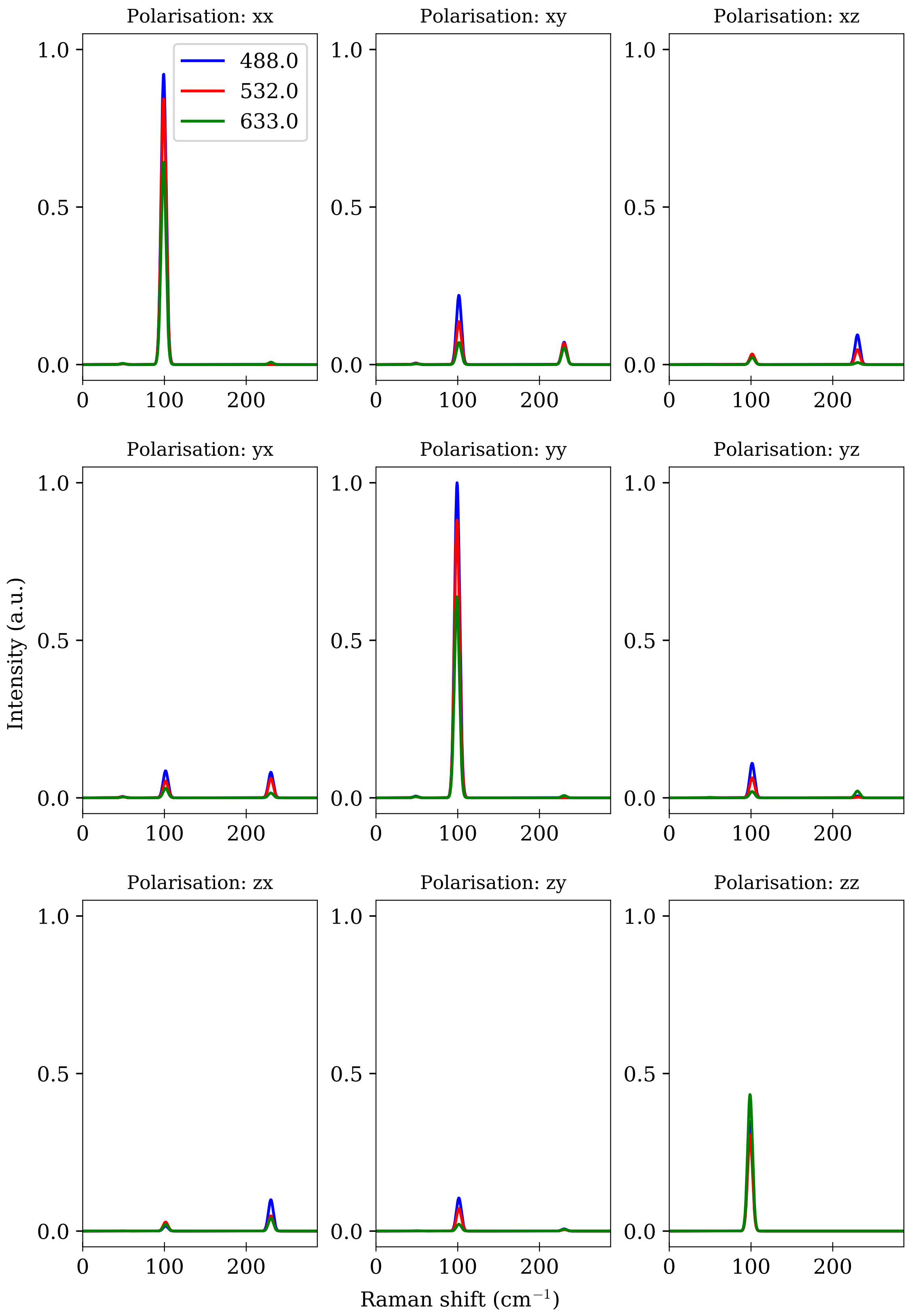

Supplementary Figure 64: Raman spectrum of Bi_2Nb with space group 164 and Wyckoff positions b-d-0.

Bi_2Nb, 187, a-i-0

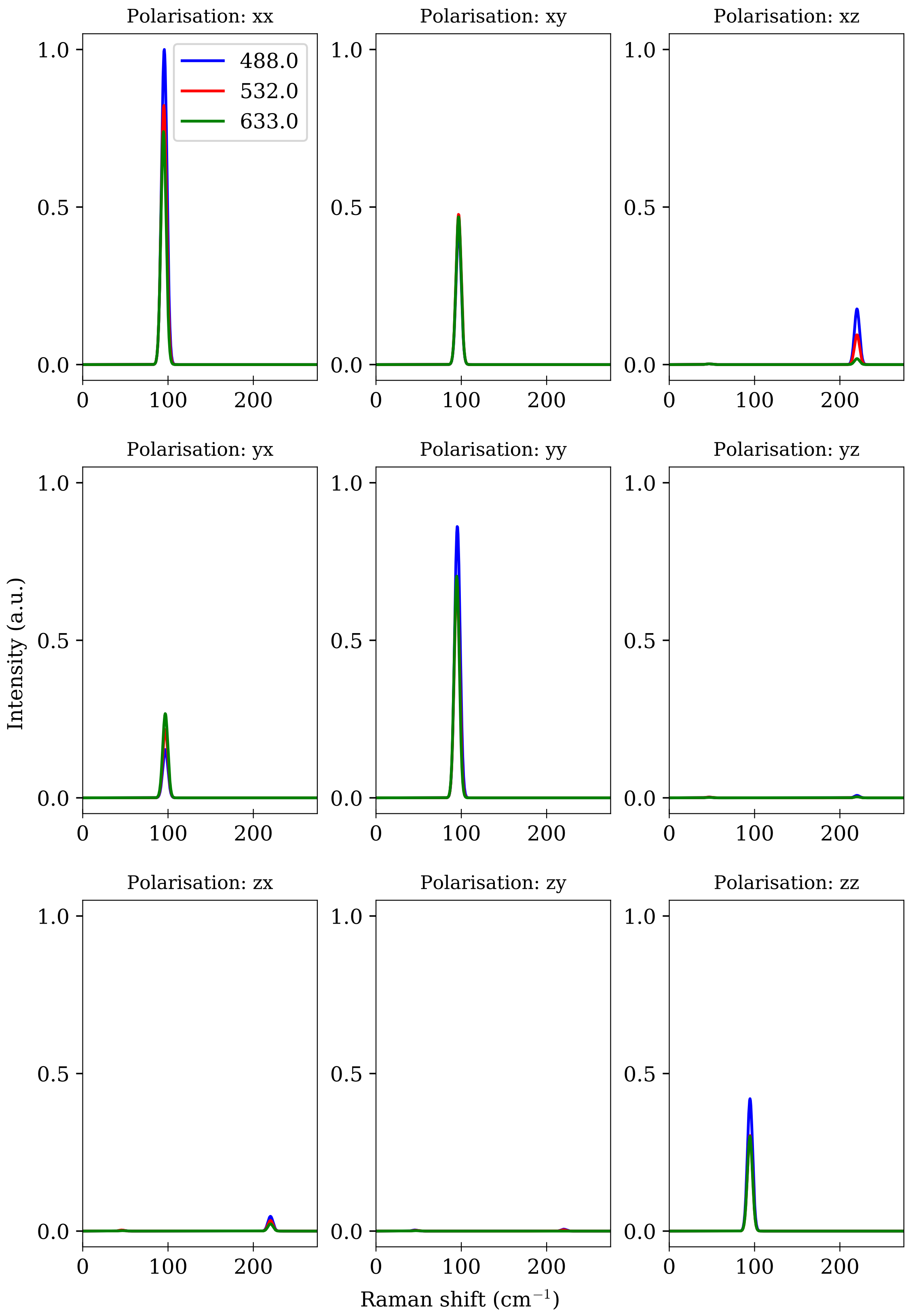

Supplementary Figure 65: Raman spectrum of Bi_2Nb with space group 187 and Wyckoff positions a-i-0.

Bi_2Ta, 164, b-d-0

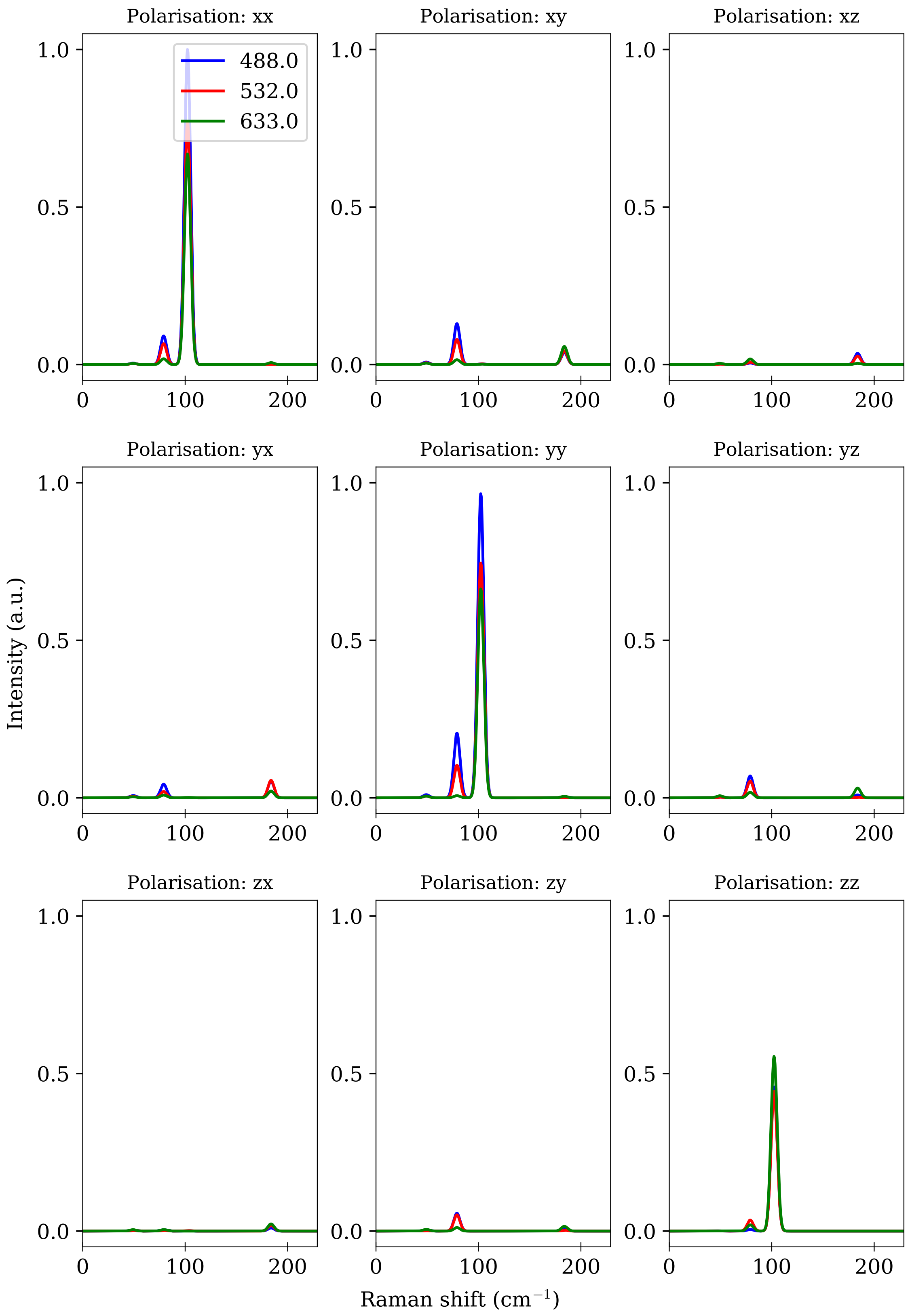

Supplementary Figure 66: Raman spectrum of Bi_2Ta with space group 164 and Wyckoff positions b-d-0.

Bi_2Ti, 164, b-d-0

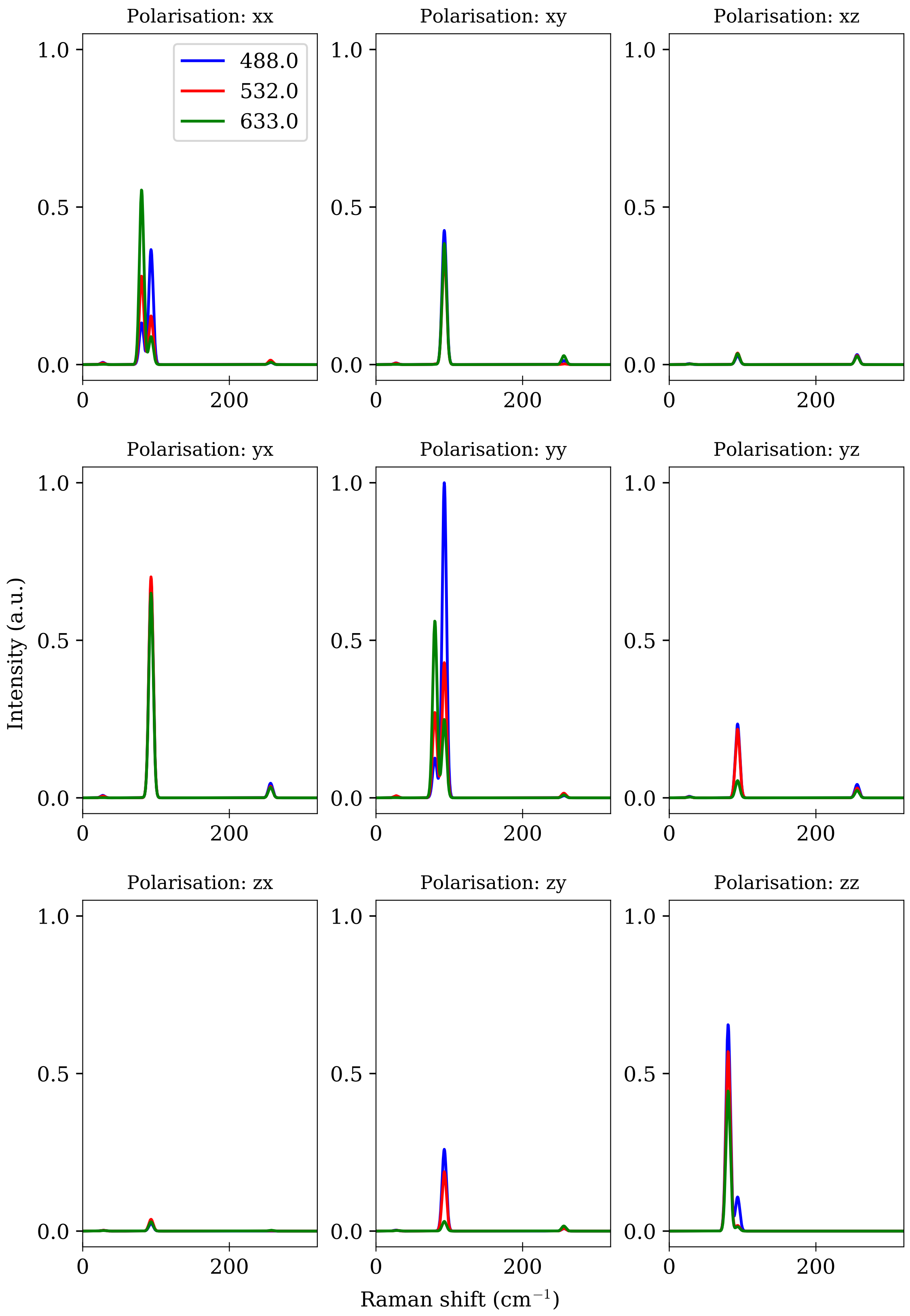

Supplementary Figure 67: Raman spectrum of Bi_2Ti with space group 164 and Wyckoff positions b-d-0.

Bi_2Ti, 187, b-i-0

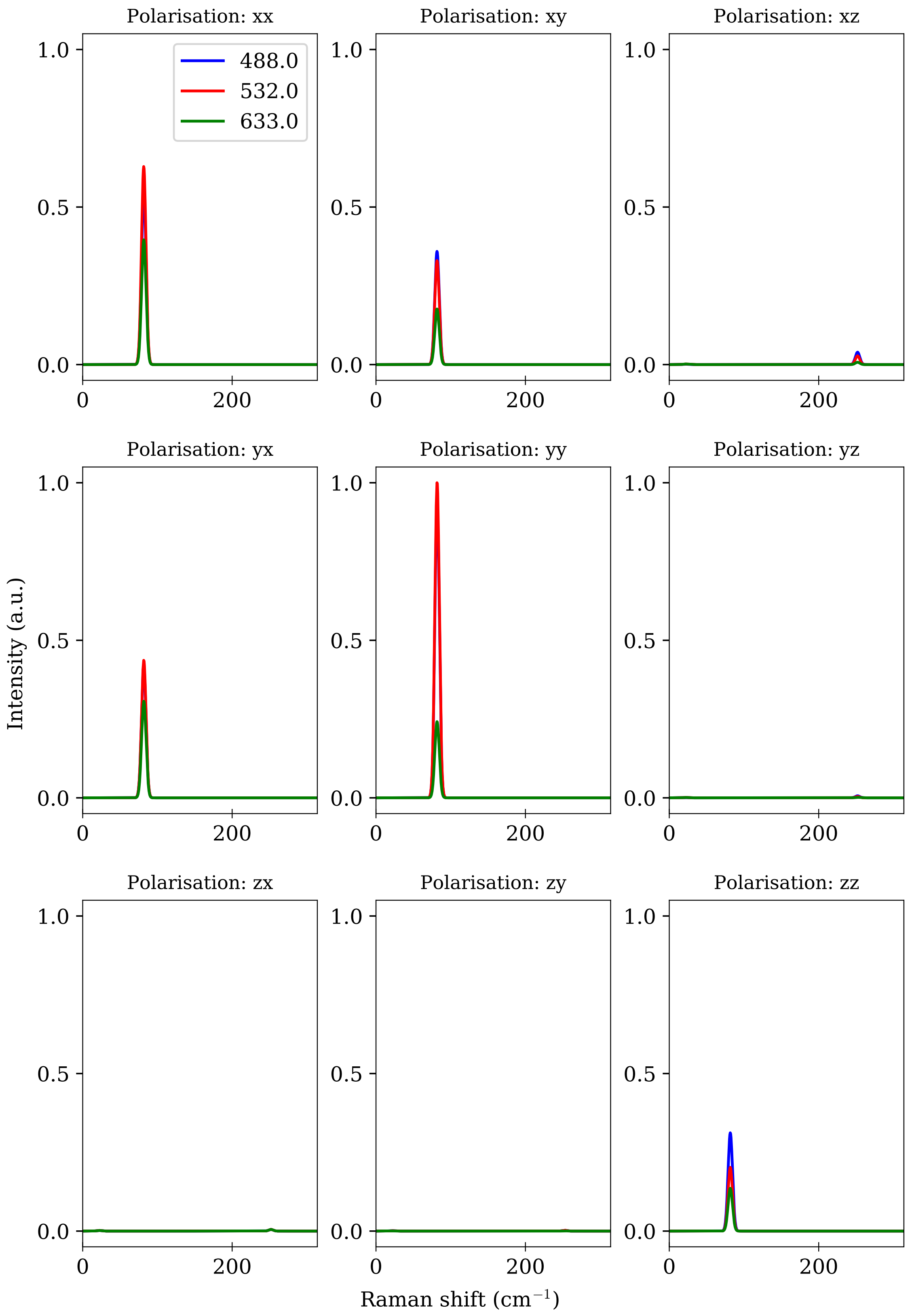

Supplementary Figure 68: Raman spectrum of Bi_2Ti with space group 187 and Wyckoff positions b-i-0.

Bi_2V, 164, b-d-0

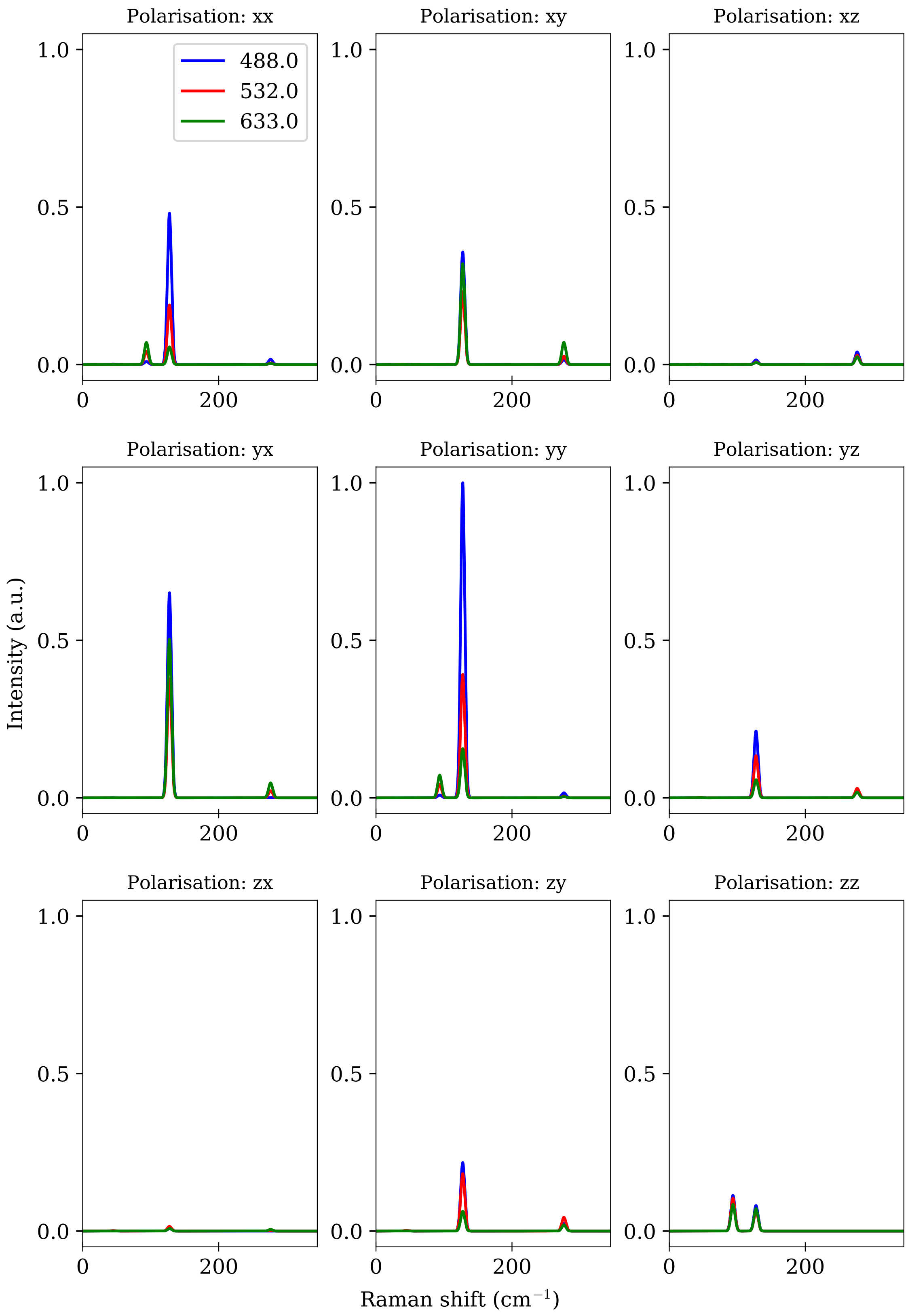

Supplementary Figure 69: Raman spectrum of Bi_2V with space group 164 and Wyckoff positions b-d-0.

Bi_2W, 164, b-d-0

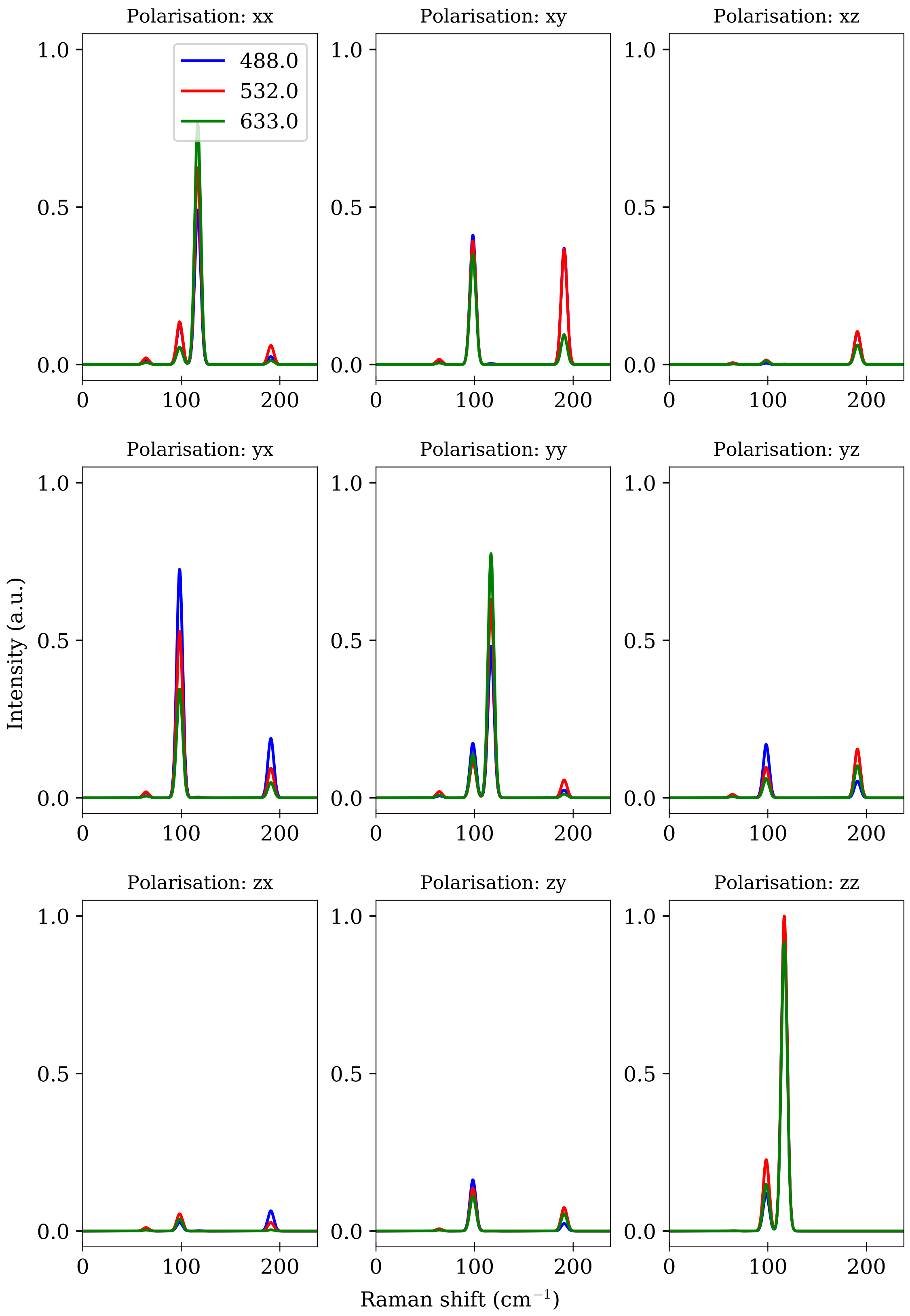

Supplementary Figure 70: Raman spectrum of Bi_2W with space group 164 and Wyckoff positions b-d-0.

Bi_2Zr, 164, b-d-0

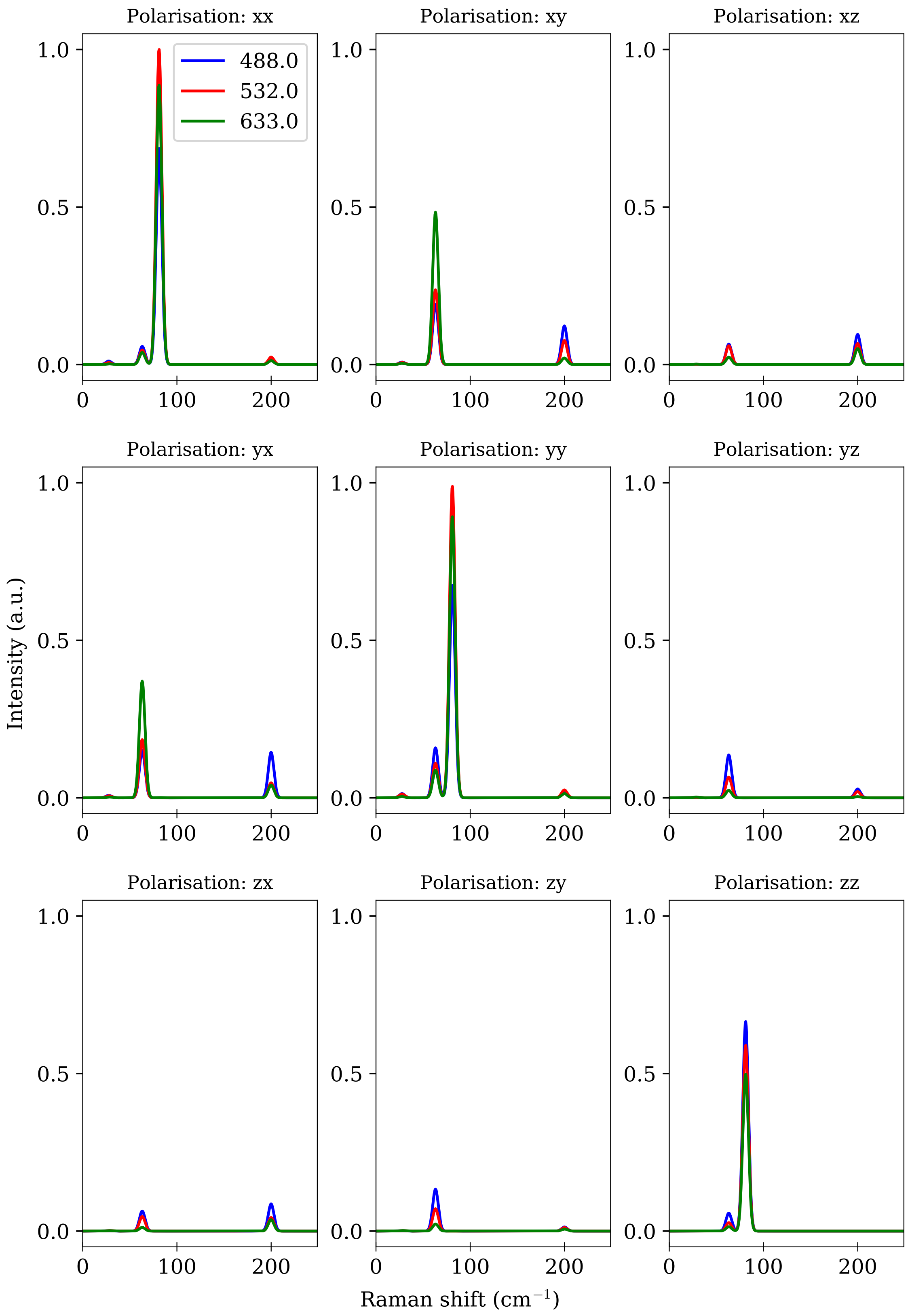

Supplementary Figure 71: Raman spectrum of Bi_2Zr with space group 164 and Wyckoff positions b-d-0.

Bi_2Zr, 187, b-i-0

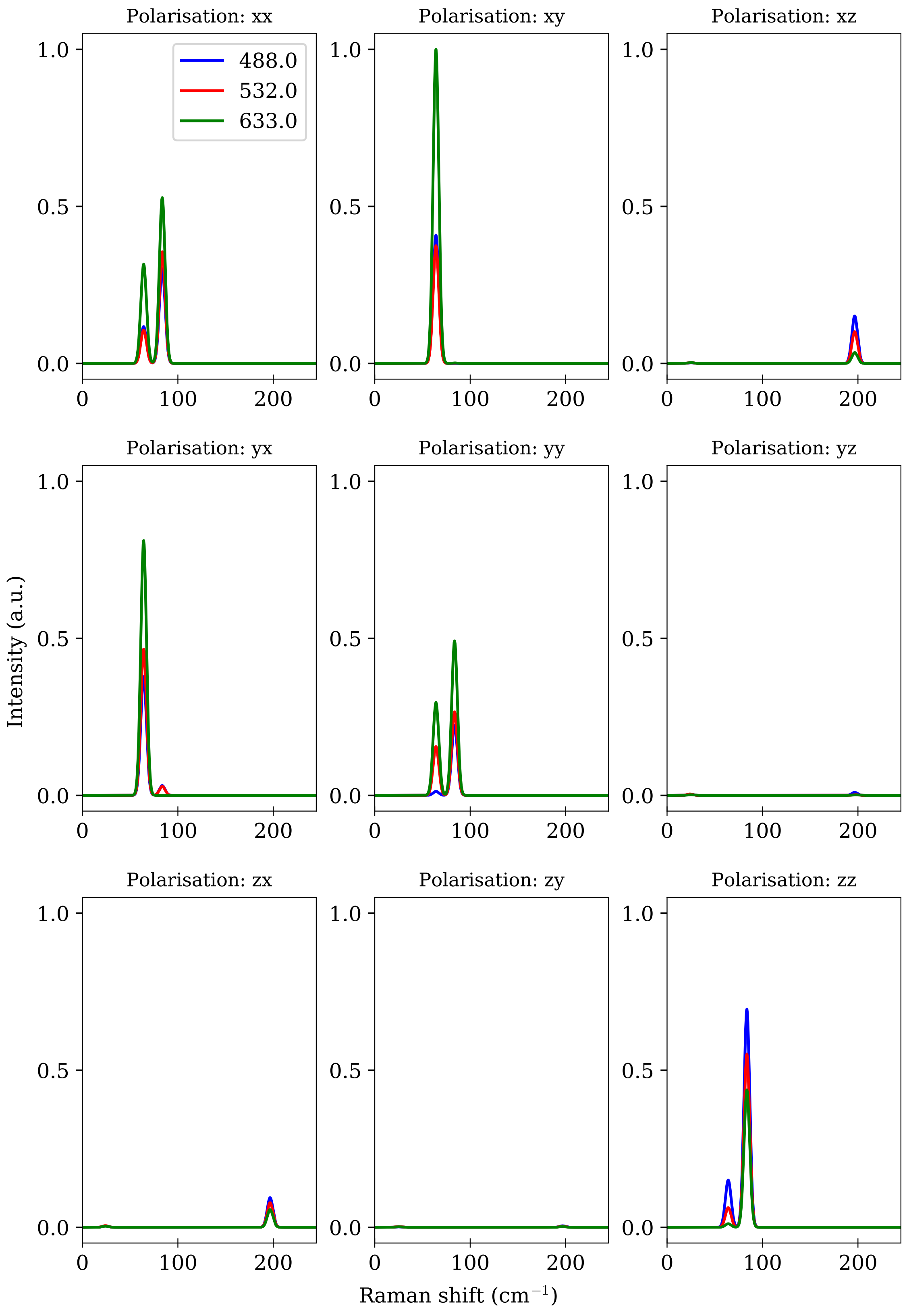

Supplementary Figure 72: Raman spectrum of Bi_2Zr with space group 187 and Wyckoff positions b-i-0.

BiBrSe, 156, a-b-c-0

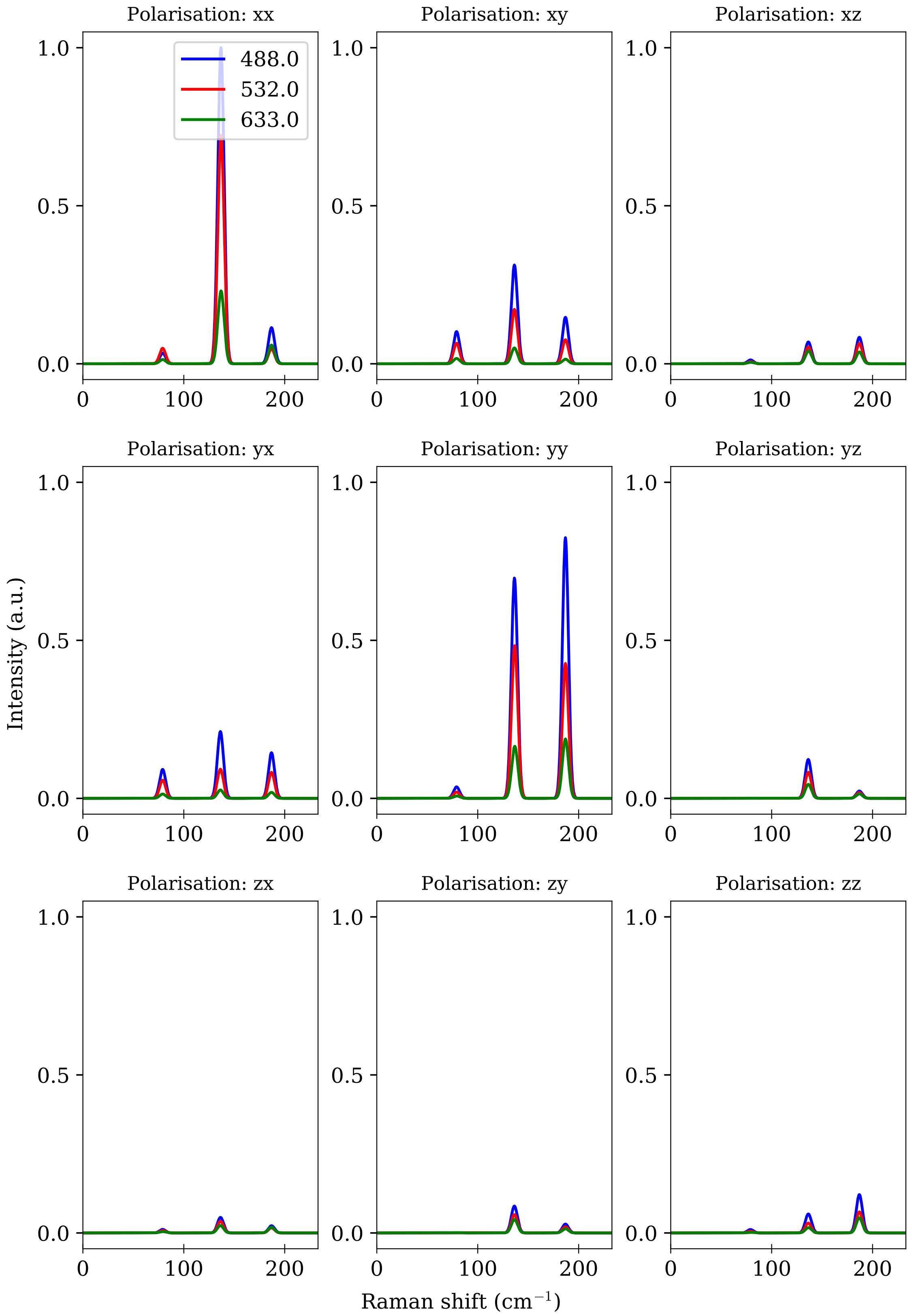

Supplementary Figure 73: Raman spectrum of BiBrSe with space group 156 and Wyckoff positions a-b-c-0.

BiBrSe, 156, a-c-0

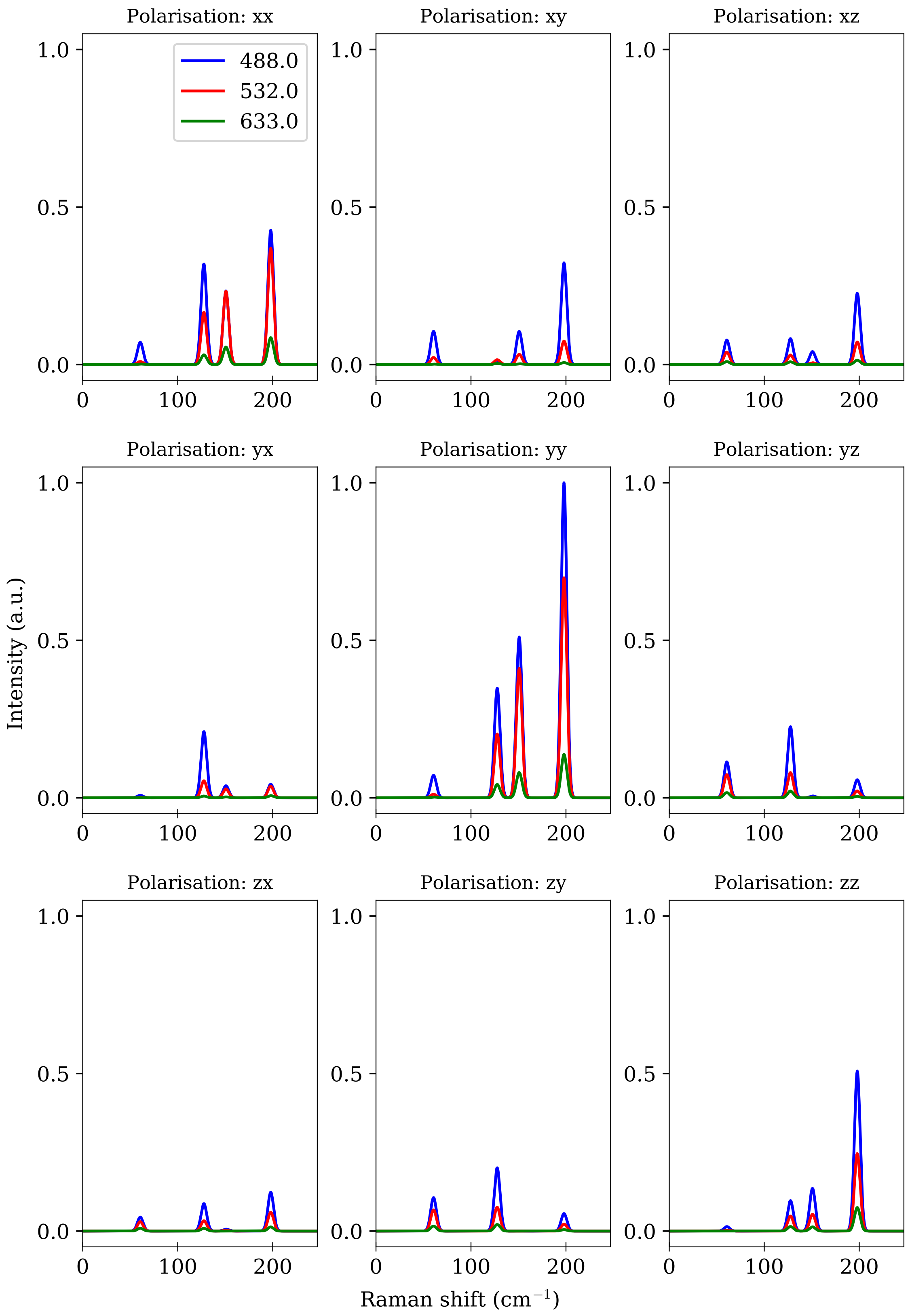

Supplementary Figure 74: Raman spectrum of BiBrSe with space group 156 and Wyckoff positions a-c-0.

BiBrS, 156, a-b-c-0

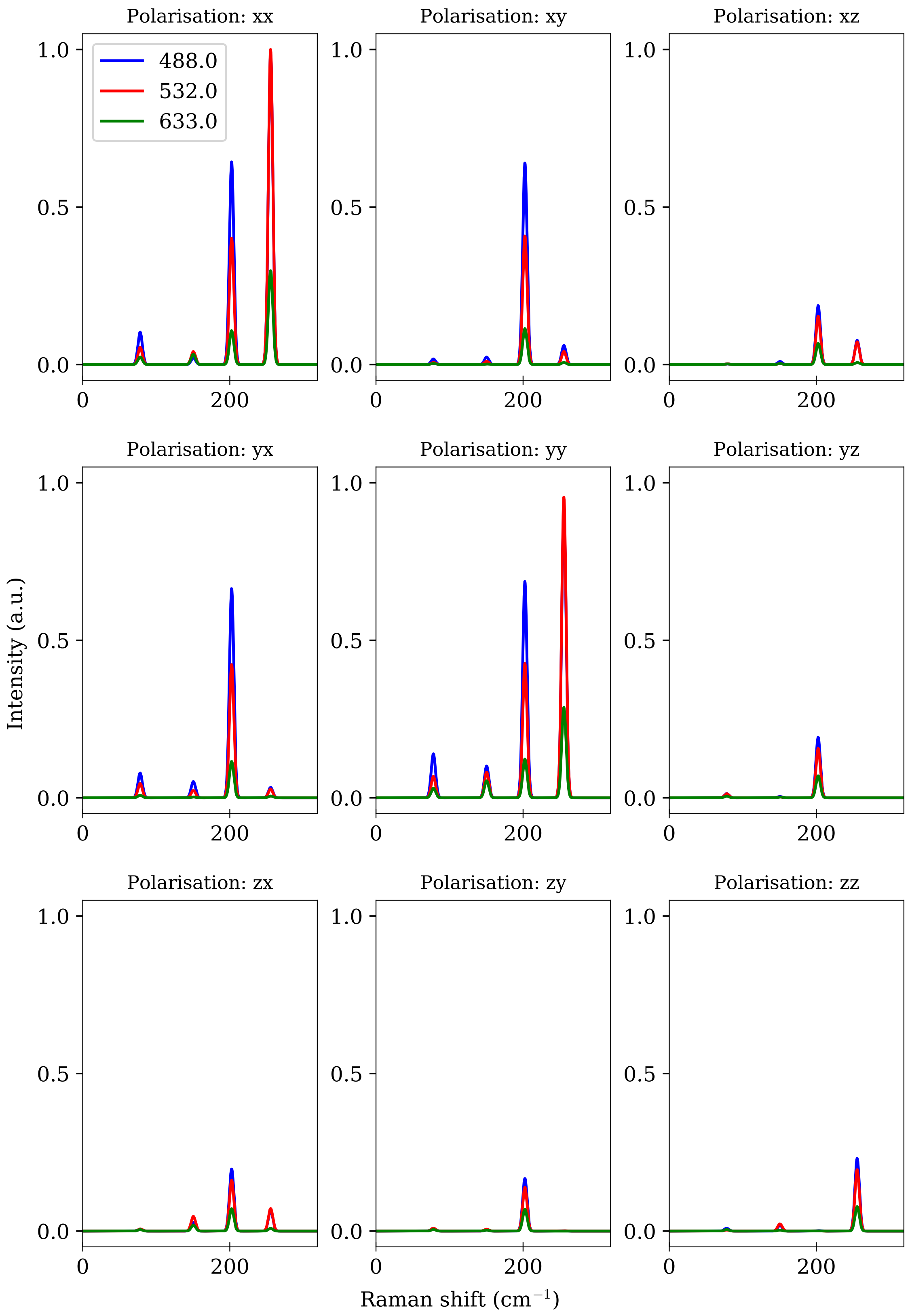

Supplementary Figure 75: Raman spectrum of BiBrS with space group 156 and Wyckoff positions a-b-c-0.

BiBrS, 156, a-c-0

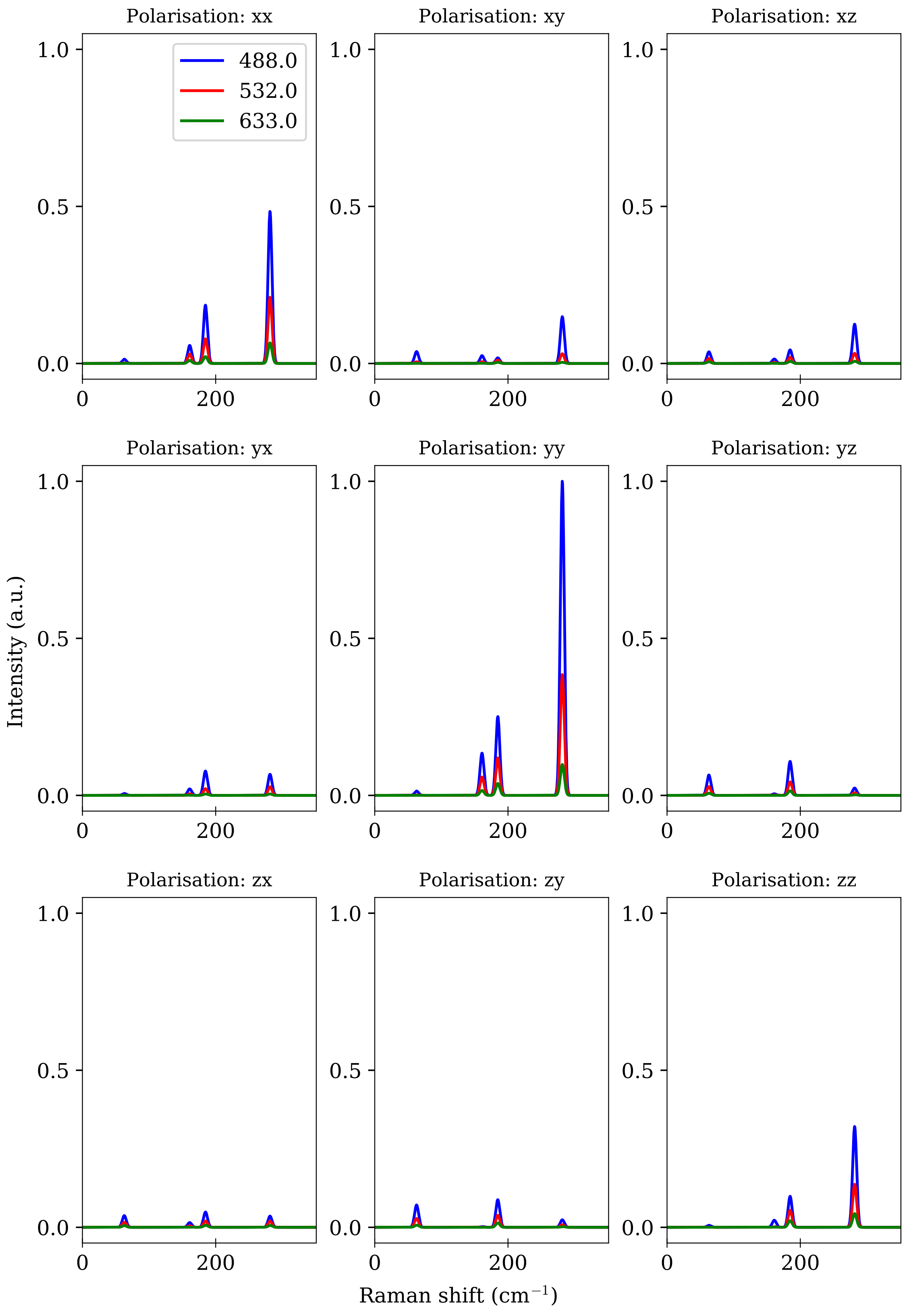

Supplementary Figure 76: Raman spectrum of BiBrS with space group 156 and Wyckoff positions a-c-0.

BiBrTe, 156, a-b-c-0

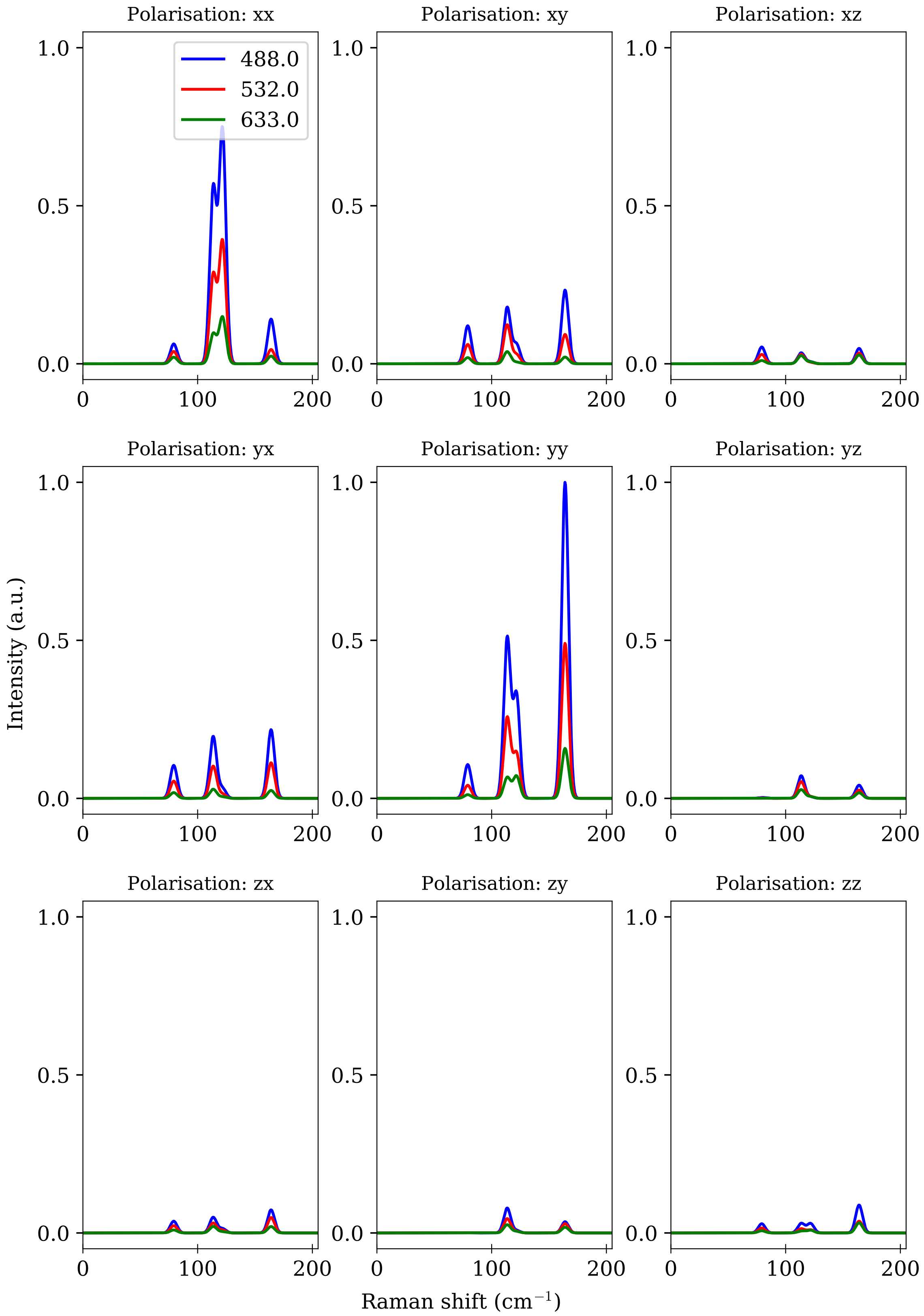

Supplementary Figure 77: Raman spectrum of BiBrTe with space group 156 and Wyckoff positions a-b-c-0.

BiBrTe, 156, a-c-0

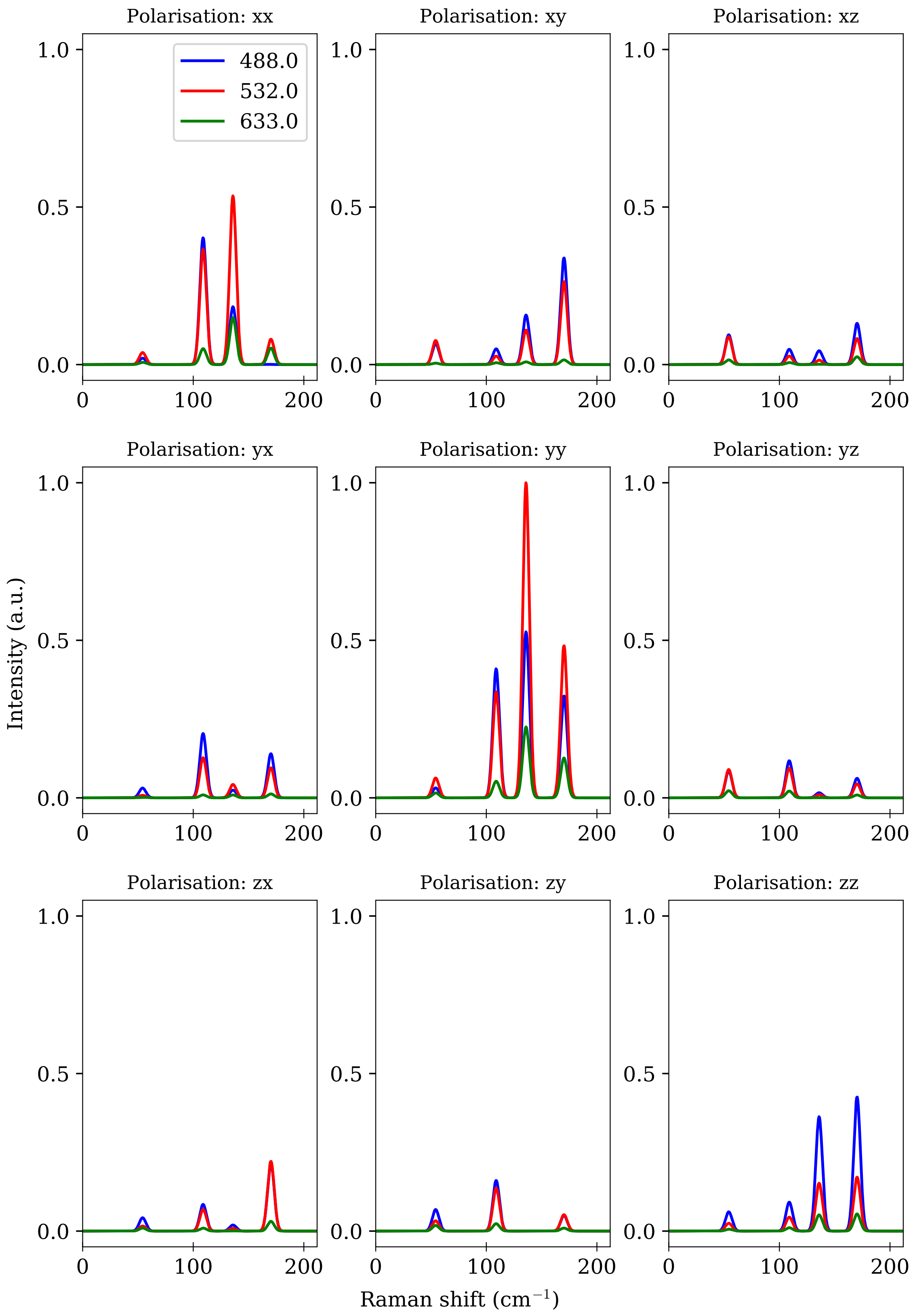

Supplementary Figure 78: Raman spectrum of BiBrTe with space group 156 and Wyckoff positions a-c-0.

BiClSe, 156, a-b-c-0

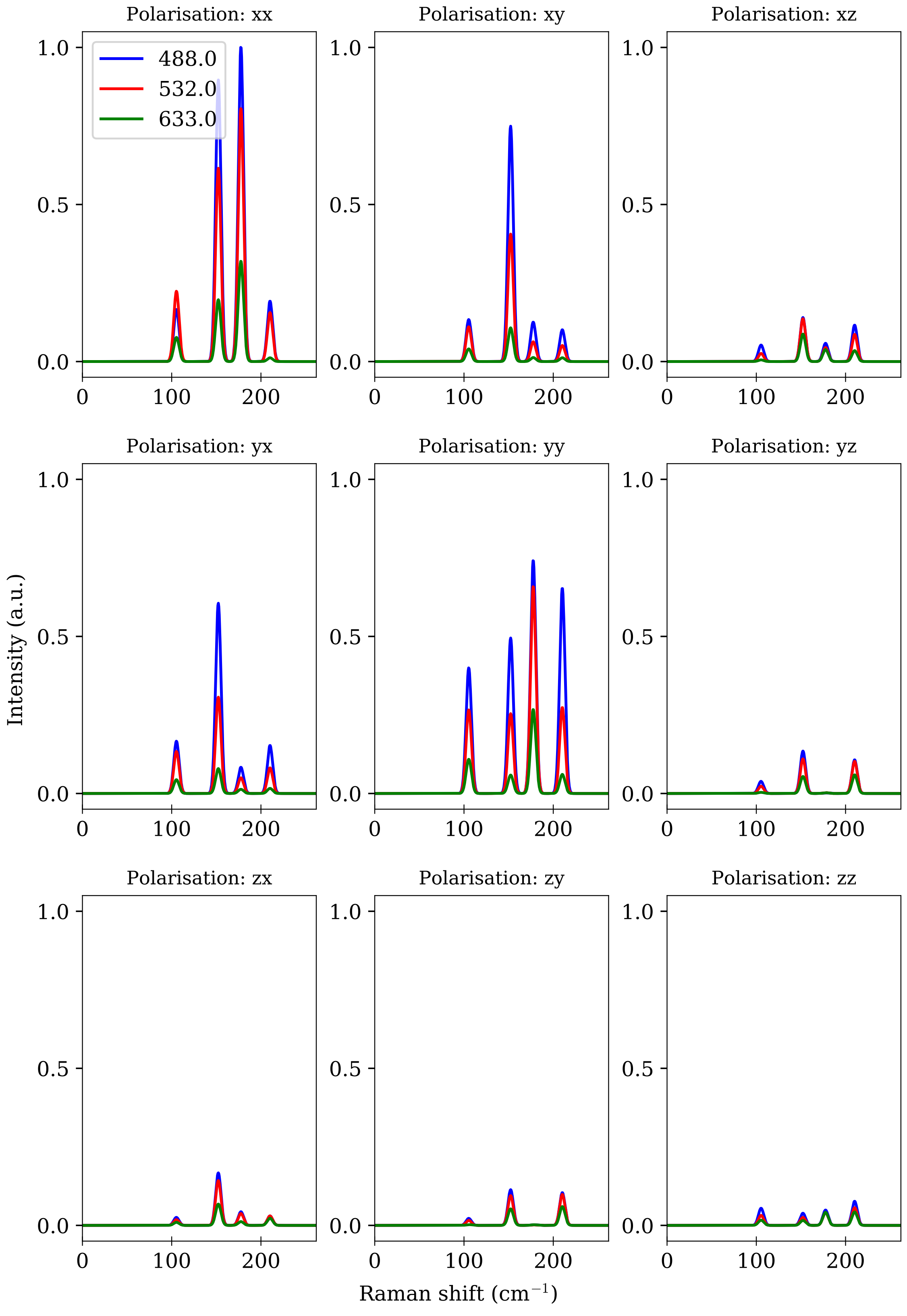

Supplementary Figure 79: Raman spectrum of BiClSe with space group 156 and Wyckoff positions a-b-c-0.

BiClSe, 156, a-c-0

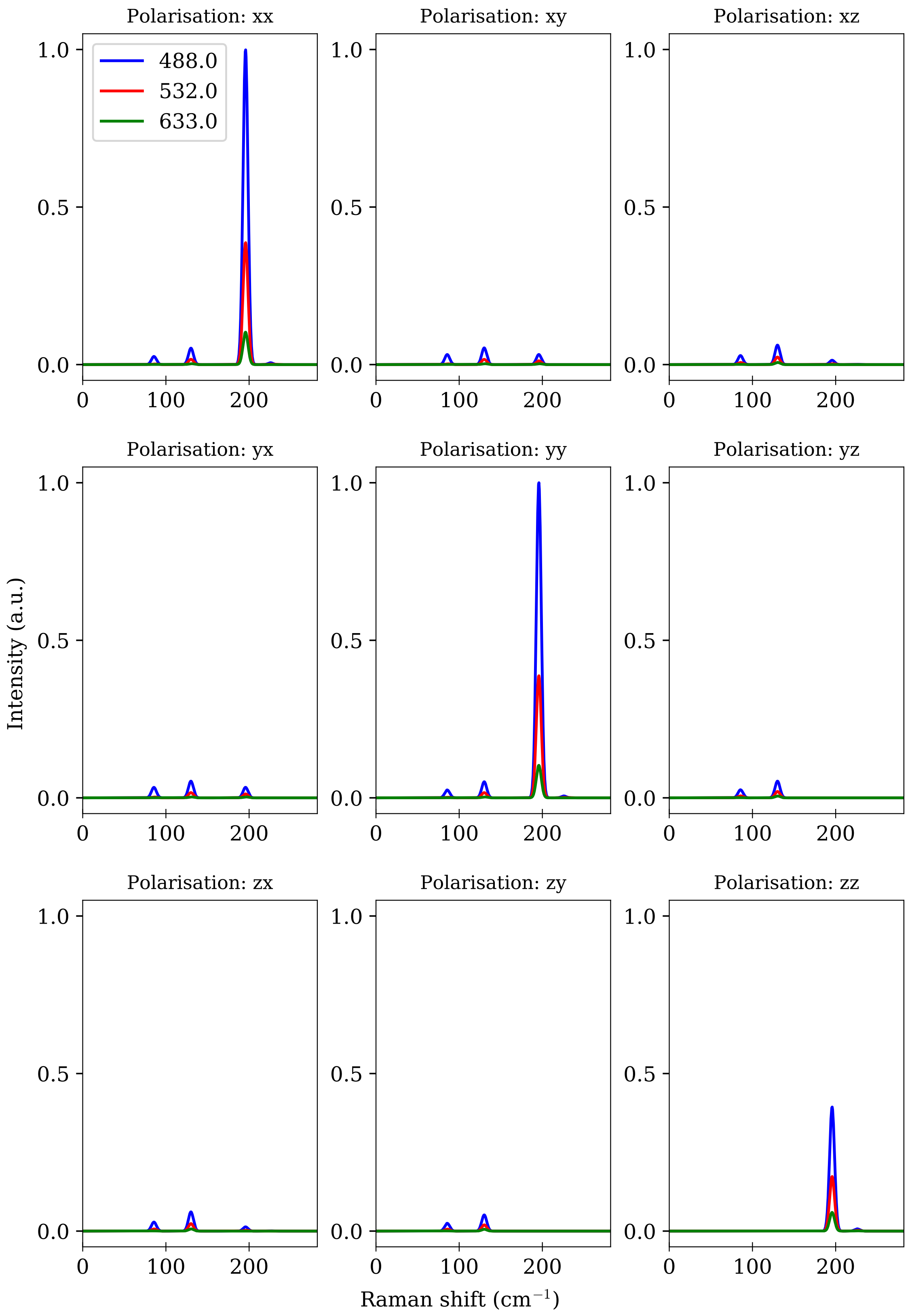

Supplementary Figure 80: Raman spectrum of BiClSe with space group 156 and Wyckoff positions a-c-0.

BiClS, 156, a-b-c-0

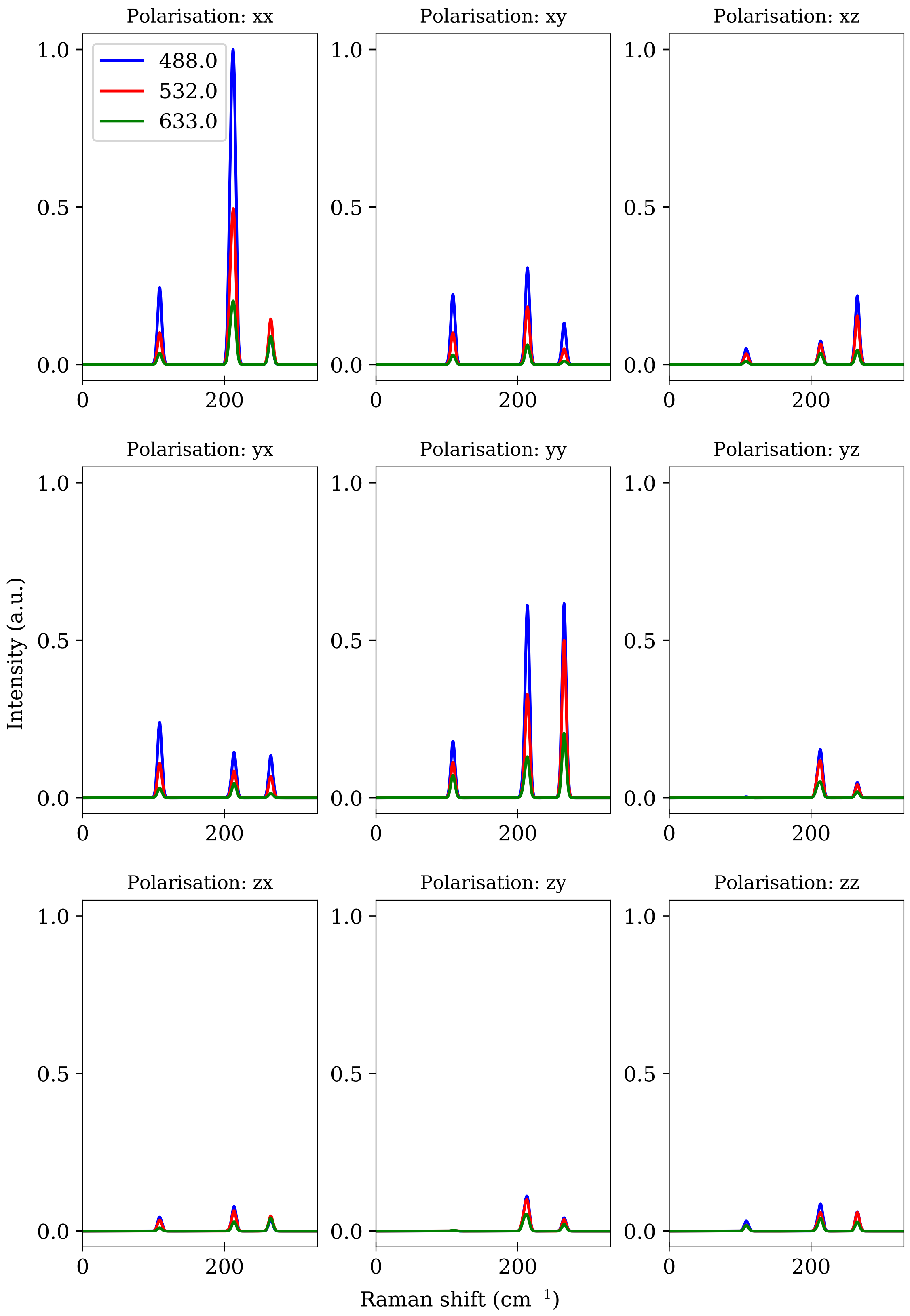

Supplementary Figure 81: Raman spectrum of BiClS with space group 156 and Wyckoff positions a-b-c-0.

BiClS, 156, a-c-0

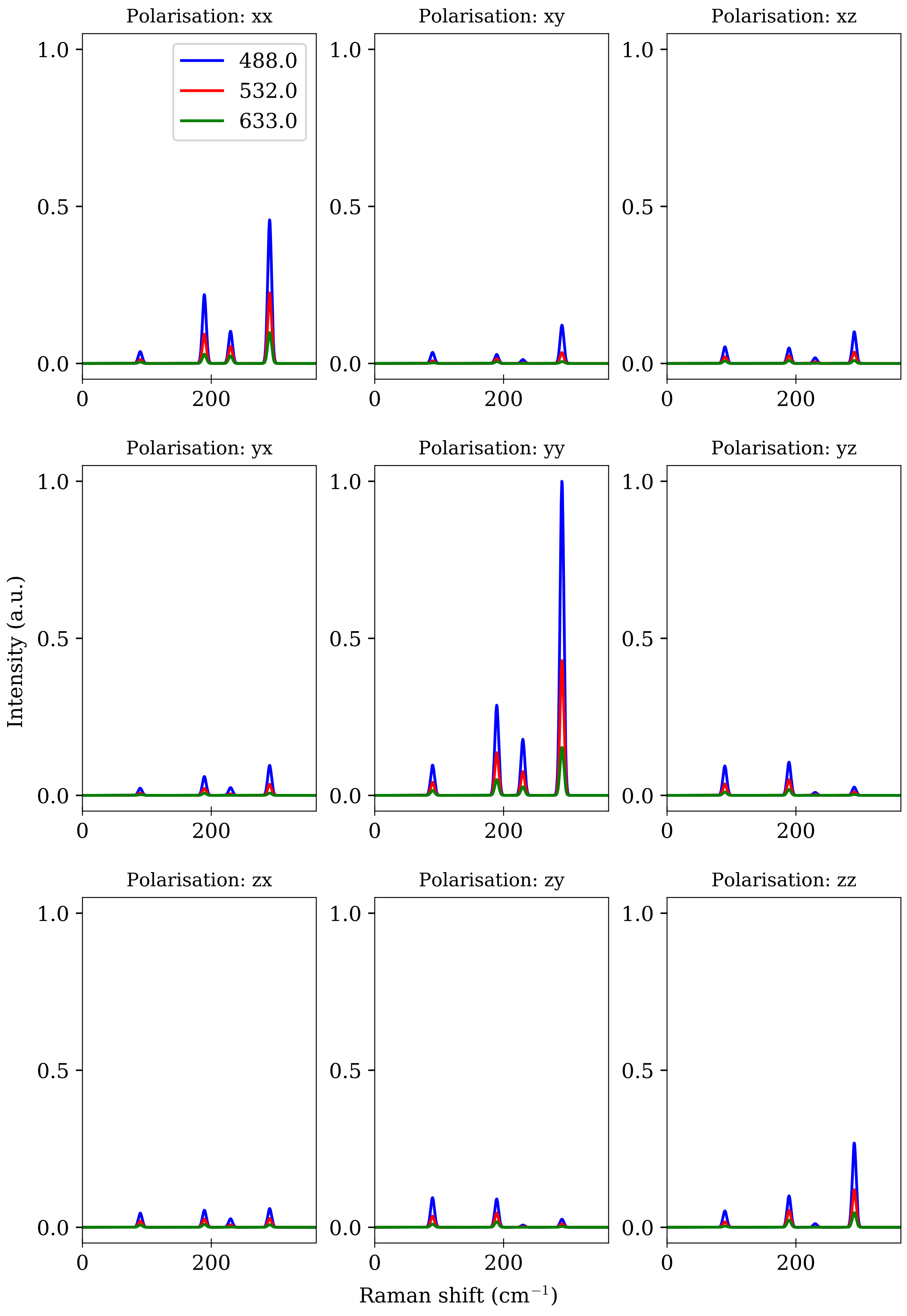

Supplementary Figure 82: Raman spectrum of BiClS with space group 156 and Wyckoff positions a-c-0.

BiClTe, 156, a-b-c-0

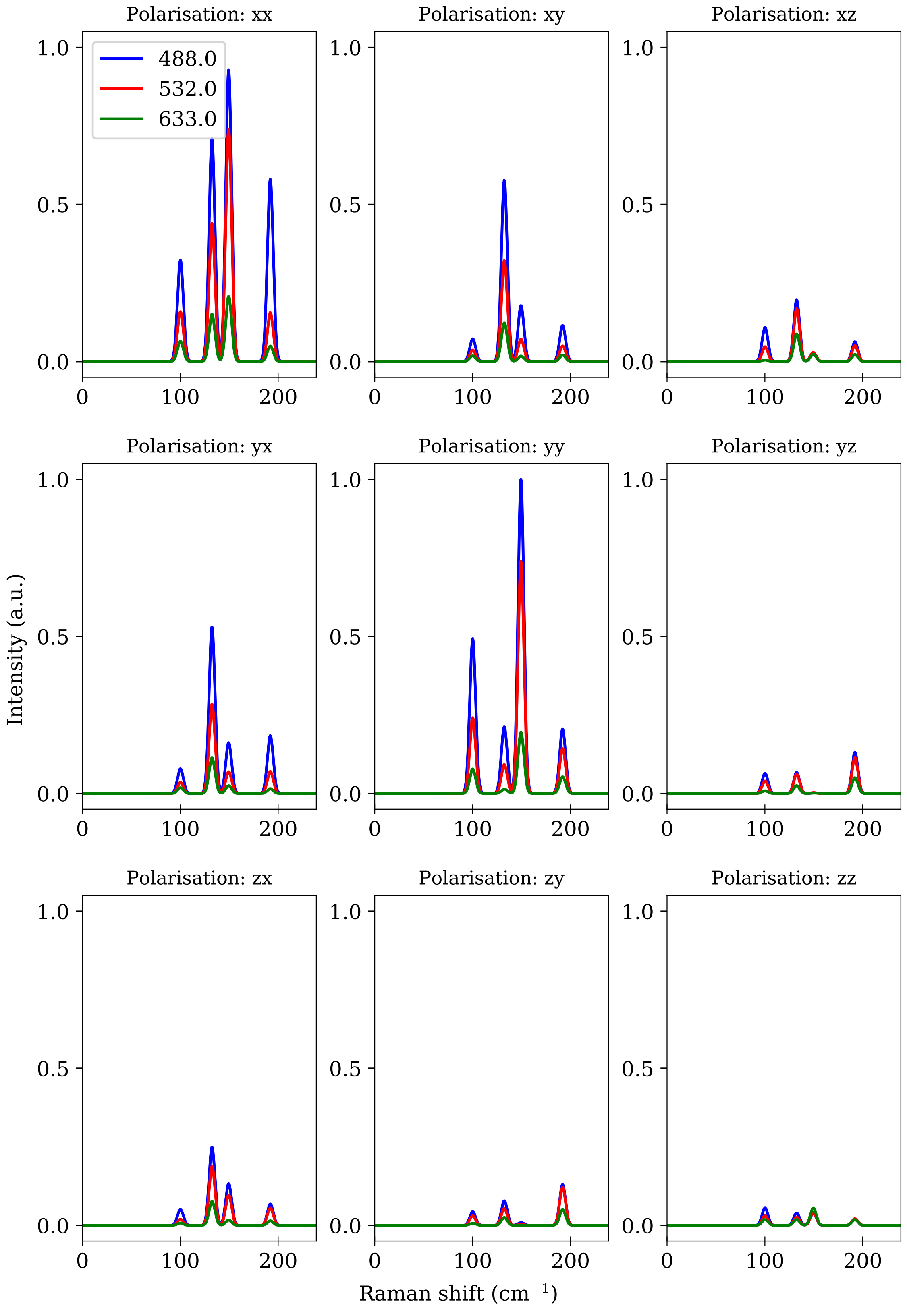

Supplementary Figure 83: Raman spectrum of BiClTe with space group 156 and Wyckoff positions a-b-c-0.

BiCrAs, 156, a-b-c-0

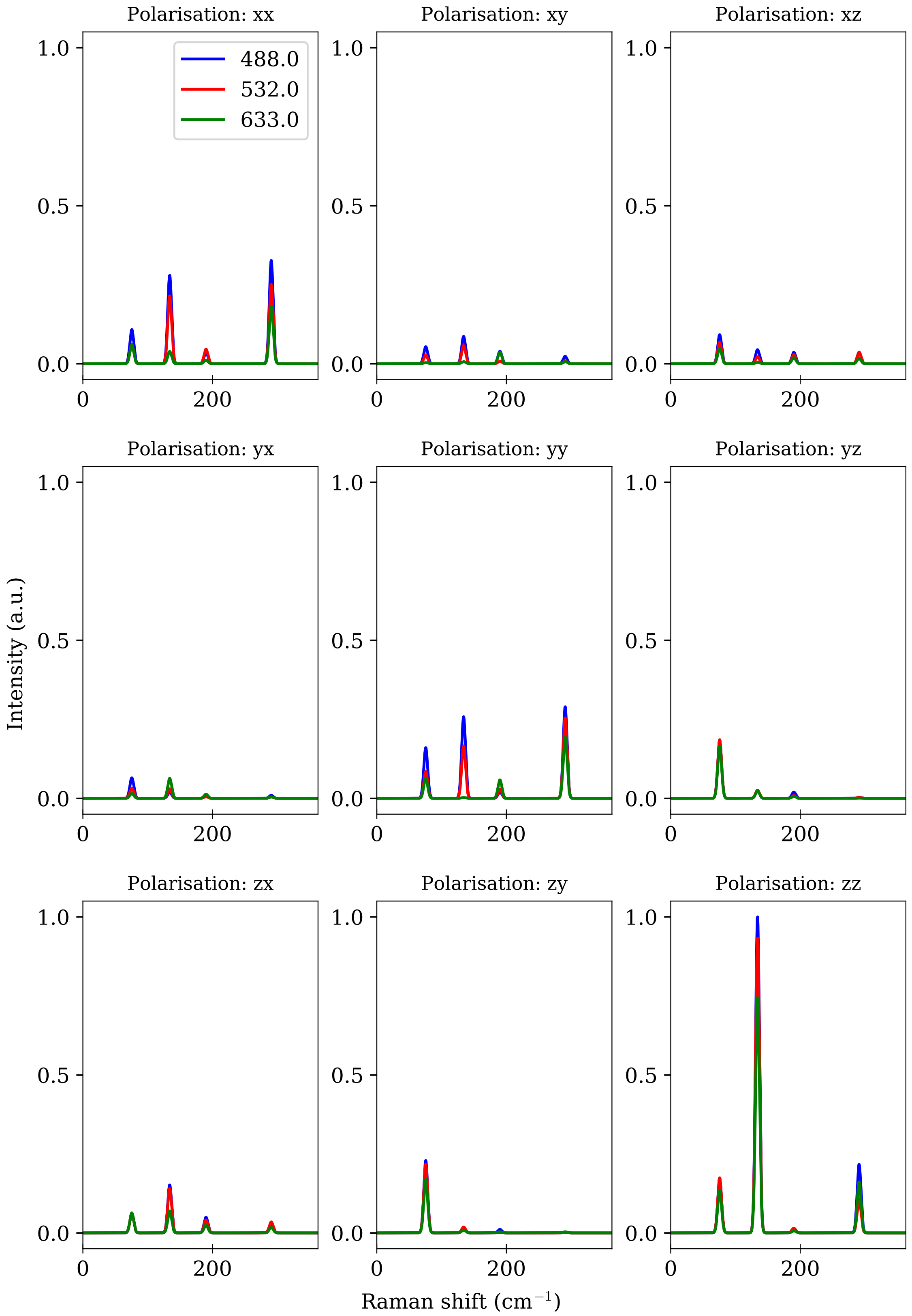

Supplementary Figure 84: Raman spectrum of BiCrAs with space group 156 and Wyckoff positions a-b-c-0.

BiHfSb, 156, a-b-c-0

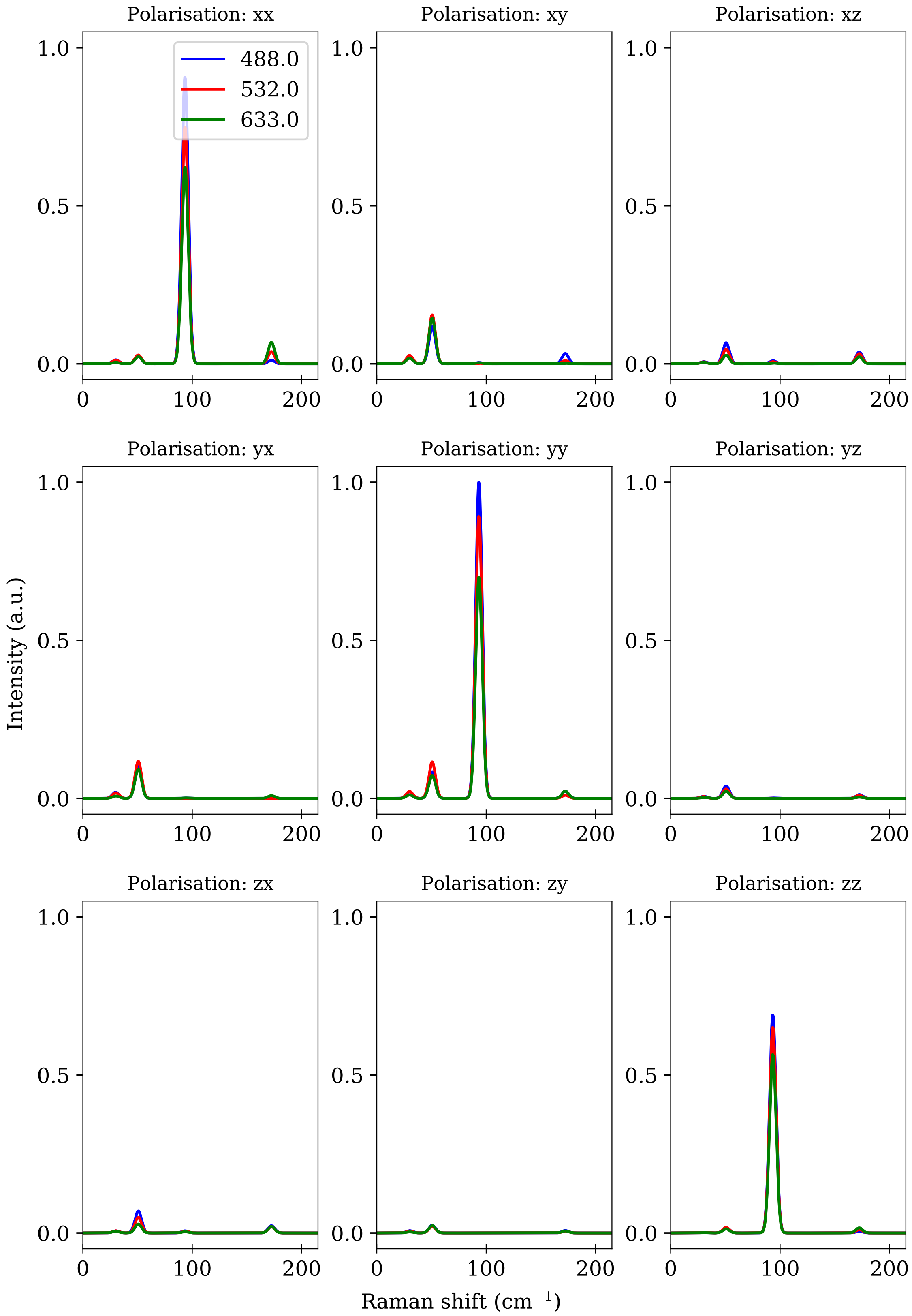

Supplementary Figure 85: Raman spectrum of BiHfSb with space group 156 and Wyckoff positions a-b-c-0.

BiHfSb, 156, a-c-0

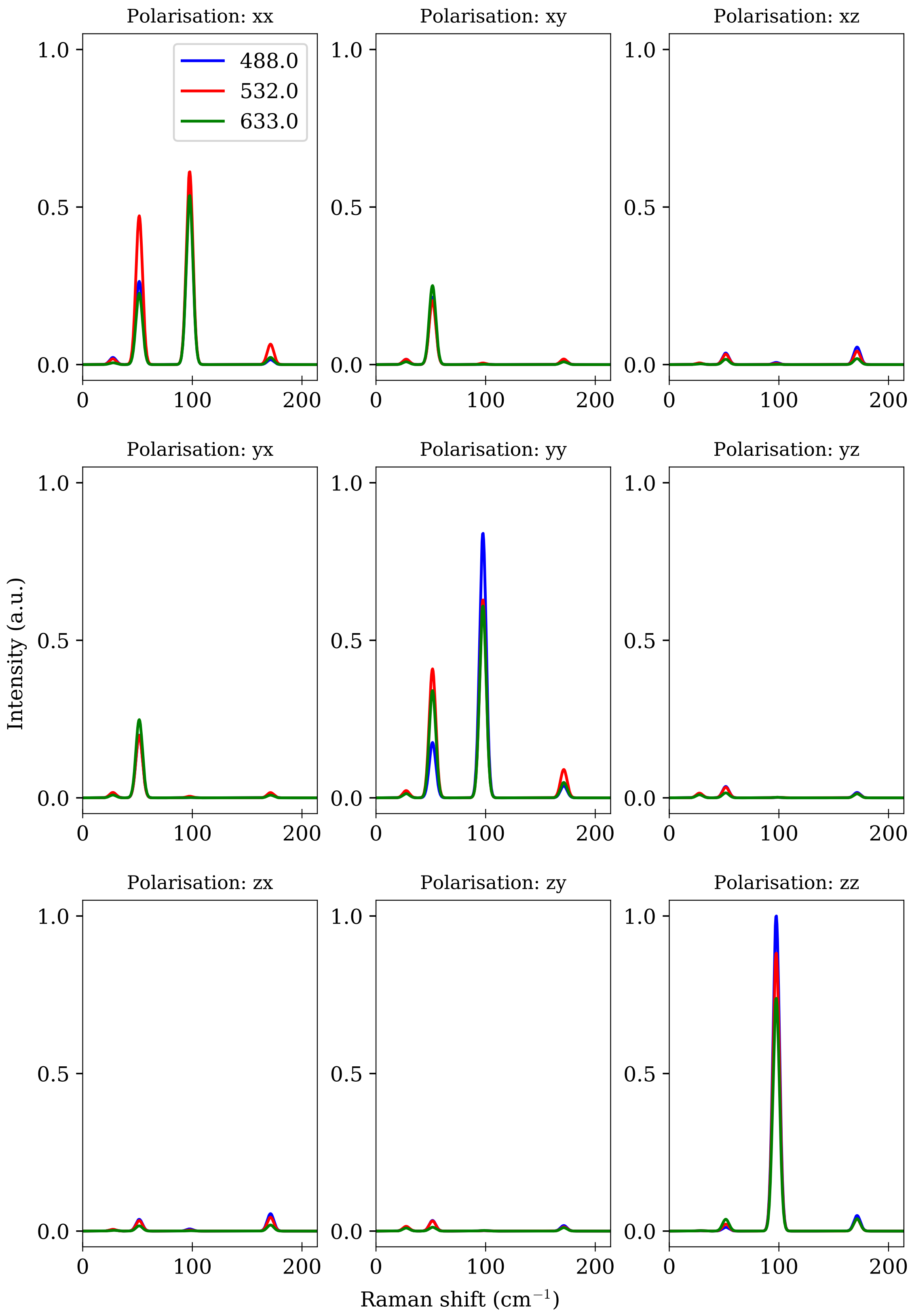

Supplementary Figure 86: Raman spectrum of BiHfSb with space group 156 and Wyckoff positions a-c-0.

BiISe, 156, a-b-c-0

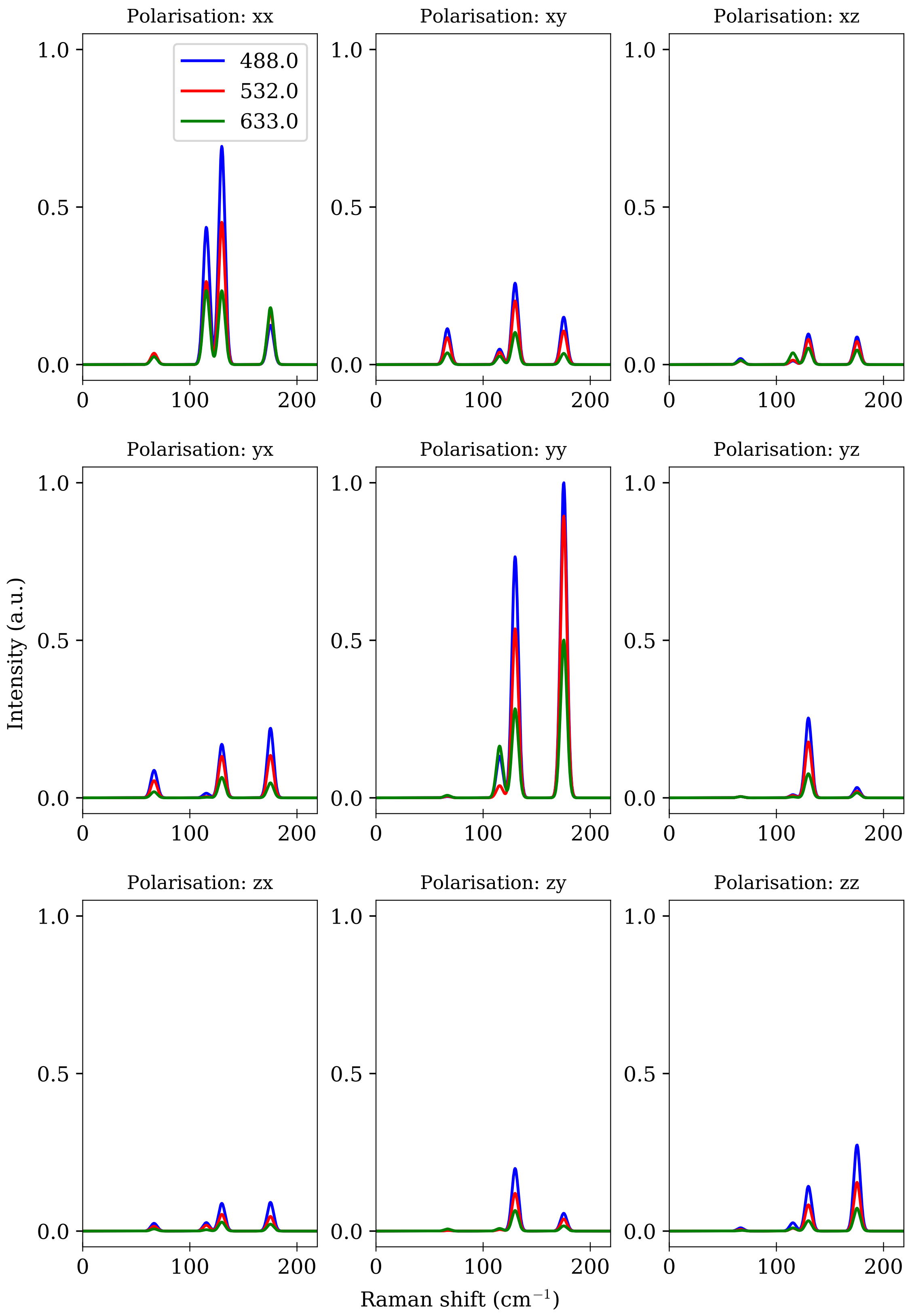

Supplementary Figure 87: Raman spectrum of BiISe with space group 156 and Wyckoff positions a-b-c-0.

BiISe, 156, a-c-0

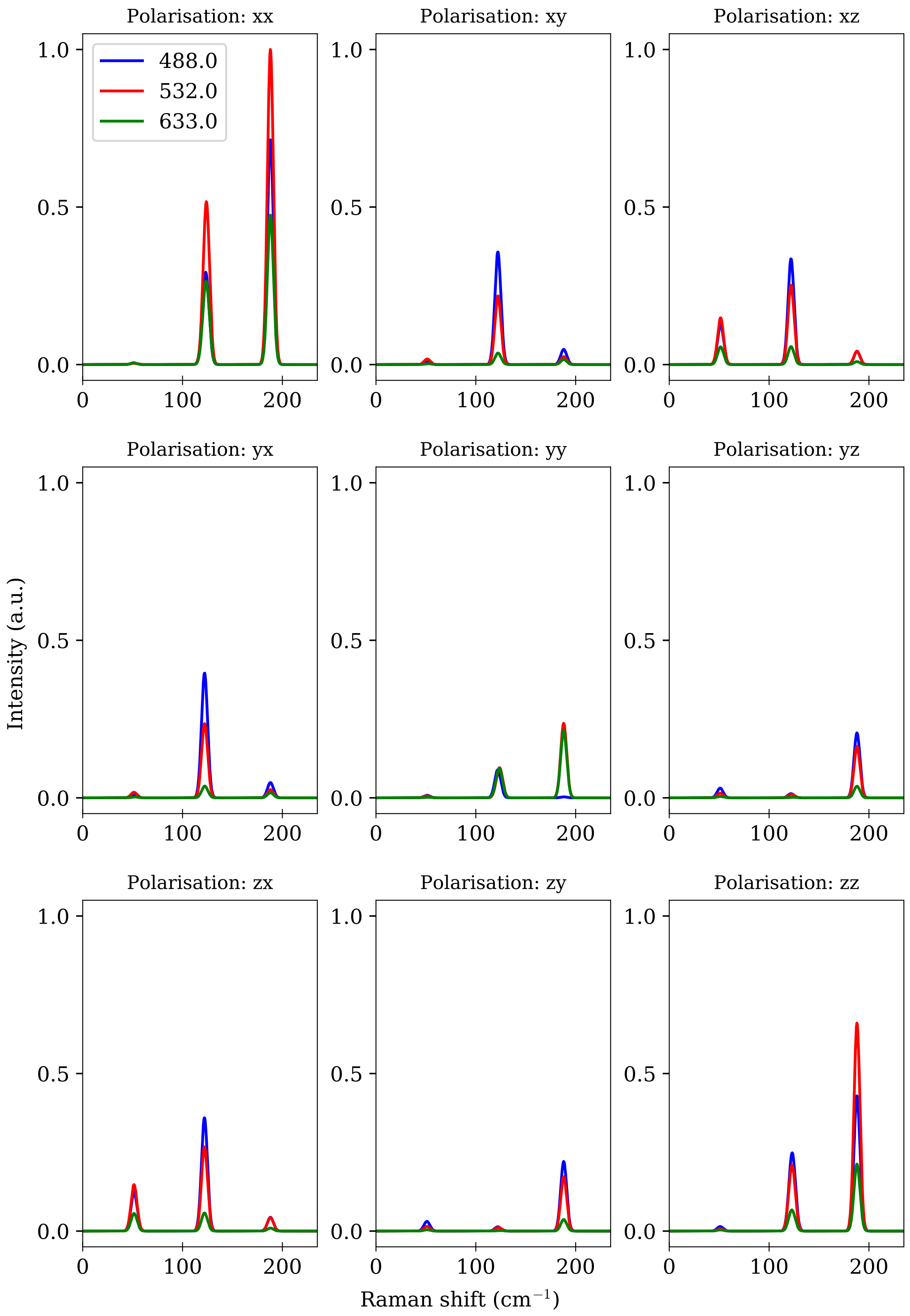

Supplementary Figure 88: Raman spectrum of BiISe with space group 156 and Wyckoff positions a-c-0.

BiIS, 156, a-b-c-0

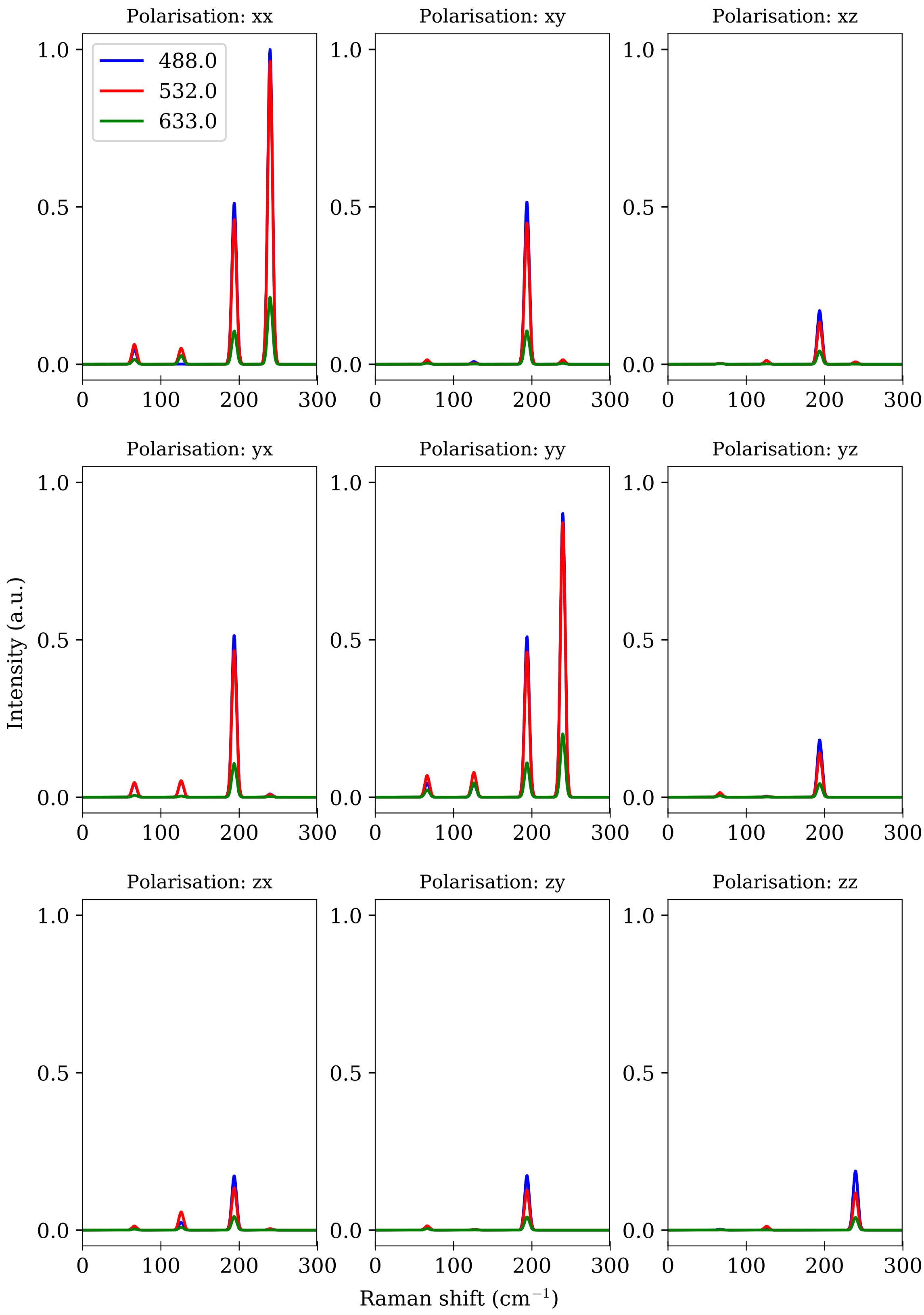

Supplementary Figure 89: Raman spectrum of BiIS with space group 156 and Wyckoff positions a-b-c-0.

BiITe, 156, a-b-c-0

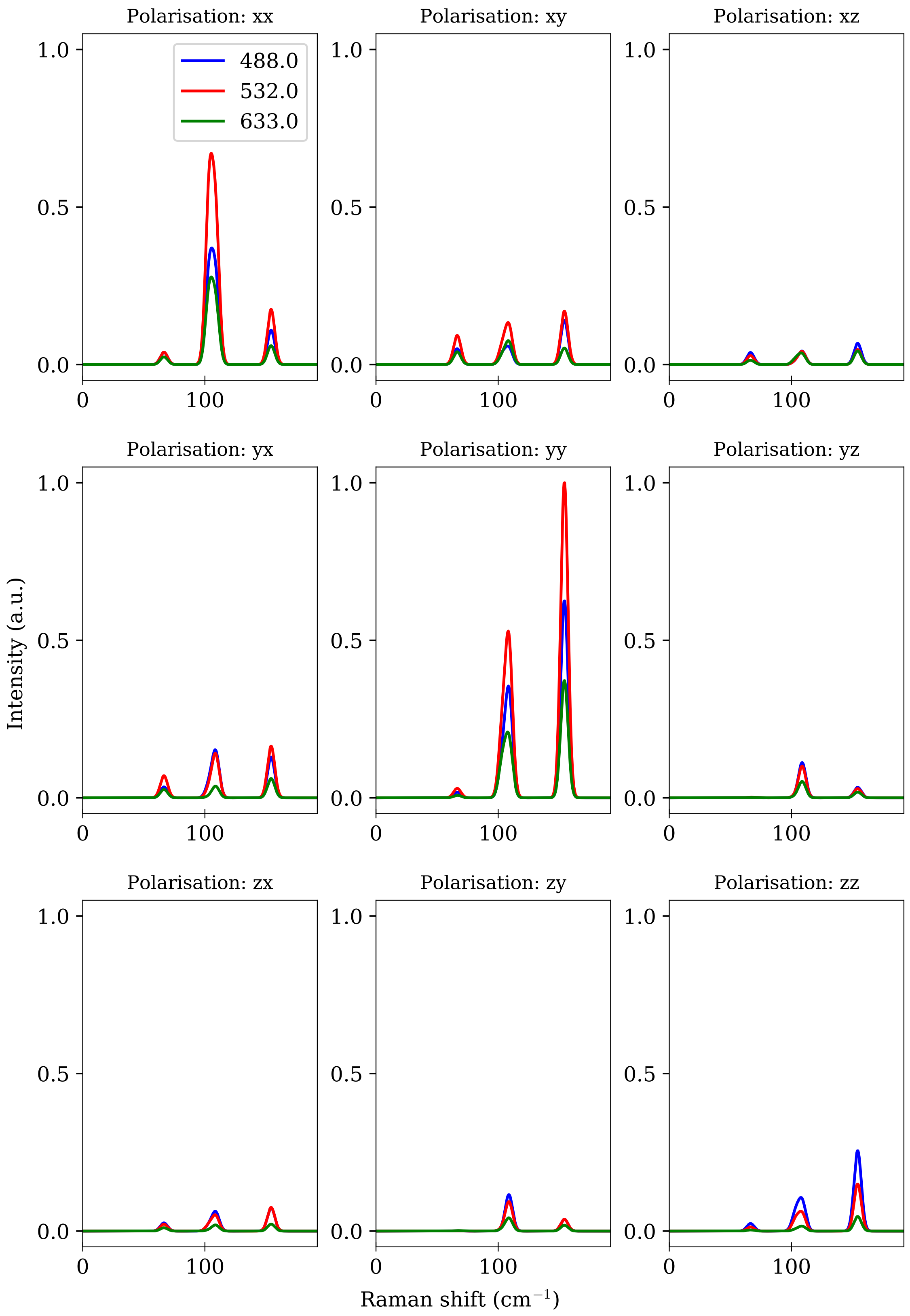

Supplementary Figure 90: Raman spectrum of BiITe with space group 156 and Wyckoff positions a-b-c-0.

BiITe, 156, a-c-0

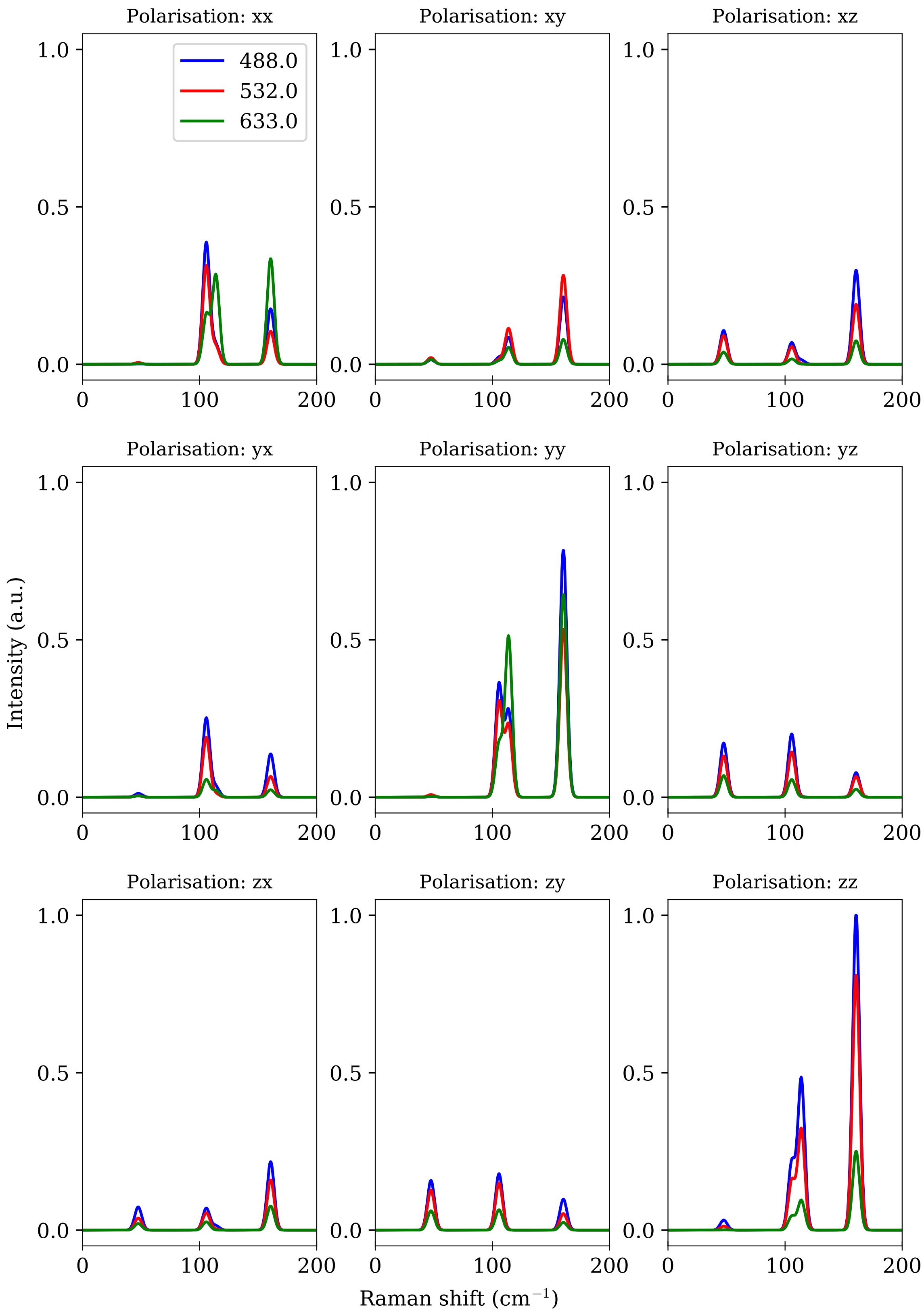

Supplementary Figure 91: Raman spectrum of BiITe with space group 156 and Wyckoff positions a-c-0.

BiMoAs, 156, a-b-c-0

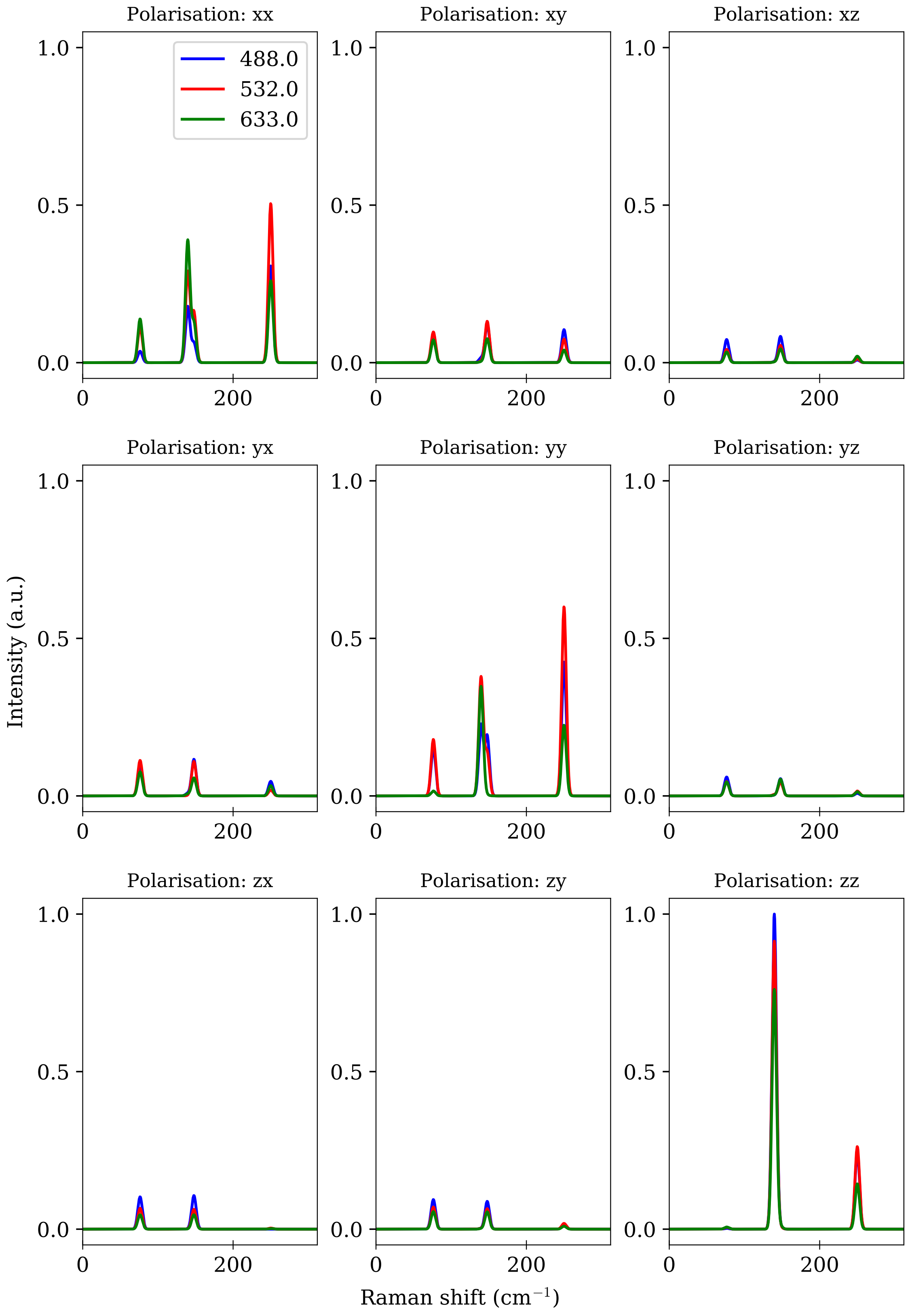

Supplementary Figure 92: Raman spectrum of BiMoAs with space group 156 and Wyckoff positions a-b-c-0.

BiMoAs, 156, a-c-0

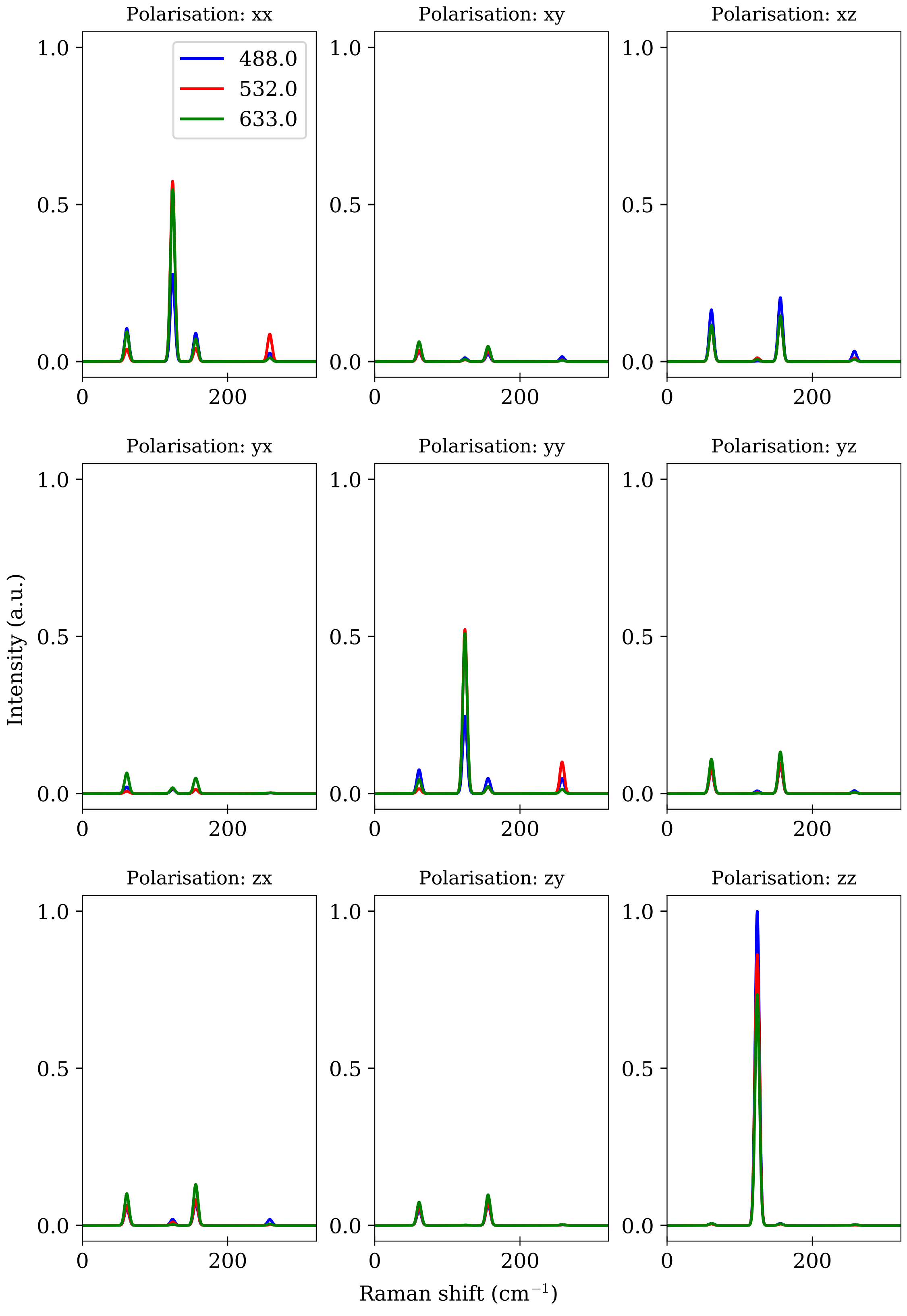

Supplementary Figure 93: Raman spectrum of BiMoAs with space group 156 and Wyckoff positions a-c-0.

BiMoSb, 156, a-b-c-0

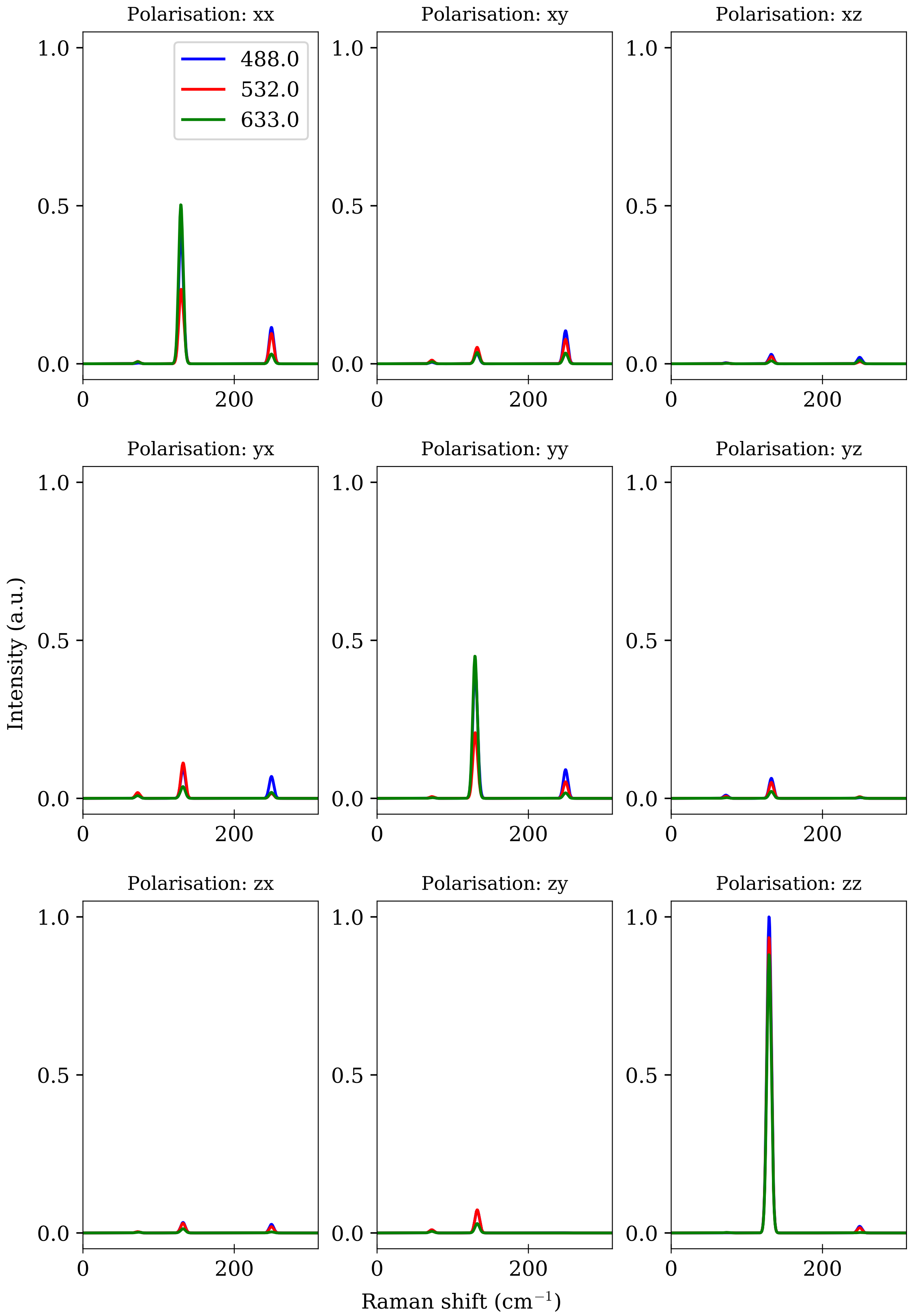

Supplementary Figure 94: Raman spectrum of BiMoSb with space group 156 and Wyckoff positions a-b-c-0.

BiNbAs, 156, a-b-c-0

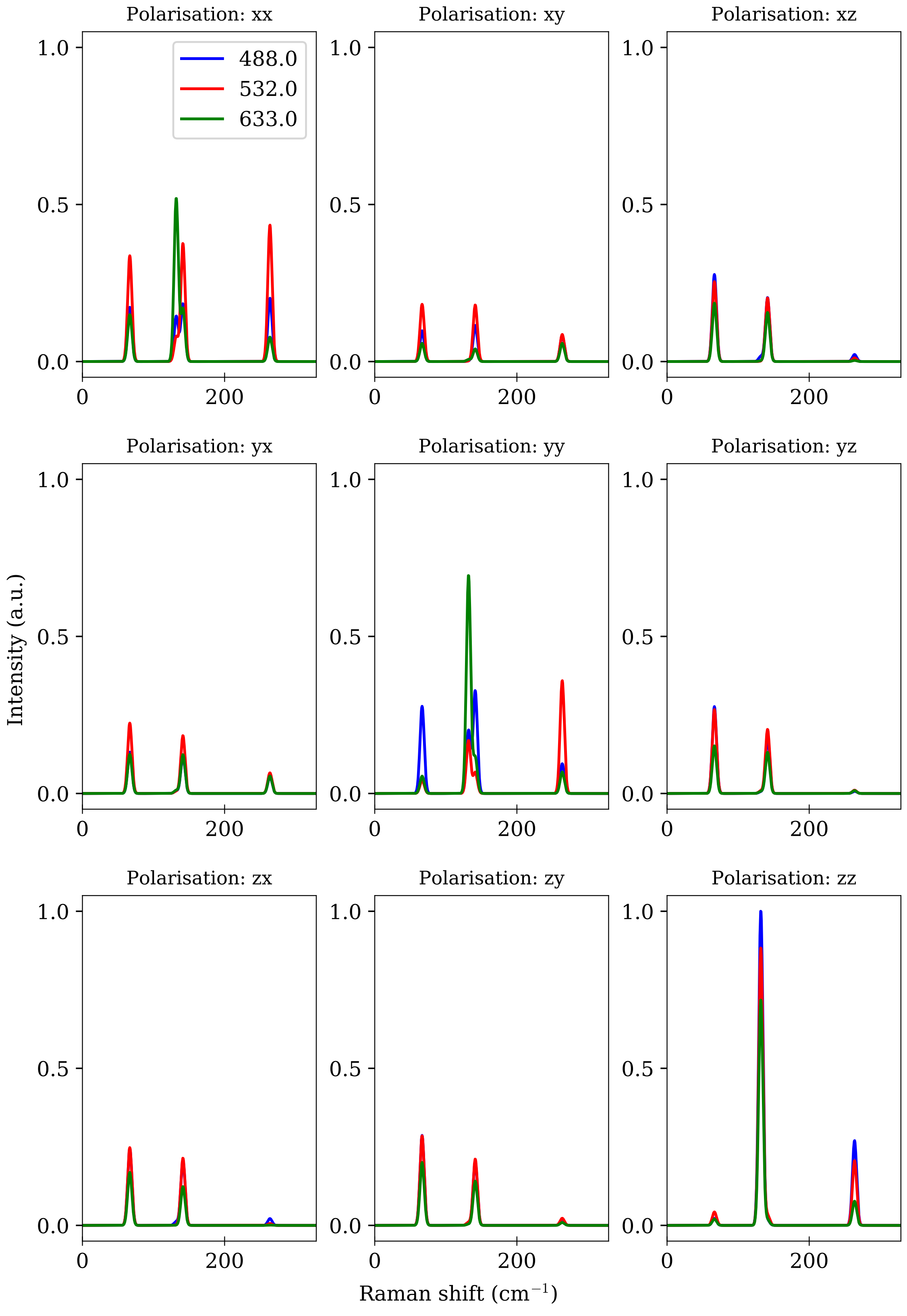

Supplementary Figure 95: Raman spectrum of BiNbAs with space group 156 and Wyckoff positions a-b-c-0.

BiNbAs, 156, a-c-0

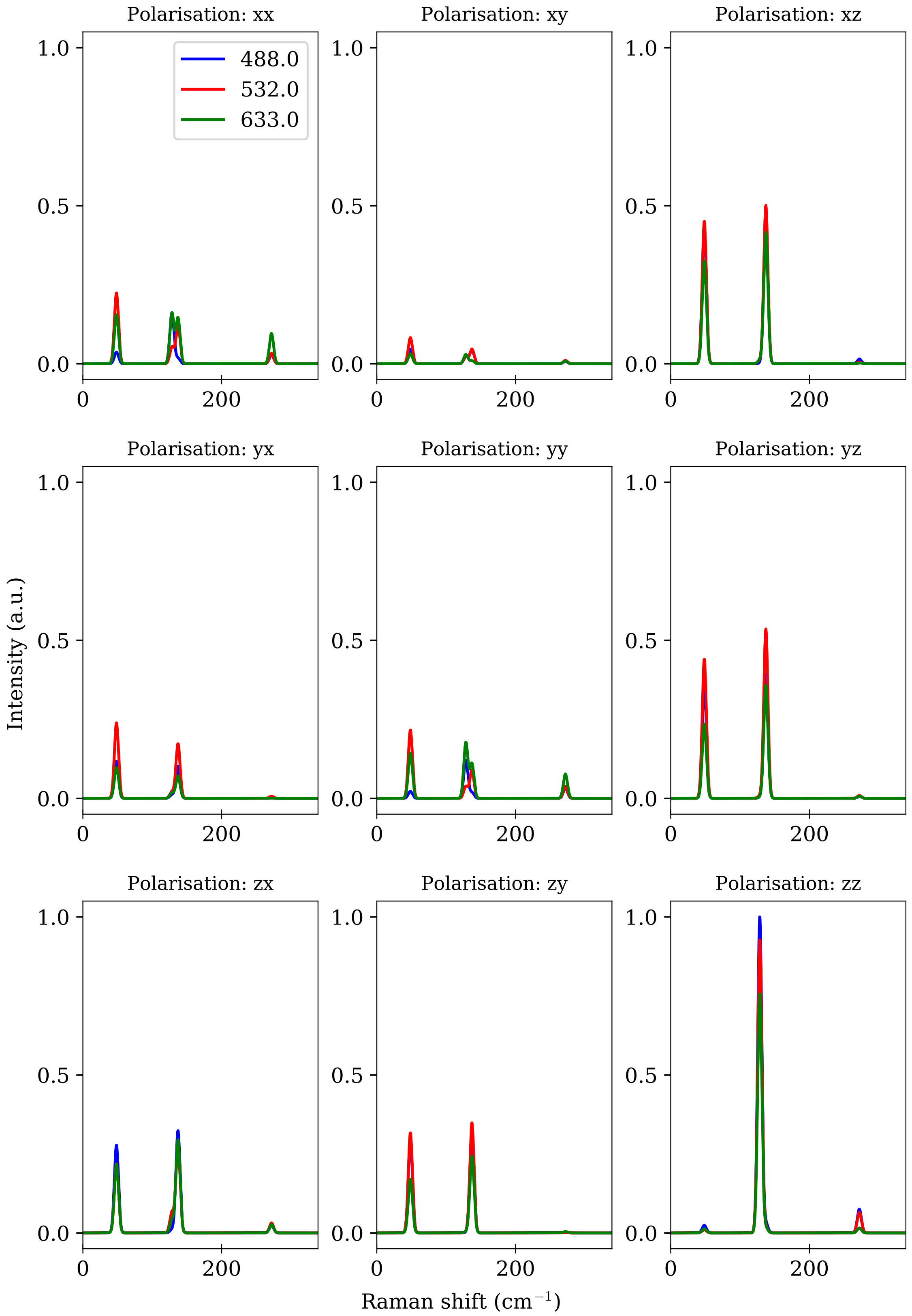

Supplementary Figure 96: Raman spectrum of BiNbAs with space group 156 and Wyckoff positions a-c-0.

BiNbSb, 156, a-b-c-0

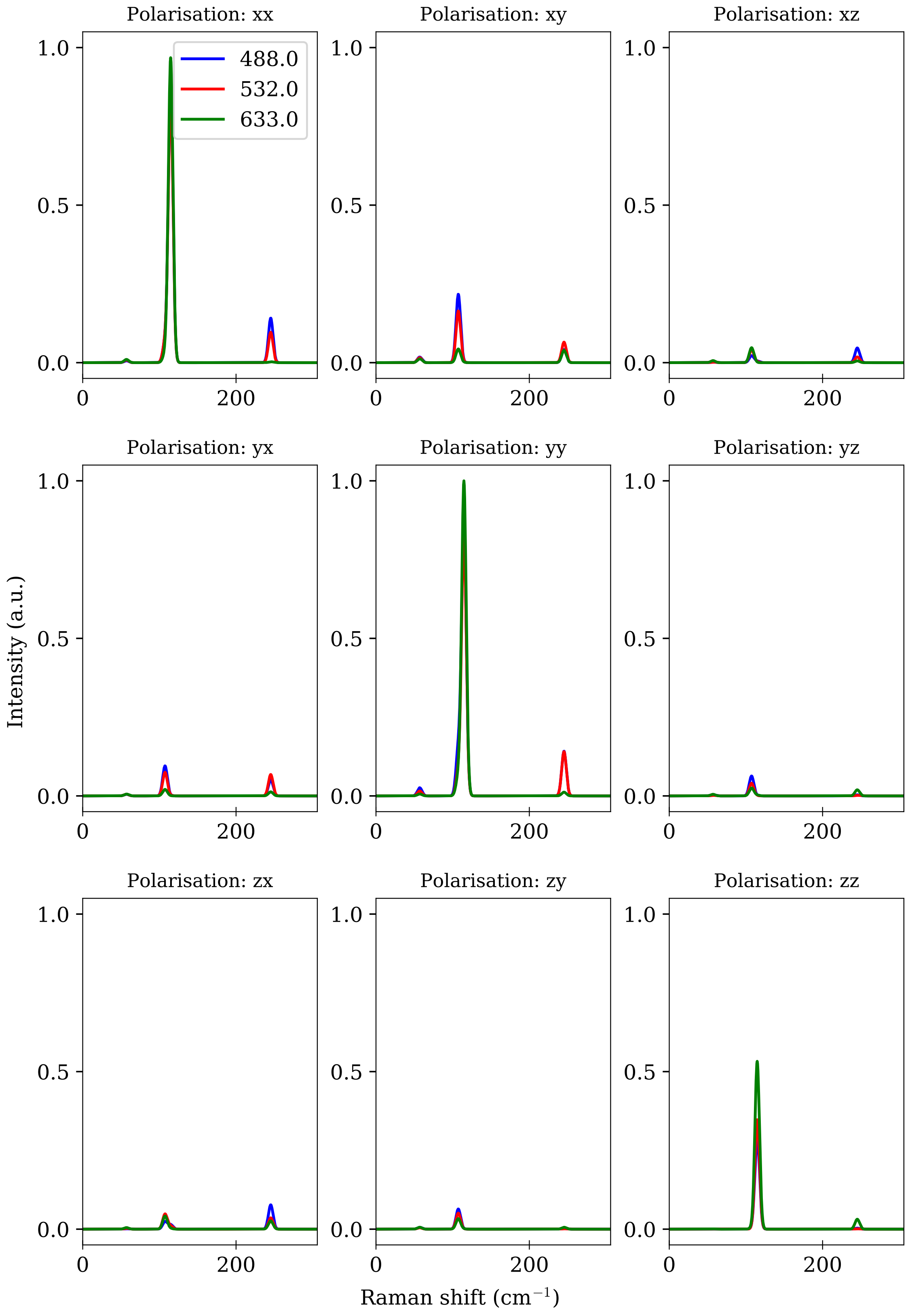

Supplementary Figure 97: Raman spectrum of BiNbSb with space group 156 and Wyckoff positions a-b-c-0.

BiNbSb, 156, a-c-0

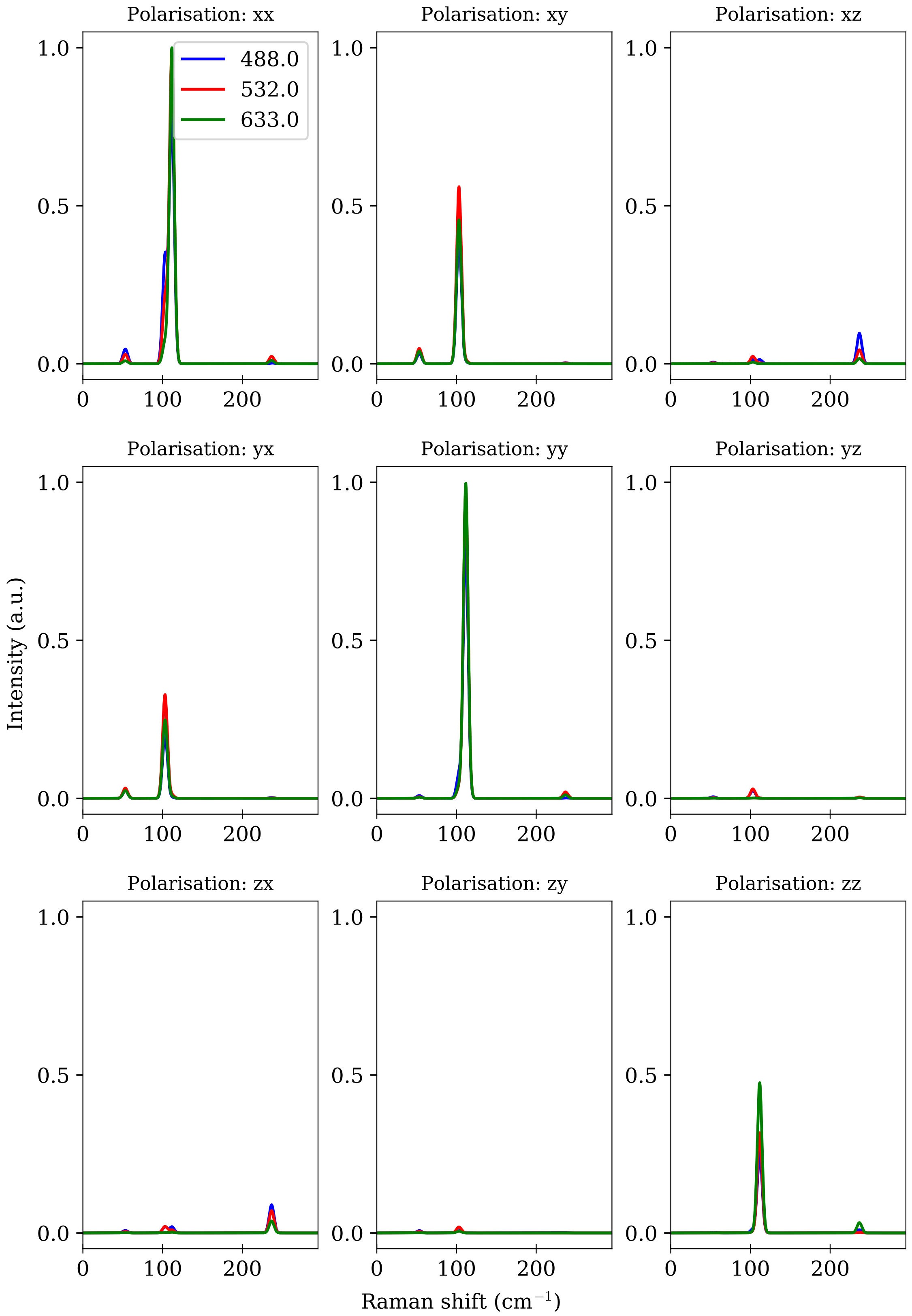

Supplementary Figure 98: Raman spectrum of BiNbSb with space group 156 and Wyckoff positions a-c-0.

BiTaAs, 156, a-b-c-0

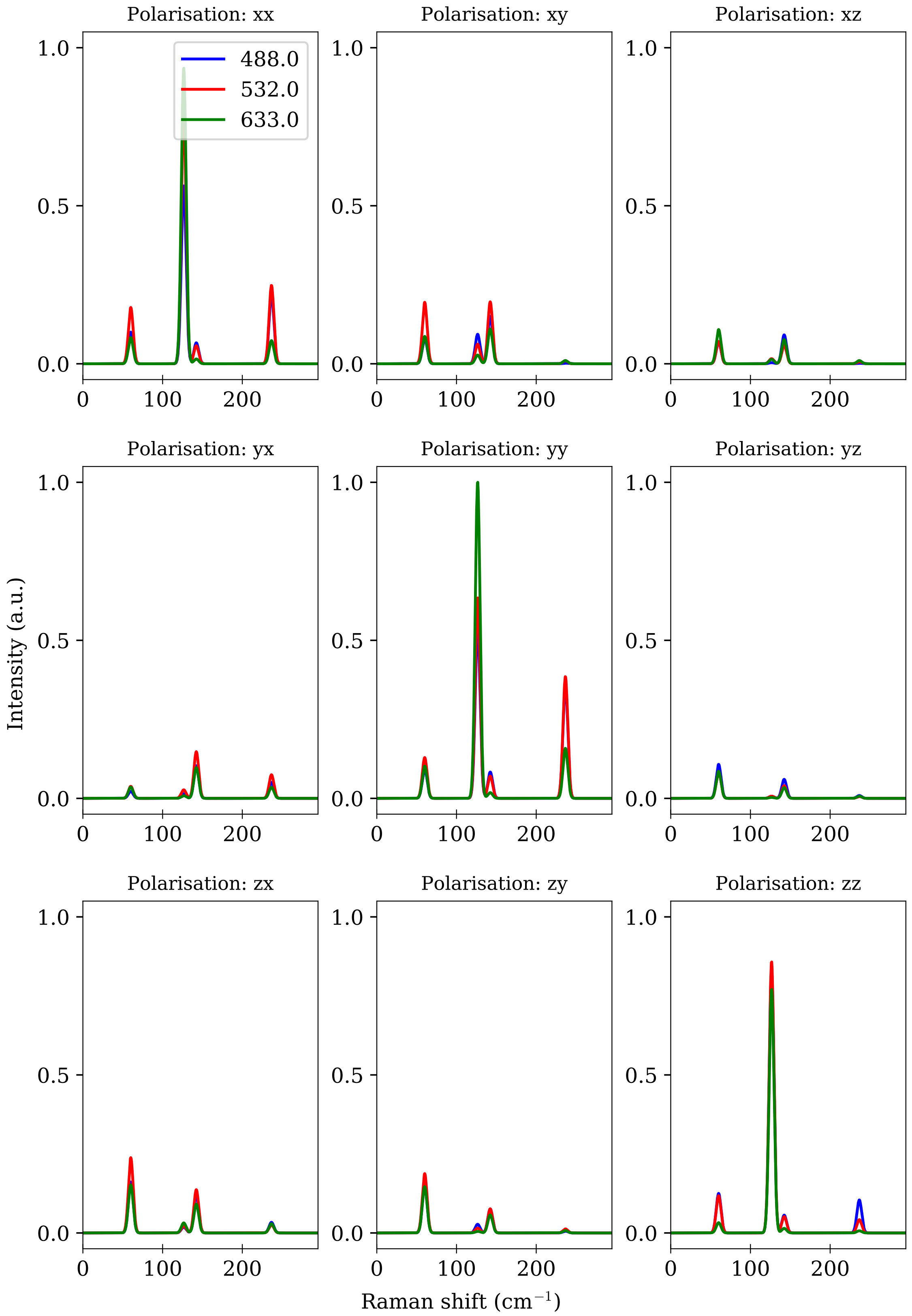

Supplementary Figure 99: Raman spectrum of BiTaAs with space group 156 and Wyckoff positions a-b-c-0.

BiTaAs, 156, a-c-0

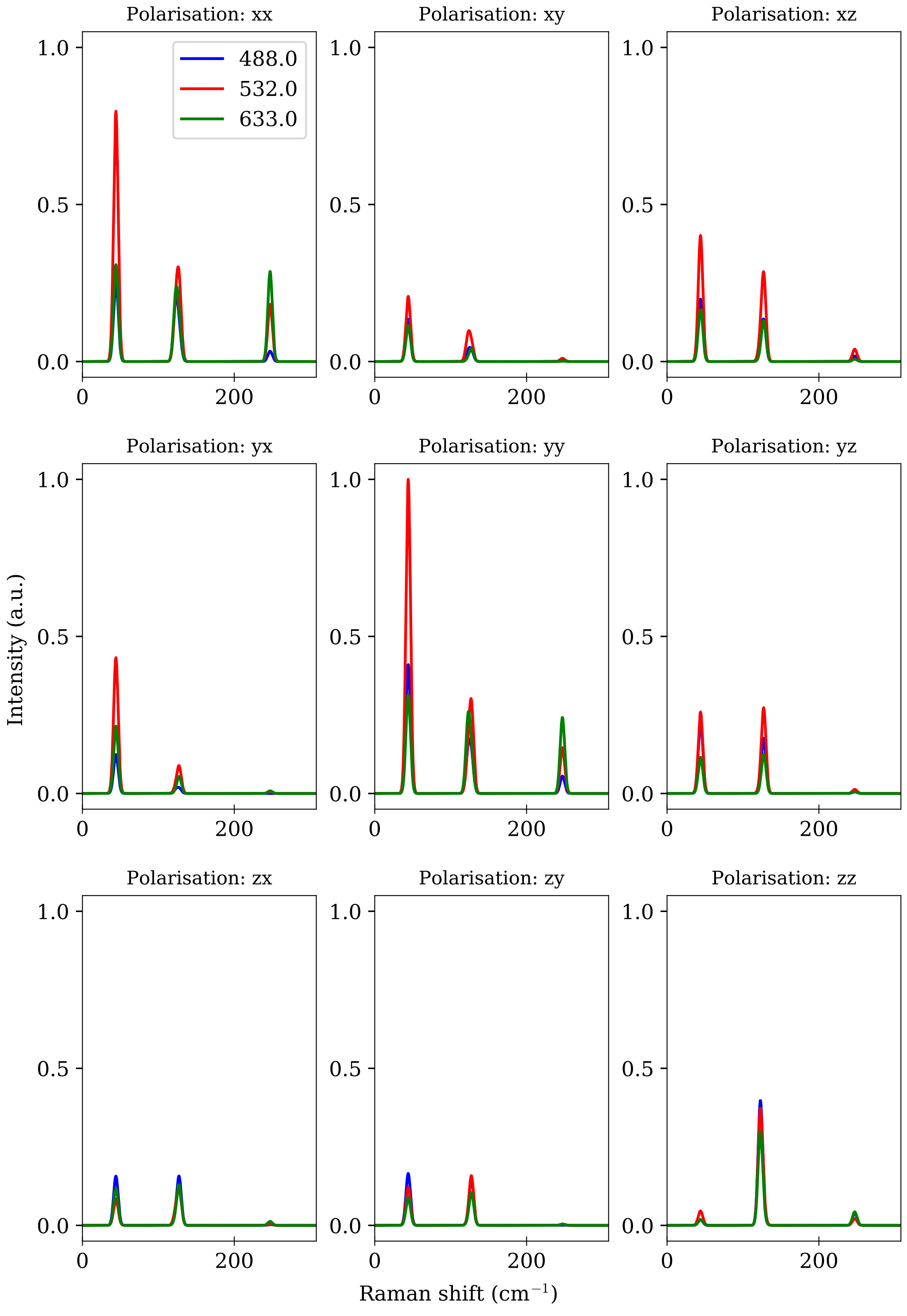

Supplementary Figure 100: Raman spectrum of BiTaAs with space group 156 and Wyckoff positions a-c-0.

BiTaSb, 156, a-b-c-0

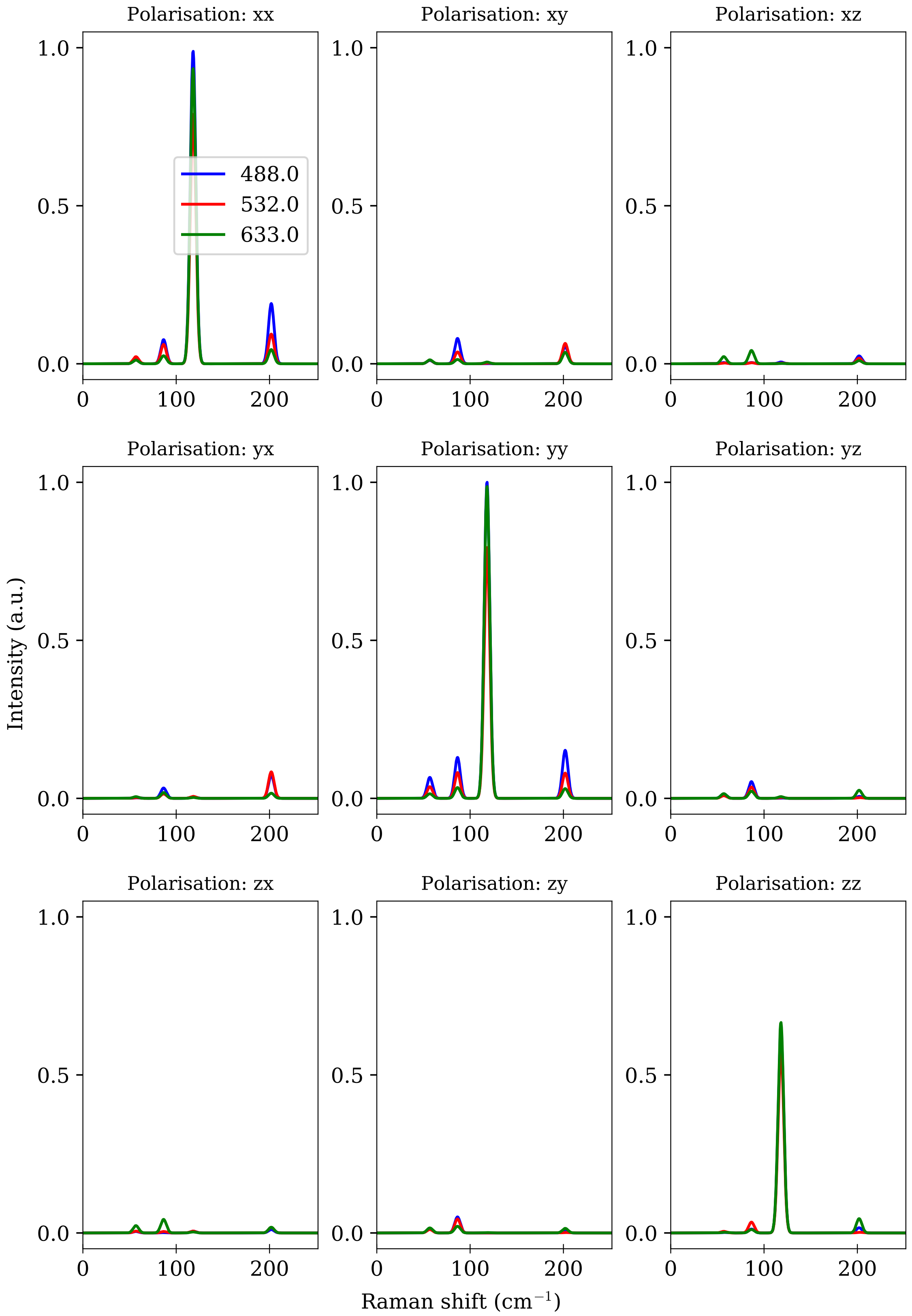

Supplementary Figure 101: Raman spectrum of BiTaSb with space group 156 and Wyckoff positions a-b-c-0.

BiTaSb, 156, a-c-0

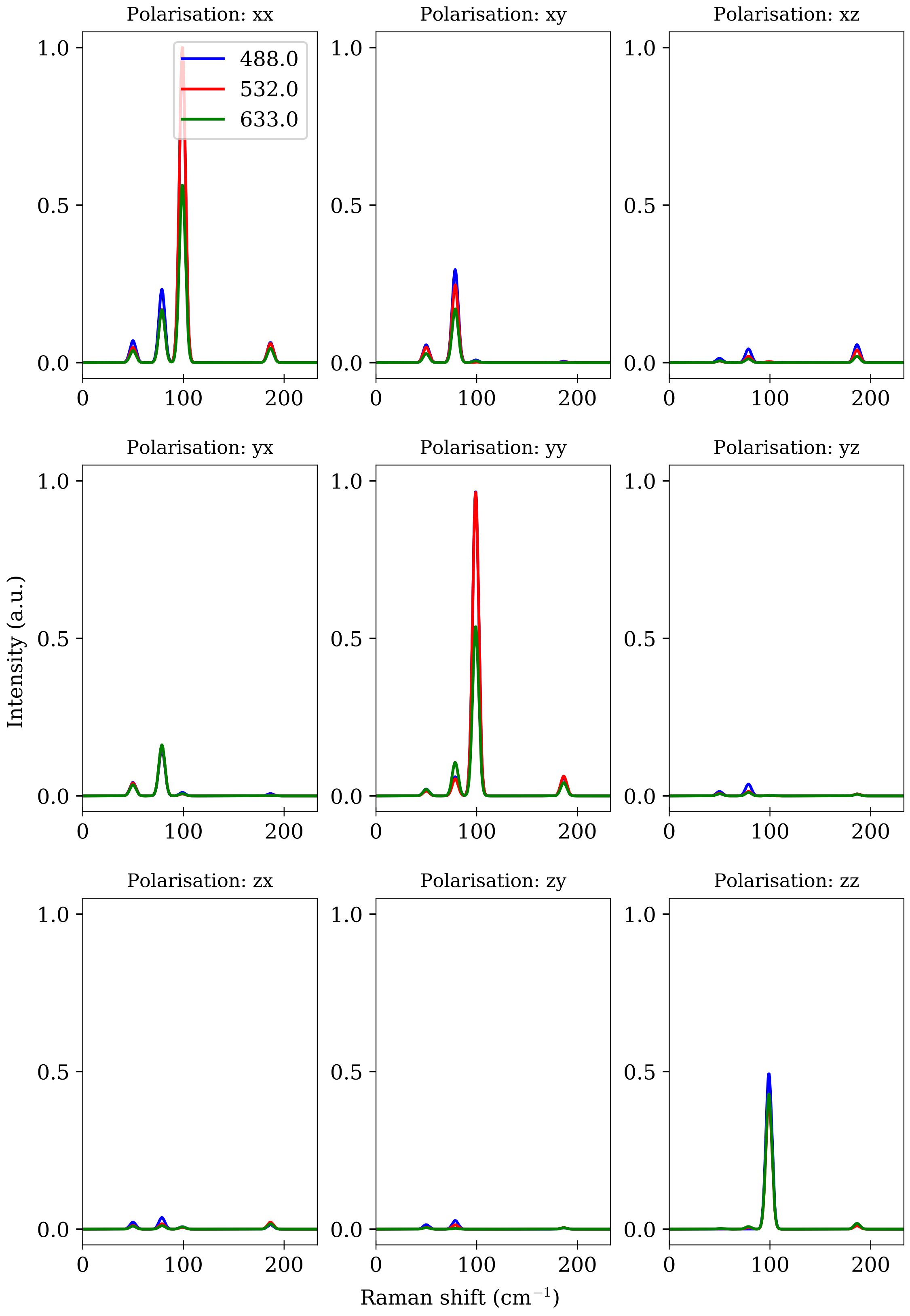

Supplementary Figure 102: Raman spectrum of BiTaSb with space group 156 and Wyckoff positions a-c-0.

BiTiAs, 156, a-b-c-0

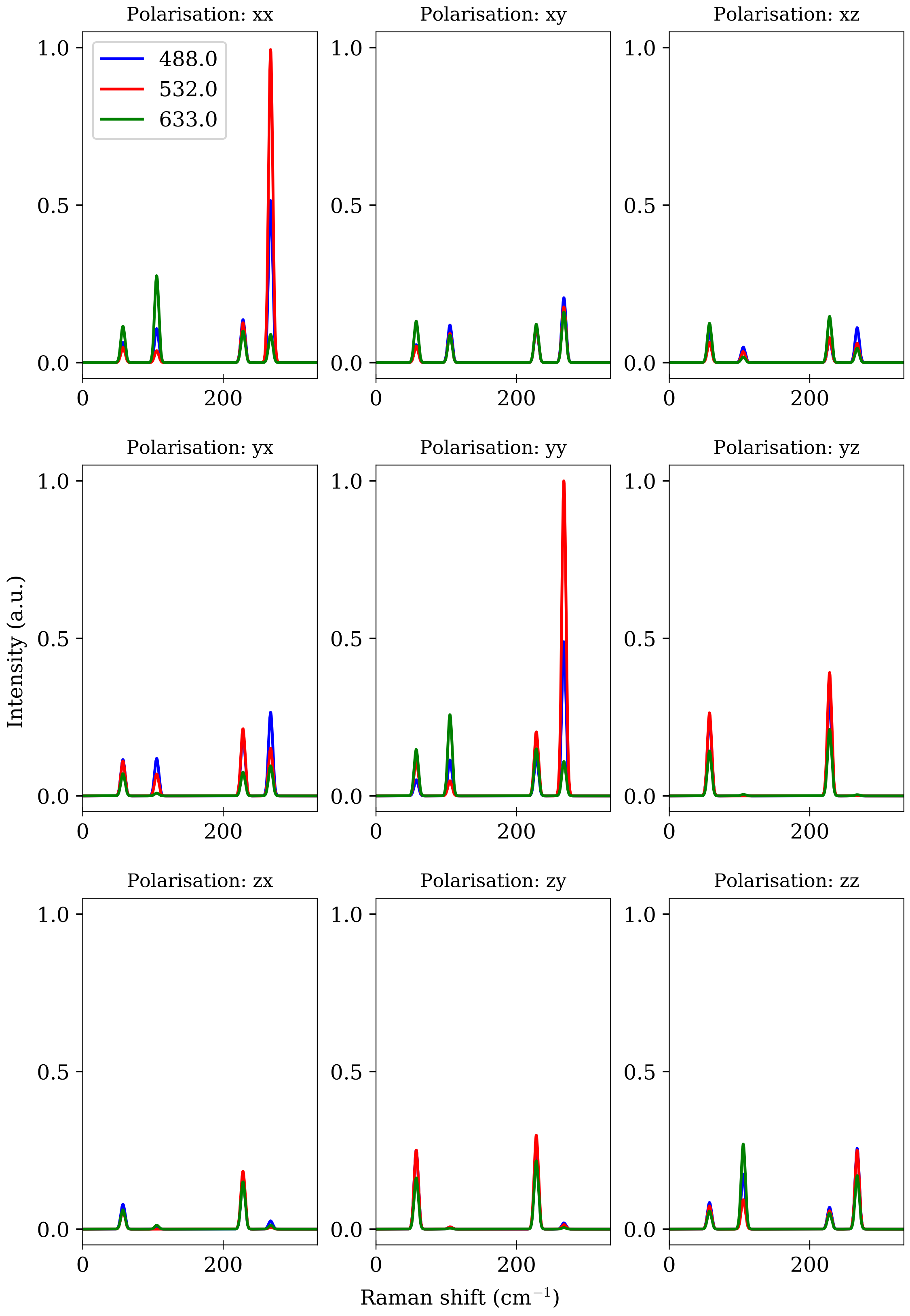

Supplementary Figure 103: Raman spectrum of BiTiAs with space group 156 and Wyckoff positions a-b-c-0.

BiTiSb, 156, a-b-c-0

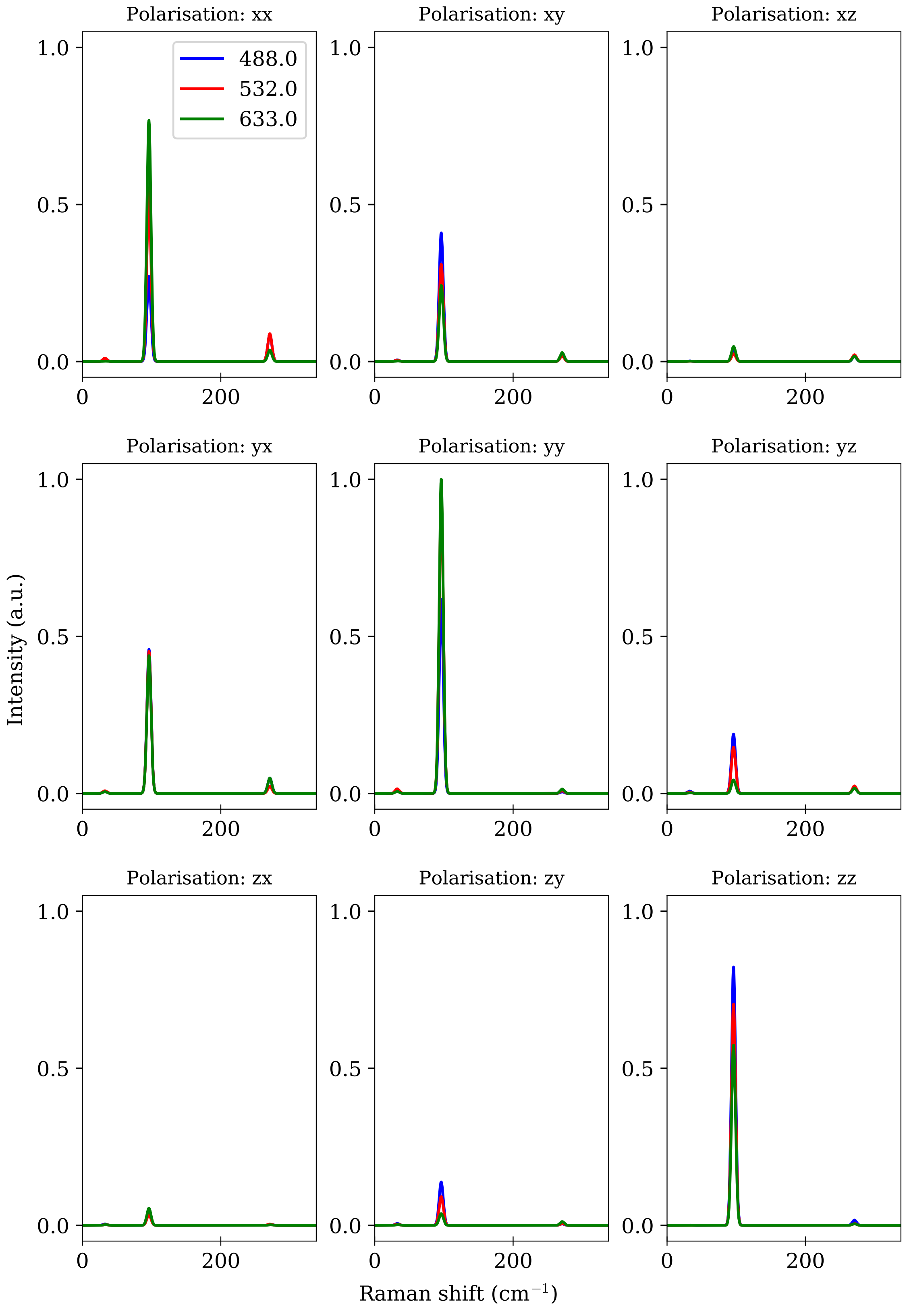

Supplementary Figure 104: Raman spectrum of BiTiSb with space group 156 and Wyckoff positions a-b-c-0.

BiTiSb, 156, a-c-0

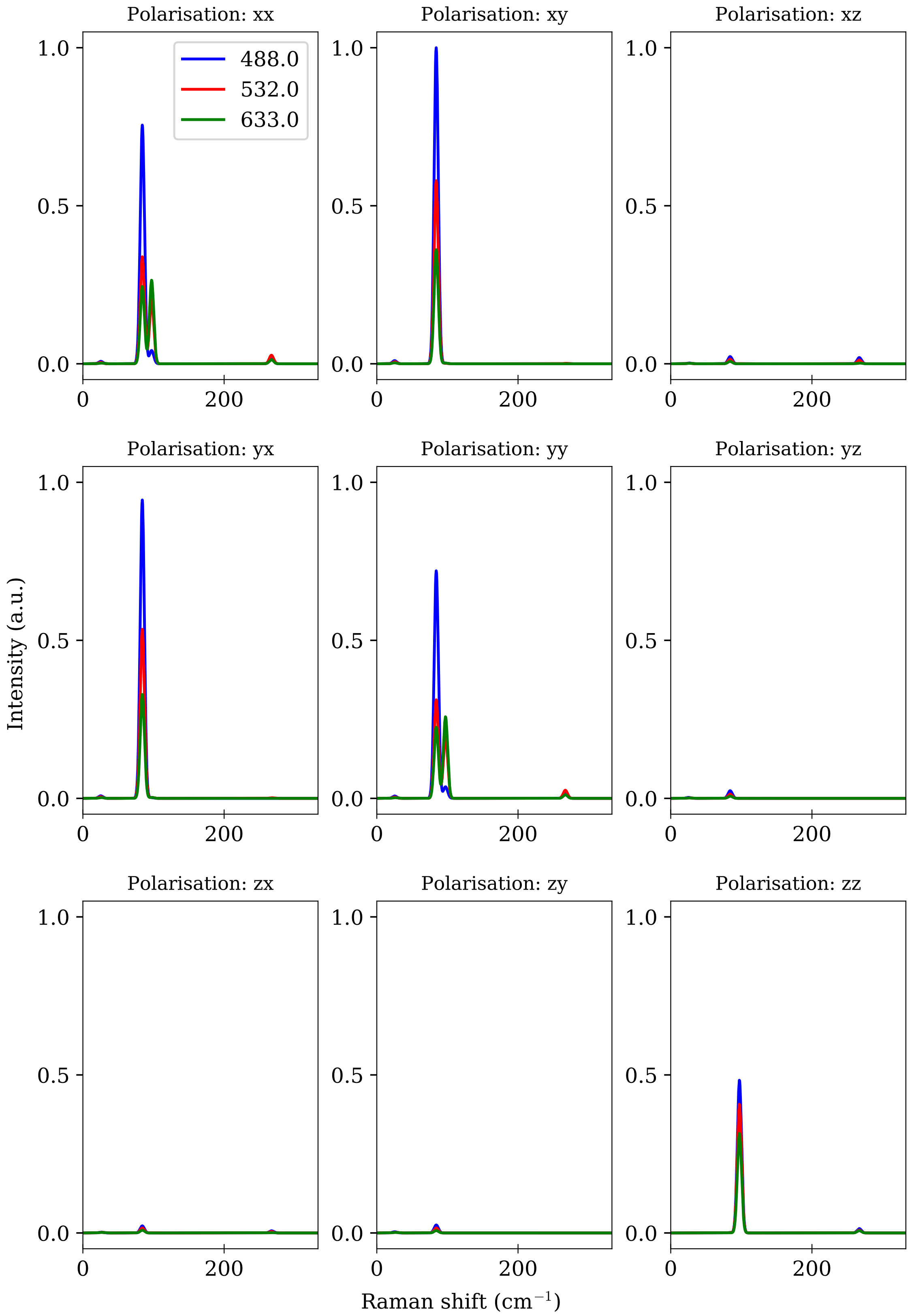

Supplementary Figure 105: Raman spectrum of BiTiSb with space group 156 and Wyckoff positions a-c-0.

BiVAs, 156, a-b-c-0

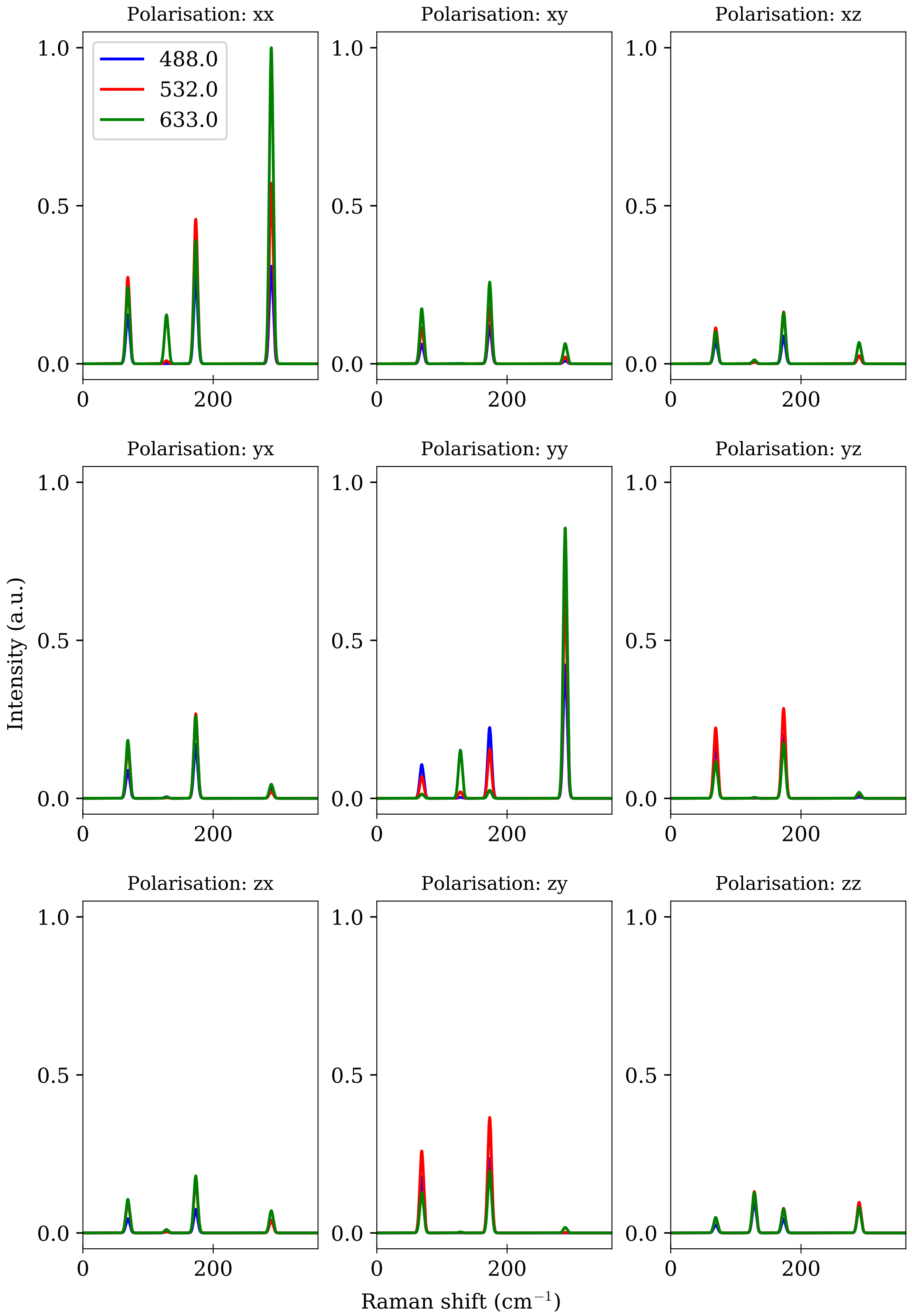

Supplementary Figure 106: Raman spectrum of BiVAs with space group 156 and Wyckoff positions a-b-c-0.

BiVAs, 156, a-c-0

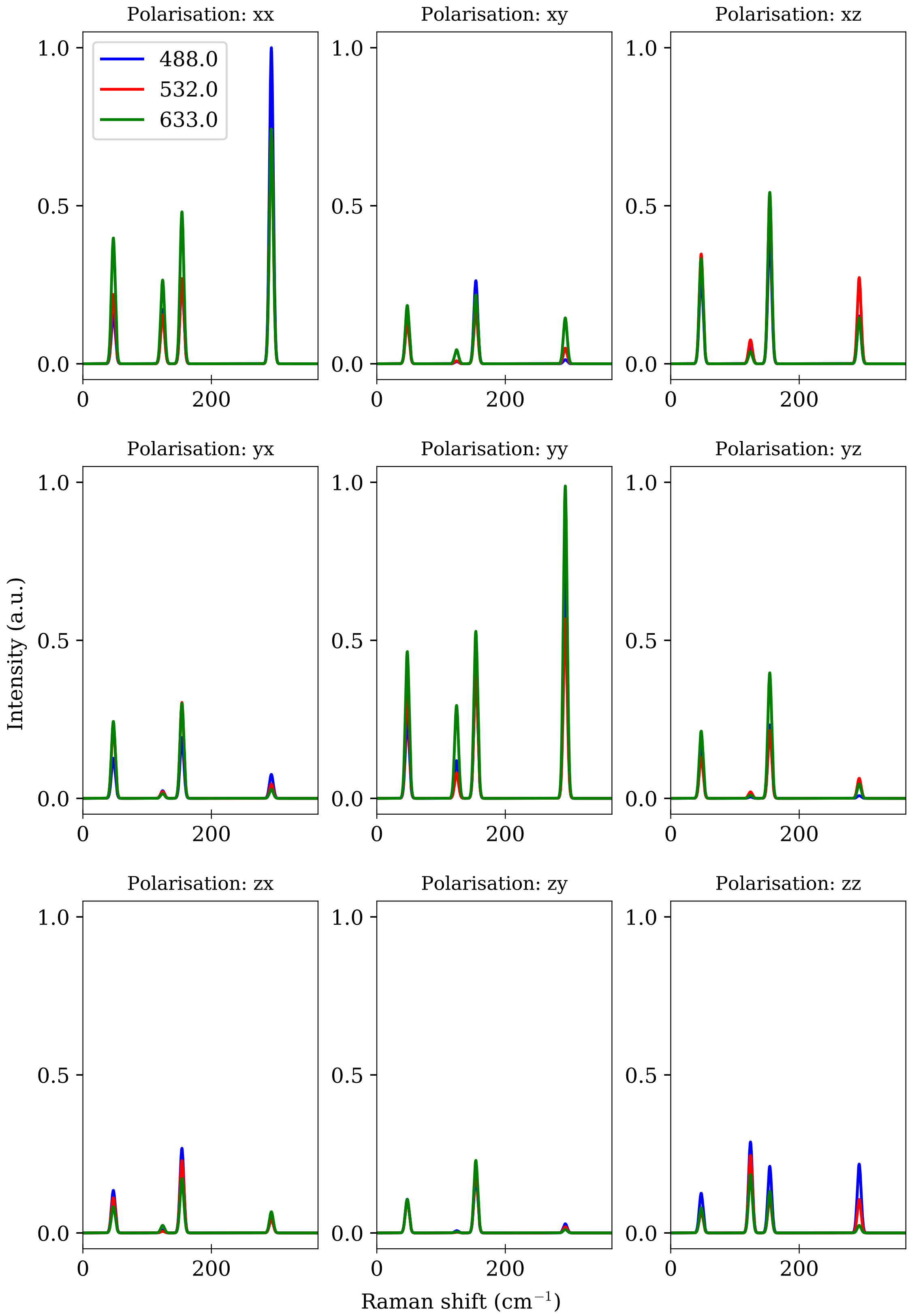

Supplementary Figure 107: Raman spectrum of BiVAs with space group 156 and Wyckoff positions a-c-0.

BiVSb, 156, a-b-c-0

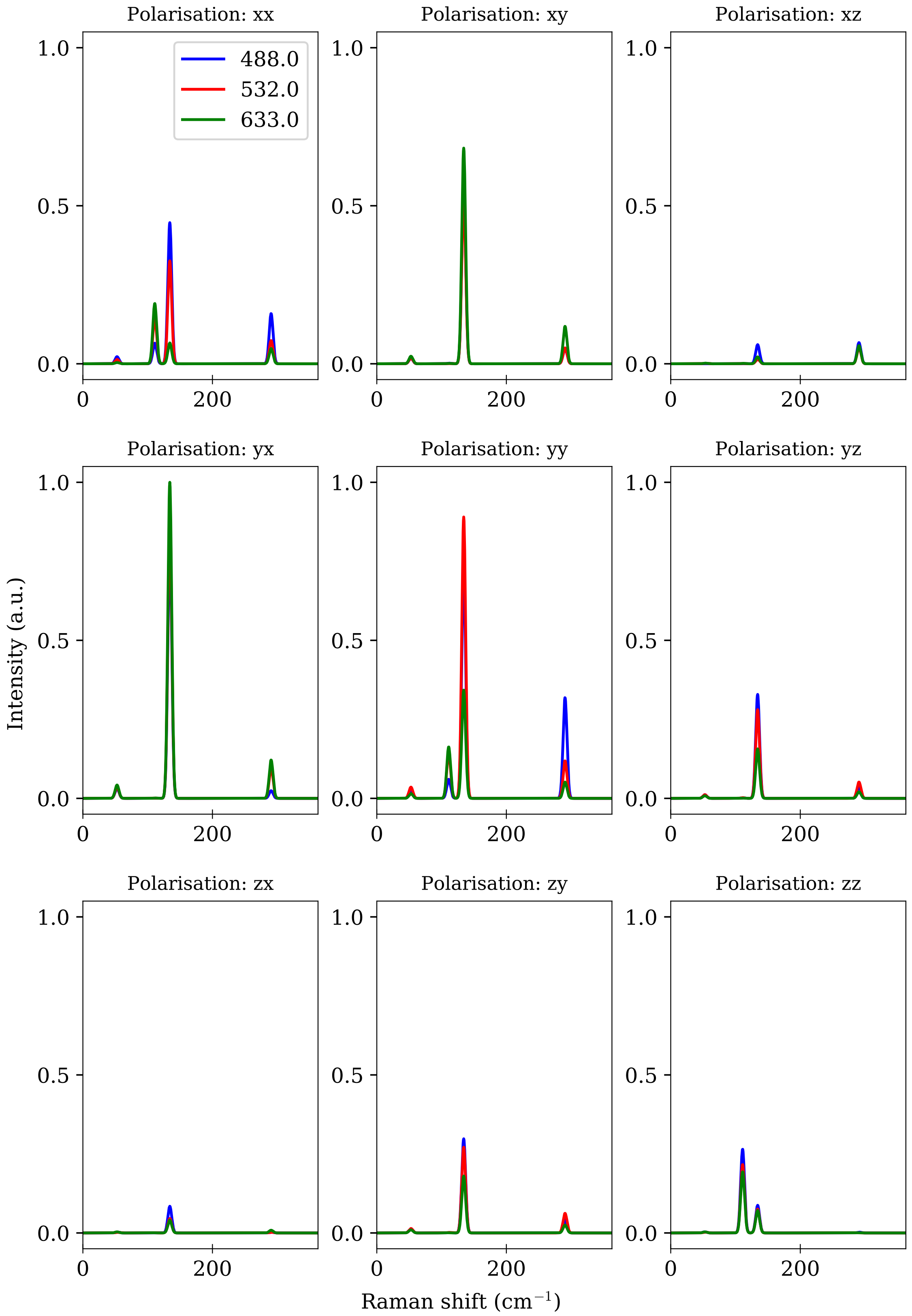

Supplementary Figure 108: Raman spectrum of BiVSb with space group 156 and Wyckoff positions a-b-c-0.

BiWAs, 156, a-b-c-0

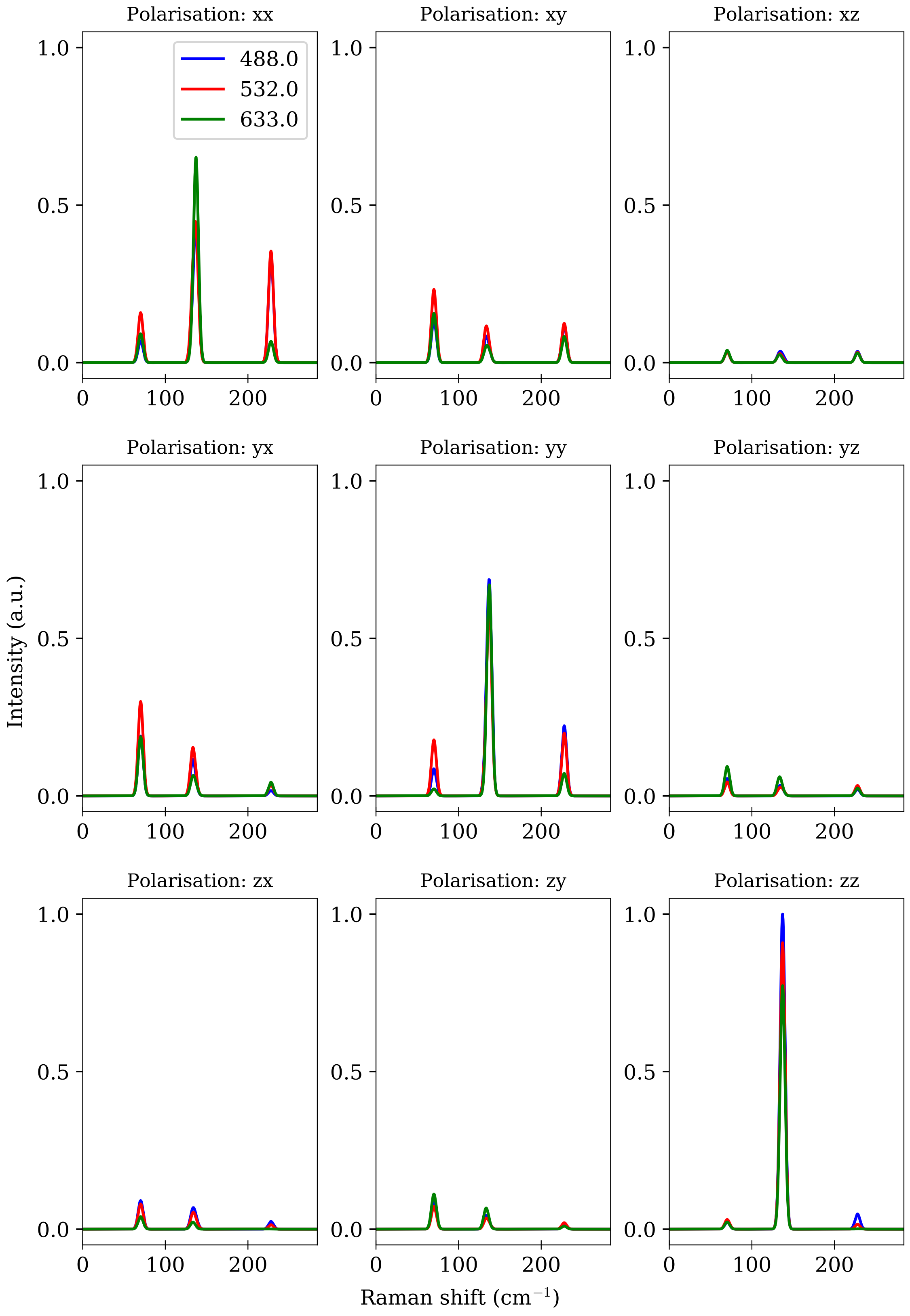

Supplementary Figure 109: Raman spectrum of BiWAs with space group 156 and Wyckoff positions a-b-c-0.

BiWSb, 156, a-b-c-0

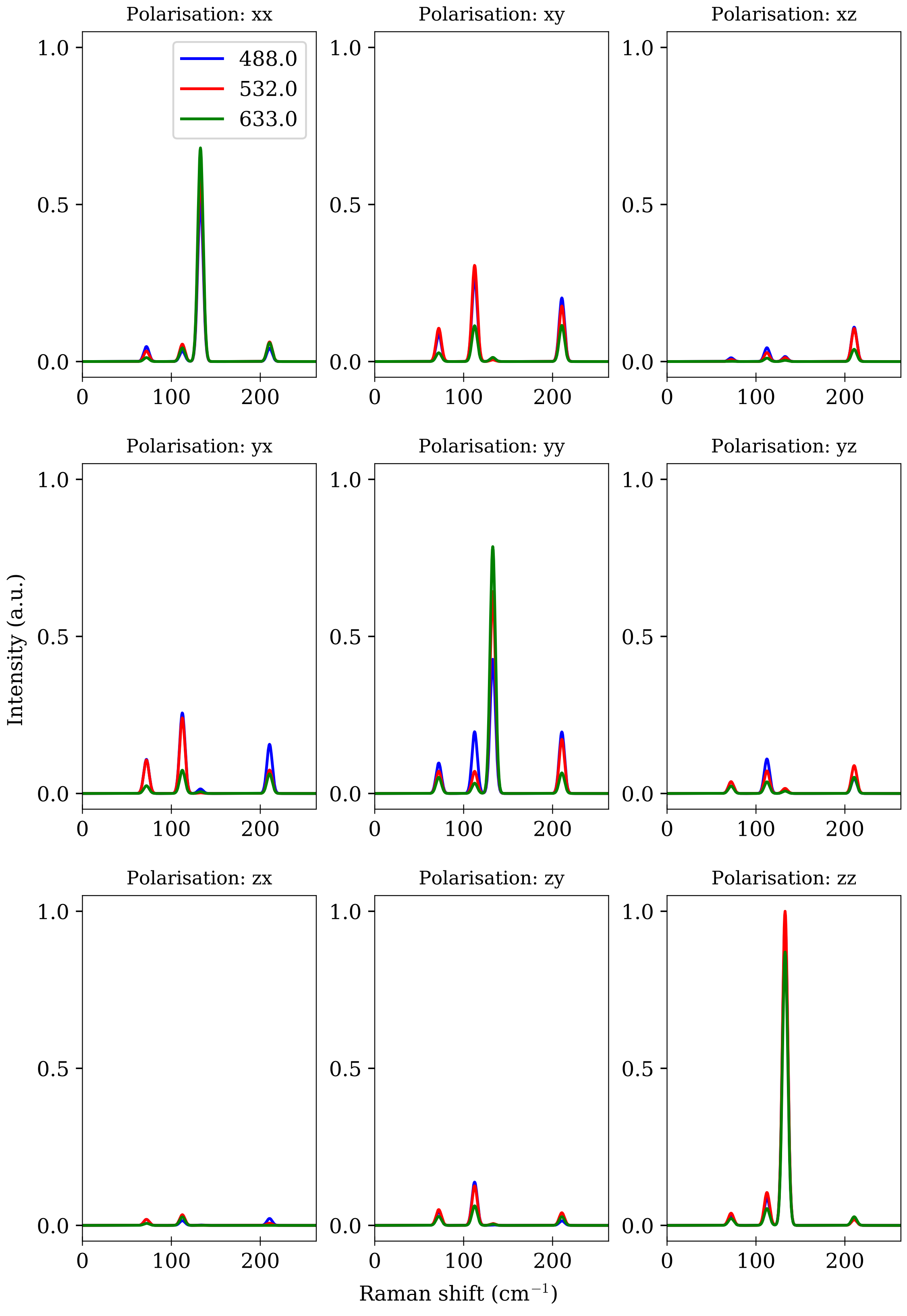

Supplementary Figure 110: Raman spectrum of BiWSb with space group 156 and Wyckoff positions a-b-c-0.

BiZrSb, 156, a-b-c-0

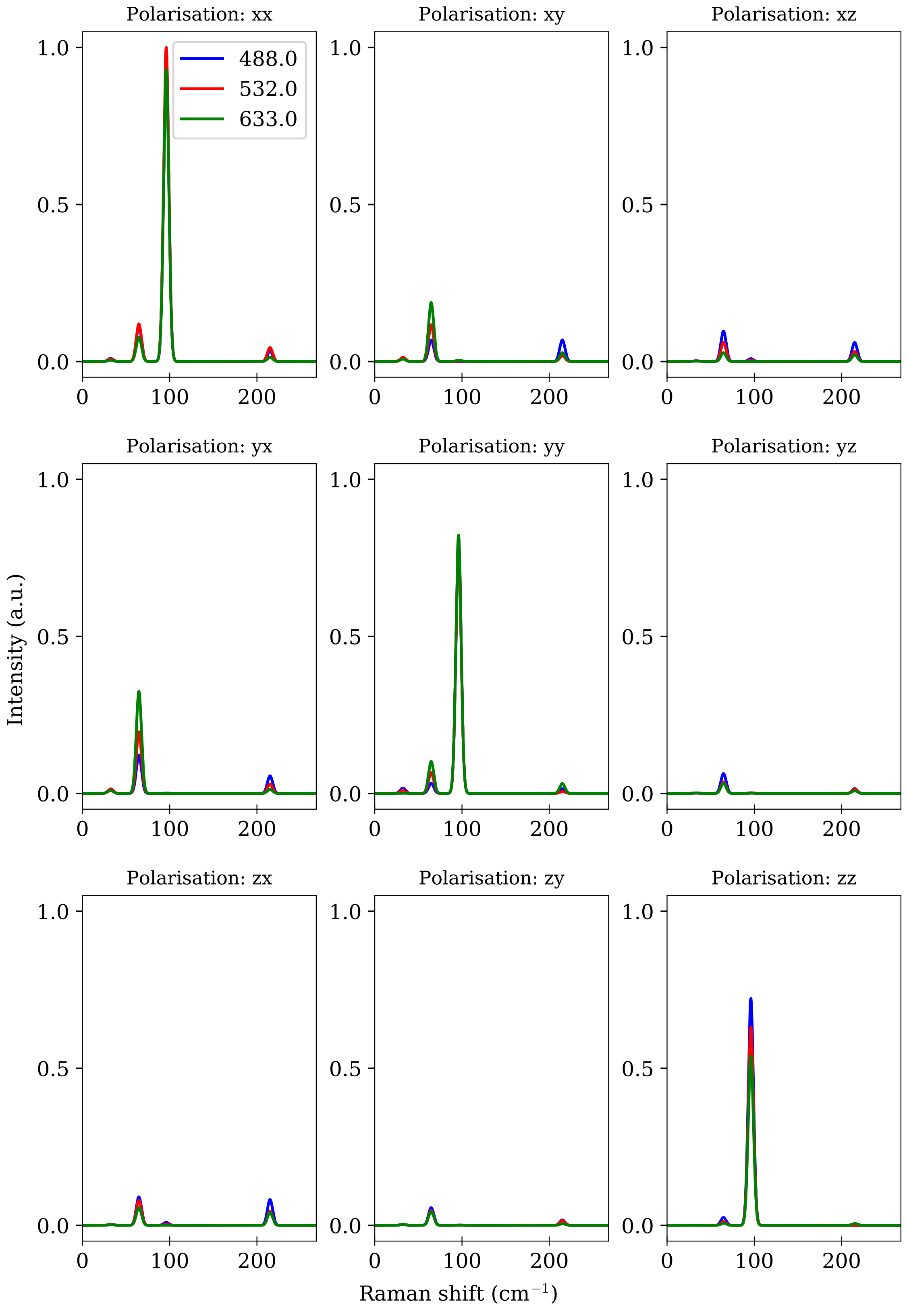

Supplementary Figure 111: Raman spectrum of BiZrSb with space group 156 and Wyckoff positions a-b-c-0.

BiZrSb, 156, a-c-0

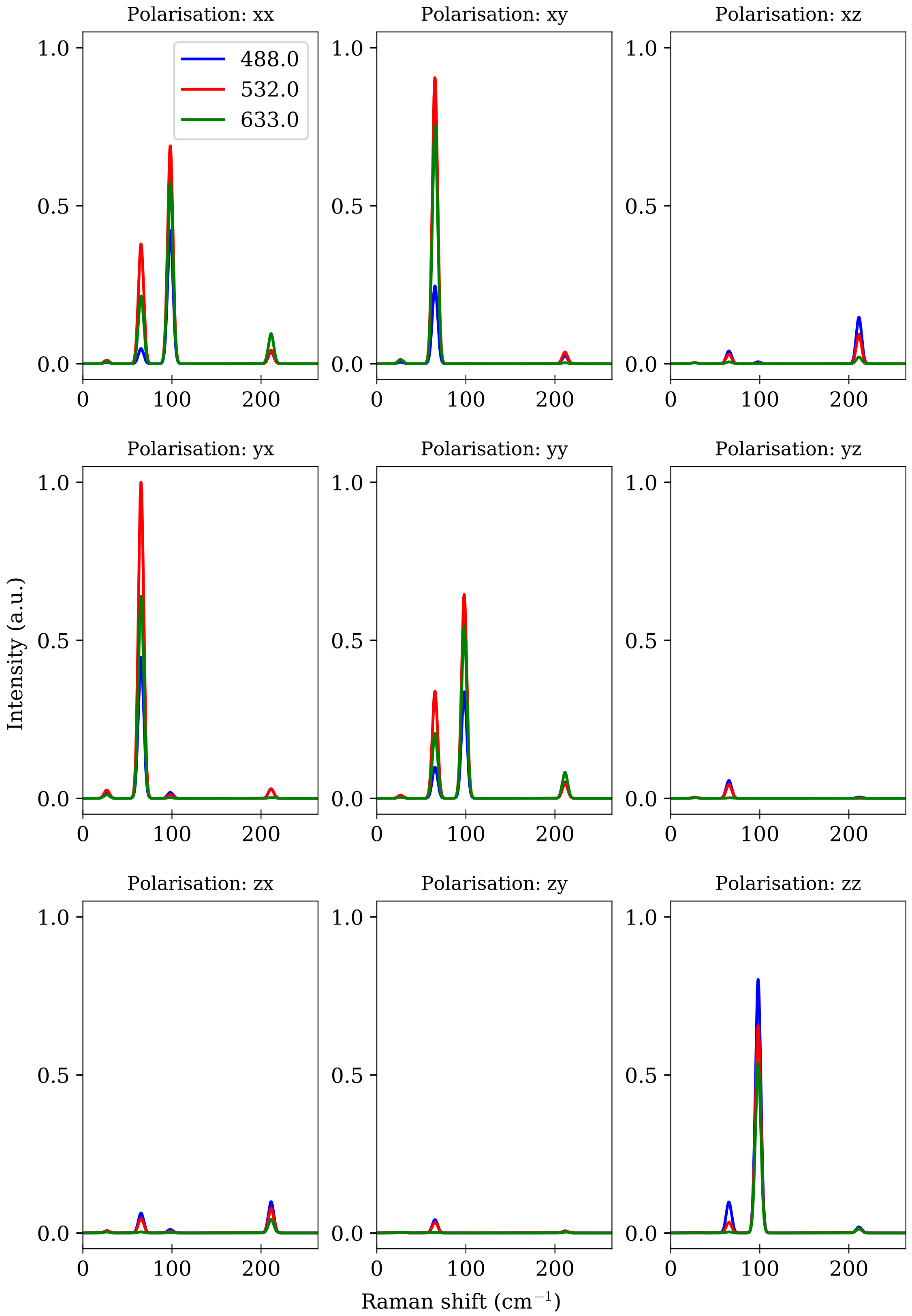

Supplementary Figure 112: Raman spectrum of BiZrSb with space group 156 and Wyckoff positions a-c-0.

BN, 187, d-f-0

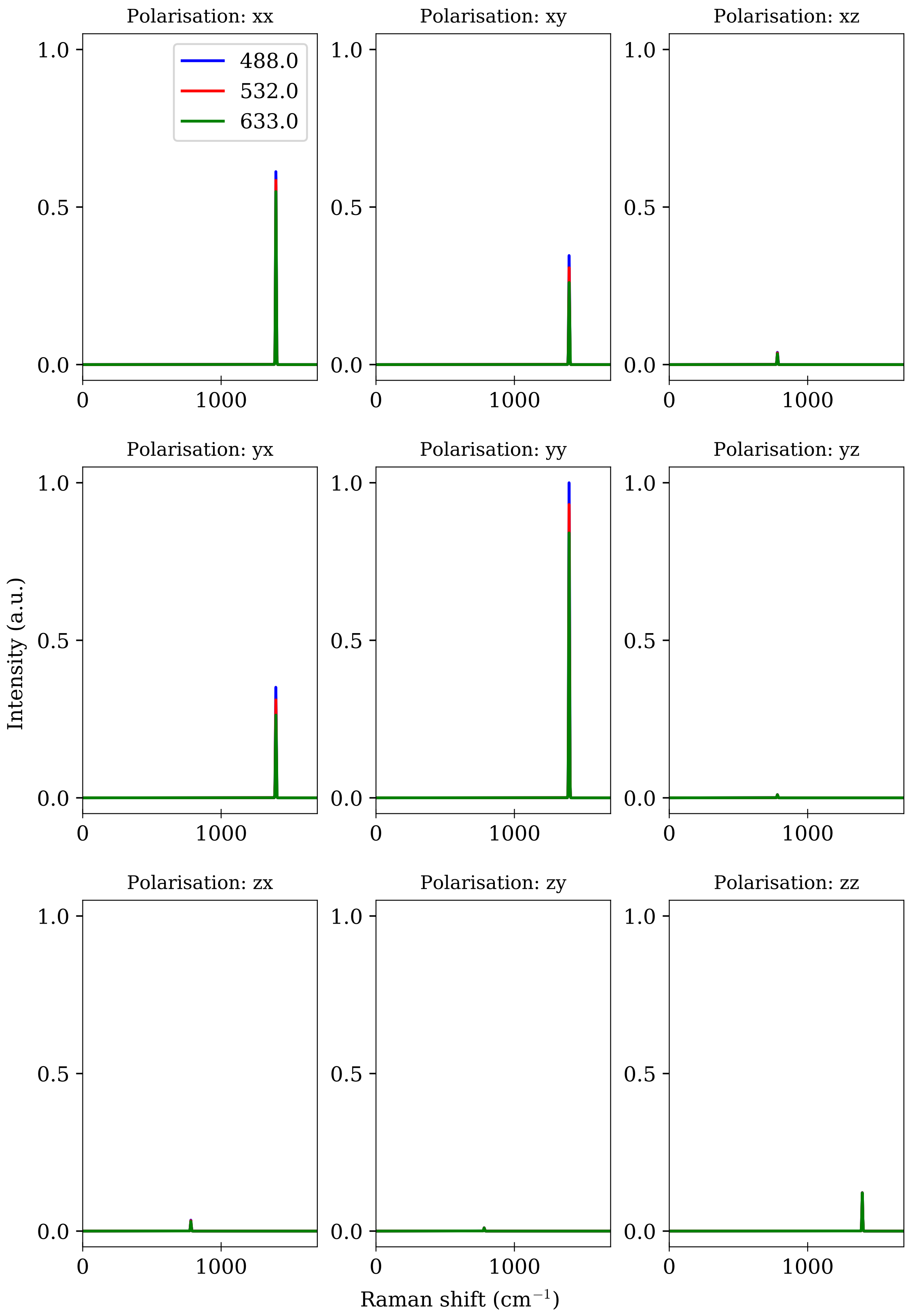

Supplementary Figure 113: Raman spectrum of BN with space group 187 and Wyckoff positions d-f-0.

BP, 187, d-f-0

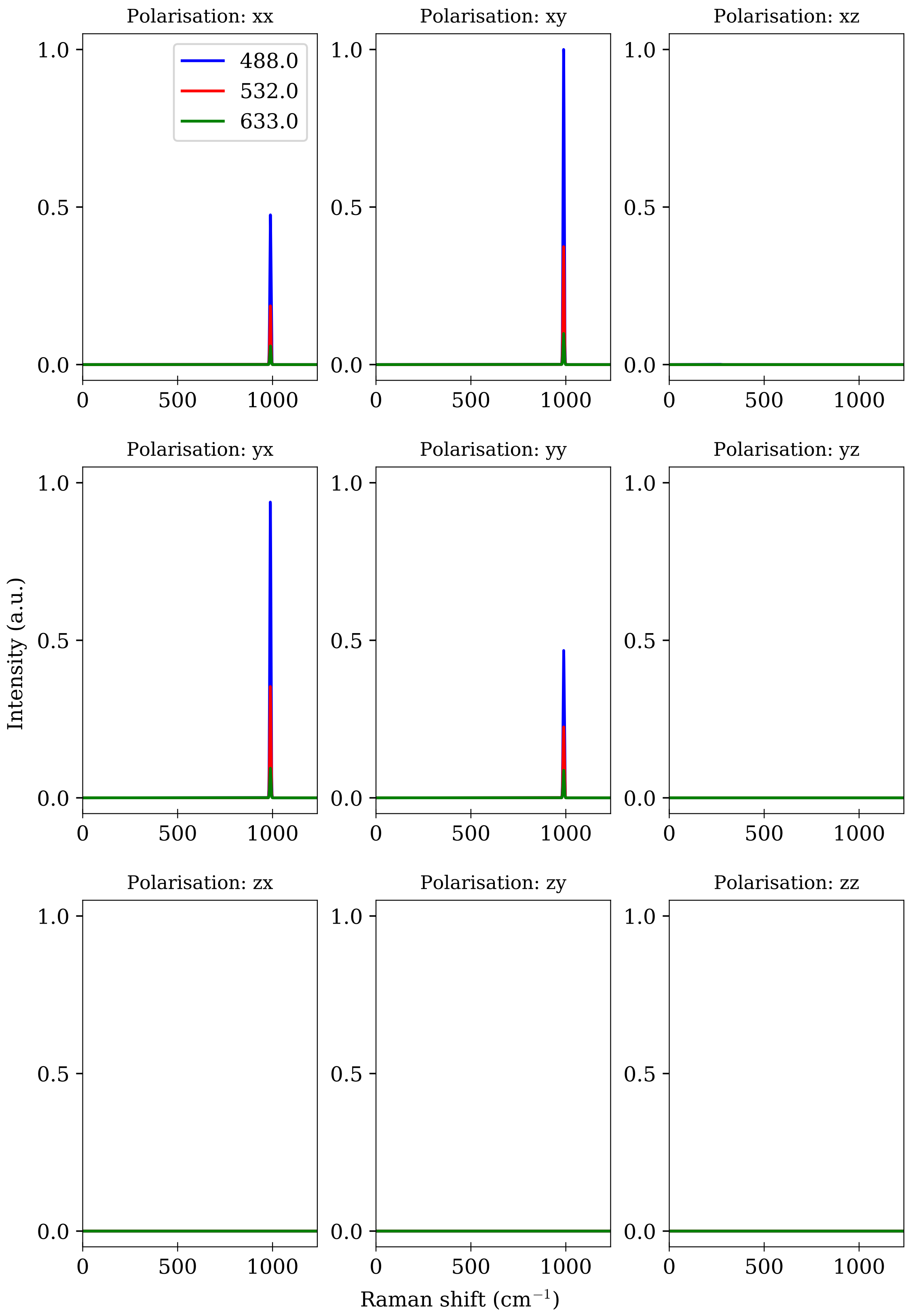

Supplementary Figure 114: Raman spectrum of BP with space group 187 and Wyckoff positions d-f-0.

$Br_2Ge_2N_2$, 59, a-b-0

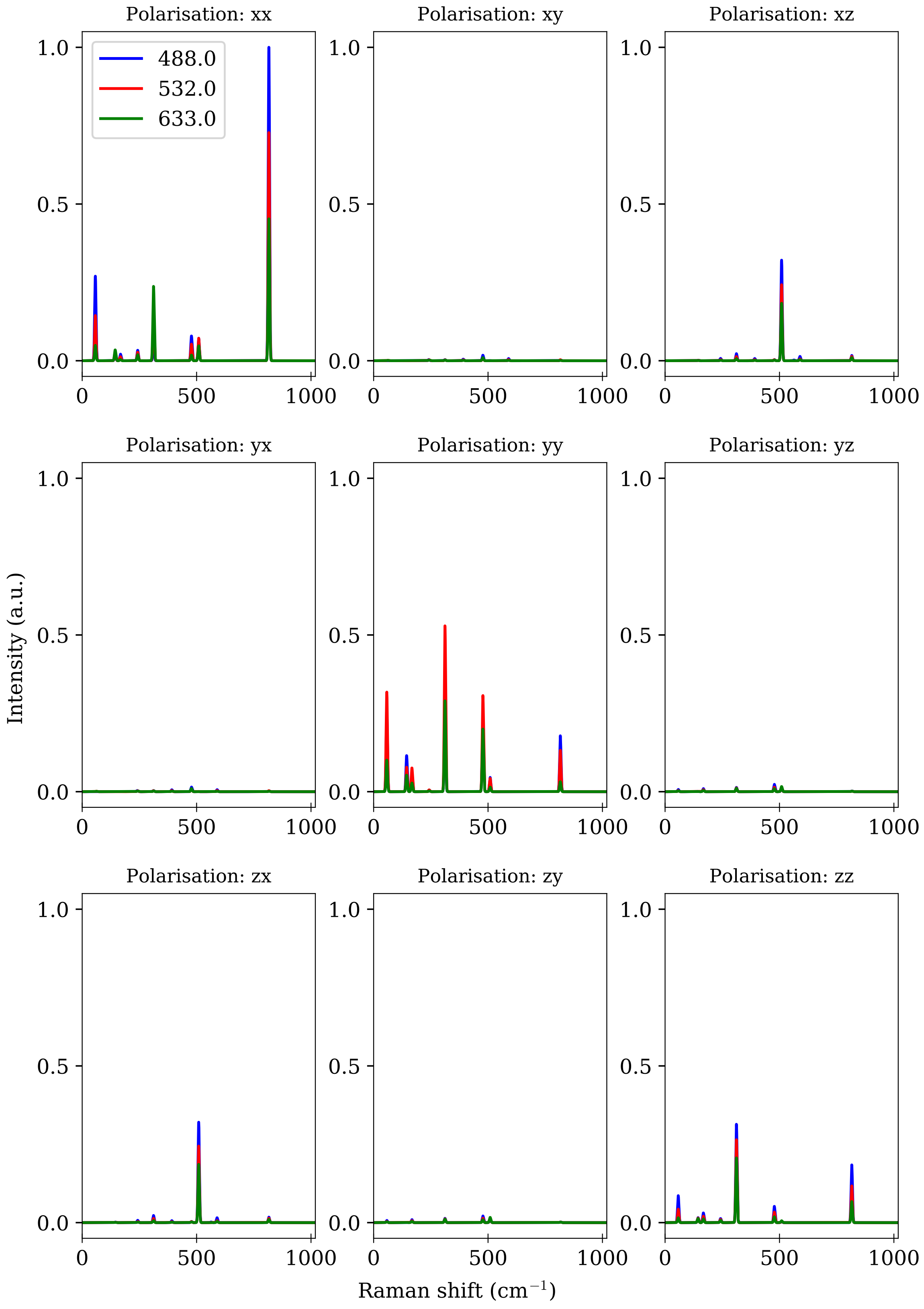

Supplementary Figure 115: Raman spectrum of $Br_2Ge_2N_2$ with space group 59 and Wyckoff positions a-b-0.

Br_2Sb_2, 2, i-0

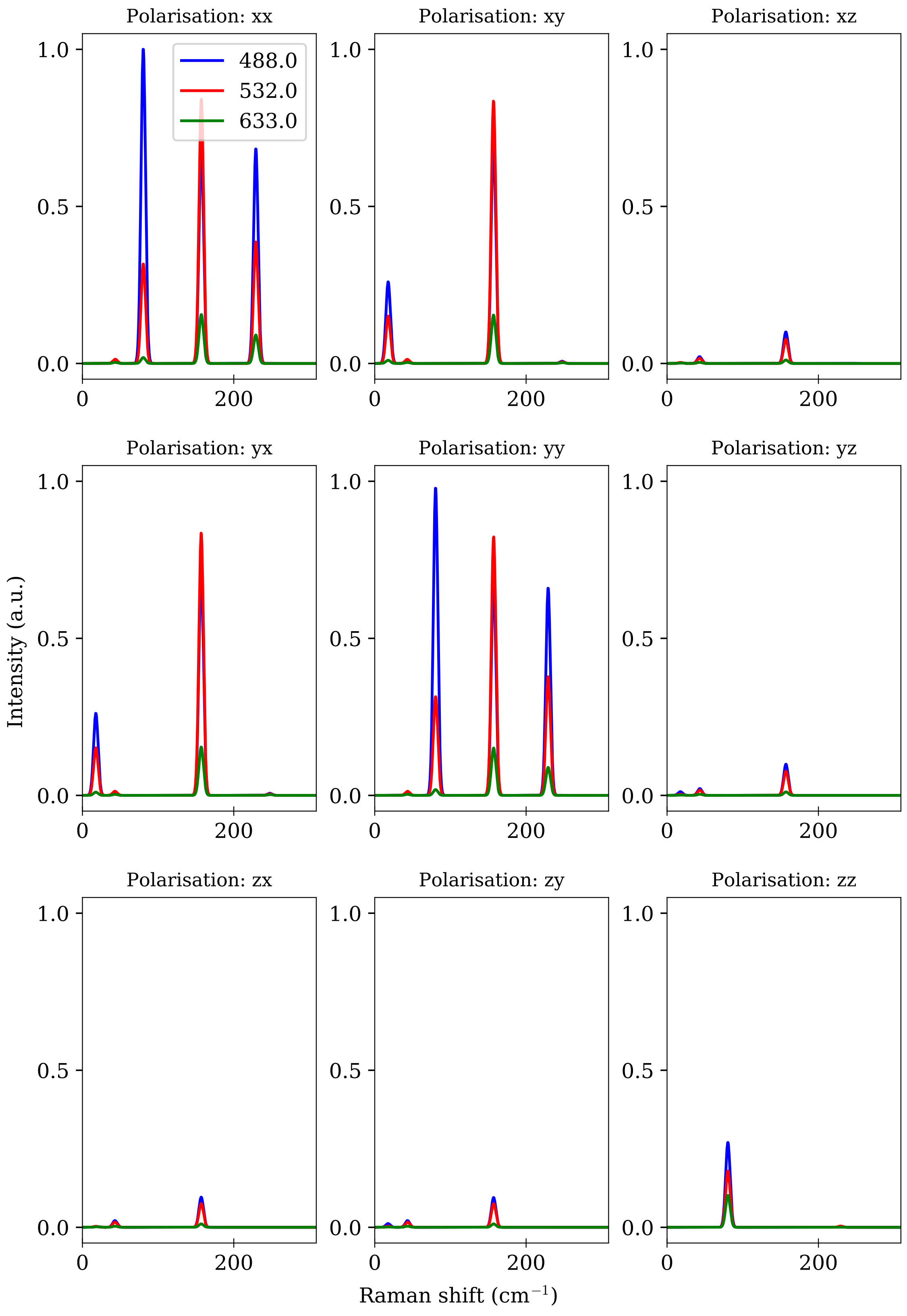

Supplementary Figure 116: Raman spectrum of Br_2Sb_2 with space group 2 and Wyckoff positions i-0.

BrSbSe, 156, a-b-c-0

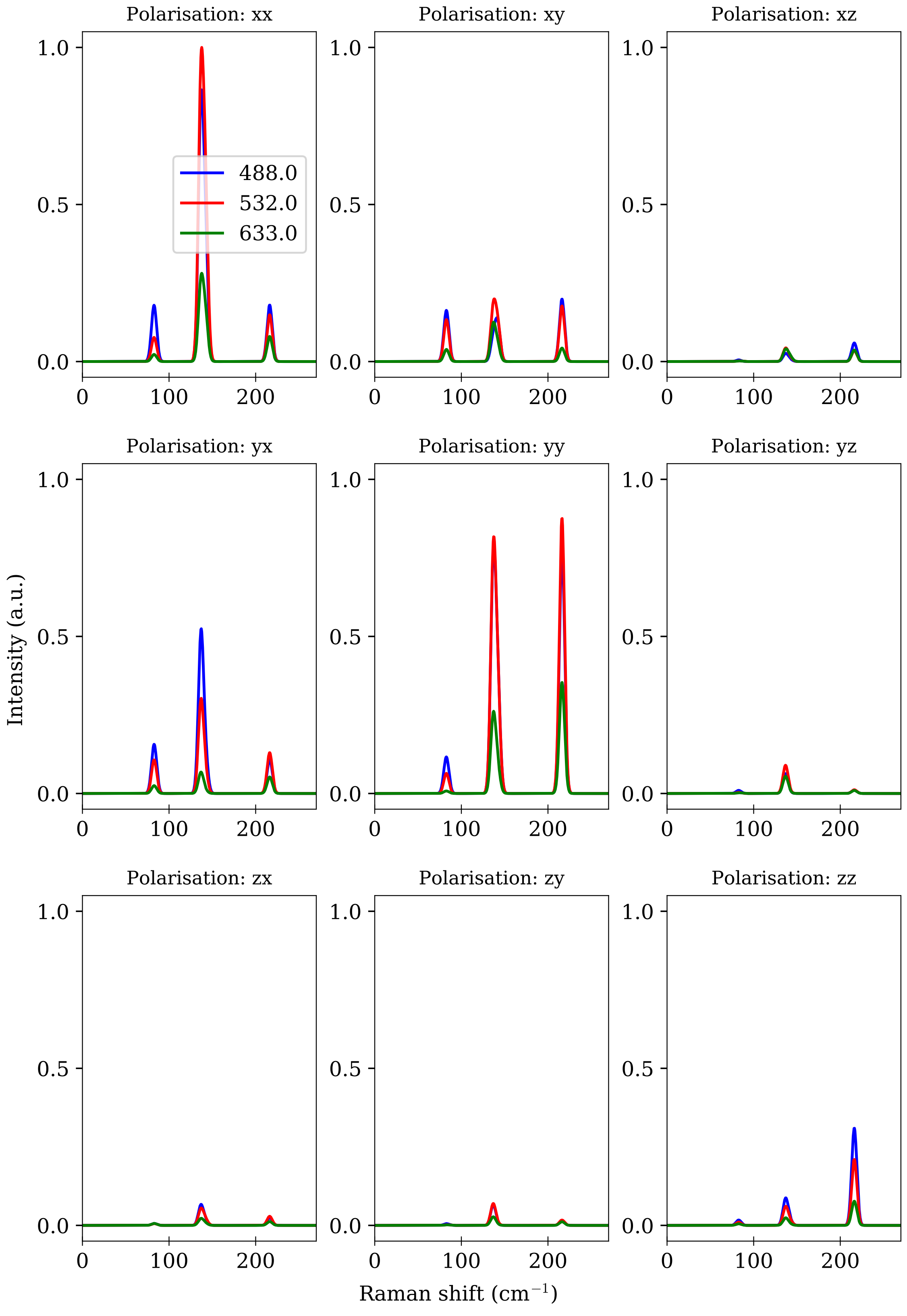

Supplementary Figure 117: Raman spectrum of BrSbSe with space group 156 and Wyckoff positions a-b-c-0.

BrSbSe, 156, a-c-0

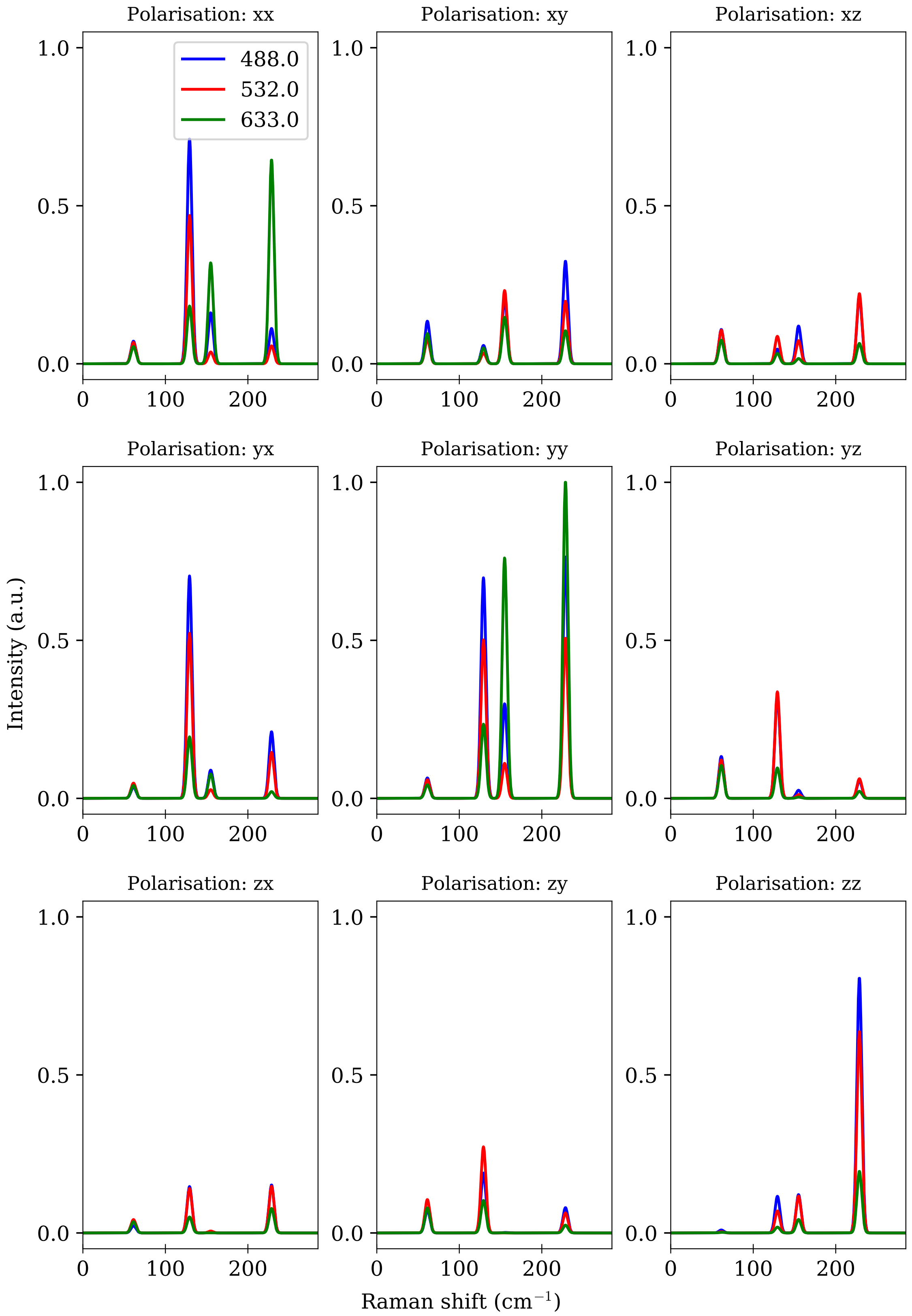

Supplementary Figure 118: Raman spectrum of BrSbSe with space group 156 and Wyckoff positions a-c-0.

BrSbTe, 156, a-b-c-0

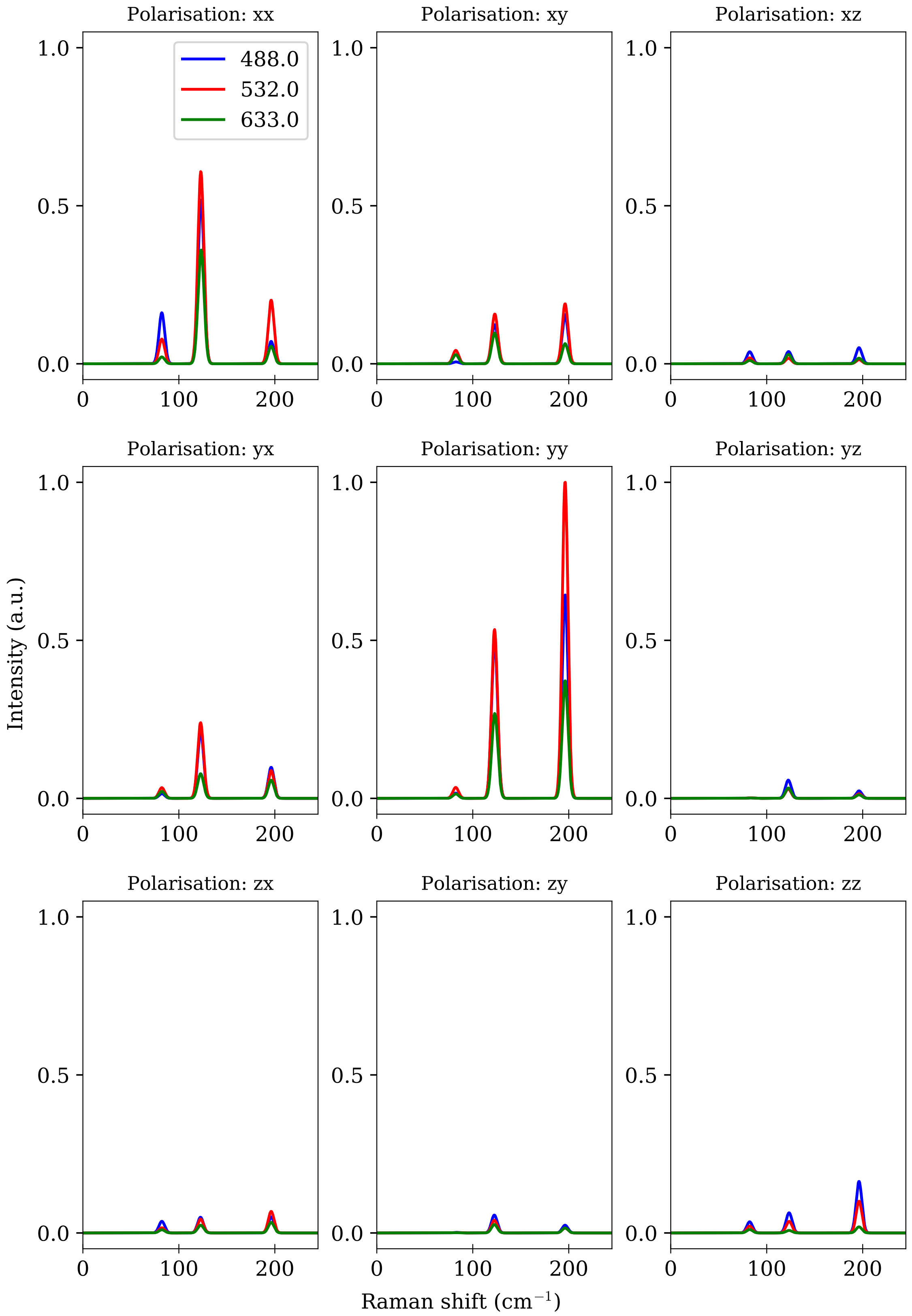

Supplementary Figure 119: Raman spectrum of BrSbTe with space group 156 and Wyckoff positions a-b-c-0.

BrSbTe, 156, a-c-0

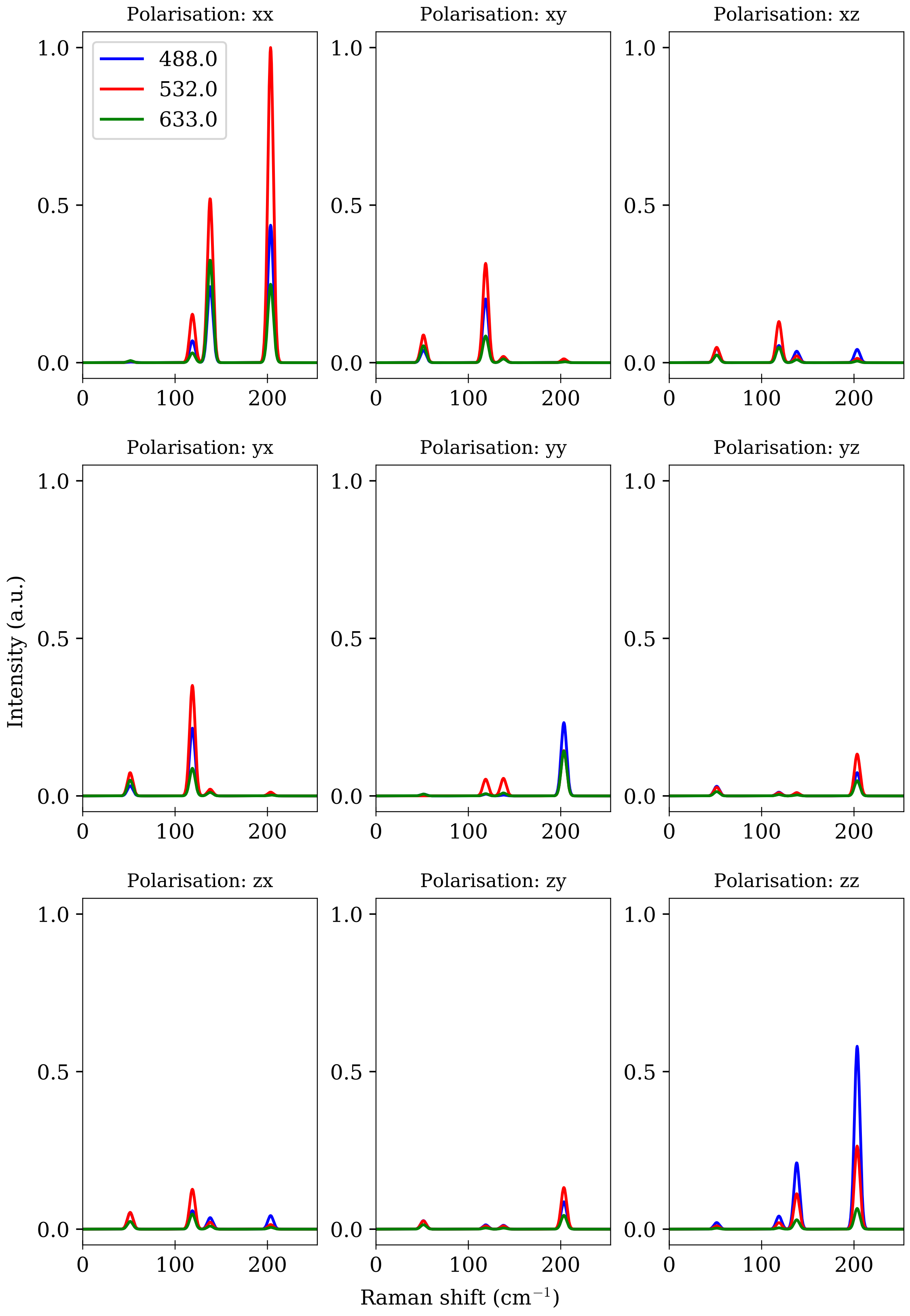

Supplementary Figure 120: Raman spectrum of BrSbTe with space group 156 and Wyckoff positions a-c-0.

BrSSb, 156, a-b-c-0

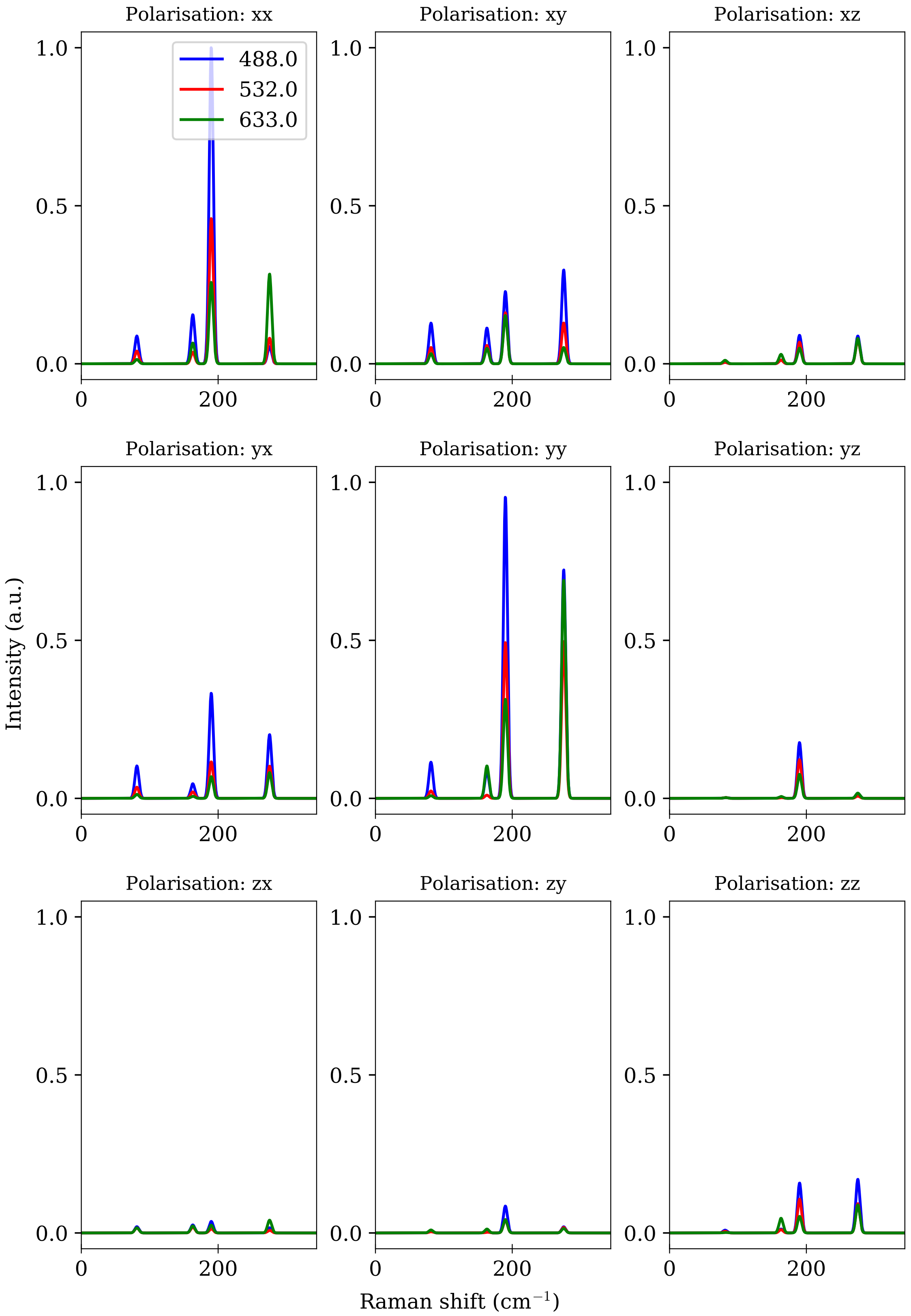

Supplementary Figure 121: Raman spectrum of BrSSb with space group 156 and Wyckoff positions a-b-c-0.

BrSSb, 156, a-c-0

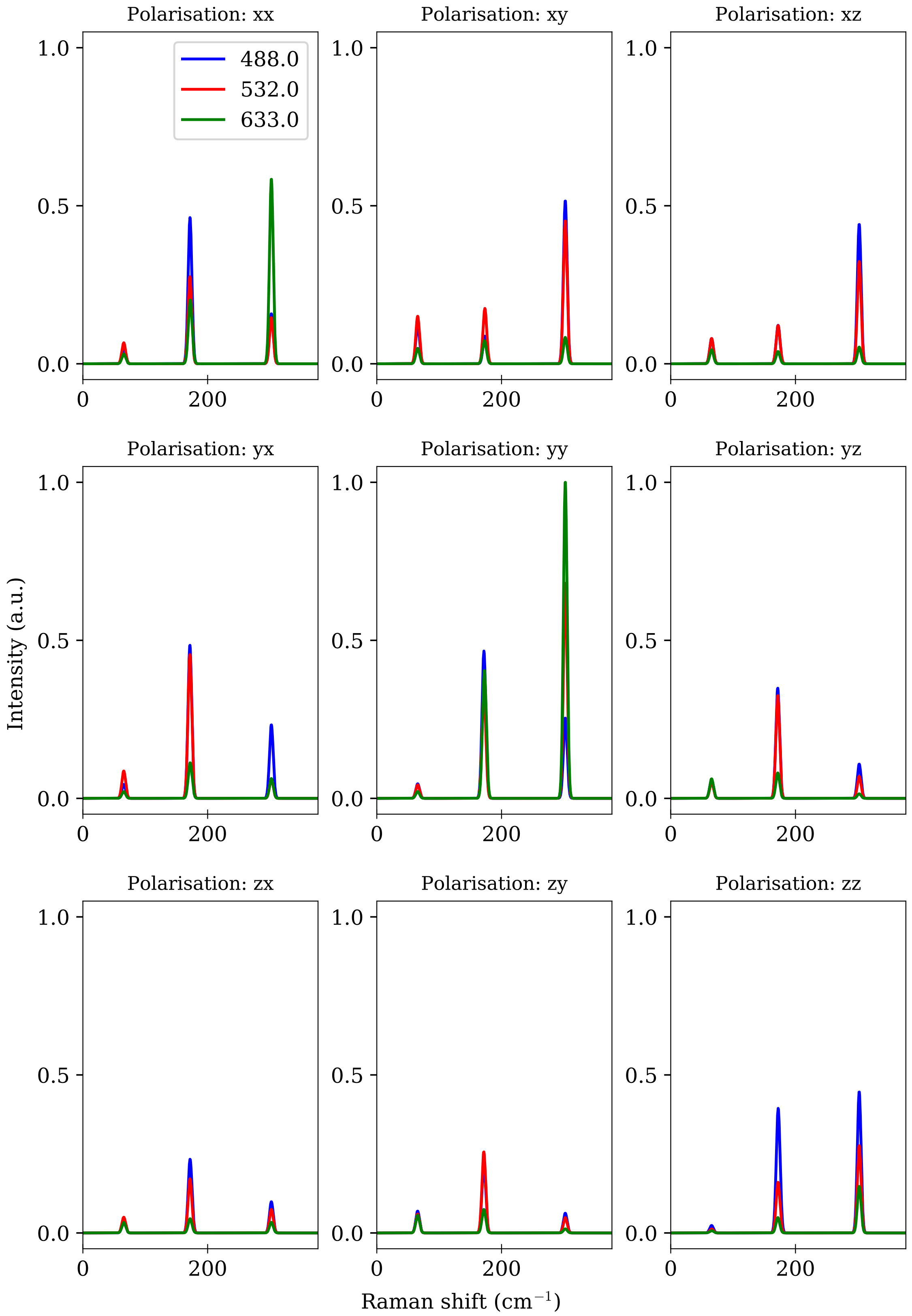

Supplementary Figure 122: Raman spectrum of BrSSb with space group 156 and Wyckoff positions a-c-0.

BSb, 187, d-f-0

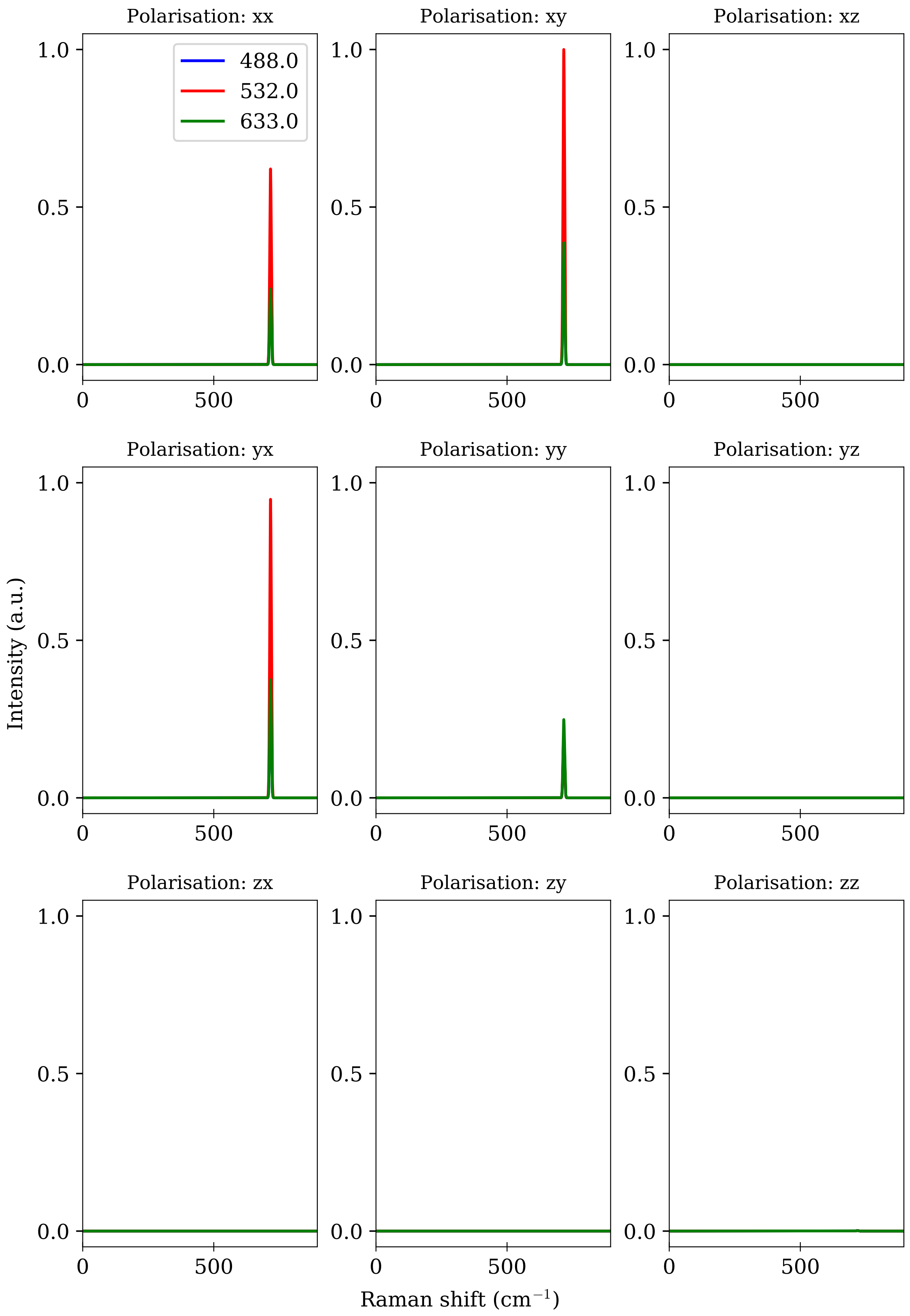

Supplementary Figure 123: Raman spectrum of BSb with space group 187 and Wyckoff positions d-f-0.

C_2F_2, 164, d-0

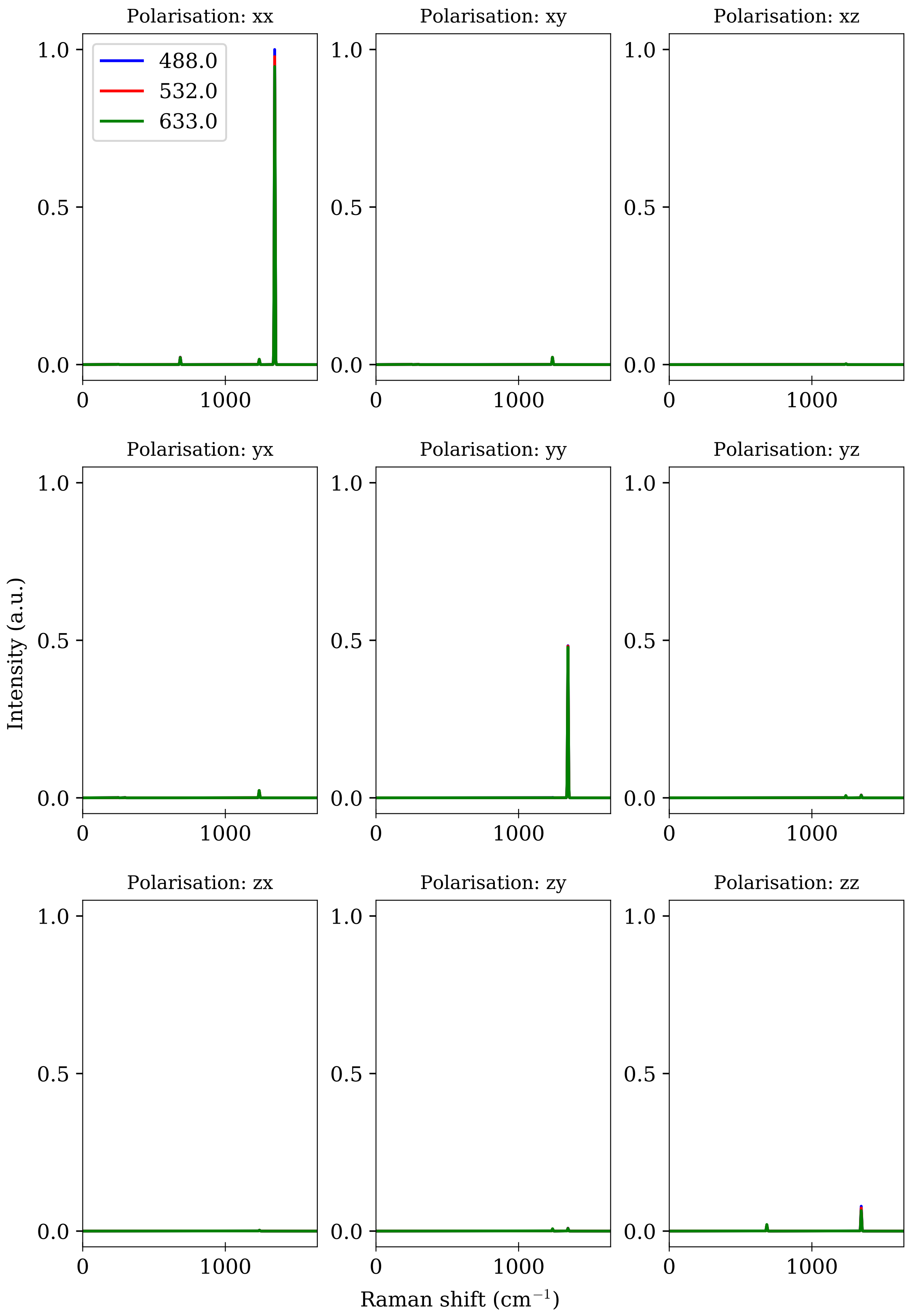

Supplementary Figure 124: Raman spectrum of C_2F_2 with space group 164 and Wyckoff positions d-0.

C_2H_2, 164, d-0

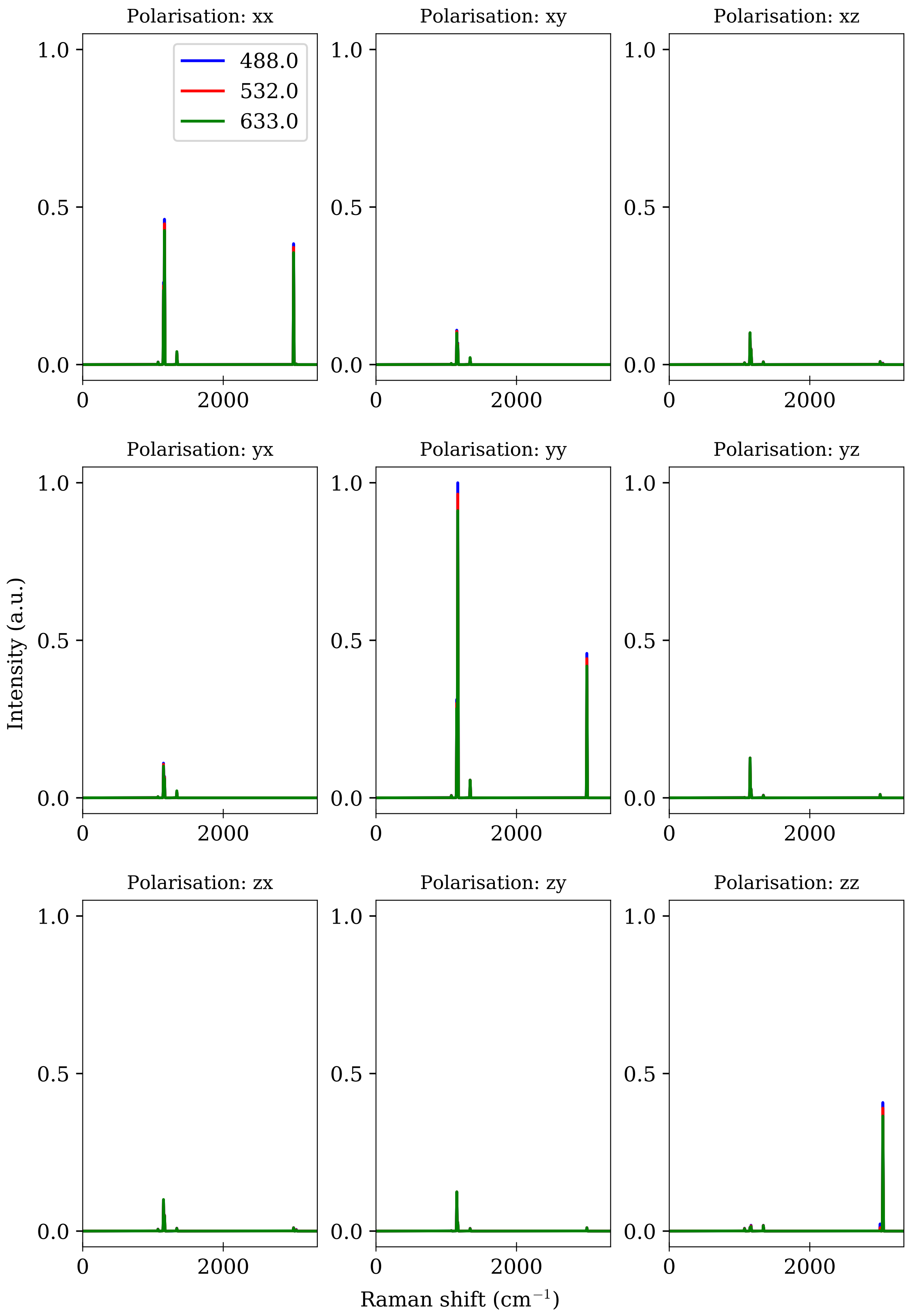

Supplementary Figure 125: Raman spectrum of C_2H_2 with space group 164 and Wyckoff positions d-0.

C_2, 191, d-0

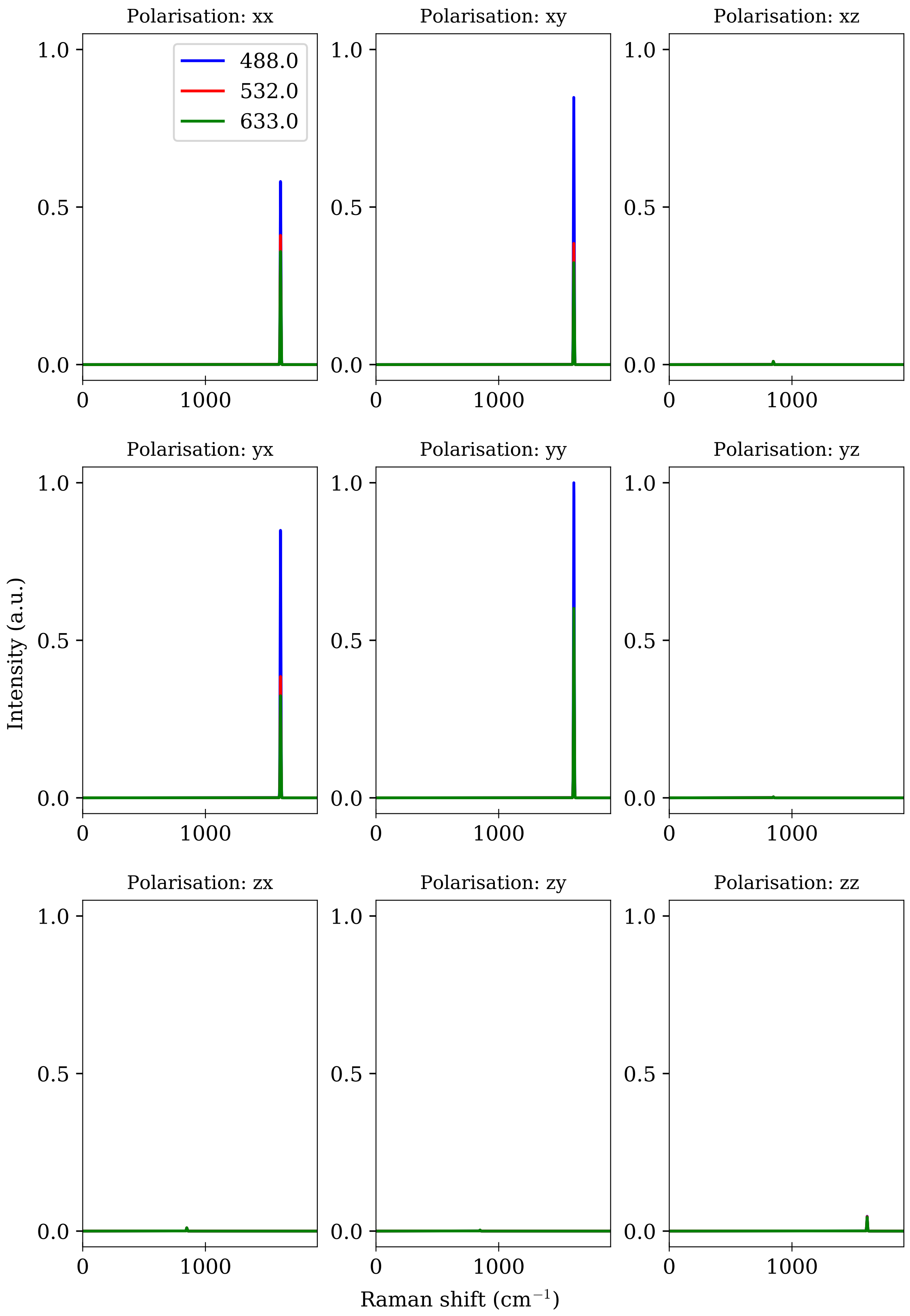

Supplementary Figure 126: Raman spectrum of C_2 with space group 191 and Wyckoff positions d-0.

$CaBr_2$, 164, b-d-0

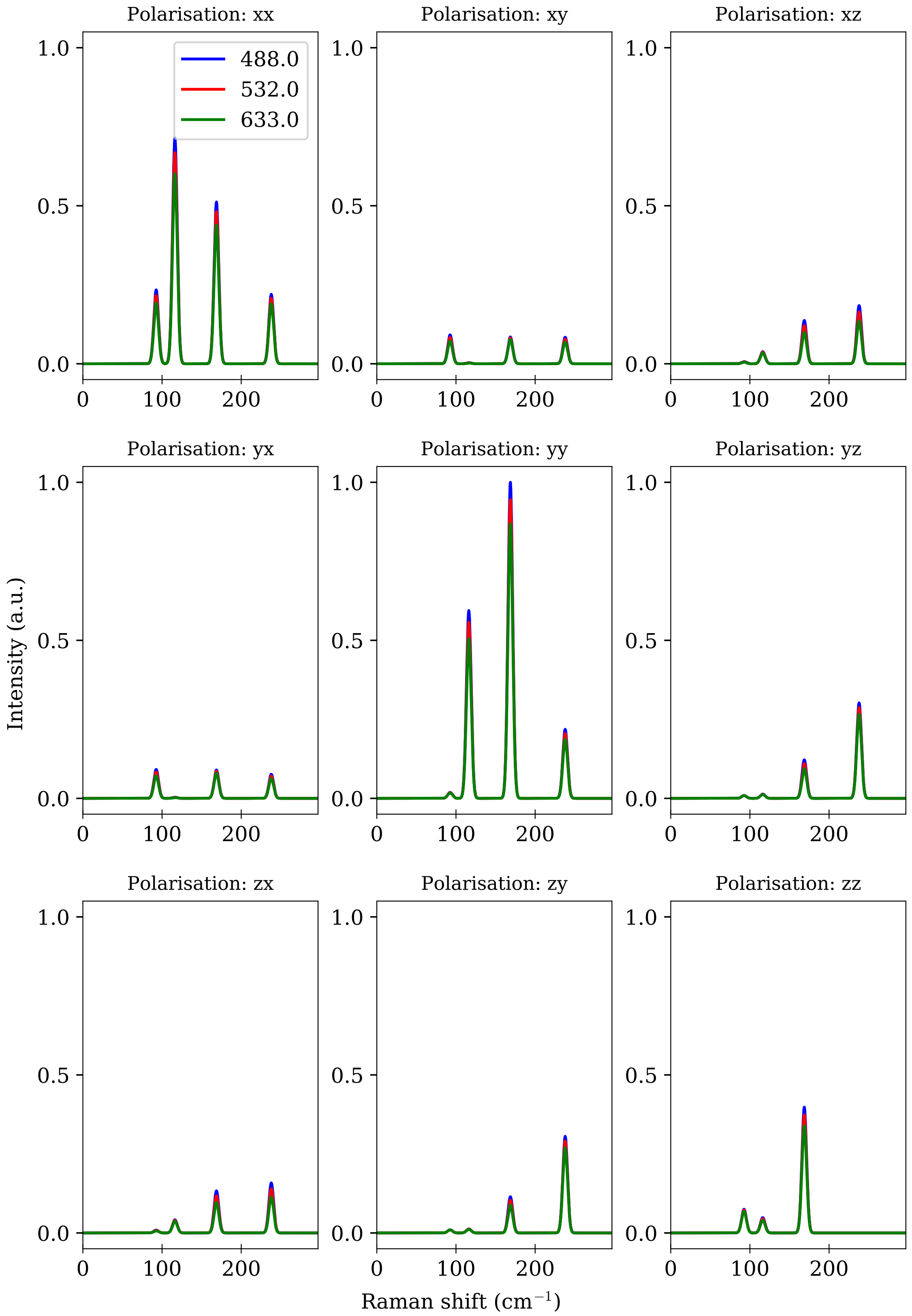

Supplementary Figure 127: Raman spectrum of $CaBr_2$ with space group 164 and Wyckoff positions b-d-0.

$CaBr_2$, 187, b-i-0

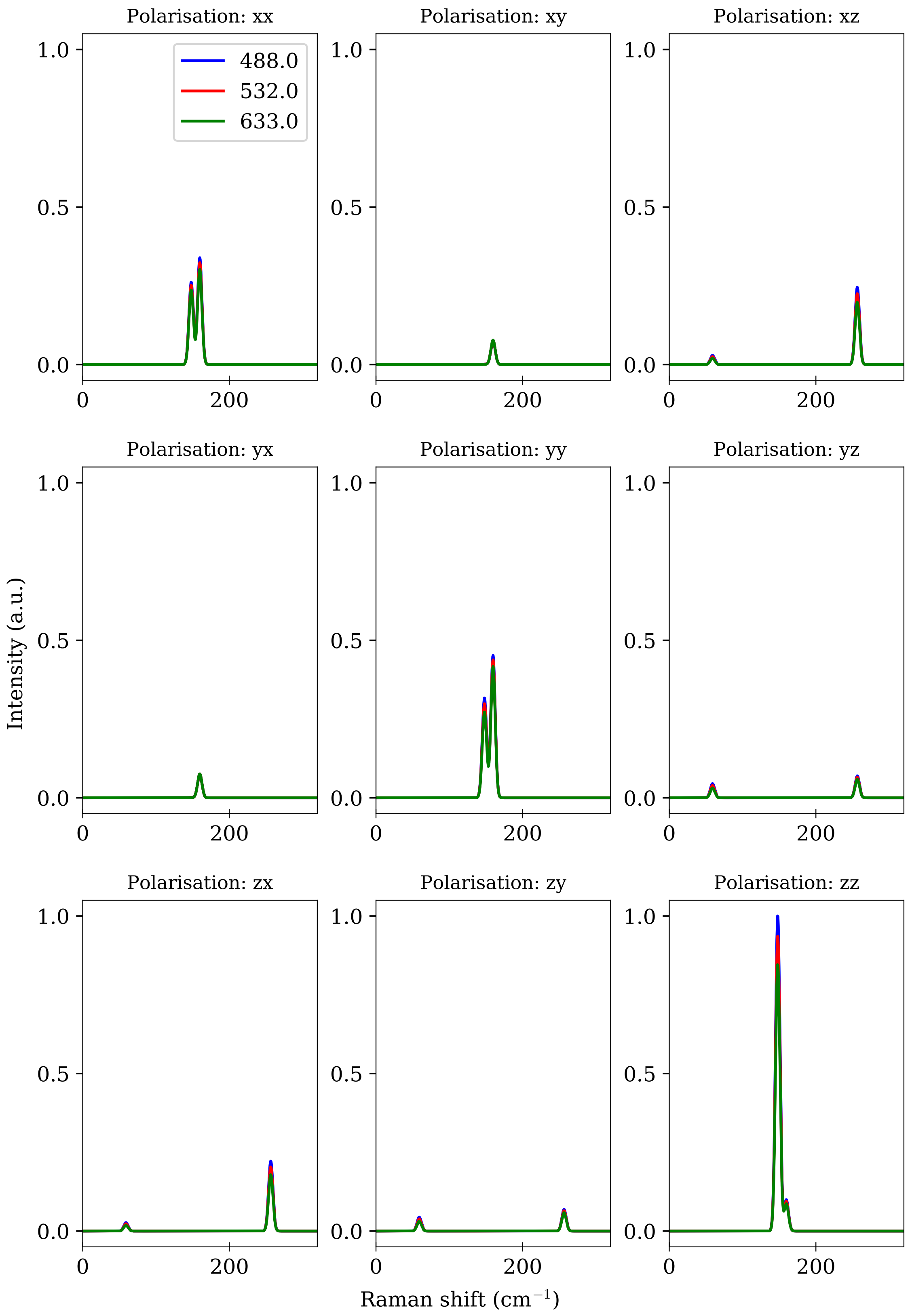

Supplementary Figure 128: Raman spectrum of $CaBr_2$ with space group 187 and Wyckoff positions b-i-0.

$CaCl_2$, 164, b-d-0

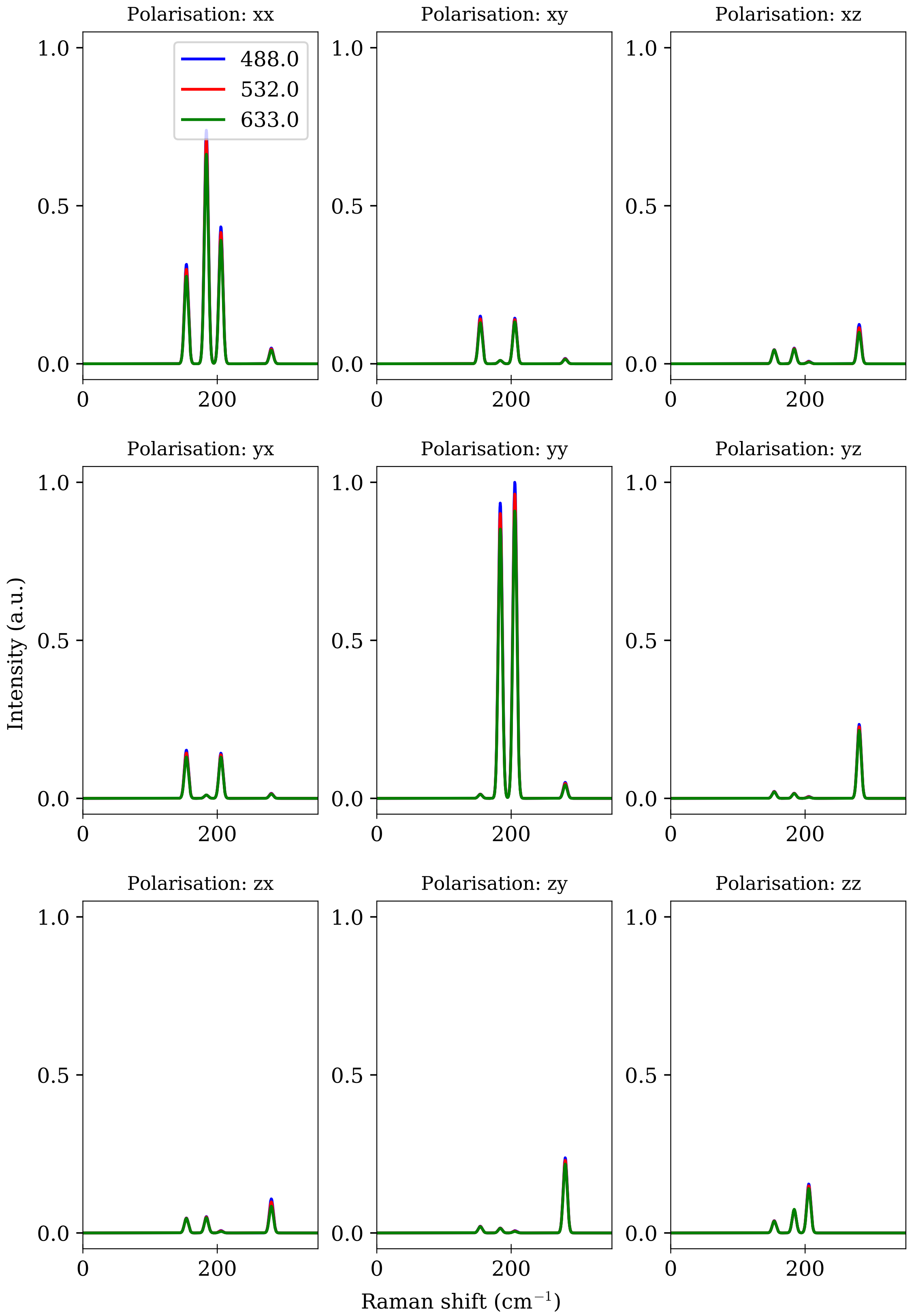

Supplementary Figure 129: Raman spectrum of $CaCl_2$ with space group 164 and Wyckoff positions b-d-0.

$CaCl_2$, 187, b-i-0

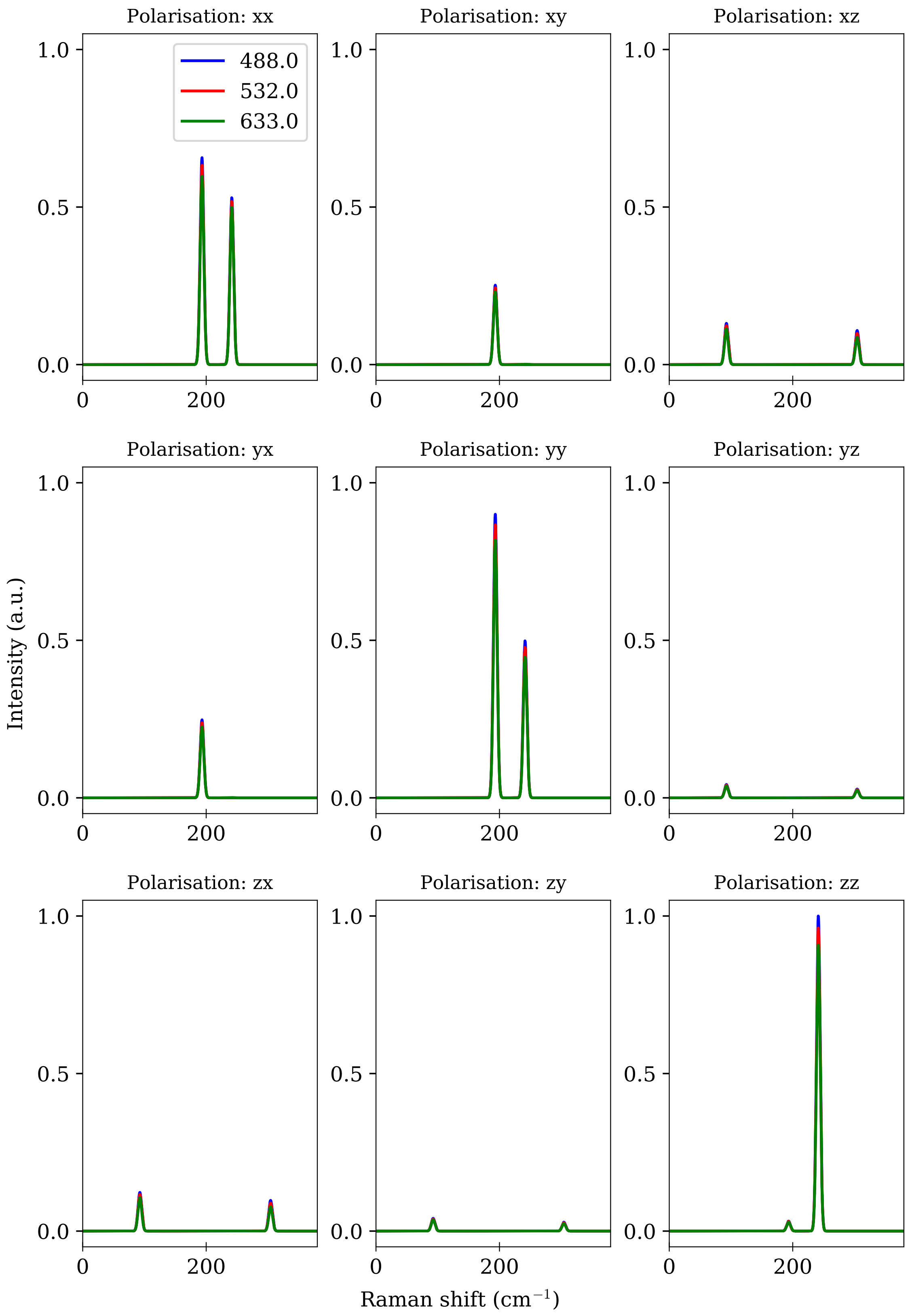

Supplementary Figure 130: Raman spectrum of $CaCl_2$ with space group 187 and Wyckoff positions b-i-0.

CaI_2, 164, b-d-0

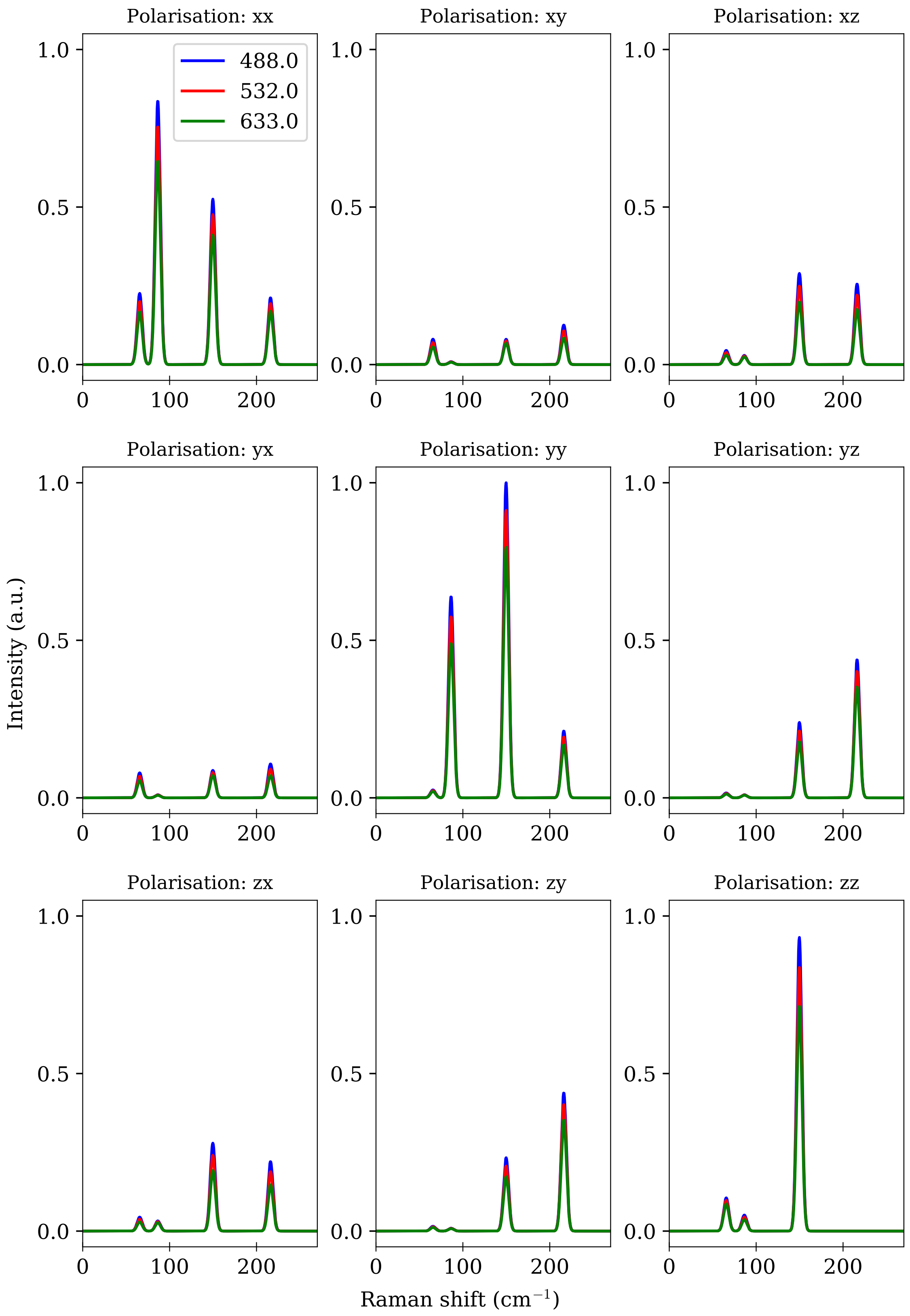

Supplementary Figure 131: Raman spectrum of CaI_2 with space group 164 and Wyckoff positions b-d-0.

CaI_2, 187, b-i-0

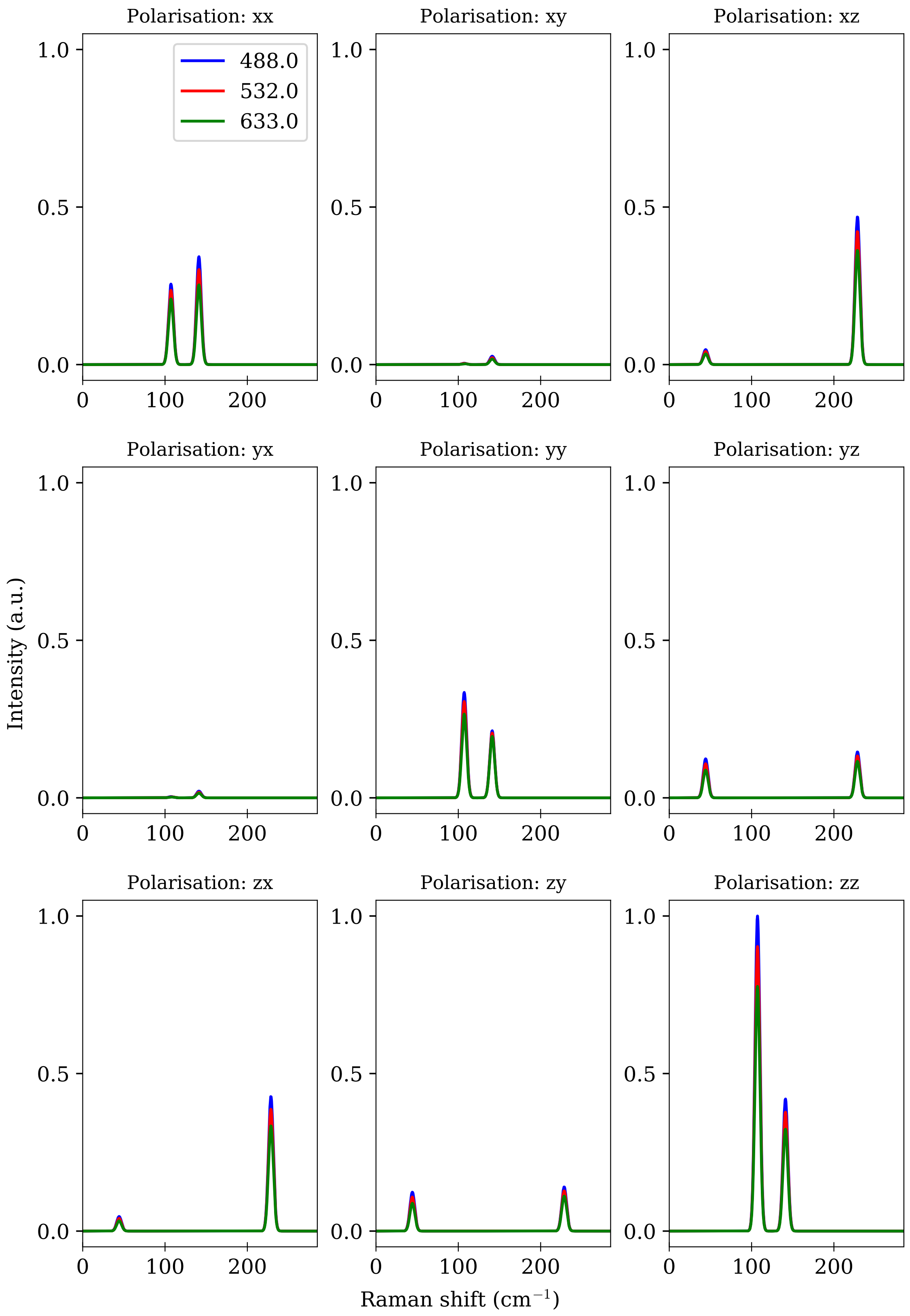

Supplementary Figure 132: Raman spectrum of CaI_2 with space group 187 and Wyckoff positions b-i-0.

Cd_2S_2, 2, i-0

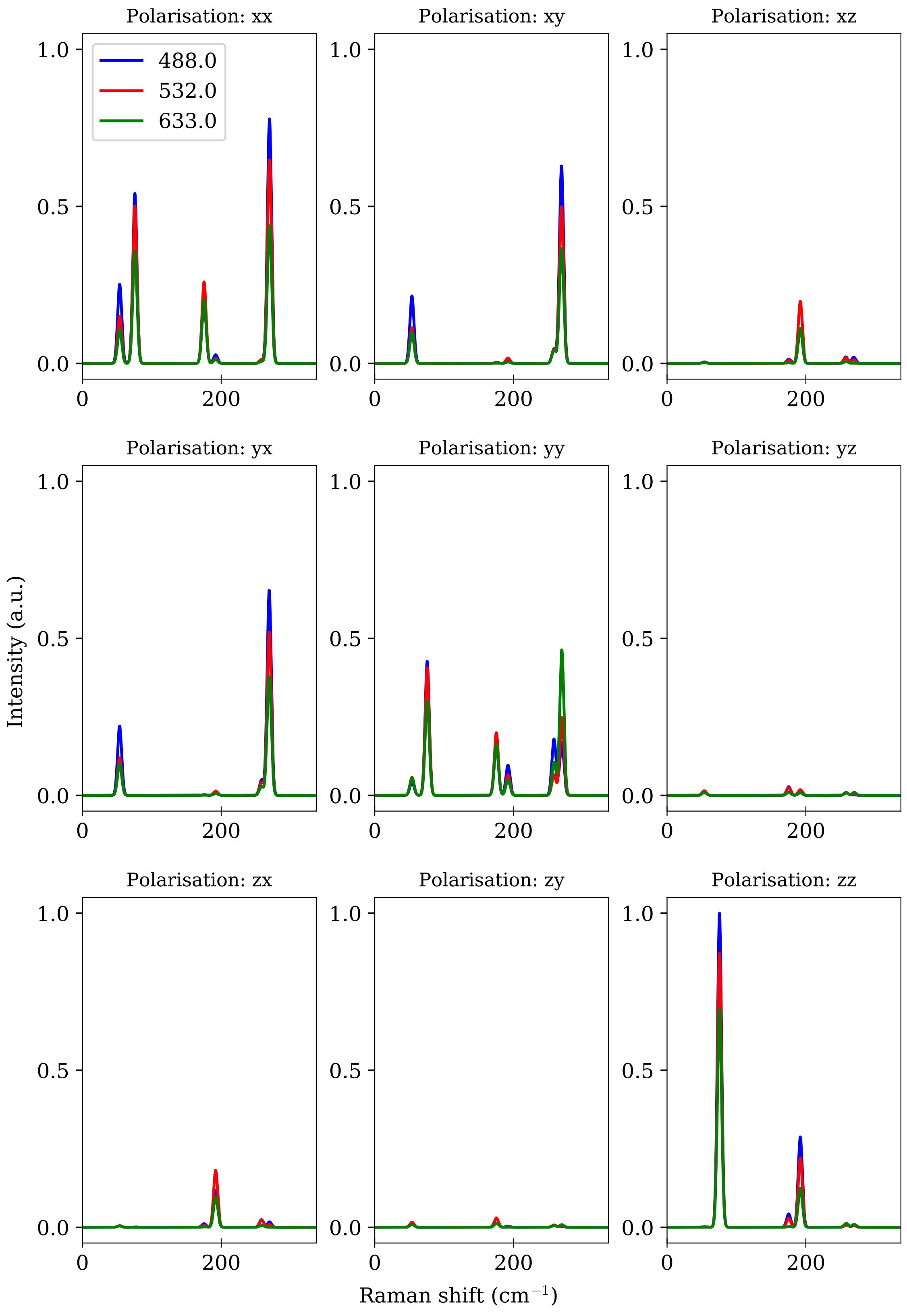

Supplementary Figure 133: Raman spectrum of Cd_2S_2 with space group 2 and Wyckoff positions i-0.

Cd_2S_4, 14, d-e-0

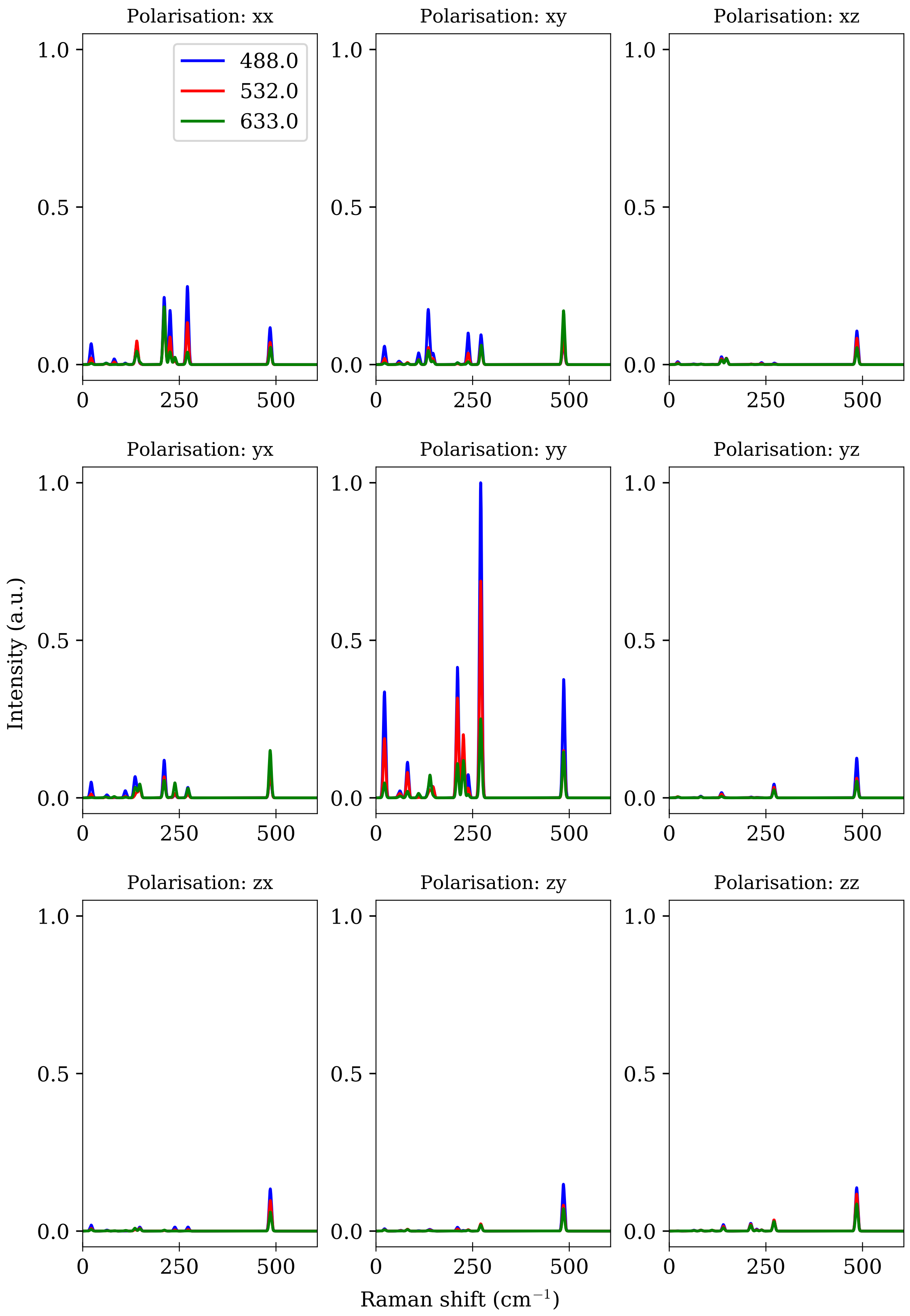

Supplementary Figure 134: Raman spectrum of Cd_2S_4 with space group 14 and Wyckoff positions d-e-0.

Cd_2Se_2, 2, i-0

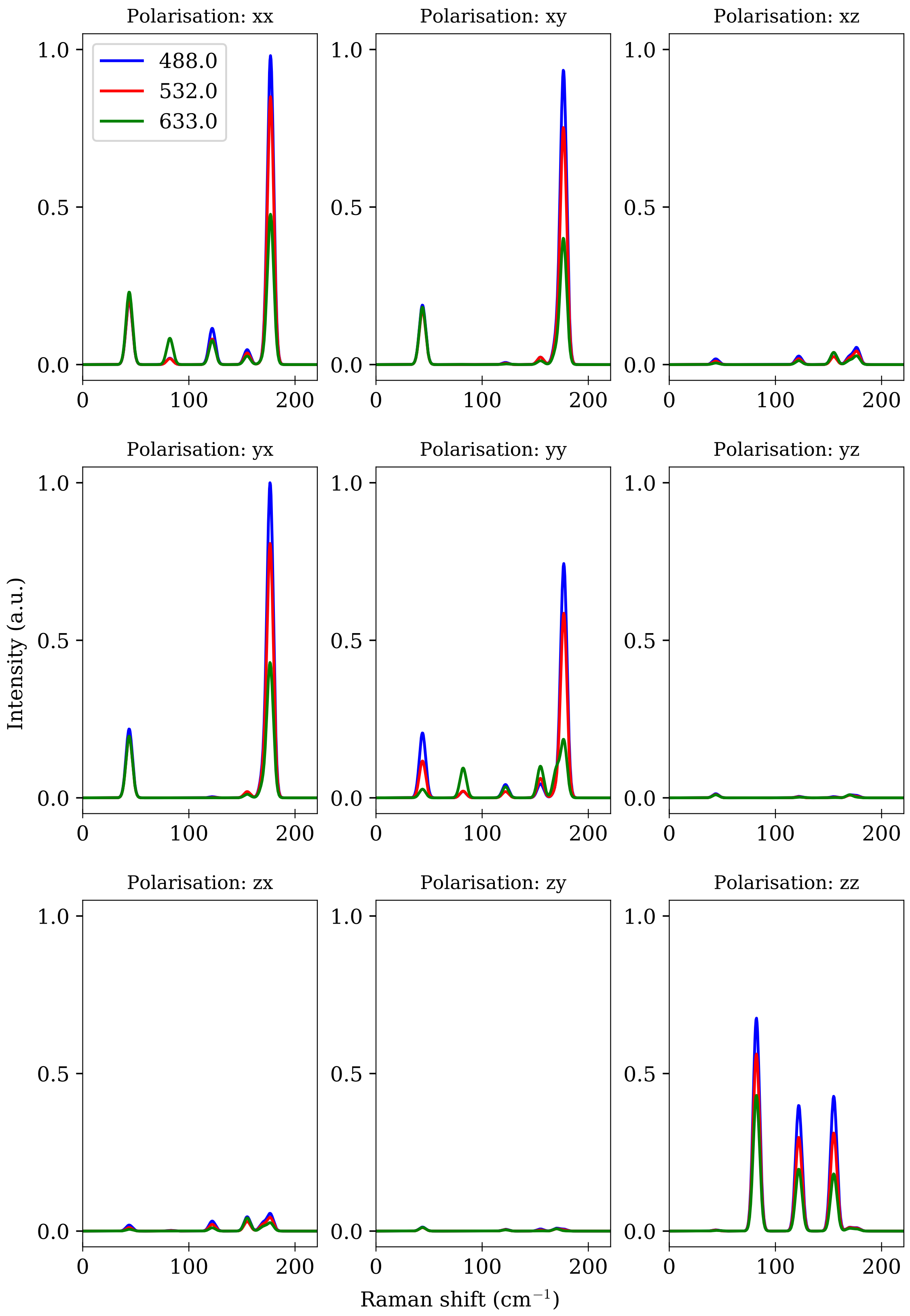

Supplementary Figure 135: Raman spectrum of Cd_2Se_2 with space group 2 and Wyckoff positions i-0.

Cd_2Te_2, 2, i-0

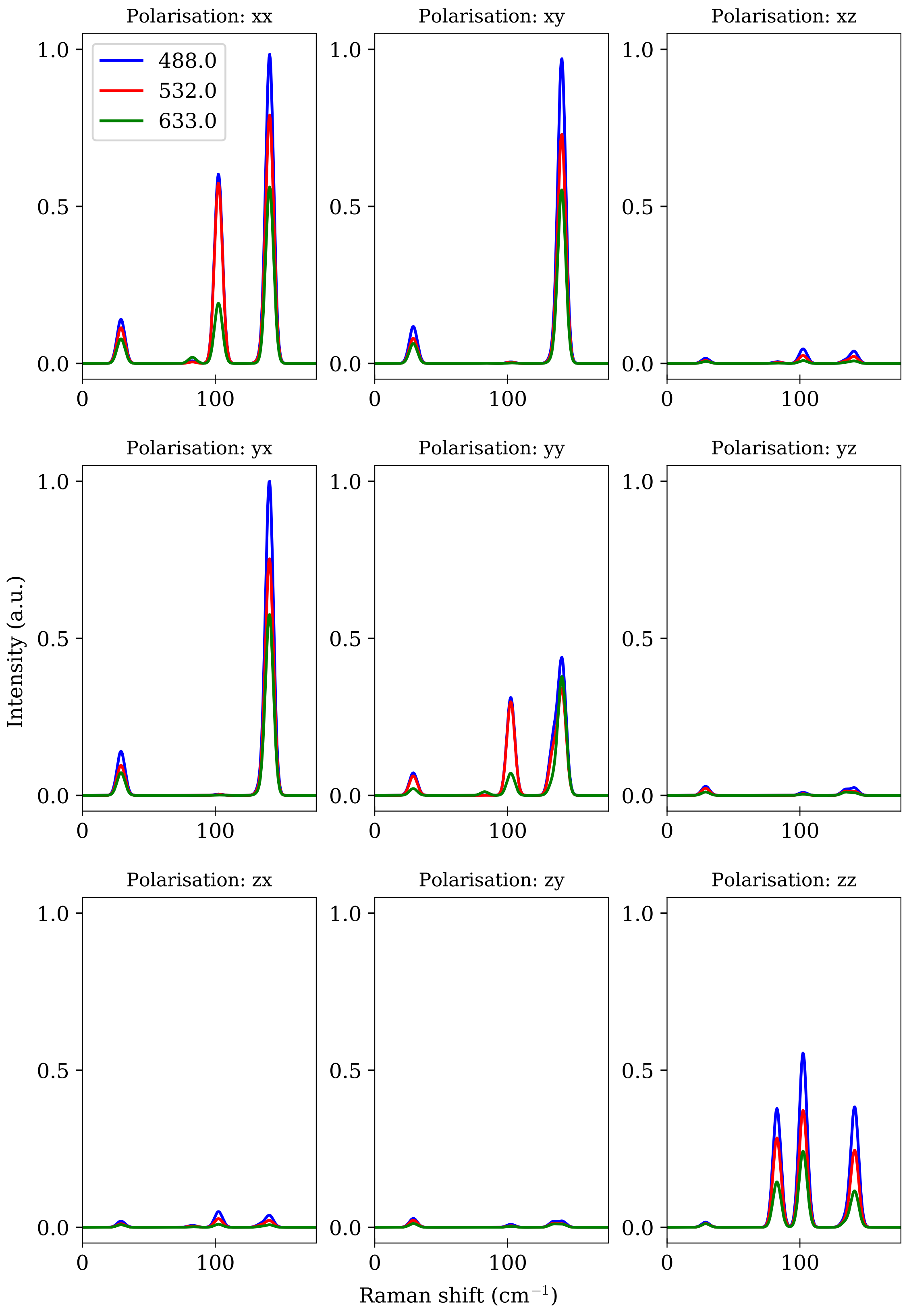

Supplementary Figure 136: Raman spectrum of Cd_2Te_2 with space group 2 and Wyckoff positions i-0.

CdI_2, 164, b-d-0

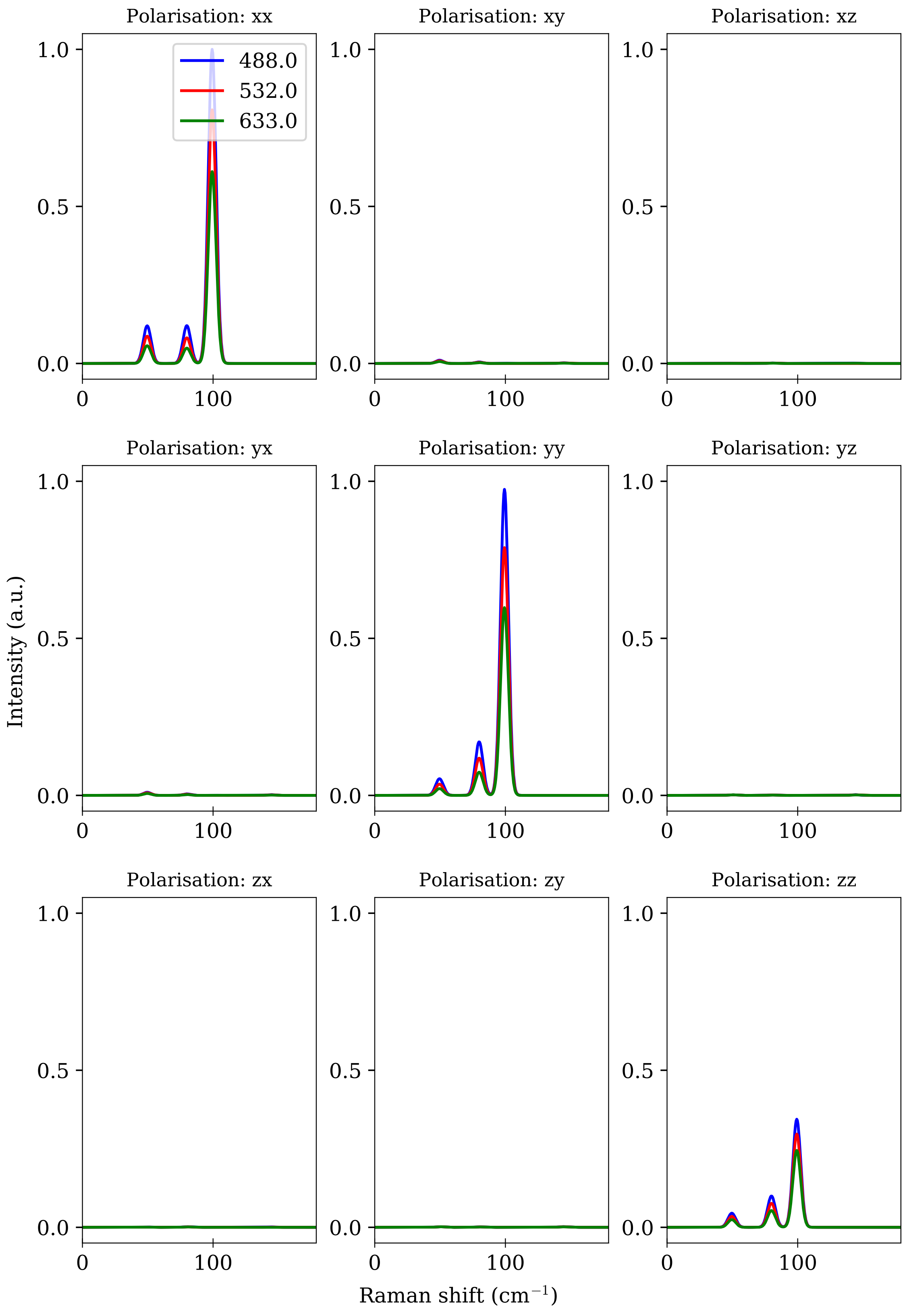

Supplementary Figure 137: Raman spectrum of CdI_2 with space group 164 and Wyckoff positions b-d-0.

CF_2Si, 156, a-b-0

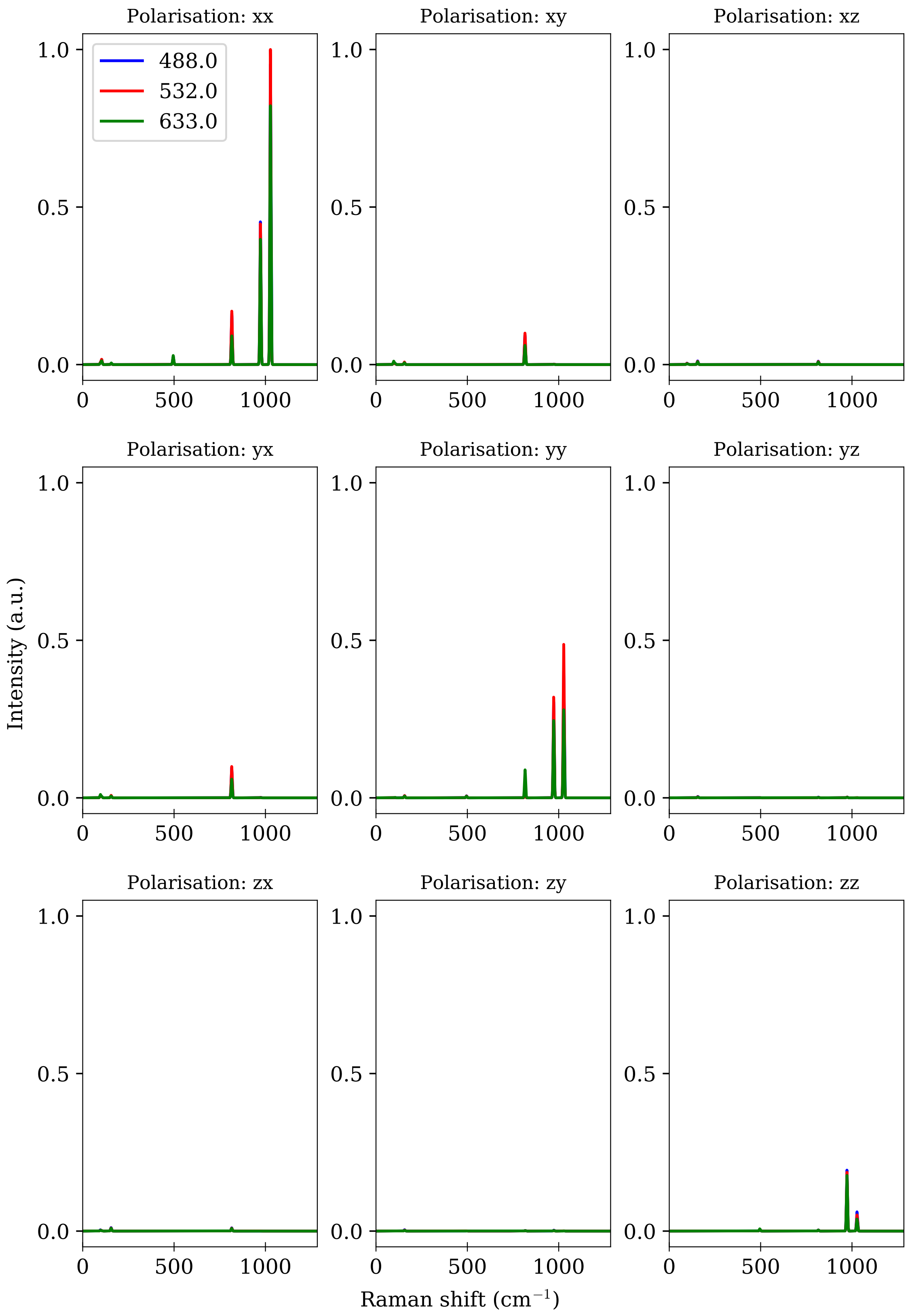

Supplementary Figure 138: Raman spectrum of CF_2Si with space group 156 and Wyckoff positions a-b-0.

CH_2Si, 156, a-b-0

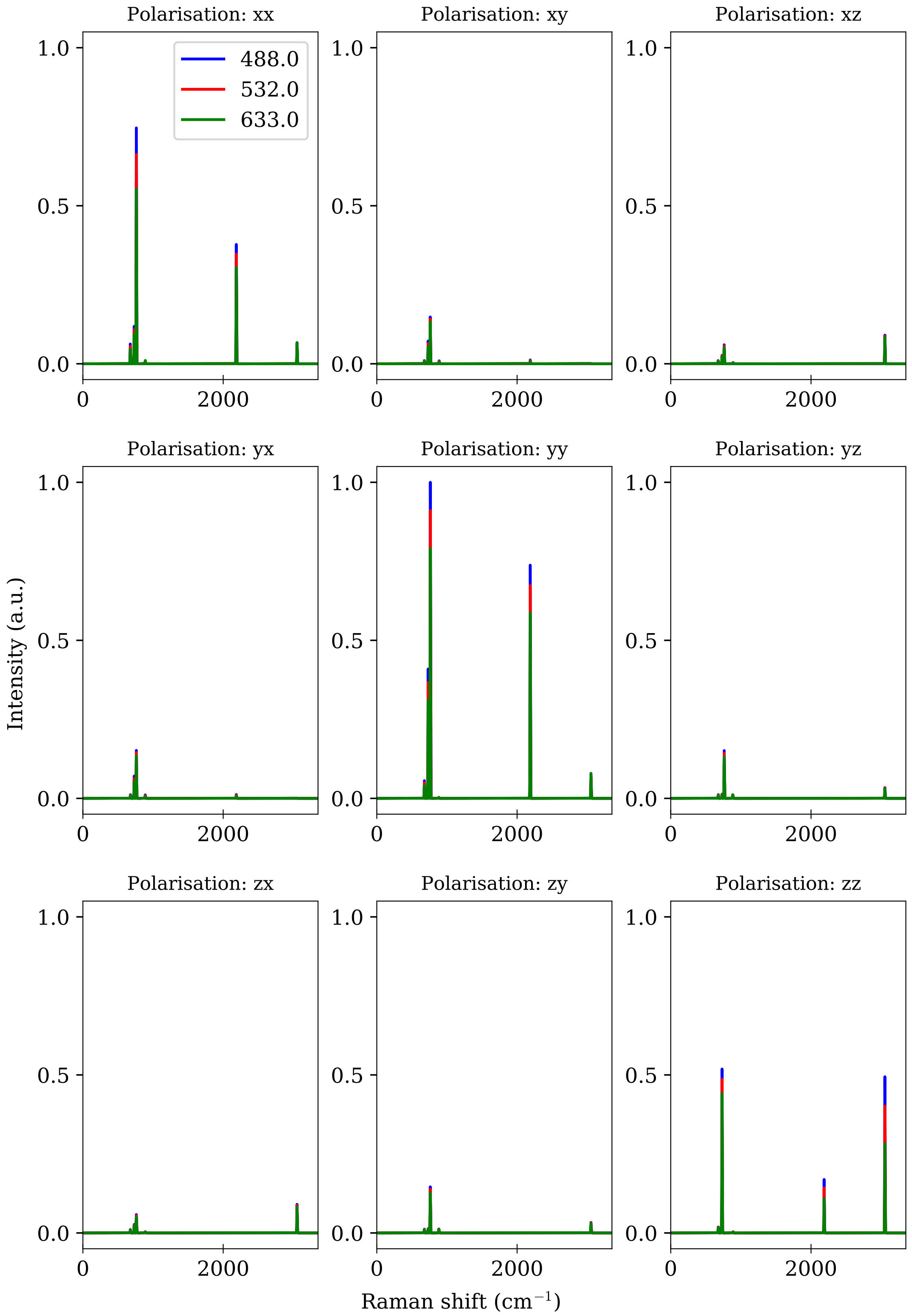

Supplementary Figure 139: Raman spectrum of CH_2Si with space group 156 and Wyckoff positions a-b-0.

Cl_2Sb_2, 2, i-0

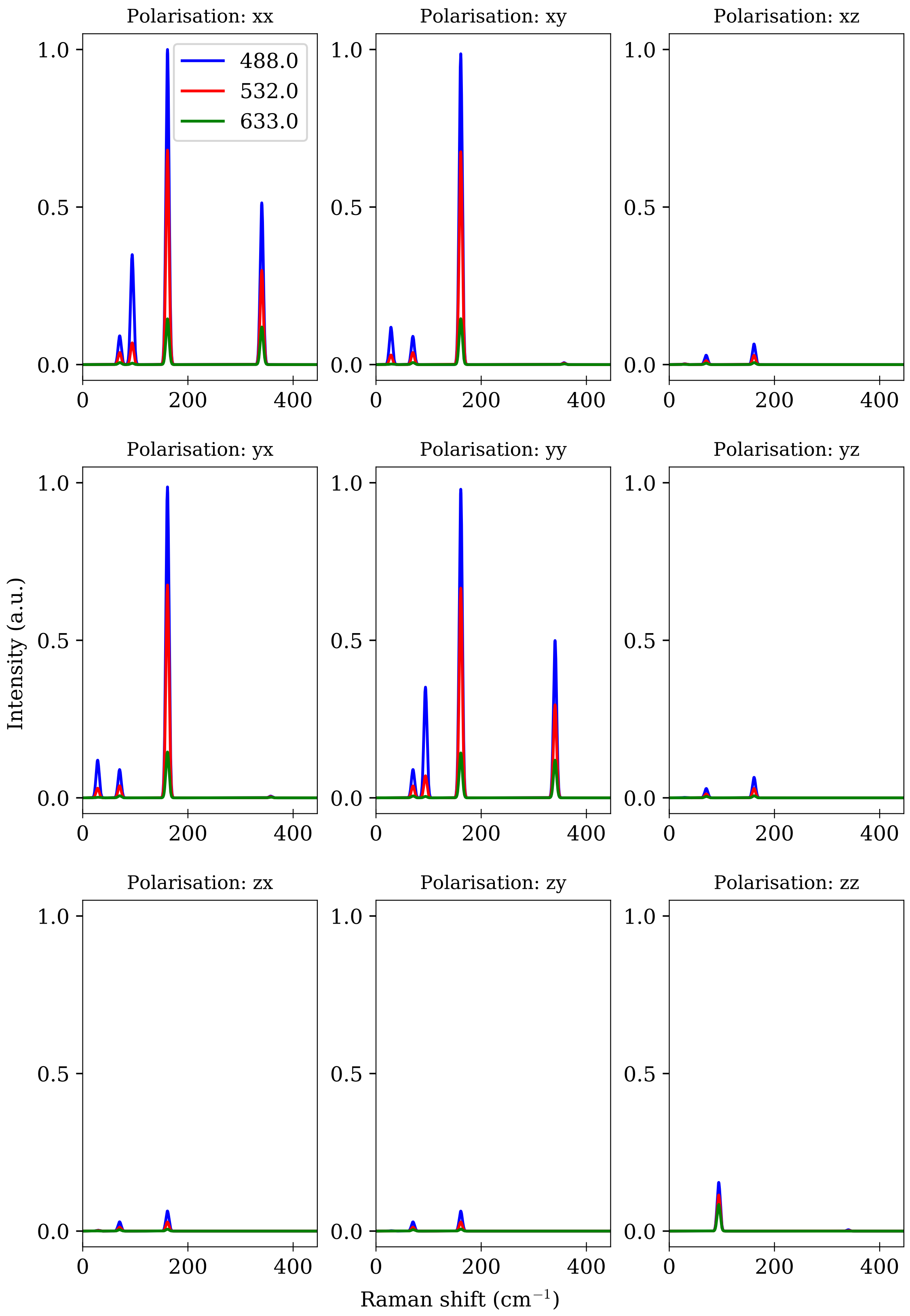

Supplementary Figure 140: Raman spectrum of Cl_2Sb_2 with space group 2 and Wyckoff positions i-0.

ClSbSe, 156, a-b-c-0

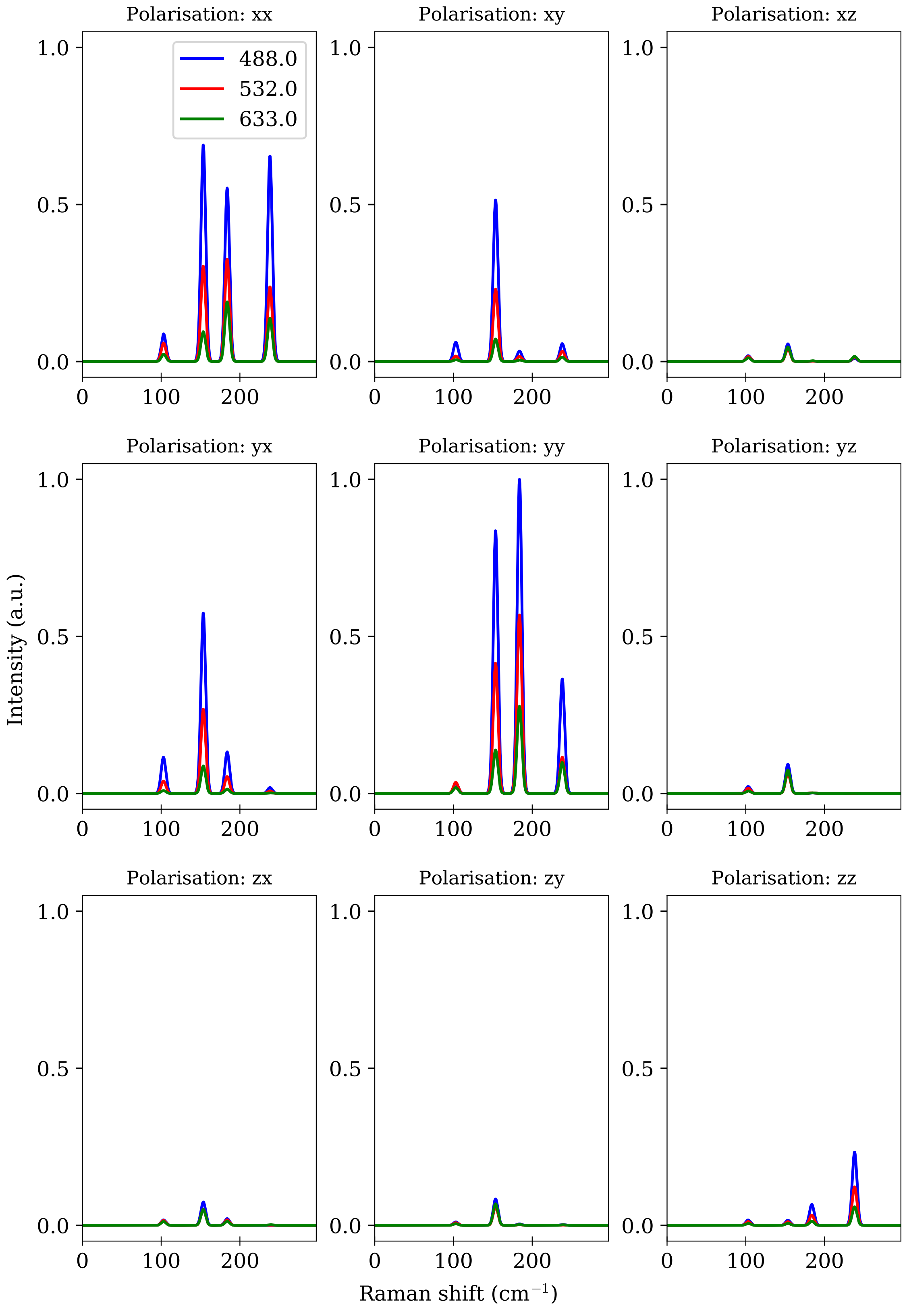

Supplementary Figure 141: Raman spectrum of ClSbSe with space group 156 and Wyckoff positions a-b-c-0.

ClSbTe, 156, a-b-c-0

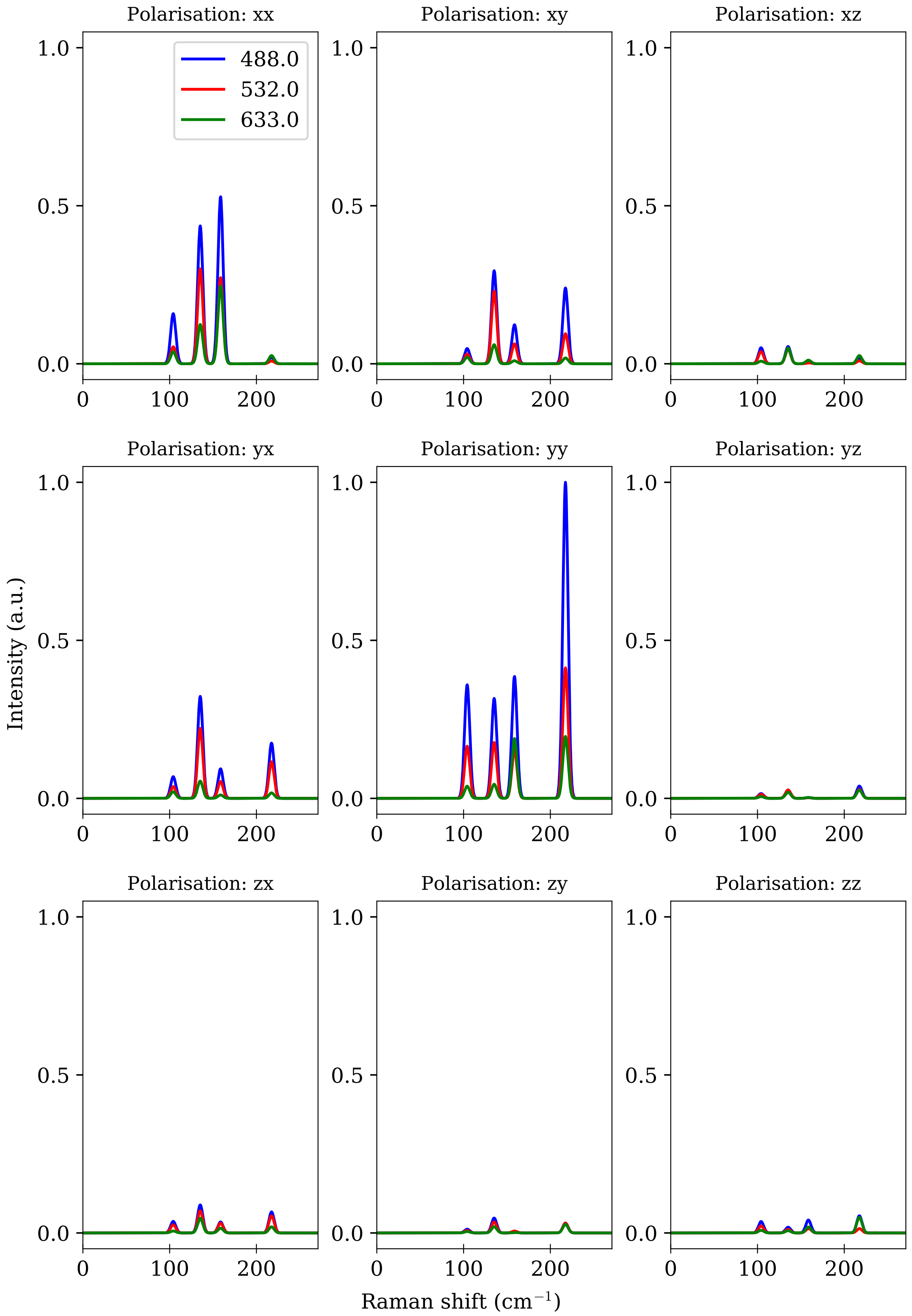

Supplementary Figure 142: Raman spectrum of ClSbTe with space group 156 and Wyckoff positions a-b-c-0.

$Co_2Br_2N_2$, 59, a-b-0

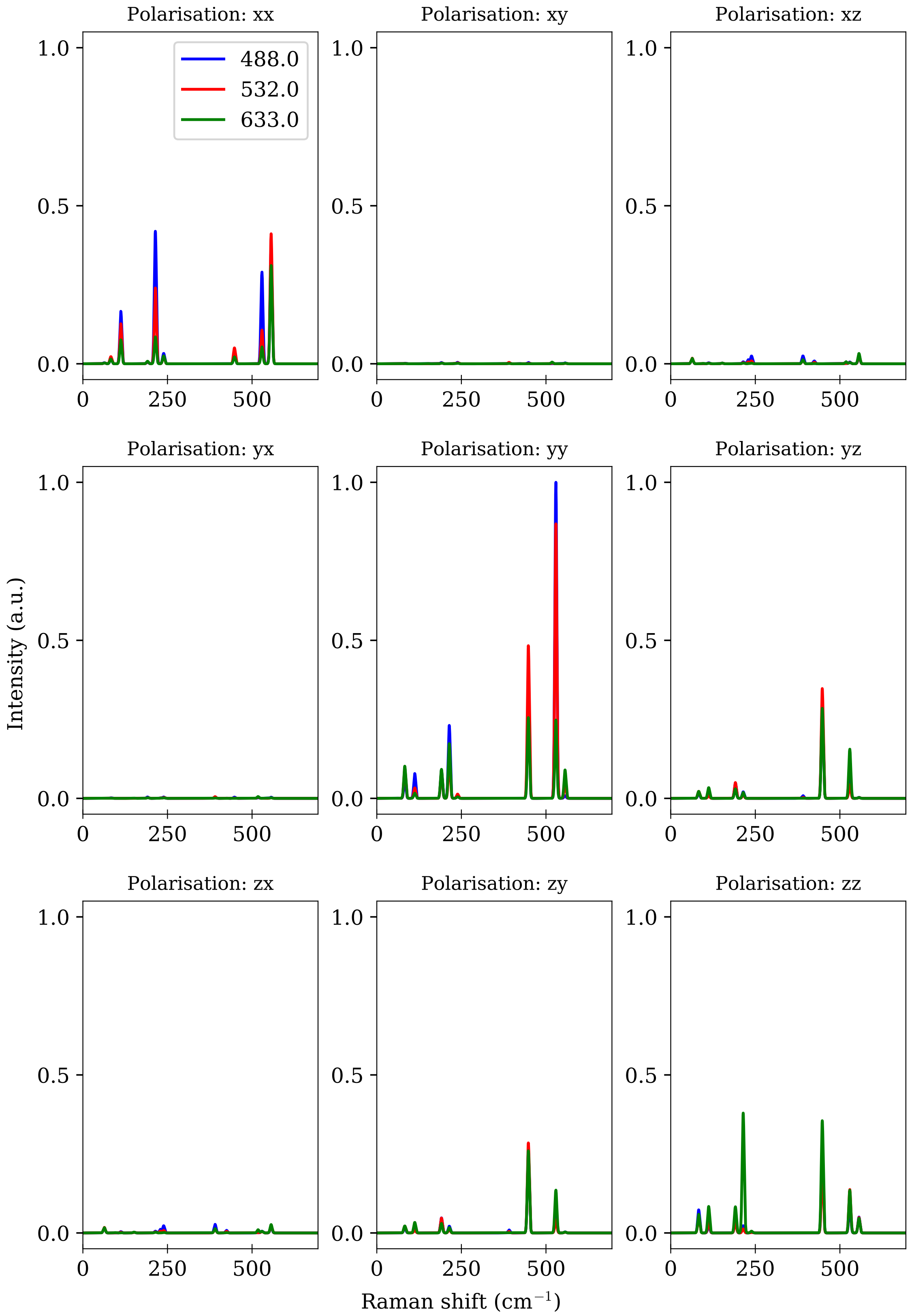

Supplementary Figure 143: Raman spectrum of $Co_2Br_2N_2$ with space group 59 and Wyckoff positions a-b-0.

$Co_2Br_2O_2$, 59, a-b-0

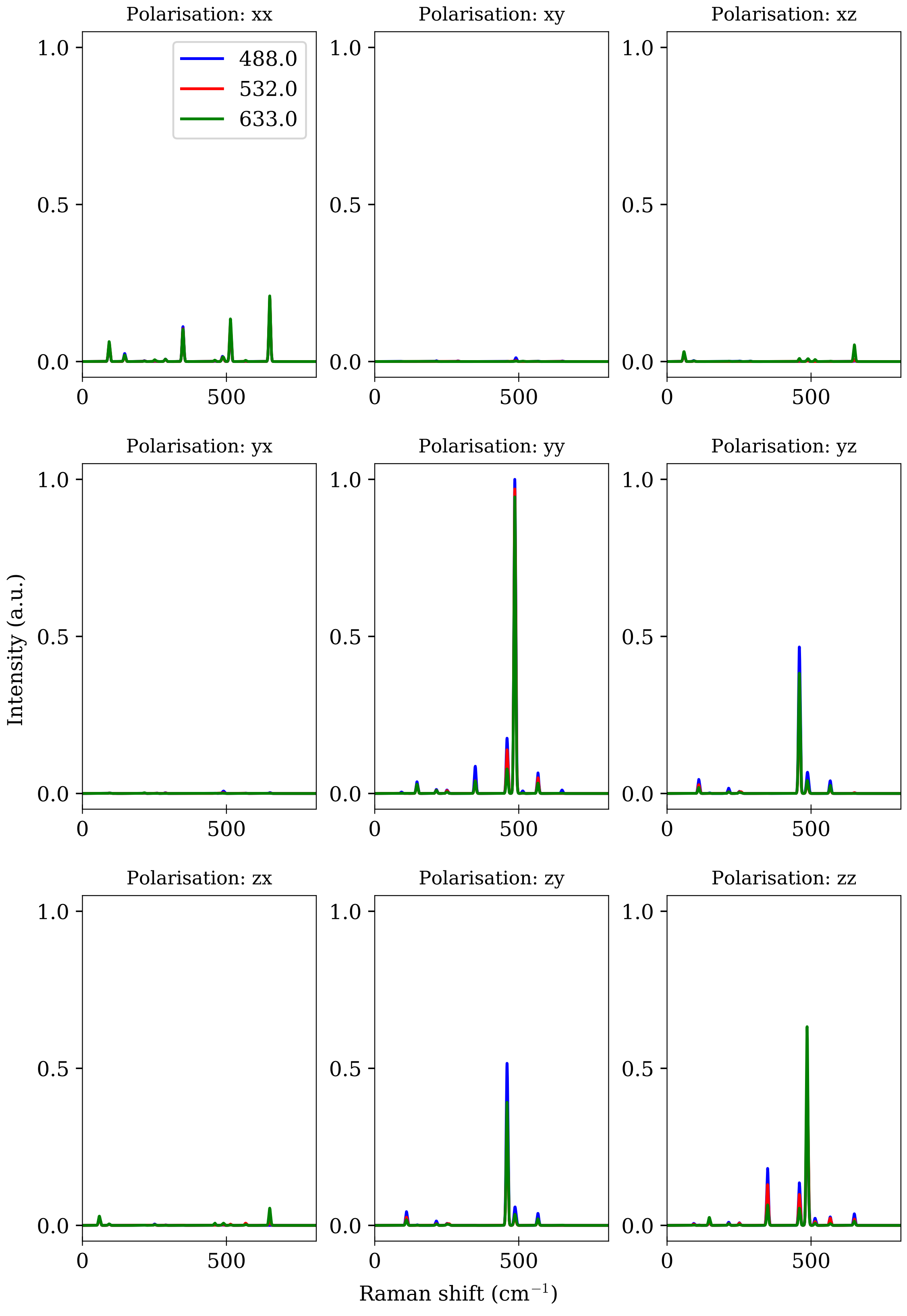

Supplementary Figure 144: Raman spectrum of $Co_2Br_2O_2$ with space group 59 and Wyckoff positions a-b-0.

$Co_2Br_2Se_2$, 59, a-b-0

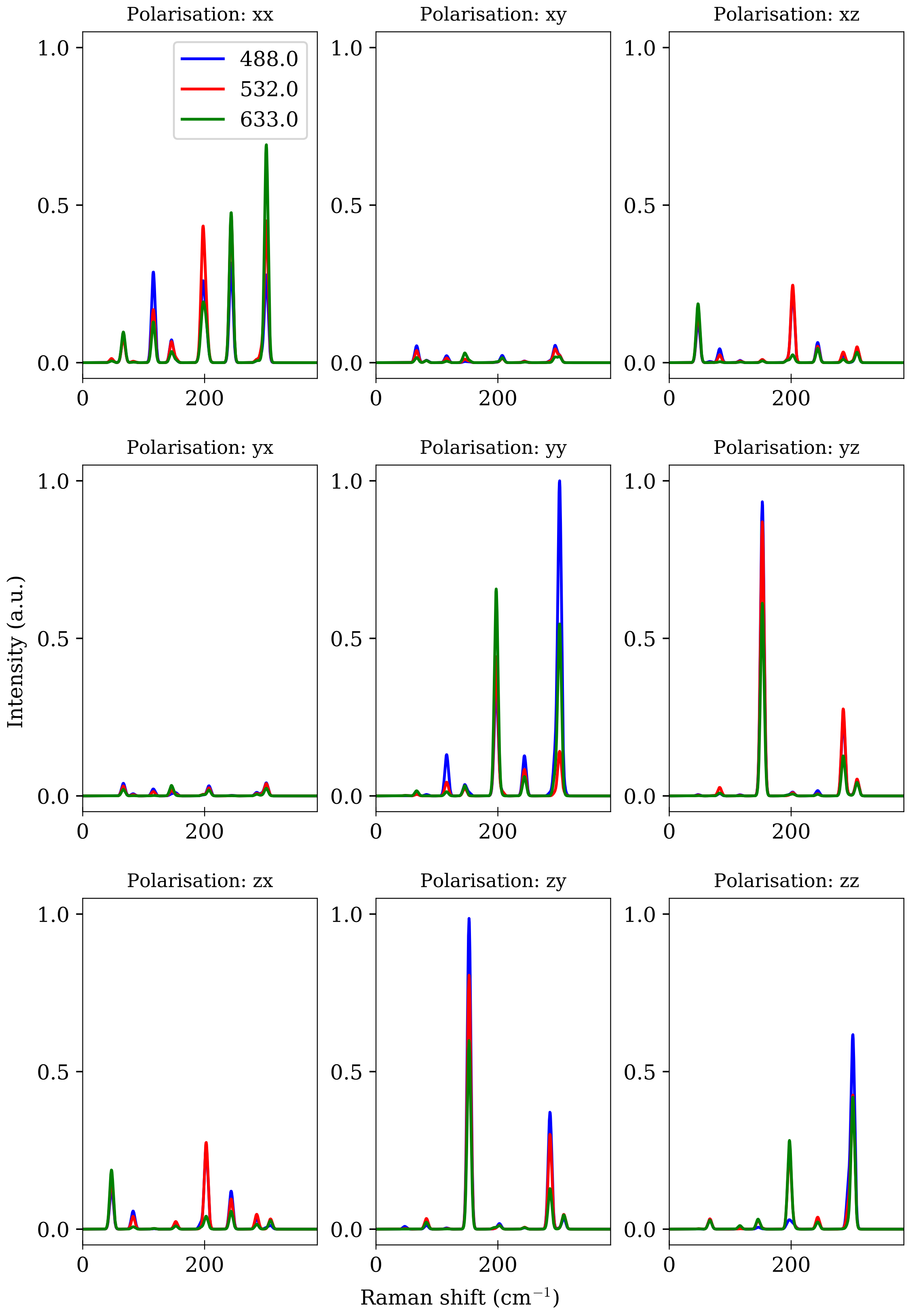

Supplementary Figure 145: Raman spectrum of $Co_2Br_2Se_2$ with space group 59 and Wyckoff positions a-b-0.

Co_2Br_6, 162, d-k-0

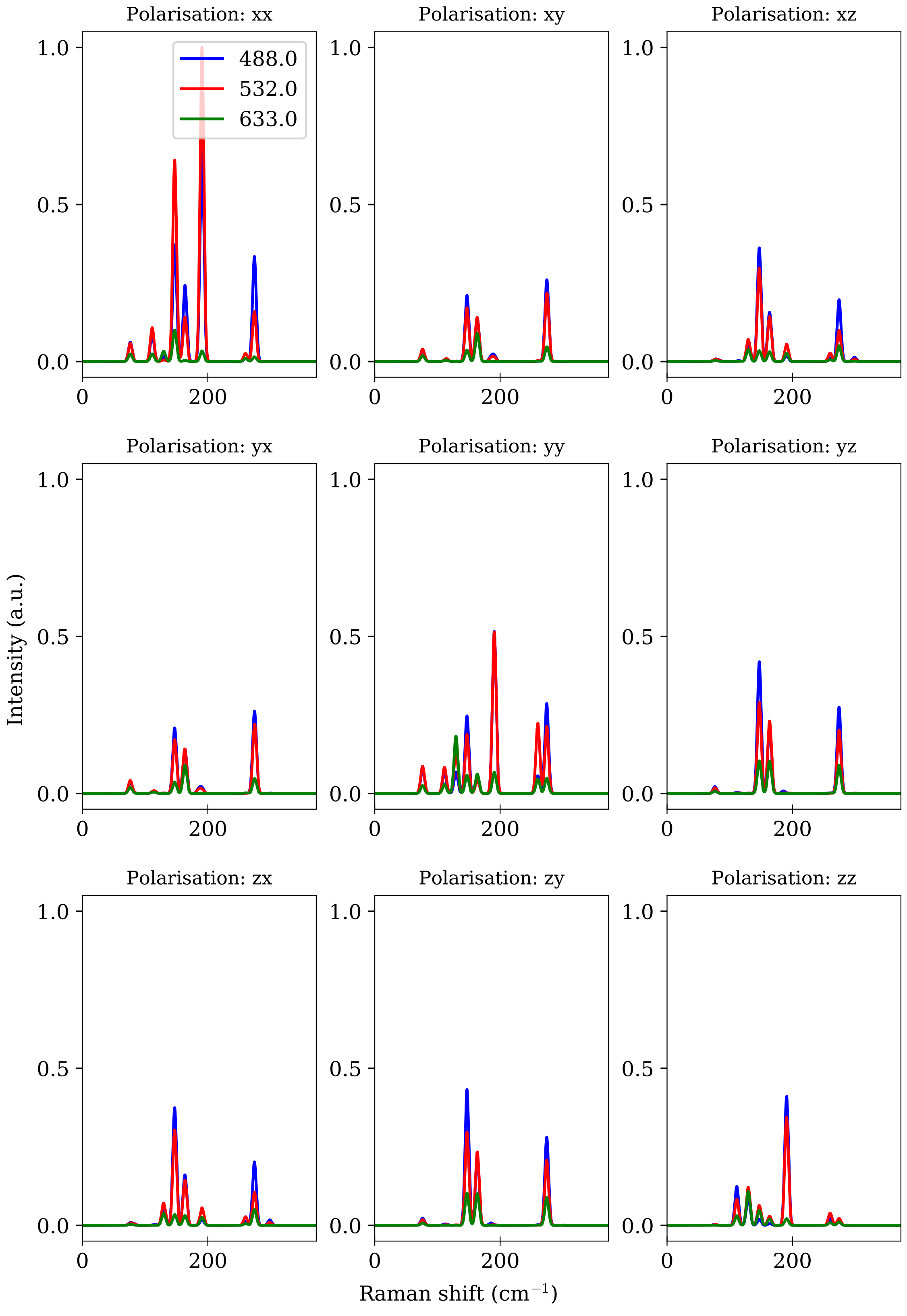

Supplementary Figure 146: Raman spectrum of Co_2Br_6 with space group 162 and Wyckoff positions d-k-0.

$Co_2Cl_2O_2$, 59, a-b-0

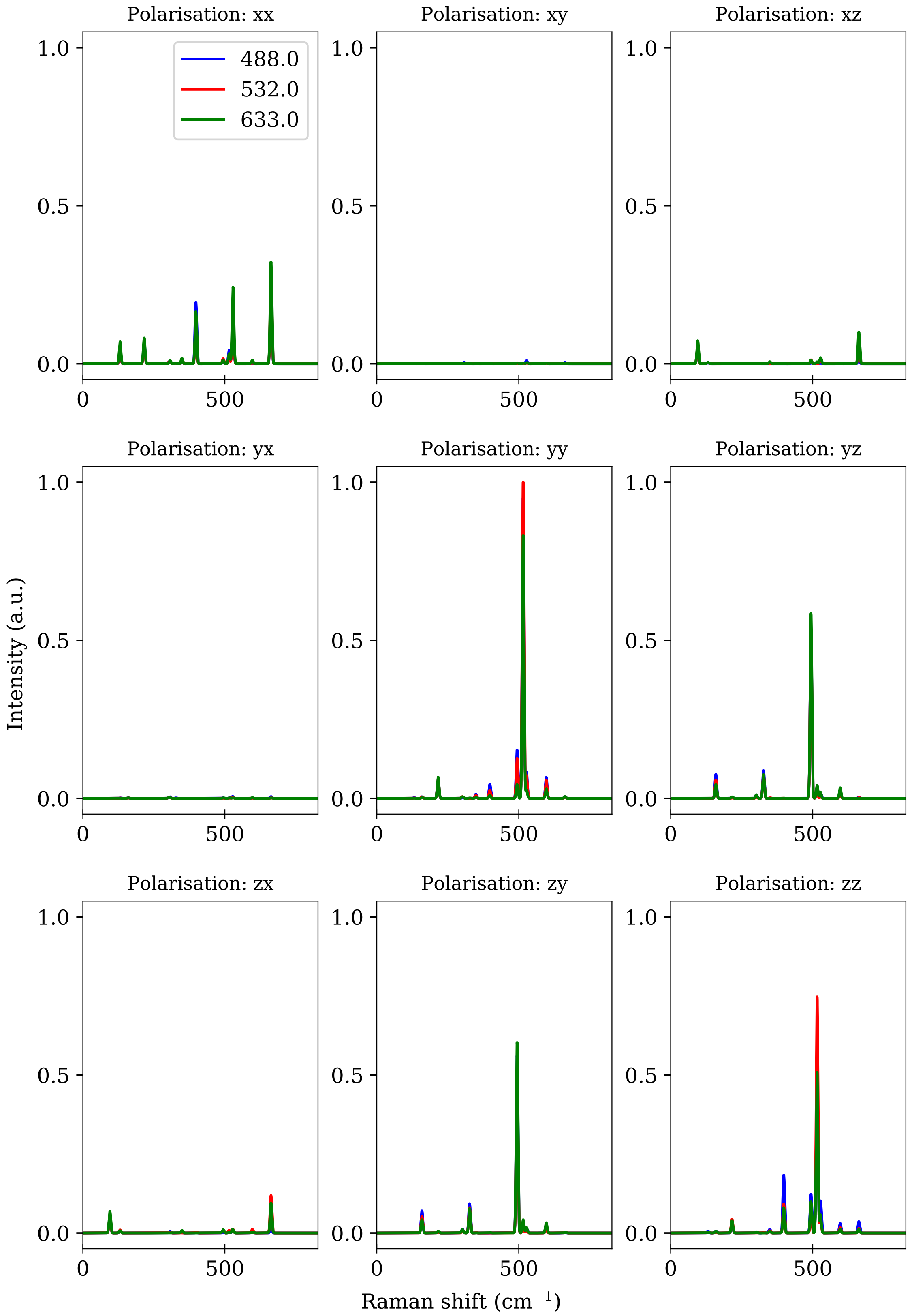

Supplementary Figure 147: Raman spectrum of $Co_2Cl_2O_2$ with space group 59 and Wyckoff positions a-b-0.

Co_2Cl_6, 162, d-k-0

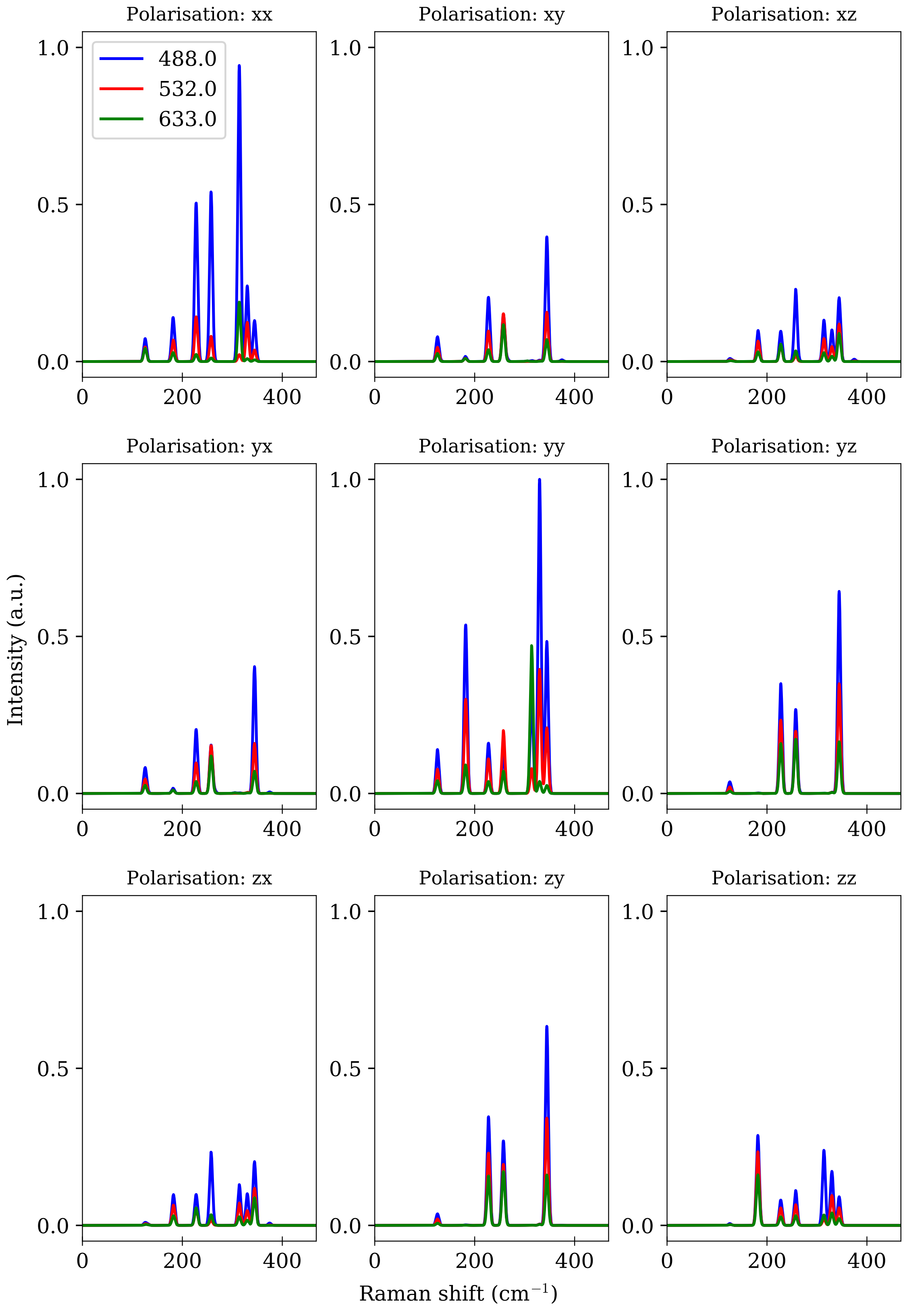

Supplementary Figure 148: Raman spectrum of Co_2Cl_6 with space group 162 and Wyckoff positions d-k-0.

$Co_2I_2S_2$, 59, a-b-0

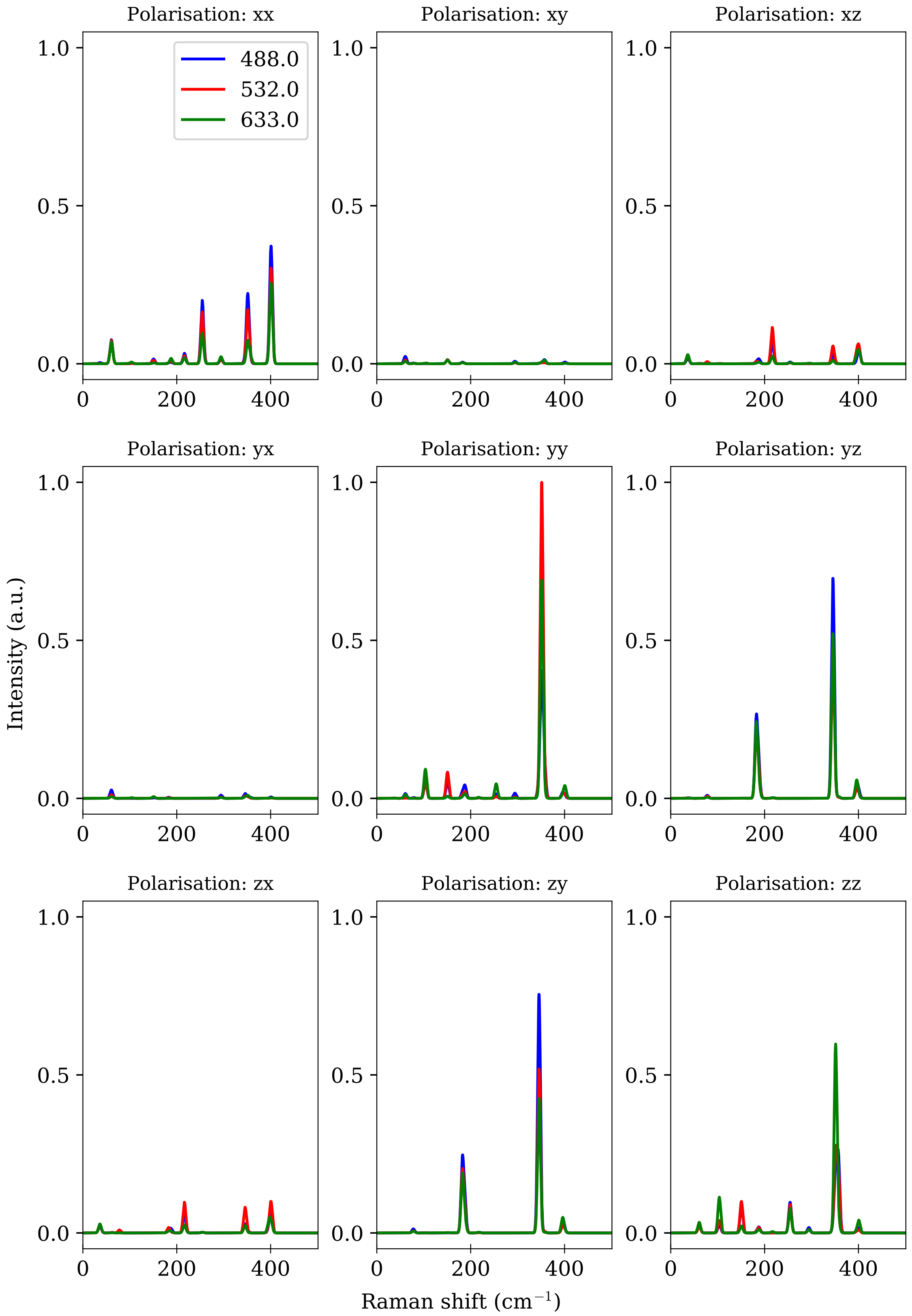

Supplementary Figure 149: Raman spectrum of $Co_2I_2S_2$ with space group 59 and Wyckoff positions a-b-0.

Co_2I_6, 162, d-k-0

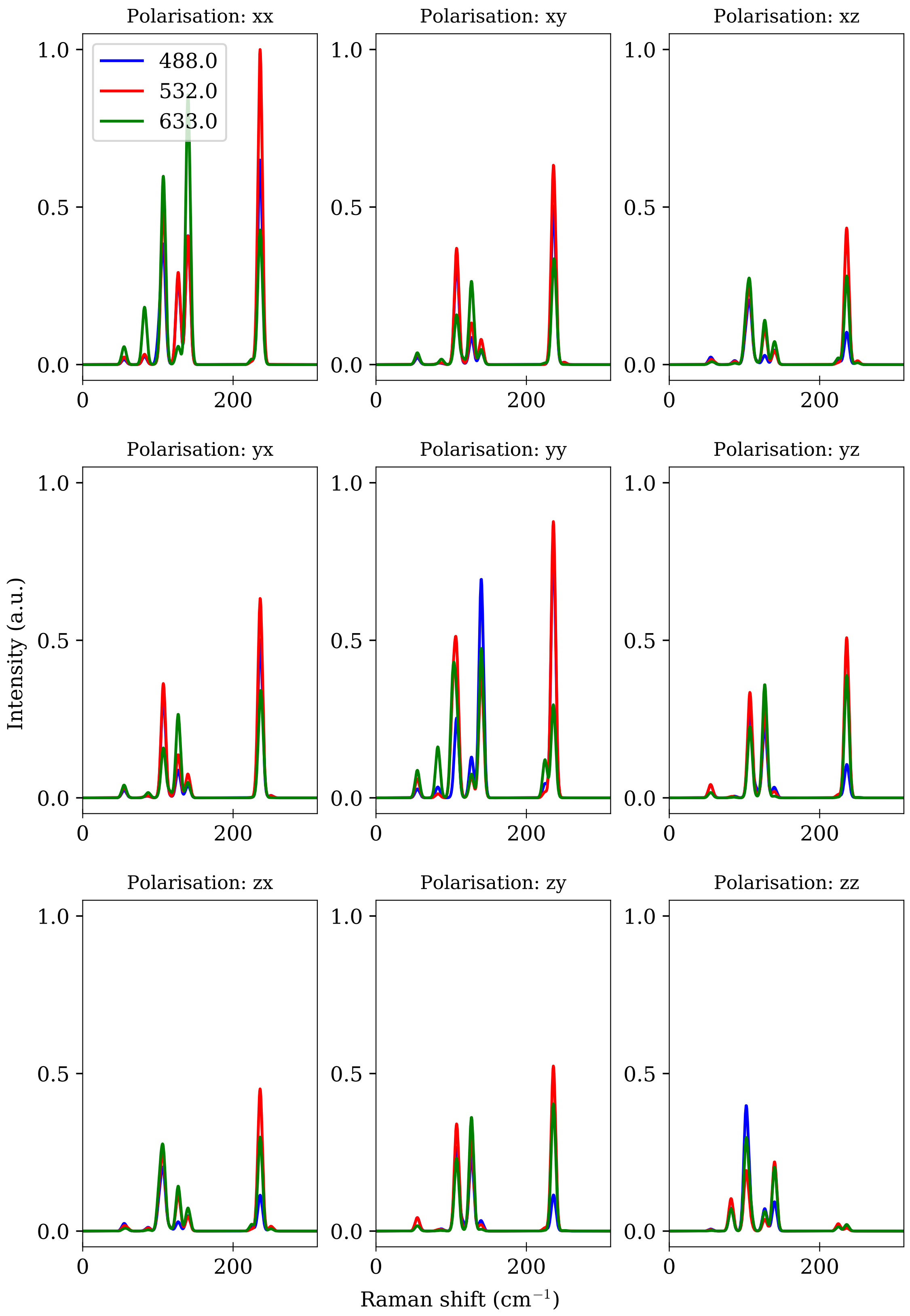

Supplementary Figure 150: Raman spectrum of Co_2I_6 with space group 162 and Wyckoff positions d-k-0.

Co_2S_2, 164, d-0

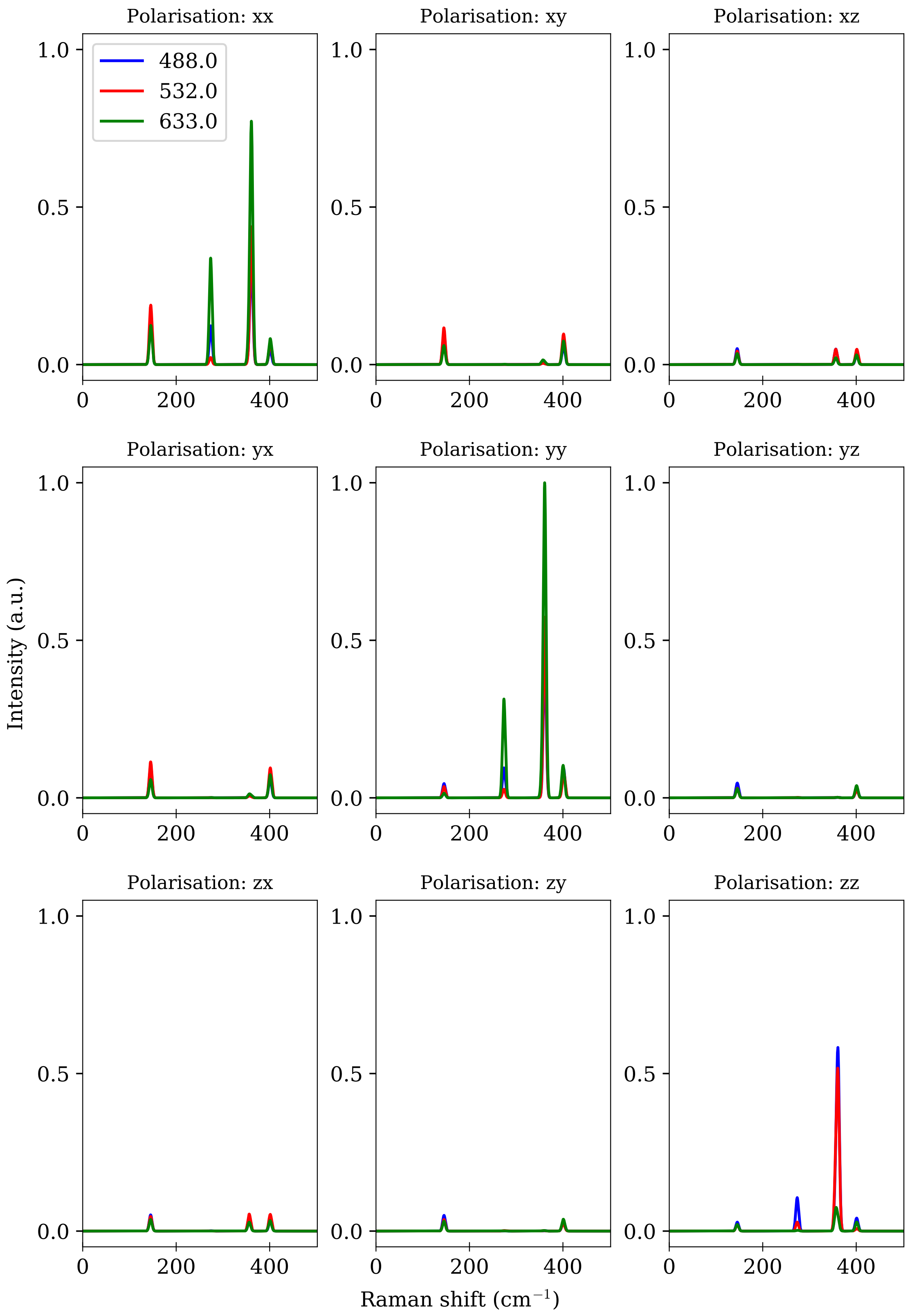

Supplementary Figure 151: Raman spectrum of Co_2S_2 with space group 164 and Wyckoff positions d-0.

Co_2Se_2, 129, b-c-0

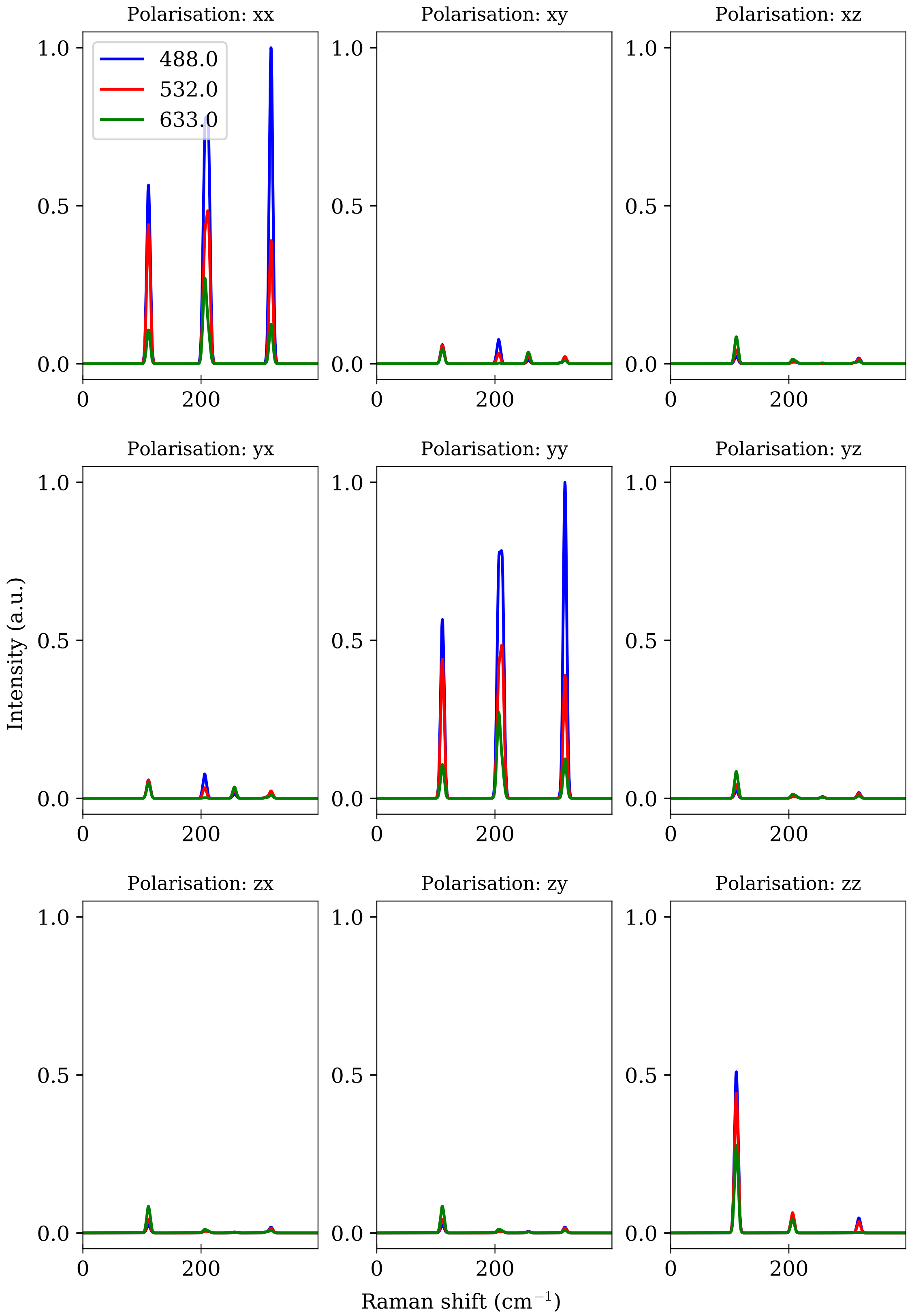

Supplementary Figure 152: Raman spectrum of Co_2Se_2 with space group 129 and Wyckoff positions b-c-0.

Co_2Se_2, 12, i-0

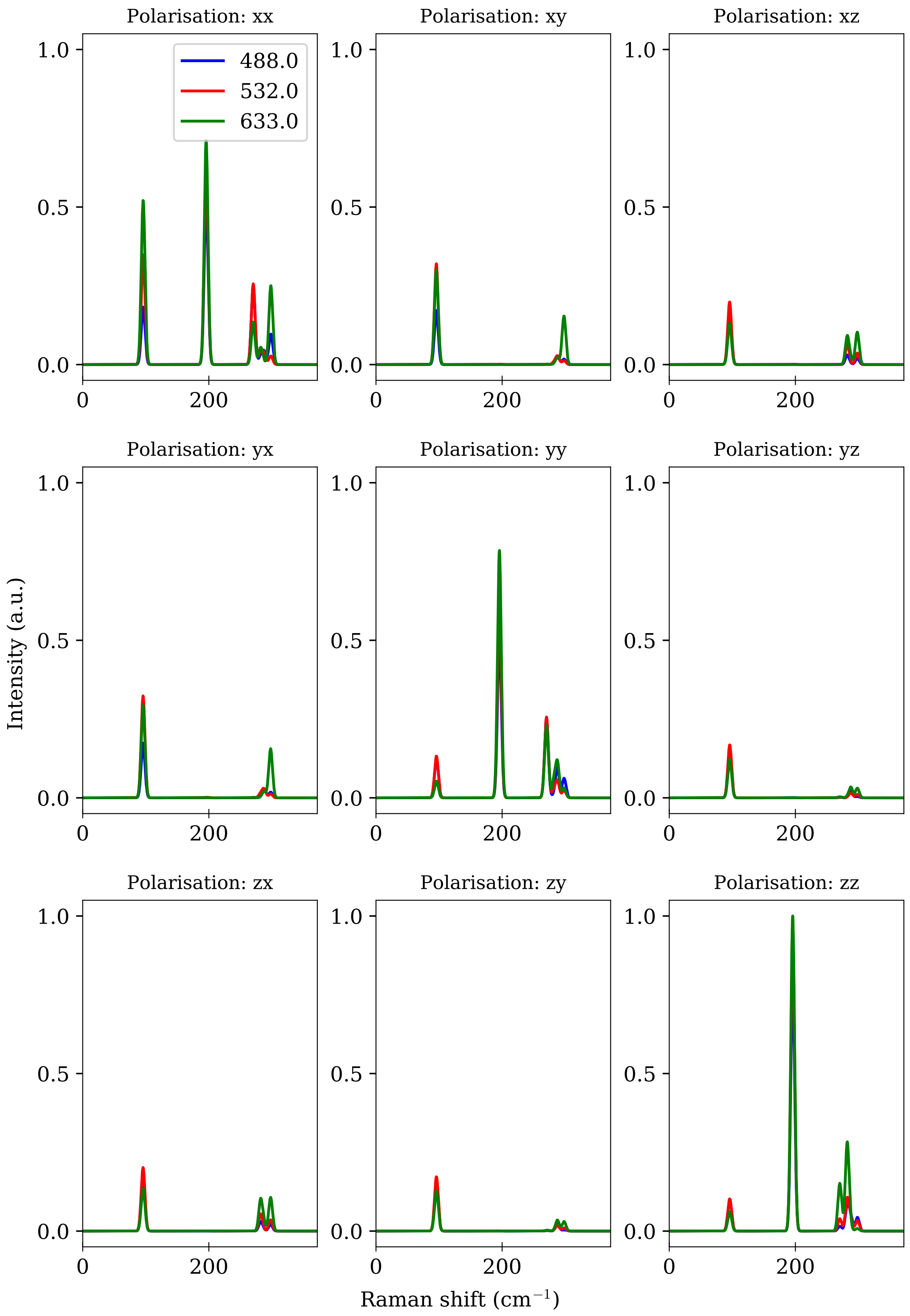

Supplementary Figure 153: Raman spectrum of Co_2Se_2 with space group 12 and Wyckoff positions i-0.

Co_2Te_2, 129, b-c-0

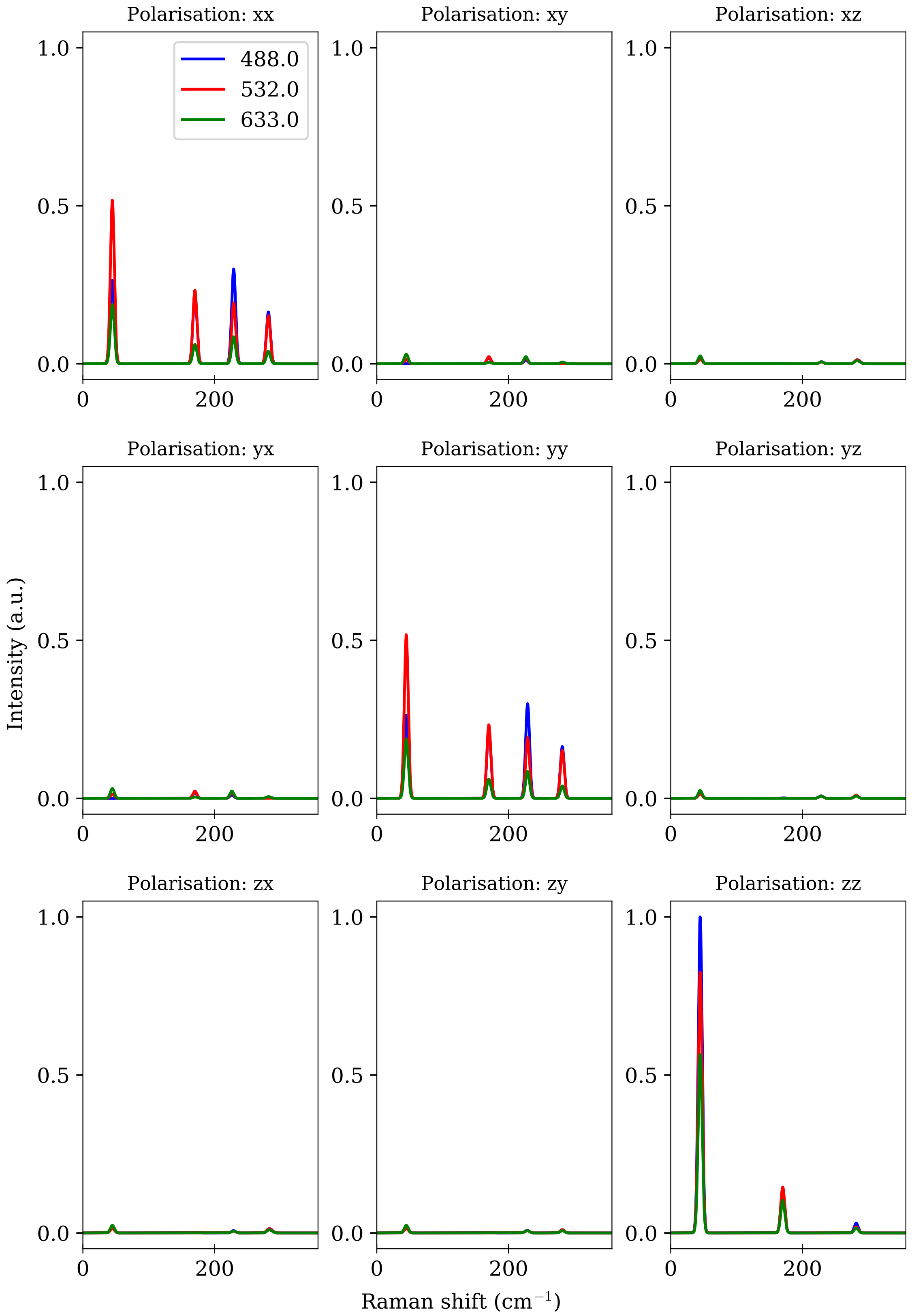

Supplementary Figure 154: Raman spectrum of Co_2Te_2 with space group 129 and Wyckoff positions b-c-0.

Co_2Te_2, 156, b-c-0

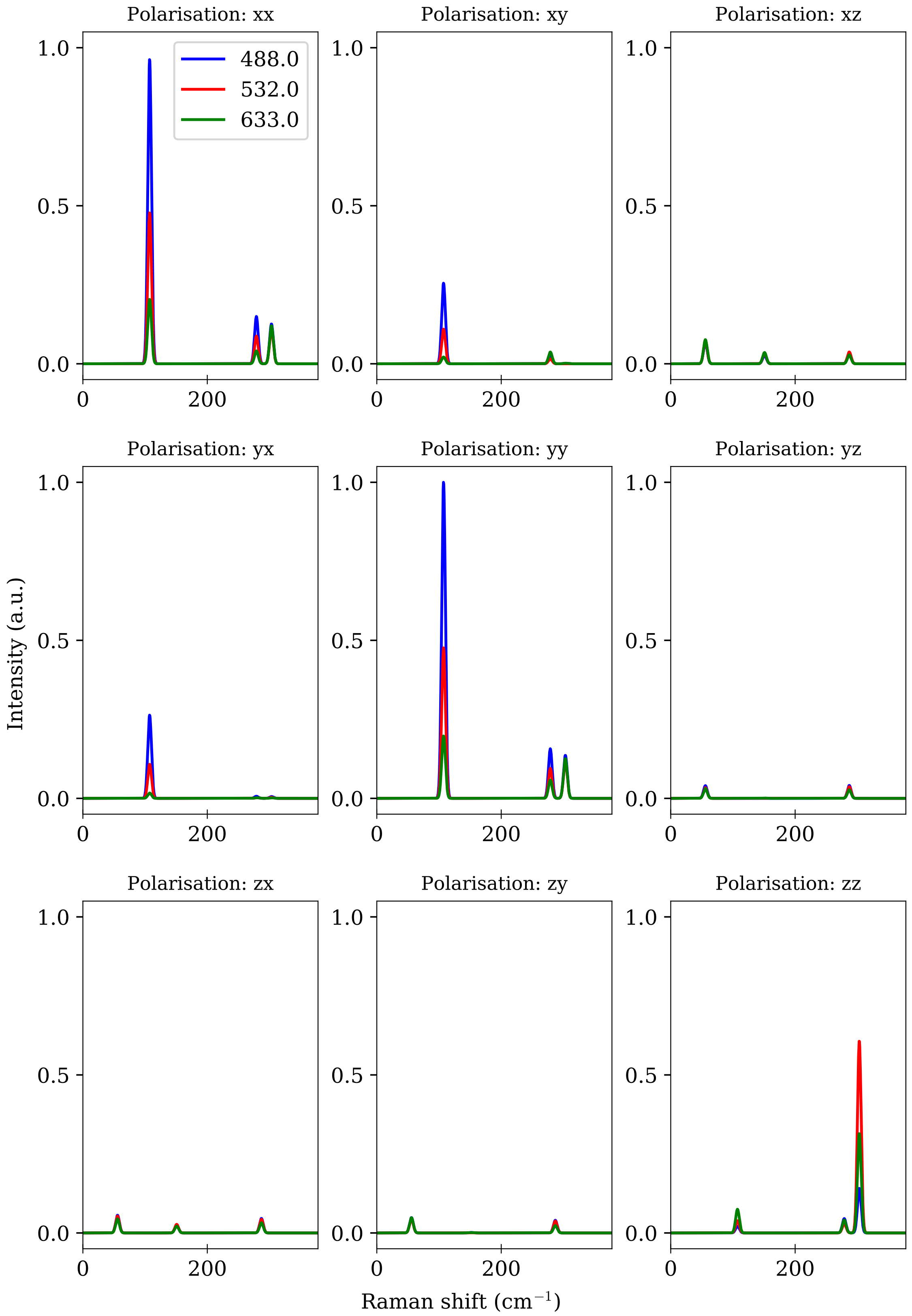

Supplementary Figure 155: Raman spectrum of Co_2Te_2 with space group 156 and Wyckoff positions b-c-0.

Co_2Te_2, 164, d-0

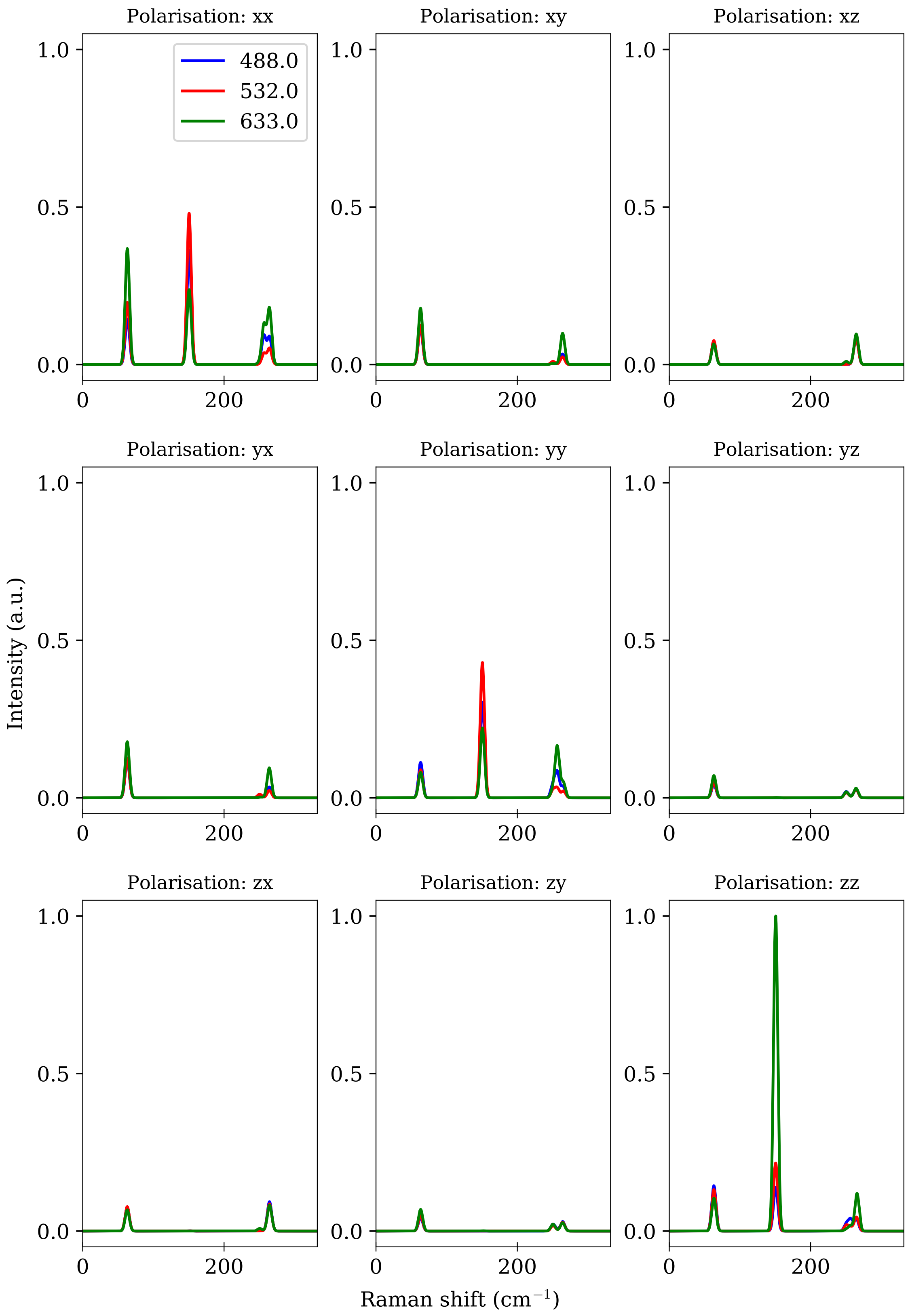

Supplementary Figure 156: Raman spectrum of Co_2Te_2 with space group 164 and Wyckoff positions d-0.

CoH_2, 187, b-i-0

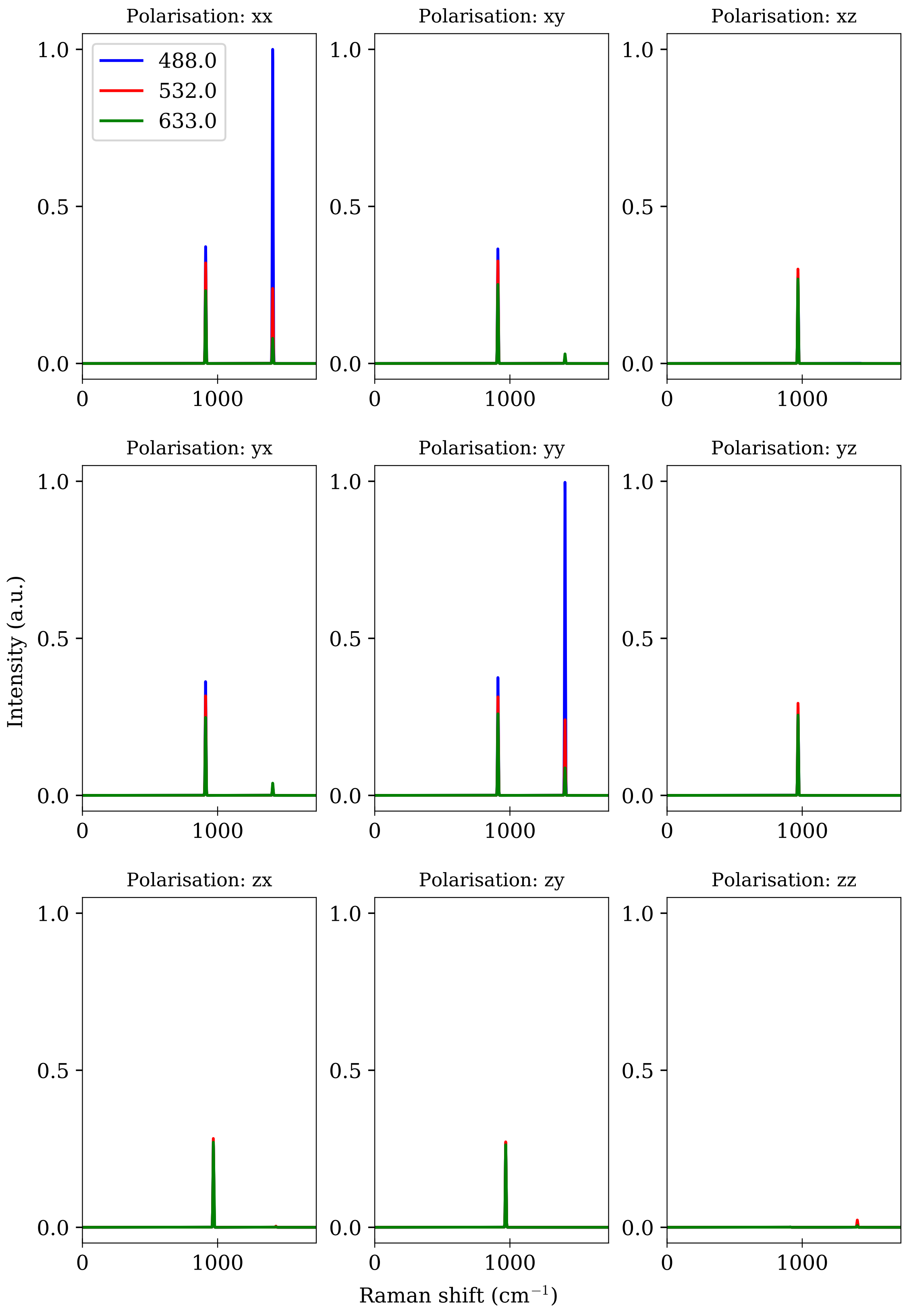

Supplementary Figure 157: Raman spectrum of CoH_2 with space group 187 and Wyckoff positions b-i-0.

CoO_2, 115, a-g-0

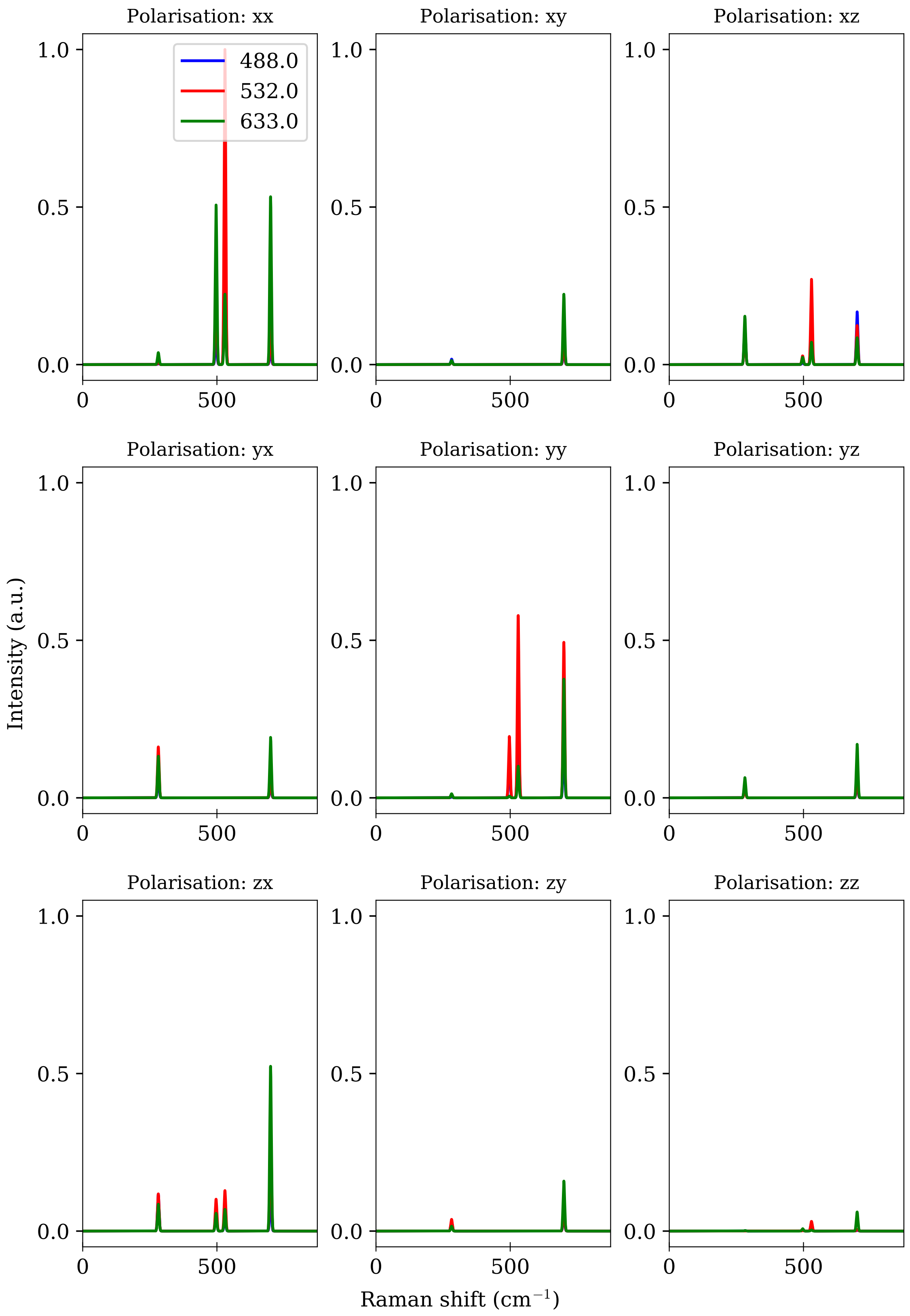

Supplementary Figure 158: Raman spectrum of CoO_2 with space group 115 and Wyckoff positions a-g-0.

$CoTe_2$, 187, b-i-0

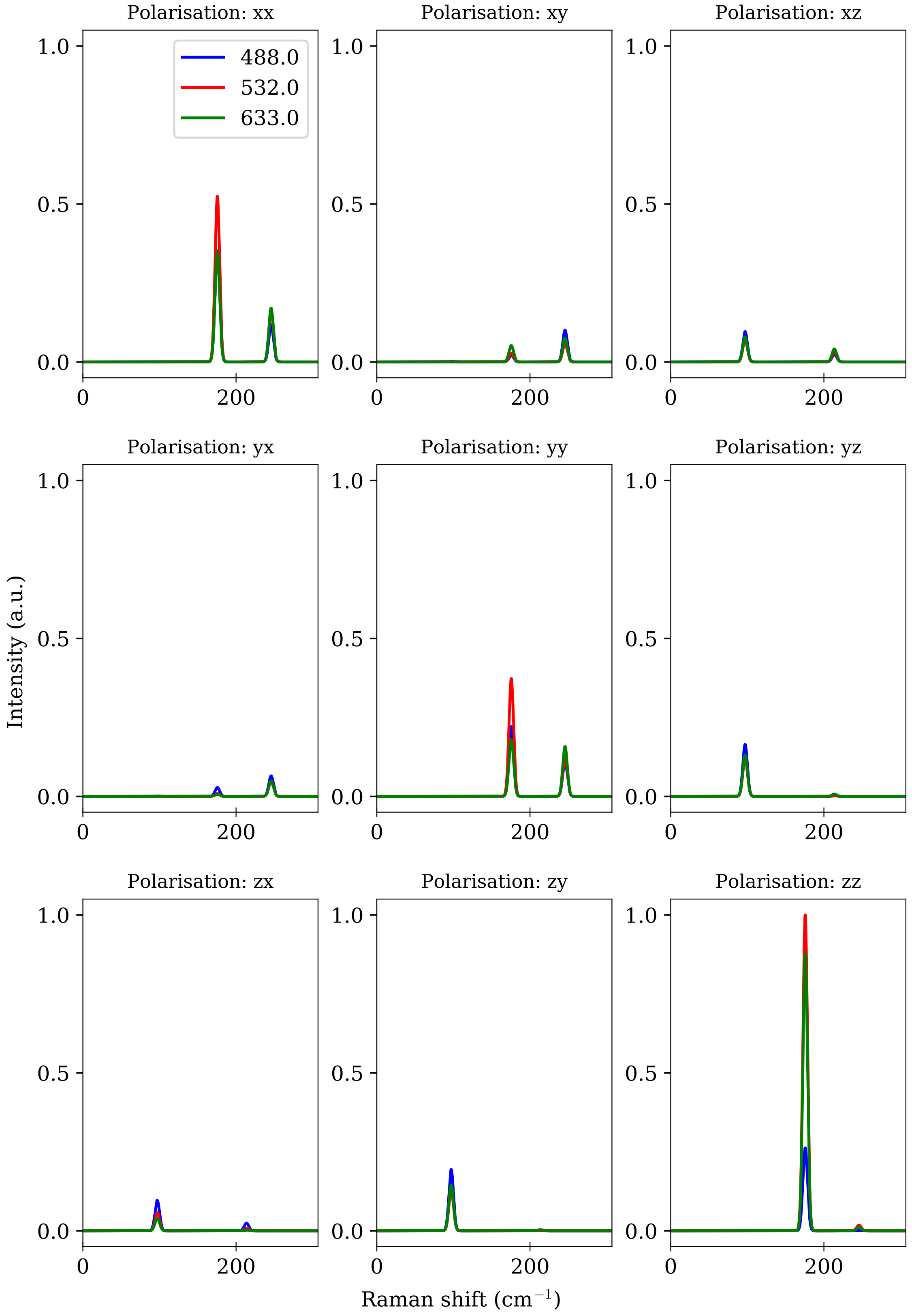

Supplementary Figure 159: Raman spectrum of $CoTe_2$ with space group 187 and Wyckoff positions b-i-0.

Cr_2C, 164, b-d-0

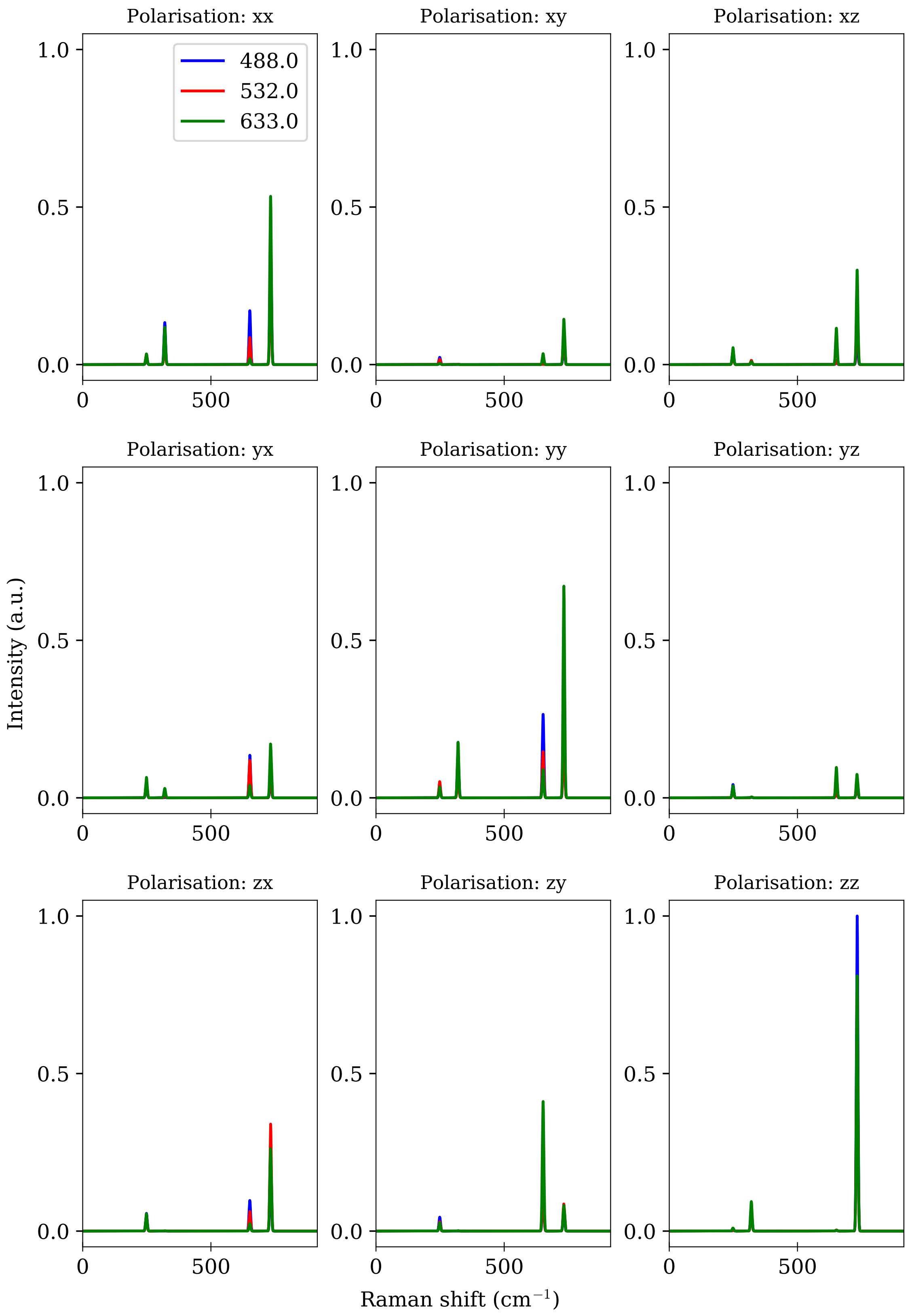

Supplementary Figure 160: Raman spectrum of Cr_2C with space group 164 and Wyckoff positions b-d-0.

Cr_2N, 164, b-d-0

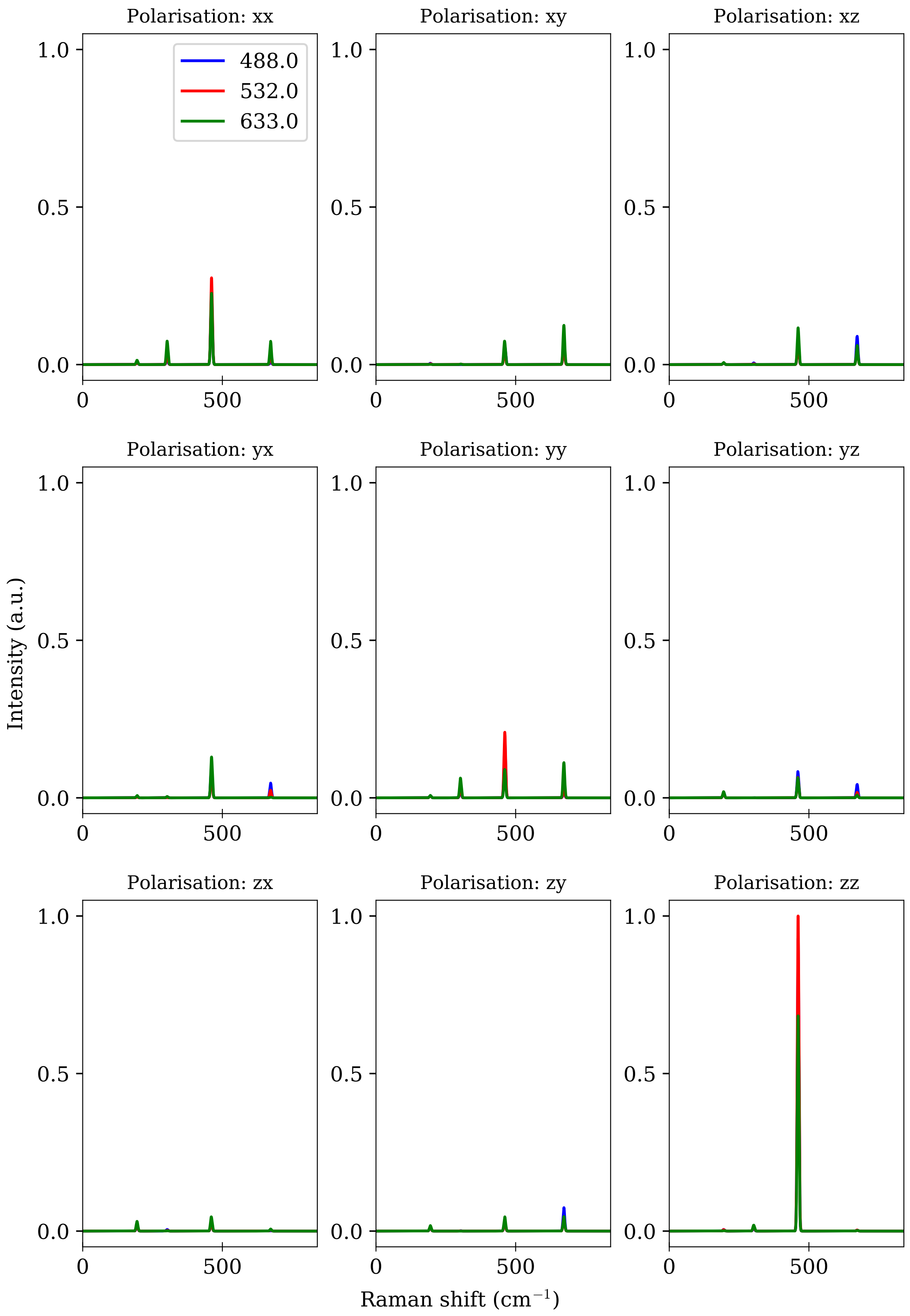

Supplementary Figure 161: Raman spectrum of Cr_2N with space group 164 and Wyckoff positions b-d-0.

Cr_2O_4, 11, e-0

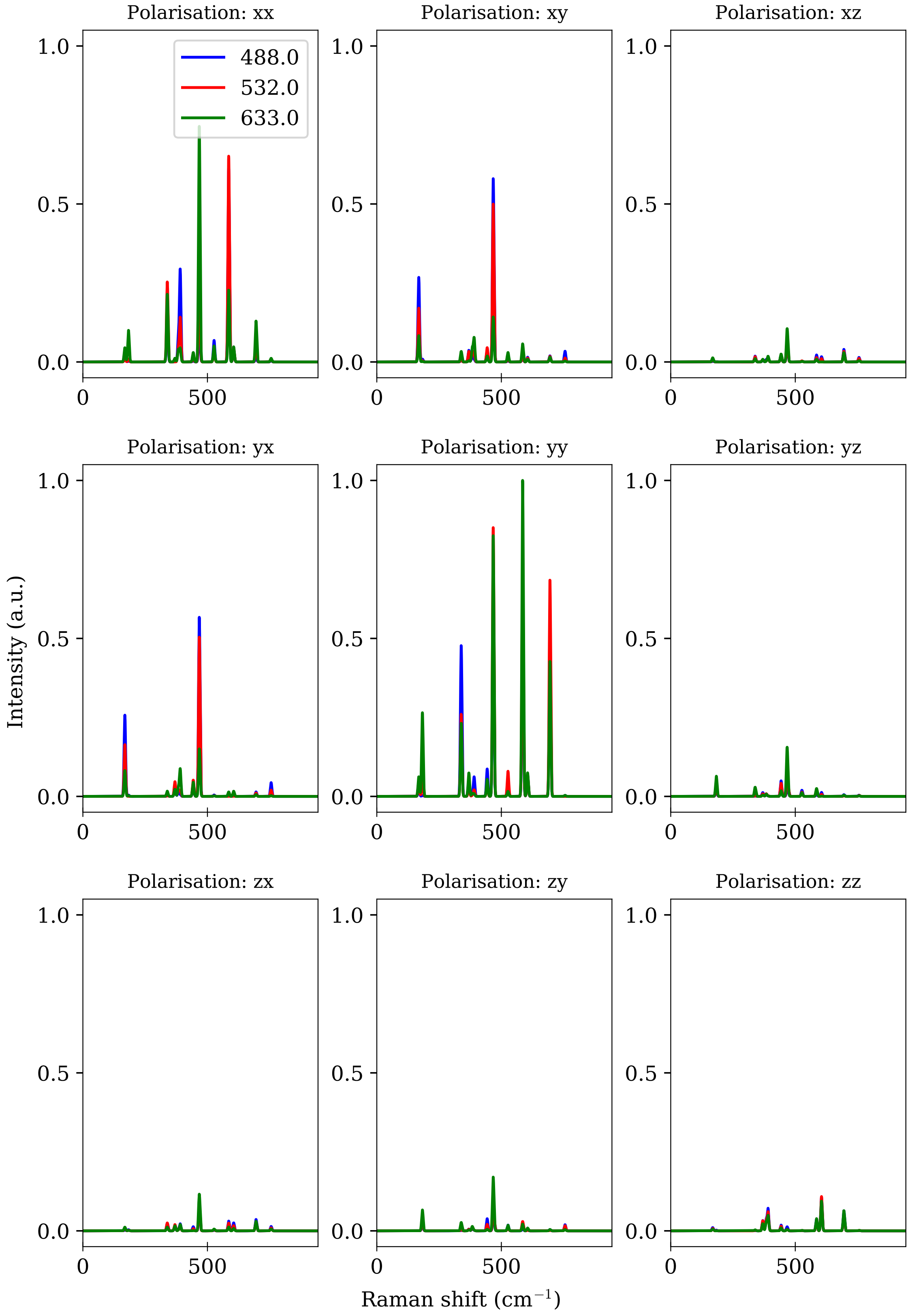

Supplementary Figure 162: Raman spectrum of Cr_2O_4 with space group 11 and Wyckoff positions e-0.

Cr_2S_4, 11, e-0

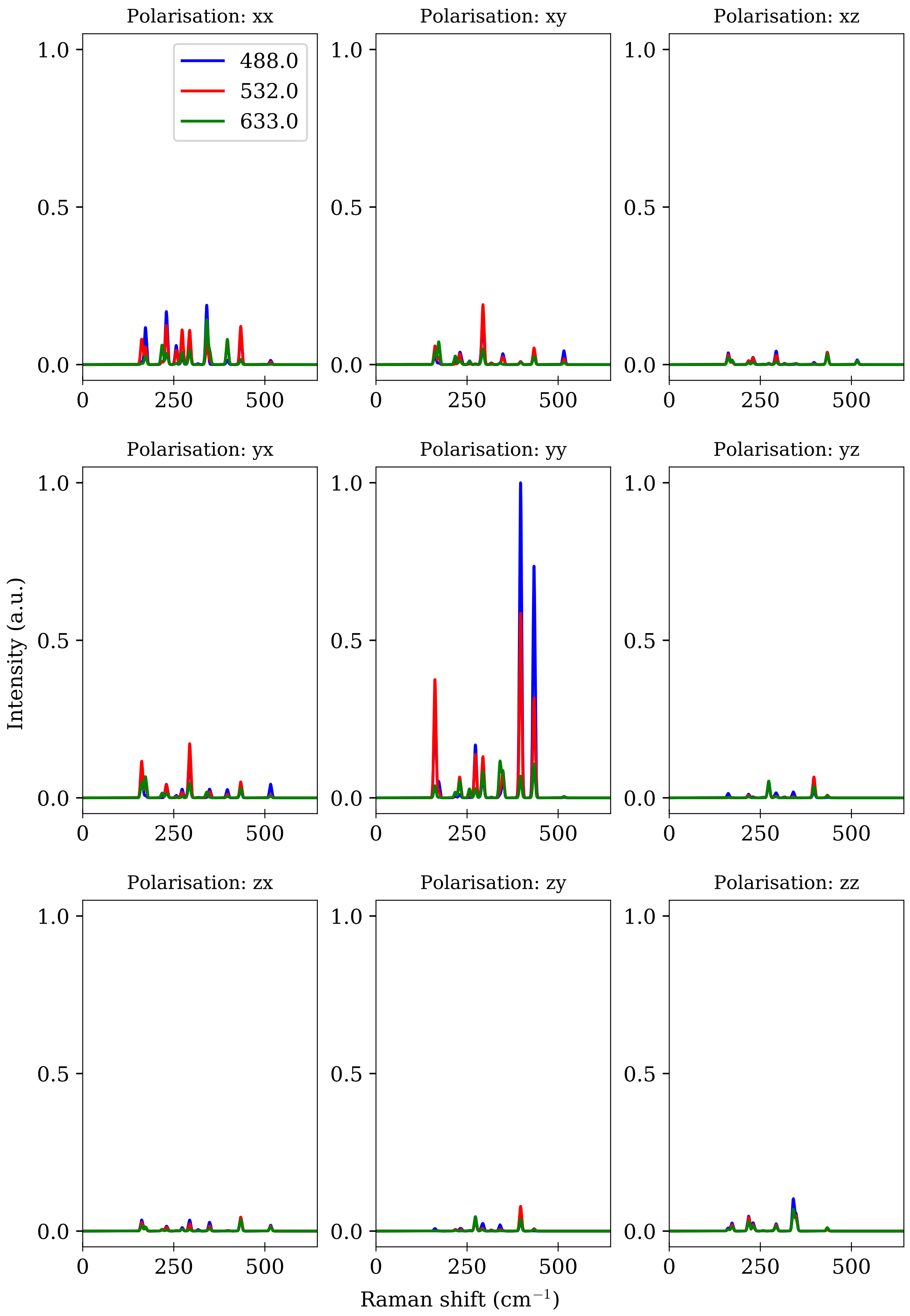

Supplementary Figure 163: Raman spectrum of Cr_2S_4 with space group 11 and Wyckoff positions e-0.

Cr_2Se_4, 11, e-0

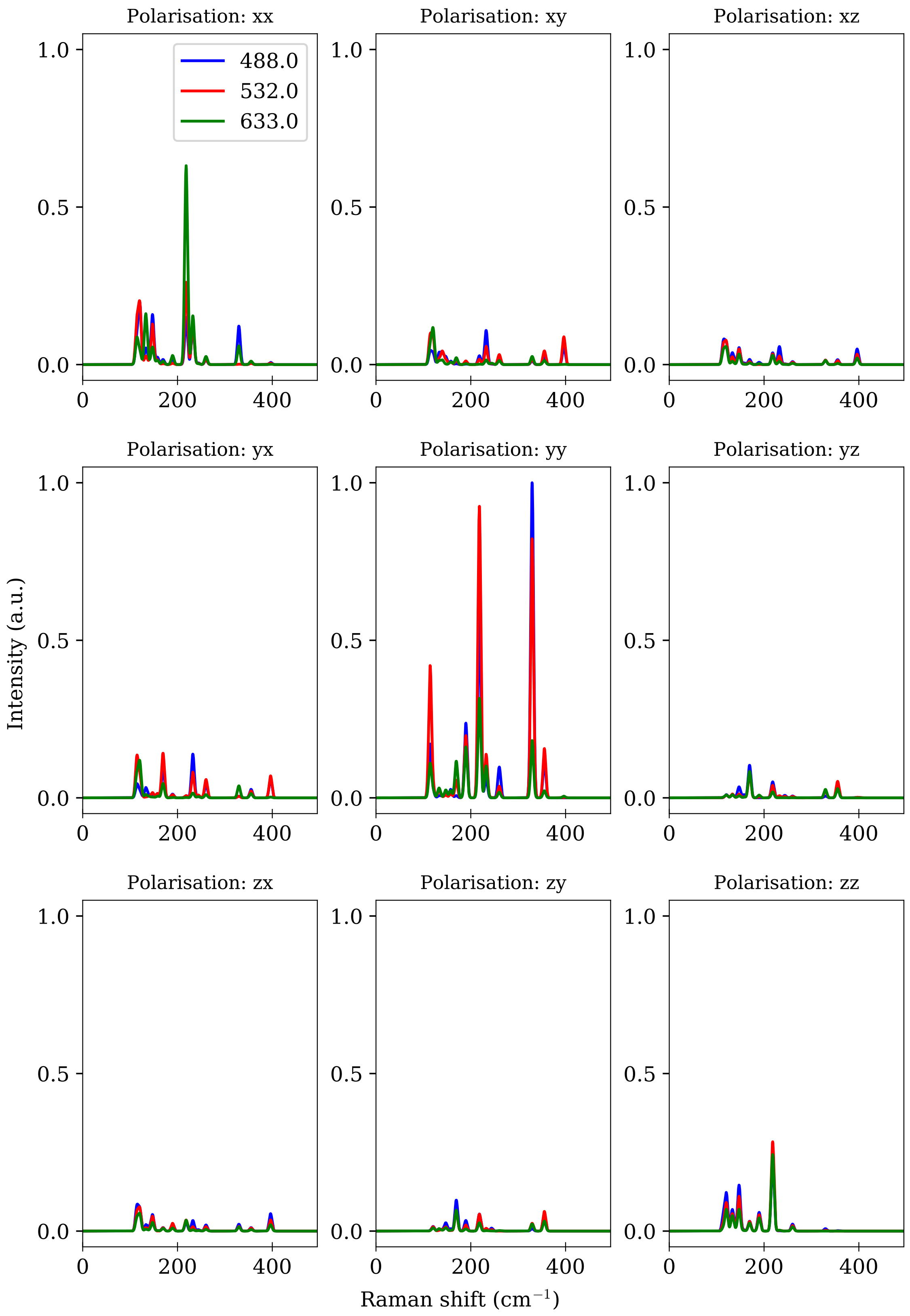

Supplementary Figure 164: Raman spectrum of Cr_2Se_4 with space group 11 and Wyckoff positions e-0.

Cr_3N_2, 187, a-h-i-0

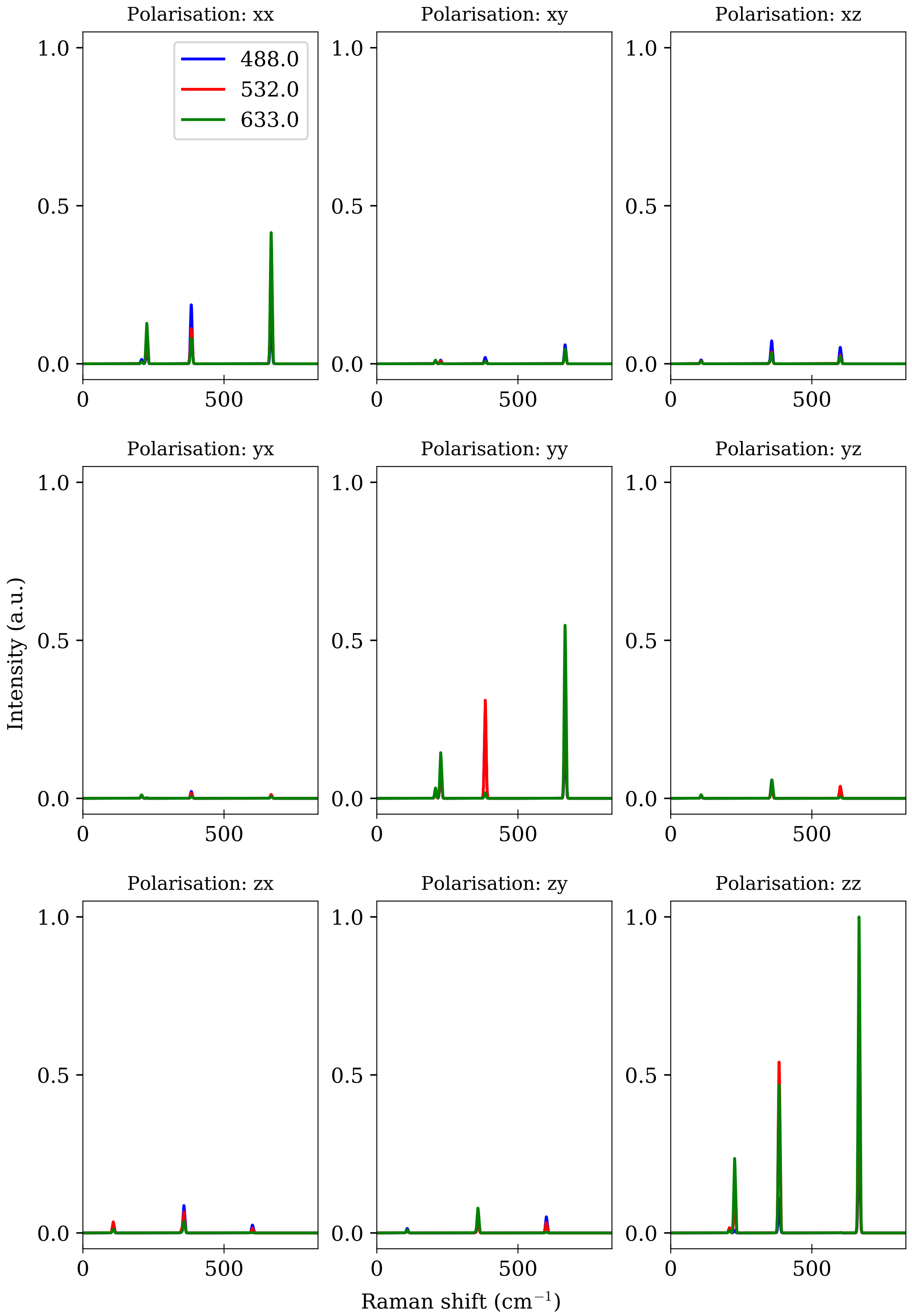

Supplementary Figure 165: Raman spectrum of Cr_3N_2 with space group 187 and Wyckoff positions a-h-i-0.

$CrAs_2$, 164, b-d-0

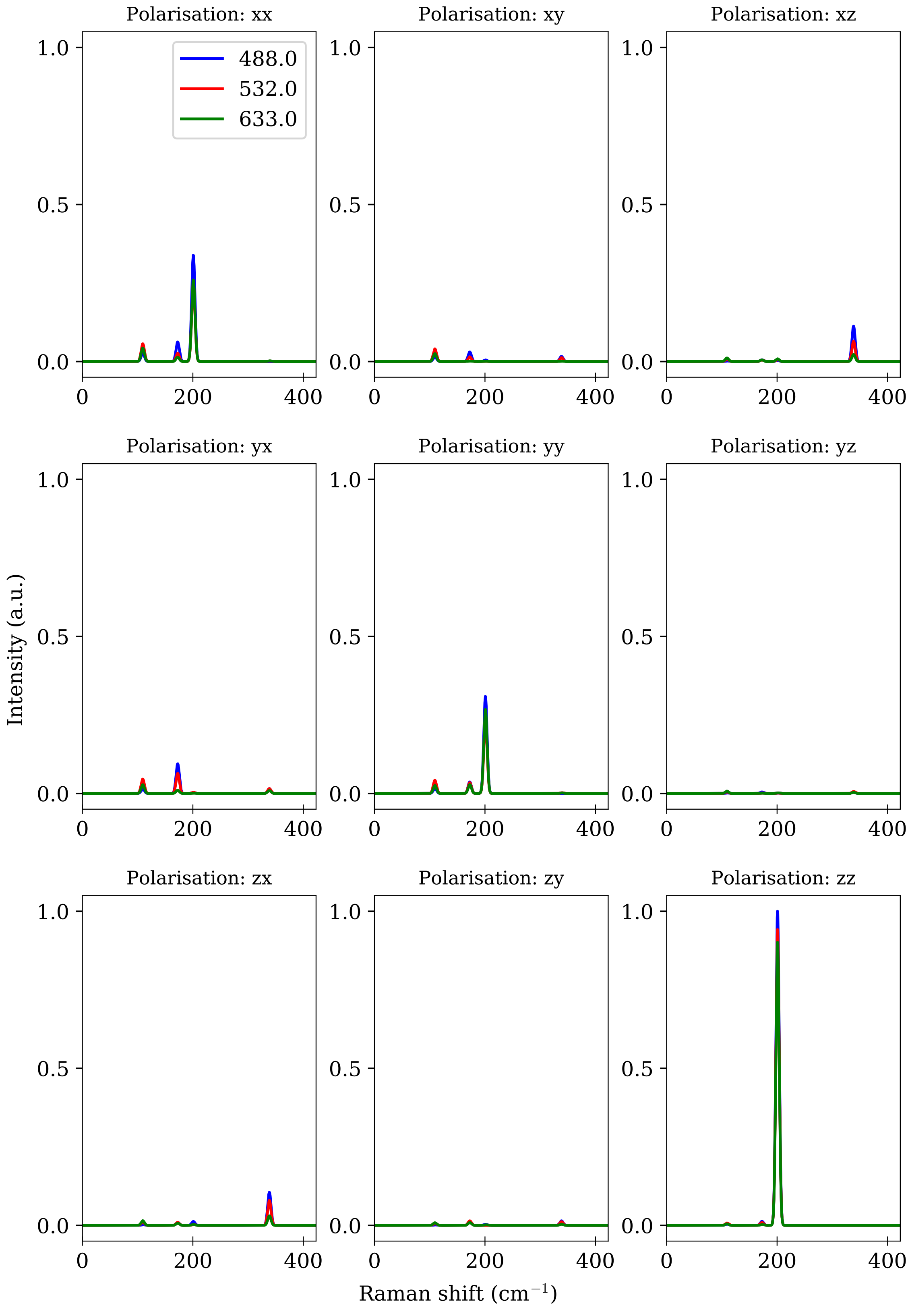

Supplementary Figure 166: Raman spectrum of $CrAs_2$ with space group 164 and Wyckoff positions b-d-0.

$CrAs_2$, 187, b-i-0

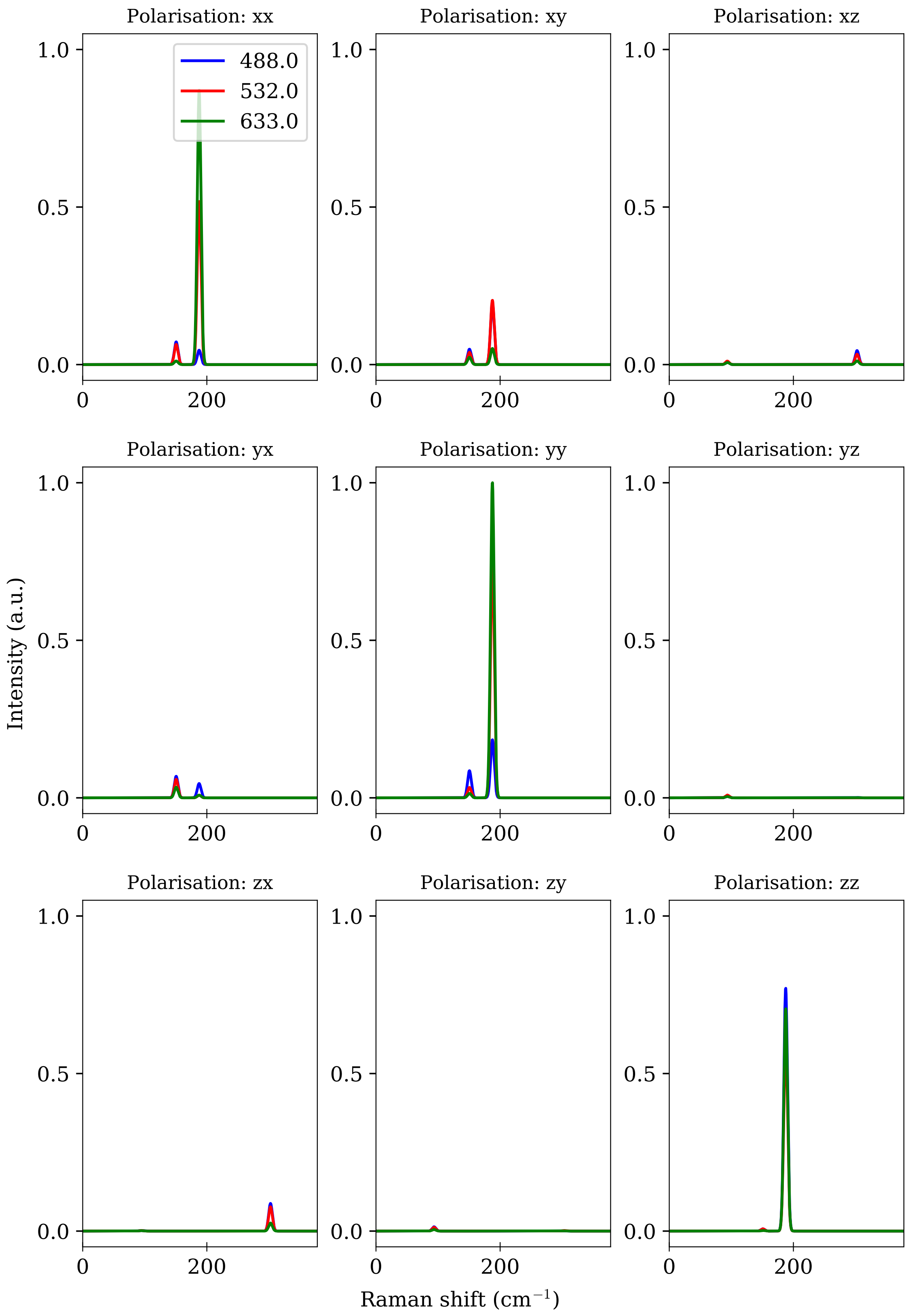

Supplementary Figure 167: Raman spectrum of $CrAs_2$ with space group 187 and Wyckoff positions b-i-0.

CrAsSb, 156, a-b-c-0

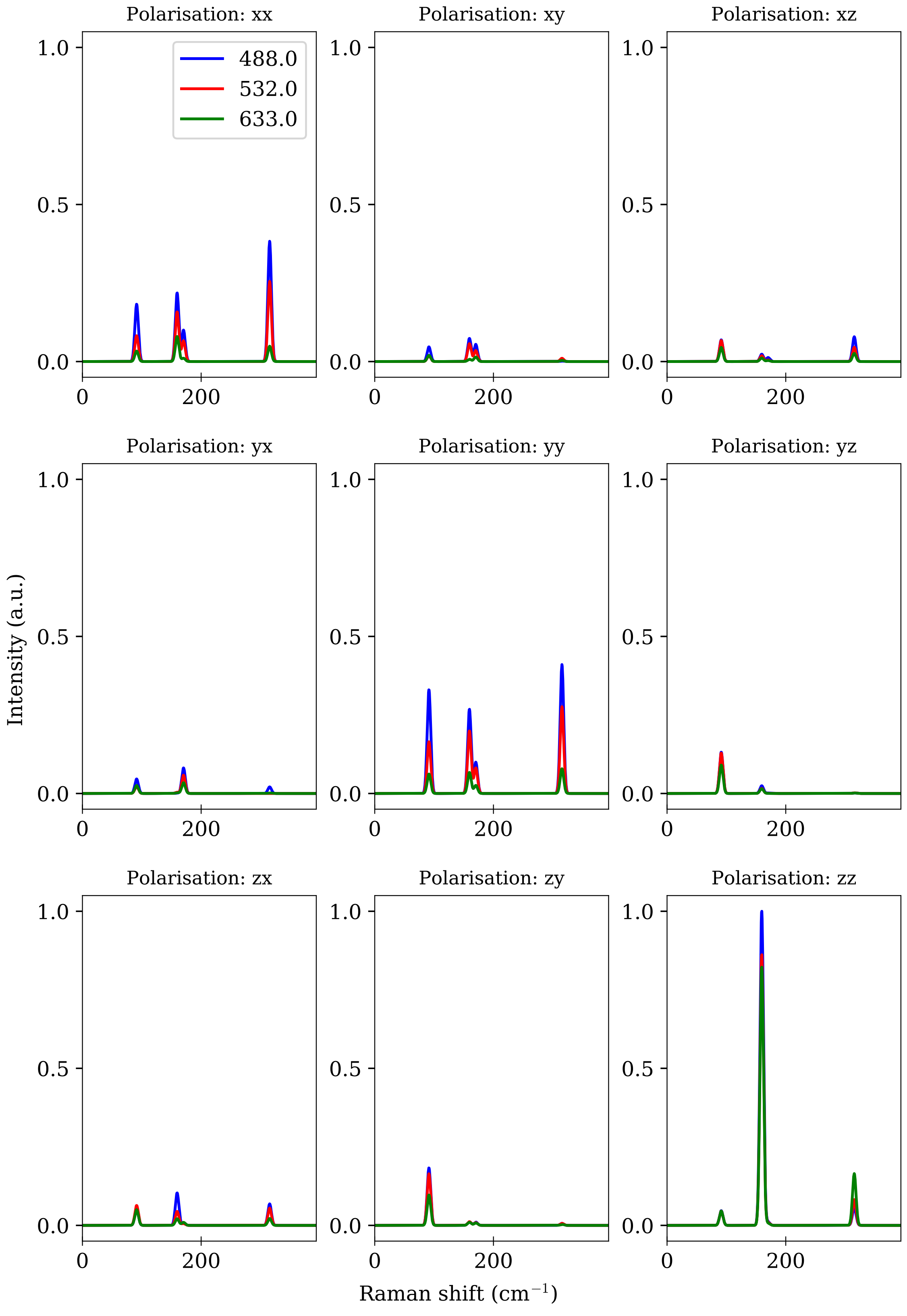

Supplementary Figure 168: Raman spectrum of CrAsSb with space group 156 and Wyckoff positions a-b-c-0.

CrO_2, 187, b-i-0

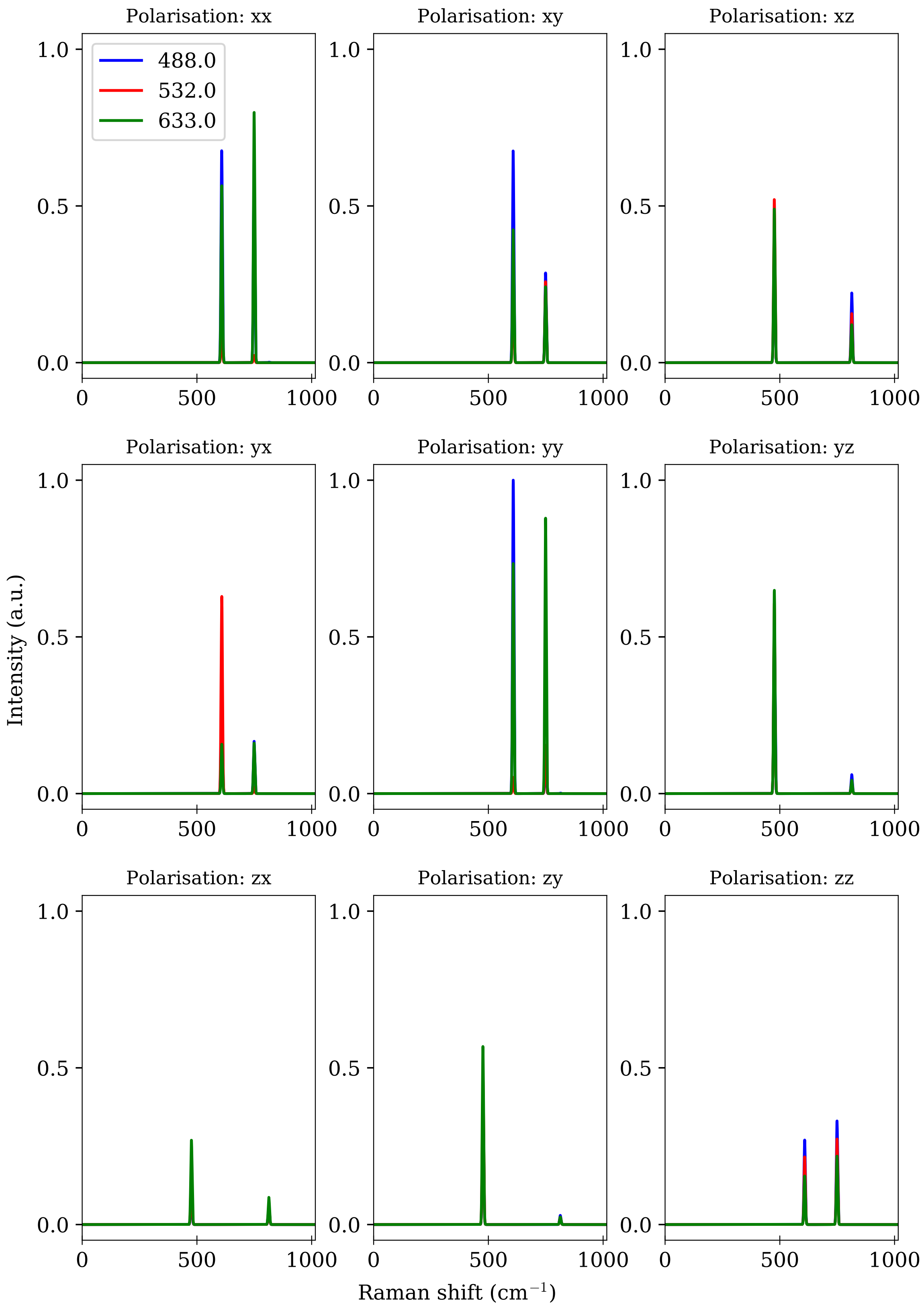

Supplementary Figure 169: Raman spectrum of CrO_2 with space group 187 and Wyckoff positions b-i-0.

CrS_2, 187, b-i-0

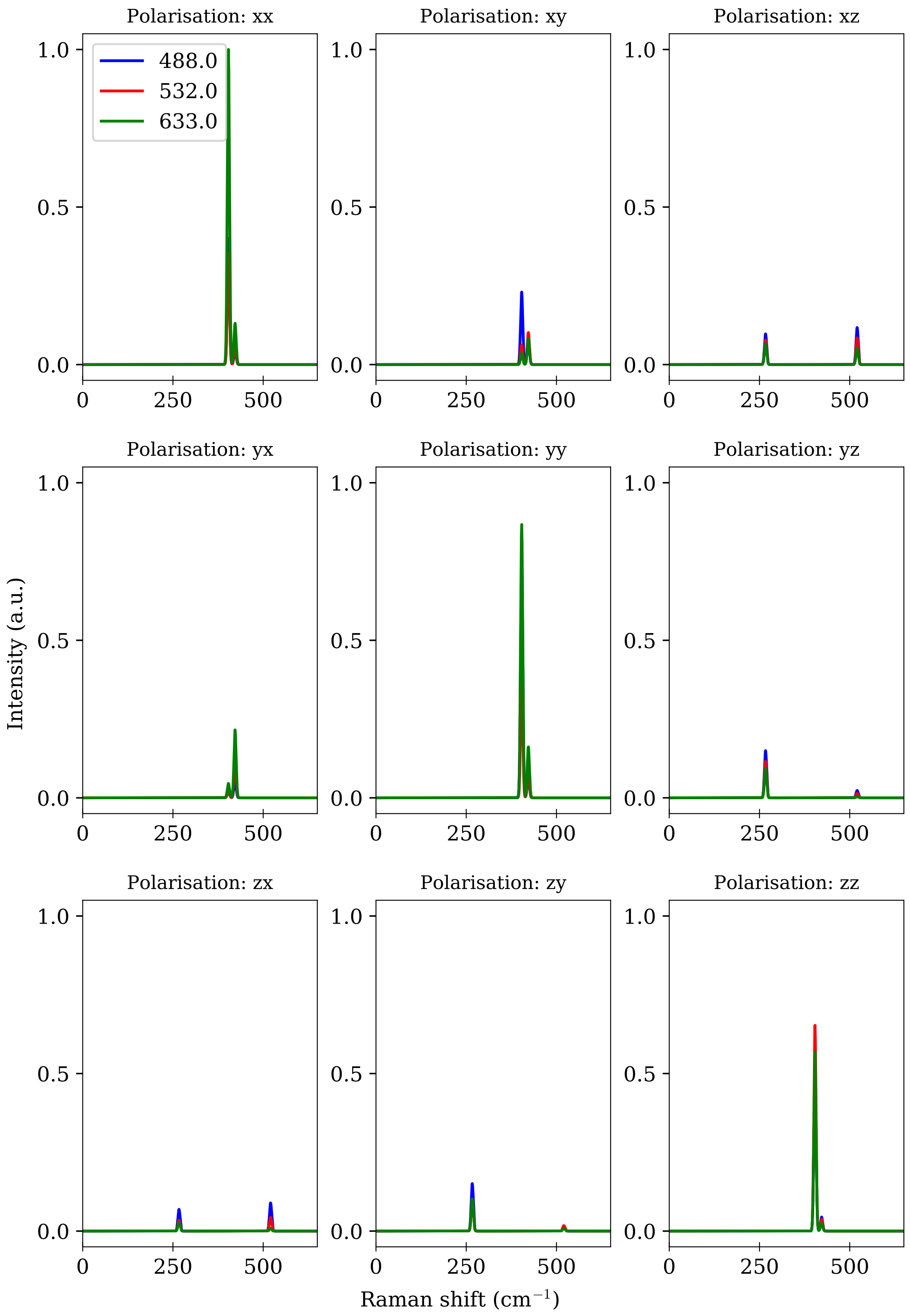

Supplementary Figure 170: Raman spectrum of CrS_2 with space group 187 and Wyckoff positions b-i-0.

$CrSe_2$, 187, b-i-0

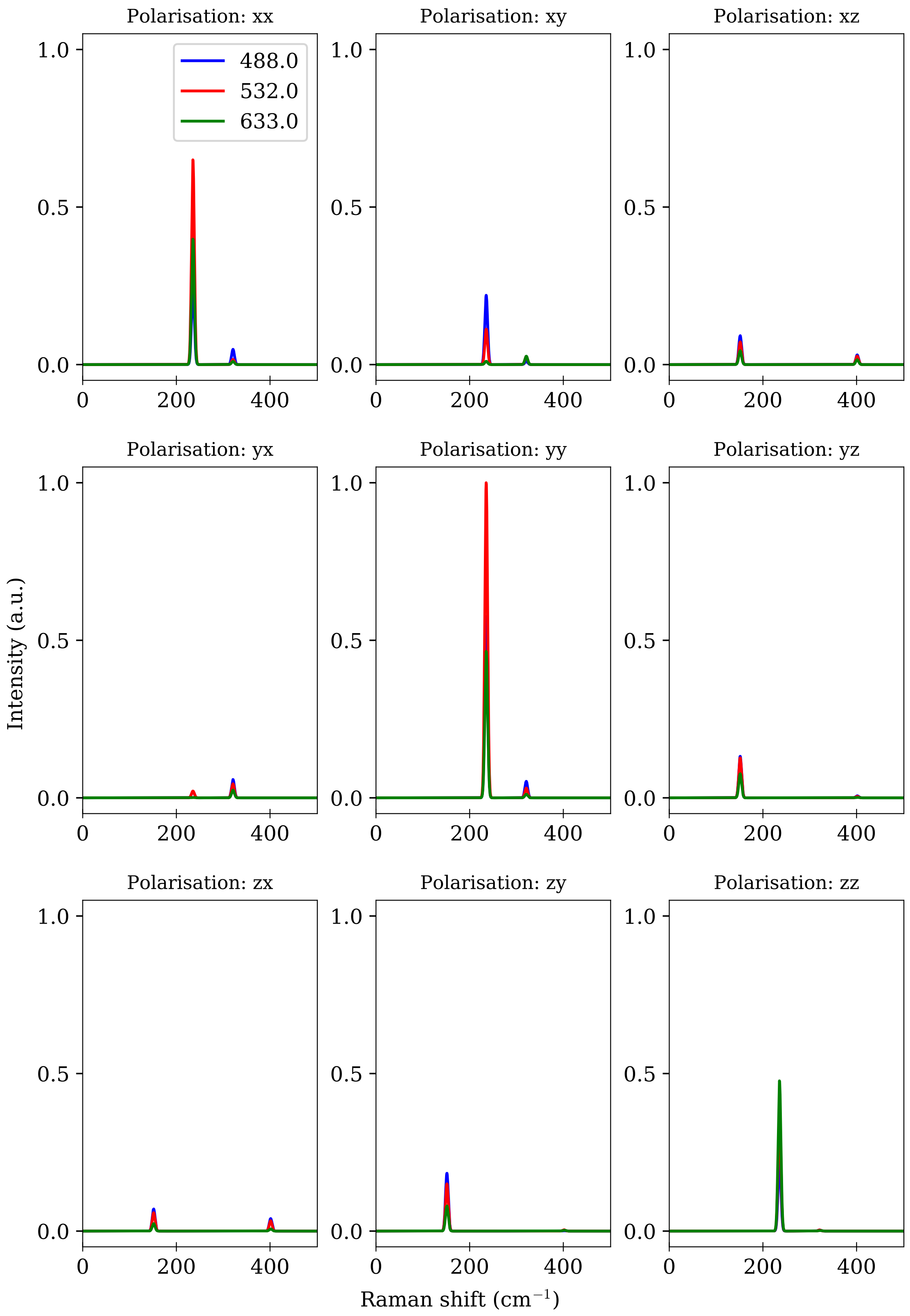

Supplementary Figure 171: Raman spectrum of $CrSe_2$ with space group 187 and Wyckoff positions b-i-0.

CrSeTe, 156, a-c-0

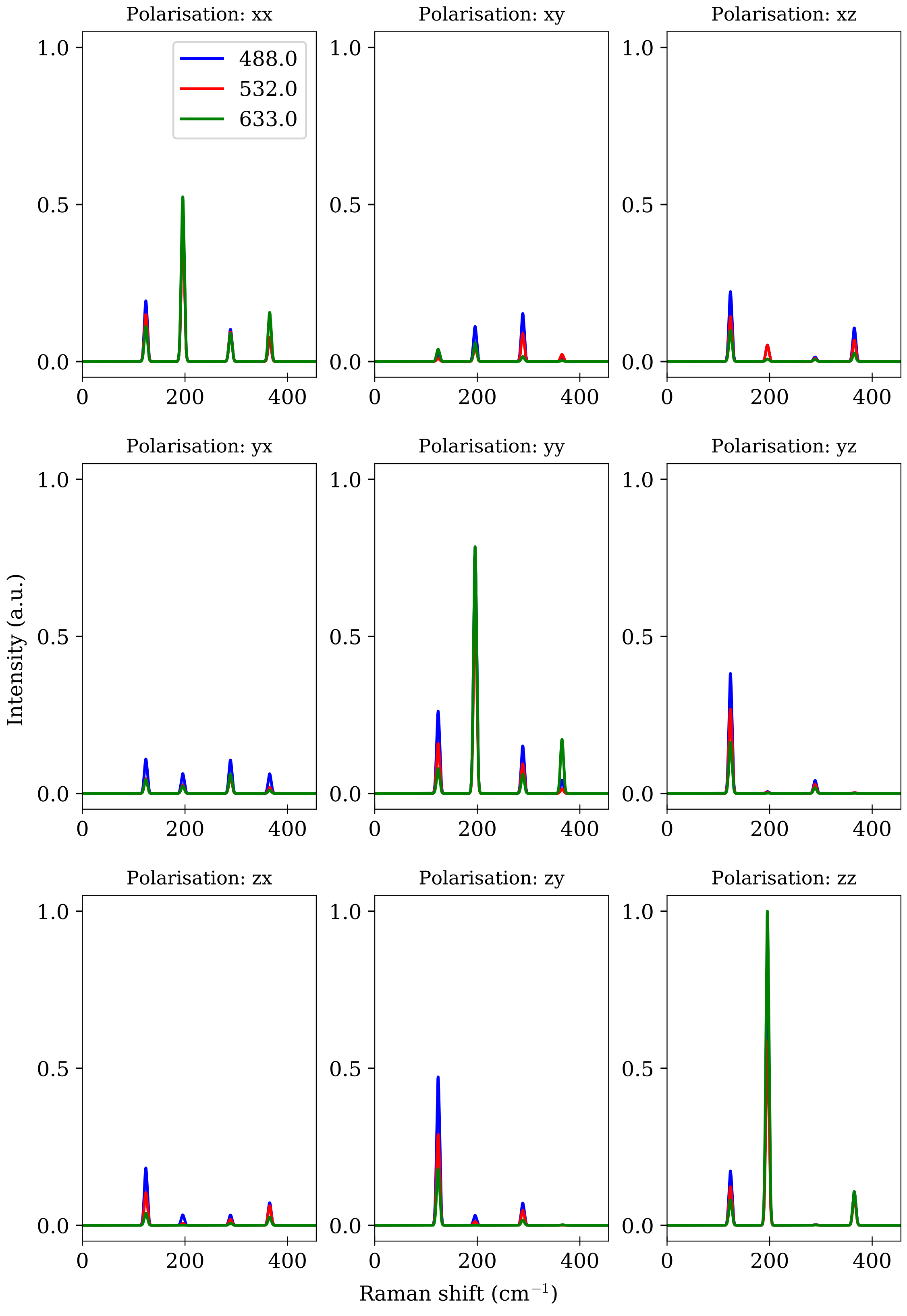

Supplementary Figure 172: Raman spectrum of CrSeTe with space group 156 and Wyckoff positions a-c-0.

CrSSe, 156, a-c-0

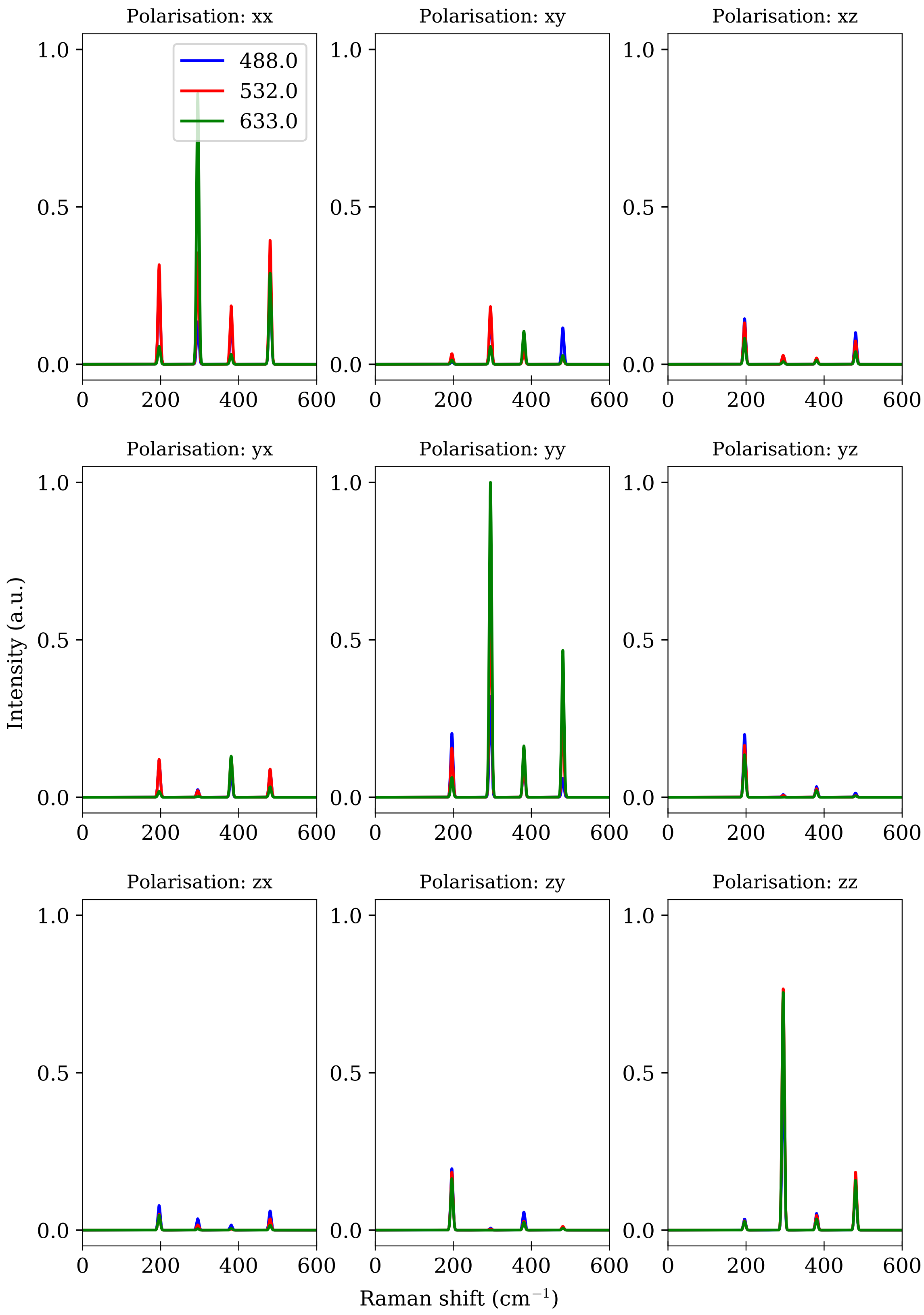

Supplementary Figure 173: Raman spectrum of CrSSe with space group 156 and Wyckoff positions a-c-0.

CrSTe, 156, a-c-0

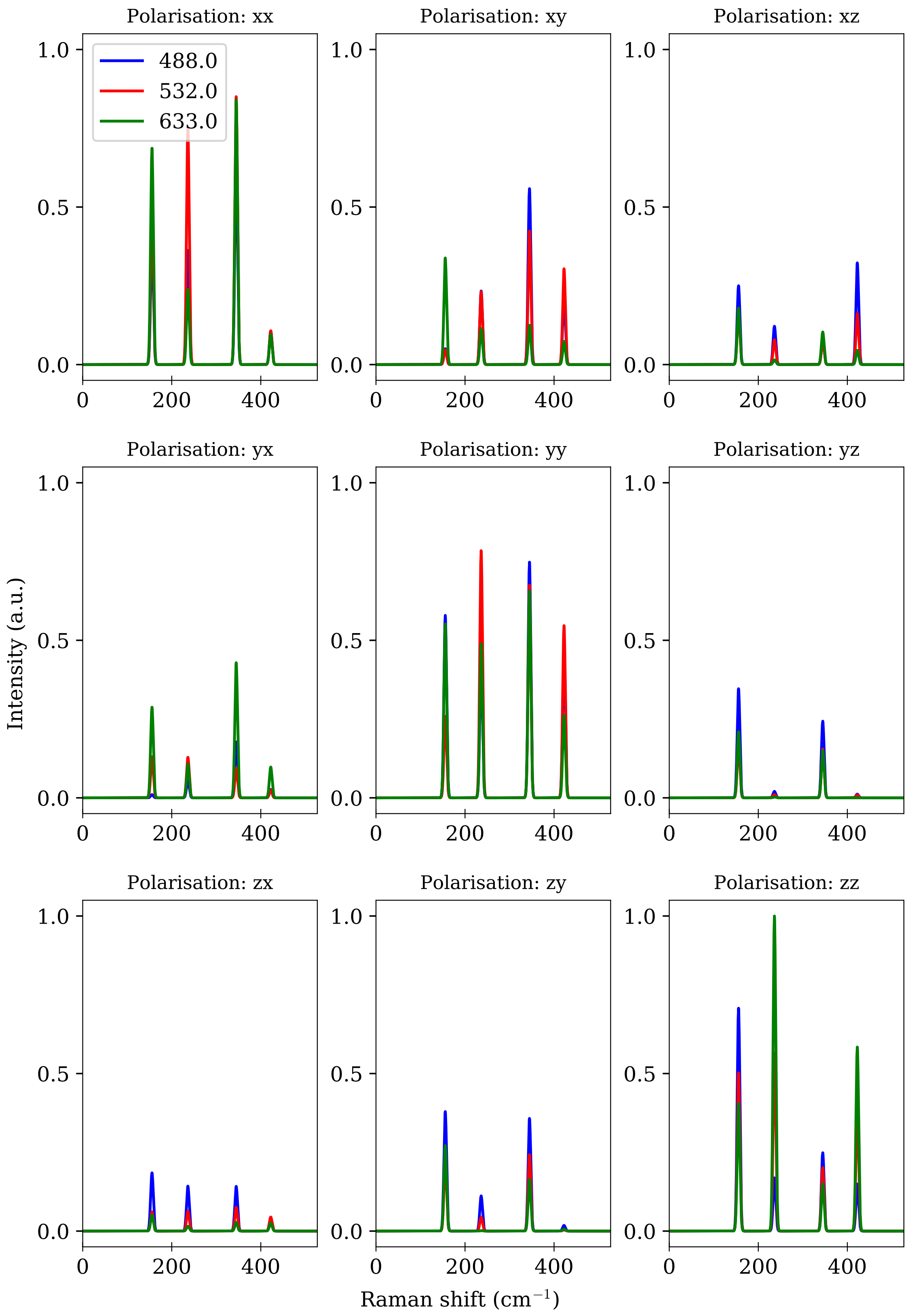

Supplementary Figure 174: Raman spectrum of CrSTe with space group 156 and Wyckoff positions a-c-0.

$CrTe_2$, 187, b-i-0

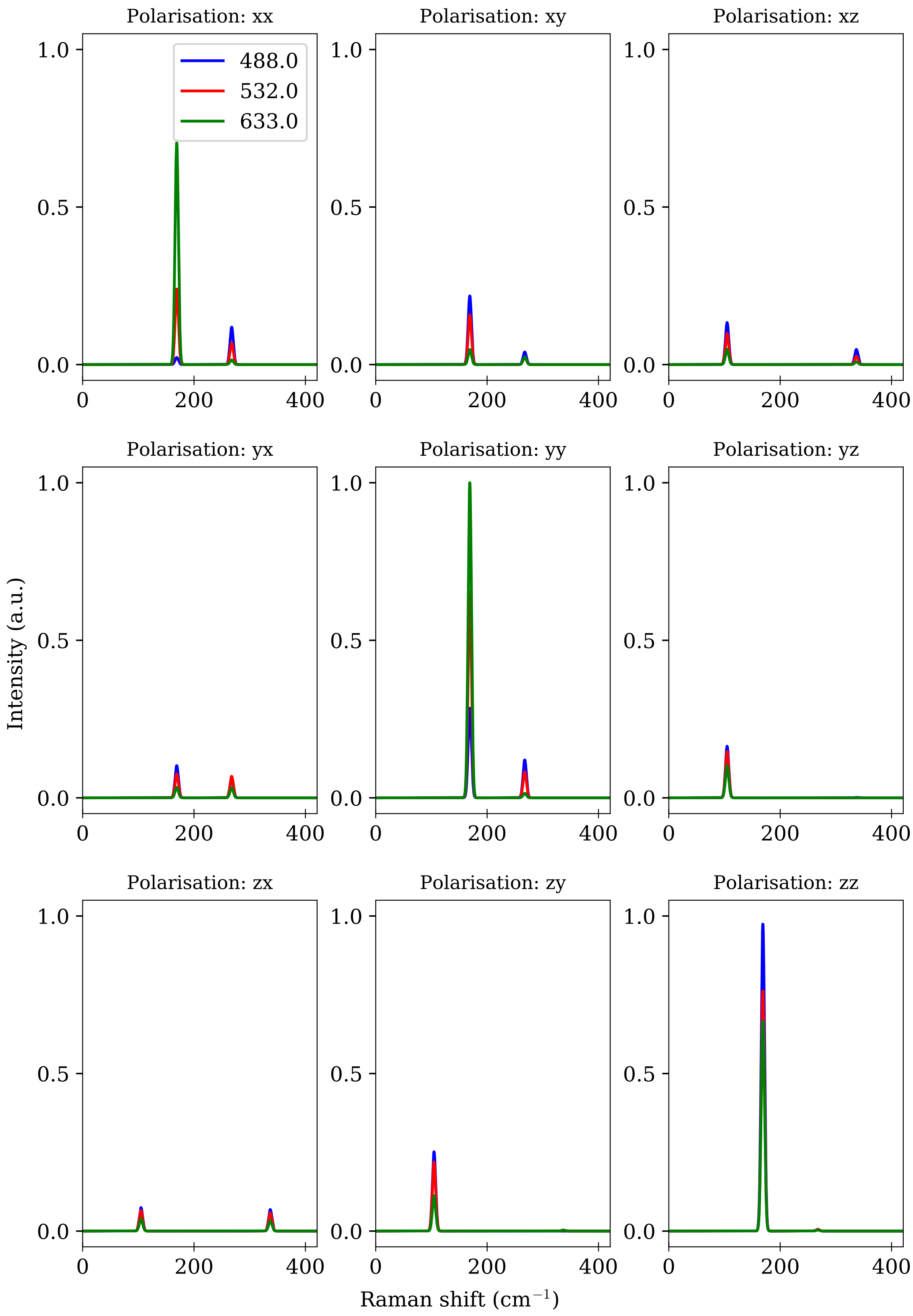

Supplementary Figure 175: Raman spectrum of $CrTe_2$ with space group 187 and Wyckoff positions b-i-0.

Cs_2F_2, 129, b-c-0

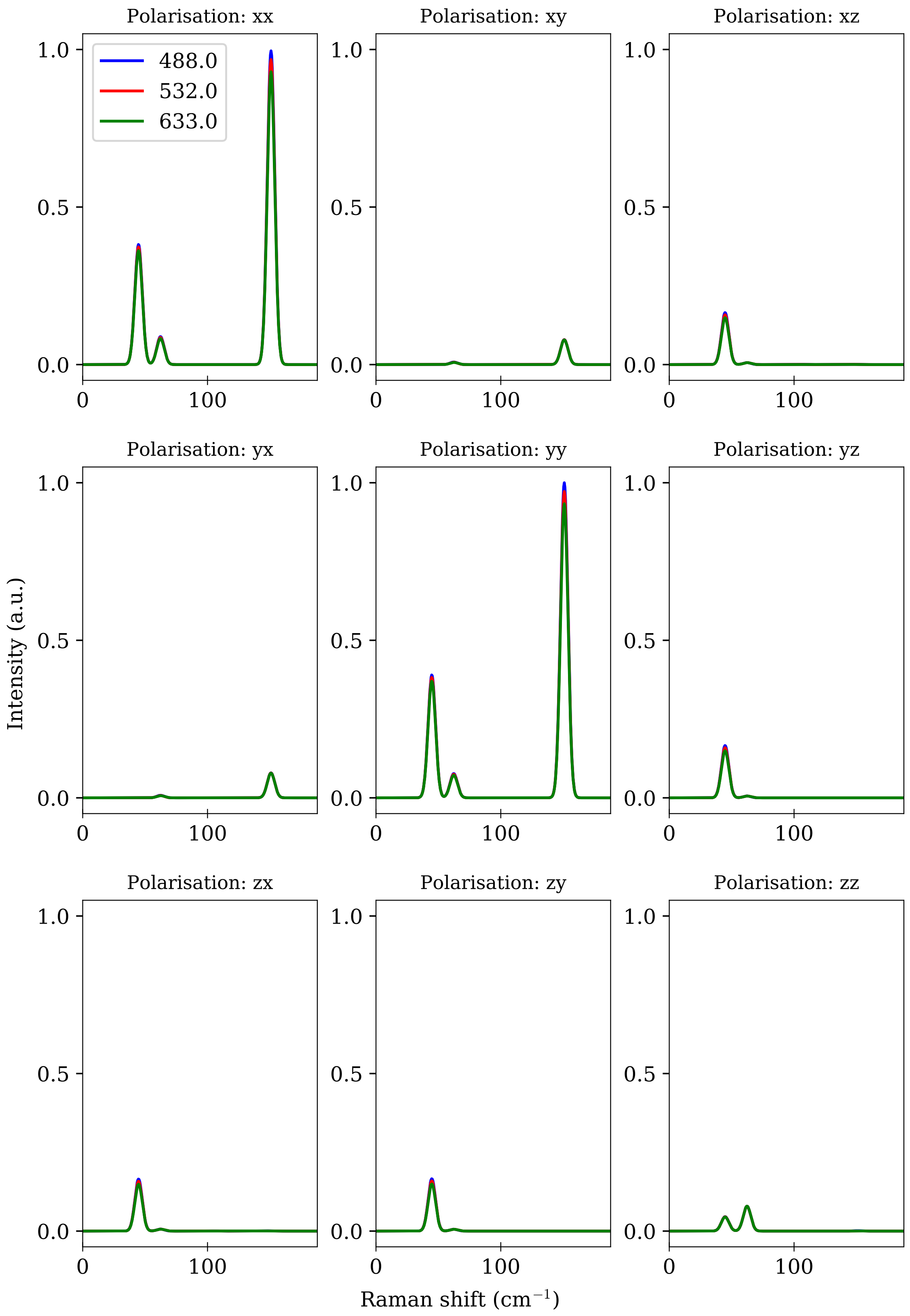

Supplementary Figure 176: Raman spectrum of Cs_2F_2 with space group 129 and Wyckoff positions b-c-0.

Cu_2Br_2, 129, b-c-0

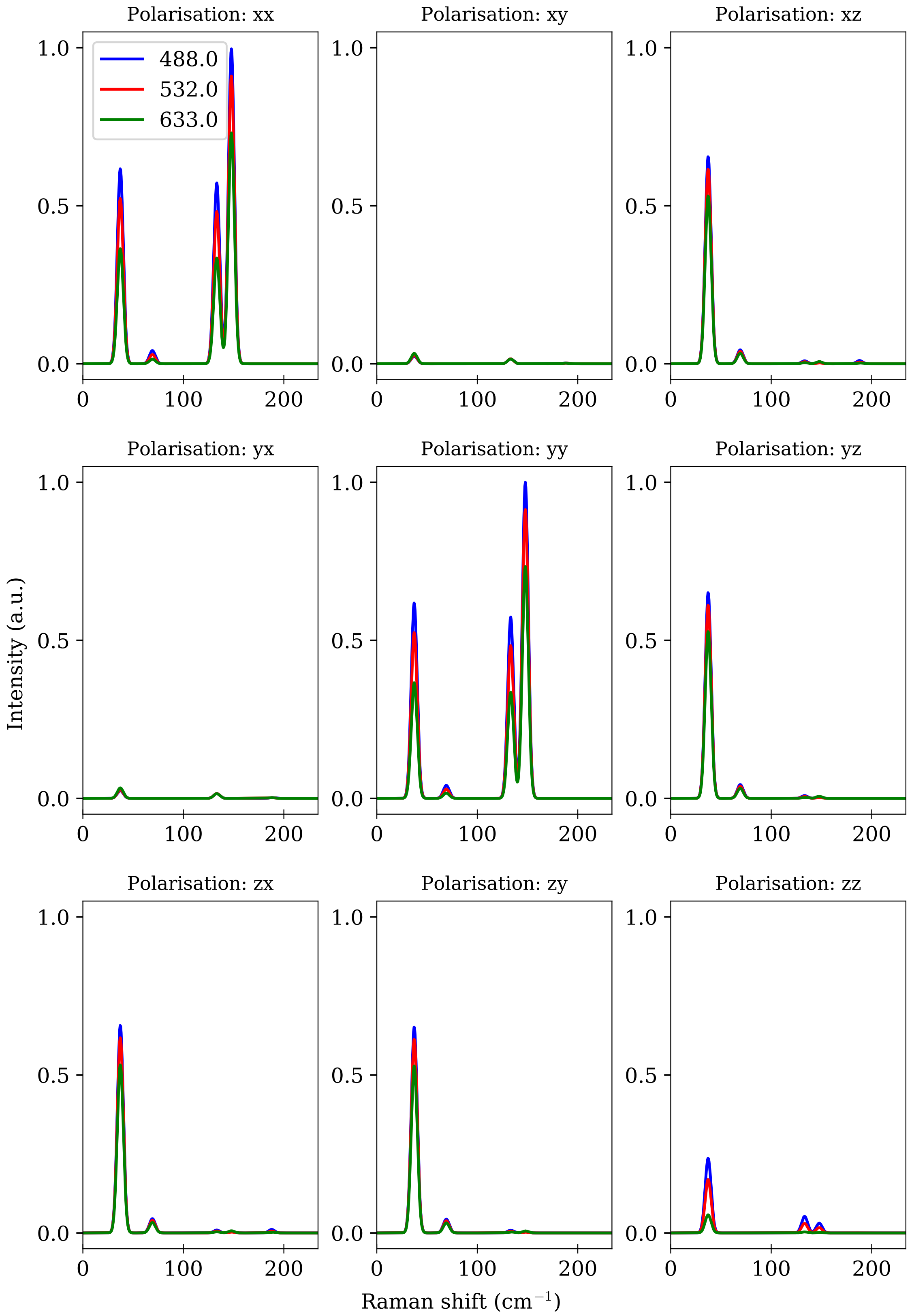

Supplementary Figure 177: Raman spectrum of Cu_2Br_2 with space group 129 and Wyckoff positions b-c-0.

Cu_2Br_2, 2, i-0

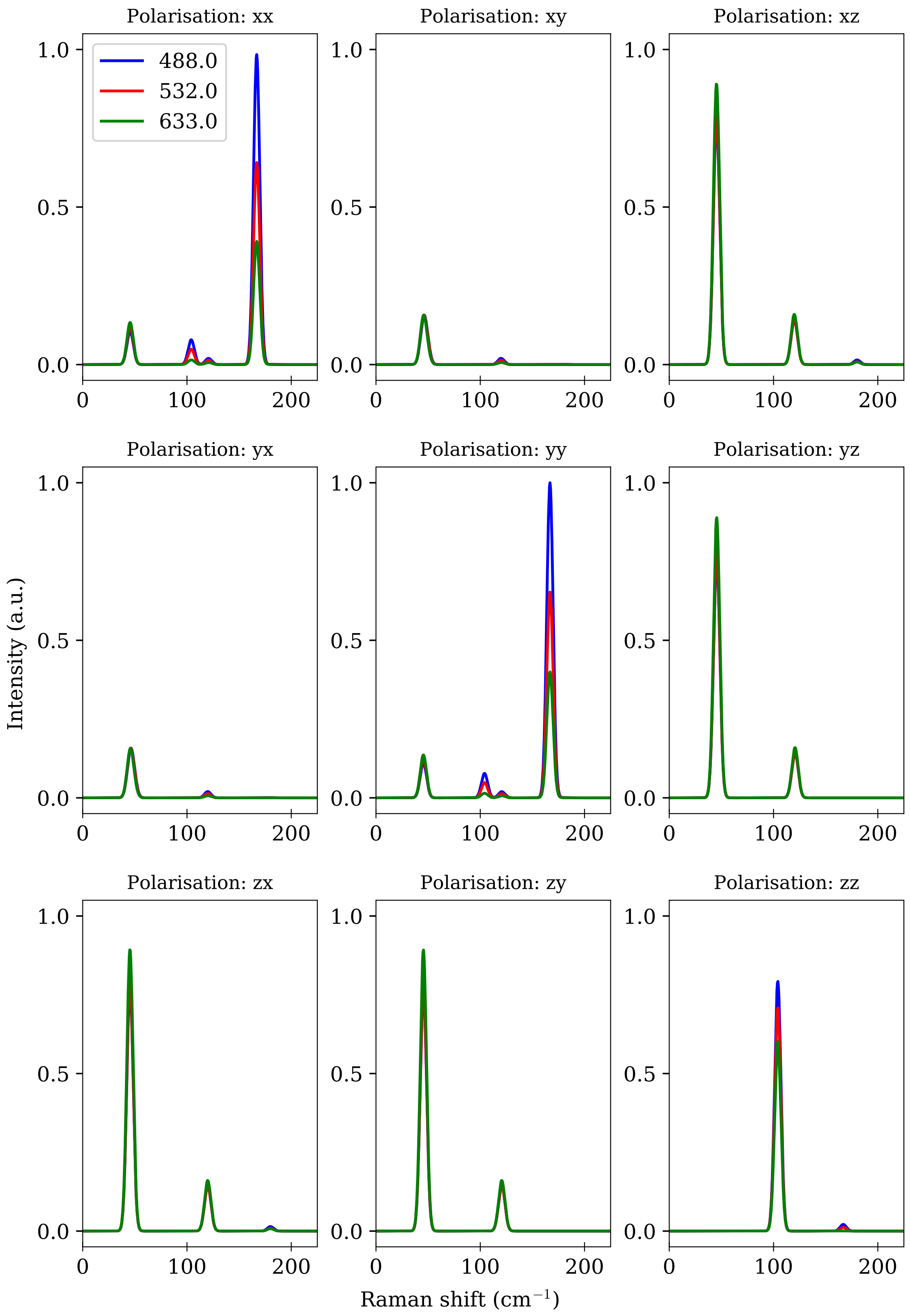

Supplementary Figure 178: Raman spectrum of Cu_2Br_2 with space group 2 and Wyckoff positions i-0.

Cu_2I_2, 156, a-b-0

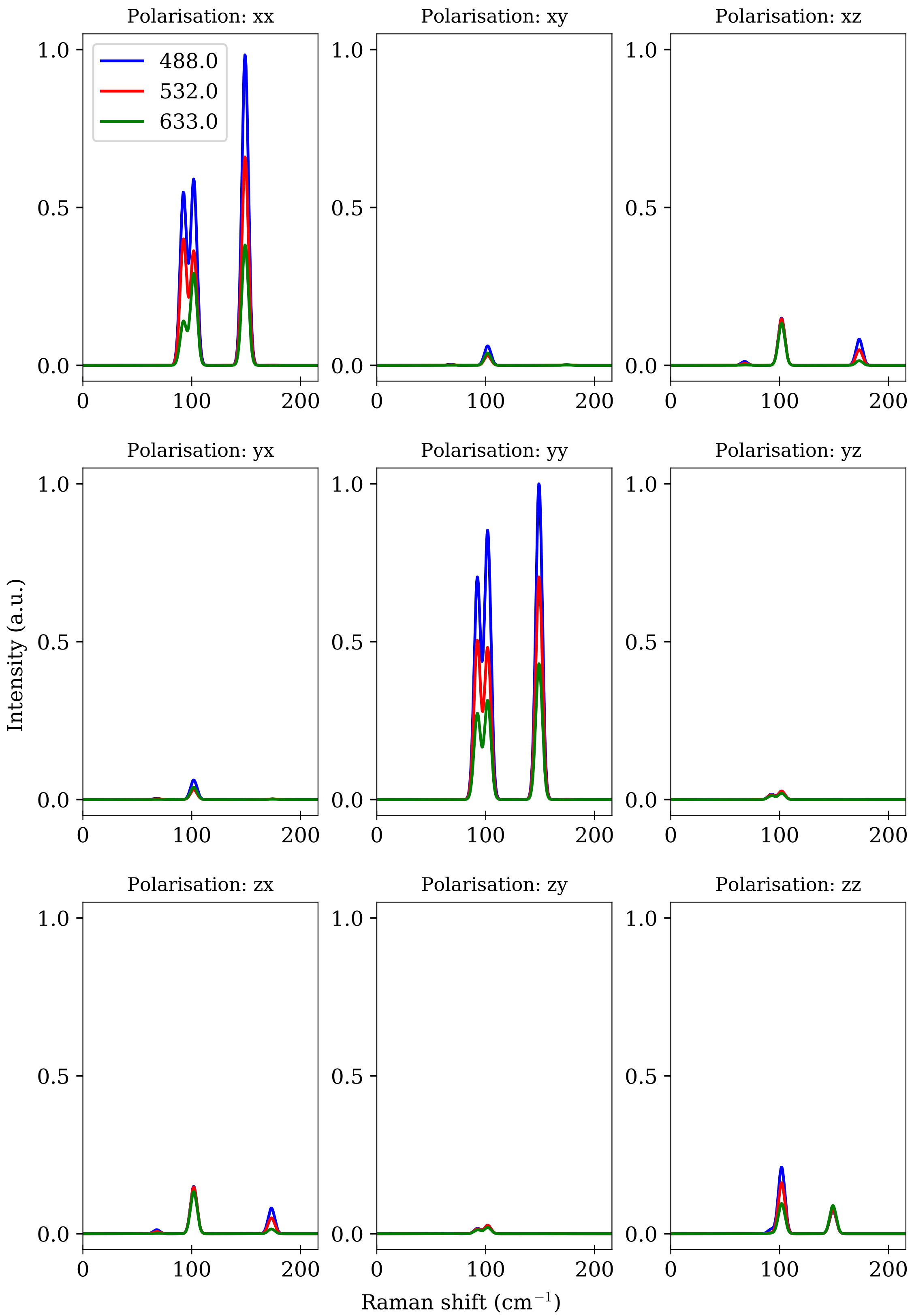

Supplementary Figure 179: Raman spectrum of Cu_2I_2 with space group 156 and Wyckoff positions a-b-0.

Cu_2O_2, 10, f-g-m-0

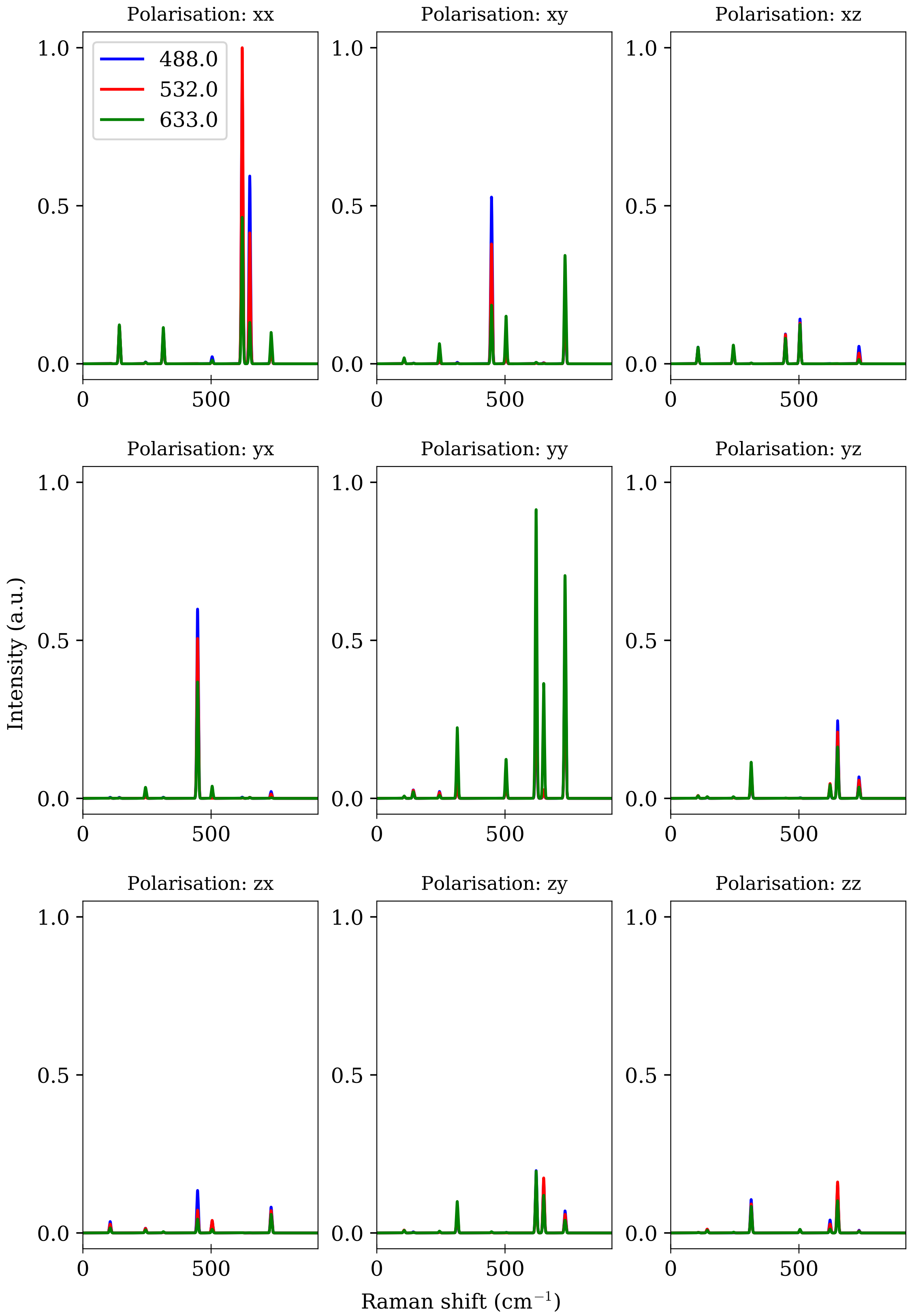

Supplementary Figure 180: Raman spectrum of Cu_2O_2 with space group 10 and Wyckoff positions f-g-m-0.

Cu_2S_2, 2, i-0

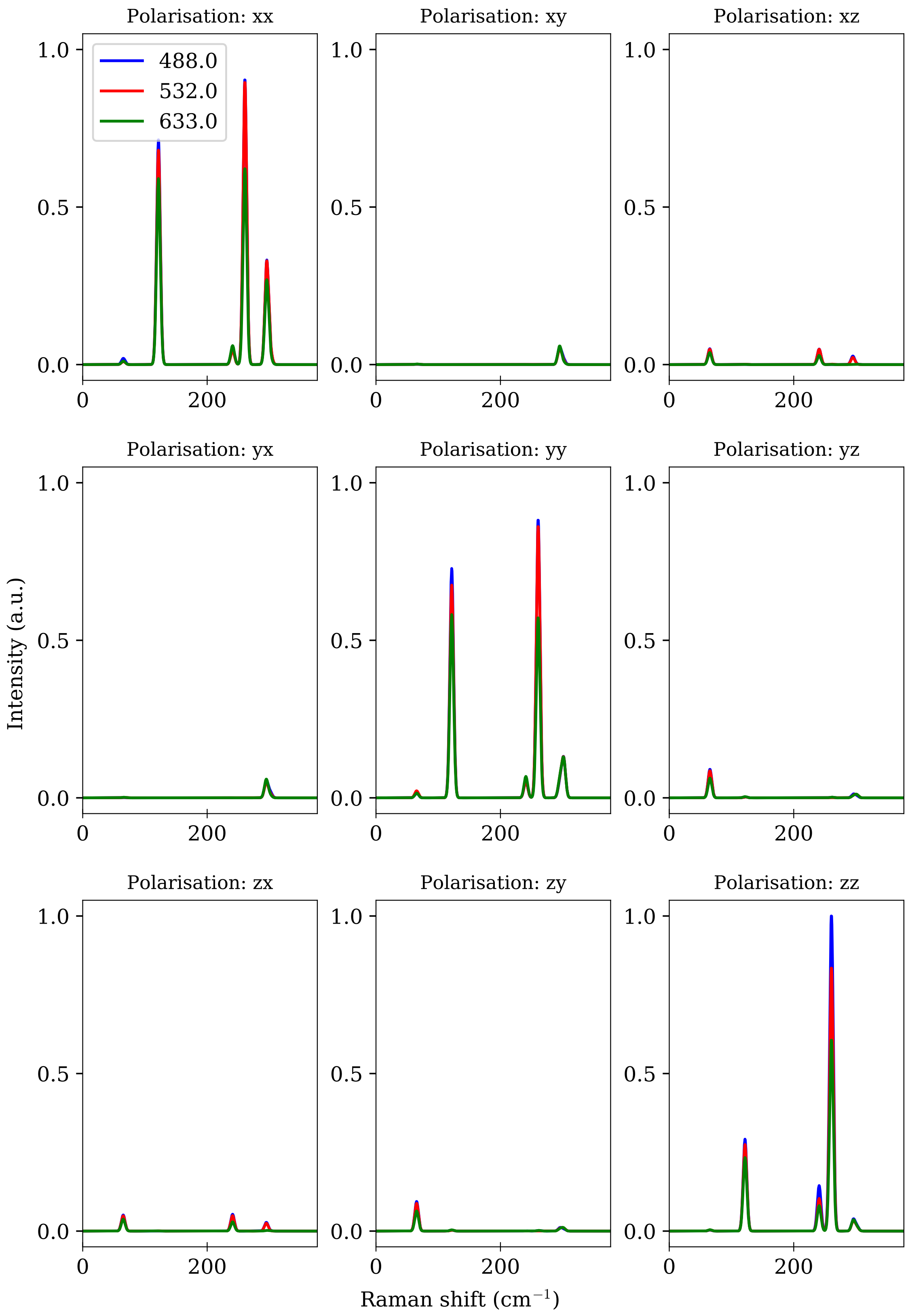

Supplementary Figure 181: Raman spectrum of Cu_2S_2 with space group 2 and Wyckoff positions i-0.

Cu_2S_2, 6, a-b-0

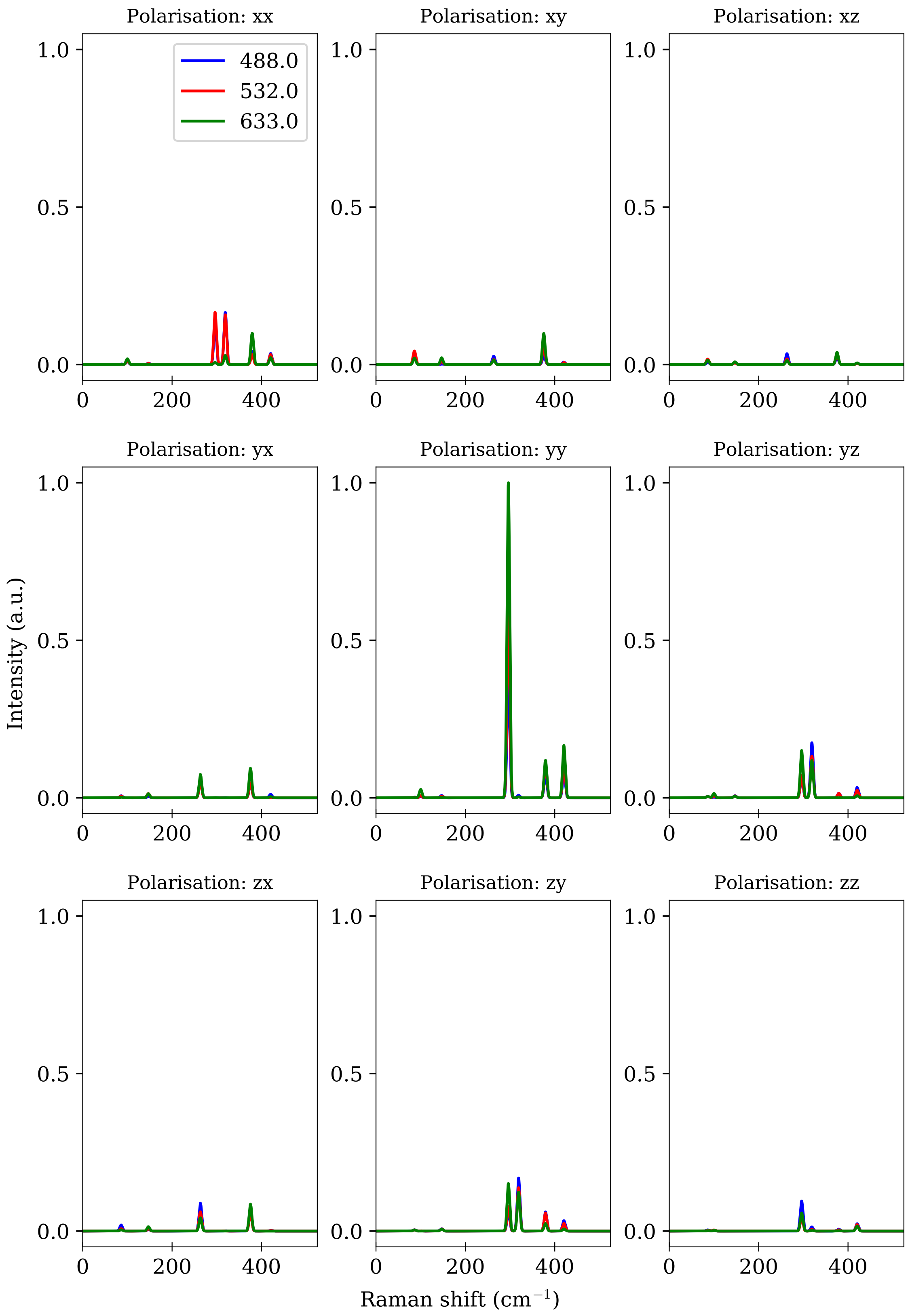

Supplementary Figure 182: Raman spectrum of Cu_2S_2 with space group 6 and Wyckoff positions a-b-0.

Cu_2S_4, 14, d-e-0

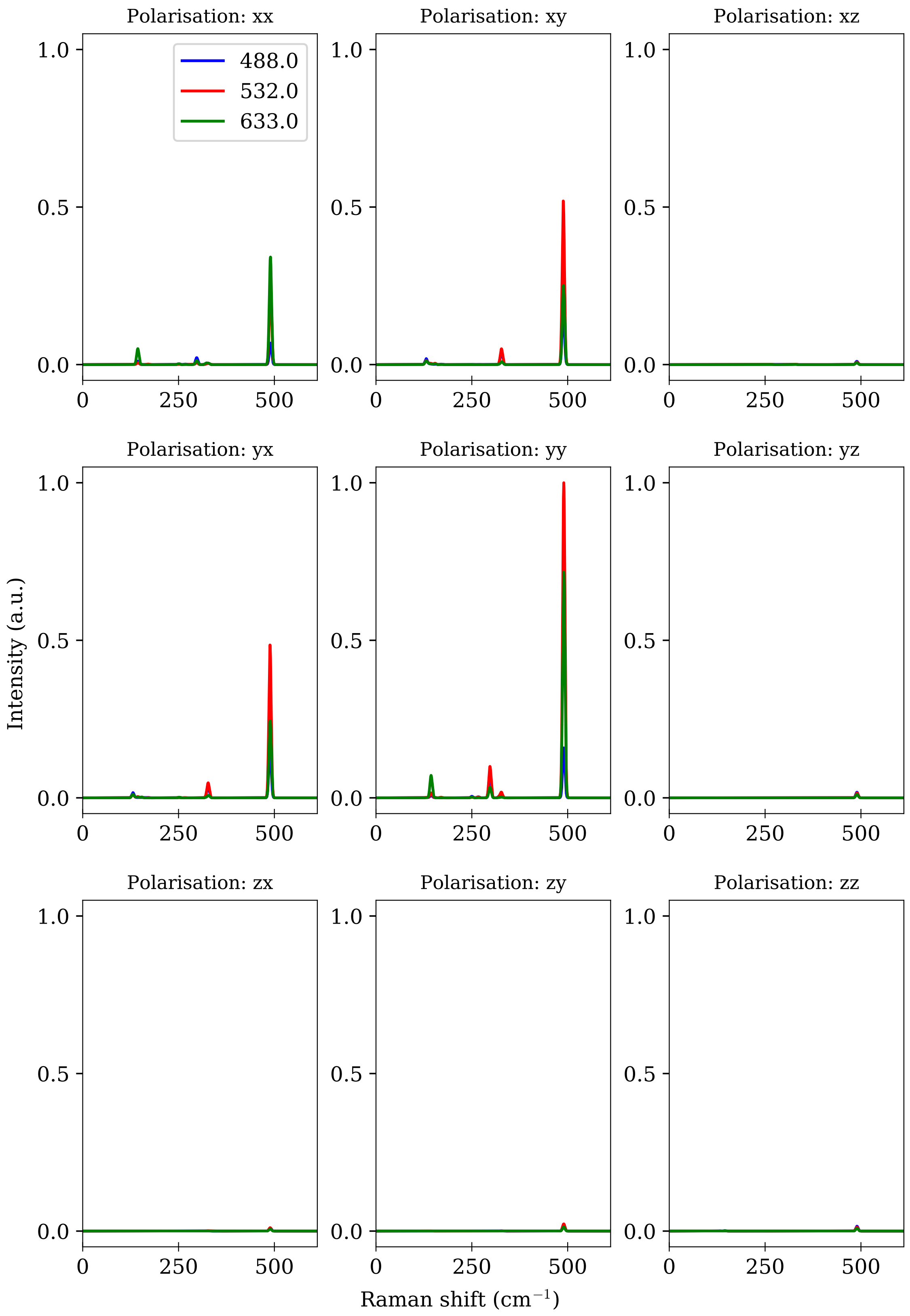

Supplementary Figure 183: Raman spectrum of Cu_2S_4 with space group 14 and Wyckoff positions d-e-0.

Cu_2Se_2, 187, h-i-0

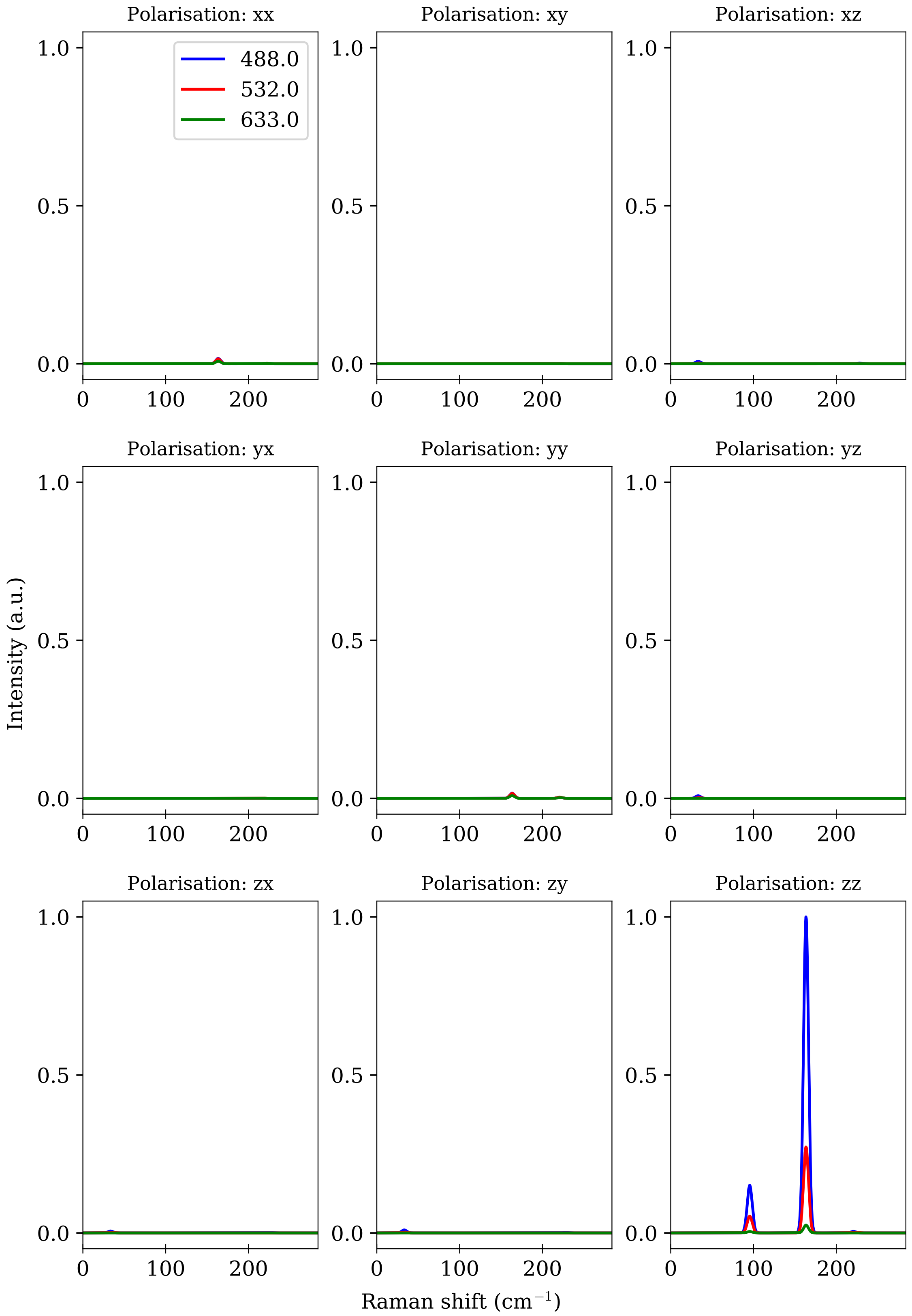

Supplementary Figure 184: Raman spectrum of Cu_2Se_2 with space group 187 and Wyckoff positions h-i-0.

Cu_2Se_2, 8, a-0

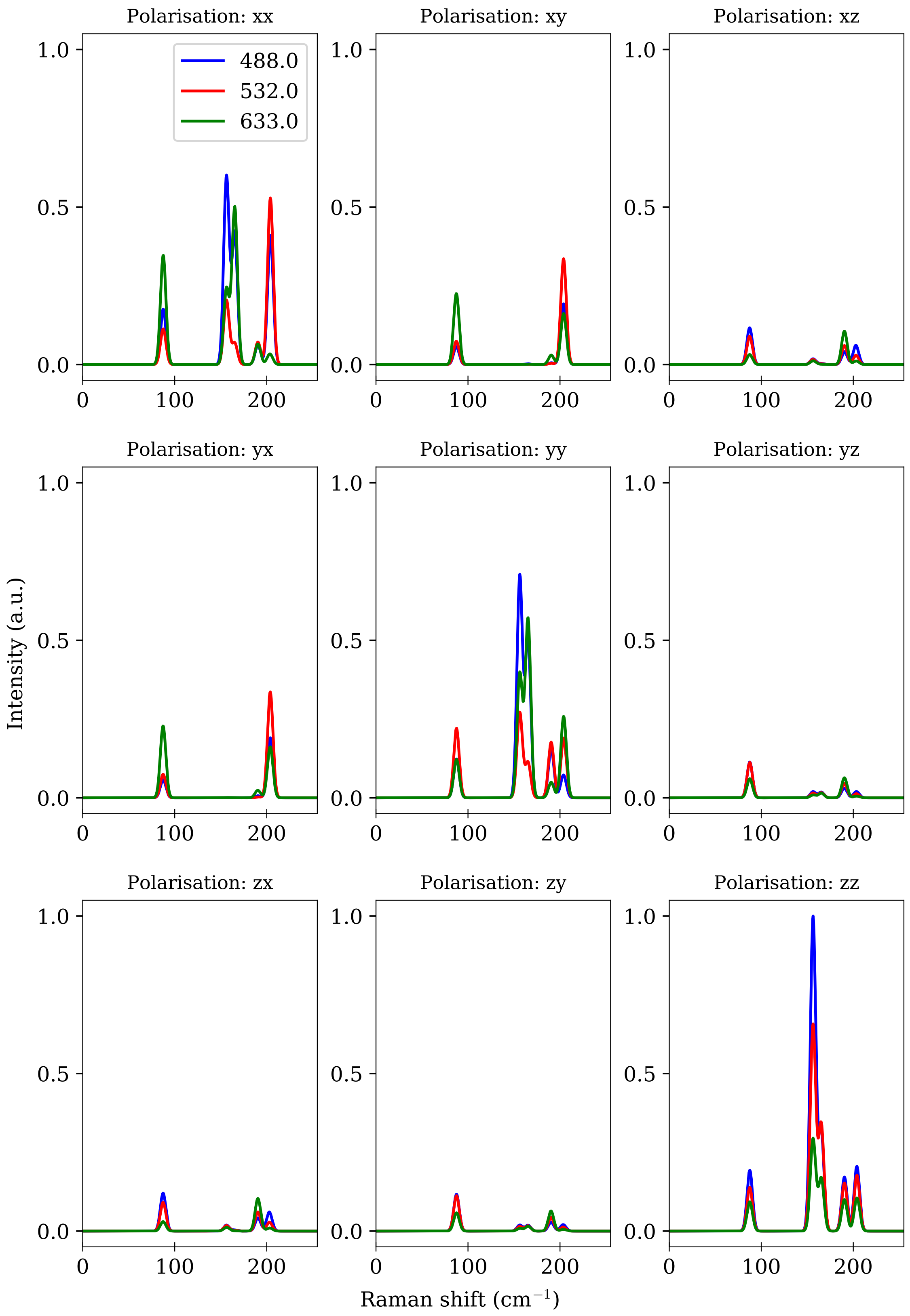

Supplementary Figure 185: Raman spectrum of Cu_2Se_2 with space group 8 and Wyckoff positions a-0.

Cu_2Se_4, 14, d-e-0

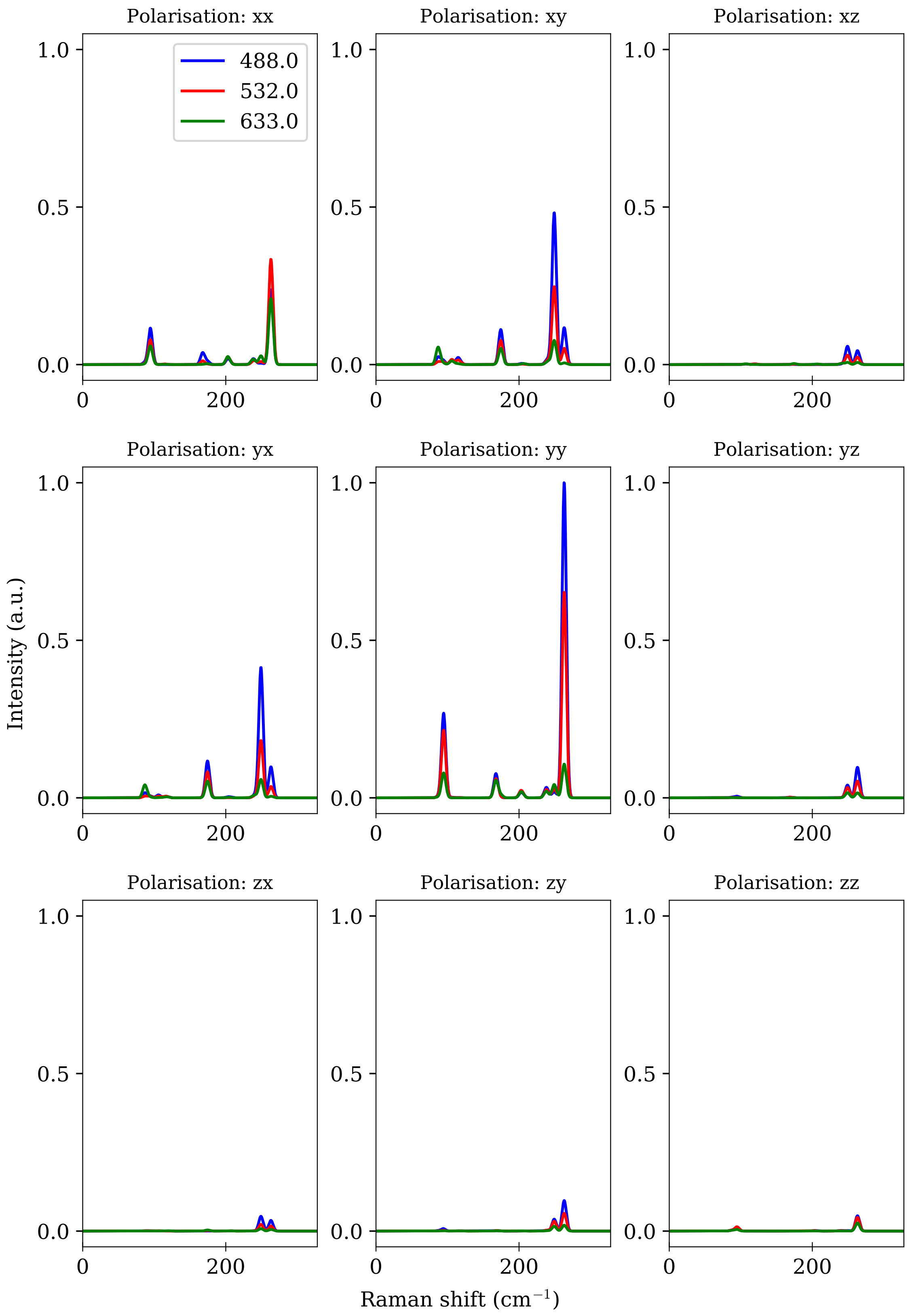

Supplementary Figure 186: Raman spectrum of Cu_2Se_4 with space group 14 and Wyckoff positions d-e-0.

Cu_2Te_2, 164, d-0

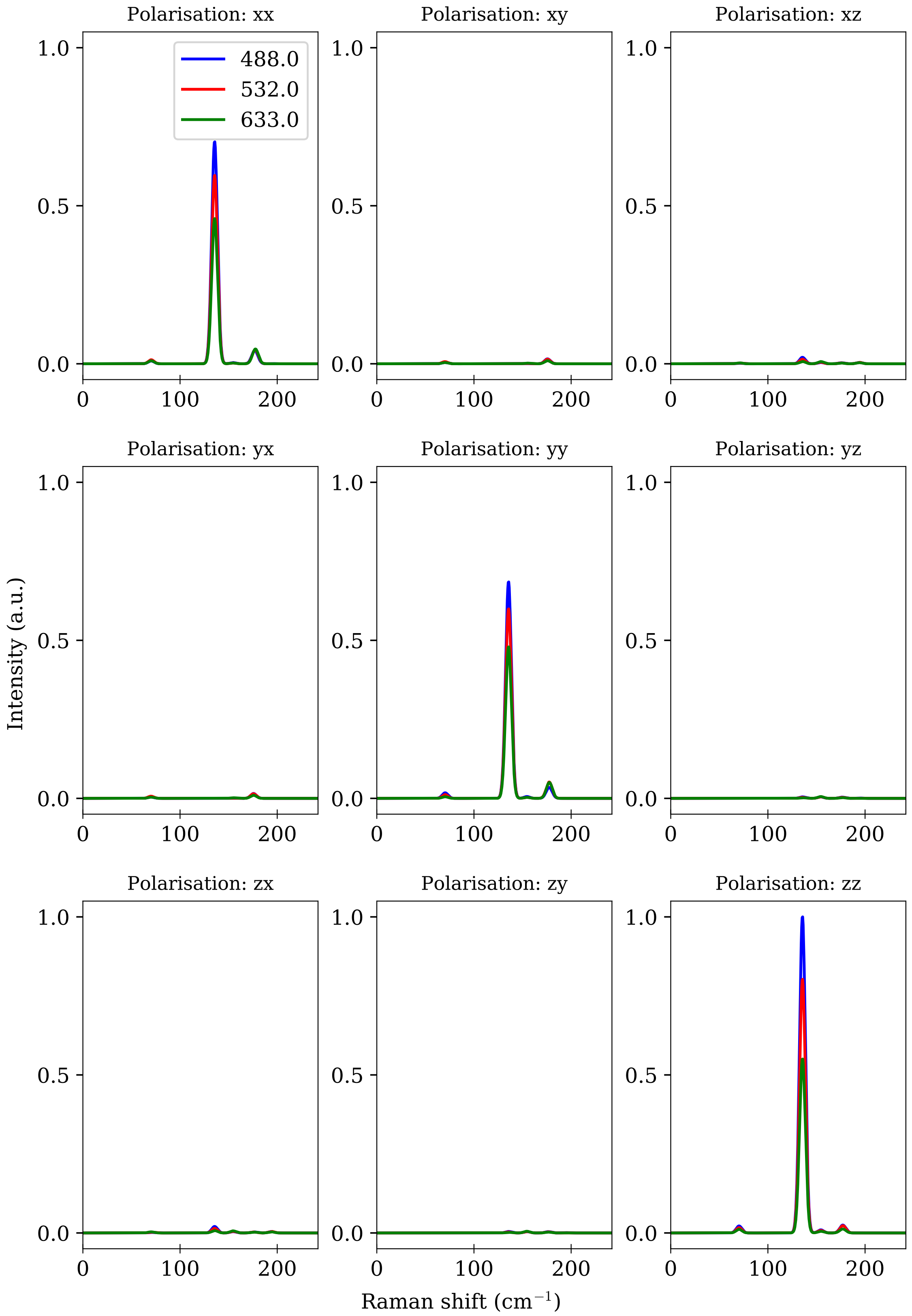

Supplementary Figure 187: Raman spectrum of Cu_2Te_2 with space group 164 and Wyckoff positions d-0.

Cu_2Te_2, 6, a-b-0

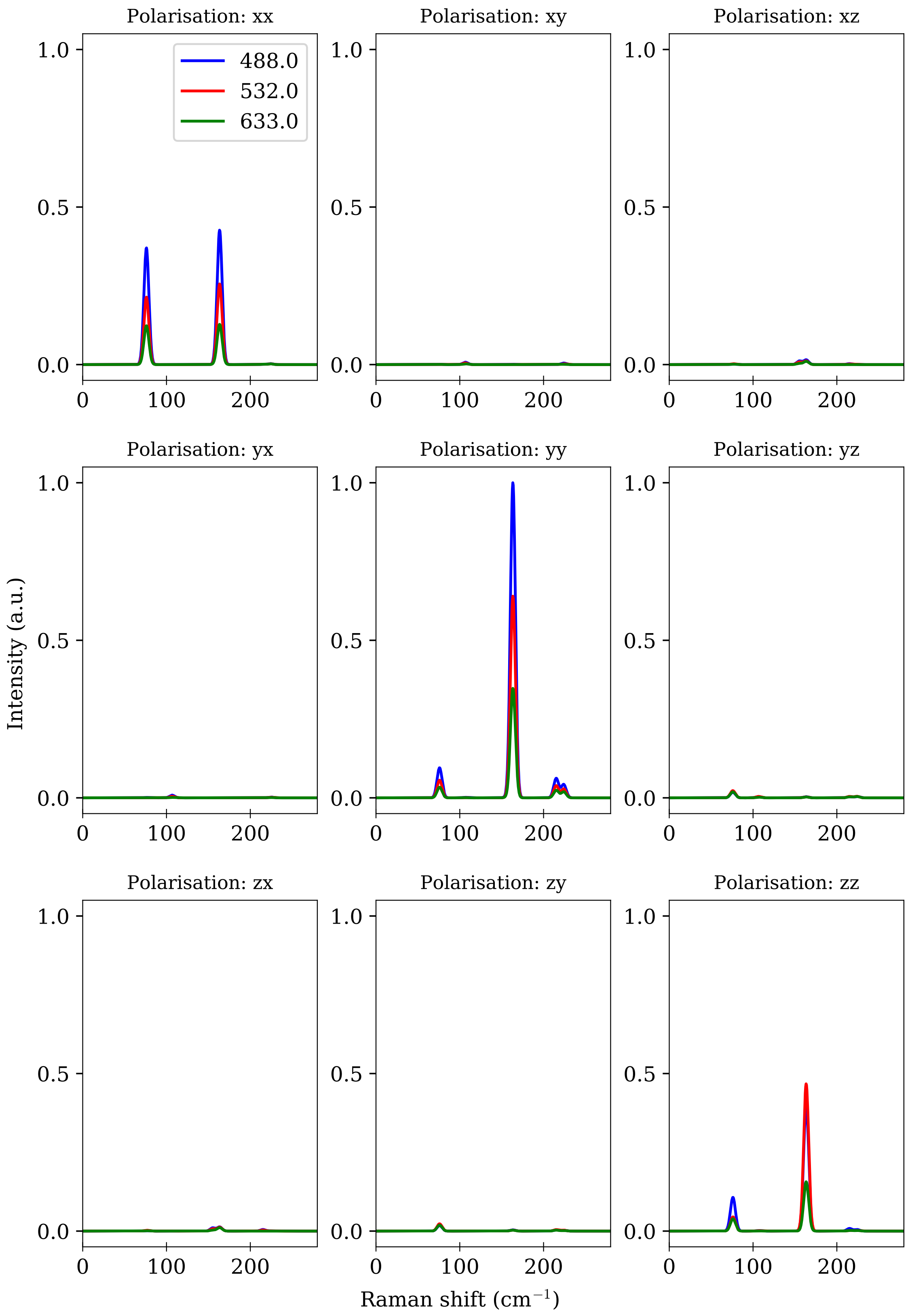

Supplementary Figure 188: Raman spectrum of Cu_2Te_2 with space group 6 and Wyckoff positions a-b-0.

$CuBr_2$, 115, a-g-0

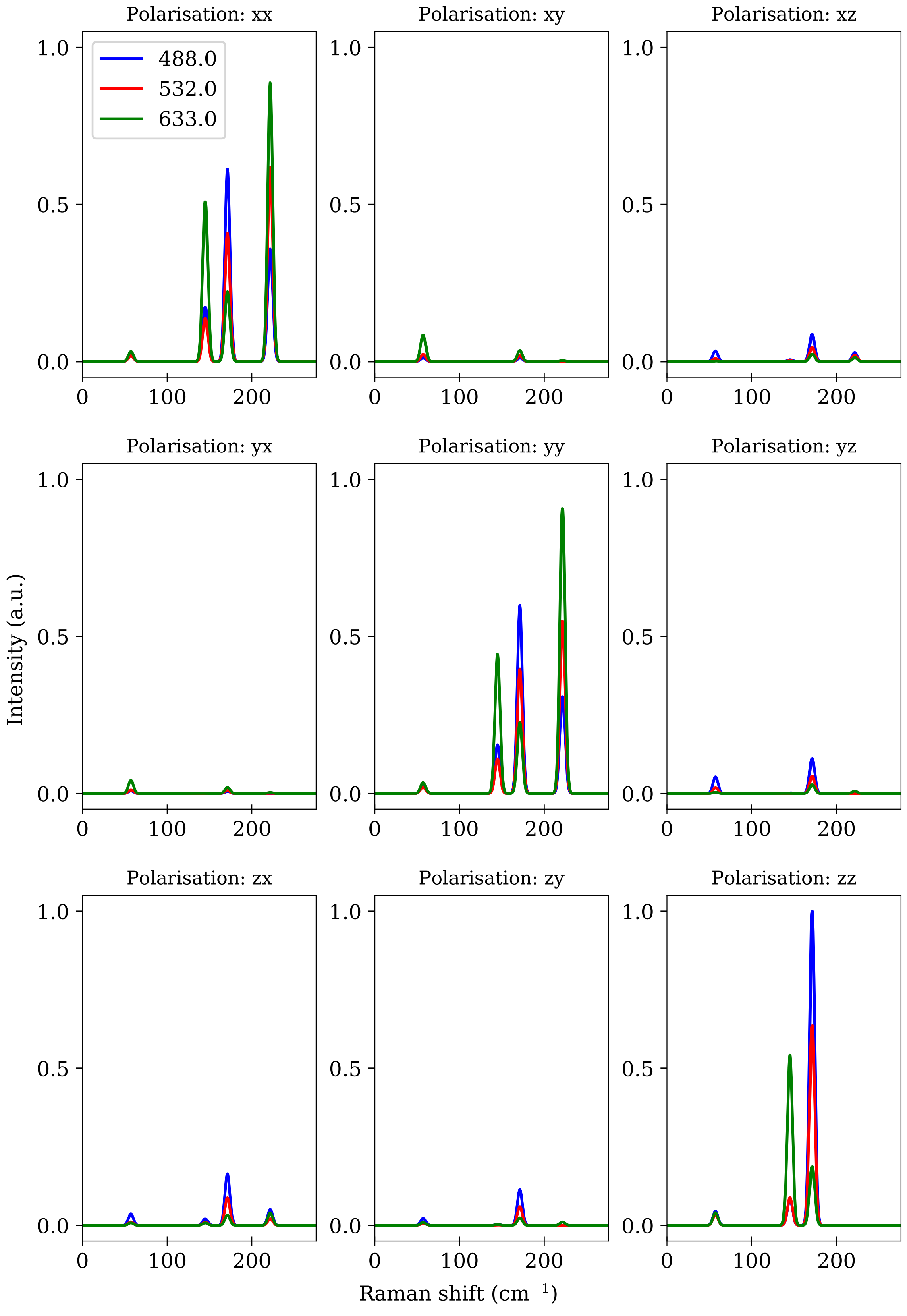

Supplementary Figure 189: Raman spectrum of $CuBr_2$ with space group 115 and Wyckoff positions a-g-0.

CuH_2, 187, b-i-0

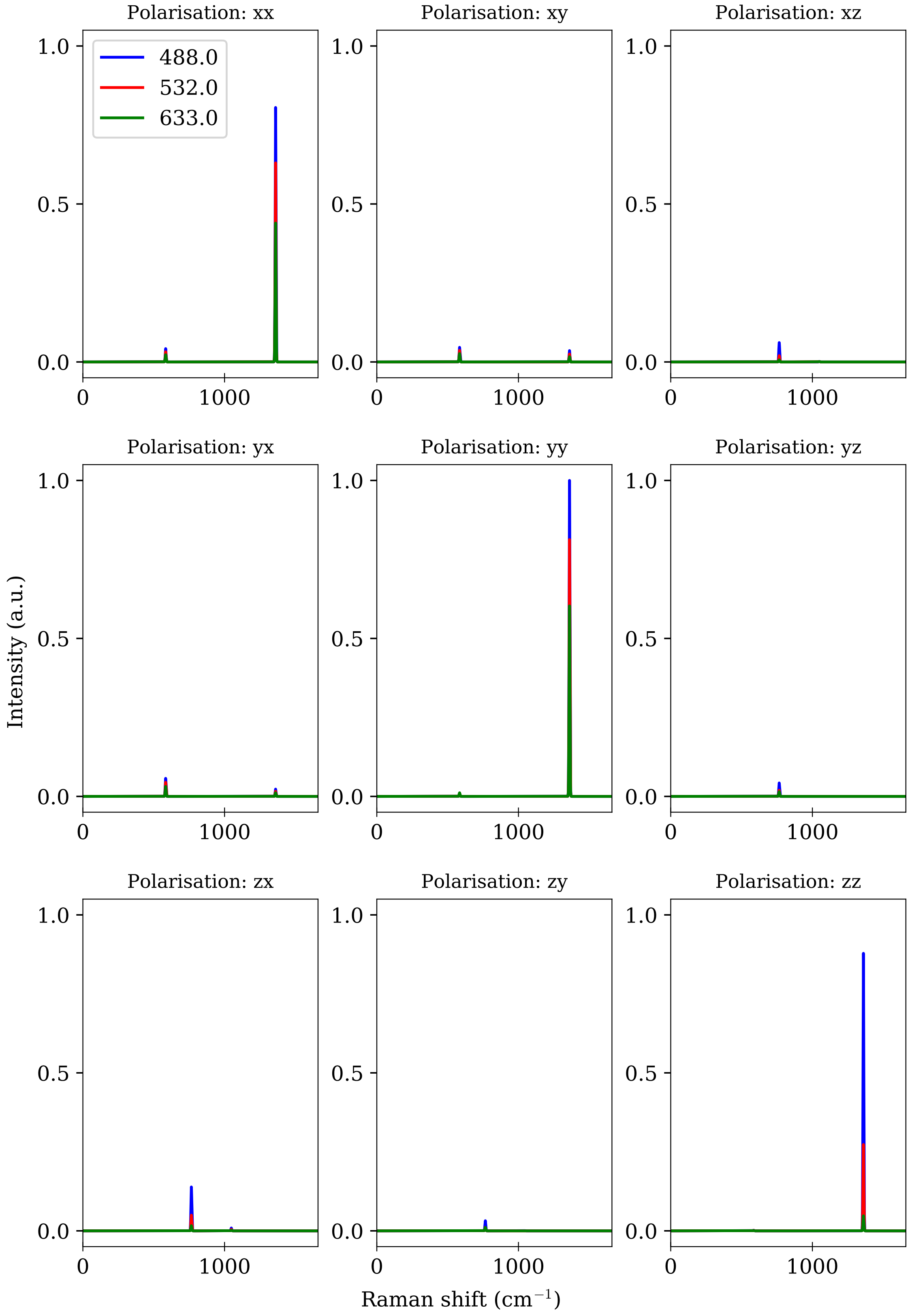

Supplementary Figure 190: Raman spectrum of CuH_2 with space group 187 and Wyckoff positions b-i-0.

CuI_2, 164, b-d-0

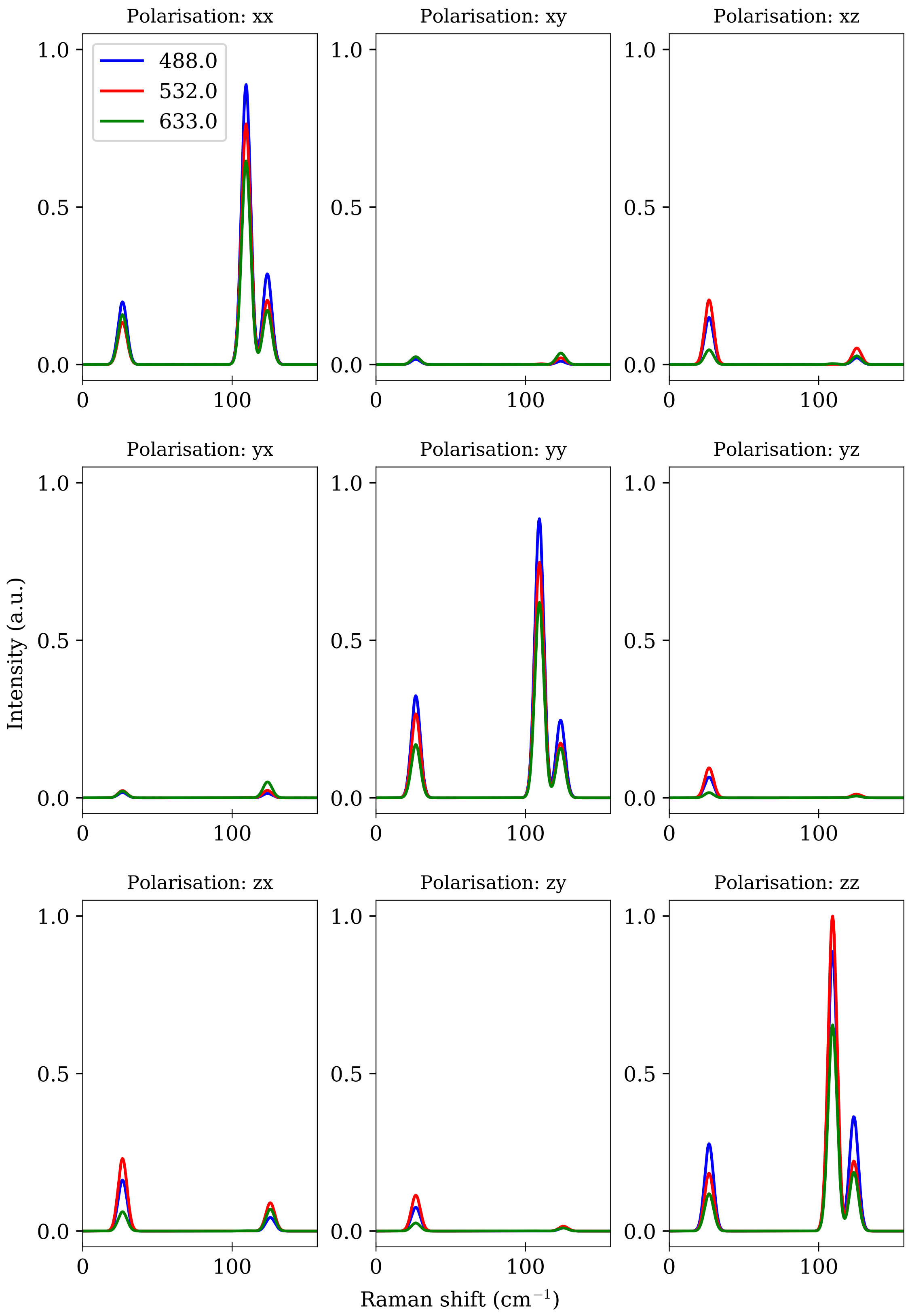

Supplementary Figure 191: Raman spectrum of CuI_2 with space group 164 and Wyckoff positions b-d-0.

$CuLi_2$, 187, a-i-0

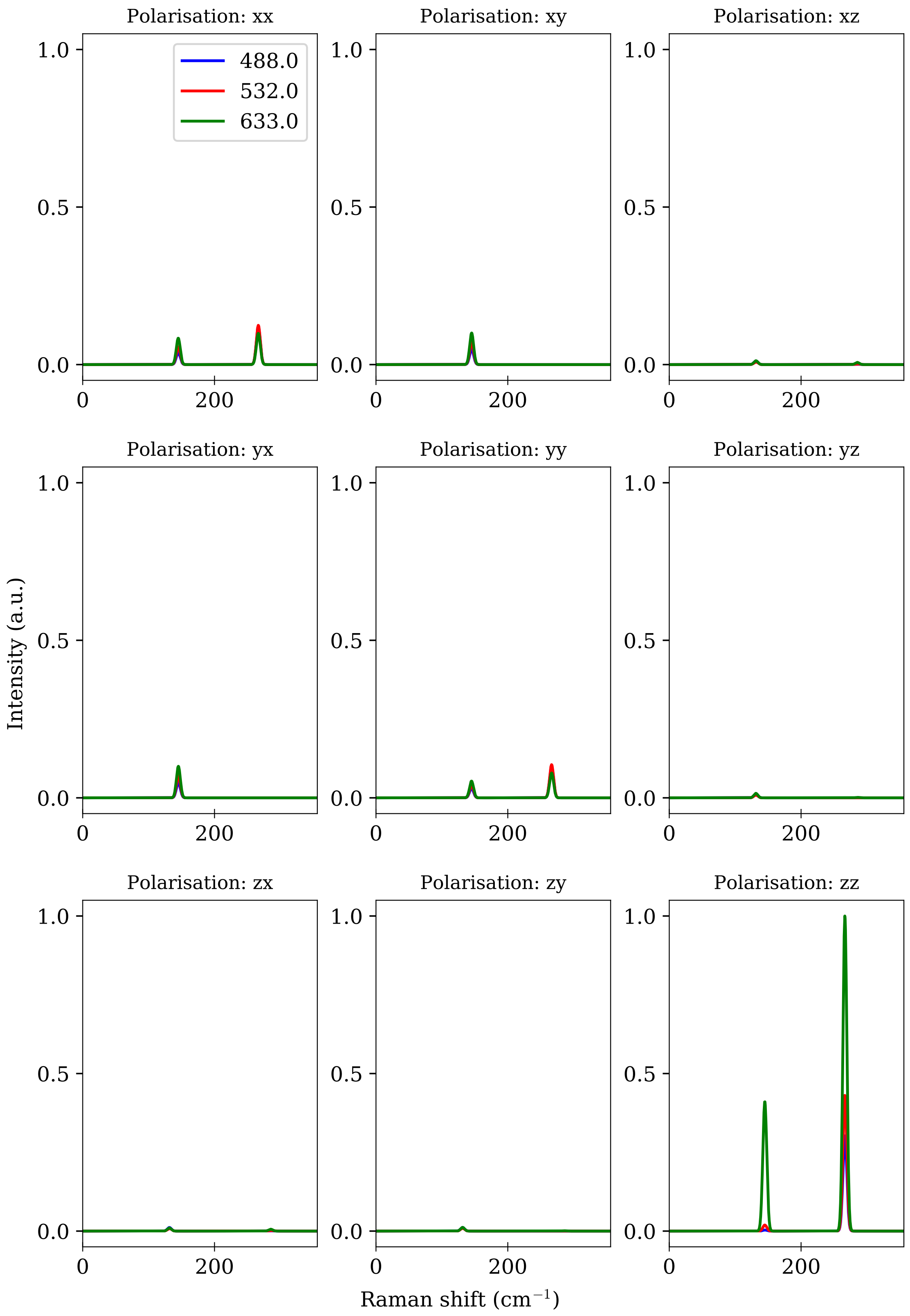

Supplementary Figure 192: Raman spectrum of $CuLi_2$ with space group 187 and Wyckoff positions a-i-0.

CuS_2, 164, b-d-0

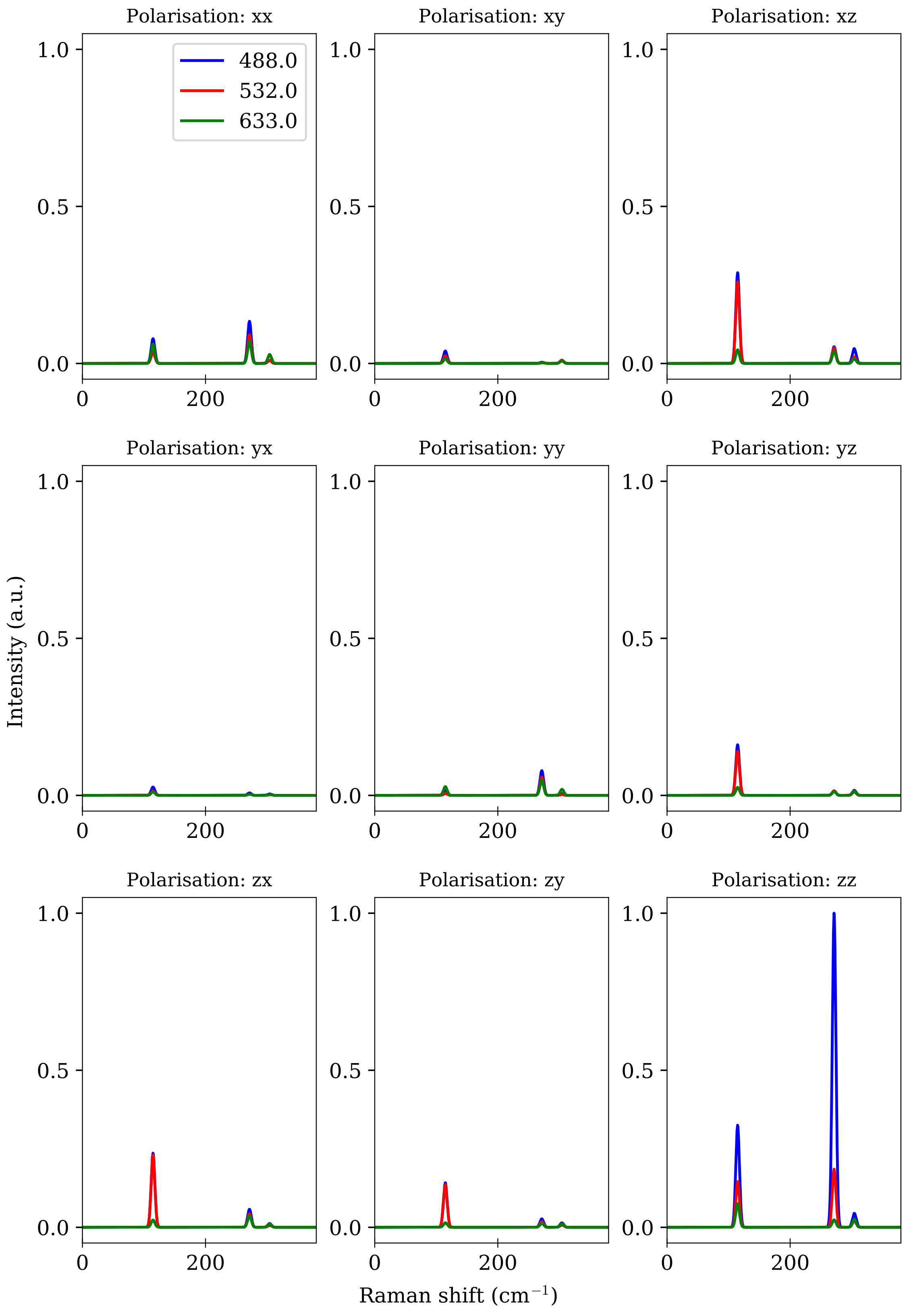

Supplementary Figure 193: Raman spectrum of CuS_2 with space group 164 and Wyckoff positions b-d-0.

$CuSe_2$, 115, d-g-0

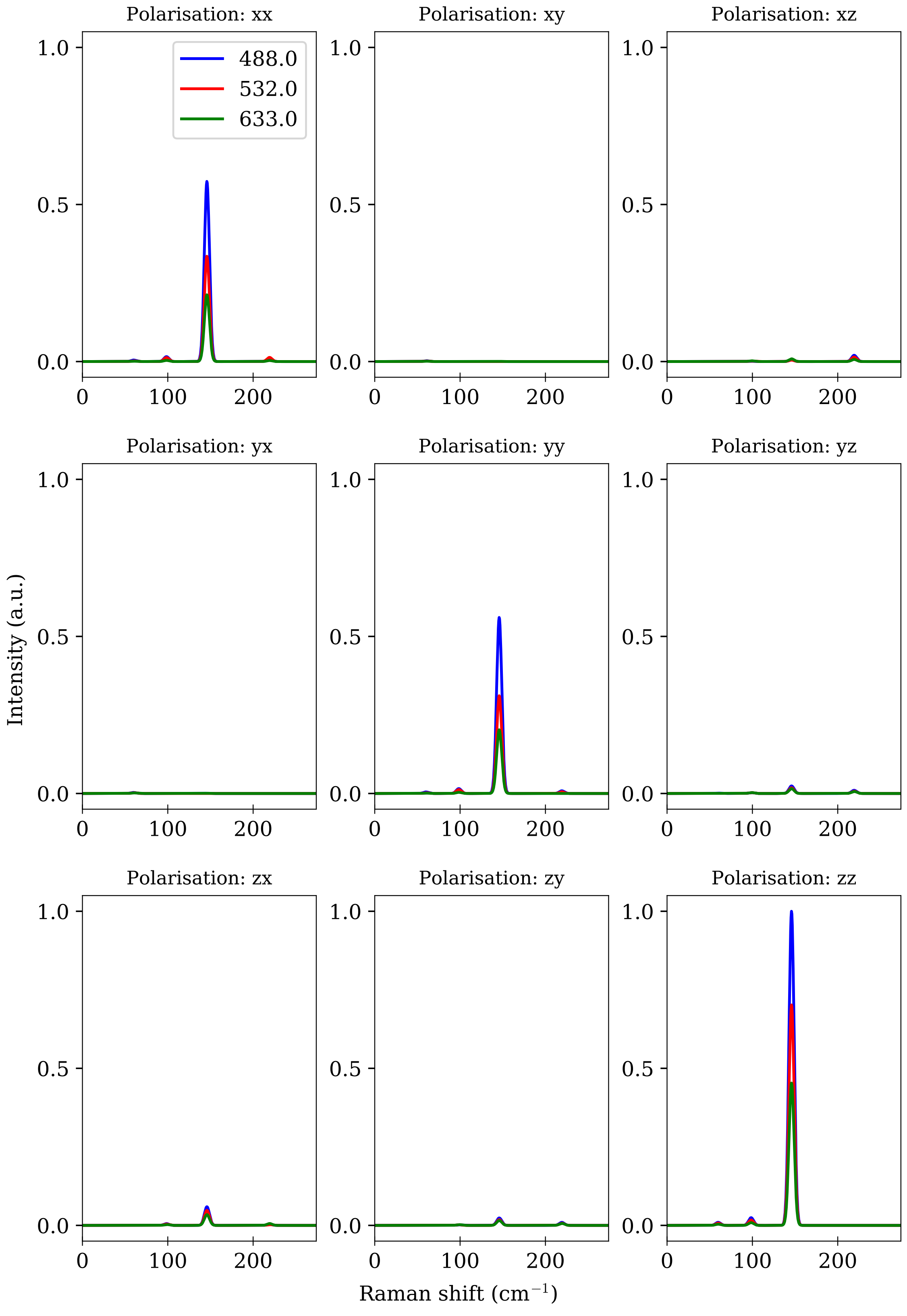

Supplementary Figure 194: Raman spectrum of $CuSe_2$ with space group 115 and Wyckoff positions d-g-0.

$CuSe_2$, 164, b-d-0

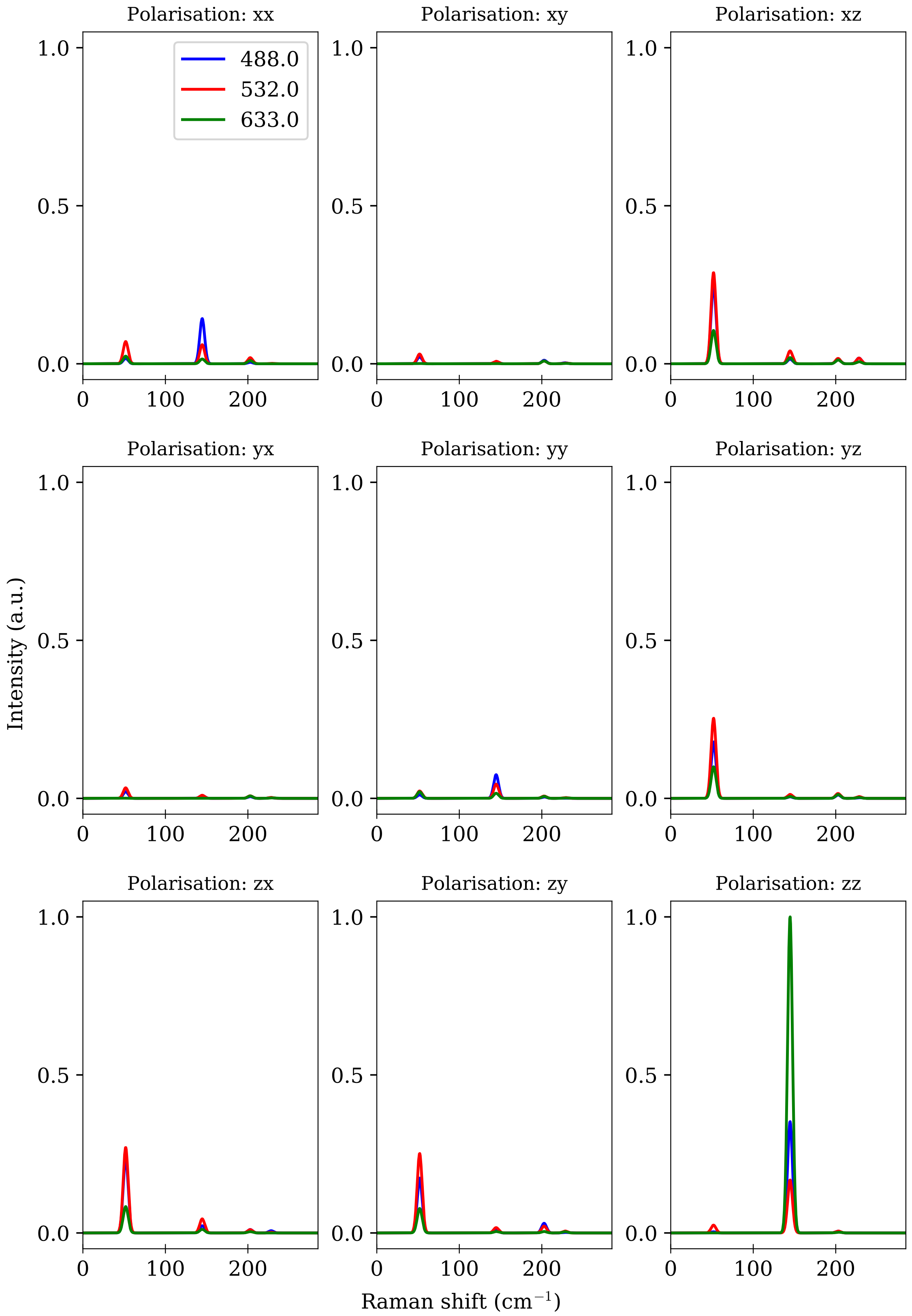

Supplementary Figure 195: Raman spectrum of $CuSe_2$ with space group 164 and Wyckoff positions b-d-0.

$CuTe_2$, 187, b-i-0

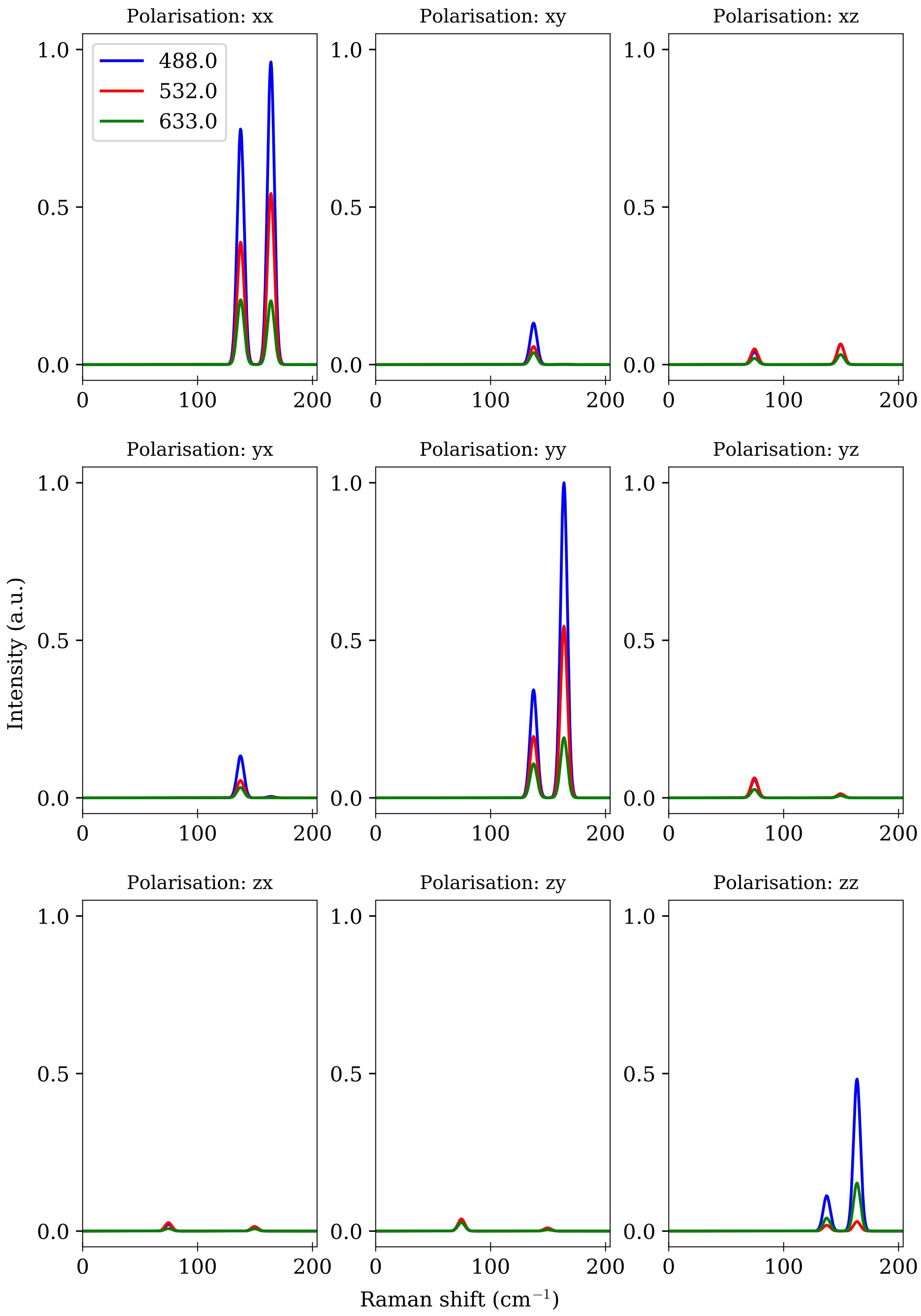

Supplementary Figure 196: Raman spectrum of $CuTe_2$ with space group 187 and Wyckoff positions b-i-0.

Fe_2O_2, 187, h-i-0

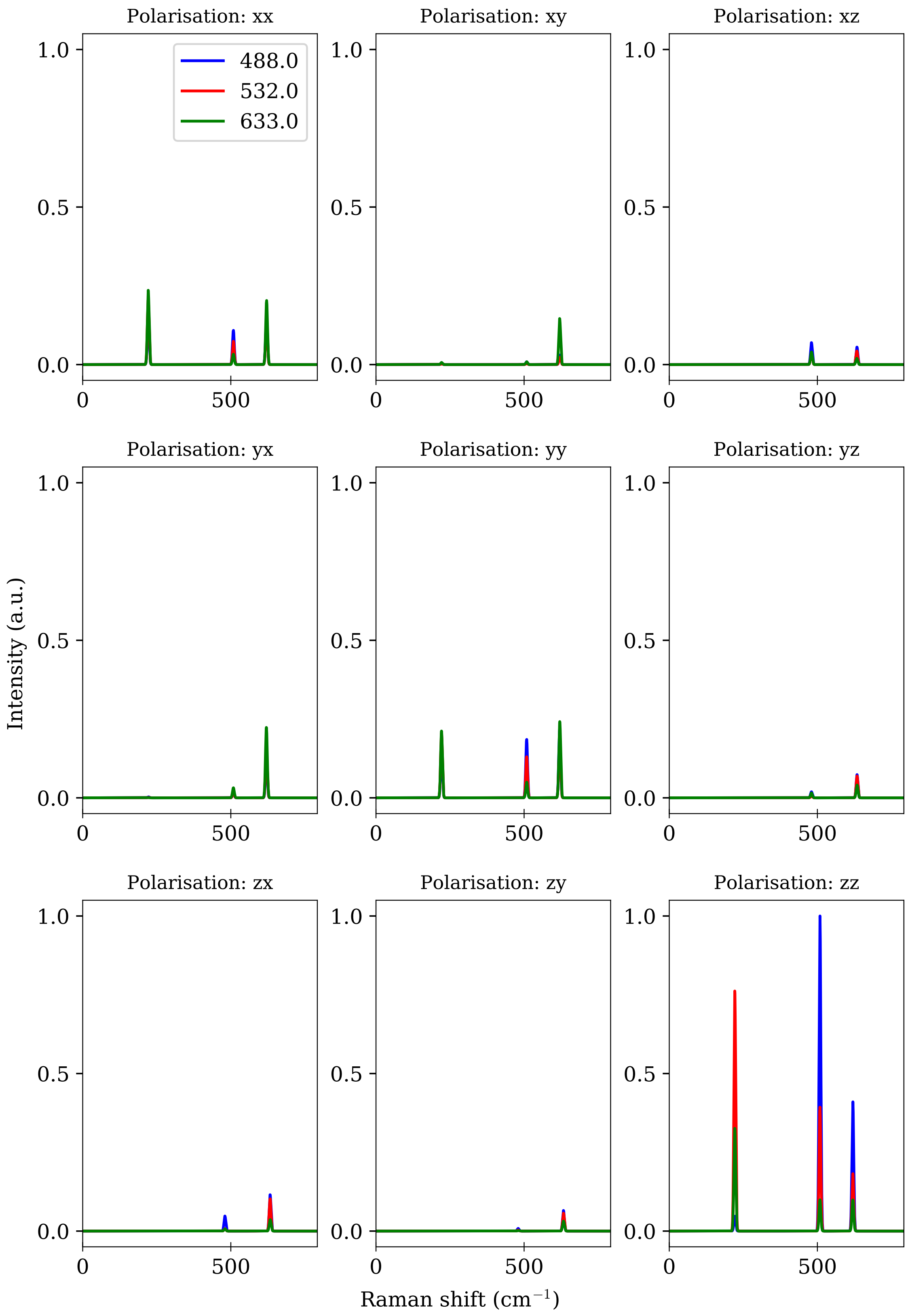

Supplementary Figure 197: Raman spectrum of Fe_2O_2 with space group 187 and Wyckoff positions h-i-0.

Fe_2S_2, 129, b-c-0

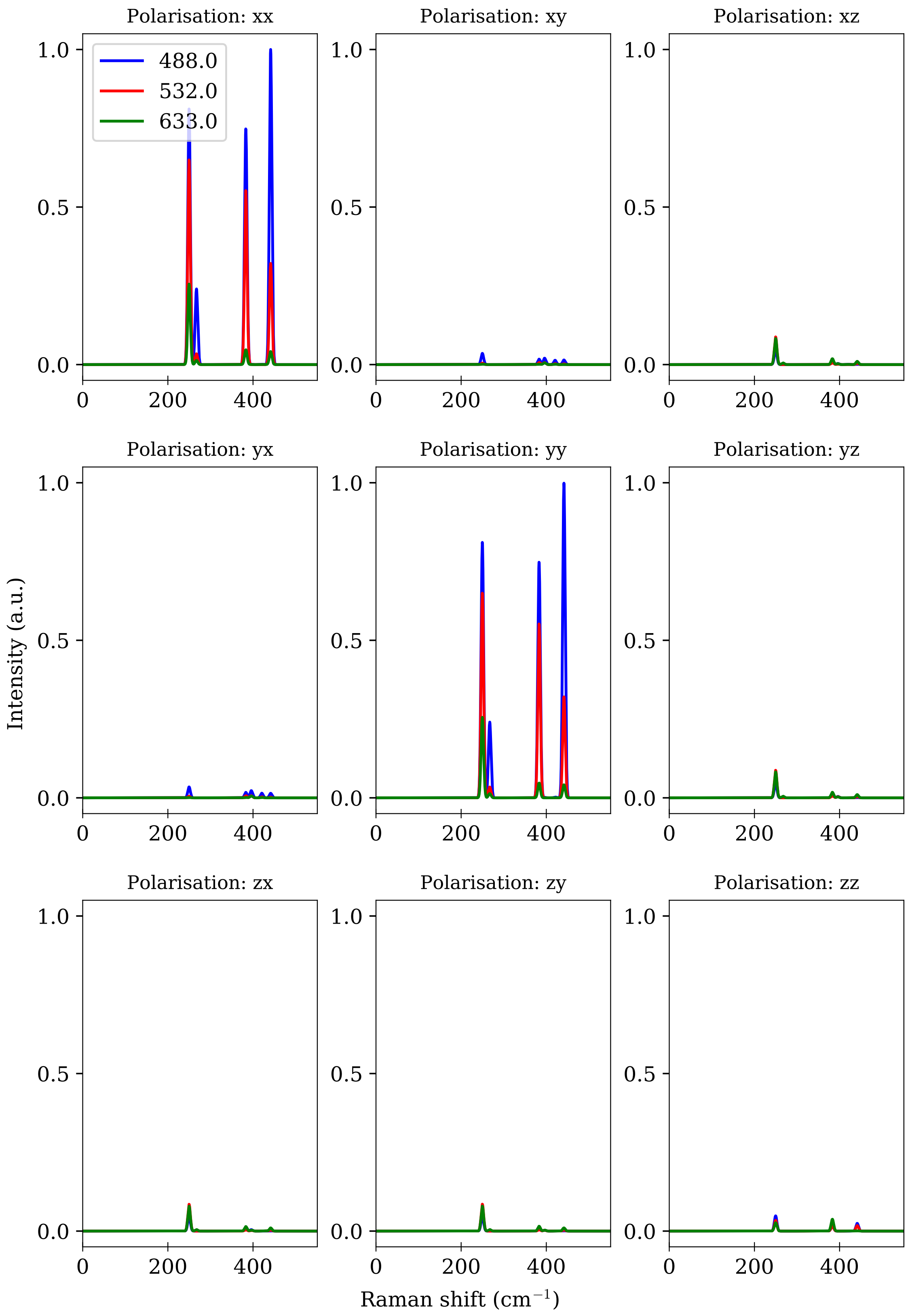

Supplementary Figure 198: Raman spectrum of Fe_2S_2 with space group 129 and Wyckoff positions b-c-0.

Fe_2S_4, 11, e-0

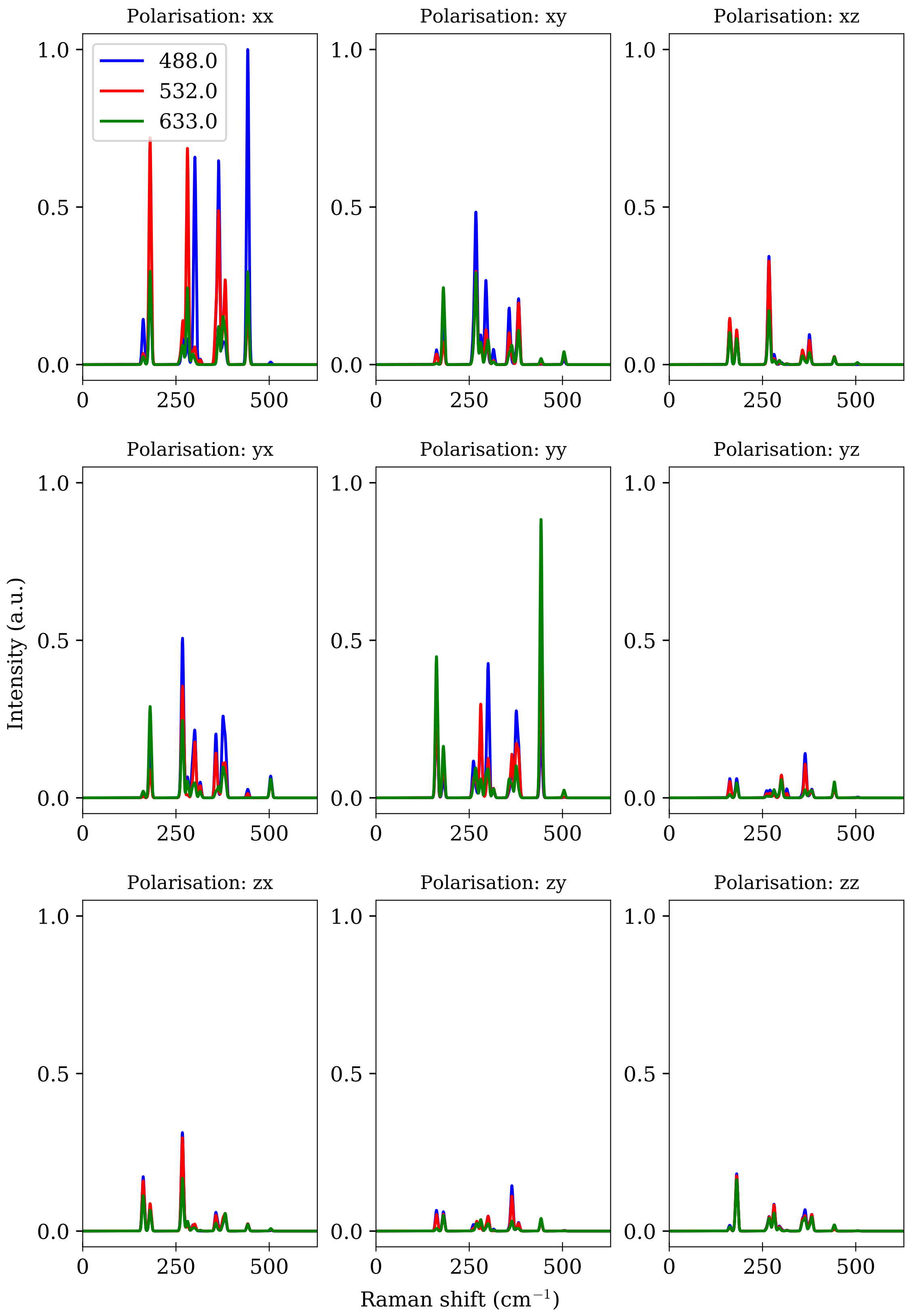

Supplementary Figure 199: Raman spectrum of Fe_2S_4 with space group 11 and Wyckoff positions e-0.

Fe_2Se_4, 11, e-0

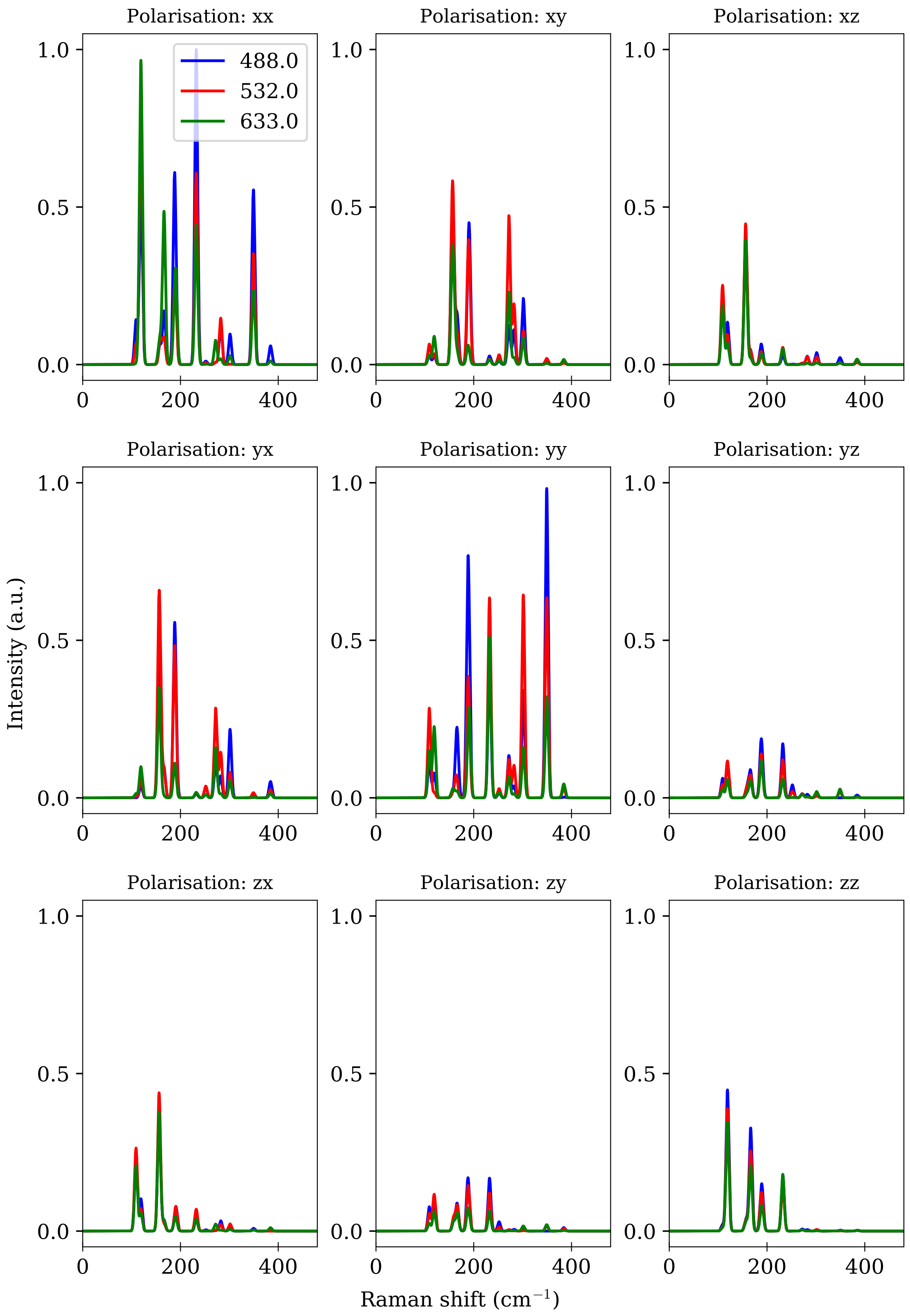

Supplementary Figure 200: Raman spectrum of Fe_2Se_4 with space group 11 and Wyckoff positions e-0.

Fe_2Te_4, 11, e-0

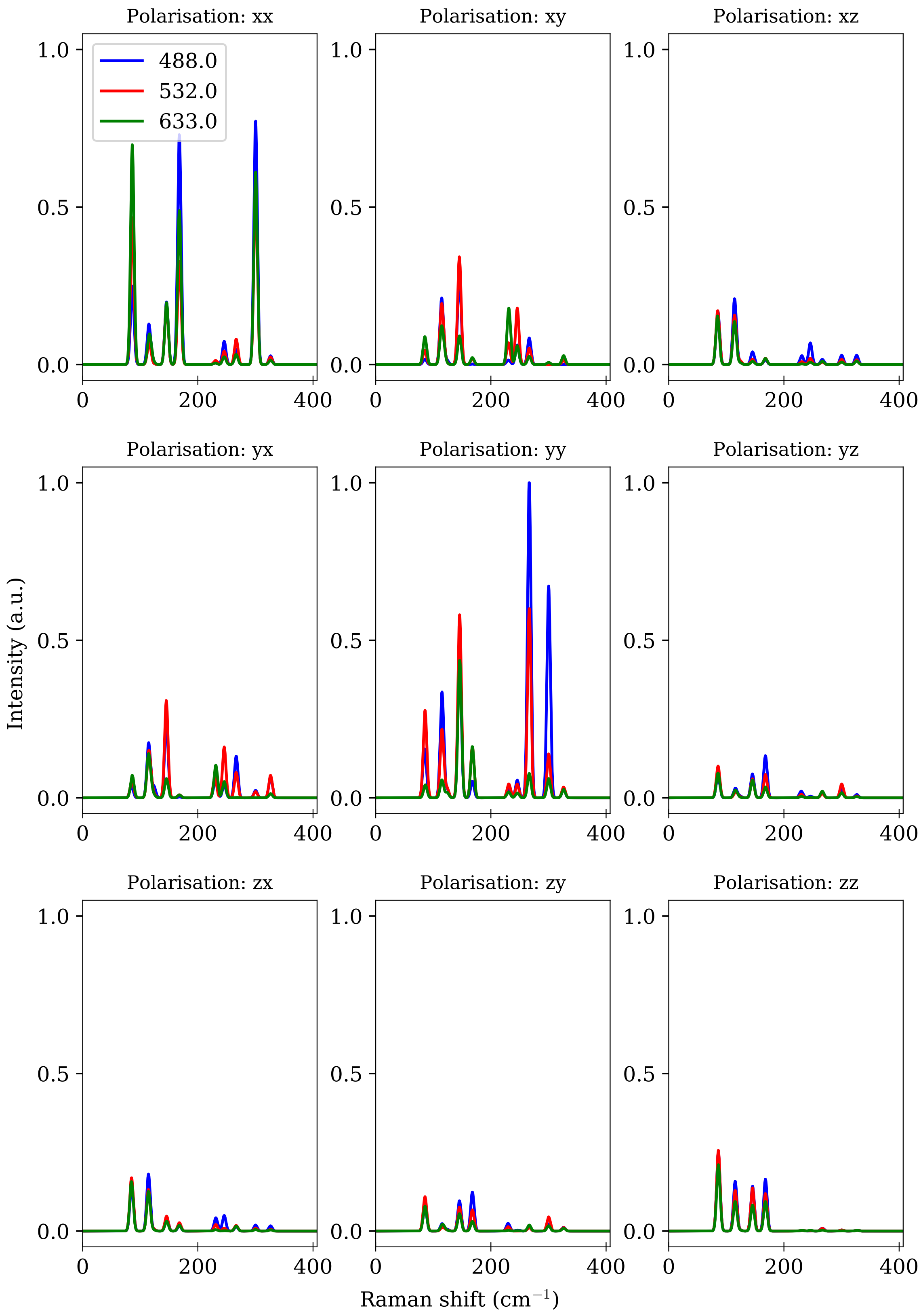

Supplementary Figure 201: Raman spectrum of Fe_2Te_4 with space group 11 and Wyckoff positions e-0.

$FeBr_2$, 164, b-d-0

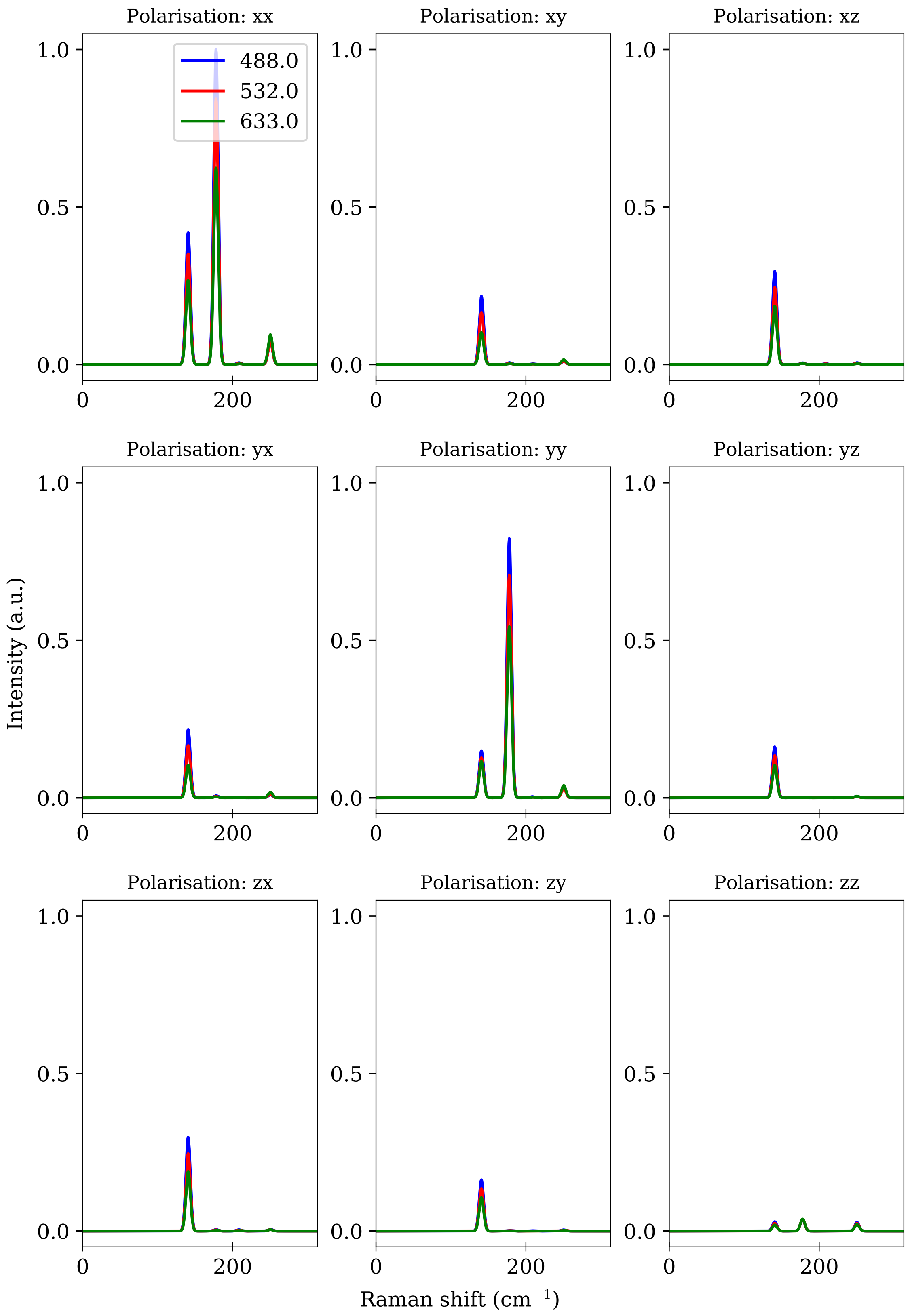

Supplementary Figure 202: Raman spectrum of $FeBr_2$ with space group 164 and Wyckoff positions b-d-0.

$FeCl_2$, 164, b-d-0

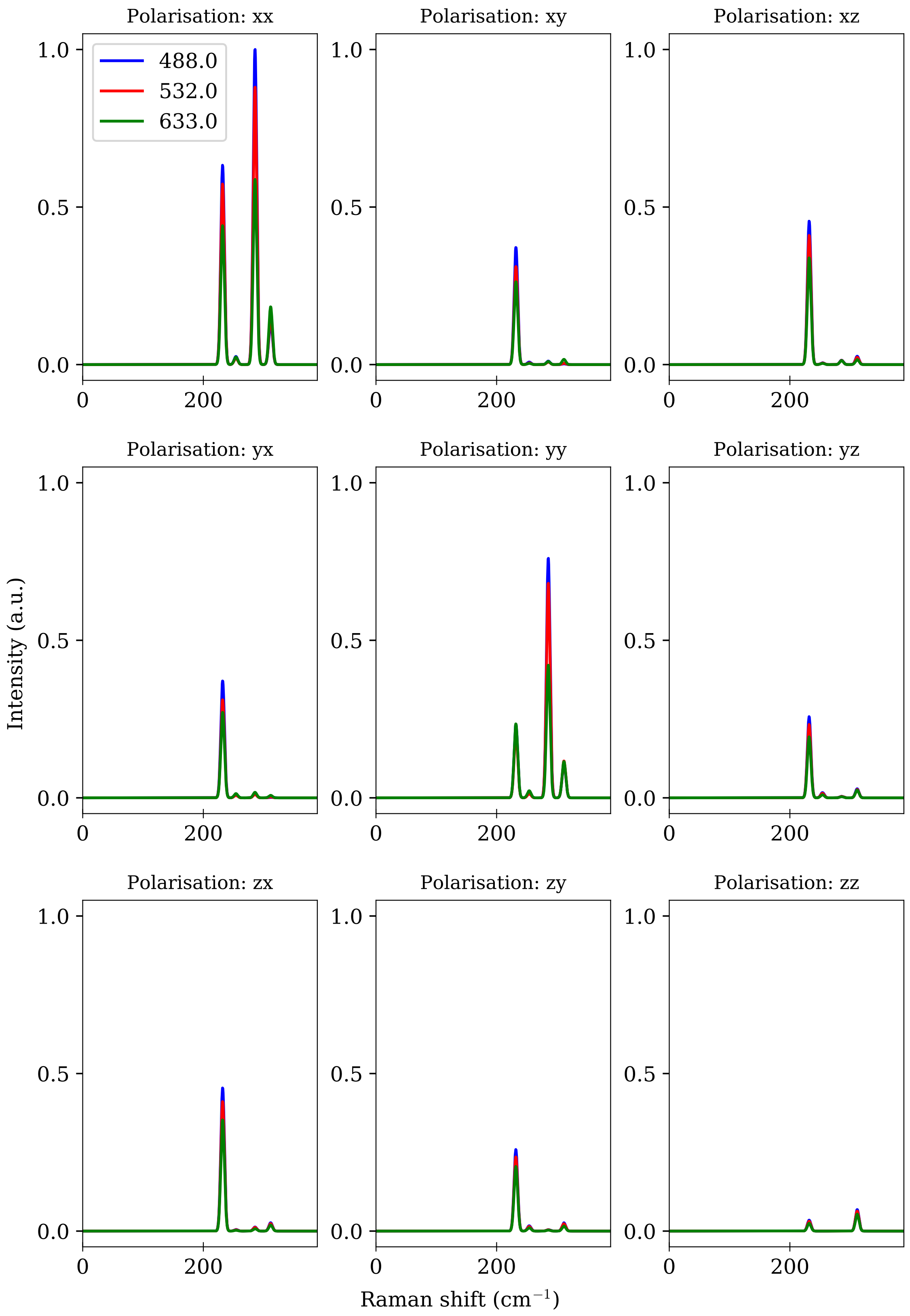

Supplementary Figure 203: Raman spectrum of $FeCl_2$ with space group 164 and Wyckoff positions b-d-0.

FeI_2, 164, b-d-0

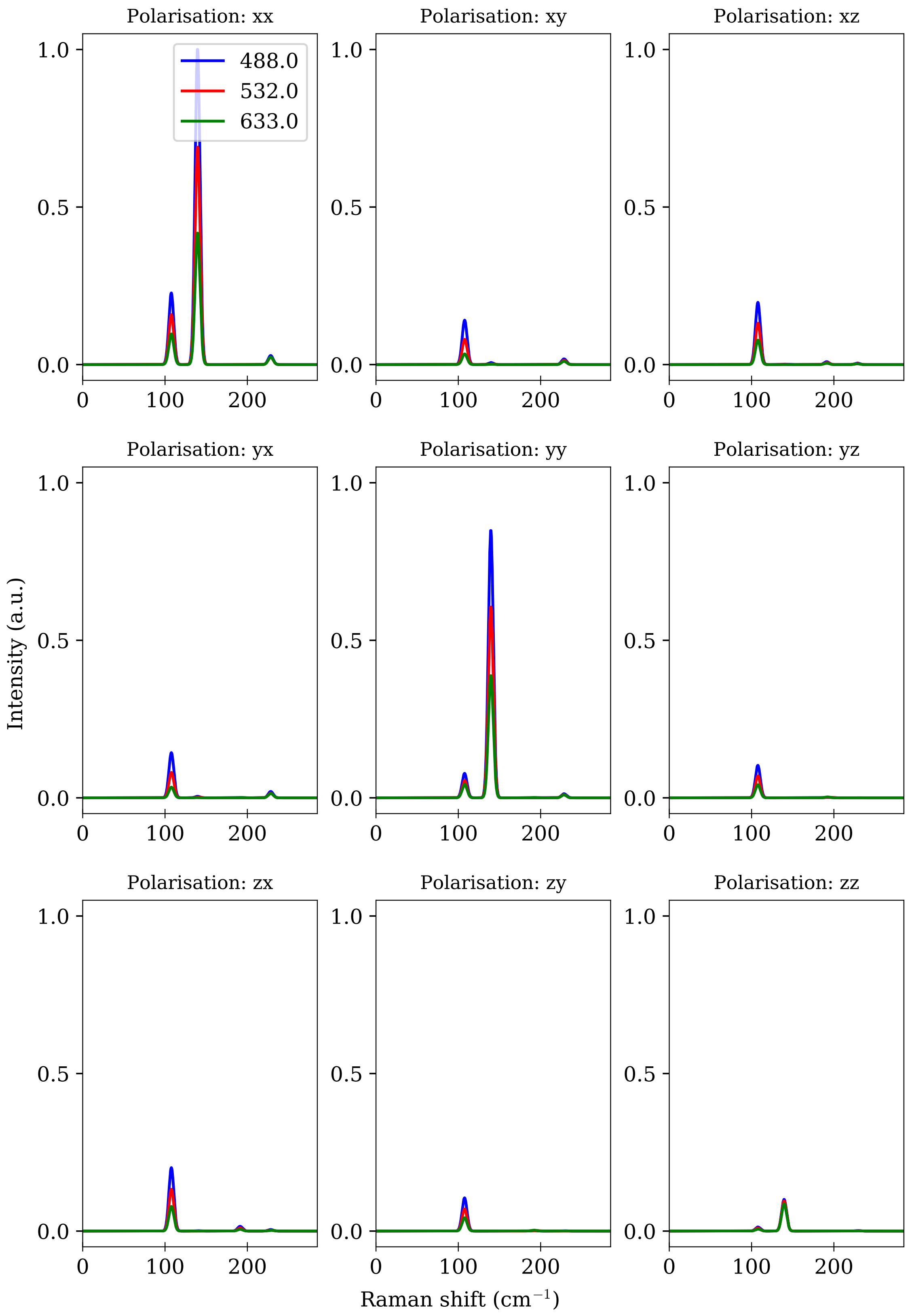

Supplementary Figure 204: Raman spectrum of FeI_2 with space group 164 and Wyckoff positions b-d-0.

FeO_2, 115, a-g-0

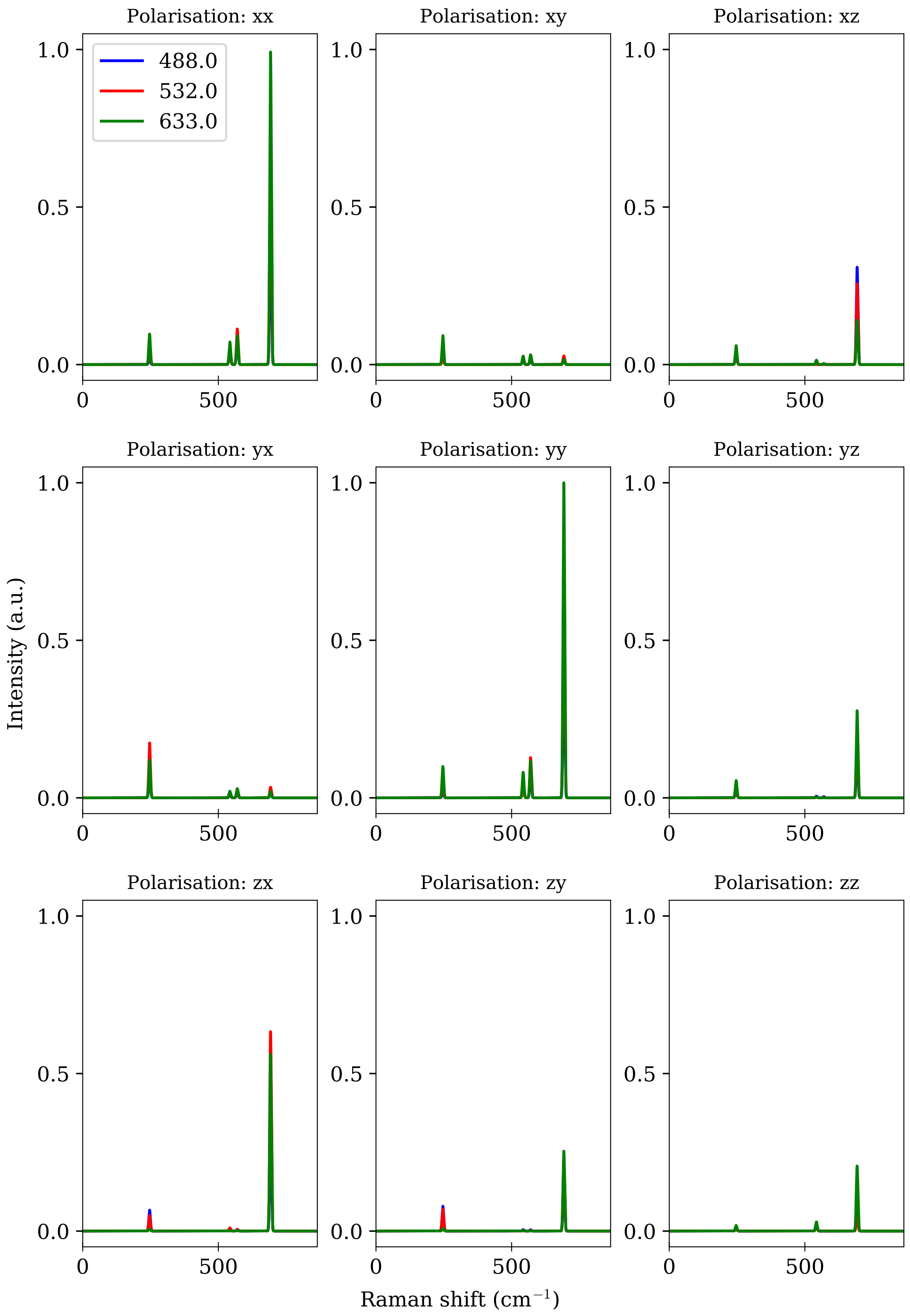

Supplementary Figure 205: Raman spectrum of FeO_2 with space group 115 and Wyckoff positions a-g-0.

FeS_2, 115, d-g-0

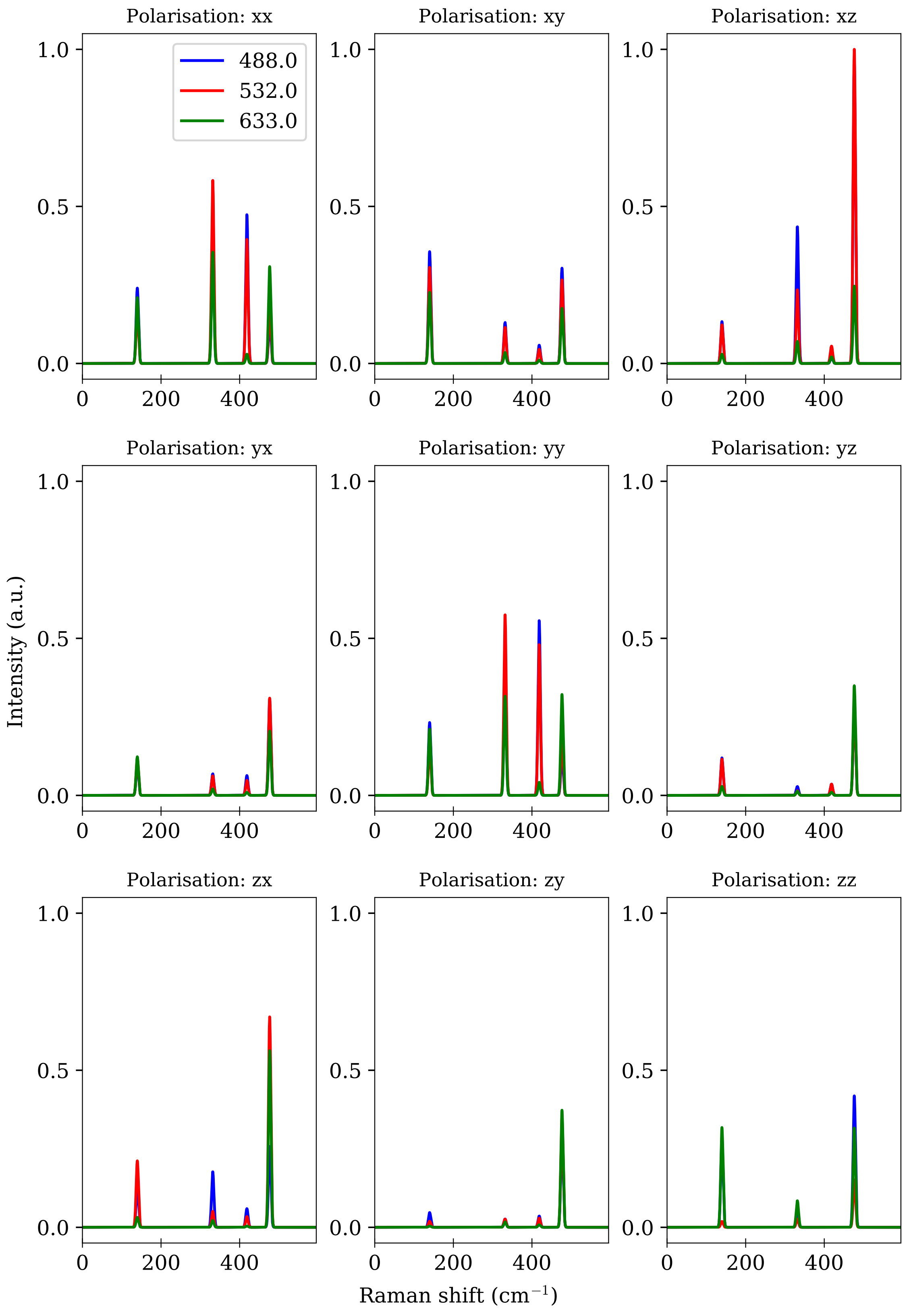

Supplementary Figure 206: Raman spectrum of FeS_2 with space group 115 and Wyckoff positions d-g-0.

$FeSe_2$, 115, a-g-0

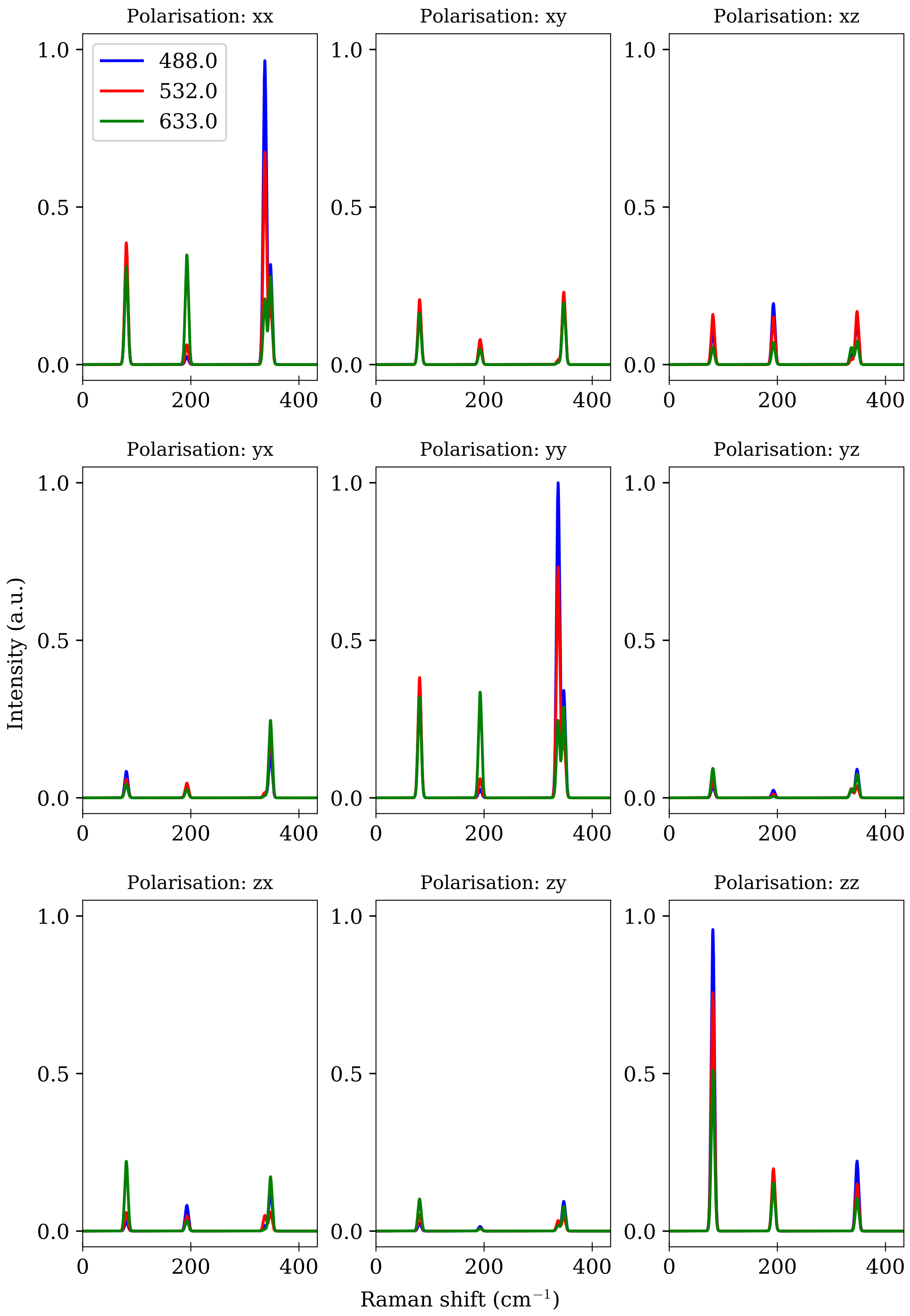

Supplementary Figure 207: Raman spectrum of $FeSe_2$ with space group 115 and Wyckoff positions a-g-0.

$Ga_2Br_2O_2$, 59, a-b-0

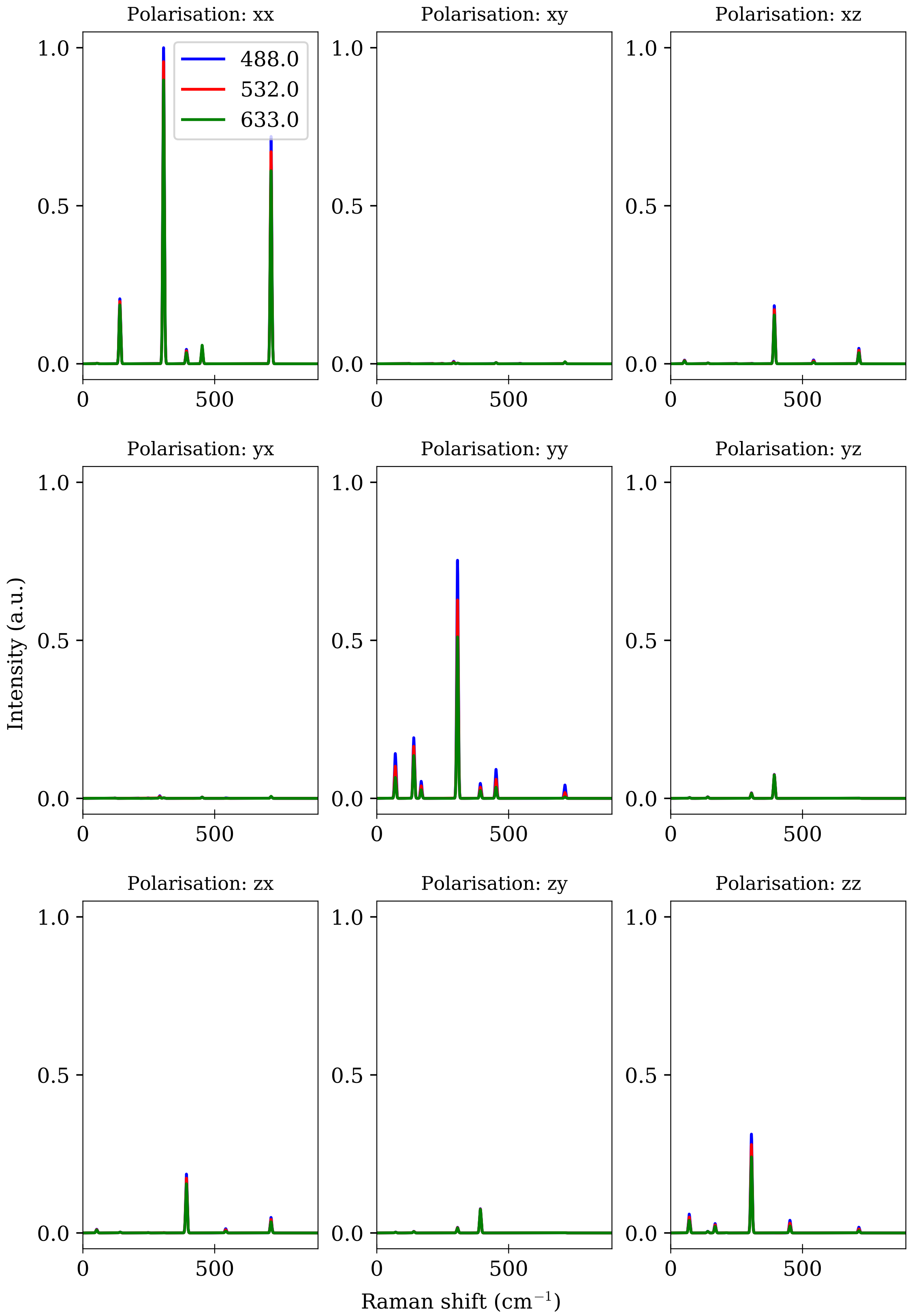

Supplementary Figure 208: Raman spectrum of $Ga_2Br_2O_2$ with space group 59 and Wyckoff positions a-b-0.

$Ga_2I_2S_2$, 59, a-b-0

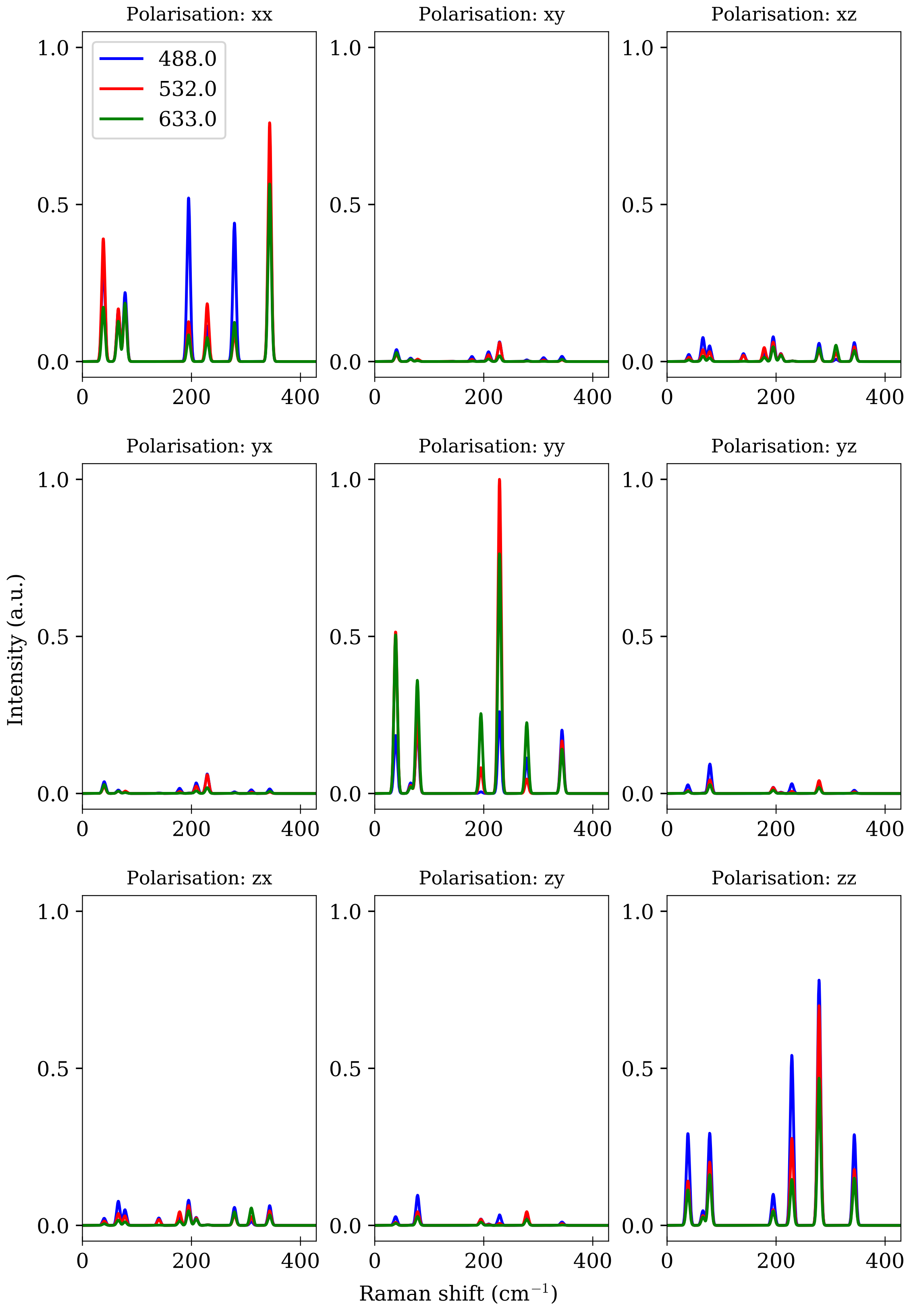

Supplementary Figure 209: Raman spectrum of $Ga_2I_2S_2$ with space group 59 and Wyckoff positions a-b-0.

Ga_2O_2, 187, h-i-0

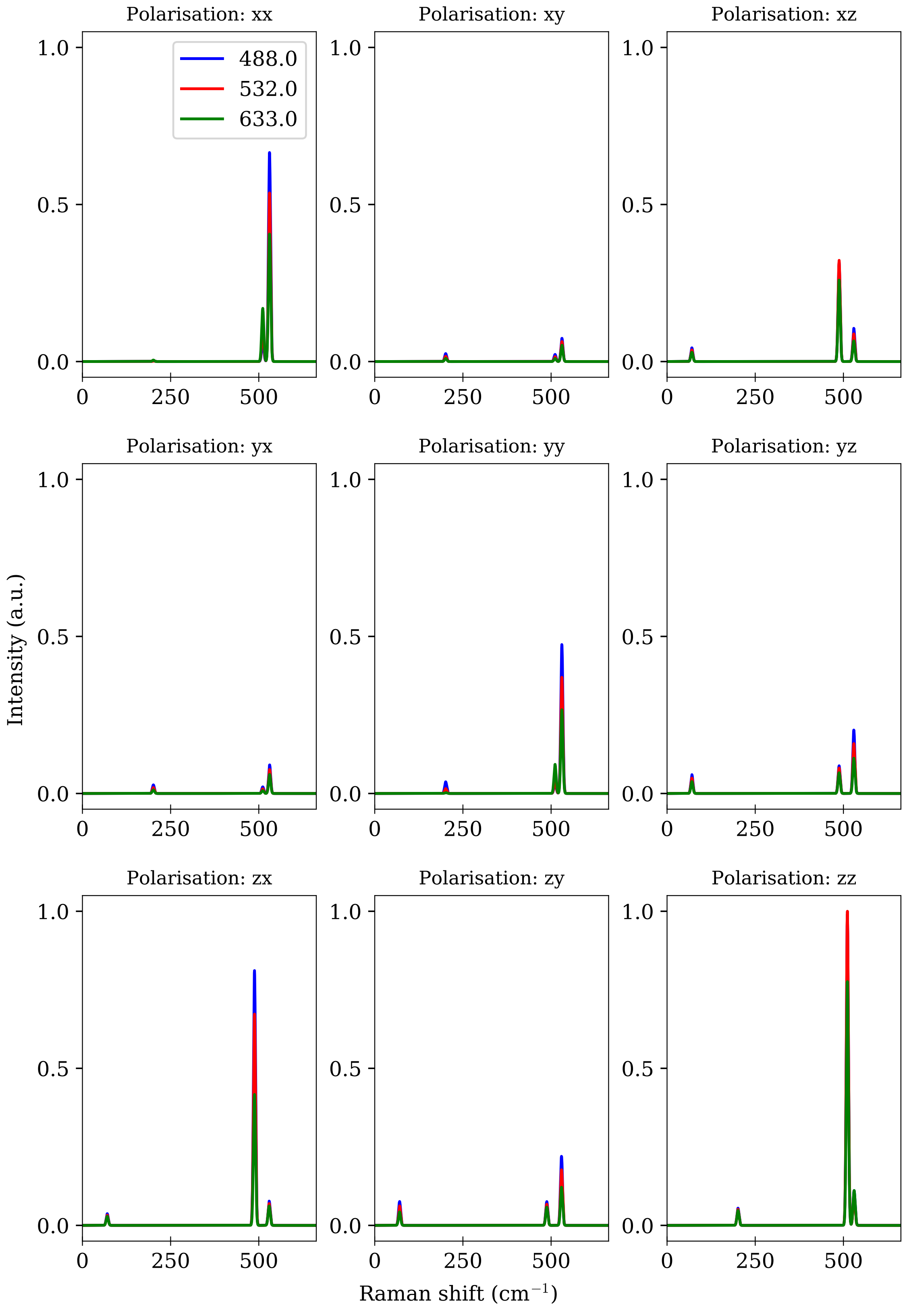

Supplementary Figure 210: Raman spectrum of Ga_2O_2 with space group 187 and Wyckoff positions h-i-0.

Ga_2S_2, 164, c-d-0

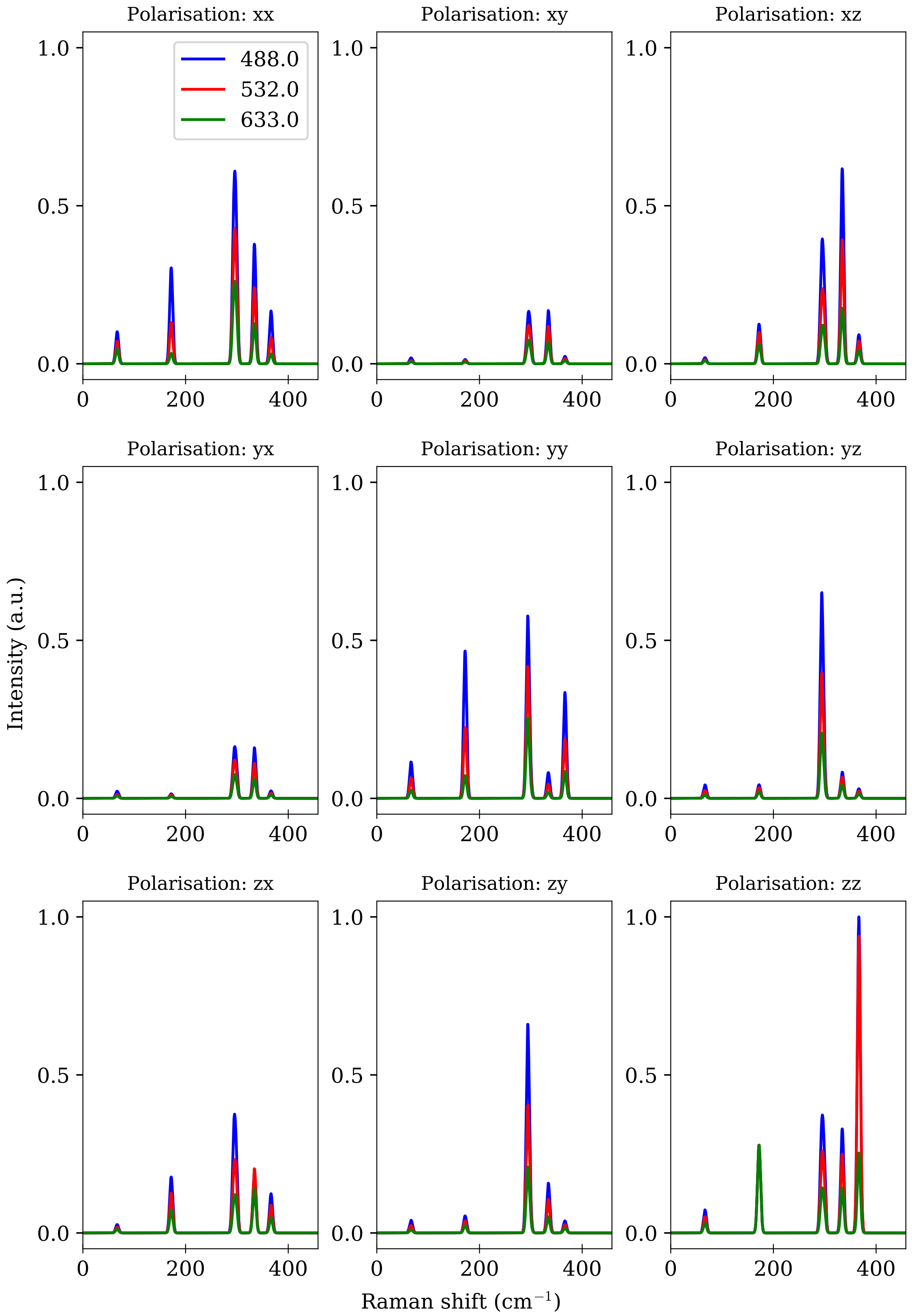

Supplementary Figure 211: Raman spectrum of Ga_2S_2 with space group 164 and Wyckoff positions c-d-0.

Ga_2S_2, 187, h-i-0

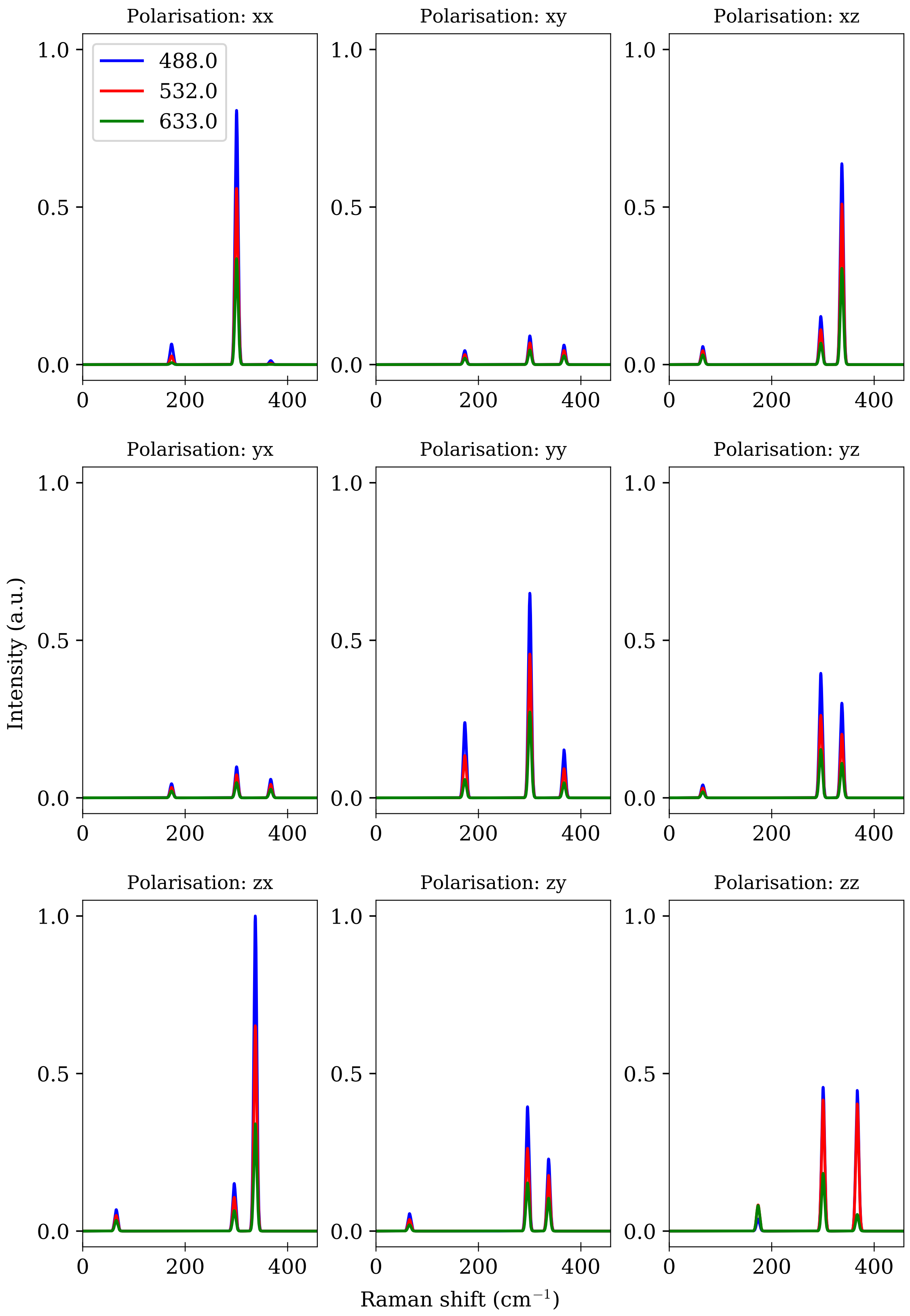

Supplementary Figure 212: Raman spectrum of Ga_2S_2 with space group 187 and Wyckoff positions h-i-0.

Ga_2Se_2, 187, h-i-0

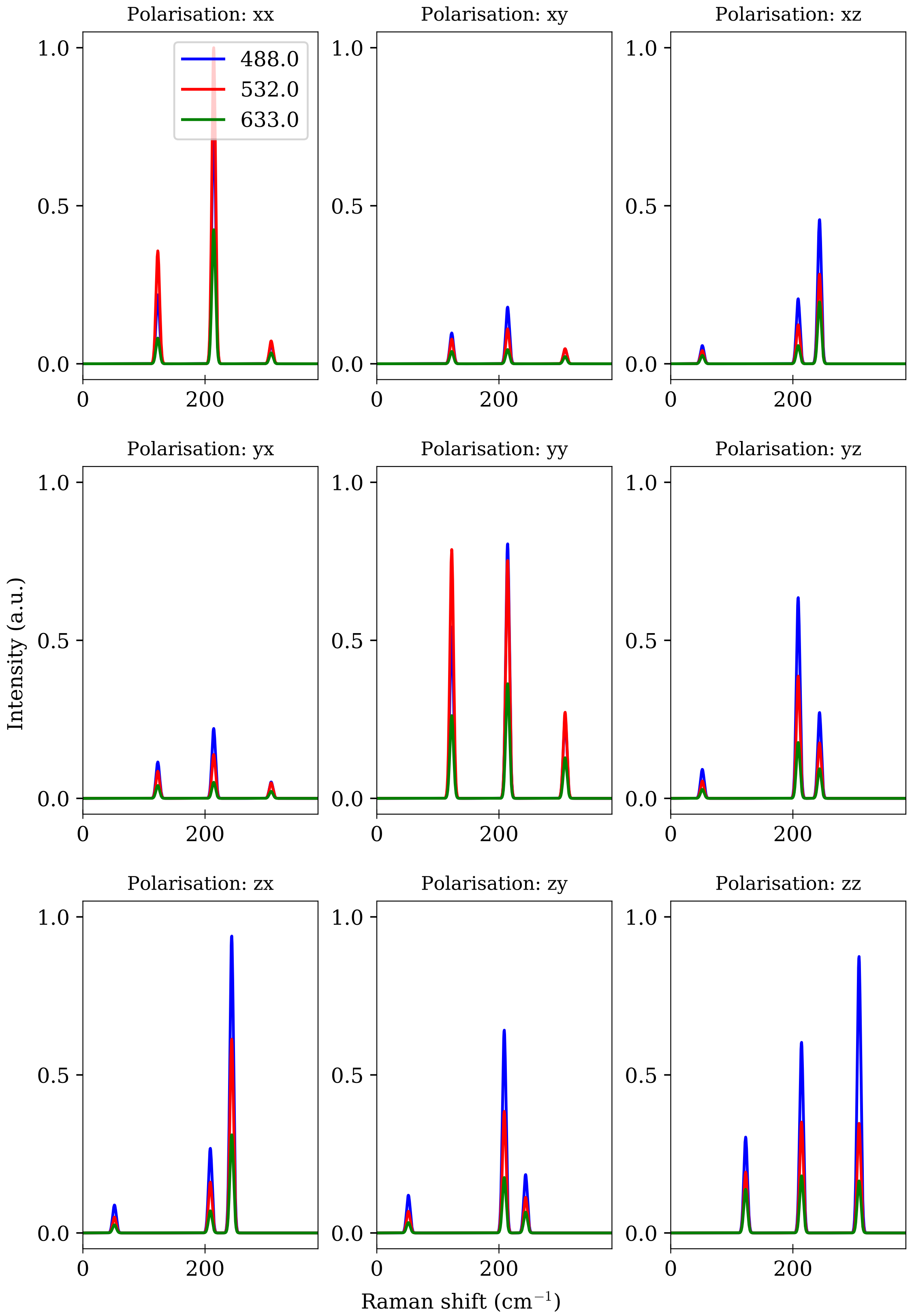

Supplementary Figure 213: Raman spectrum of Ga_2Se_2 with space group 187 and Wyckoff positions h-i-0.

Ga_2Te_2, 123, b-d-0

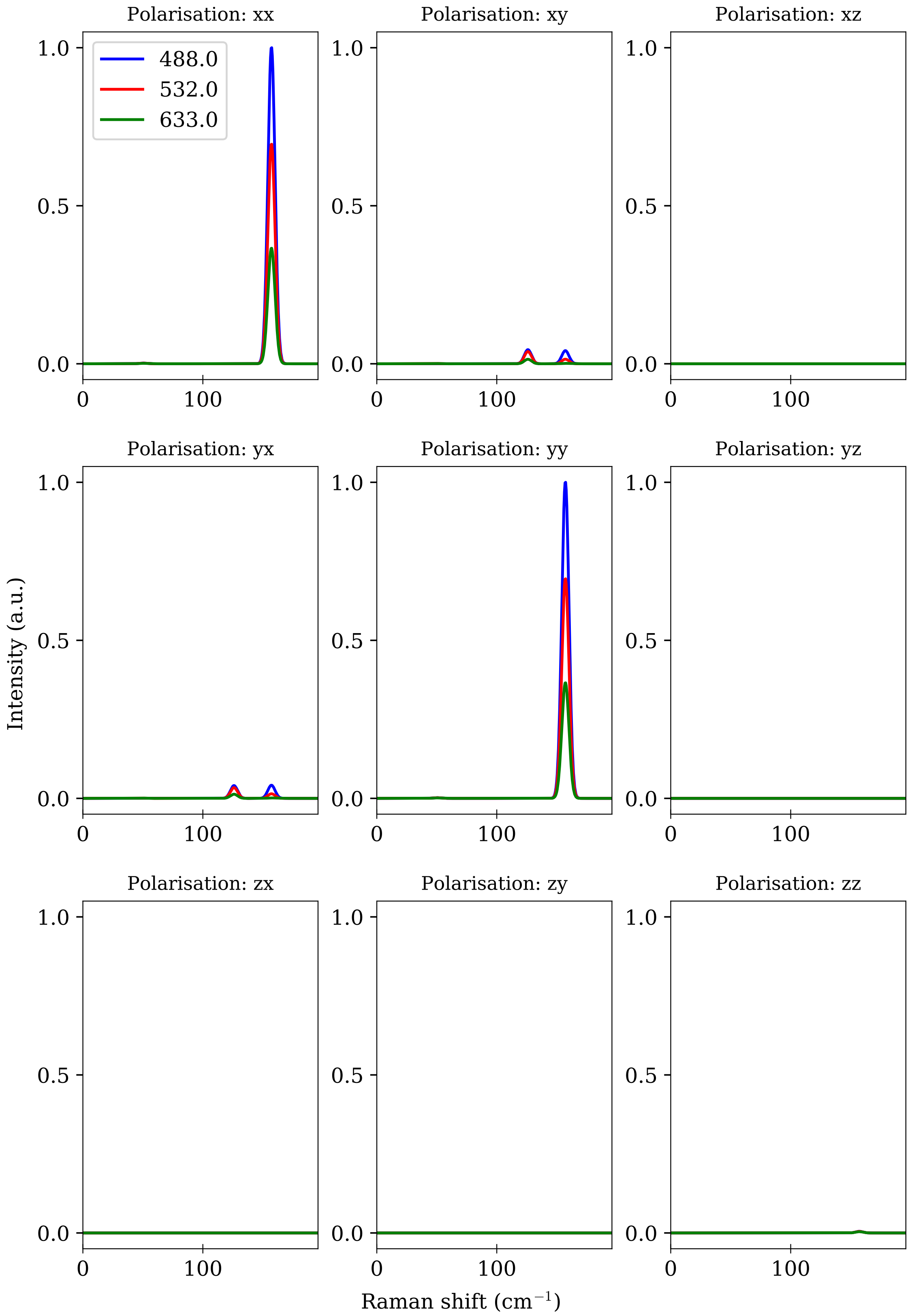

Supplementary Figure 214: Raman spectrum of Ga_2Te_2 with space group 123 and Wyckoff positions b-d-0.

Ga_2Te_2, 2, i-0

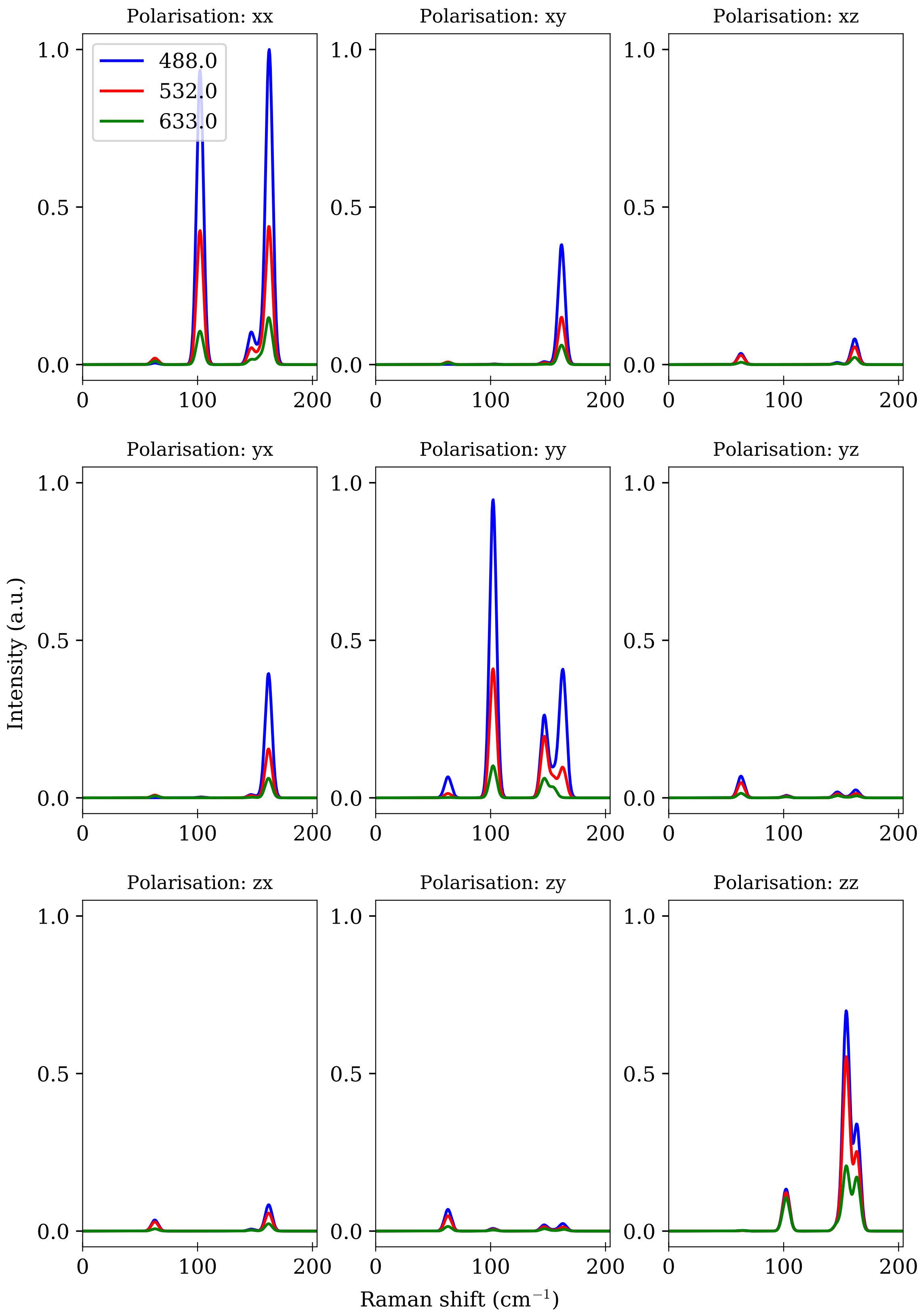

Supplementary Figure 215: Raman spectrum of Ga_2Te_2 with space group 2 and Wyckoff positions i-0.

GaAs, 156, b-c-0

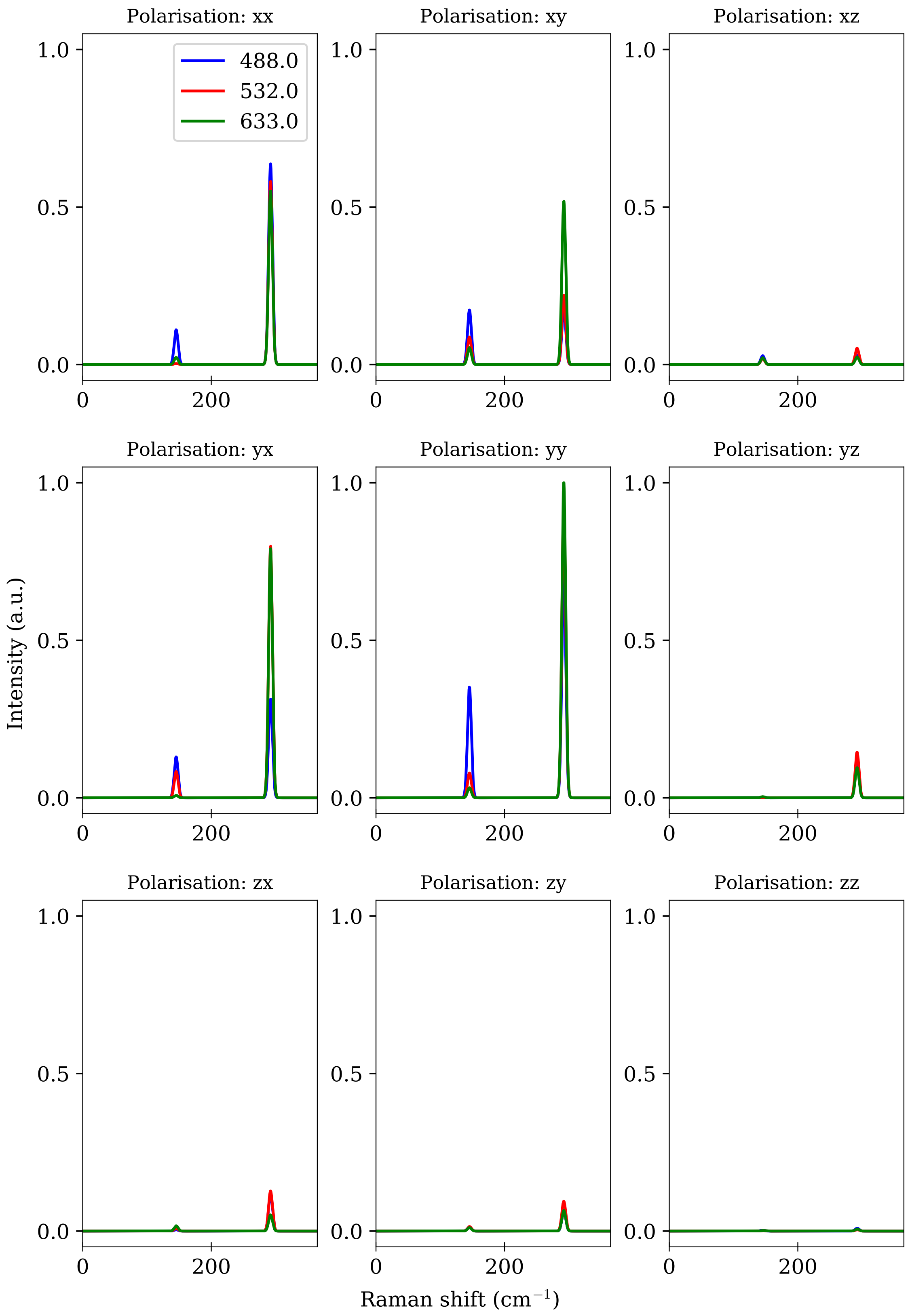

Supplementary Figure 216: Raman spectrum of GaAs with space group 156 and Wyckoff positions b-c-0.

GaI_2, 164, b-d-0

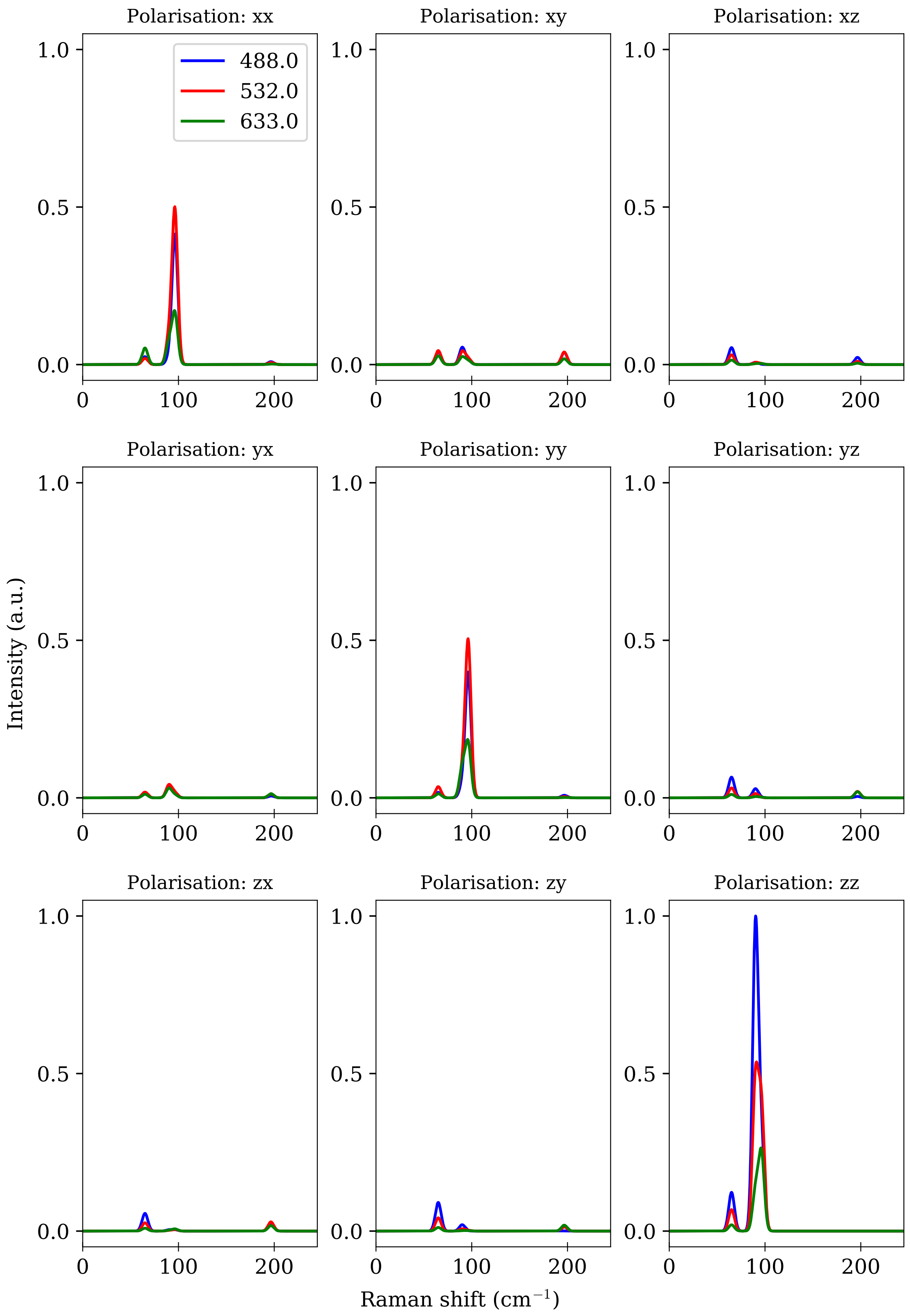

Supplementary Figure 217: Raman spectrum of GaI_2 with space group 164 and Wyckoff positions b-d-0.

GaN, 187, d-f-0

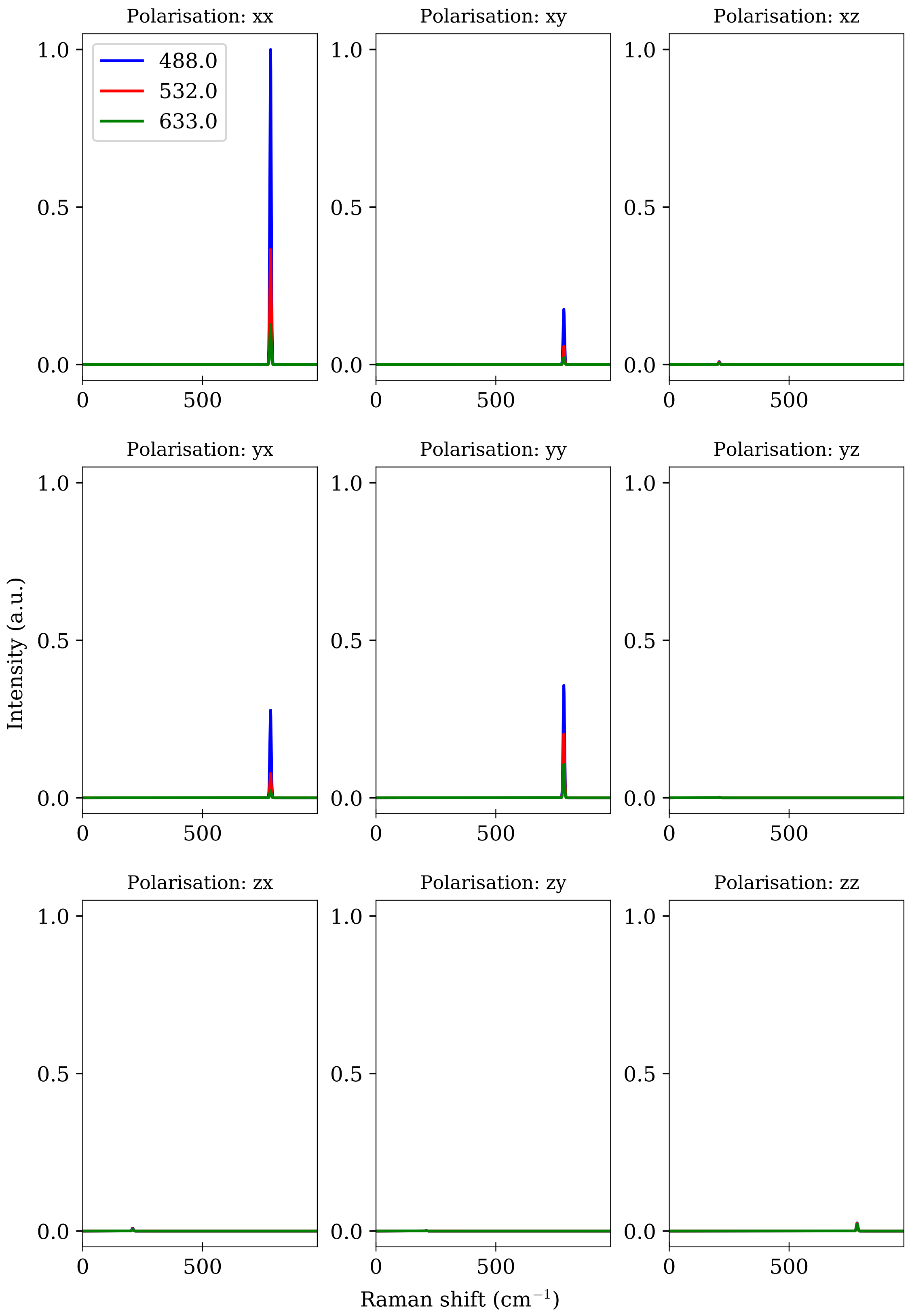

Supplementary Figure 218: Raman spectrum of GaN with space group 187 and Wyckoff positions d-f-0.

GaP, 156, b-c-0

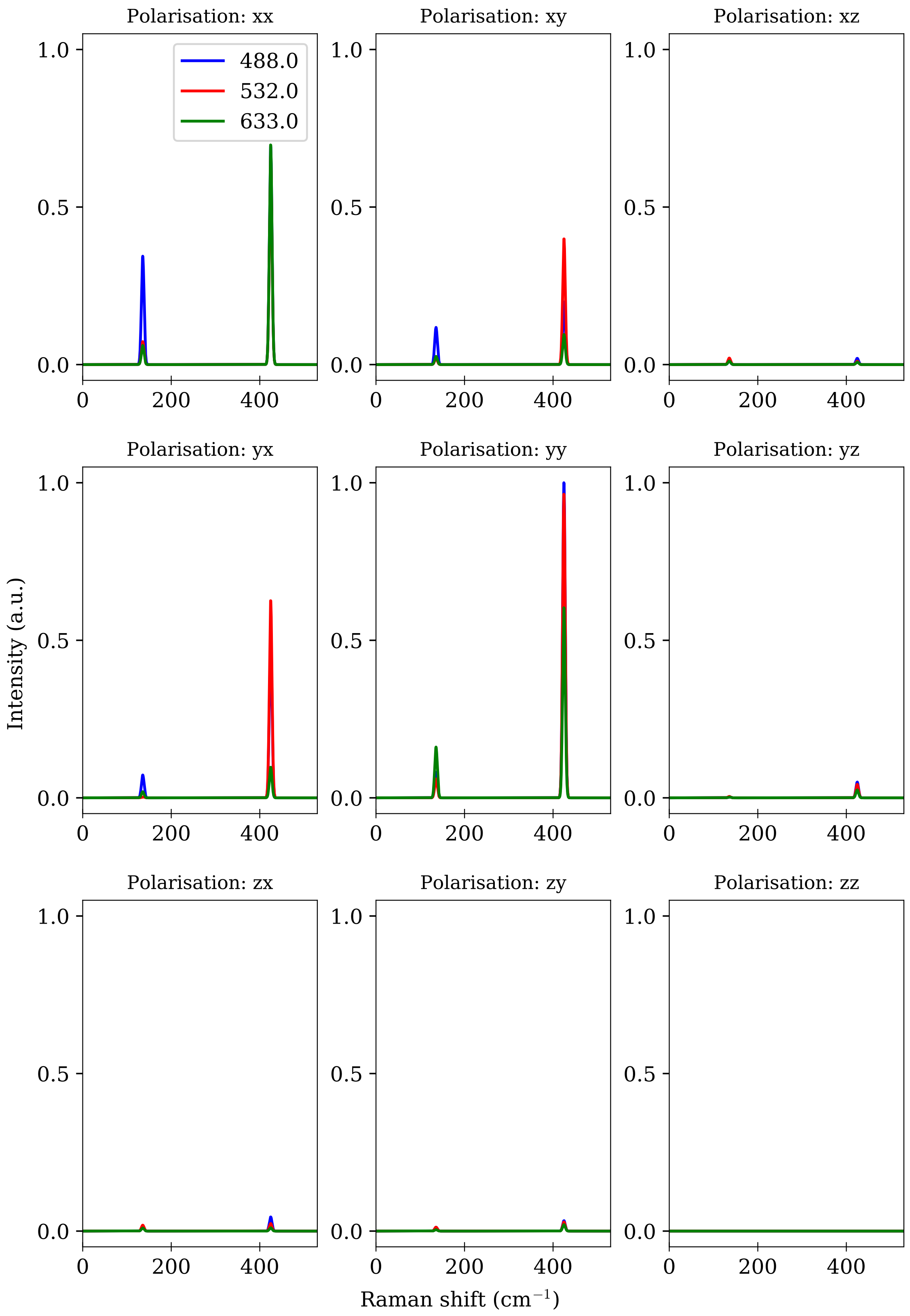

Supplementary Figure 219: Raman spectrum of GaP with space group 156 and Wyckoff positions b-c-0.

$GaSe_2$, 115, a-g-0

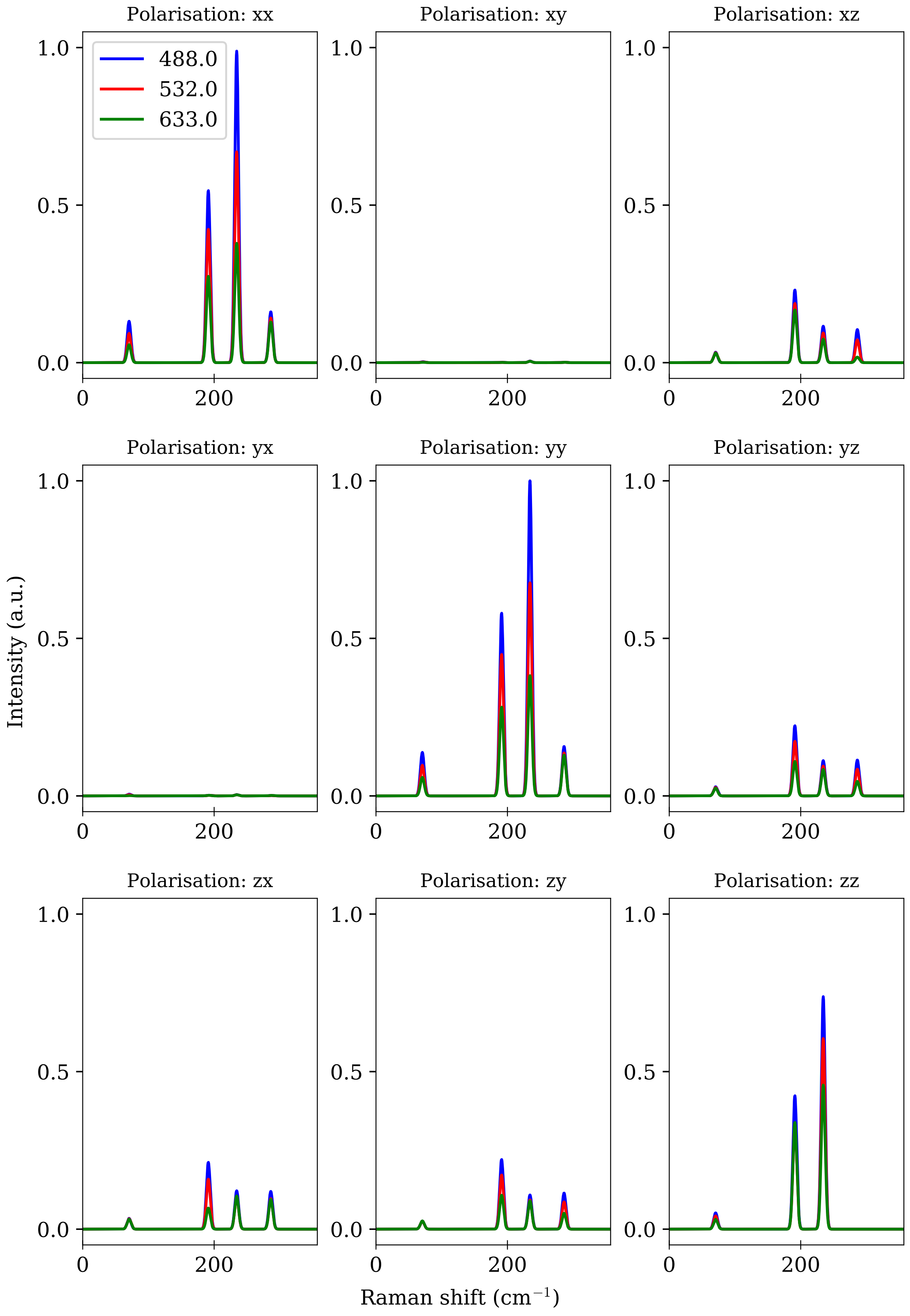

Supplementary Figure 220: Raman spectrum of $GaSe_2$ with space group 115 and Wyckoff positions a-g-0.

Ge_2, 164, d-0

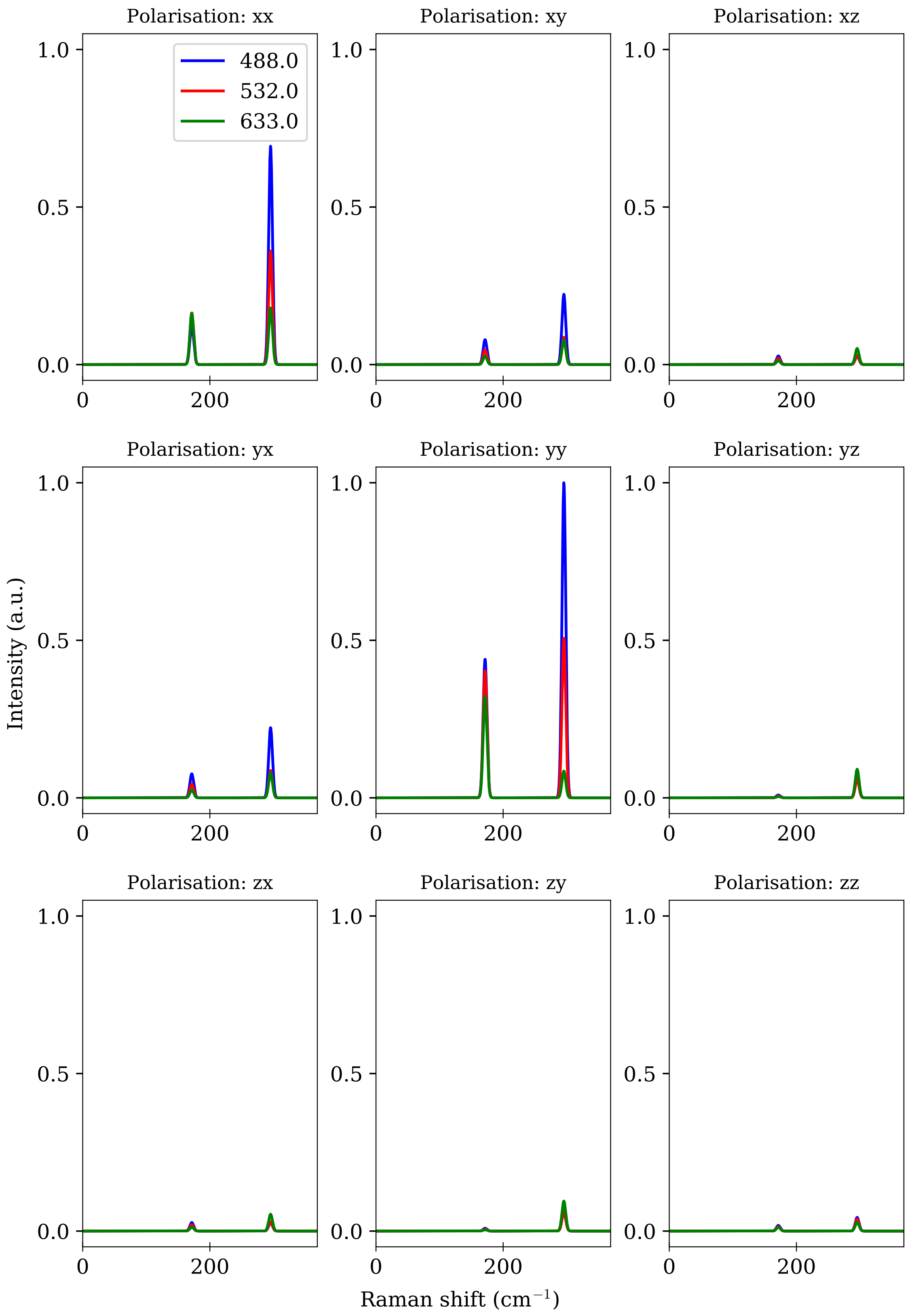

Supplementary Figure 221: Raman spectrum of Ge_2 with space group 164 and Wyckoff positions d-0.

GeI_2, 164, b-d-0

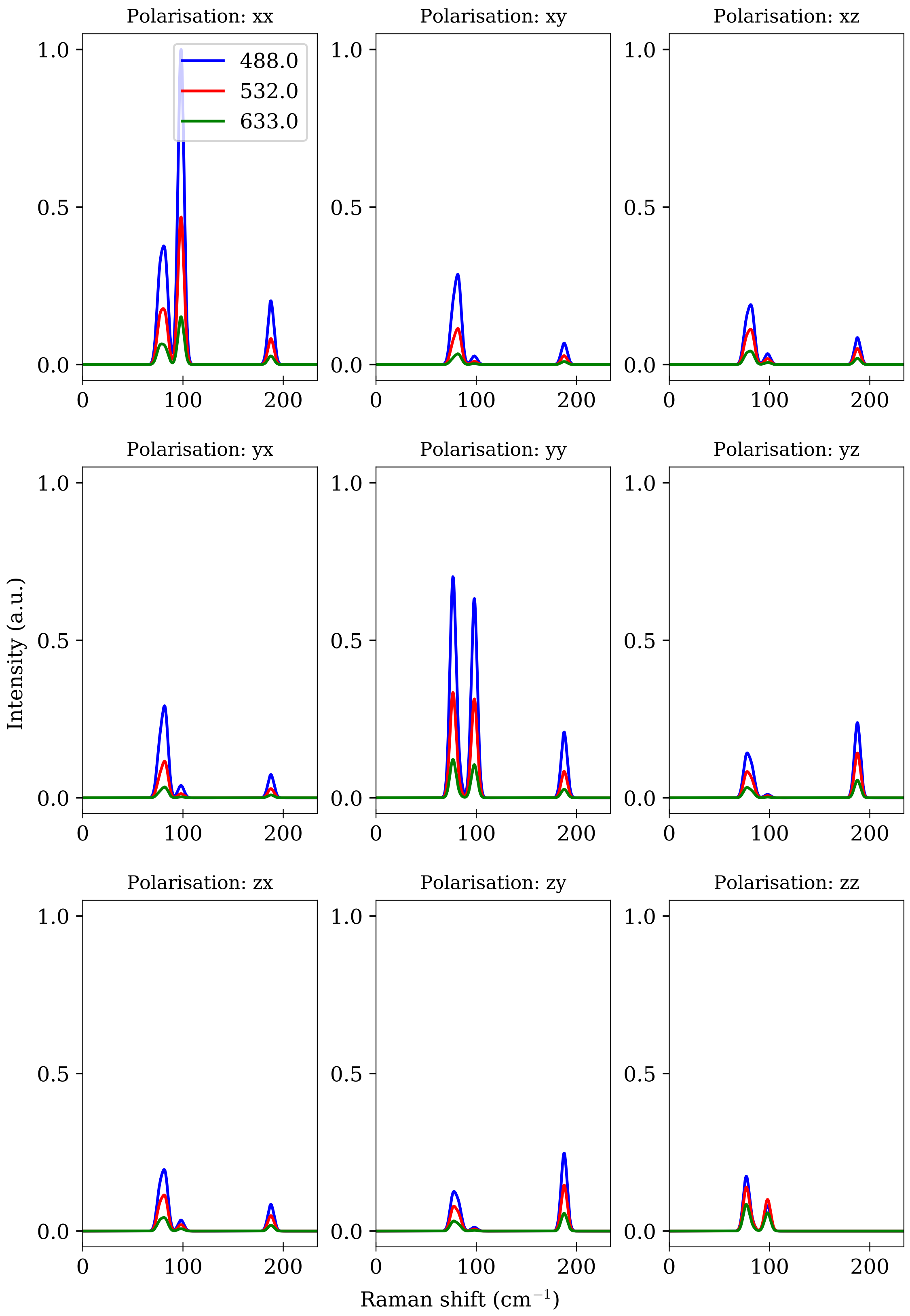

Supplementary Figure 222: Raman spectrum of GeI_2 with space group 164 and Wyckoff positions b-d-0.

GeO_2, 115, d-g-0

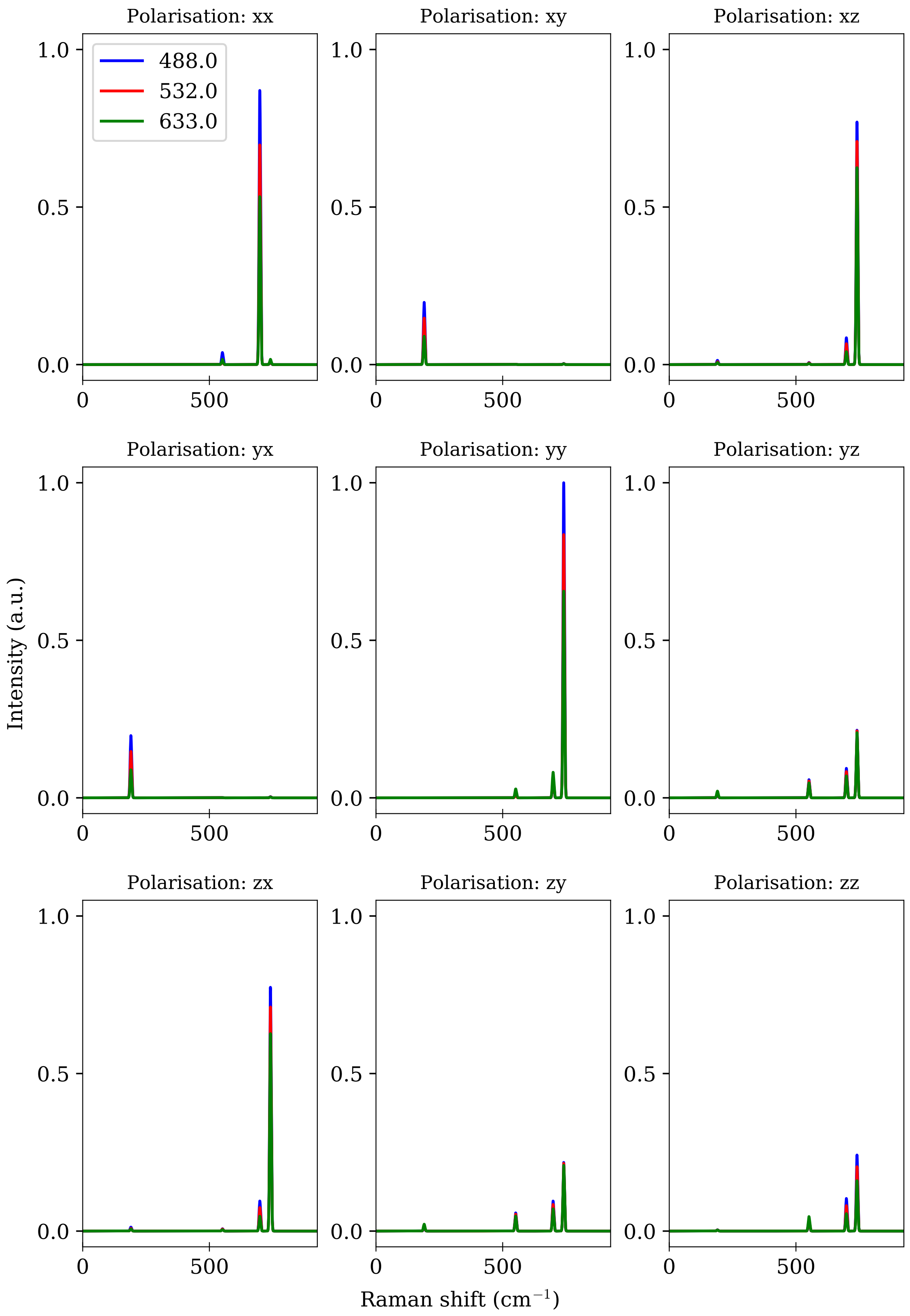

Supplementary Figure 223: Raman spectrum of GeO_2 with space group 115 and Wyckoff positions d-g-0.

GeO_2, 164, b-d-0

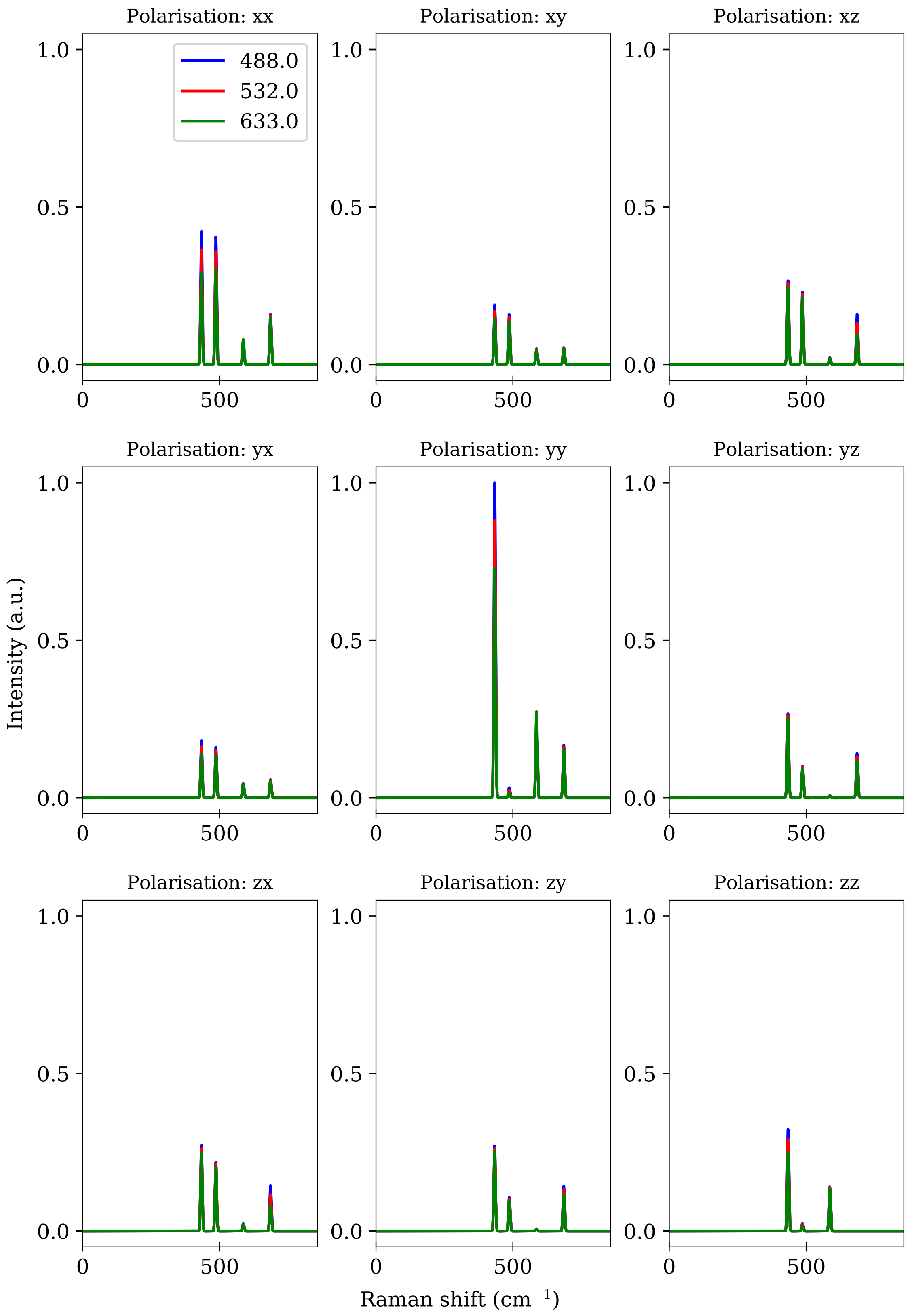

Supplementary Figure 224: Raman spectrum of GeO_2 with space group 164 and Wyckoff positions b-d-0.

GeO, 156, b-c-0

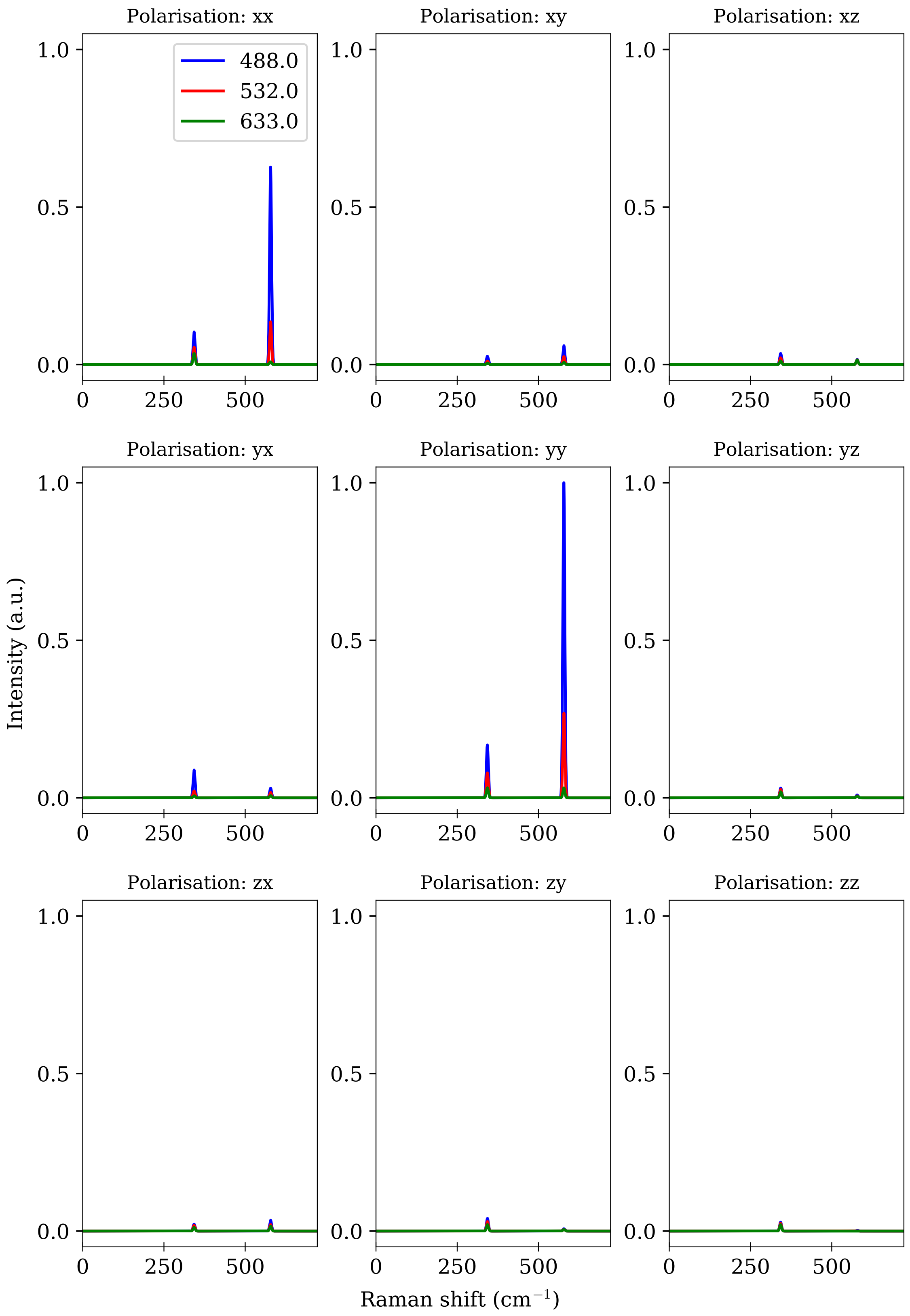

Supplementary Figure 225: Raman spectrum of GeO with space group 156 and Wyckoff positions b-c-0.

GeS_2, 115, a-g-0

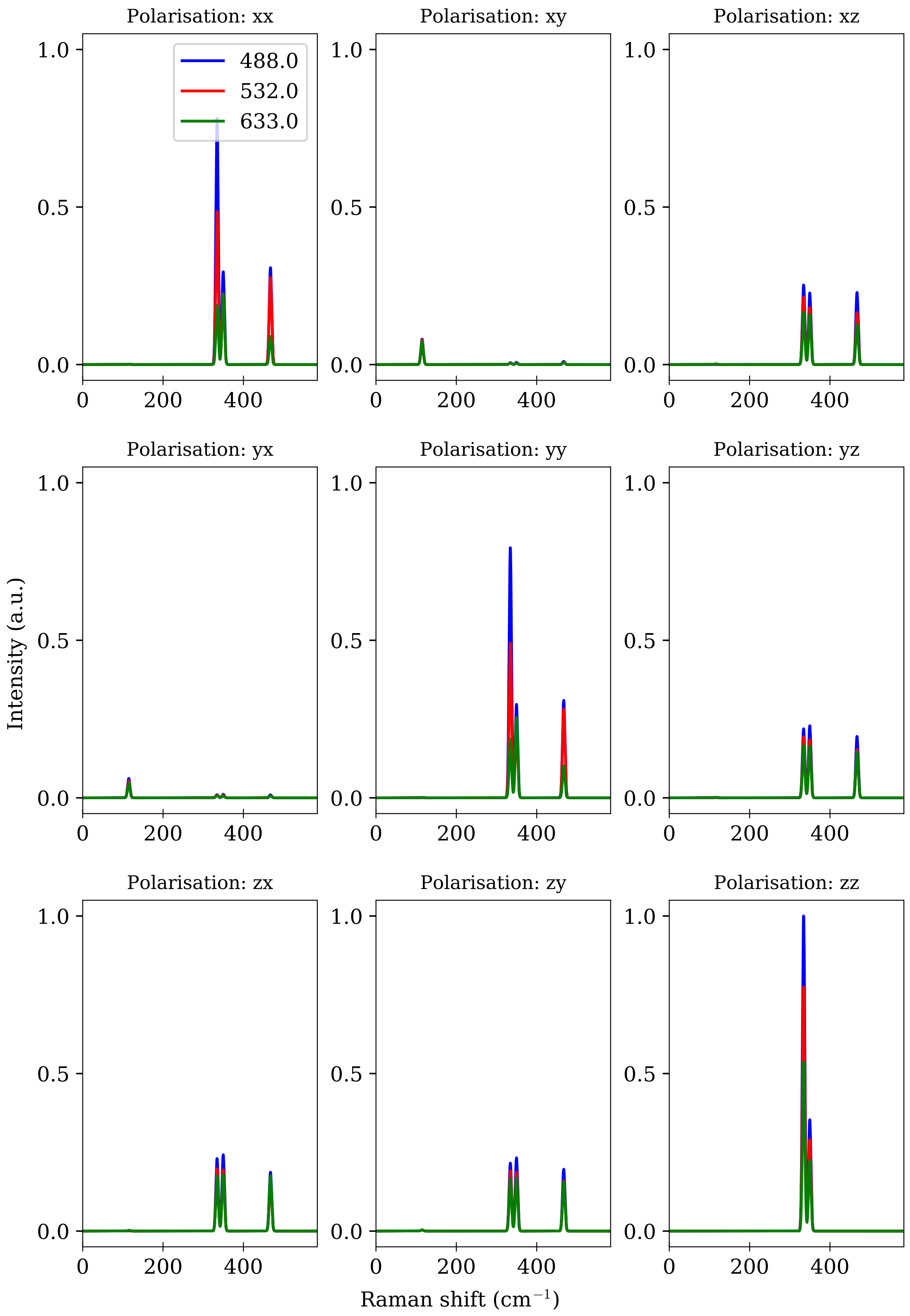

Supplementary Figure 226: Raman spectrum of GeS_2 with space group 115 and Wyckoff positions a-g-0.

GeS_2, 164, b-d-0

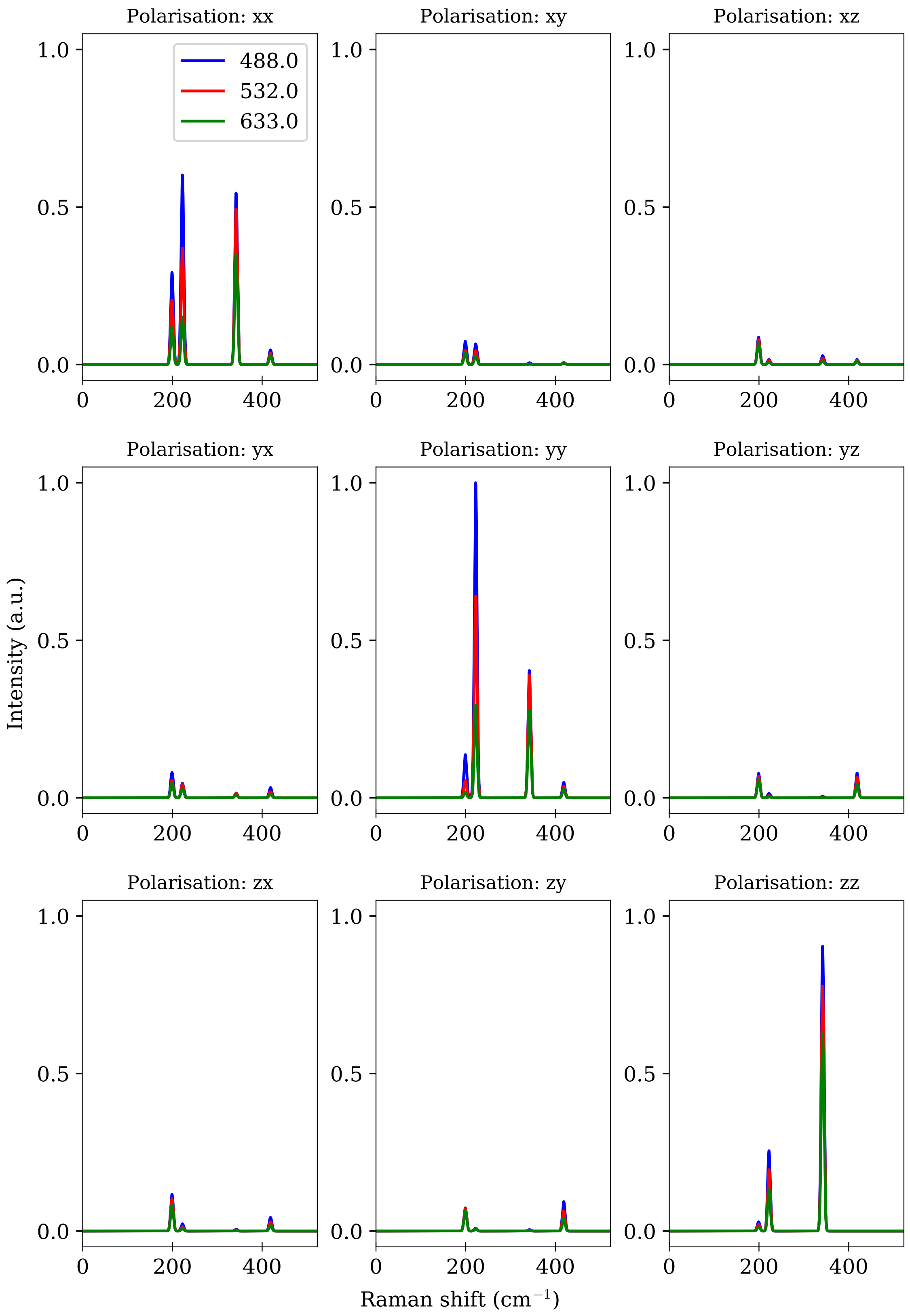

Supplementary Figure 227: Raman spectrum of GeS_2 with space group 164 and Wyckoff positions b-d-0.

$GeSe_2$, 115, d-g-0

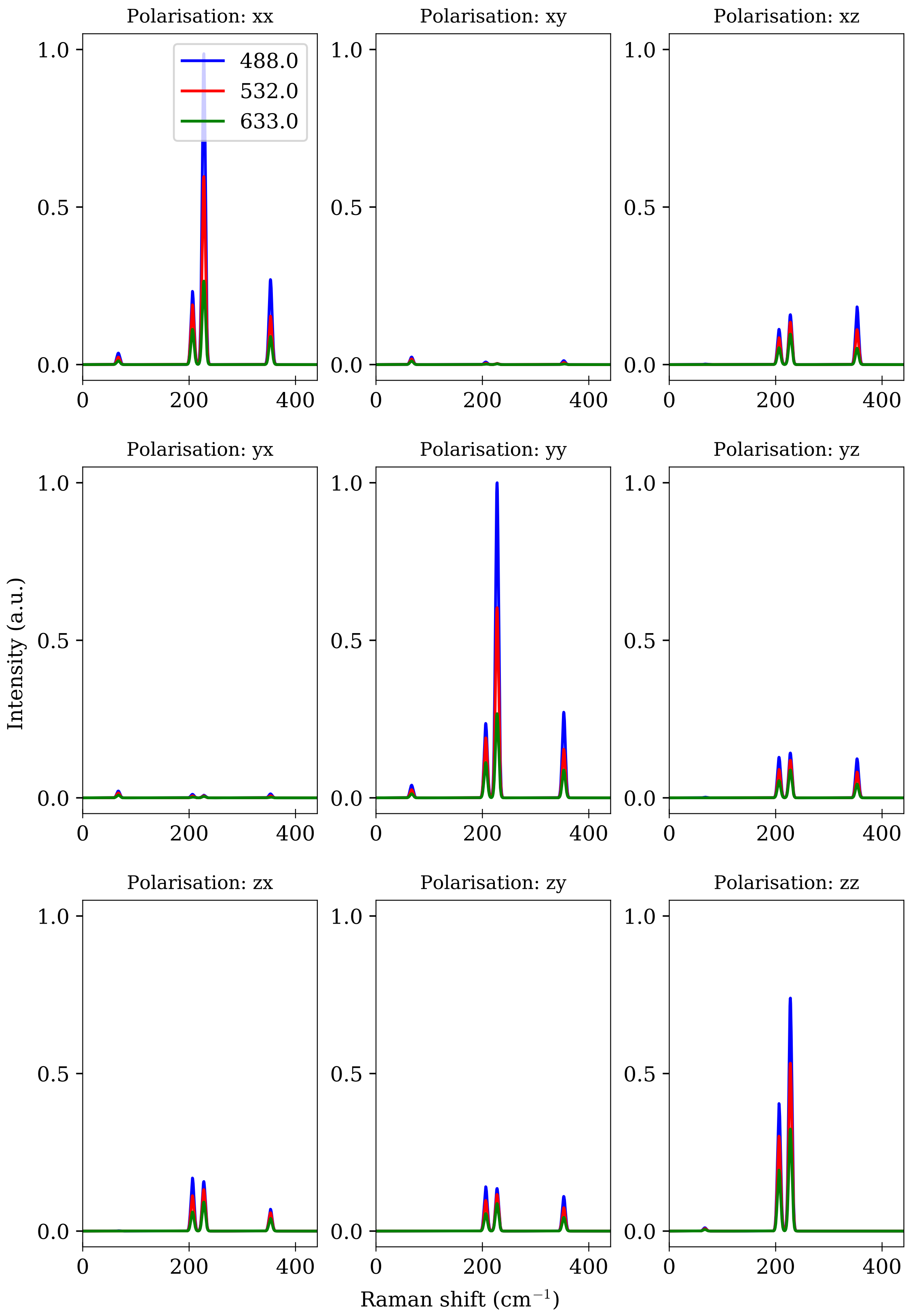

Supplementary Figure 228: Raman spectrum of $GeSe_2$ with space group 115 and Wyckoff positions d-g-0.

$GeSe_2$, 164, b-d-0

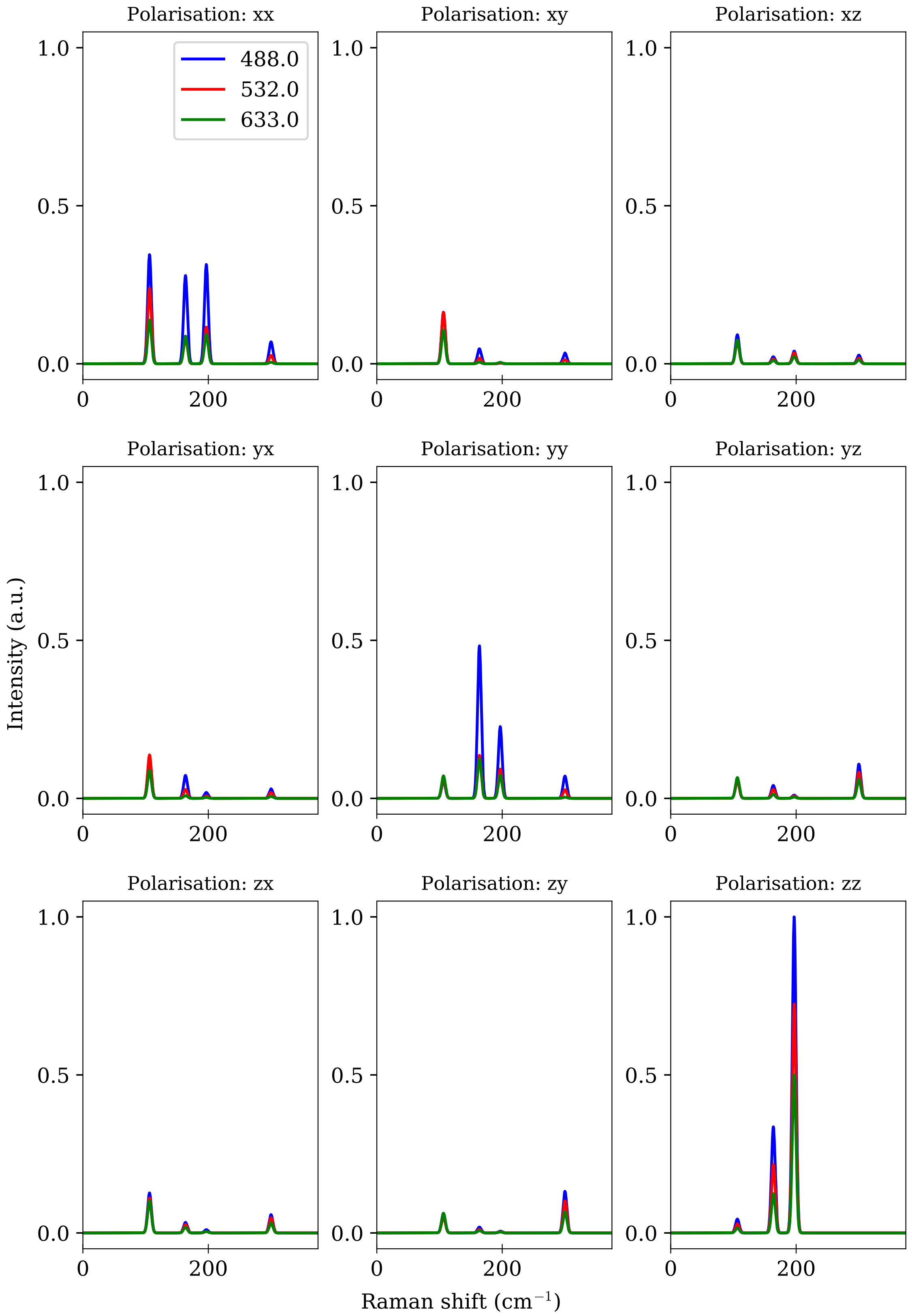

Supplementary Figure 229: Raman spectrum of $GeSe_2$ with space group 164 and Wyckoff positions b-d-0.

GeSe, 156, b-c-0

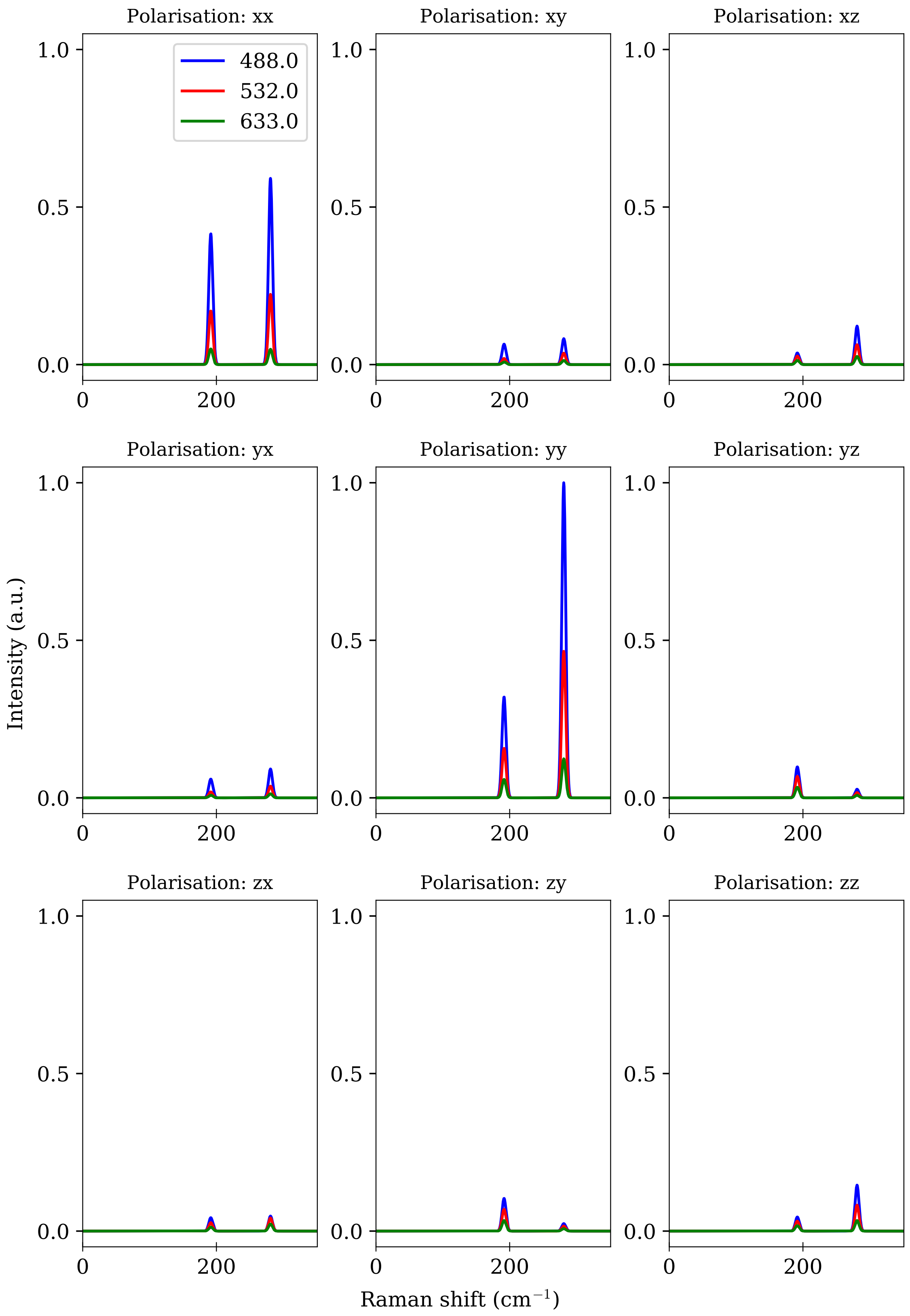

Supplementary Figure 230: Raman spectrum of GeSe with space group 156 and Wyckoff positions b-c-0.

GeS, 156, b-c-0

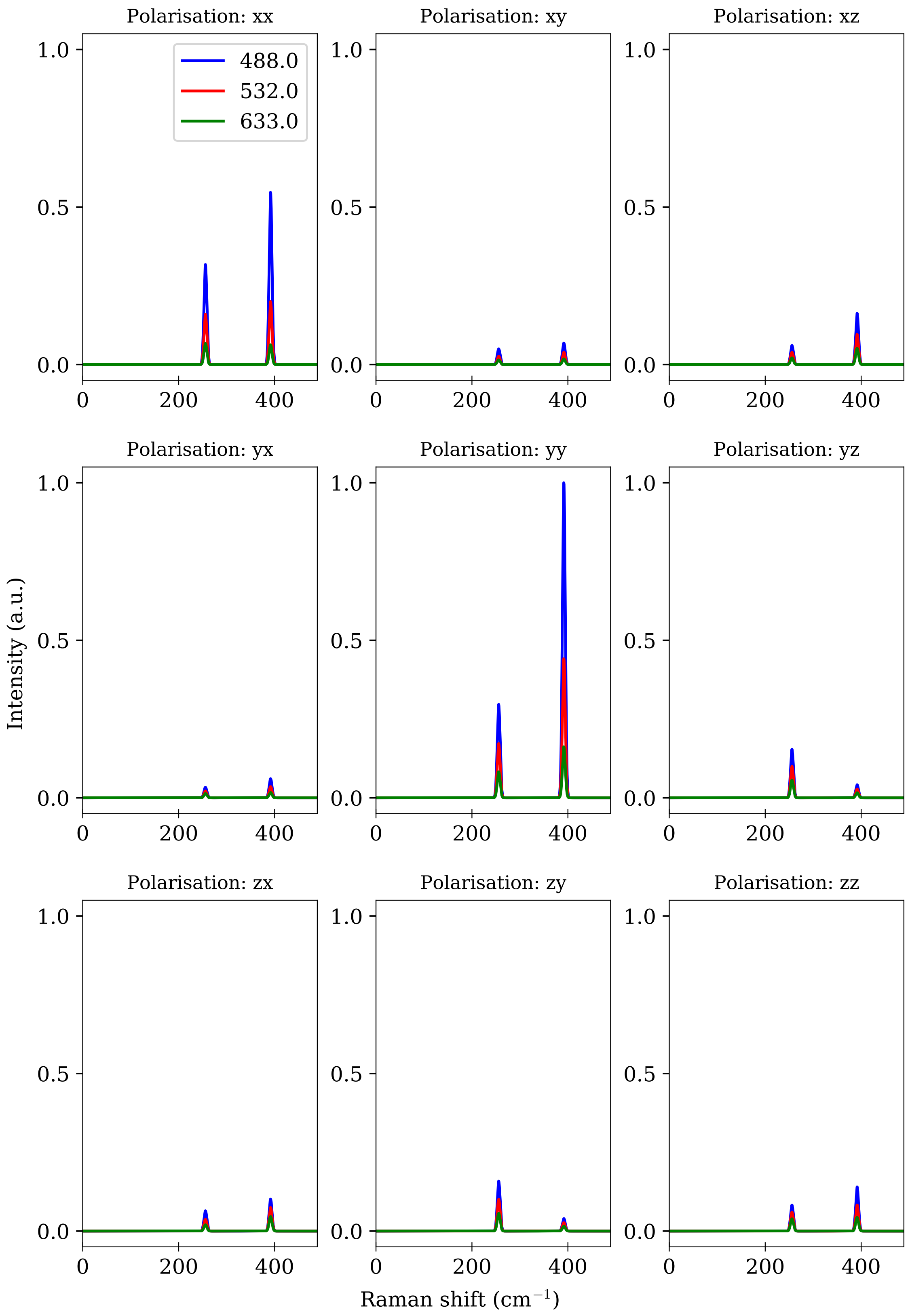

Supplementary Figure 231: Raman spectrum of GeS with space group 156 and Wyckoff positions b-c-0.

$GeTe_2$, 164, b-d-0

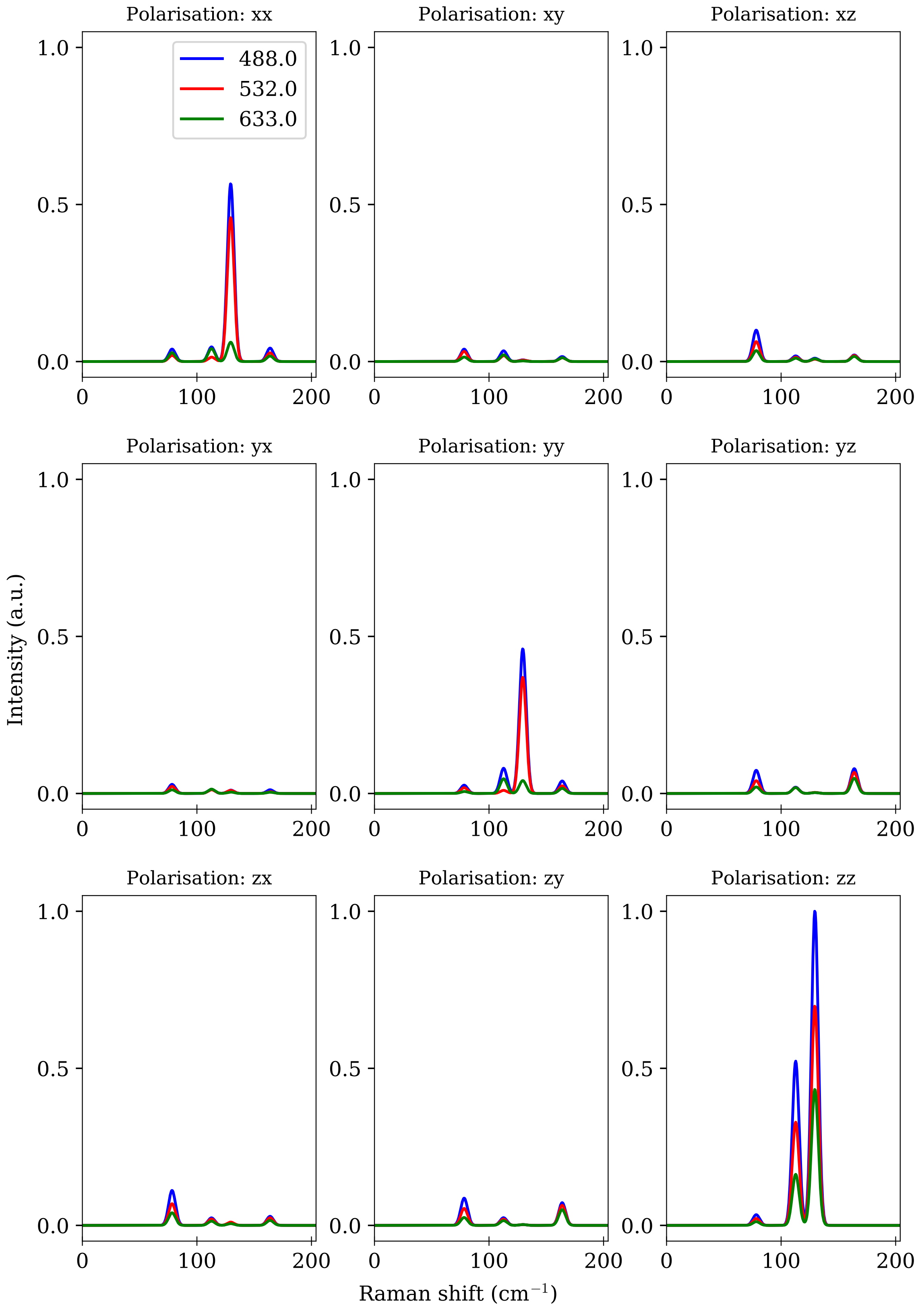

Supplementary Figure 232: Raman spectrum of $GeTe_2$ with space group 164 and Wyckoff positions b-d-0.

$GeTe_2$, 187, b-i-0

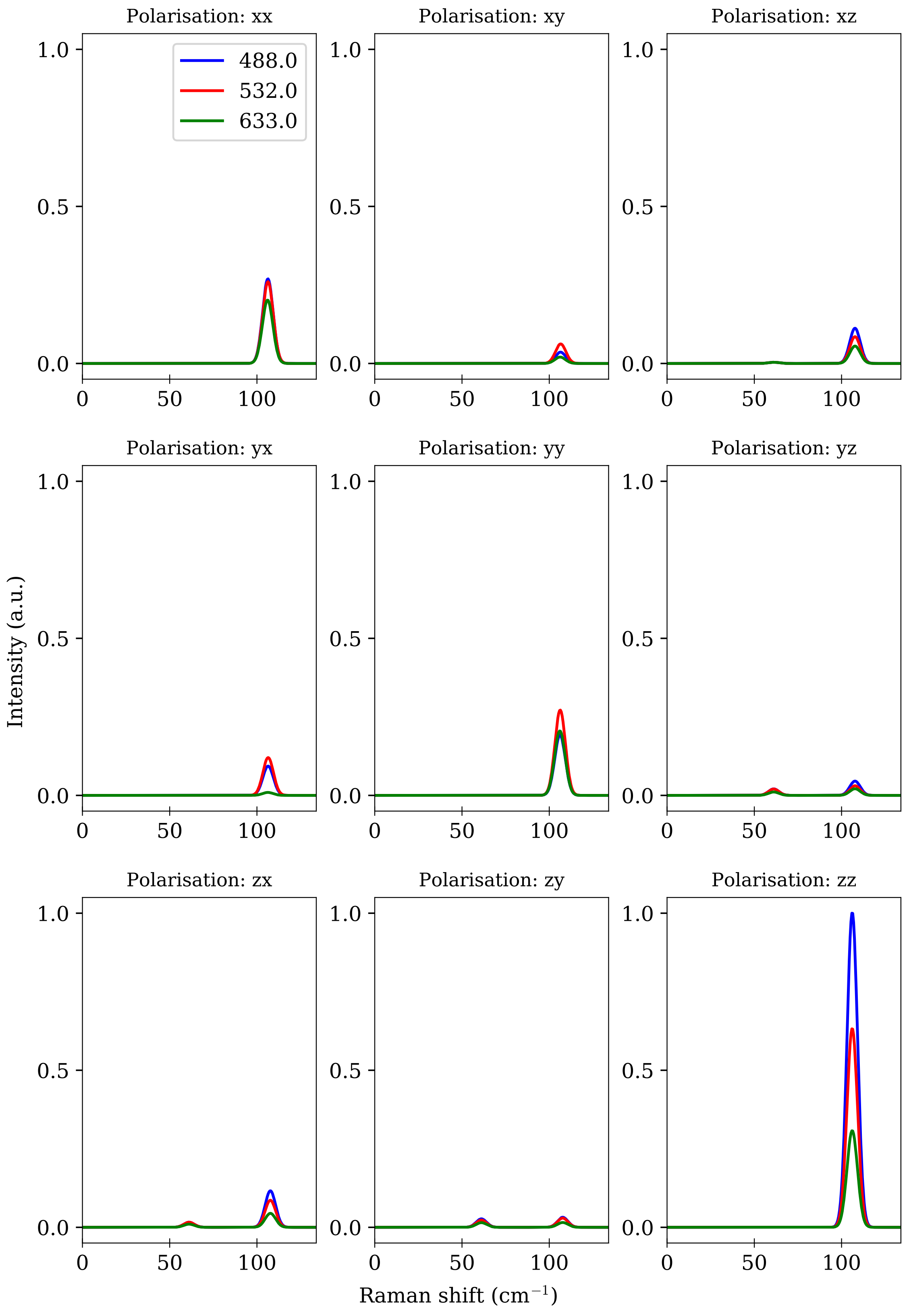

Supplementary Figure 233: Raman spectrum of $GeTe_2$ with space group 187 and Wyckoff positions b-i-0.

GeTe, 156, b-c-0

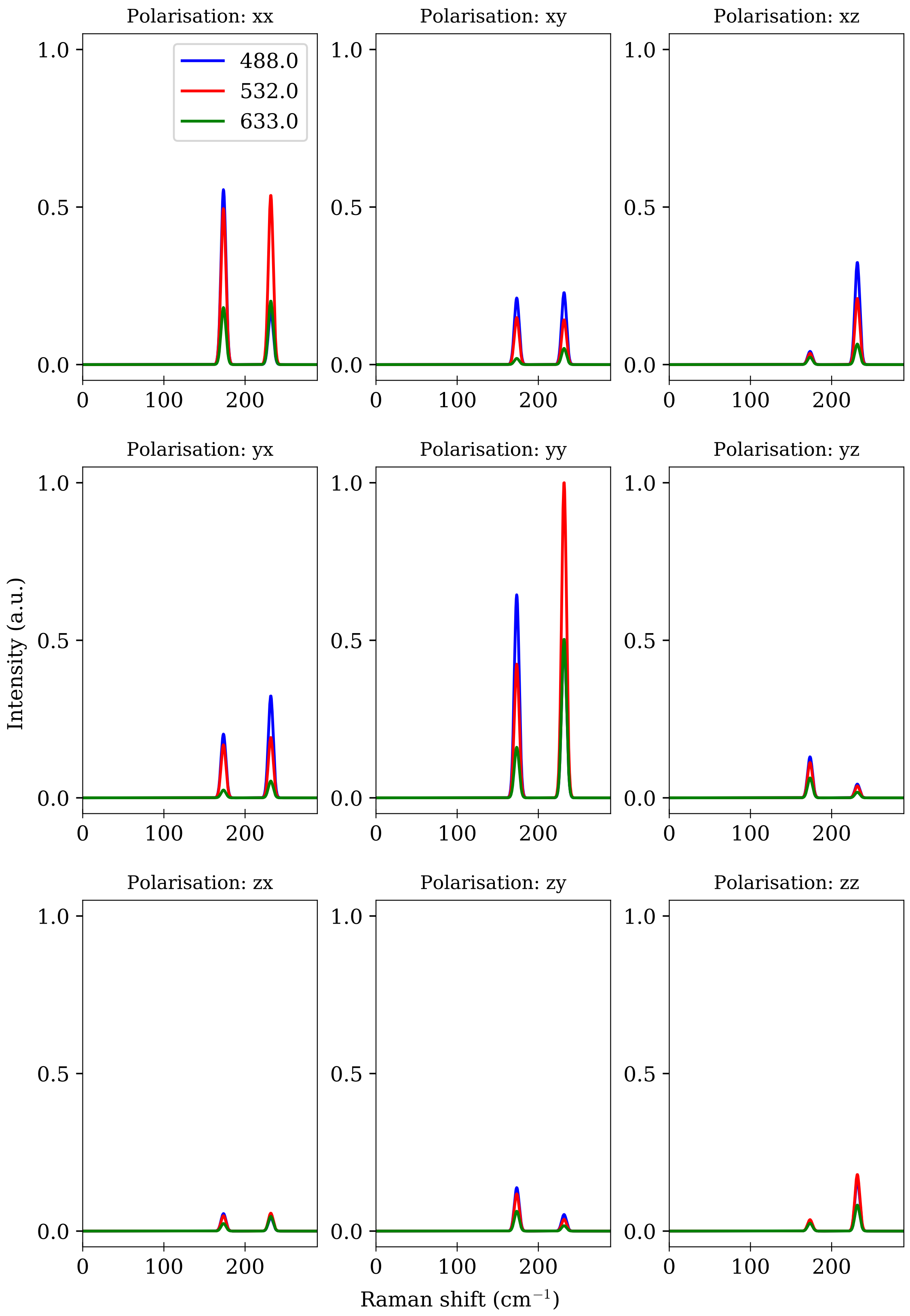

Supplementary Figure 234: Raman spectrum of GeTe with space group 156 and Wyckoff positions b-c-0.

$Hf_2Br_2N_2$, 59, a-b-0

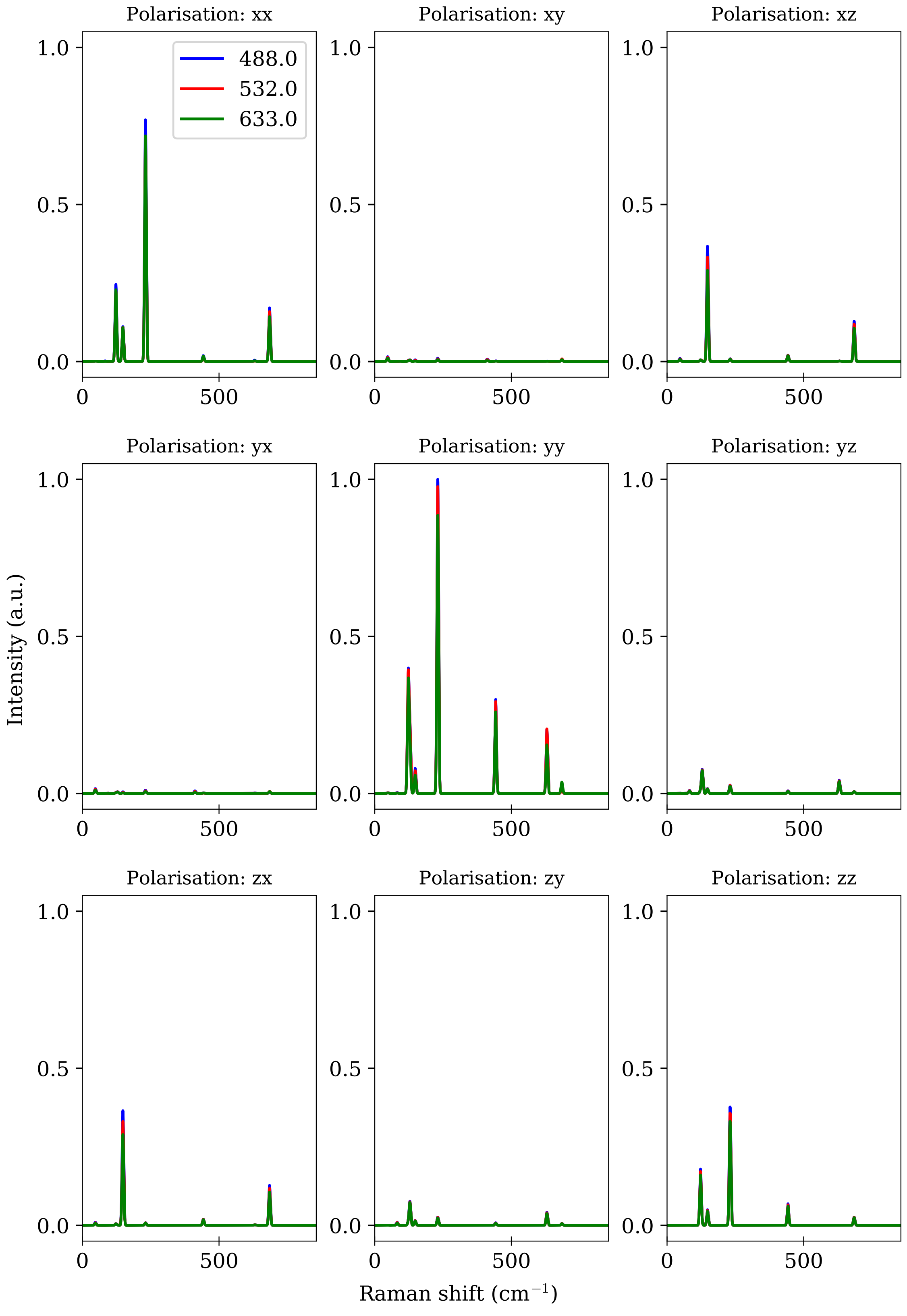

Supplementary Figure 235: Raman spectrum of $Hf_2Br_2N_2$ with space group 59 and Wyckoff positions a-b-0.

$Hf_2Br_2Se_2$, 59, a-b-0

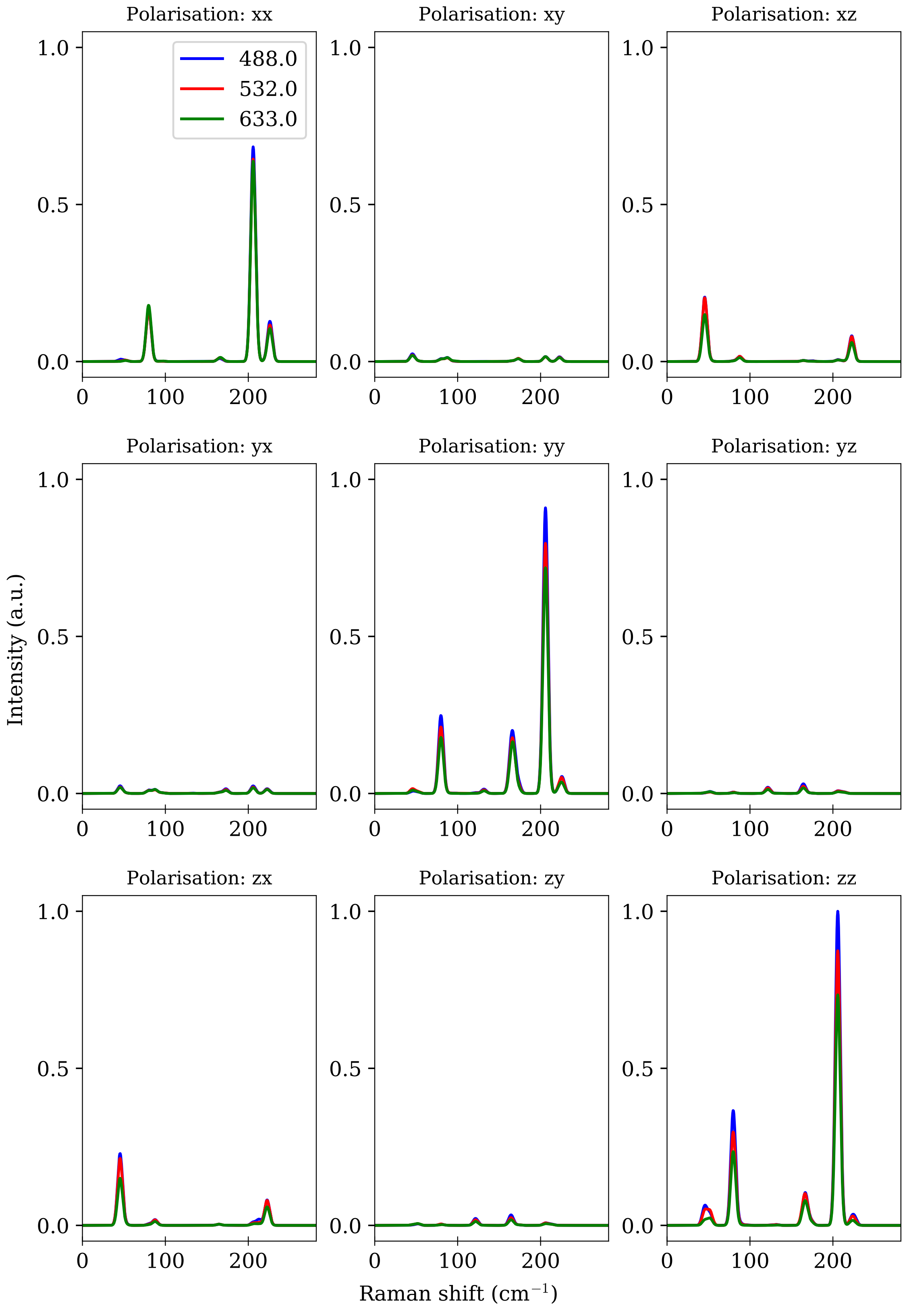

Supplementary Figure 236: Raman spectrum of $Hf_2Br_2Se_2$ with space group 59 and Wyckoff positions a-b-0.

Hf_2Br_2, 2, i-0

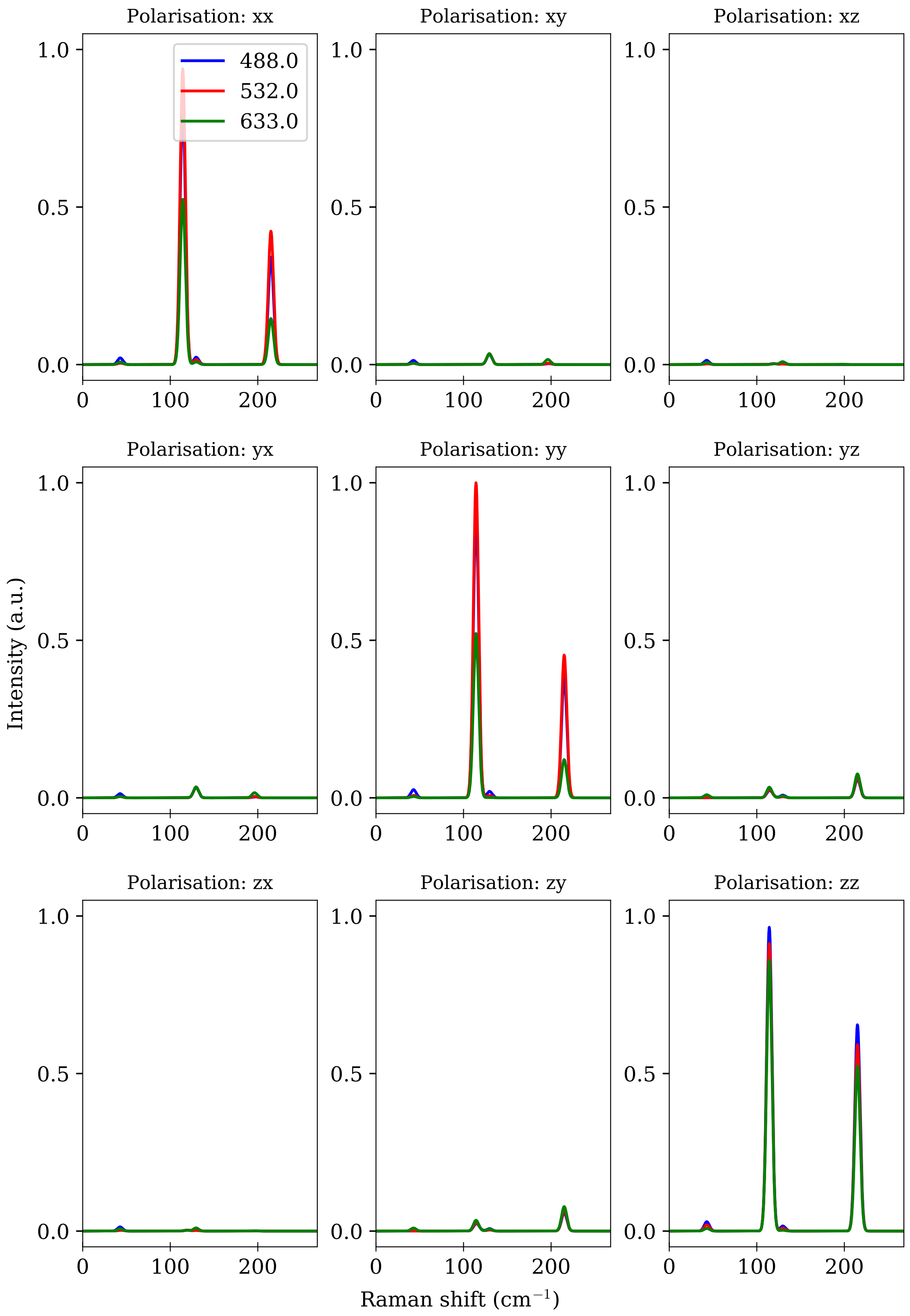

Supplementary Figure 237: Raman spectrum of Hf_2Br_2 with space group 2 and Wyckoff positions i-0.

Hf_2Br_4, 11, e-0

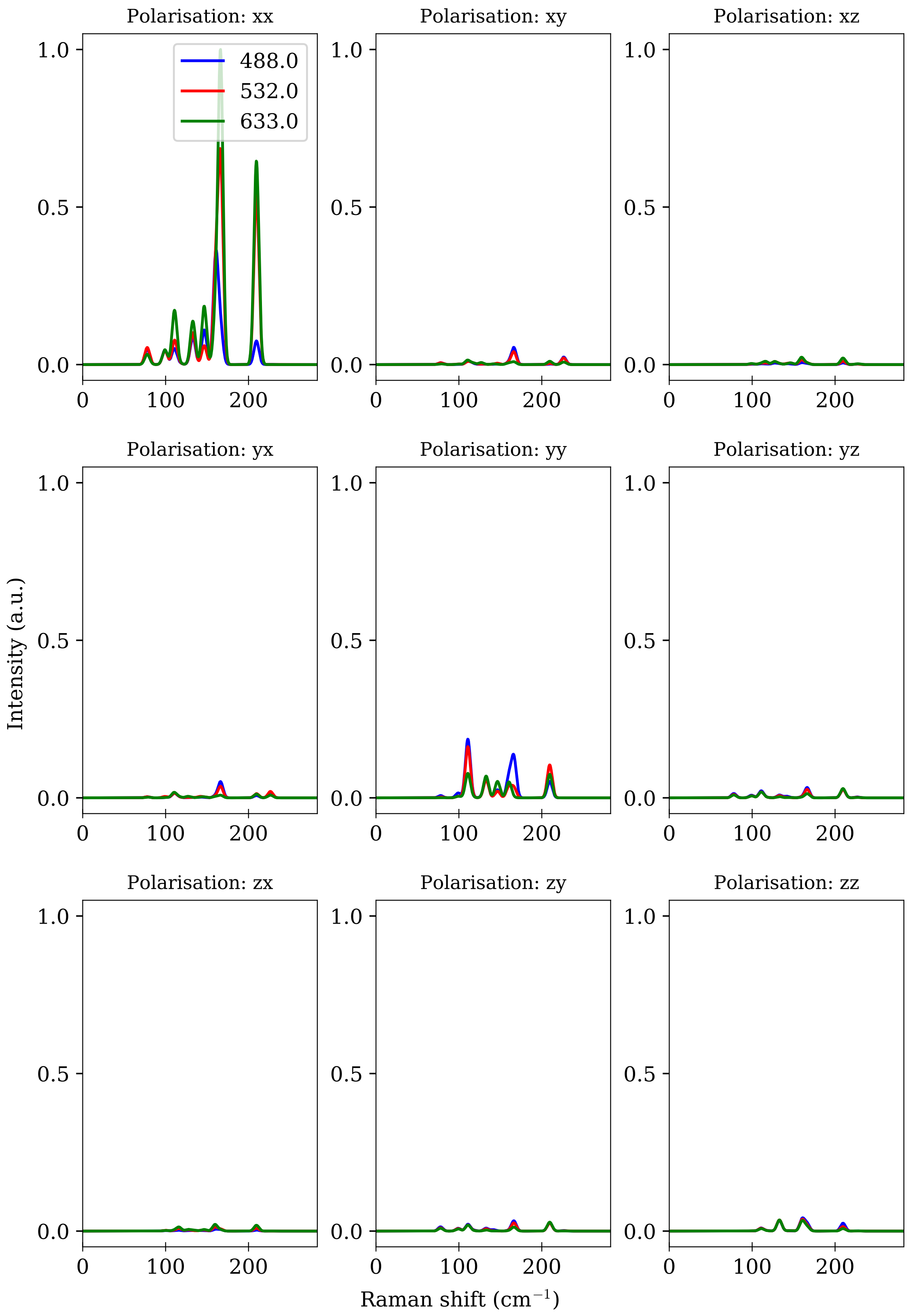

Supplementary Figure 238: Raman spectrum of Hf_2Br_4 with space group 11 and Wyckoff positions e-0.

Hf_2CF_2, 164, b-d-0

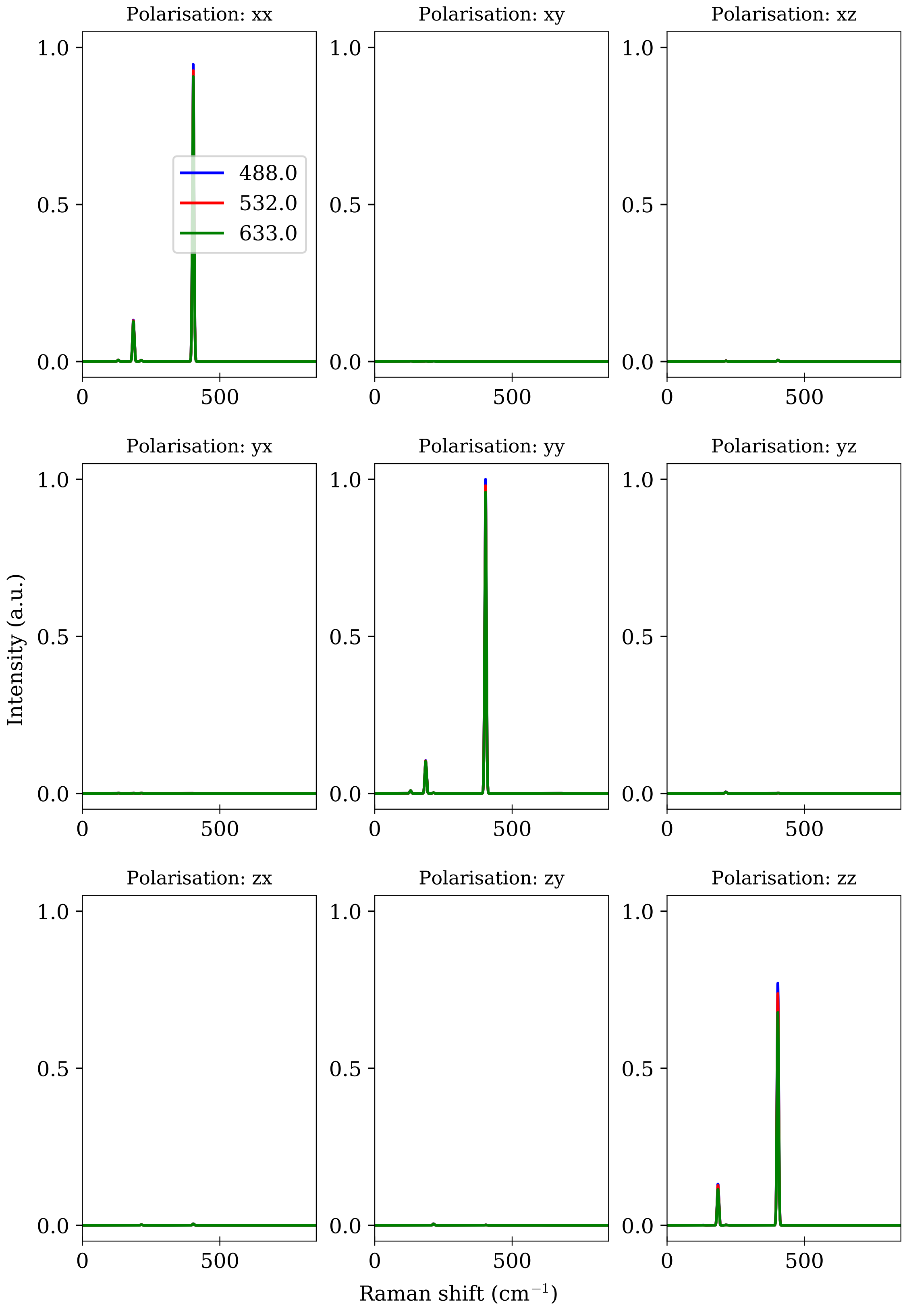

Supplementary Figure 239: Raman spectrum of Hf_2CF_2 with space group 164 and Wyckoff positions b-d-0.

$Hf_2CH_2O_2$, 164, b-d-0

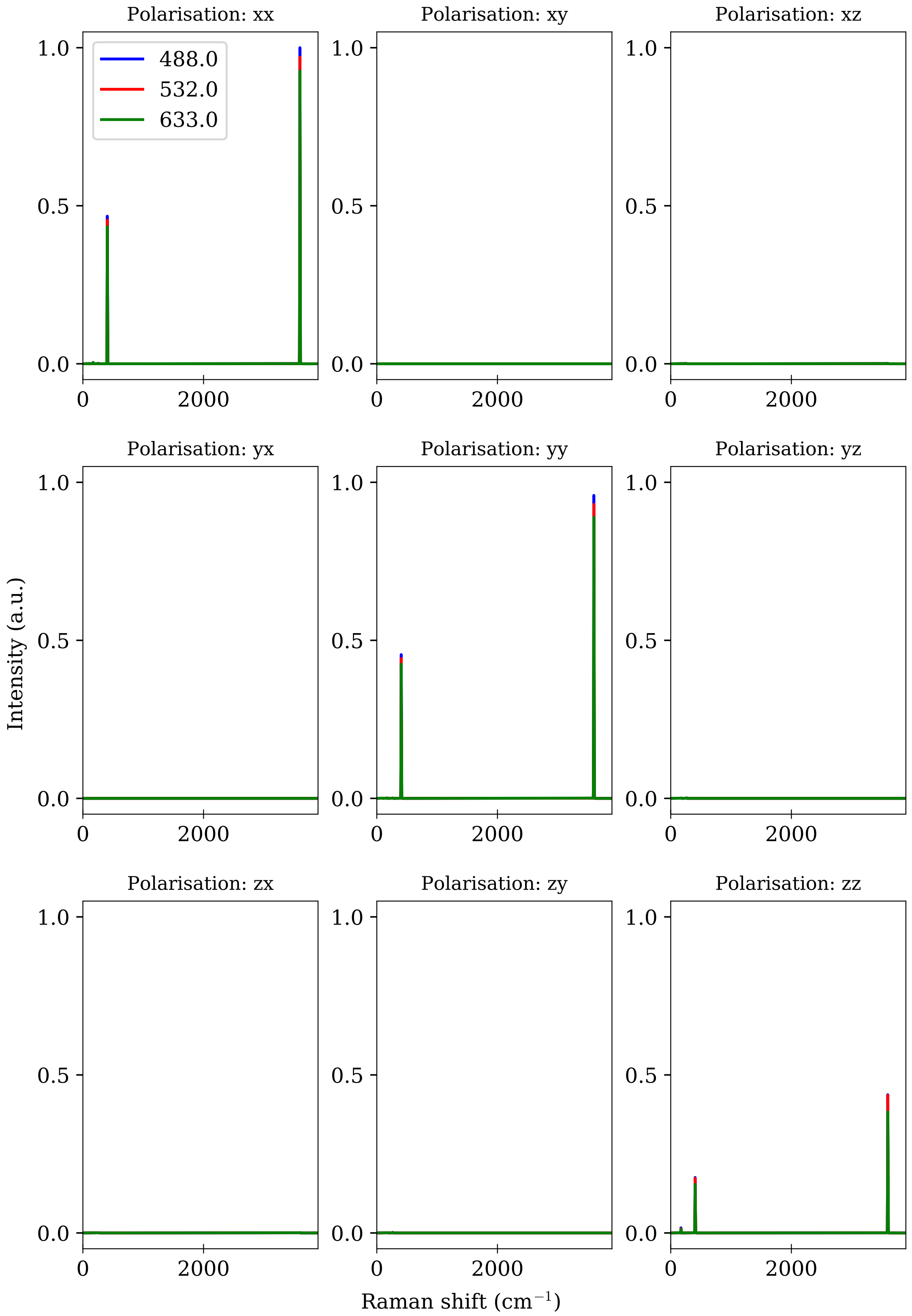

Supplementary Figure 240: Raman spectrum of $Hf_2CH_2O_2$ with space group 164 and Wyckoff positions b-d-0.

$Hf_2Cl_2N_2$, 59, a-b-0

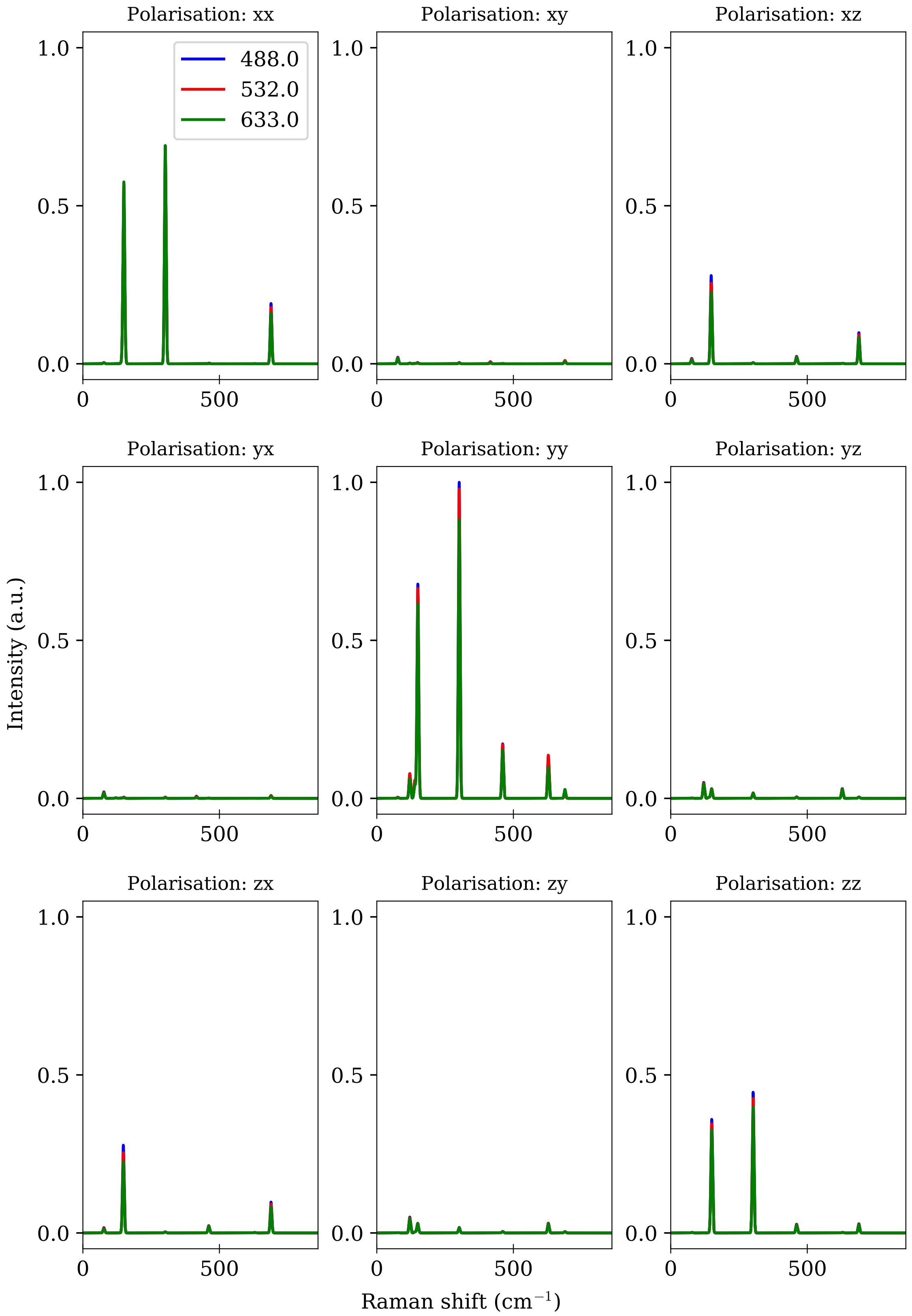

Supplementary Figure 241: Raman spectrum of $Hf_2Cl_2N_2$ with space group 59 and Wyckoff positions a-b-0.

$Hf_2Cl_2S_2$, 59, a-b-0

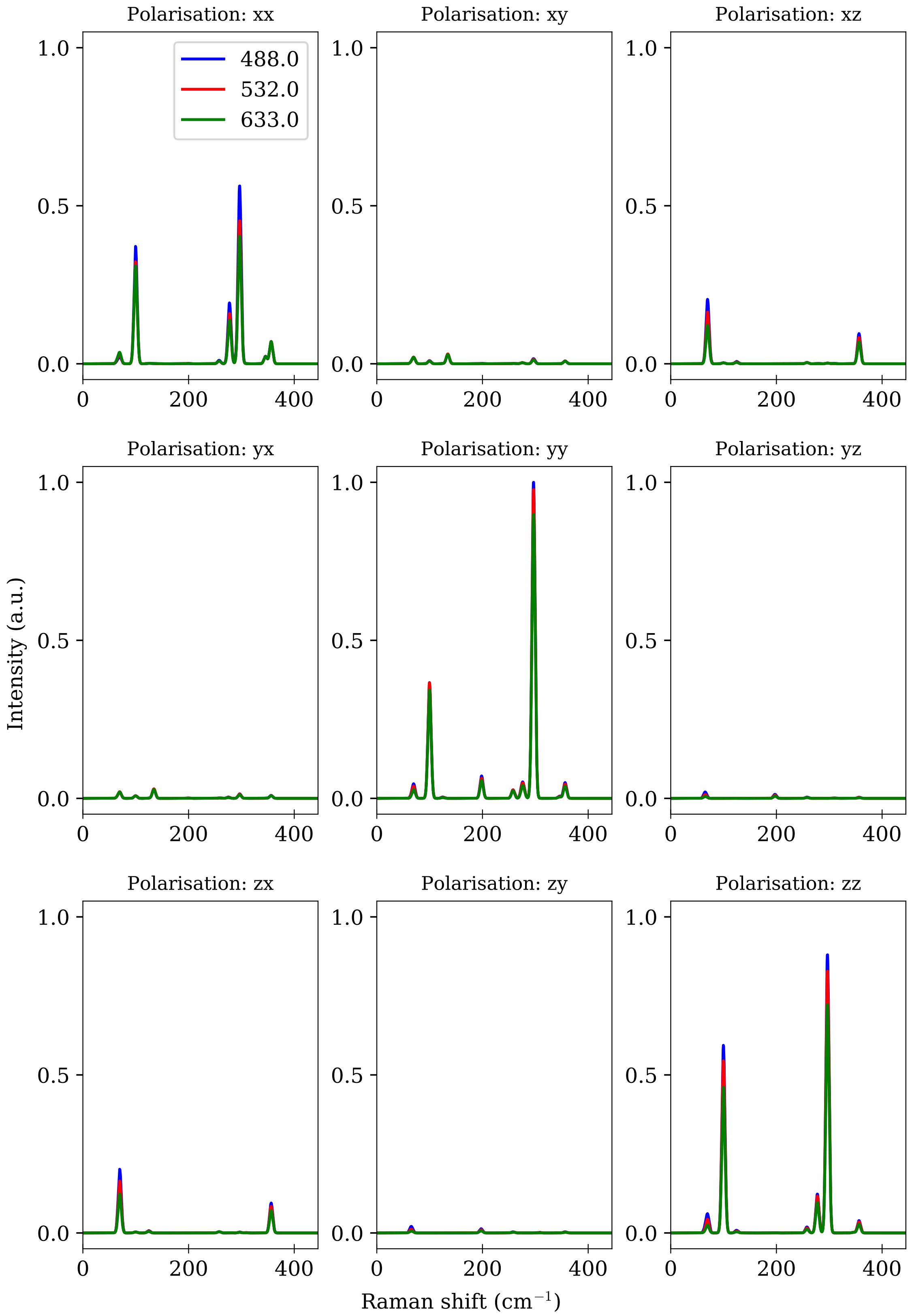

Supplementary Figure 242: Raman spectrum of $Hf_2Cl_2S_2$ with space group 59 and Wyckoff positions a-b-0.

Hf_2Cl_2, 2, i-0

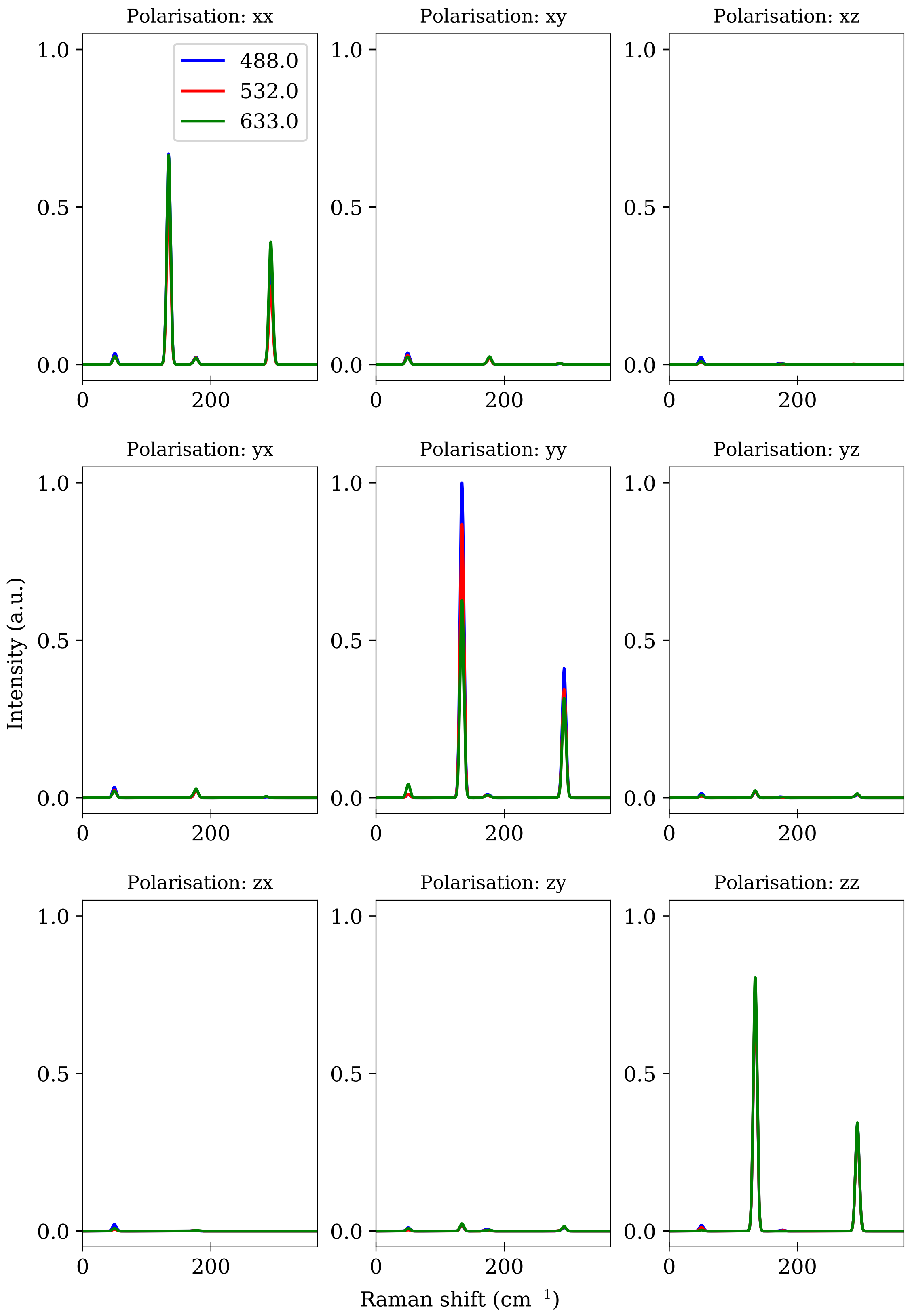

Supplementary Figure 243: Raman spectrum of Hf_2Cl_2 with space group 2 and Wyckoff positions i-0.

Hf_2Cl_4, 11, e-0

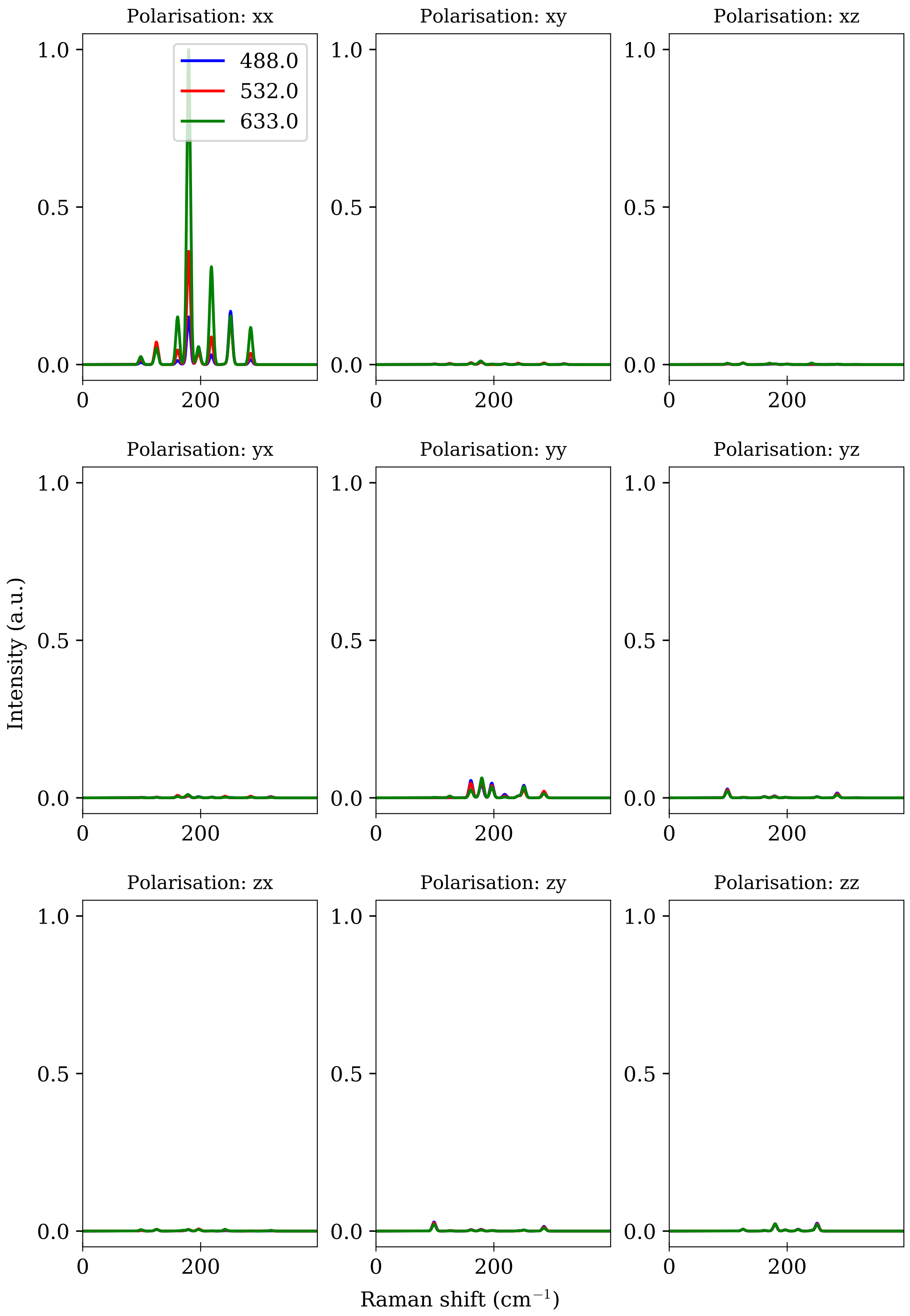

Supplementary Figure 244: Raman spectrum of Hf_2Cl_4 with space group 11 and Wyckoff positions e-0.

Hf_2CO_2, 164, b-d-0

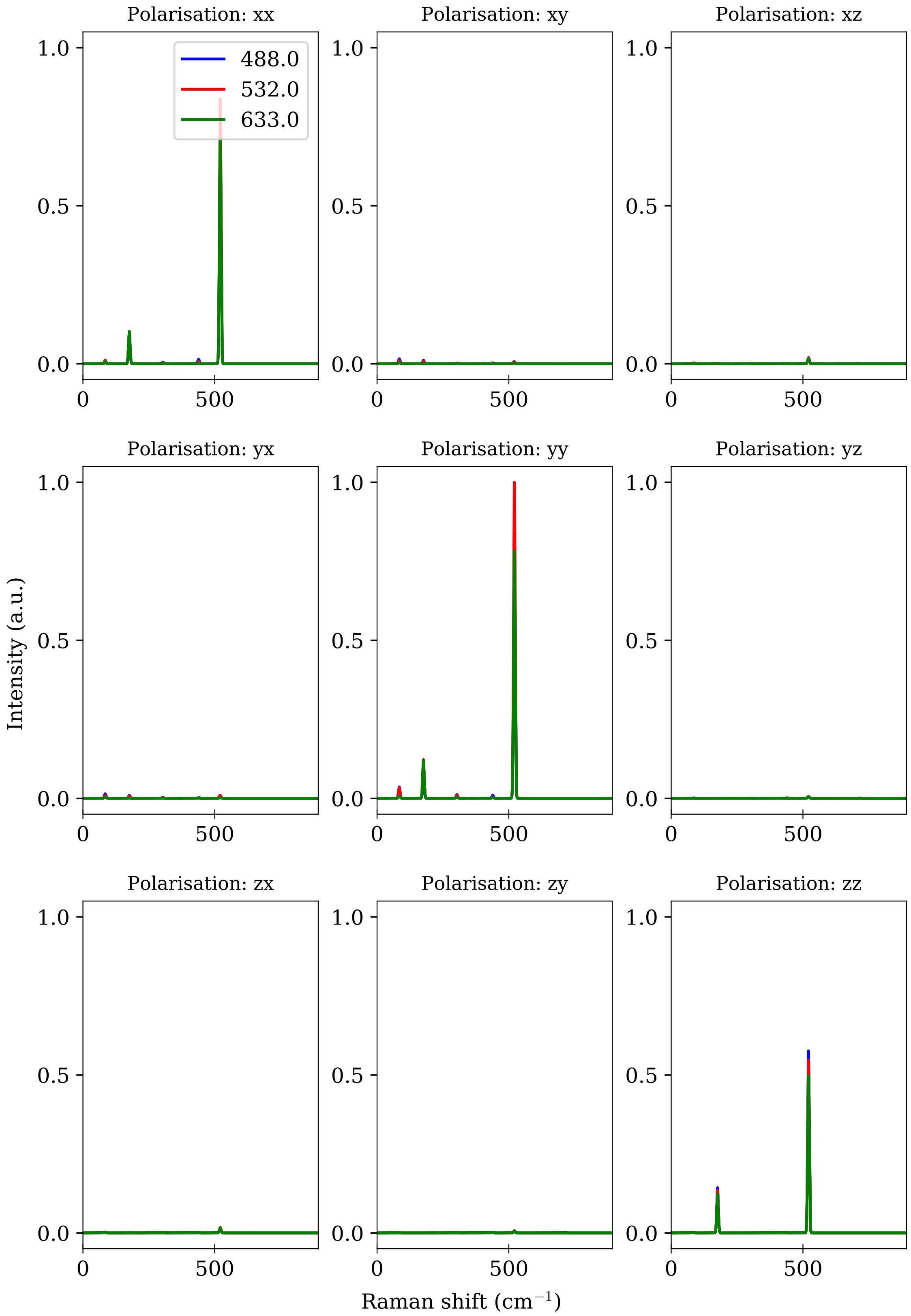

Supplementary Figure 245: Raman spectrum of Hf_2CO_2 with space group 164 and Wyckoff positions b-d-0.

Hf_2C, 164, b-d-0

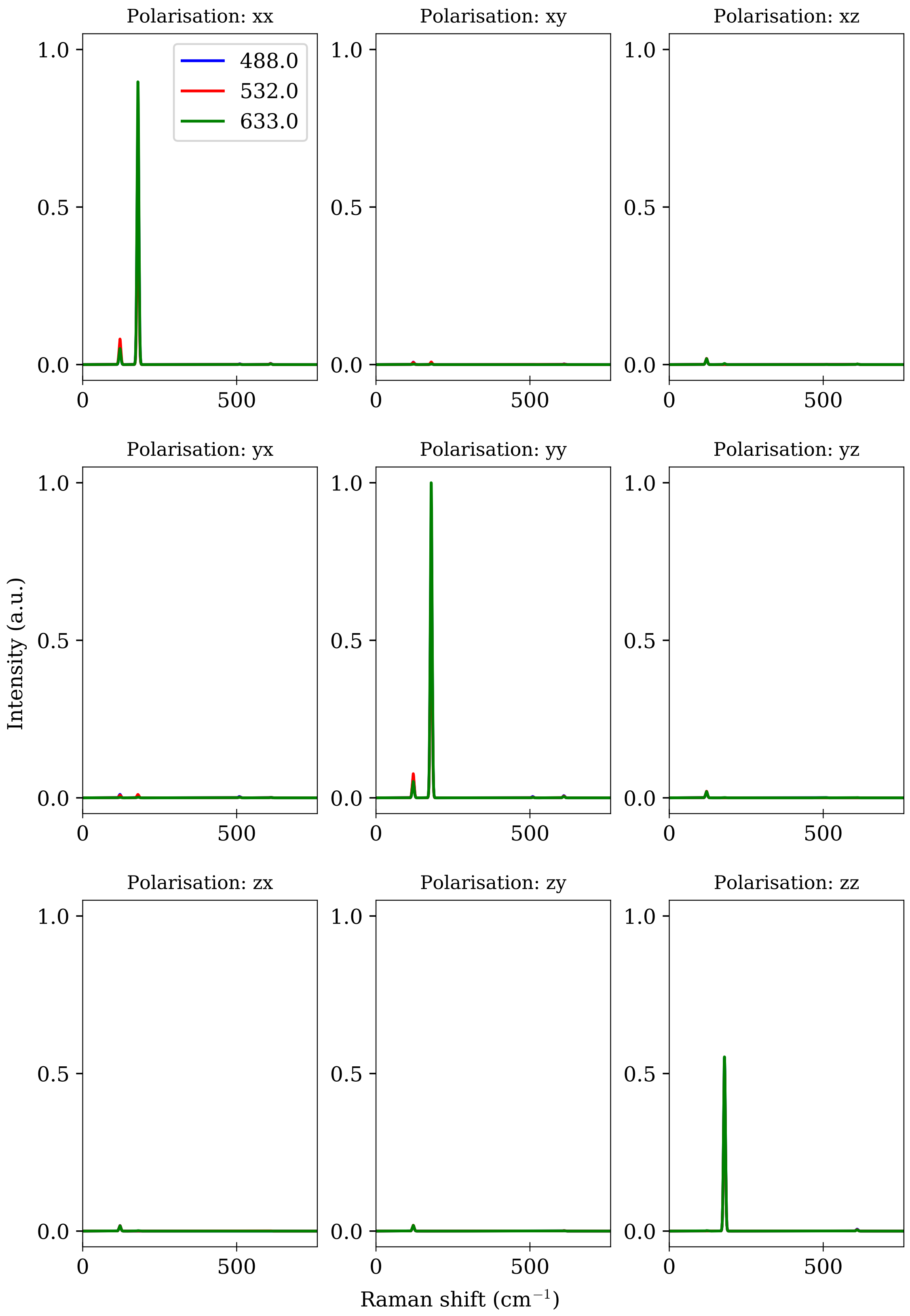

Supplementary Figure 246: Raman spectrum of Hf_2C with space group 164 and Wyckoff positions b-d-0.

Hf_2F_2, 12, i-0

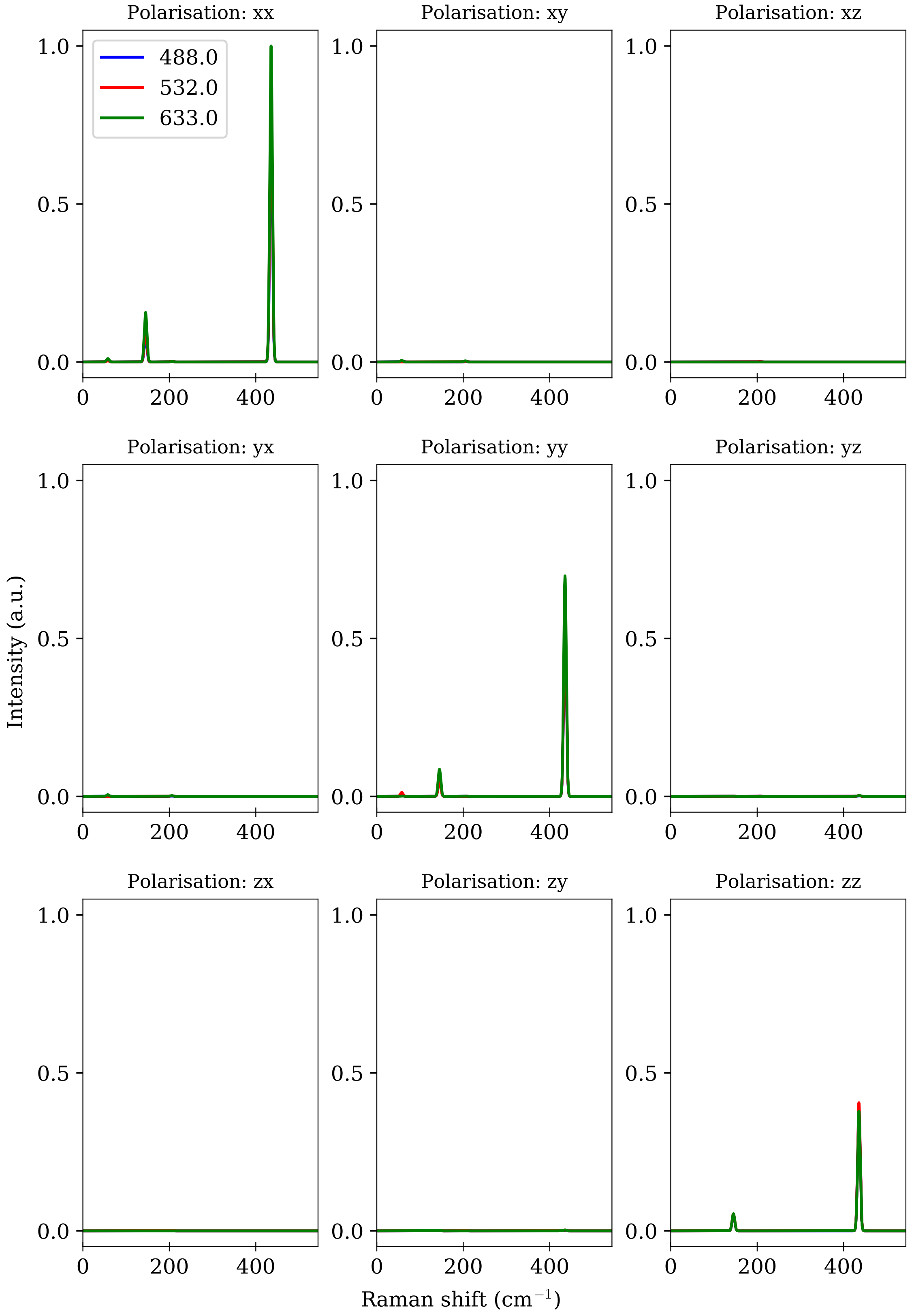

Supplementary Figure 247: Raman spectrum of Hf_2F_2 with space group 12 and Wyckoff positions i-0.

$Hf_2H_2NO_2$, 164, b-d-0

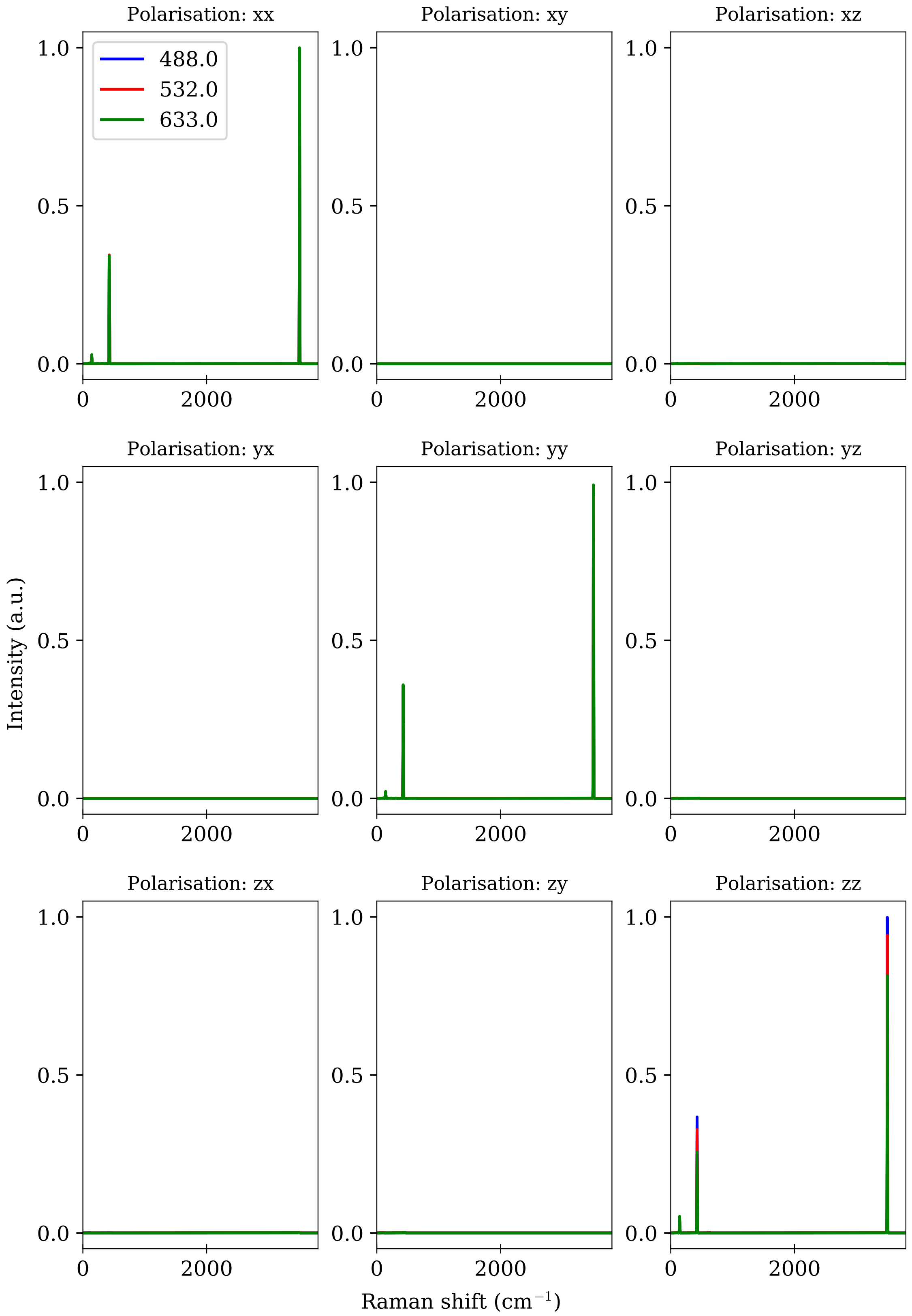

Supplementary Figure 248: Raman spectrum of $Hf_2H_2NO_2$ with space group 164 and Wyckoff positions b-d-0.

Hf_2I_2, 12, i-0

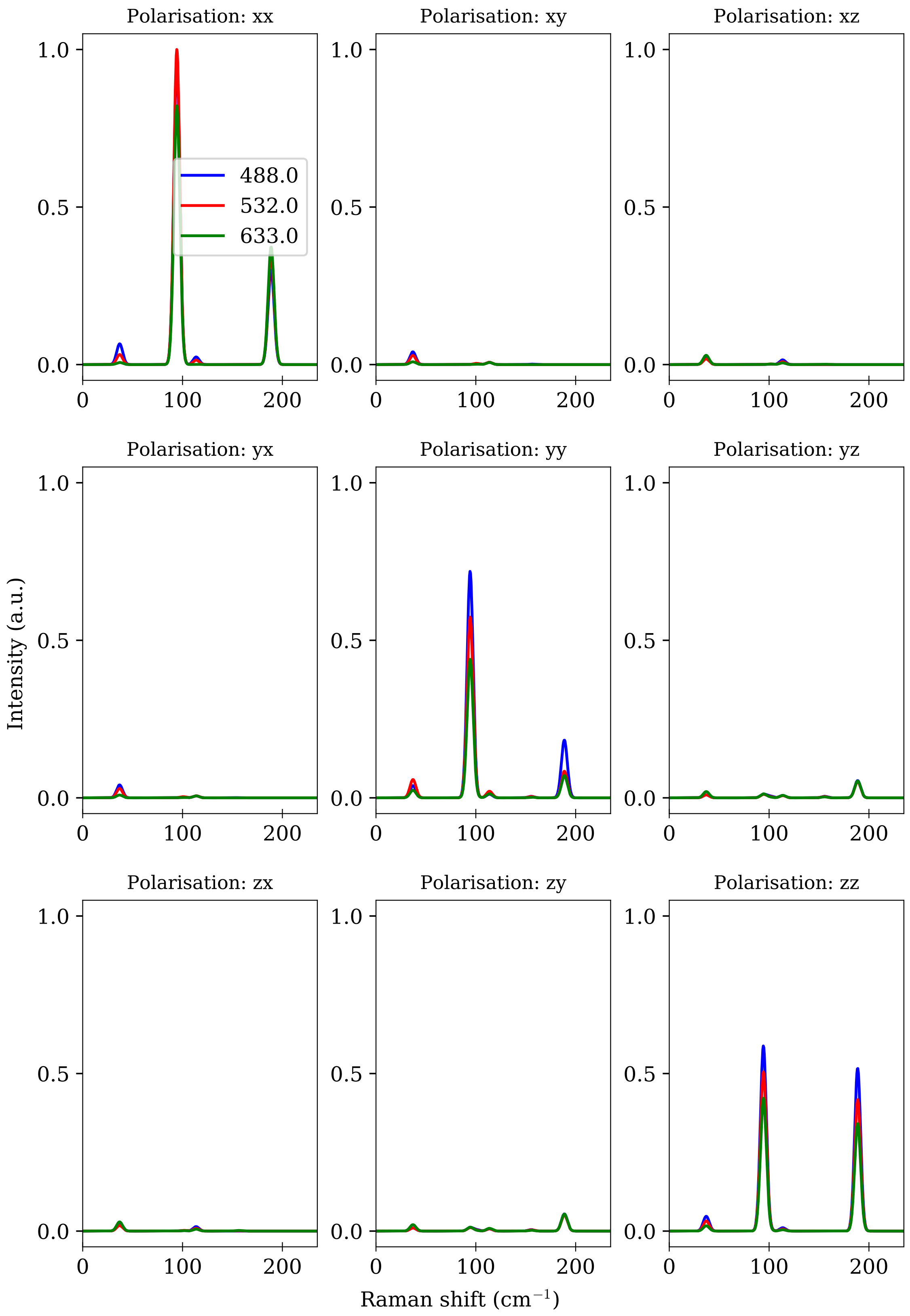

Supplementary Figure 249: Raman spectrum of Hf_2I_2 with space group 12 and Wyckoff positions i-0.

Hf_2NO_2, 164, b-d-0

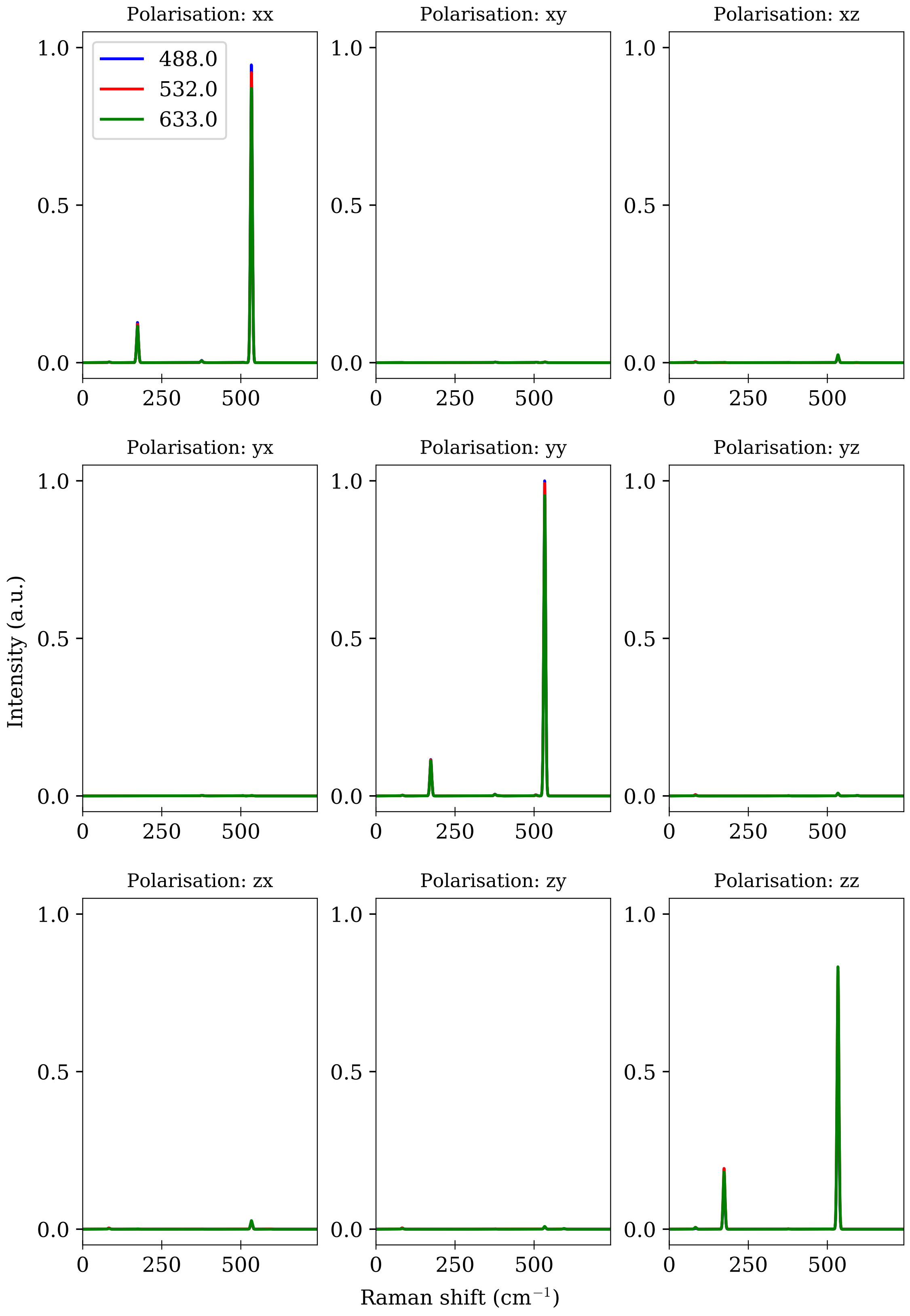

Supplementary Figure 250: Raman spectrum of Hf_2NO_2 with space group 164 and Wyckoff positions b-d-0.

Hf_2N, 164, b-d-0

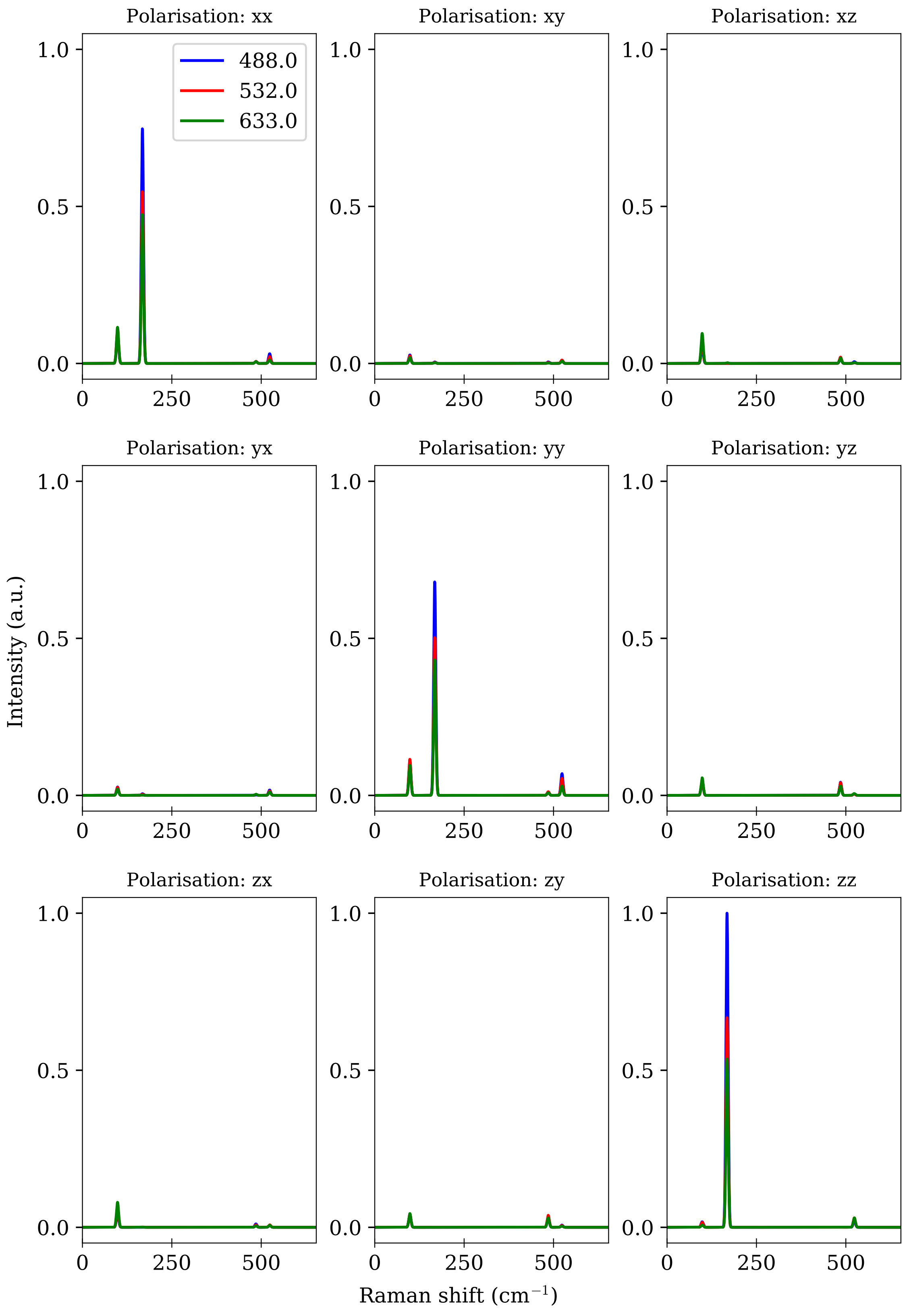

Supplementary Figure 251: Raman spectrum of Hf_2N with space group 164 and Wyckoff positions b-d-0.

Hf_2O_2, 10, b-d-m-0

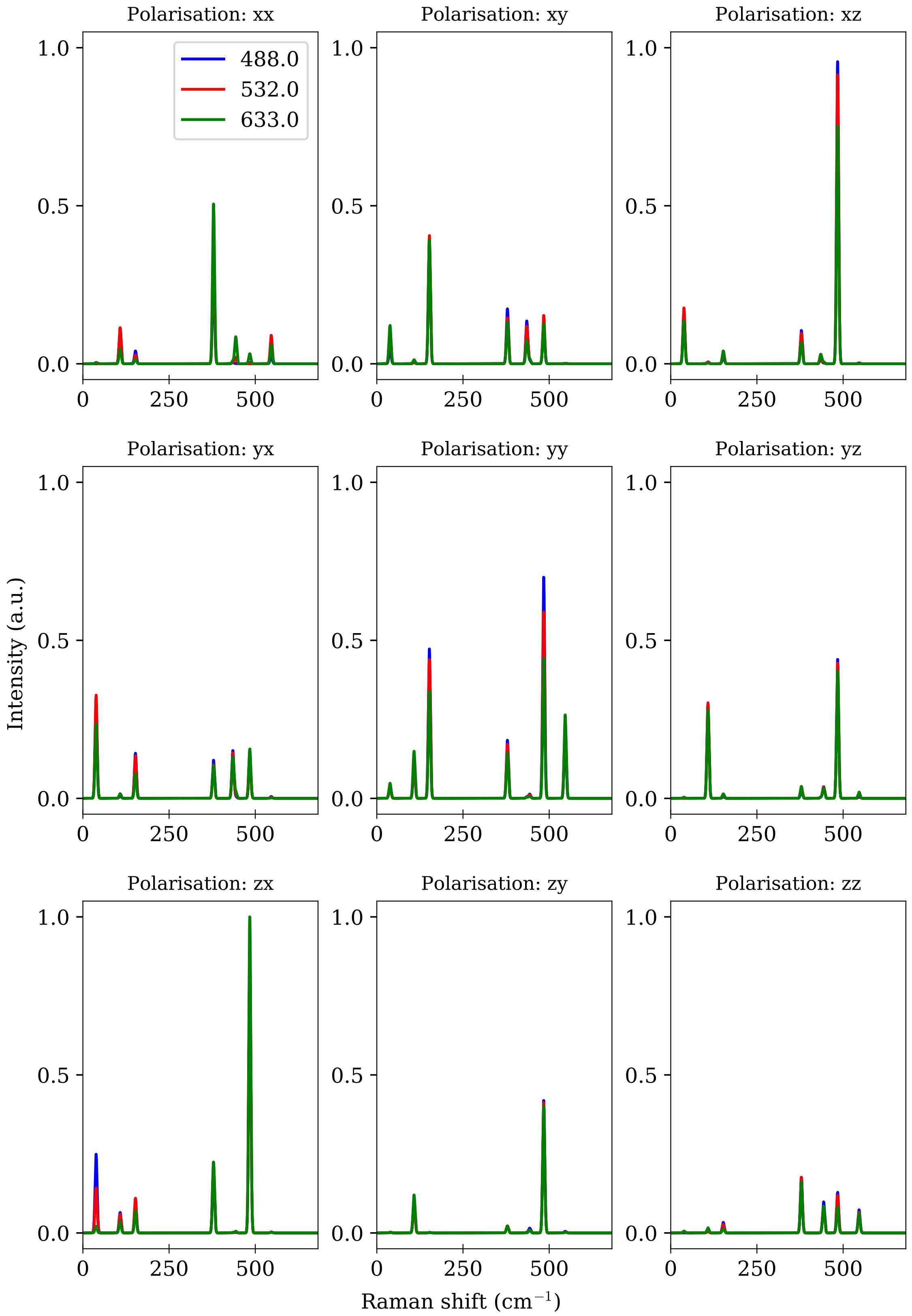

Supplementary Figure 252: Raman spectrum of Hf_2O_2 with space group 10 and Wyckoff positions b-d-m-0.

Hf_2O_6, 59, a-b-e-0

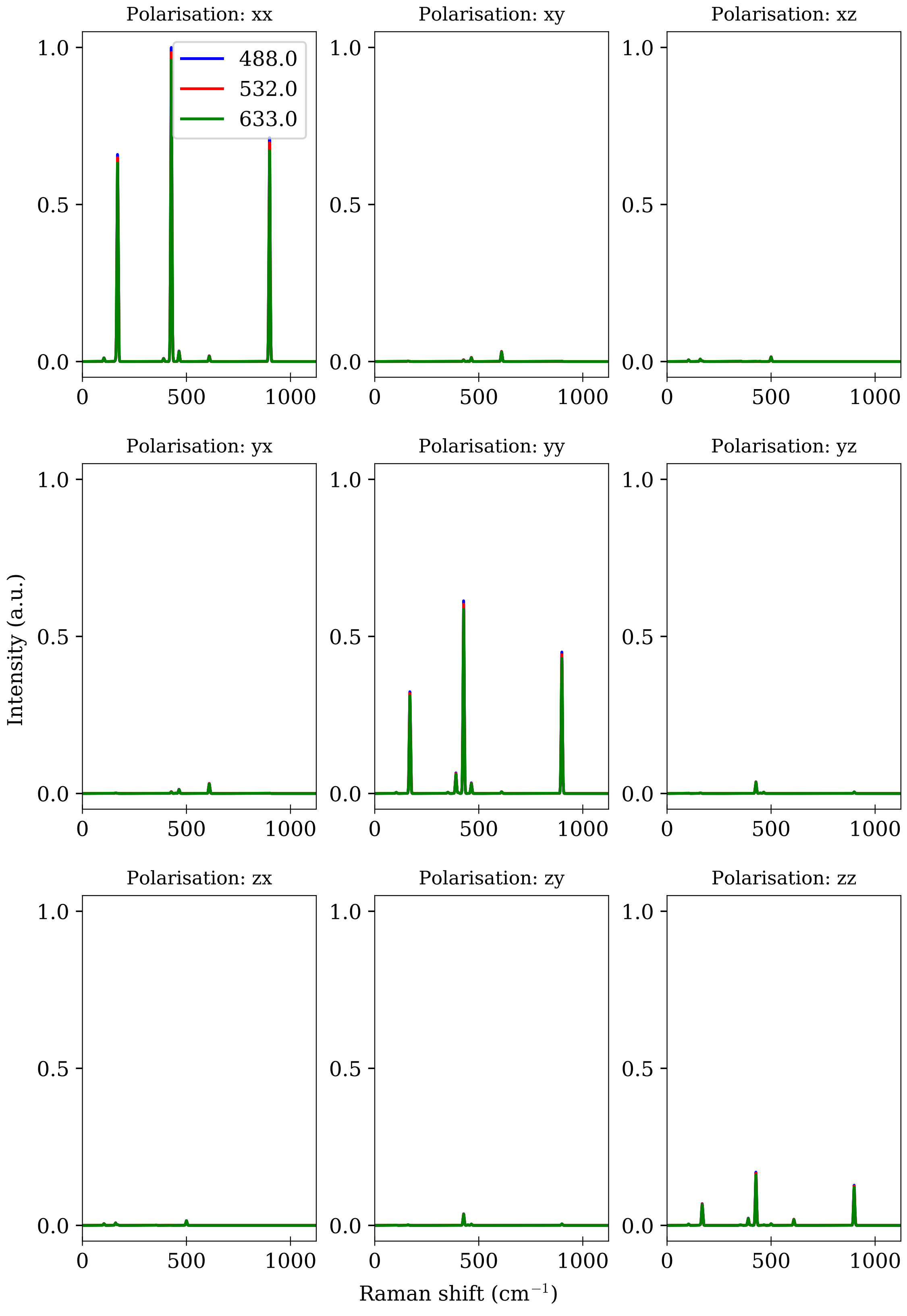

Supplementary Figure 253: Raman spectrum of Hf_2O_6 with space group 59 and Wyckoff positions a-b-e-0.

Hf_2S_2, 12, i-0

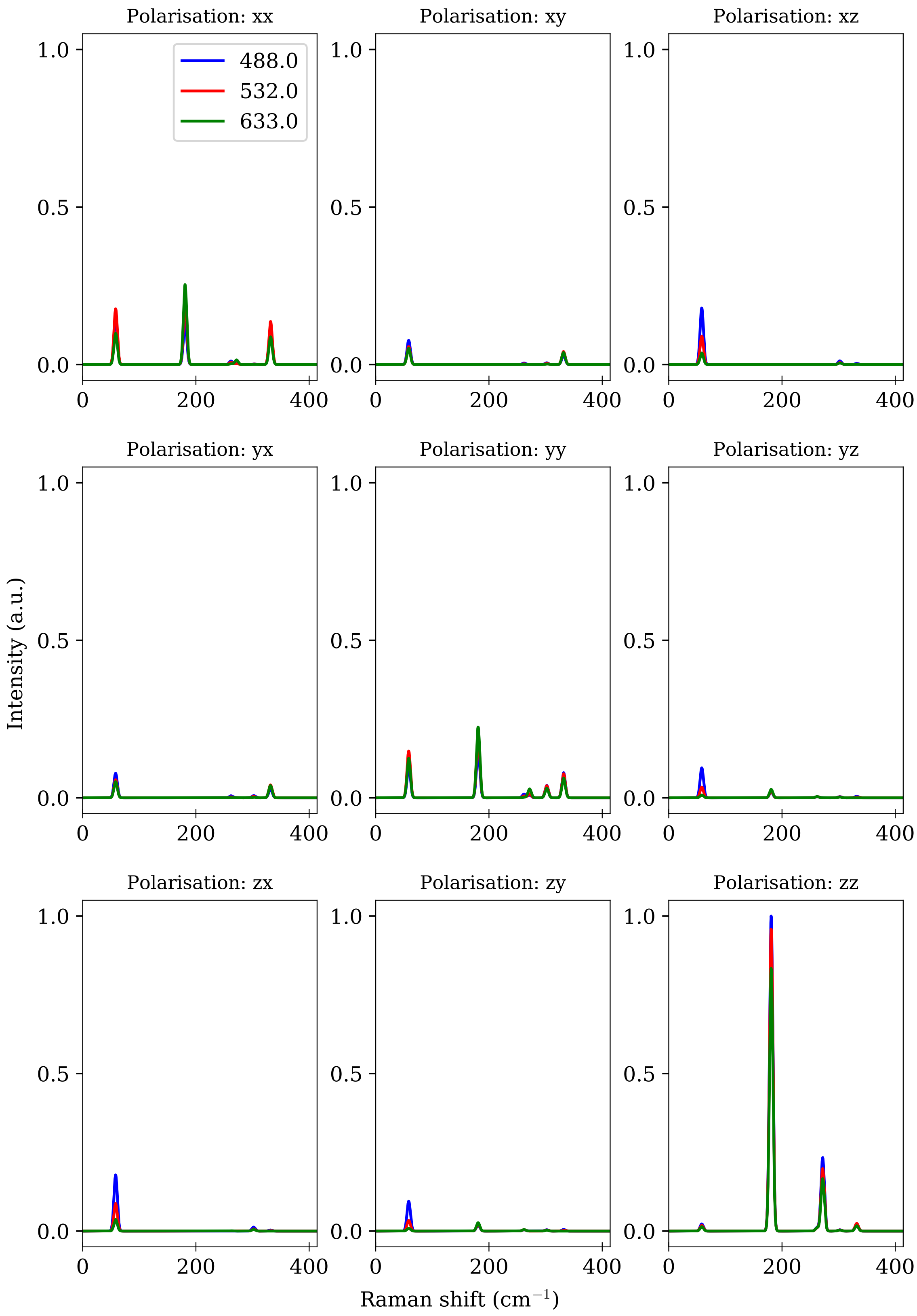

Supplementary Figure 254: Raman spectrum of Hf_2S_2 with space group 12 and Wyckoff positions i-0.

Hf_2Se_2, 12, i-0

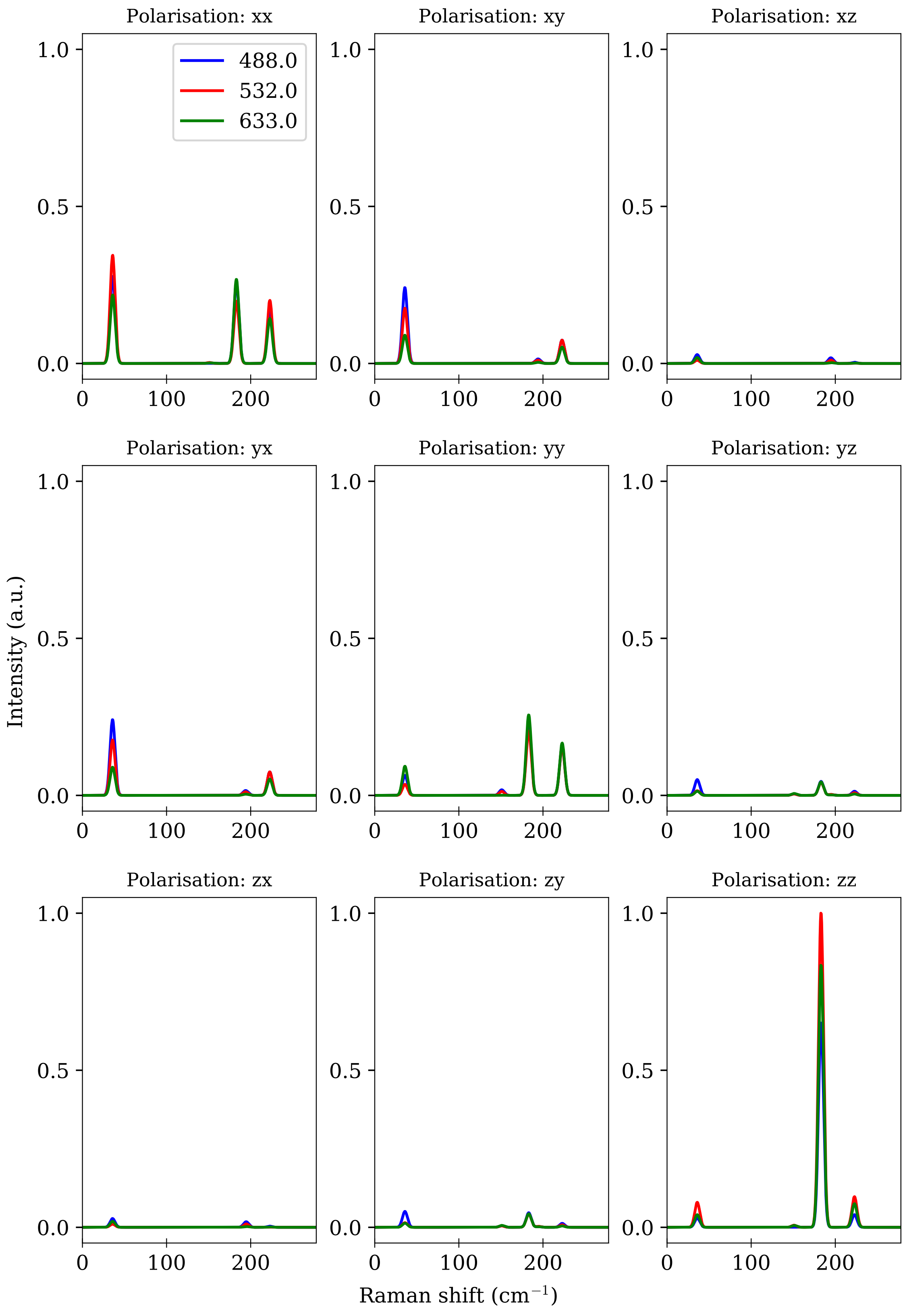

Supplementary Figure 255: Raman spectrum of Hf_2Se_2 with space group 12 and Wyckoff positions i-0.

$Hf_3C_2F_2$, 187, b-g-h-i-0

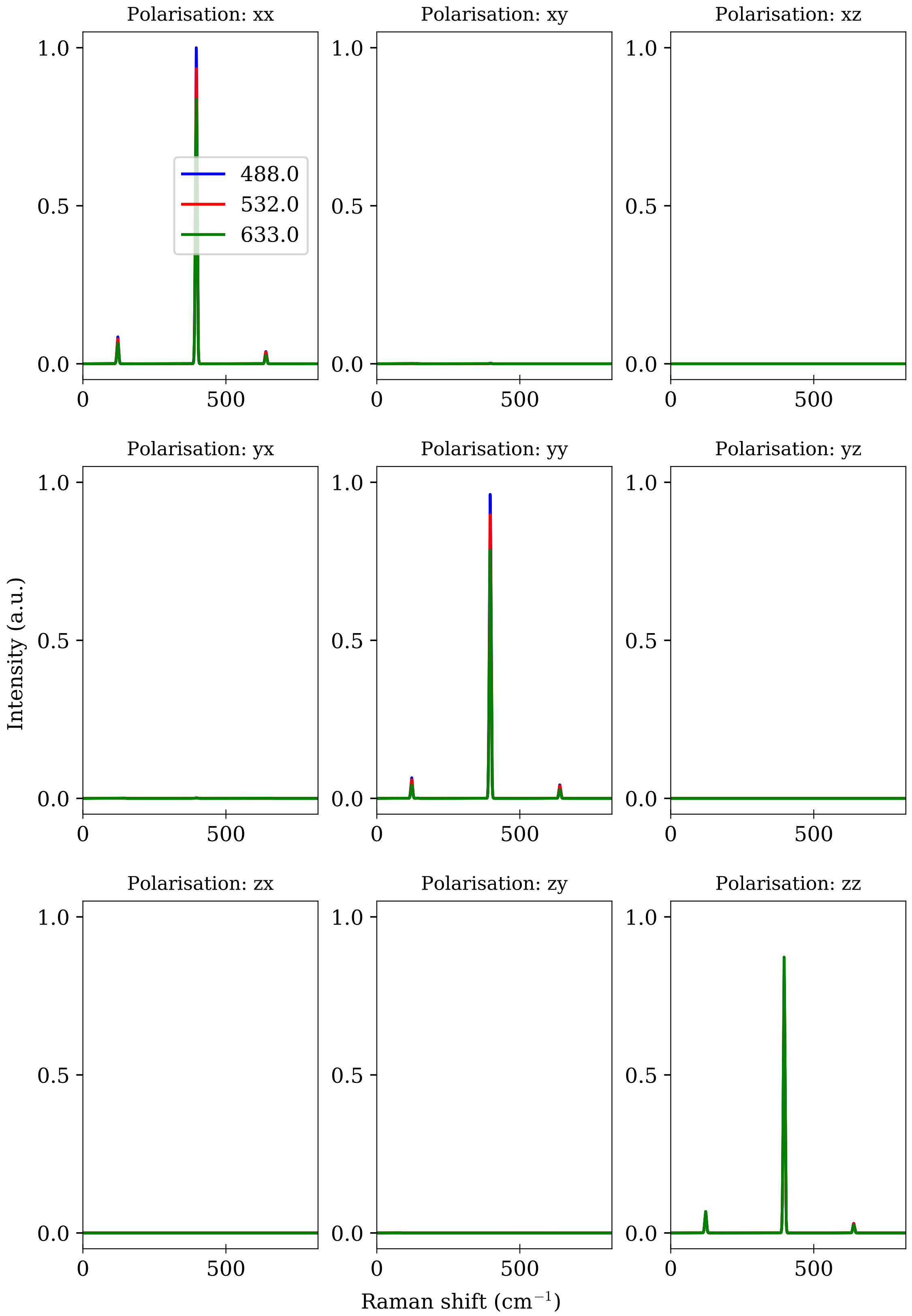

Supplementary Figure 256: Raman spectrum of $Hf_3C_2F_2$ with space group 187 and Wyckoff positions b-g-h-i-0.

$Hf_3C_2O_2$, 187, b-g-h-i-0

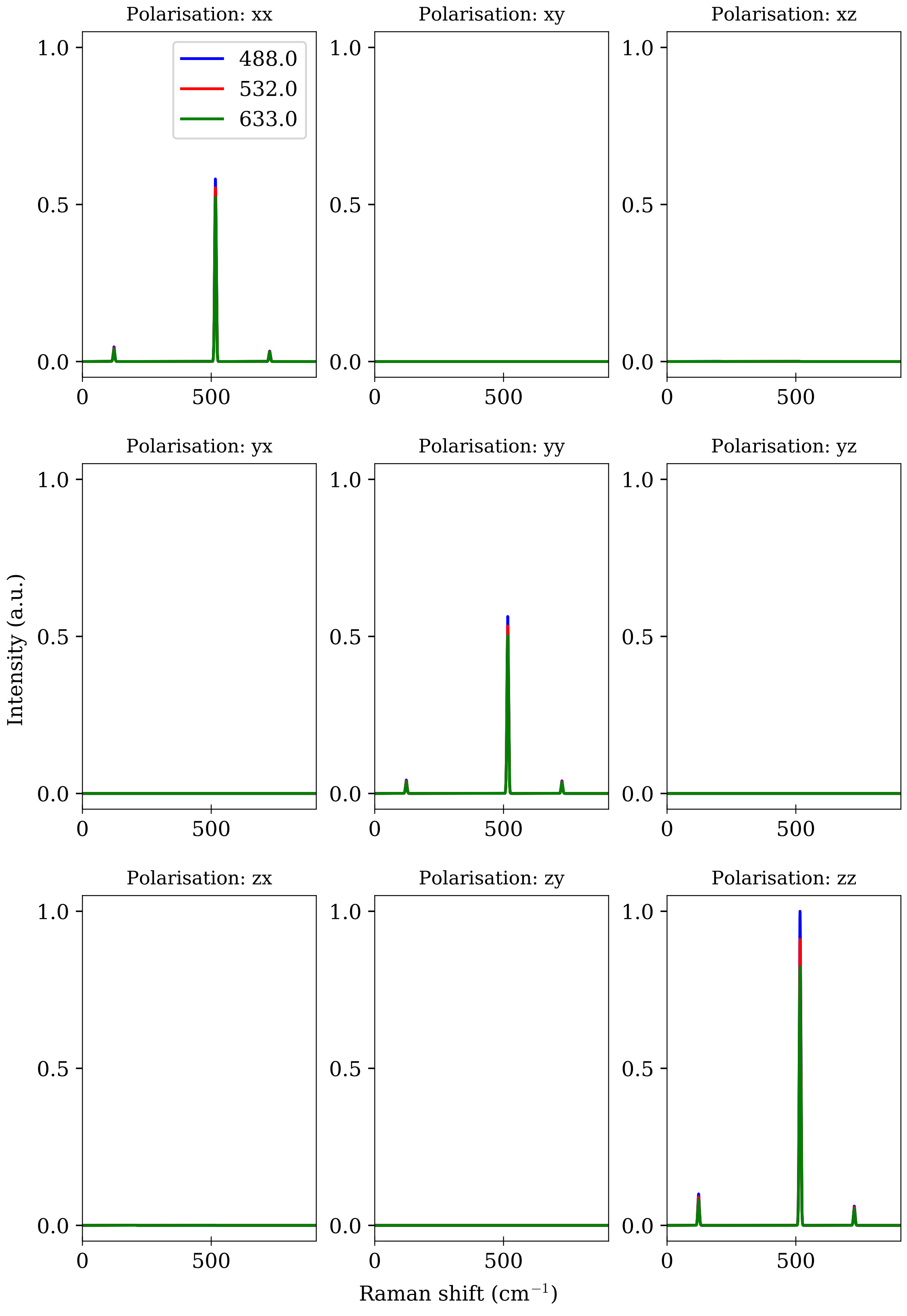

Supplementary Figure 257: Raman spectrum of $Hf_3C_2O_2$ with space group 187 and Wyckoff positions b-g-h-i-0.

$Hf_3N_2O_2$, 187, b-g-h-i-0

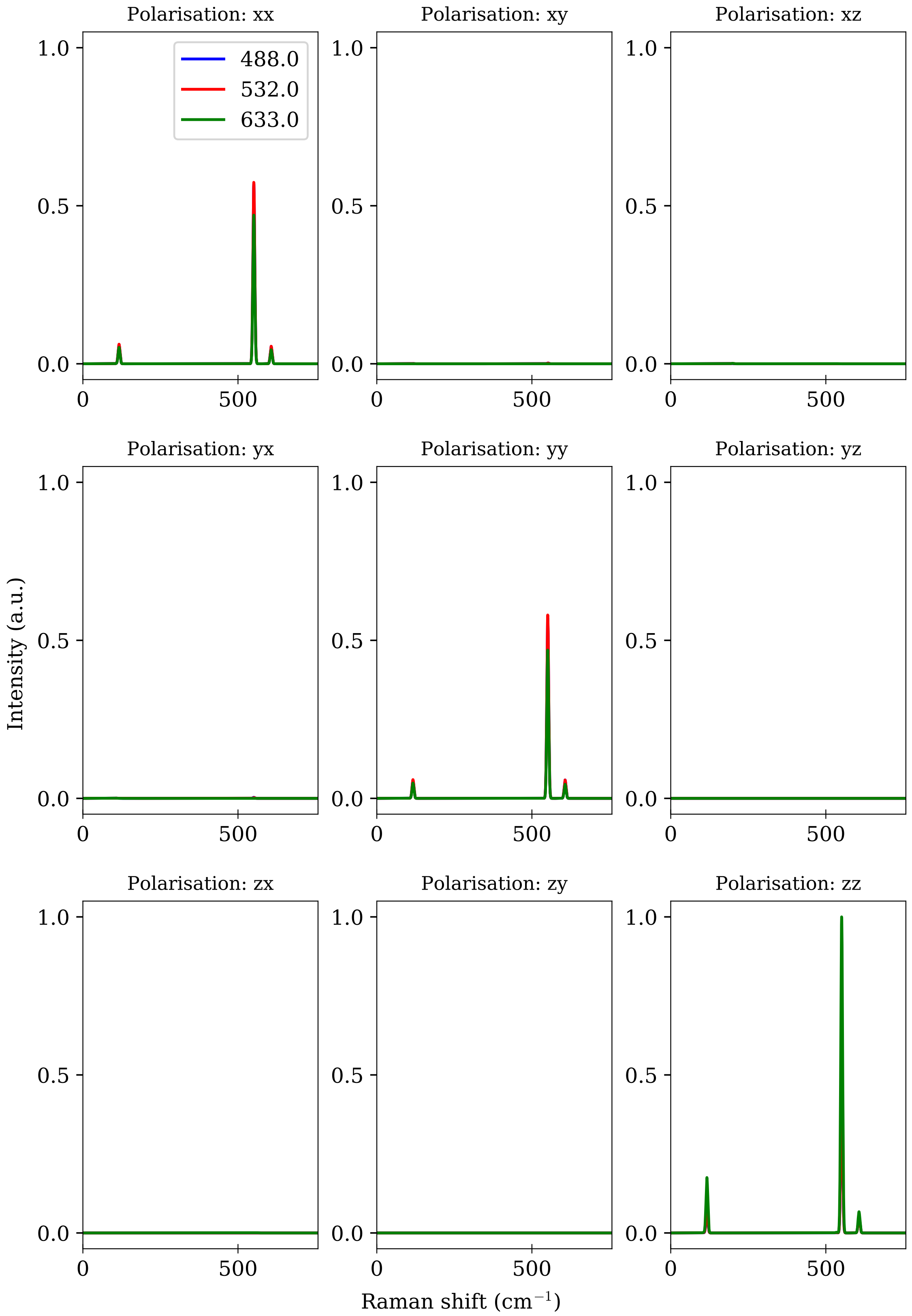

Supplementary Figure 258: Raman spectrum of $Hf_3N_2O_2$ with space group 187 and Wyckoff positions b-g-h-i-0.

Hf_3N_2, 187, a-h-i-0

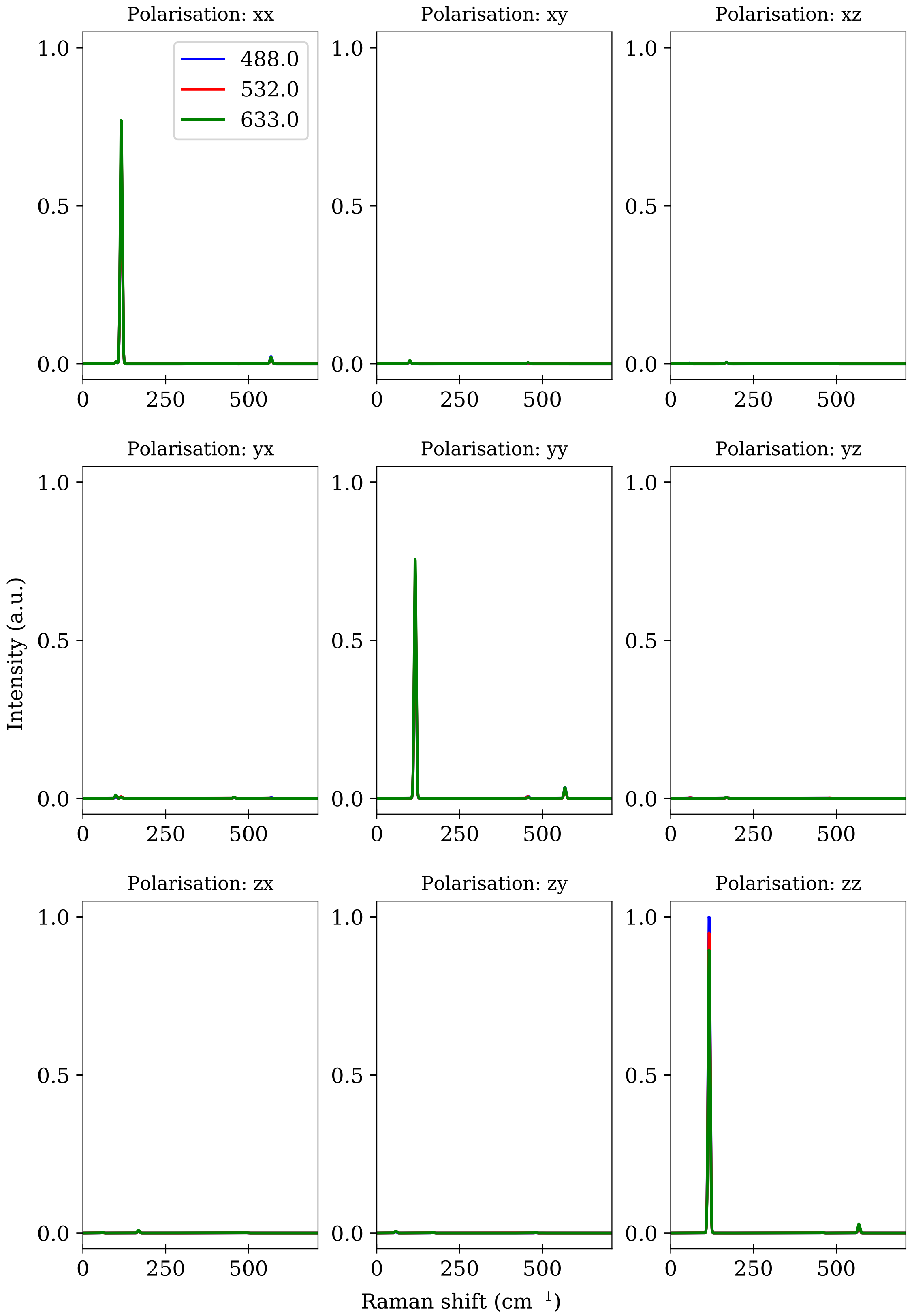

Supplementary Figure 259: Raman spectrum of Hf_3N_2 with space group 187 and Wyckoff positions a-h-i-0.

Hf_4C_3, 164, b-c-d-0

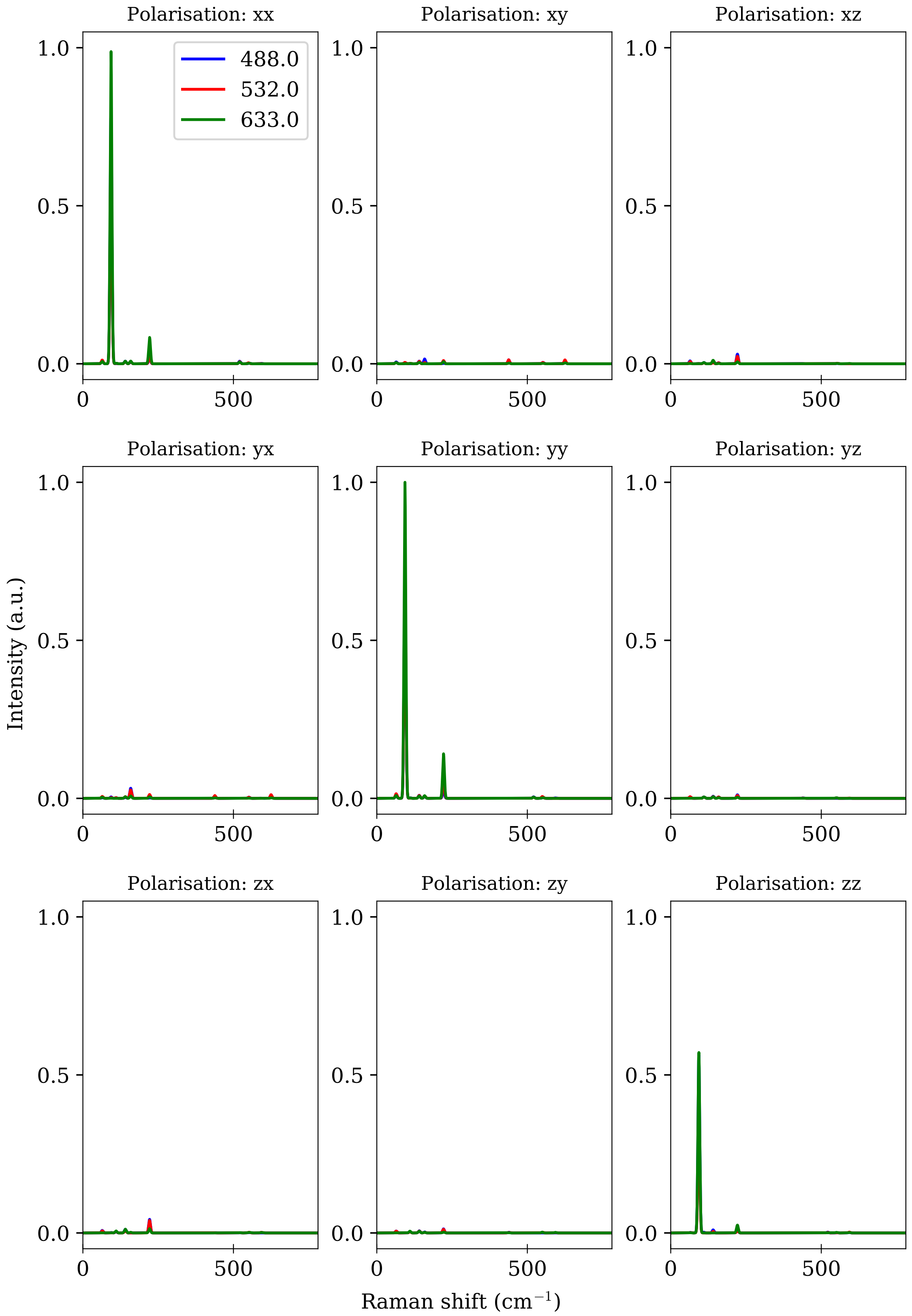

Supplementary Figure 260: Raman spectrum of Hf_4C_3 with space group 164 and Wyckoff positions b-c-d-0.

Hf_4N_3, 164, b-c-d-0

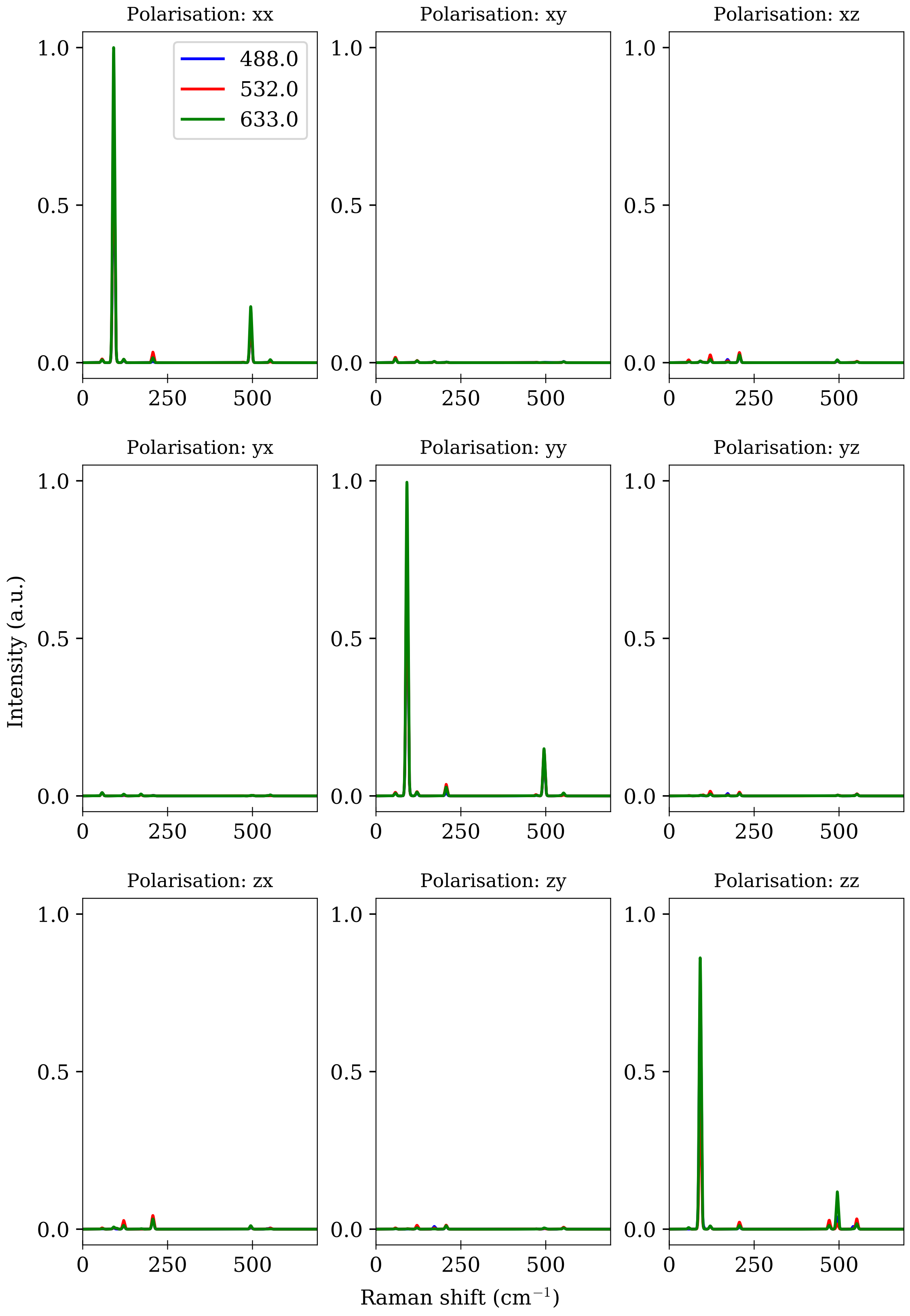

Supplementary Figure 261: Raman spectrum of Hf_4N_3 with space group 164 and Wyckoff positions b-c-d-0.

$HfAs_2$, 164, b-d-0

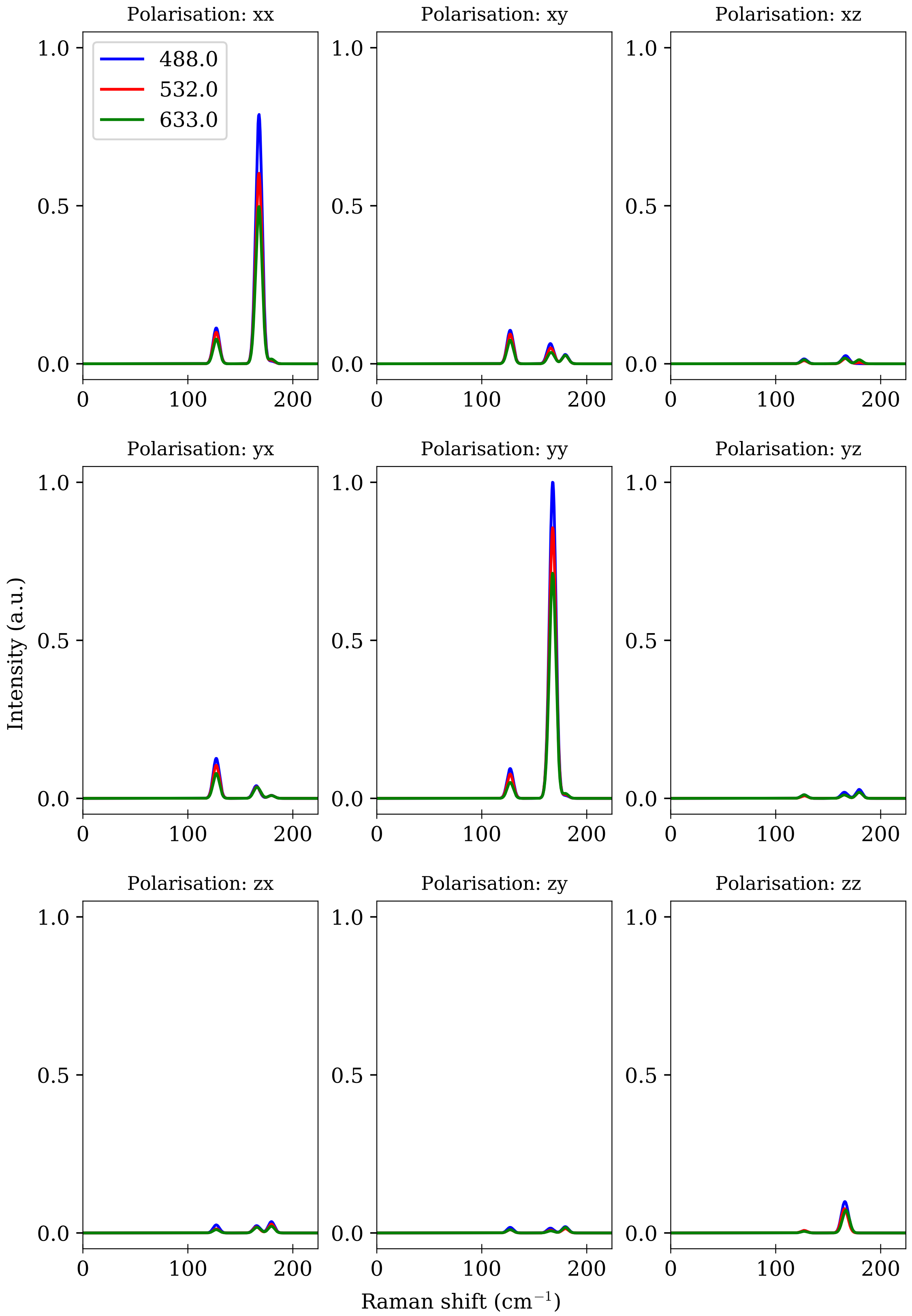

Supplementary Figure 262: Raman spectrum of $HfAs_2$ with space group 164 and Wyckoff positions b-d-0.

$HfBr_2$, 187, b-i-0

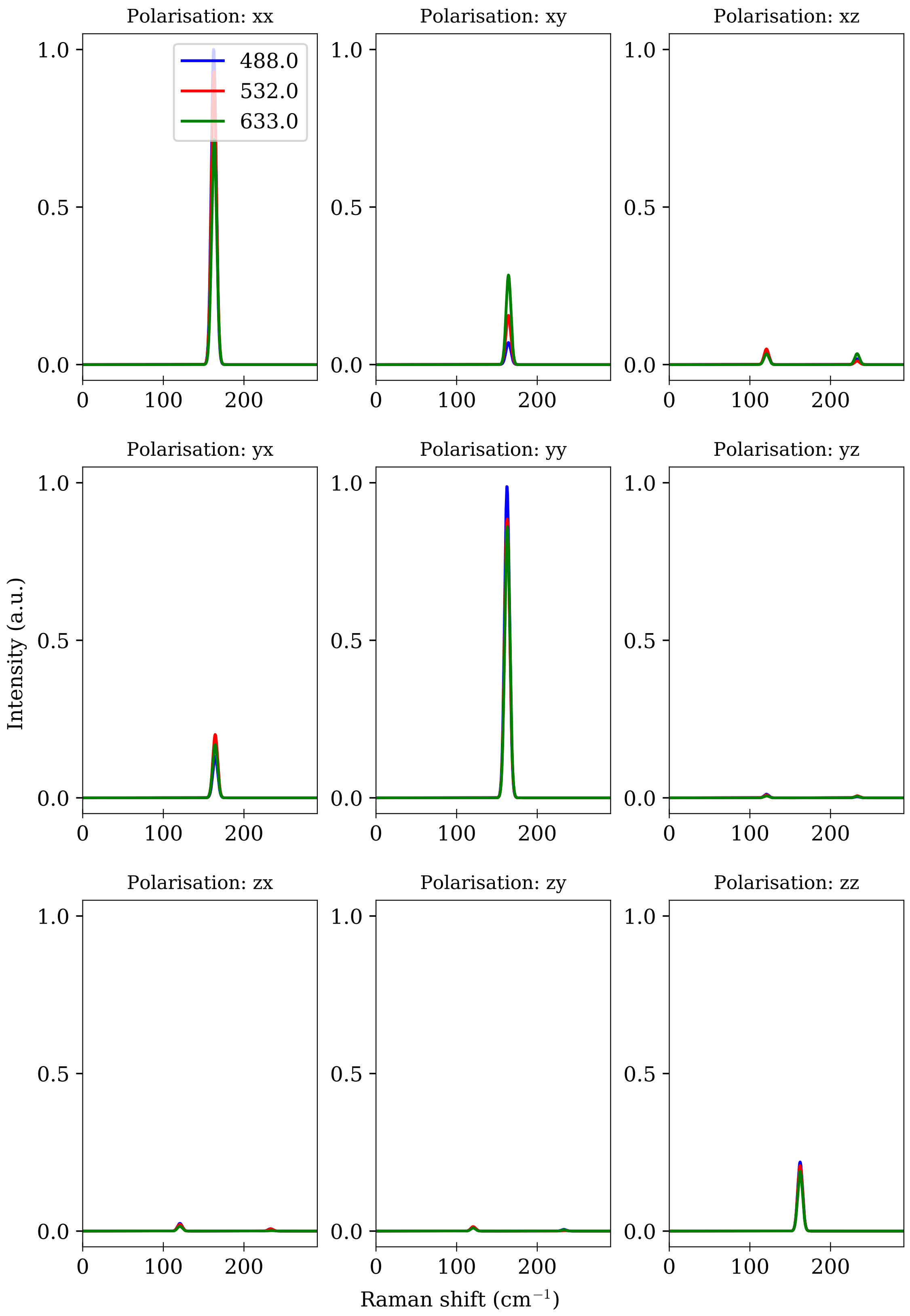

Supplementary Figure 263: Raman spectrum of $HfBr_2$ with space group 187 and Wyckoff positions b-i-0.

HfBrCl, 156, a-c-0

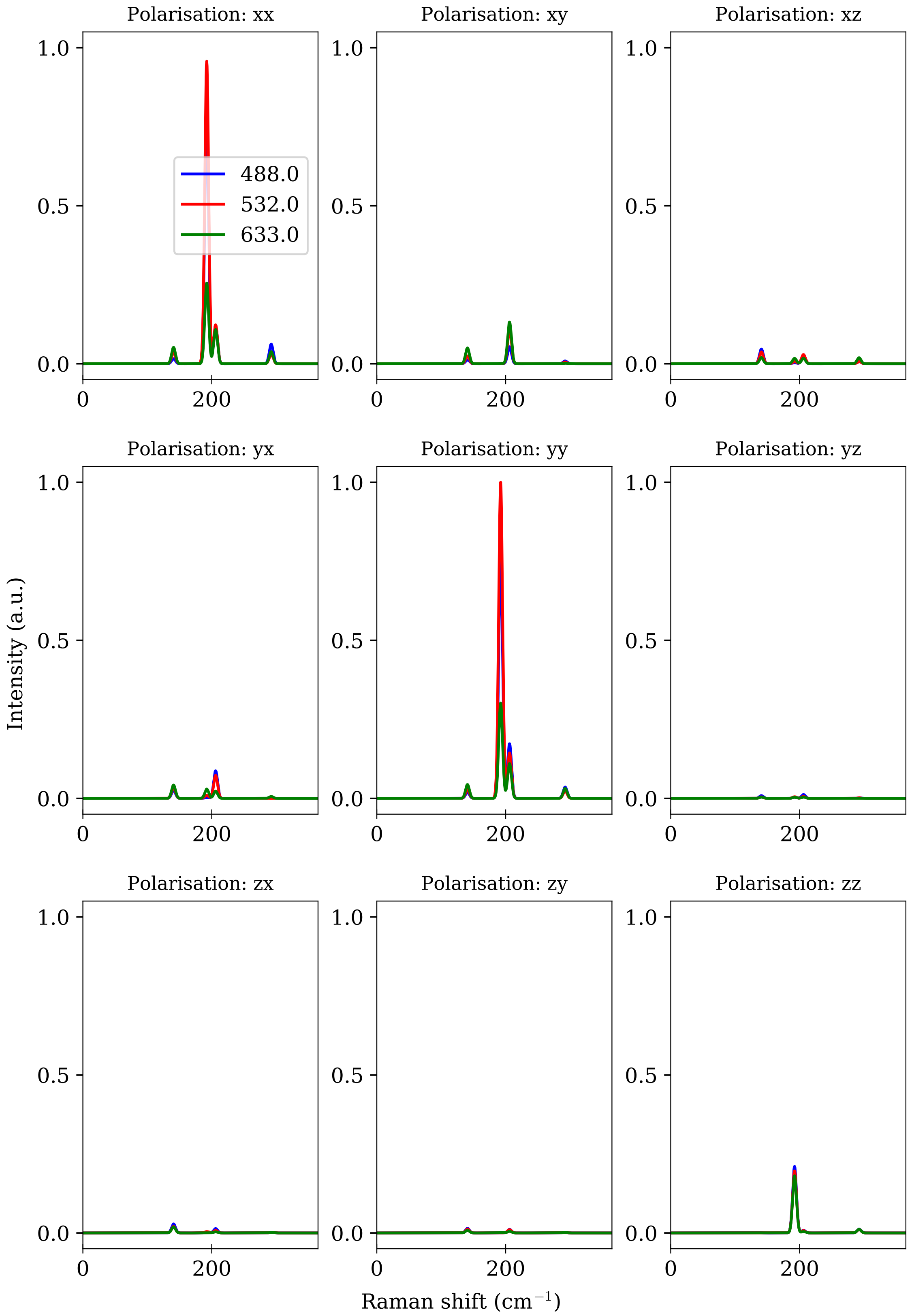

Supplementary Figure 264: Raman spectrum of HfBrCl with space group 156 and Wyckoff positions a-c-0.

HfBrI, 156, a-c-0

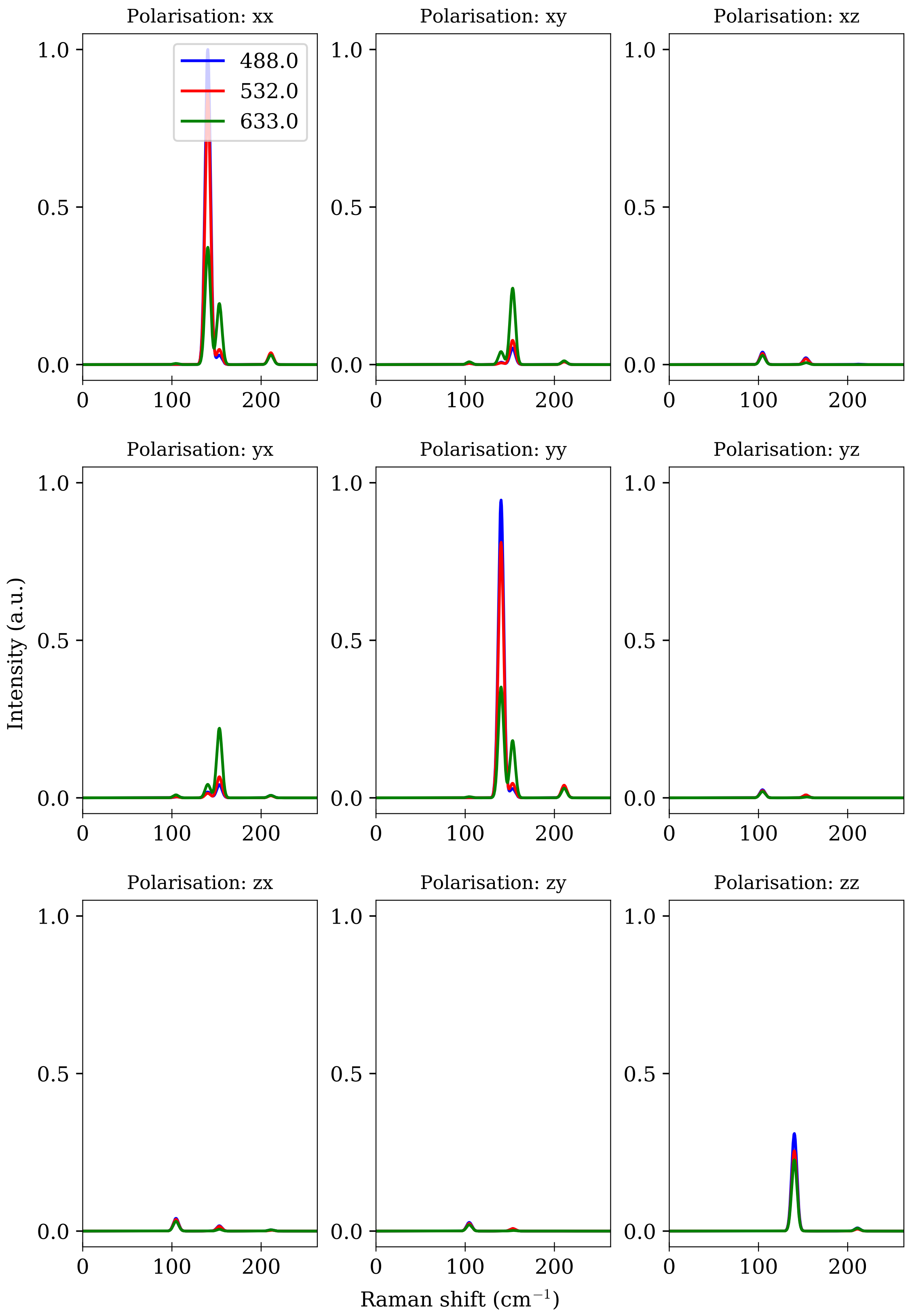

Supplementary Figure 265: Raman spectrum of HfBrI with space group 156 and Wyckoff positions a-c-0.

$HfCl_2$, 187, b-i-0

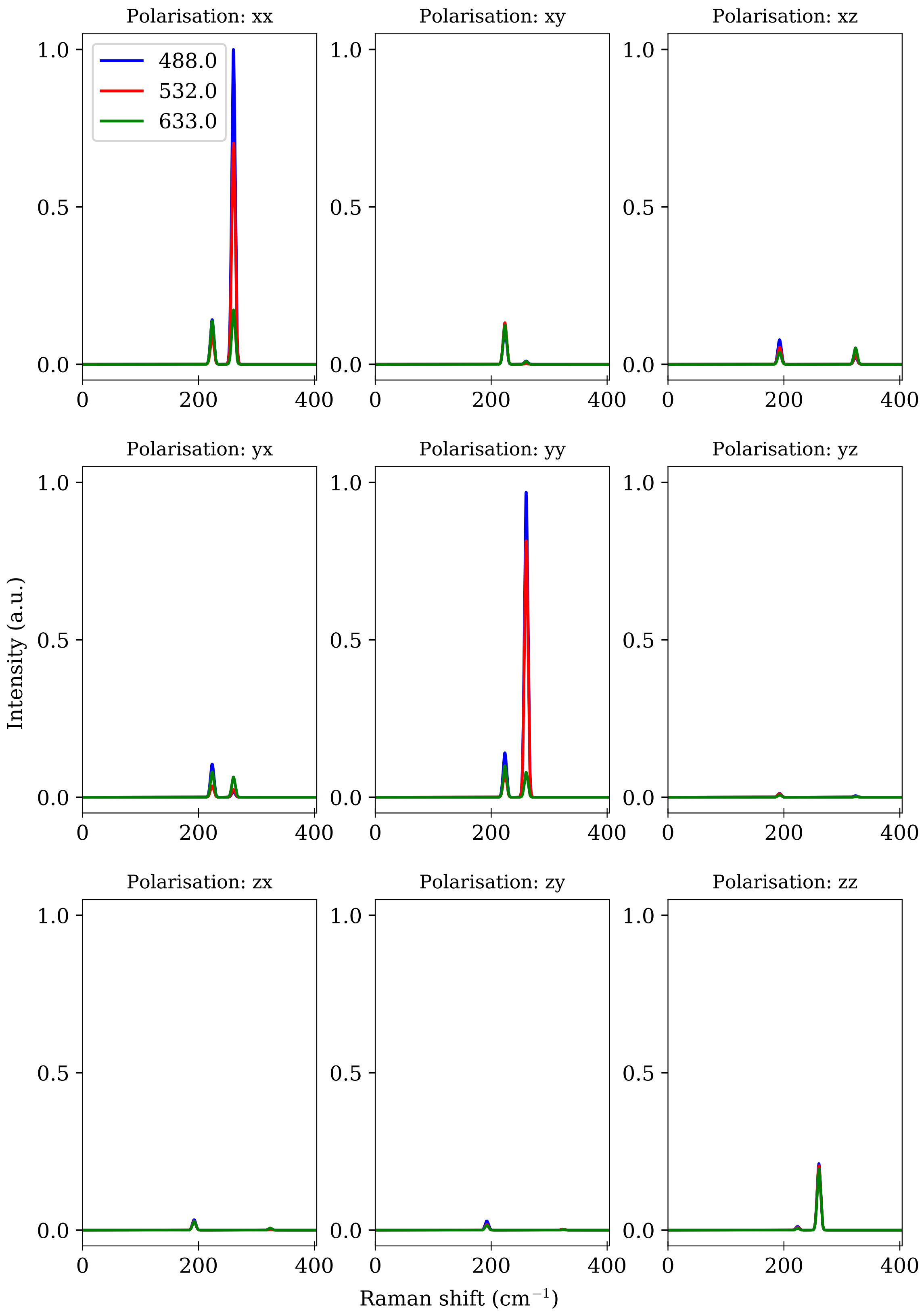

Supplementary Figure 266: Raman spectrum of $HfCl_2$ with space group 187 and Wyckoff positions b-i-0.

HfClI, 156, a-c-0

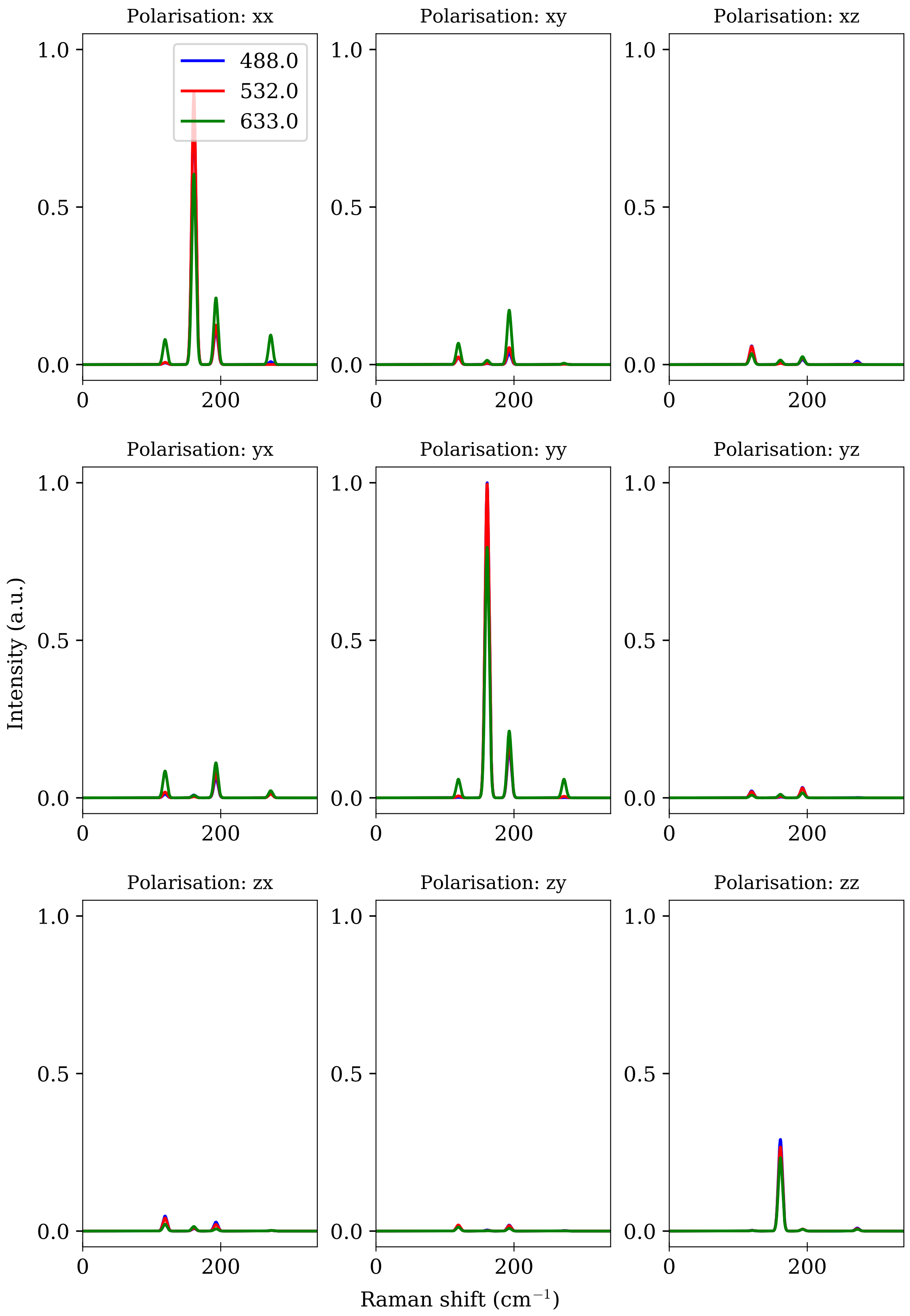

Supplementary Figure 267: Raman spectrum of HfClI with space group 156 and Wyckoff positions a-c-0.

HfH_2, 187, a-i-0

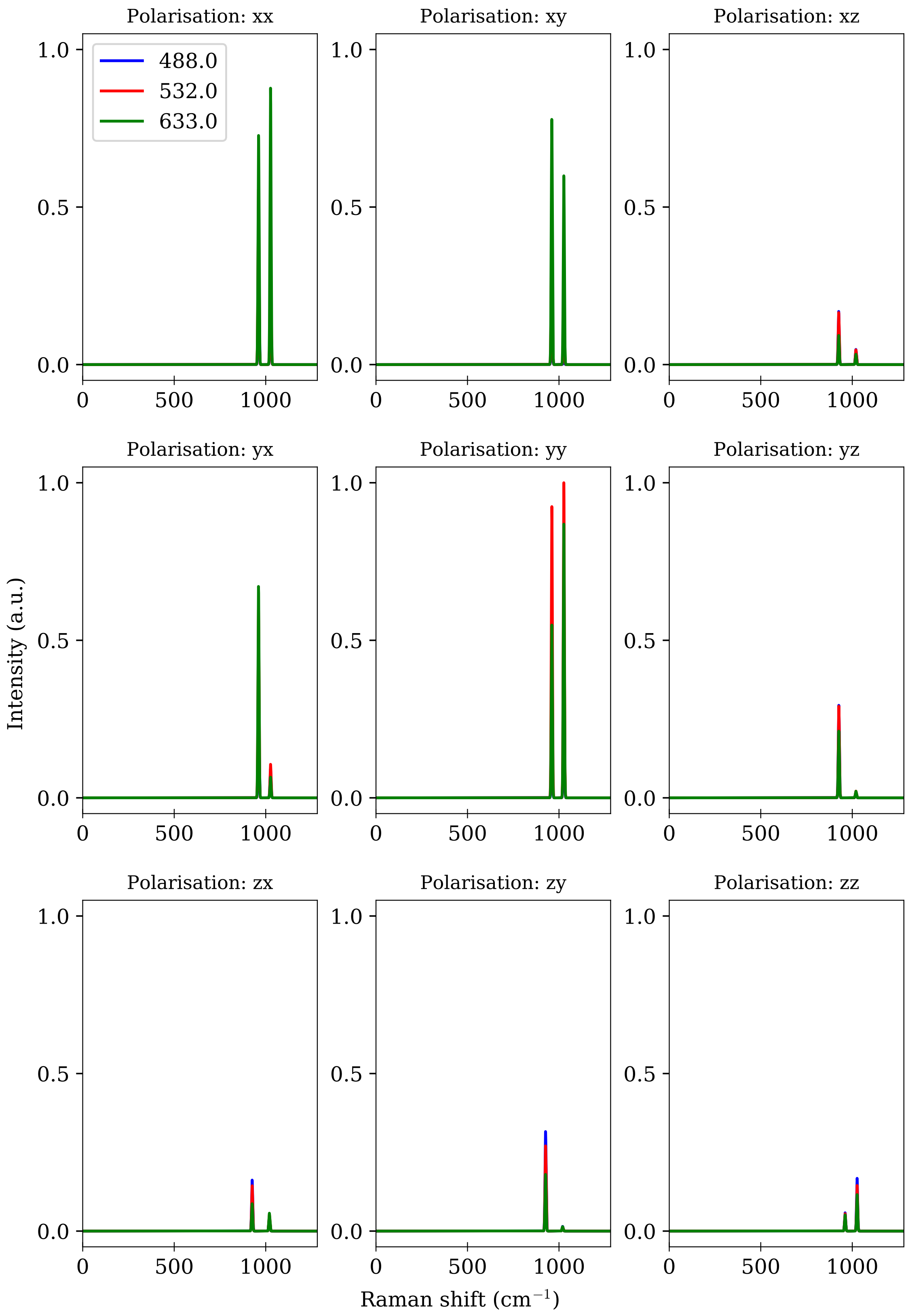

Supplementary Figure 268: Raman spectrum of HfH_2 with space group 187 and Wyckoff positions a-i-0.

HfI_2, 187, b-i-0

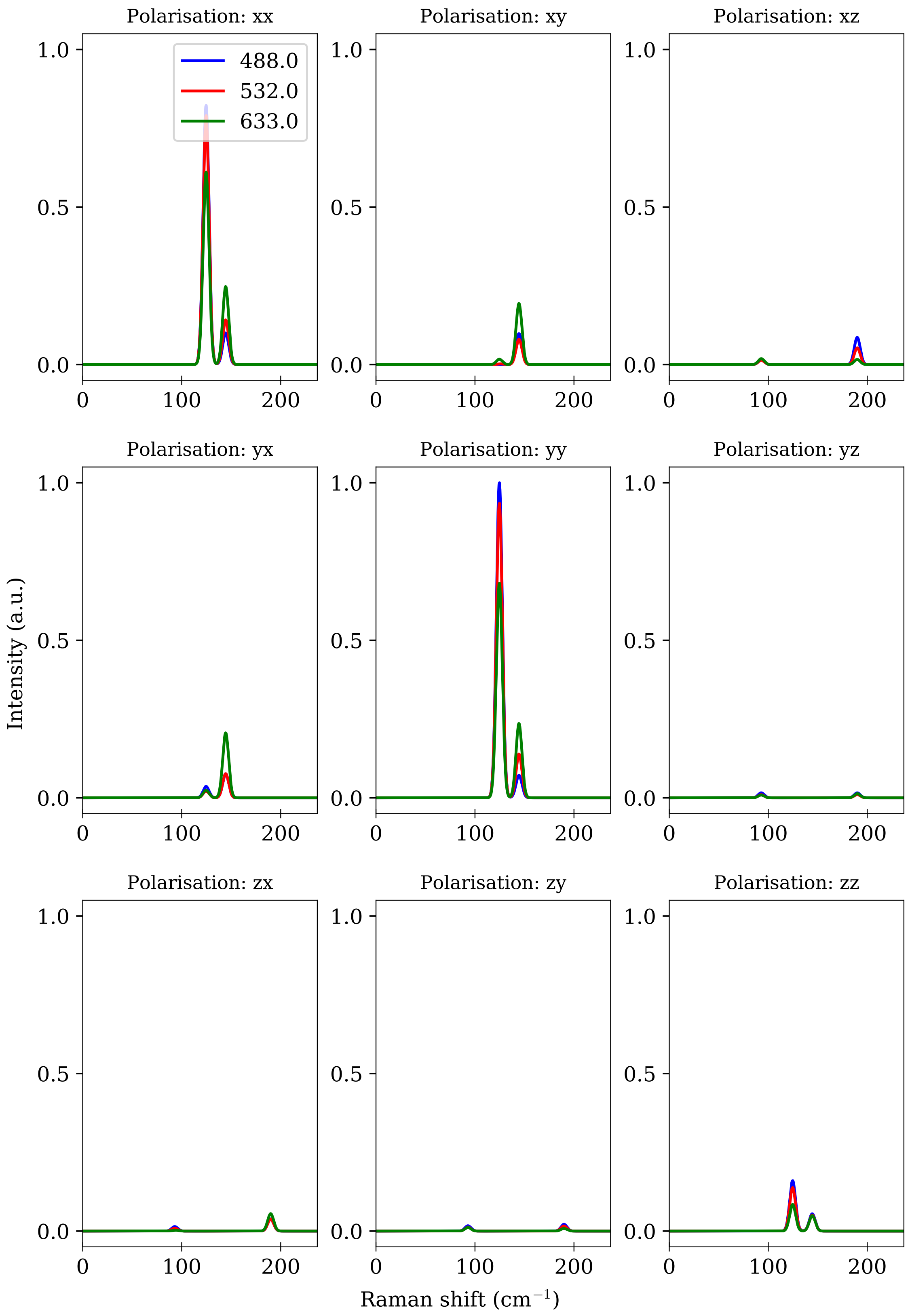

Supplementary Figure 269: Raman spectrum of HfI_2 with space group 187 and Wyckoff positions b-i-0.

$HfLi_2$, 187, a-i-0

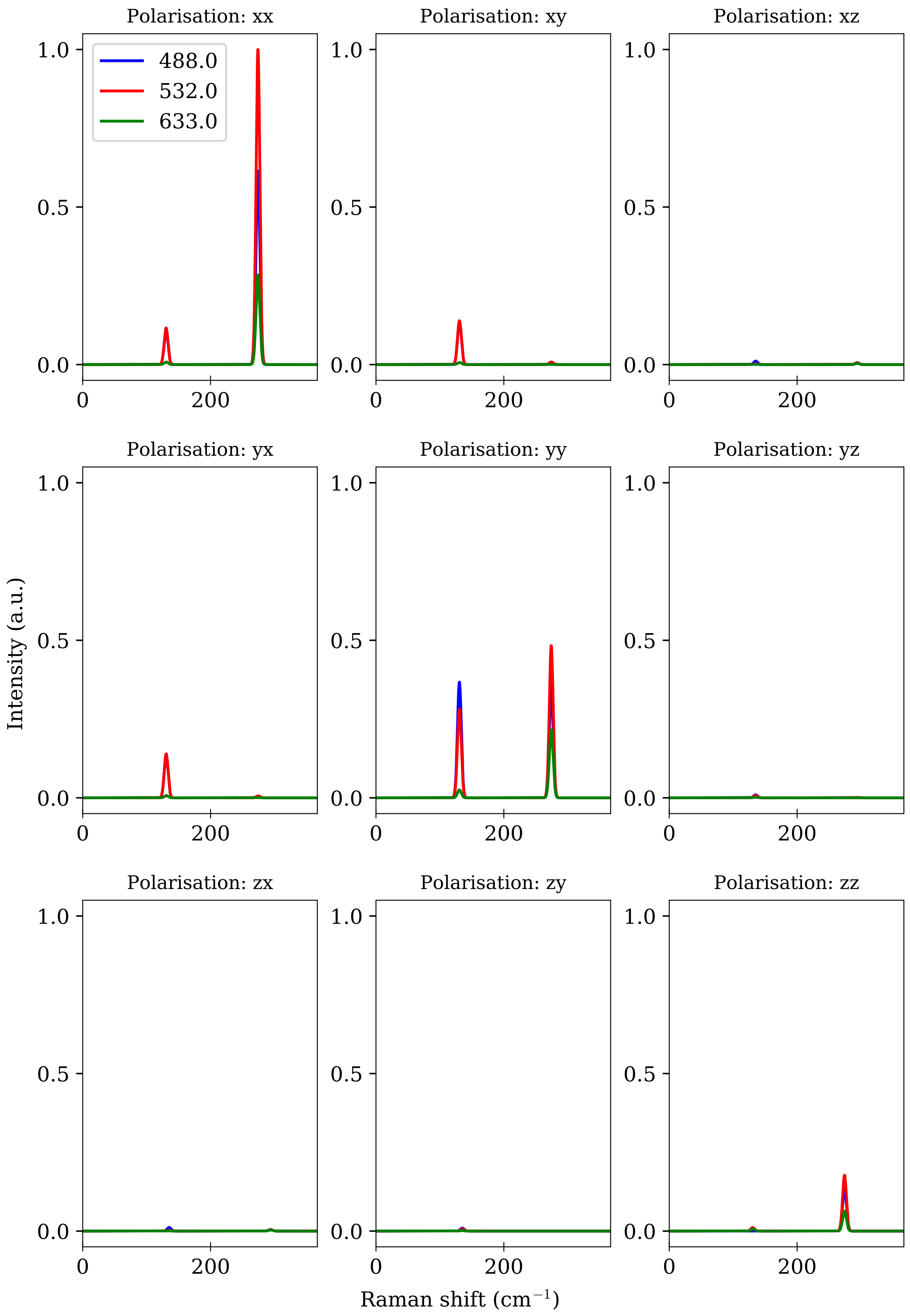

Supplementary Figure 270: Raman spectrum of $HfLi_2$ with space group 187 and Wyckoff positions a-i-0.

HfO_2, 115, d-g-0

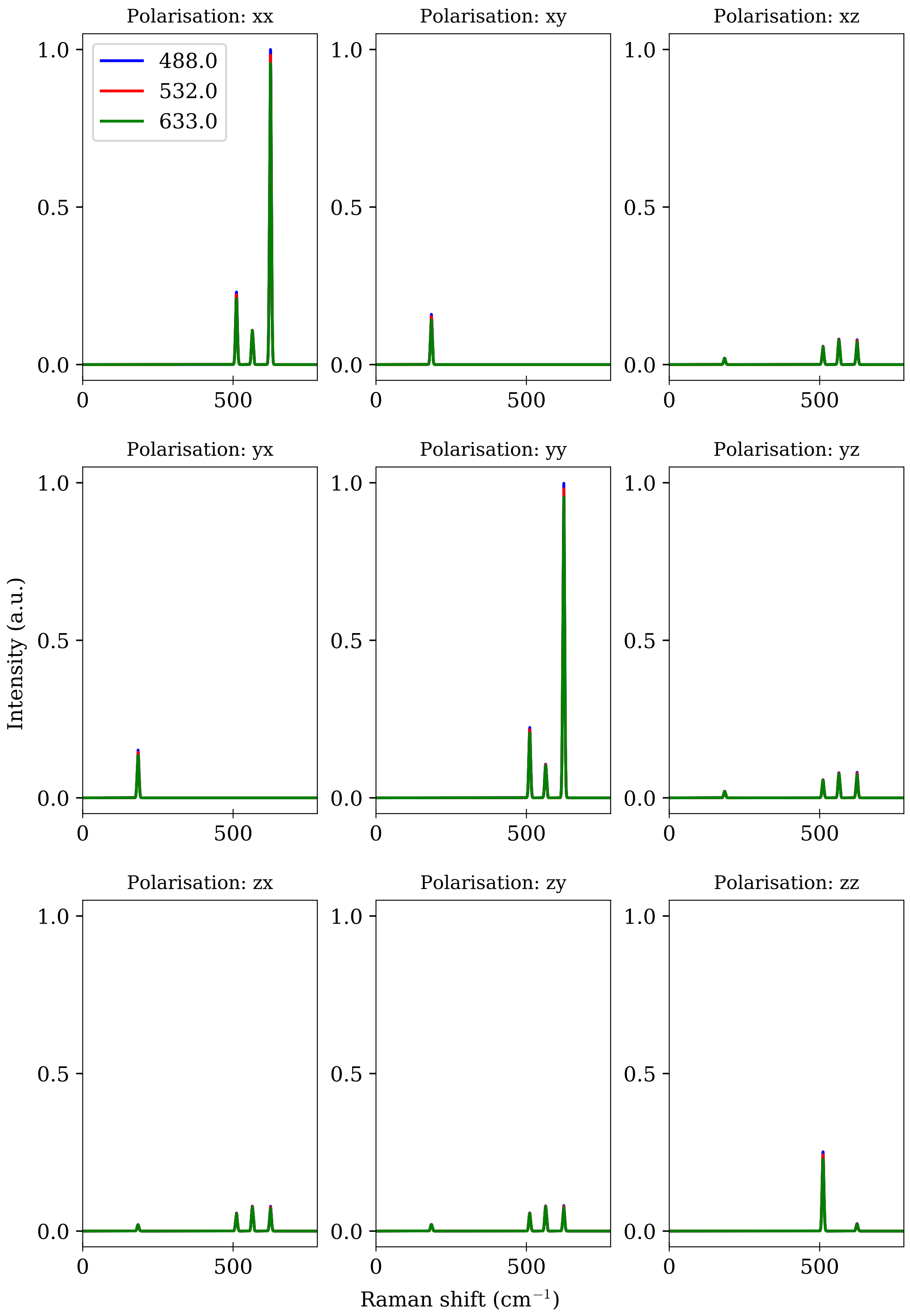

Supplementary Figure 271: Raman spectrum of HfO_2 with space group 115 and Wyckoff positions d-g-0.

HfO_2, 164, b-d-0

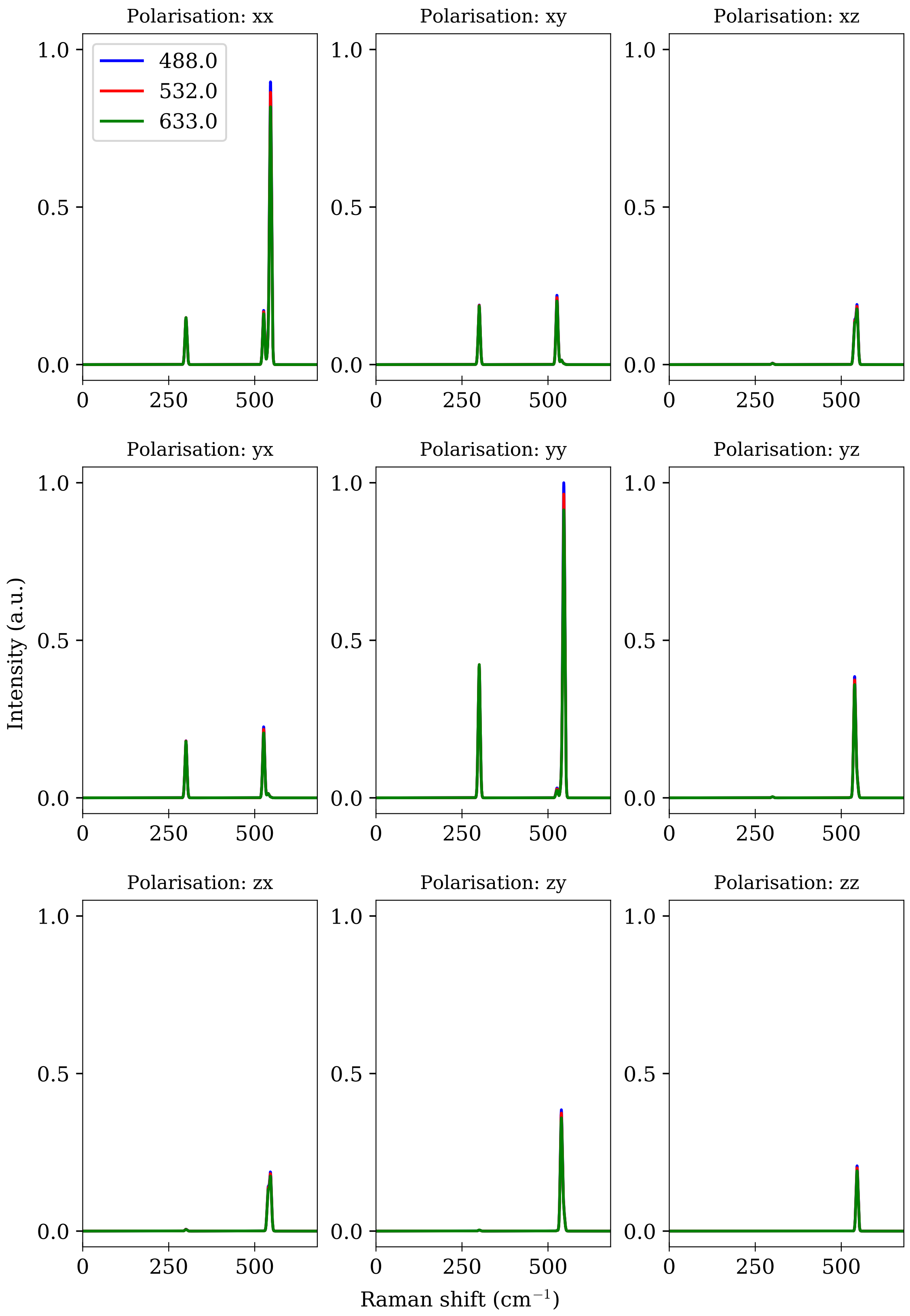

Supplementary Figure 272: Raman spectrum of HfO_2 with space group 164 and Wyckoff positions b-d-0.

HfS_2, 115, d-g-0

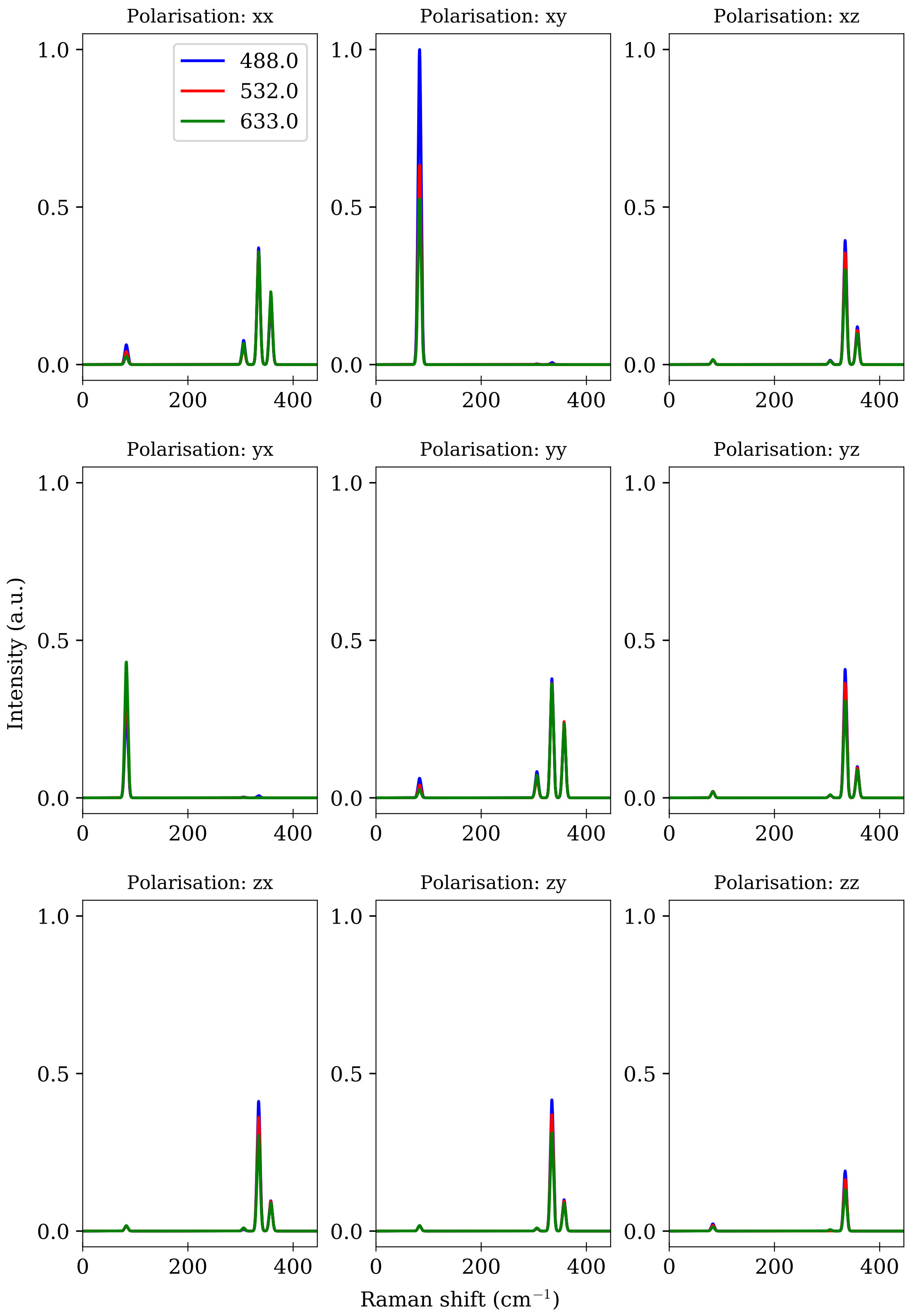

Supplementary Figure 273: Raman spectrum of HfS_2 with space group 115 and Wyckoff positions d-g-0.

HfS_2, 164, b-d-0

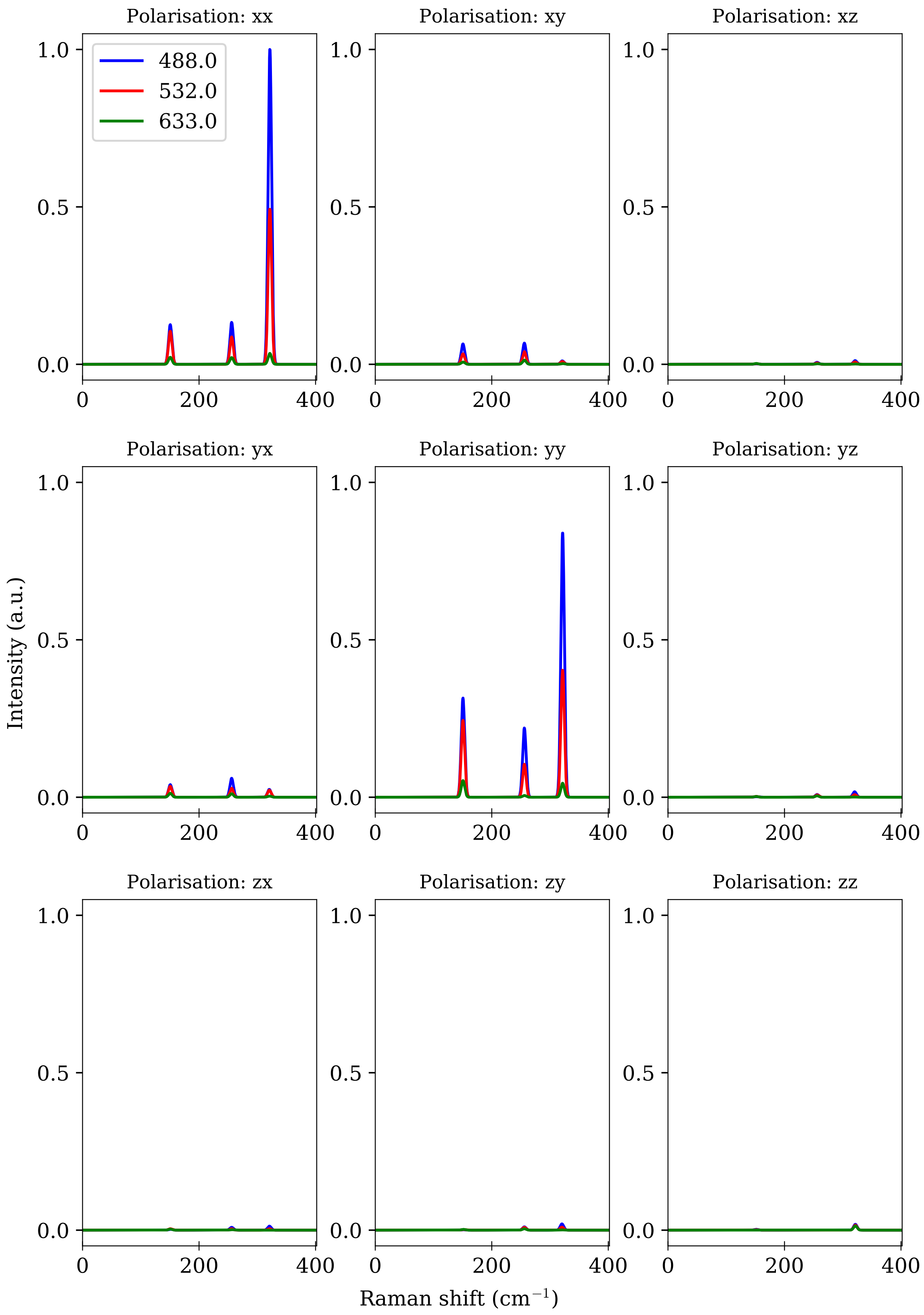

Supplementary Figure 274: Raman spectrum of HfS_2 with space group 164 and Wyckoff positions b-d-0.

$HfSb_2$, 164, b-d-0

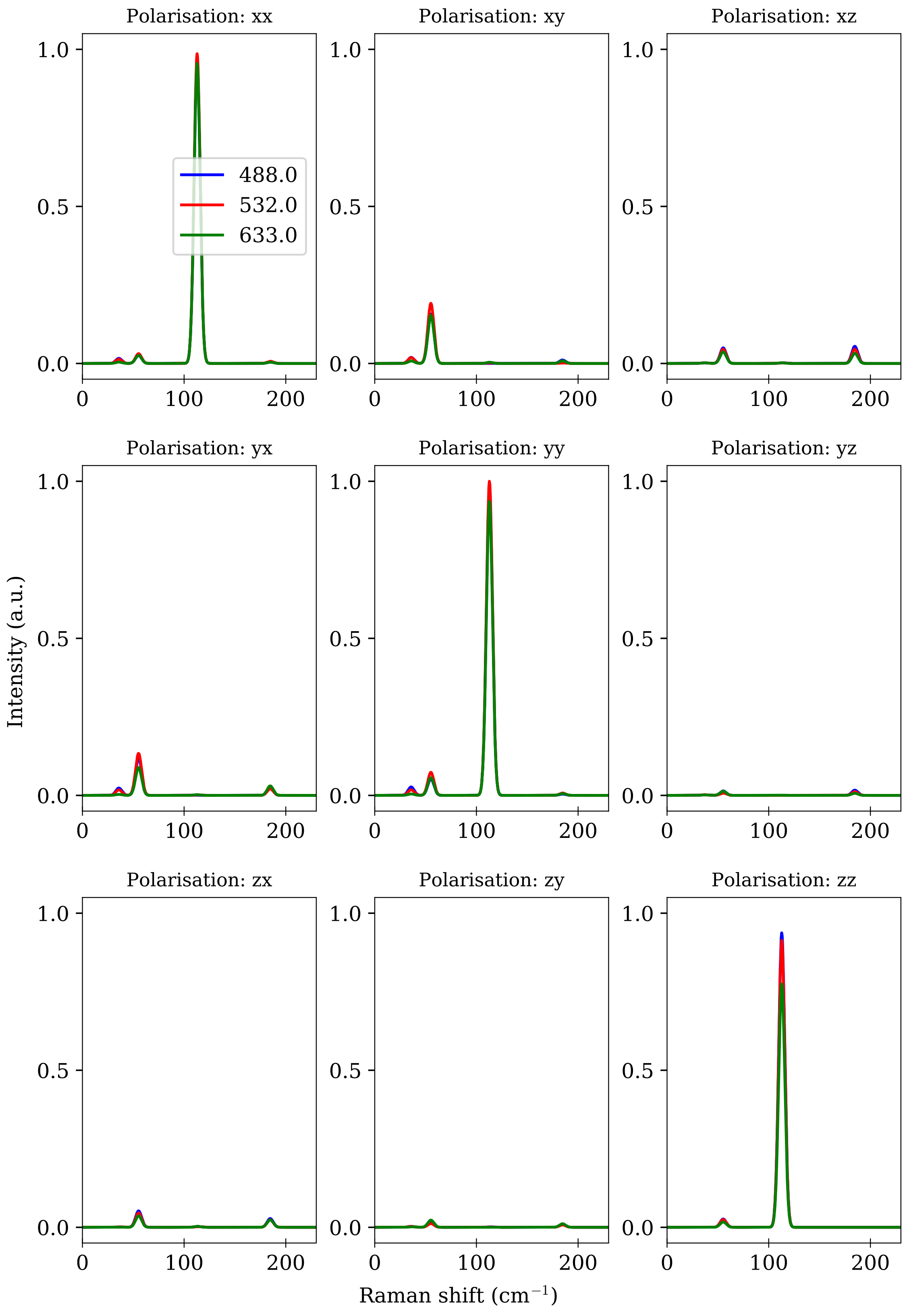

Supplementary Figure 275: Raman spectrum of $HfSb_2$ with space group 164 and Wyckoff positions b-d-0.

$HfSb_2$, 187, b-i-0

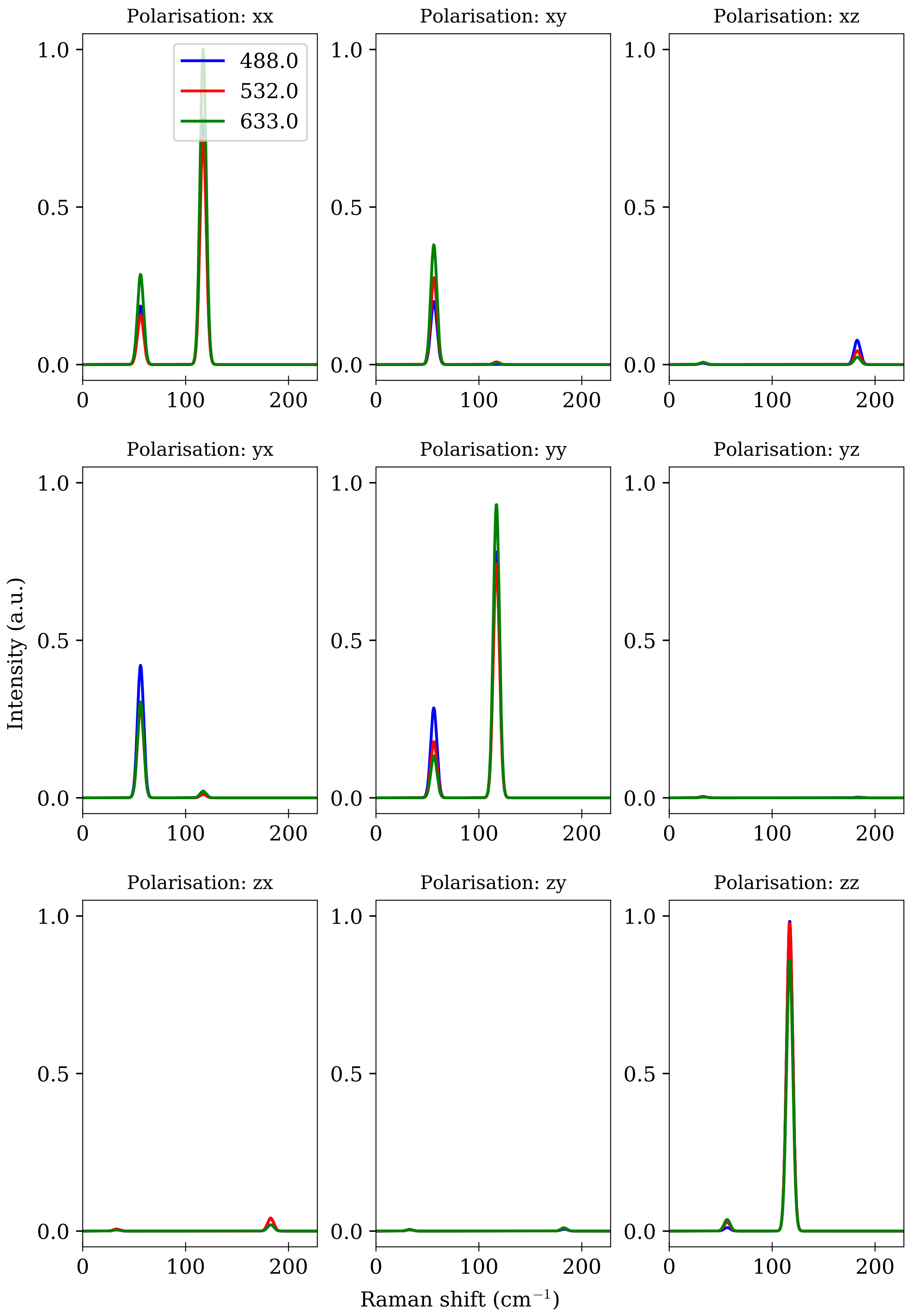

Supplementary Figure 276: Raman spectrum of $HfSb_2$ with space group 187 and Wyckoff positions b-i-0.

$HfSe_2$, 115, a-g-0

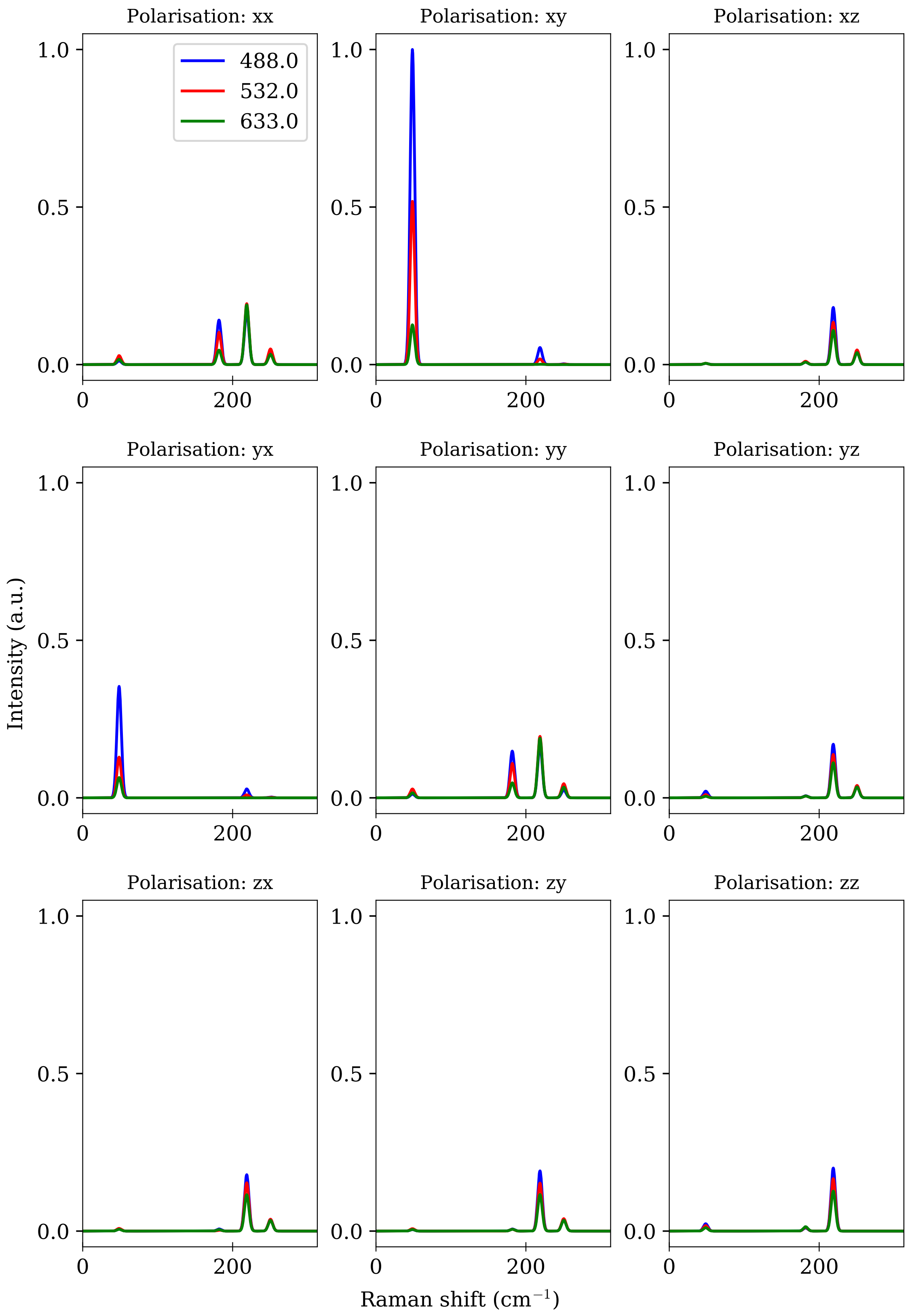

Supplementary Figure 277: Raman spectrum of $HfSe_2$ with space group 115 and Wyckoff positions a-g-0.

$HfSe_2$, 164, b-d-0

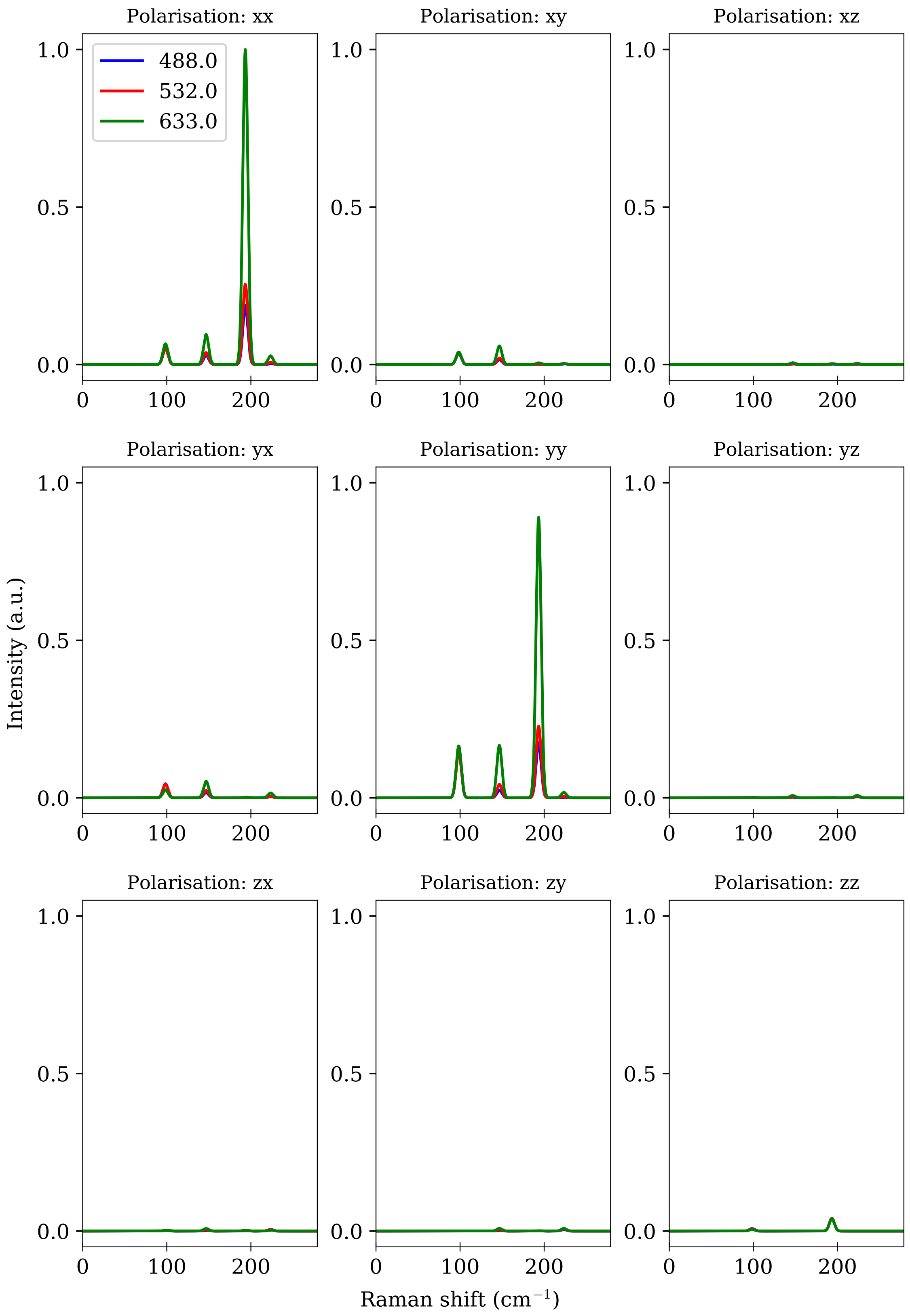

Supplementary Figure 278: Raman spectrum of $HfSe_2$ with space group 164 and Wyckoff positions b-d-0.

HfSeTe, 156, a-b-c-0

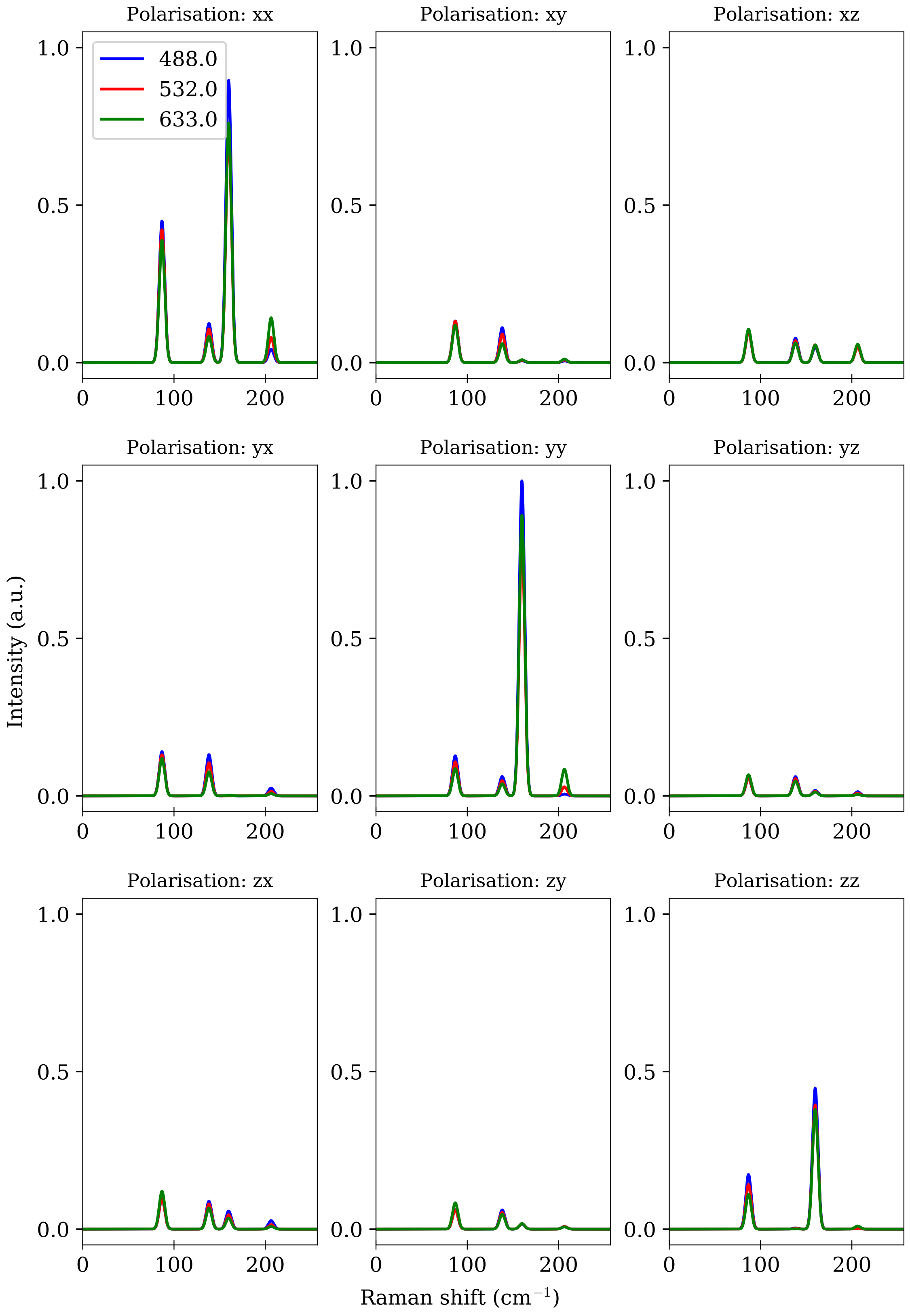

Supplementary Figure 279: Raman spectrum of HfSeTe with space group 156 and Wyckoff positions a-b-c-0.

HfSeTe, 156, a-c-0

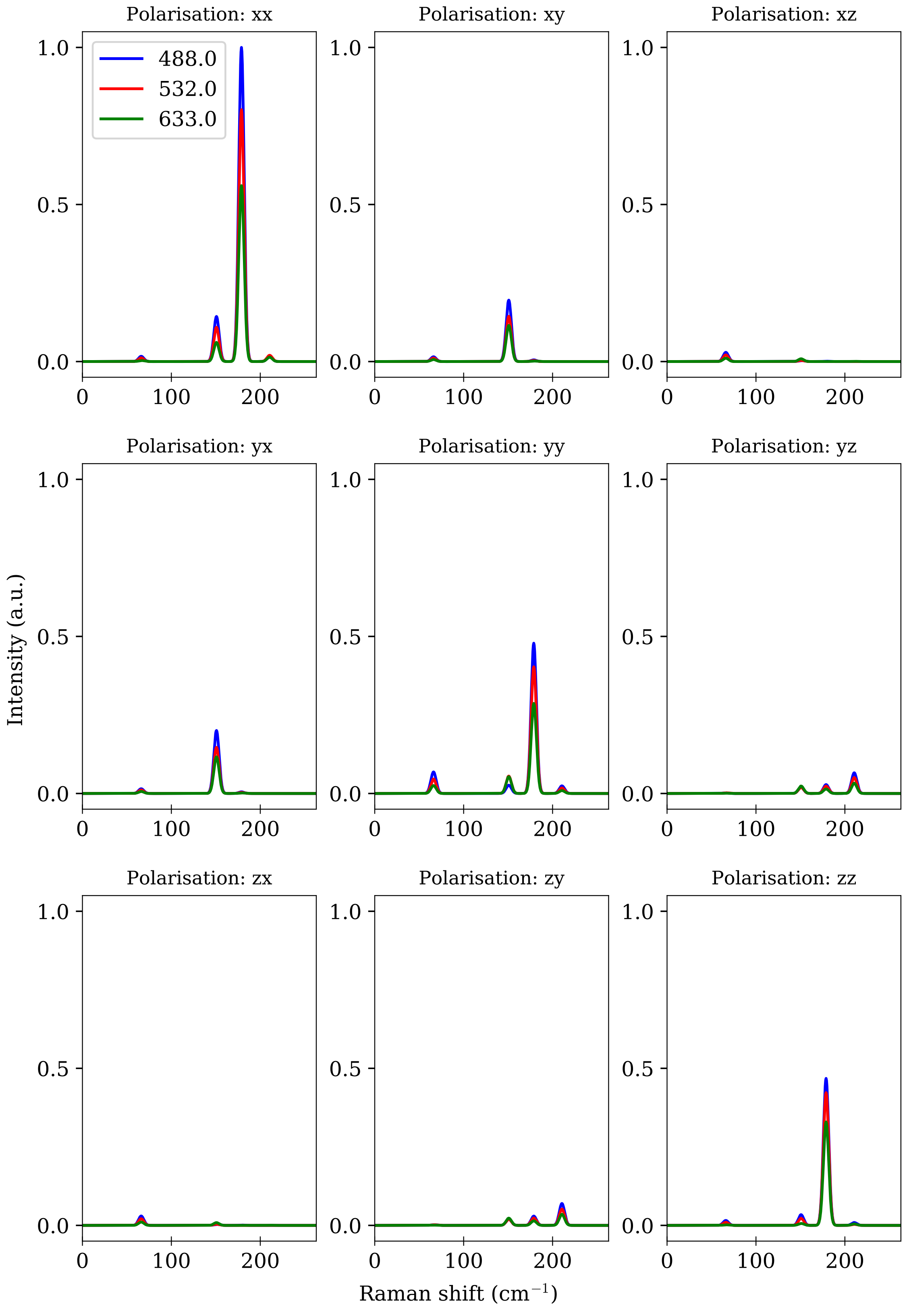

Supplementary Figure 280: Raman spectrum of HfSeTe with space group 156 and Wyckoff positions a-c-0.

HfSSe, 156, a-b-c-0

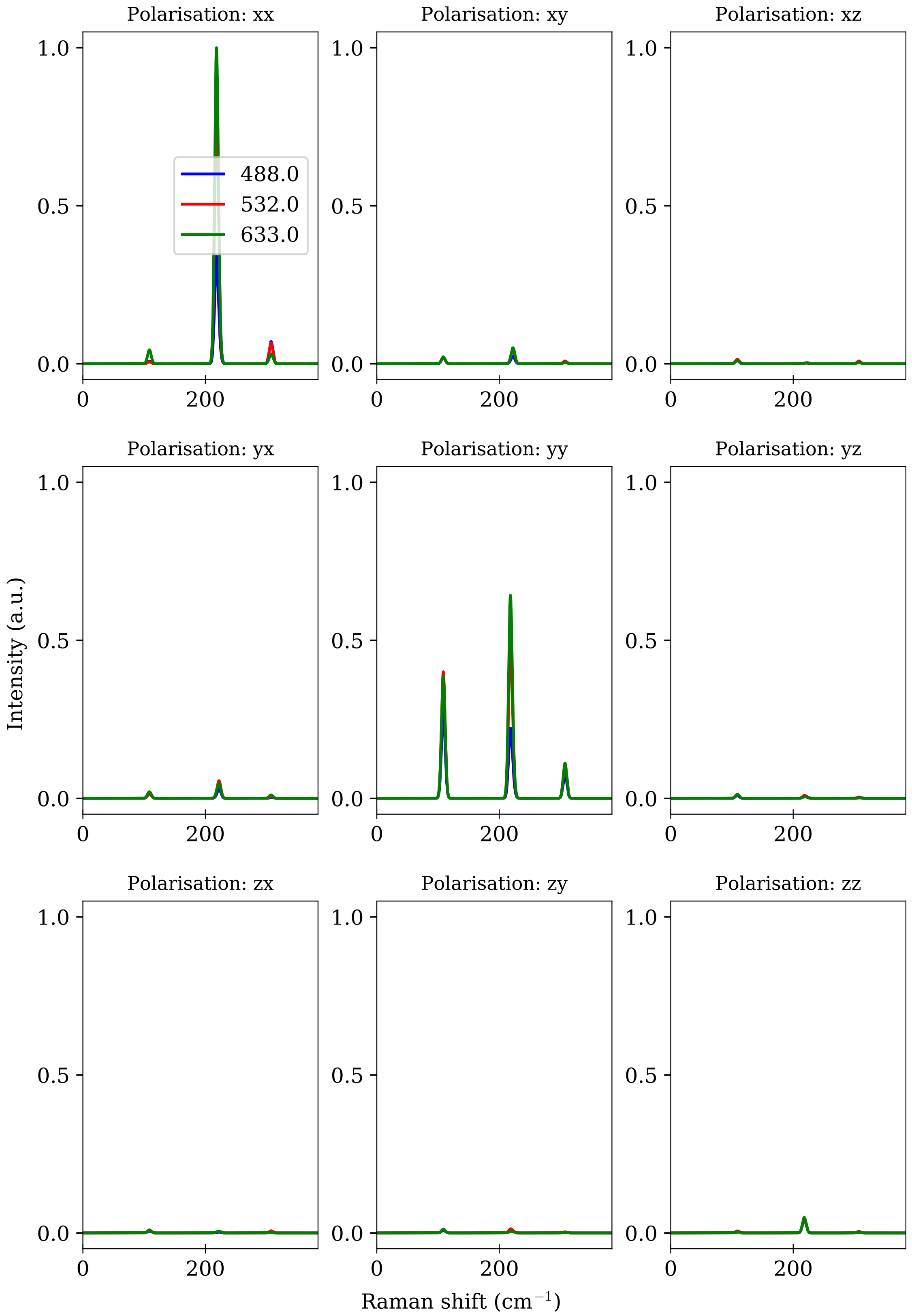

Supplementary Figure 281: Raman spectrum of HfSSe with space group 156 and Wyckoff positions a-b-c-0.

HfSTe, 156, a-b-c-0

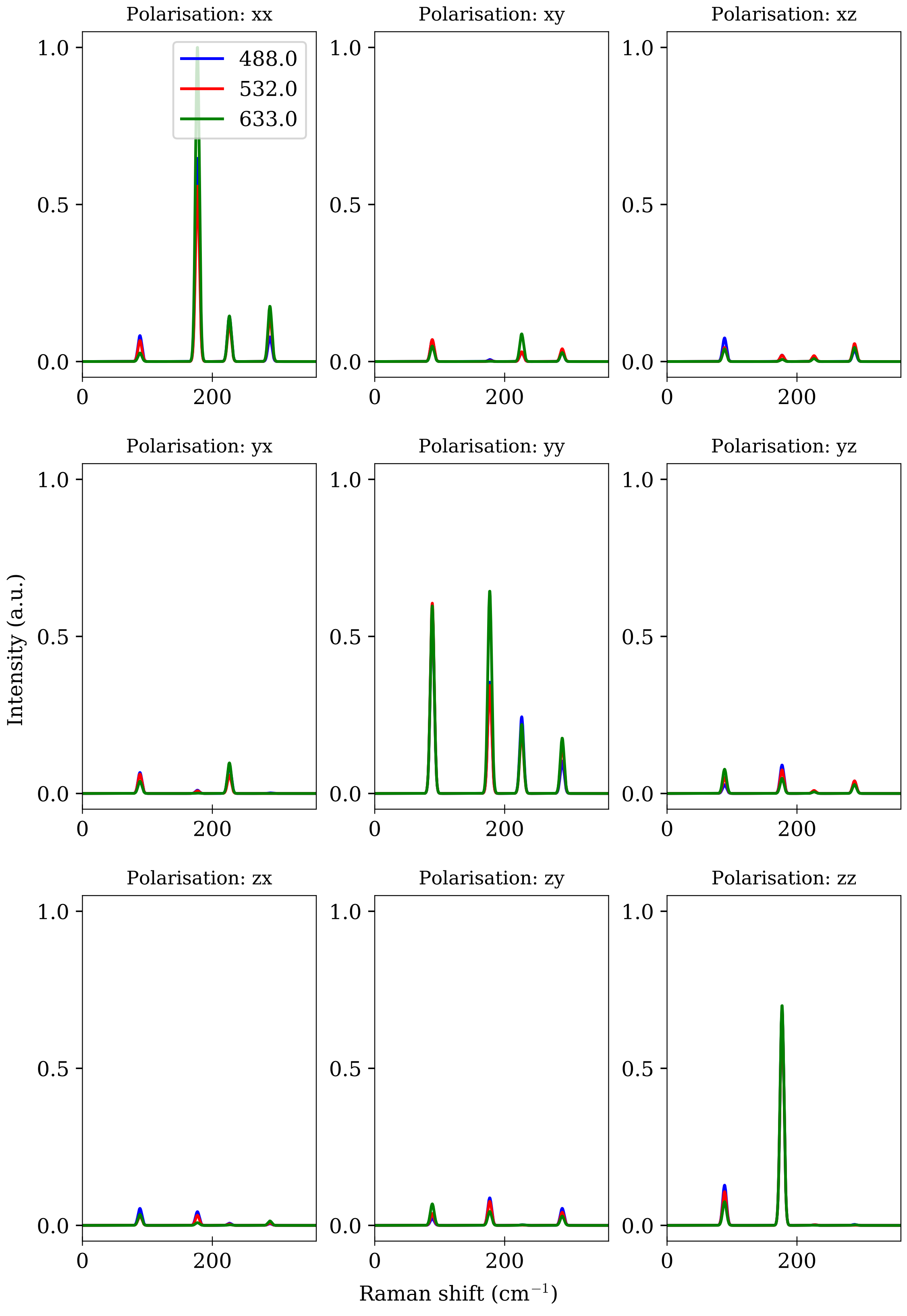

Supplementary Figure 282: Raman spectrum of HfSTe with space group 156 and Wyckoff positions a-b-c-0.

HfSTe, 156, a-c-0

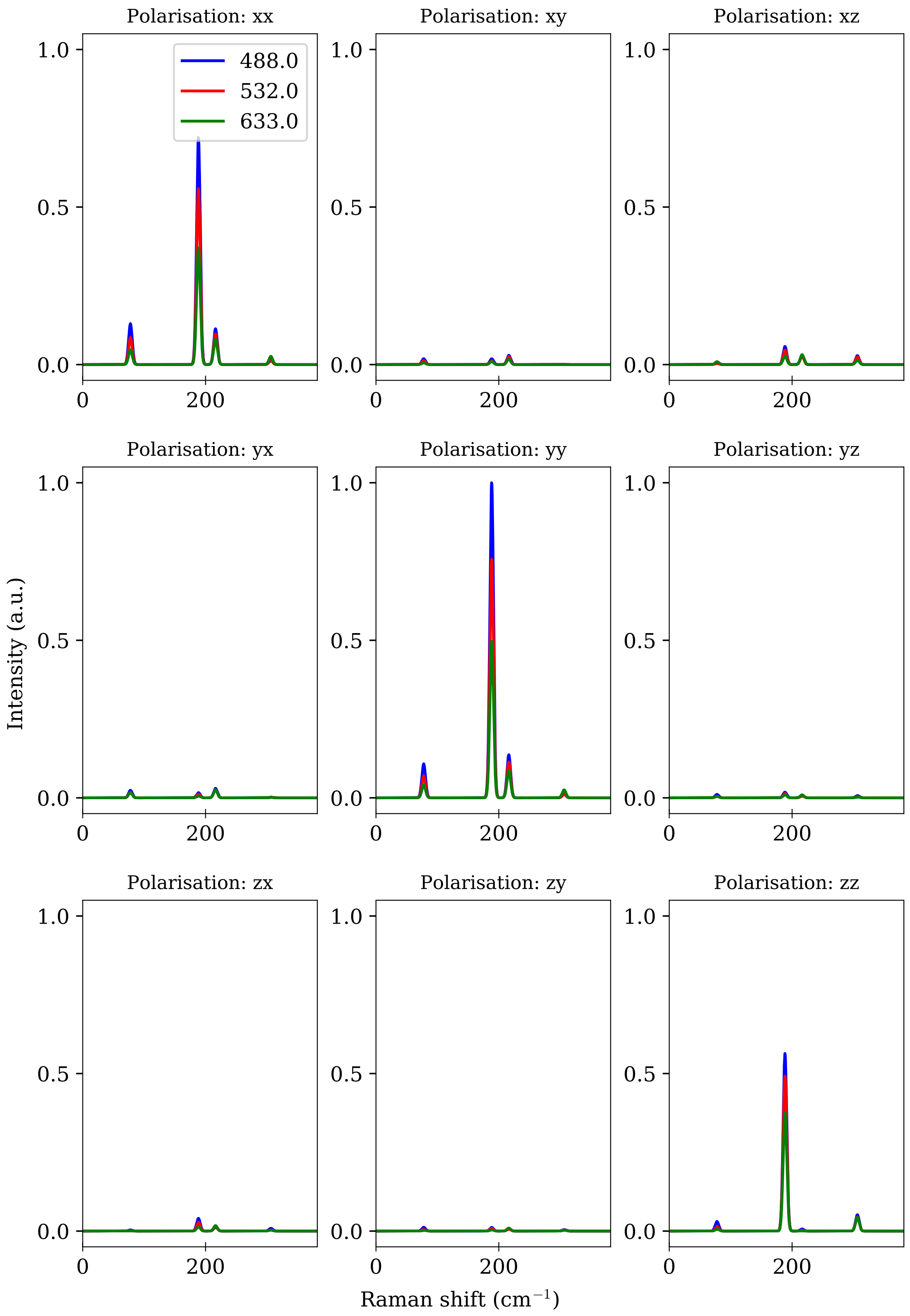

Supplementary Figure 283: Raman spectrum of HfSTe with space group 156 and Wyckoff positions a-c-0.

$HfTe_2$, 164, b-d-0

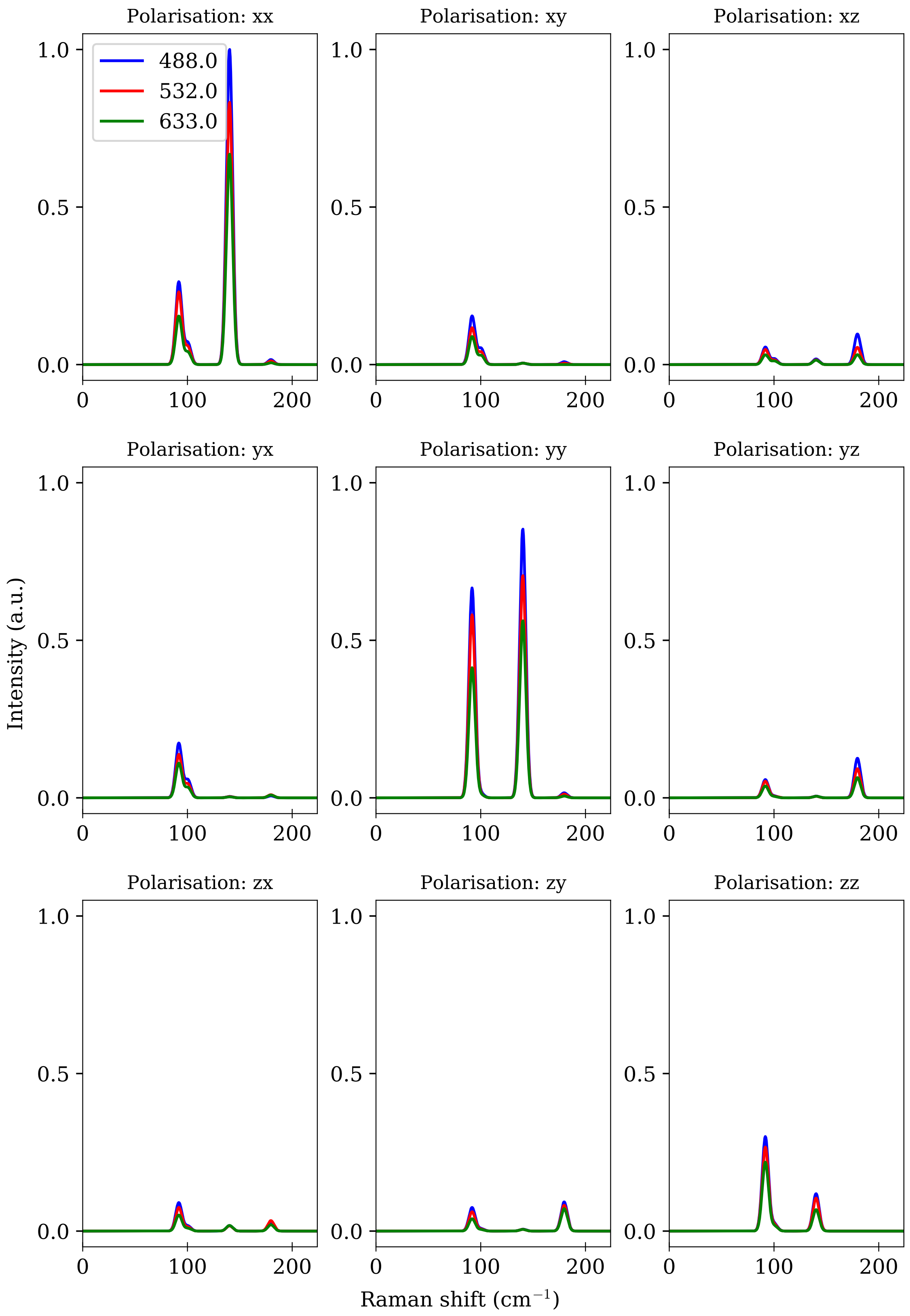

Supplementary Figure 284: Raman spectrum of $HfTe_2$ with space group 164 and Wyckoff positions b-d-0.

$HfTe_2$, 187, b-i-0

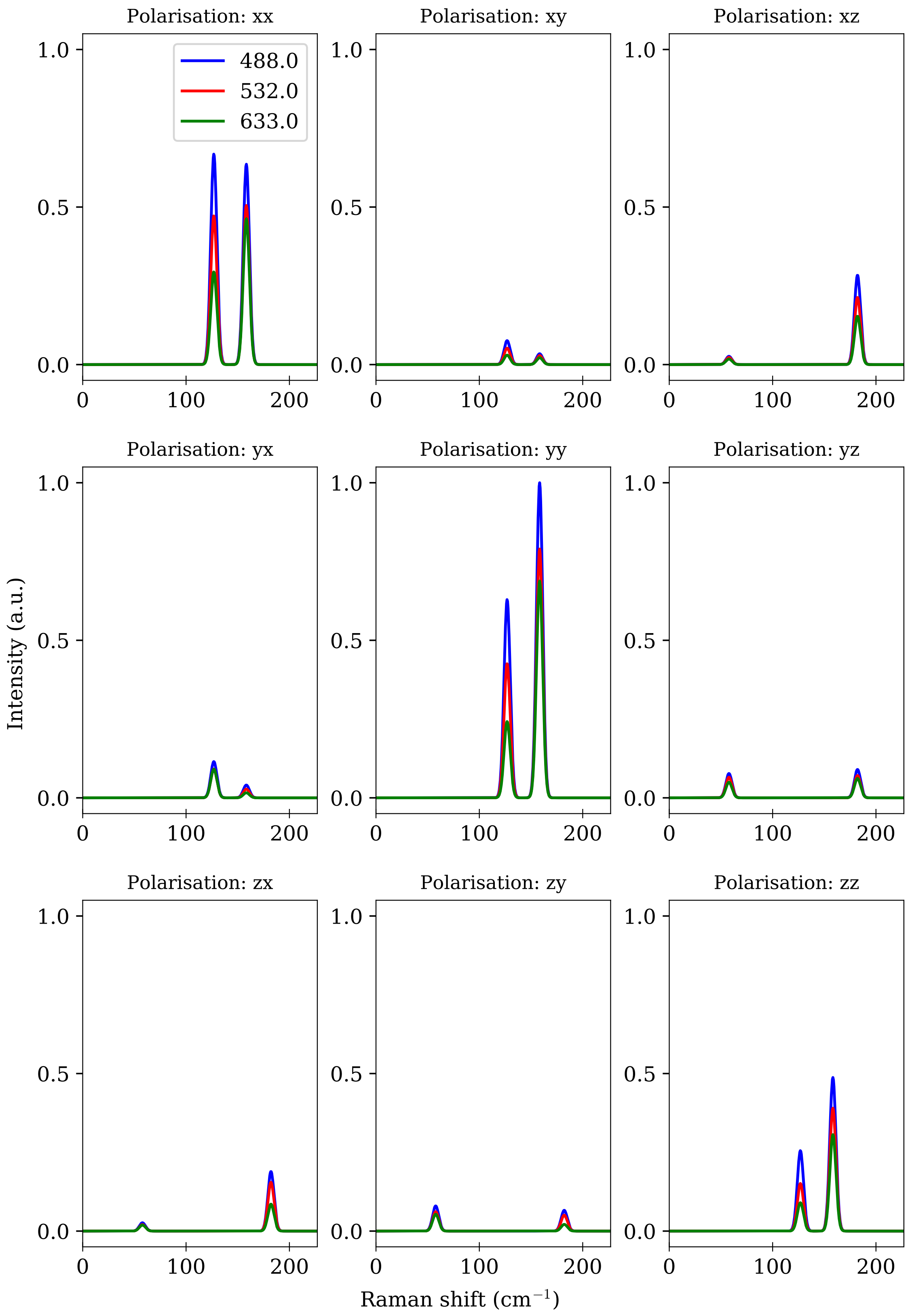

Supplementary Figure 285: Raman spectrum of $HfTe_2$ with space group 187 and Wyckoff positions b-i-0.

Hg_2I_2, 1, a-0

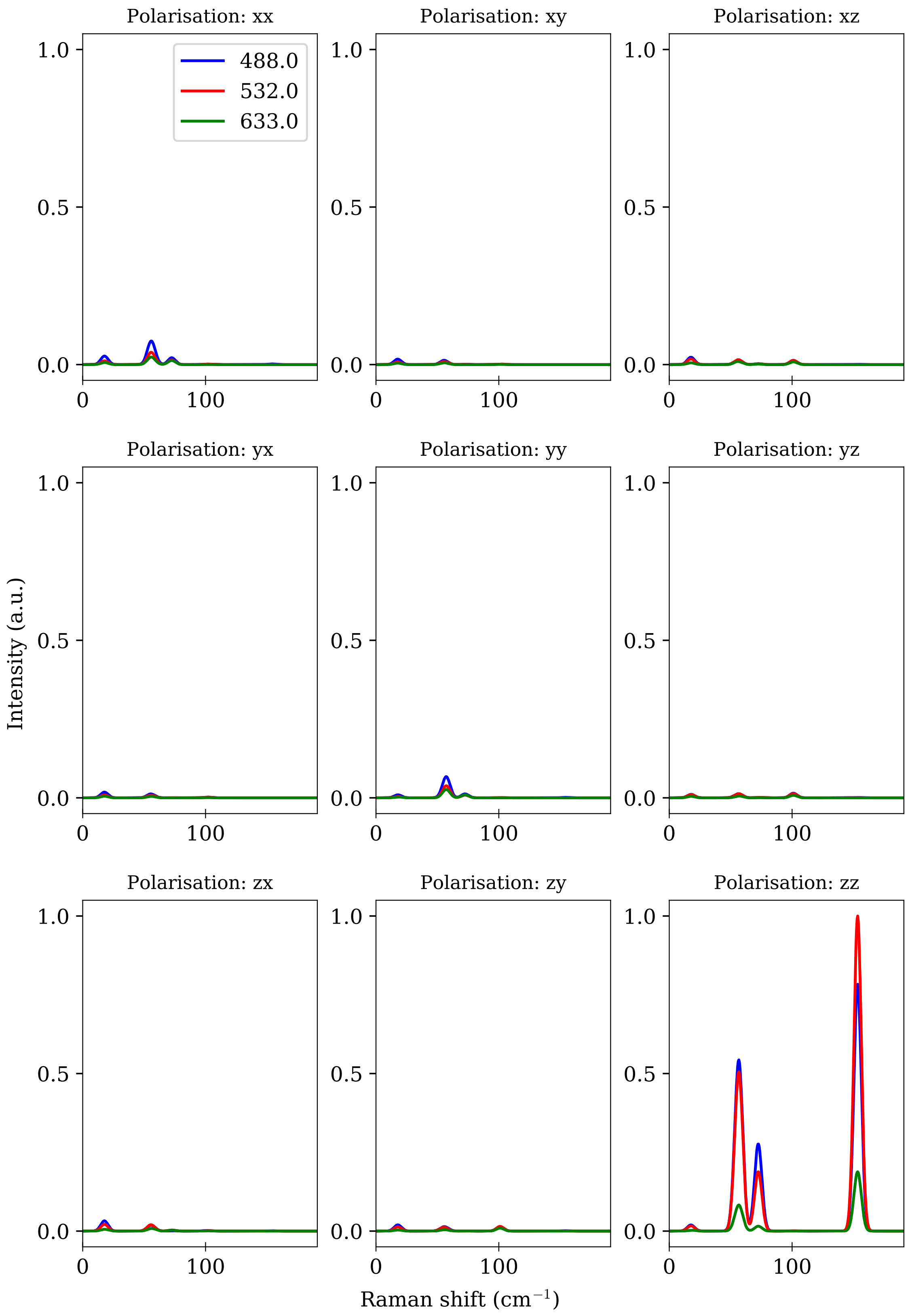

Supplementary Figure 286: Raman spectrum of Hg_2I_2 with space group 1 and Wyckoff positions a-0.

$HgBr_2$, 164, b-d-0

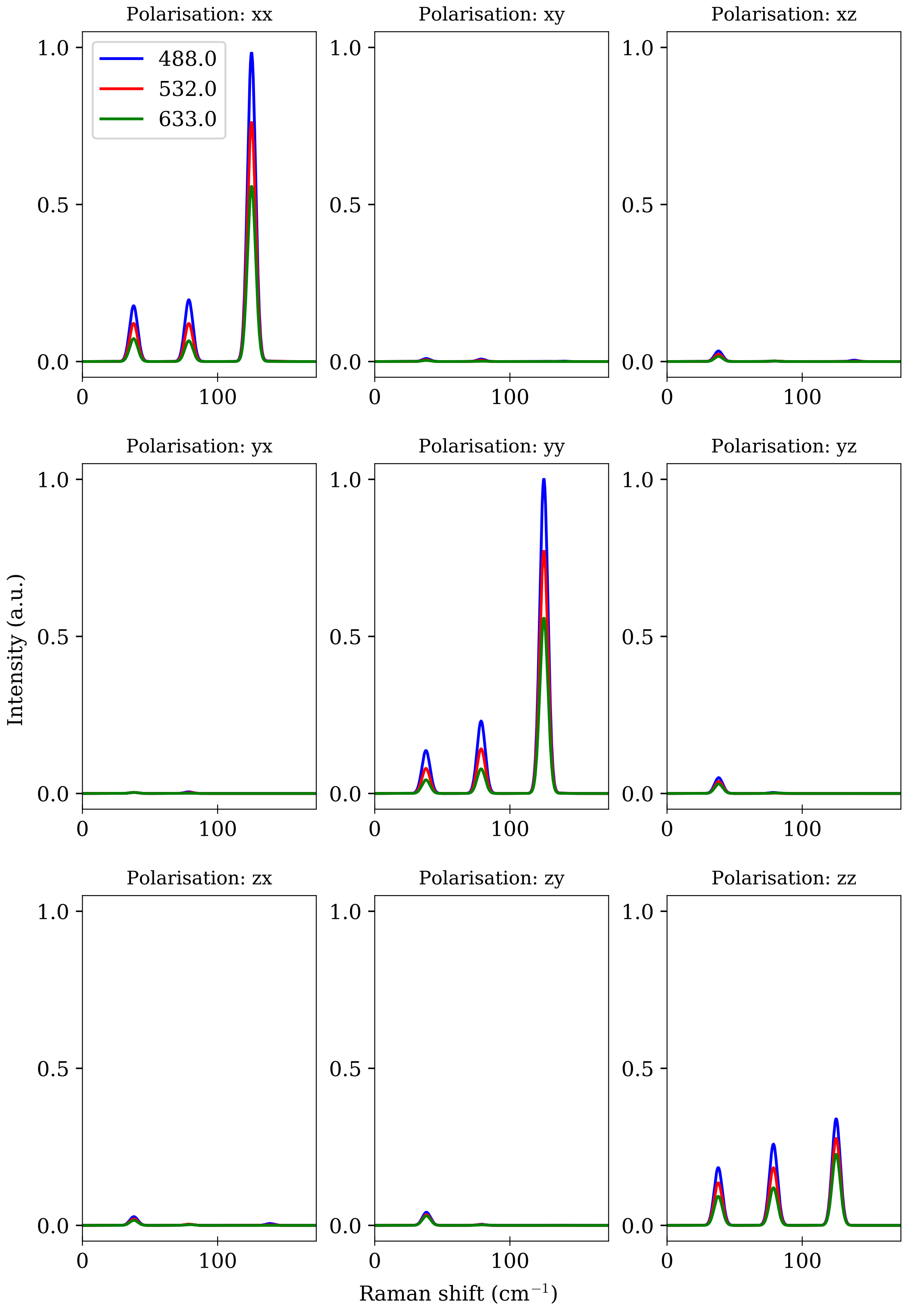

Supplementary Figure 287: Raman spectrum of $HgBr_2$ with space group 164 and Wyckoff positions b-d-0.

HgSe, 156, b-c-0

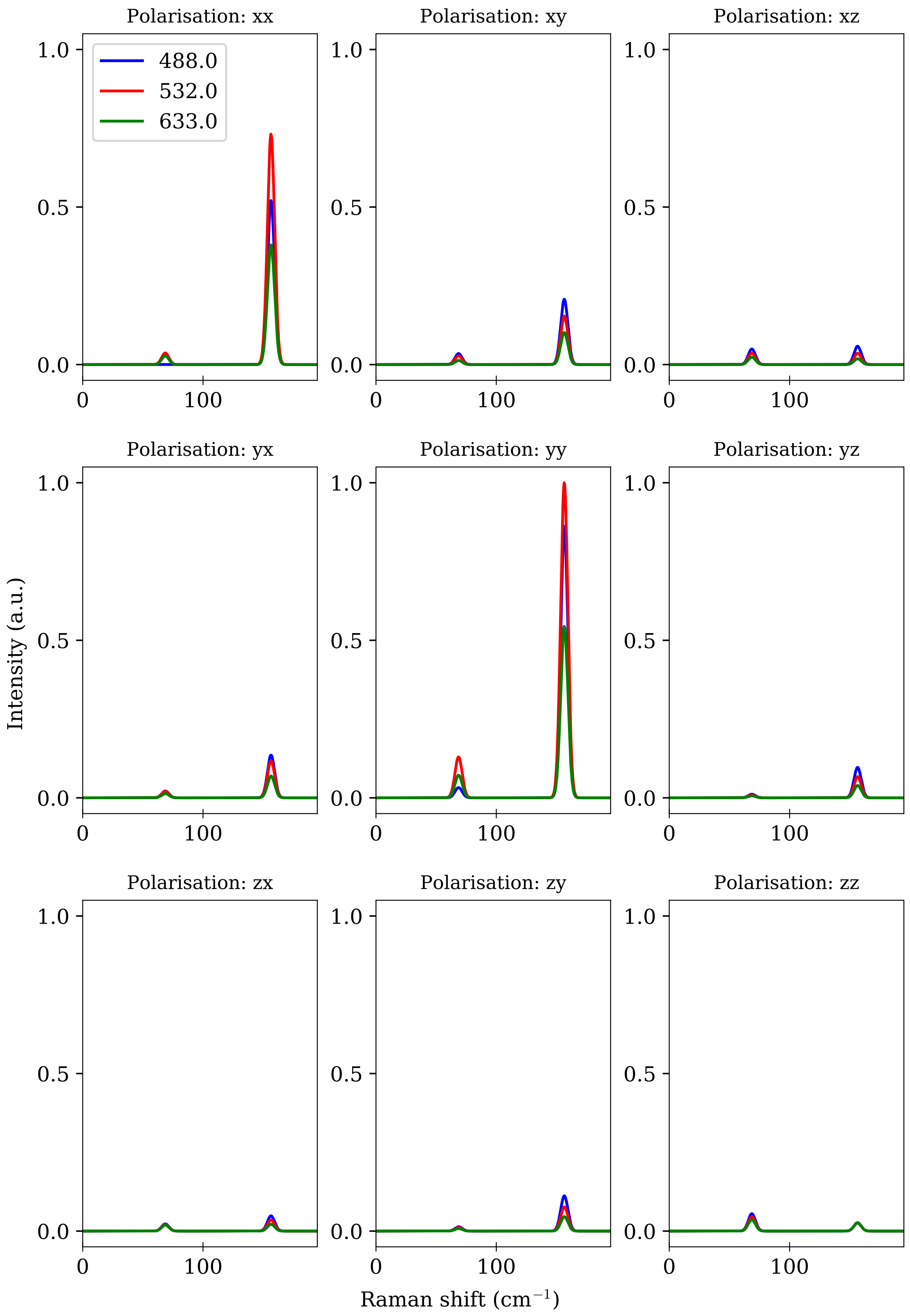

Supplementary Figure 288: Raman spectrum of HgSe with space group 156 and Wyckoff positions b-c-0.

HgTe, 156, b-c-0

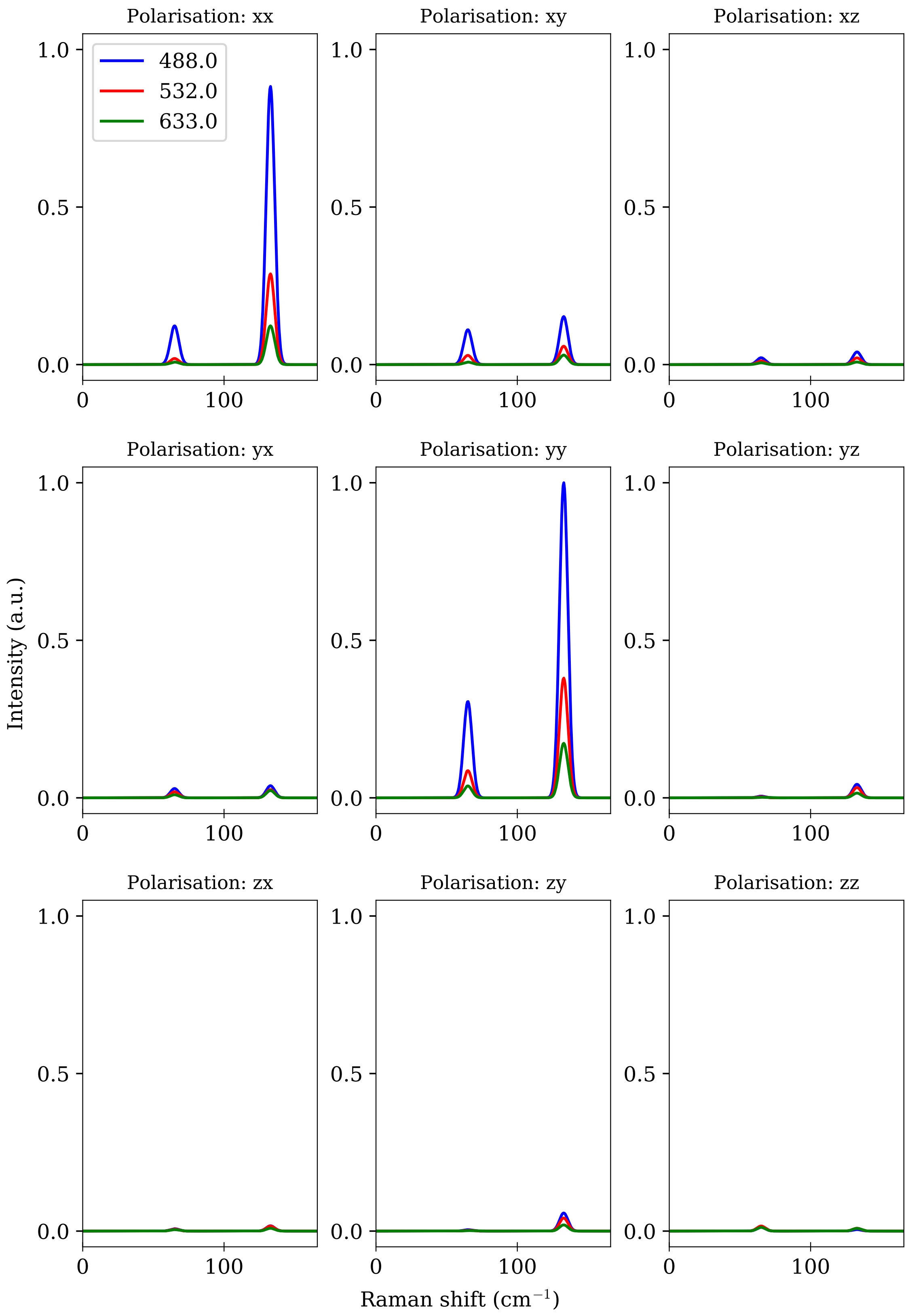

Supplementary Figure 289: Raman spectrum of HgTe with space group 156 and Wyckoff positions b-c-0.

I_2Sb_2, 1, a-0

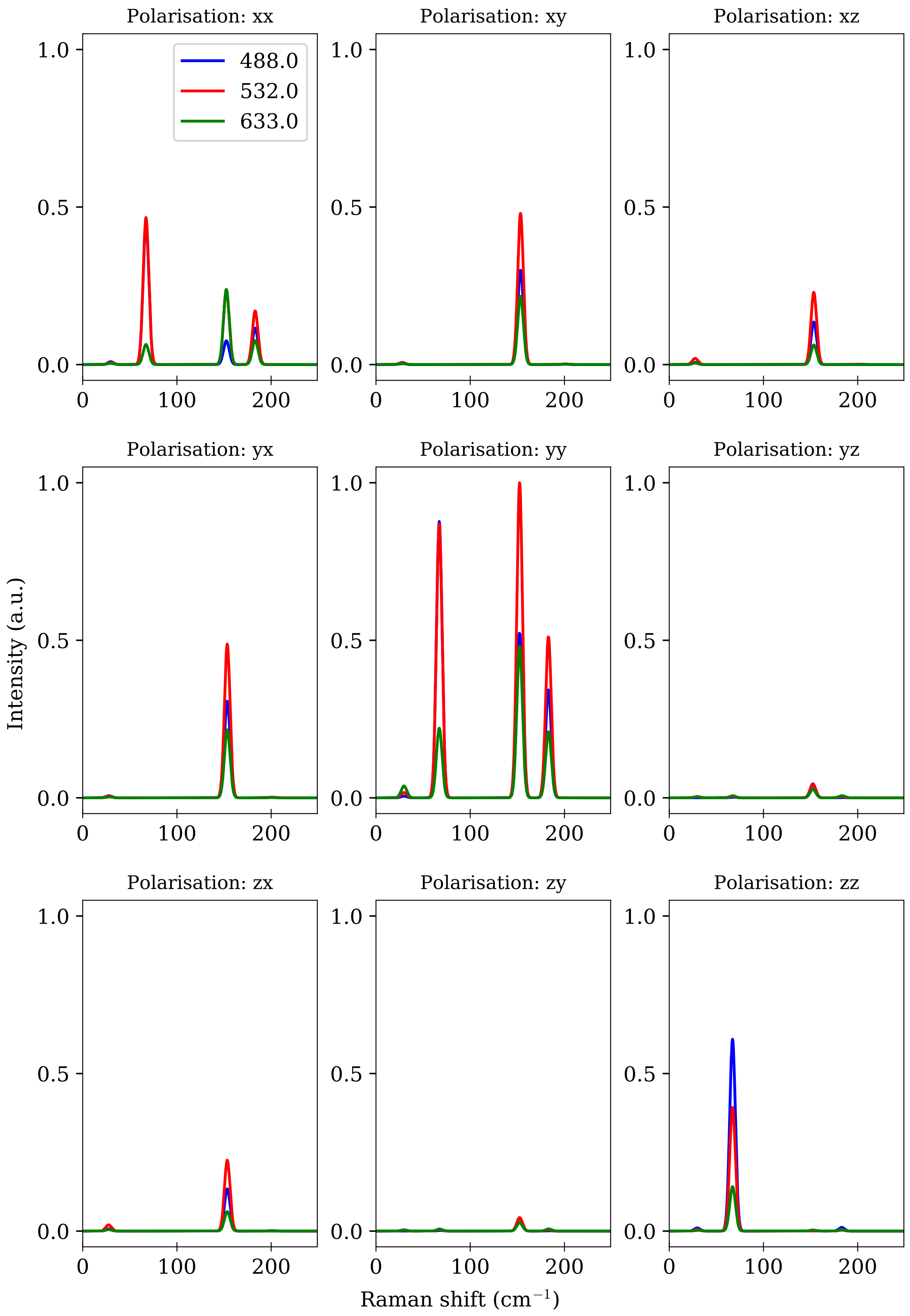

Supplementary Figure 290: Raman spectrum of I_2Sb_2 with space group 1 and Wyckoff positions a-0.

In_2I_2, 2, i-0

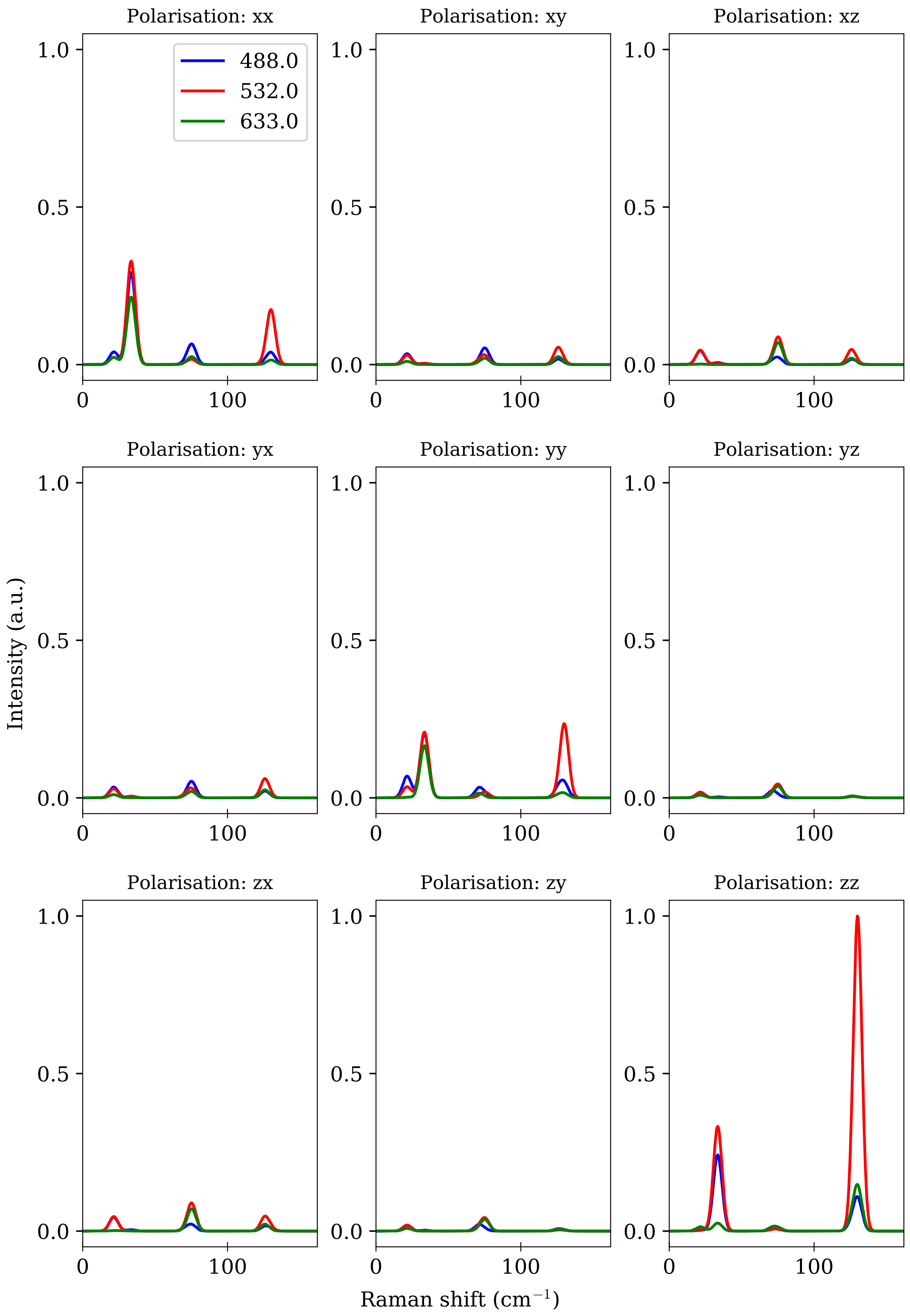

Supplementary Figure 291: Raman spectrum of In_2I_2 with space group 2 and Wyckoff positions i-0.

In_2O_2, 187, h-i-0

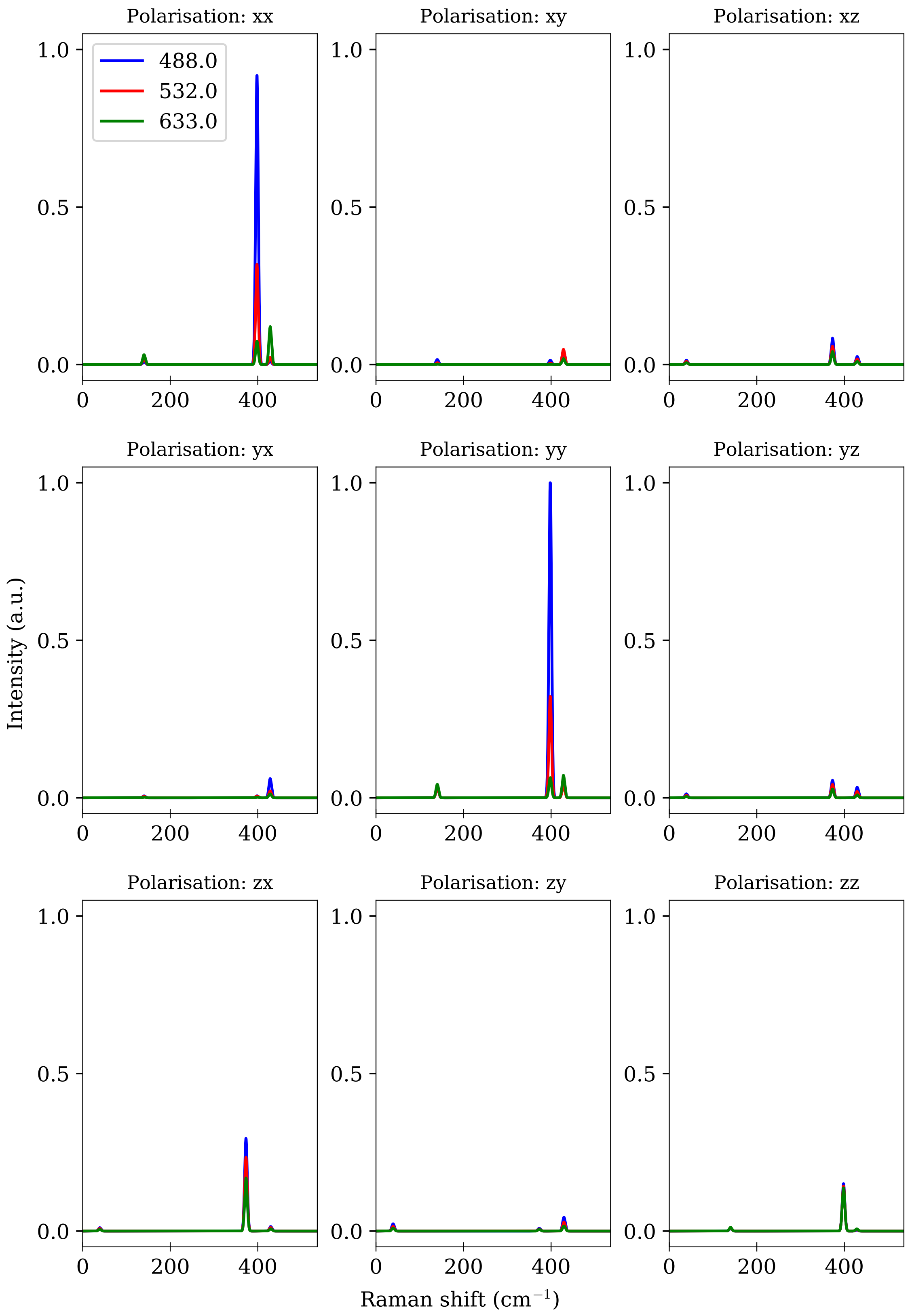

Supplementary Figure 292: Raman spectrum of In_2O_2 with space group 187 and Wyckoff positions h-i-0.

In_2S_2, 164, c-d-0

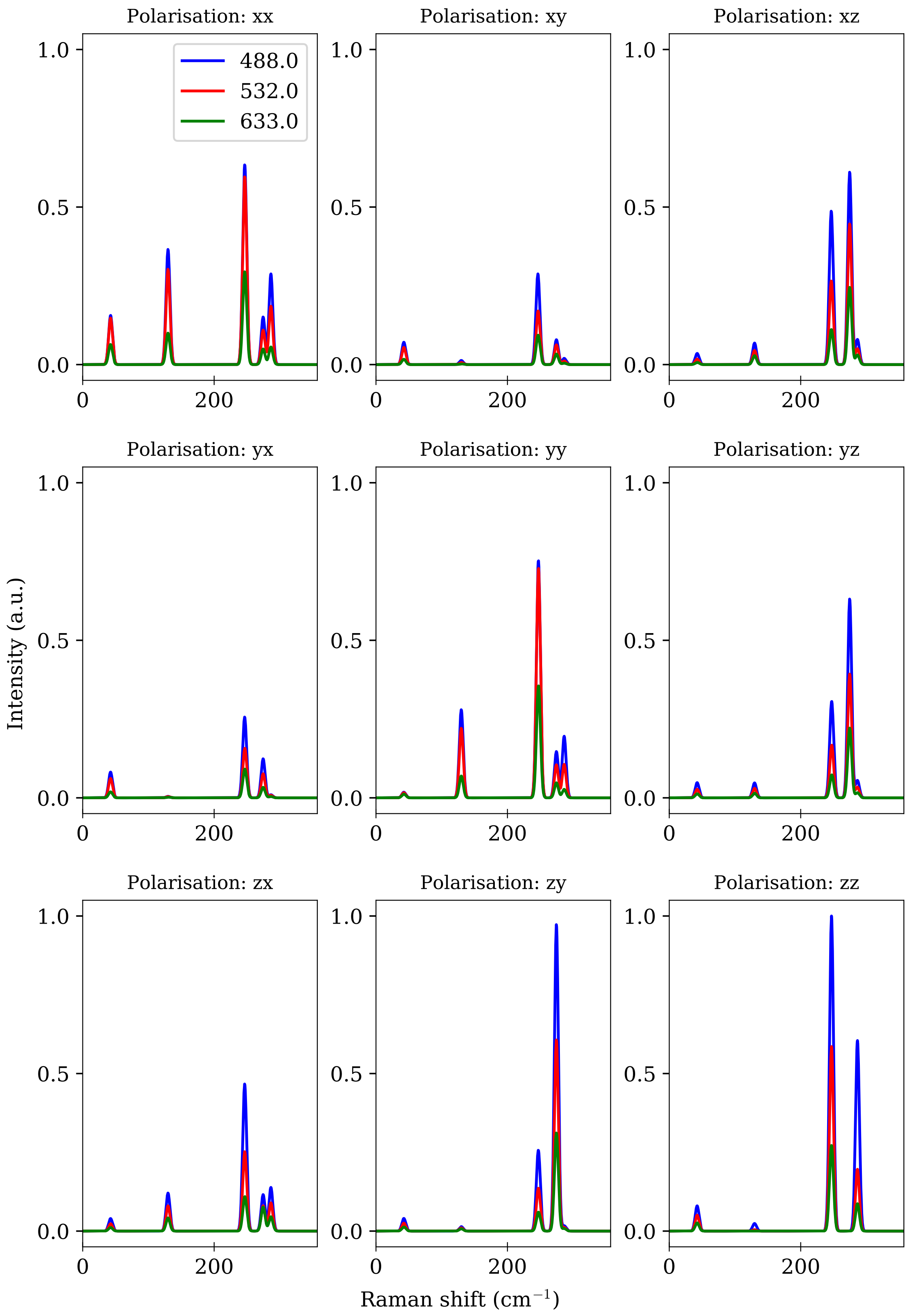

Supplementary Figure 293: Raman spectrum of In_2S_2 with space group 164 and Wyckoff positions c-d-0.

In_2S_2, 187, h-i-0

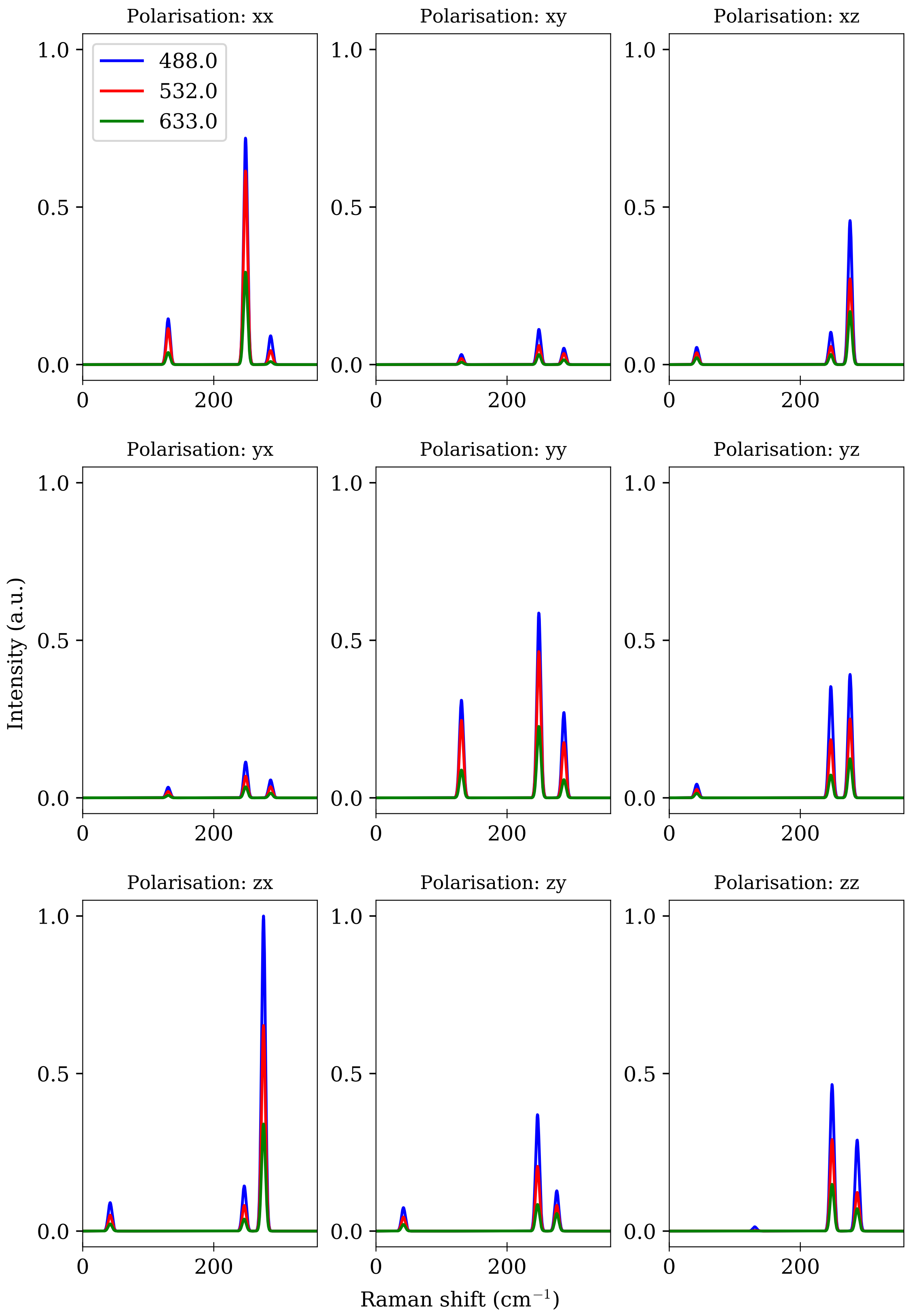

Supplementary Figure 294: Raman spectrum of In_2S_2 with space group 187 and Wyckoff positions h-i-0.

In_2Se_2, 123, b-d-0

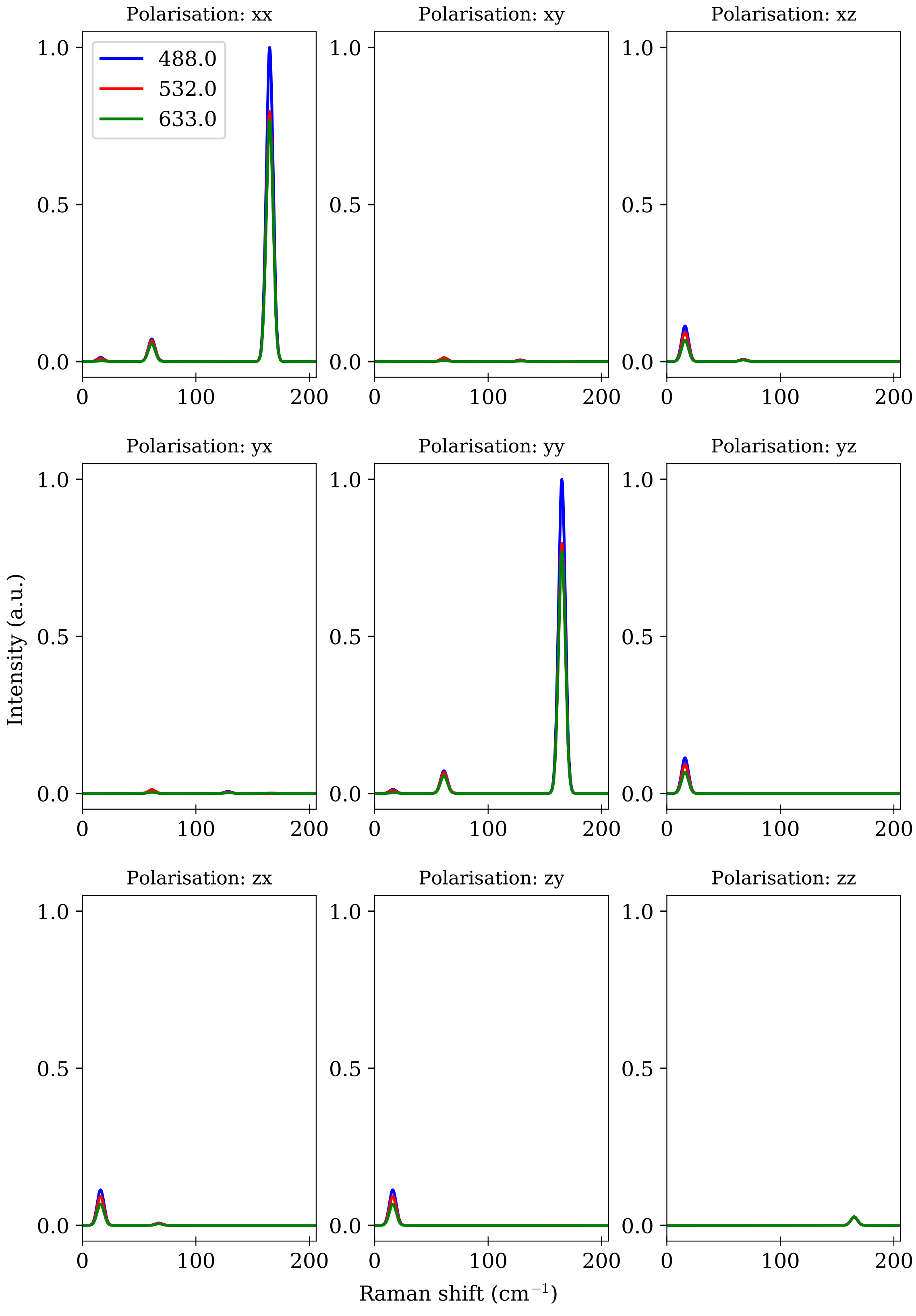

Supplementary Figure 295: Raman spectrum of In_2Se_2 with space group 123 and Wyckoff positions b-d-0.

InAs, 156, b-c-0

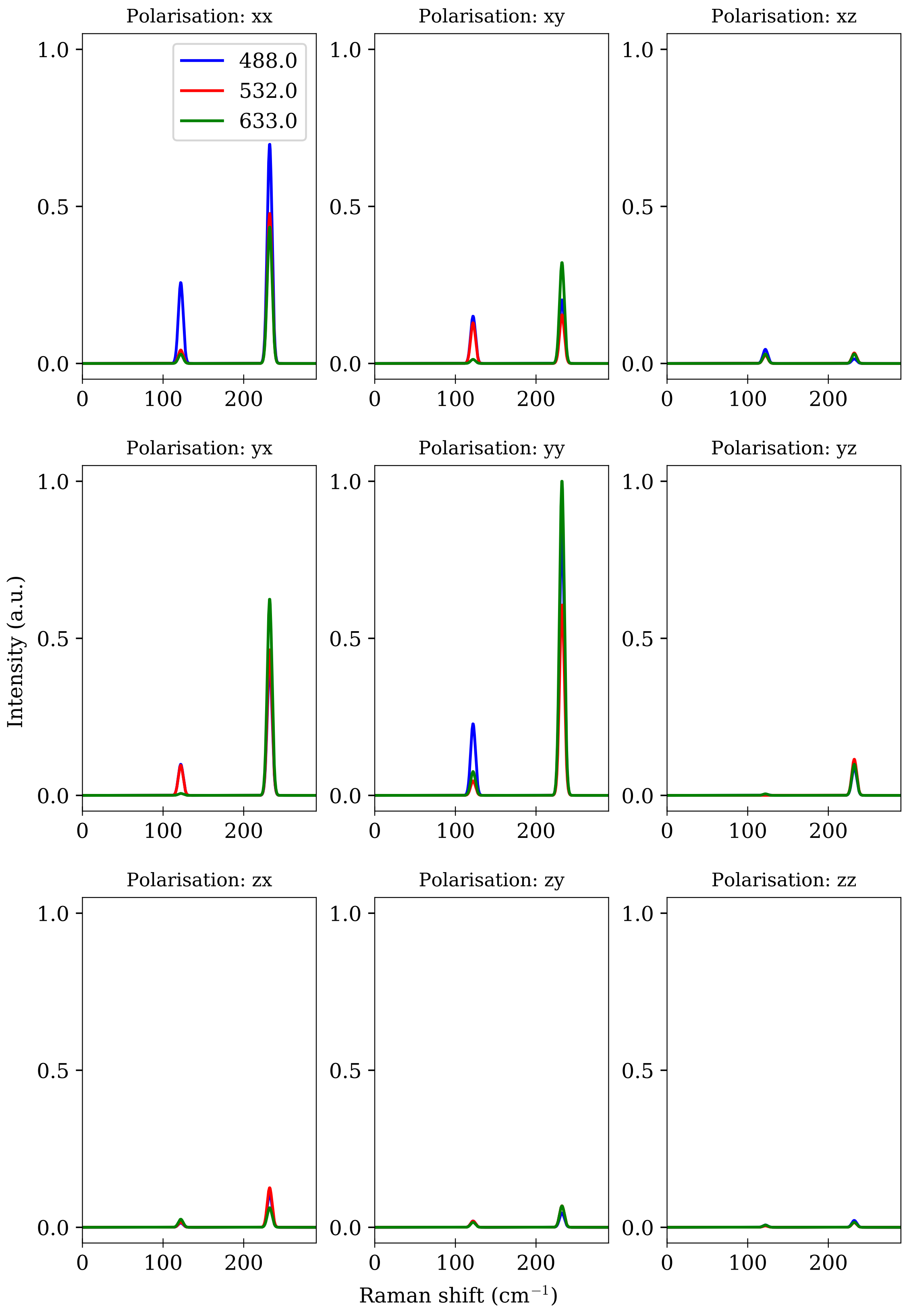

Supplementary Figure 296: Raman spectrum of InAs with space group 156 and Wyckoff positions b-c-0.

InN, 187, d-f-0

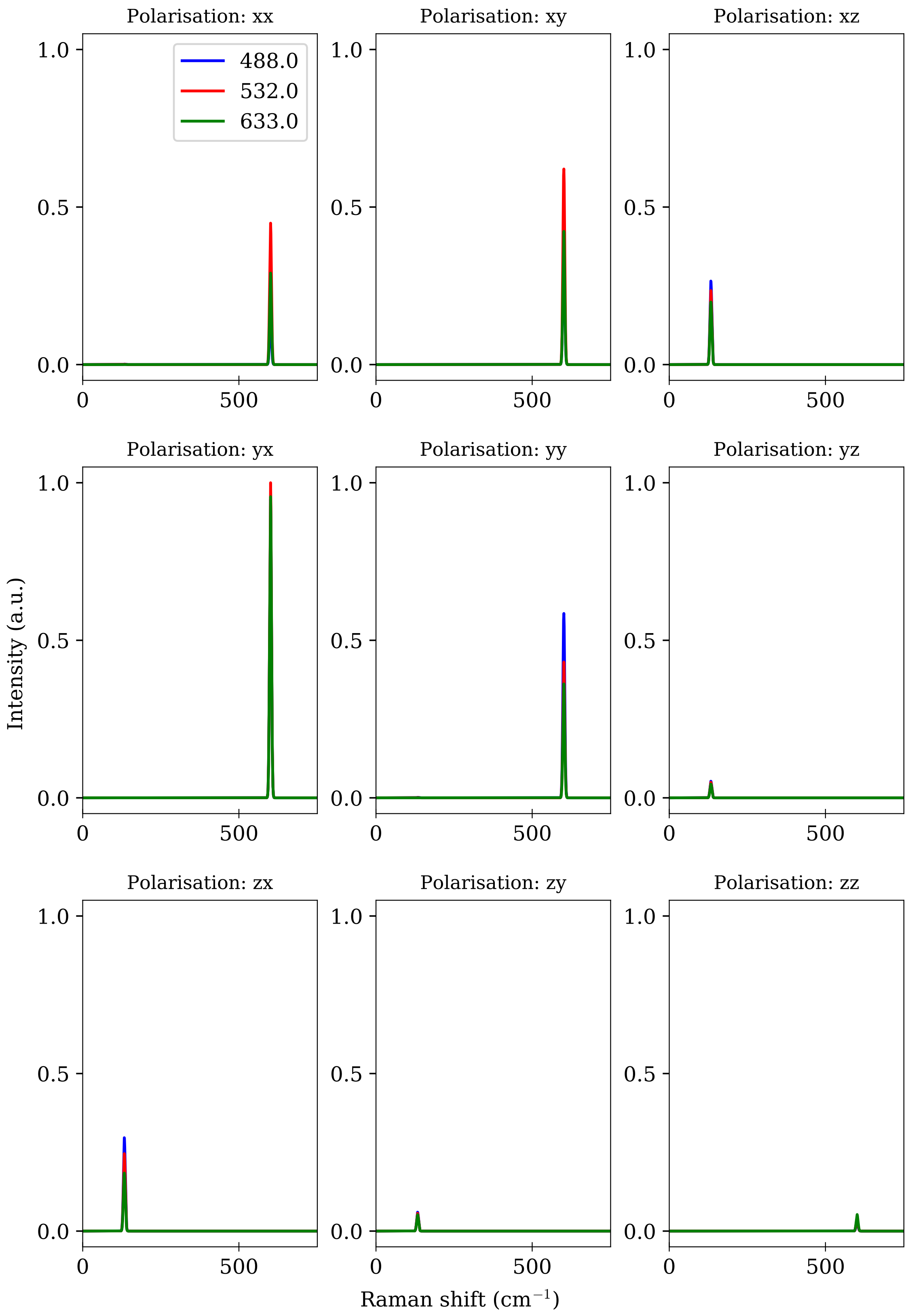

Supplementary Figure 297: Raman spectrum of InN with space group 187 and Wyckoff positions d-f-0.

InP, 156, b-c-0

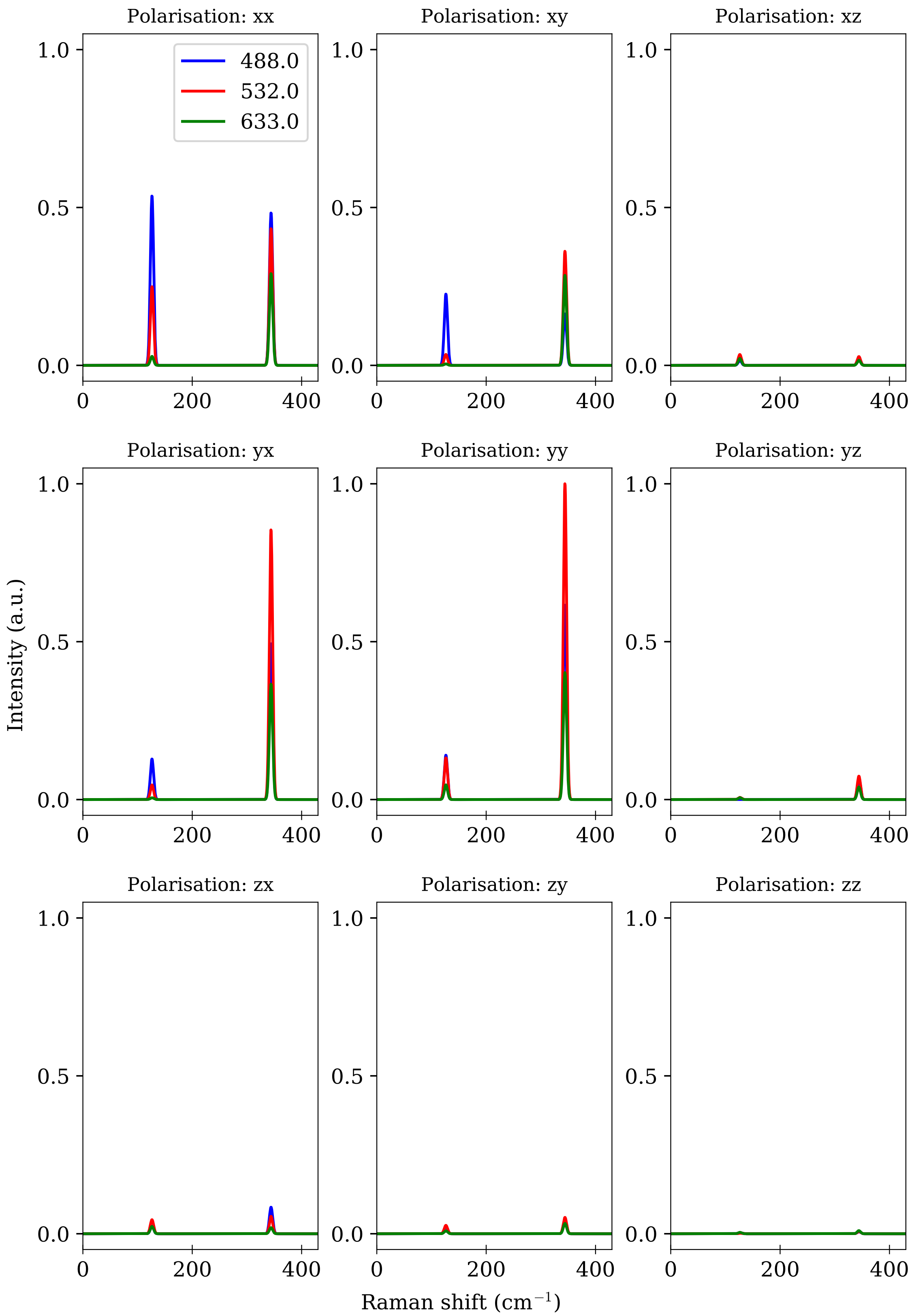

Supplementary Figure 298: Raman spectrum of InP with space group 156 and Wyckoff positions b-c-0.

InSb, 156, b-c-0

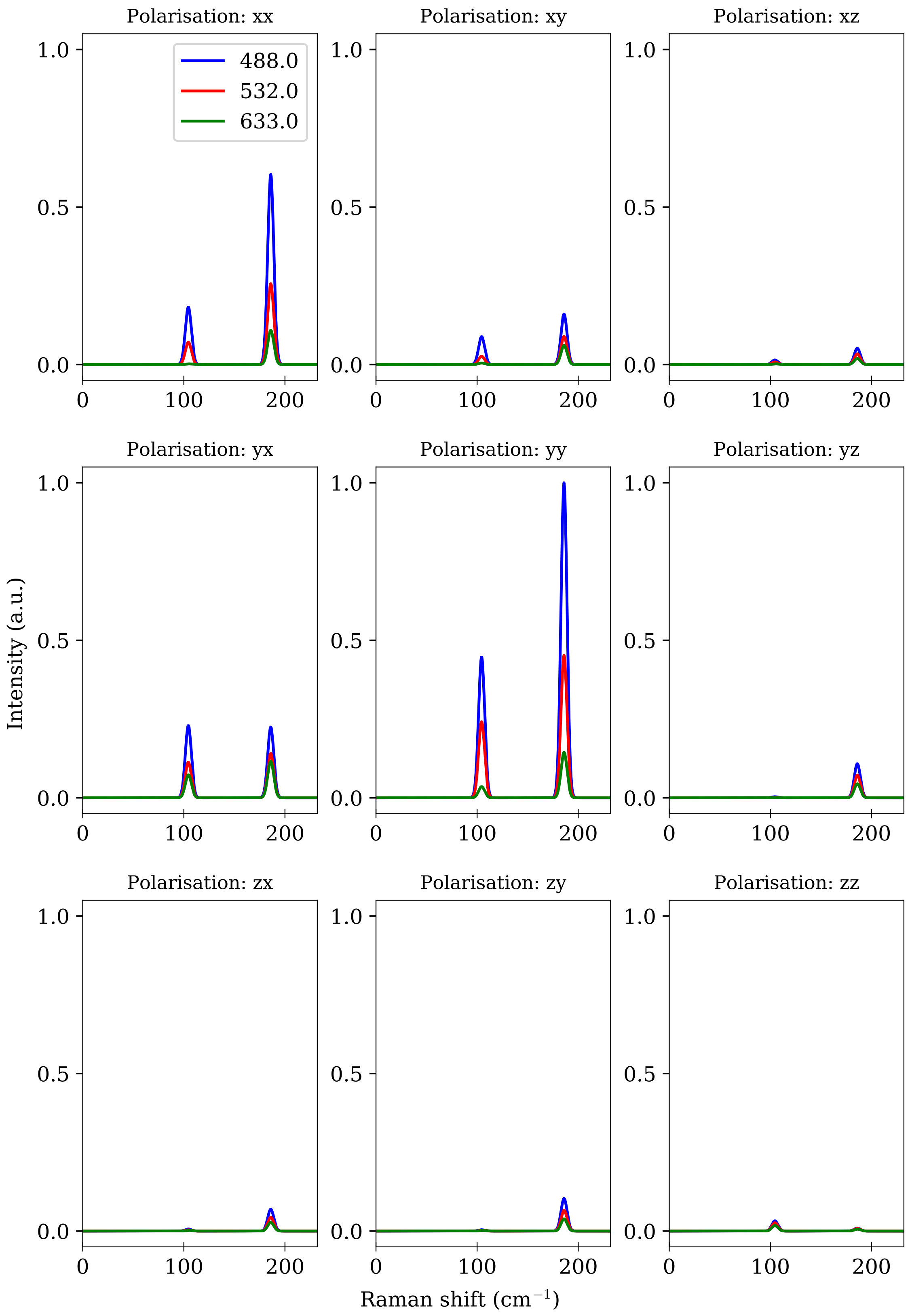

Supplementary Figure 299: Raman spectrum of InSb with space group 156 and Wyckoff positions b-c-0.

$Ir_2Br_2O_2$, 59, a-b-0

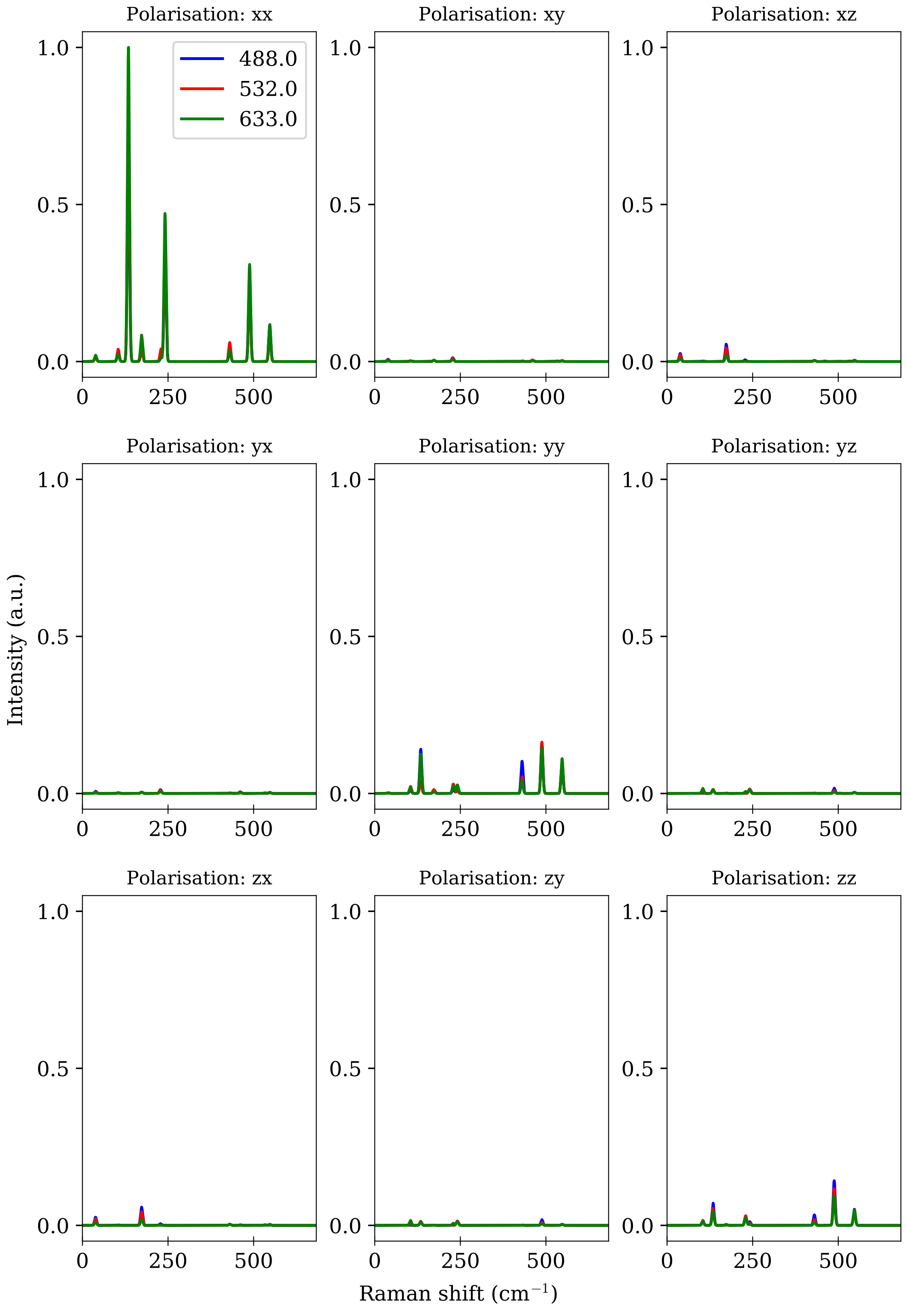

Supplementary Figure 300: Raman spectrum of $Ir_2Br_2O_2$ with space group 59 and Wyckoff positions a-b-0.

$Ir_2Cl_2O_2$, 59, a-b-0

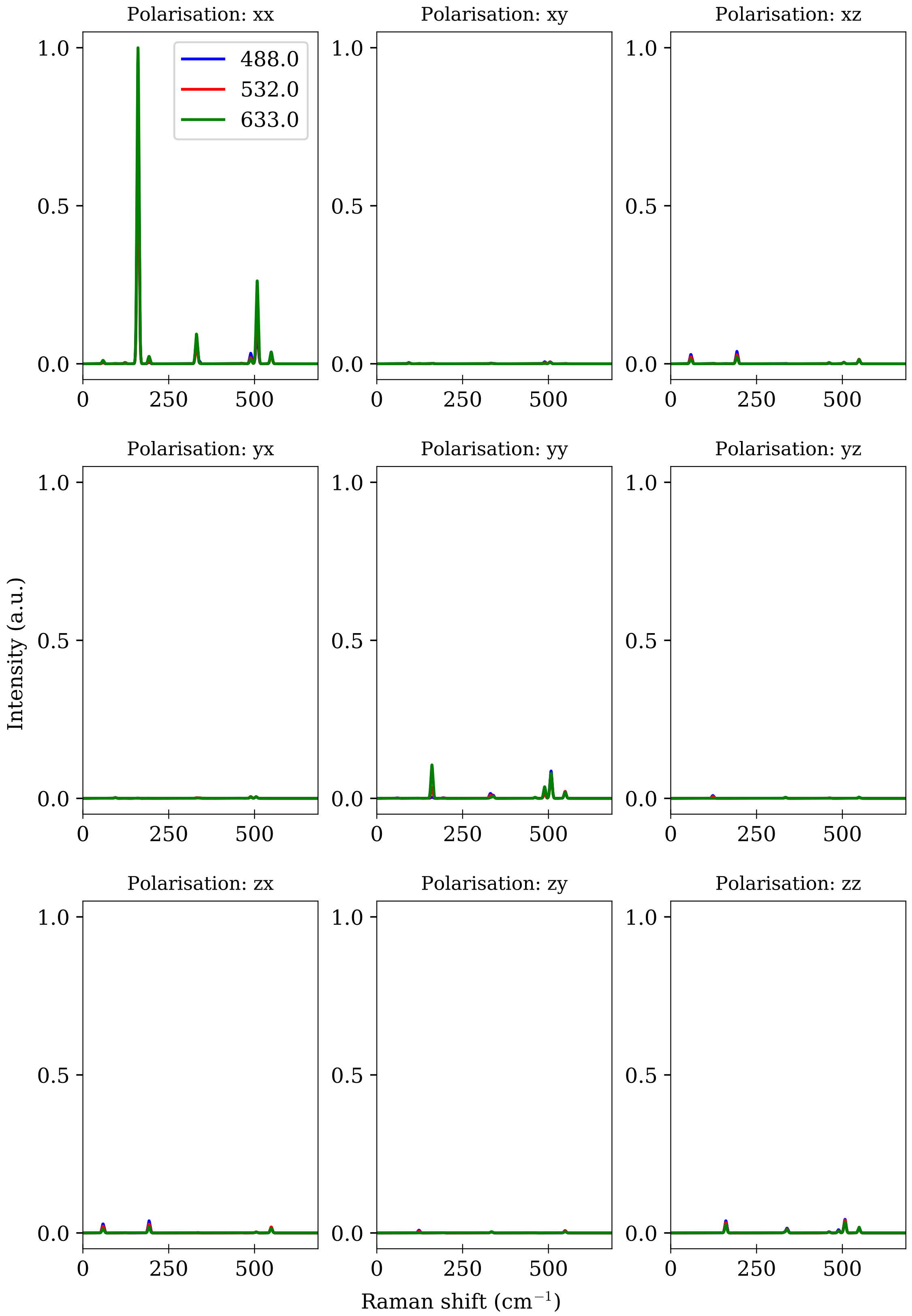

Supplementary Figure 301: Raman spectrum of $Ir_2Cl_2O_2$ with space group 59 and Wyckoff positions a-b-0.

$Ir_2Cl_2Se_2$, 59, a-b-0

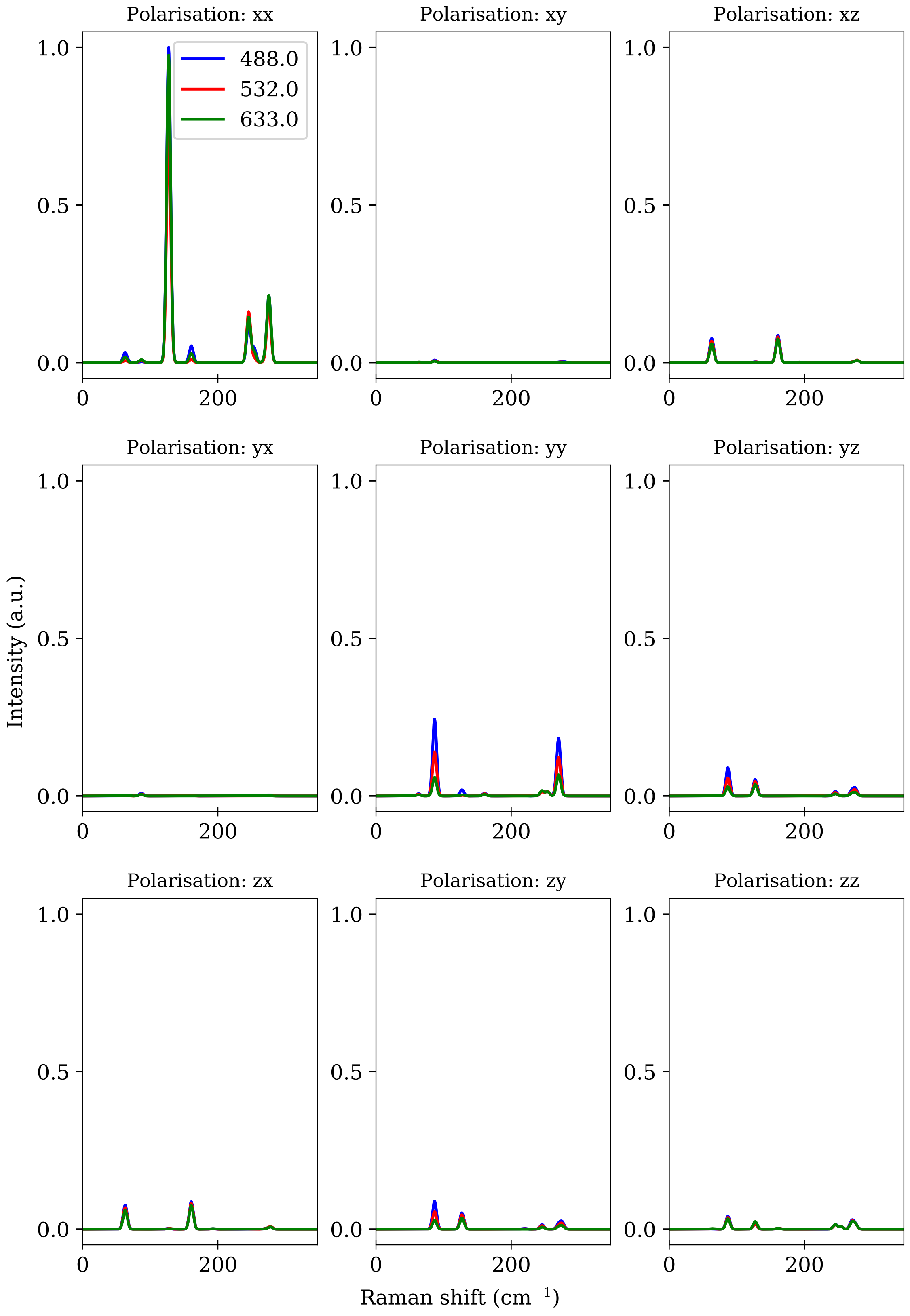

Supplementary Figure 302: Raman spectrum of $Ir_2Cl_2Se_2$ with space group 59 and Wyckoff positions a-b-0.

Ir_2O_4, 11, e-0

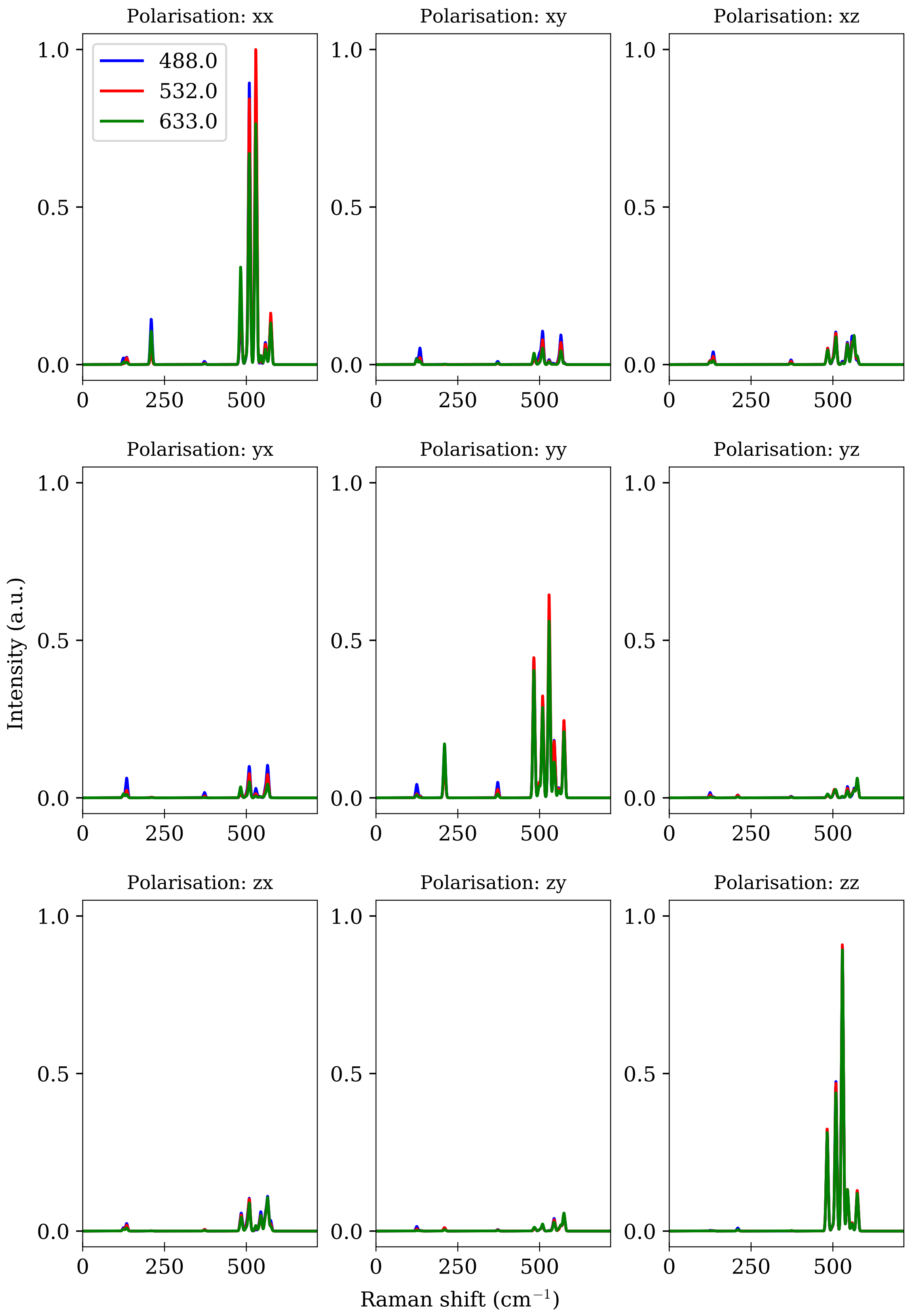

Supplementary Figure 303: Raman spectrum of Ir_2O_4 with space group 11 and Wyckoff positions e-0.

Ir_2S_2, 164, d-0

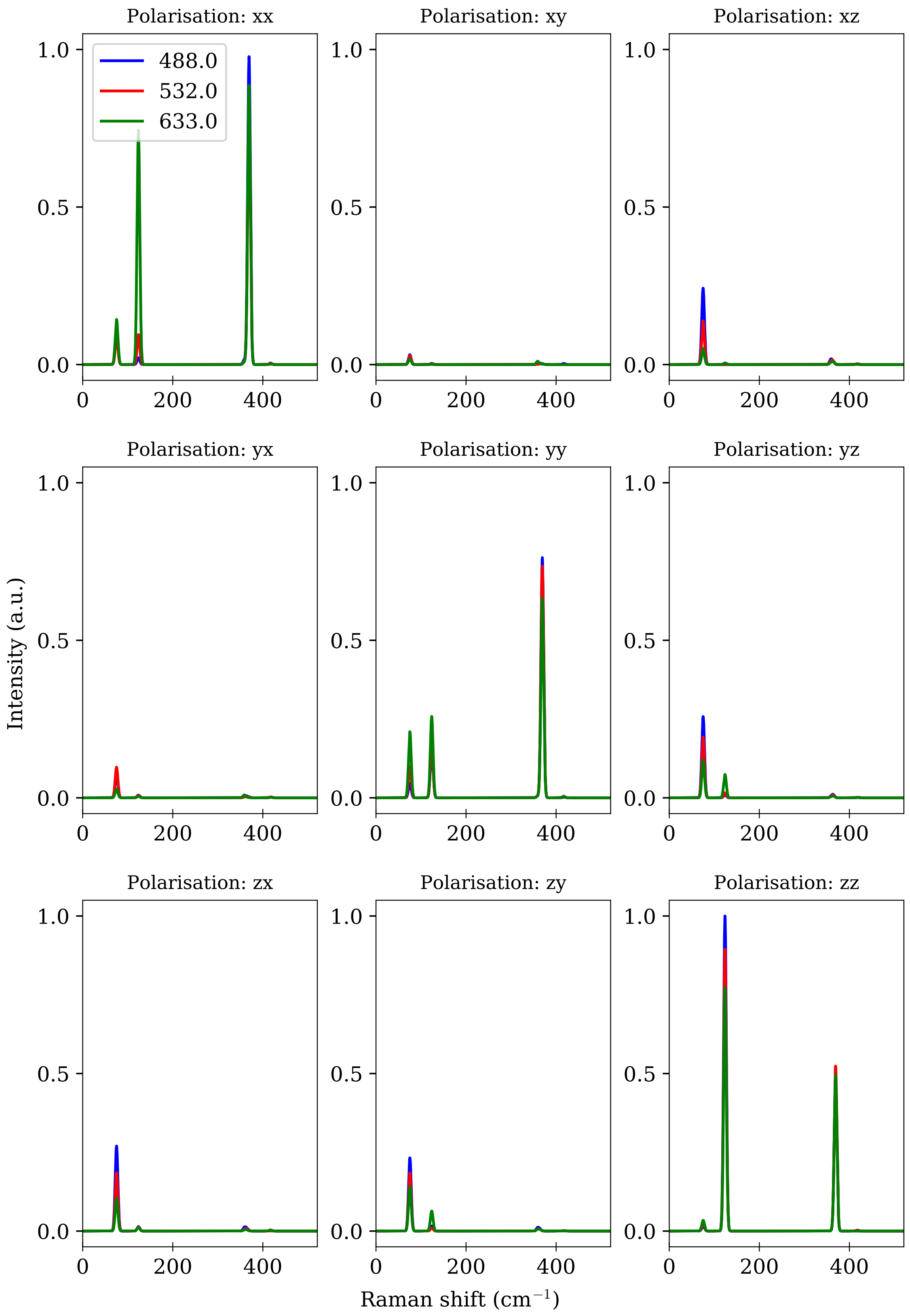

Supplementary Figure 304: Raman spectrum of Ir_2S_2 with space group 164 and Wyckoff positions d-0.

Ir_2S_2, 6, a-b-0

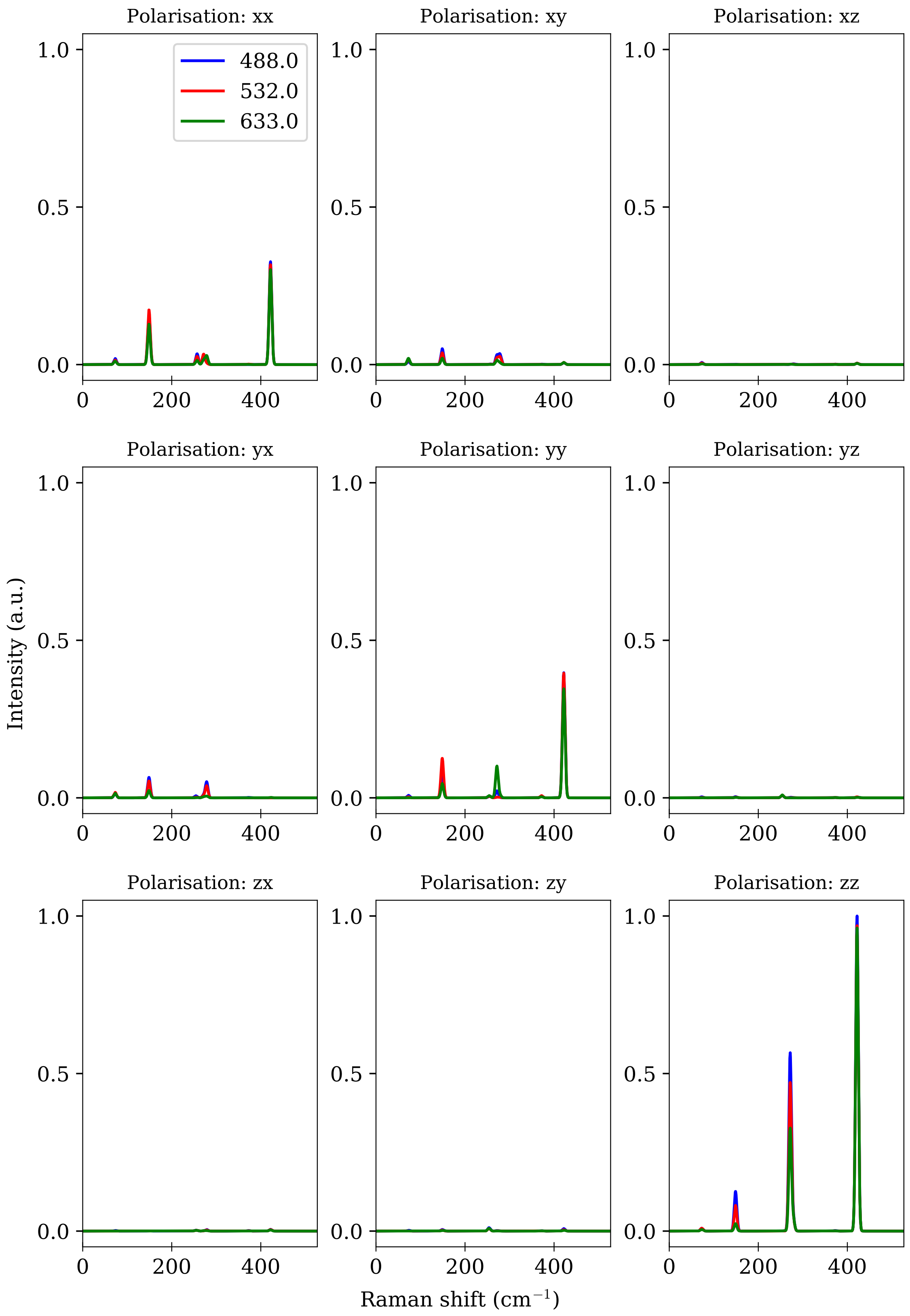

Supplementary Figure 305: Raman spectrum of Ir_2S_2 with space group 6 and Wyckoff positions a-b-0.

Ir_2Se_2, 12, i-0

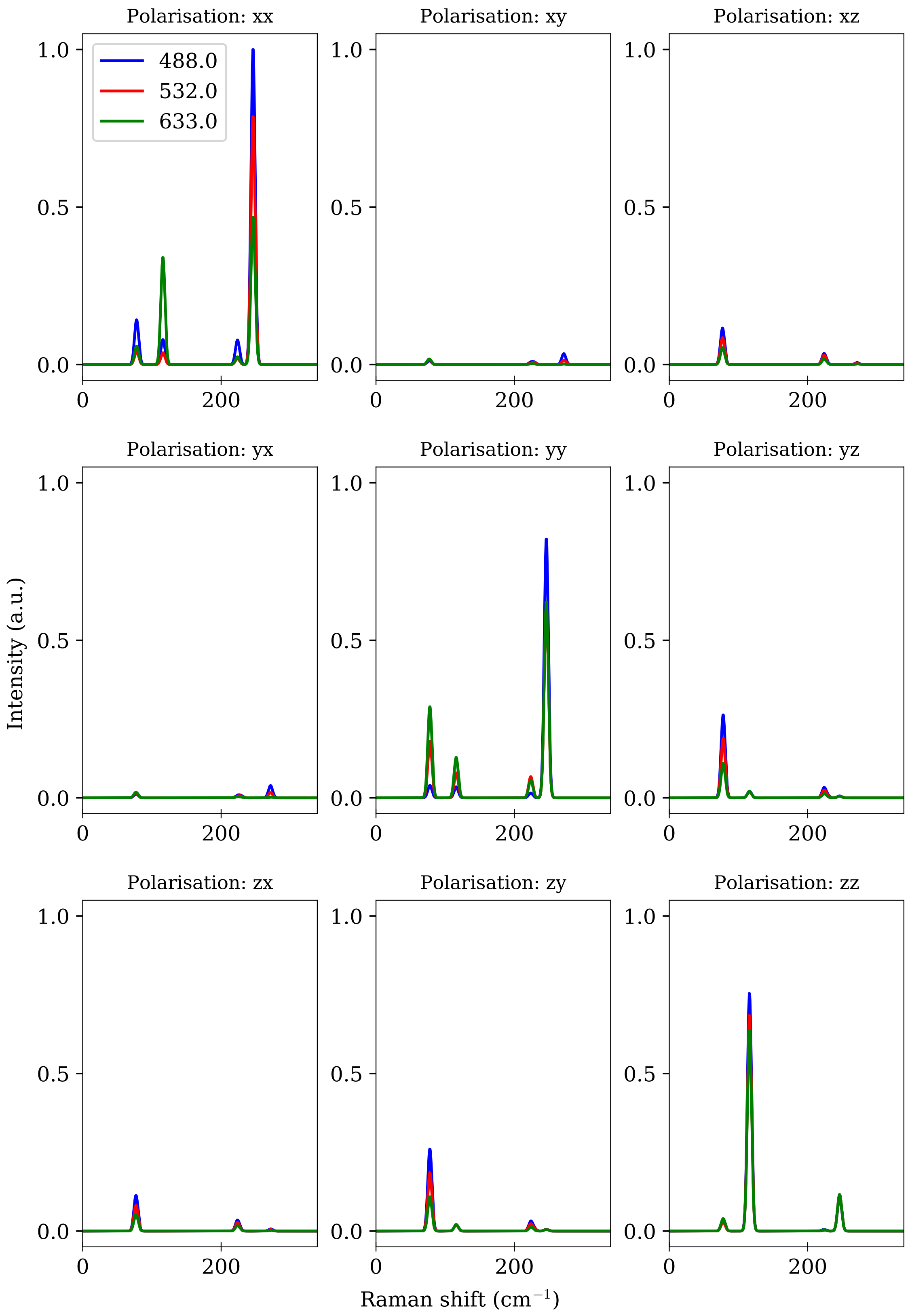

Supplementary Figure 306: Raman spectrum of Ir_2Se_2 with space group 12 and Wyckoff positions i-0.

Ir_2Te_2, 12, i-0

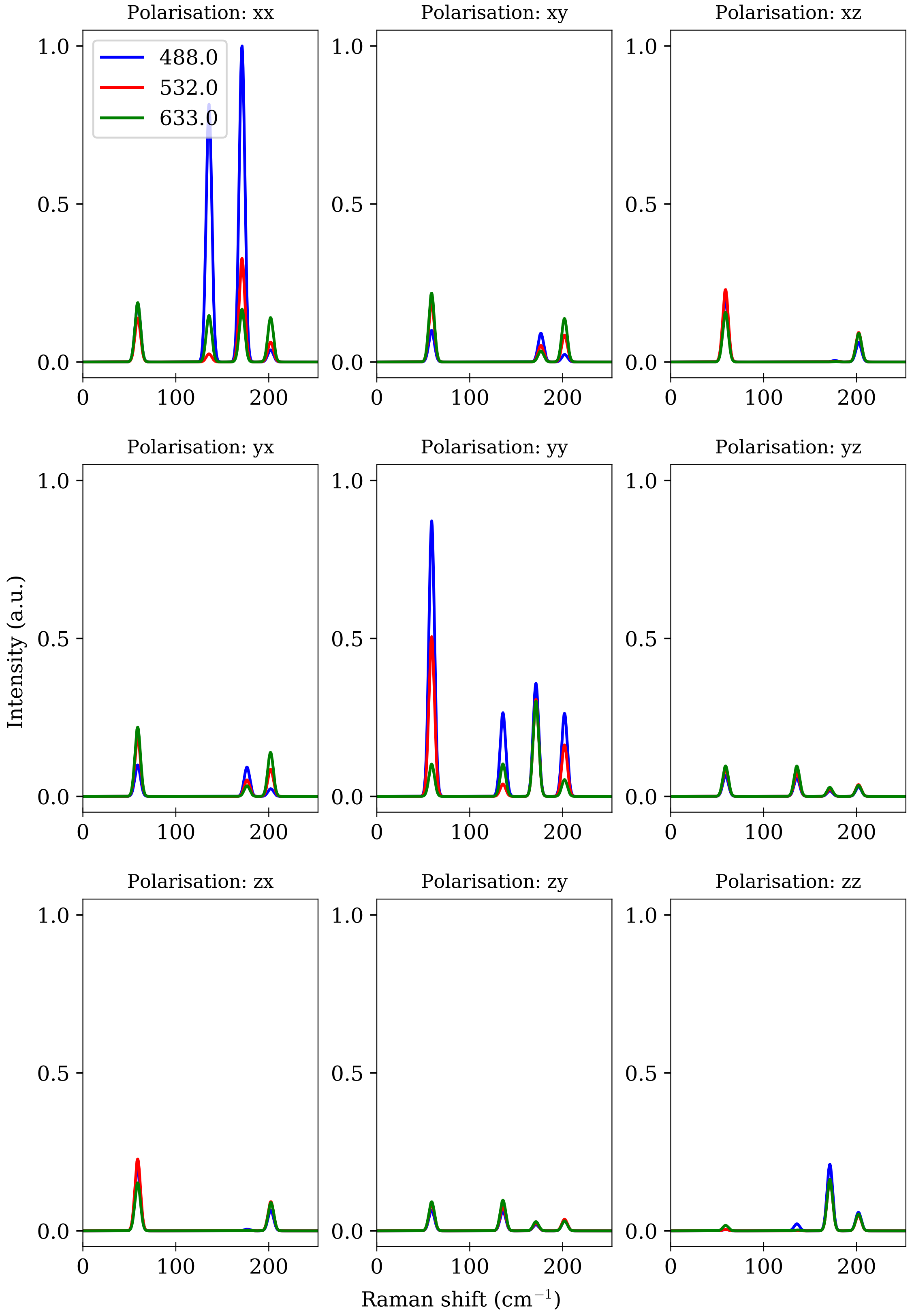

Supplementary Figure 307: Raman spectrum of Ir_2Te_2 with space group 12 and Wyckoff positions i-0.

IrH_2, 187, b-i-0

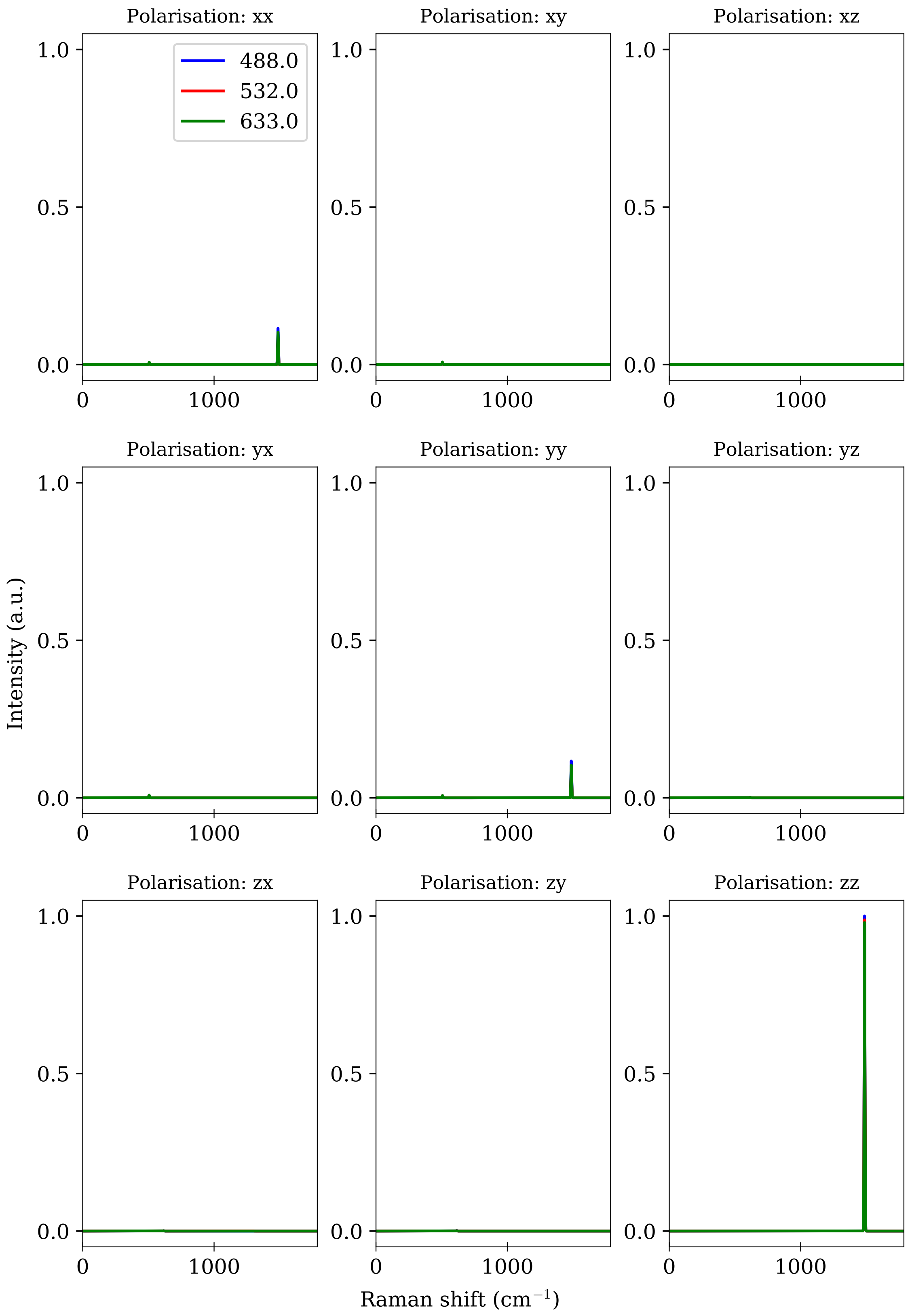

Supplementary Figure 308: Raman spectrum of IrH_2 with space group 187 and Wyckoff positions b-i-0.

ISbSe, 156, a-b-c-0

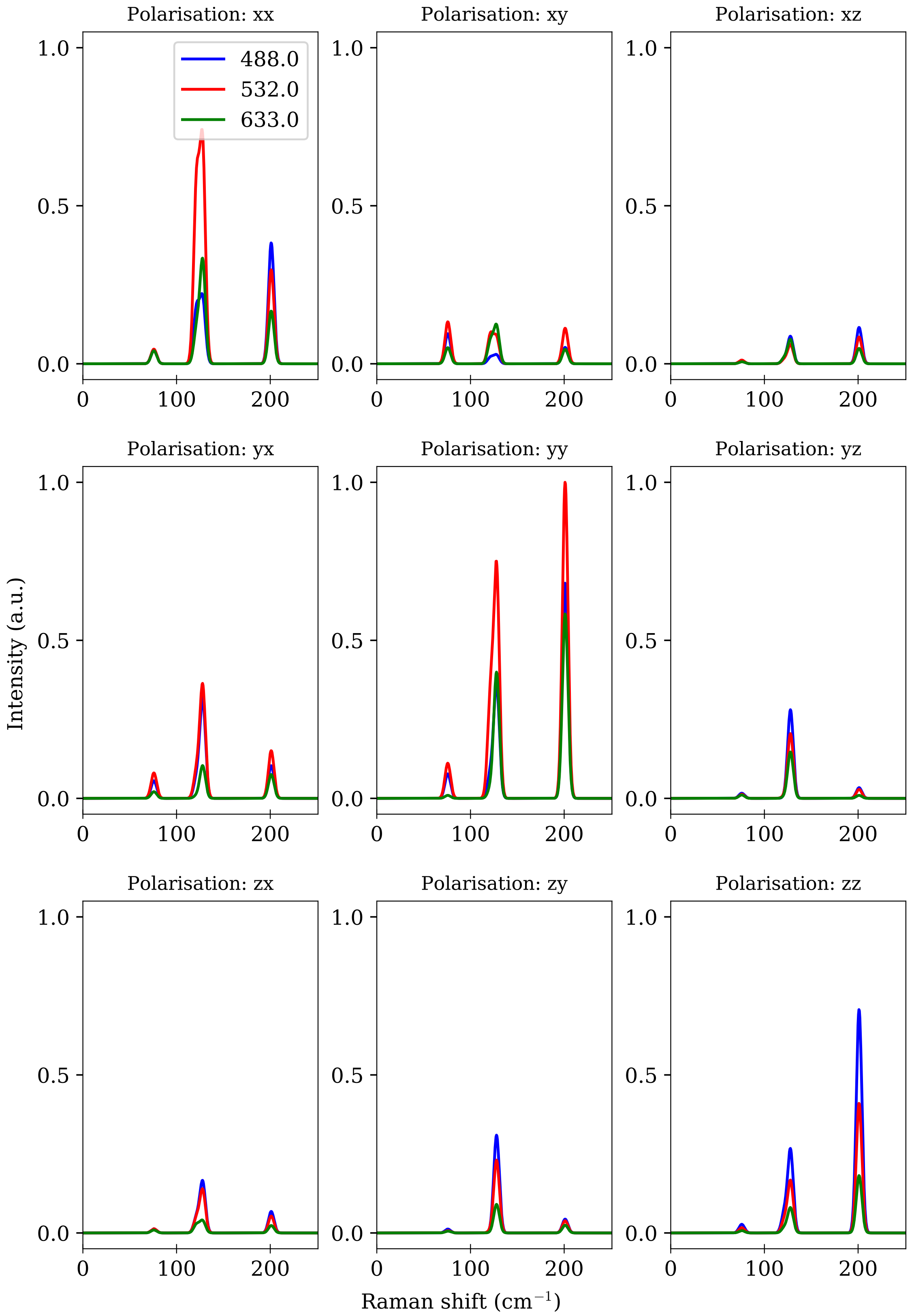

Supplementary Figure 309: Raman spectrum of ISbSe with space group 156 and Wyckoff positions a-b-c-0.

ISbSe, 156, a-c-0

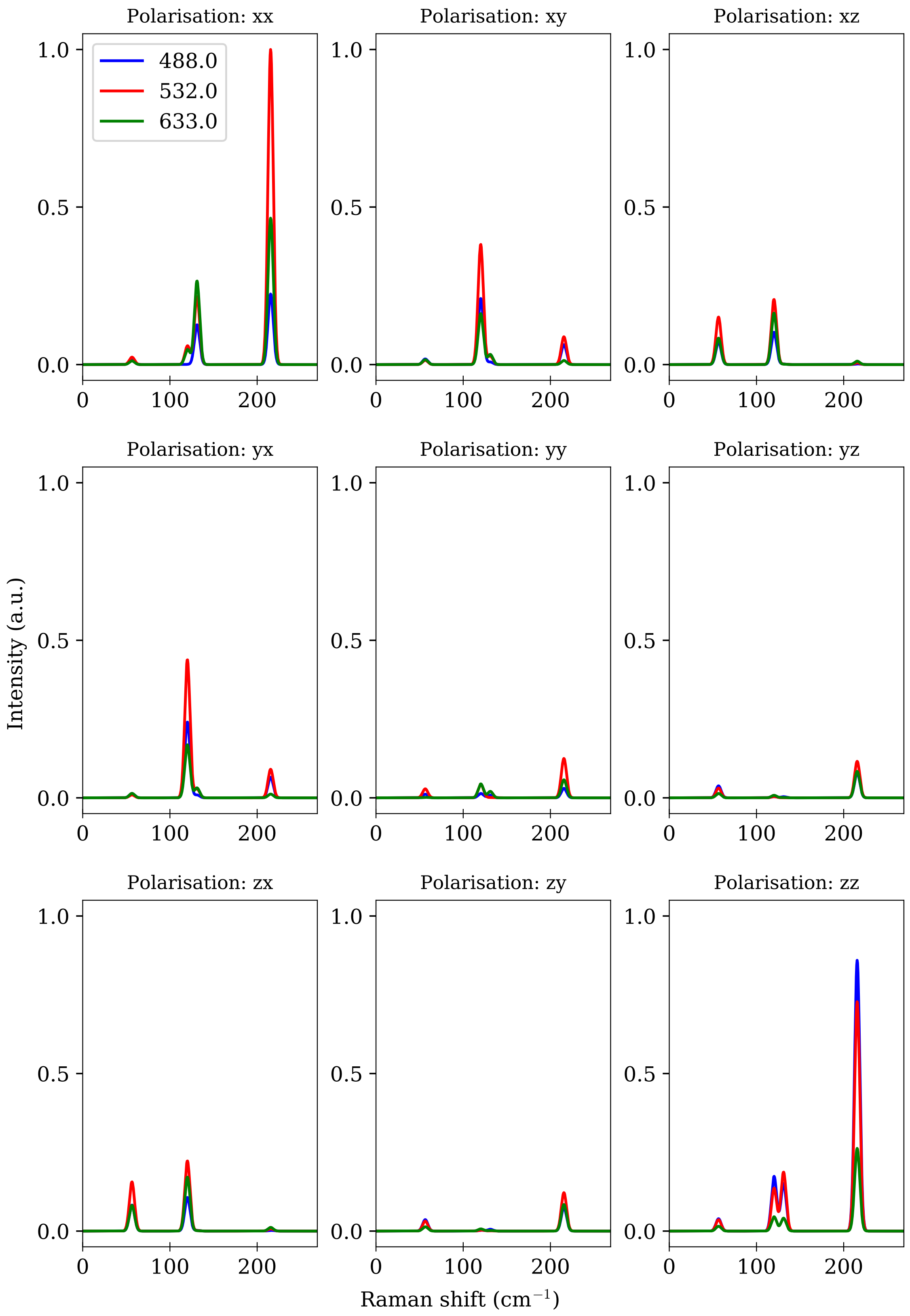

Supplementary Figure 310: Raman spectrum of ISbSe with space group 156 and Wyckoff positions a-c-0.

ISbTe, 156, a-b-c-0

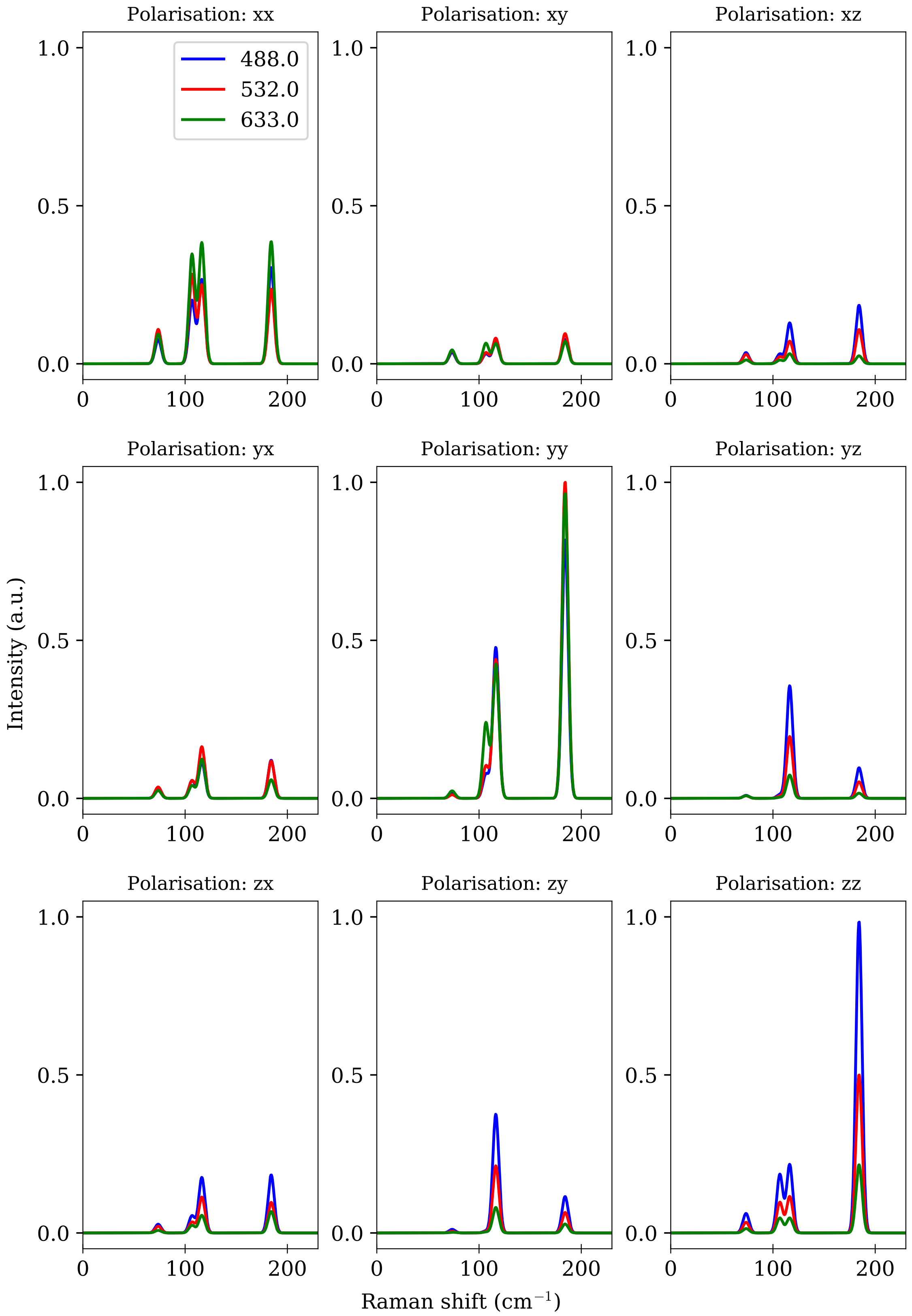

Supplementary Figure 311: Raman spectrum of ISbTe with space group 156 and Wyckoff positions a-b-c-0.

ISbTe, 156, a-c-0

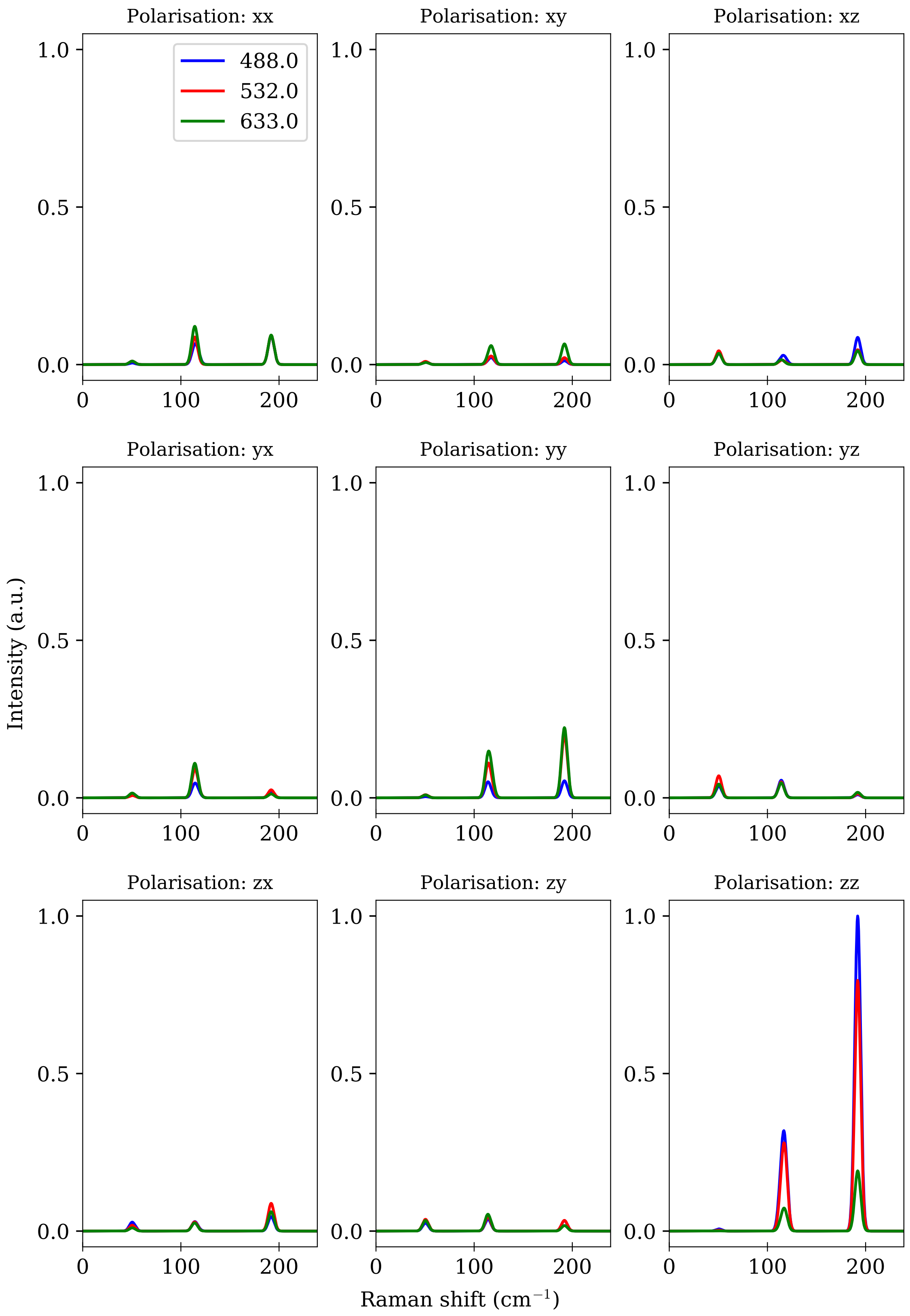

Supplementary Figure 312: Raman spectrum of ISbTe with space group 156 and Wyckoff positions a-c-0.

ISSb, 156, a-b-c-0

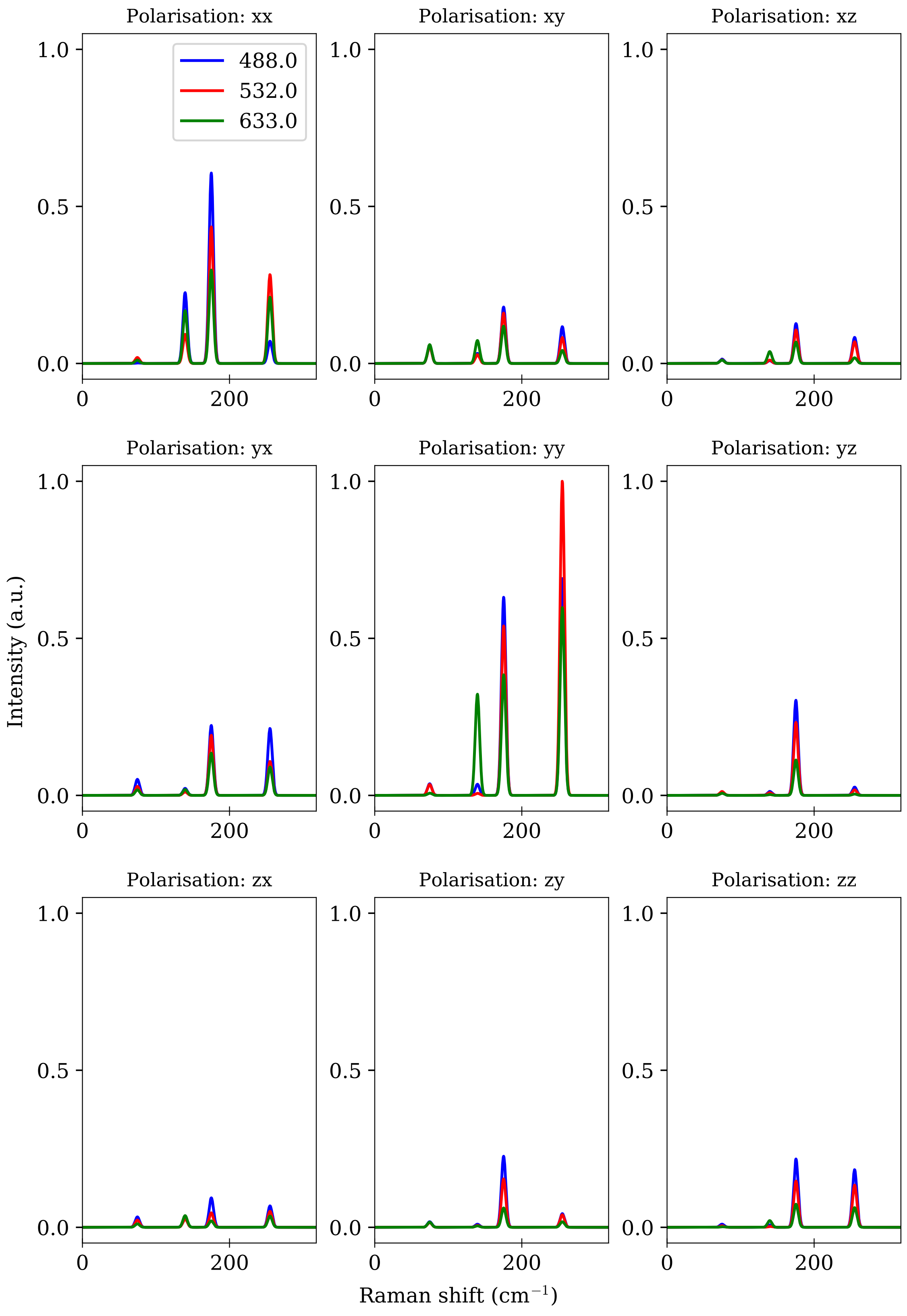

Supplementary Figure 313: Raman spectrum of ISSb with space group 156 and Wyckoff positions a-b-c-0.

ISSb, 156, a-c-0

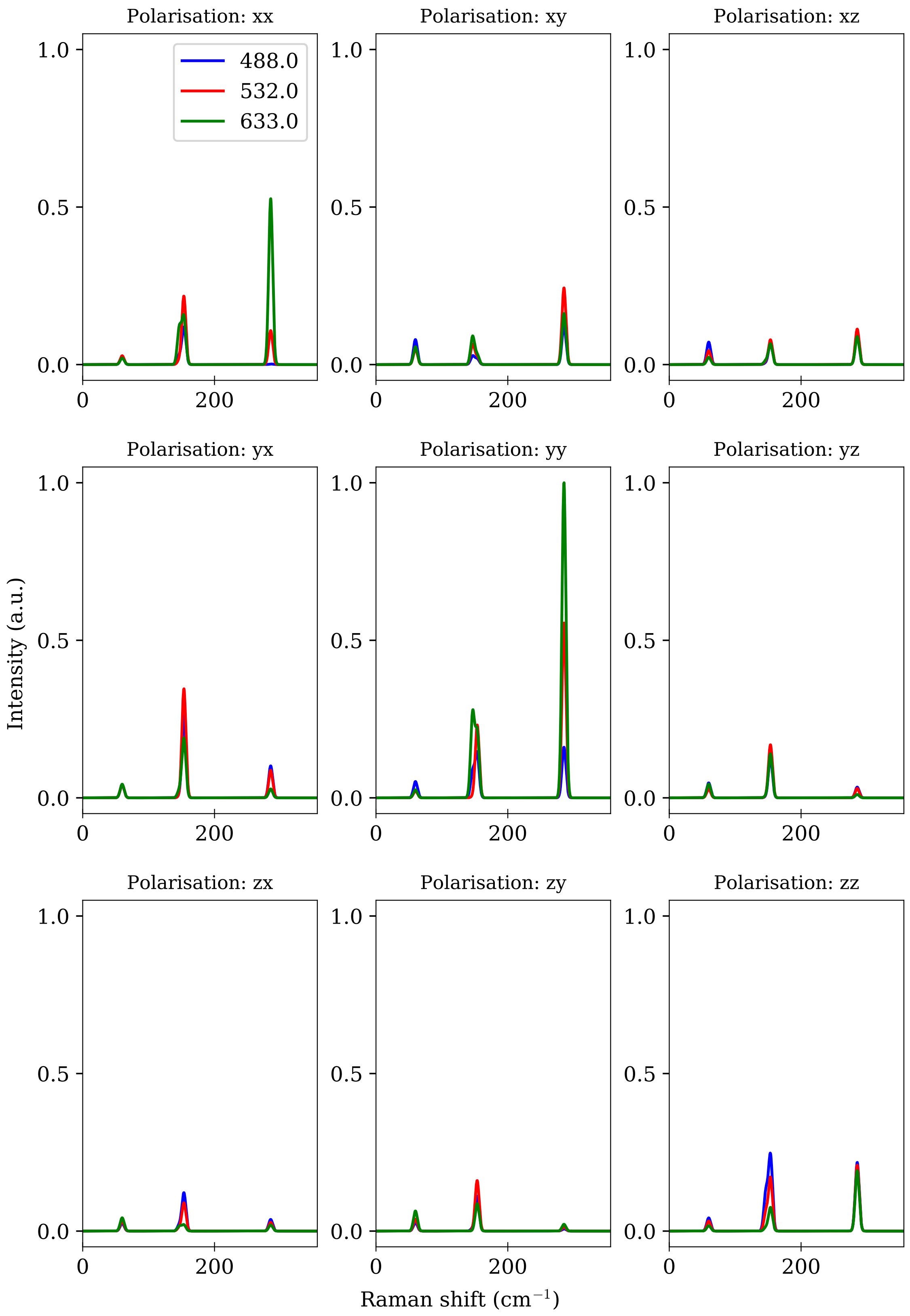

Supplementary Figure 314: Raman spectrum of ISSb with space group 156 and Wyckoff positions a-c-0.

Li_2Ni, 187, b-i-0

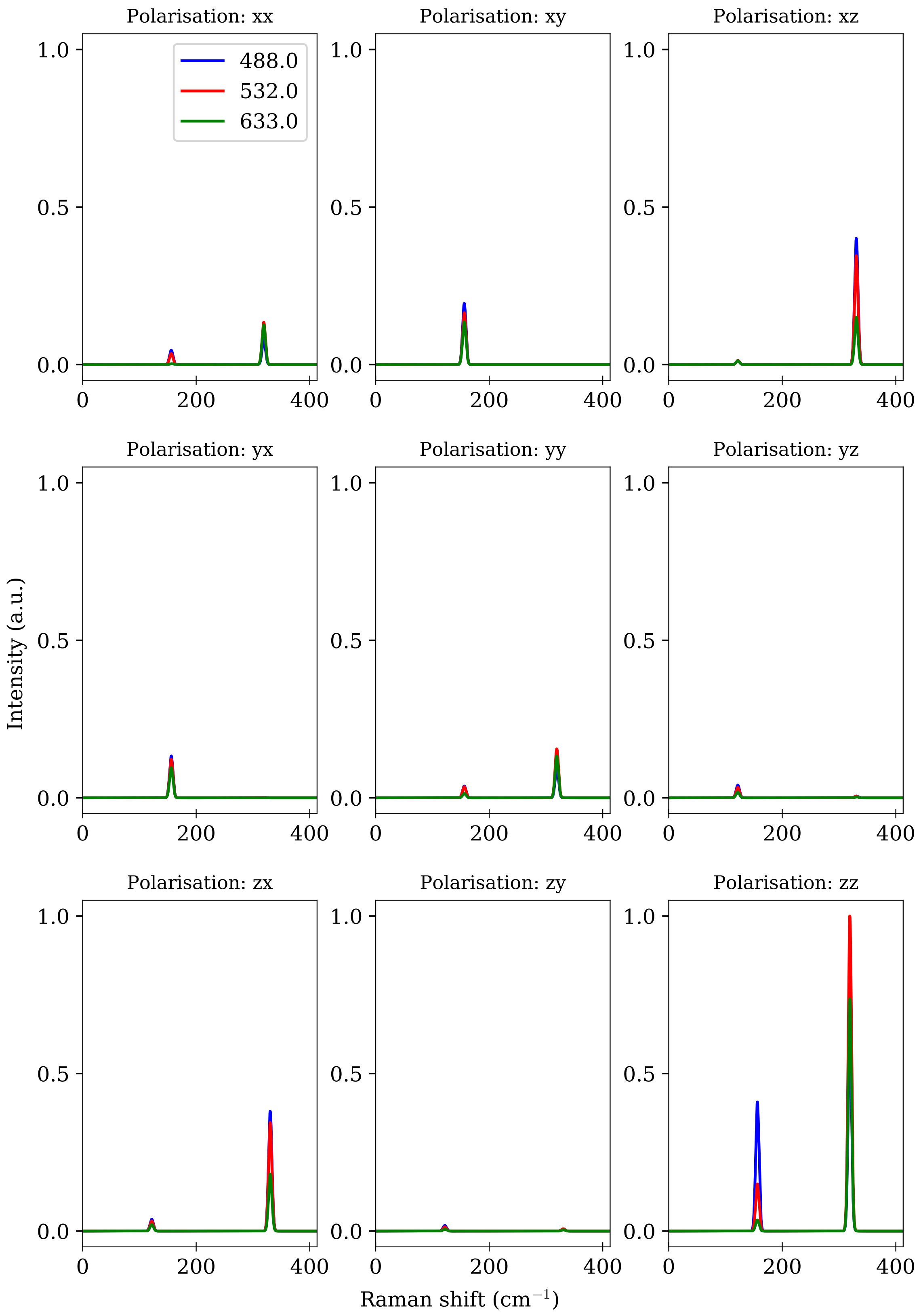

Supplementary Figure 315: Raman spectrum of Li_2Ni with space group 187 and Wyckoff positions b-i-0.

Li_2Os, 187, b-i-0

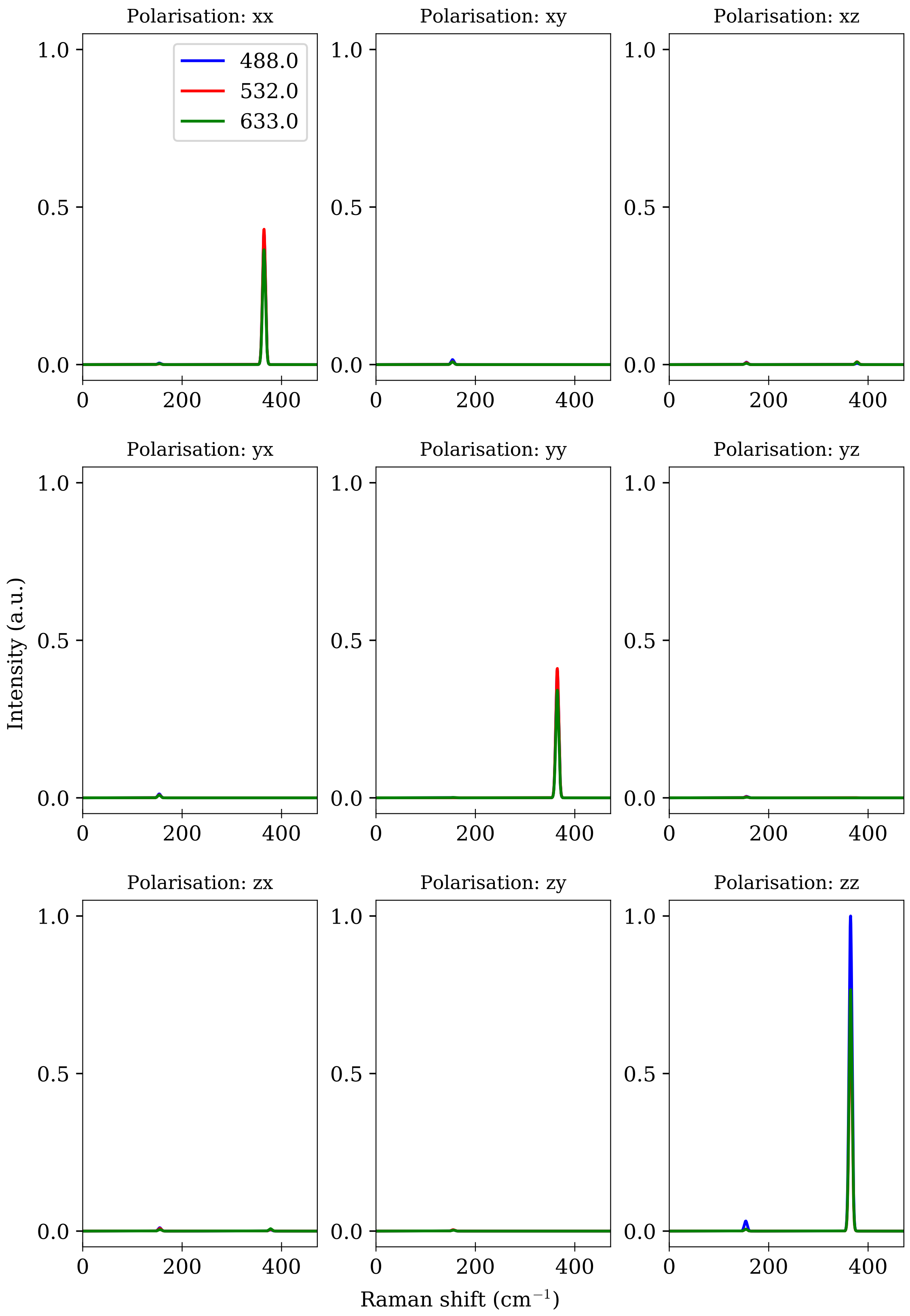

Supplementary Figure 316: Raman spectrum of Li_2Os with space group 187 and Wyckoff positions b-i-0.

Li_2Pt, 187, b-i-0

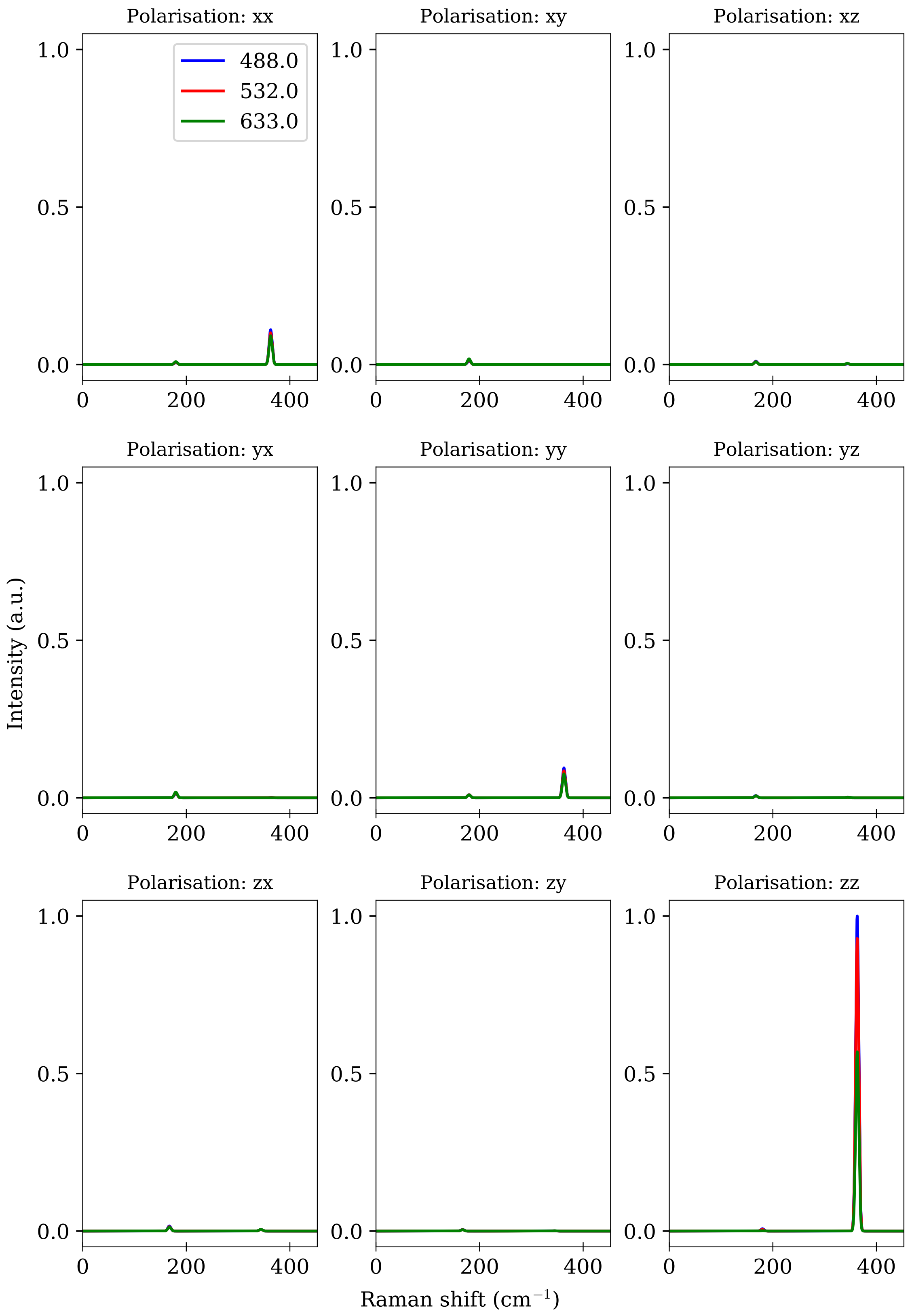

Supplementary Figure 317: Raman spectrum of Li_2Pt with space group 187 and Wyckoff positions b-i-0.

Li_2Ru, 187, b-i-0

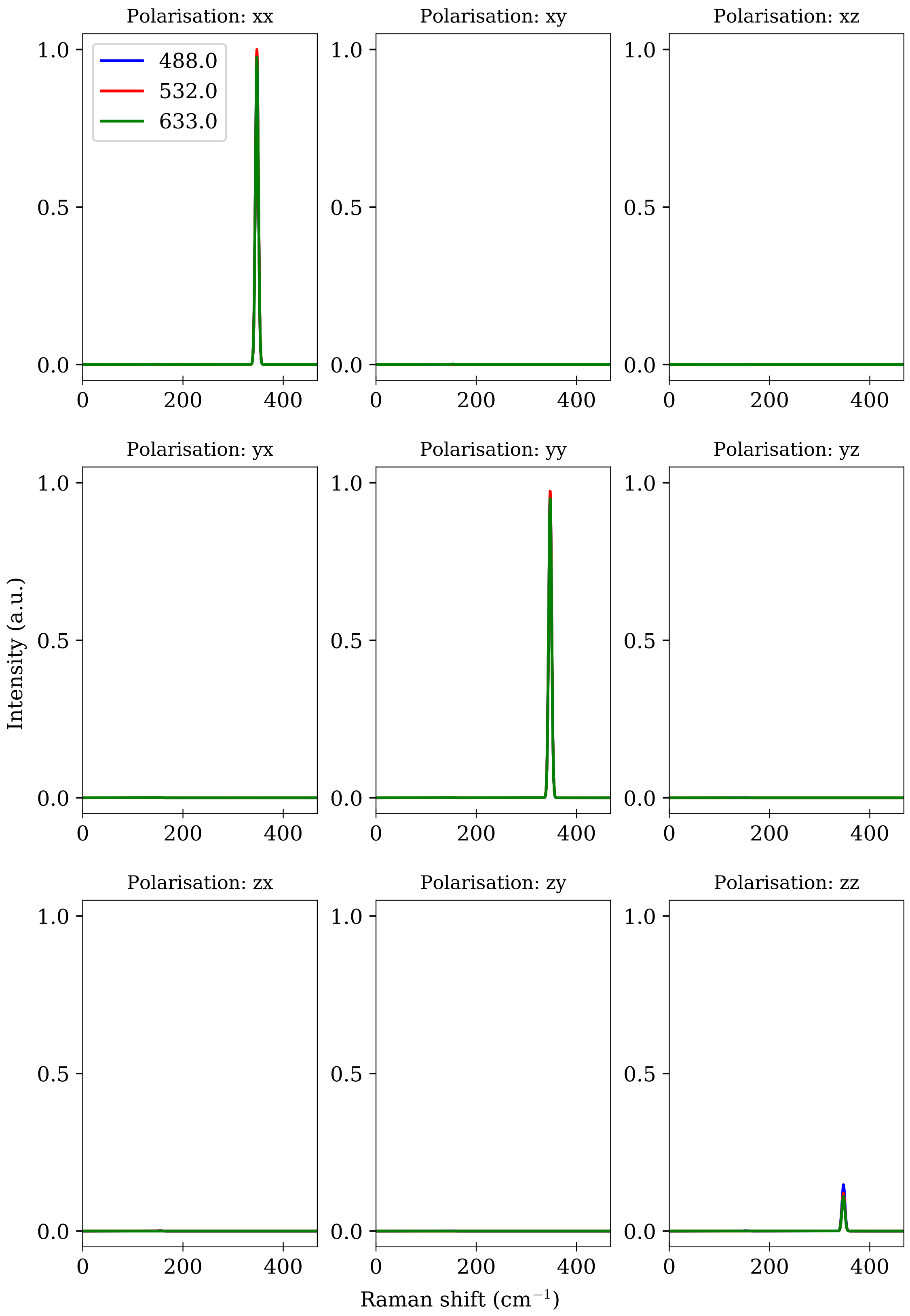

Supplementary Figure 318: Raman spectrum of Li_2Ru with space group 187 and Wyckoff positions b-i-0.

Li_2Ti, 187, a-i-0

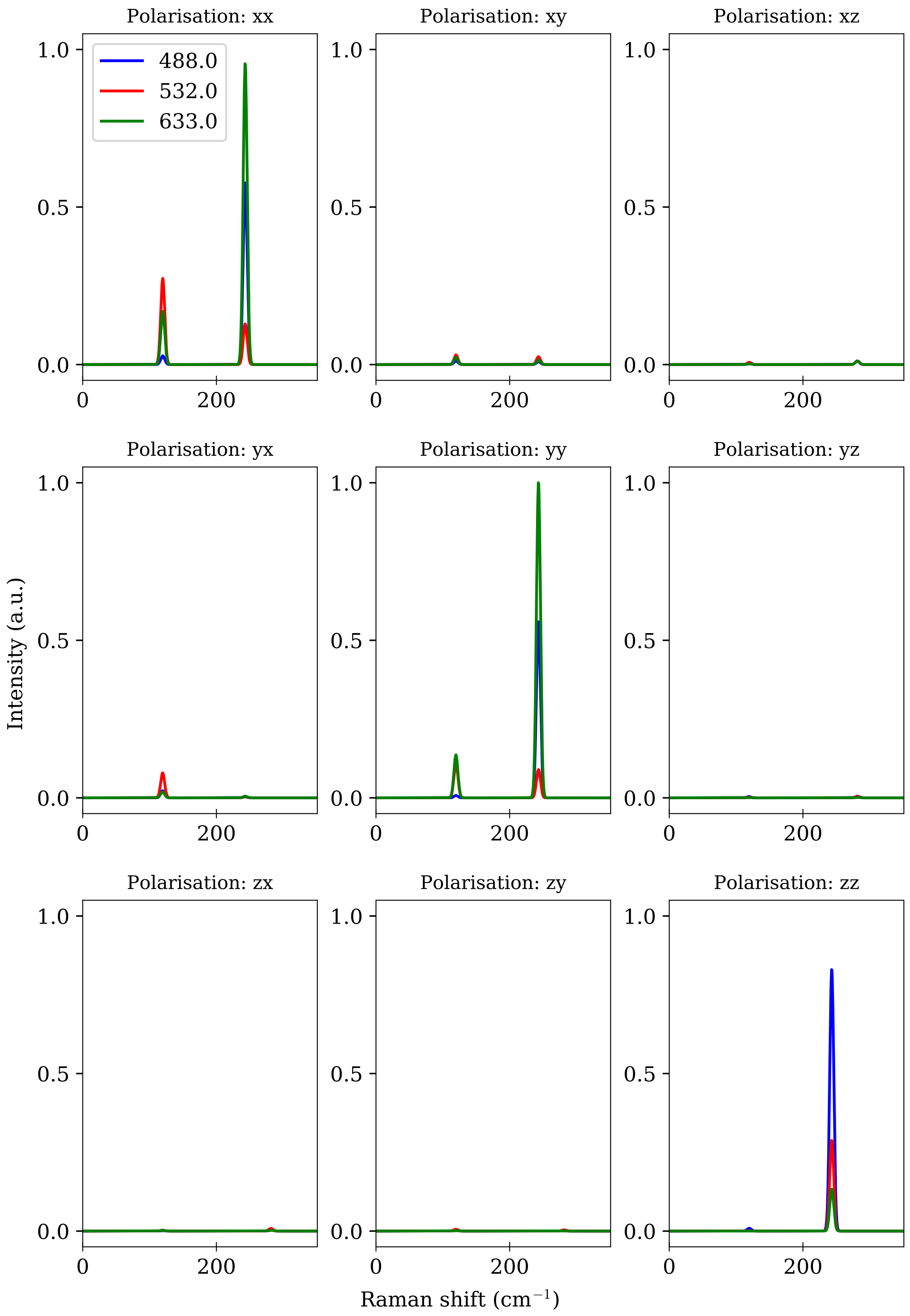

Supplementary Figure 319: Raman spectrum of Li_2Ti with space group 187 and Wyckoff positions a-i-0.

Li_2V, 187, a-i-0

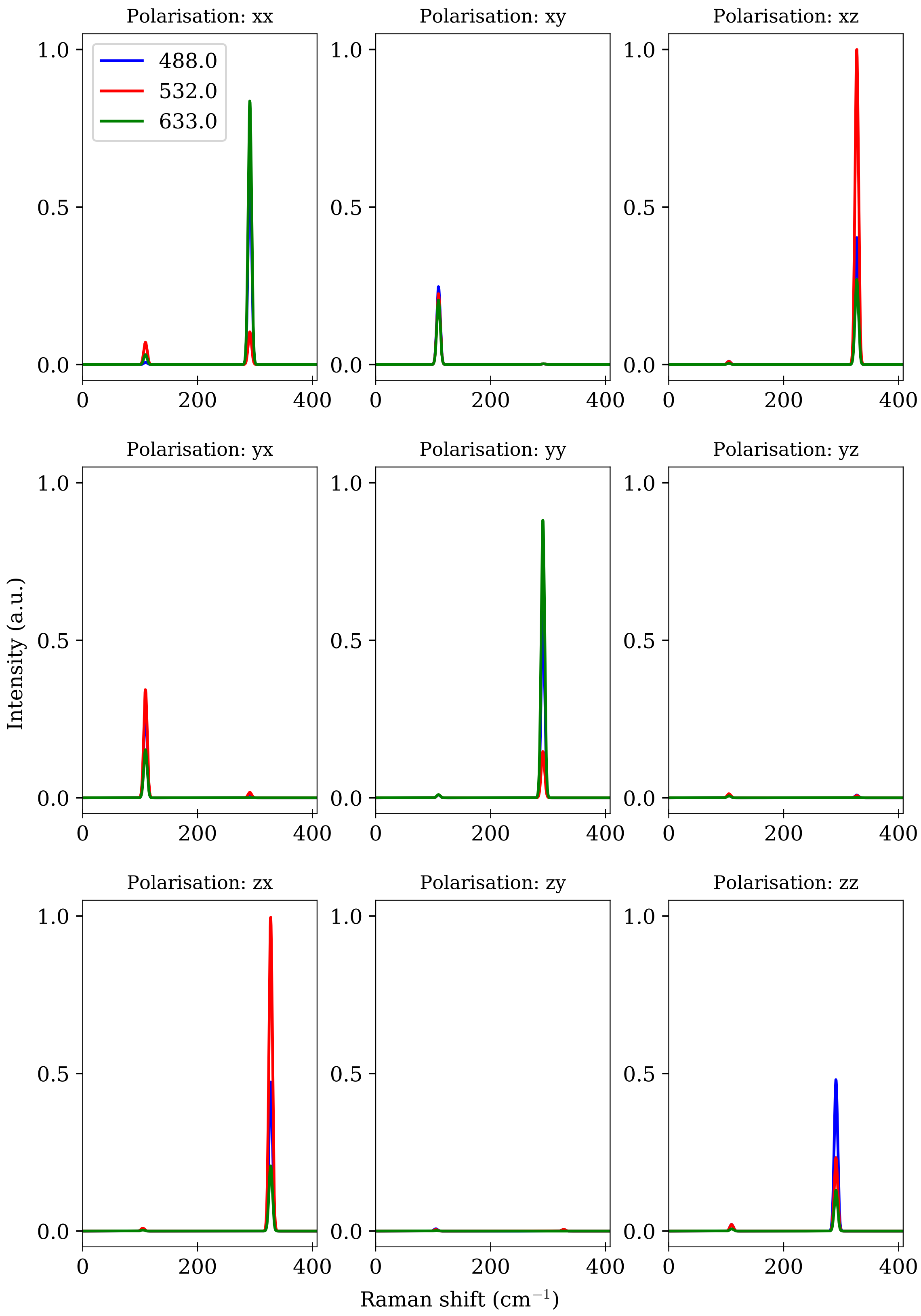

Supplementary Figure 320: Raman spectrum of Li_2V with space group 187 and Wyckoff positions a-i-0.

Li_2Zr, 187, b-i-0

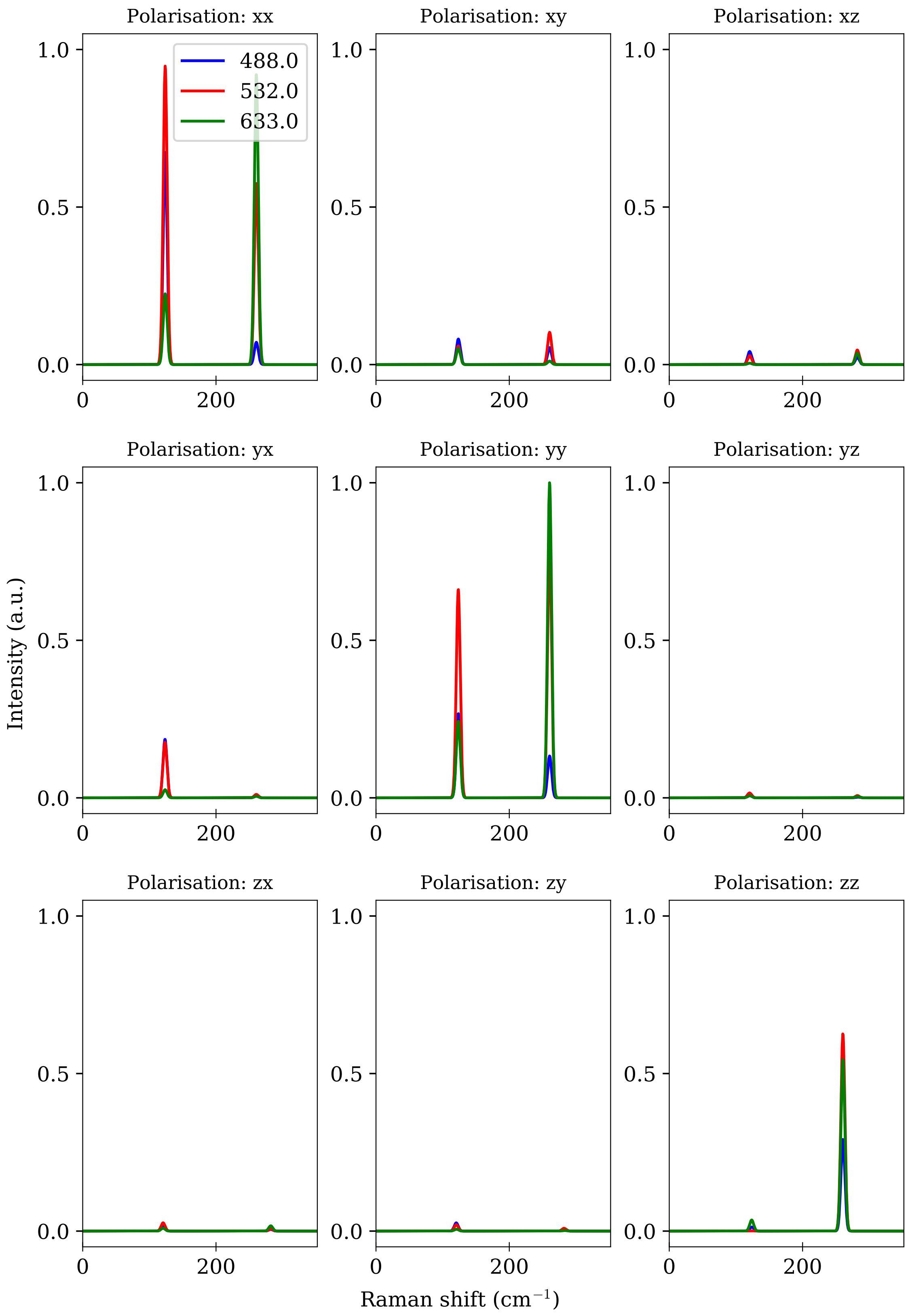

Supplementary Figure 321: Raman spectrum of Li_2Zr with space group 187 and Wyckoff positions b-i-0.

MgBr$_2$, 164, b-d-0

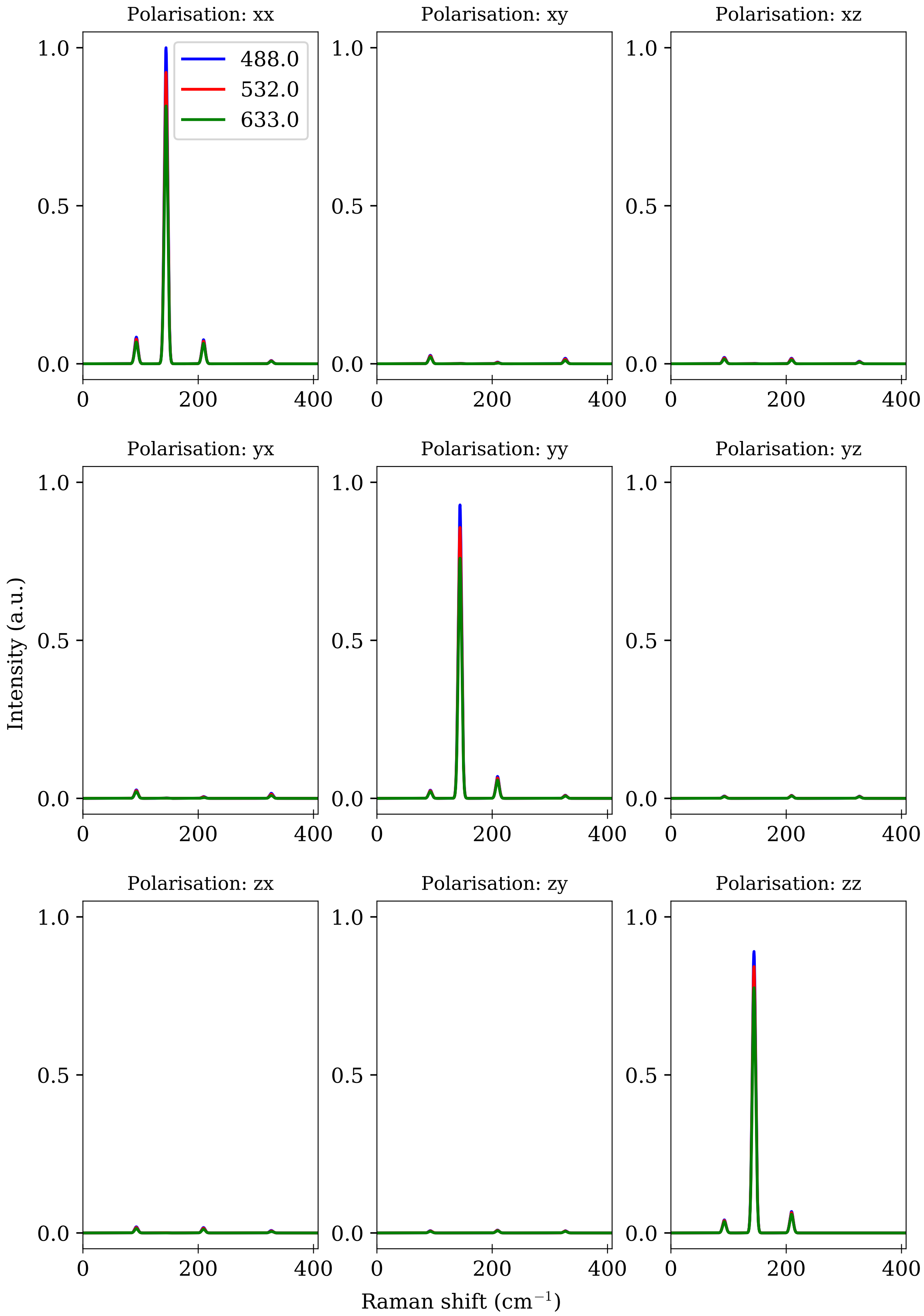

Supplementary Figure 322: Raman spectrum of MgBr$_2$ with space group 164 and Wyckoff positions b-d-0.

$MgCl_2$, 164, b-d-0

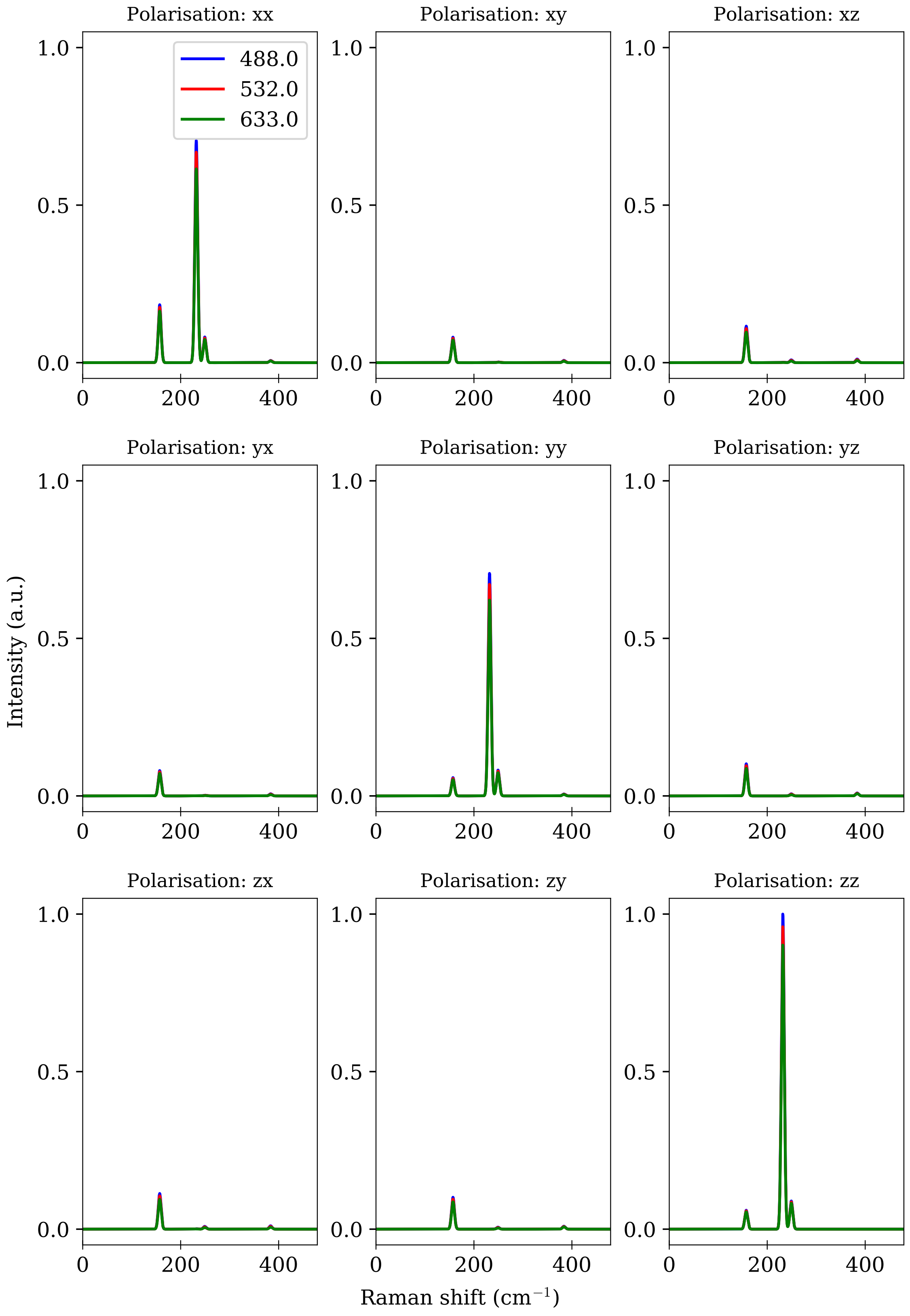

Supplementary Figure 323: Raman spectrum of $MgCl_2$ with space group 164 and Wyckoff positions b-d-0.

$MgCl_2$, 187, b-i-0

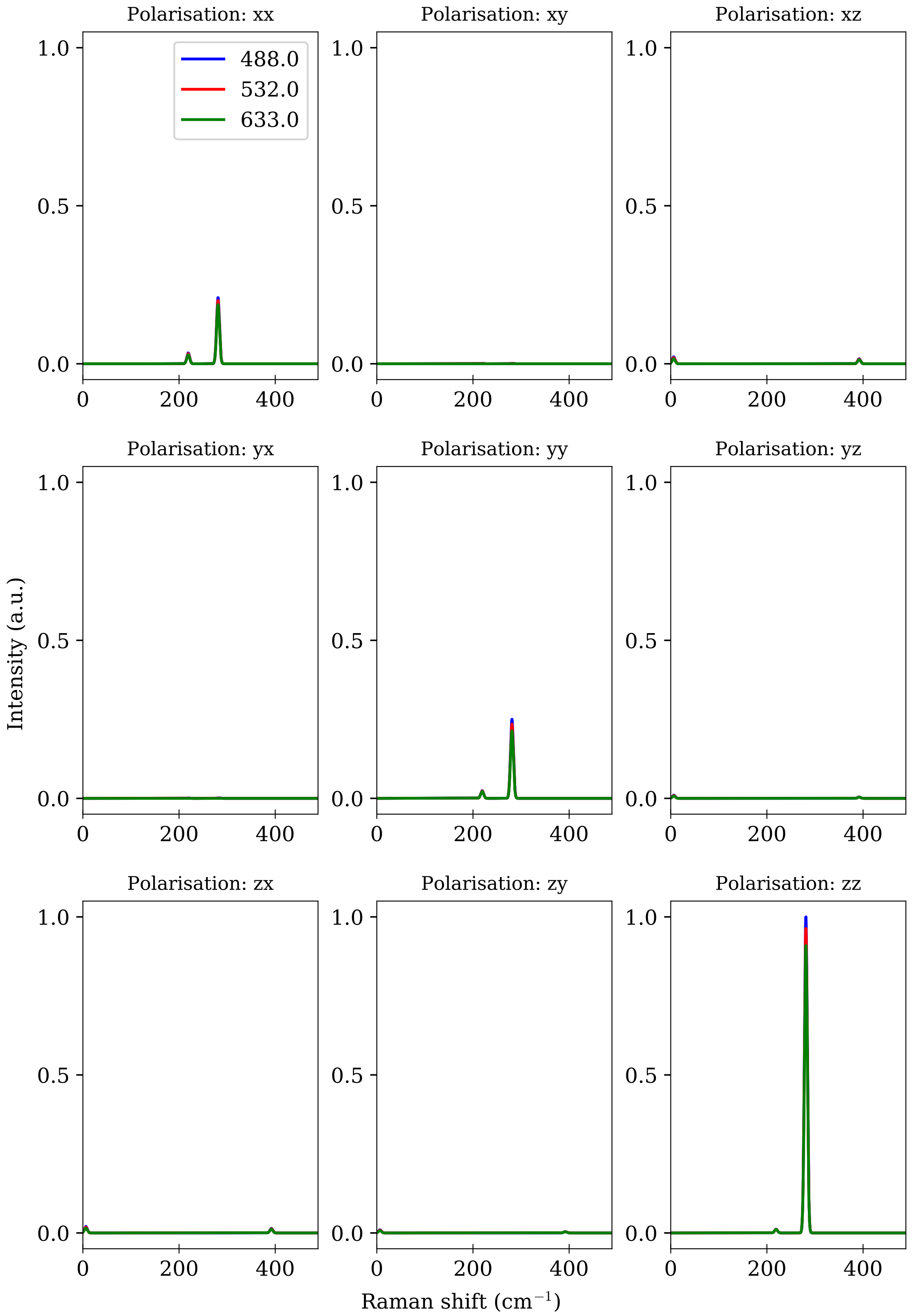

Supplementary Figure 324: Raman spectrum of $MgCl_2$ with space group 187 and Wyckoff positions b-i-0.

MgI_2, 164, b-d-0

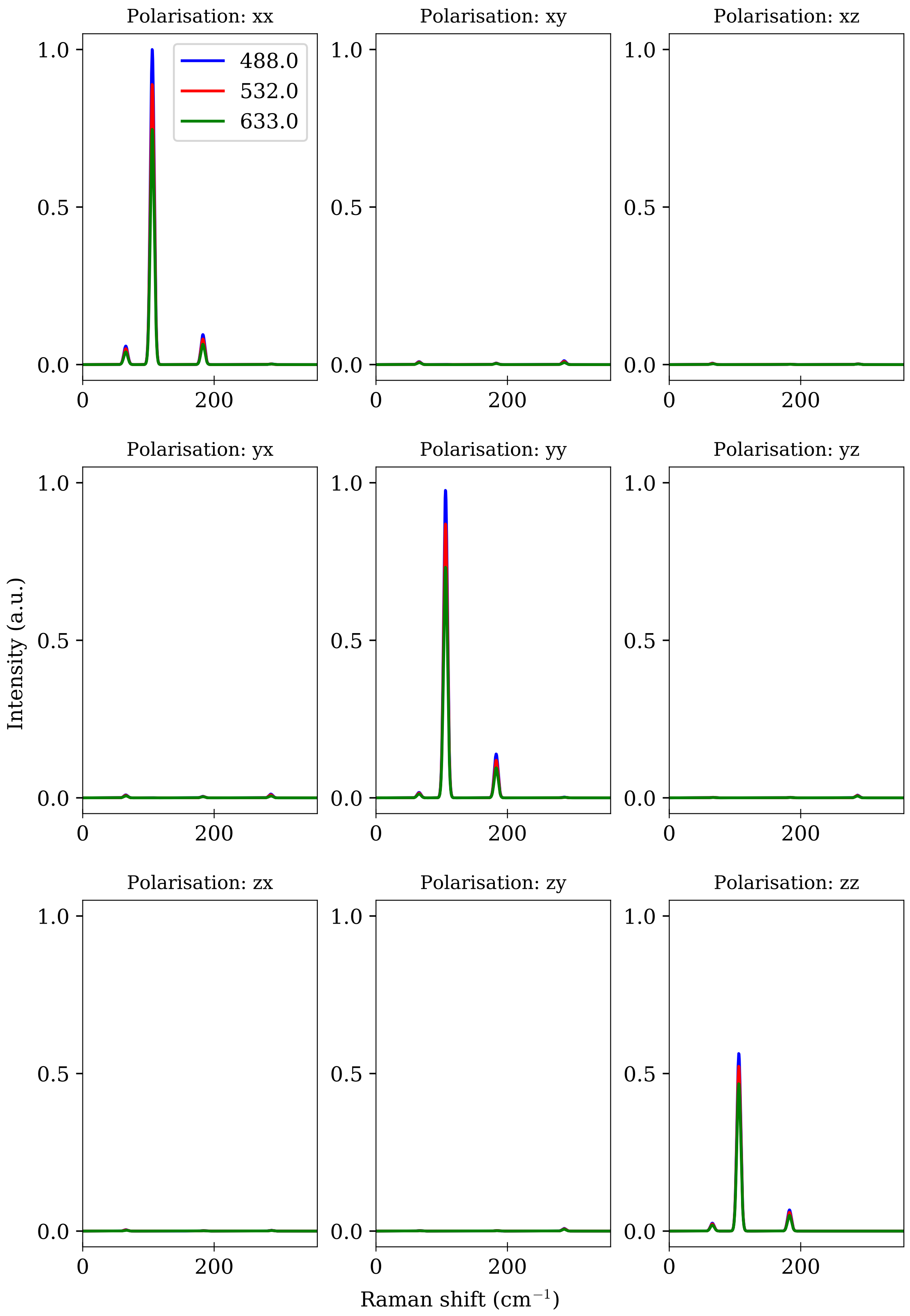

Supplementary Figure 325: Raman spectrum of MgI_2 with space group 164 and Wyckoff positions b-d-0.

MnS_2, 115, d-g-0

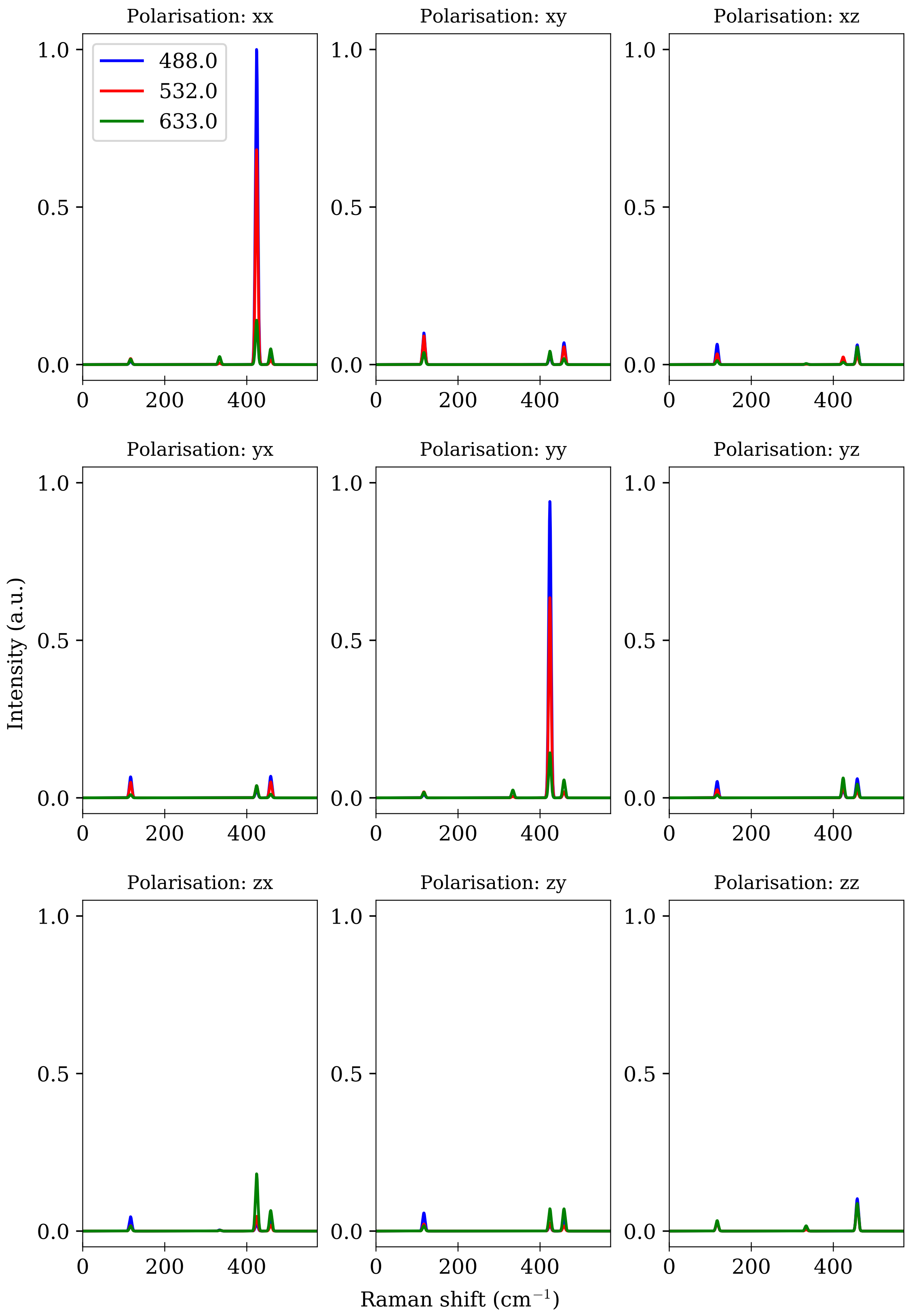

Supplementary Figure 326: Raman spectrum of MnS_2 with space group 115 and Wyckoff positions d-g-0.

Mo_2Cl_2, 12, i-0

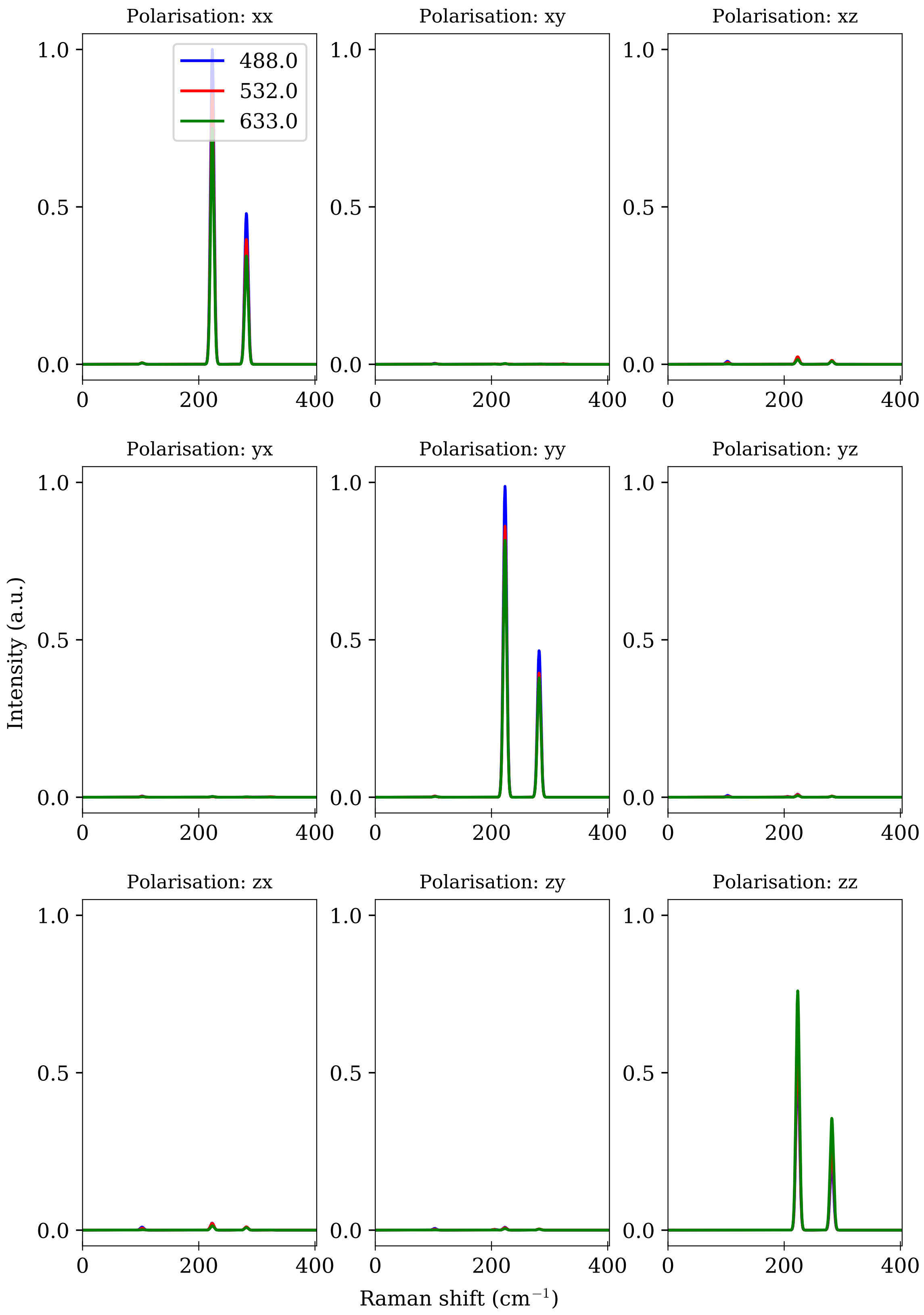

Supplementary Figure 327: Raman spectrum of Mo_2Cl_2 with space group 12 and Wyckoff positions i-0.

Mo_2Cl_6, 189, d-i-0

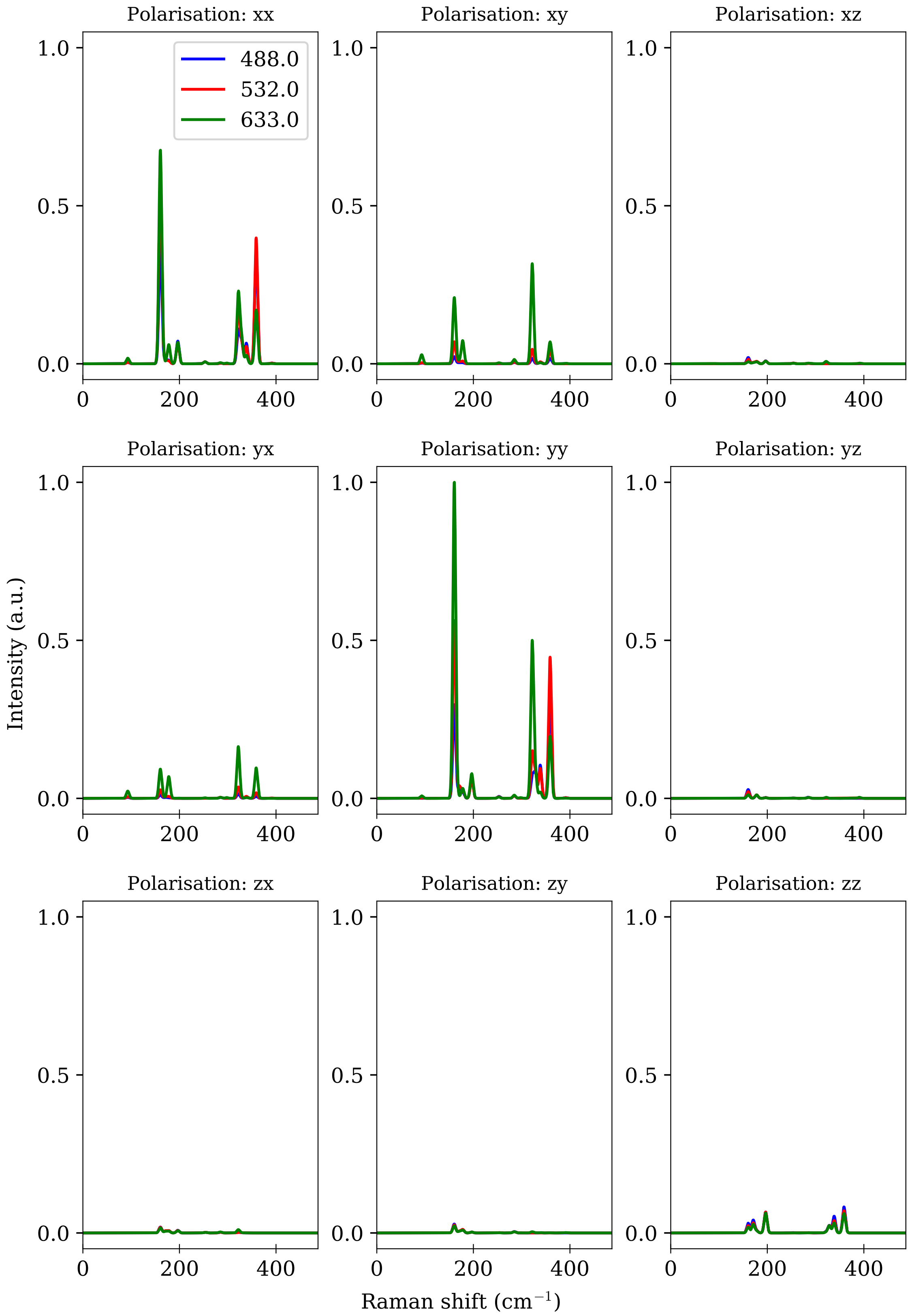

Supplementary Figure 328: Raman spectrum of Mo_2Cl_6 with space group 189 and Wyckoff positions d-i-0.

Mo_2F_2, 12, i-0

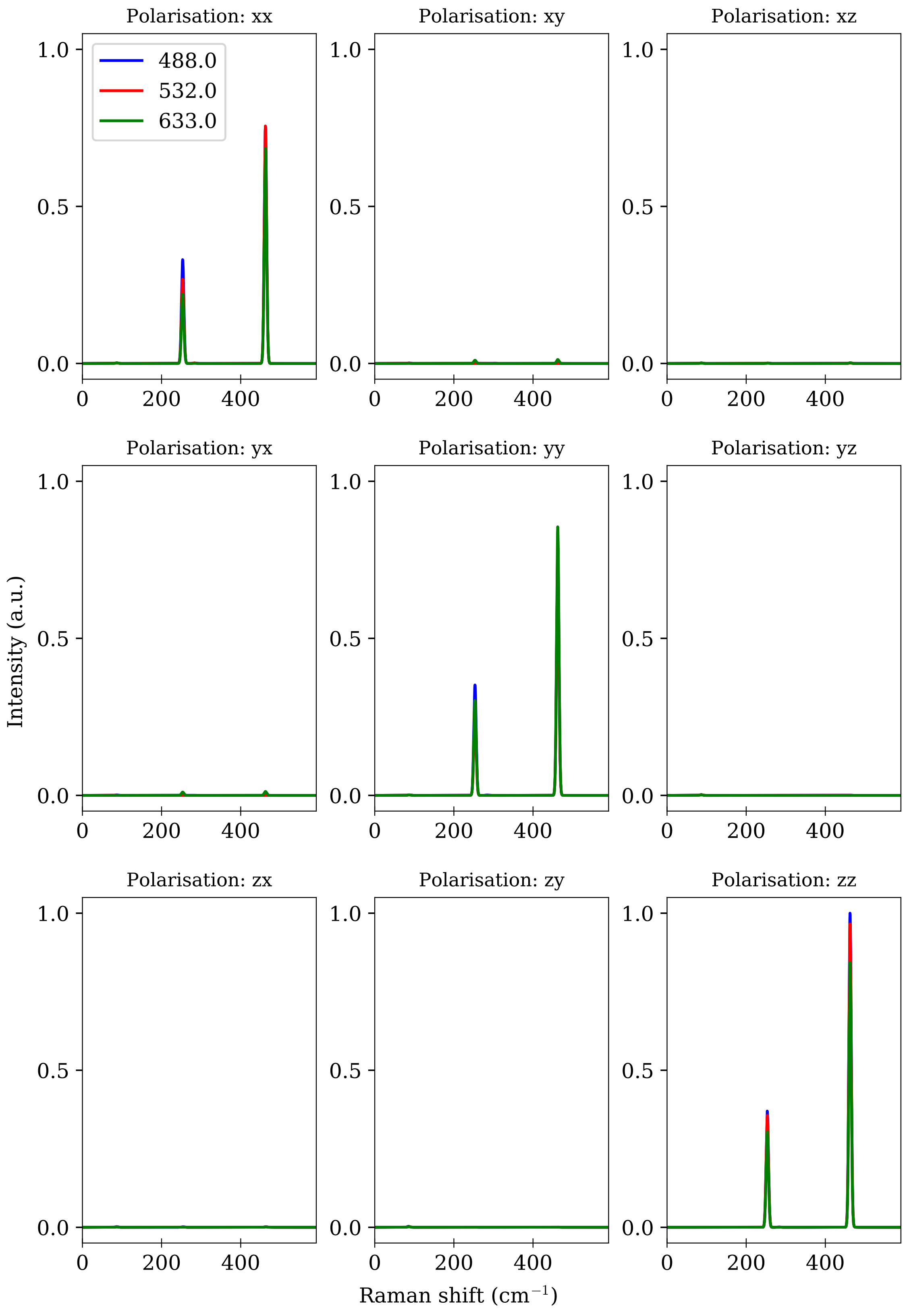

Supplementary Figure 329: Raman spectrum of Mo_2F_2 with space group 12 and Wyckoff positions i-0.

Mo_2I_4, 11, e-0

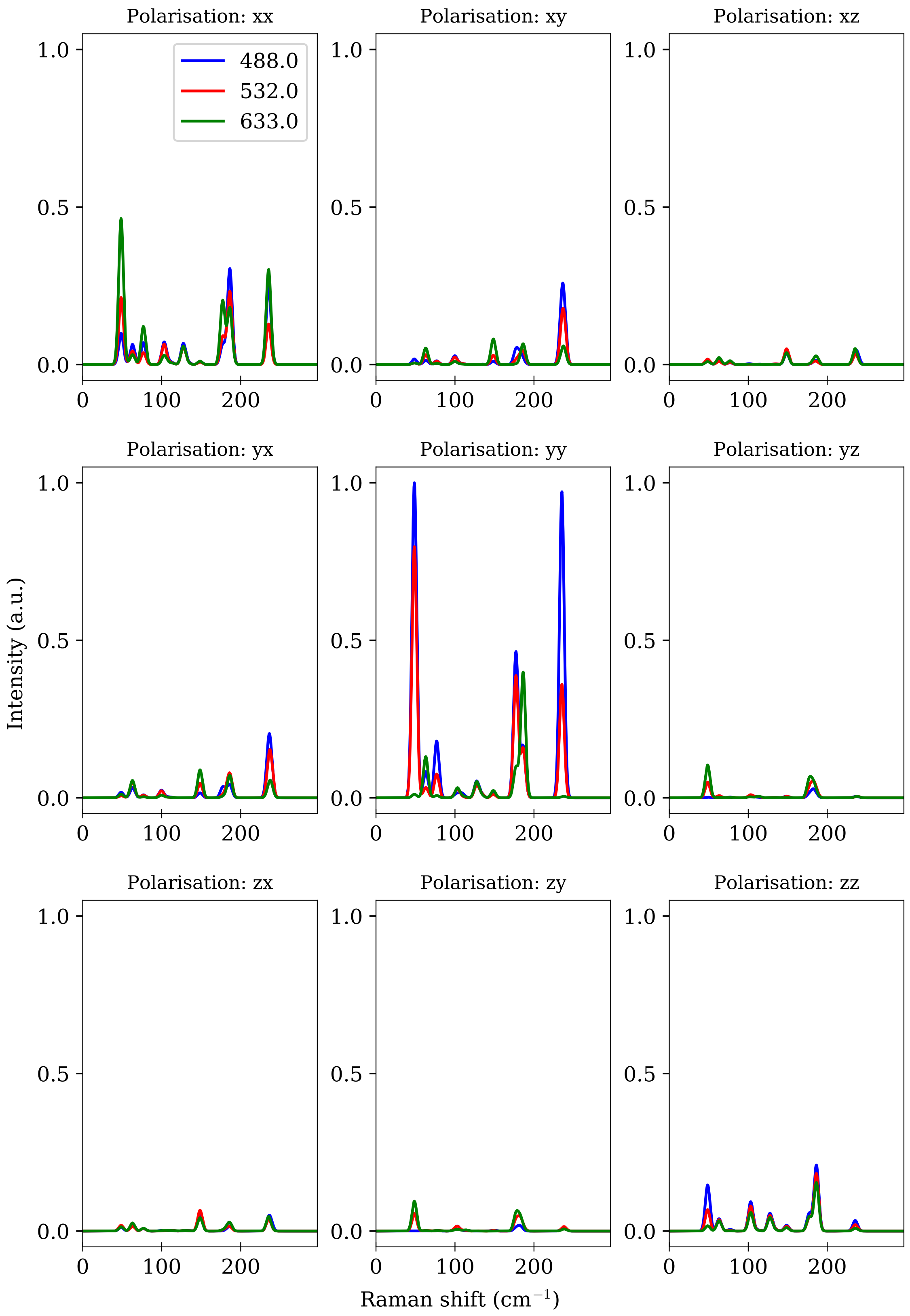

Supplementary Figure 330: Raman spectrum of Mo_2I_4 with space group 11 and Wyckoff positions e-0.

Mo_2N, 164, b-d-0

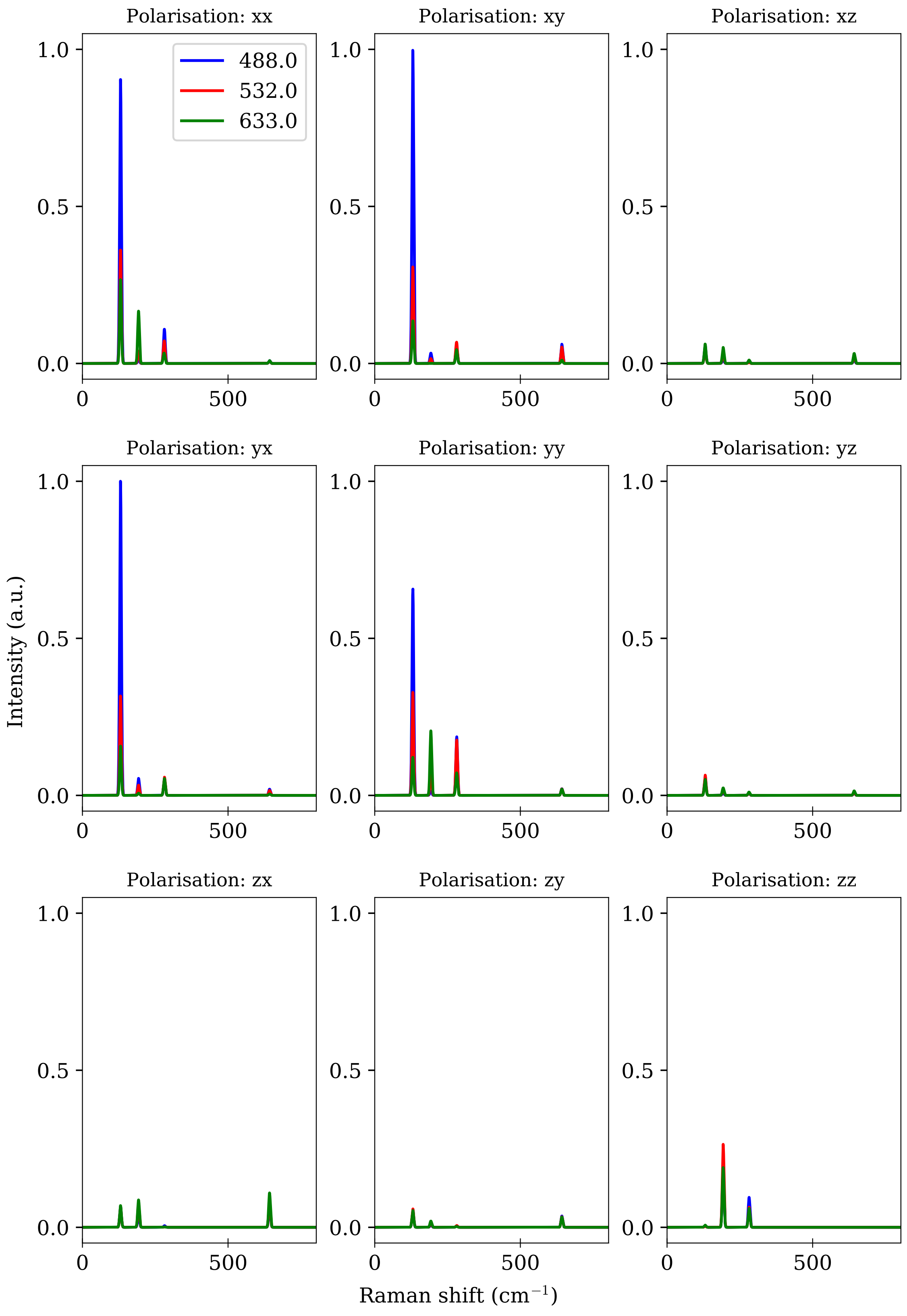

Supplementary Figure 331: Raman spectrum of Mo_2N with space group 164 and Wyckoff positions b-d-0.

Mo_2S_2, 129, b-c-0

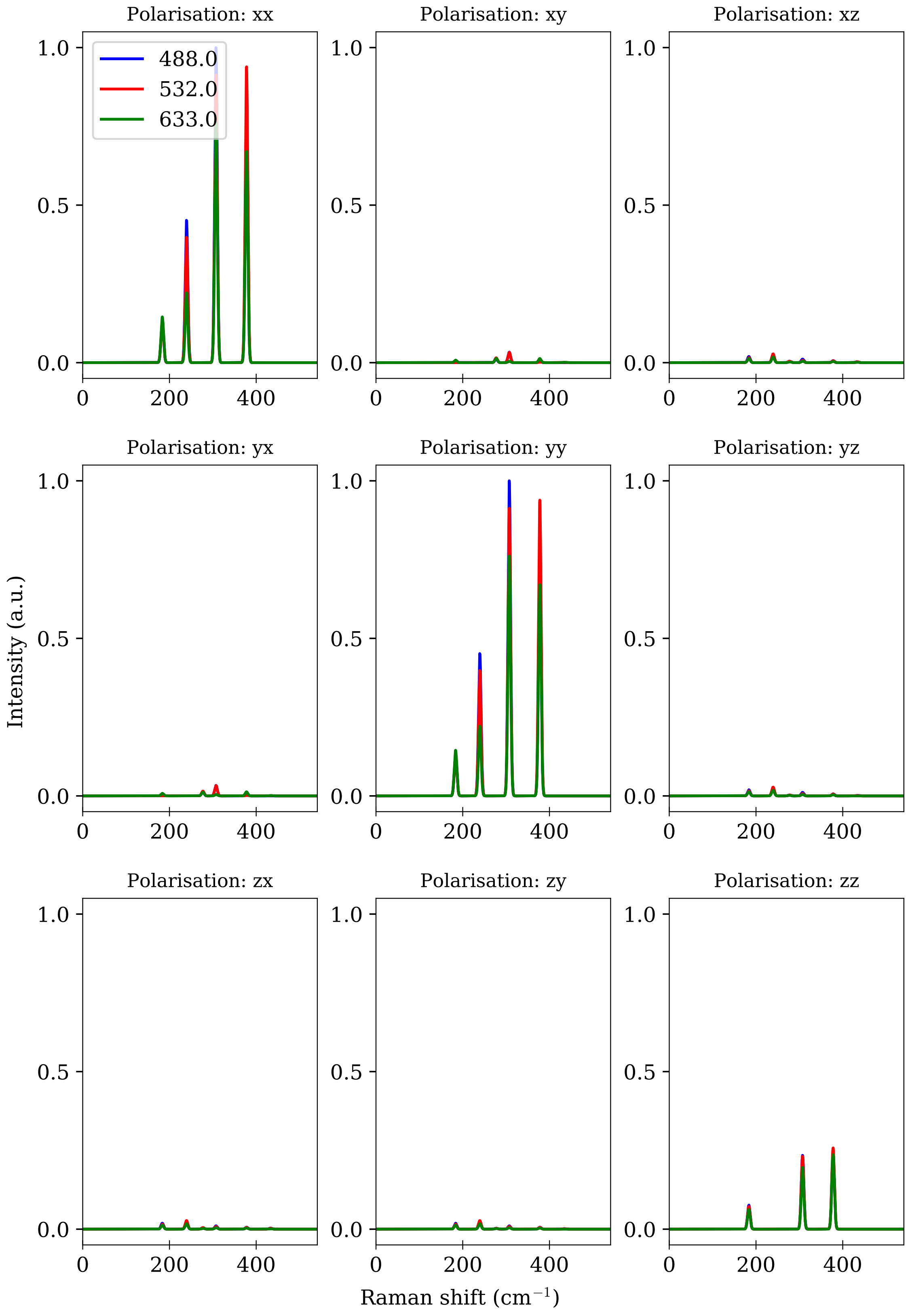

Supplementary Figure 332: Raman spectrum of Mo_2S_2 with space group 129 and Wyckoff positions b-c-0.

Mo_2S_4, 11, e-0

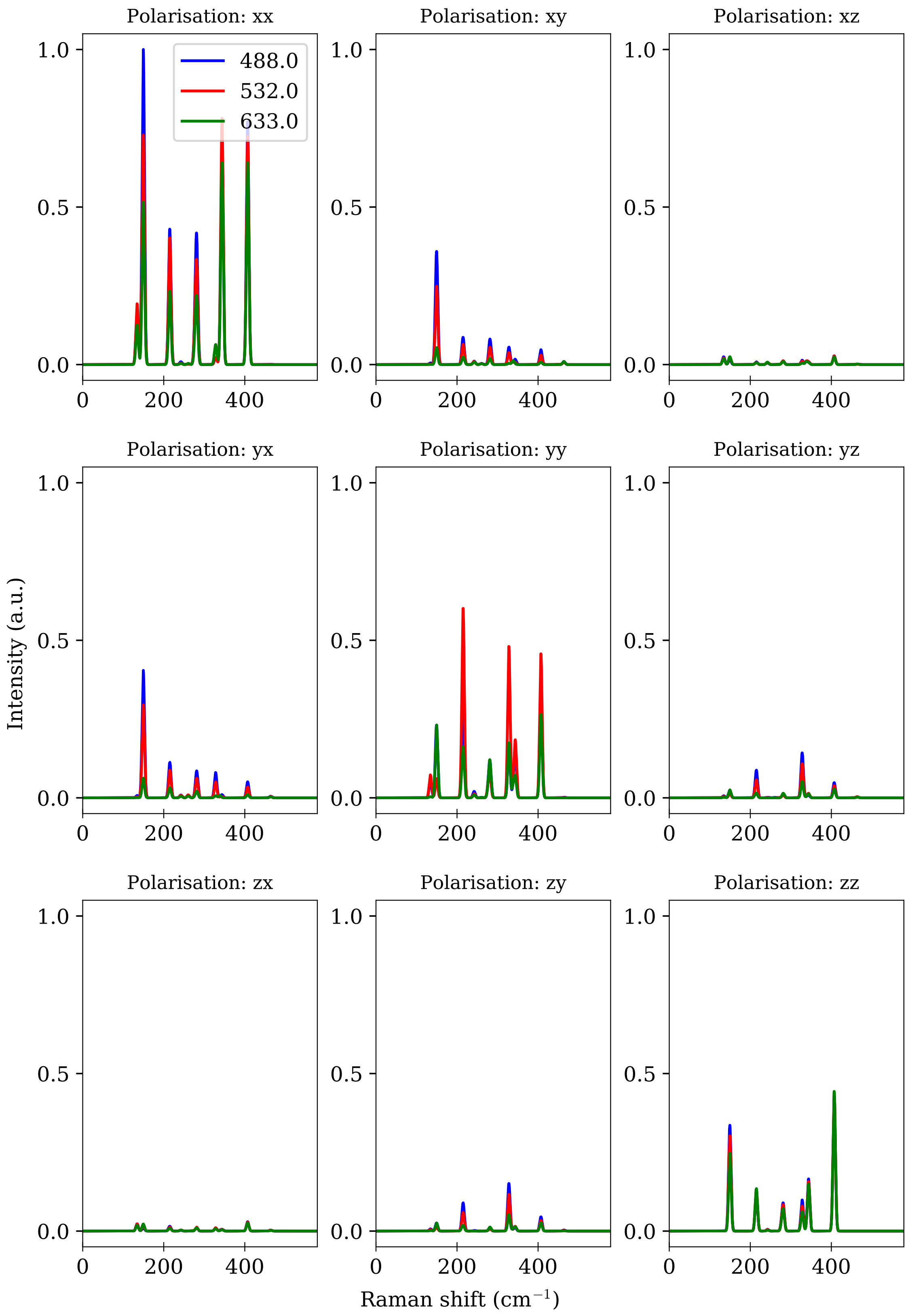

Supplementary Figure 333: Raman spectrum of Mo_2S_4 with space group 11 and Wyckoff positions e-0.

Mo_2Se_2, 129, b-c-0

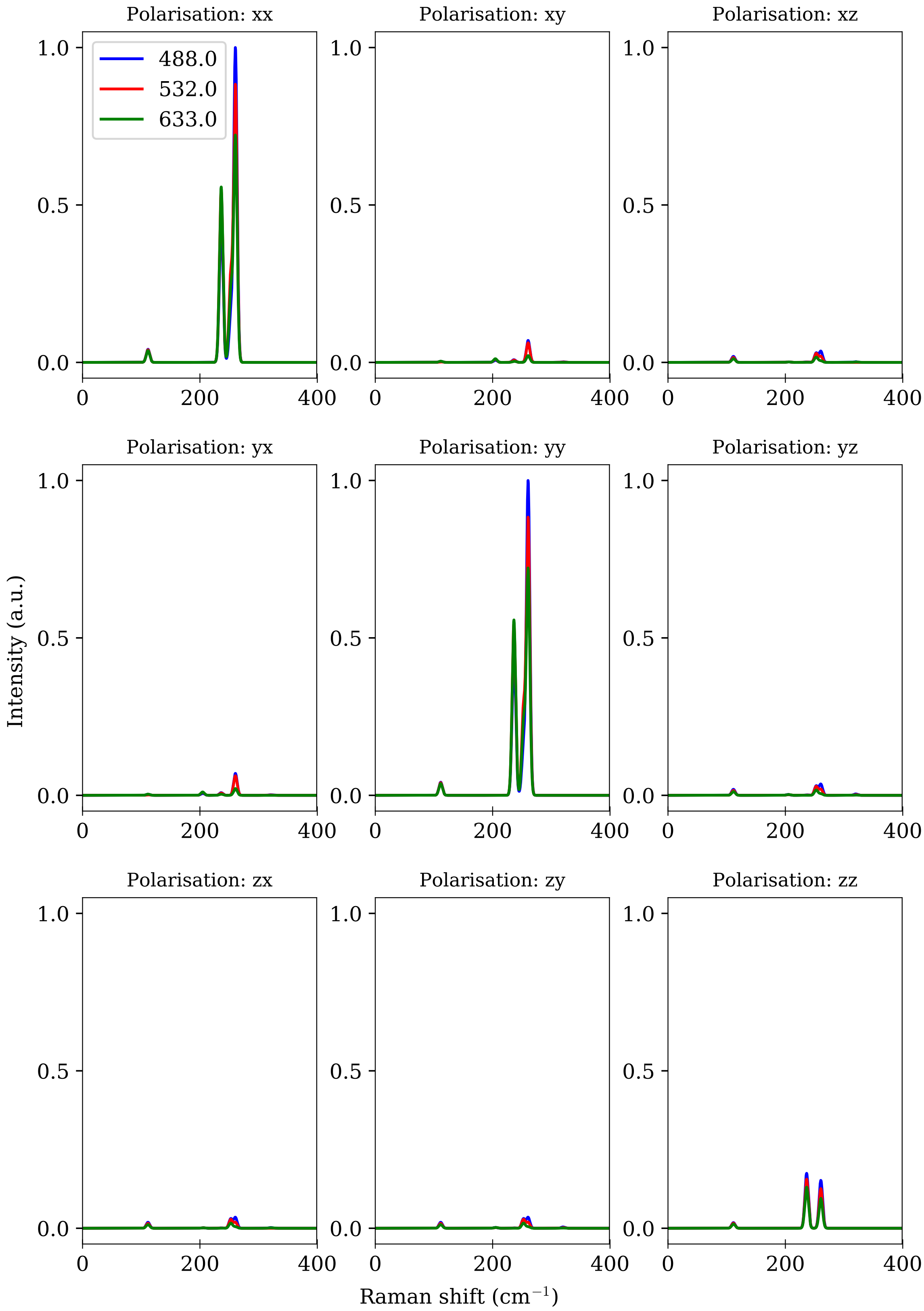

Supplementary Figure 334: Raman spectrum of Mo_2Se_2 with space group 129 and Wyckoff positions b-c-0.

Mo_2Se_2, 2, i-0

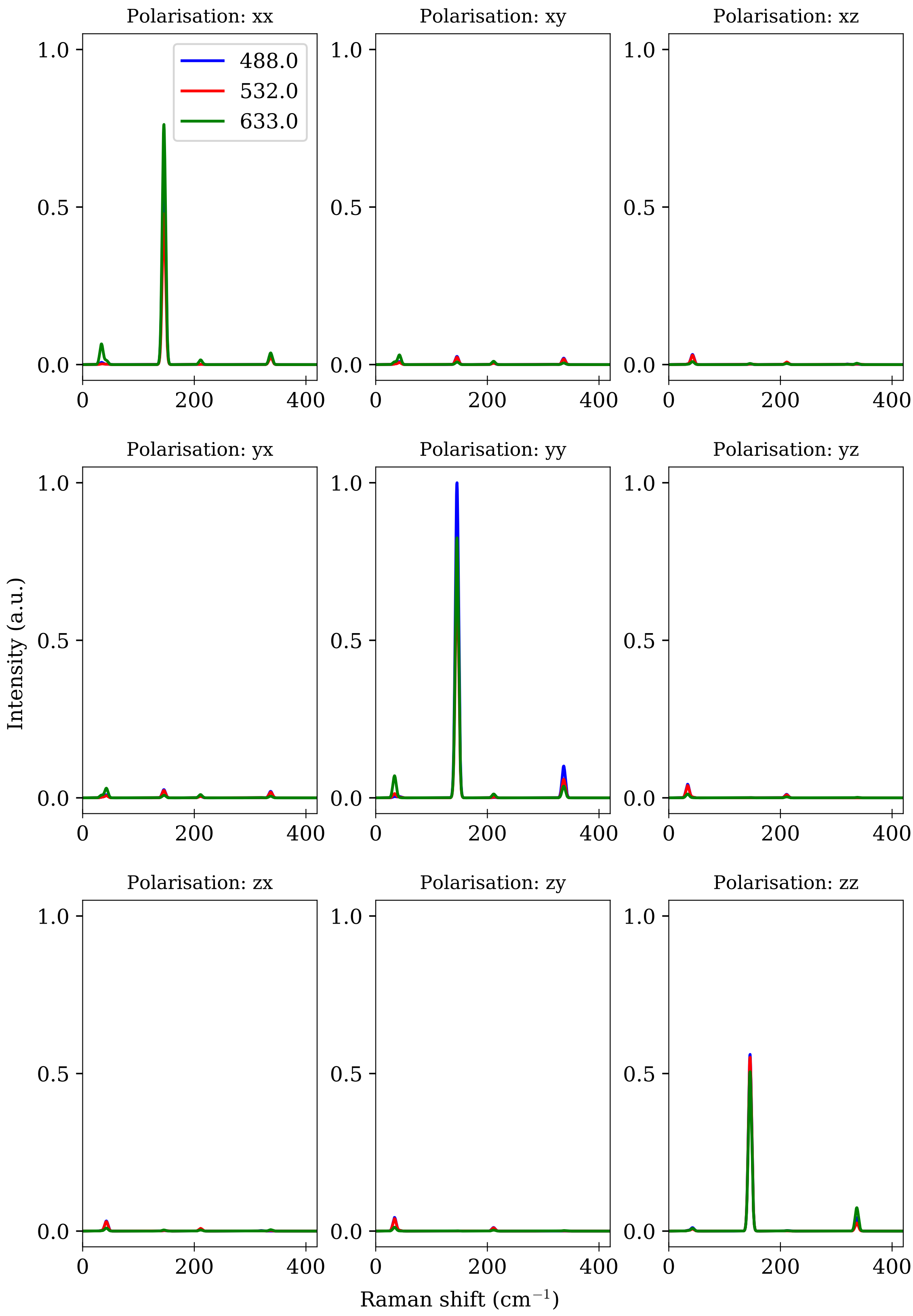

Supplementary Figure 335: Raman spectrum of Mo_2Se_2 with space group 2 and Wyckoff positions i-0.

Mo_2Se_4, 11, e-0

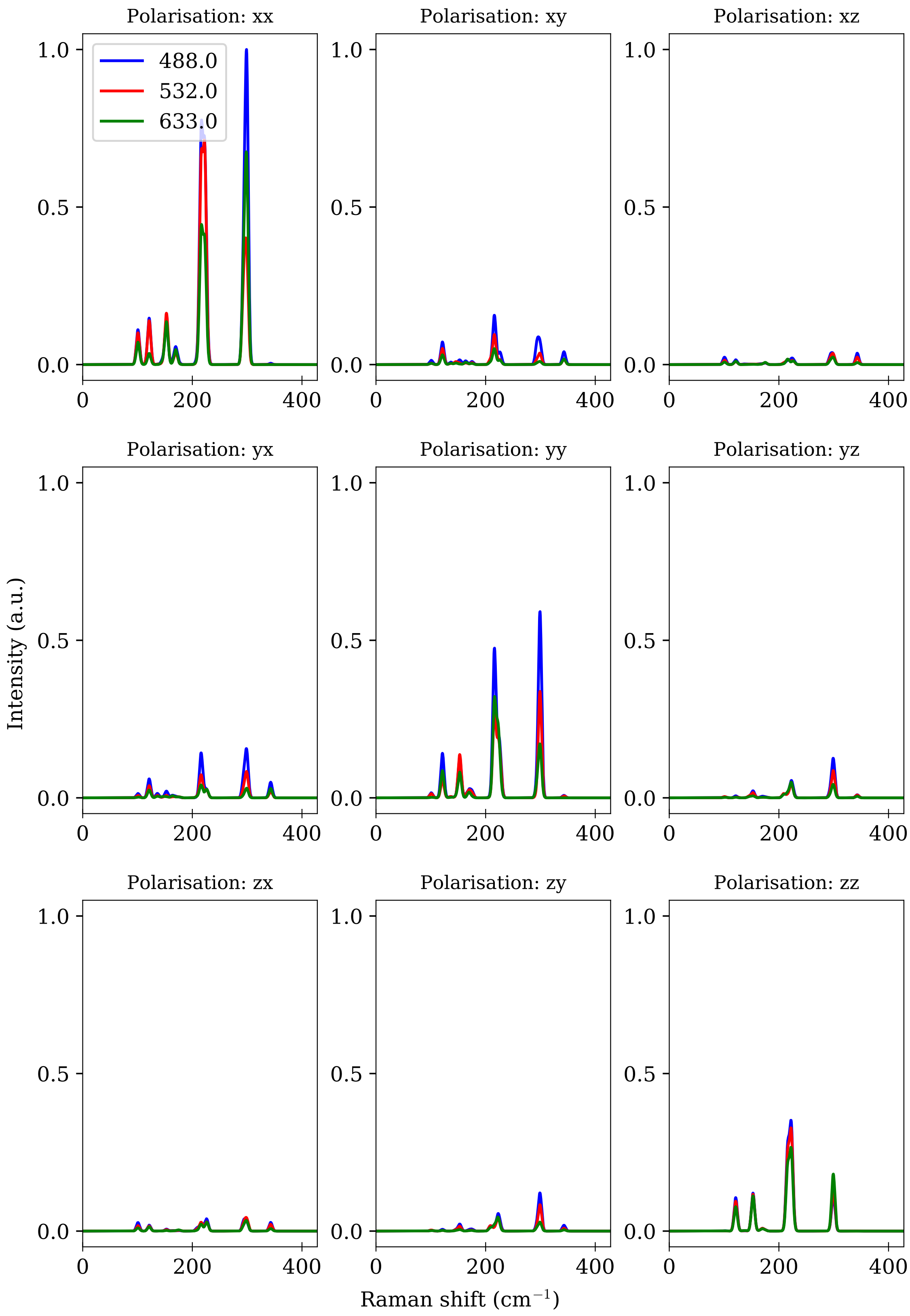

Supplementary Figure 336: Raman spectrum of Mo_2Se_4 with space group 11 and Wyckoff positions e-0.

Mo_2Te_2, 129, b-c-0

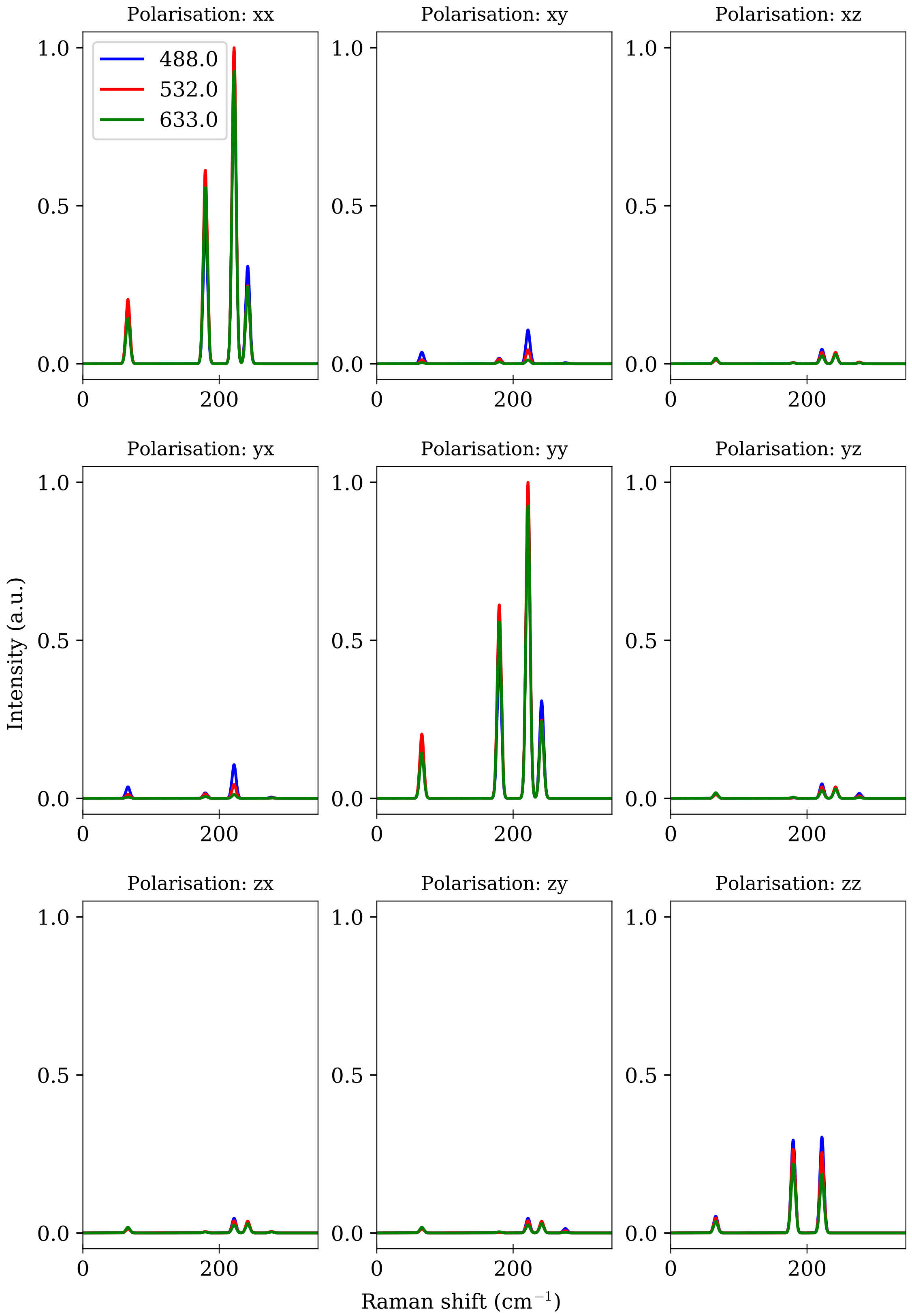

Supplementary Figure 337: Raman spectrum of Mo_2Te_2 with space group 129 and Wyckoff positions b-c-0.

Mo_2Te_4, 11, e-0

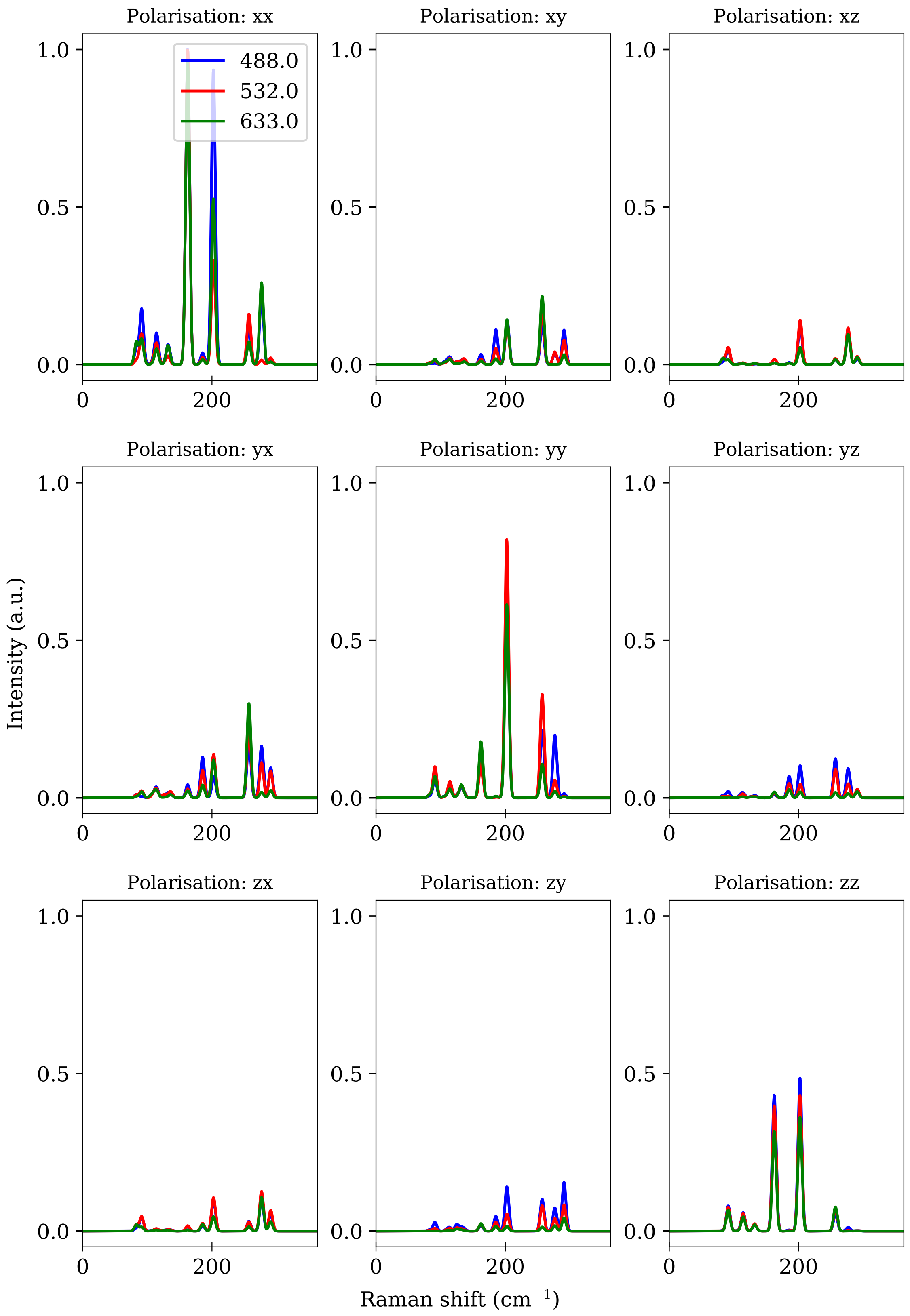

Supplementary Figure 338: Raman spectrum of Mo_2Te_4 with space group 11 and Wyckoff positions e-0.

Mo_3C_2, 187, b-h-i-0

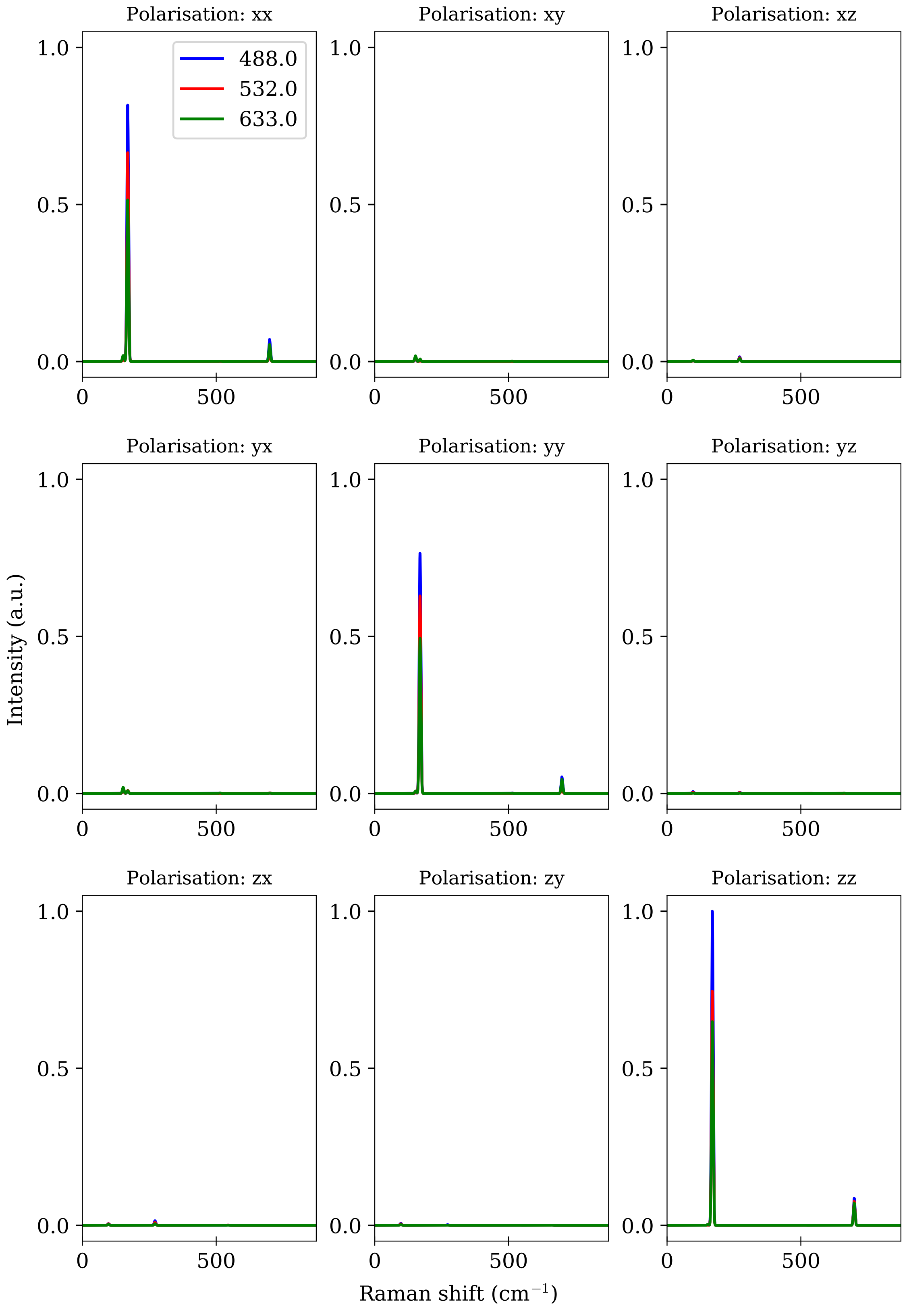

Supplementary Figure 339: Raman spectrum of Mo_3C_2 with space group 187 and Wyckoff positions b-h-i-0.

MoAs$_2$, 164, b-d-0

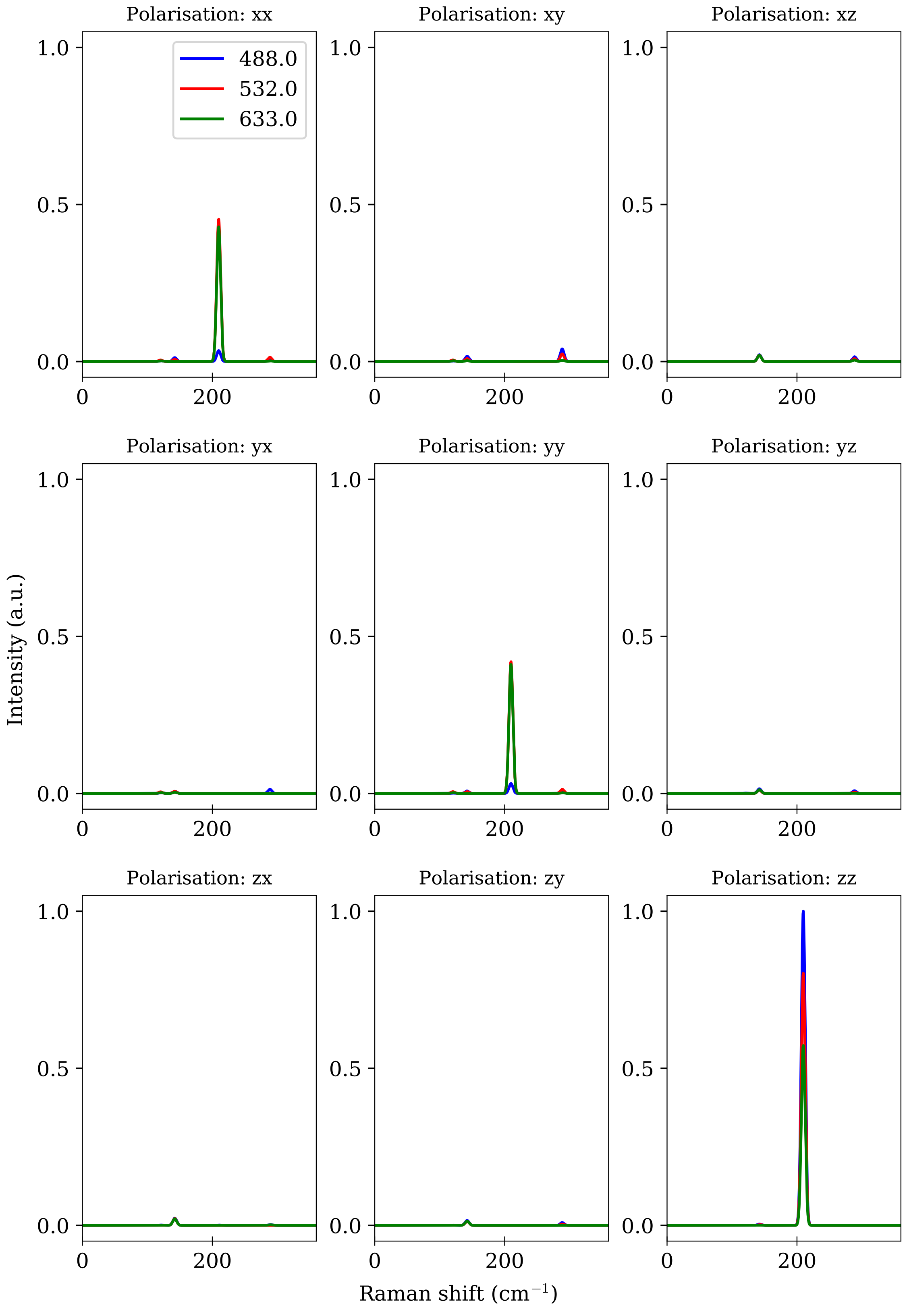

Supplementary Figure 340: Raman spectrum of MoAs$_2$ with space group 164 and Wyckoff positions b-d-0.

MoAsSb, 156, a-b-c-0

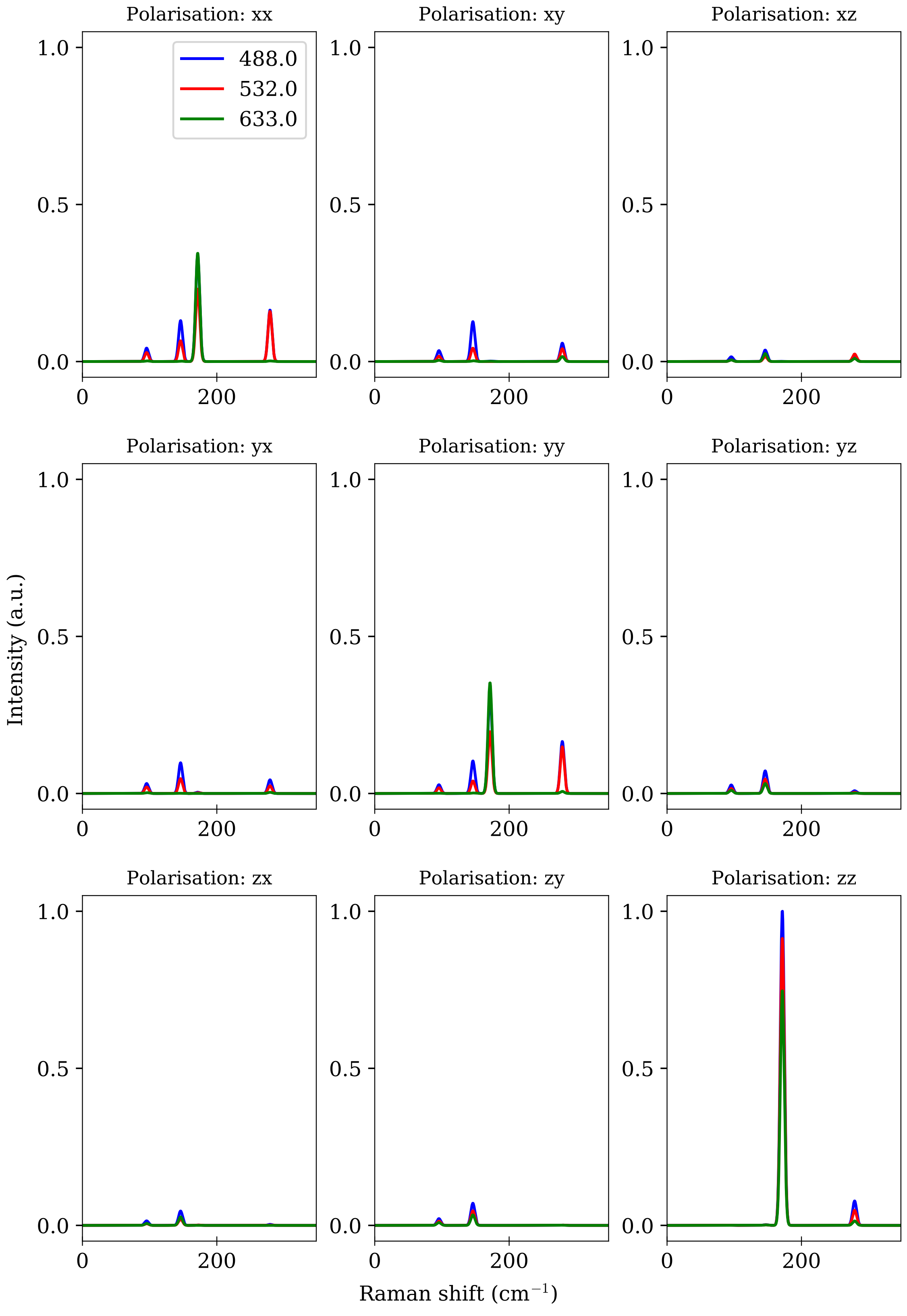

Supplementary Figure 341: Raman spectrum of MoAsSb with space group 156 and Wyckoff positions a-b-c-0.

MoAsSb, 156, a-c-0

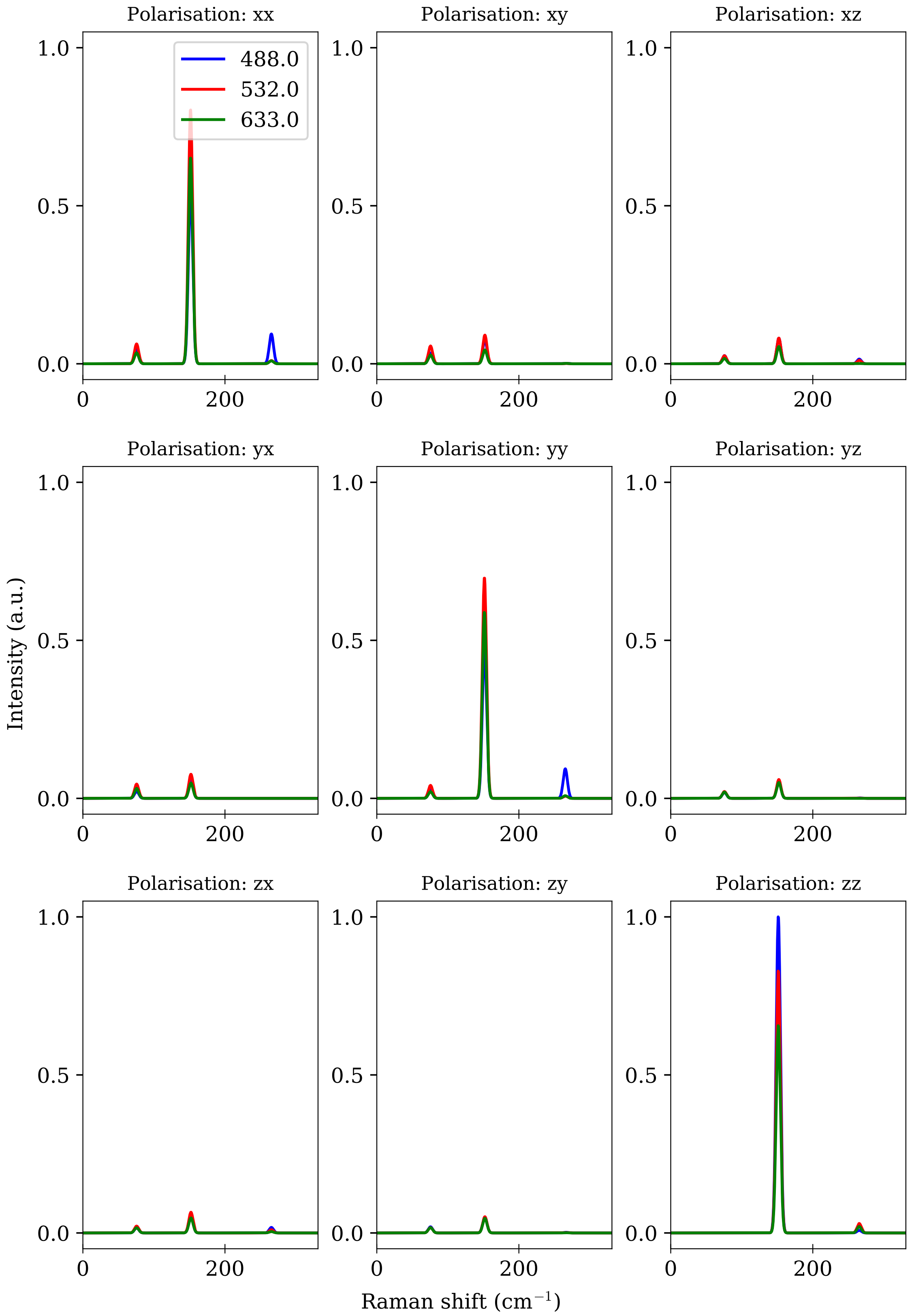

Supplementary Figure 342: Raman spectrum of MoAsSb with space group 156 and Wyckoff positions a-c-0.

MoH_2, 187, a-i-0

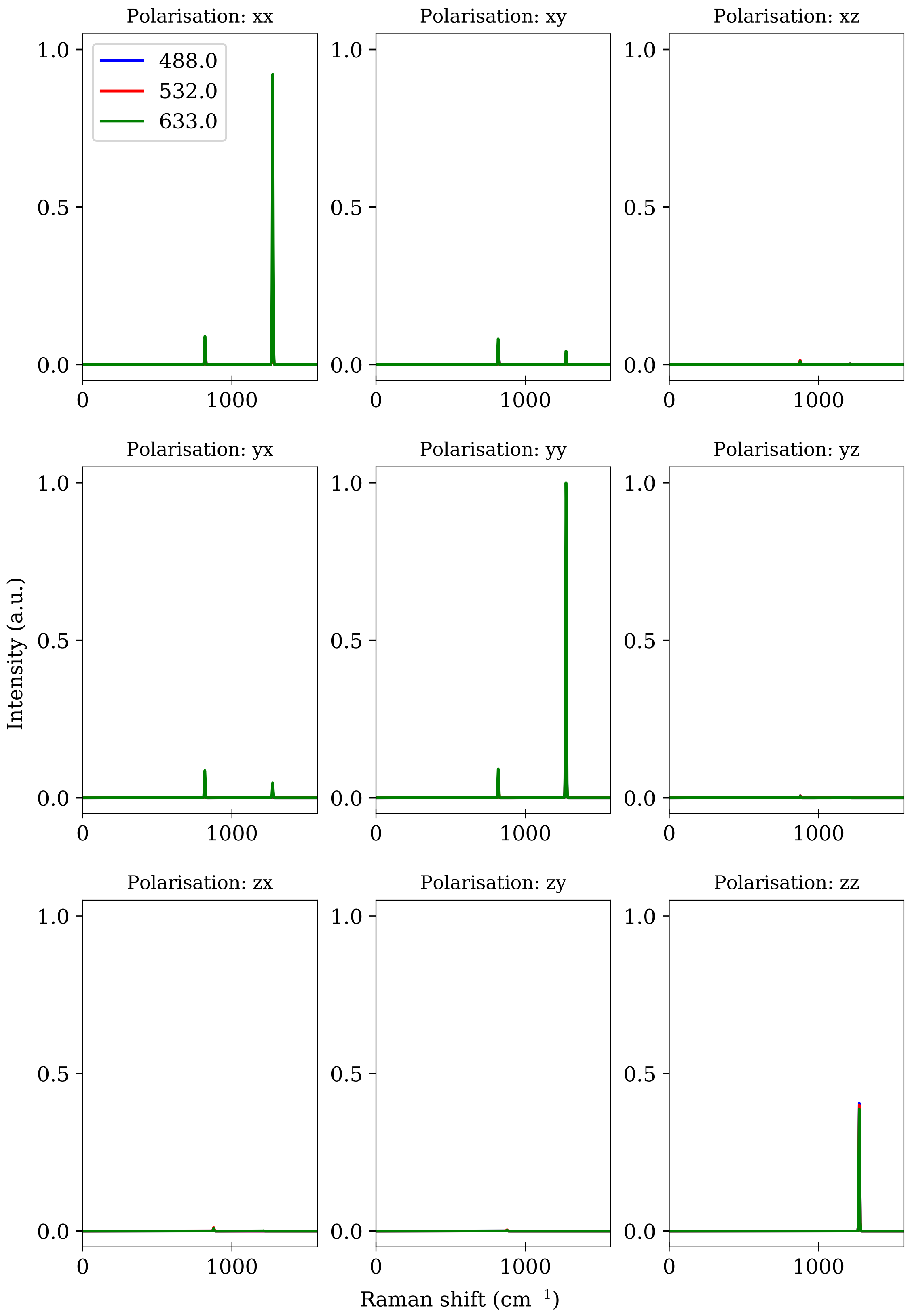

Supplementary Figure 343: Raman spectrum of MoH_2 with space group 187 and Wyckoff positions a-i-0.

MoO_2, 187, b-i-0

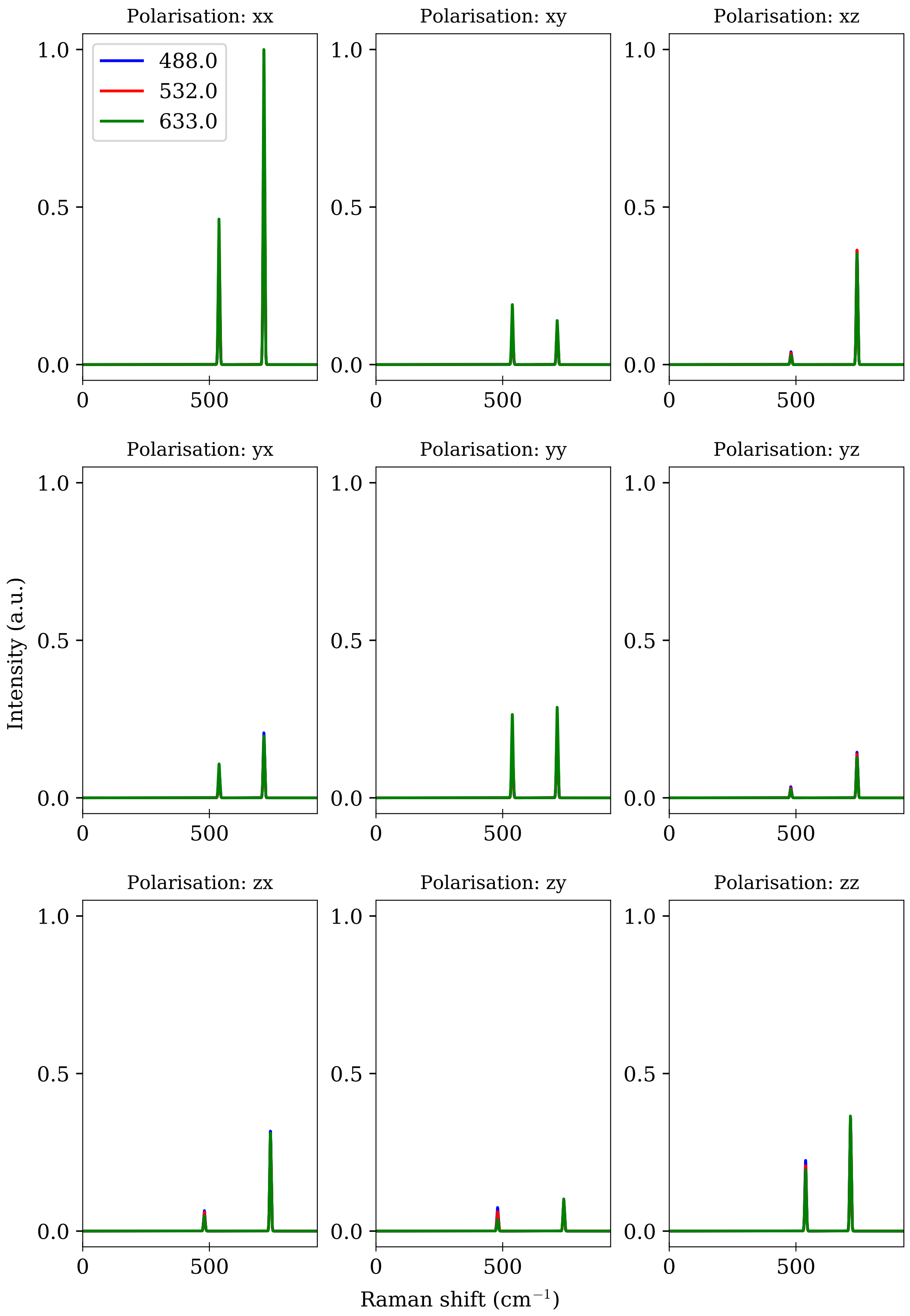

Supplementary Figure 344: Raman spectrum of MoO_2 with space group 187 and Wyckoff positions b-i-0.

MoS_2, 115, d-g-0

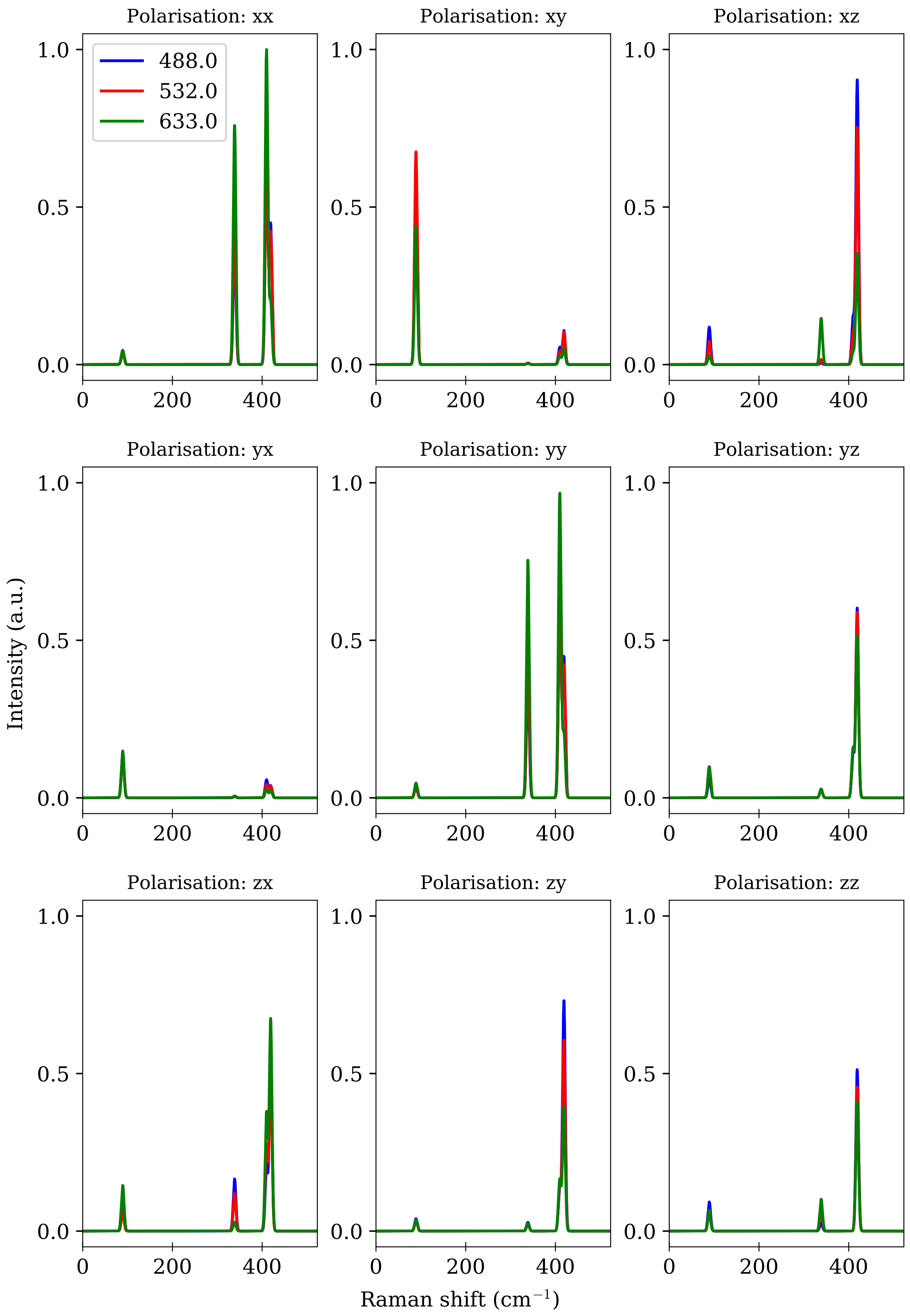

Supplementary Figure 345: Raman spectrum of MoS_2 with space group 115 and Wyckoff positions d-g-0.

MoS_2, 187, b-i-0

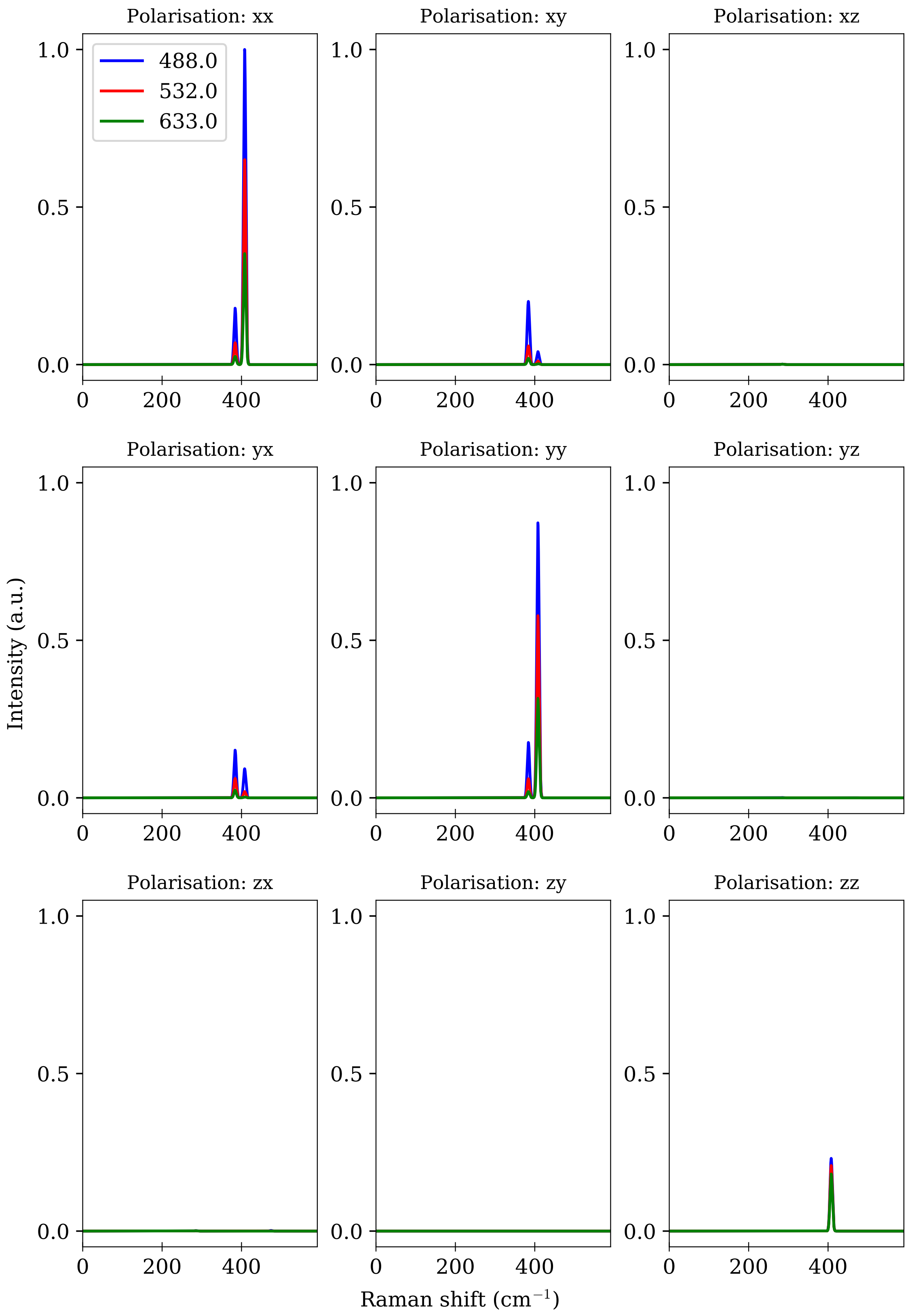

Supplementary Figure 346: Raman spectrum of MoS_2 with space group 187 and Wyckoff positions b-i-0.

$MoSb_2$, 164, b-d-0

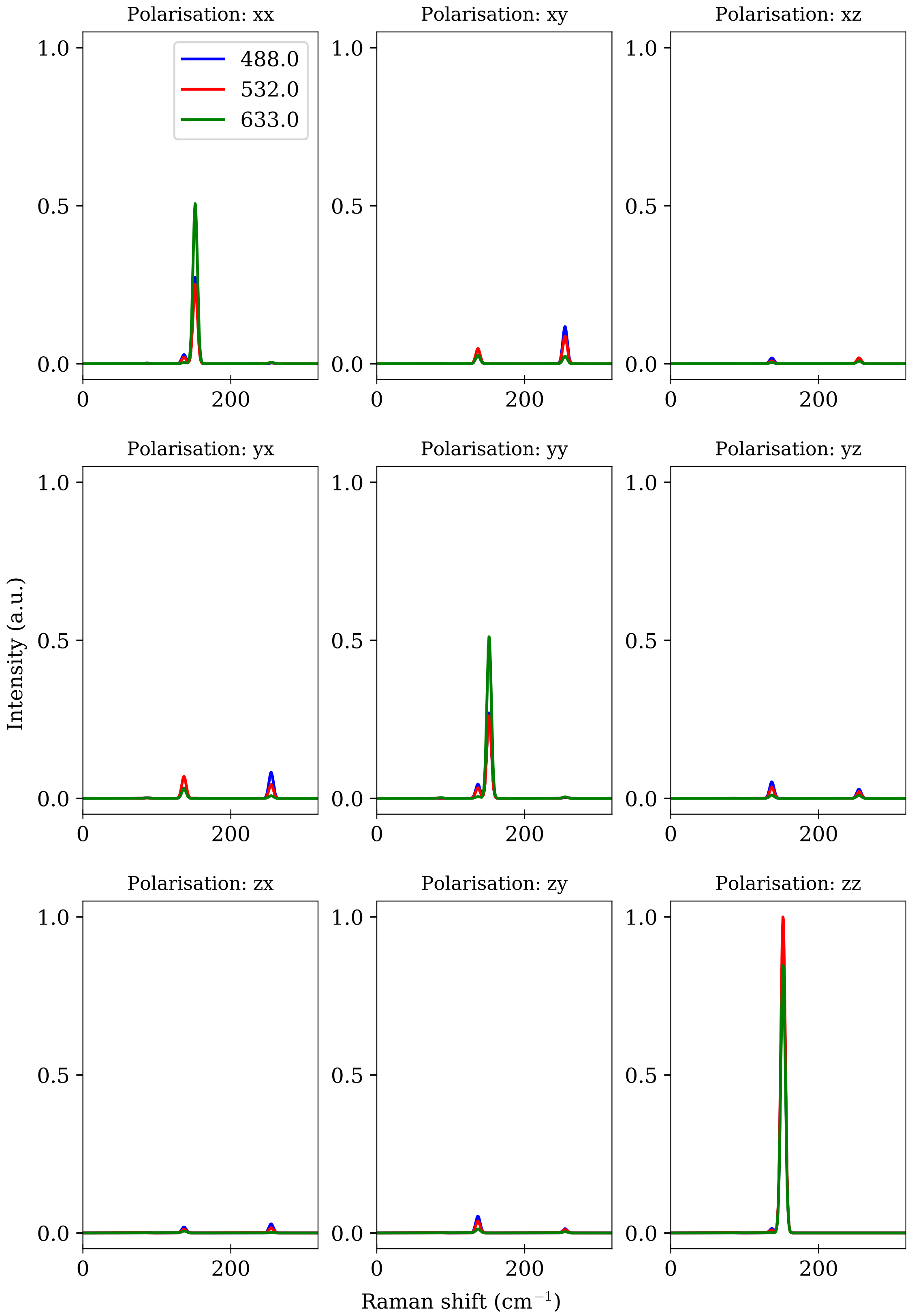

Supplementary Figure 347: Raman spectrum of $MoSb_2$ with space group 164 and Wyckoff positions b-d-0.

$MoSb_2$, 187, b-i-0

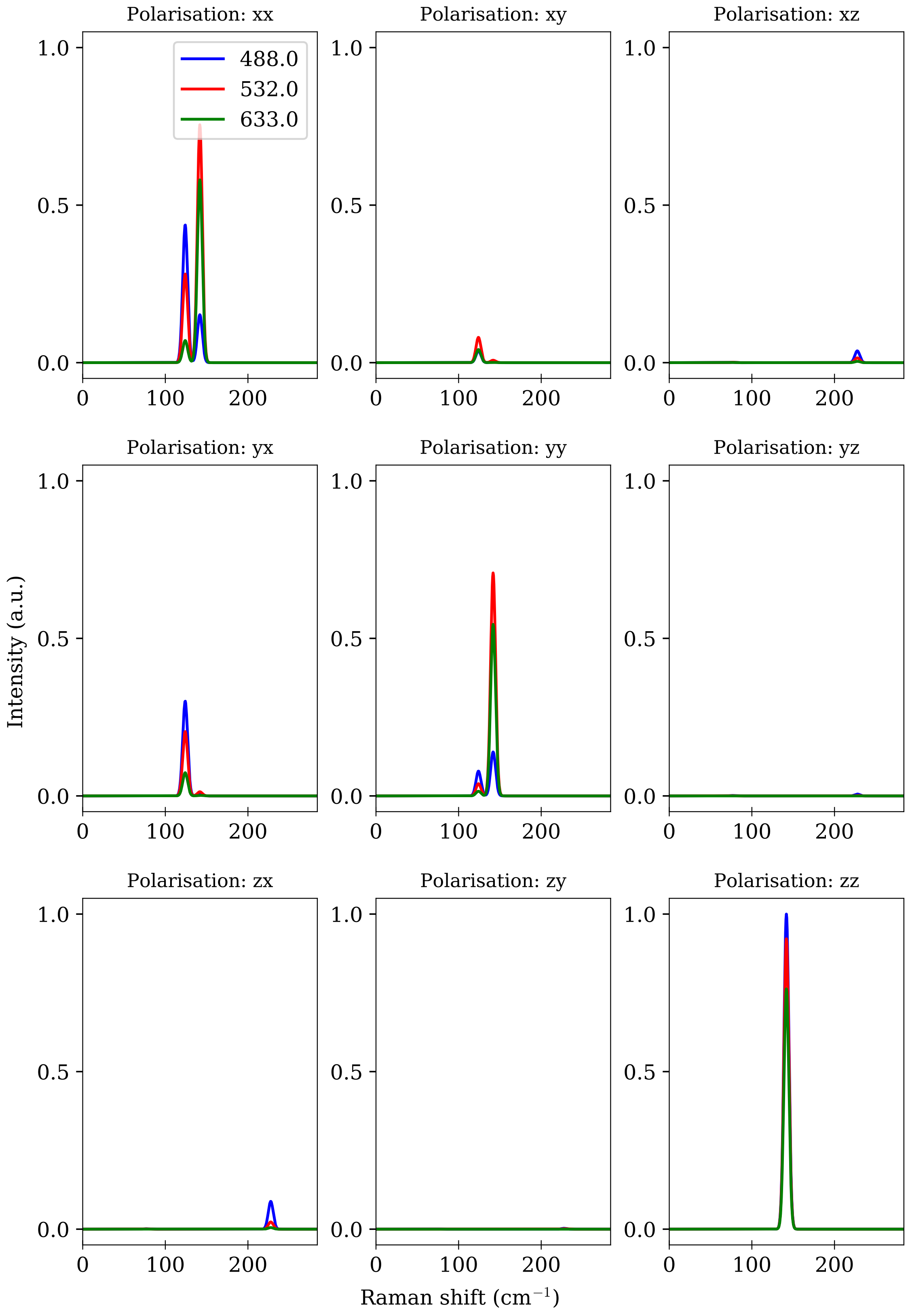

Supplementary Figure 348: Raman spectrum of $MoSb_2$ with space group 187 and Wyckoff positions b-i-0.

$MoSe_2$, 115, d-g-0

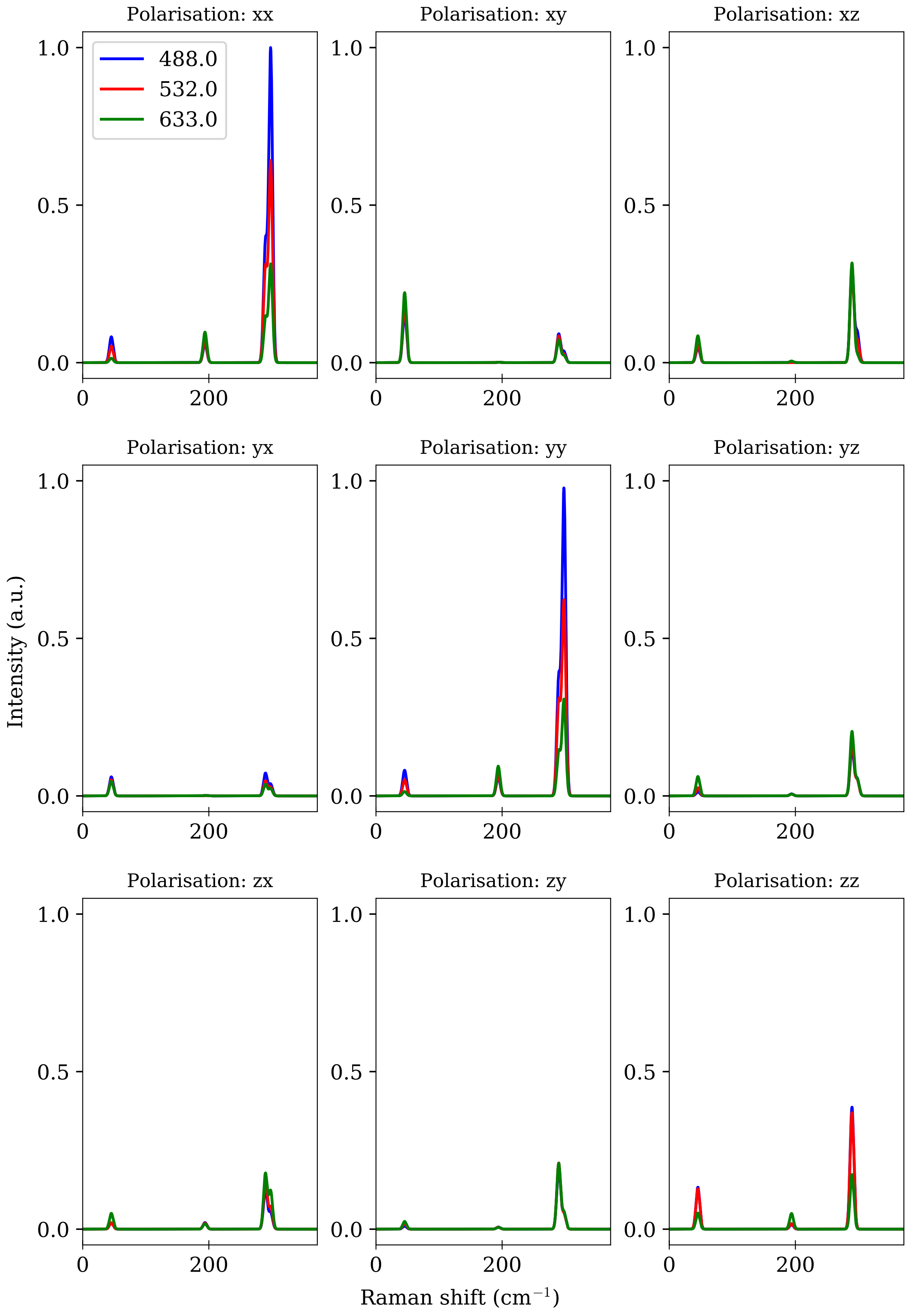

Supplementary Figure 349: Raman spectrum of $MoSe_2$ with space group 115 and Wyckoff positions d-g-0.

$MoSe_2$, 187, b-i-0

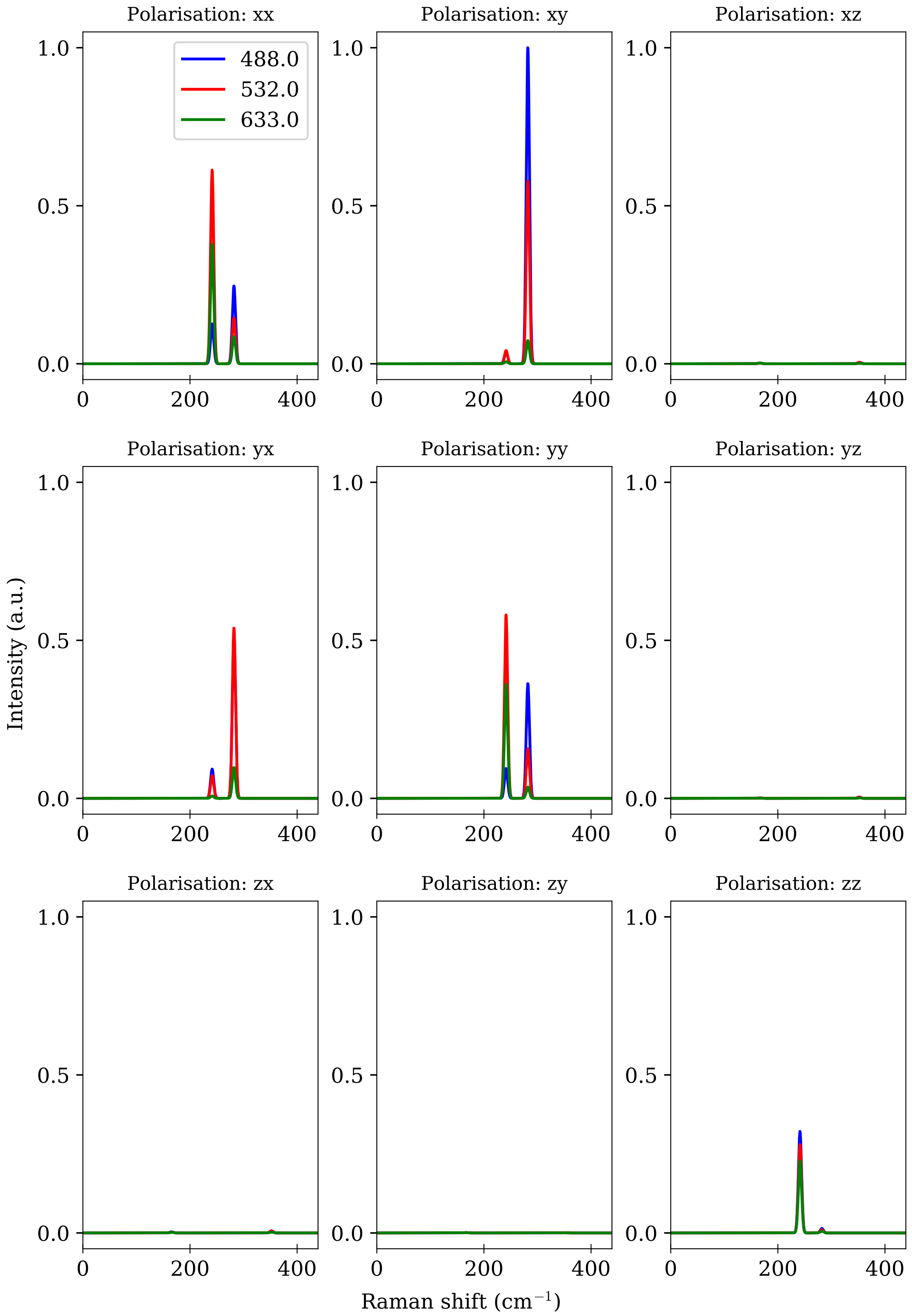

Supplementary Figure 350: Raman spectrum of $MoSe_2$ with space group 187 and Wyckoff positions b-i-0.

MoSeTe, 156, a-c-0

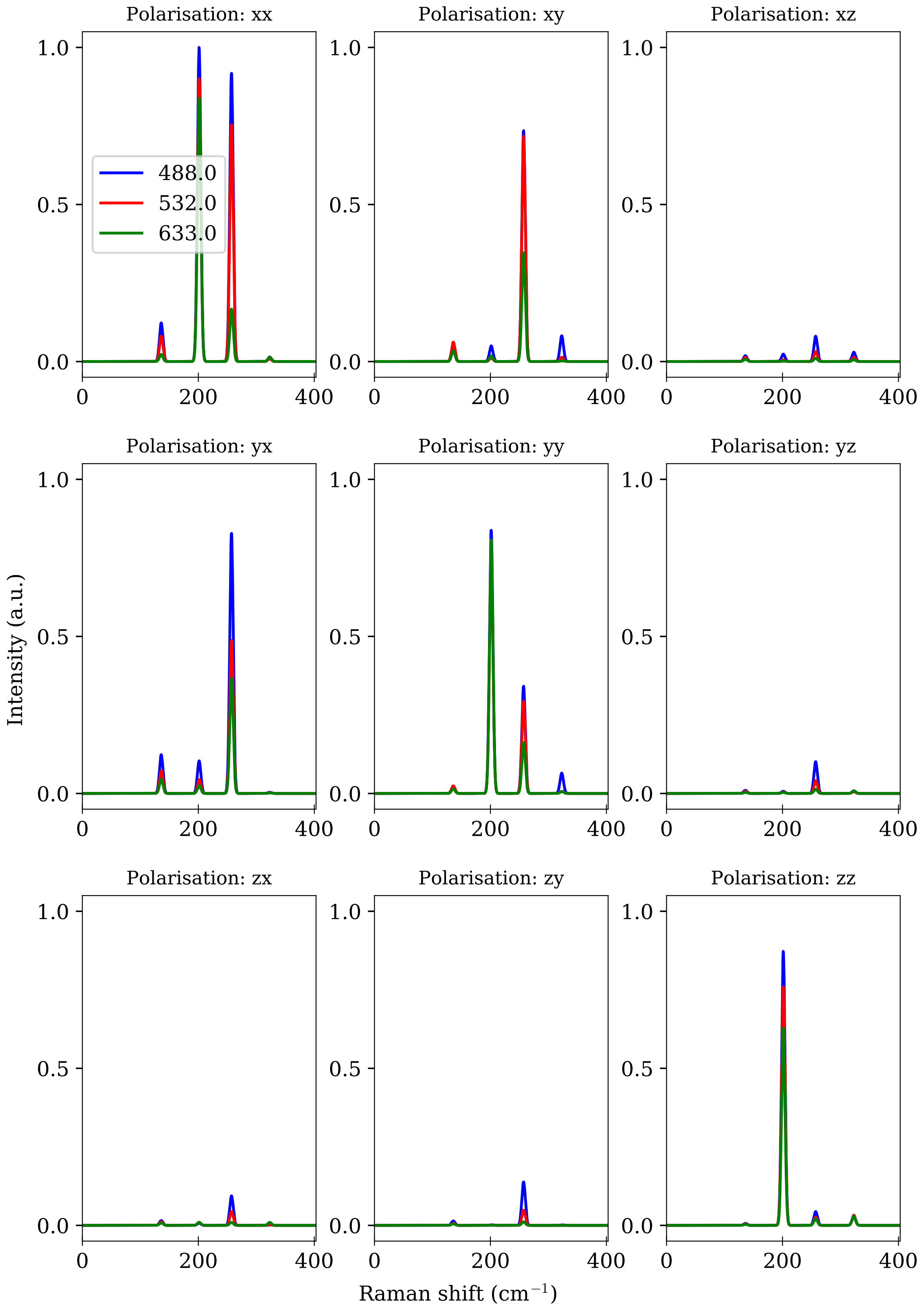

Supplementary Figure 351: Raman spectrum of MoSeTe with space group 156 and Wyckoff positions a-c-0.

MoSSe, 156, a-c-0

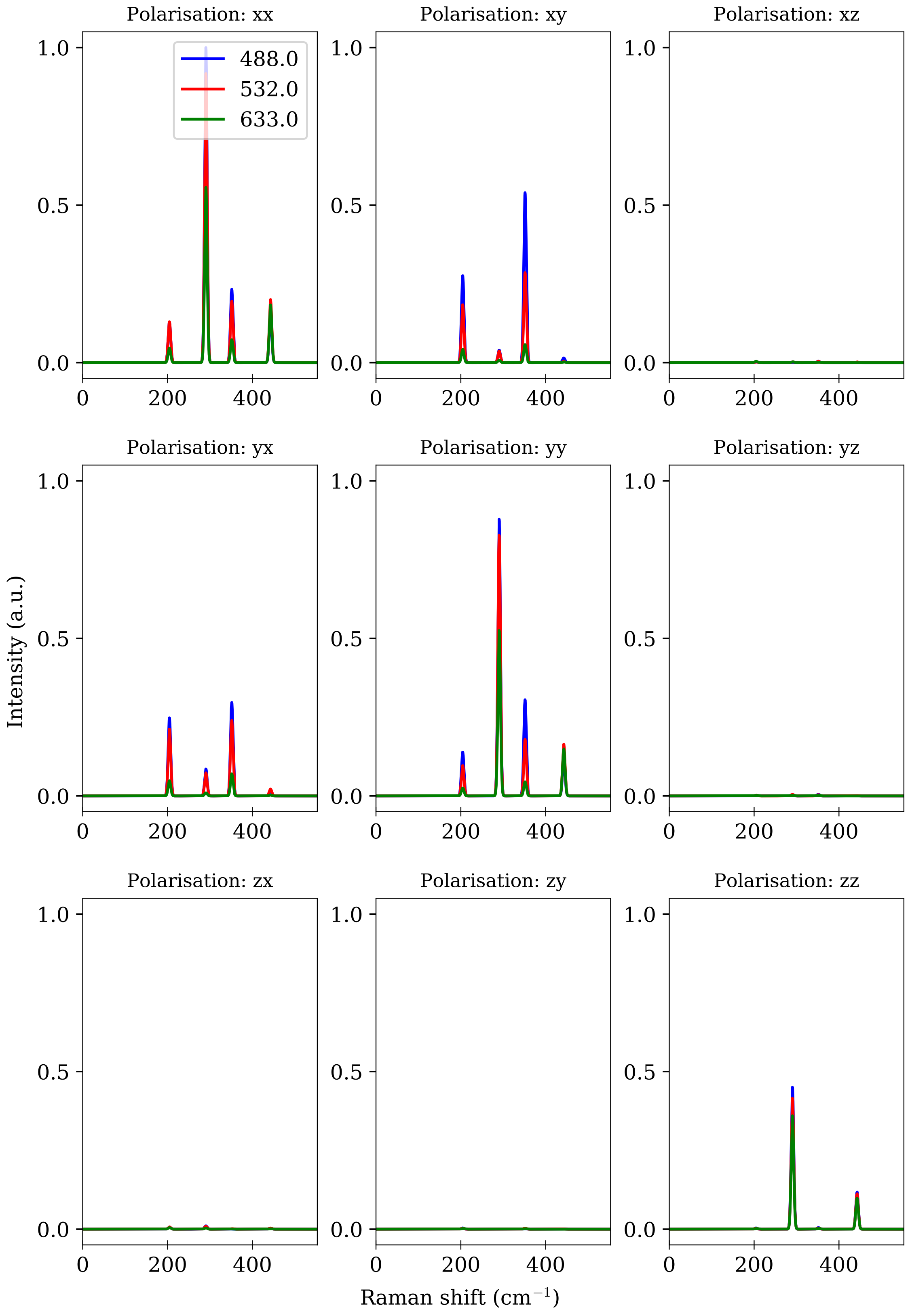

Supplementary Figure 352: Raman spectrum of MoSSe with space group 156 and Wyckoff positions a-c-0.

MoSTe, 156, a-c-0

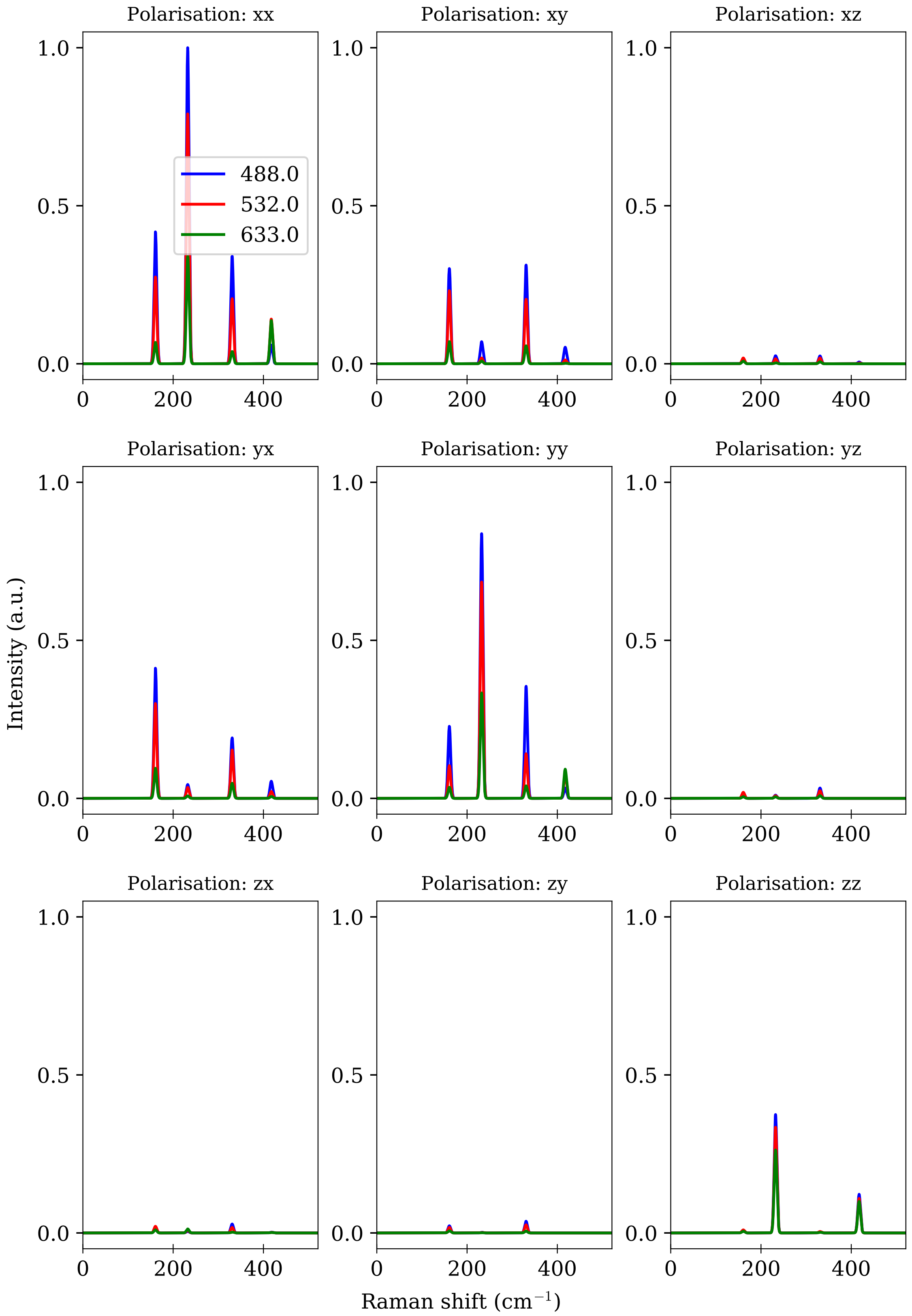

Supplementary Figure 353: Raman spectrum of MoSTe with space group 156 and Wyckoff positions a-c-0.

MoTe$_2$, 187, b-i-0

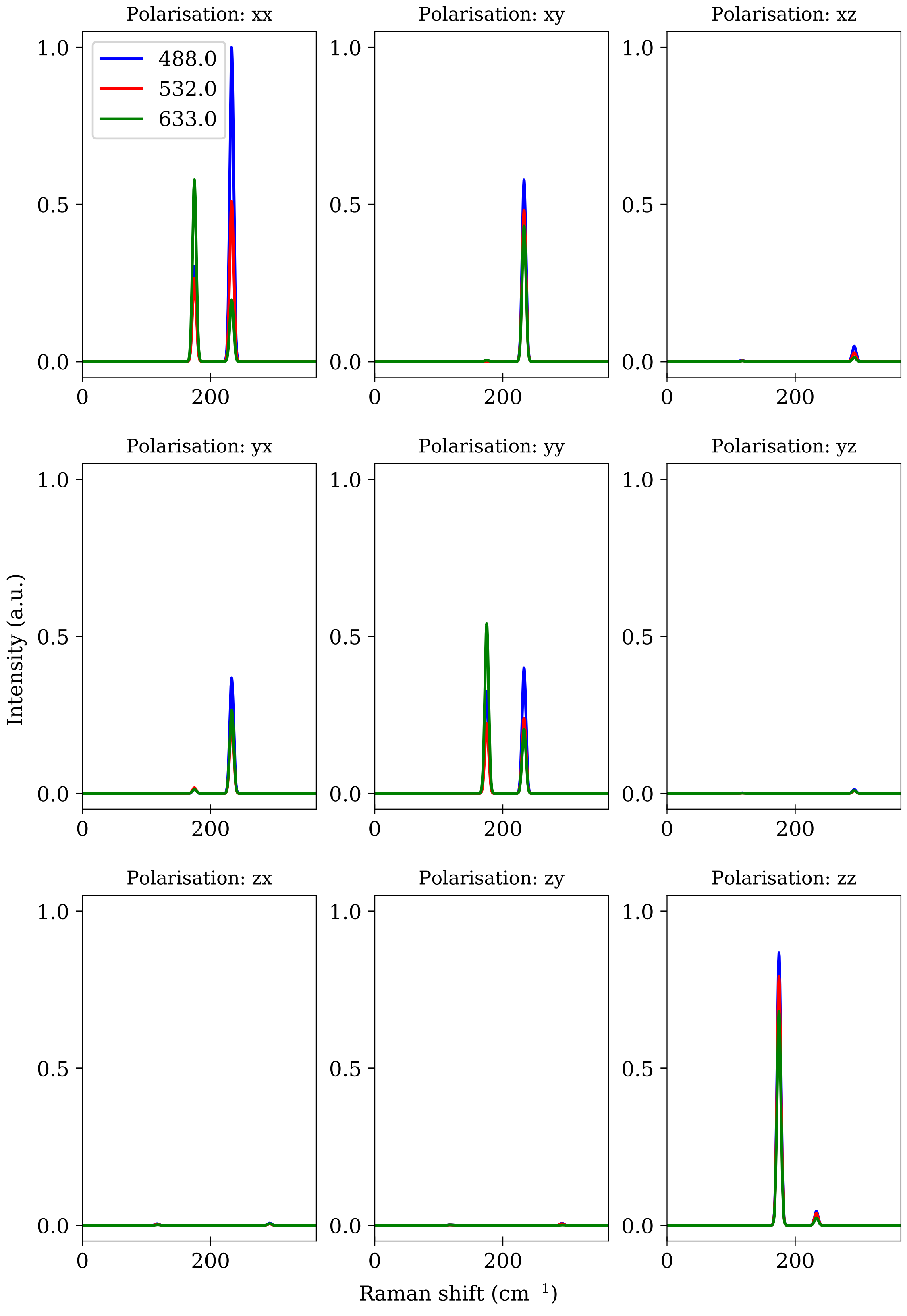

Supplementary Figure 354: Raman spectrum of MoTe$_2$ with space group 187 and Wyckoff positions b-i-0.

$Nb_2Br_2N_2$, 59, a-b-0

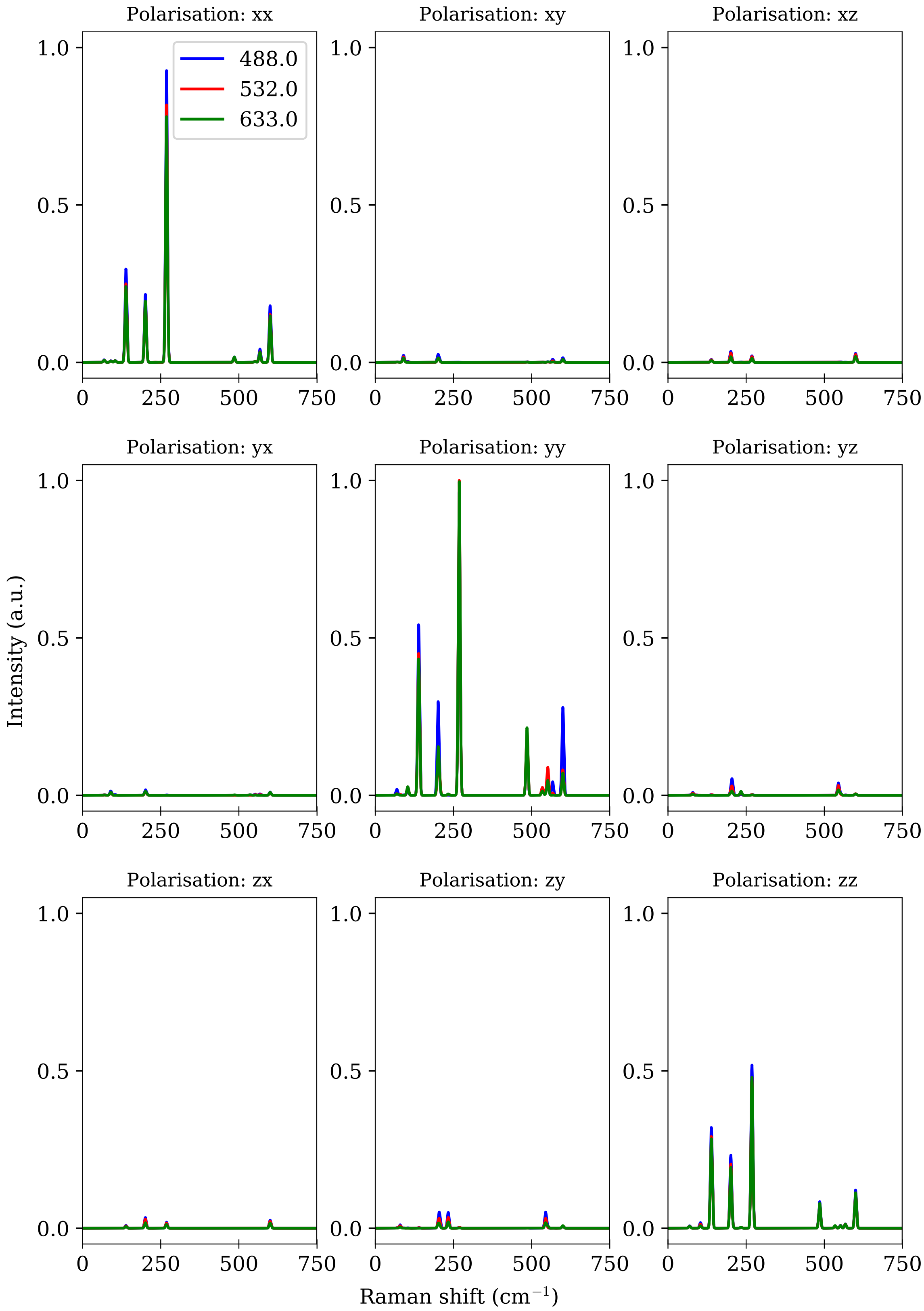

Supplementary Figure 355: Raman spectrum of $Nb_2Br_2N_2$ with space group 59 and Wyckoff positions a-b-0.

Nb_2CO_2, 164, b-d-0

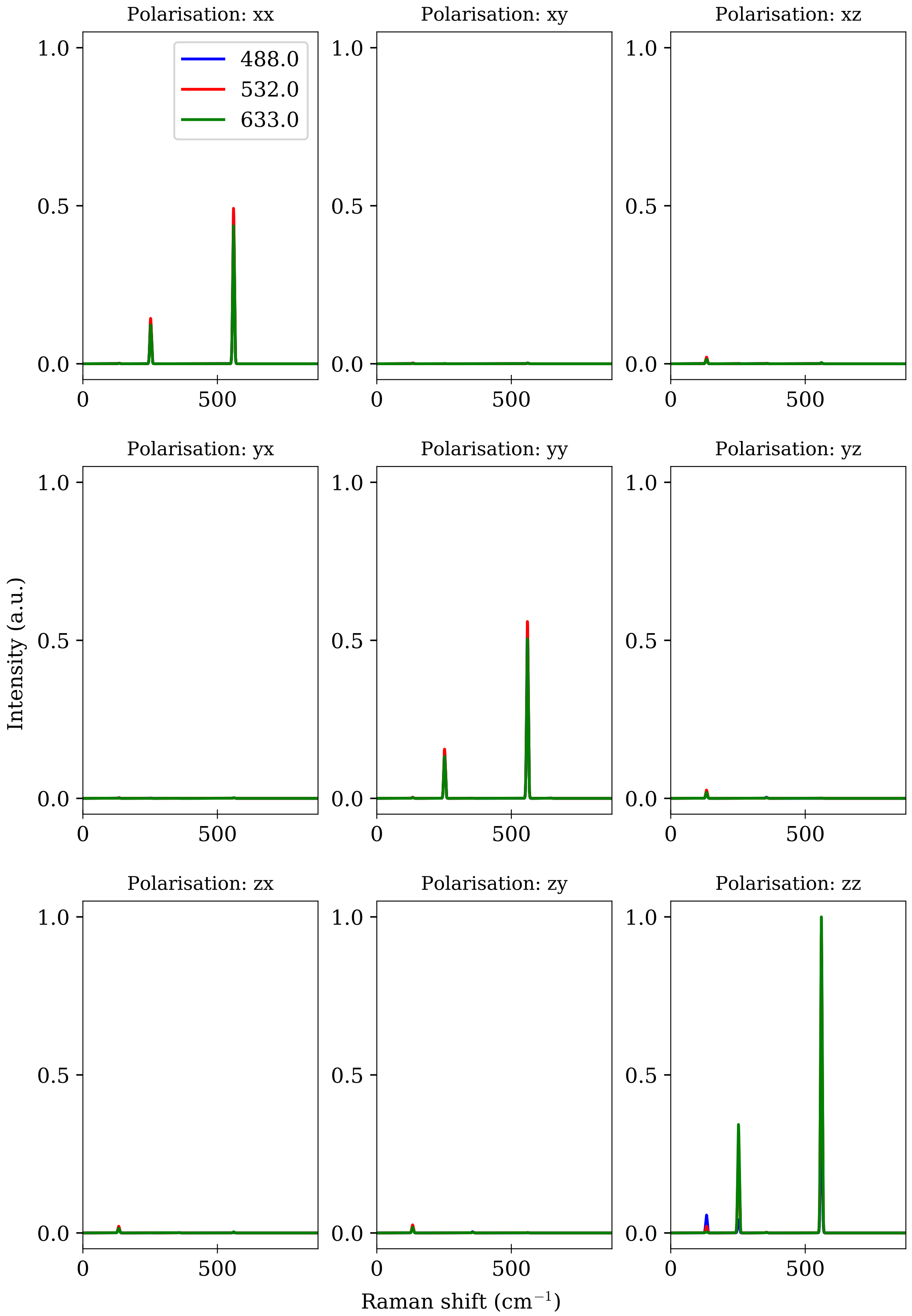

Supplementary Figure 356: Raman spectrum of Nb_2CO_2 with space group 164 and Wyckoff positions b-d-0.

Nb_2C, 164, b-d-0

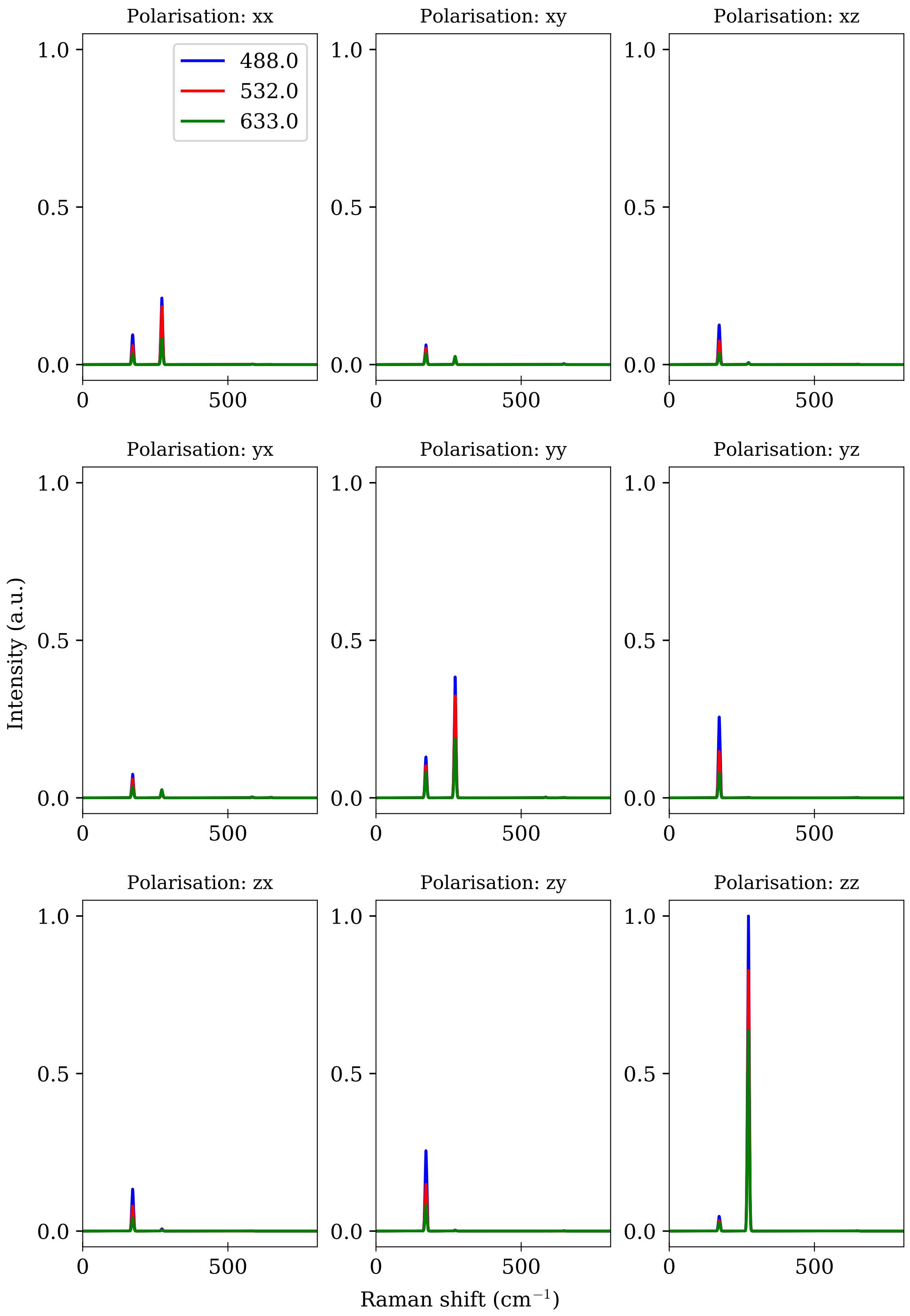

Supplementary Figure 357: Raman spectrum of Nb_2C with space group 164 and Wyckoff positions b-d-0.

Nb_2I_4, 11, e-0

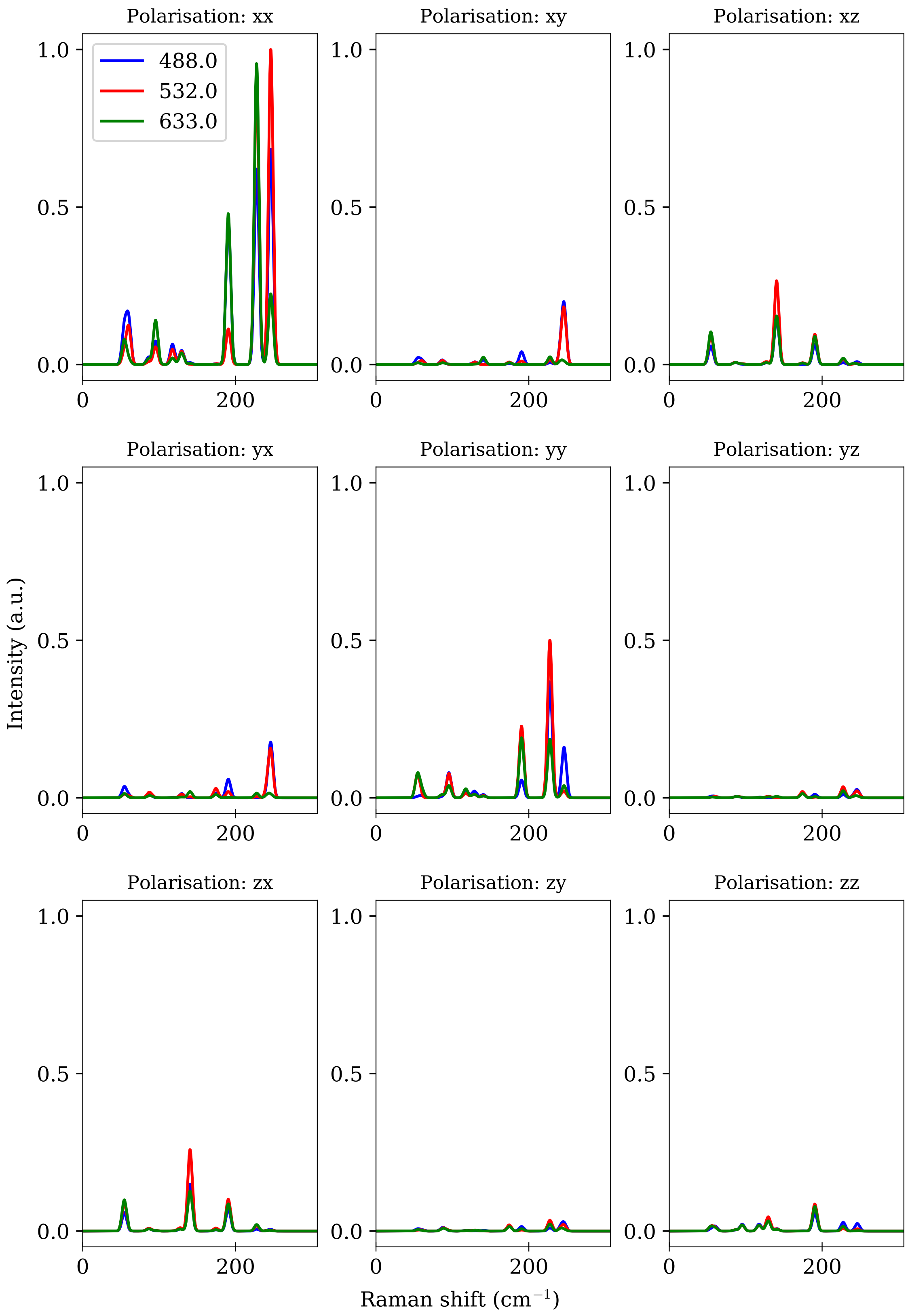

Supplementary Figure 358: Raman spectrum of Nb_2I_4 with space group 11 and Wyckoff positions e-0.

Nb_2N, 164, b-d-0

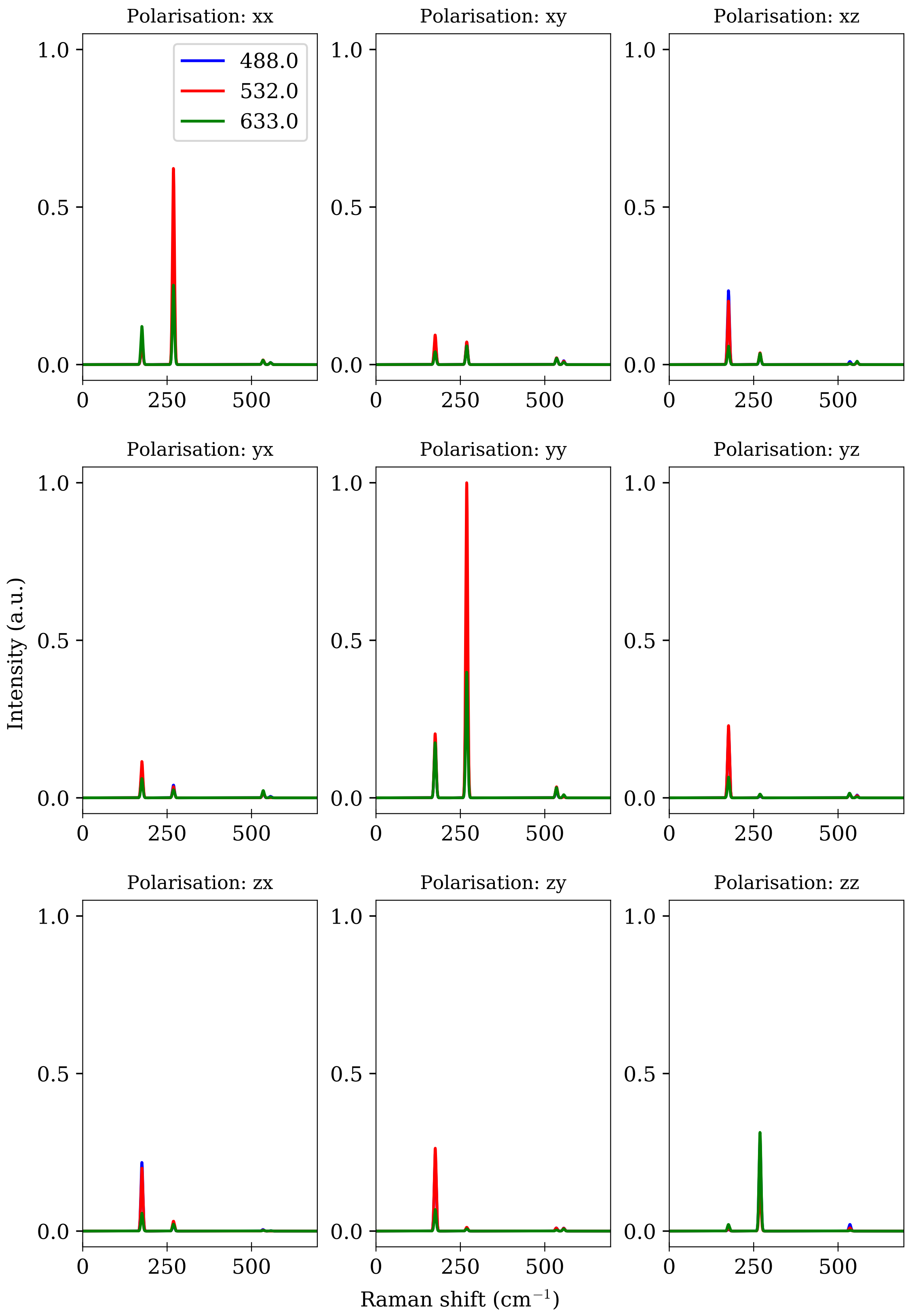

Supplementary Figure 359: Raman spectrum of Nb_2N with space group 164 and Wyckoff positions b-d-0.

Nb_2O_2, 6, a-b-0

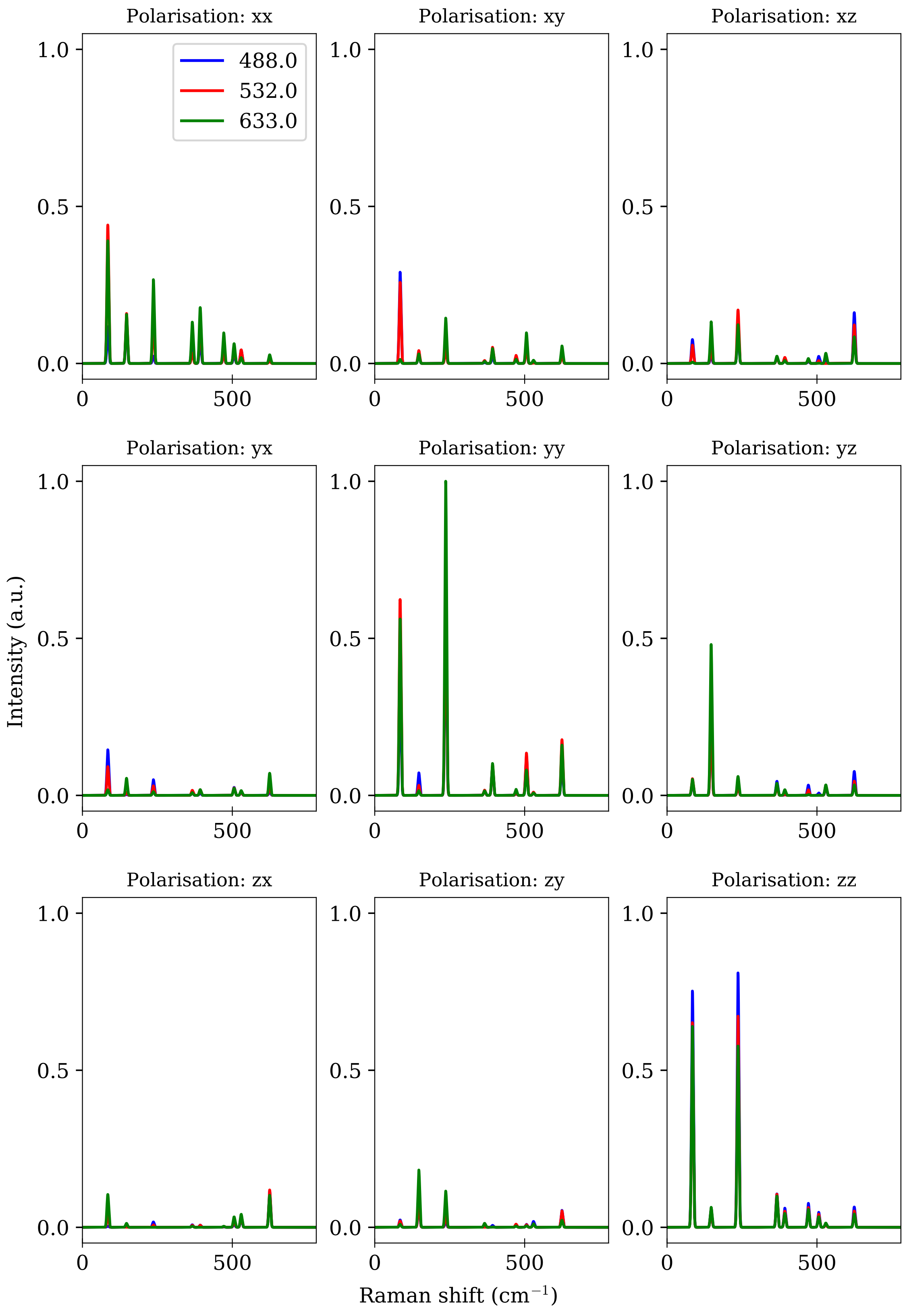

Supplementary Figure 360: Raman spectrum of Nb_2O_2 with space group 6 and Wyckoff positions a-b-0.

$Nb_2P_2S_6$, 2, i-0

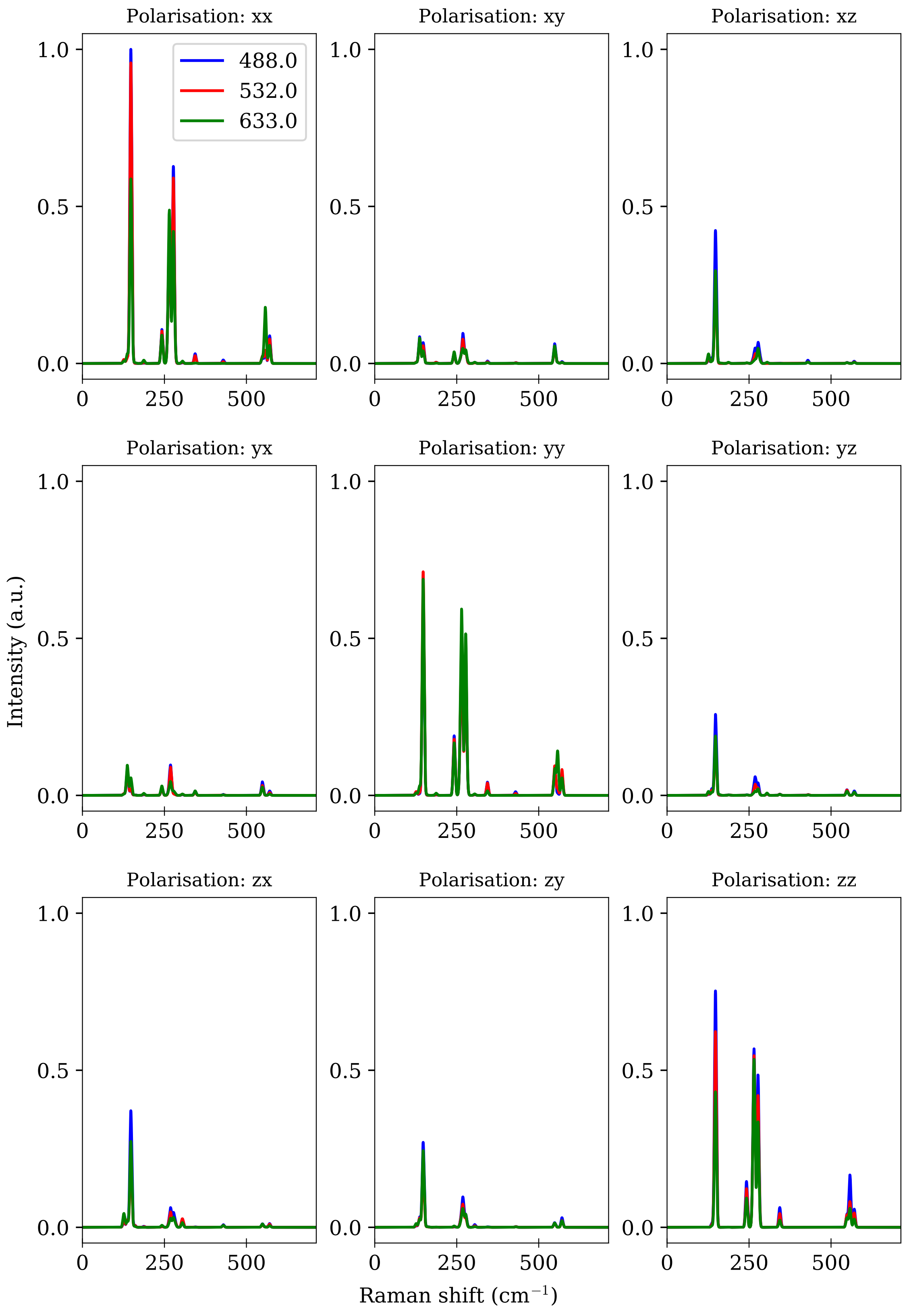

Supplementary Figure 361: Raman spectrum of $Nb_2P_2S_6$ with space group 2 and Wyckoff positions i-0.

Nb_2S_2, 164, d-0

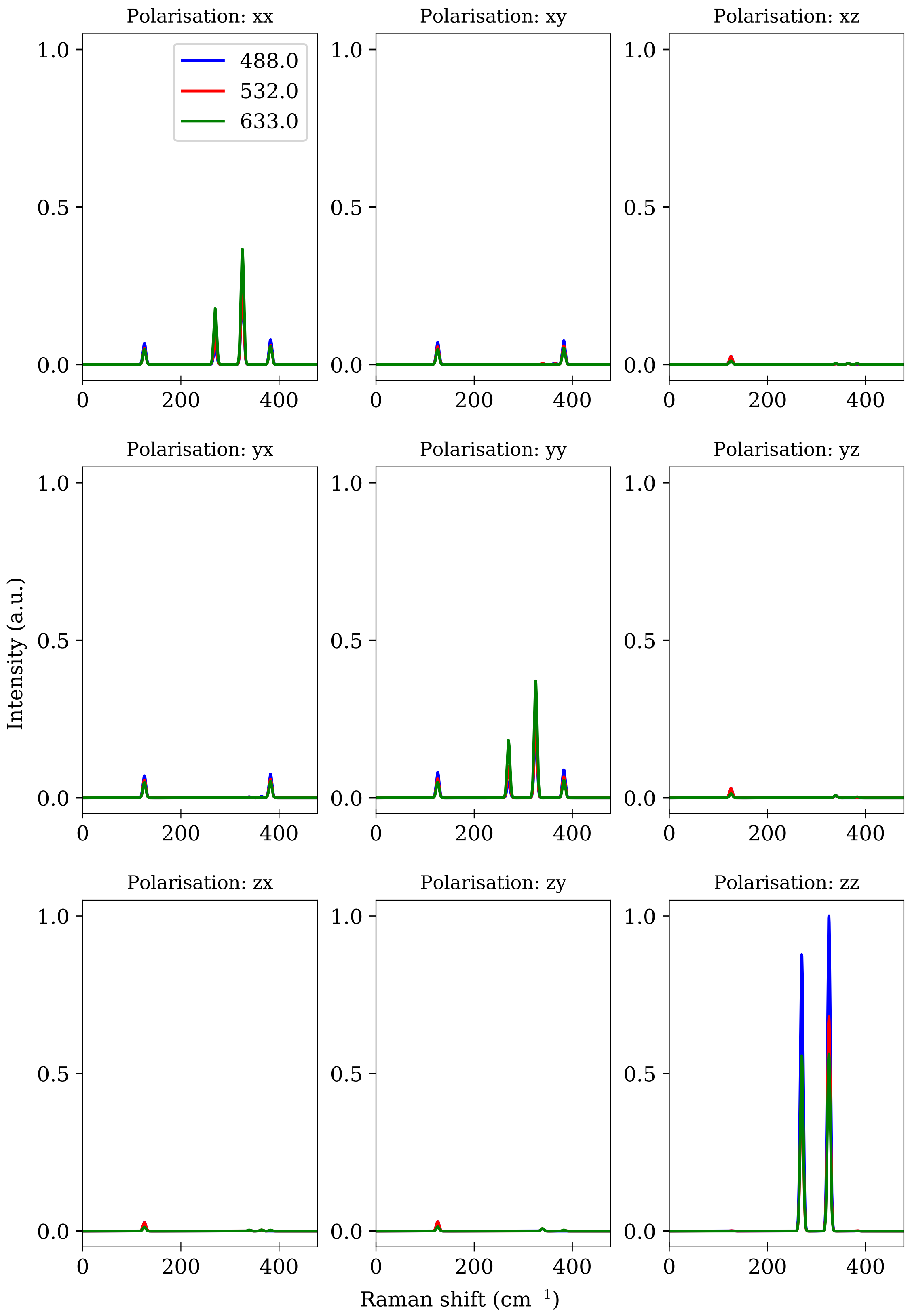

Supplementary Figure 362: Raman spectrum of Nb_2S_2 with space group 164 and Wyckoff positions d-0.

Nb_2Se_2, 164, d-0

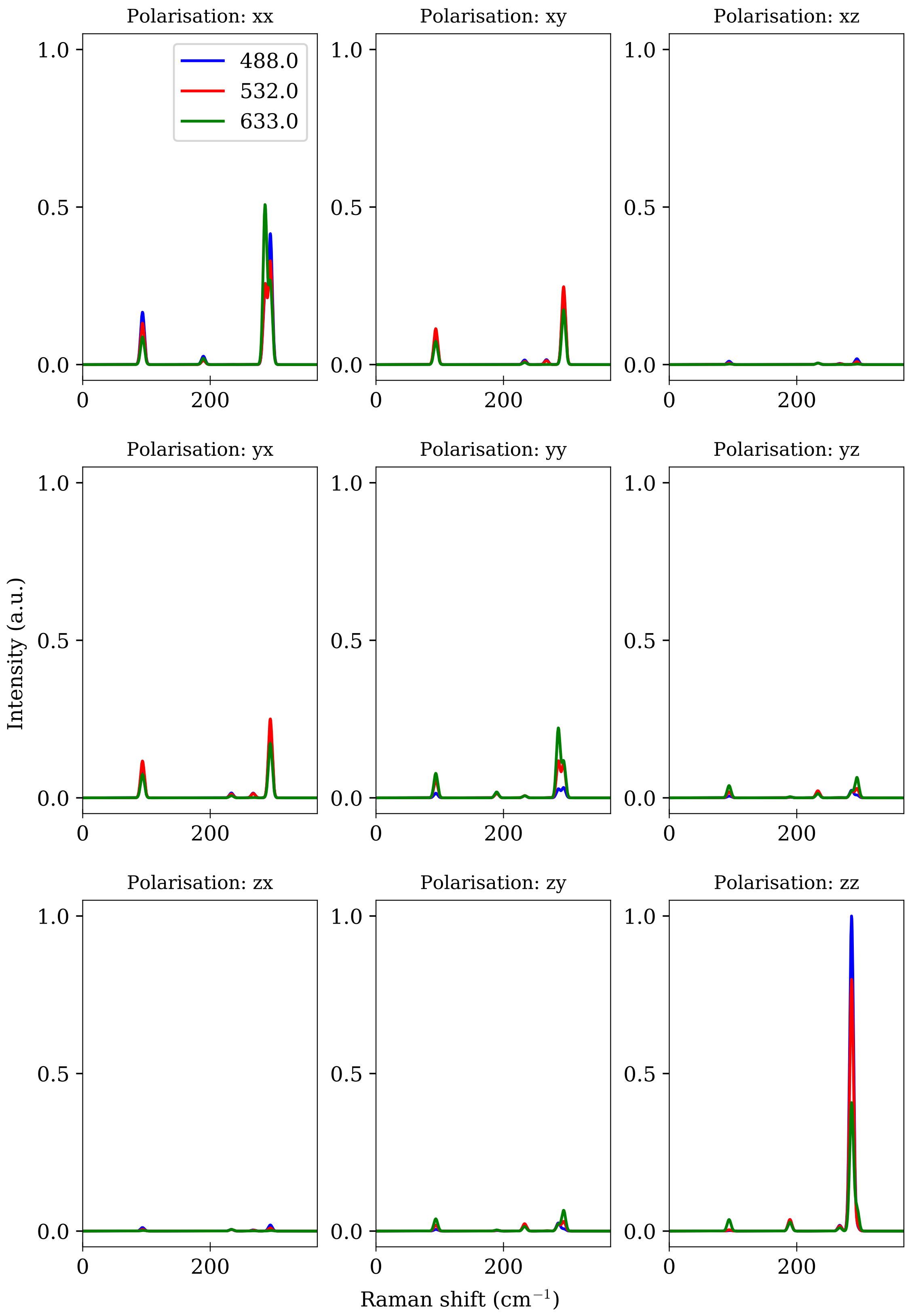

Supplementary Figure 363: Raman spectrum of Nb_2Se_2 with space group 164 and Wyckoff positions d-0.

Nb_2Te_2, 129, b-c-0

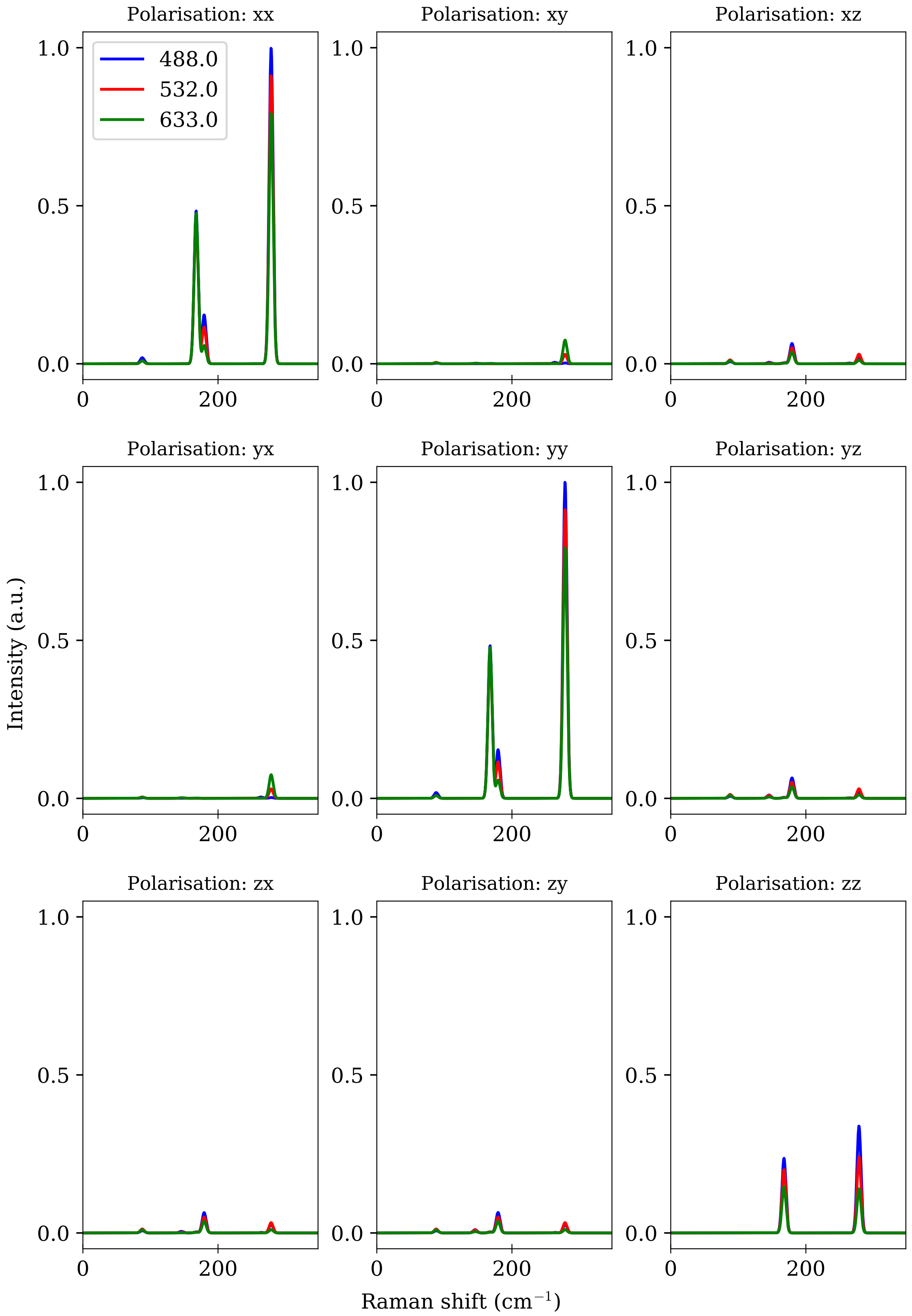

Supplementary Figure 364: Raman spectrum of Nb_2Te_2 with space group 129 and Wyckoff positions b-c-0.

Nb_2Te_2, 164, d-0

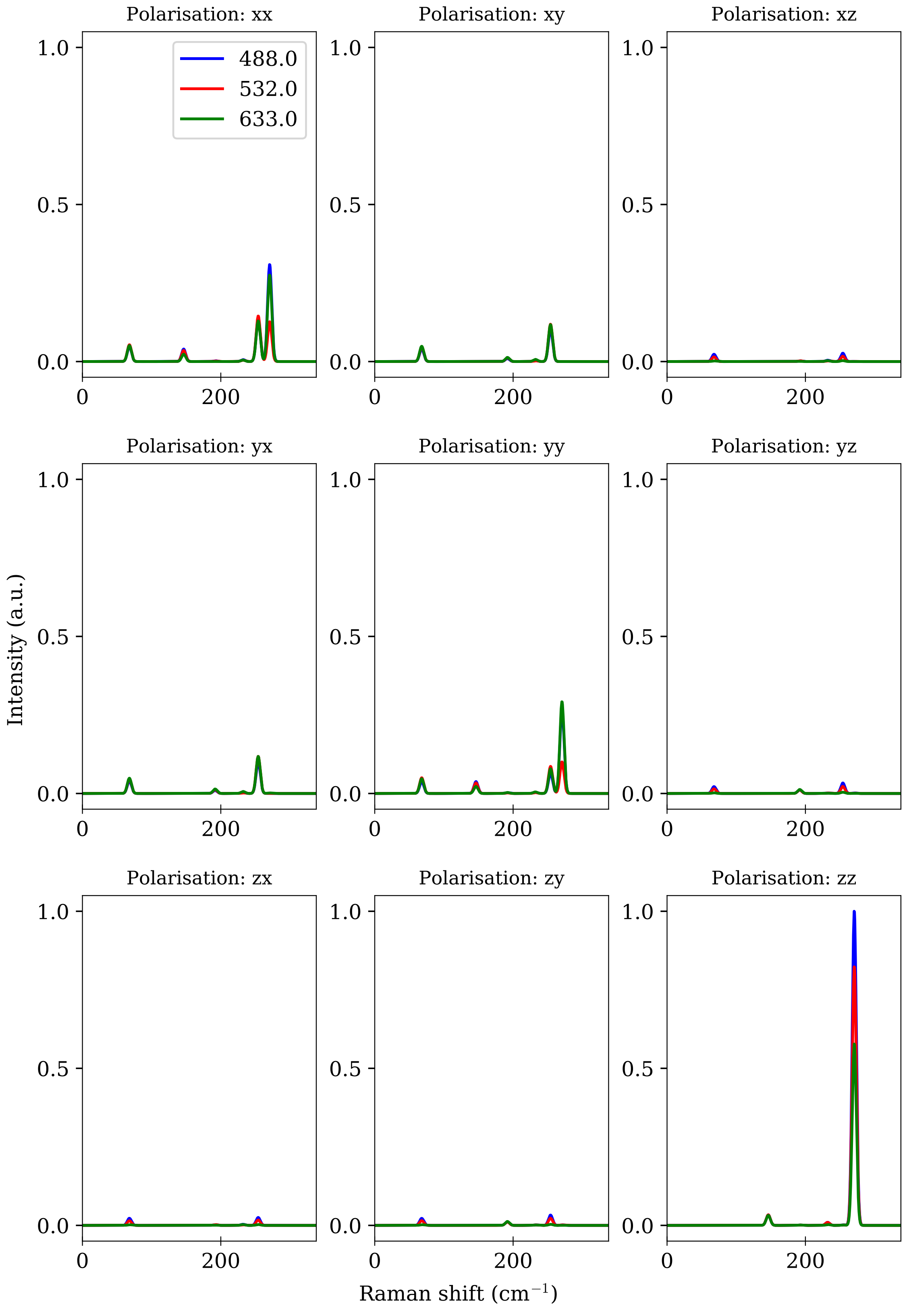

Supplementary Figure 365: Raman spectrum of Nb_2Te_2 with space group 164 and Wyckoff positions d-0.

$Nb_3C_2O_2$, 187, a-g-h-i-0

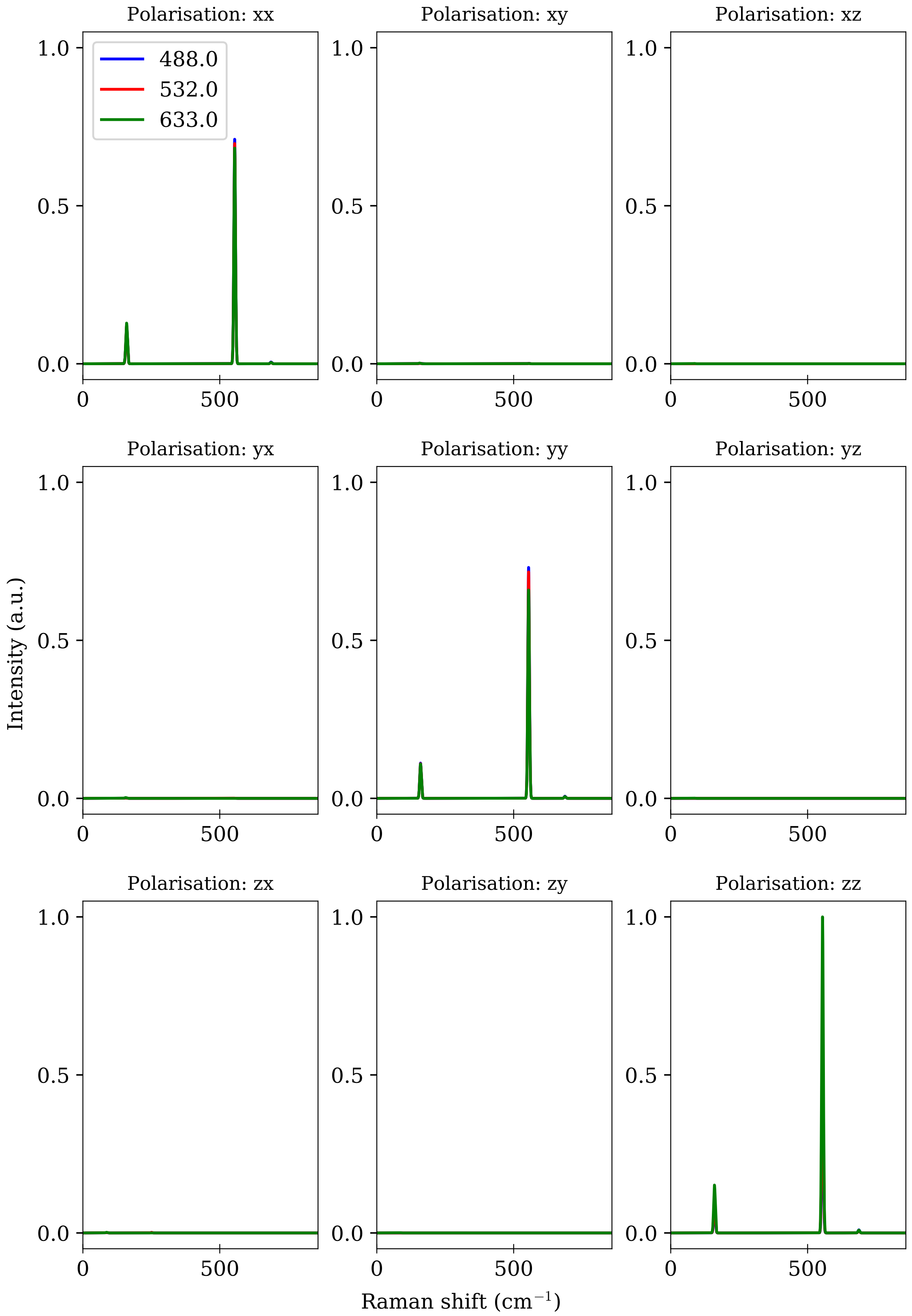

Supplementary Figure 366: Raman spectrum of $Nb_3C_2O_2$ with space group 187 and Wyckoff positions a-g-h-i-0.

Nb_3C_2, 187, b-h-i-0

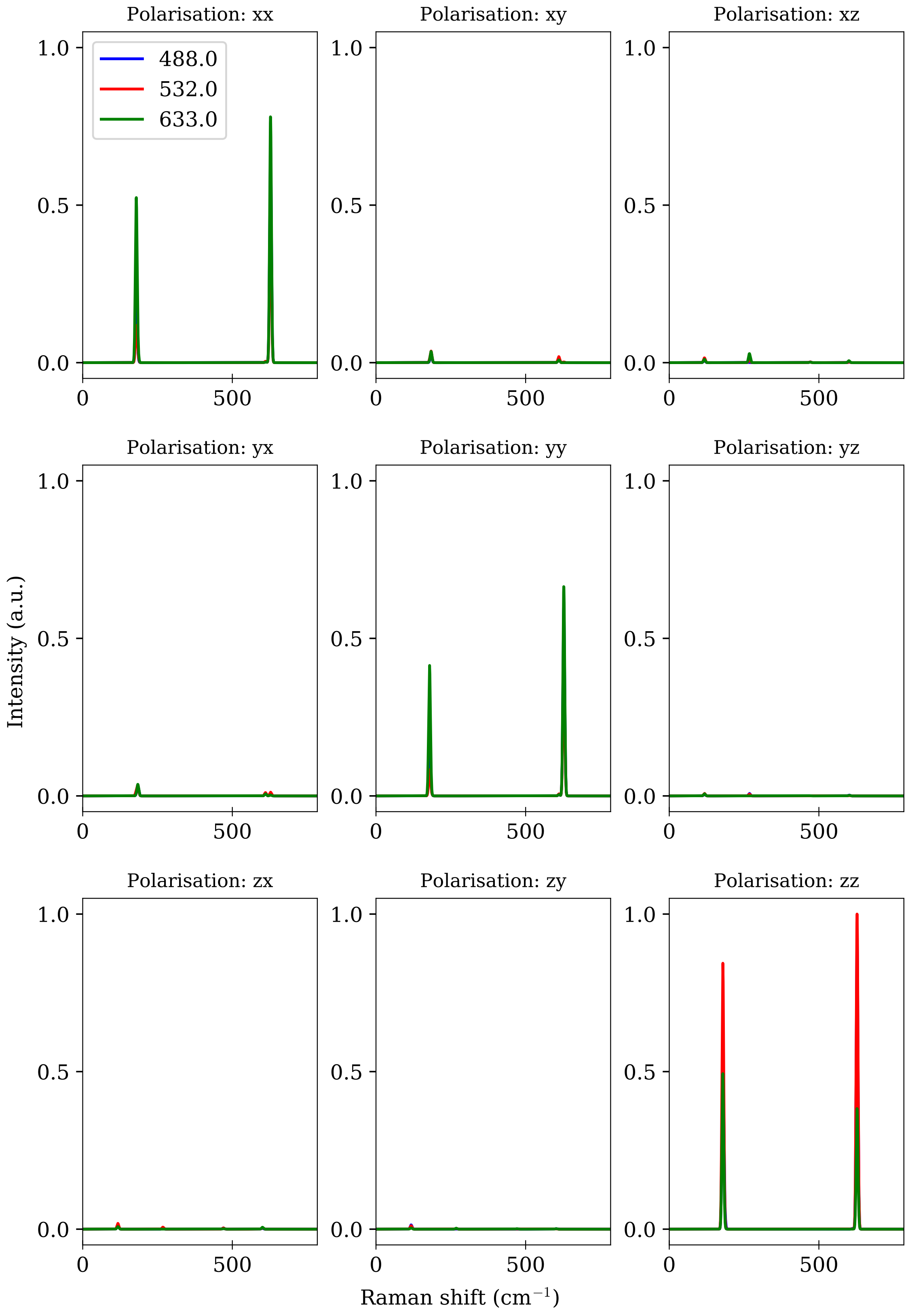

Supplementary Figure 367: Raman spectrum of Nb_3C_2 with space group 187 and Wyckoff positions b-h-i-0.

Nb_3N_2, 187, b-h-i-0

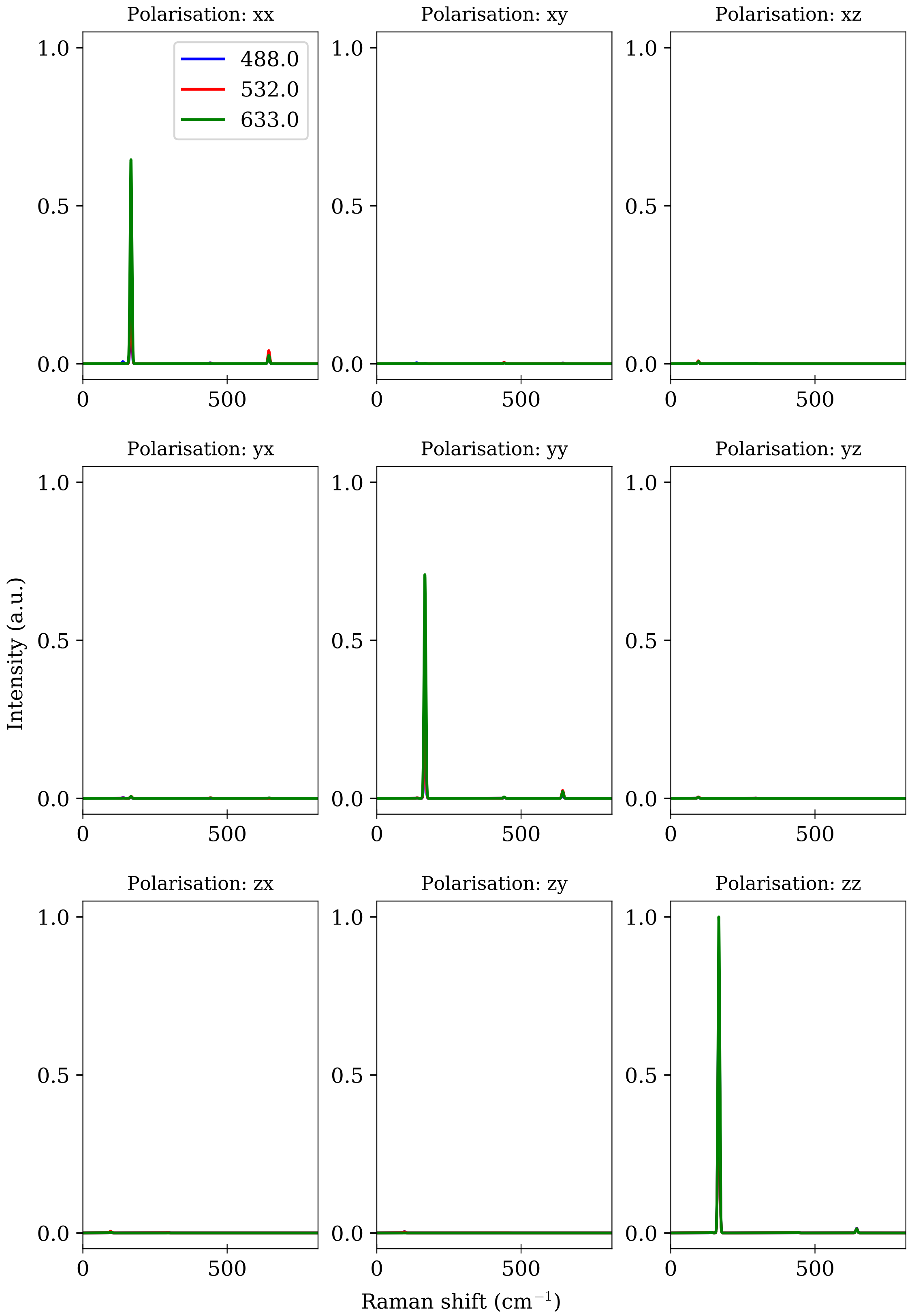

Supplementary Figure 368: Raman spectrum of Nb_3N_2 with space group 187 and Wyckoff positions b-h-i-0.

NbAsSb, 156, a-b-c-0

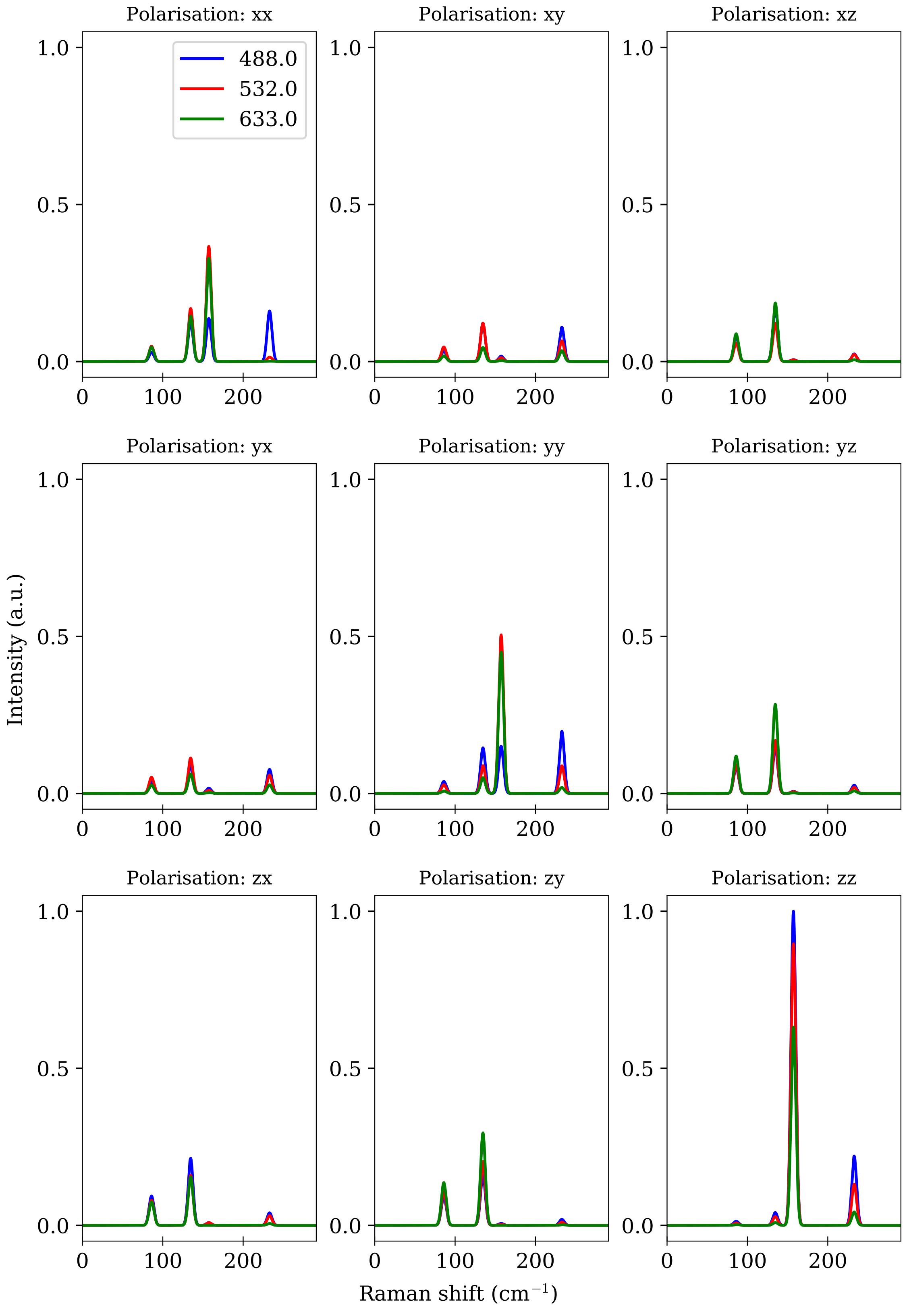

Supplementary Figure 369: Raman spectrum of NbAsSb with space group 156 and Wyckoff positions a-b-c-0.

NbH_2, 187, a-i-0

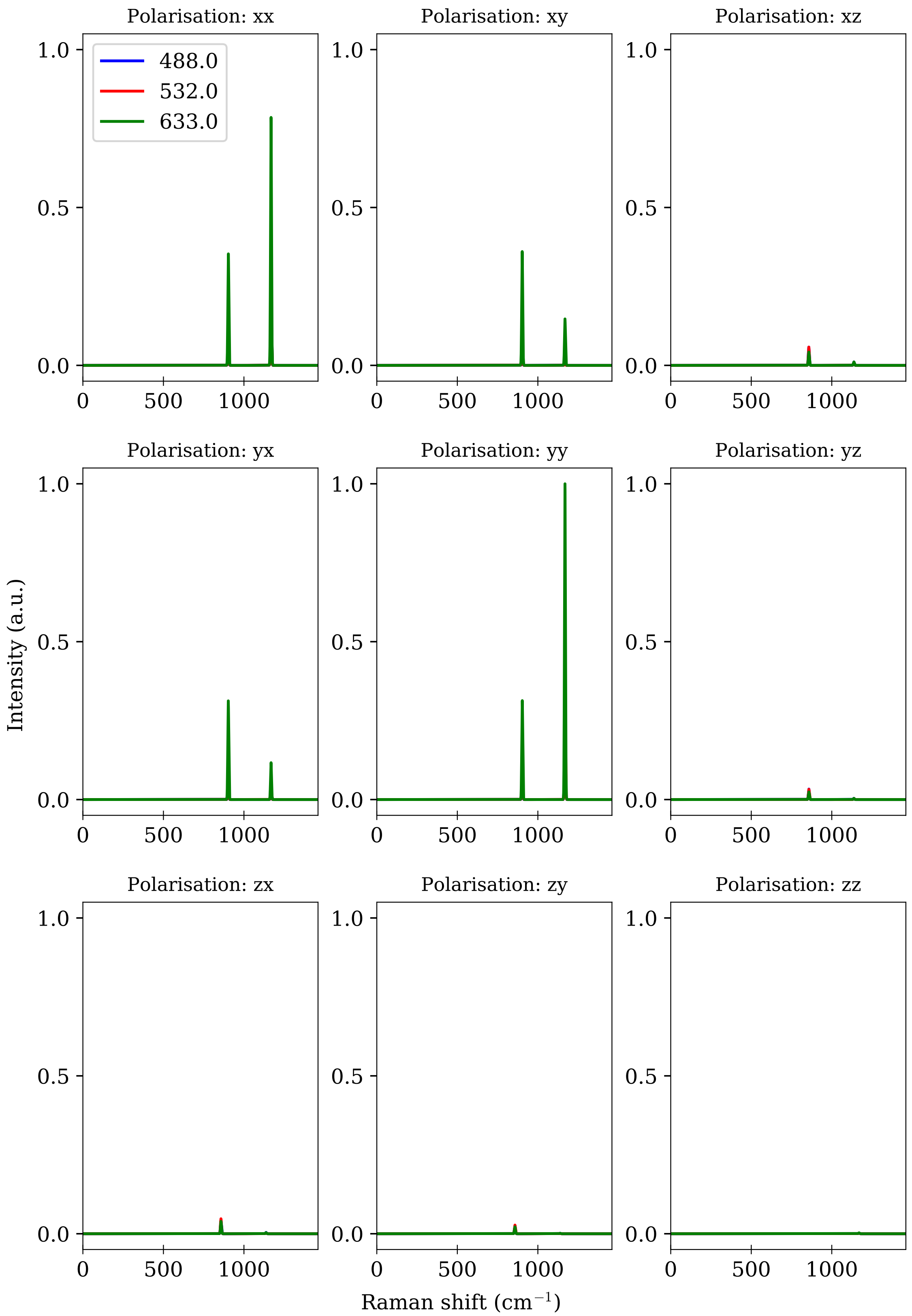

Supplementary Figure 370: Raman spectrum of NbH_2 with space group 187 and Wyckoff positions a-i-0.

NbS_2, 115, d-g-0

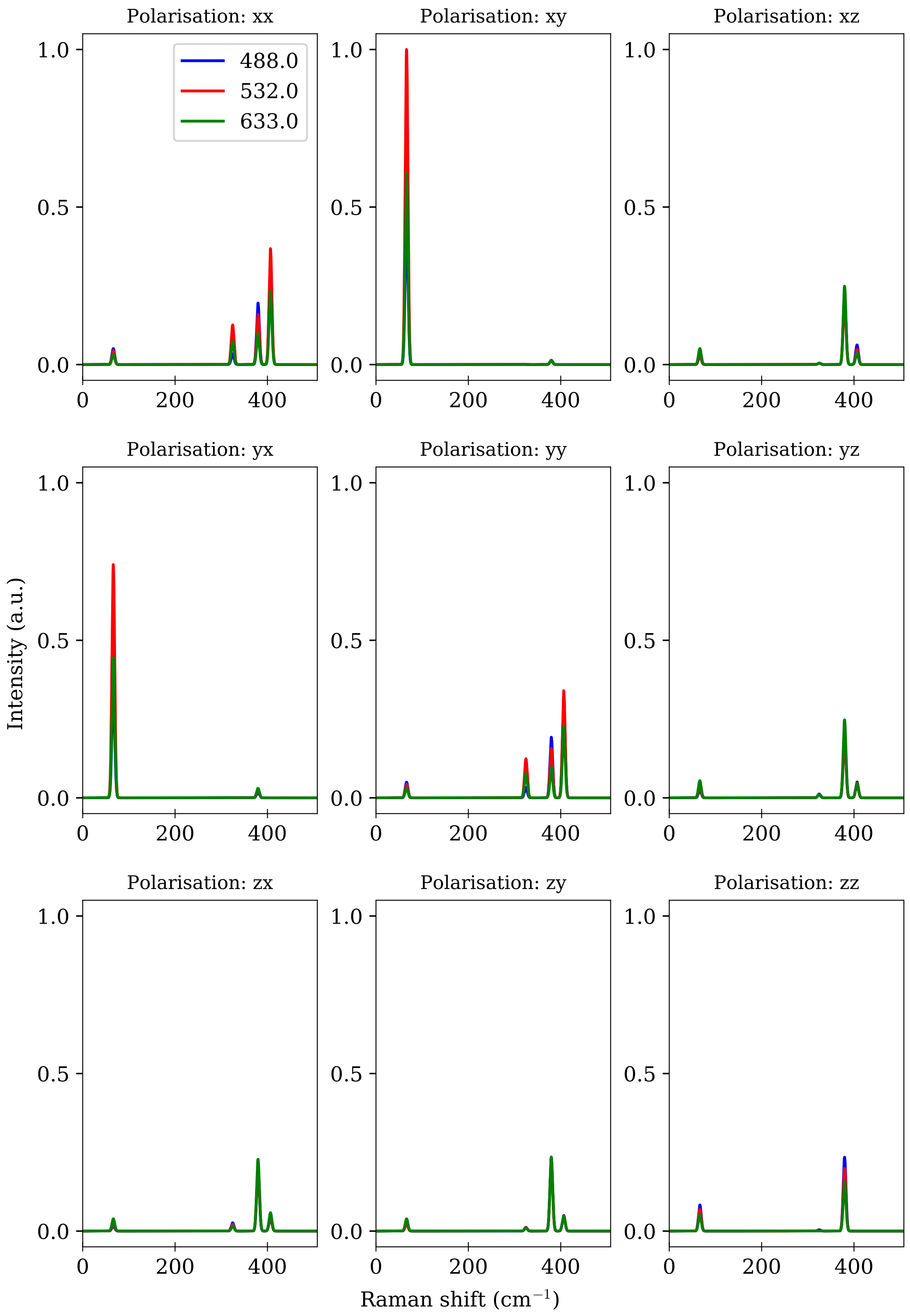

Supplementary Figure 371: Raman spectrum of NbS_2 with space group 115 and Wyckoff positions d-g-0.

NbS_2, 164, b-d-0

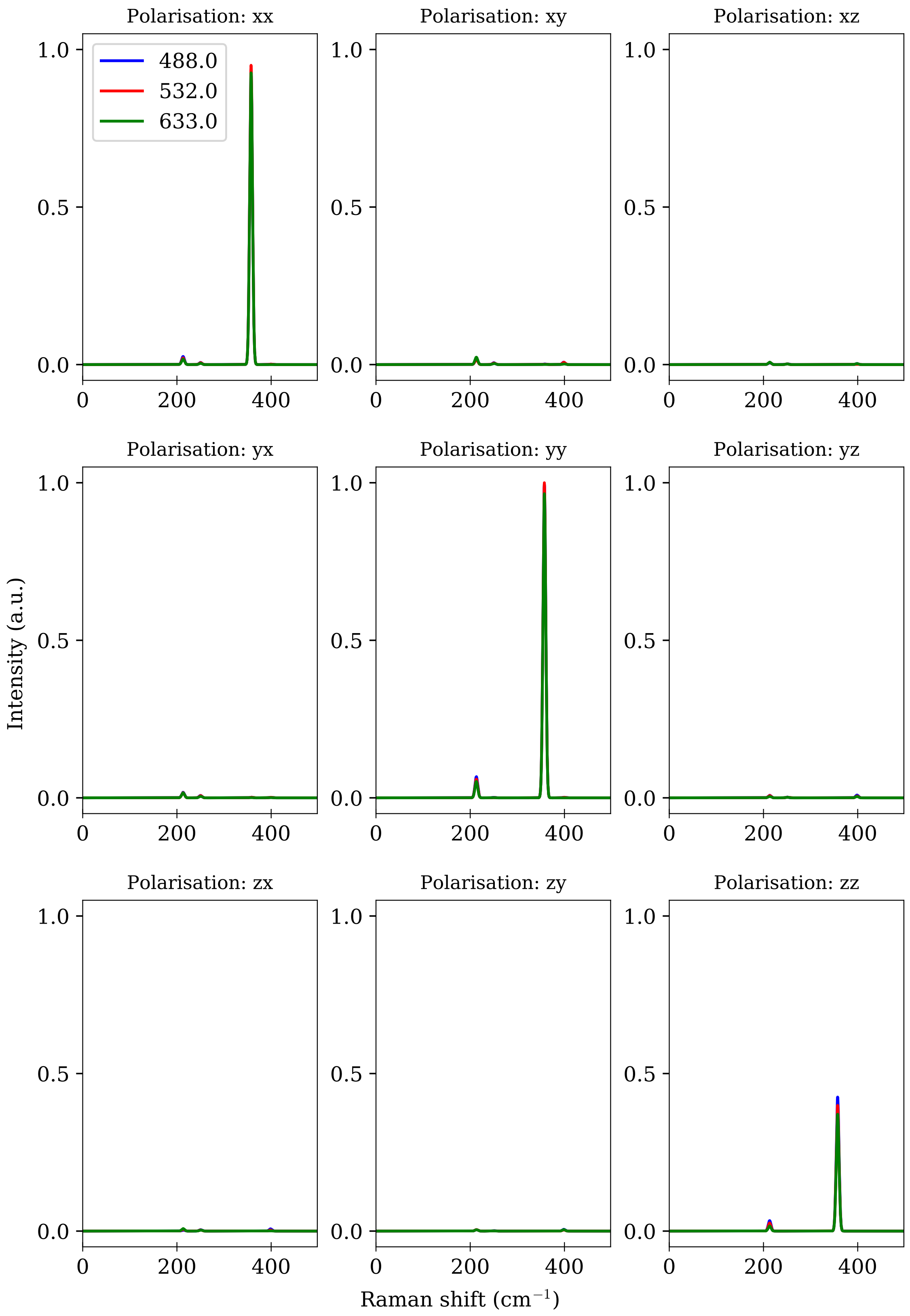

Supplementary Figure 372: Raman spectrum of NbS_2 with space group 164 and Wyckoff positions b-d-0.

$NbSb_2$, 164, b-d-0

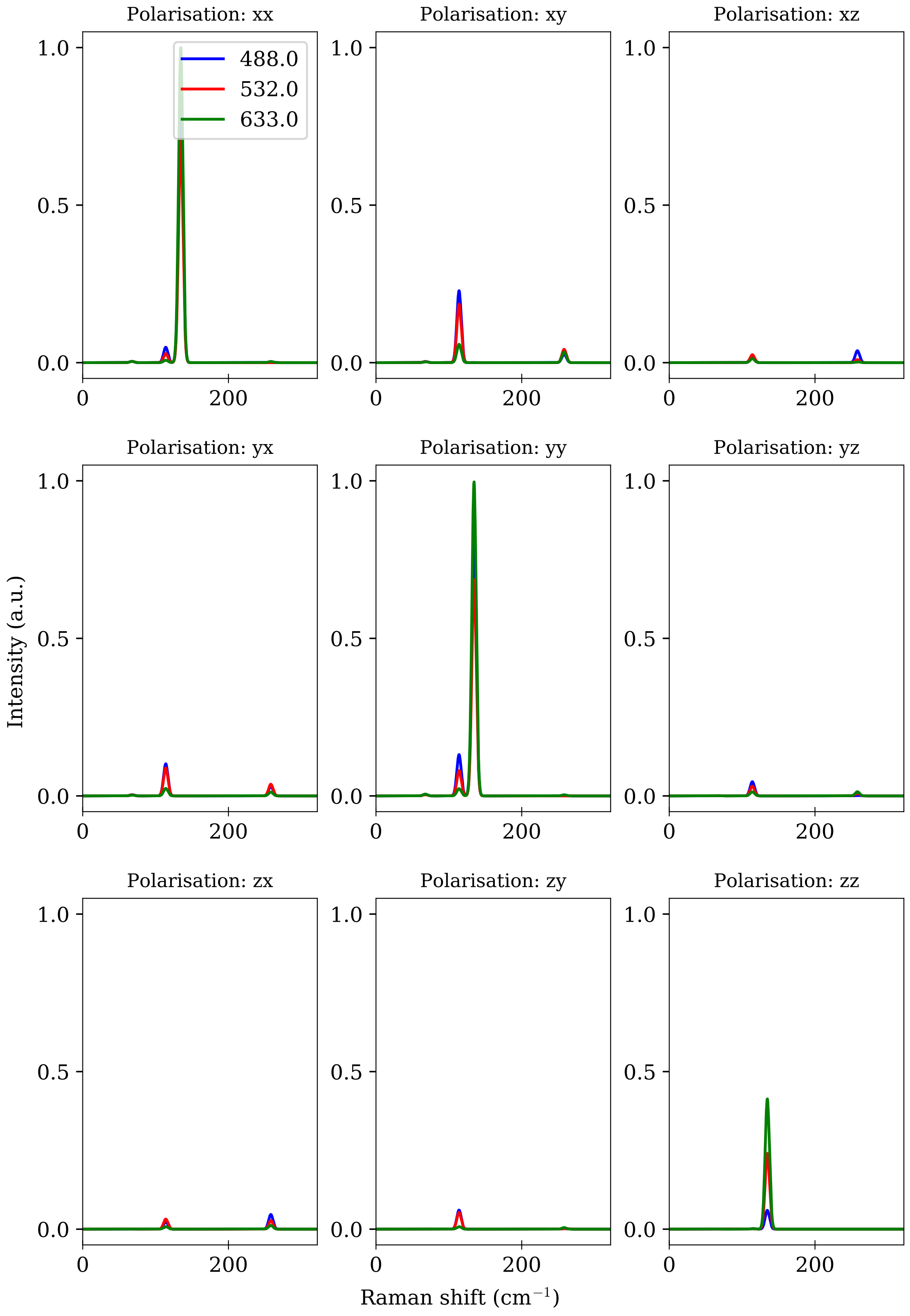

Supplementary Figure 373: Raman spectrum of $NbSb_2$ with space group 164 and Wyckoff positions b-d-0.

$NbSb_2$, 187, a-i-0

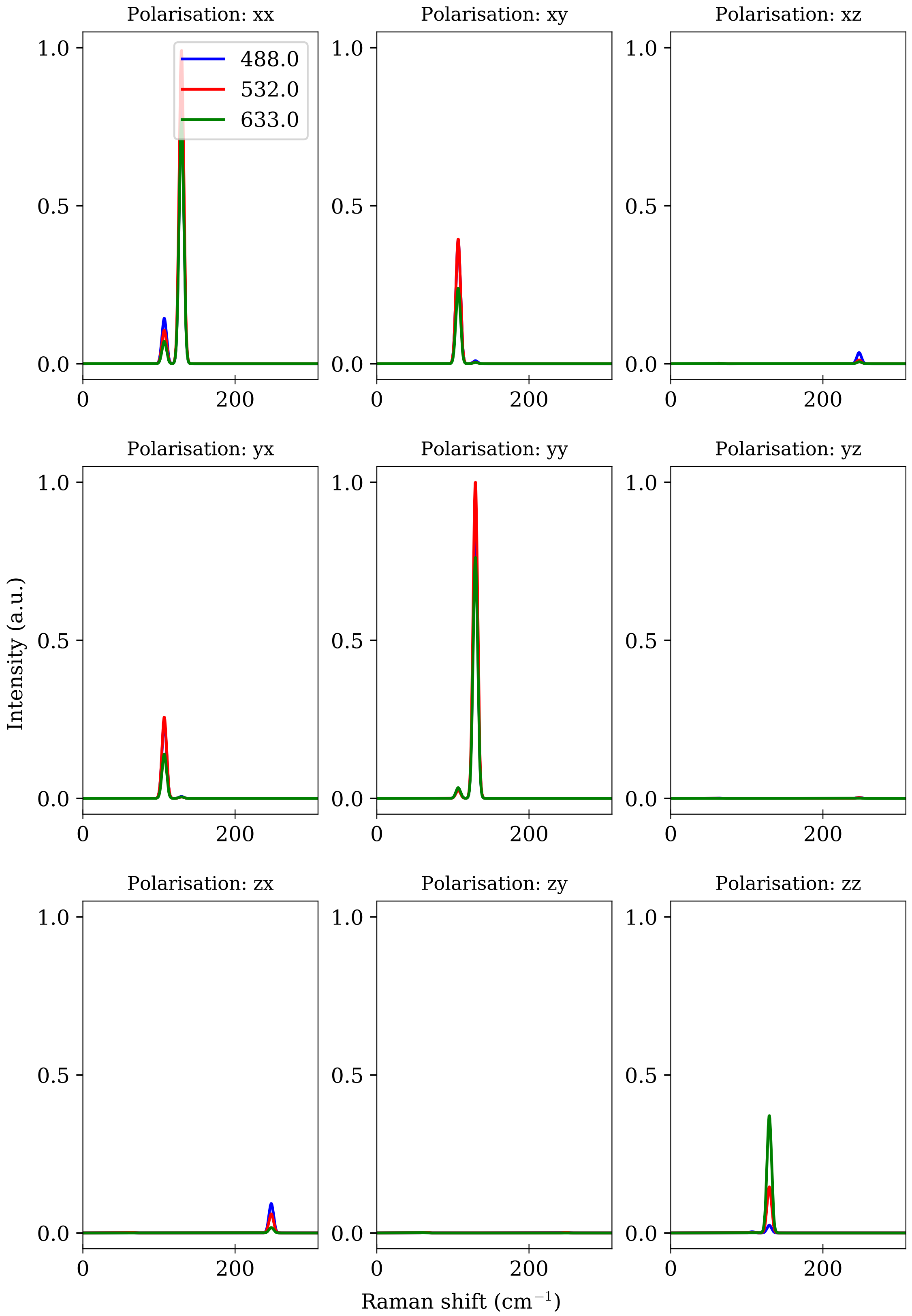

Supplementary Figure 374: Raman spectrum of $NbSb_2$ with space group 187 and Wyckoff positions a-i-0.

$NbSe_2$, 164, b-d-0

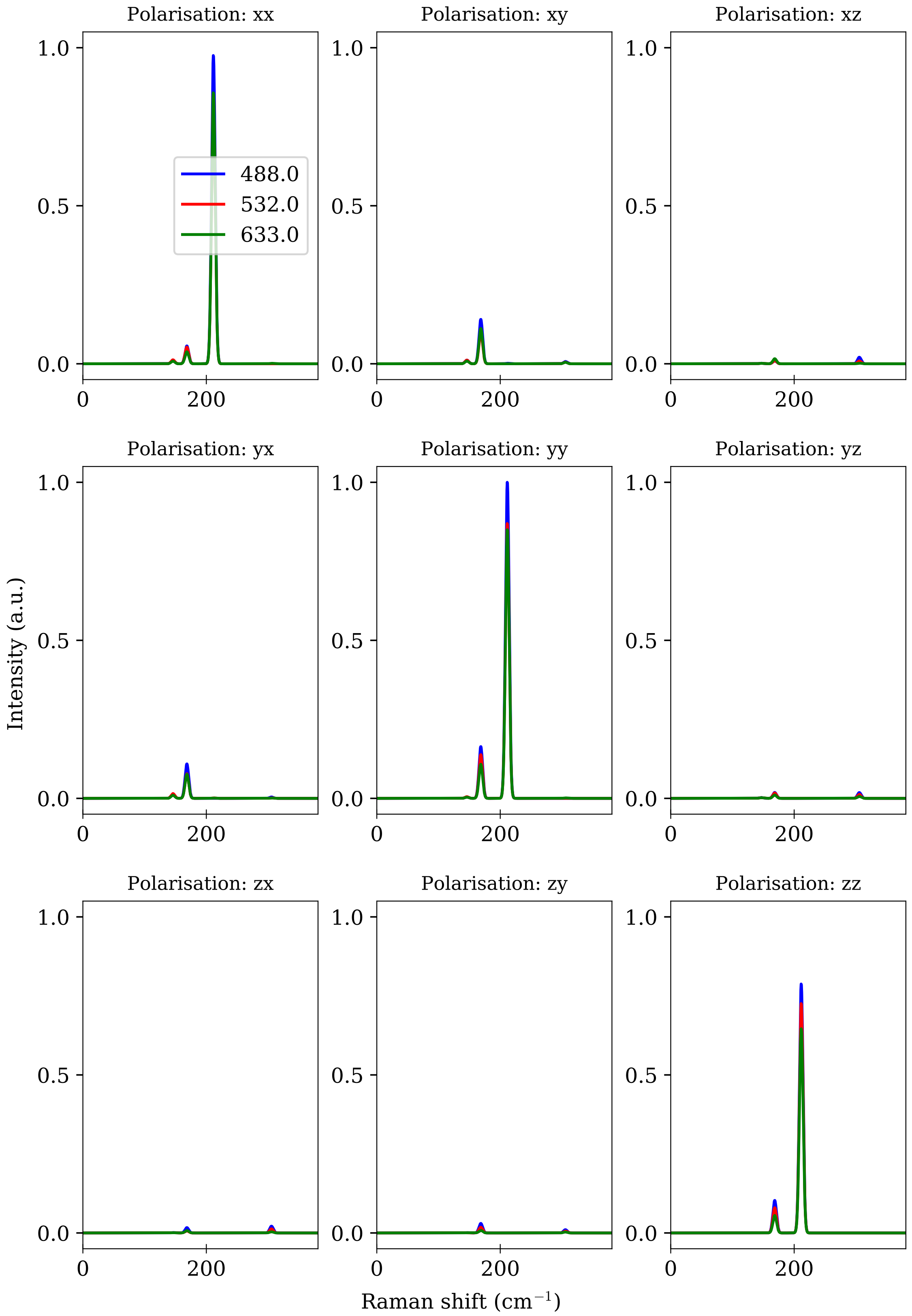

Supplementary Figure 375: Raman spectrum of $NbSe_2$ with space group 164 and Wyckoff positions b-d-0.

NbSeTe, 156, a-b-c-0

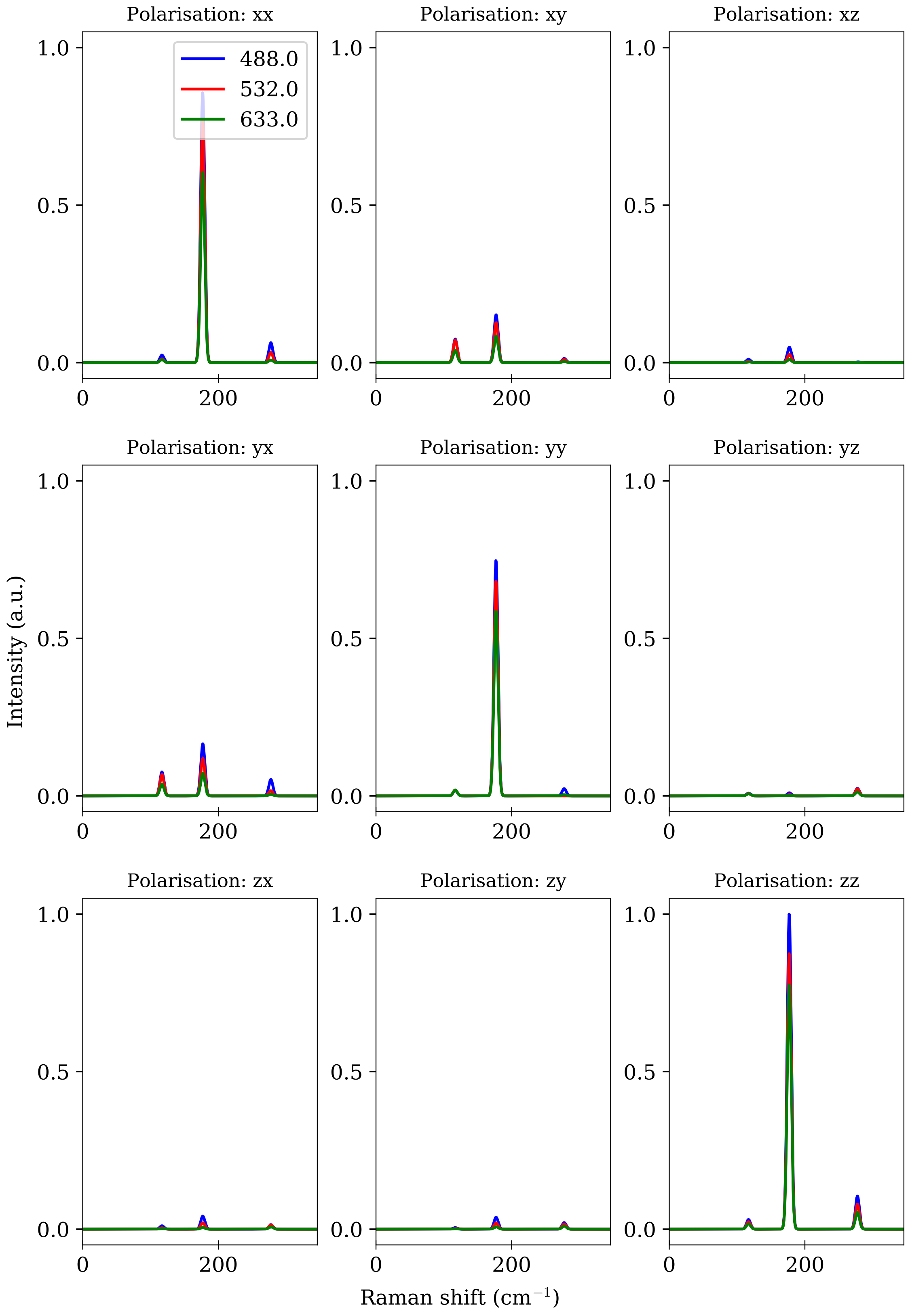

Supplementary Figure 376: Raman spectrum of NbSeTe with space group 156 and Wyckoff positions a-b-c-0.

NbSSe, 156, a-b-c-0

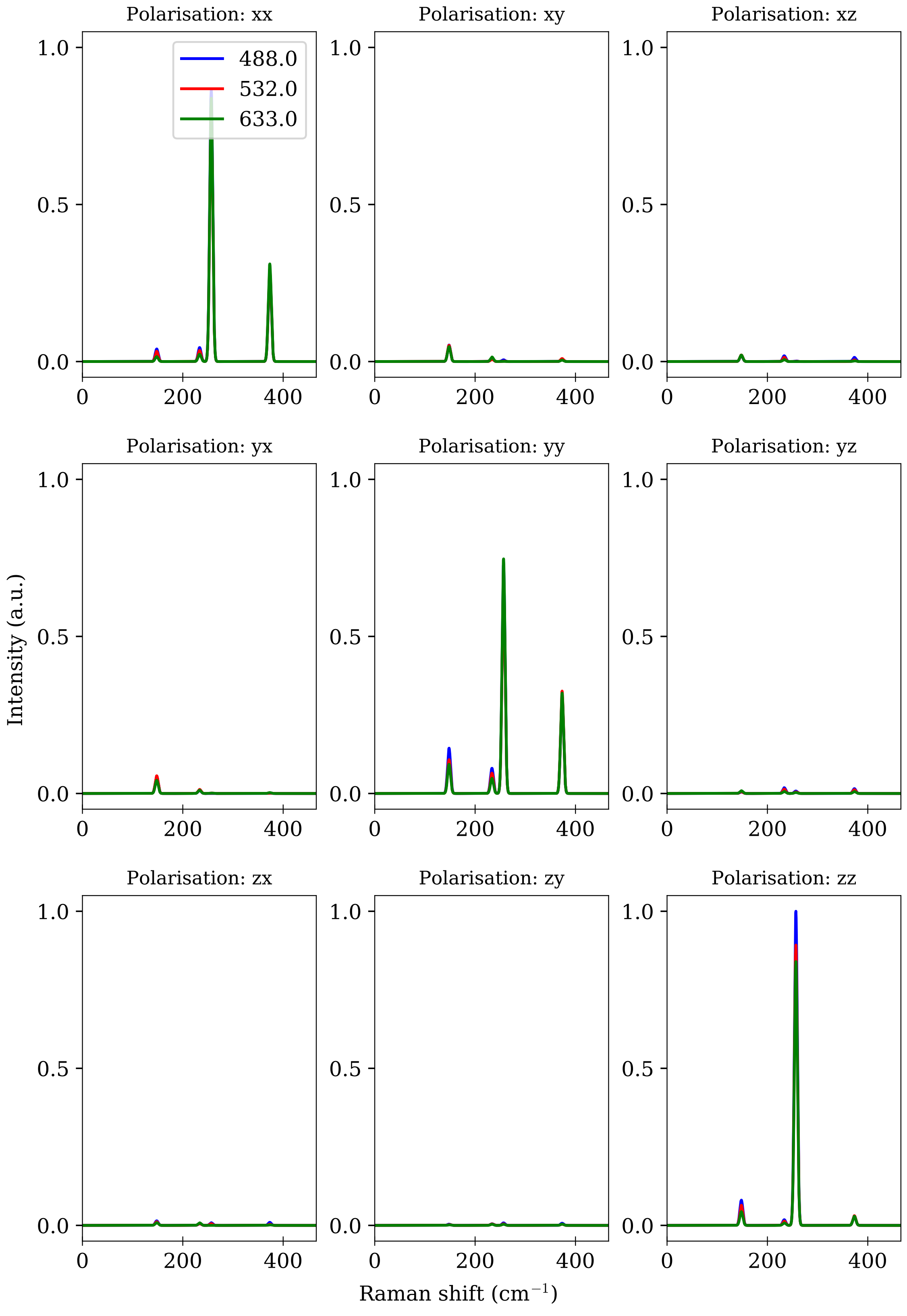

Supplementary Figure 377: Raman spectrum of NbSSe with space group 156 and Wyckoff positions a-b-c-0.

NbSTe, 156, a-b-c-0

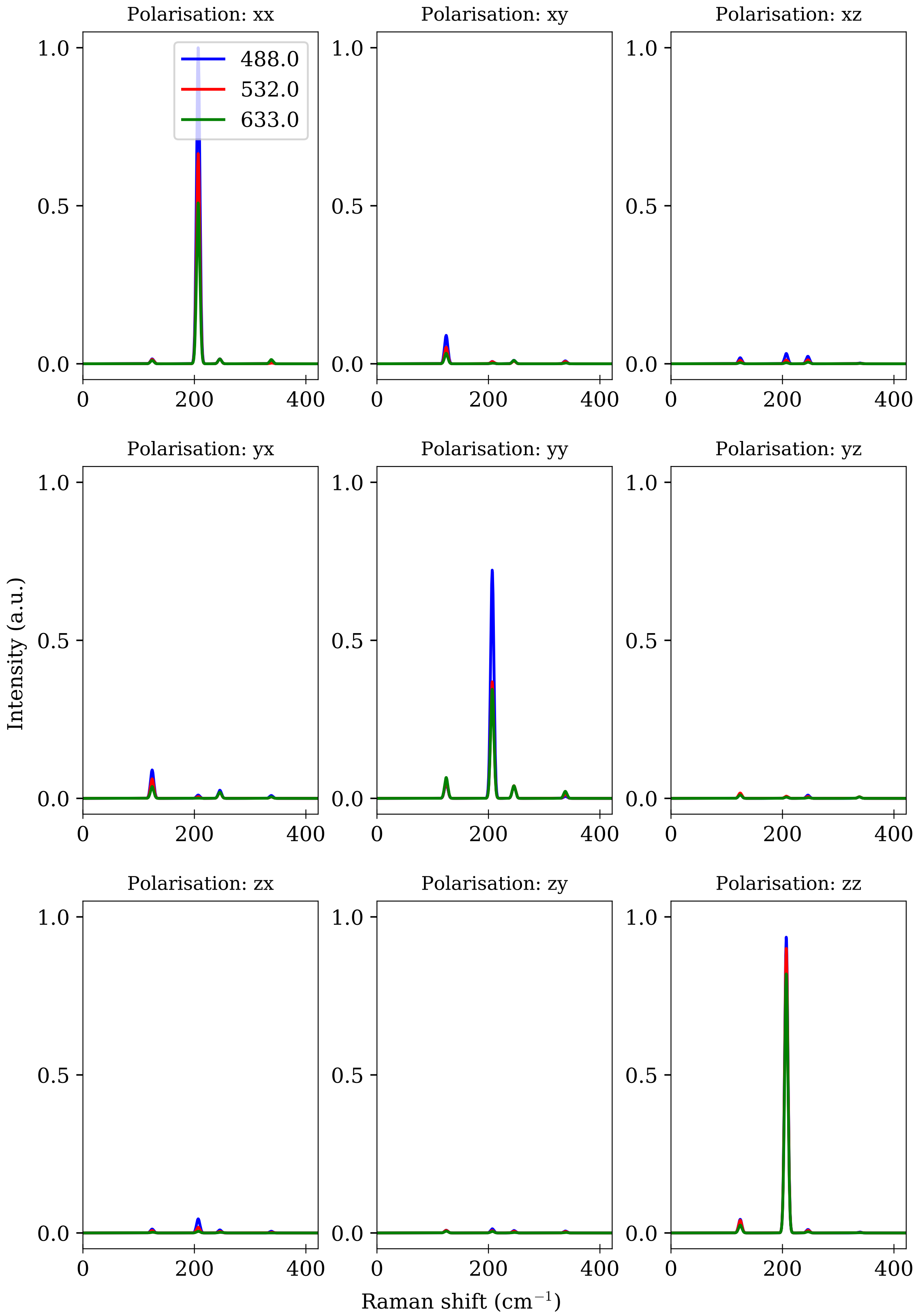

Supplementary Figure 378: Raman spectrum of NbSTe with space group 156 and Wyckoff positions a-b-c-0.

$NbTe_2$, 164, b-d-0

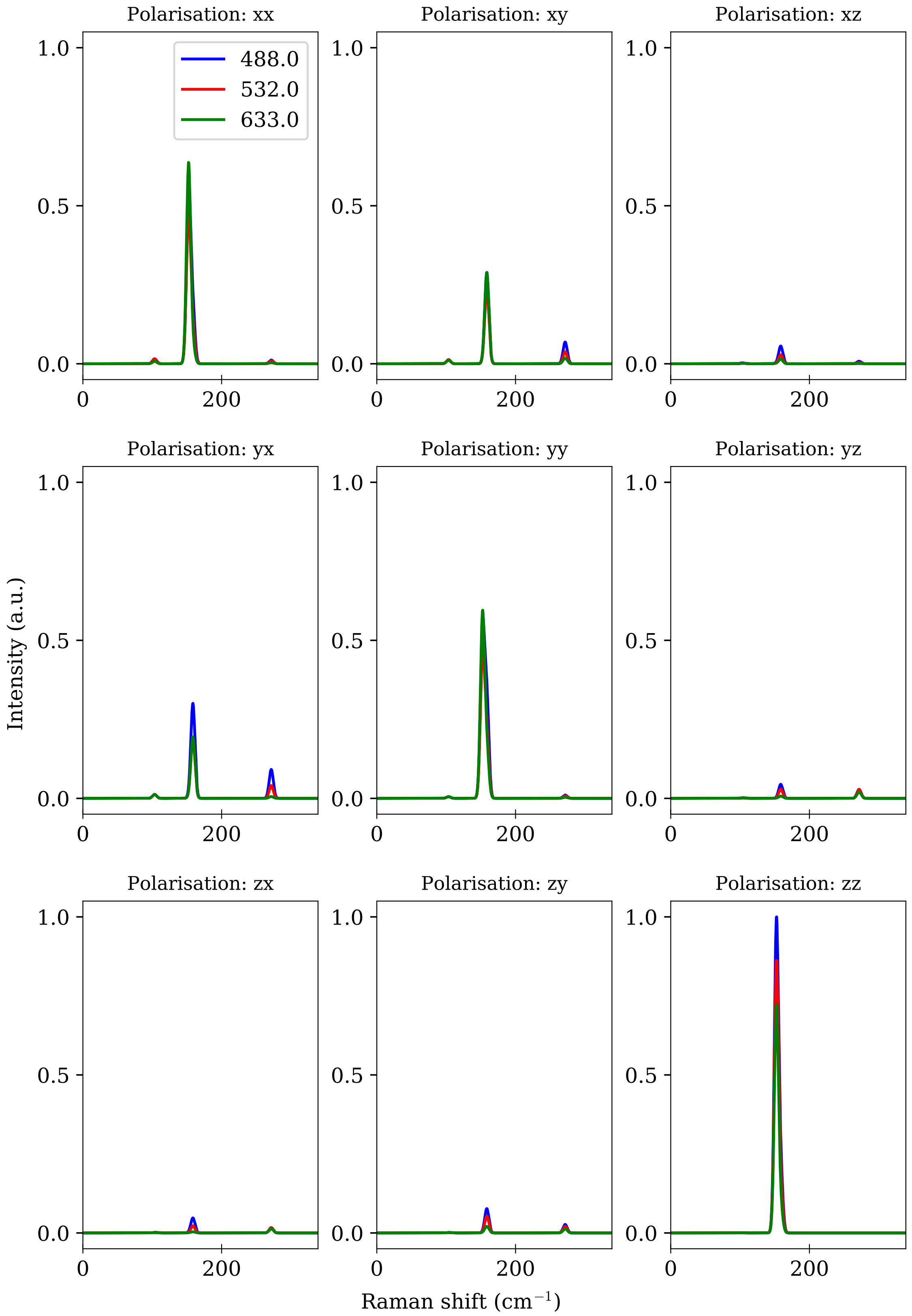

Supplementary Figure 379: Raman spectrum of $NbTe_2$ with space group 164 and Wyckoff positions b-d-0.

$Ni_2Br_2S_2$, 59, a-b-0

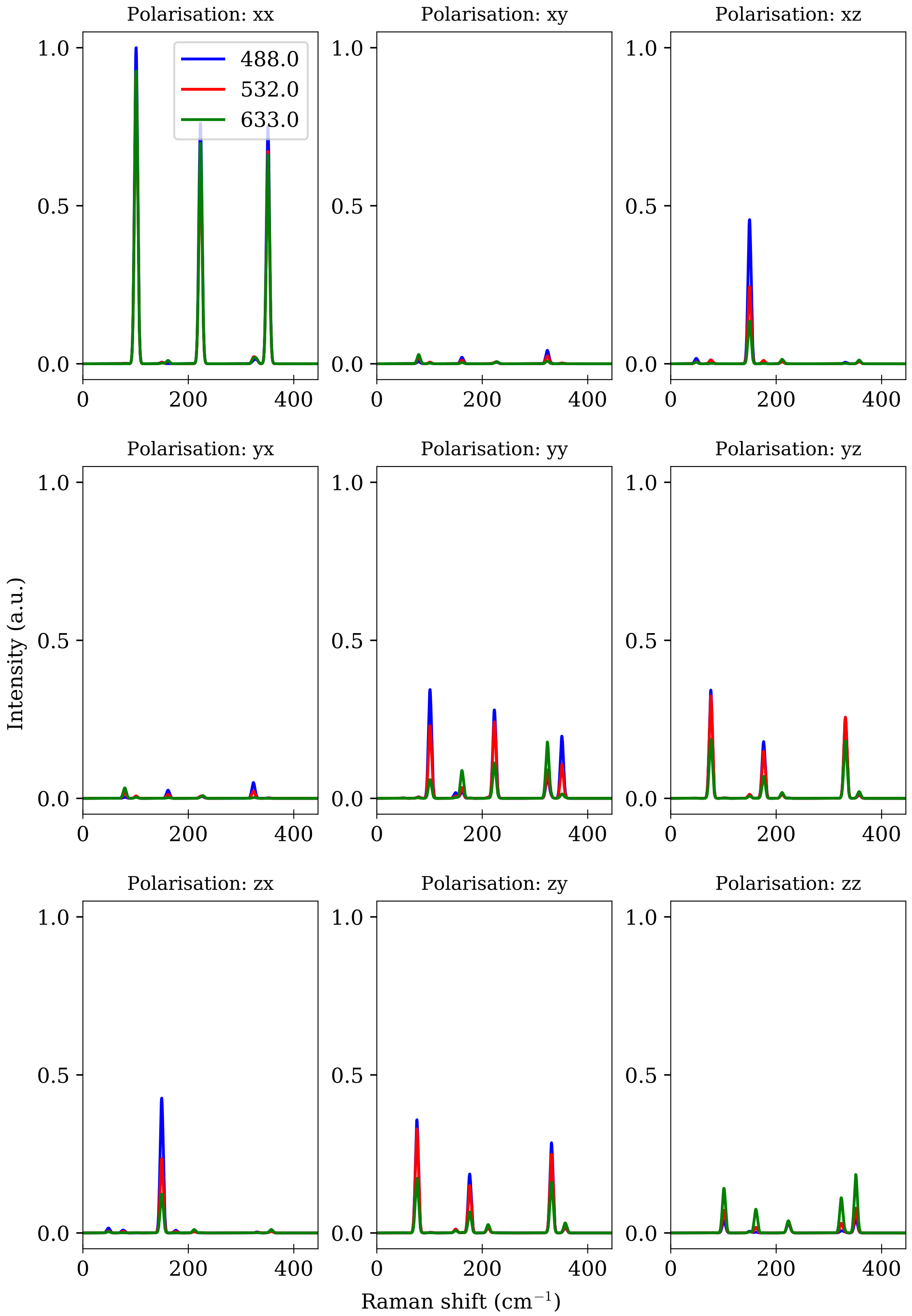

Supplementary Figure 380: Raman spectrum of $Ni_2Br_2S_2$ with space group 59 and Wyckoff positions a-b-0.

$Ni_2Br_2Se_2$, 59, a-b-0

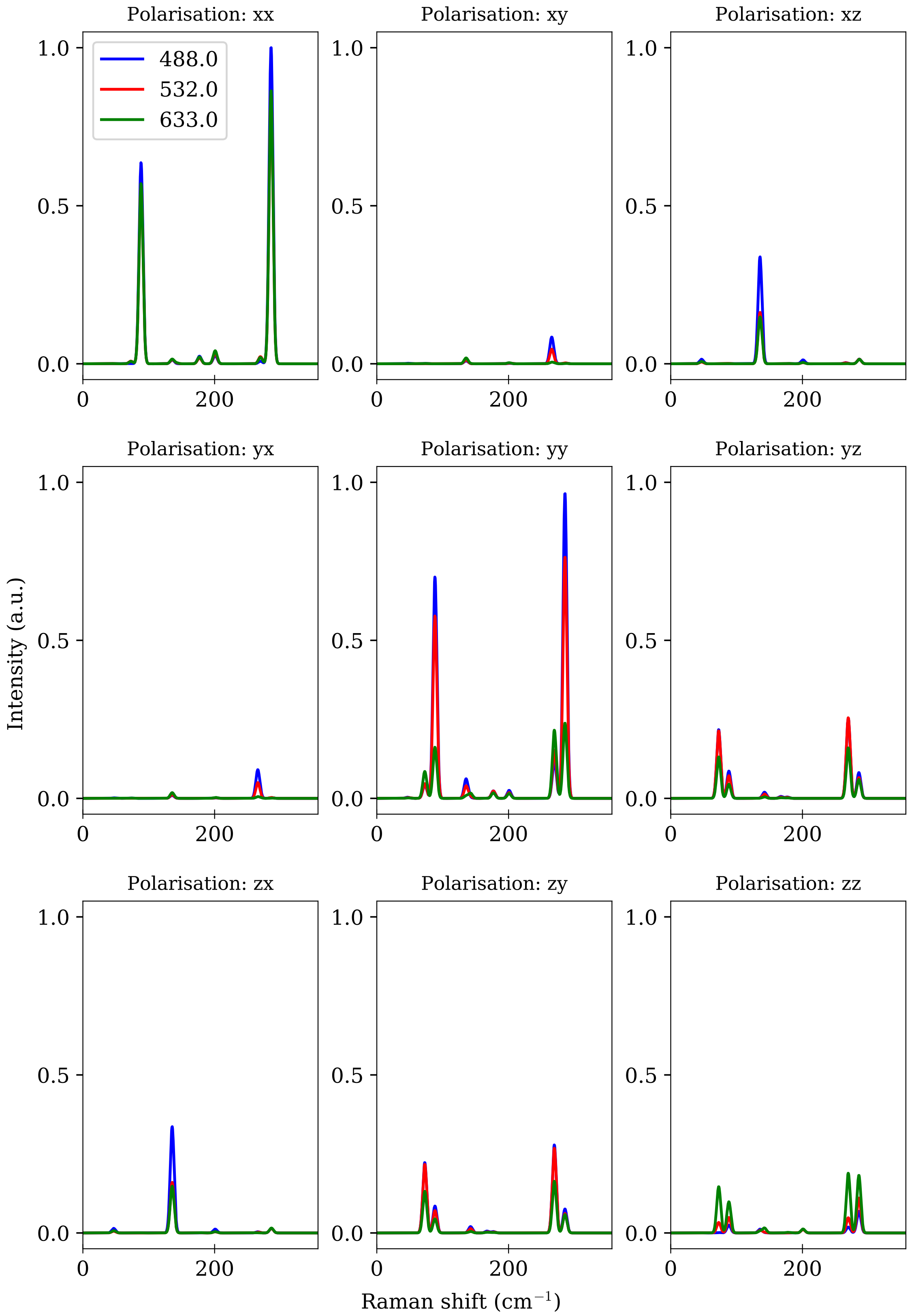

Supplementary Figure 381: Raman spectrum of $Ni_2Br_2Se_2$ with space group 59 and Wyckoff positions a-b-0.

$Ni_2Cl_2S_2$, 59, a-b-0

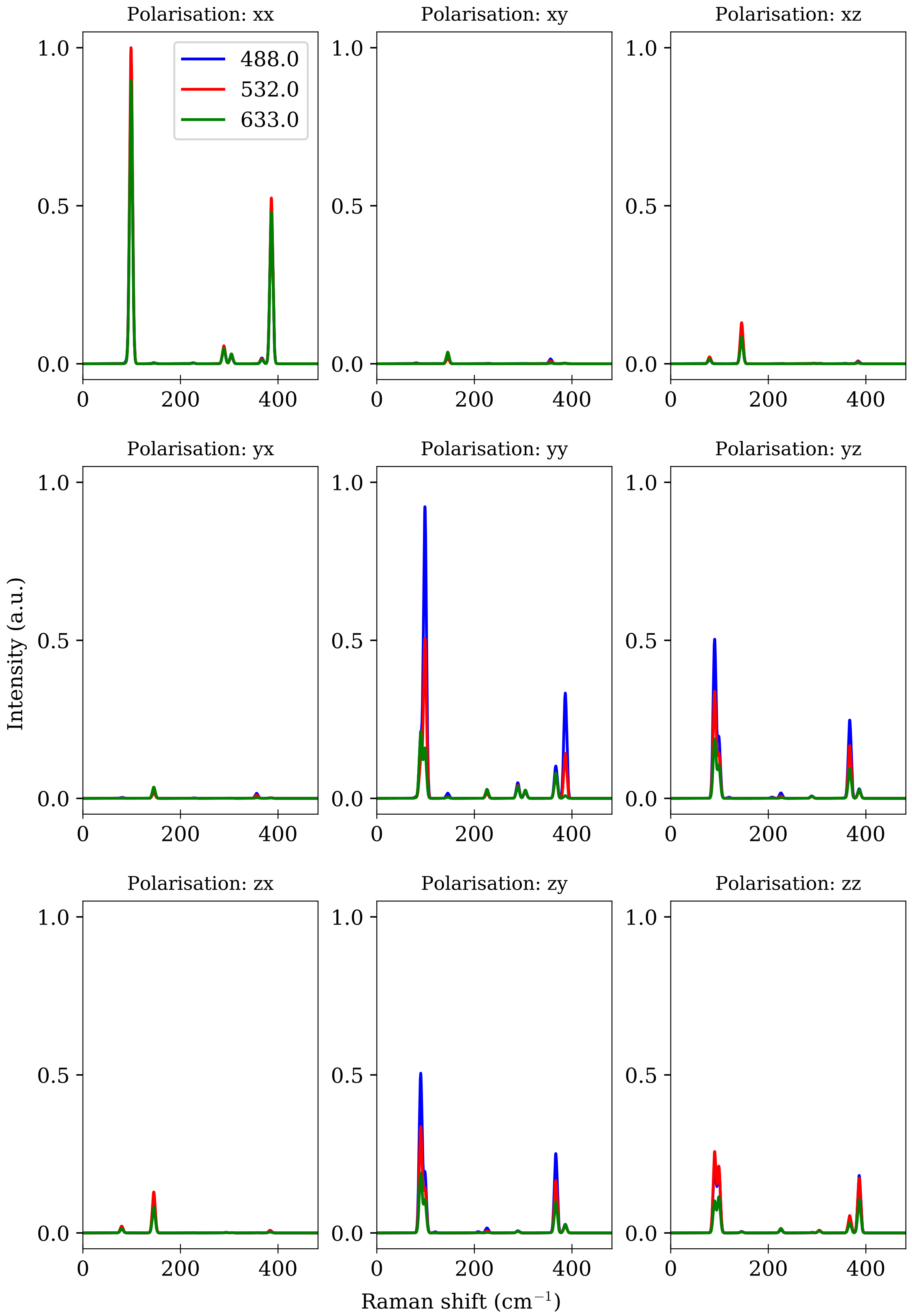

Supplementary Figure 382: Raman spectrum of $Ni_2Cl_2S_2$ with space group 59 and Wyckoff positions a-b-0.

Ni_2O_2, 10, f-g-m-0

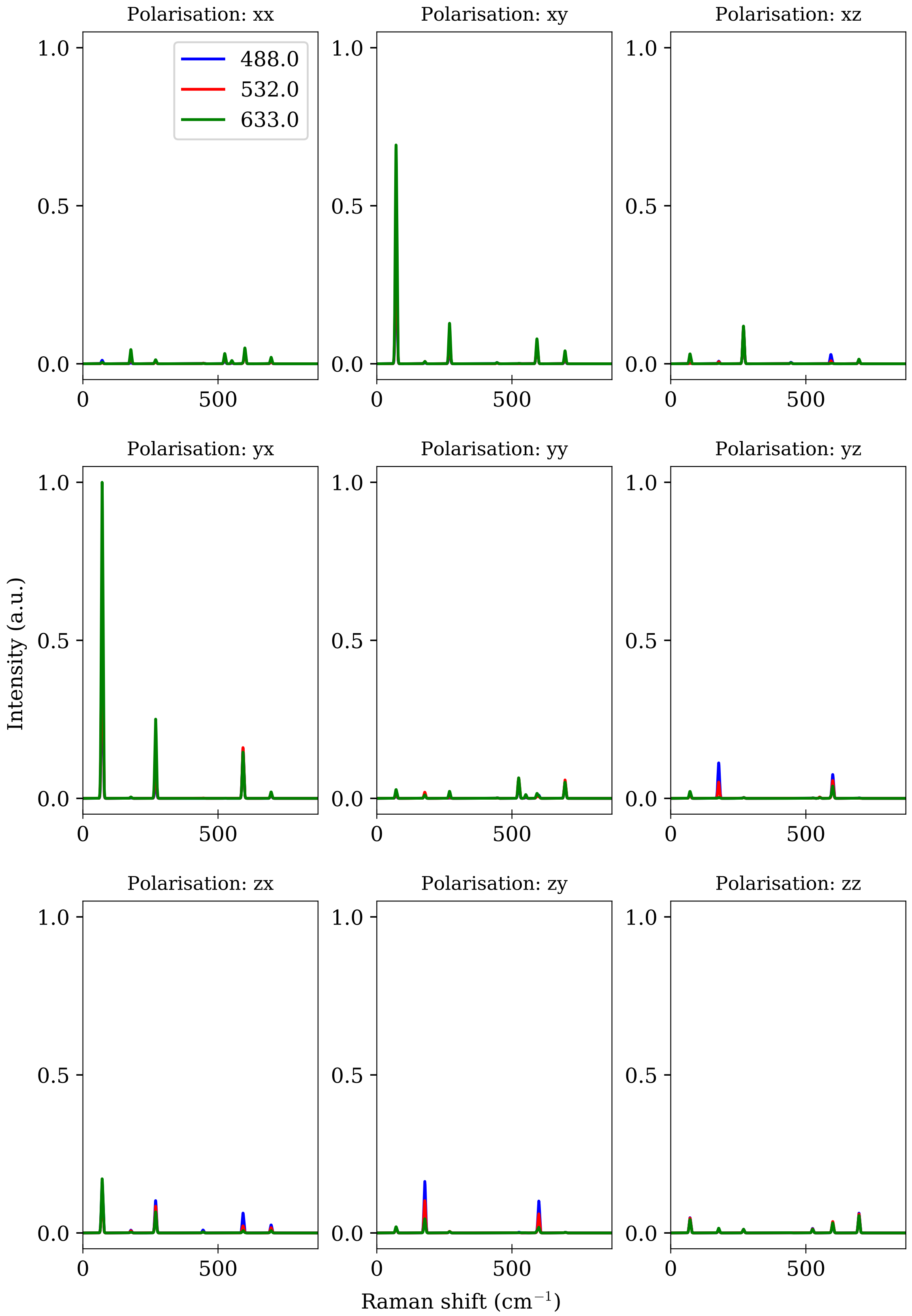

Supplementary Figure 383: Raman spectrum of Ni_2O_2 with space group 10 and Wyckoff positions f-g-m-0.

Ni_2S_2, 129, b-c-0

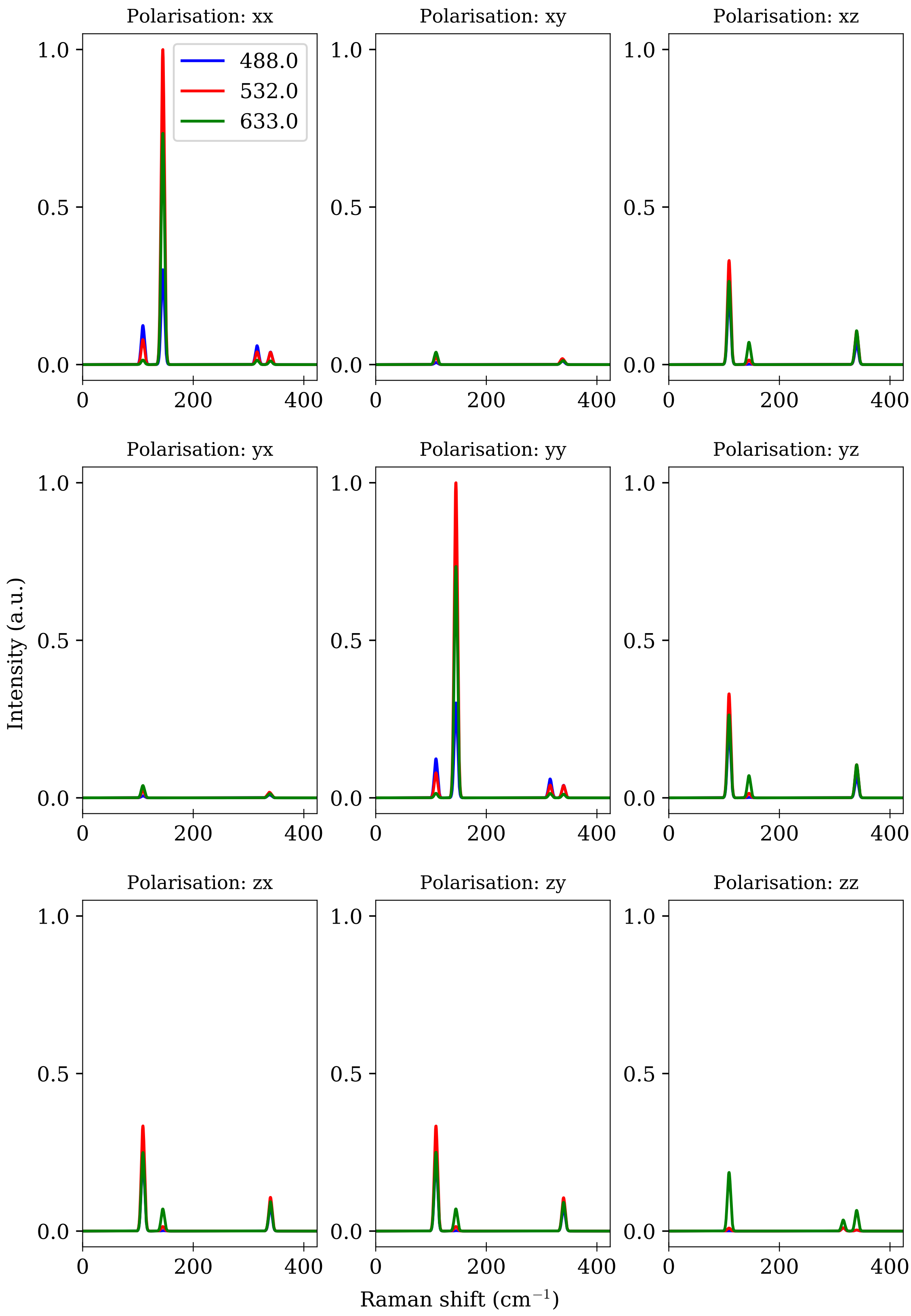

Supplementary Figure 384: Raman spectrum of Ni_2S_2 with space group 129 and Wyckoff positions b-c-0.

Ni_2S_2, 12, i-0

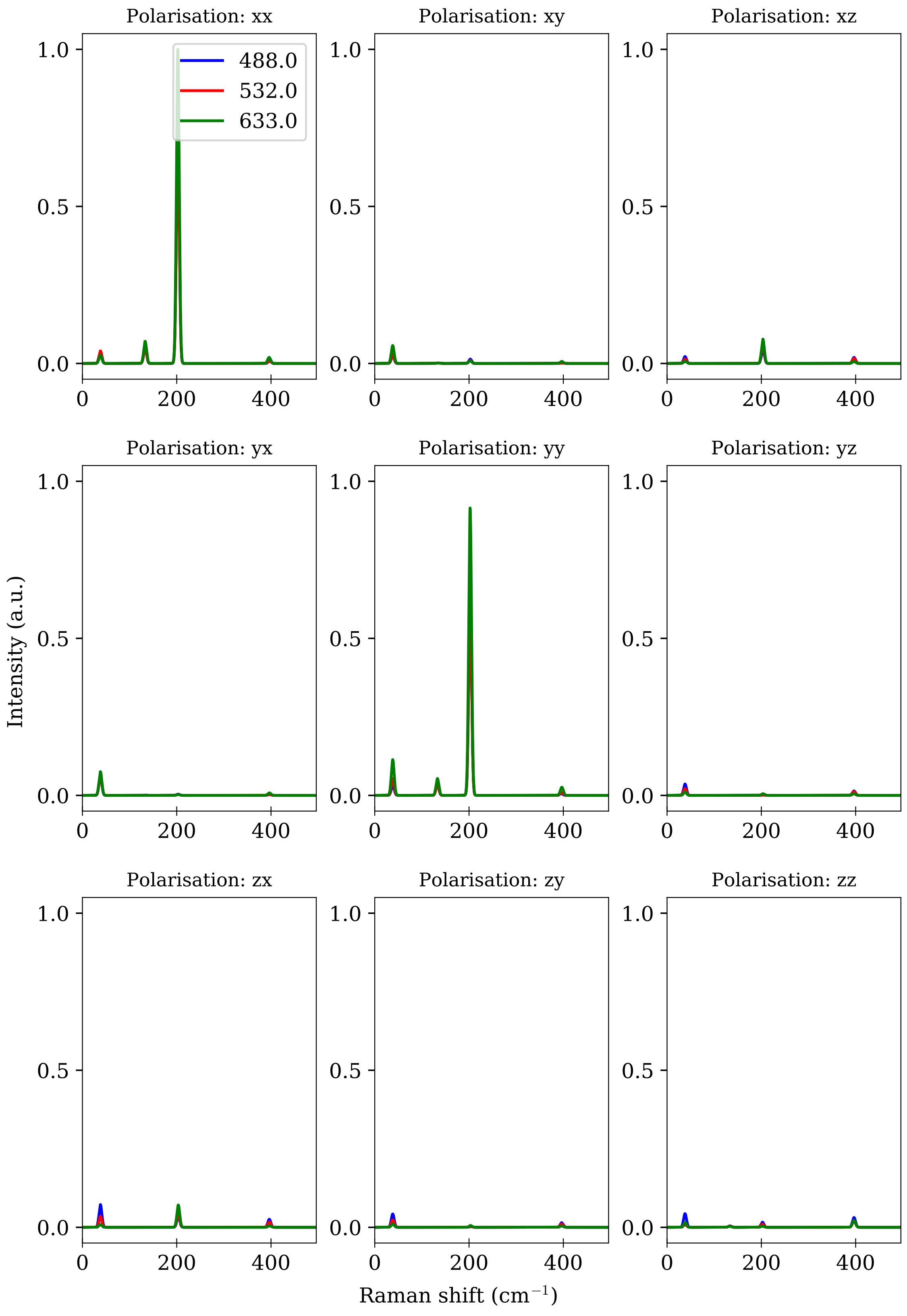

Supplementary Figure 385: Raman spectrum of Ni_2S_2 with space group 12 and Wyckoff positions i-0.

Ni_2S_2, 164, d-0

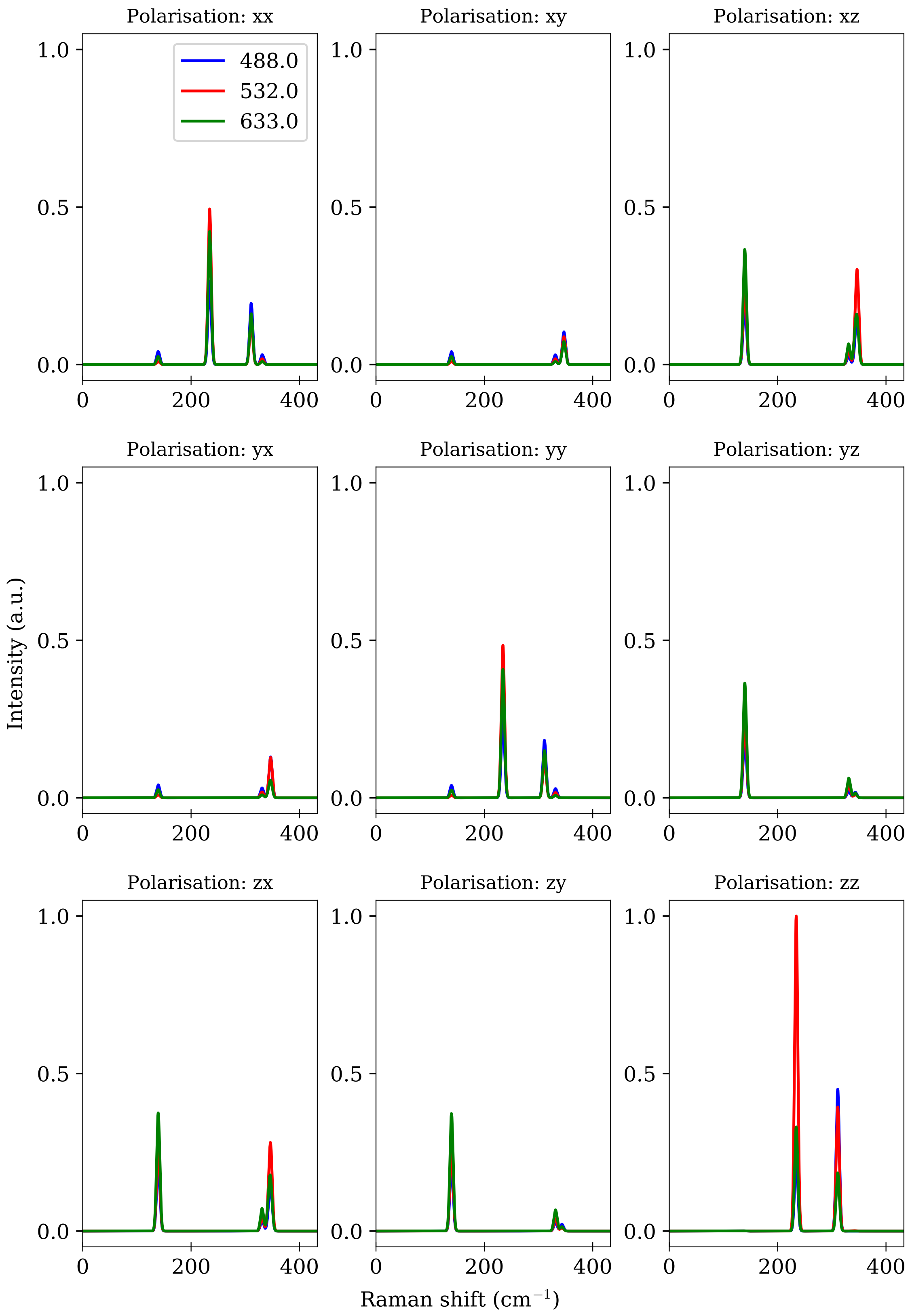

Supplementary Figure 386: Raman spectrum of Ni_2S_2 with space group 164 and Wyckoff positions d-0.

Ni_2S_2, 187, h-i-0

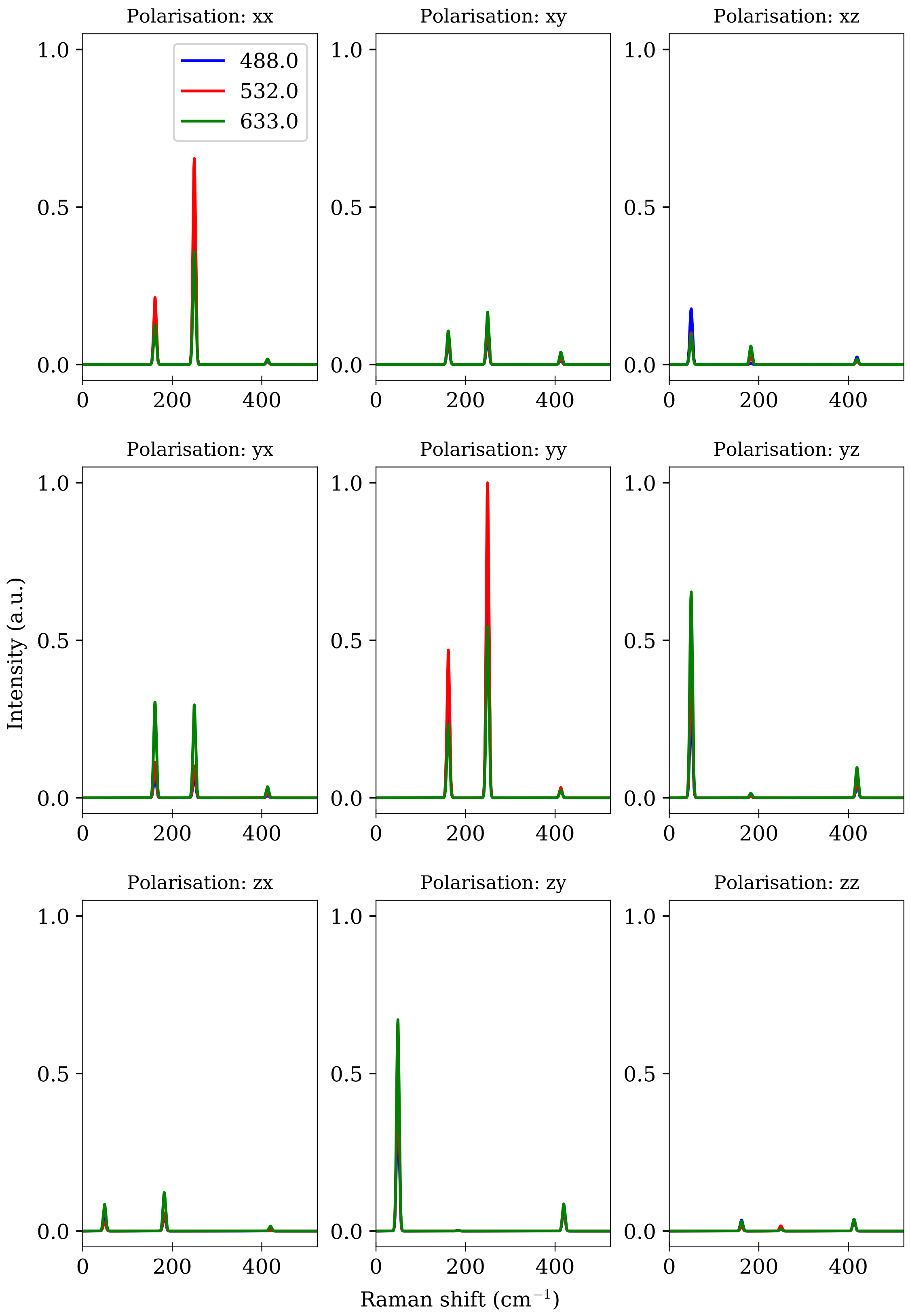

Supplementary Figure 387: Raman spectrum of Ni_2S_2 with space group 187 and Wyckoff positions h-i-0.

Ni_2S_4, 14, c-e-0

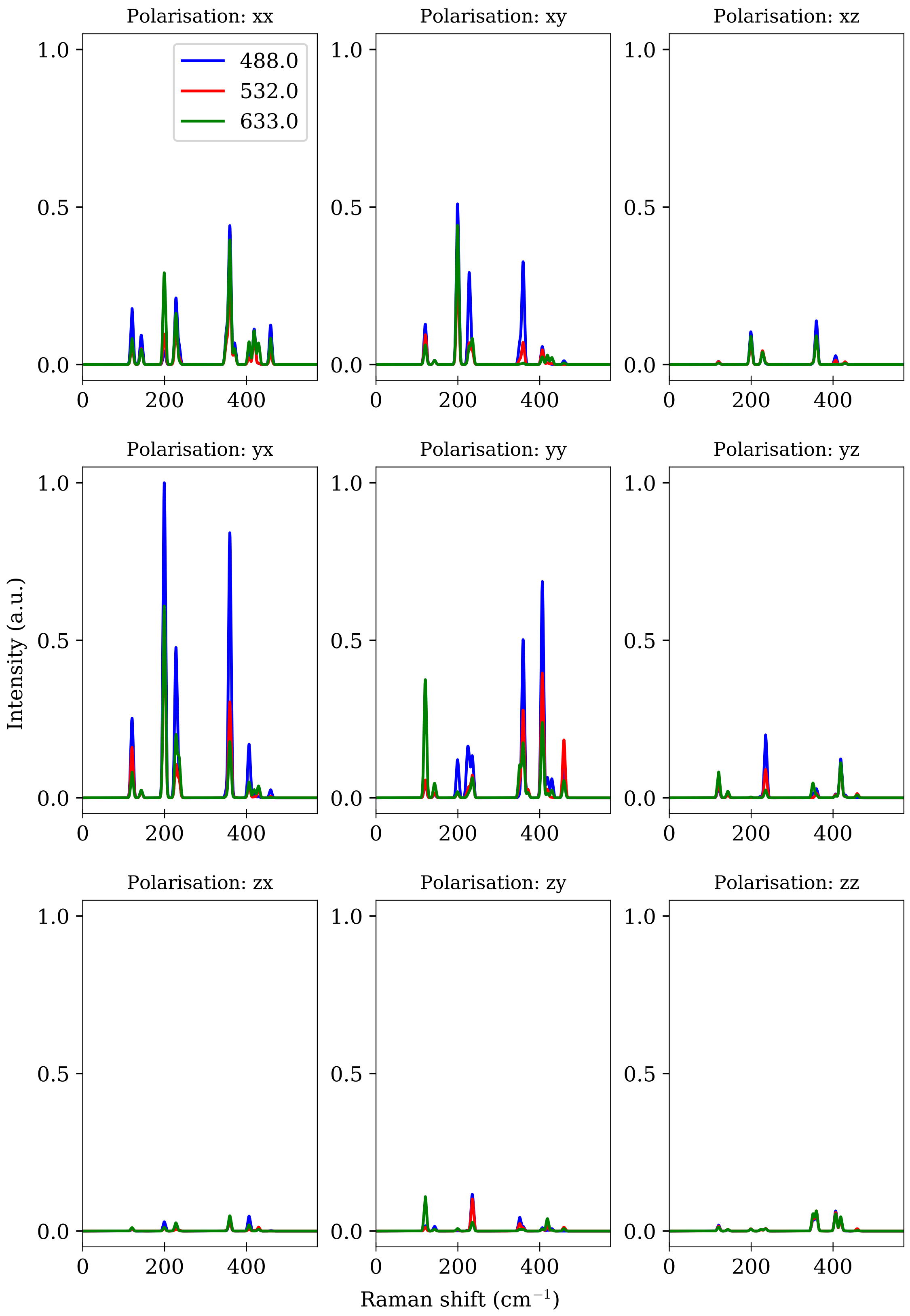

Supplementary Figure 388: Raman spectrum of Ni_2S_4 with space group 14 and Wyckoff positions c-e-0.

Ni_2Se_2, 164, d-0

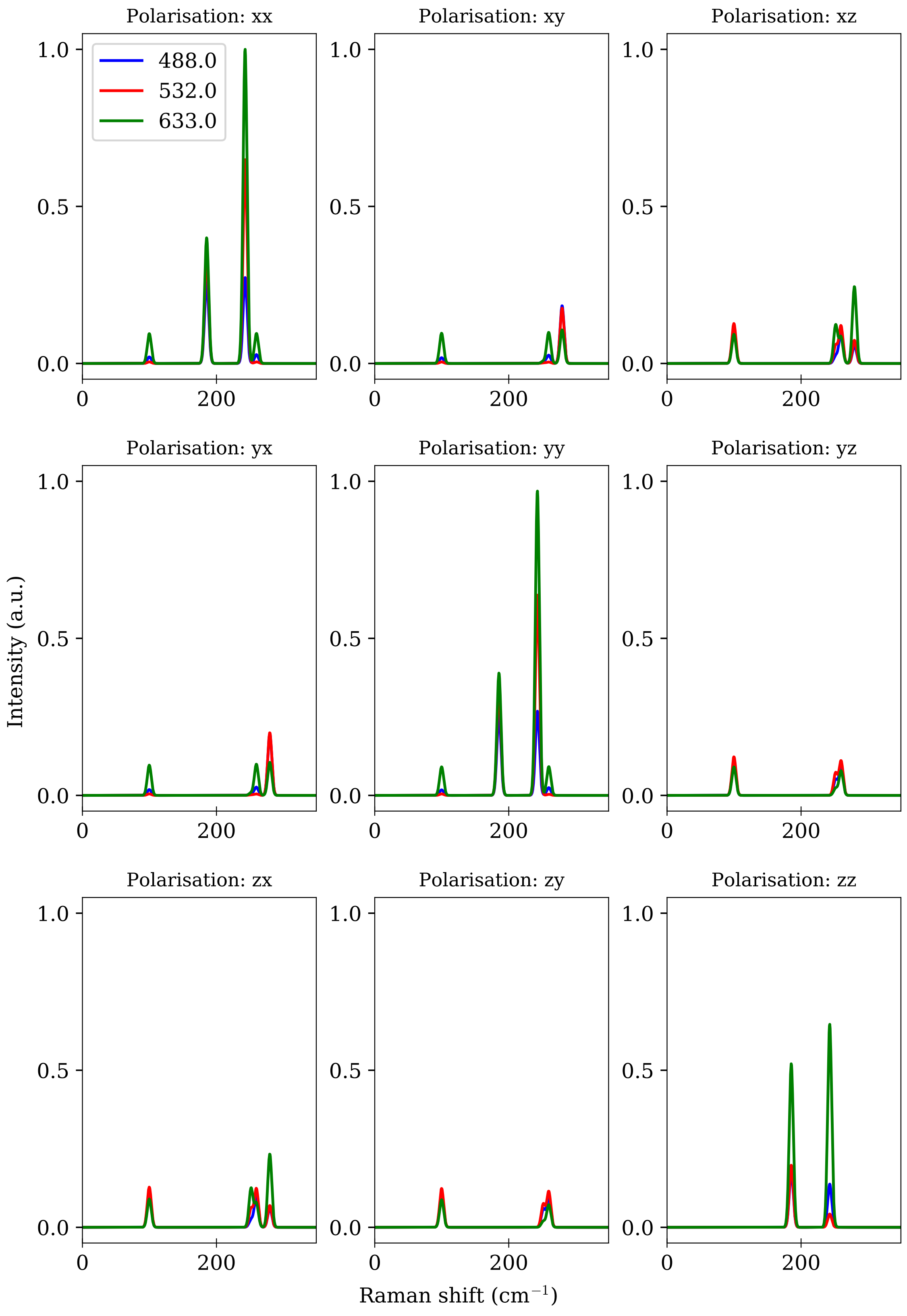

Supplementary Figure 389: Raman spectrum of Ni_2Se_2 with space group 164 and Wyckoff positions d-0.

Ni_2Se_2, 187, h-i-0

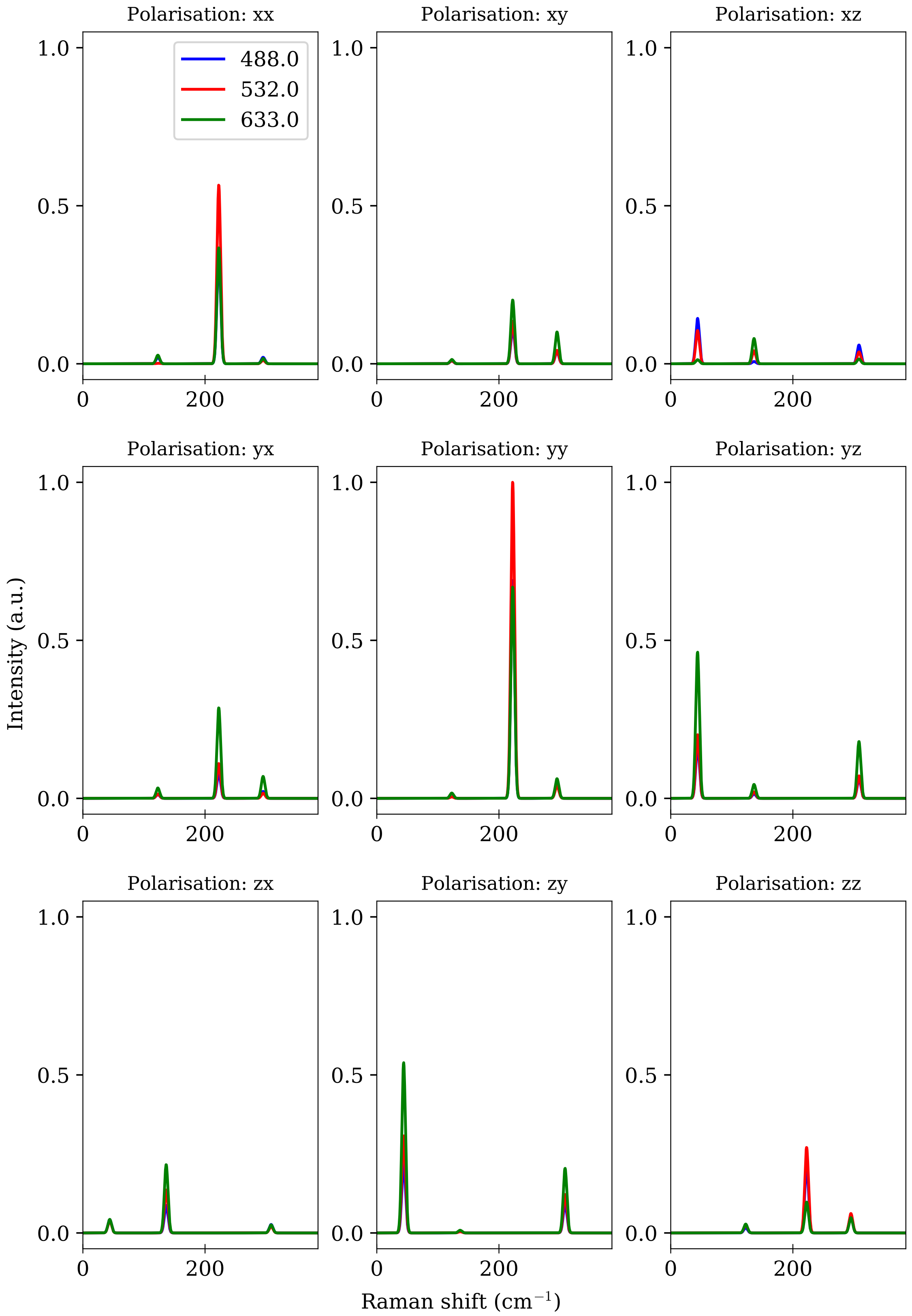

Supplementary Figure 390: Raman spectrum of Ni_2Se_2 with space group 187 and Wyckoff positions h-i-0.

Ni_2Se_4, 14, c-e-0

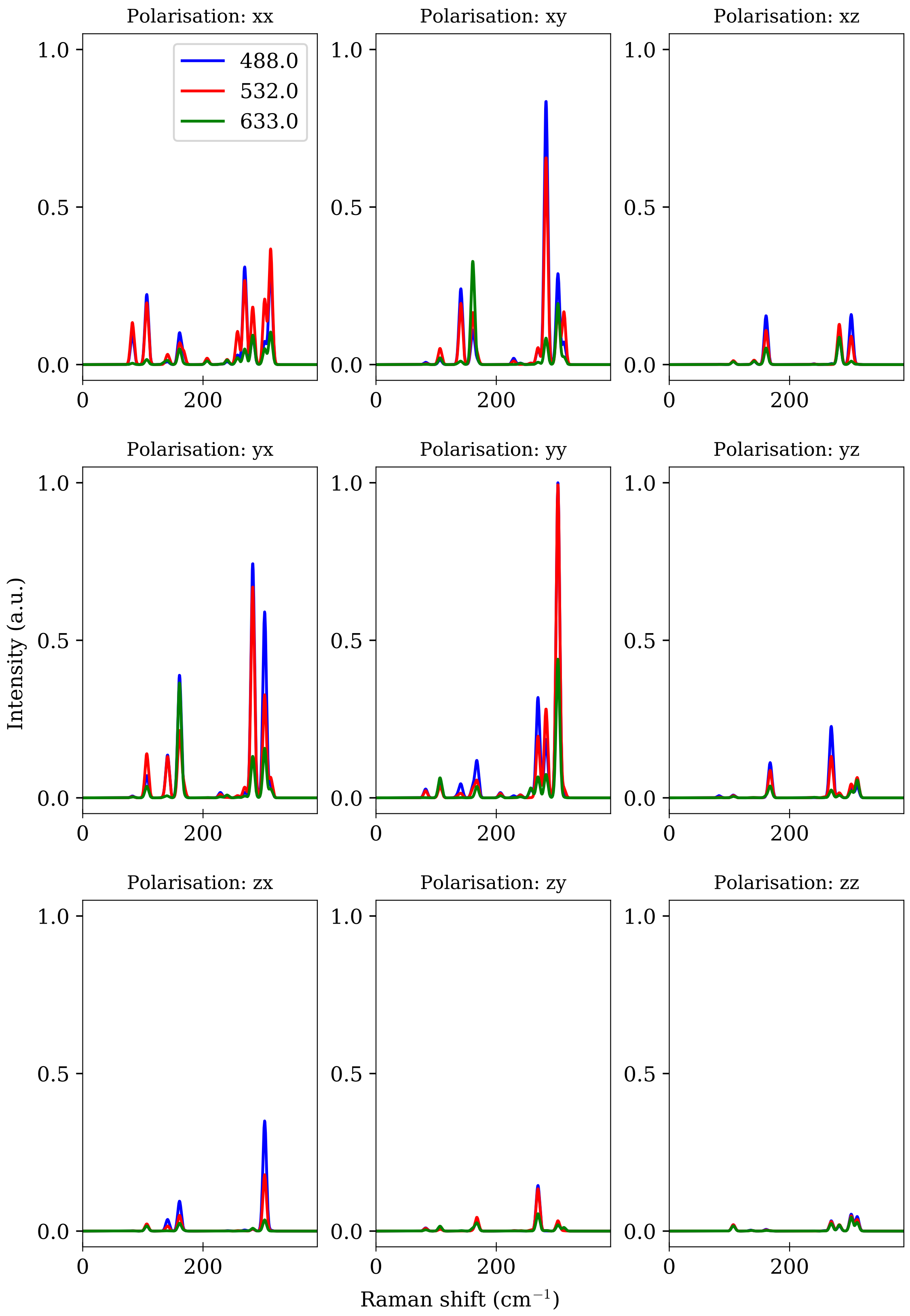

Supplementary Figure 391: Raman spectrum of Ni_2Se_4 with space group 14 and Wyckoff positions c-e-0.

Ni_2Te_2, 164, d-0

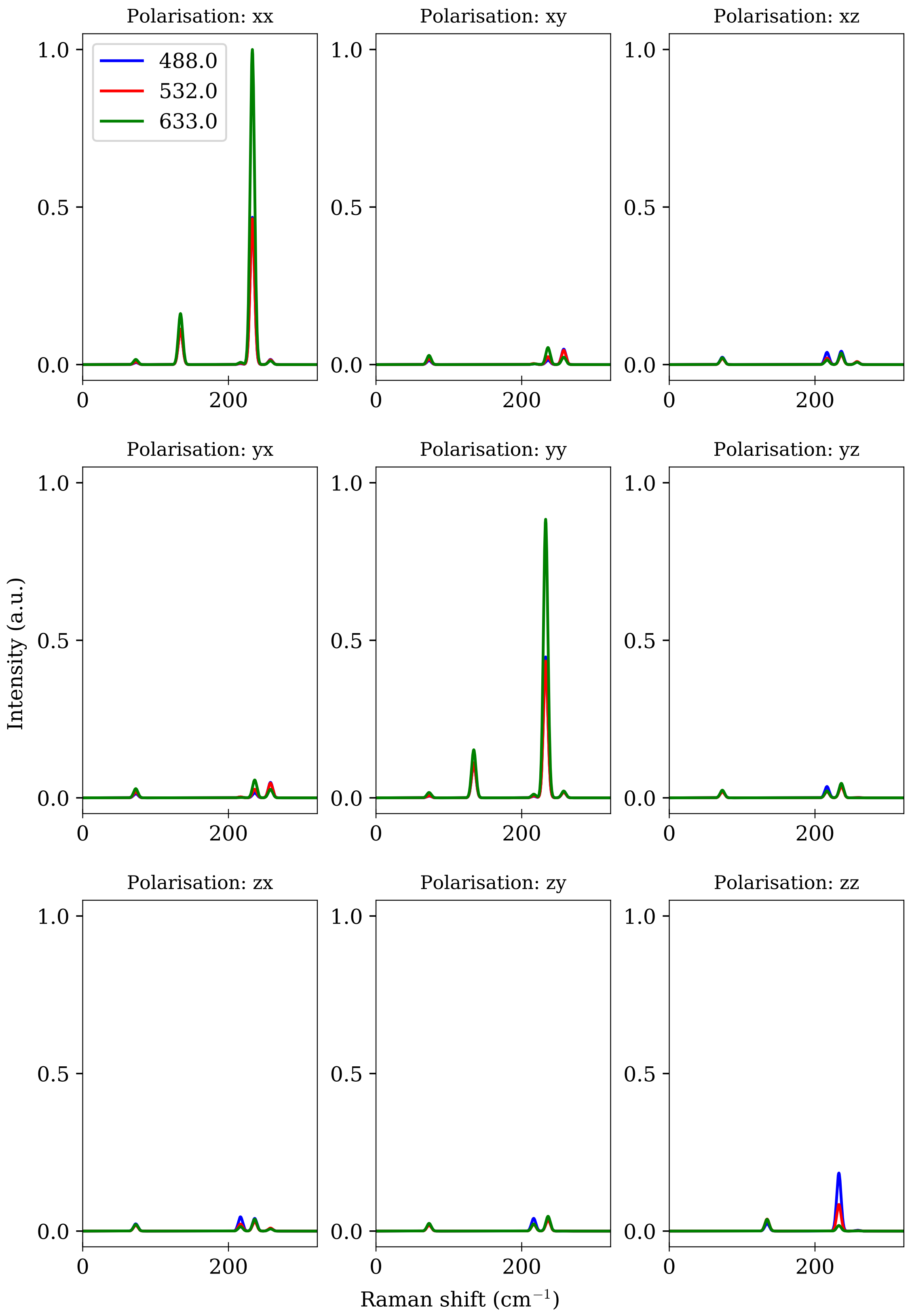

Supplementary Figure 392: Raman spectrum of Ni_2Te_2 with space group 164 and Wyckoff positions d-0.

Ni_2Te_2, 187, h-i-0

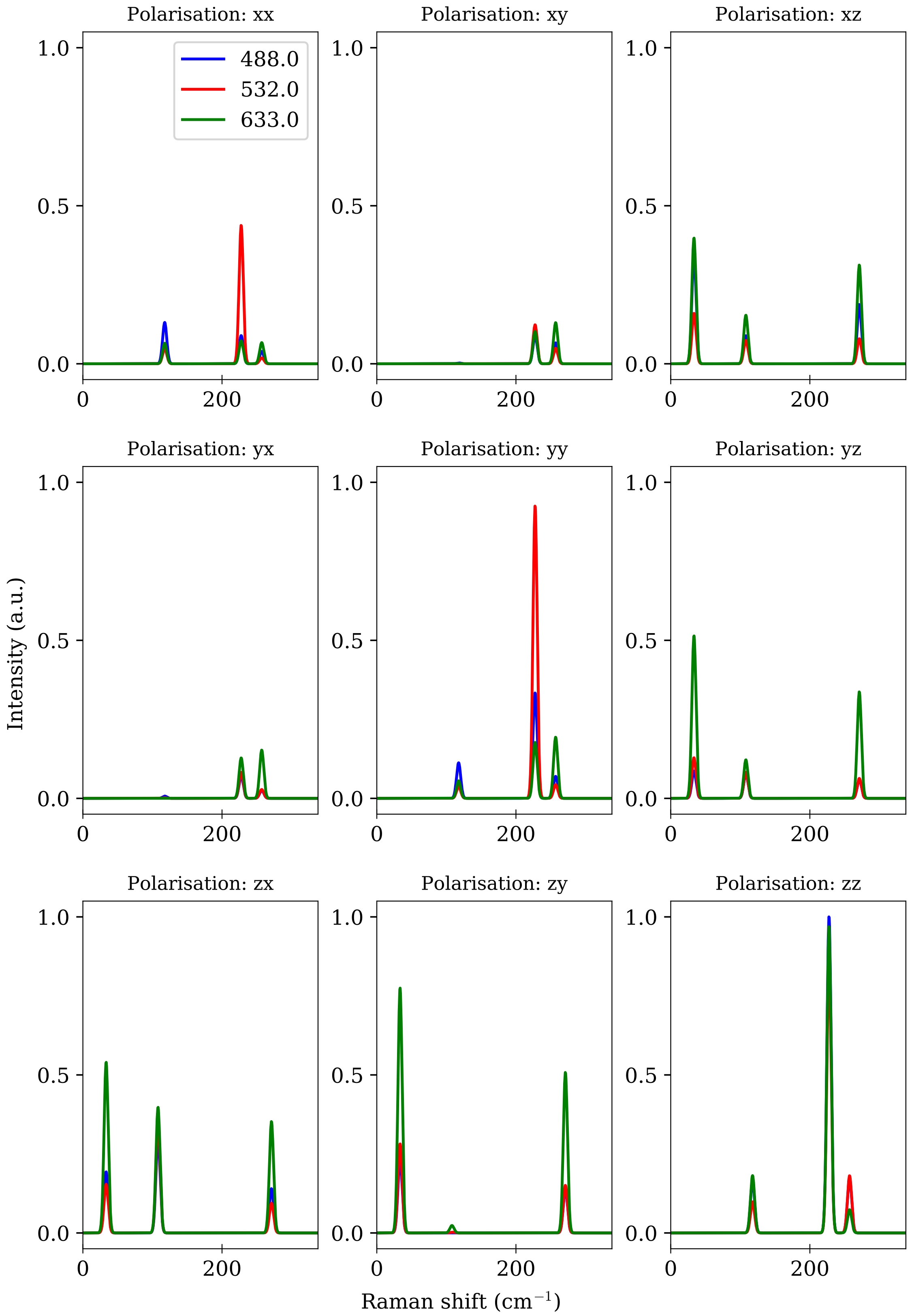

Supplementary Figure 393: Raman spectrum of Ni_2Te_2 with space group 187 and Wyckoff positions h-i-0.

Ni_2Te_4, 14, a-e-0

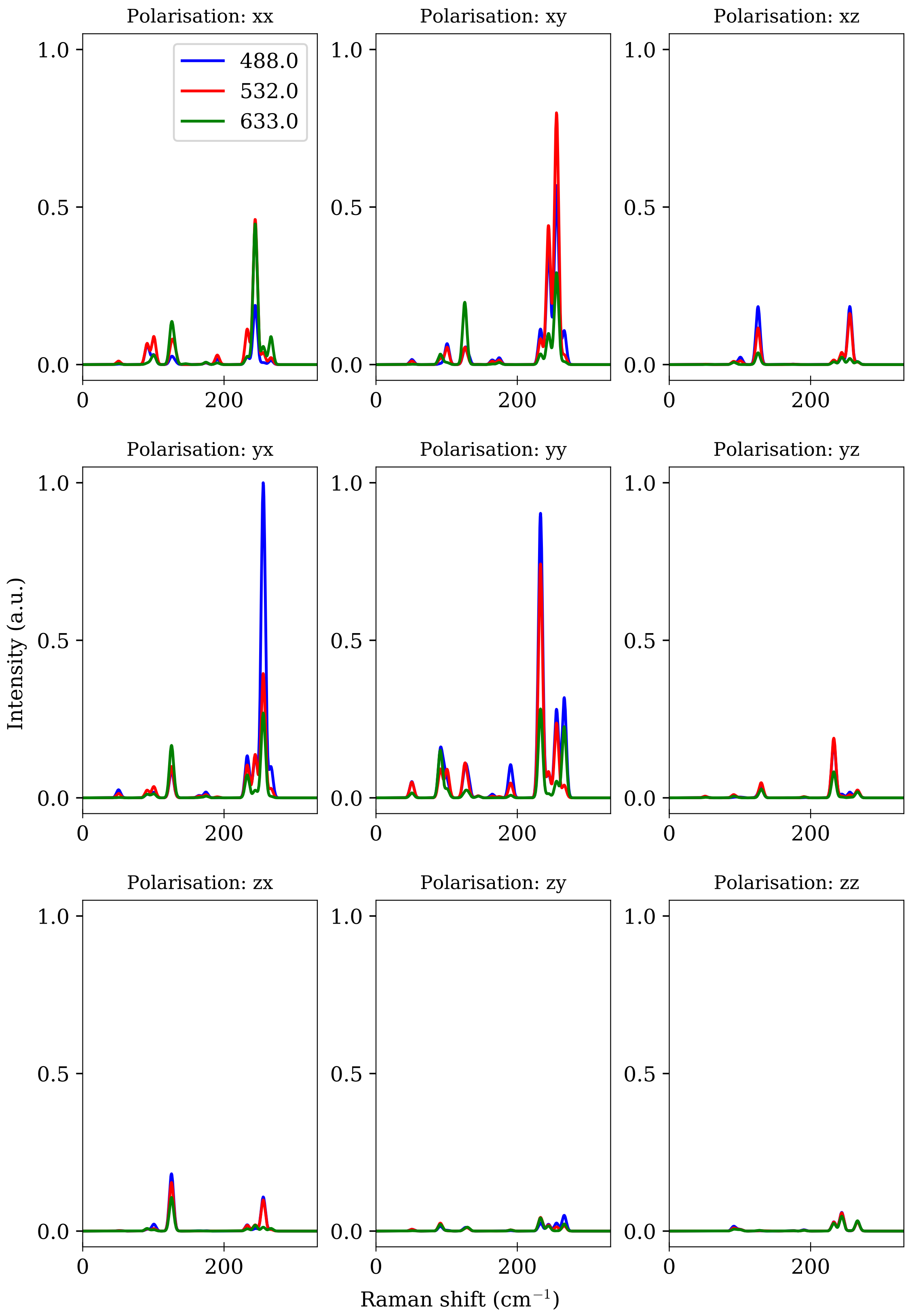

Supplementary Figure 394: Raman spectrum of Ni_2Te_4 with space group 14 and Wyckoff positions a-e-0.

$NiCl_2$, 115, a-g-0

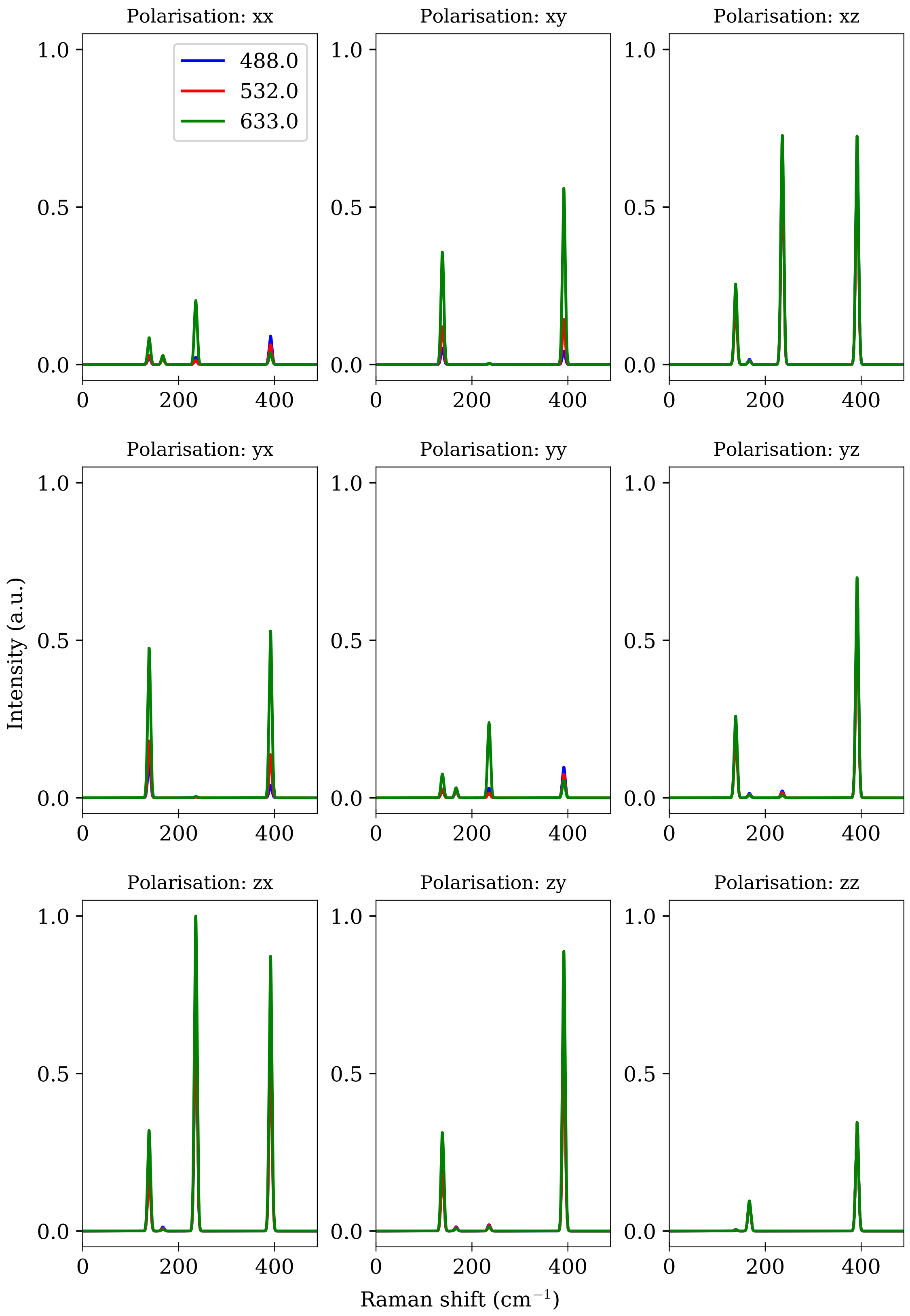

Supplementary Figure 395: Raman spectrum of $NiCl_2$ with space group 115 and Wyckoff positions a-g-0.

NiH_2, 187, b-i-0

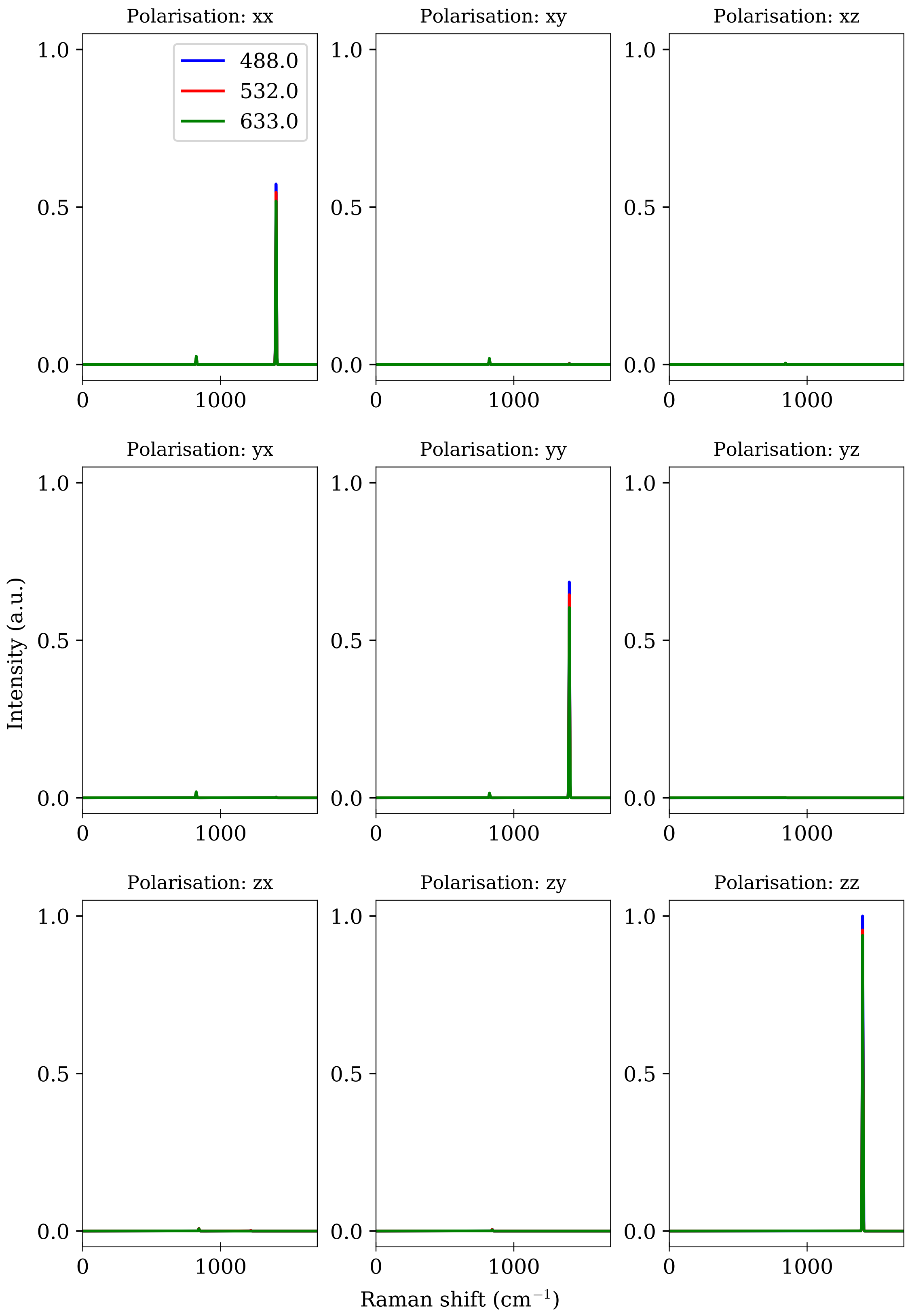

Supplementary Figure 396: Raman spectrum of NiH_2 with space group 187 and Wyckoff positions b-i-0.

NiO_2, 164, b-d-0

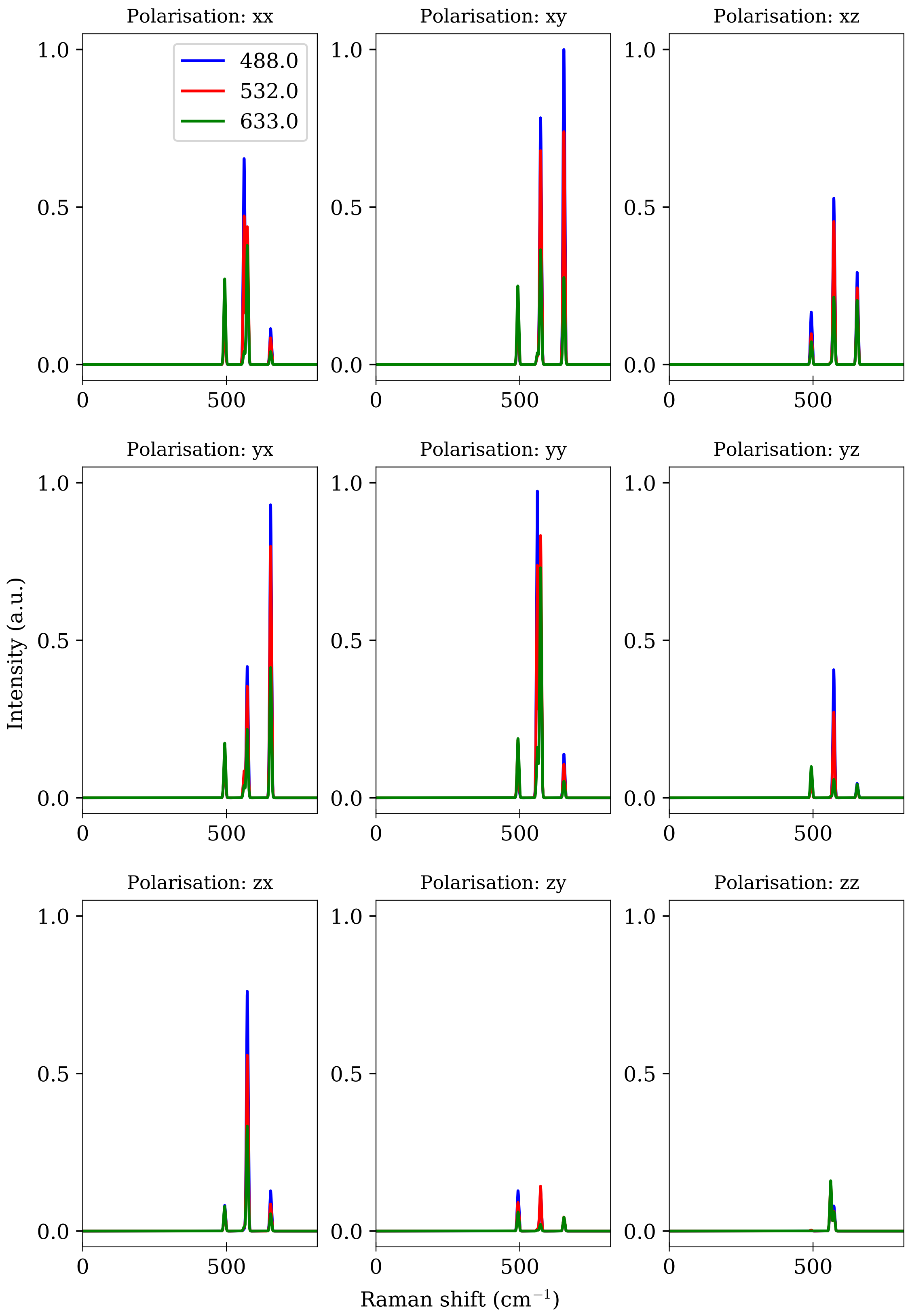

Supplementary Figure 397: Raman spectrum of NiO_2 with space group 164 and Wyckoff positions b-d-0.

NiS_2, 164, b-d-0

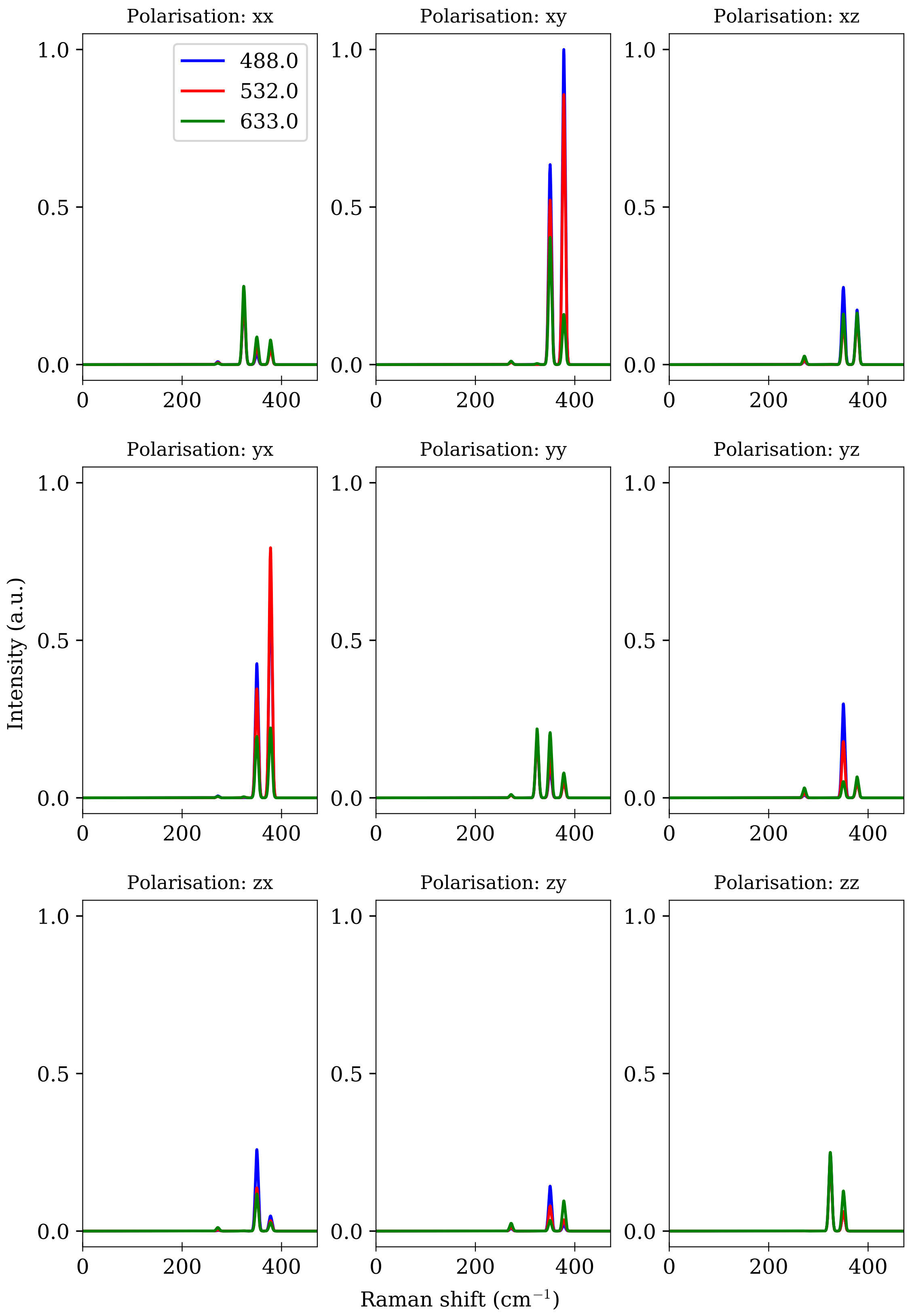

Supplementary Figure 398: Raman spectrum of NiS_2 with space group 164 and Wyckoff positions b-d-0.

NiS_2, 187, b-i-0

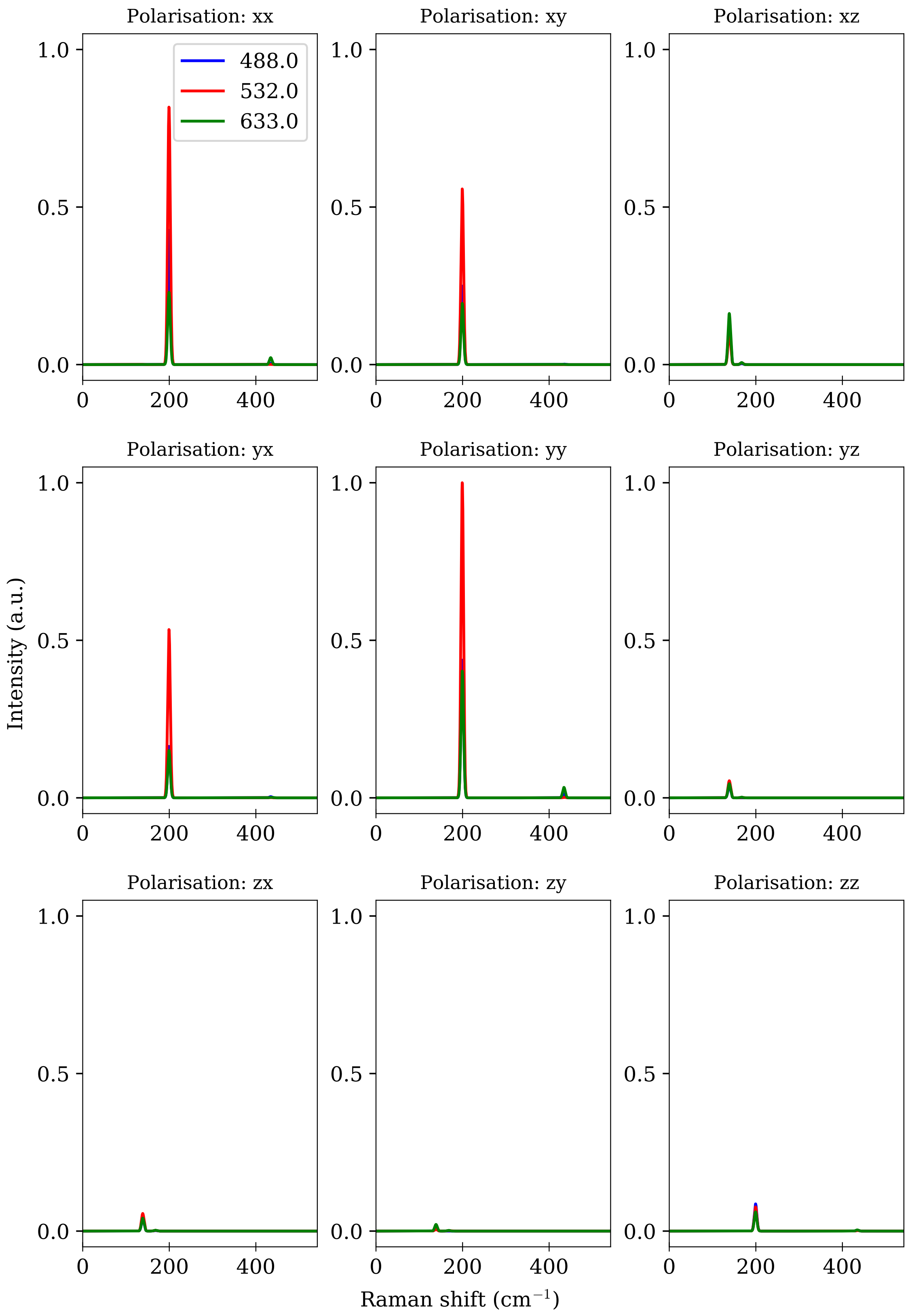

Supplementary Figure 399: Raman spectrum of NiS_2 with space group 187 and Wyckoff positions b-i-0.

$NiSe_2$, 164, b-d-0

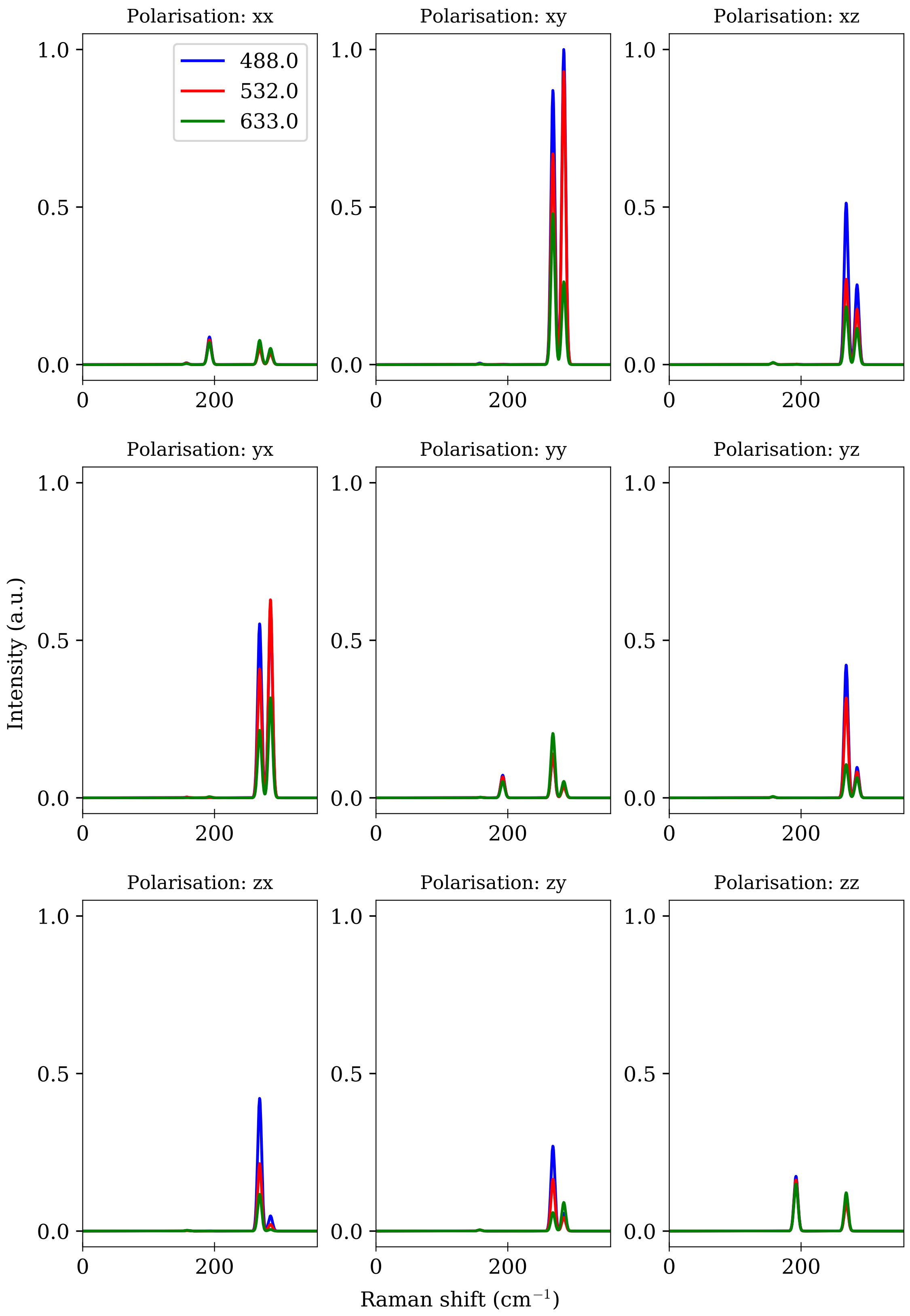

Supplementary Figure 400: Raman spectrum of $NiSe_2$ with space group 164 and Wyckoff positions b-d-0.

$NiSe_2$, 187, b-i-0

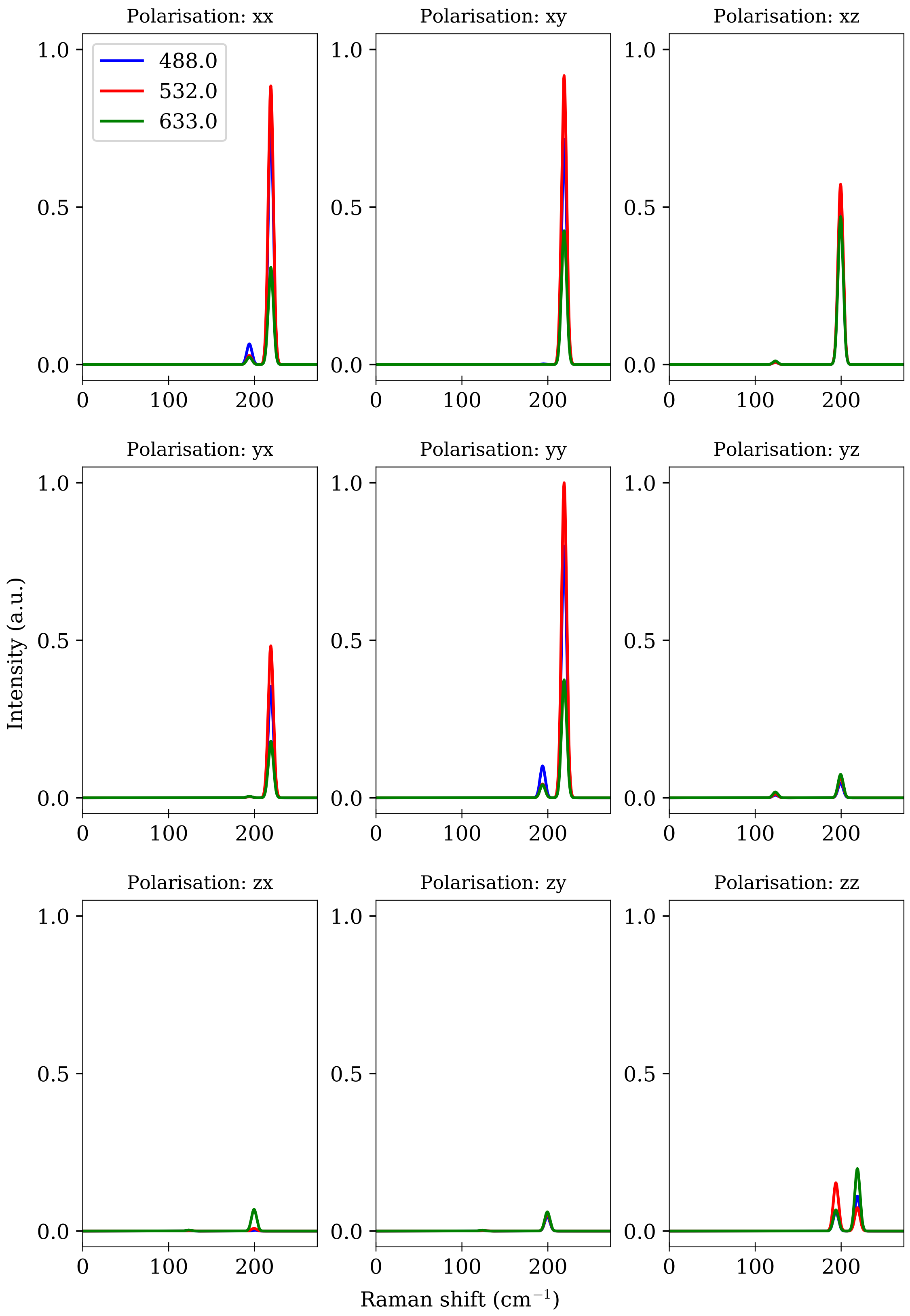

Supplementary Figure 401: Raman spectrum of $NiSe_2$ with space group 187 and Wyckoff positions b-i-0.

$NiTe_2$, 164, b-d-0

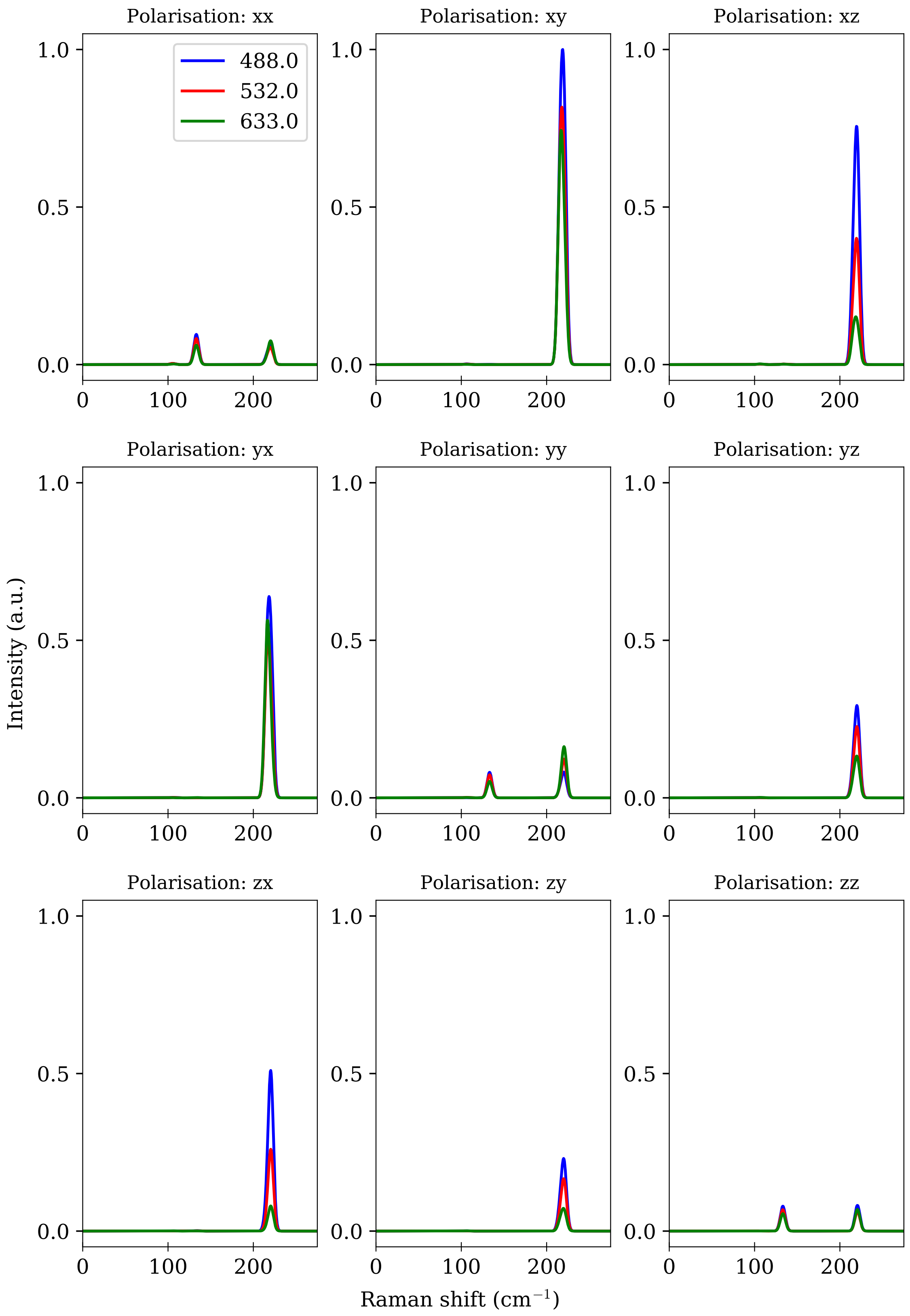

Supplementary Figure 402: Raman spectrum of $NiTe_2$ with space group 164 and Wyckoff positions b-d-0.

$NiTe_2$, 187, b-i-0

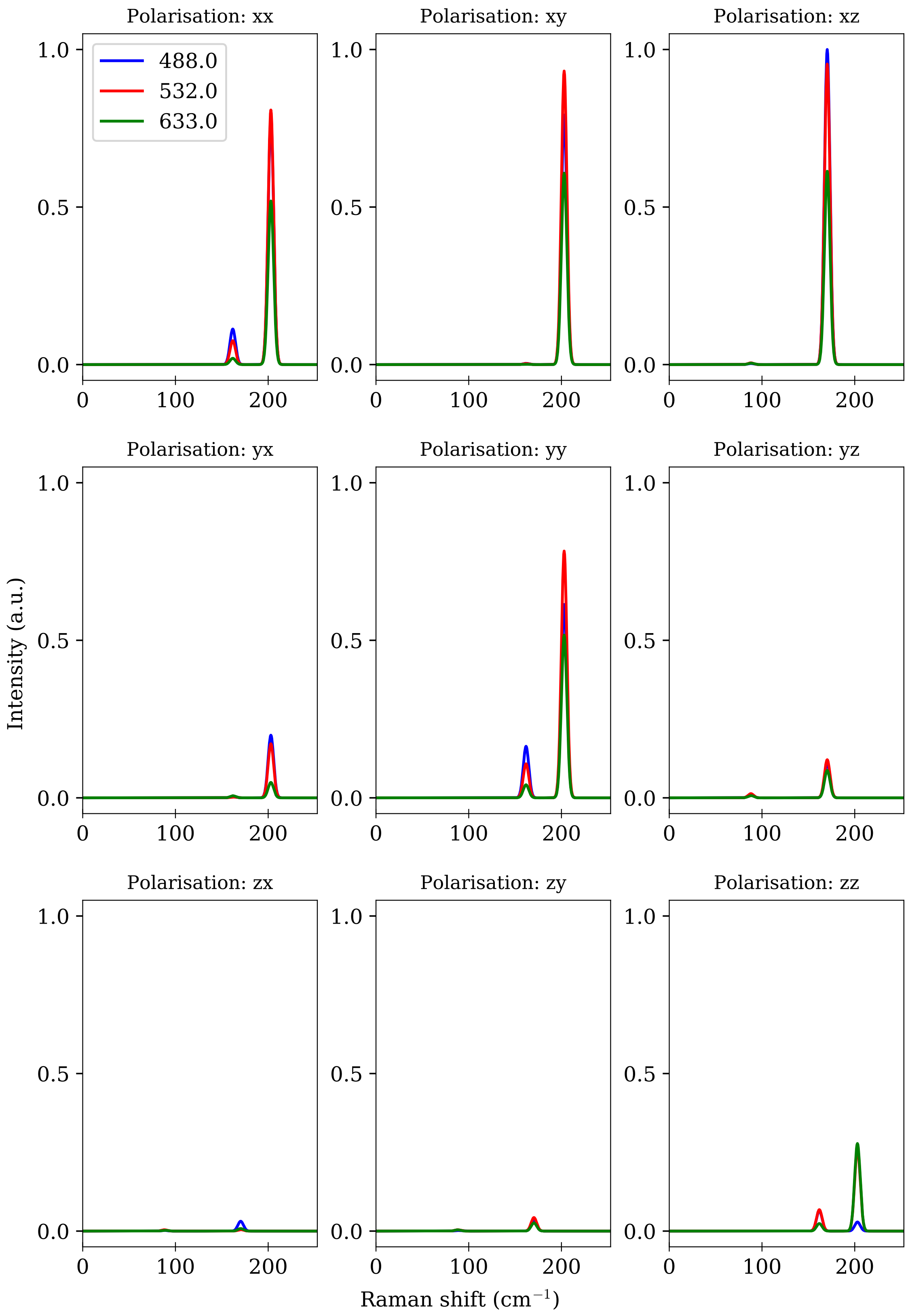

Supplementary Figure 403: Raman spectrum of $NiTe_2$ with space group 187 and Wyckoff positions b-i-0.

$Os_2Br_2S_2$, 59, a-b-0

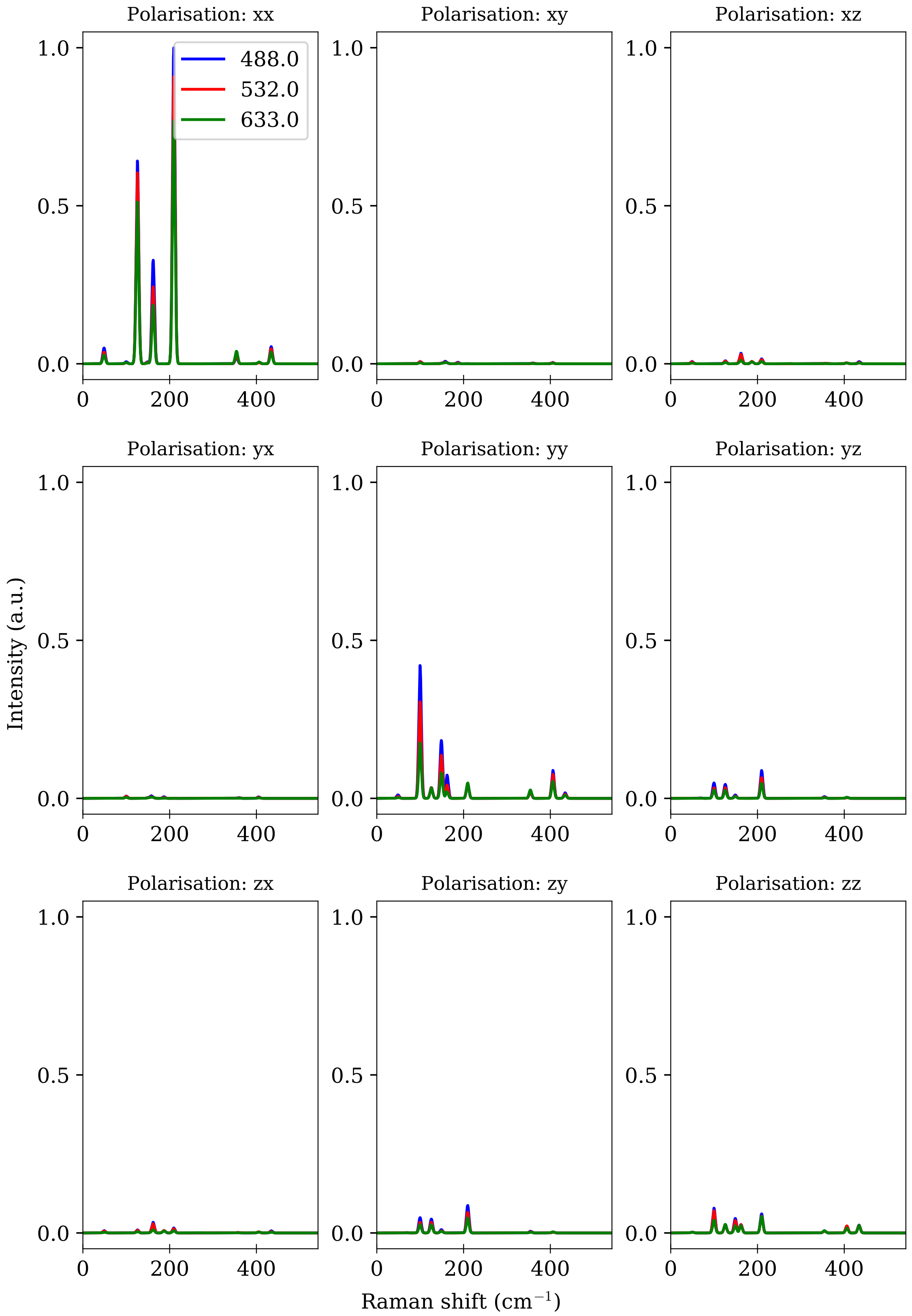

Supplementary Figure 404: Raman spectrum of $Os_2Br_2S_2$ with space group 59 and Wyckoff positions a-b-0.

$Os_2Cl_2S_2$, 59, a-b-0

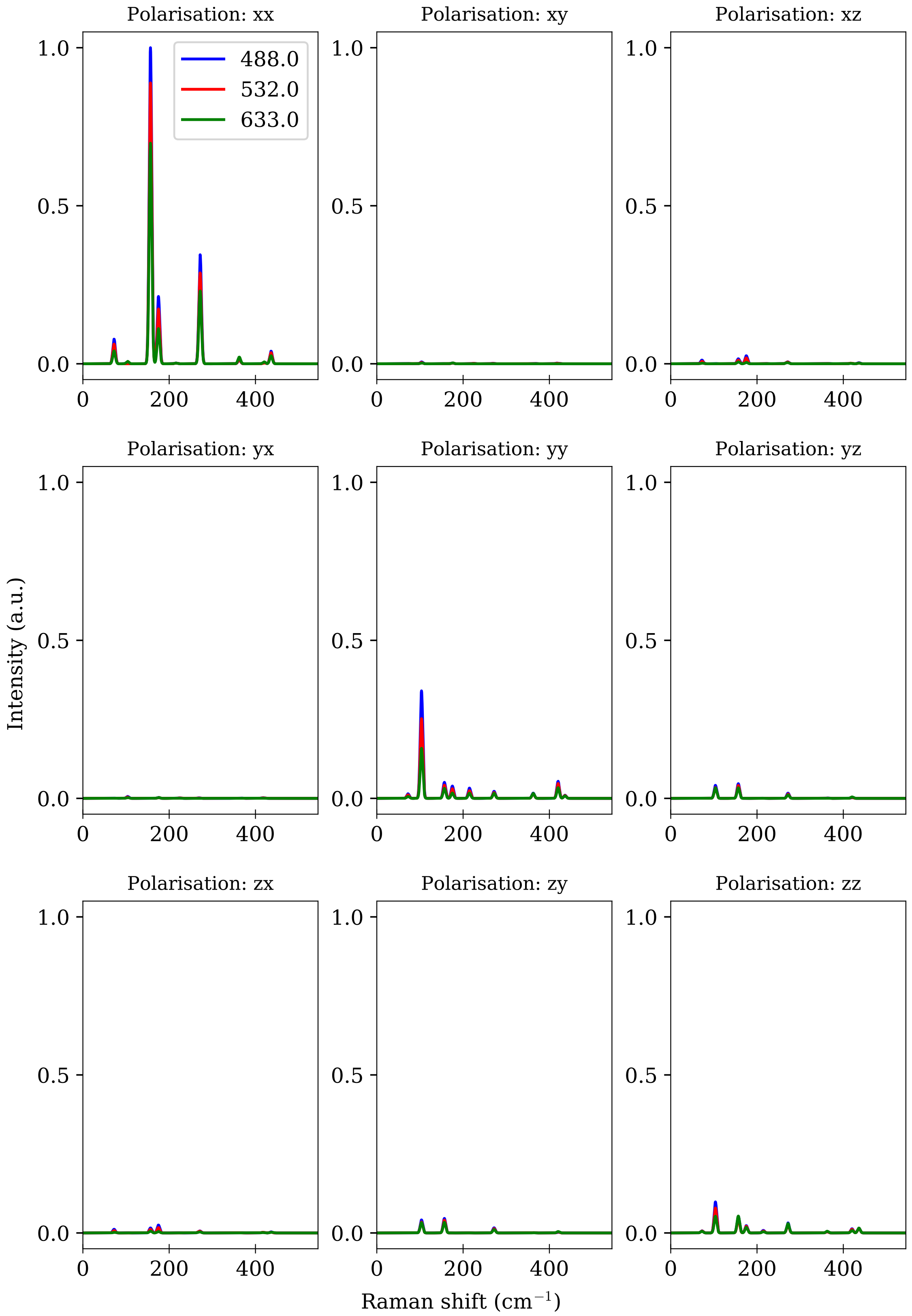

Supplementary Figure 405: Raman spectrum of $Os_2Cl_2S_2$ with space group 59 and Wyckoff positions a-b-0.

$Os_2I_2S_2$, 59, a-b-0

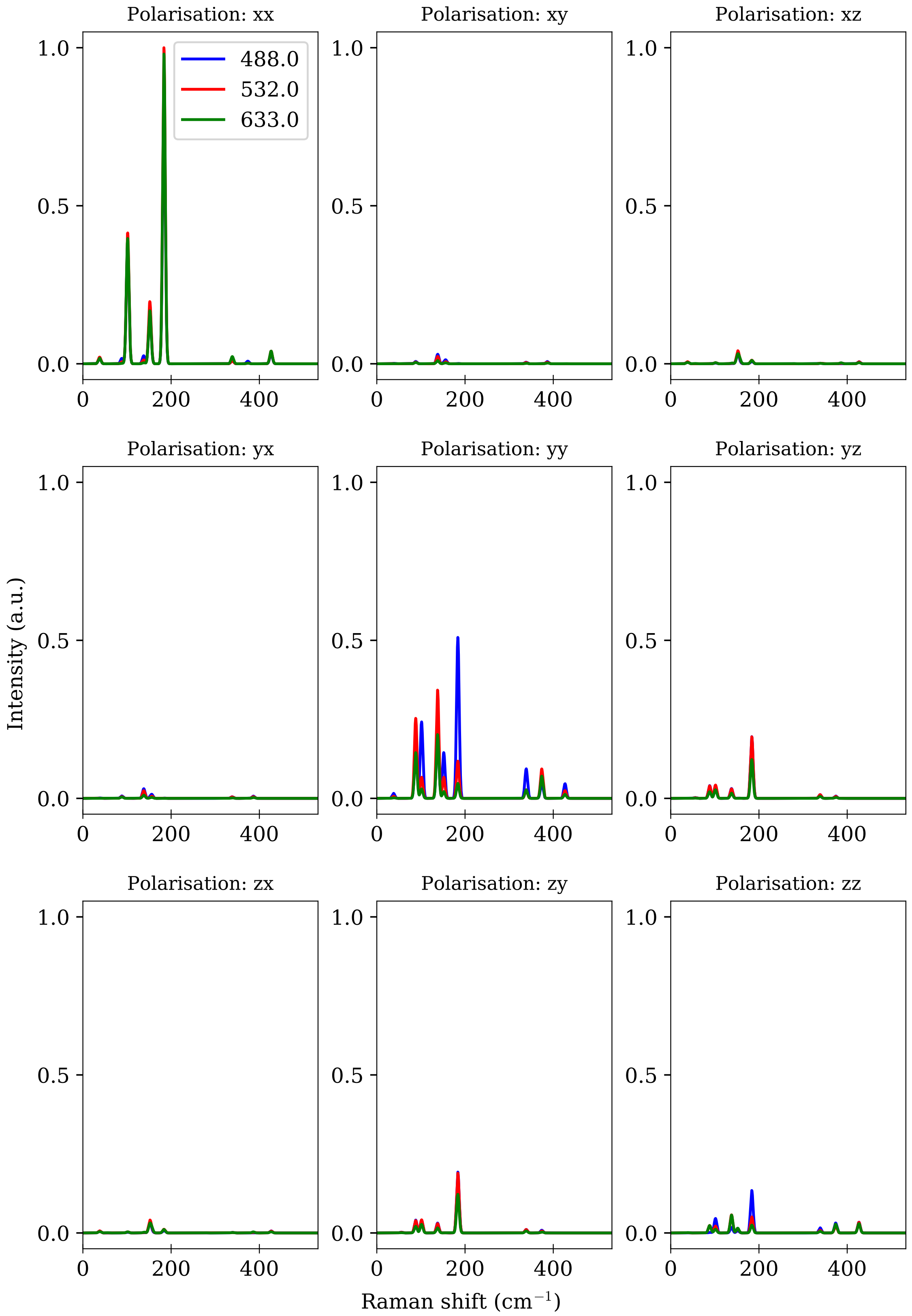

Supplementary Figure 406: Raman spectrum of $Os_2I_2S_2$ with space group 59 and Wyckoff positions a-b-0.

Os_2O_4, 11, e-0

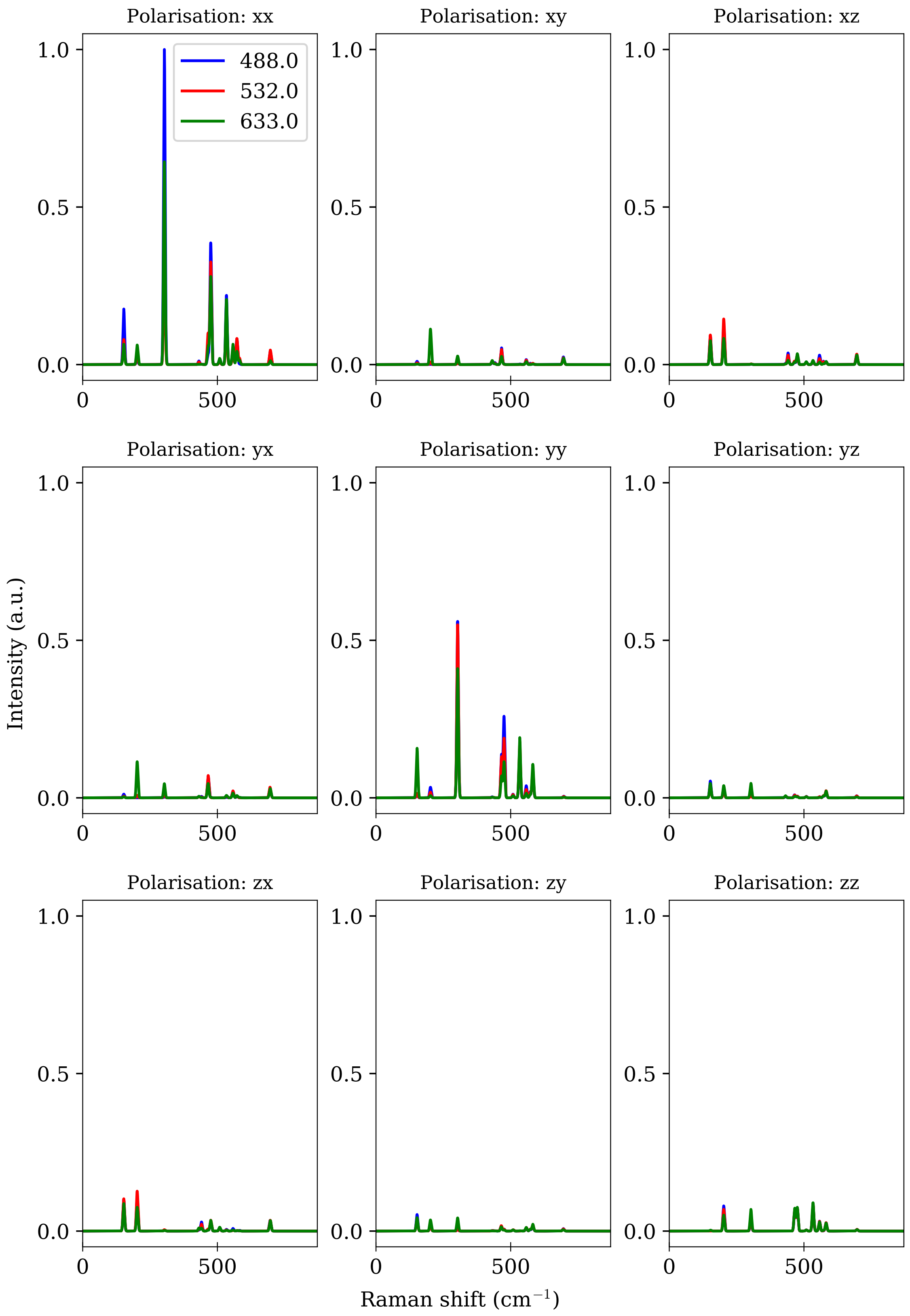

Supplementary Figure 407: Raman spectrum of Os_2O_4 with space group 11 and Wyckoff positions e-0.

Os_2S_2, 129, b-c-0

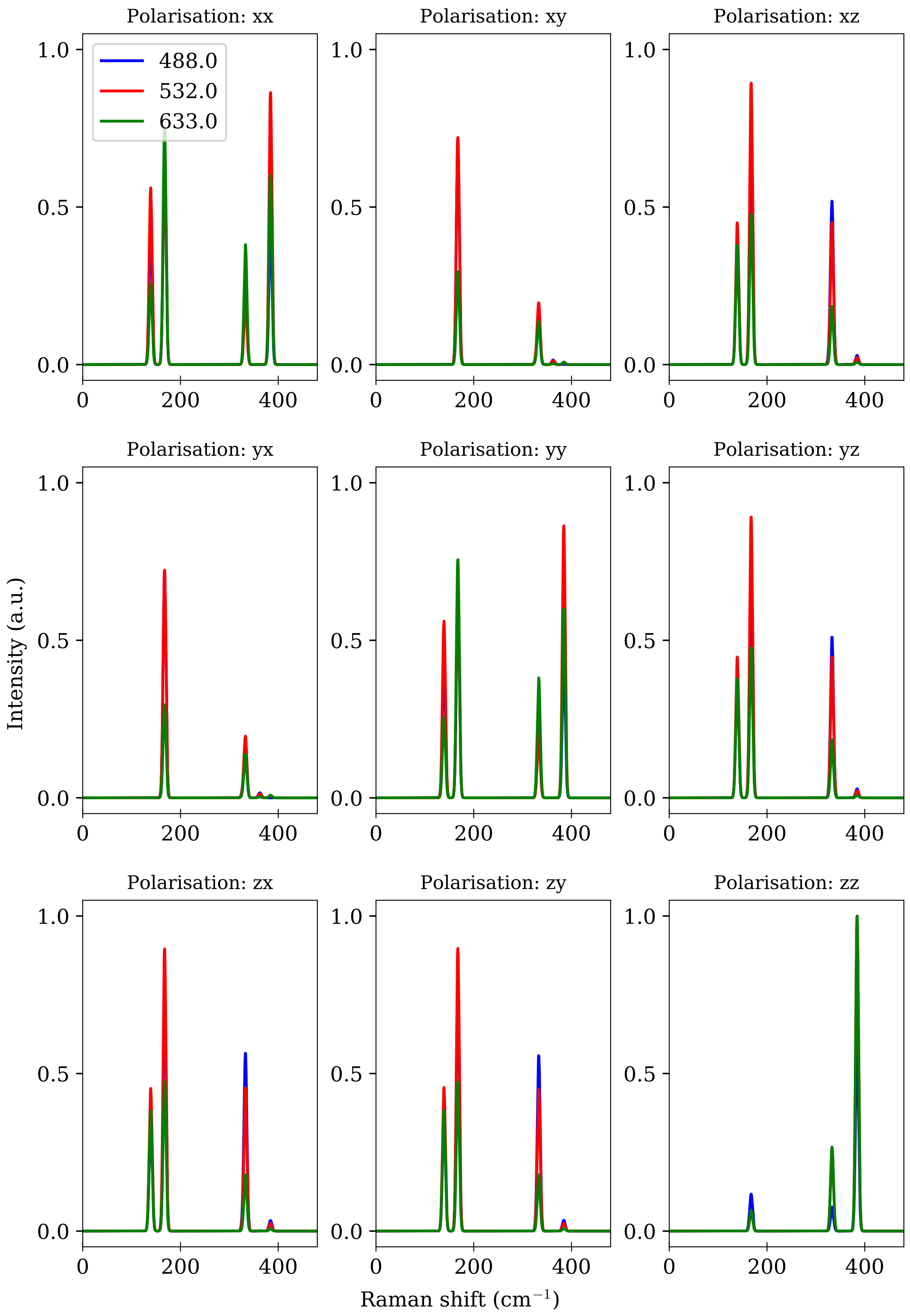

Supplementary Figure 408: Raman spectrum of Os_2S_2 with space group 129 and Wyckoff positions b-c-0.

Os_2S_4, 11, e-0

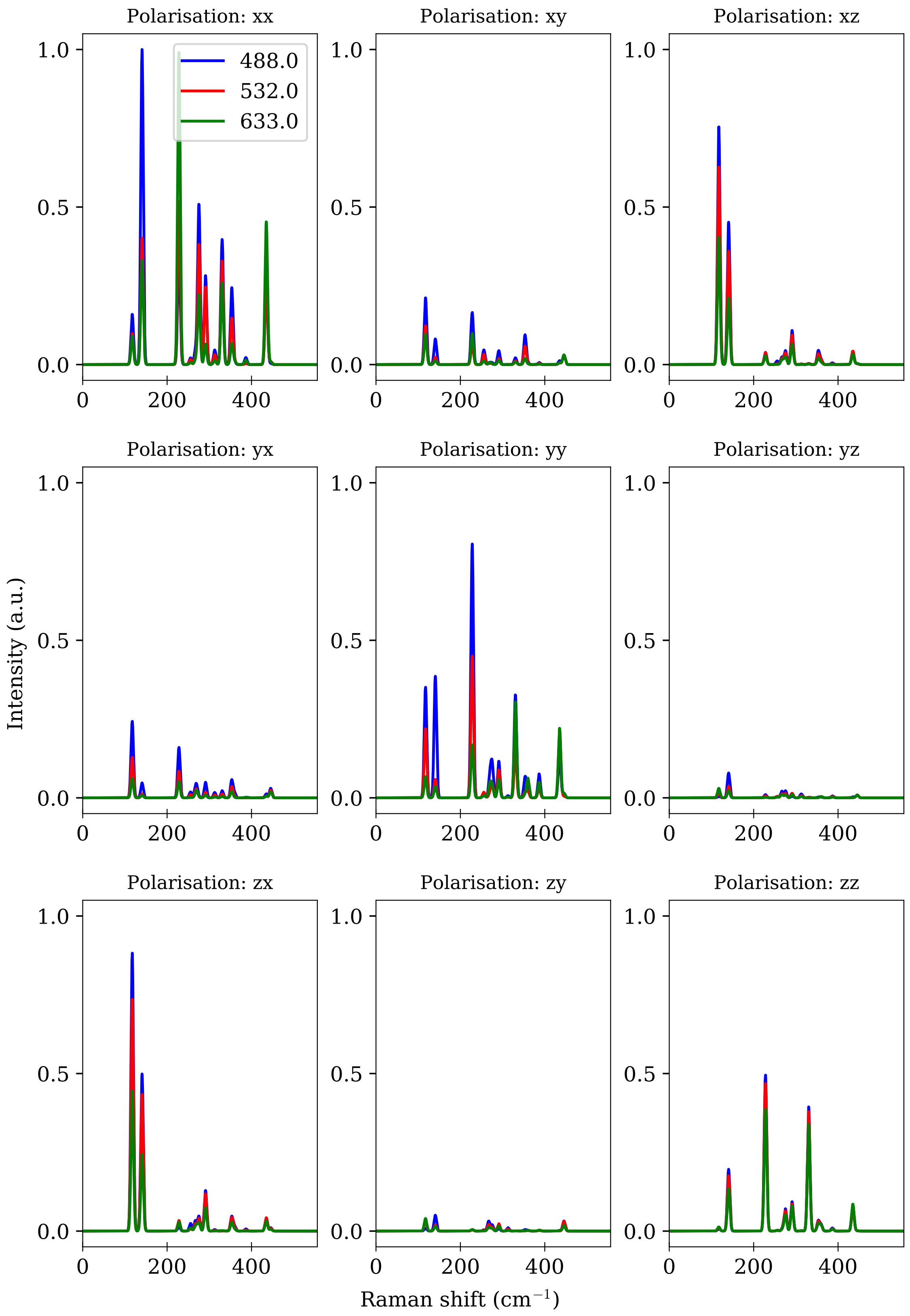

Supplementary Figure 409: Raman spectrum of Os_2S_4 with space group 11 and Wyckoff positions e-0.

Os_2Se_2, 129, b-c-0

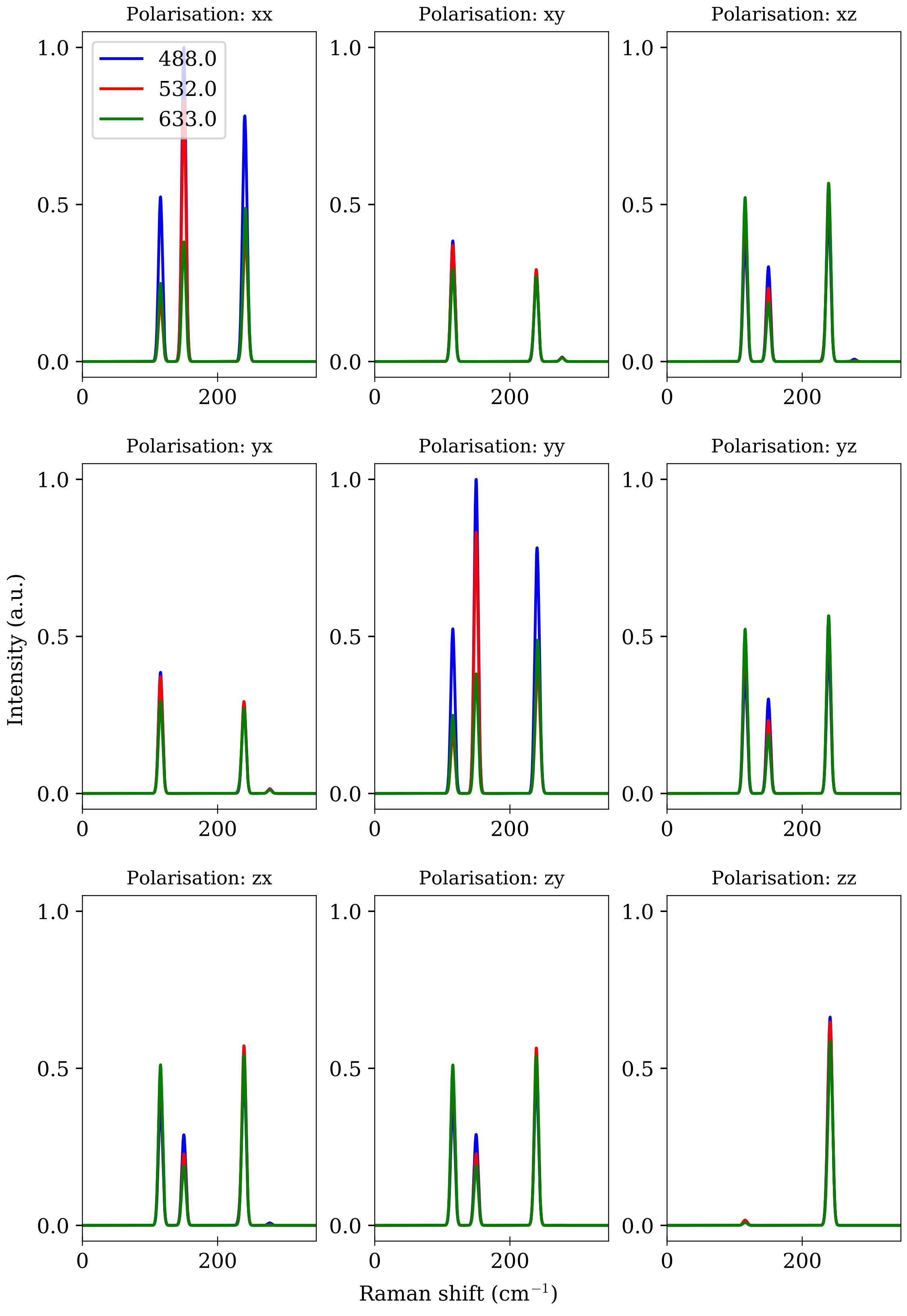

Supplementary Figure 410: Raman spectrum of Os_2Se_2 with space group 129 and Wyckoff positions b-c-0.

Os_2Se_4, 11, e-0

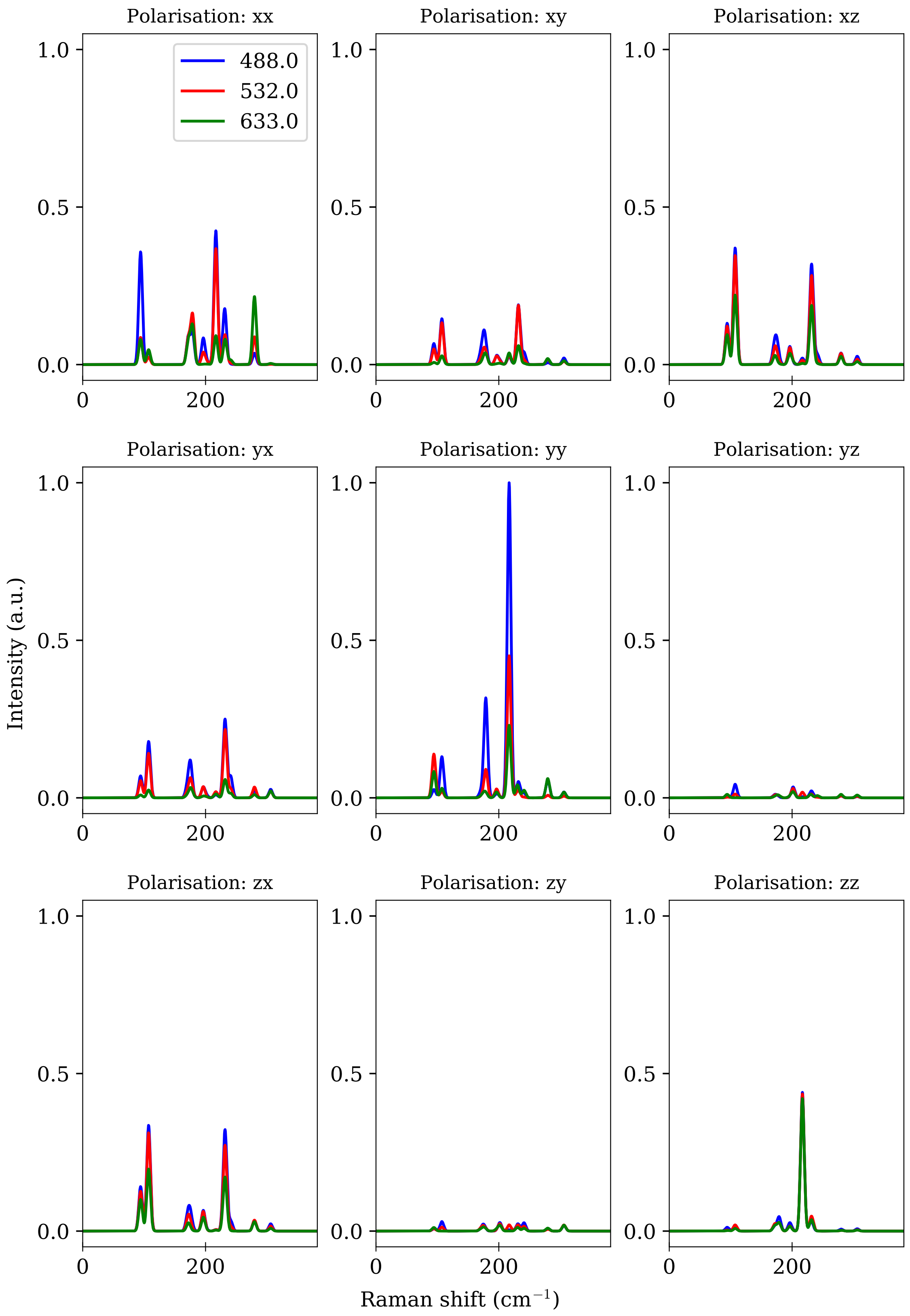

Supplementary Figure 411: Raman spectrum of Os_2Se_4 with space group 11 and Wyckoff positions e-0.

Os_2Te_2, 129, b-c-0

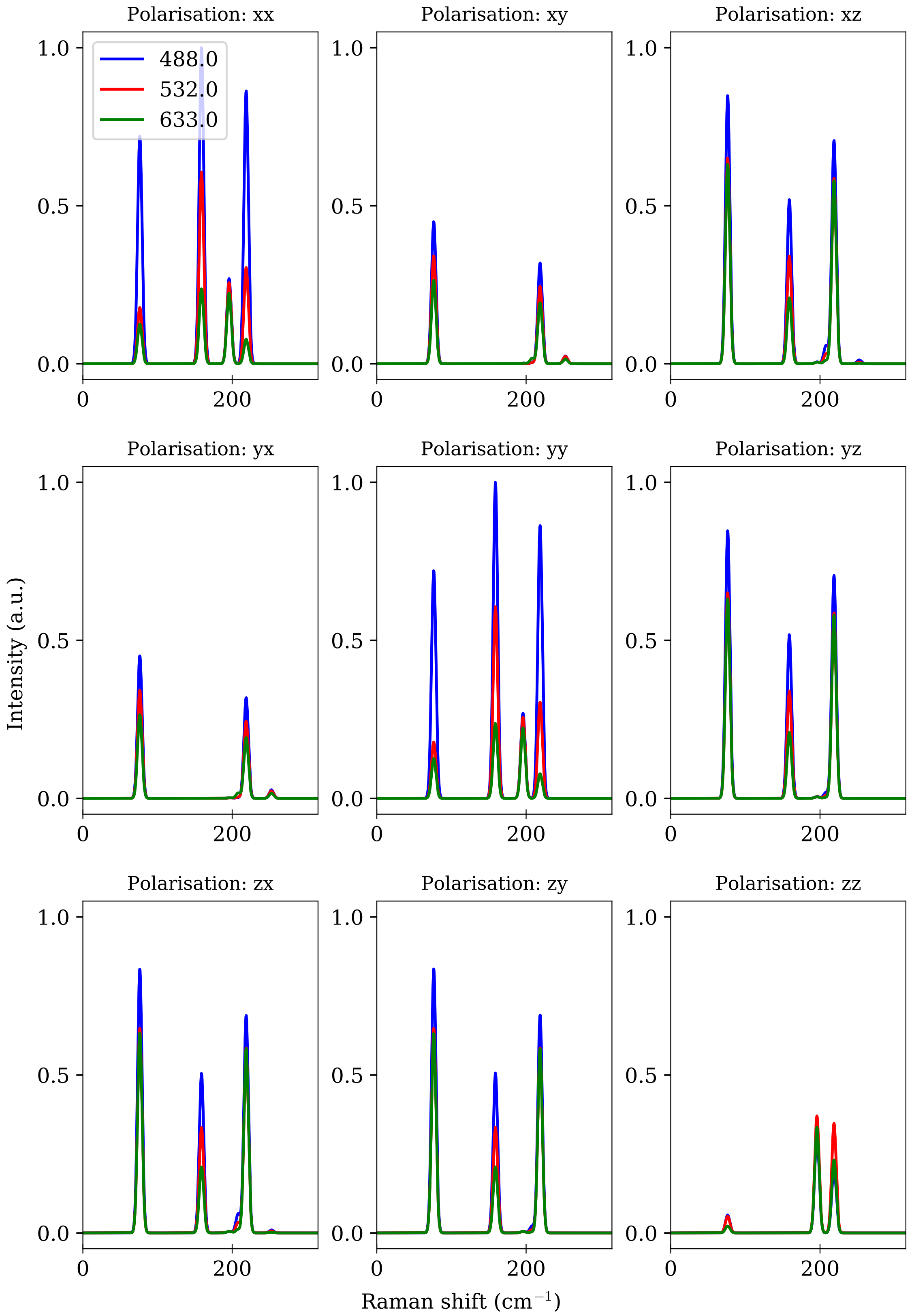

Supplementary Figure 412: Raman spectrum of Os_2Te_2 with space group 129 and Wyckoff positions b-c-0.

Os_2Te_2, 12, i-0

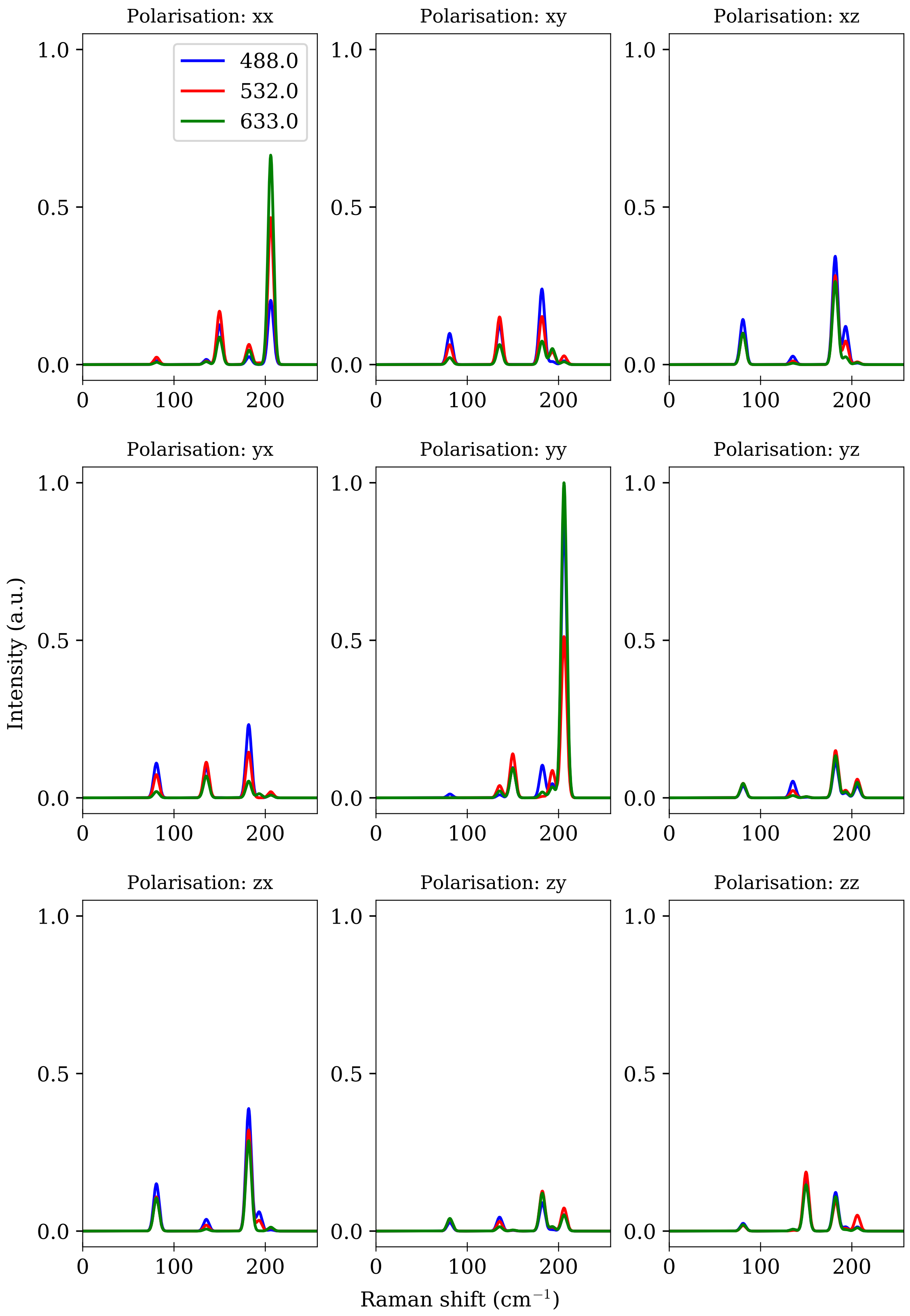

Supplementary Figure 413: Raman spectrum of Os_2Te_2 with space group 12 and Wyckoff positions i-0.

Os_2Te_4, 11, e-0

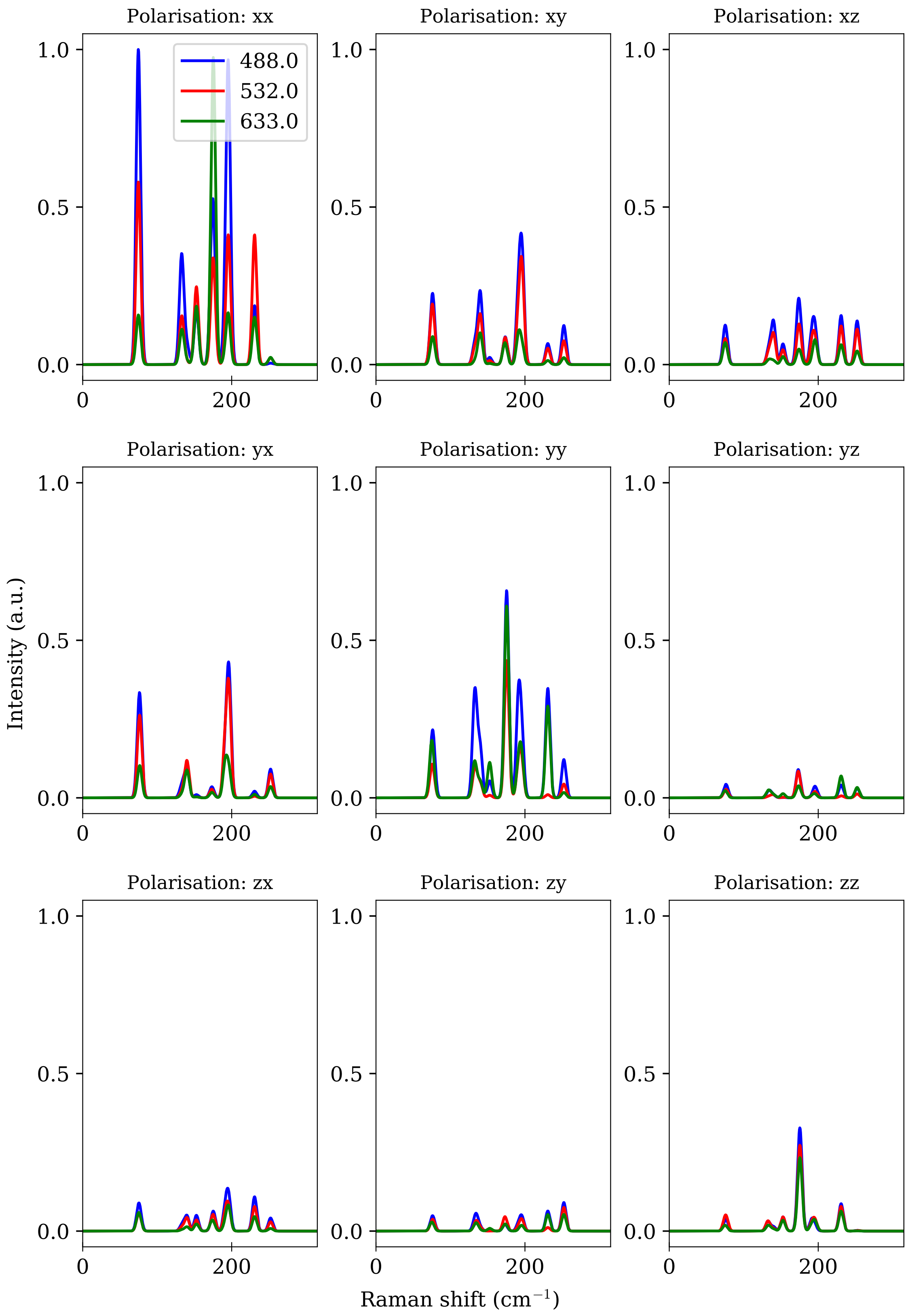

Supplementary Figure 414: Raman spectrum of Os_2Te_4 with space group 11 and Wyckoff positions e-0.

$OsBr_2$, 164, b-d-0

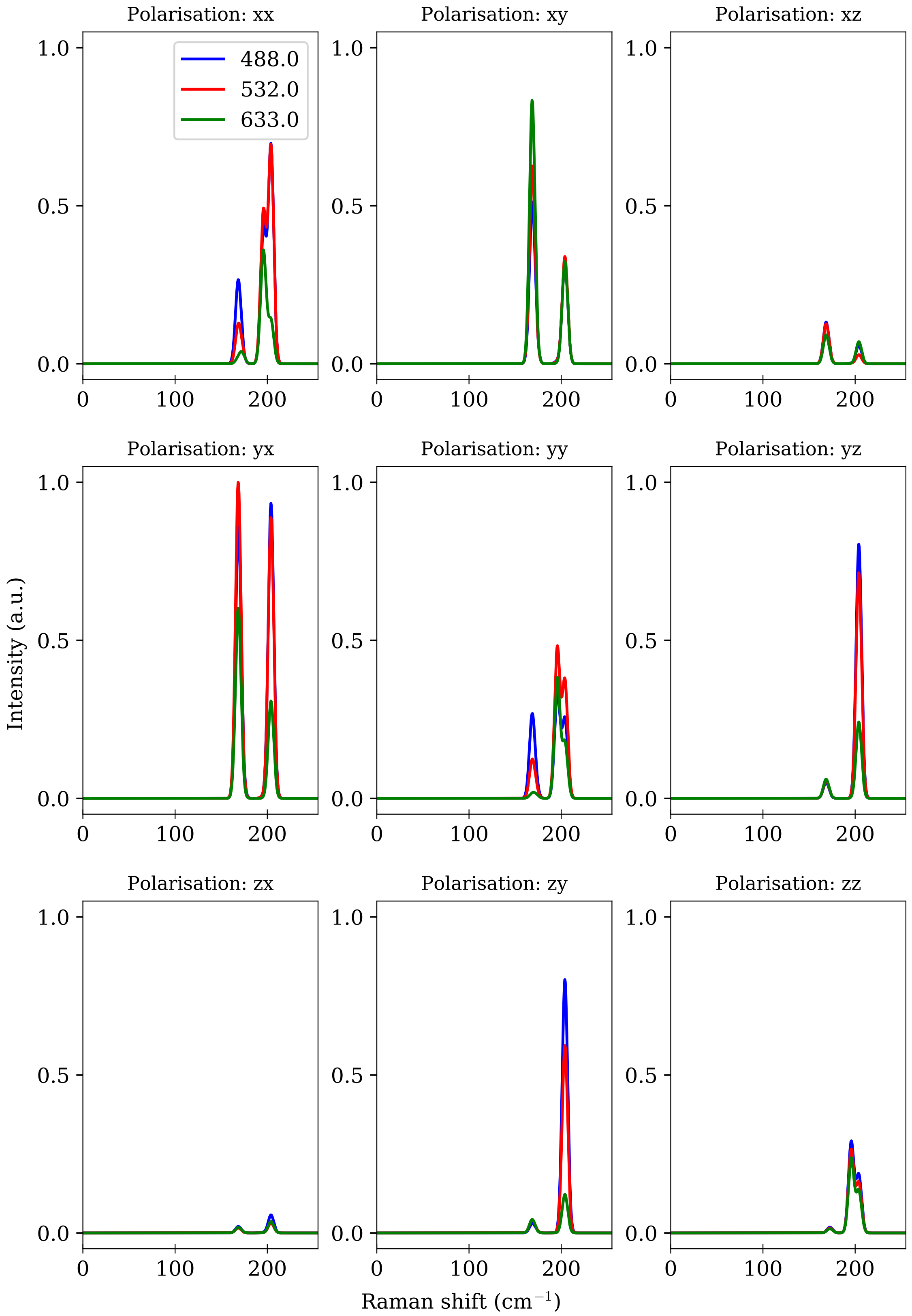

Supplementary Figure 415: Raman spectrum of $OsBr_2$ with space group 164 and Wyckoff positions b-d-0.

$OsCl_2$, 164, b-d-0

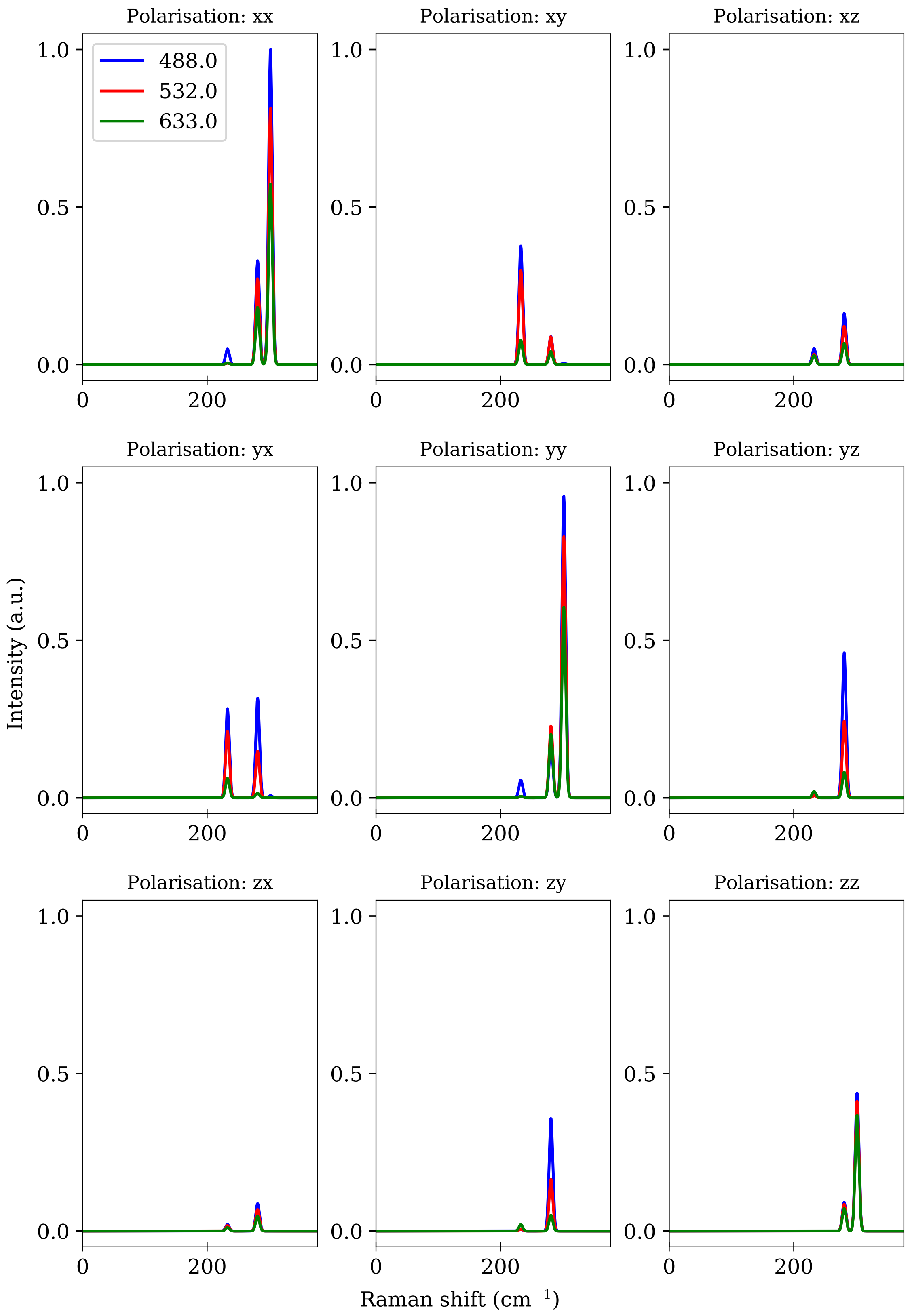

Supplementary Figure 416: Raman spectrum of $OsCl_2$ with space group 164 and Wyckoff positions b-d-0.

OsH_2, 187, b-i-0

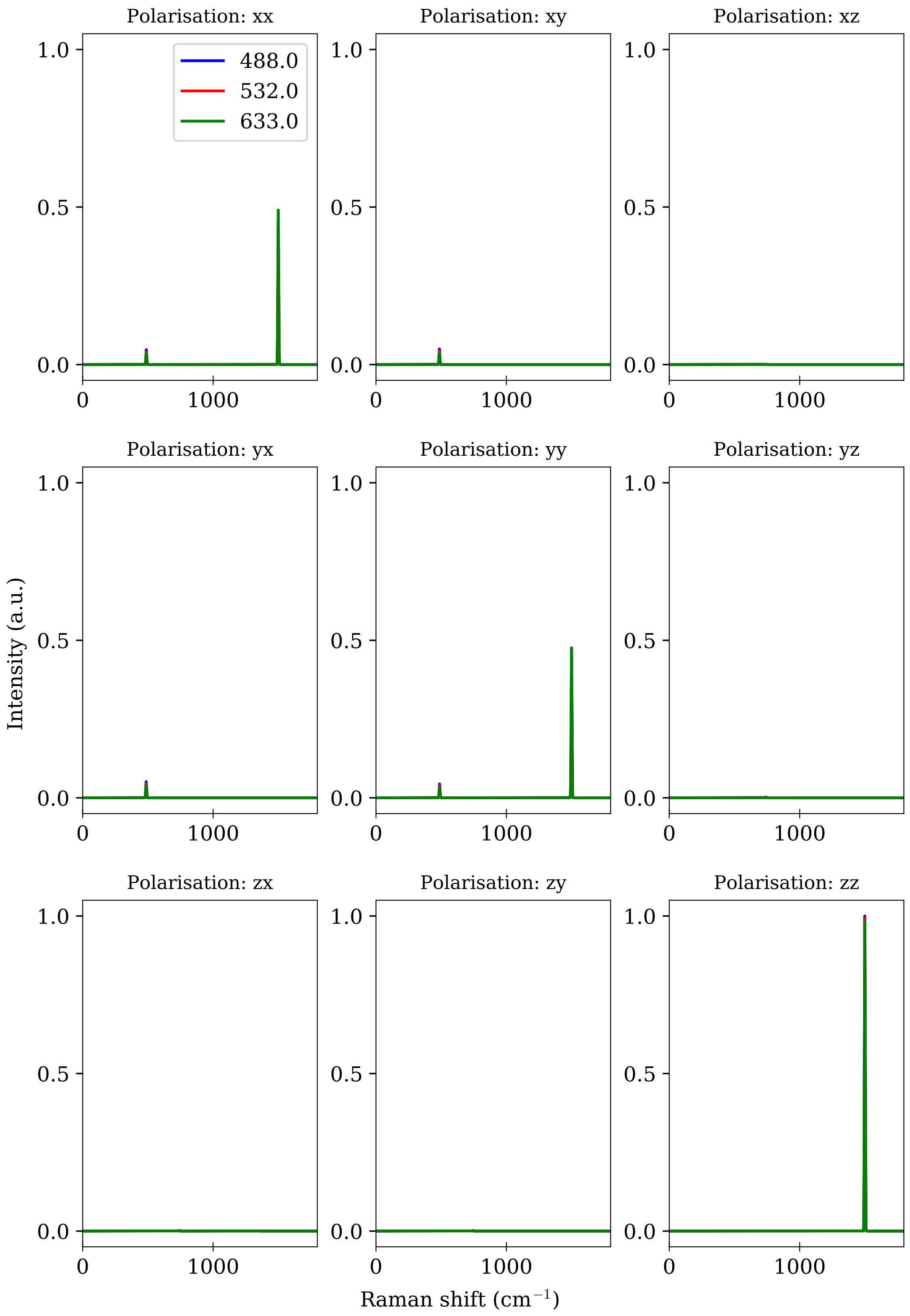

Supplementary Figure 417: Raman spectrum of OsH_2 with space group 187 and Wyckoff positions b-i-0.

OsI_2, 164, b-d-0

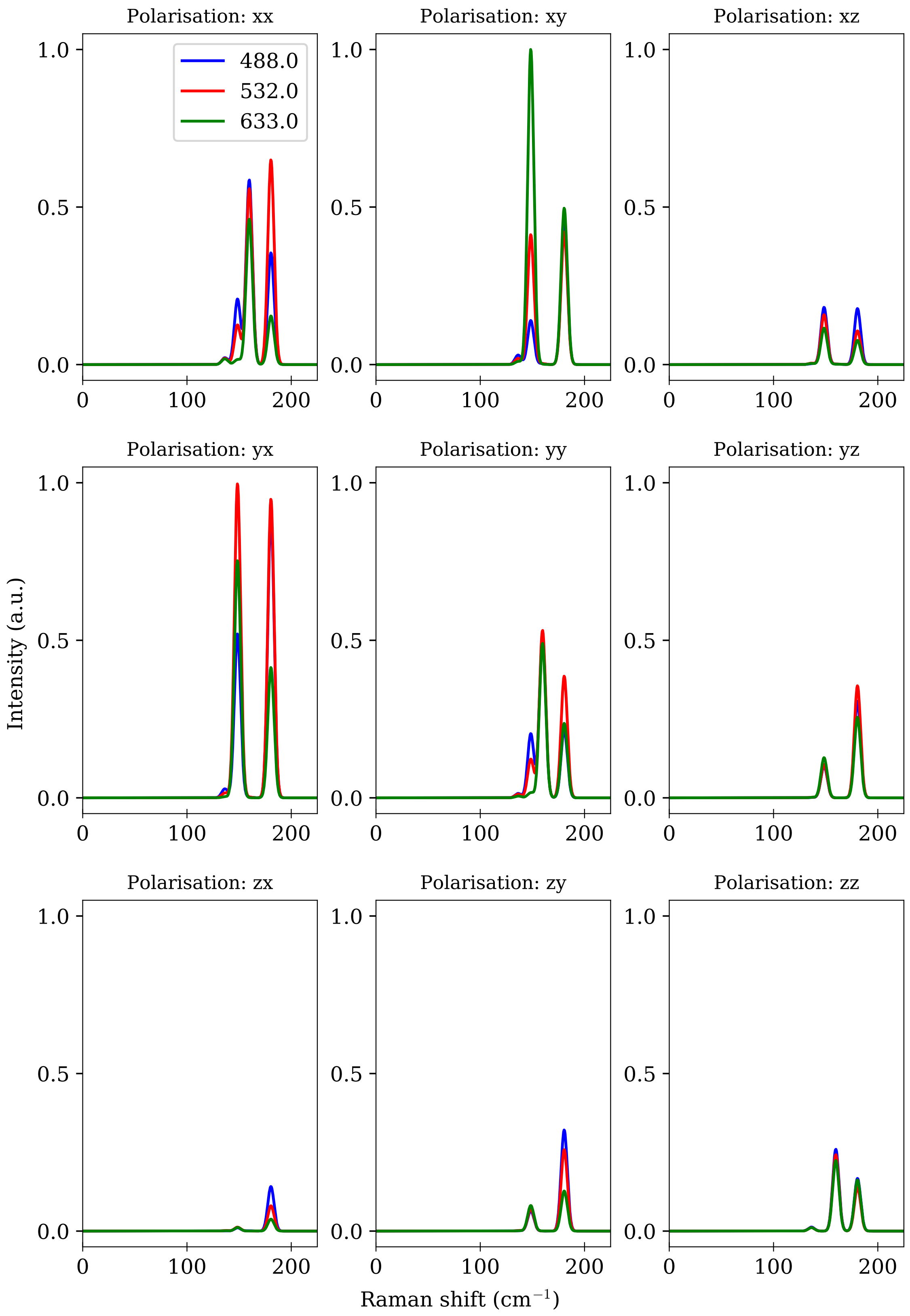

Supplementary Figure 418: Raman spectrum of OsI_2 with space group 164 and Wyckoff positions b-d-0.

P_4, 53, h-0

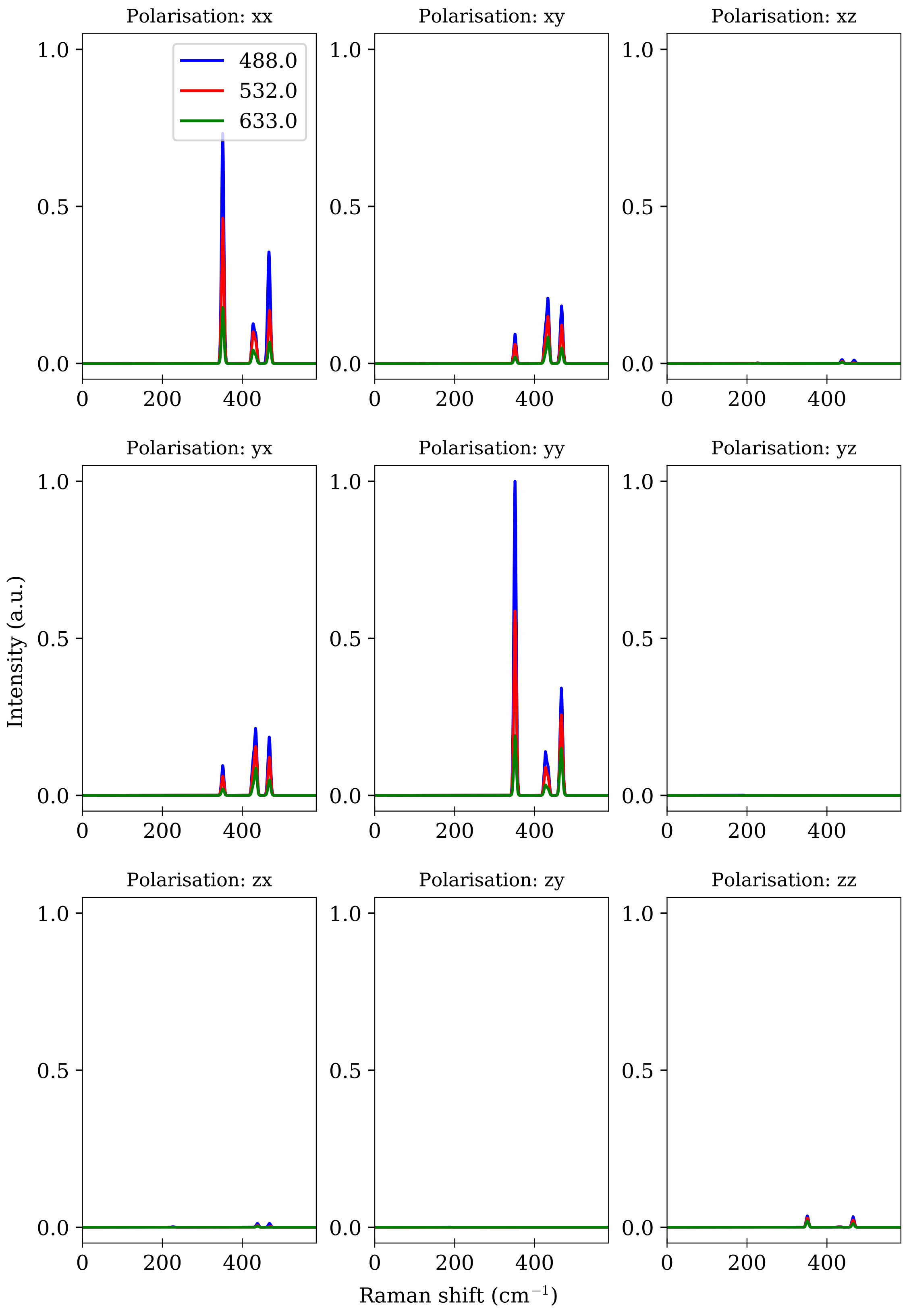

Supplementary Figure 419: Raman spectrum of P_4 with space group 53 and Wyckoff positions h-0.

Pb_2Se_2, 31, a-0

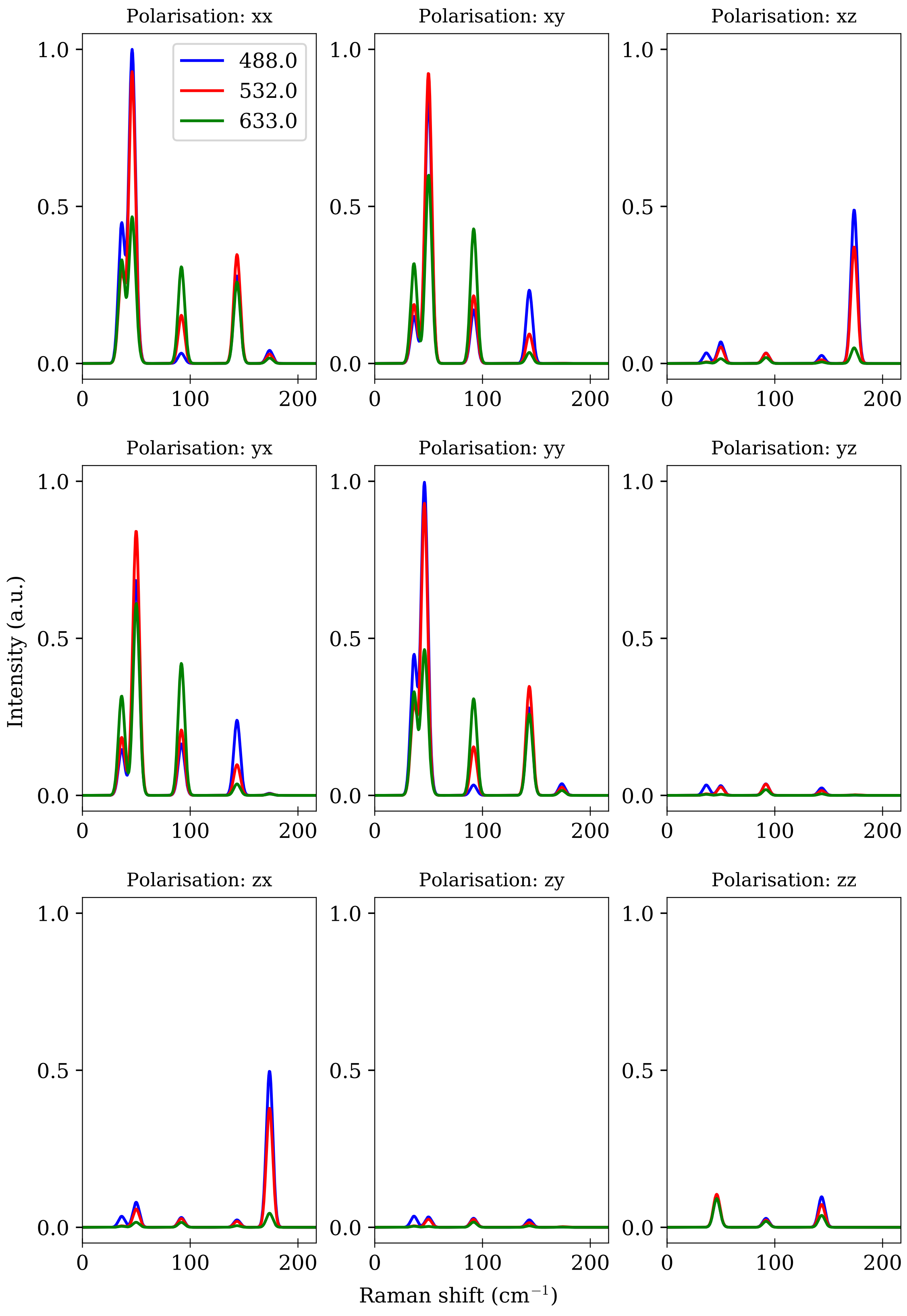

Supplementary Figure 420: Raman spectrum of Pb_2Se_2 with space group 31 and Wyckoff positions a-0.

Pb_2Te_2, 2, i-0

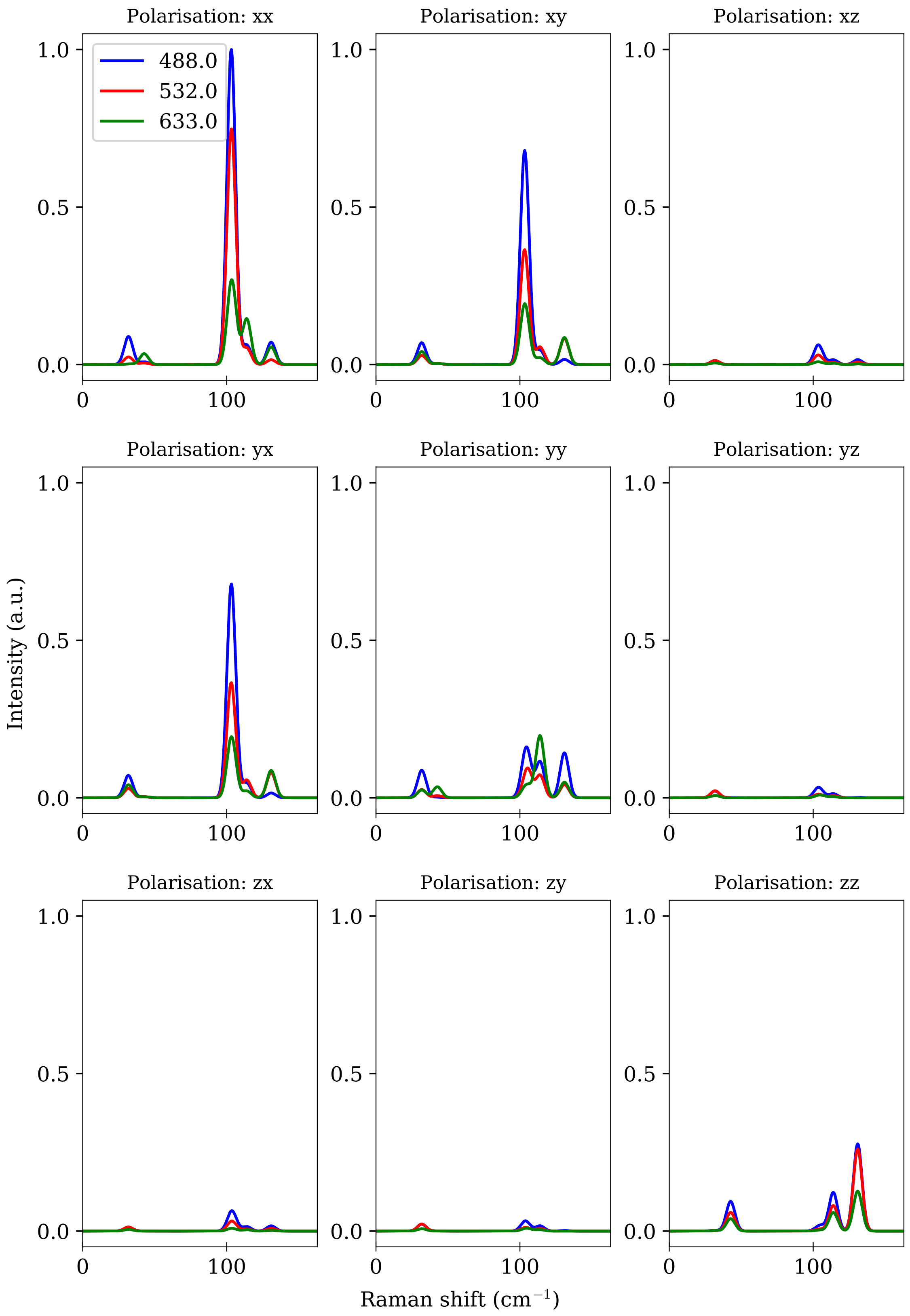

Supplementary Figure 421: Raman spectrum of Pb_2Te_2 with space group 2 and Wyckoff positions i-0.

$PbBr_2$, 164, b-d-0

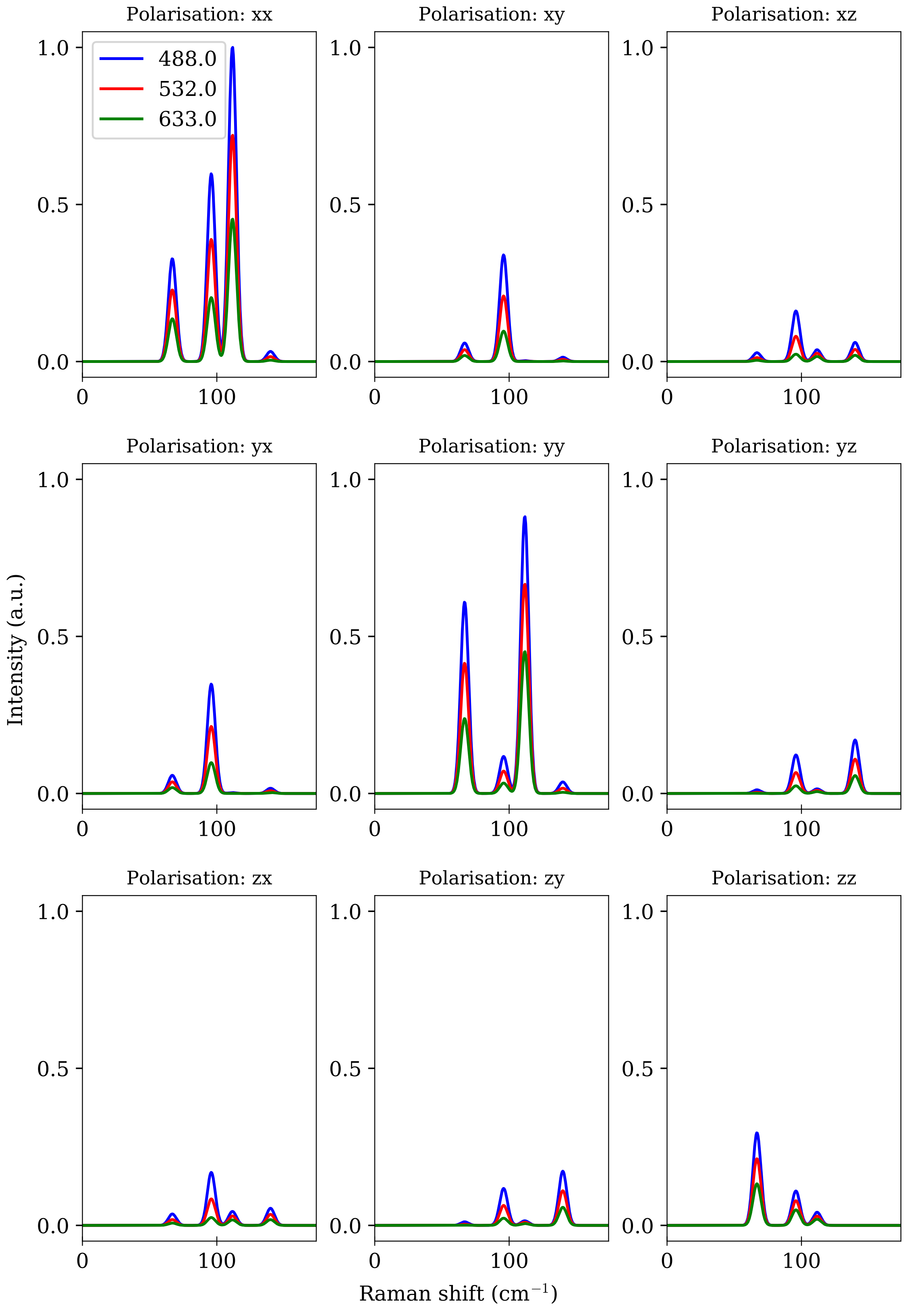

Supplementary Figure 422: Raman spectrum of $PbBr_2$ with space group 164 and Wyckoff positions b-d-0.

PbO_2, 164, b-d-0

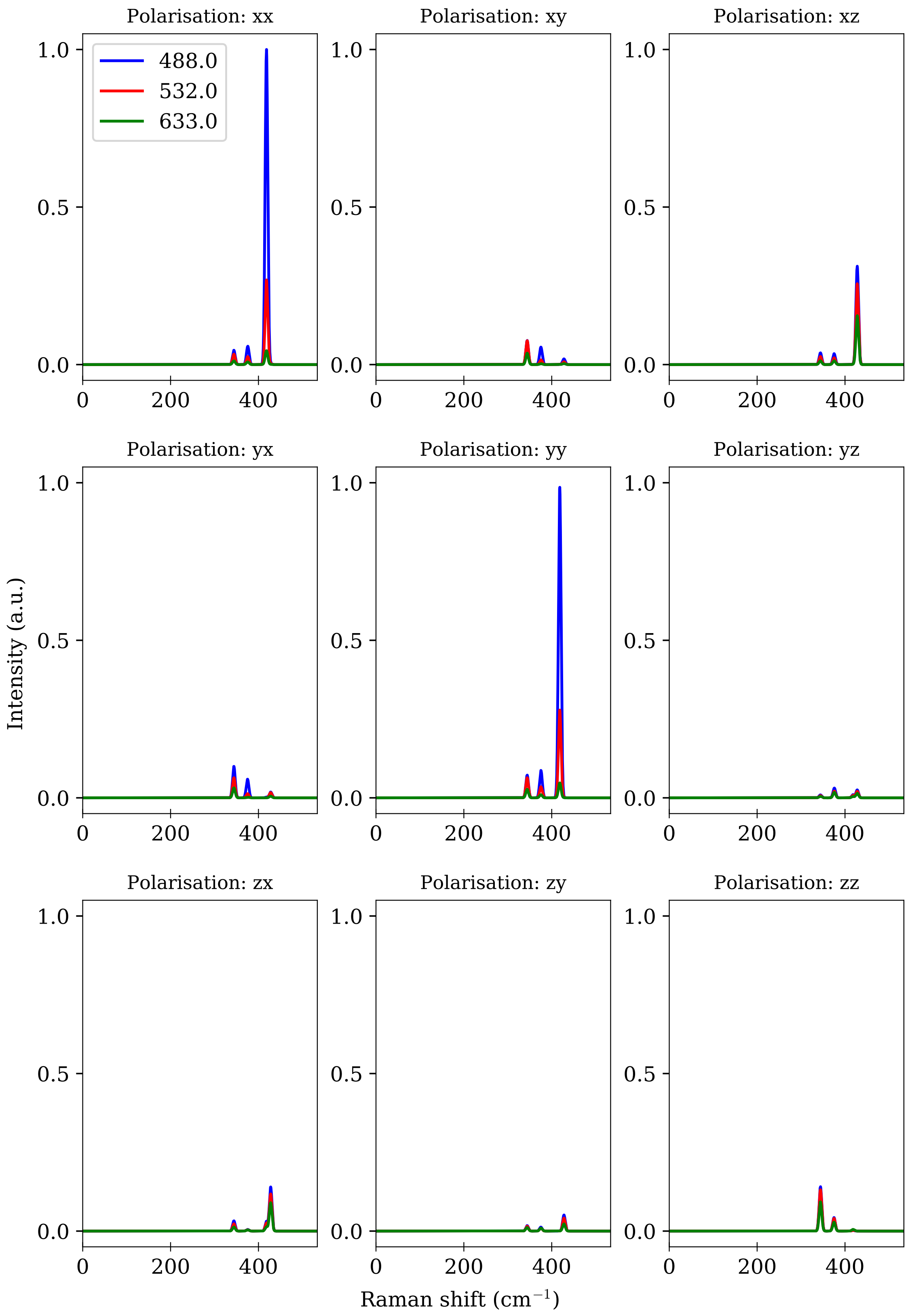

Supplementary Figure 423: Raman spectrum of PbO_2 with space group 164 and Wyckoff positions b-d-0.

PbO, 156, b-c-0

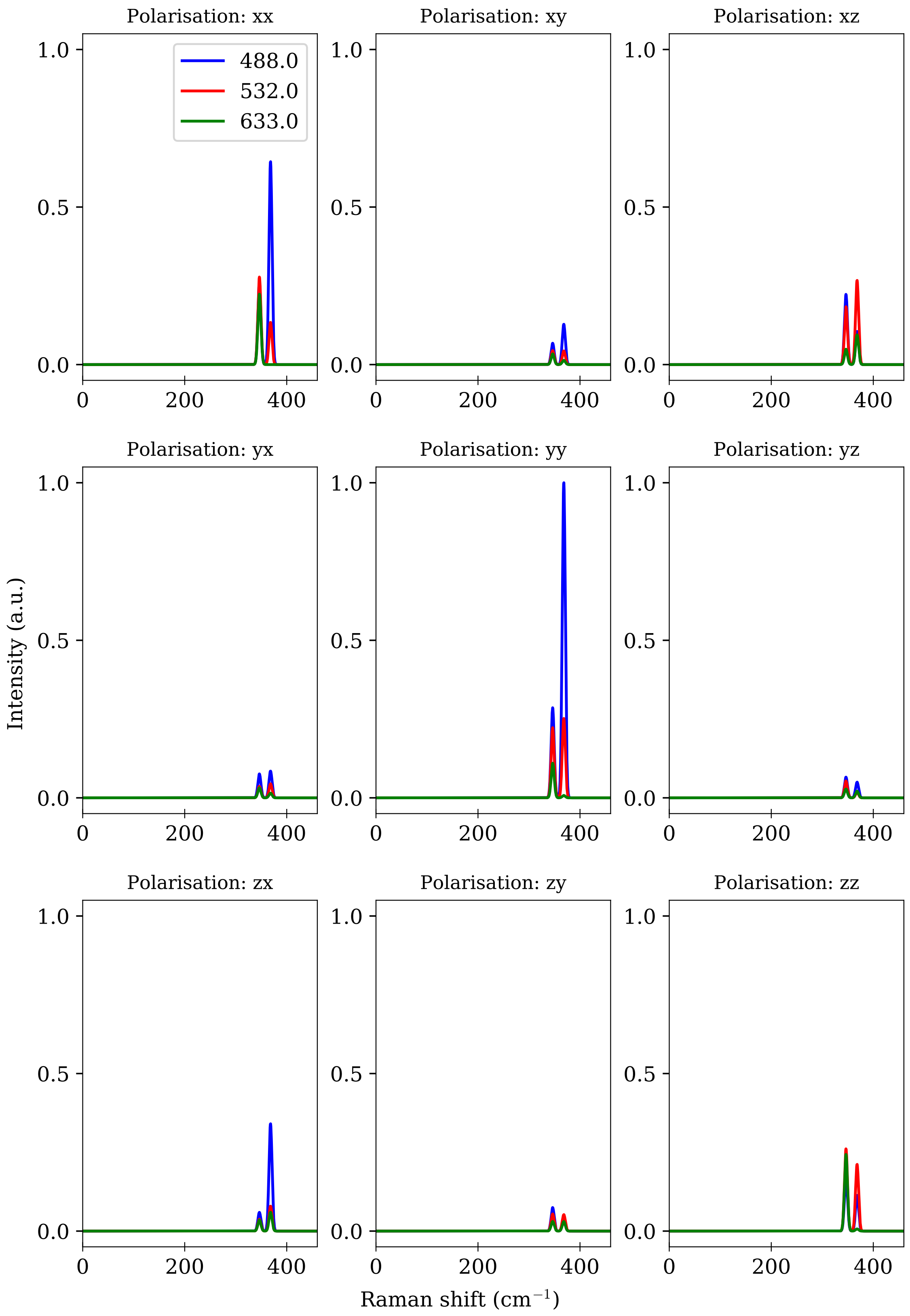

Supplementary Figure 424: Raman spectrum of PbO with space group 156 and Wyckoff positions b-c-0.

PbS_2, 164, b-d-0

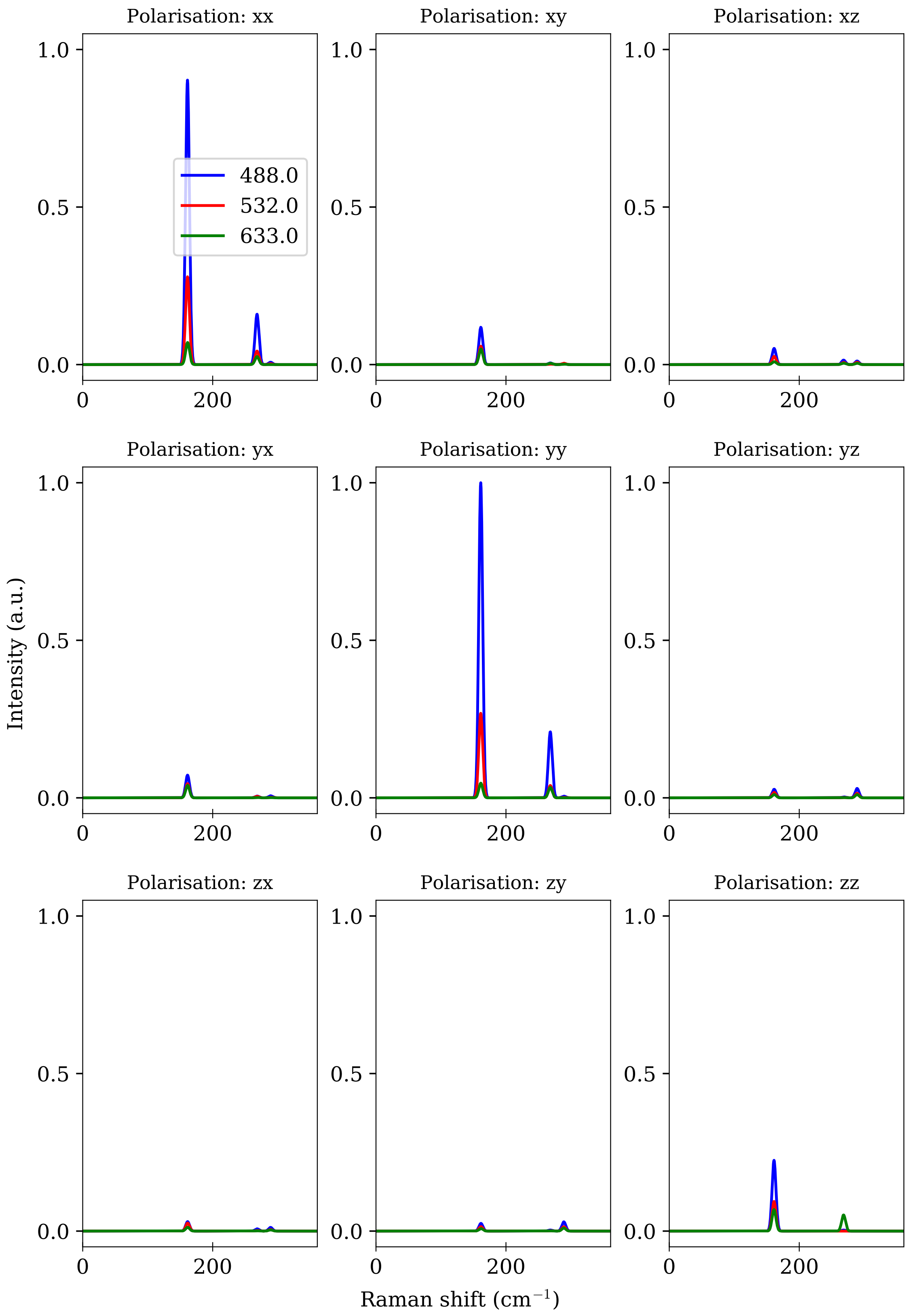

Supplementary Figure 425: Raman spectrum of PbS_2 with space group 164 and Wyckoff positions b-d-0.

PbS_2, 187, b-i-0

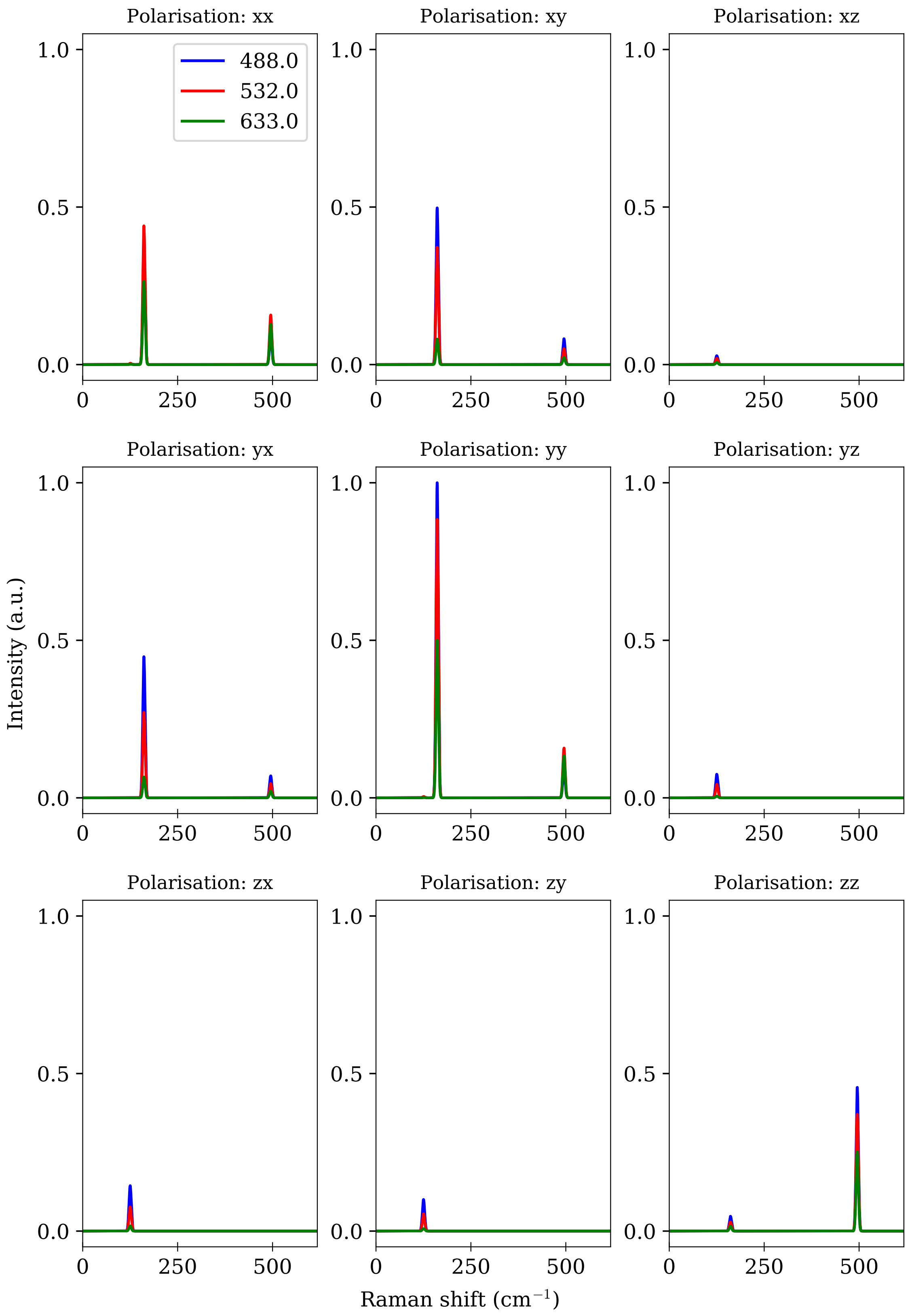

Supplementary Figure 426: Raman spectrum of PbS_2 with space group 187 and Wyckoff positions b-i-0.

$PbSe_2$, 164, b-d-0

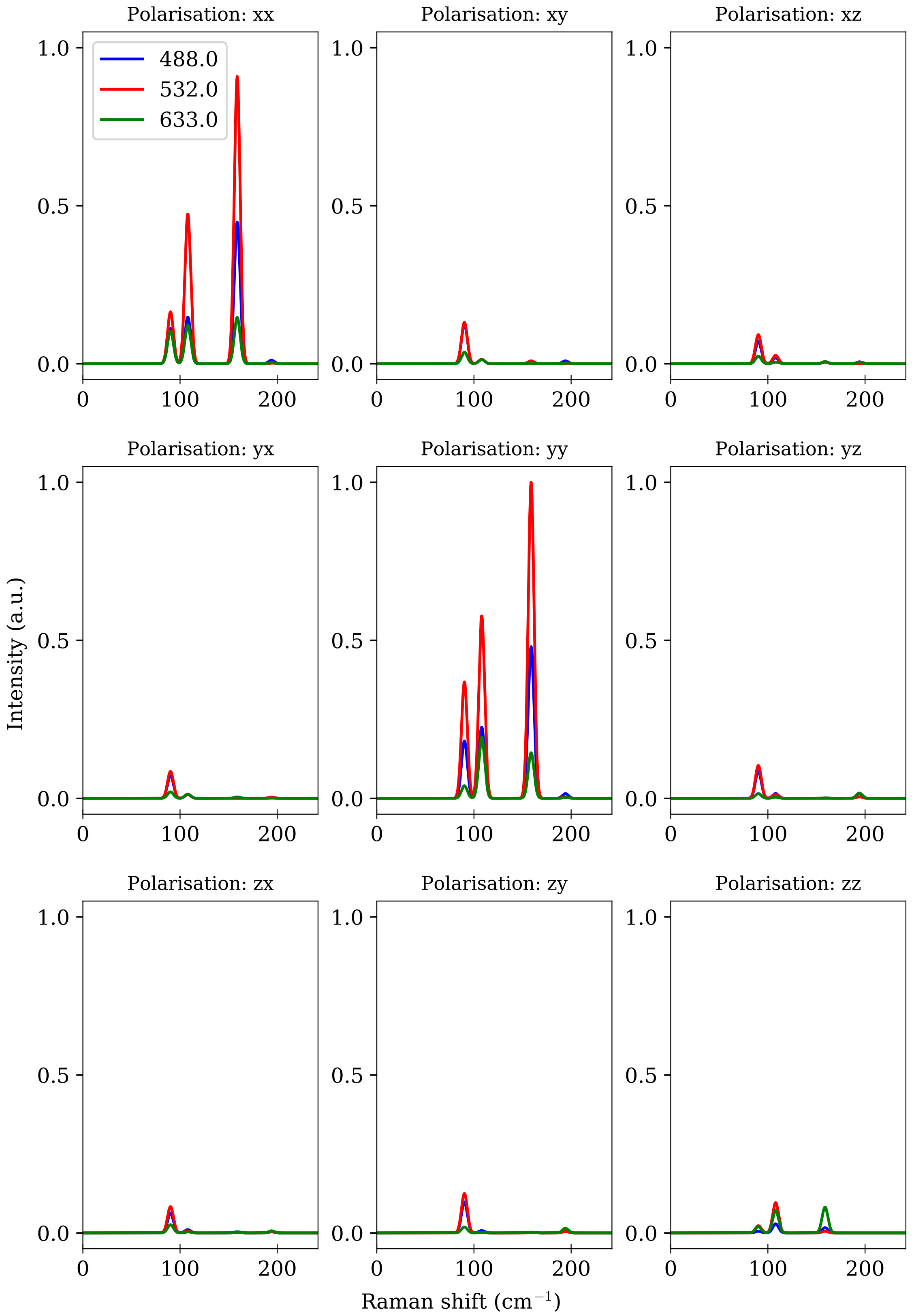

Supplementary Figure 427: Raman spectrum of $PbSe_2$ with space group 164 and Wyckoff positions b-d-0.

PbSe, 156, b-c-0

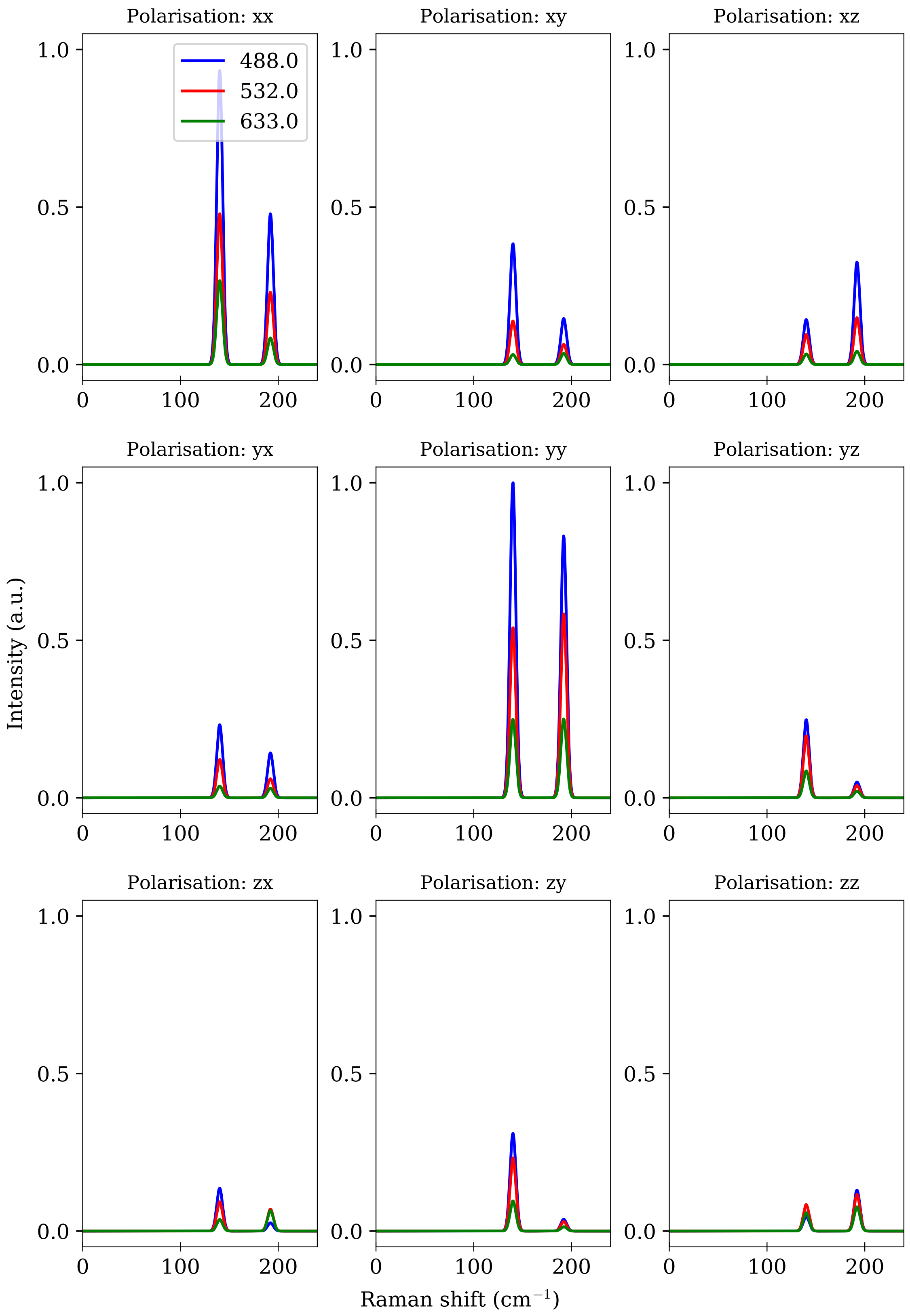

Supplementary Figure 428: Raman spectrum of PbSe with space group 156 and Wyckoff positions b-c-0.

PbS, 156, b-c-0

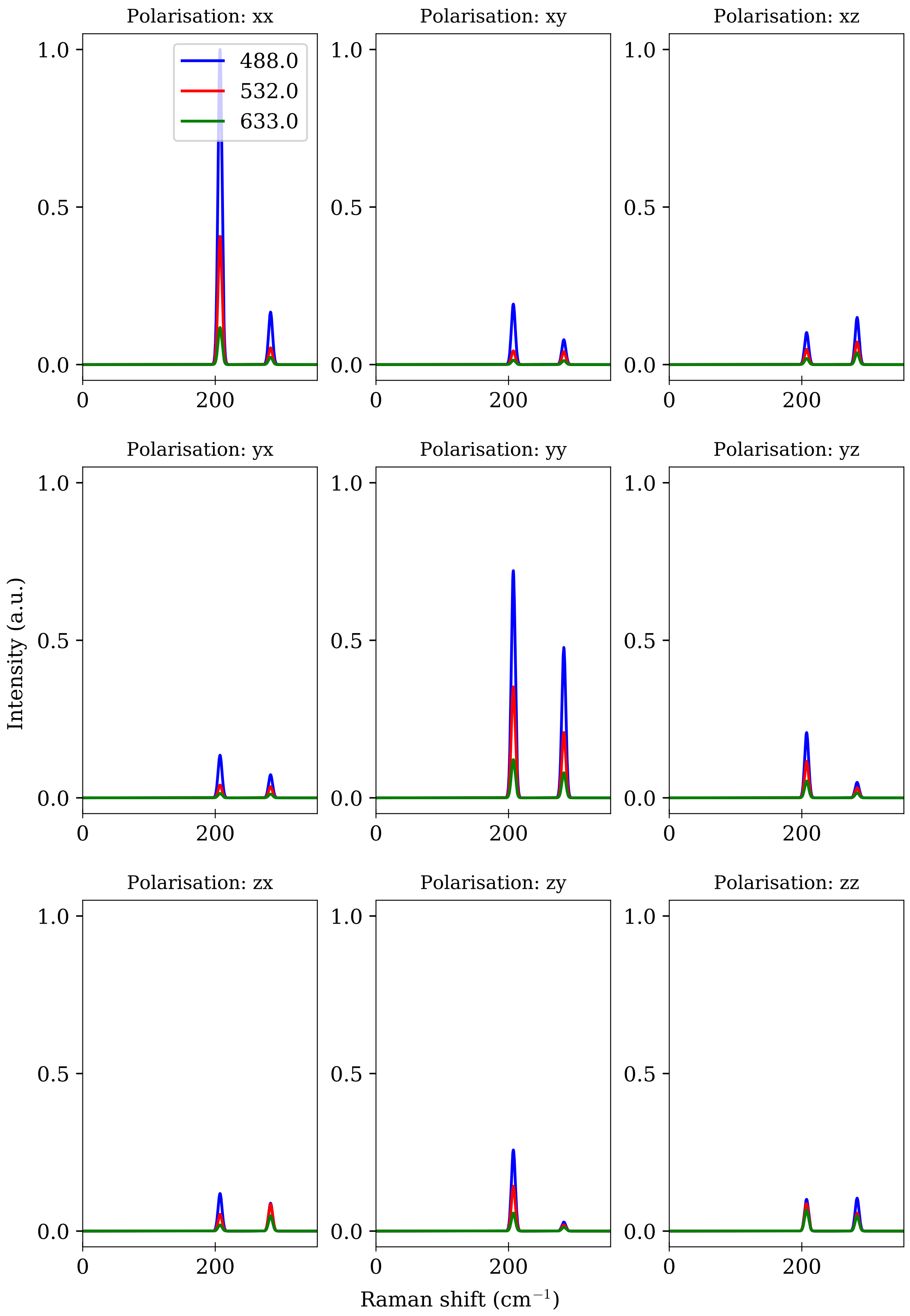

Supplementary Figure 429: Raman spectrum of PbS with space group 156 and Wyckoff positions b-c-0.

PbTe, 156, b-c-0

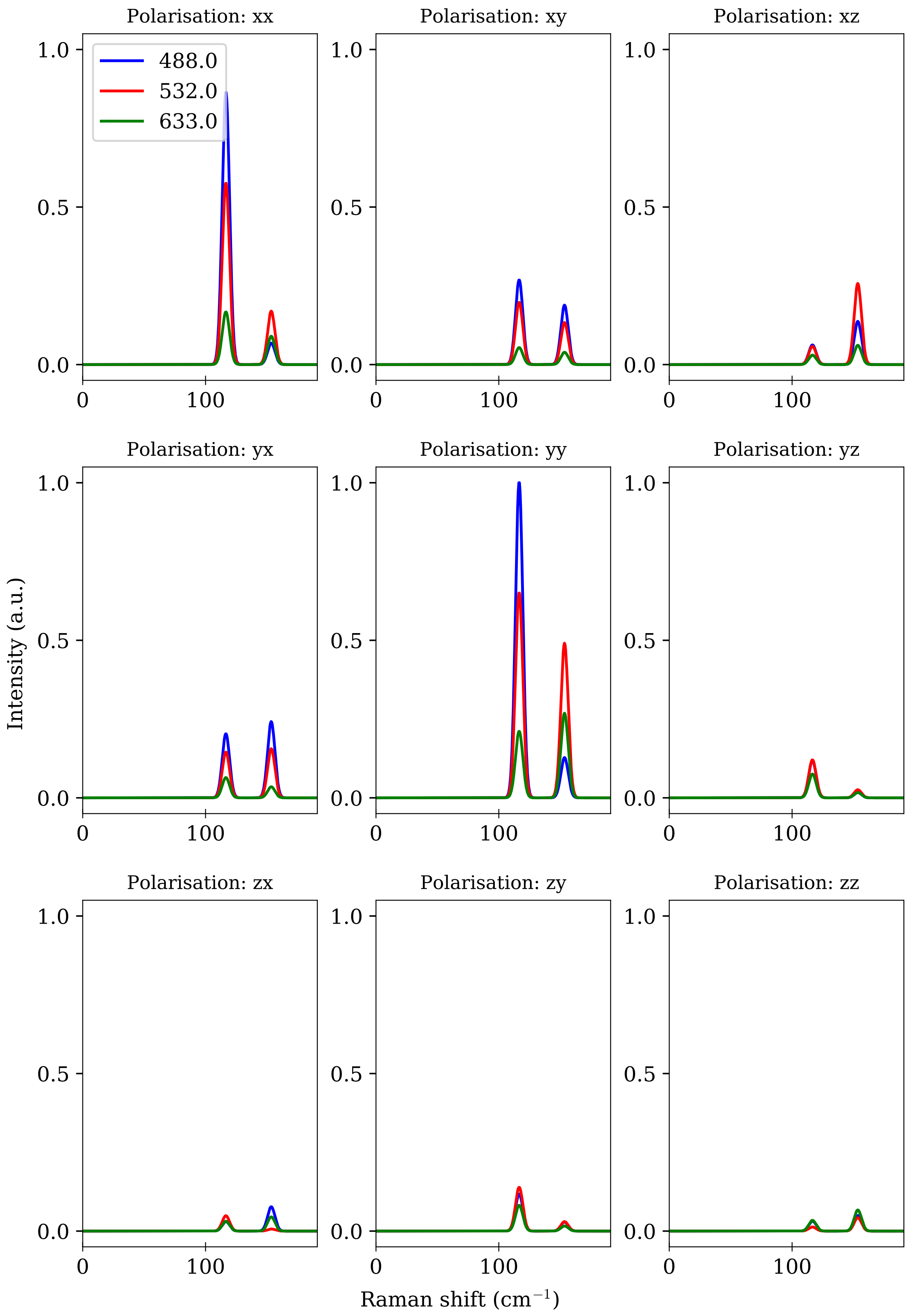

Supplementary Figure 430: Raman spectrum of PbTe with space group 156 and Wyckoff positions b-c-0.

$Pd_2Cl_2S_2$, 59, a-b-0

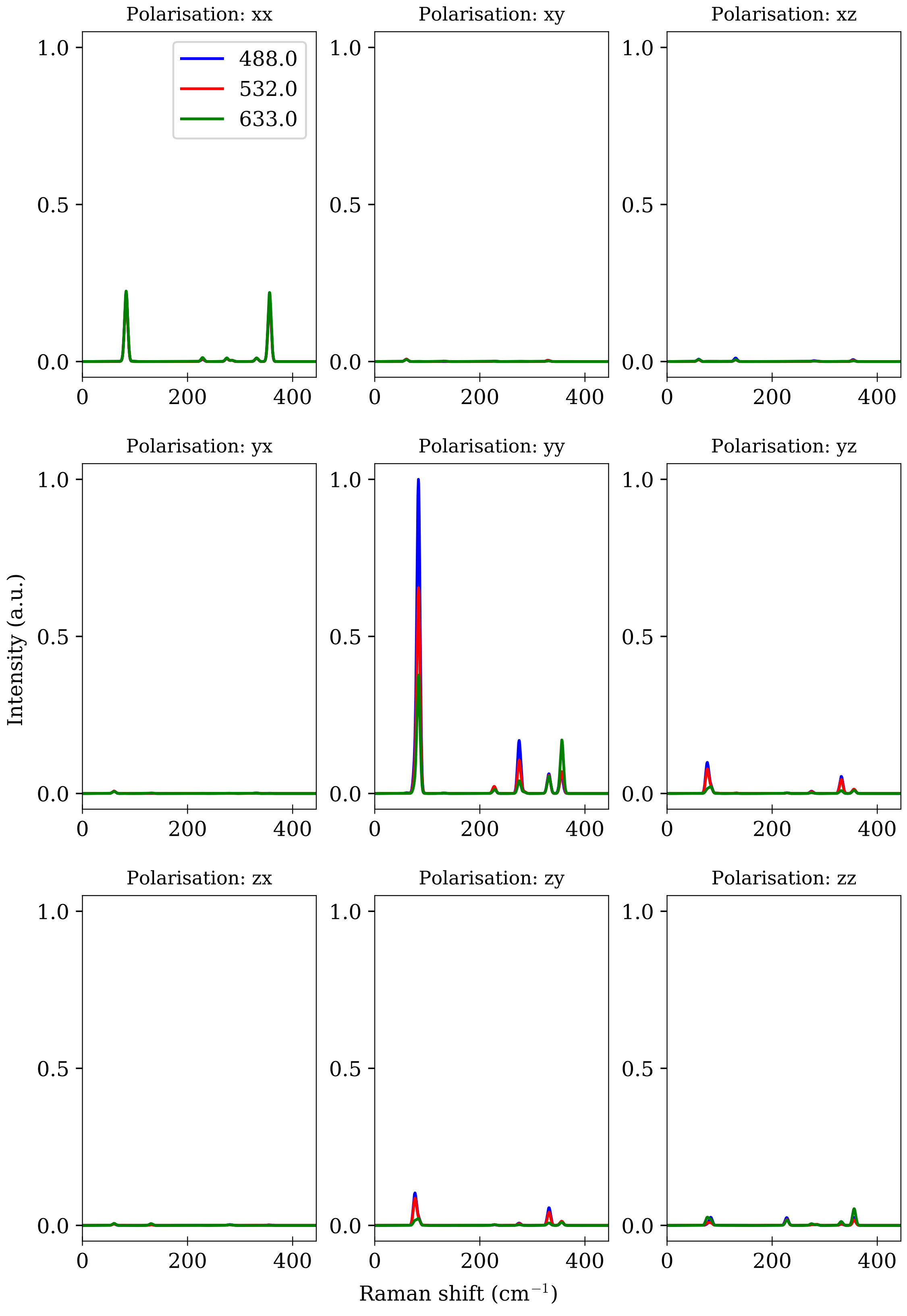

Supplementary Figure 431: Raman spectrum of $Pd_2Cl_2S_2$ with space group 59 and Wyckoff positions a-b-0.

Pd_2F_2, 2, i-0

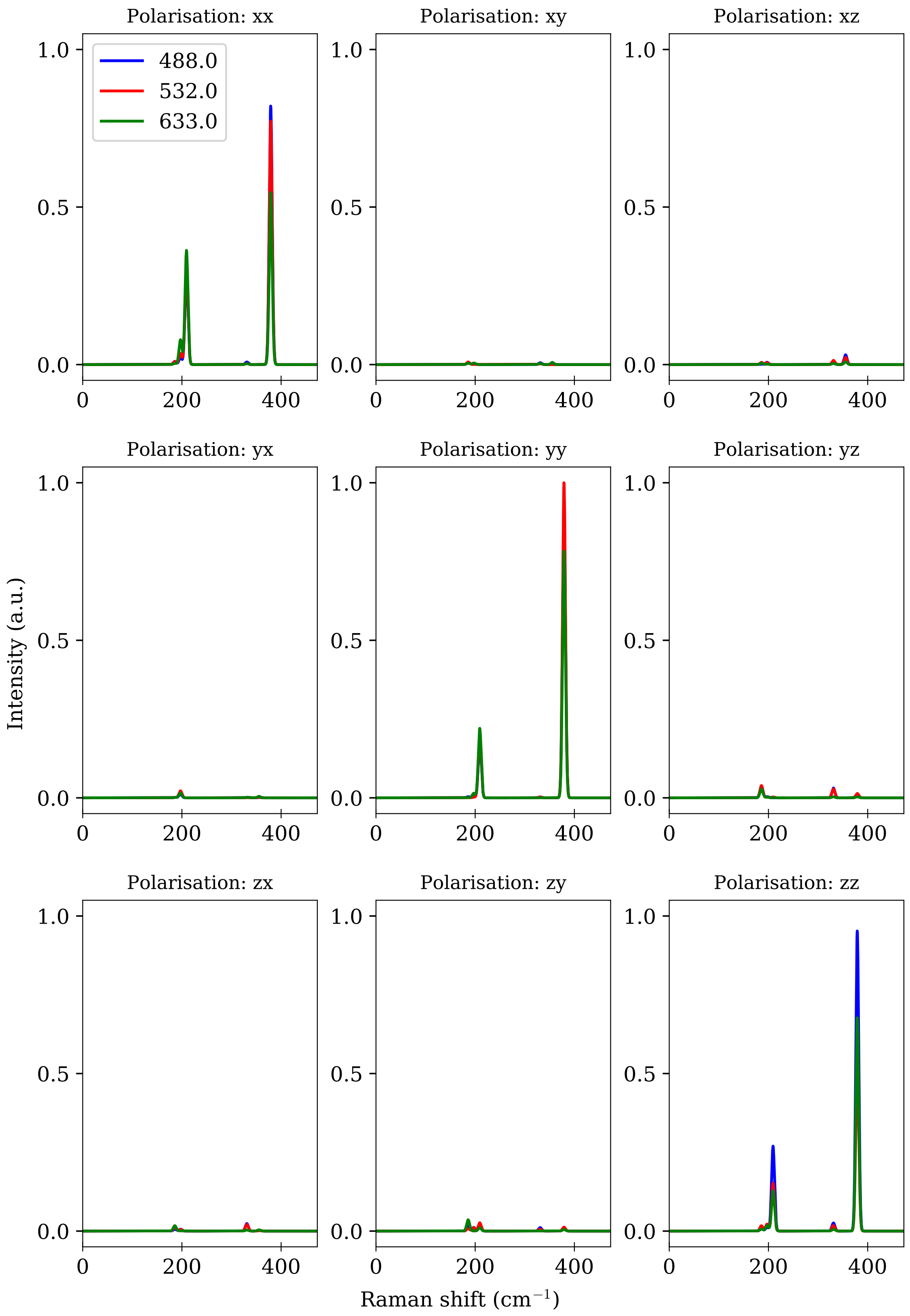

Supplementary Figure 432: Raman spectrum of Pd_2F_2 with space group 2 and Wyckoff positions i-0.

Pd_2I_2, 2, i-0

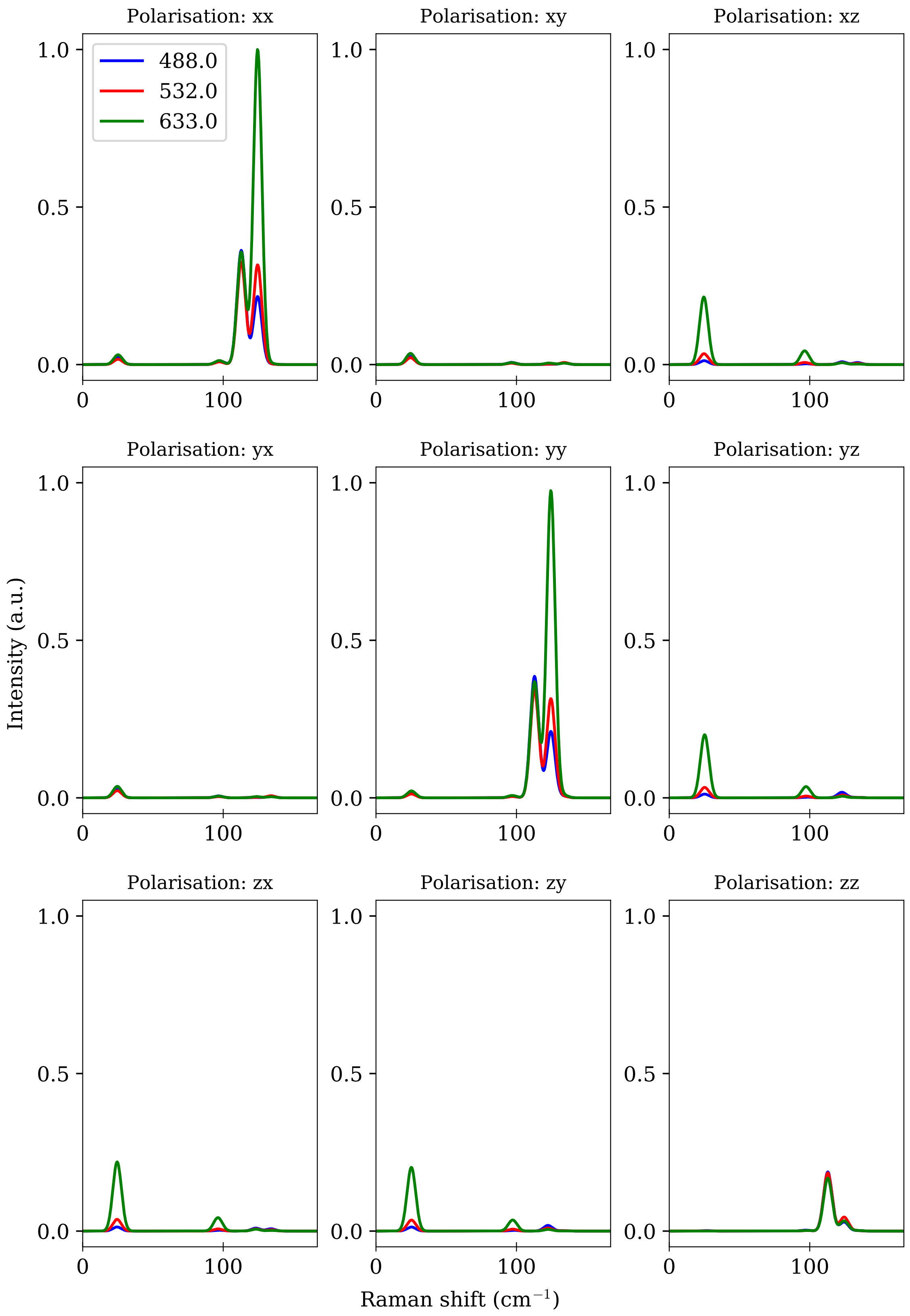

Supplementary Figure 433: Raman spectrum of Pd_2I_2 with space group 2 and Wyckoff positions i-0.

$Pd_2P_2S_6$, 2, i-0

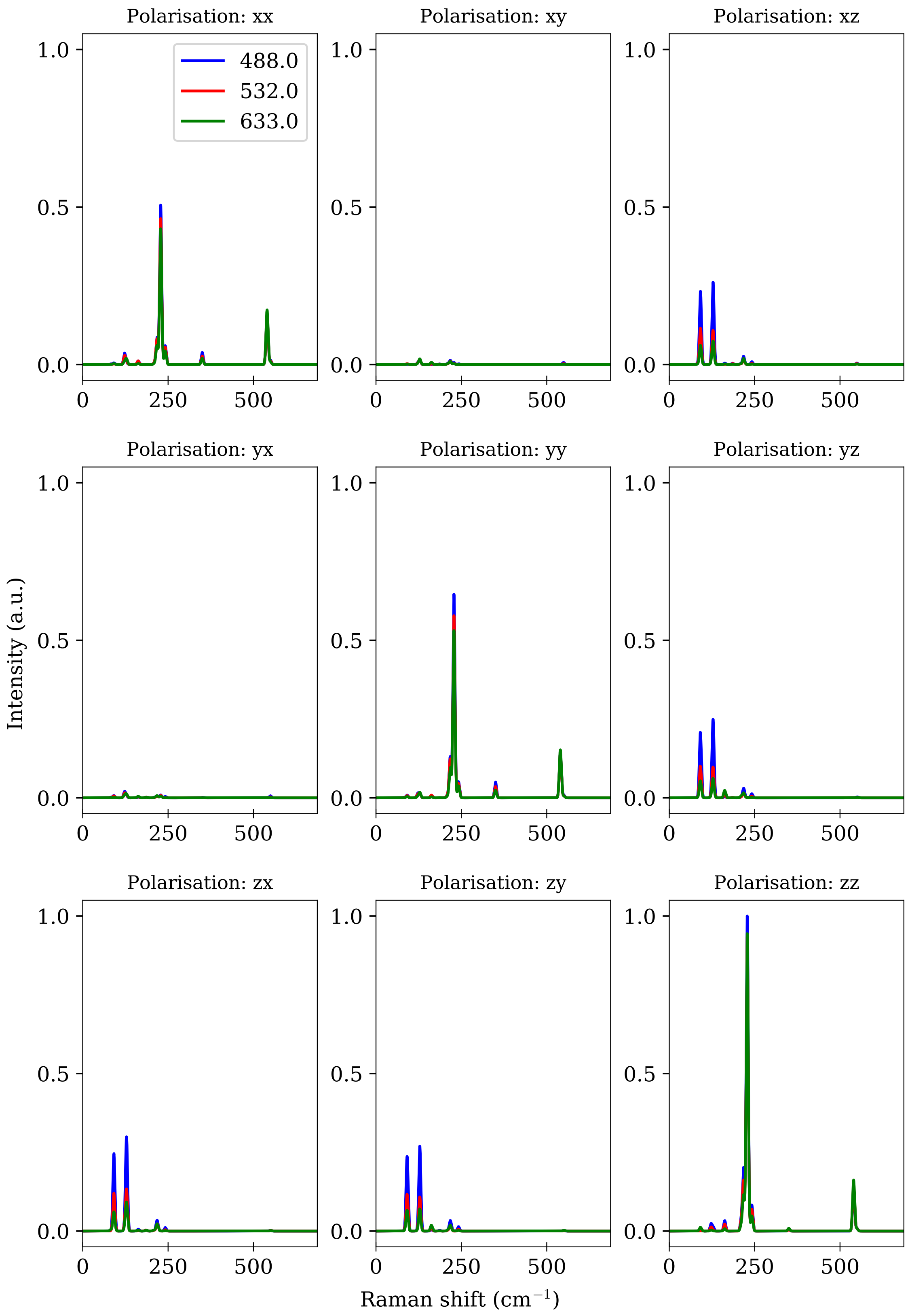

Supplementary Figure 434: Raman spectrum of $Pd_2P_2S_6$ with space group 2 and Wyckoff positions i-0.

Pd_2S_2, 12, i-0

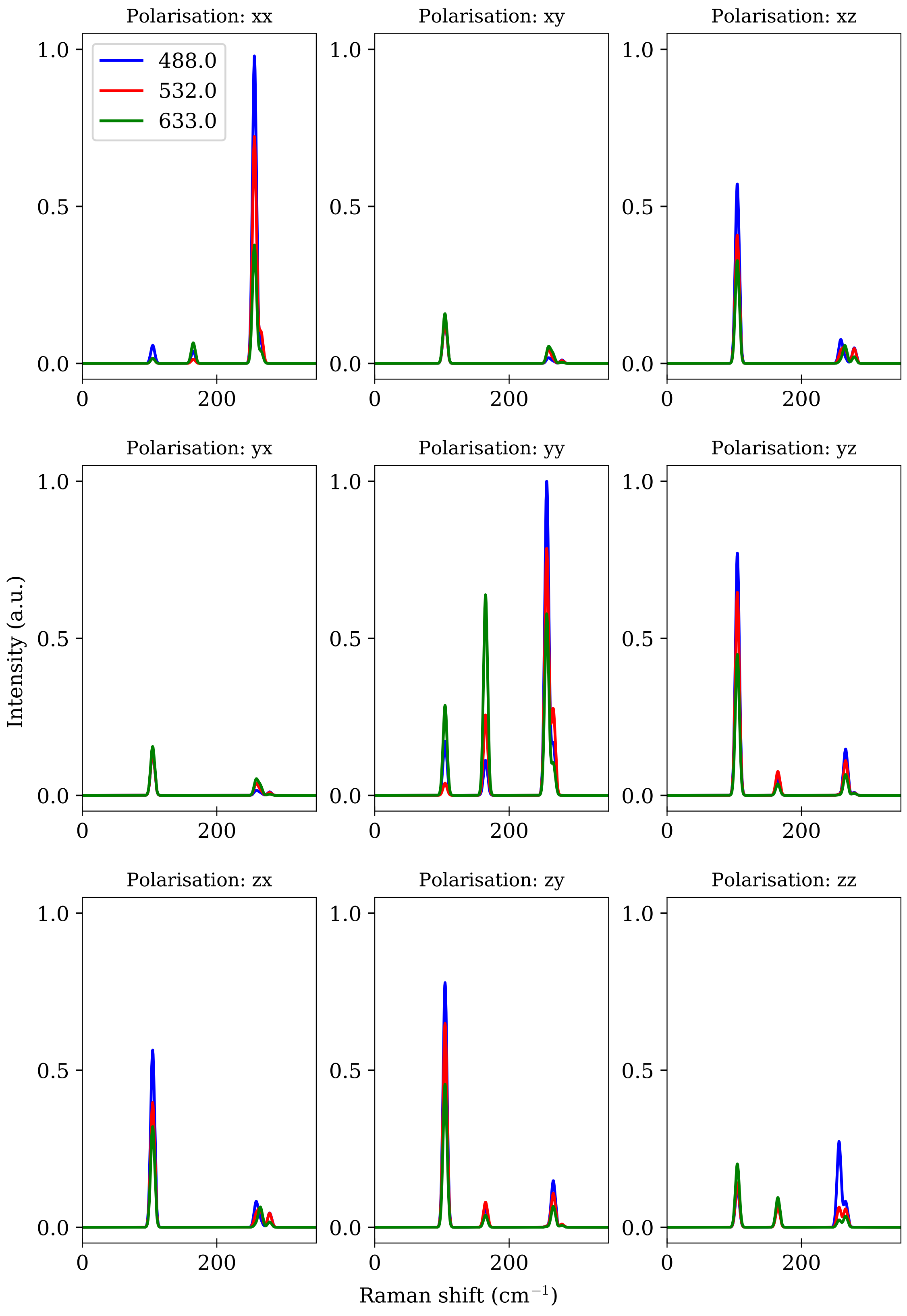

Supplementary Figure 435: Raman spectrum of Pd_2S_2 with space group 12 and Wyckoff positions i-0.

Pd_2S_4, 14, d-e-0

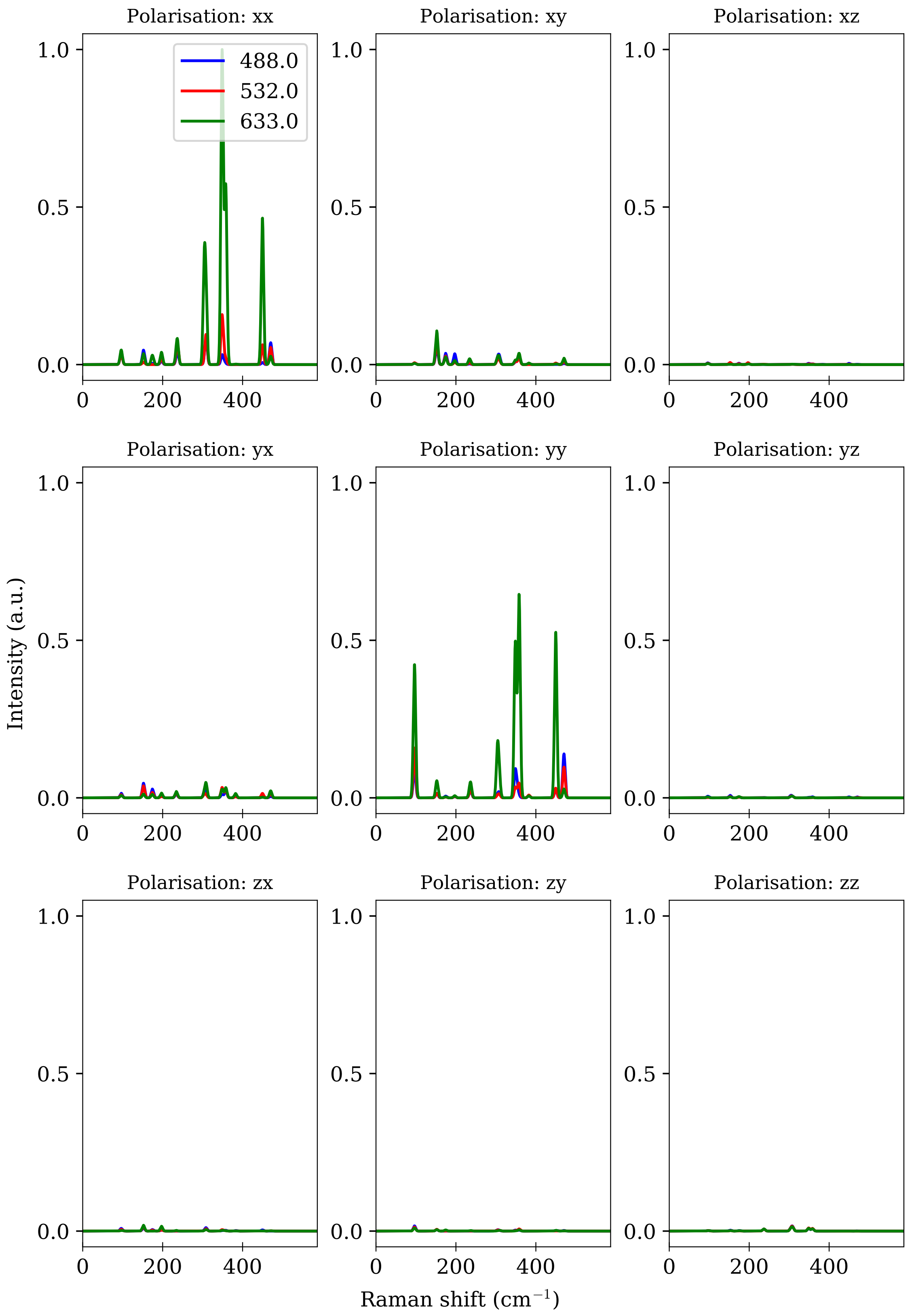

Supplementary Figure 436: Raman spectrum of Pd_2S_4 with space group 14 and Wyckoff positions d-e-0.

Pd_2Se_2, 12, i-0

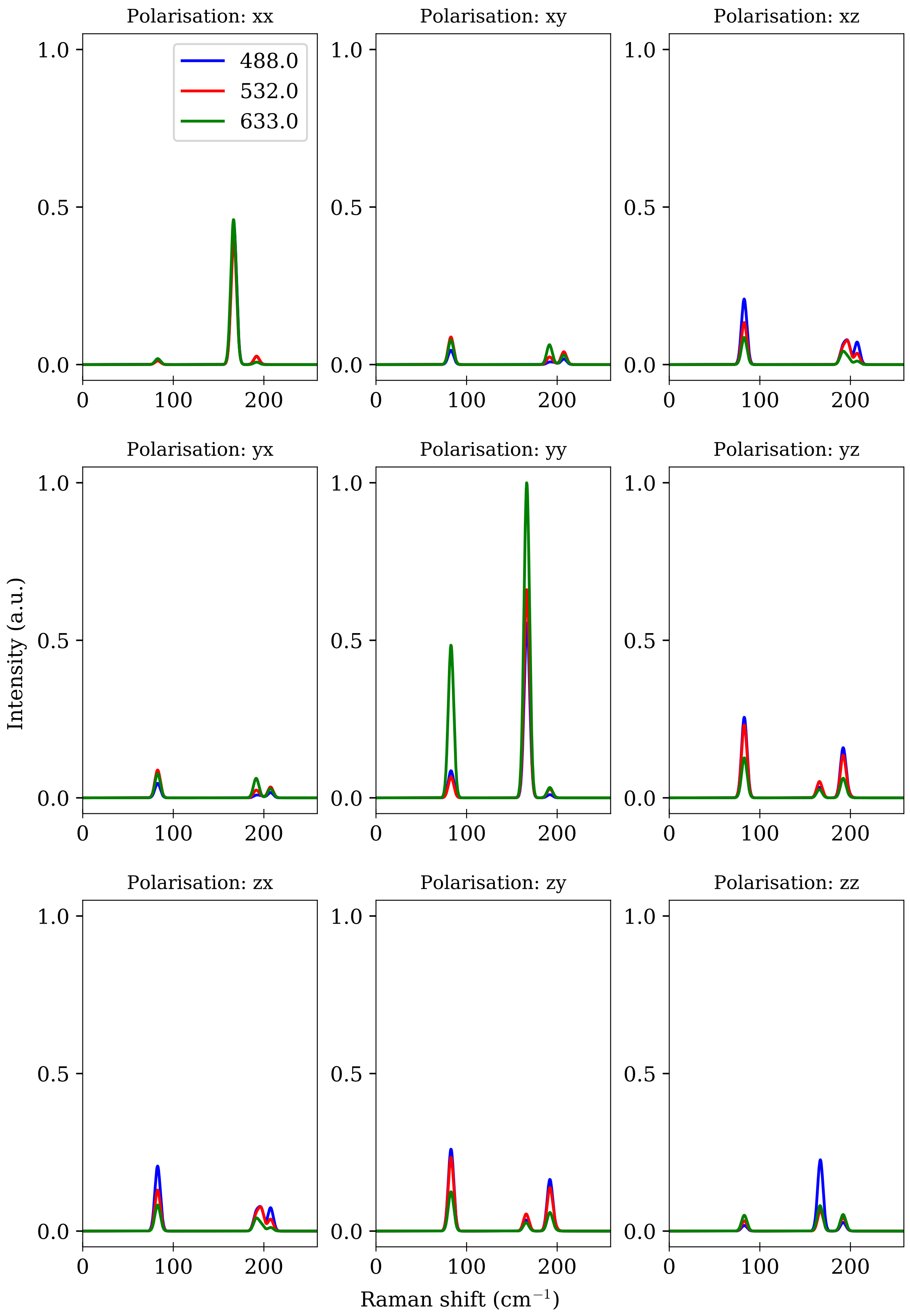

Supplementary Figure 437: Raman spectrum of Pd_2Se_2 with space group 12 and Wyckoff positions i-0.

Pd_2Se_4, 14, a-e-0

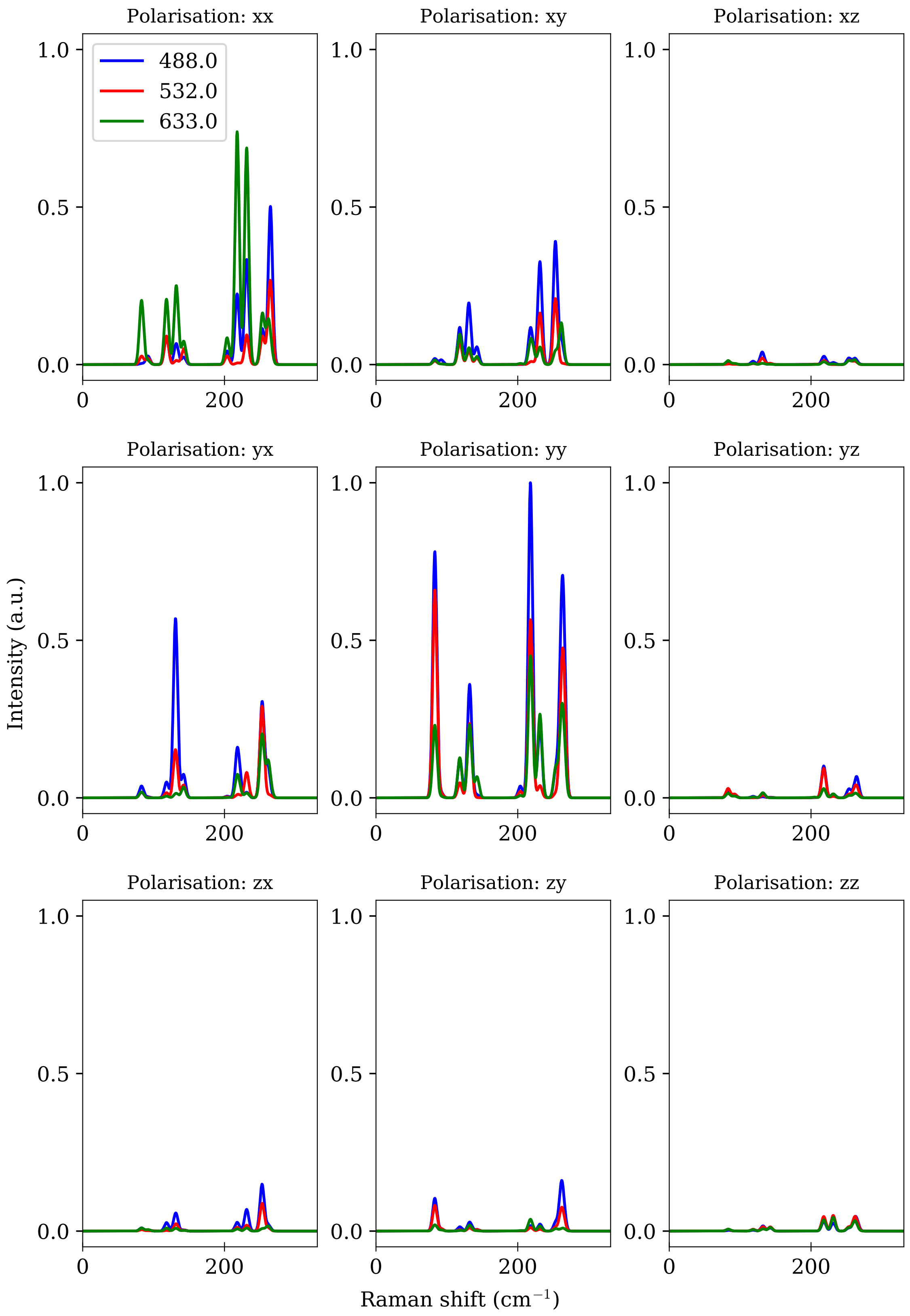

Supplementary Figure 438: Raman spectrum of Pd_2Se_4 with space group 14 and Wyckoff positions a-e-0.

Pd_2Te_2, 12, i-0

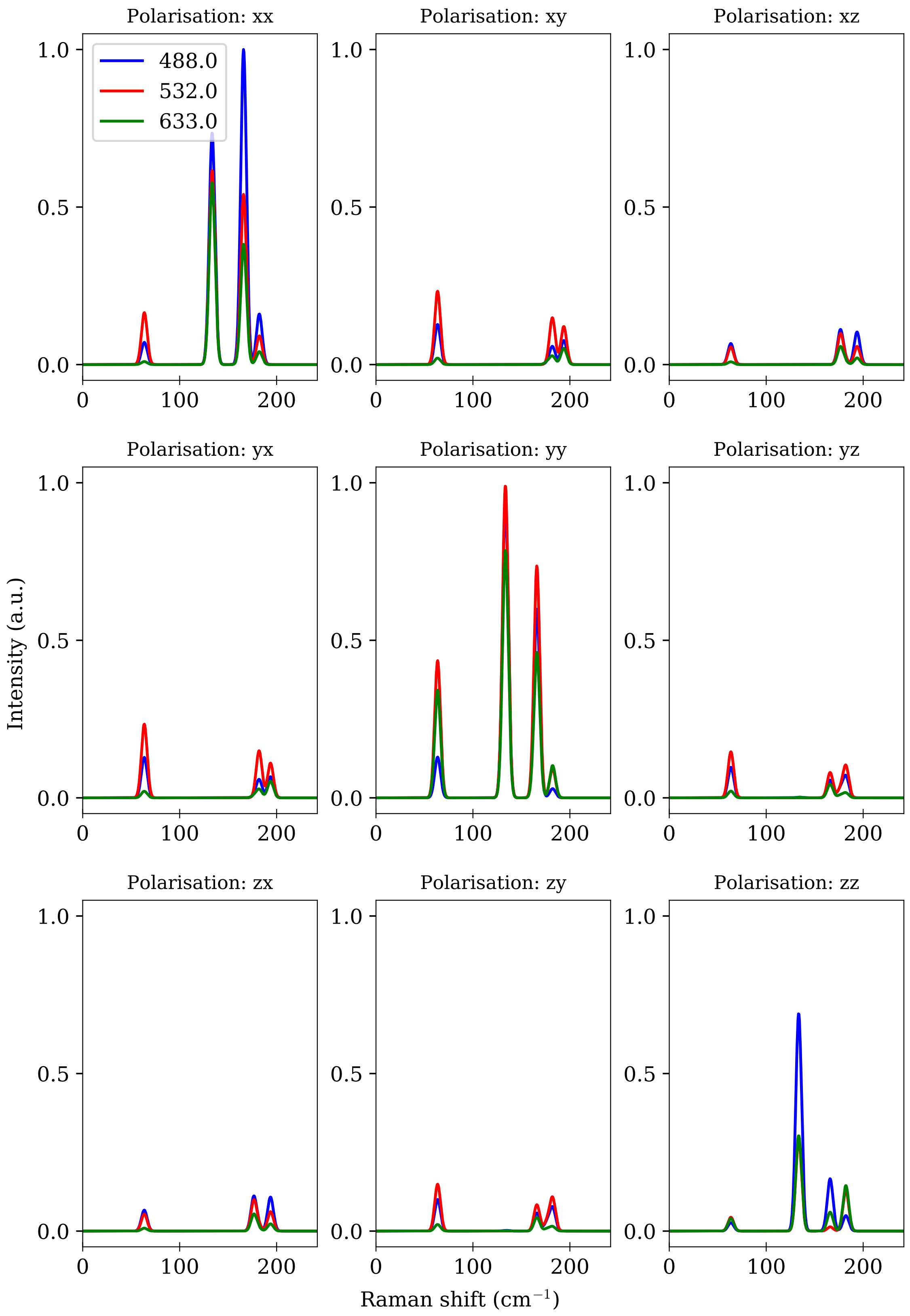

Supplementary Figure 439: Raman spectrum of Pd_2Te_2 with space group 12 and Wyckoff positions i-0.

Pd_2Te_4, 14, d-e-0

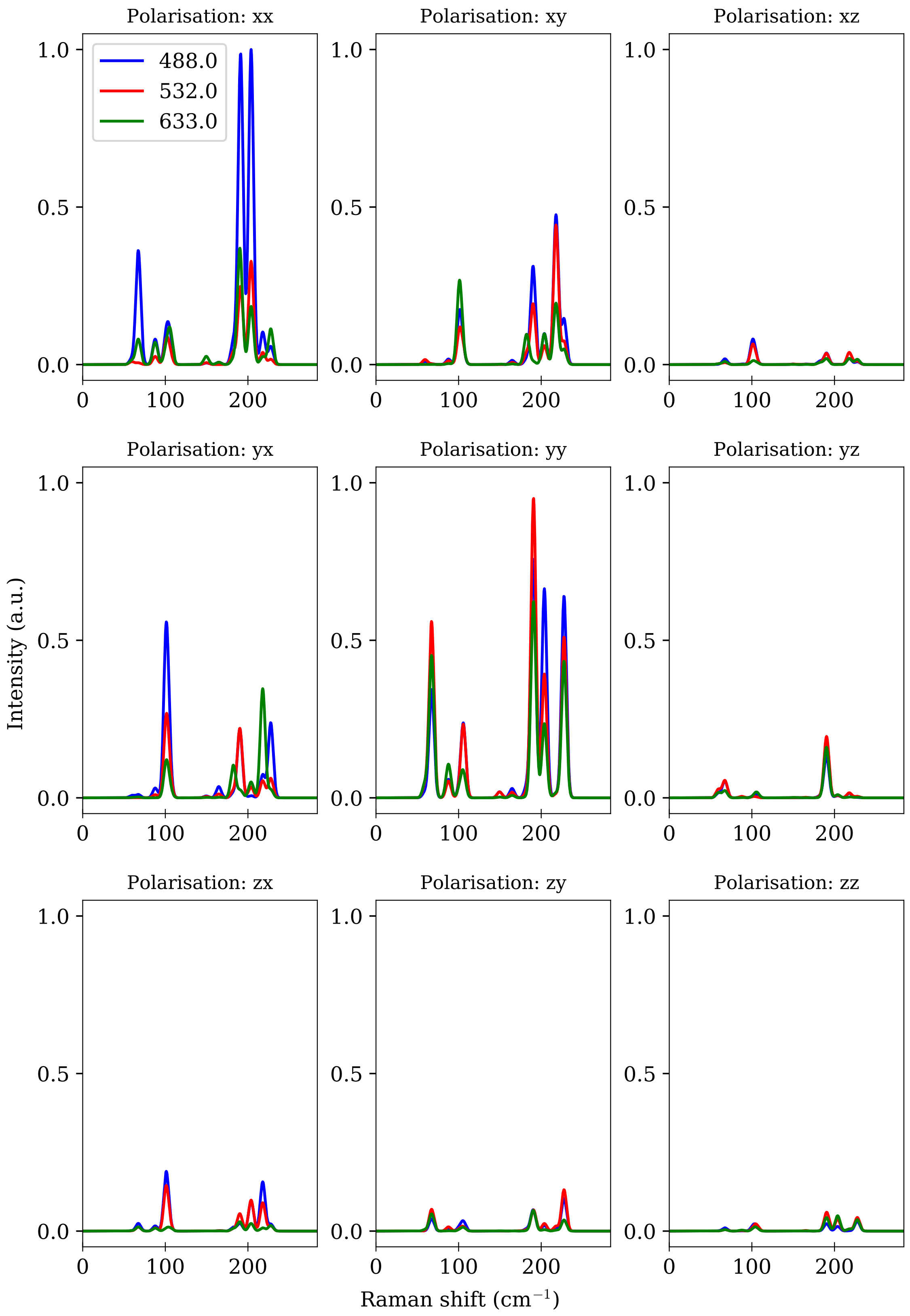

Supplementary Figure 440: Raman spectrum of Pd_2Te_4 with space group 14 and Wyckoff positions d-e-0.

PdO_2, 164, b-d-0

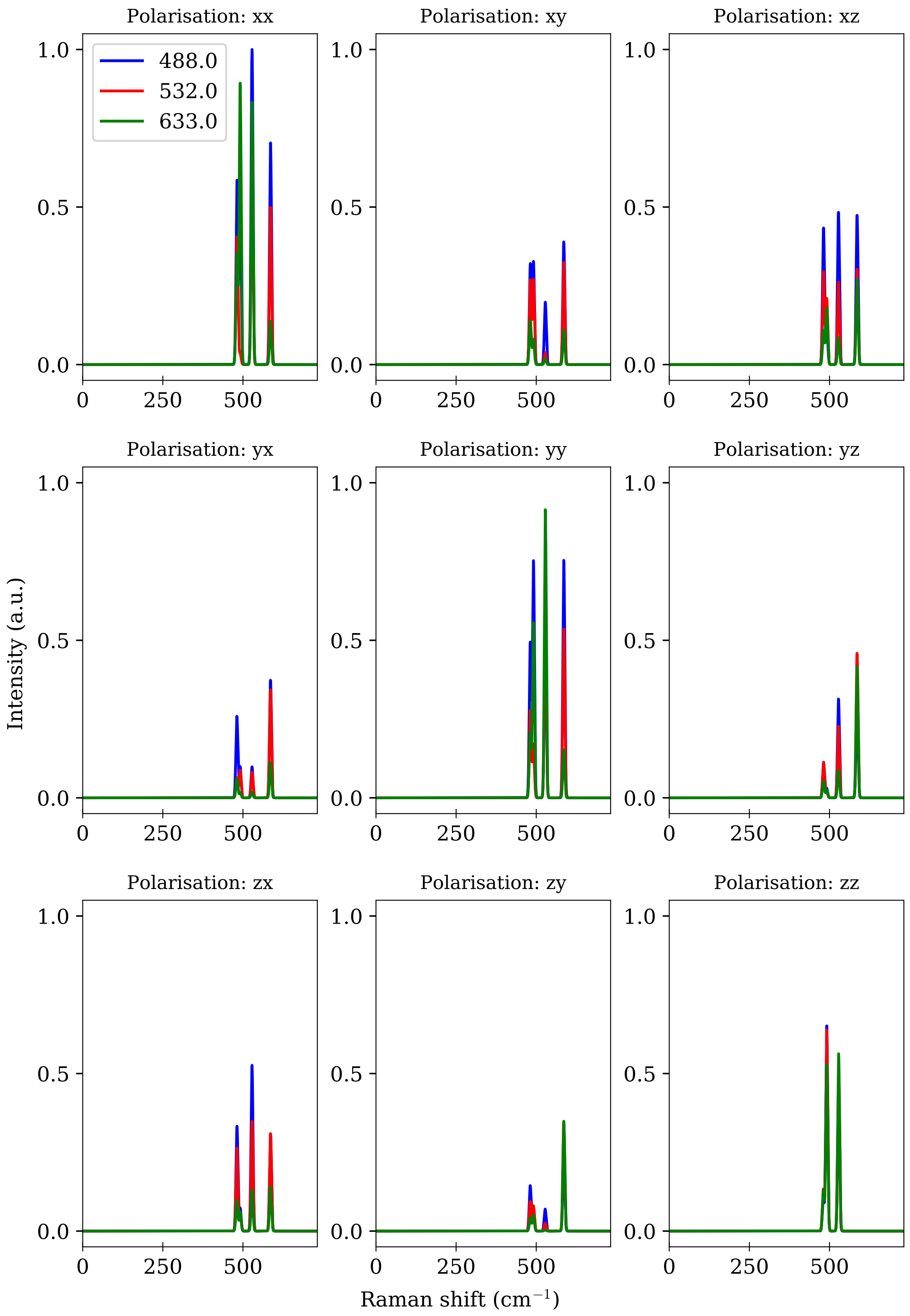

Supplementary Figure 441: Raman spectrum of PdO_2 with space group 164 and Wyckoff positions b-d-0.

PdS_2, 164, b-d-0

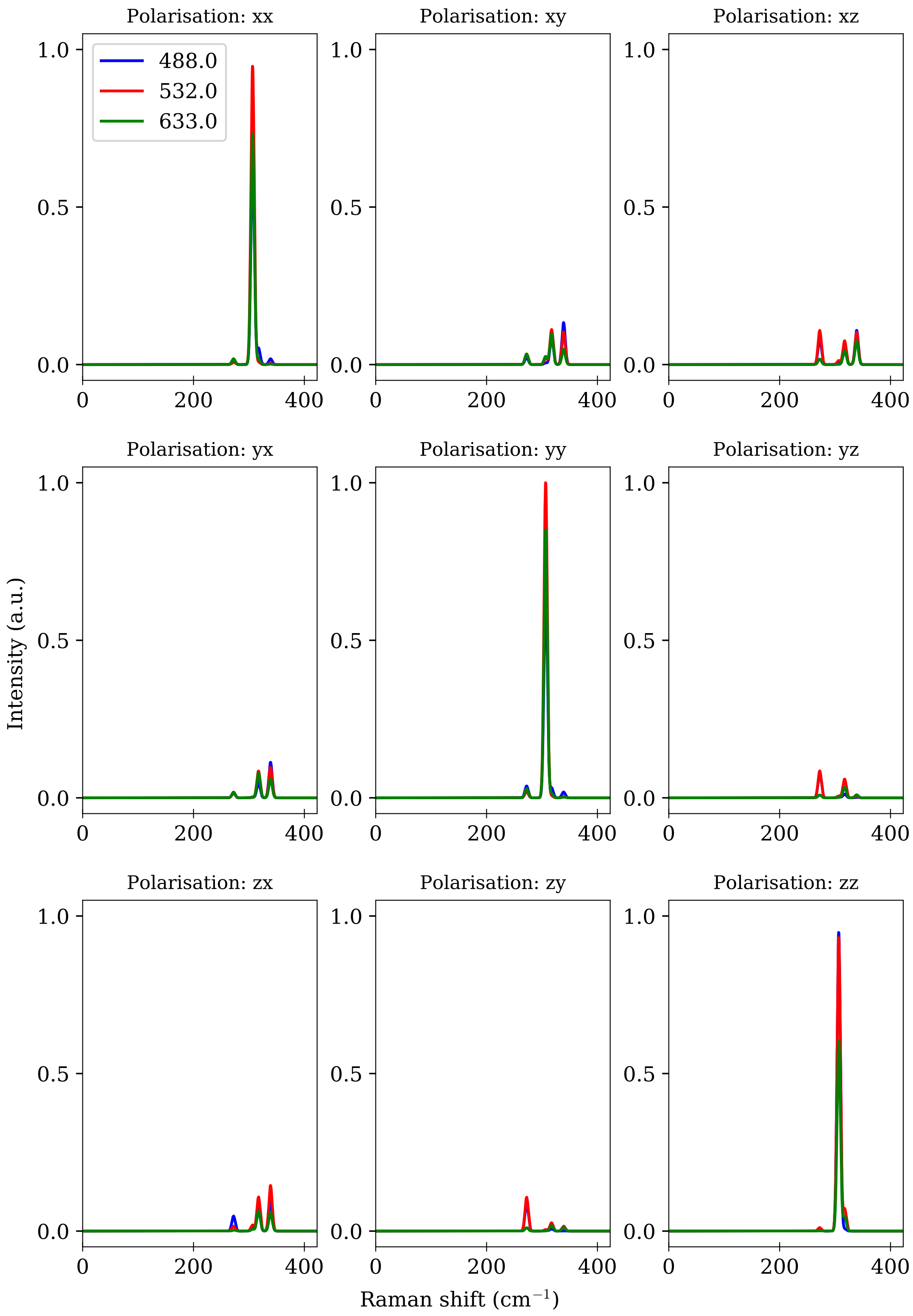

Supplementary Figure 442: Raman spectrum of PdS_2 with space group 164 and Wyckoff positions b-d-0.

$PdSe_2$, 164, b-d-0

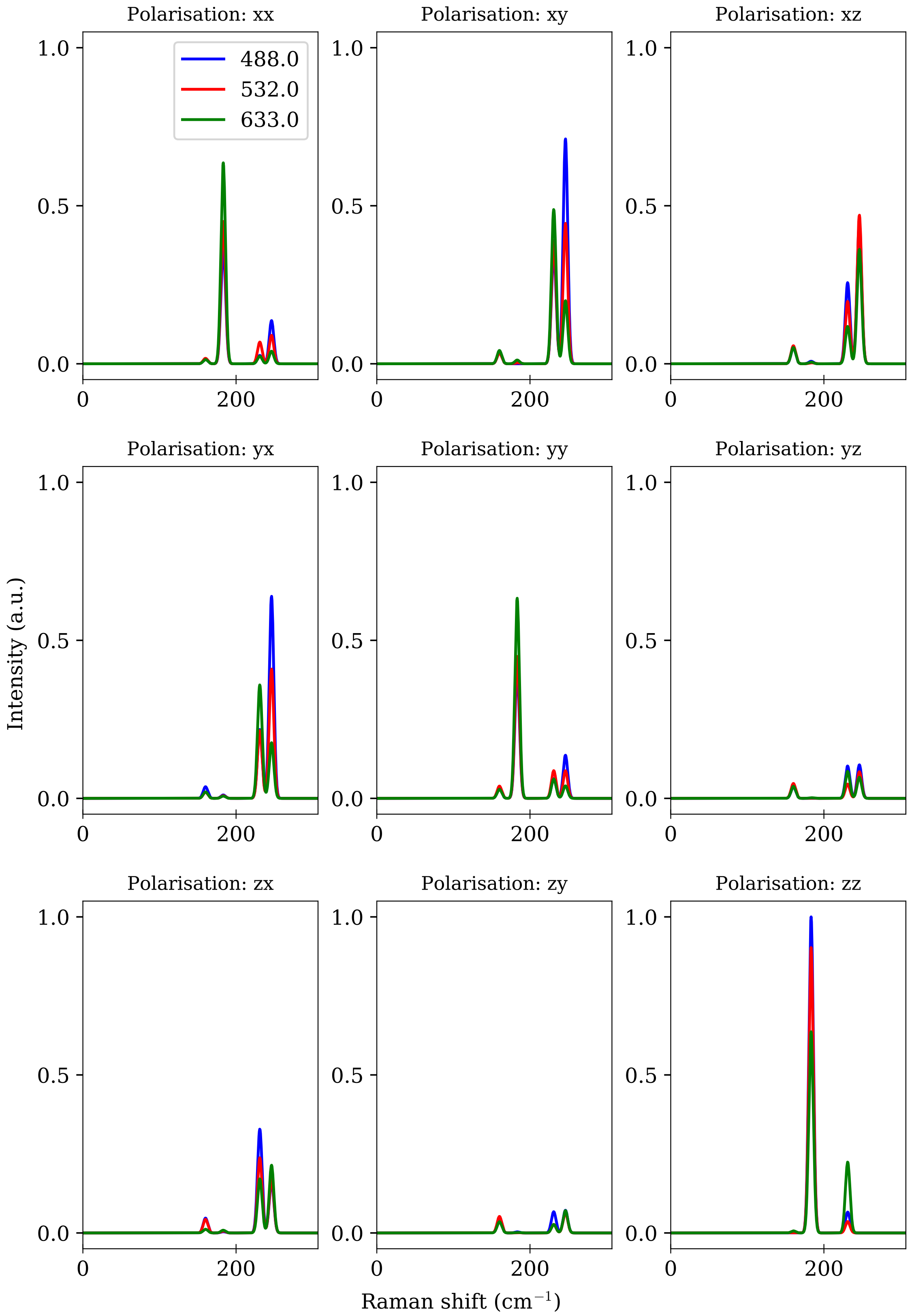

Supplementary Figure 443: Raman spectrum of $PdSe_2$ with space group 164 and Wyckoff positions b-d-0.

$PdSe_2$, 187, b-i-0

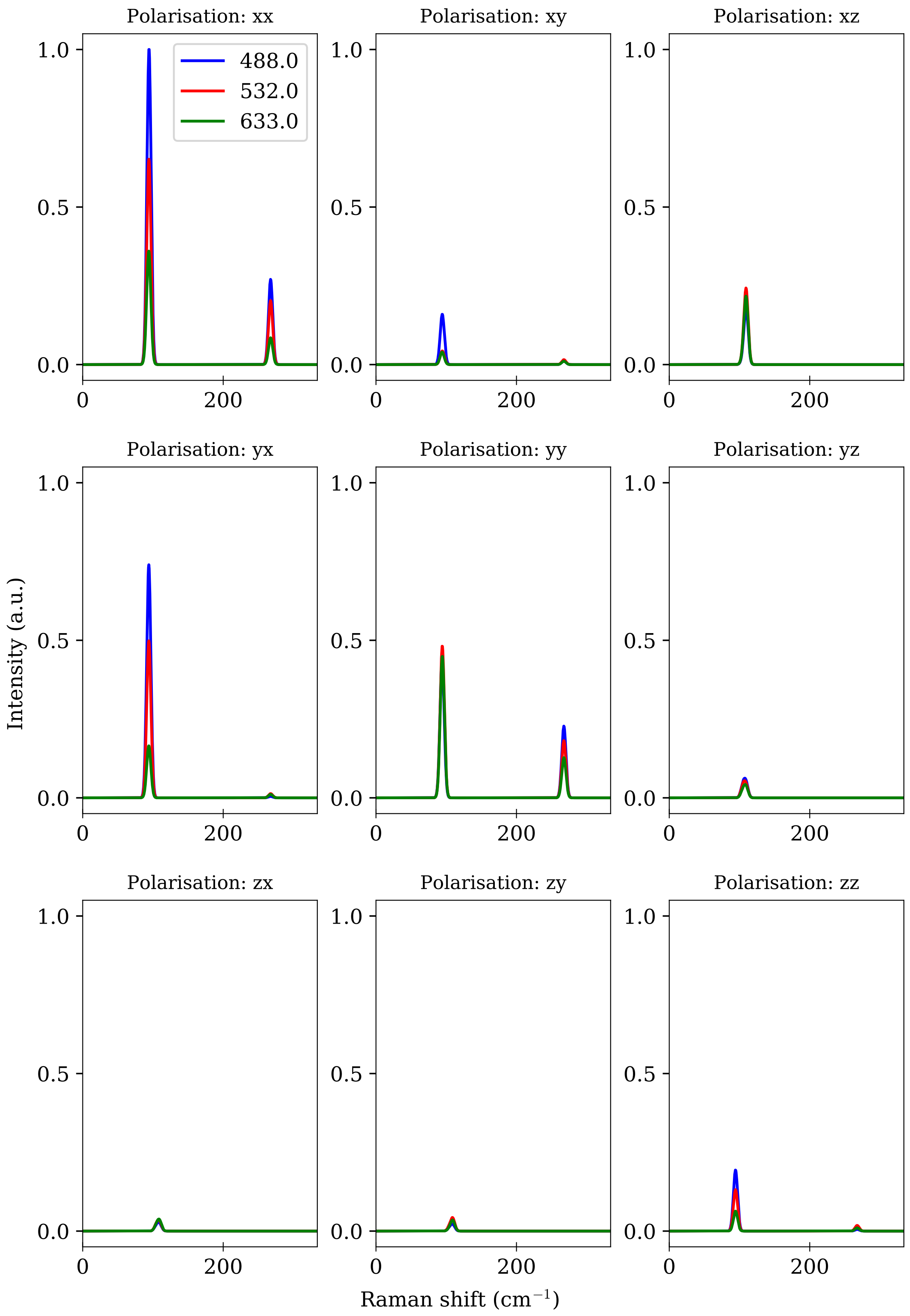

Supplementary Figure 444: Raman spectrum of $PdSe_2$ with space group 187 and Wyckoff positions b-i-0.

$PdTe_2$, 164, b-d-0

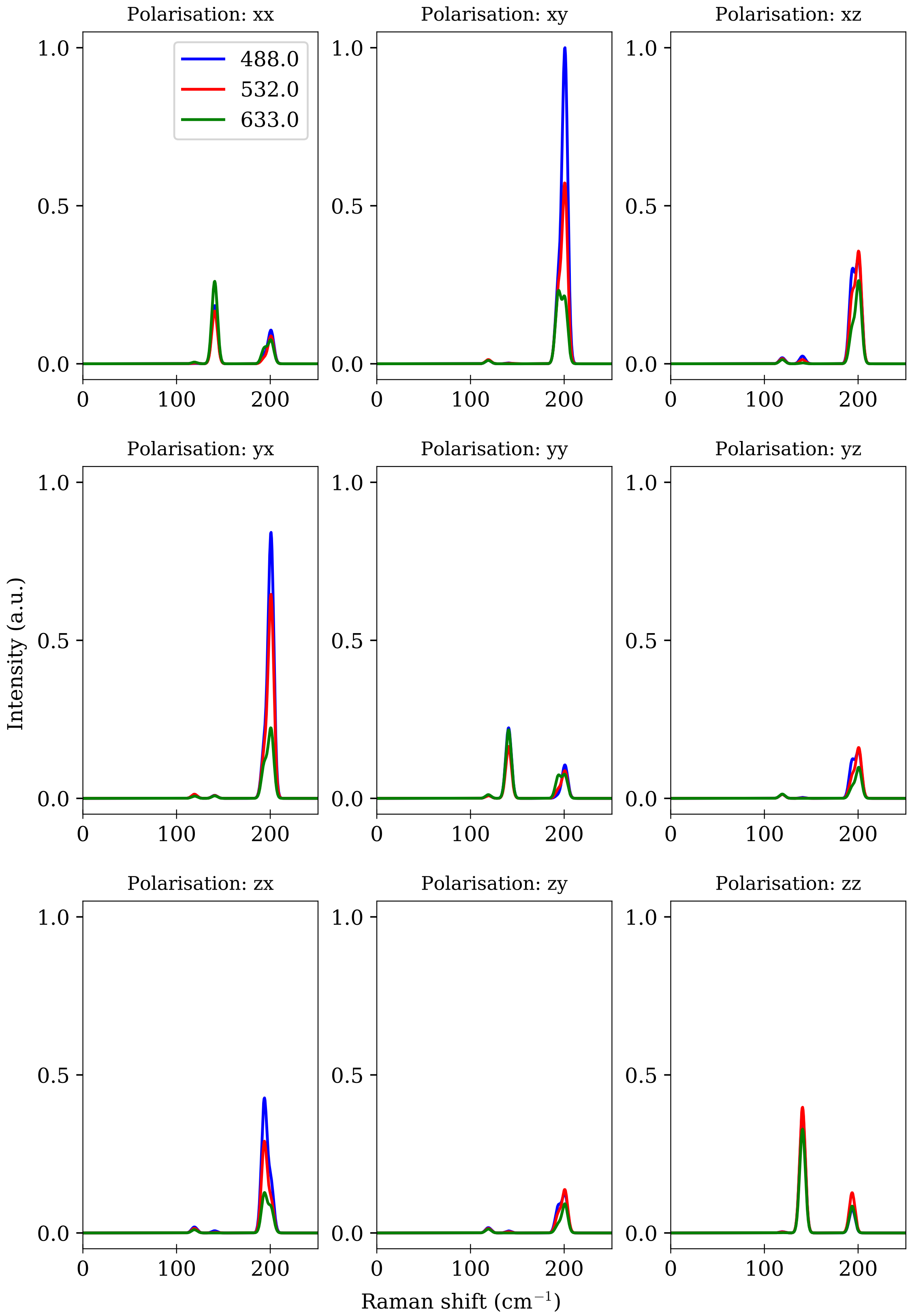

Supplementary Figure 445: Raman spectrum of $PdTe_2$ with space group 164 and Wyckoff positions b-d-0.

$PdTe_2$, 187, b-i-0

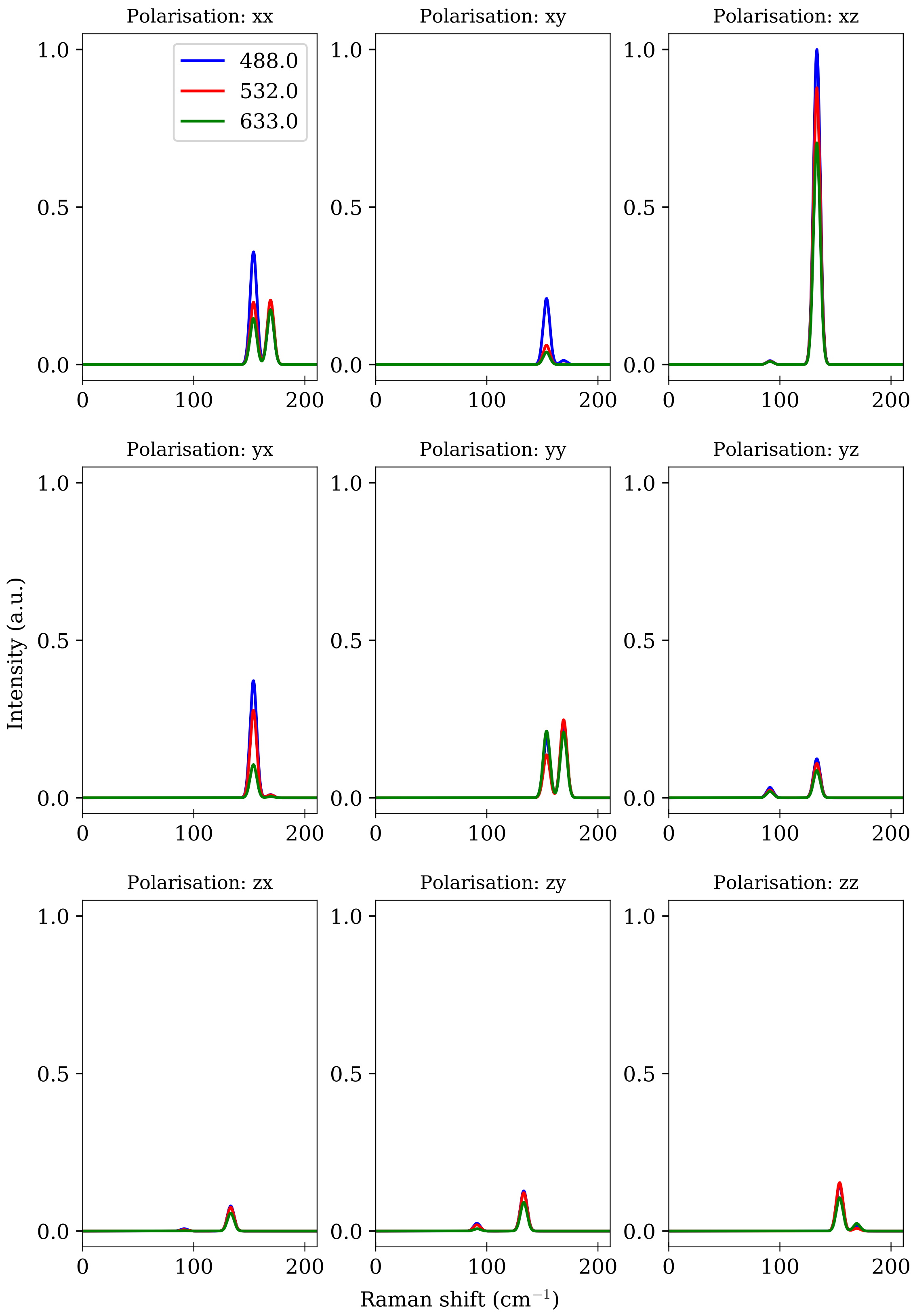

Supplementary Figure 446: Raman spectrum of $PdTe_2$ with space group 187 and Wyckoff positions b-i-0.

Pt_2Cl_2, 1, a-0

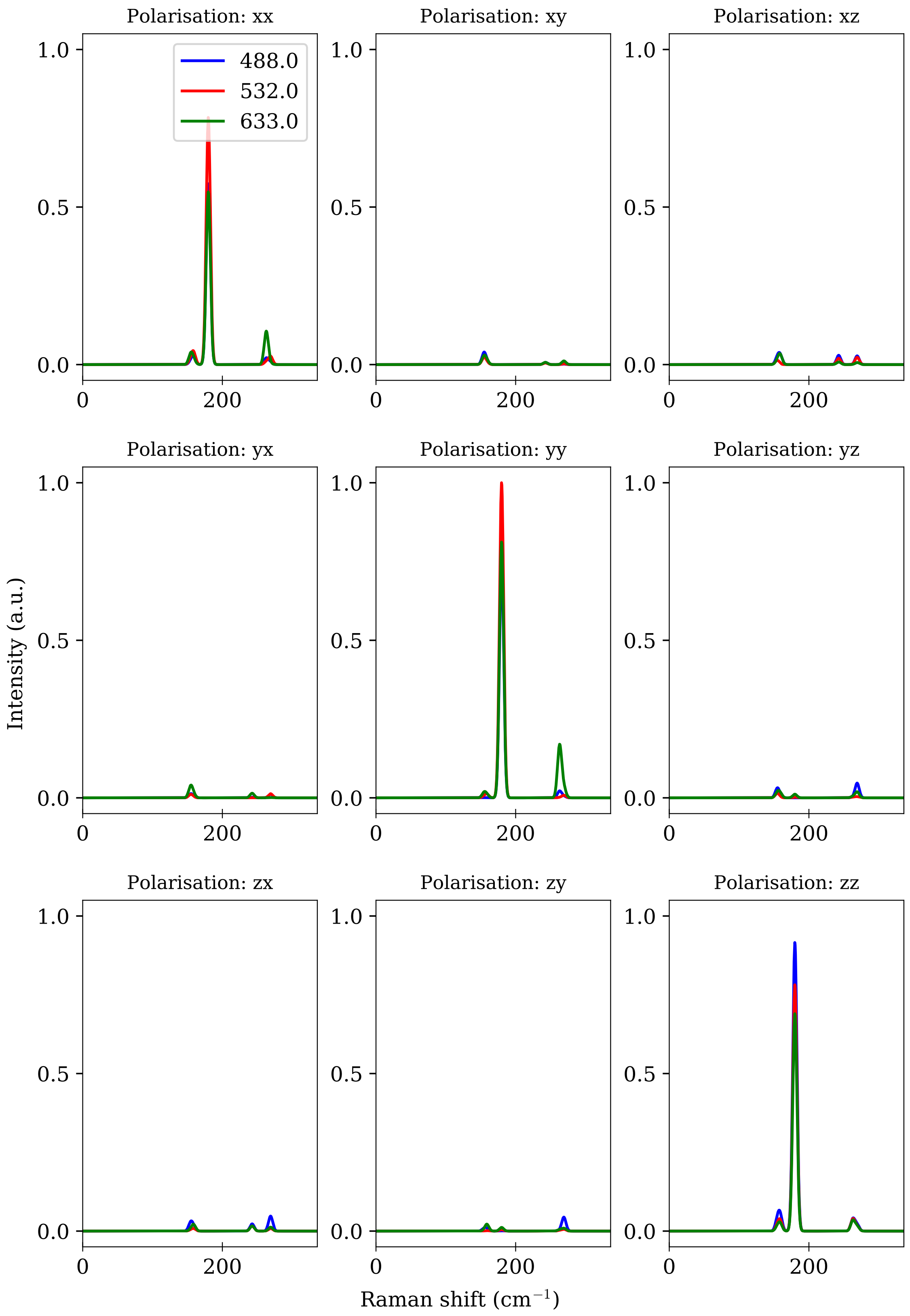

Supplementary Figure 447: Raman spectrum of Pt_2Cl_2 with space group 1 and Wyckoff positions a-0.

Pt_2O_2, 10, b-d-m-0

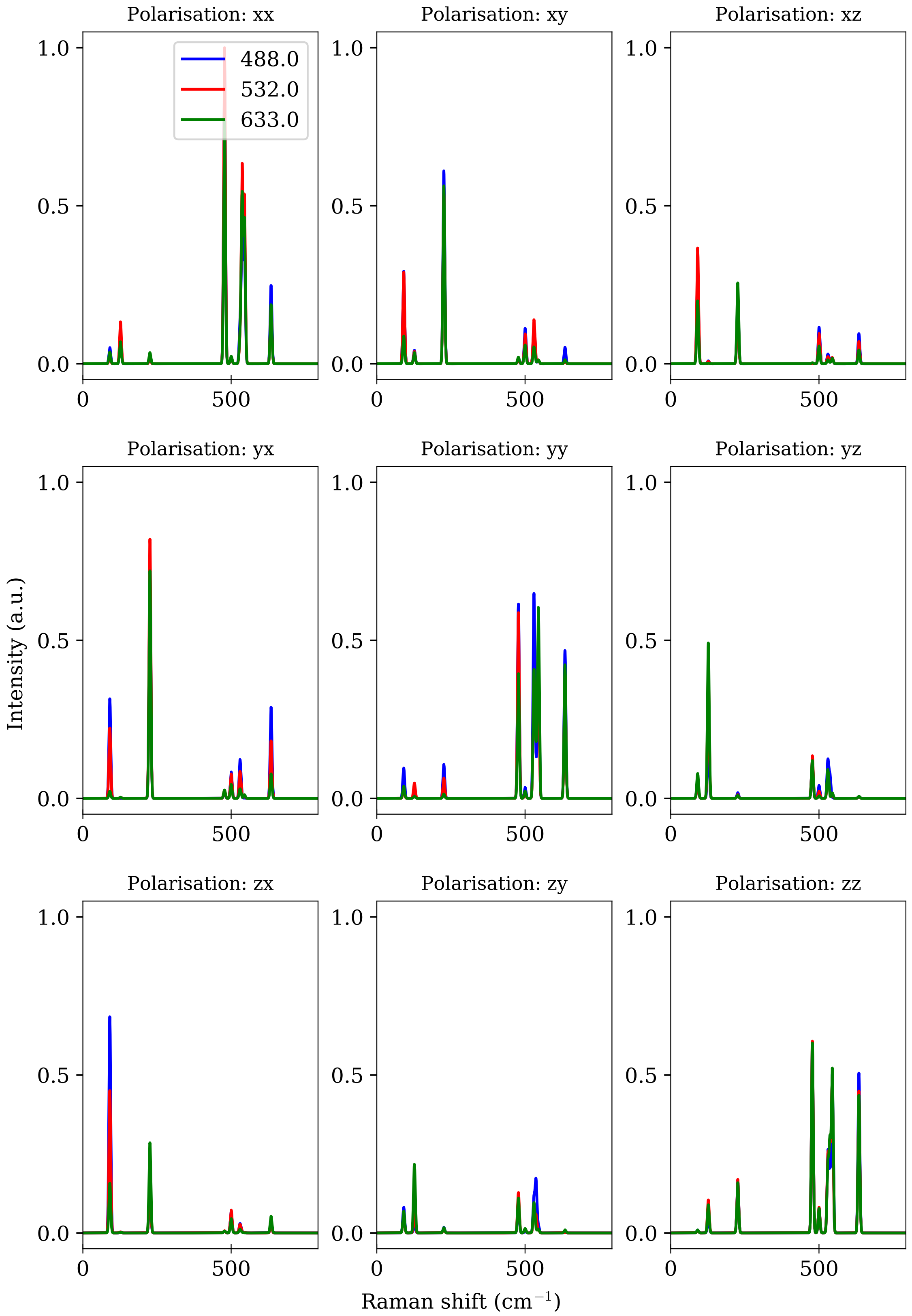

Supplementary Figure 448: Raman spectrum of Pt_2O_2 with space group 10 and Wyckoff positions b-d-m-0.

Pt_2O_2, 129, b-c-0

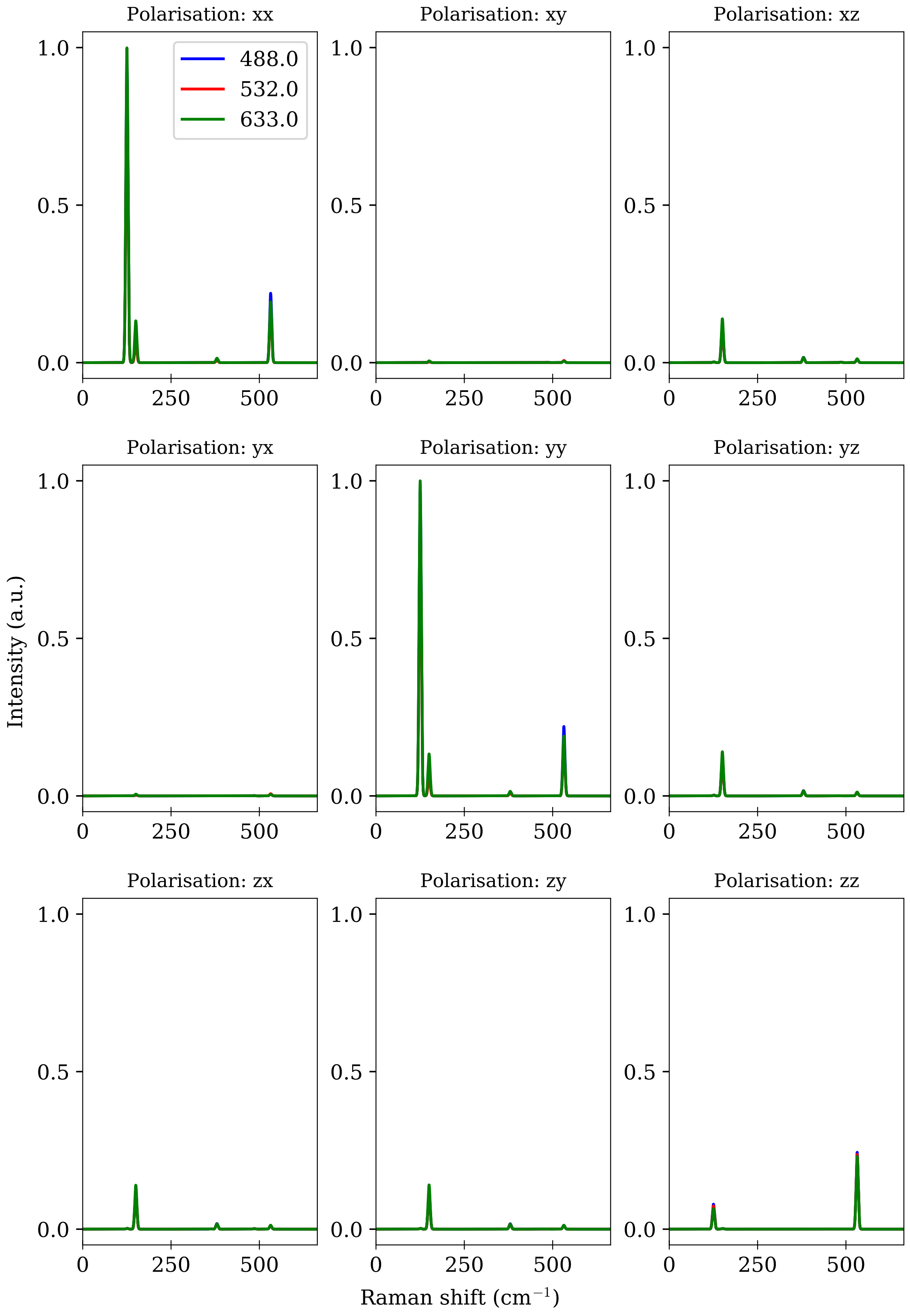

Supplementary Figure 449: Raman spectrum of Pt_2O_2 with space group 129 and Wyckoff positions b-c-0.

Pt_2S_4, 14, d-e-0

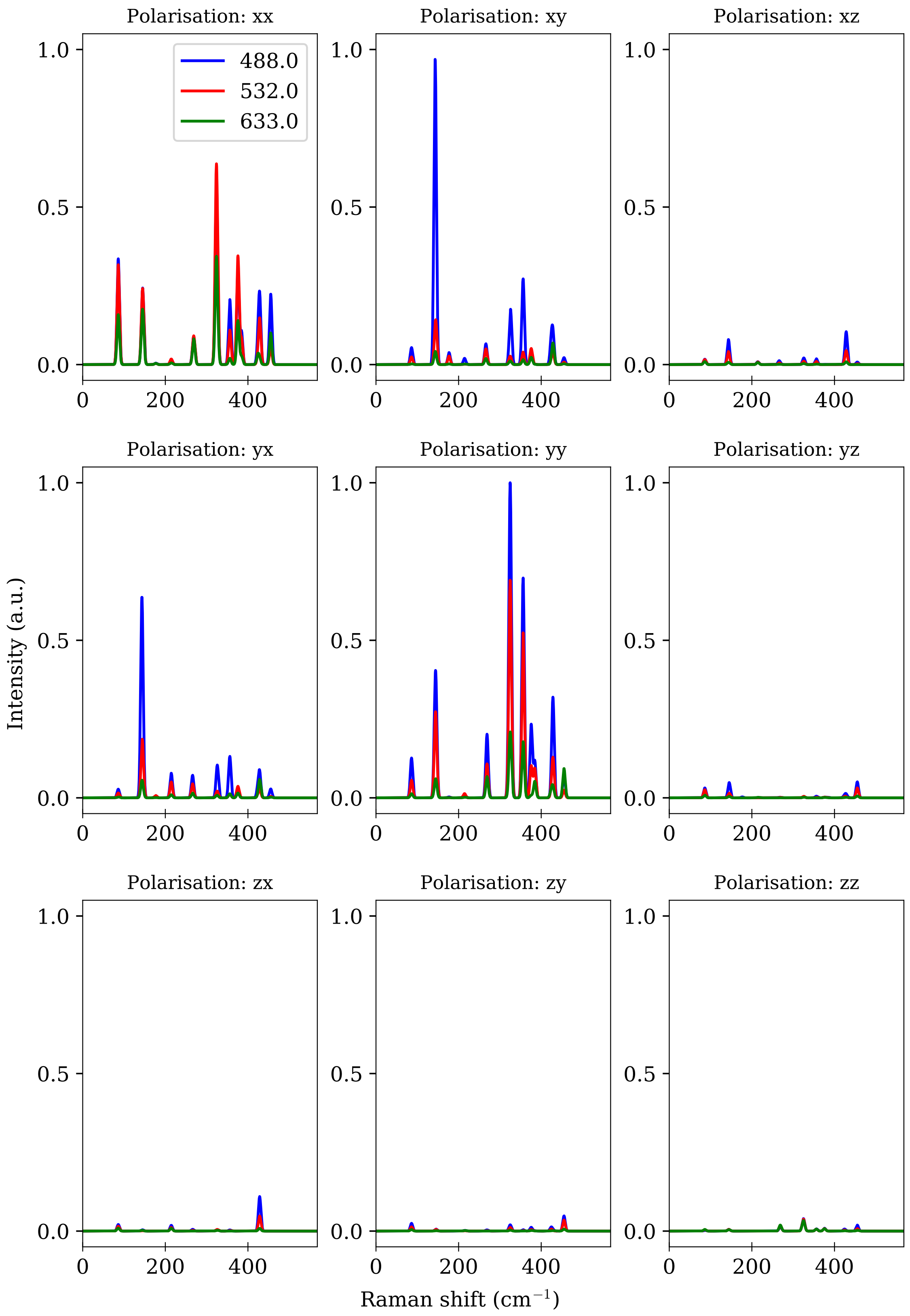

Supplementary Figure 450: Raman spectrum of Pt_2S_4 with space group 14 and Wyckoff positions d-e-0.

Pt_2Se_2, 25, b-d-g-0

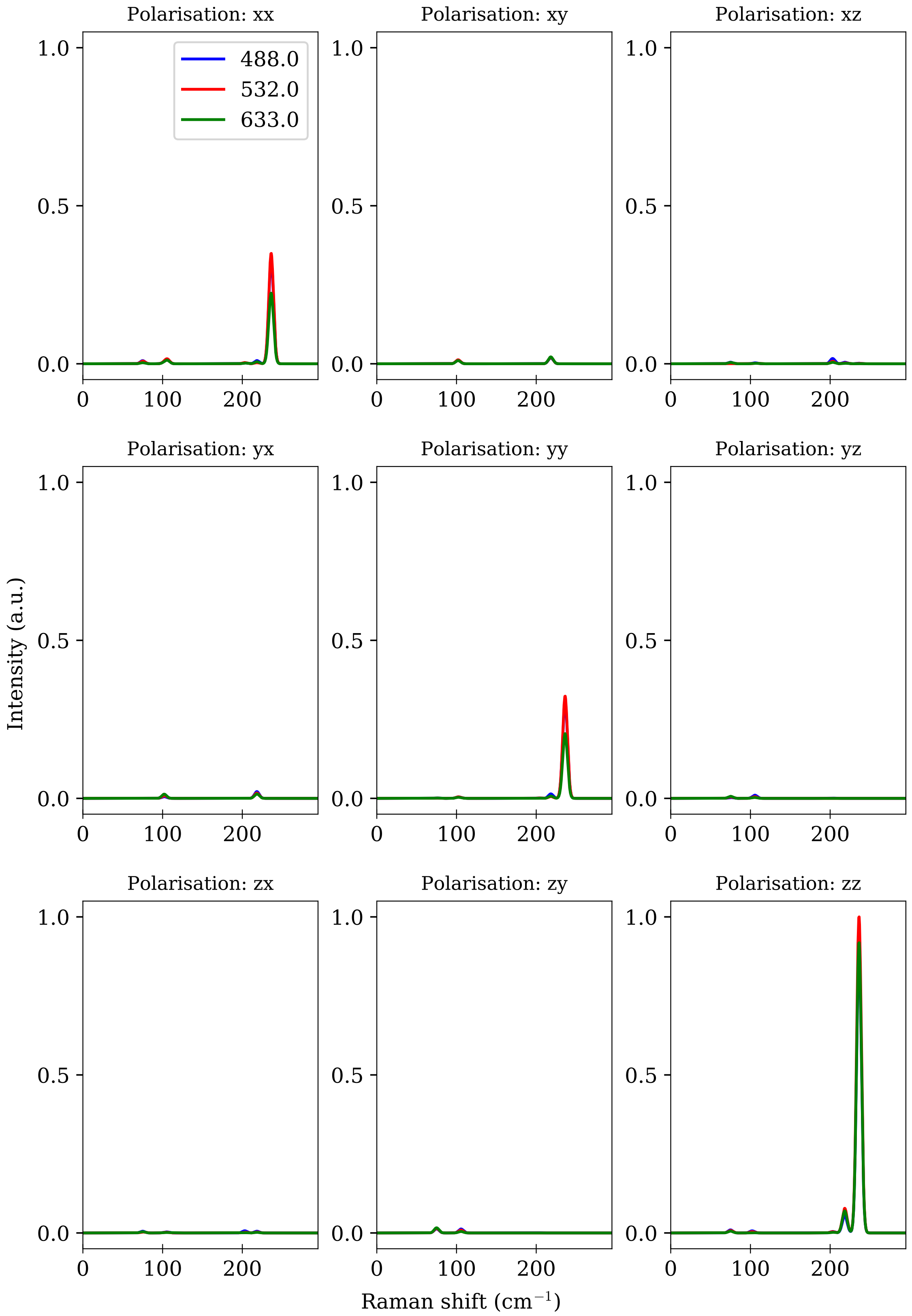

Supplementary Figure 451: Raman spectrum of Pt_2Se_2 with space group 25 and Wyckoff positions b-d-g-0.

Pt_2Se_4, 14, c-e-0

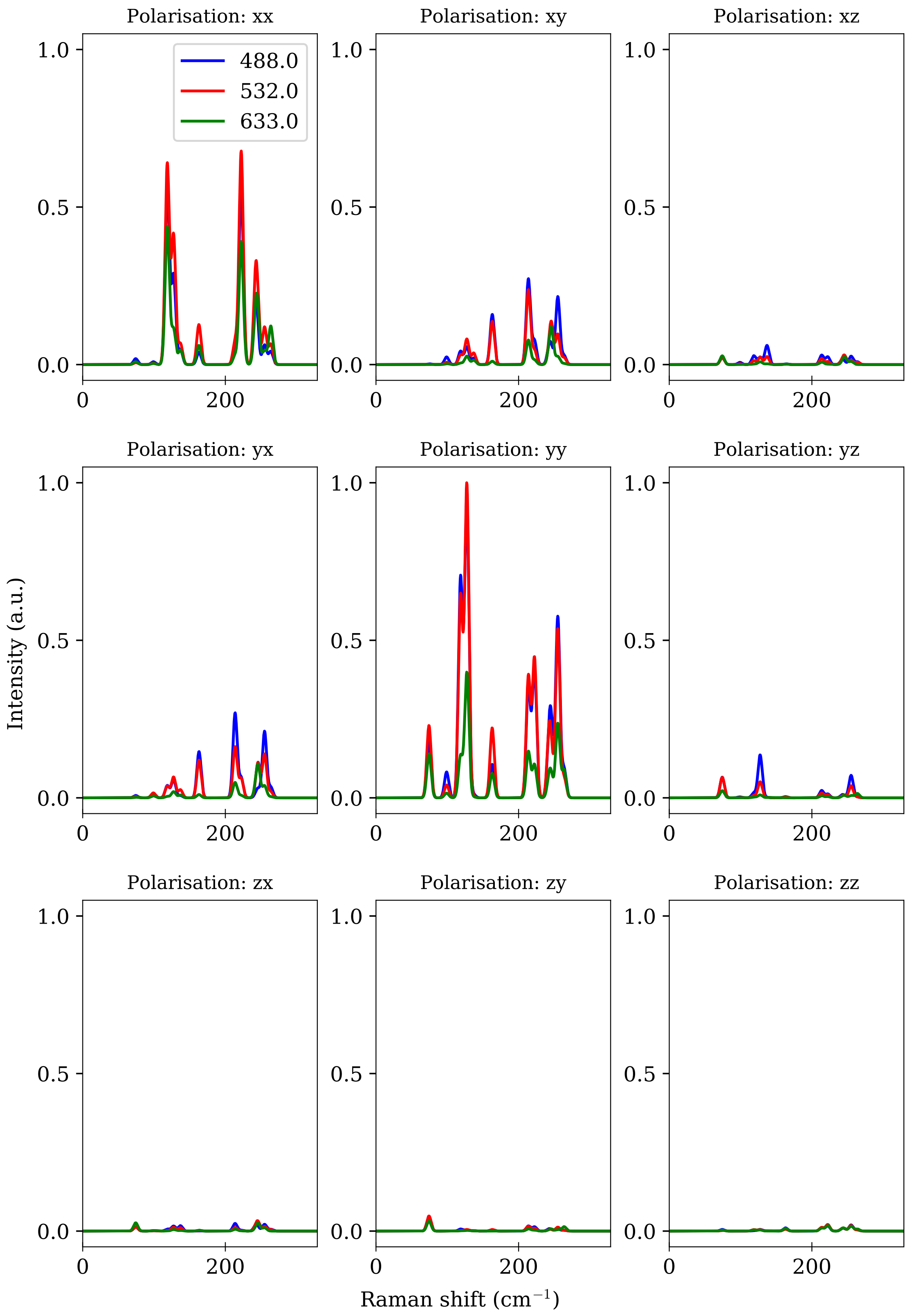

Supplementary Figure 452: Raman spectrum of Pt_2Se_4 with space group 14 and Wyckoff positions c-e-0.

Pt_2Te_2, 12, i-0

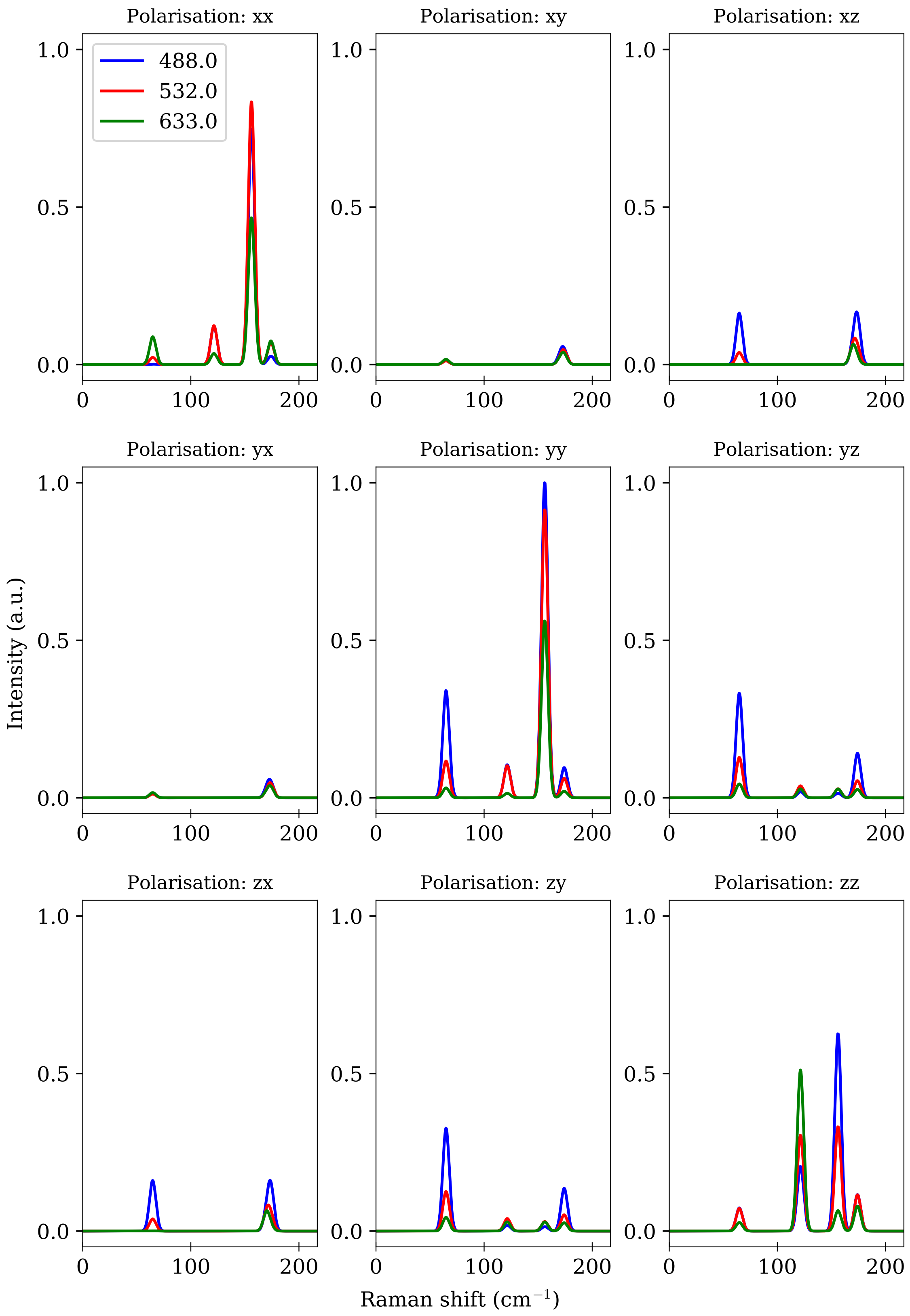

Supplementary Figure 453: Raman spectrum of Pt_2Te_2 with space group 12 and Wyckoff positions i-0.

Pt_2Te_4, 14, c-e-0

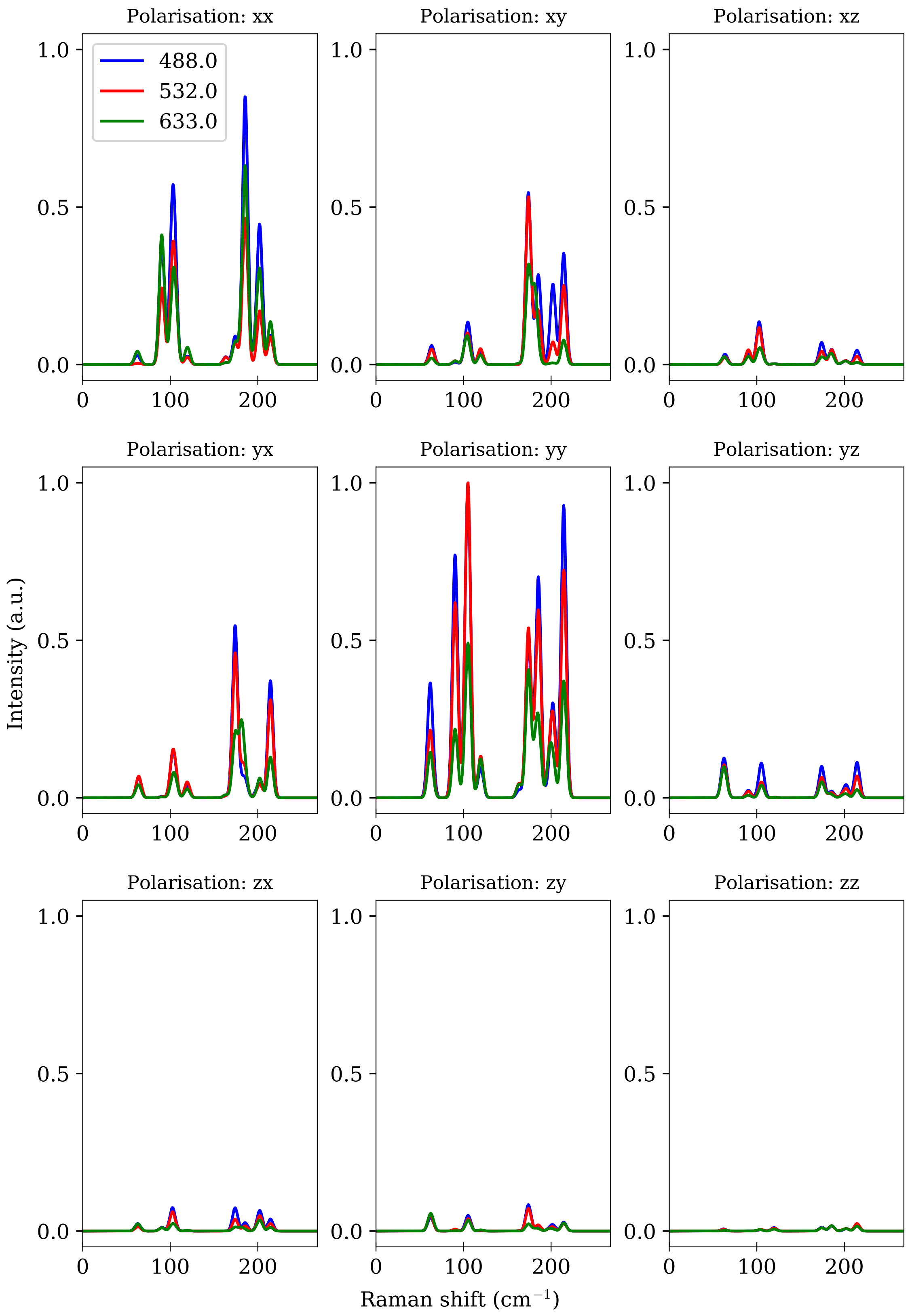

Supplementary Figure 454: Raman spectrum of Pt_2Te_4 with space group 14 and Wyckoff positions c-e-0.

PtO_2, 164, b-d-0

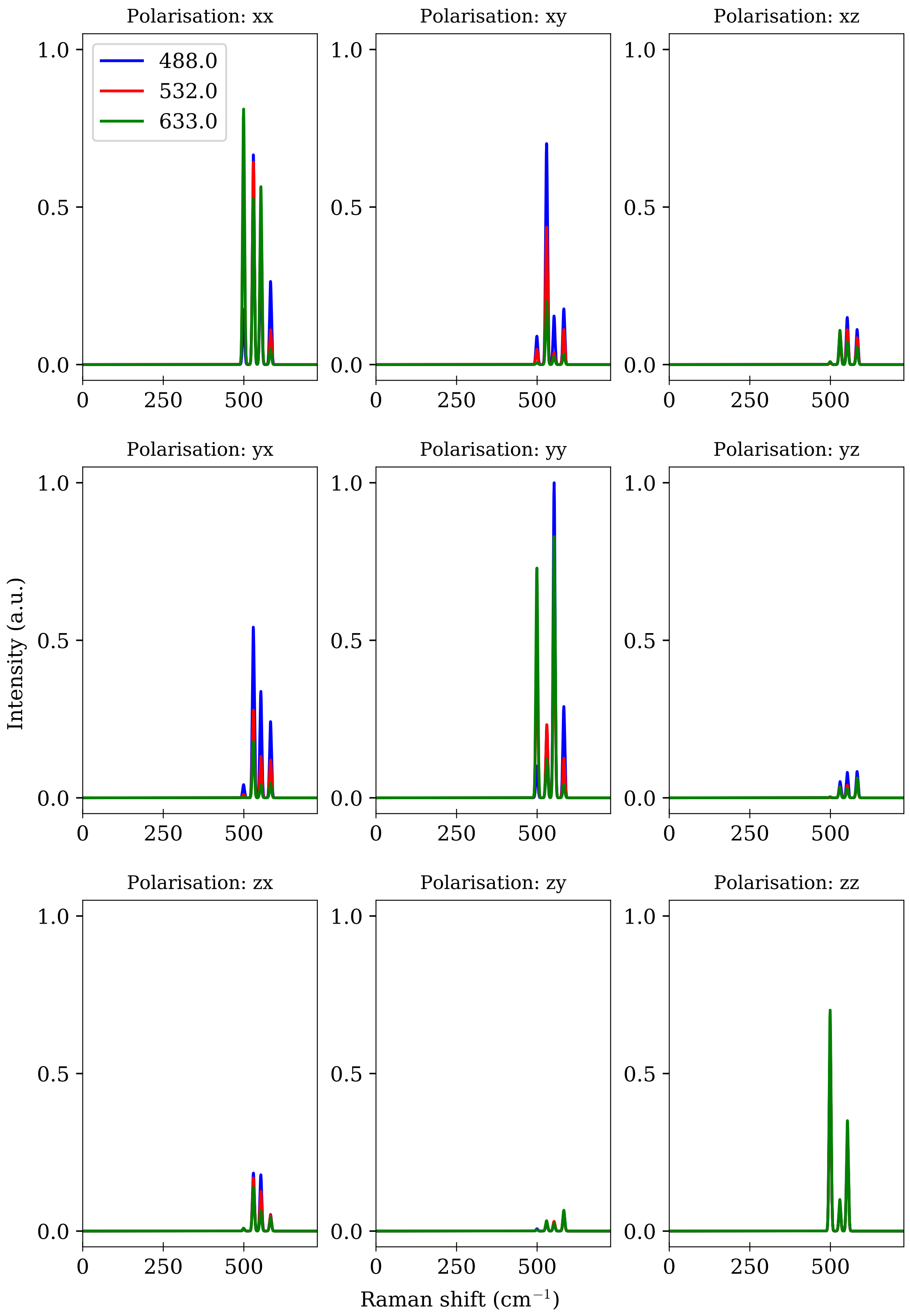

Supplementary Figure 455: Raman spectrum of PtO_2 with space group 164 and Wyckoff positions b-d-0.

PtS_2, 164, b-d-0

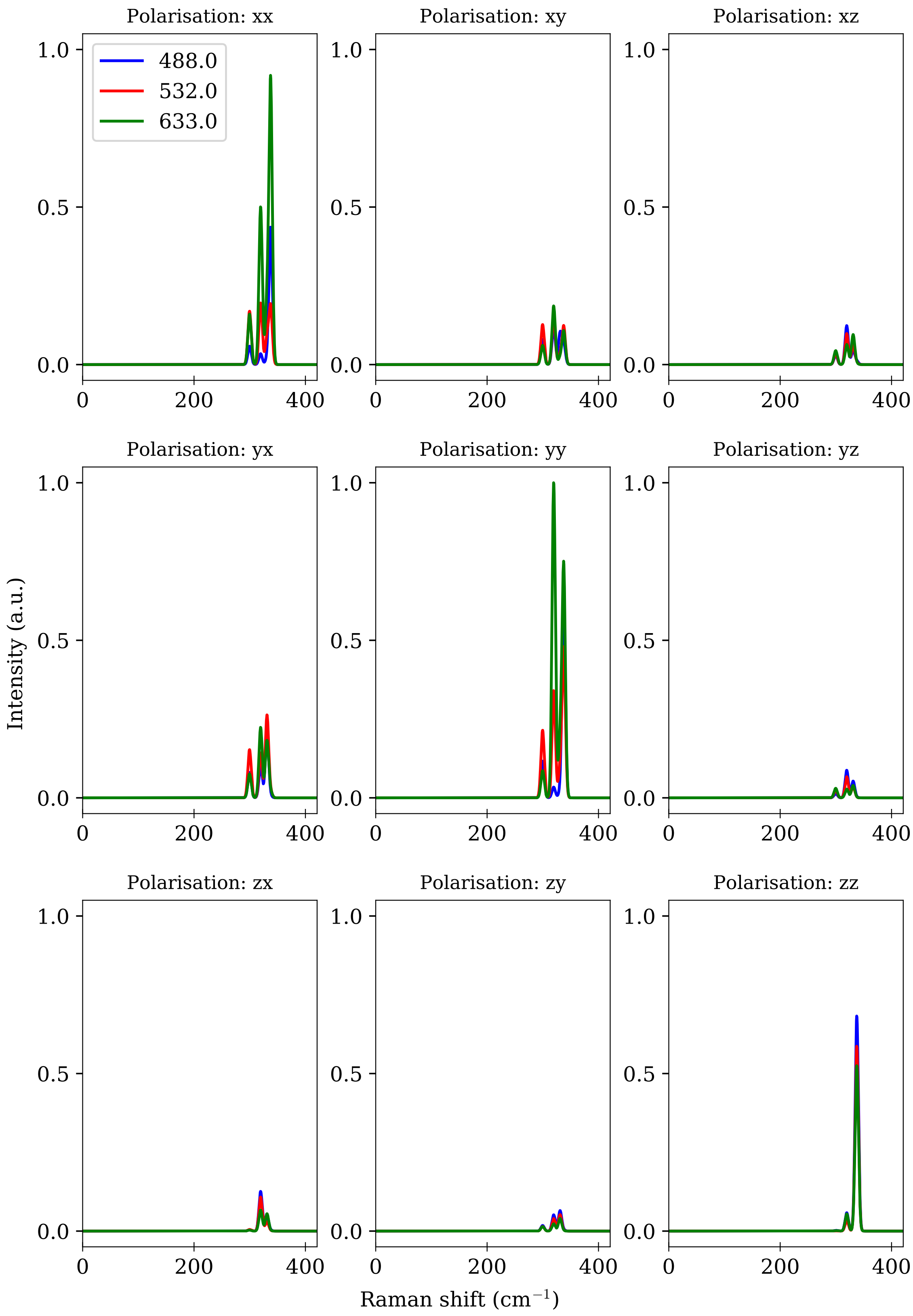

Supplementary Figure 456: Raman spectrum of PtS_2 with space group 164 and Wyckoff positions b-d-0.

$PtSe_2$, 164, b-d-0

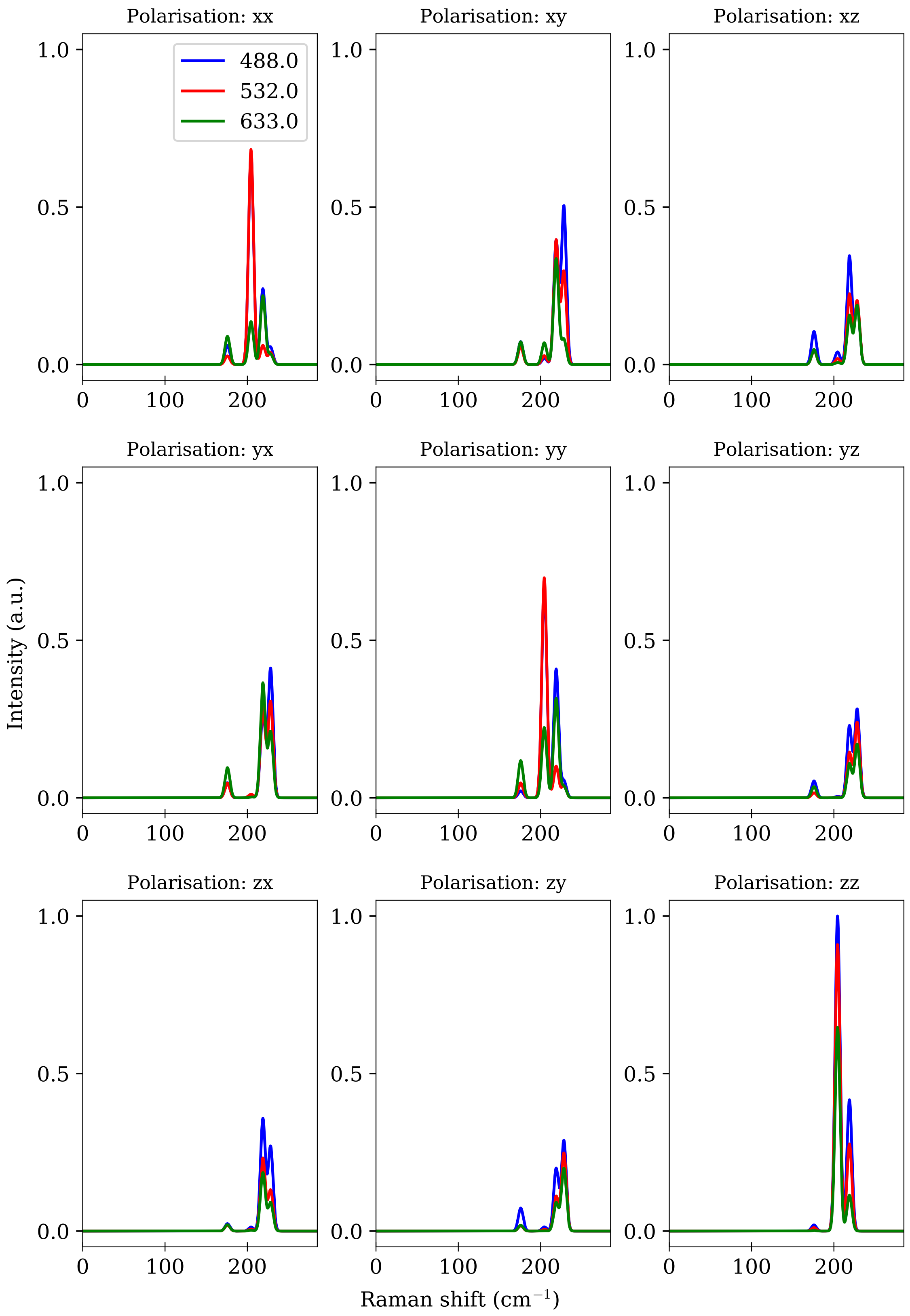

Supplementary Figure 457: Raman spectrum of $PtSe_2$ with space group 164 and Wyckoff positions b-d-0.

$PtSe_2$, 187, b-i-0

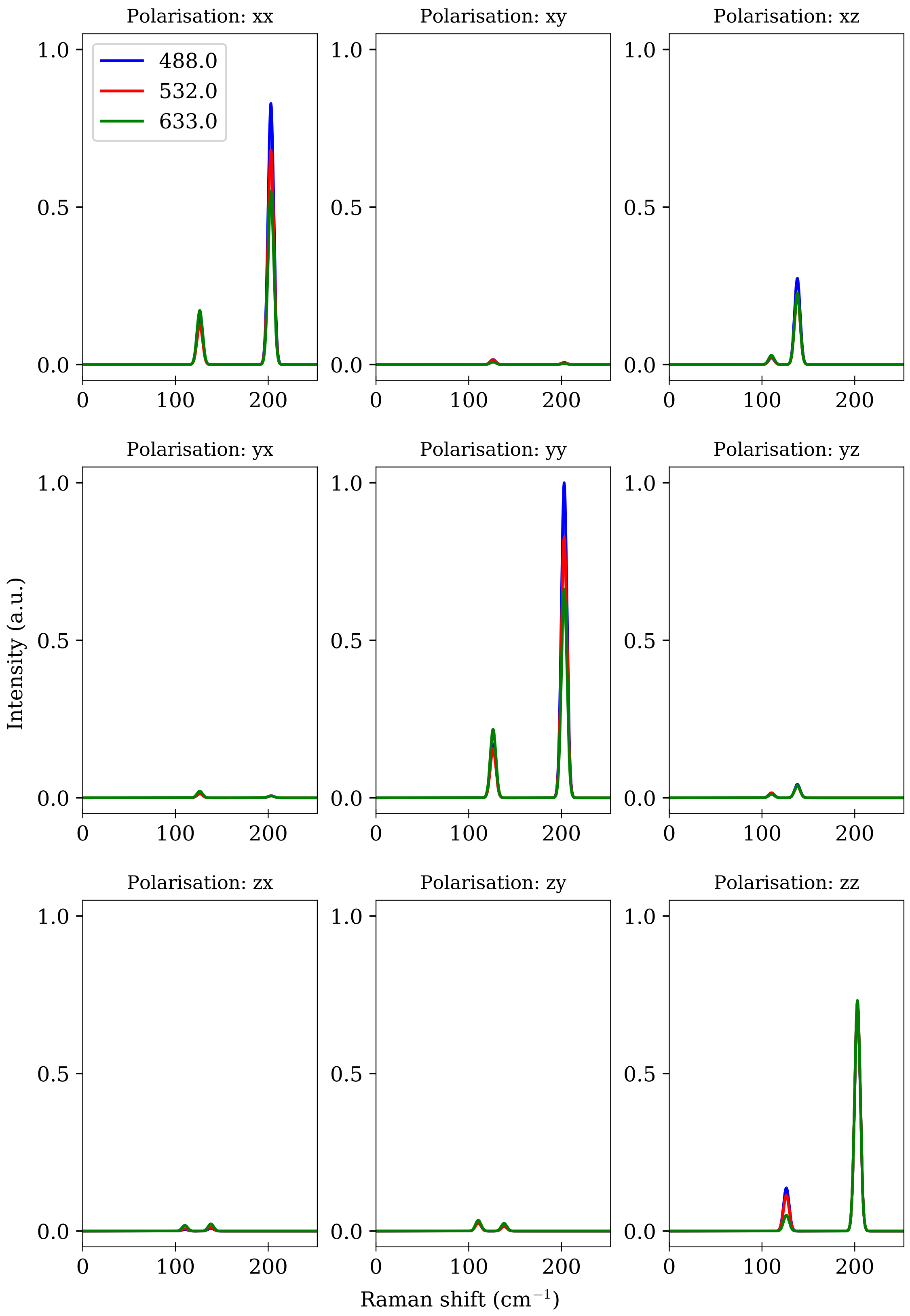

Supplementary Figure 458: Raman spectrum of $PtSe_2$ with space group 187 and Wyckoff positions b-i-0.

$PtTe_2$, 164, b-d-0

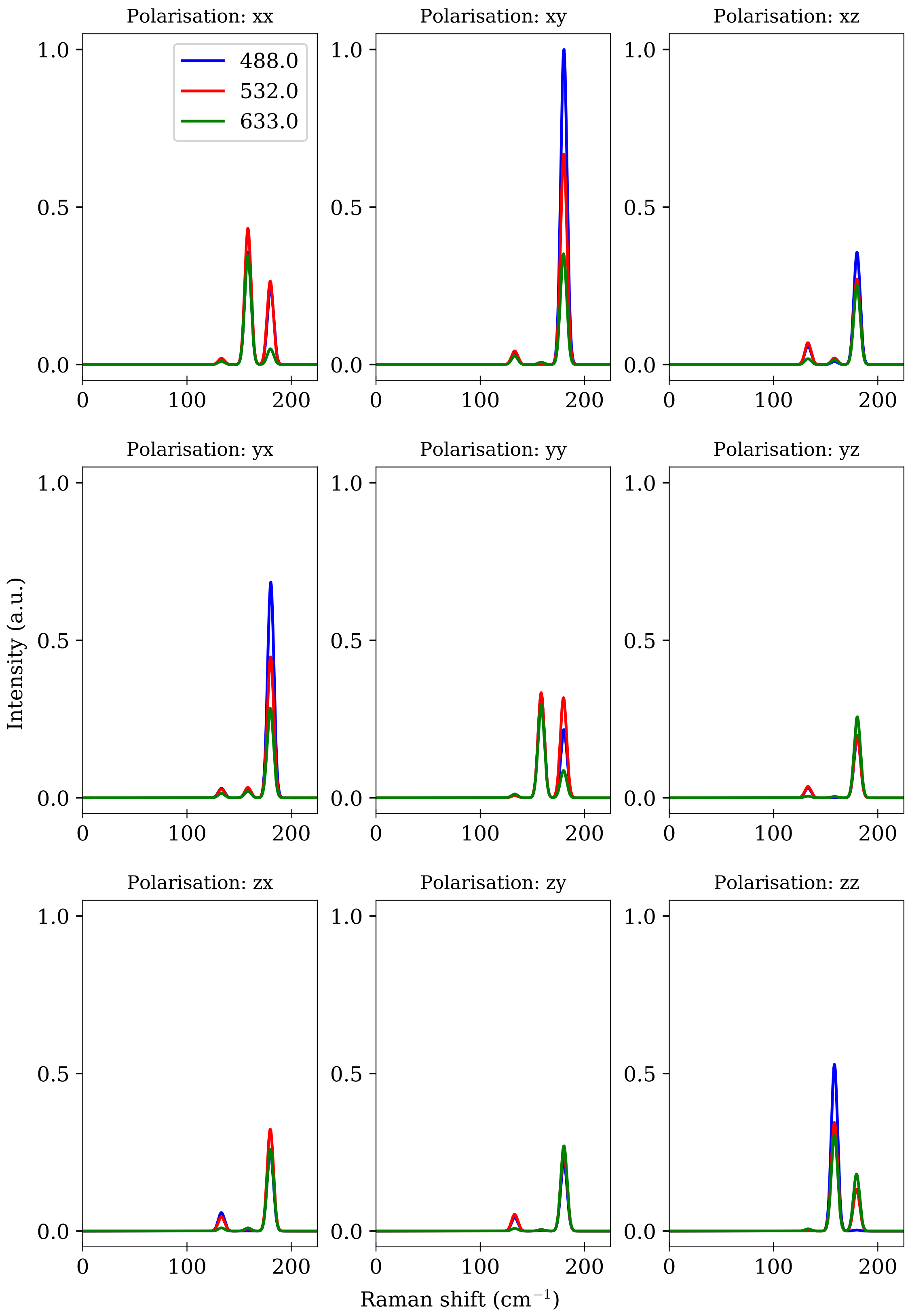

Supplementary Figure 459: Raman spectrum of $PtTe_2$ with space group 164 and Wyckoff positions b-d-0.

$PtTe_2$, 187, b-i-0

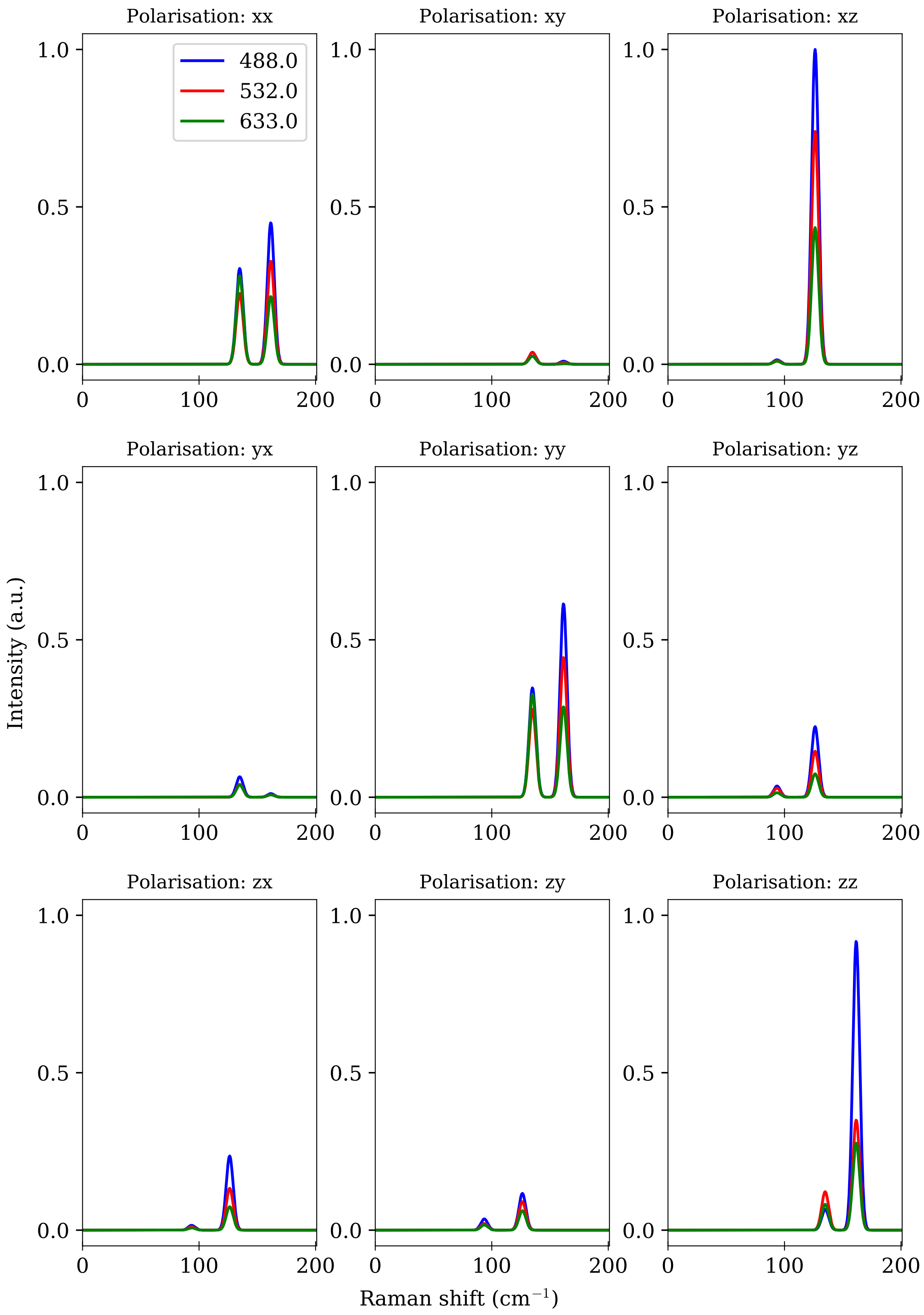

Supplementary Figure 460: Raman spectrum of $PtTe_2$ with space group 187 and Wyckoff positions b-i-0.

Rb_2F_2, 129, b-c-0

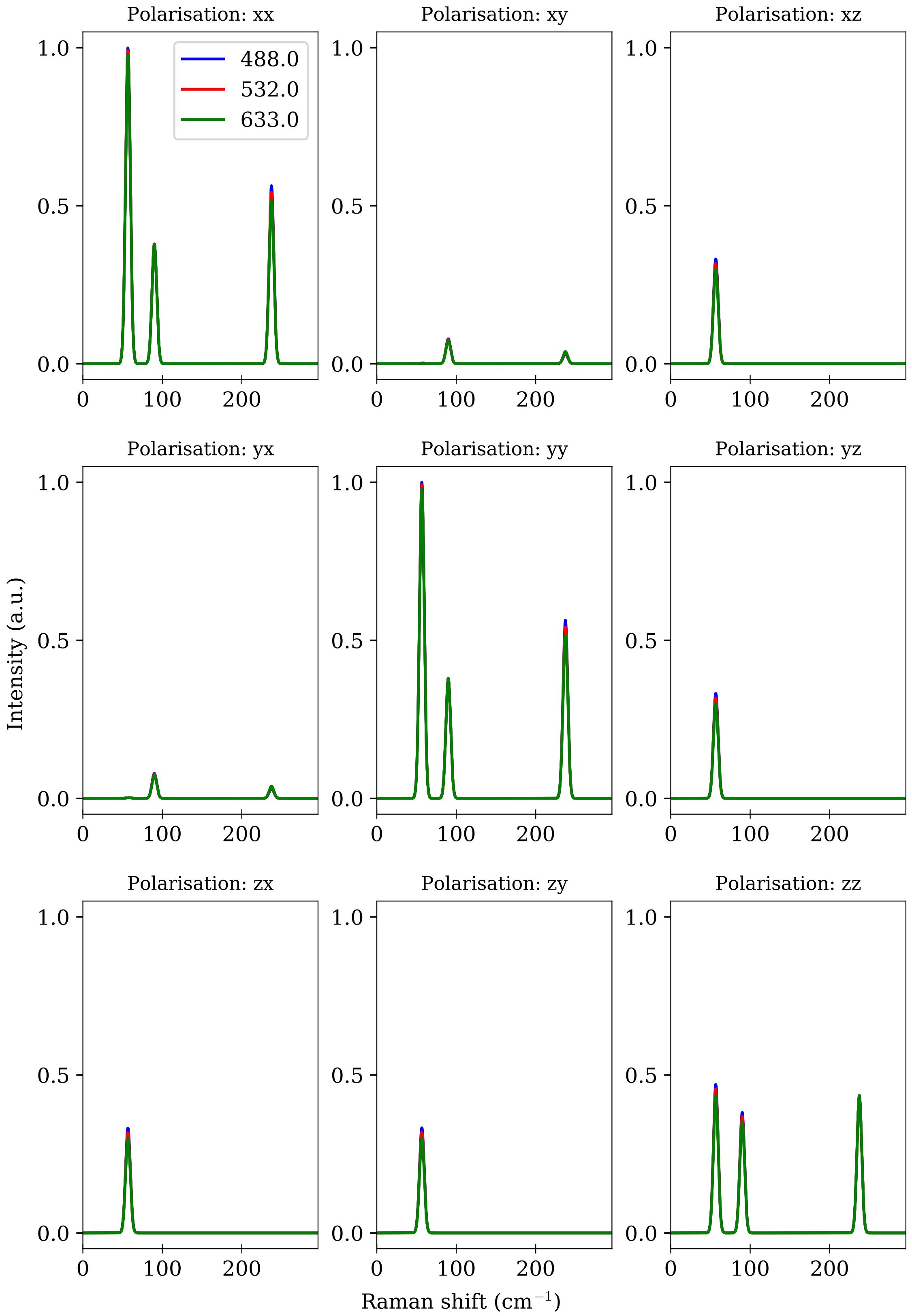

Supplementary Figure 461: Raman spectrum of Rb_2F_2 with space group 129 and Wyckoff positions b-c-0.

Re_2Cl_2, 2, i-0

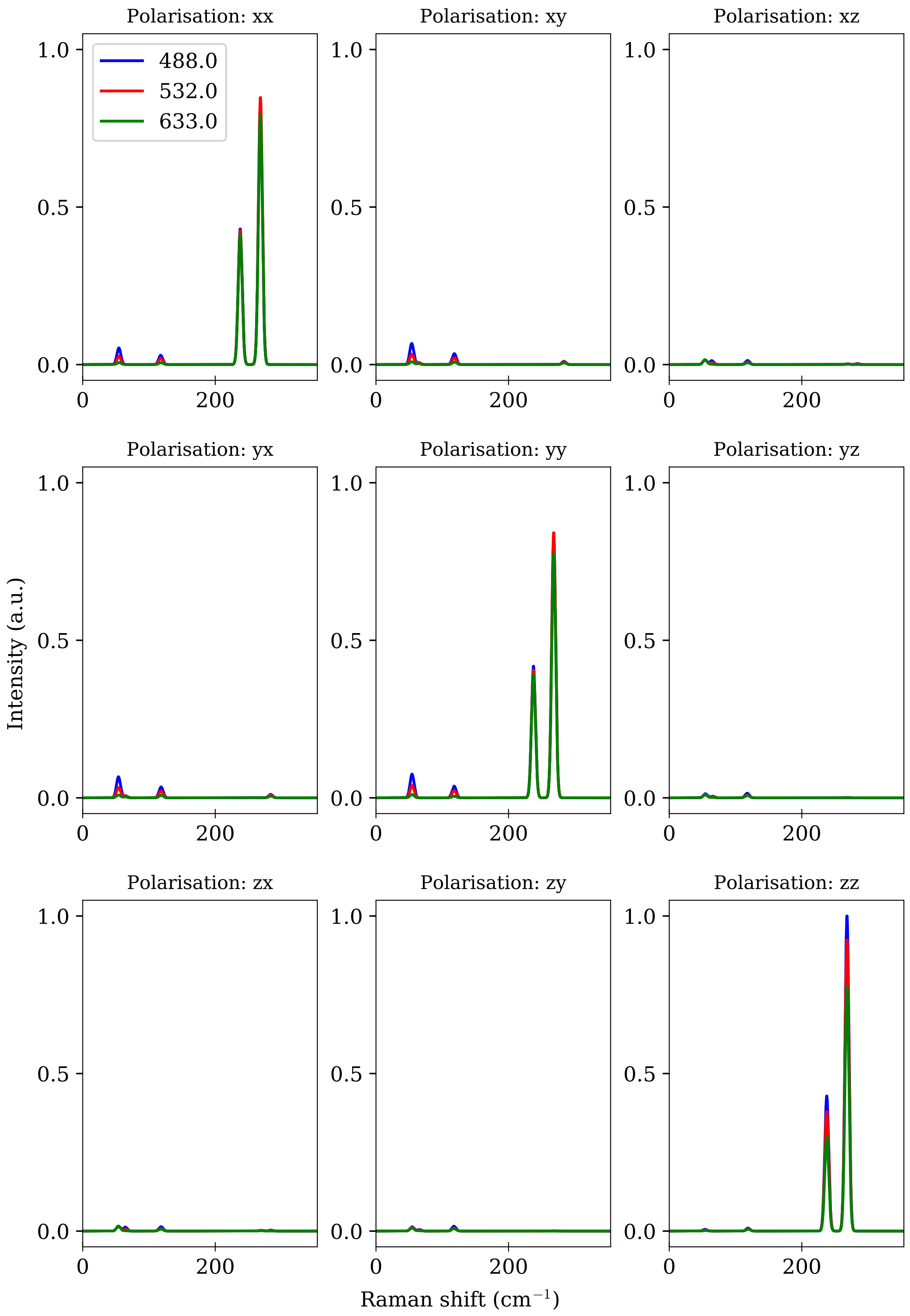

Supplementary Figure 462: Raman spectrum of Re_2Cl_2 with space group 2 and Wyckoff positions i-0.

Re_2I_2, 2, i-0

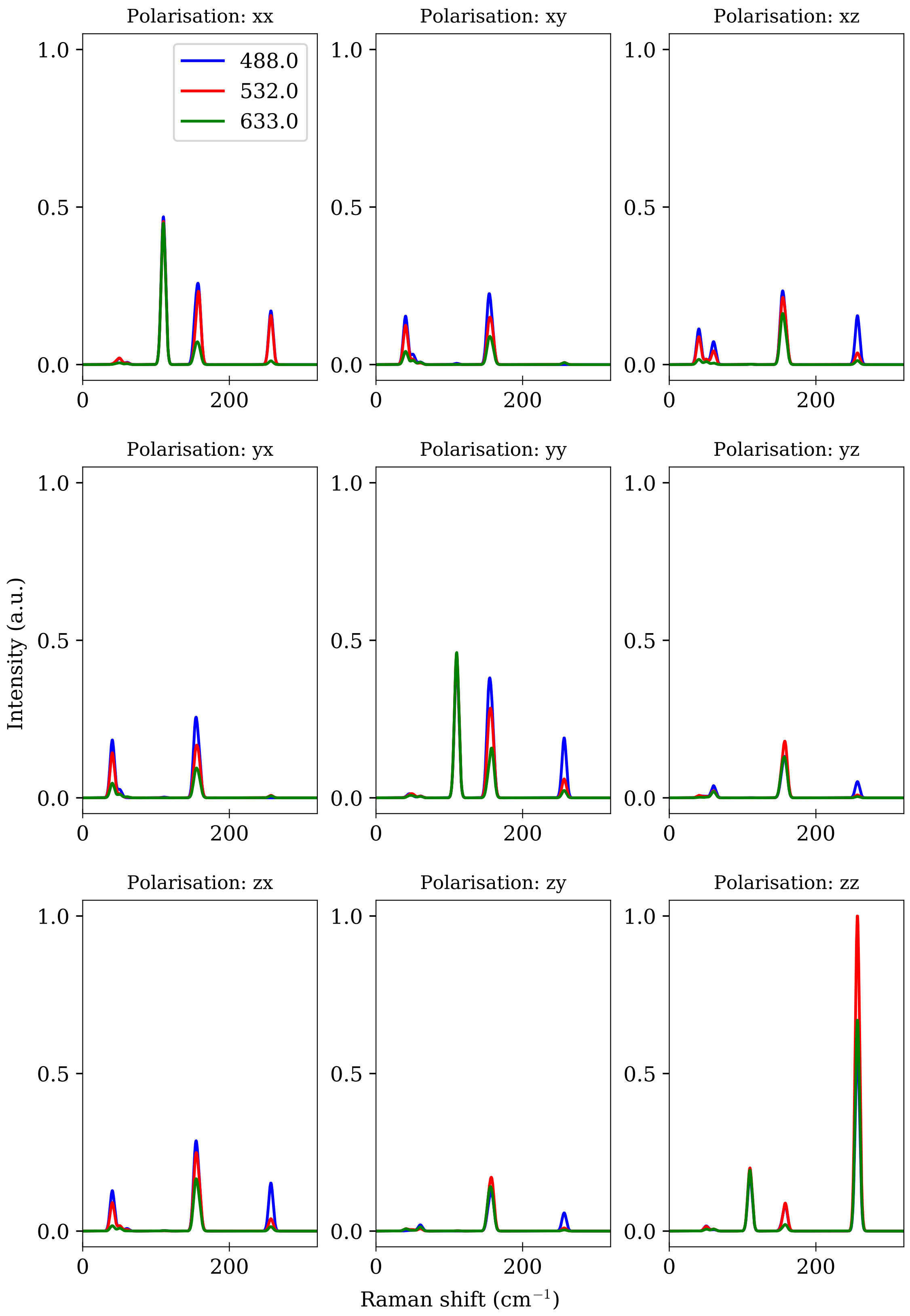

Supplementary Figure 463: Raman spectrum of Re_2I_2 with space group 2 and Wyckoff positions i-0.

Re_2S_2, 129, b-c-0

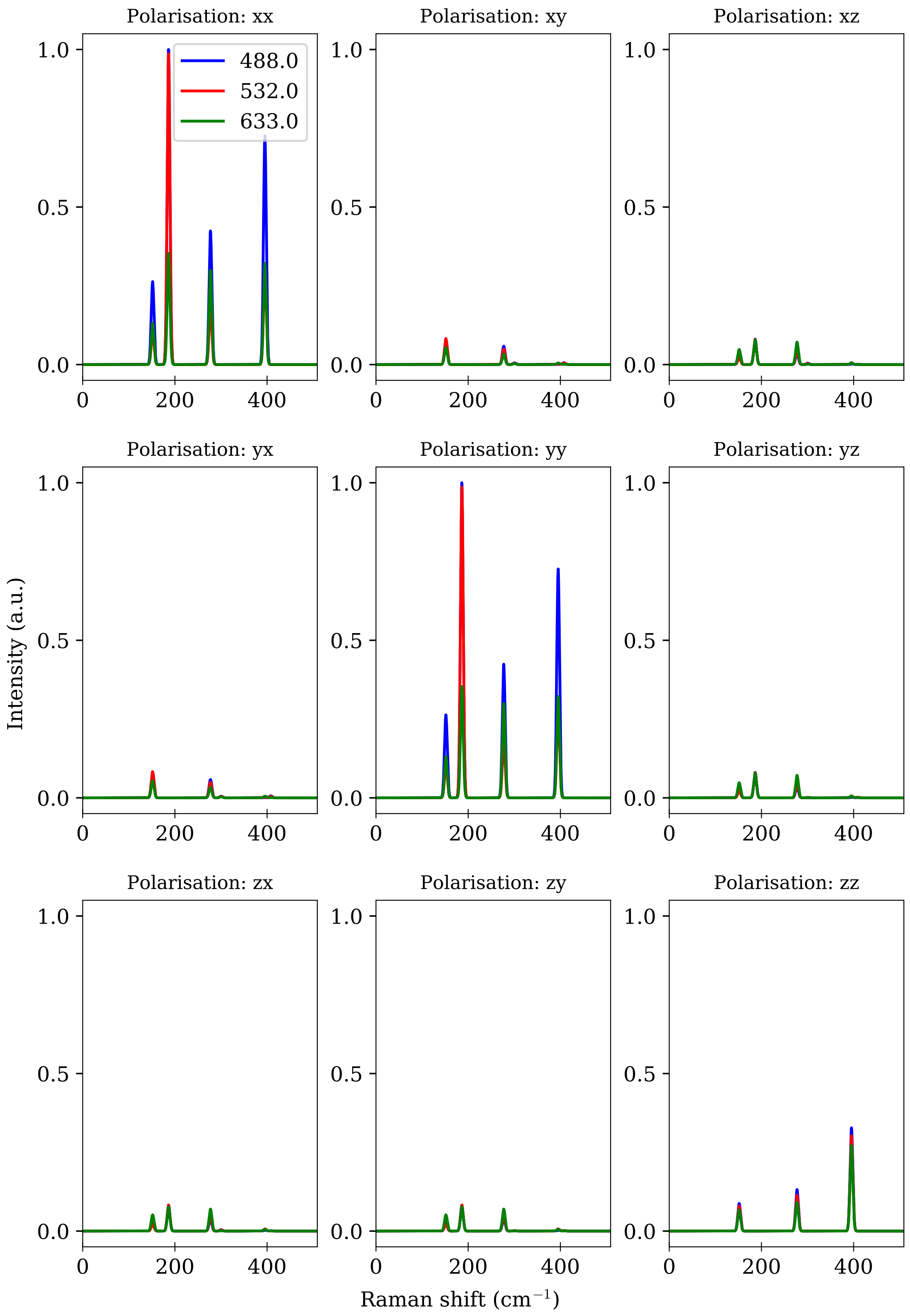

Supplementary Figure 464: Raman spectrum of Re_2S_2 with space group 129 and Wyckoff positions b-c-0.

Re_2S_2, 164, d-0

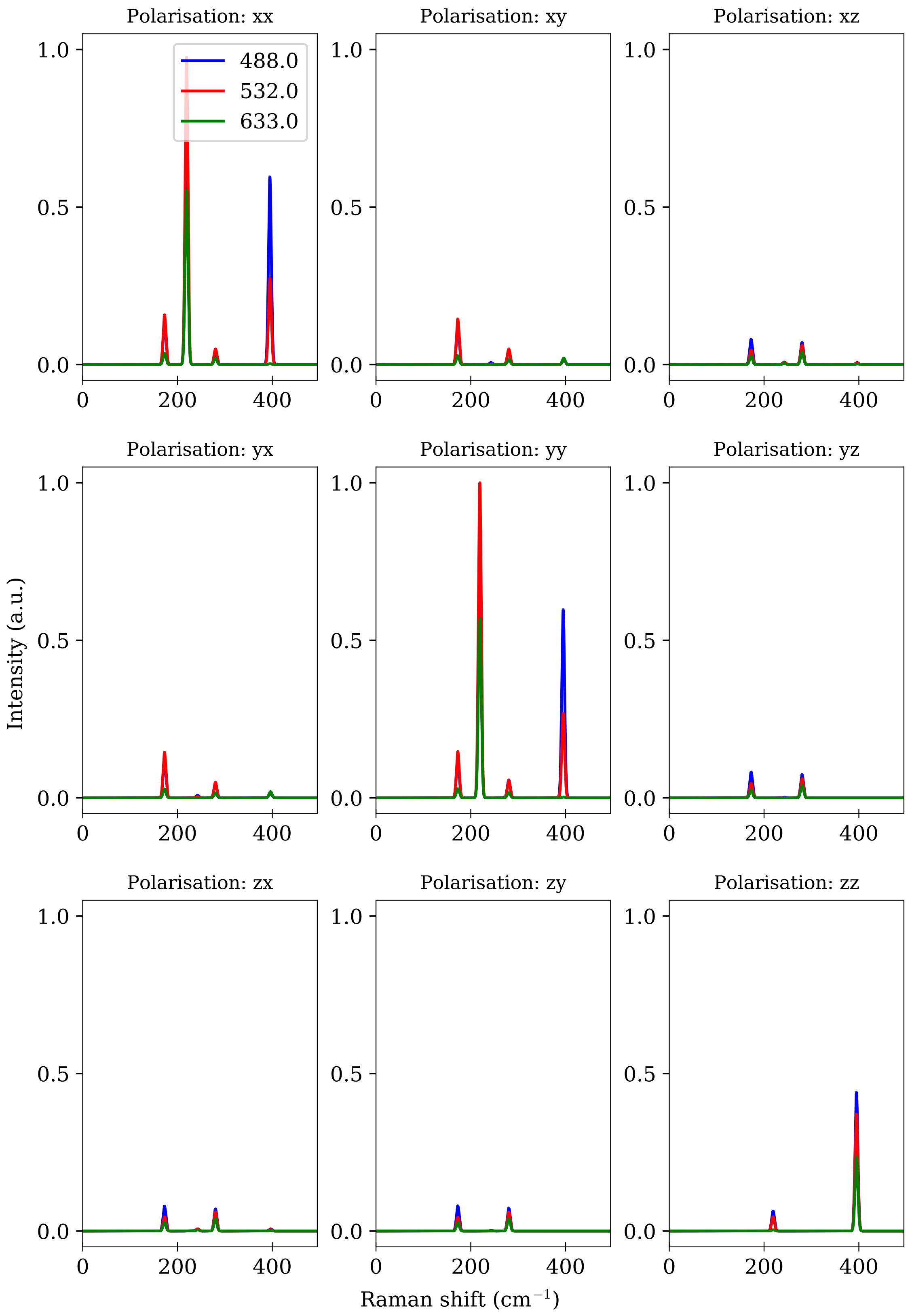

Supplementary Figure 465: Raman spectrum of Re_2S_2 with space group 164 and Wyckoff positions d-0.

Re_2Se_2, 129, b-c-0

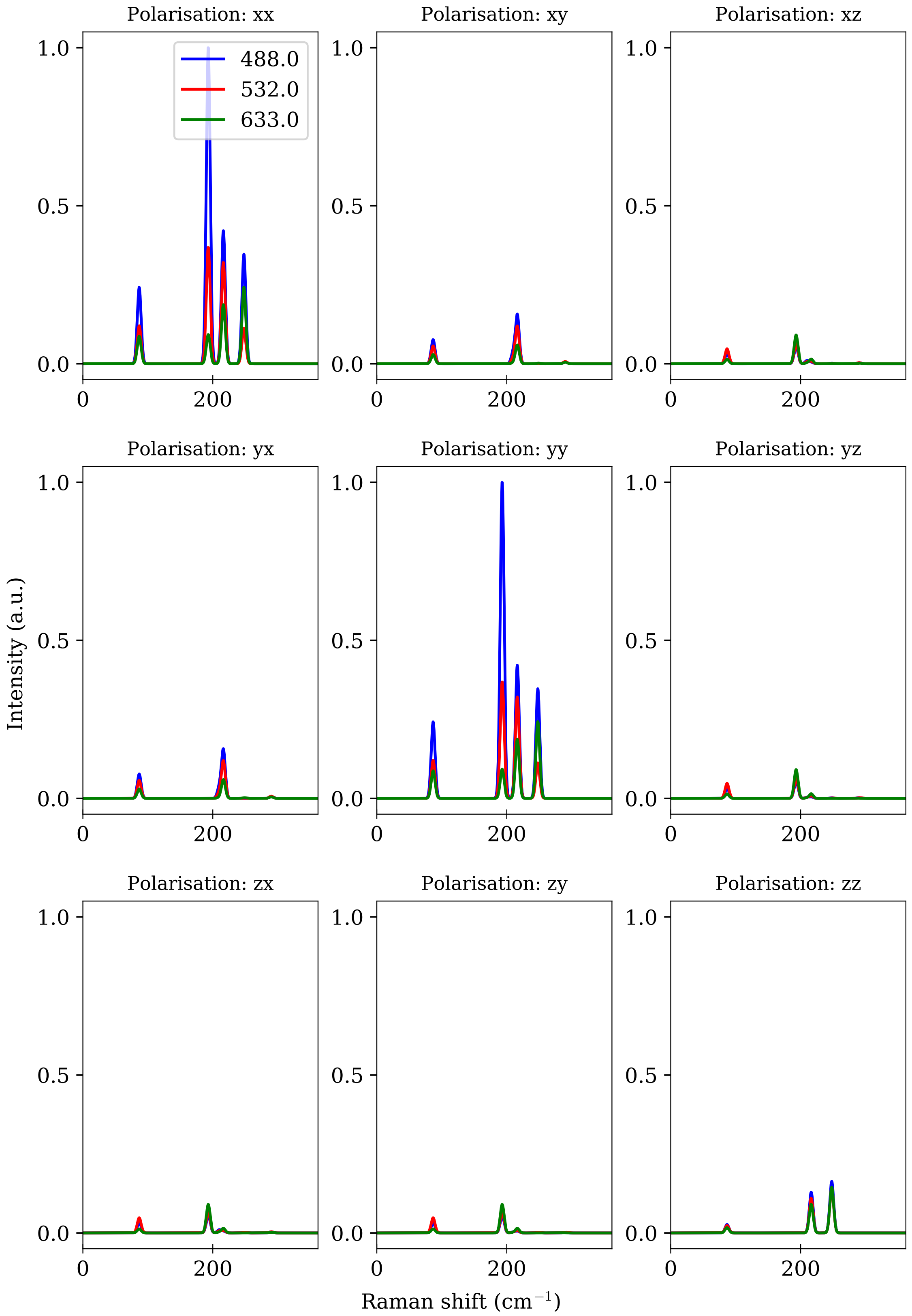

Supplementary Figure 466: Raman spectrum of Re_2Se_2 with space group 129 and Wyckoff positions b-c-0.

Re_2Se_2, 164, d-0

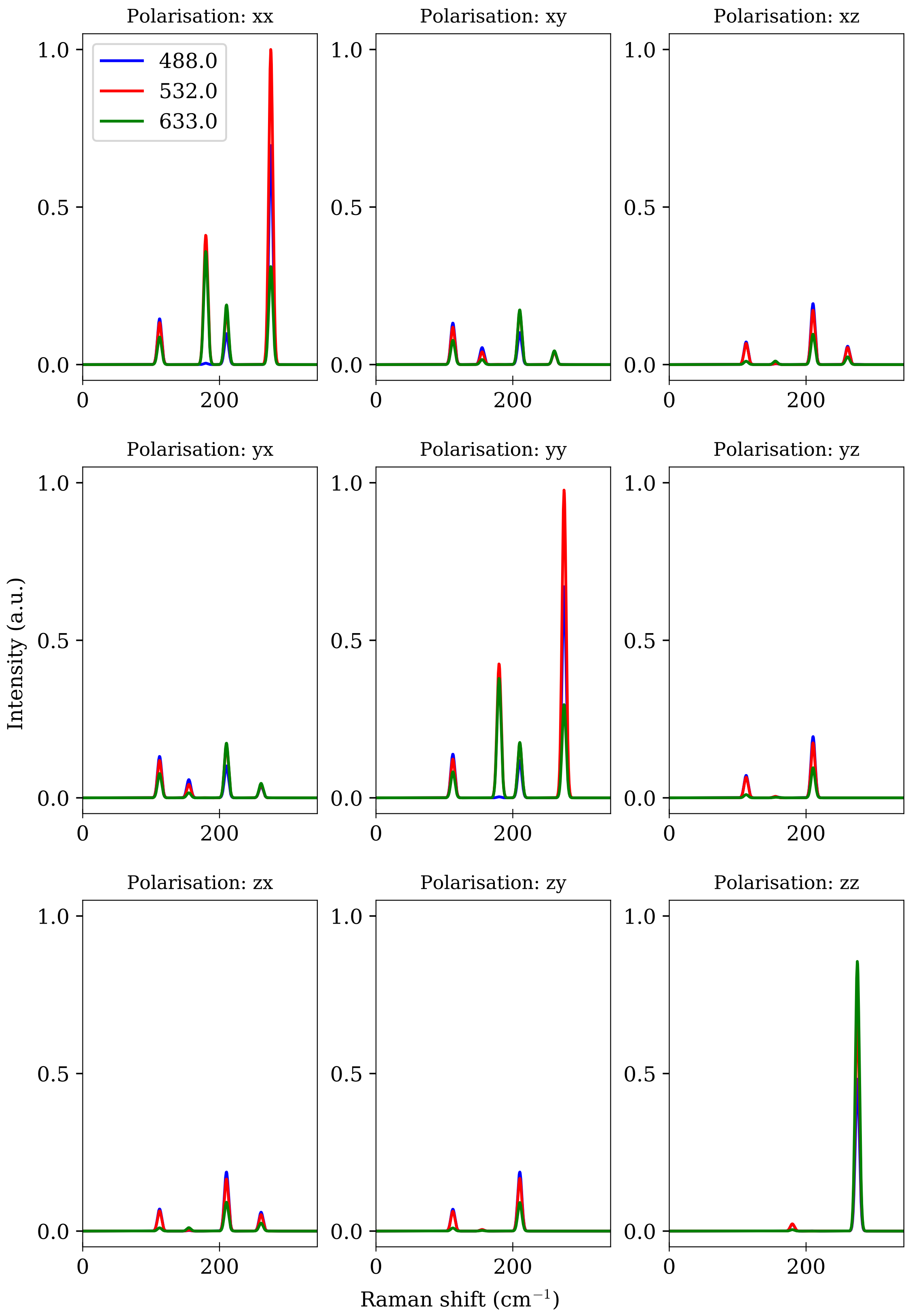

Supplementary Figure 467: Raman spectrum of Re_2Se_2 with space group 164 and Wyckoff positions d-0.

ReH_2, 187, a-i-0

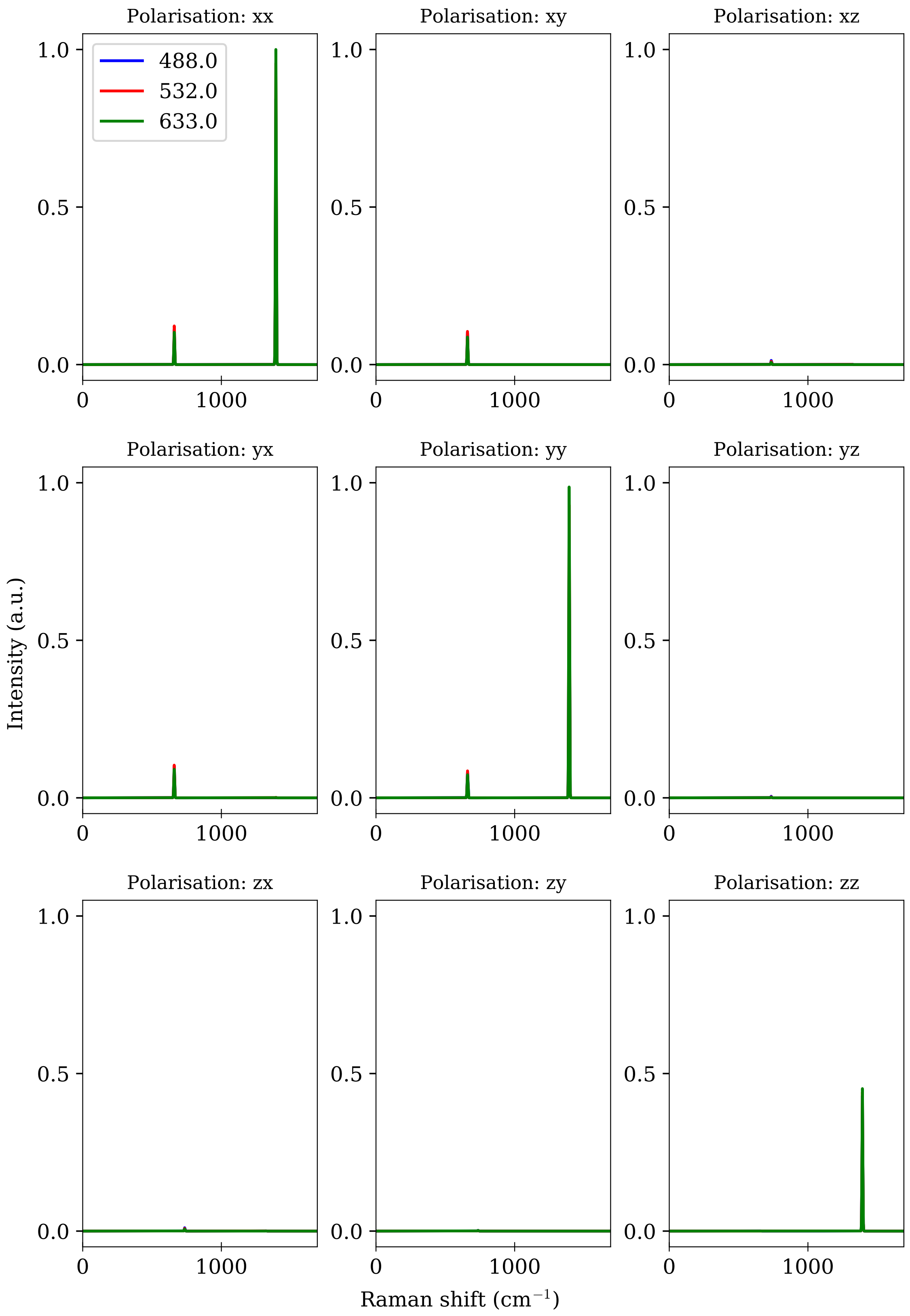

Supplementary Figure 468: Raman spectrum of ReH_2 with space group 187 and Wyckoff positions a-i-0.

ReS$_2$, 115, d-g-0

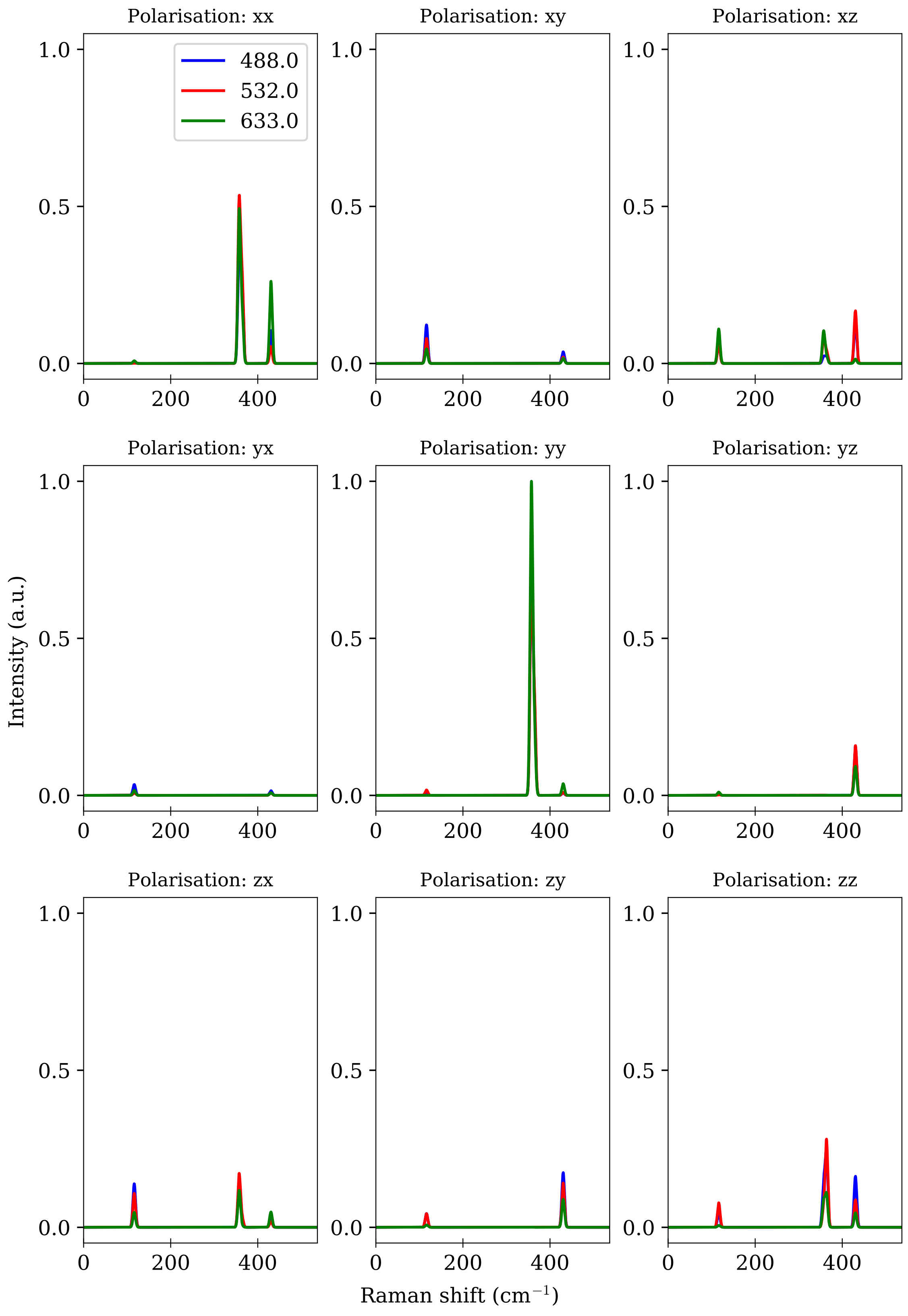

Supplementary Figure 469: Raman spectrum of ReS$_2$ with space group 115 and Wyckoff positions d-g-0.

$Rh_2Br_2O_2$, 59, a-b-0

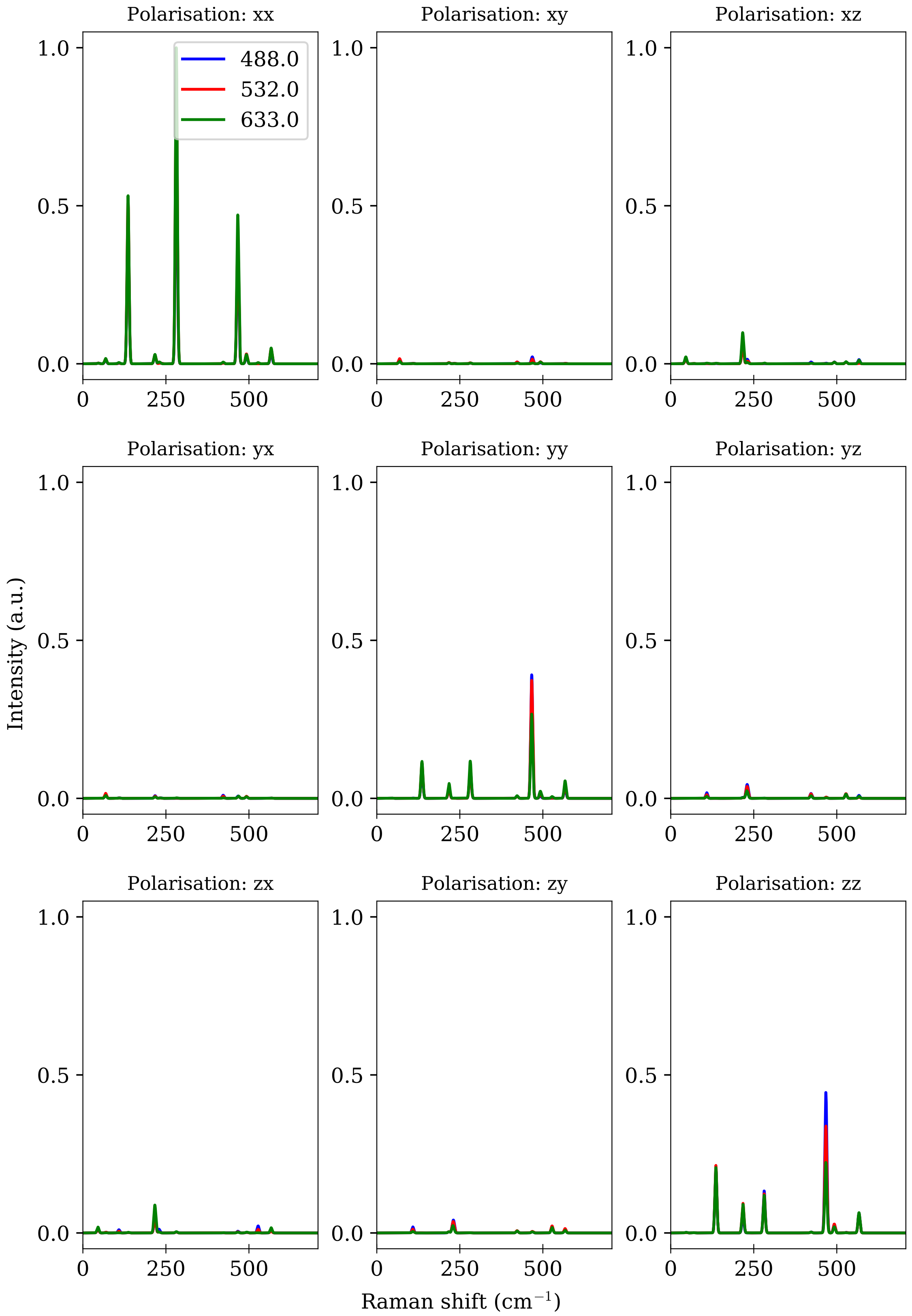

Supplementary Figure 470: Raman spectrum of $Rh_2Br_2O_2$ with space group 59 and Wyckoff positions a-b-0.

Rh_2Br_6, 162, d-k-0

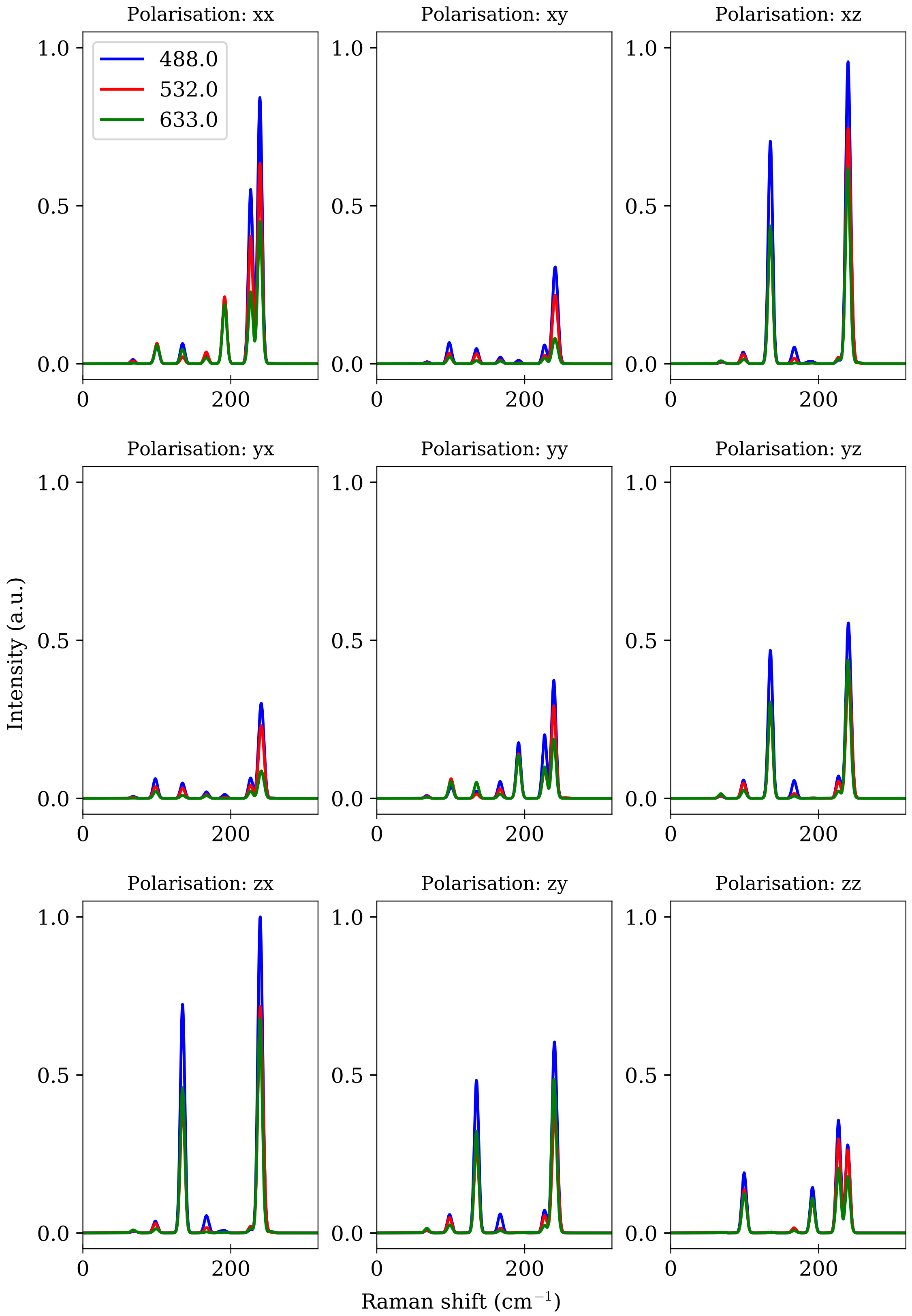

Supplementary Figure 471: Raman spectrum of Rh_2Br_6 with space group 162 and Wyckoff positions d-k-0.

$Rh_2Cl_2O_2$, 59, a-b-0

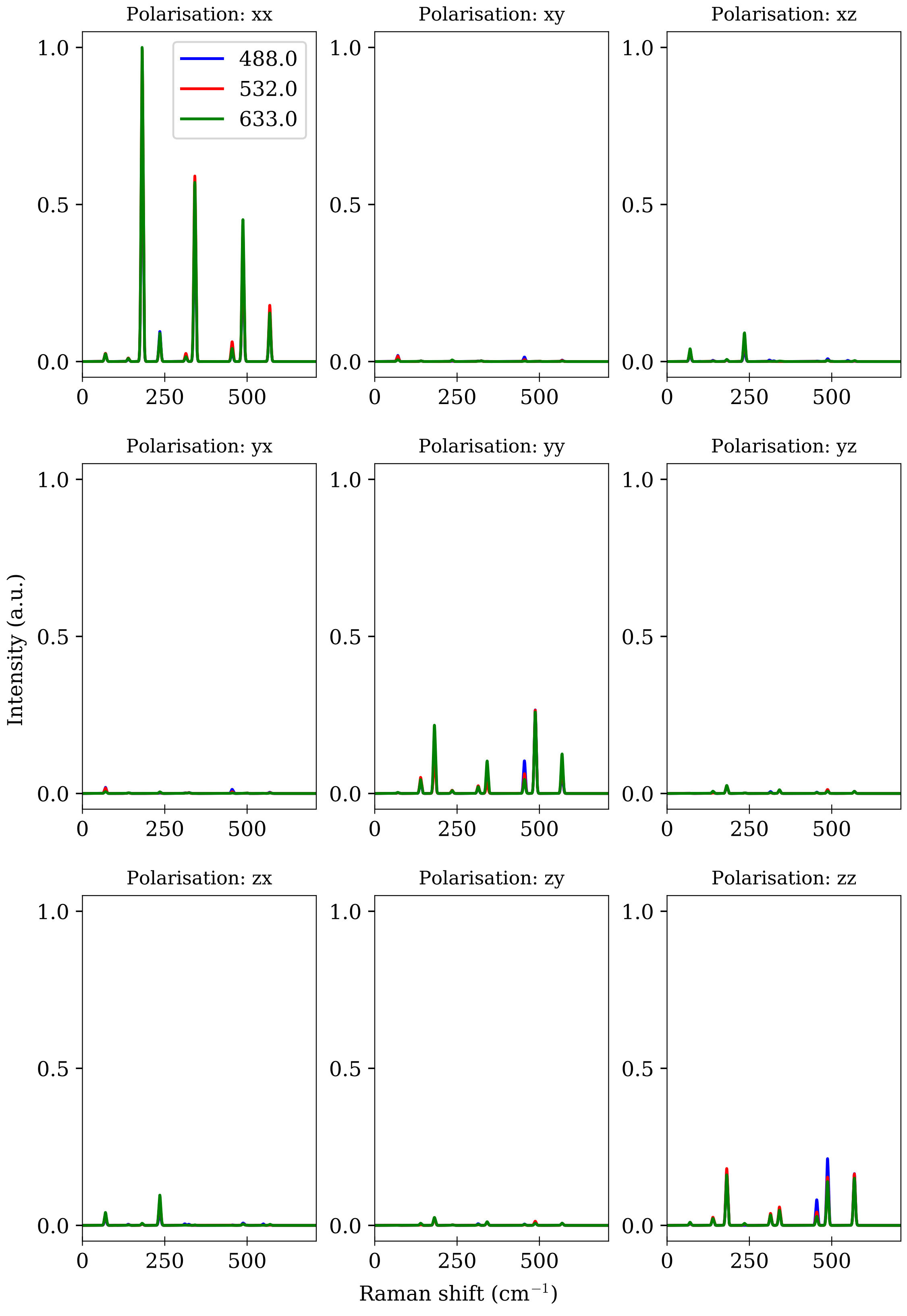

Supplementary Figure 472: Raman spectrum of $Rh_2Cl_2O_2$ with space group 59 and Wyckoff positions a-b-0.

Rh_2Cl_6, 162, d-k-0

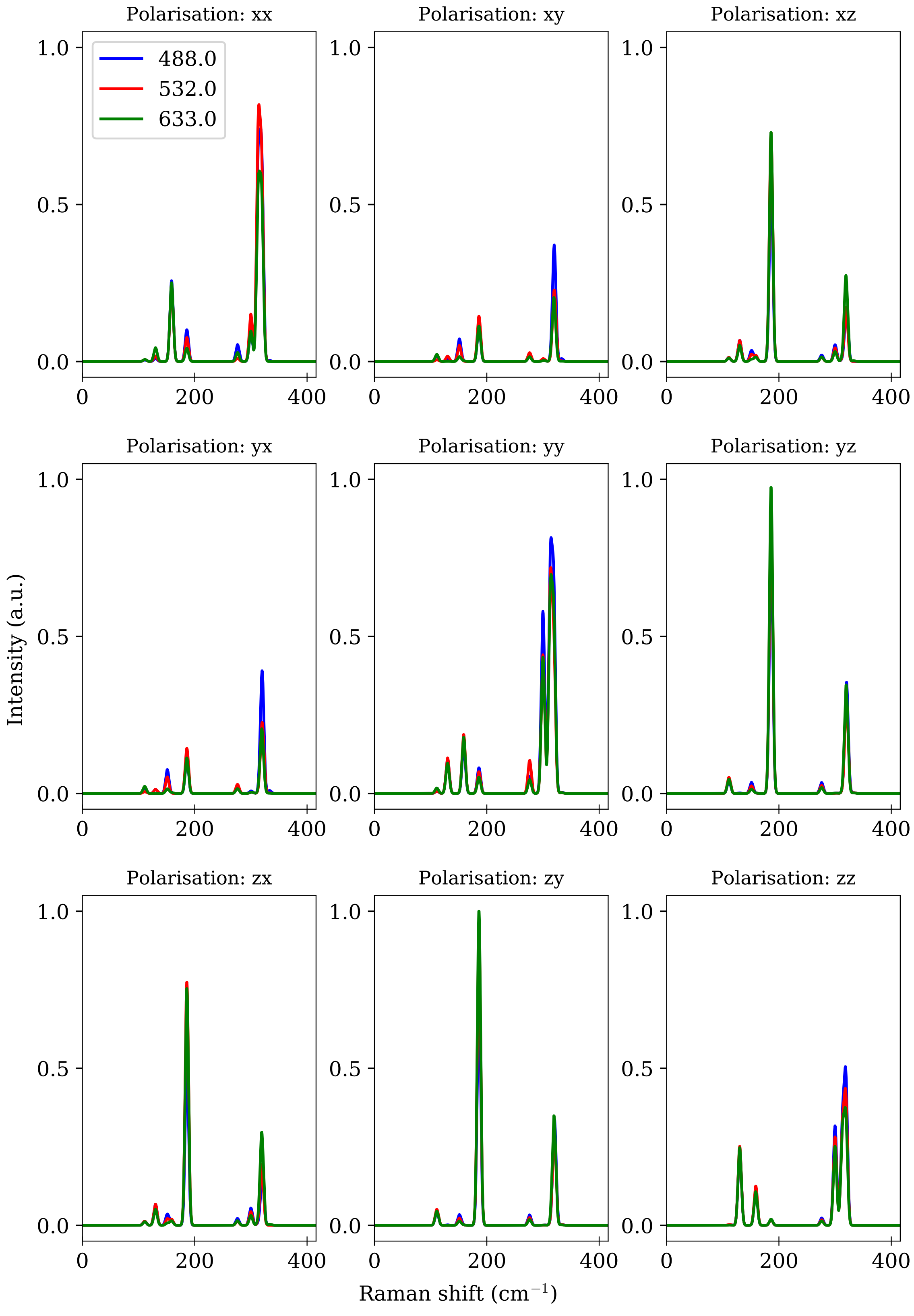

Supplementary Figure 473: Raman spectrum of Rh_2Cl_6 with space group 162 and Wyckoff positions d-k-0.

Rh_2O_2, 6, a-b-0

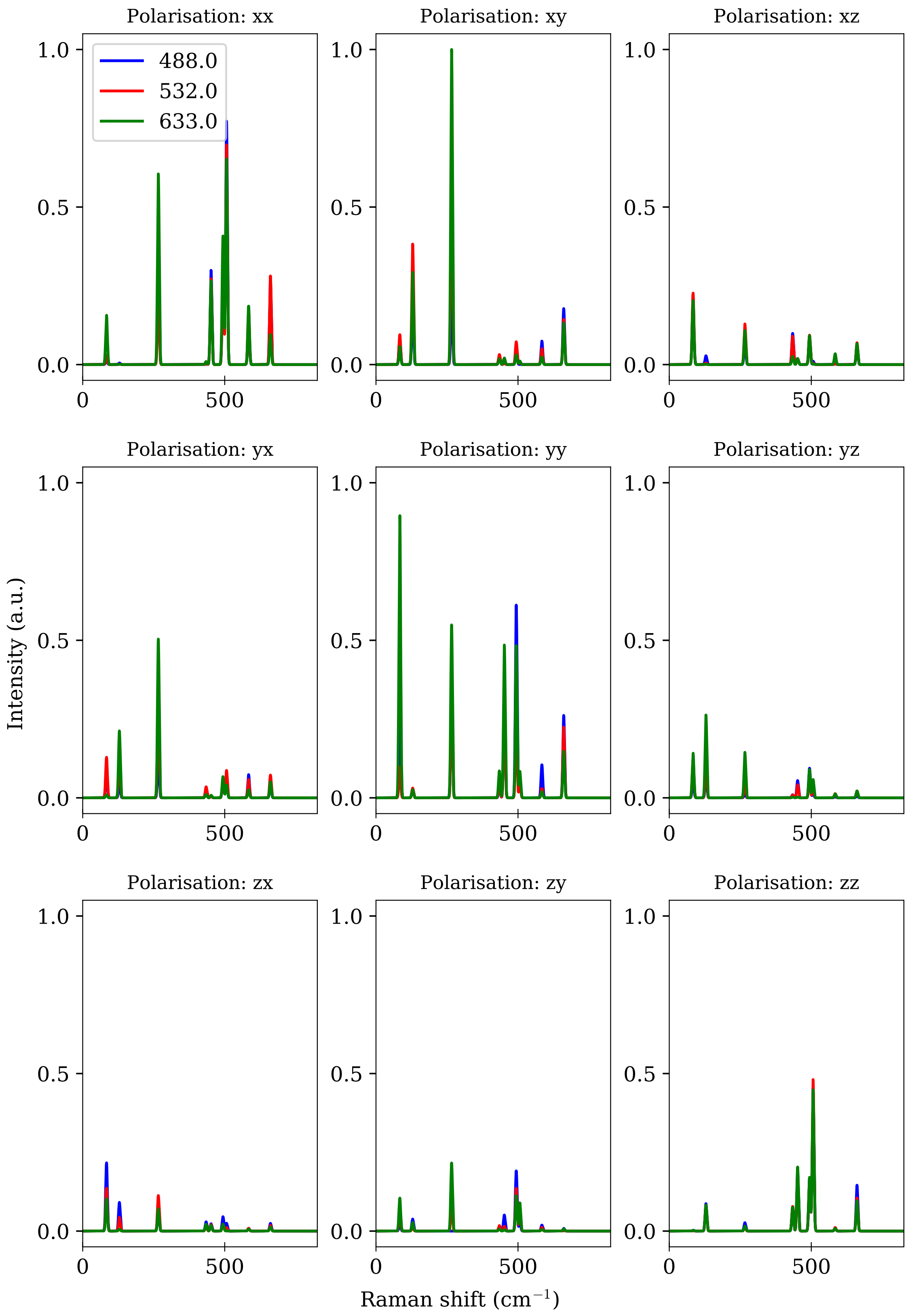

Supplementary Figure 474: Raman spectrum of Rh_2O_2 with space group 6 and Wyckoff positions a-b-0.

Rh_2S_2, 12, i-0

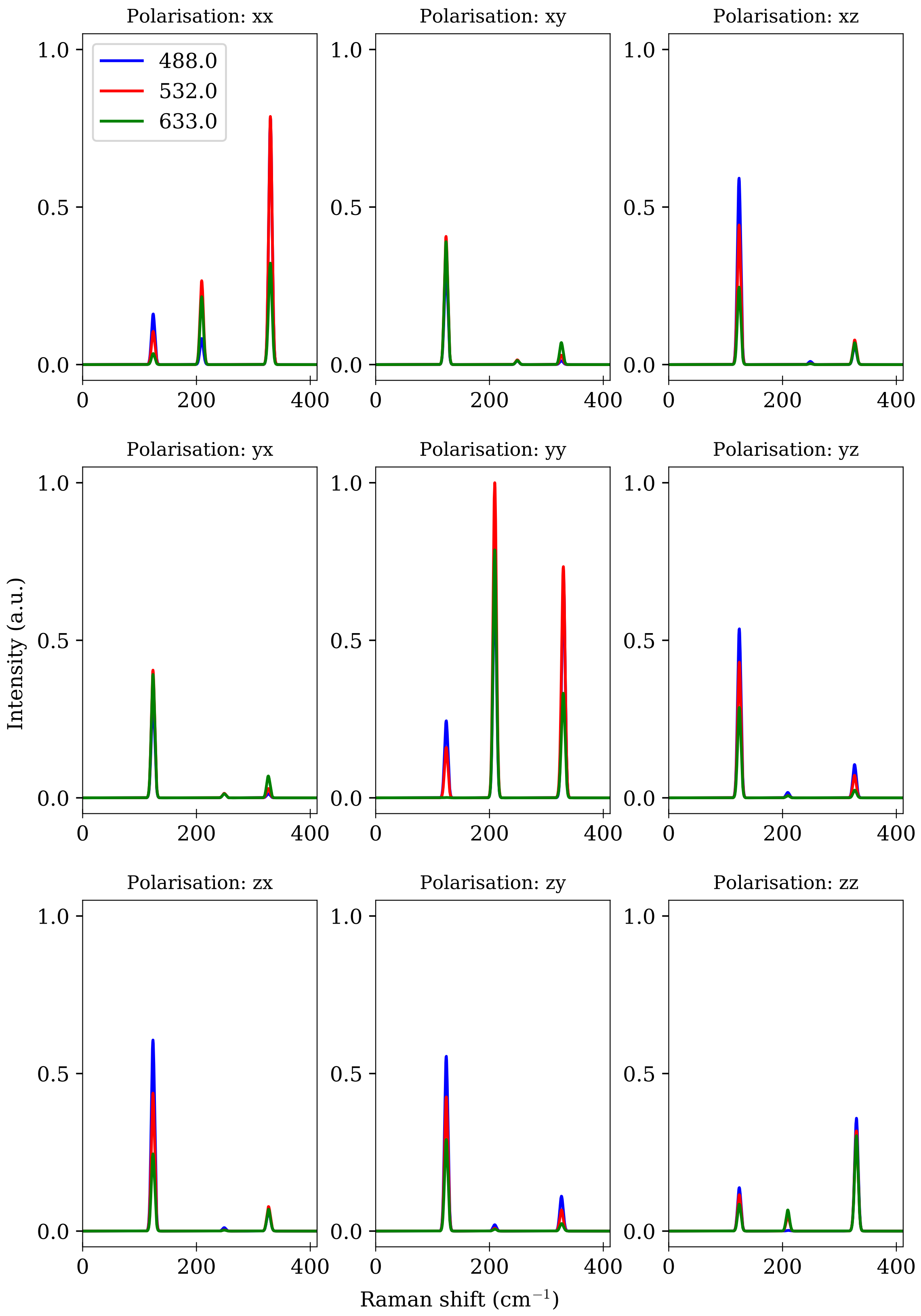

Supplementary Figure 475: Raman spectrum of Rh_2S_2 with space group 12 and Wyckoff positions i-0.

Rh_2Se_2, 12, i-0

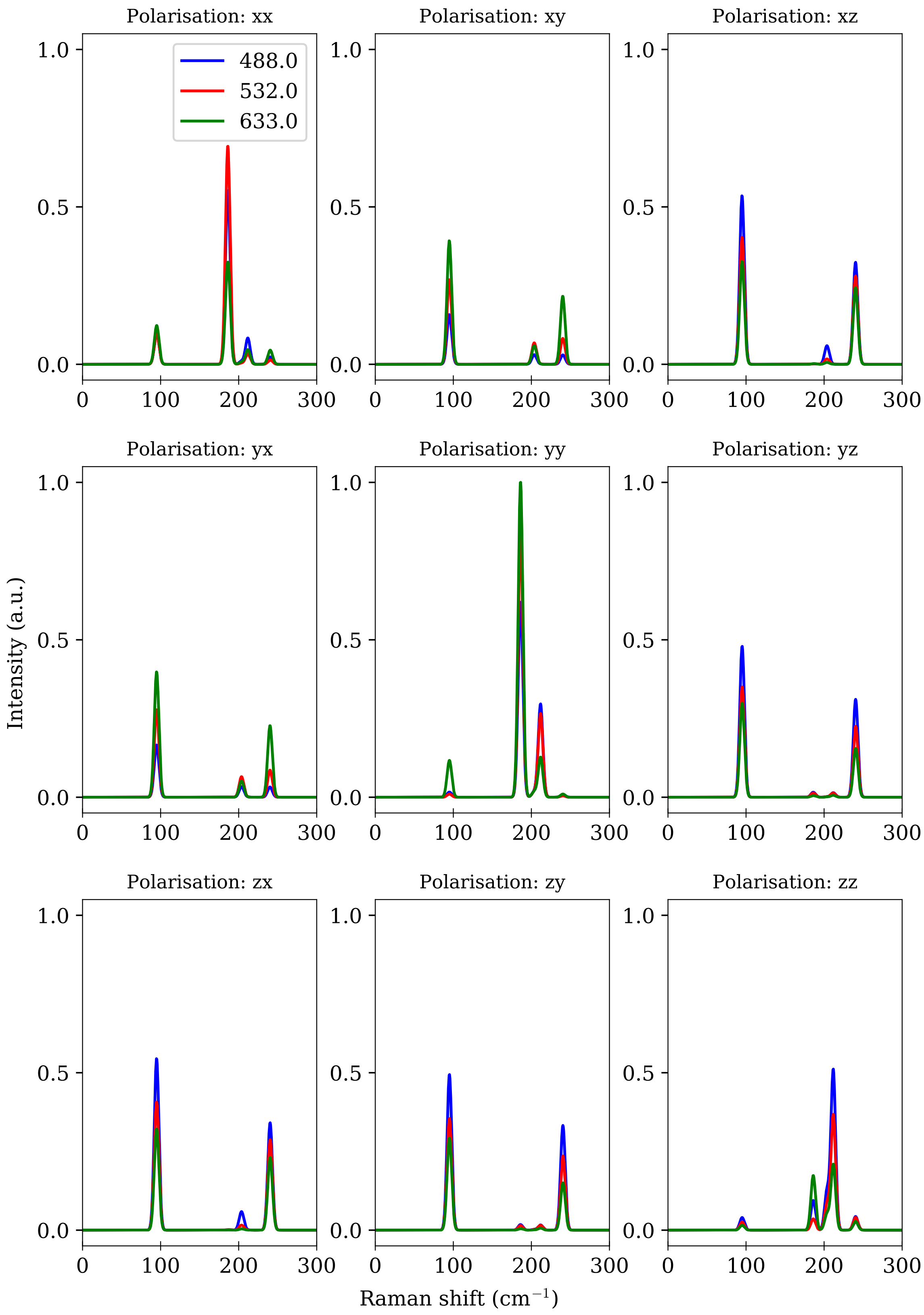

Supplementary Figure 476: Raman spectrum of Rh_2Se_2 with space group 12 and Wyckoff positions i-0.

Rh_2Te_2, 129, b-c-0

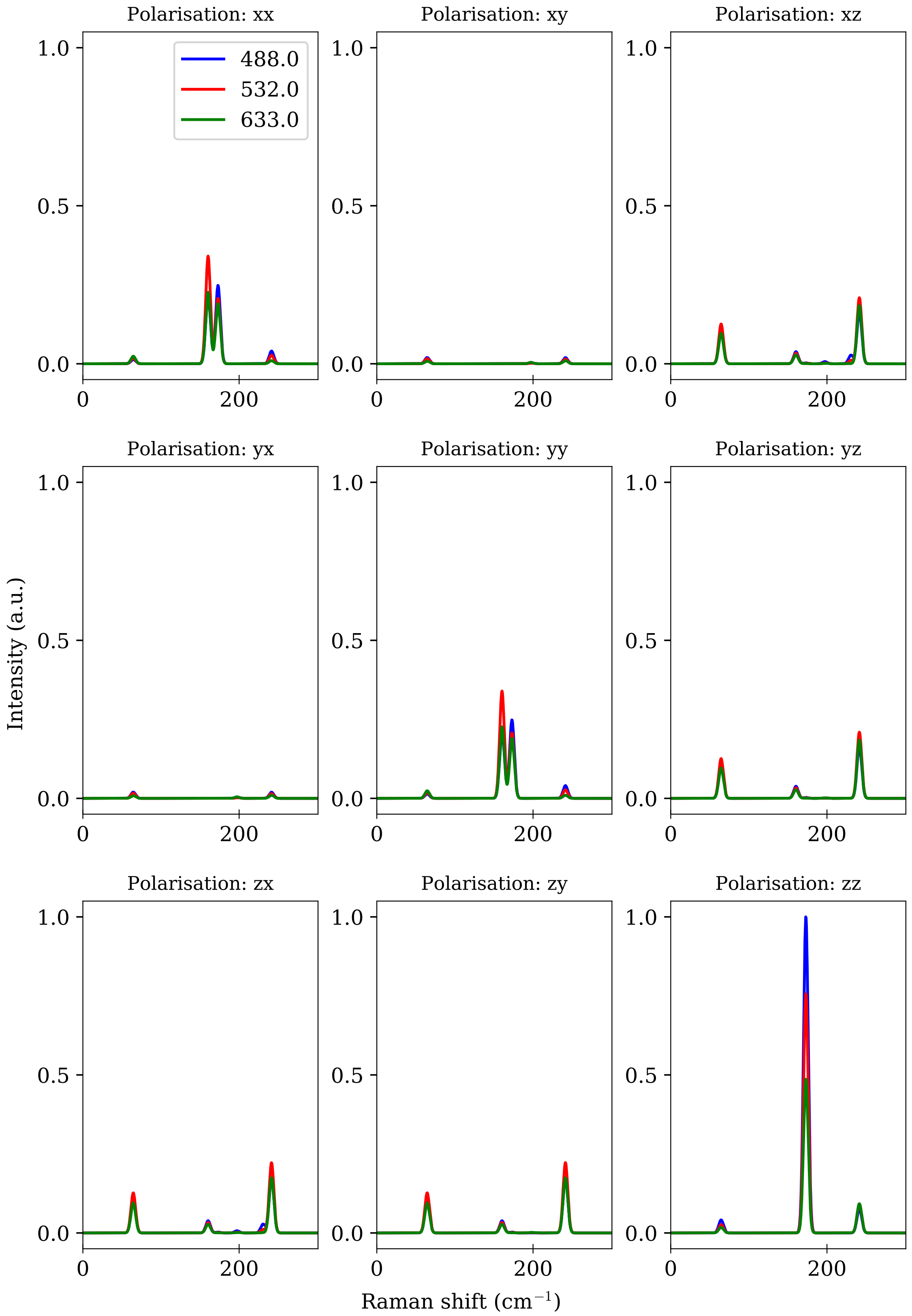

Supplementary Figure 477: Raman spectrum of Rh_2Te_2 with space group 129 and Wyckoff positions b-c-0.

Rh_2Te_2, 164, d-0

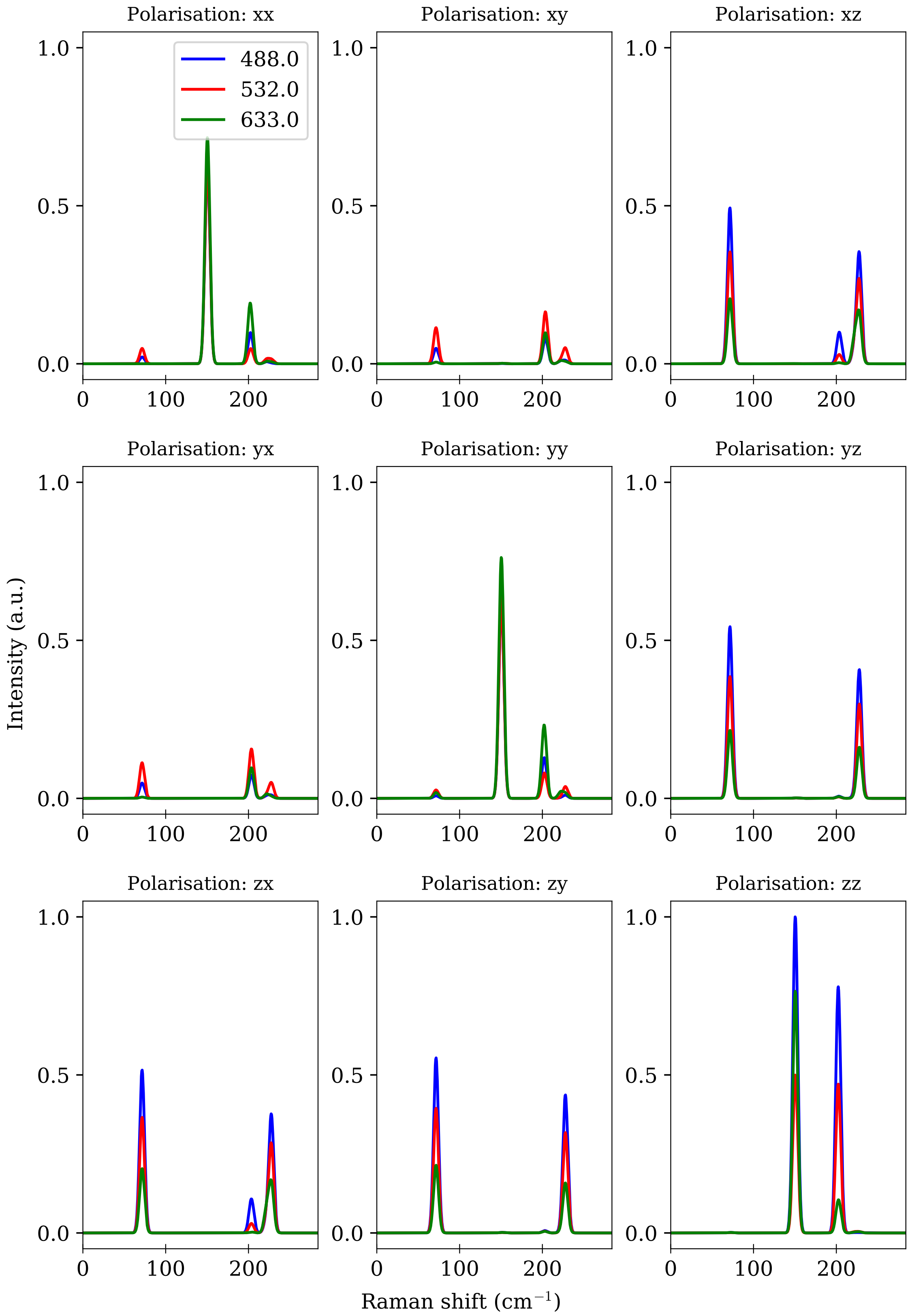

Supplementary Figure 478: Raman spectrum of Rh_2Te_2 with space group 164 and Wyckoff positions d-0.

Rh_2Te_2, 6, a-b-0

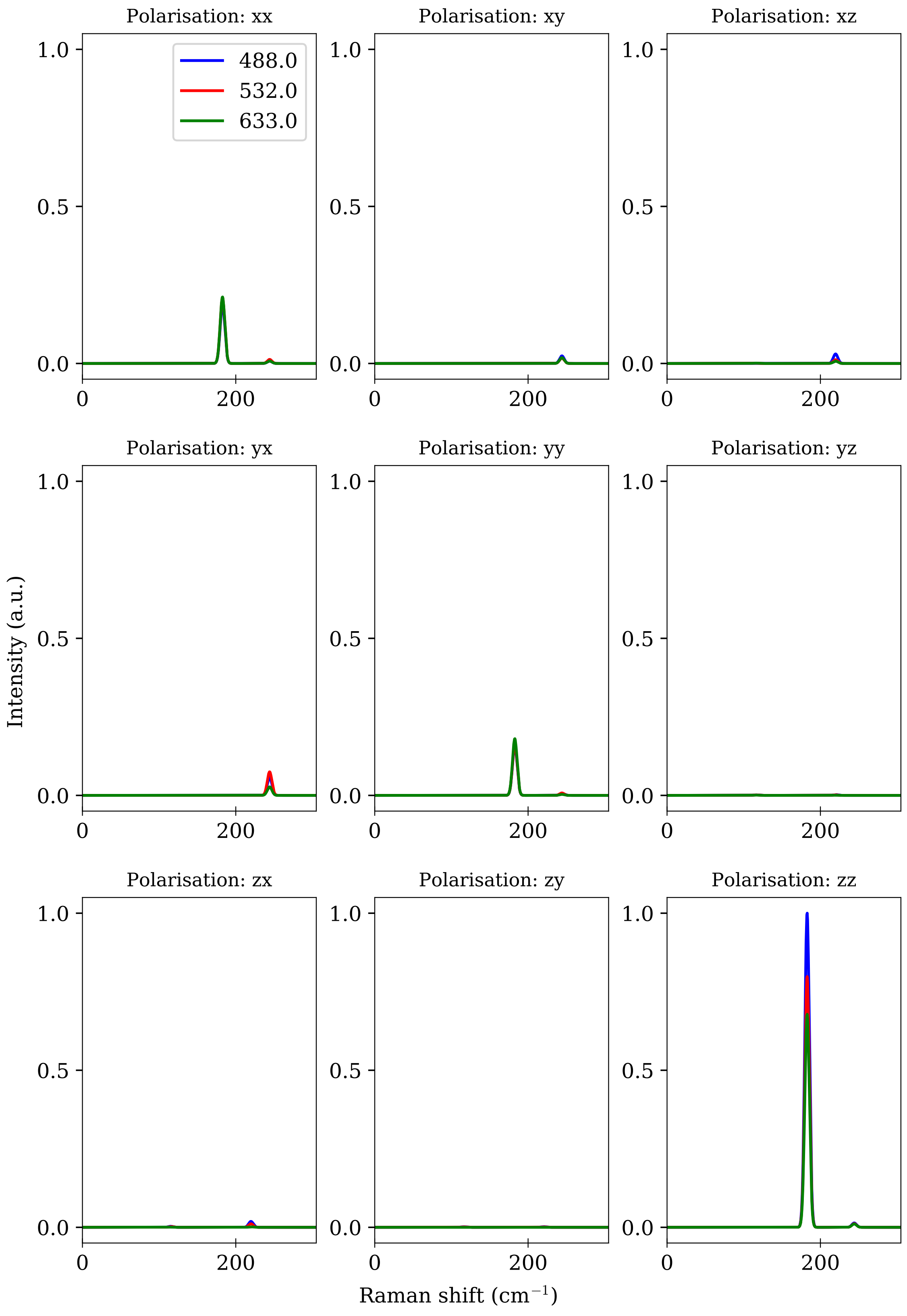

Supplementary Figure 479: Raman spectrum of Rh_2Te_2 with space group 6 and Wyckoff positions a-b-0.

RhH_2, 187, b-i-0

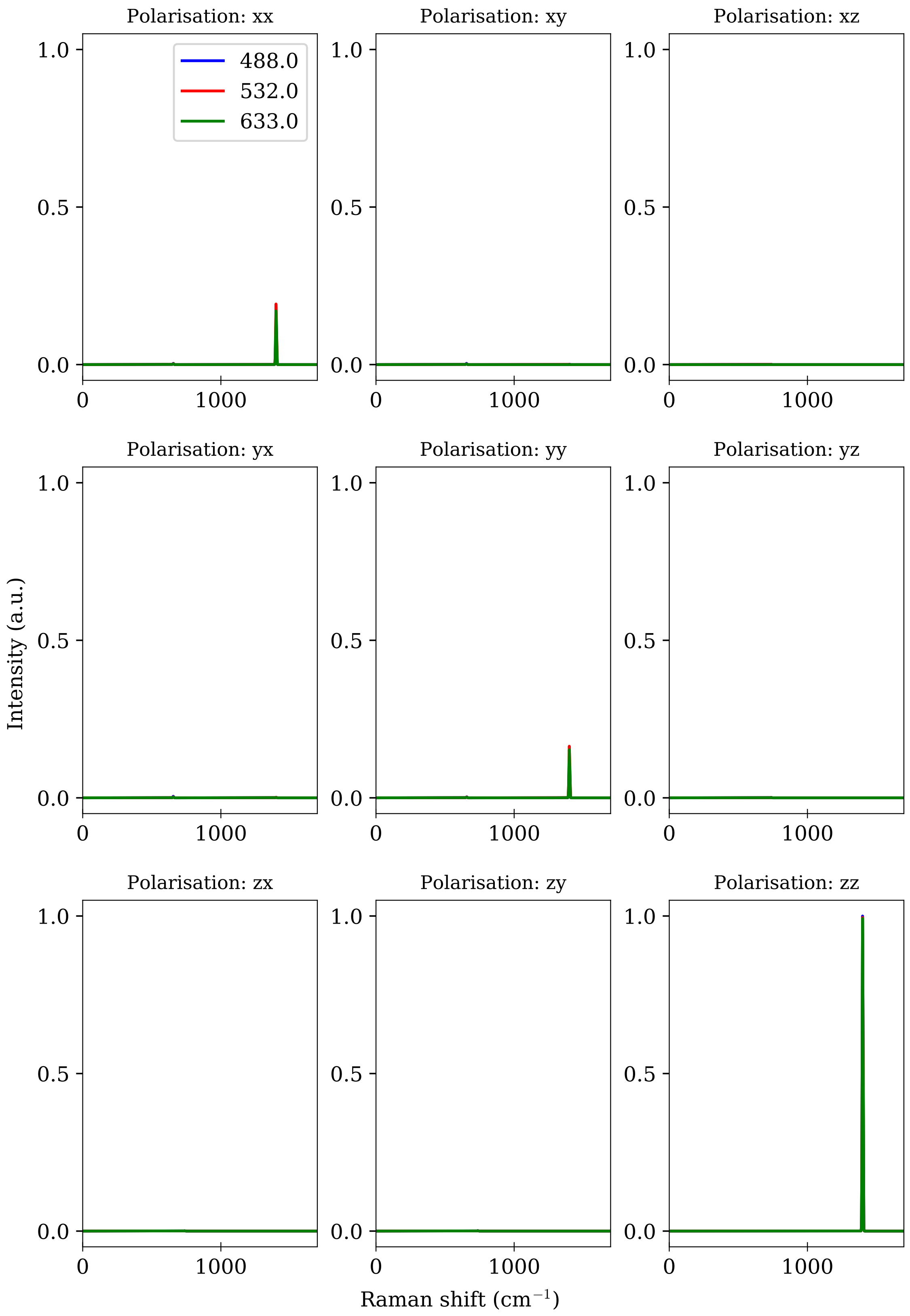

Supplementary Figure 480: Raman spectrum of RhH_2 with space group 187 and Wyckoff positions b-i-0.

RhO_2, 115, a-g-0

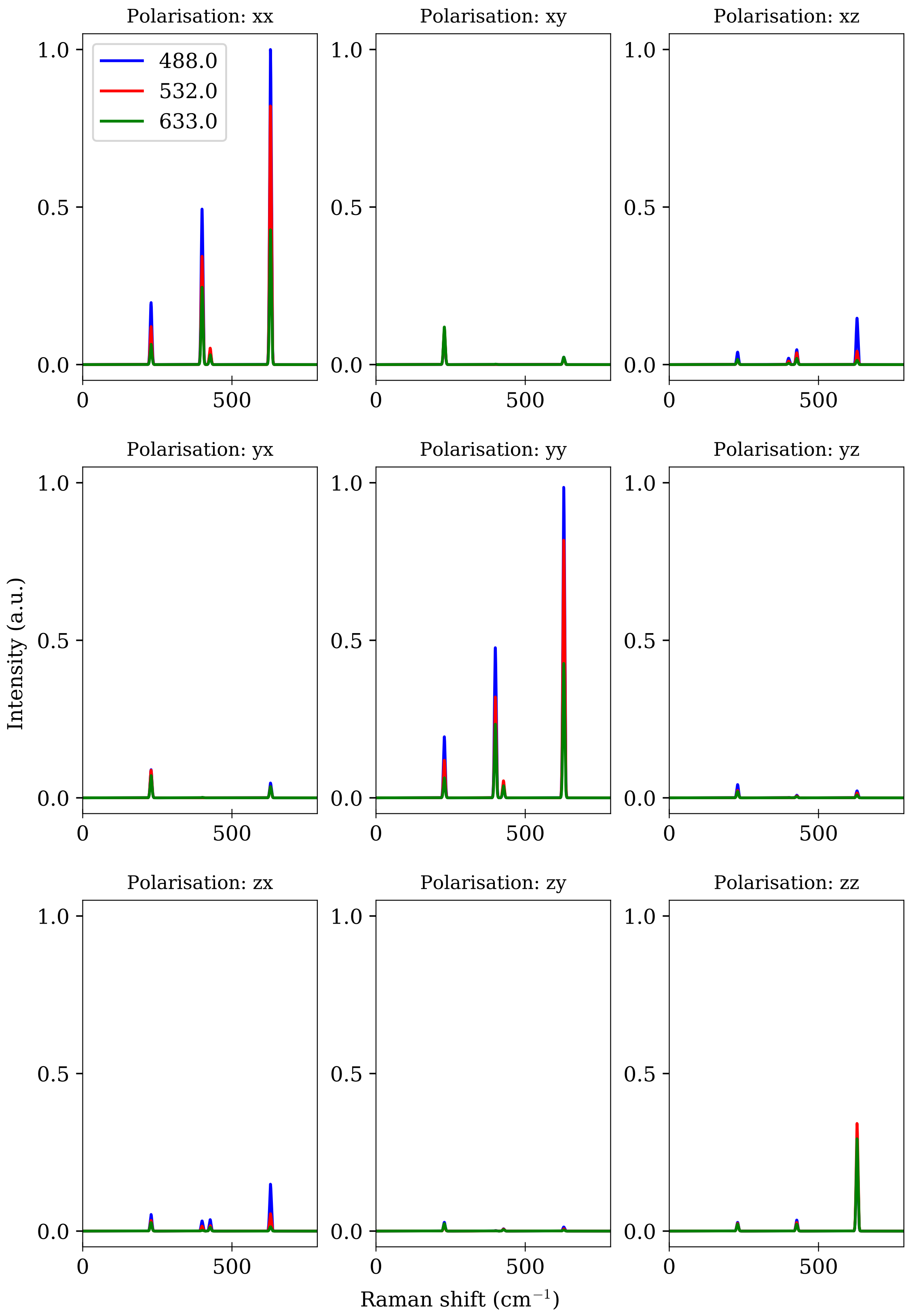

Supplementary Figure 481: Raman spectrum of RhO_2 with space group 115 and Wyckoff positions a-g-0.

$Ru_2Br_2O_2$, 59, a-b-0

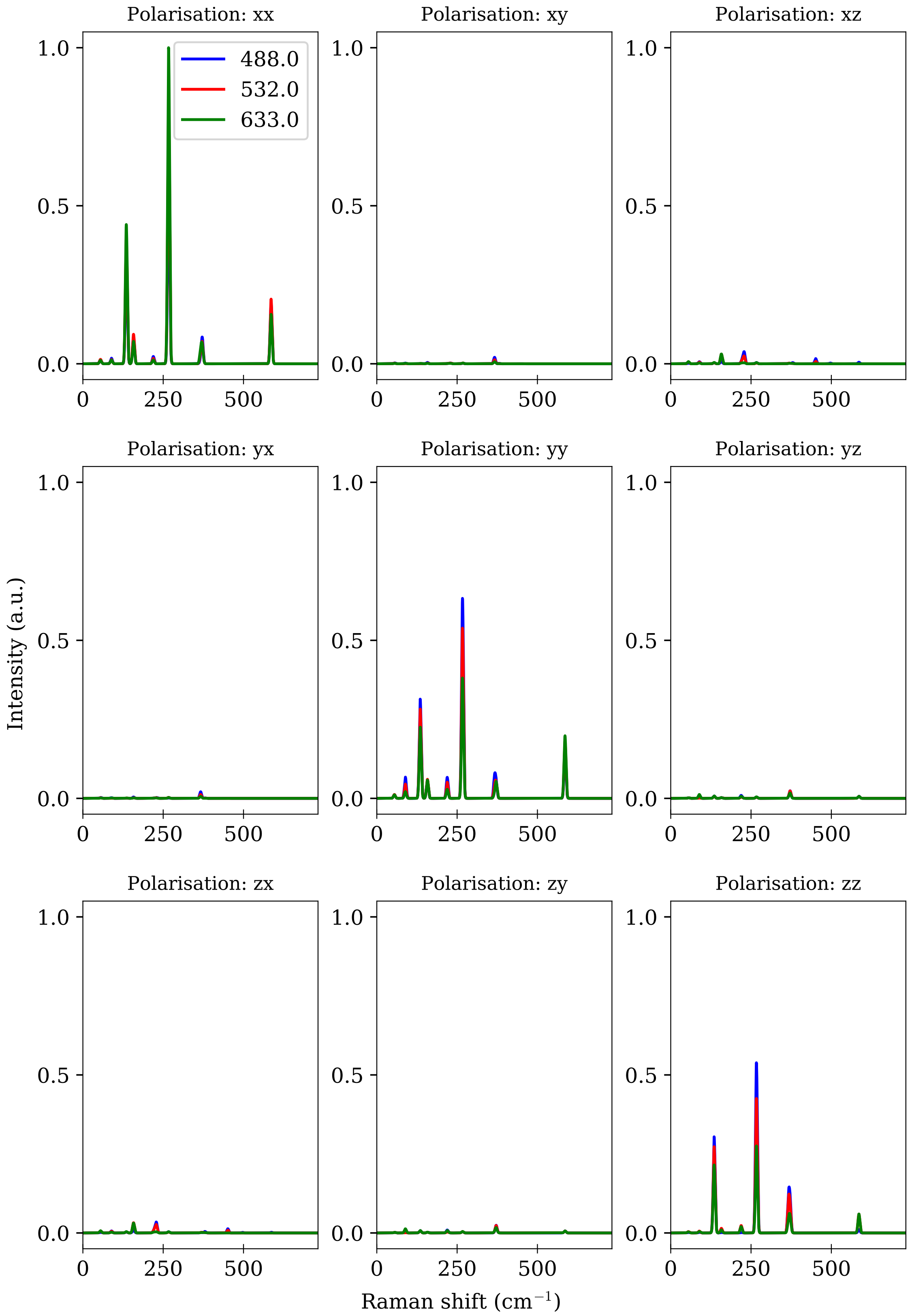

Supplementary Figure 482: Raman spectrum of $Ru_2Br_2O_2$ with space group 59 and Wyckoff positions a-b-0.

Ru_2Br_2, 12, i-0

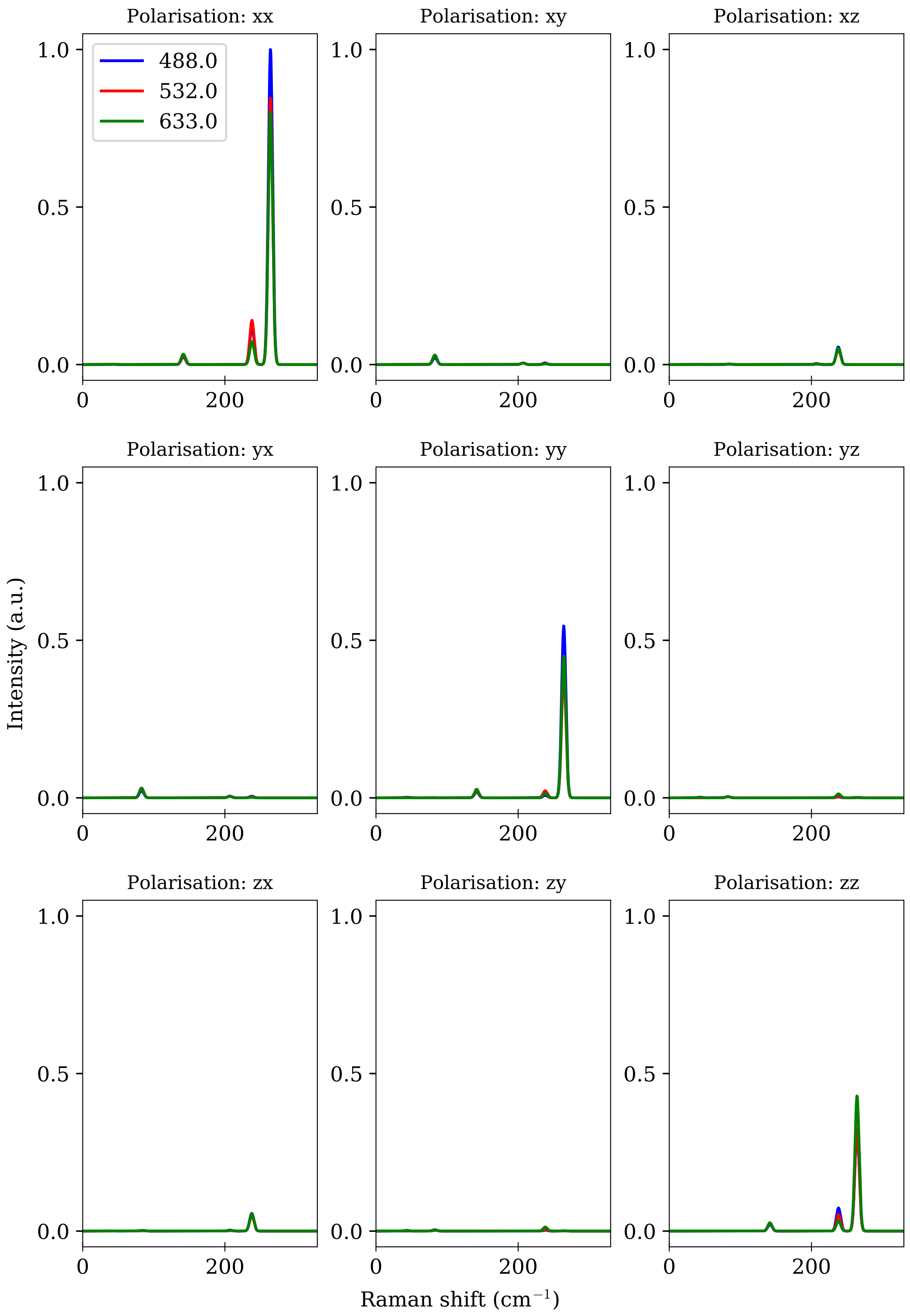

Supplementary Figure 483: Raman spectrum of Ru_2Br_2 with space group 12 and Wyckoff positions i-0.

Ru_2Cl_2, 12, i-0

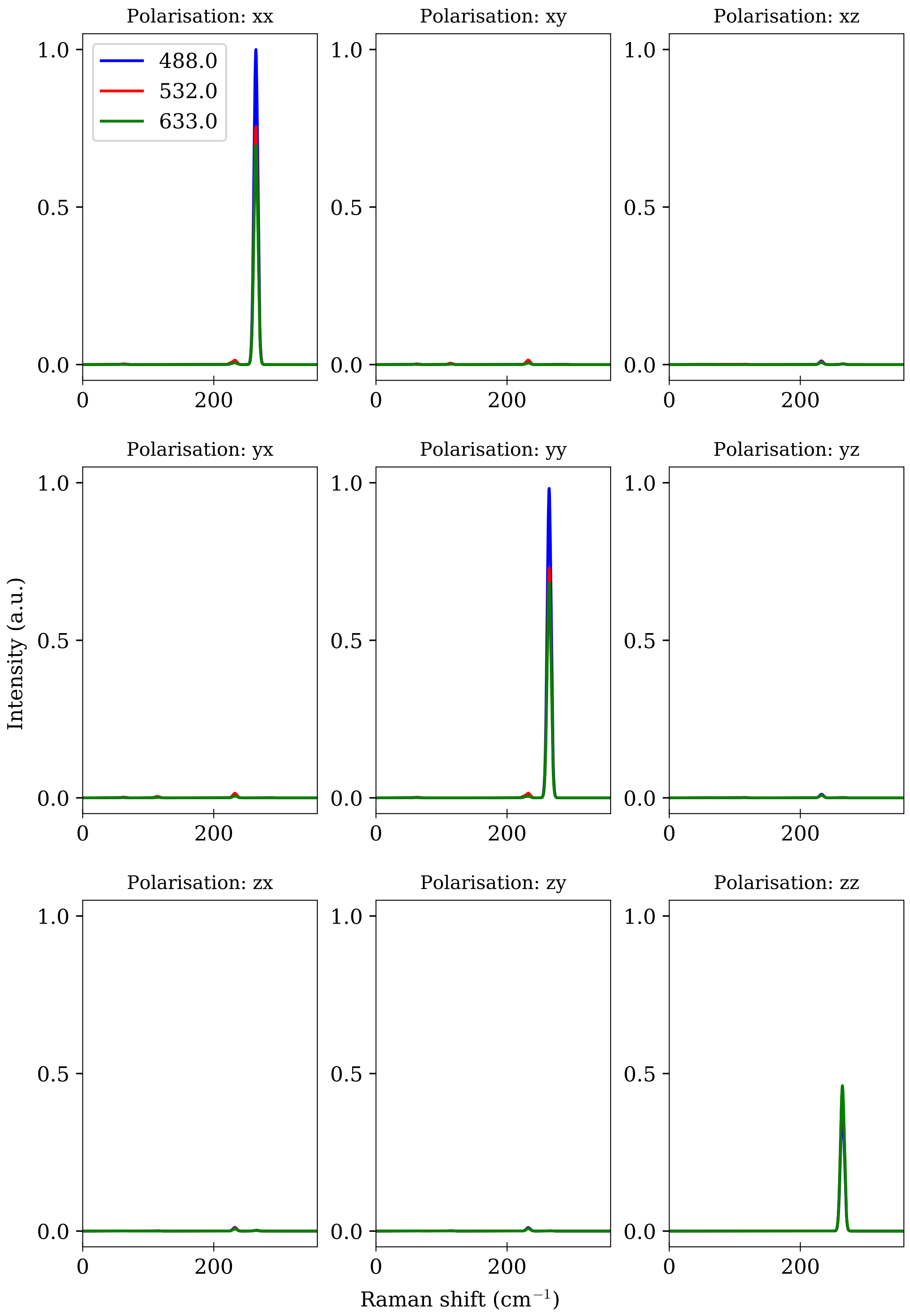

Supplementary Figure 484: Raman spectrum of Ru_2Cl_2 with space group 12 and Wyckoff positions i-0.

Ru_2F_2, 1, a-0

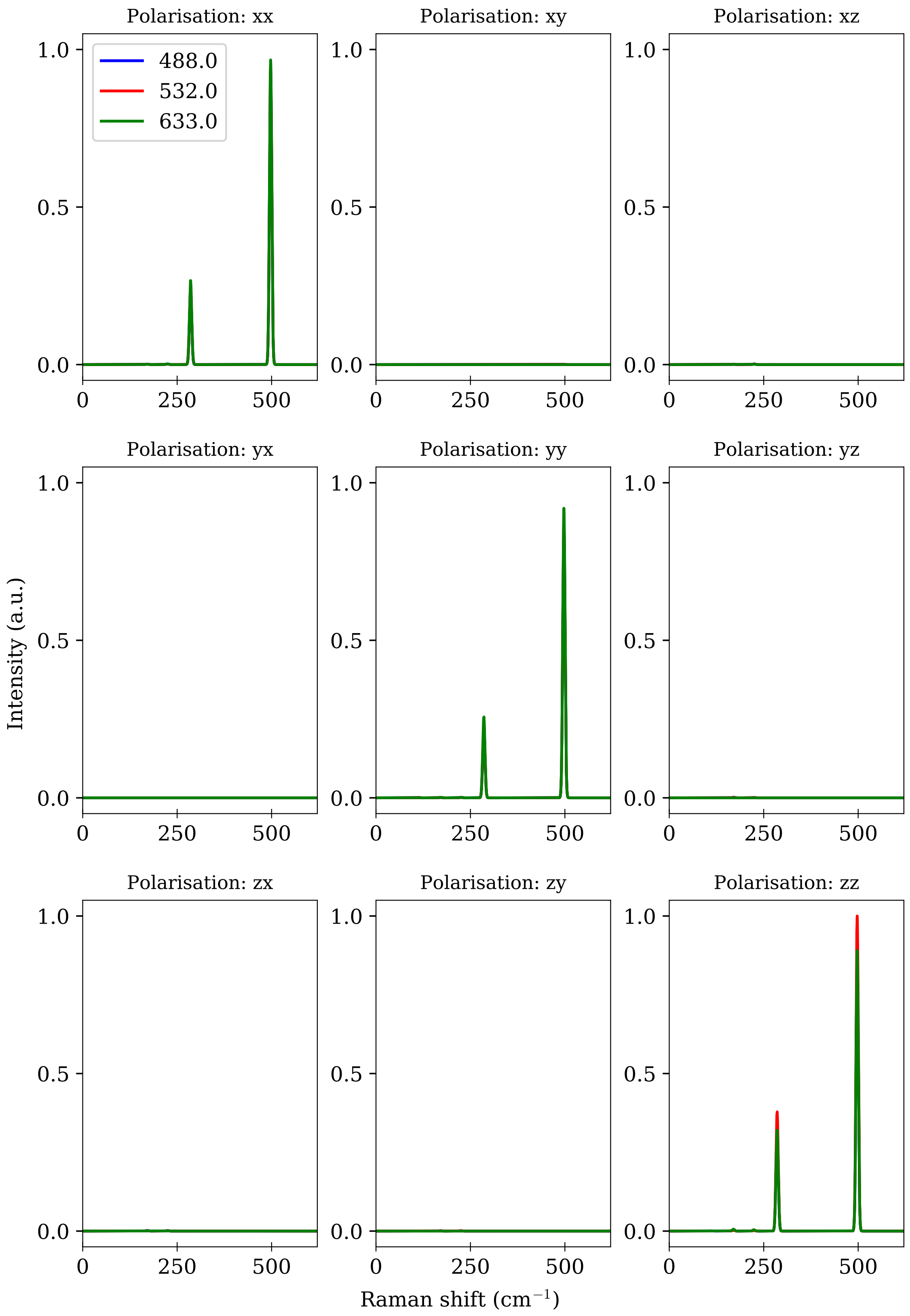

Supplementary Figure 485: Raman spectrum of Ru_2F_2 with space group 1 and Wyckoff positions a-0.

Ru_2I_2, 12, i-0

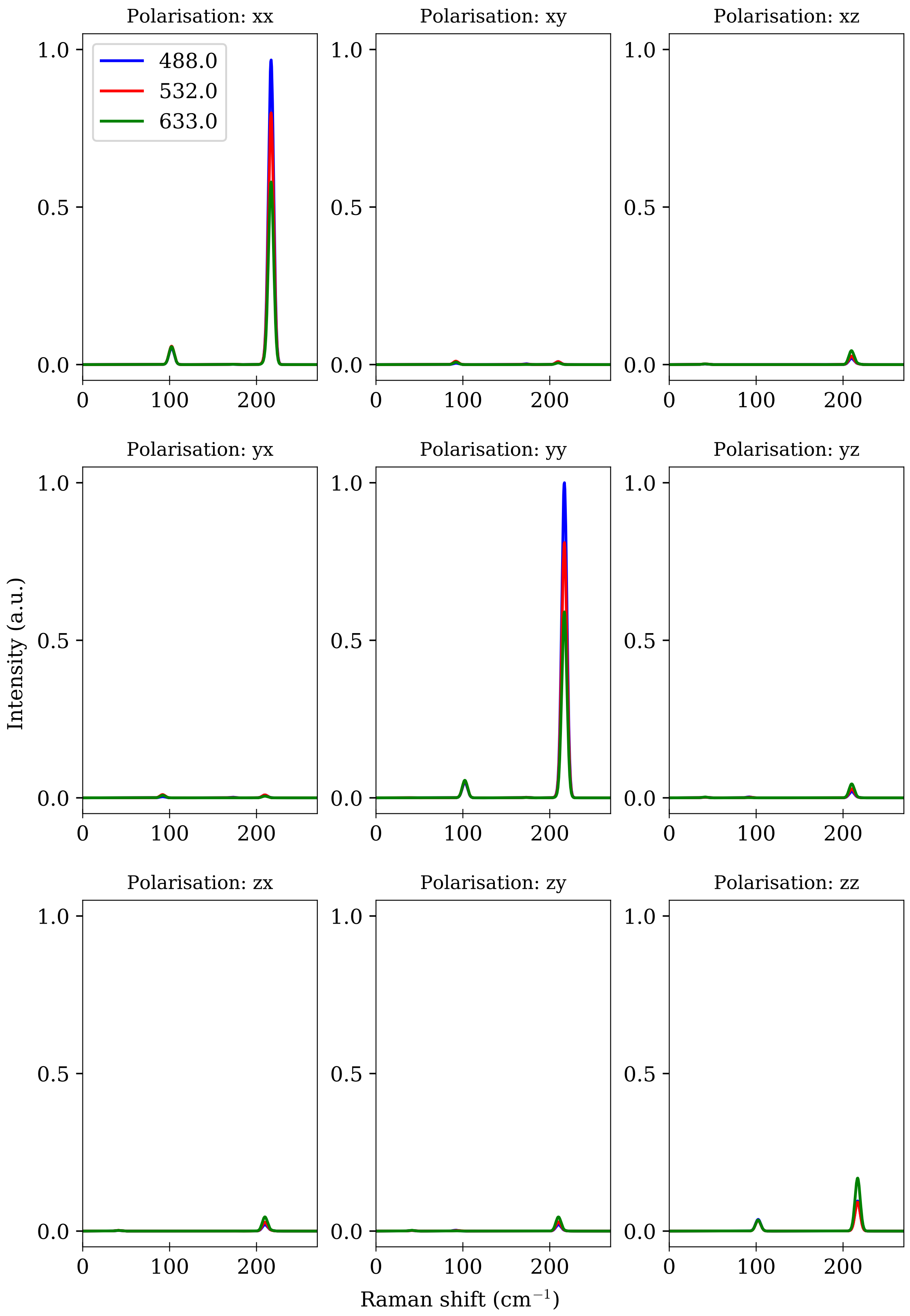

Supplementary Figure 486: Raman spectrum of Ru_2I_2 with space group 12 and Wyckoff positions i-0.

Ru_2O_4, 11, e-0

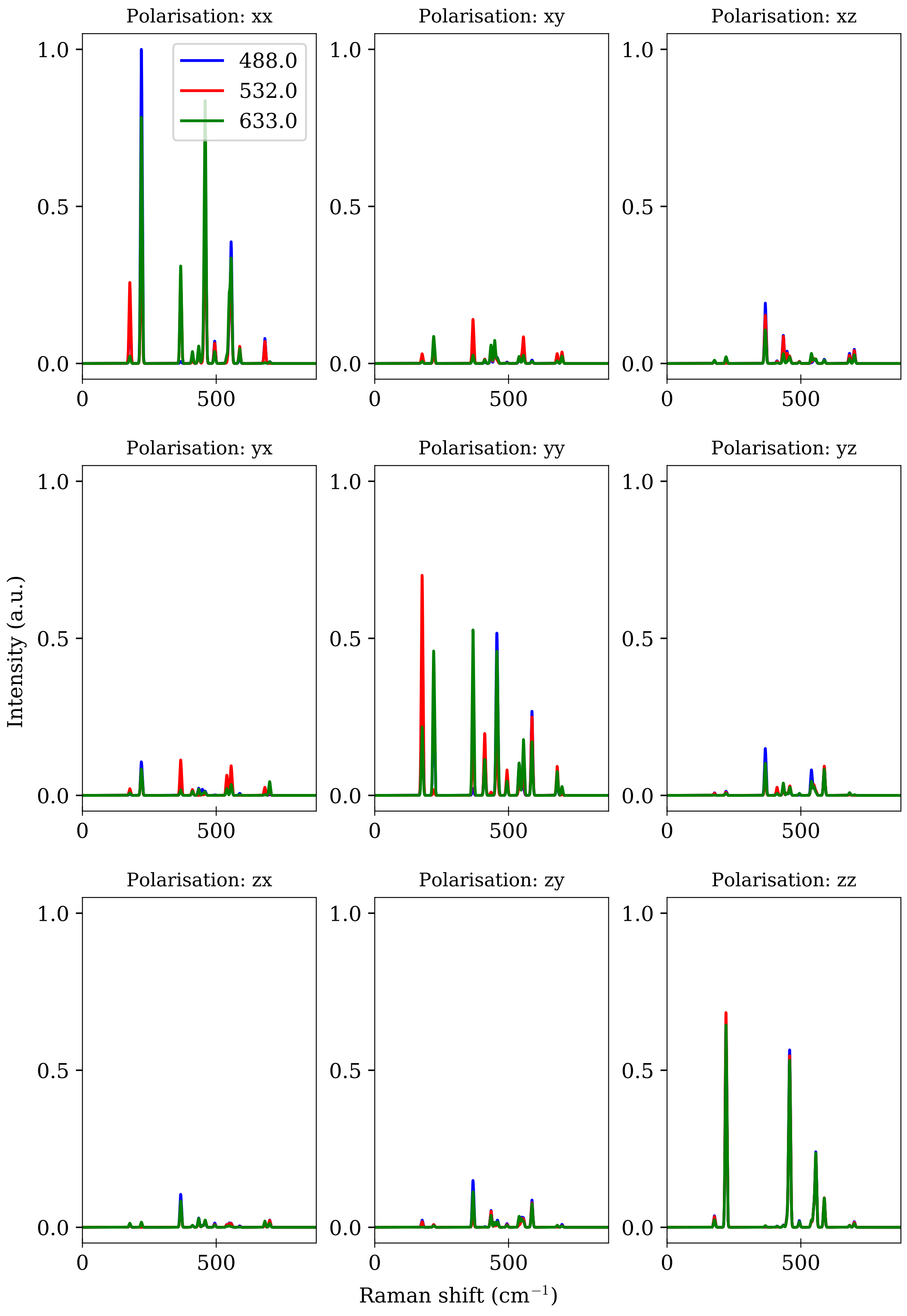

Supplementary Figure 487: Raman spectrum of Ru_2O_4 with space group 11 and Wyckoff positions e-0.

Ru_2S_2, 129, b-c-0

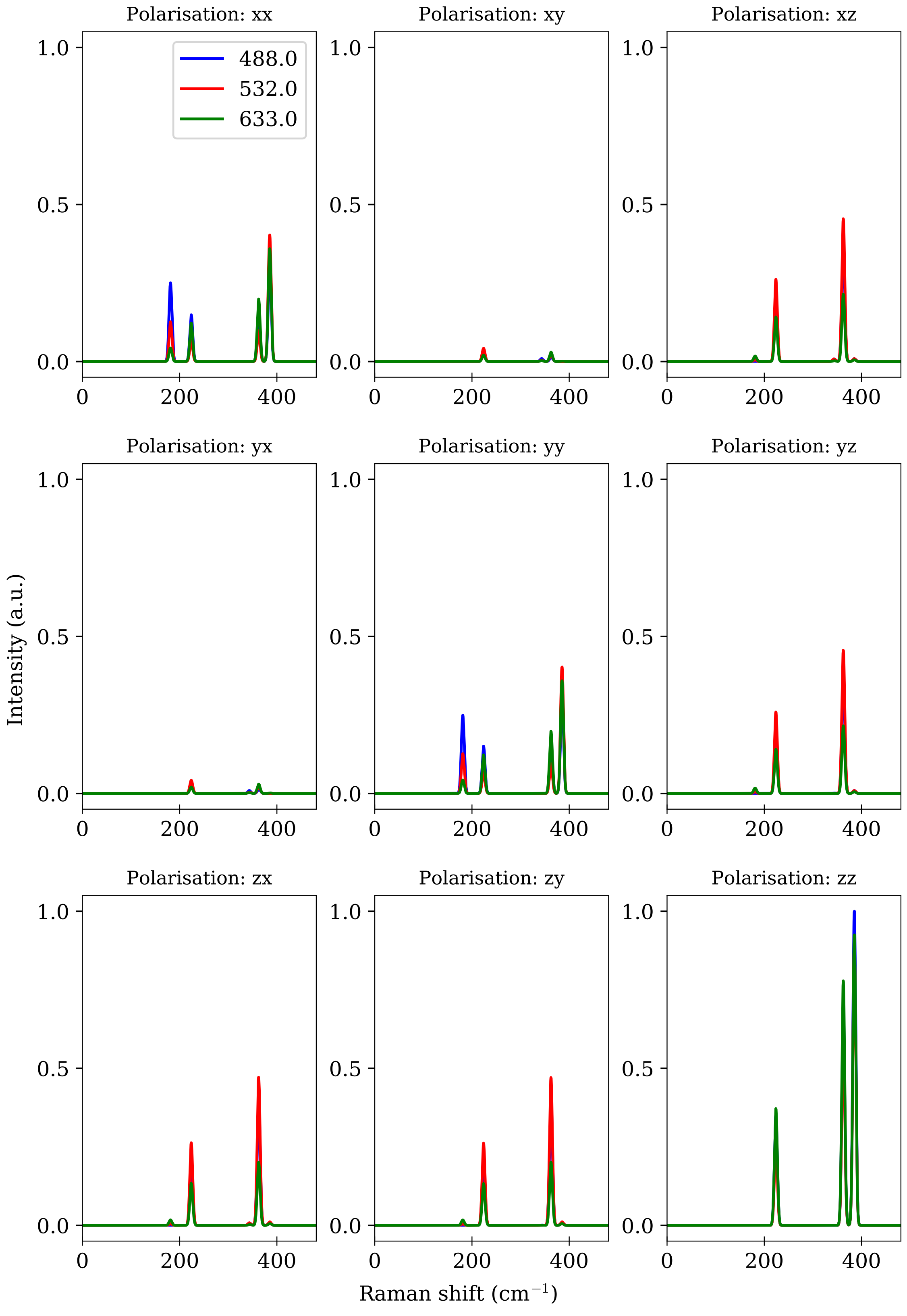

Supplementary Figure 488: Raman spectrum of Ru_2S_2 with space group 129 and Wyckoff positions b-c-0.

Ru_2S_2, 164, d-0

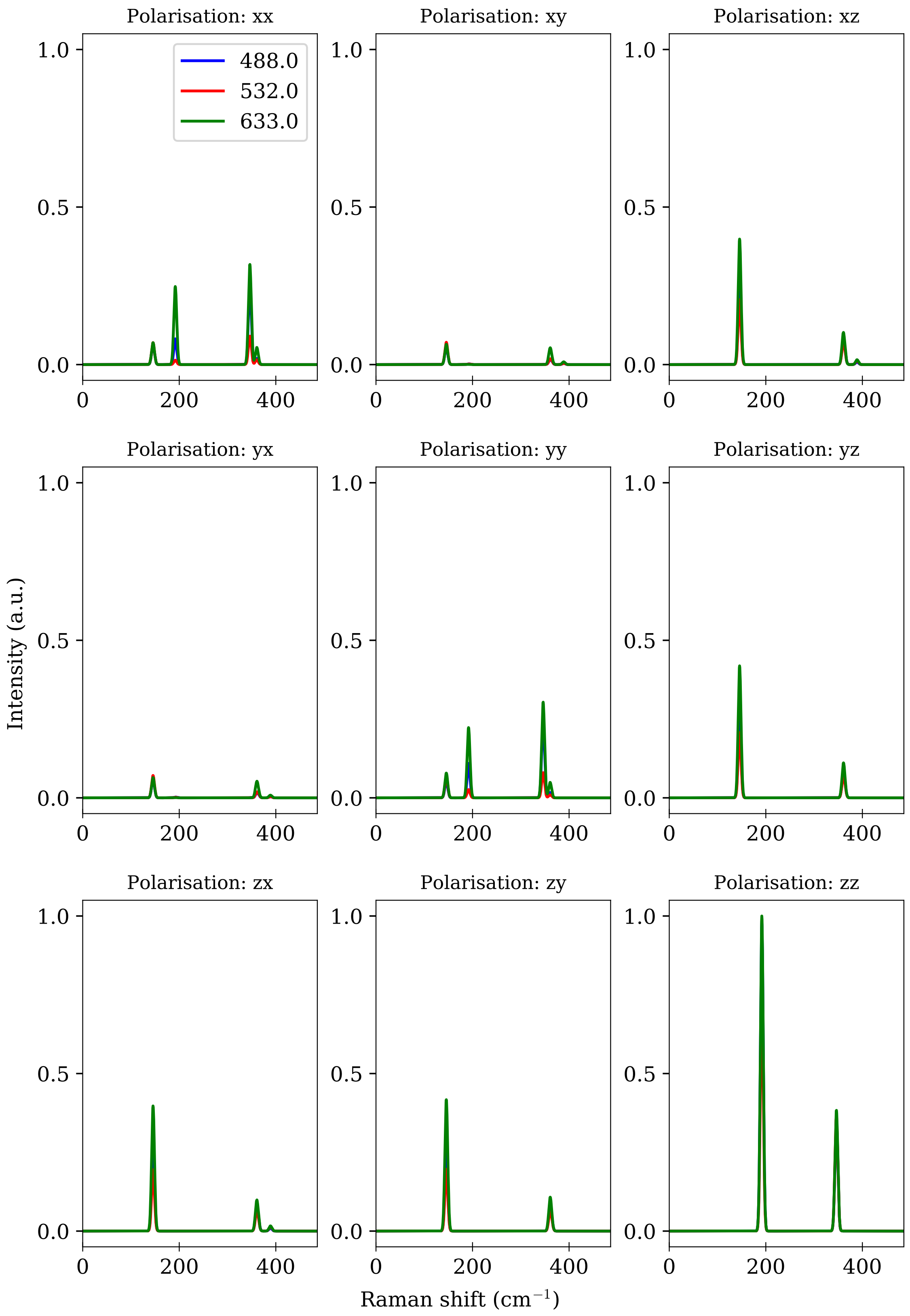

Supplementary Figure 489: Raman spectrum of Ru_2S_2 with space group 164 and Wyckoff positions d-0.

Ru_2S_4, 11, e-0

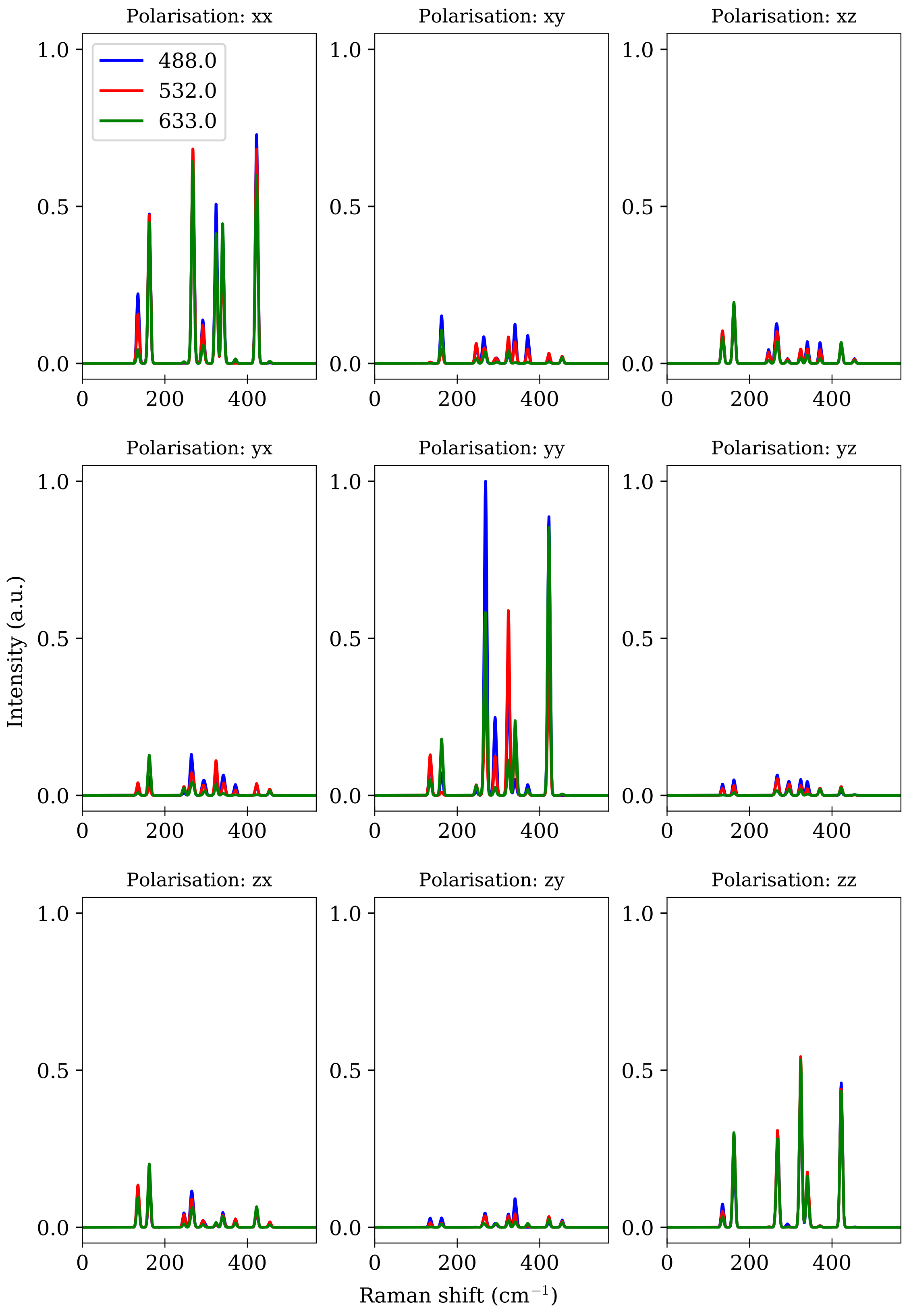

Supplementary Figure 490: Raman spectrum of Ru_2S_4 with space group 11 and Wyckoff positions e-0.

Ru_2Se_2, 129, b-c-0

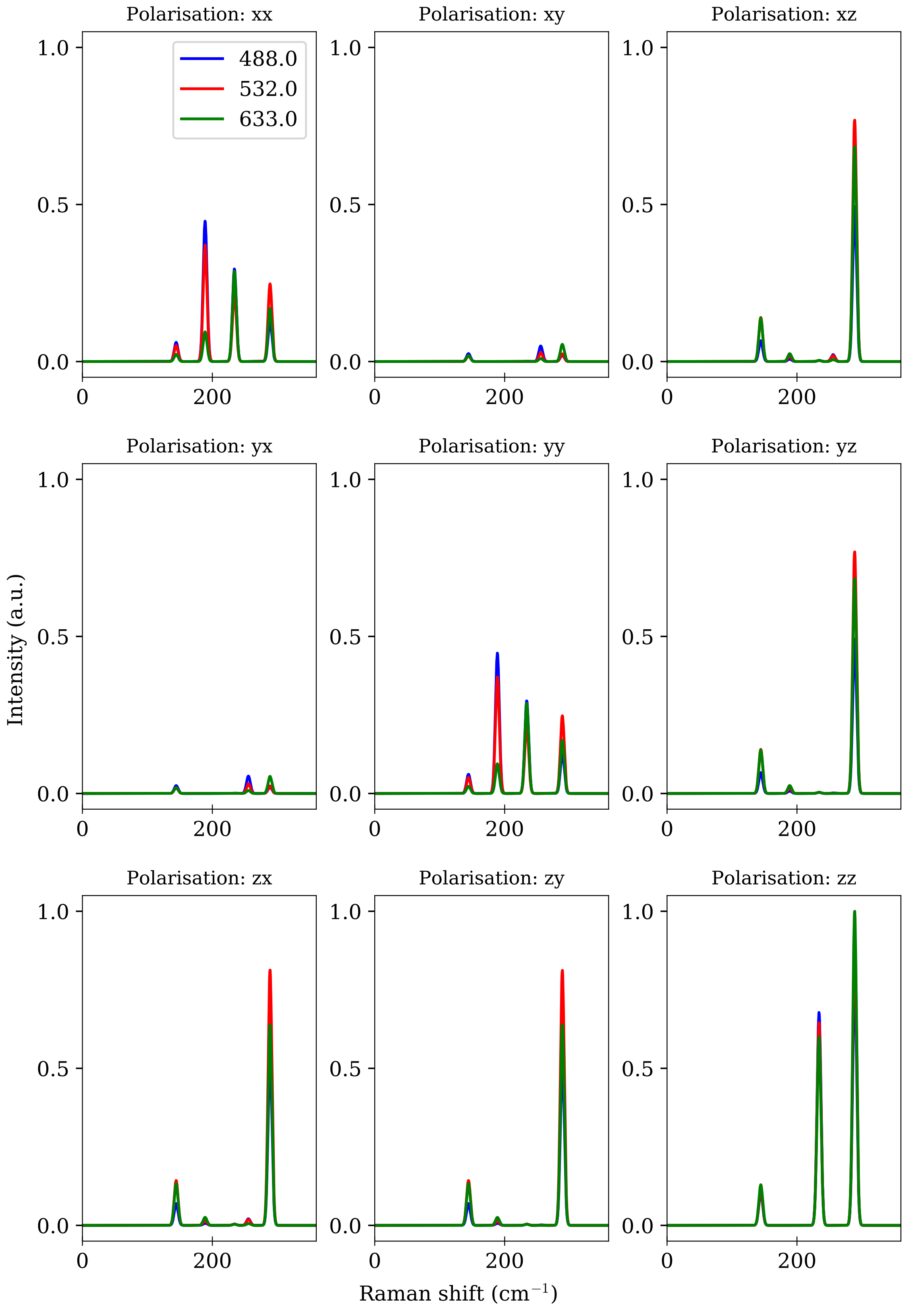

Supplementary Figure 491: Raman spectrum of Ru_2Se_2 with space group 129 and Wyckoff positions b-c-0.

Ru_2Se_2, 164, d-0

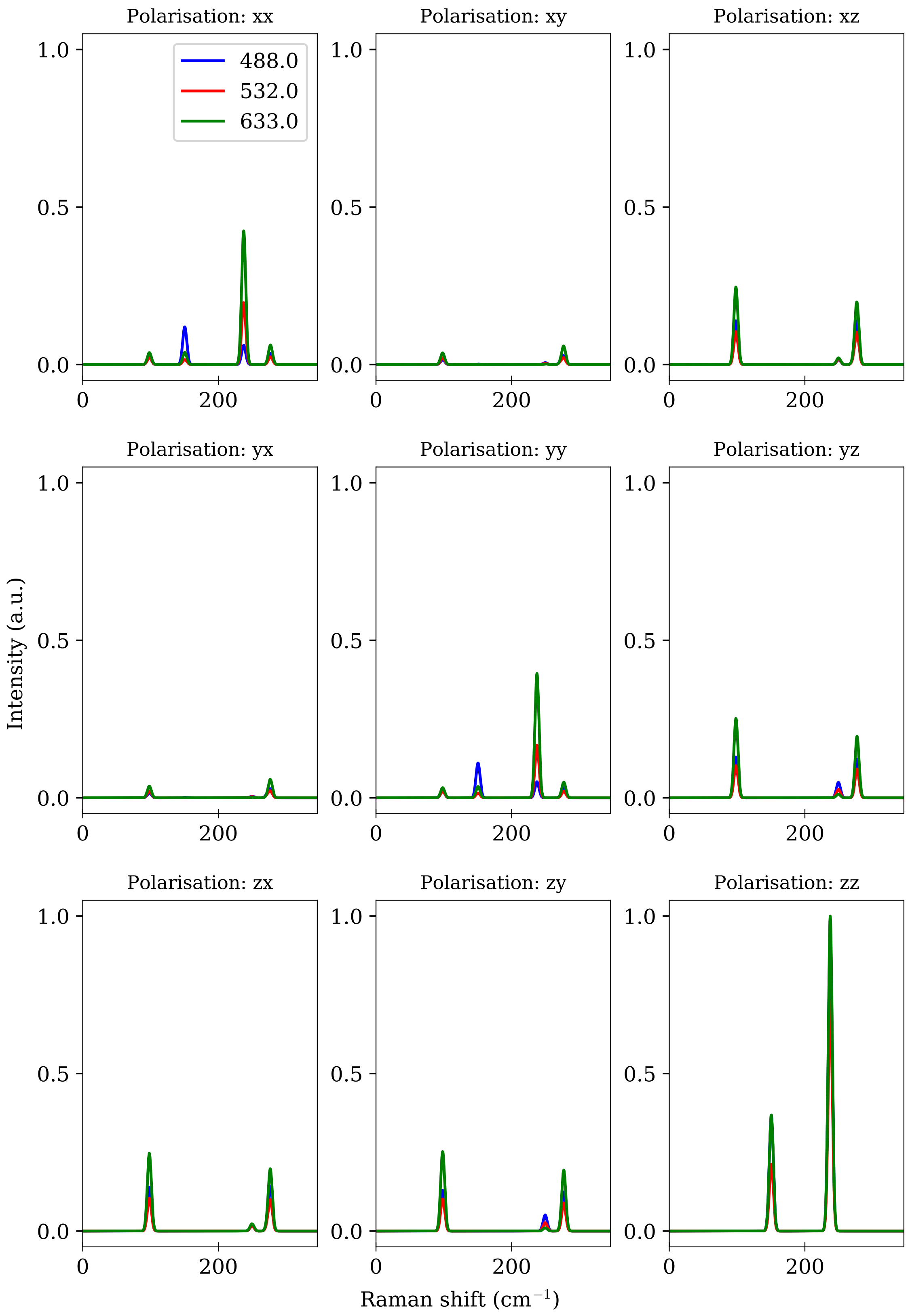

Supplementary Figure 492: Raman spectrum of Ru_2Se_2 with space group 164 and Wyckoff positions d-0.

Ru_2Se_4, 11, e-0

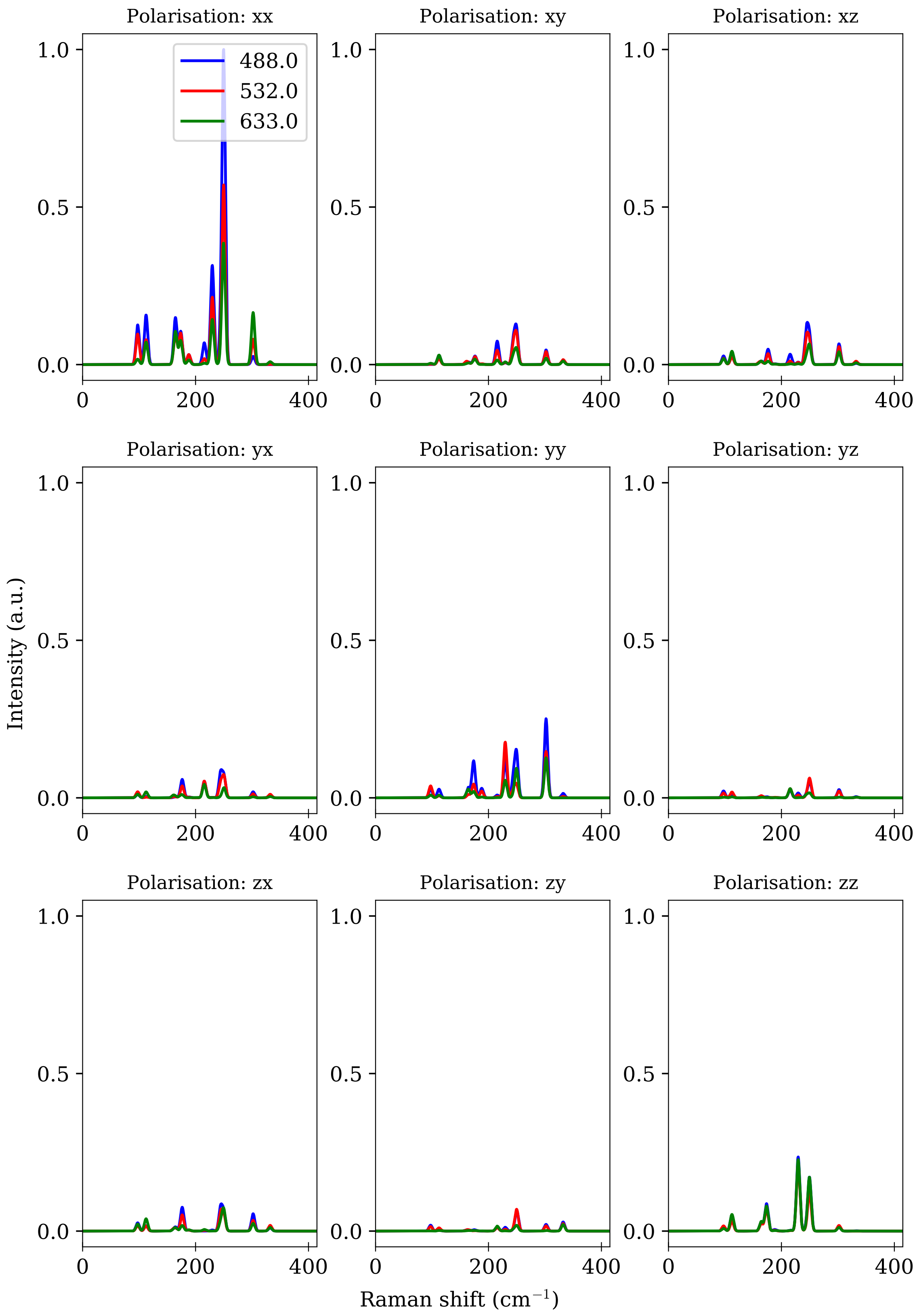

Supplementary Figure 493: Raman spectrum of Ru_2Se_4 with space group 11 and Wyckoff positions e-0.

Ru_2Te_2, 129, b-c-0

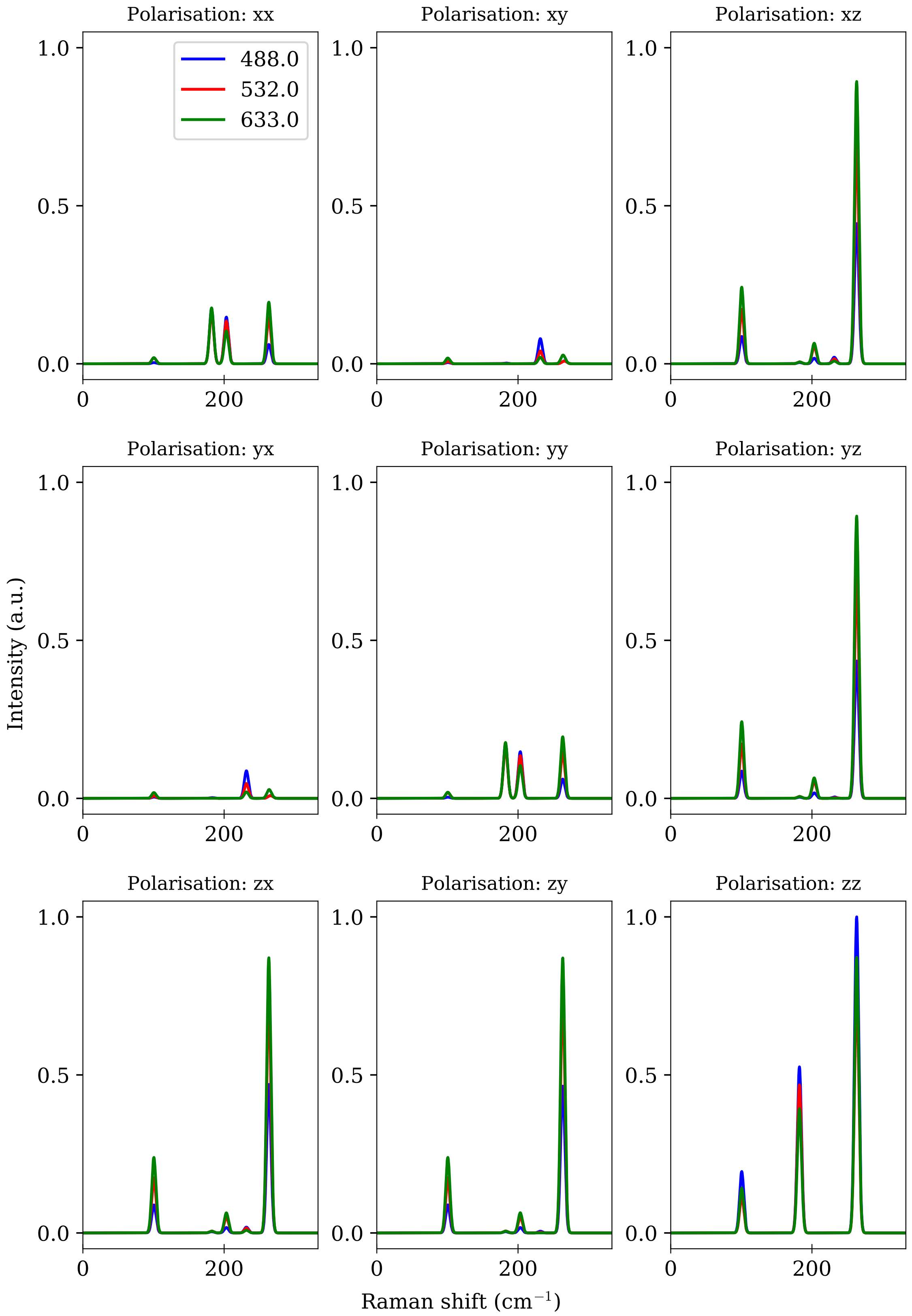

Supplementary Figure 494: Raman spectrum of Ru_2Te_2 with space group 129 and Wyckoff positions b-c-0.

Ru_2Te_2, 164, d-0

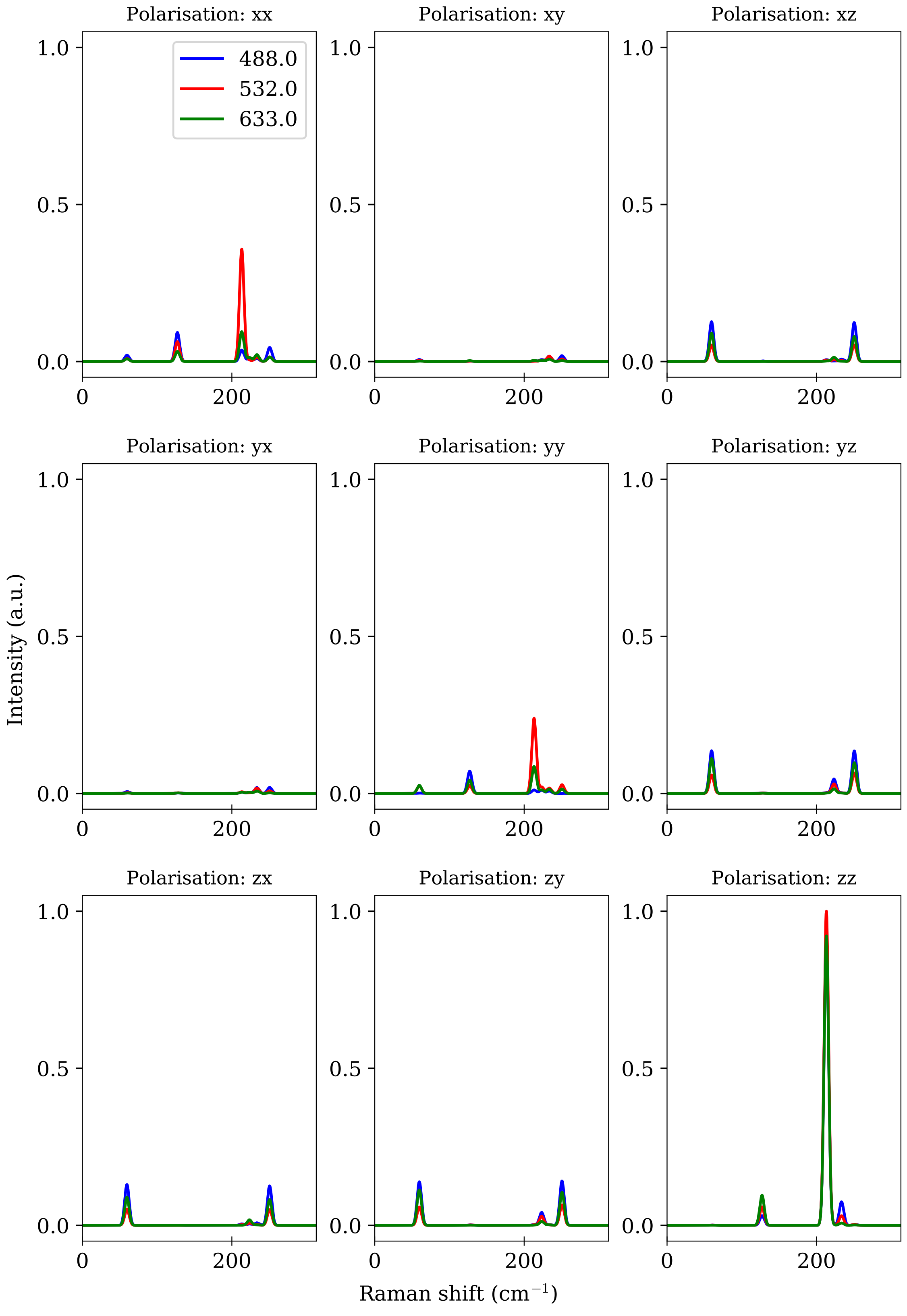

Supplementary Figure 495: Raman spectrum of Ru_2Te_2 with space group 164 and Wyckoff positions d-0.

Ru_2Te_4, 11, e-0

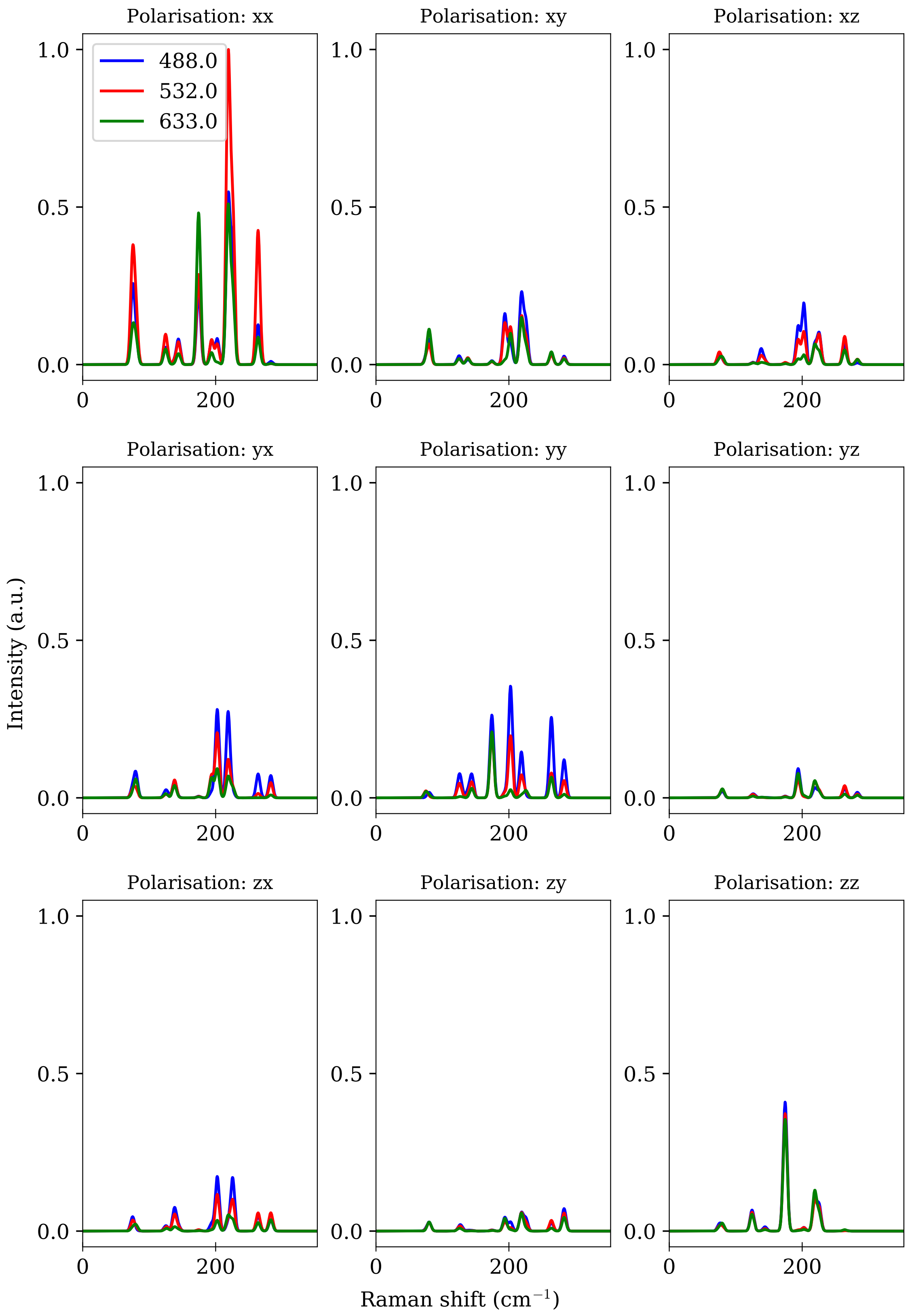

Supplementary Figure 496: Raman spectrum of Ru_2Te_4 with space group 11 and Wyckoff positions e-0.

$RuBr_2$, 164, b-d-0

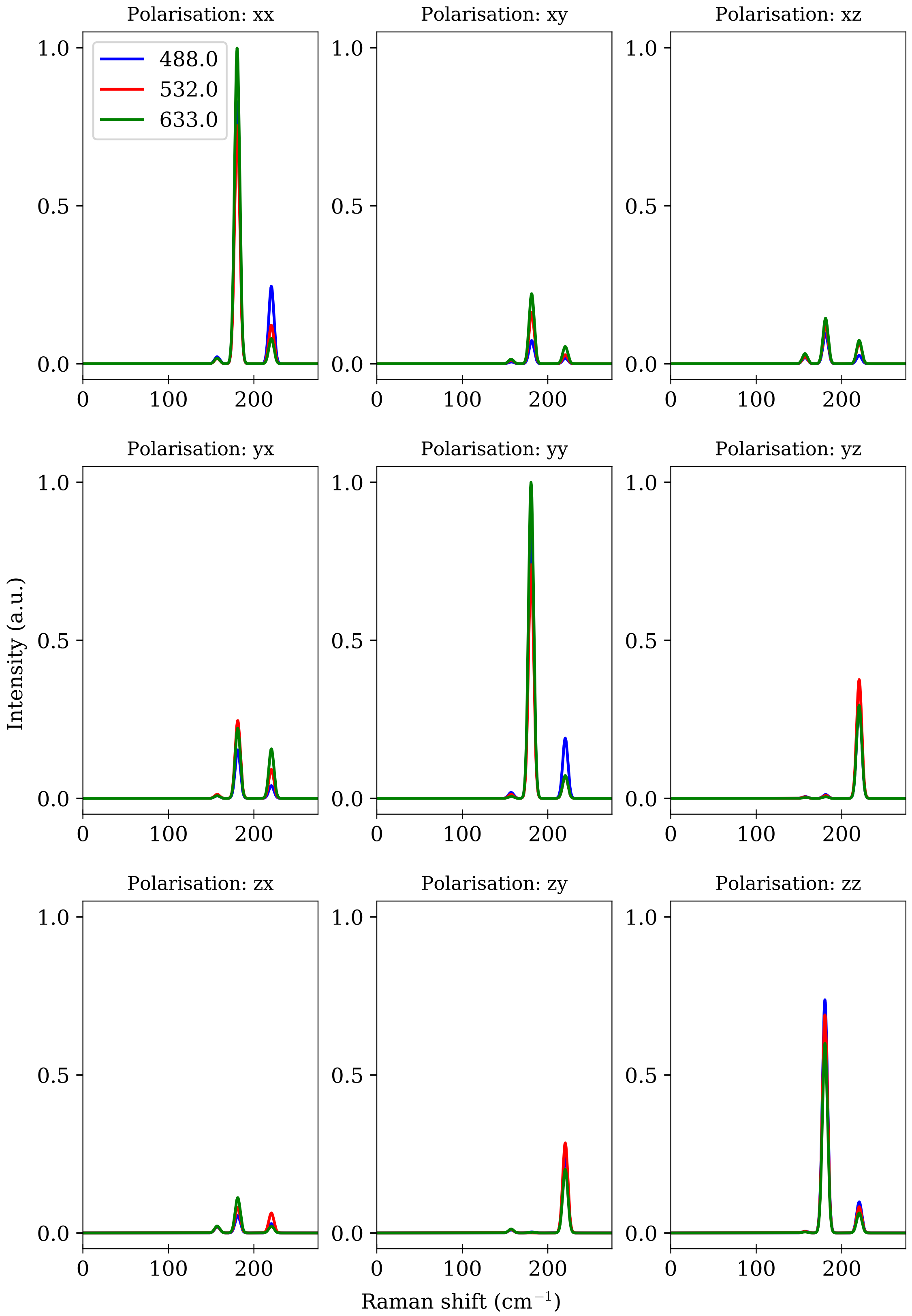

Supplementary Figure 497: Raman spectrum of $RuBr_2$ with space group 164 and Wyckoff positions b-d-0.

$RuCl_2$, 164, b-d-0

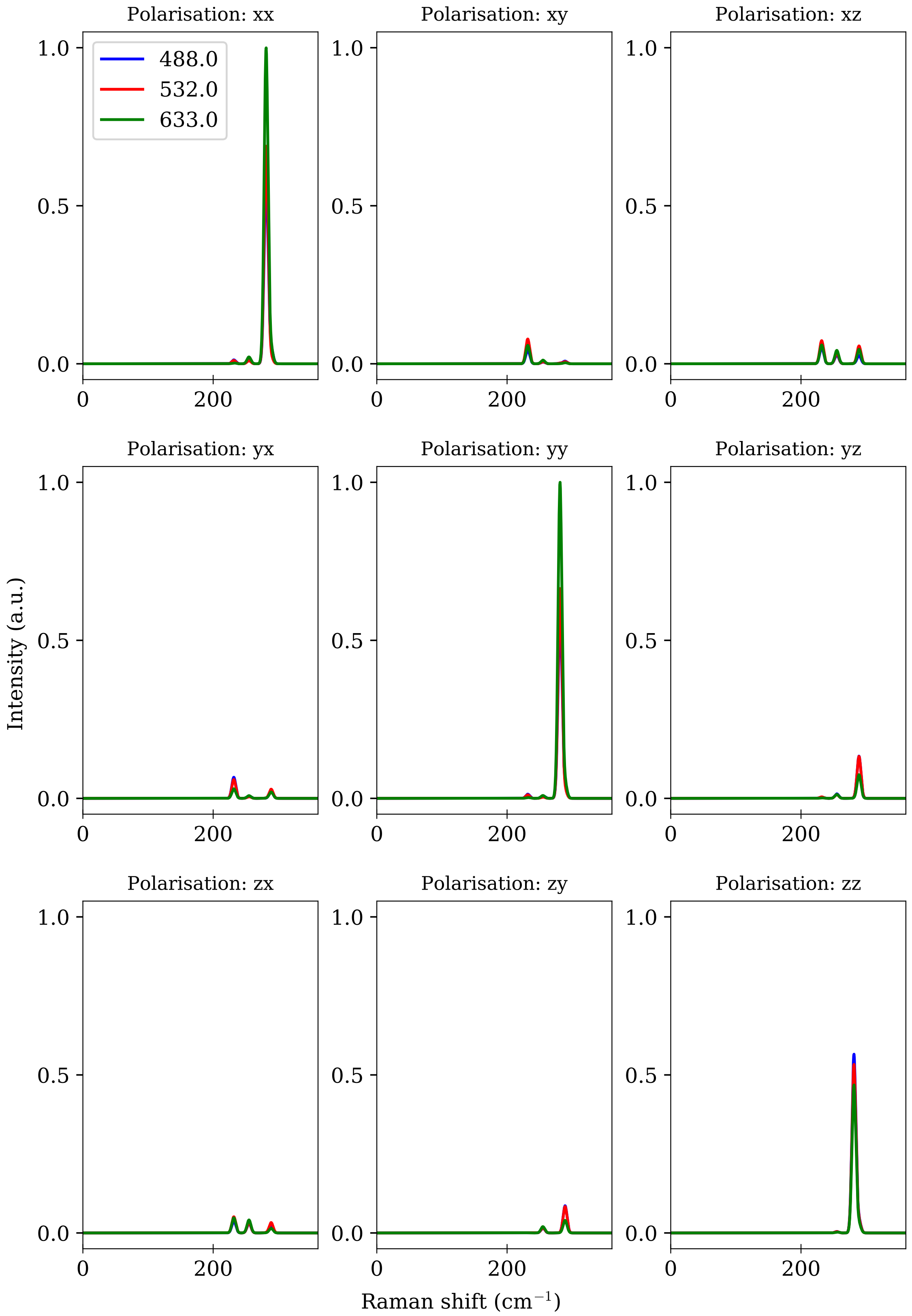

Supplementary Figure 498: Raman spectrum of $RuCl_2$ with space group 164 and Wyckoff positions b-d-0.

RuH_2, 187, b-i-0

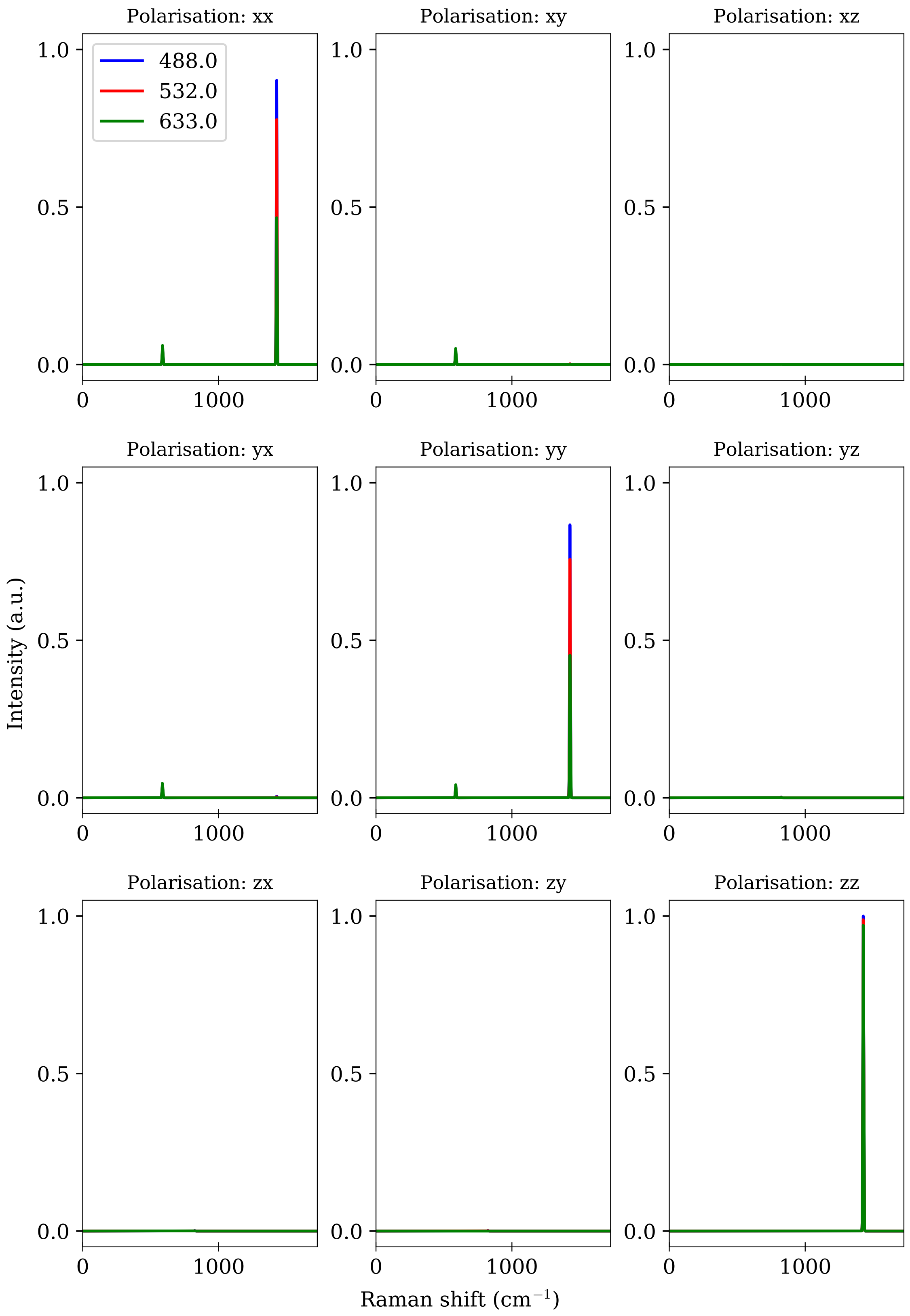

Supplementary Figure 499: Raman spectrum of RuH_2 with space group 187 and Wyckoff positions b-i-0.

RuI_2, 164, b-d-0

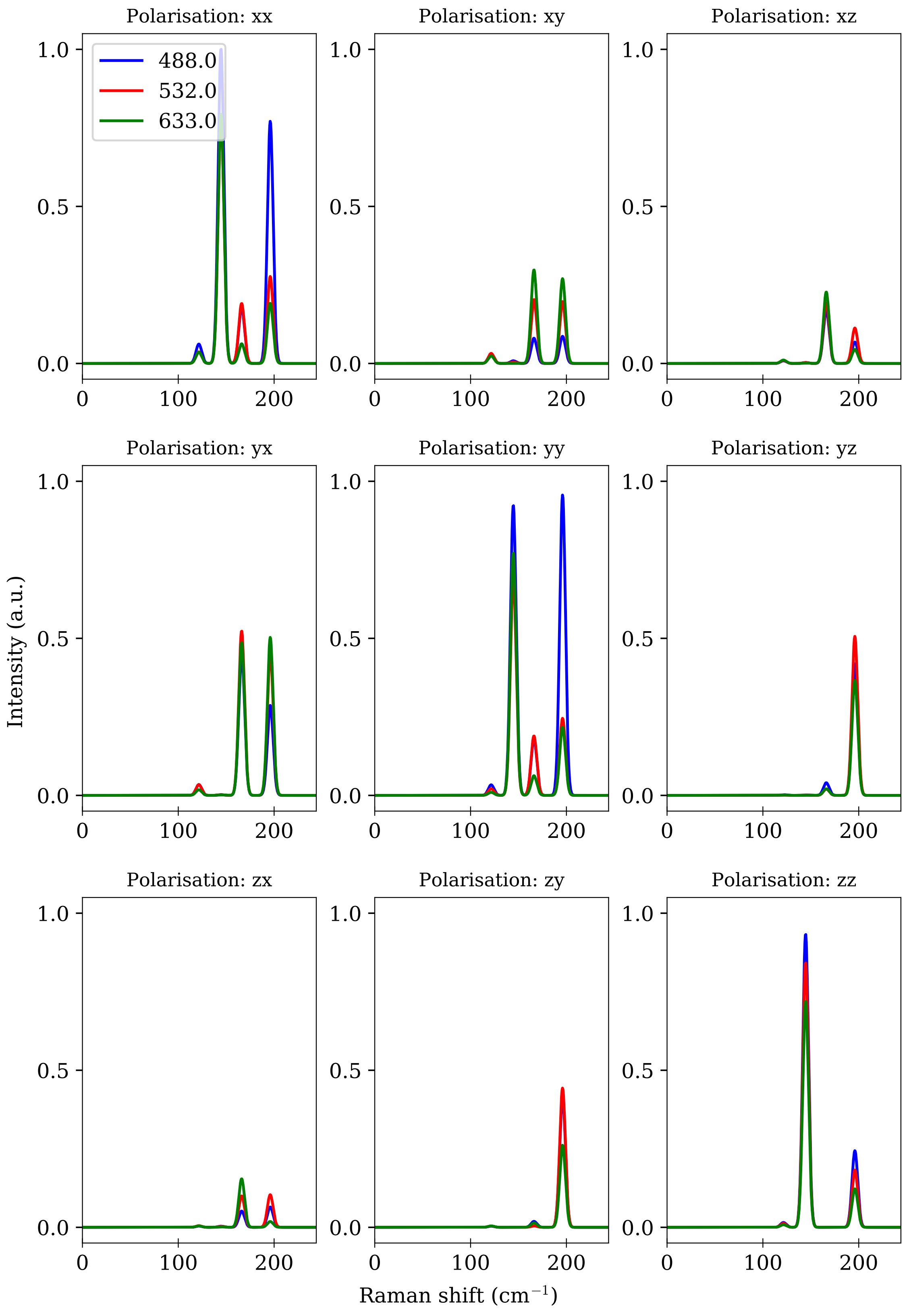

Supplementary Figure 500: Raman spectrum of RuI_2 with space group 164 and Wyckoff positions b-d-0.

RuO_2, 115, d-g-0

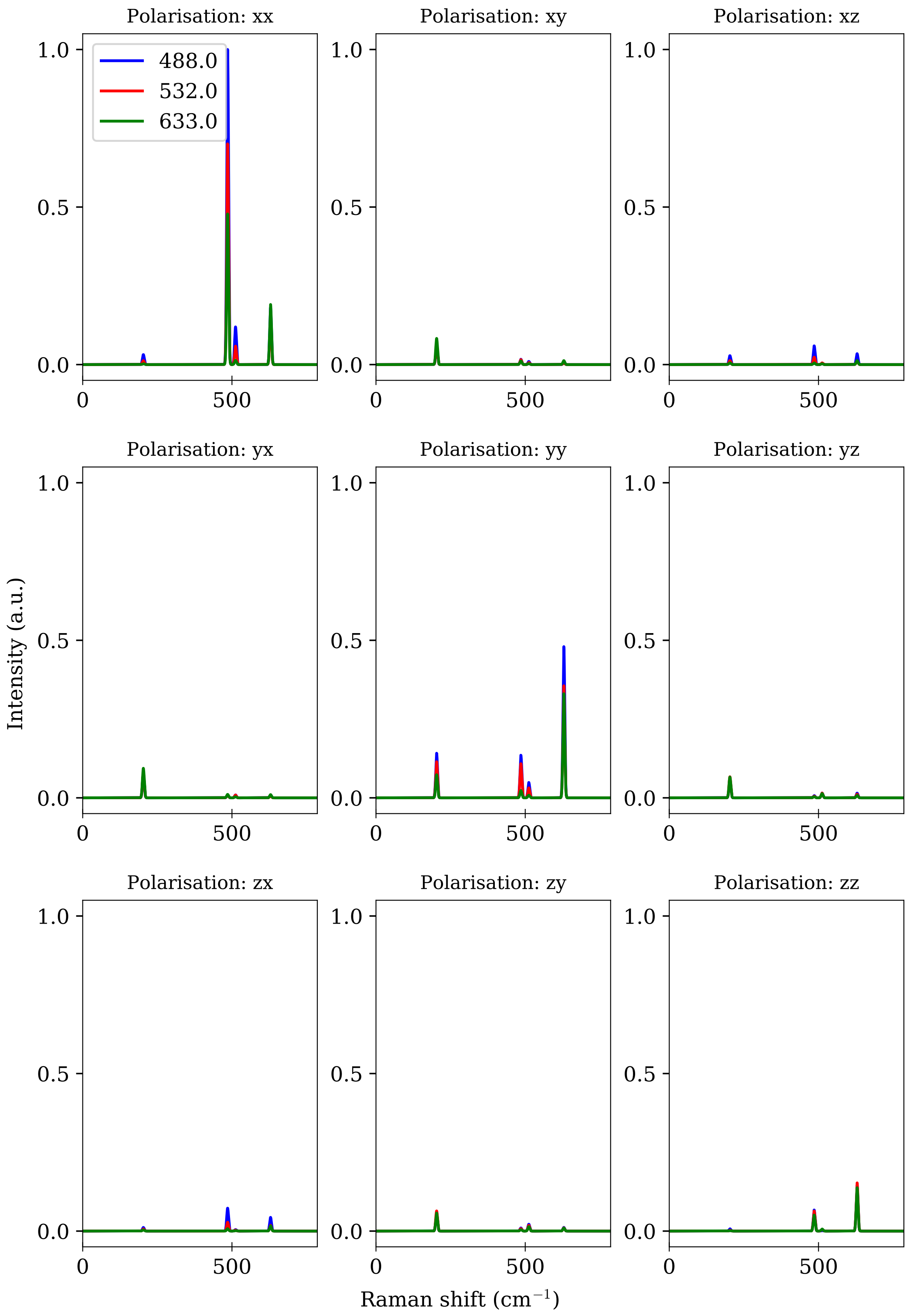

Supplementary Figure 501: Raman spectrum of RuO_2 with space group 115 and Wyckoff positions d-g-0.

S_2Sb, 164, b-d-0

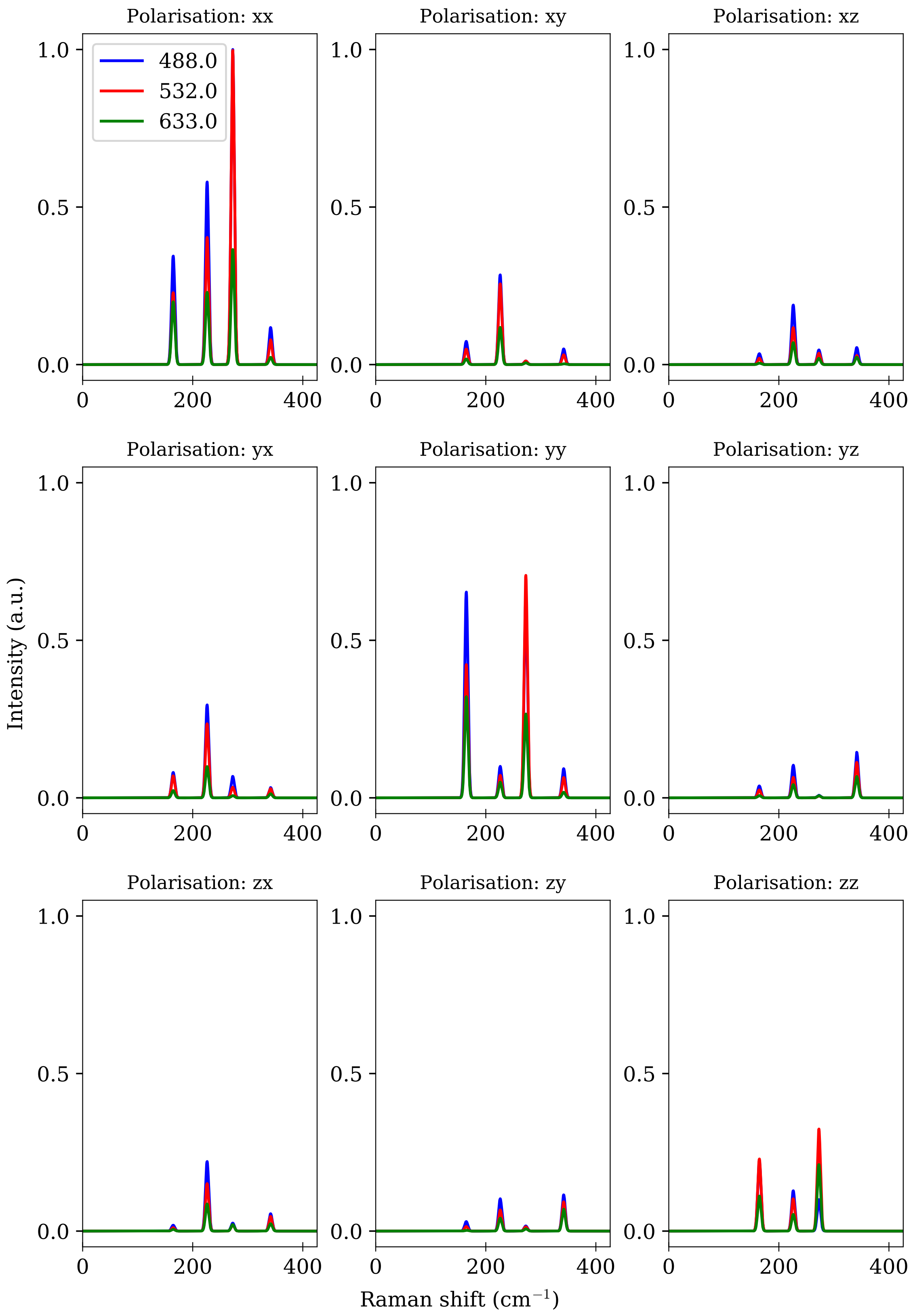

Supplementary Figure 502: Raman spectrum of S_2Sb with space group 164 and Wyckoff positions b-d-0.

$SbSe_2$, 164, b-d-0

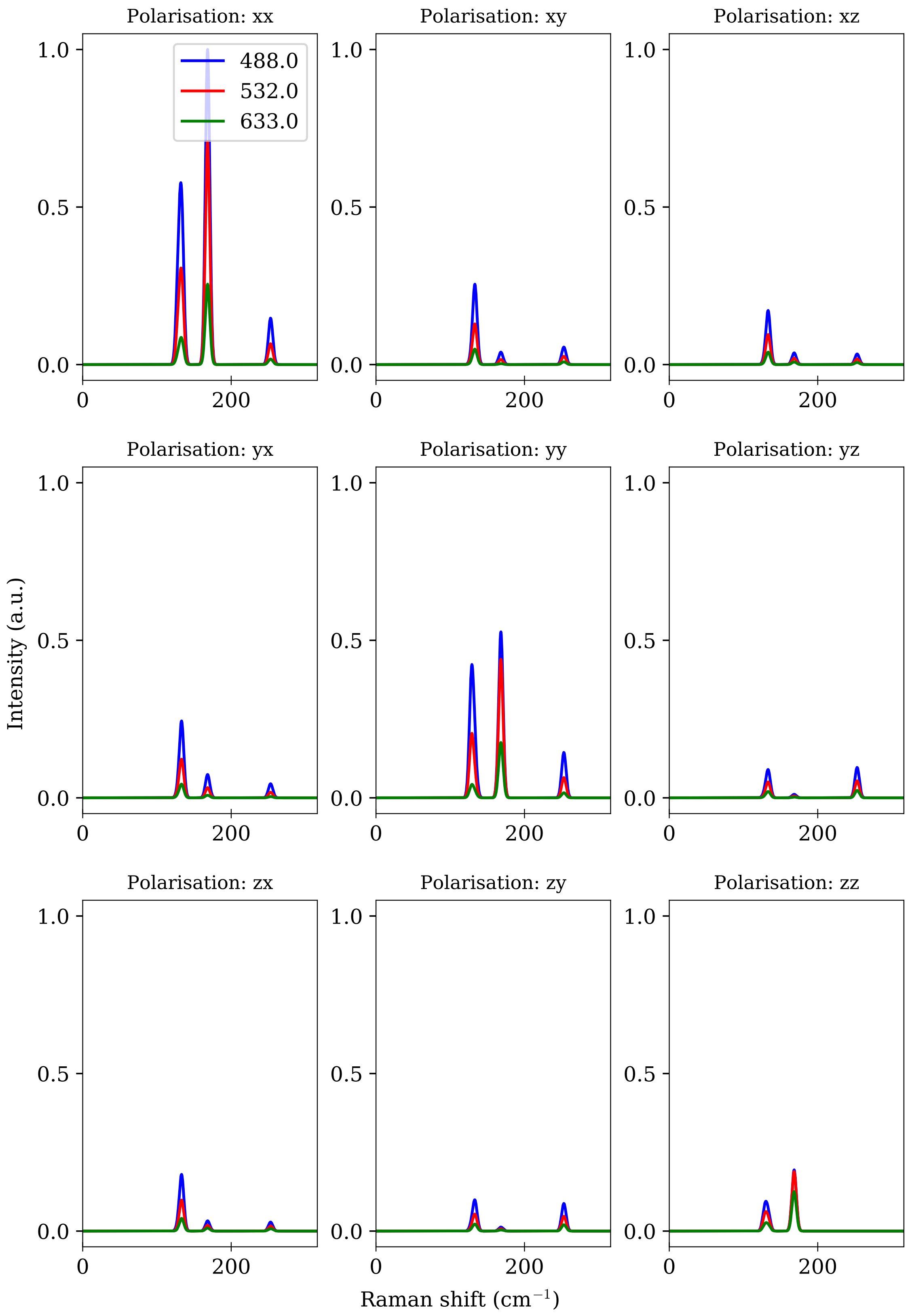

Supplementary Figure 503: Raman spectrum of $SbSe_2$ with space group 164 and Wyckoff positions b-d-0.

$SbTe_2$, 164, b-d-0

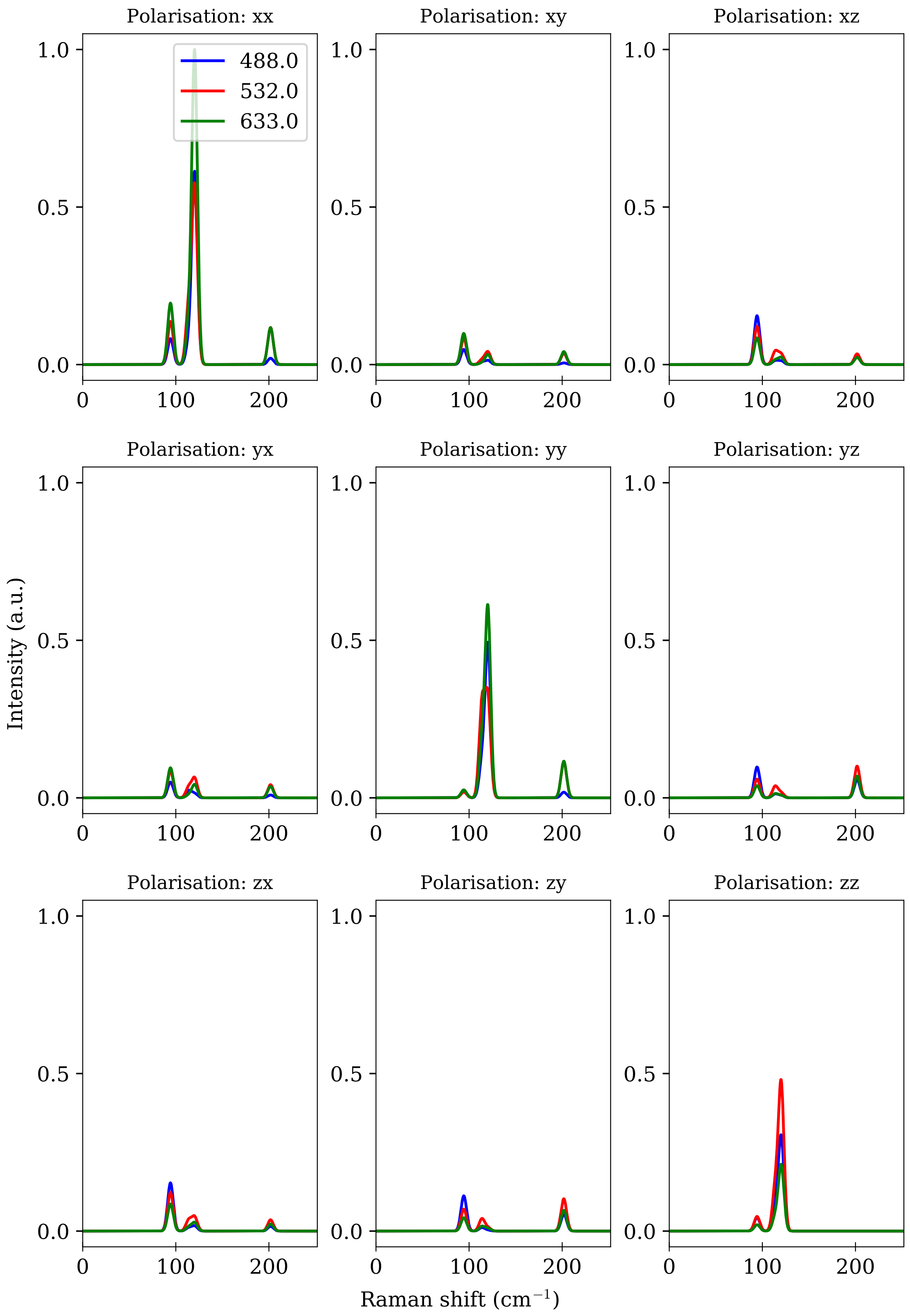

Supplementary Figure 504: Raman spectrum of $SbTe_2$ with space group 164 and Wyckoff positions b-d-0.

$Sc_2Br_2S_2$, 59, a-b-0

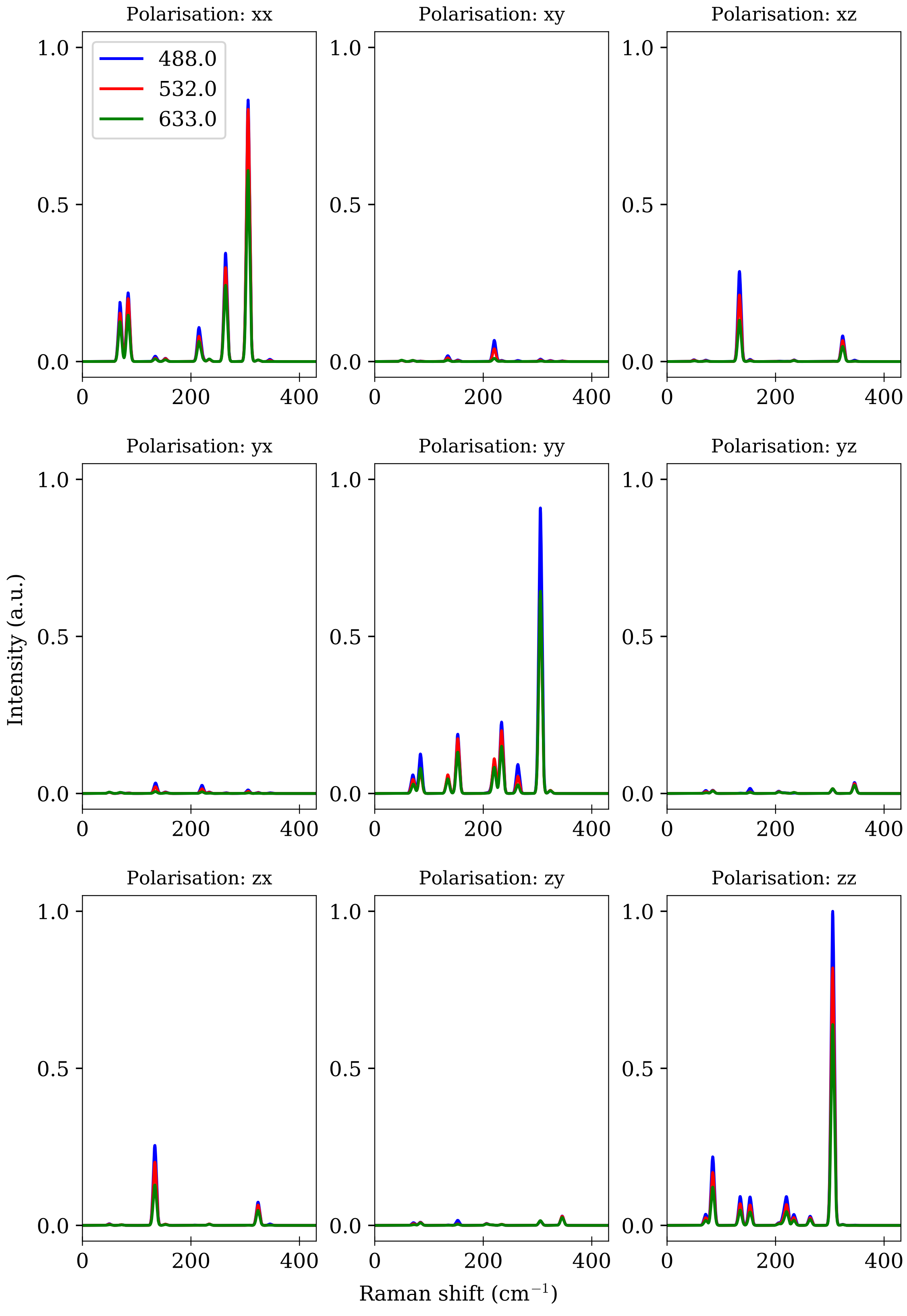

Supplementary Figure 505: Raman spectrum of $Sc_2Br_2S_2$ with space group 59 and Wyckoff positions a-b-0.

$Sc_2Br_2Se_2$, 59, a-b-0

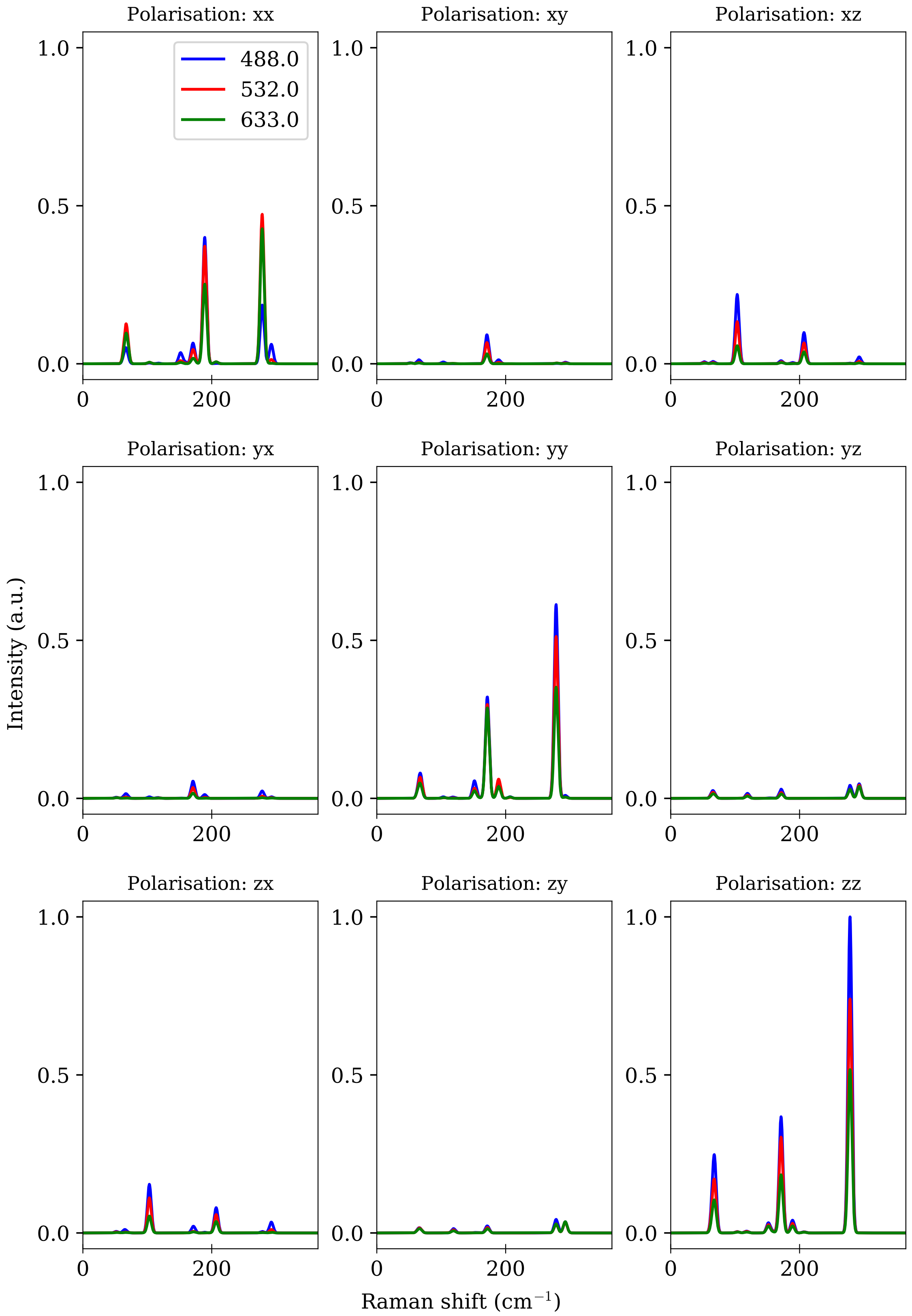

Supplementary Figure 506: Raman spectrum of $Sc_2Br_2Se_2$ with space group 59 and Wyckoff positions a-b-0.

Sc_2CF_2, 164, b-d-0

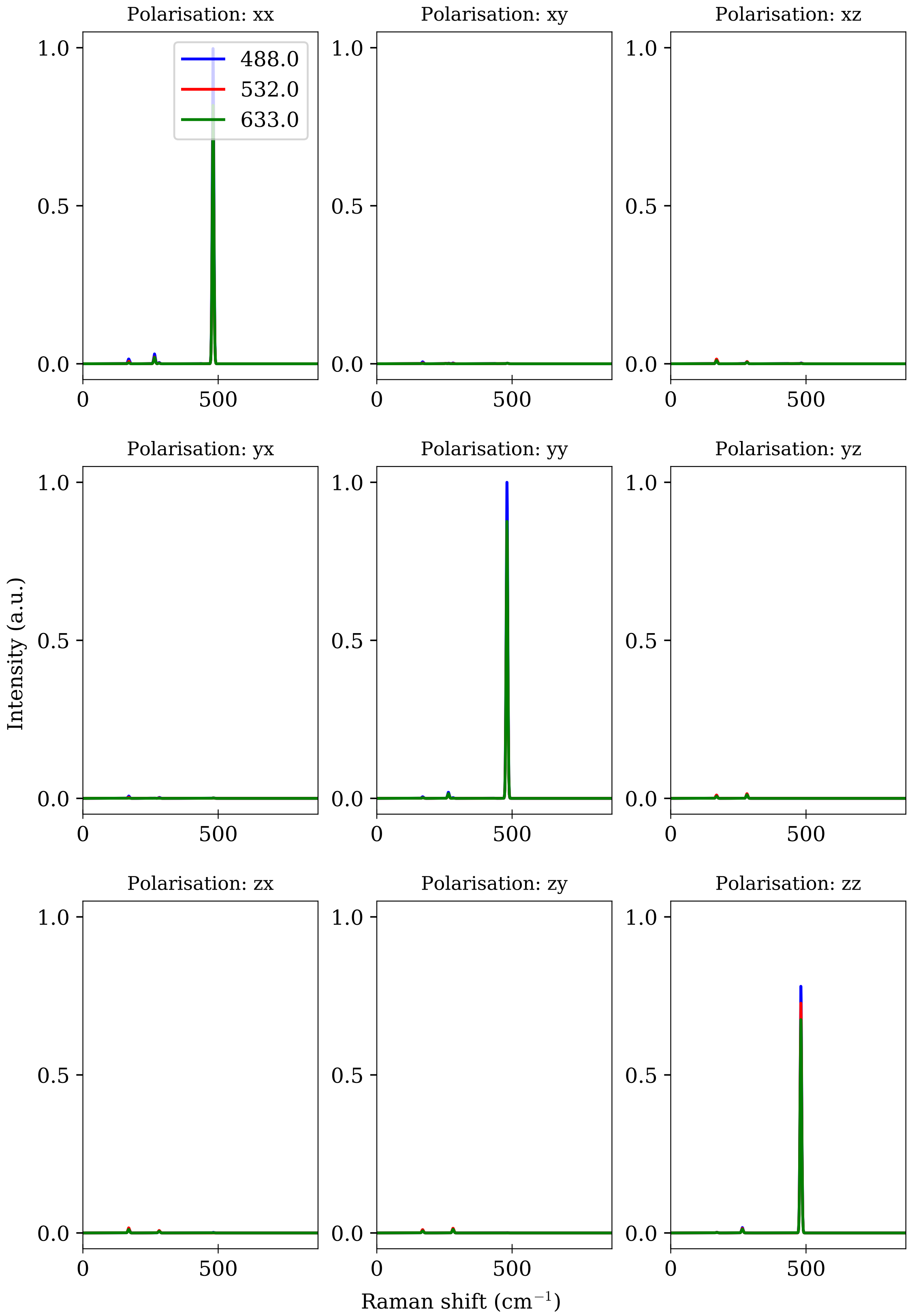

Supplementary Figure 507: Raman spectrum of Sc_2CF_2 with space group 164 and Wyckoff positions b-d-0.

$Sc_2CH_2O_2$, 164, b-d-0

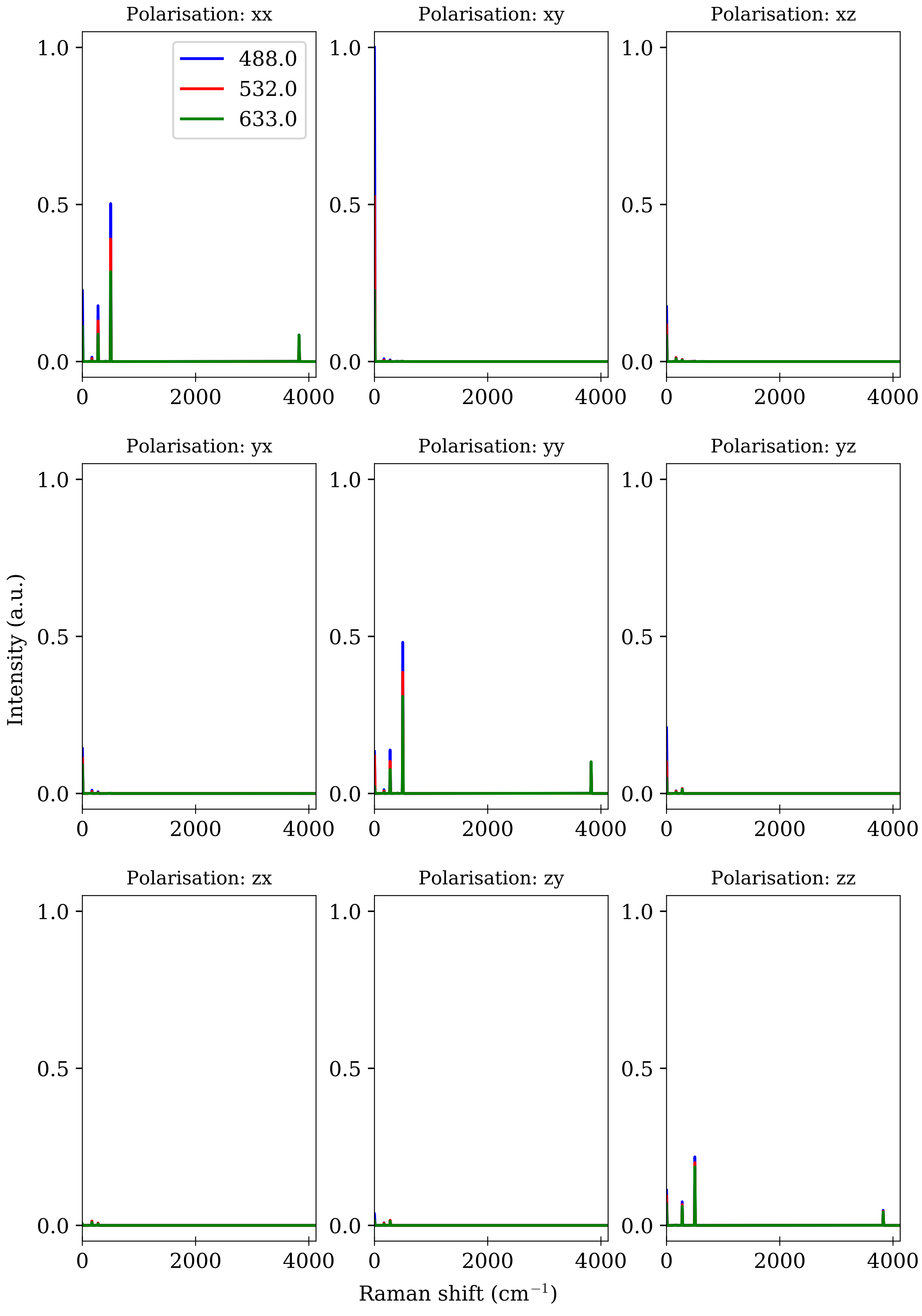

Supplementary Figure 508: Raman spectrum of $Sc_2CH_2O_2$ with space group 164 and Wyckoff positions b-d-0.

$Sc_2Cl_2Se_2$, 59, a-b-0

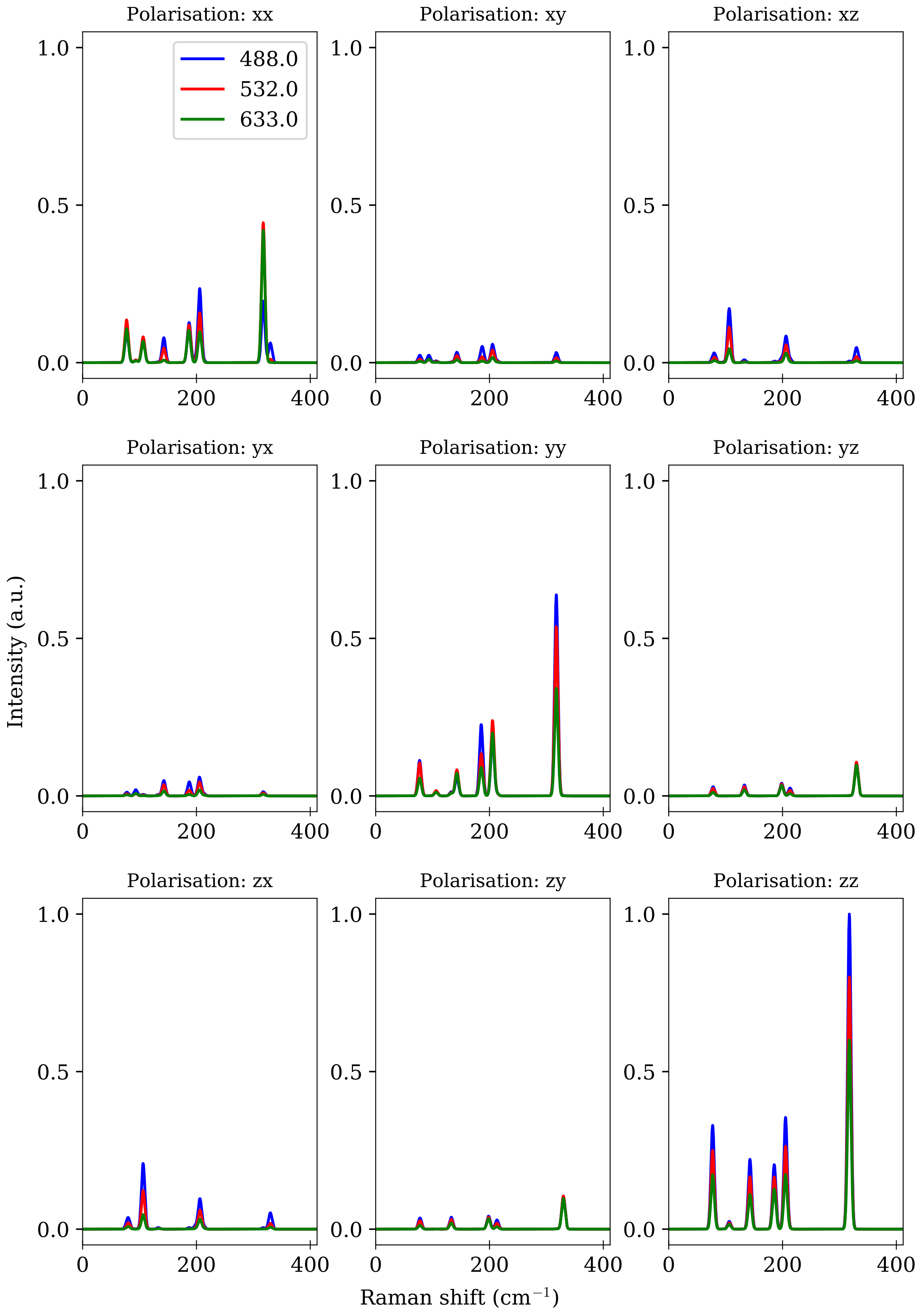

Supplementary Figure 509: Raman spectrum of $Sc_2Cl_2Se_2$ with space group 59 and Wyckoff positions a-b-0.

Sc_2F_2N, 164, b-d-0

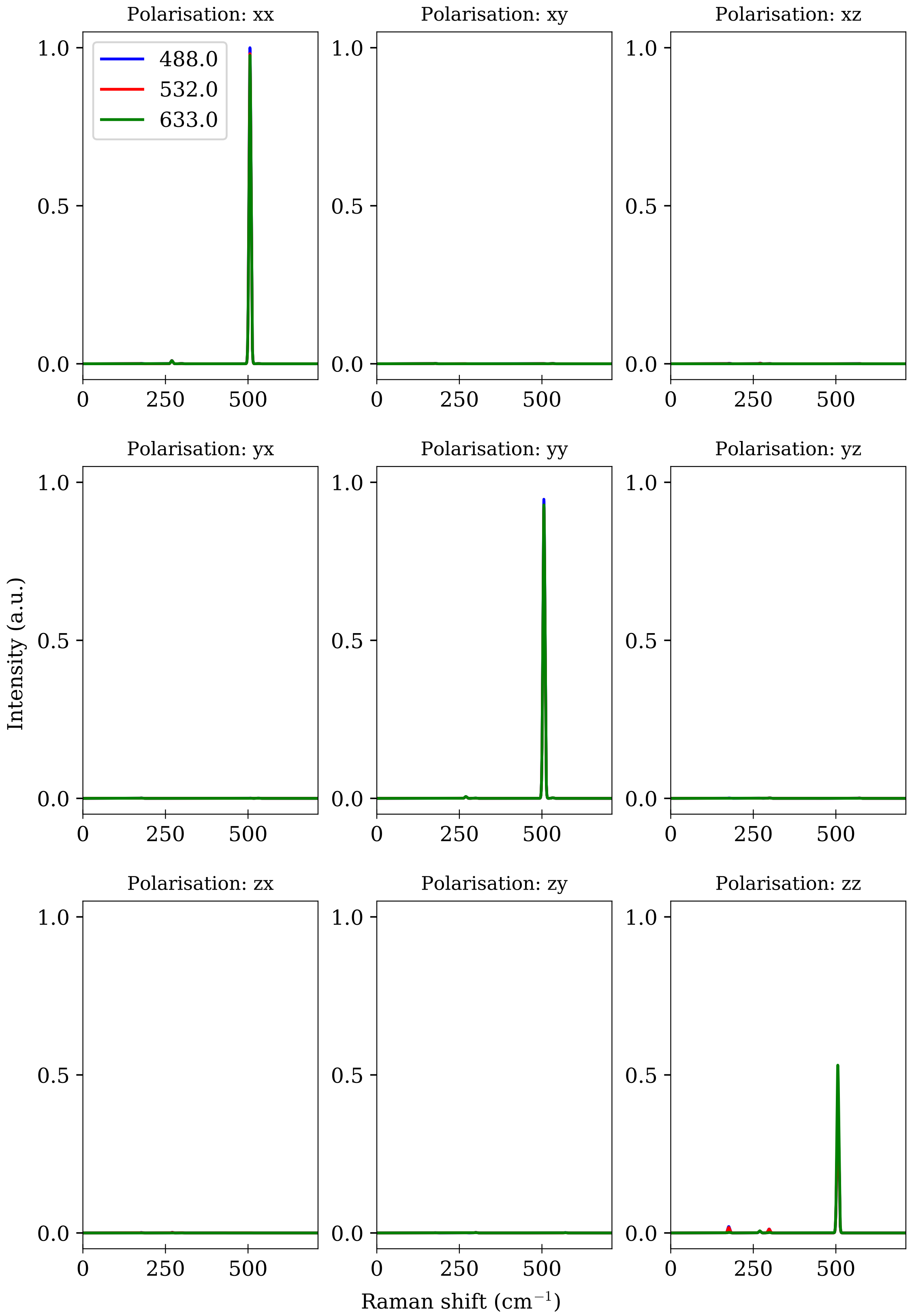

Supplementary Figure 510: Raman spectrum of Sc_2F_2N with space group 164 and Wyckoff positions b-d-0.

$Sc_2H_2NO_2$, 164, b-d-0

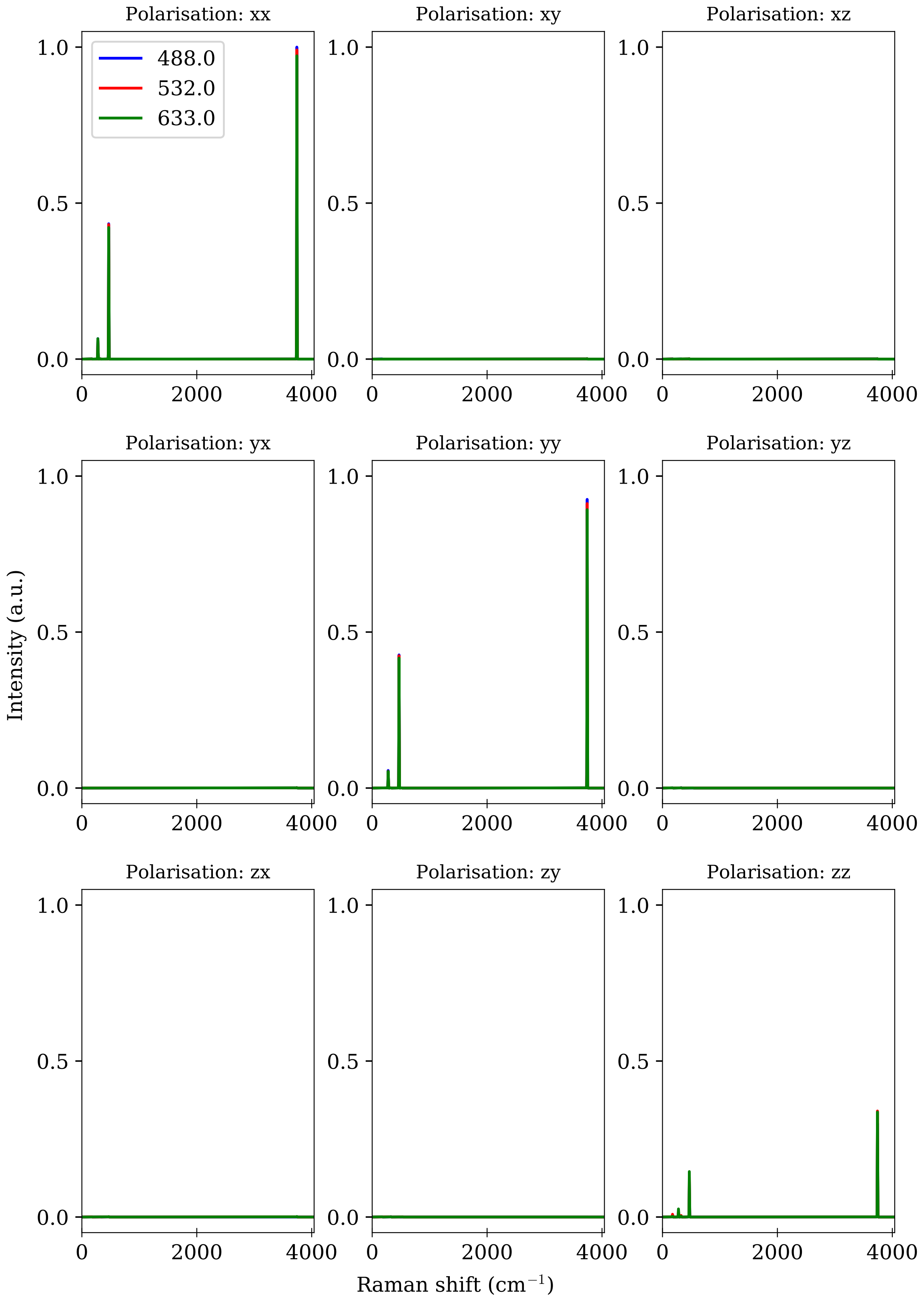

Supplementary Figure 511: Raman spectrum of $Sc_2H_2NO_2$ with space group 164 and Wyckoff positions b-d-0.

$Sc_2I_2N_2$, 59, a-b-0

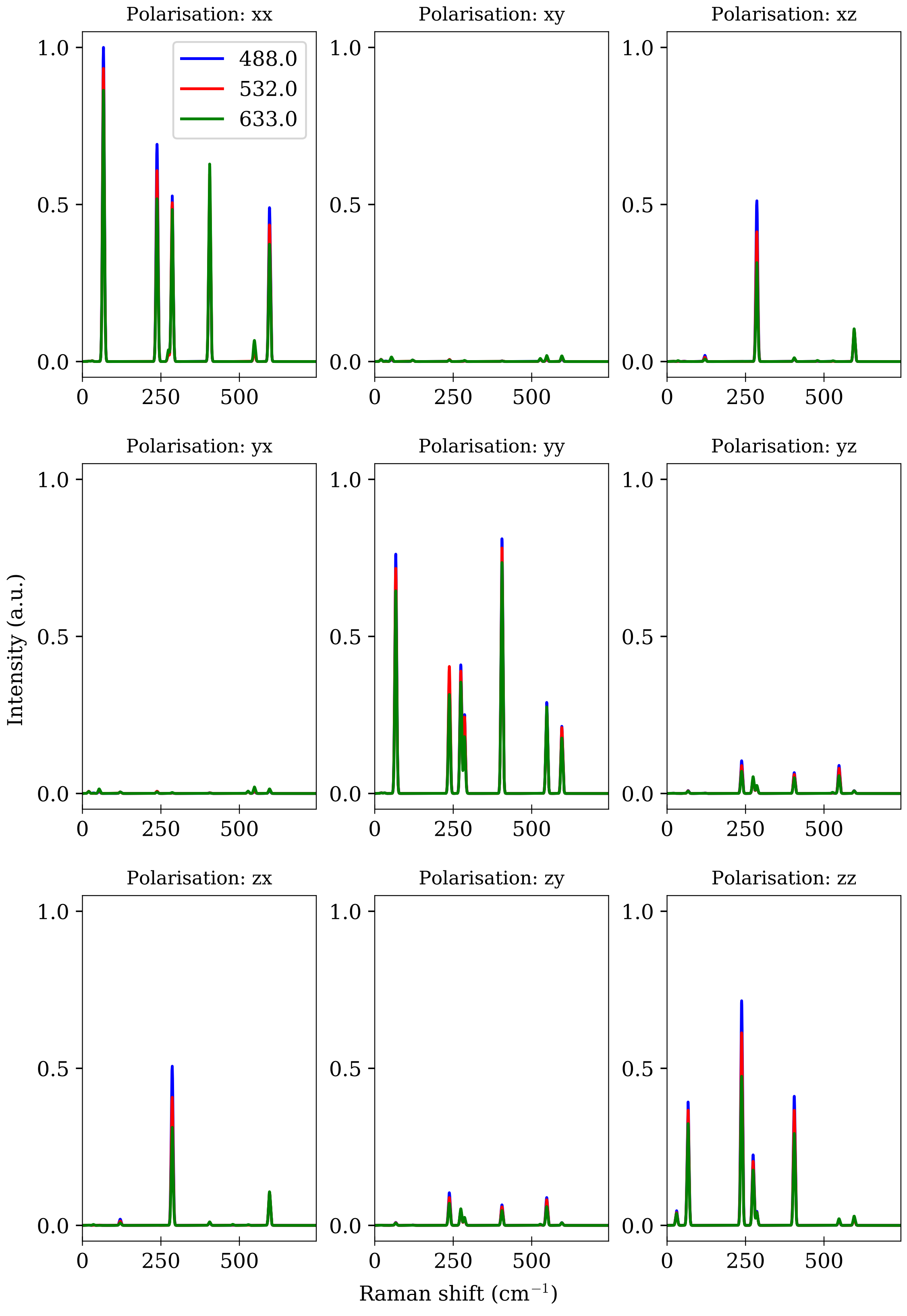

Supplementary Figure 512: Raman spectrum of $Sc_2I_2N_2$ with space group 59 and Wyckoff positions a-b-0.

$Sc_2I_2S_2$, 59, a-b-0

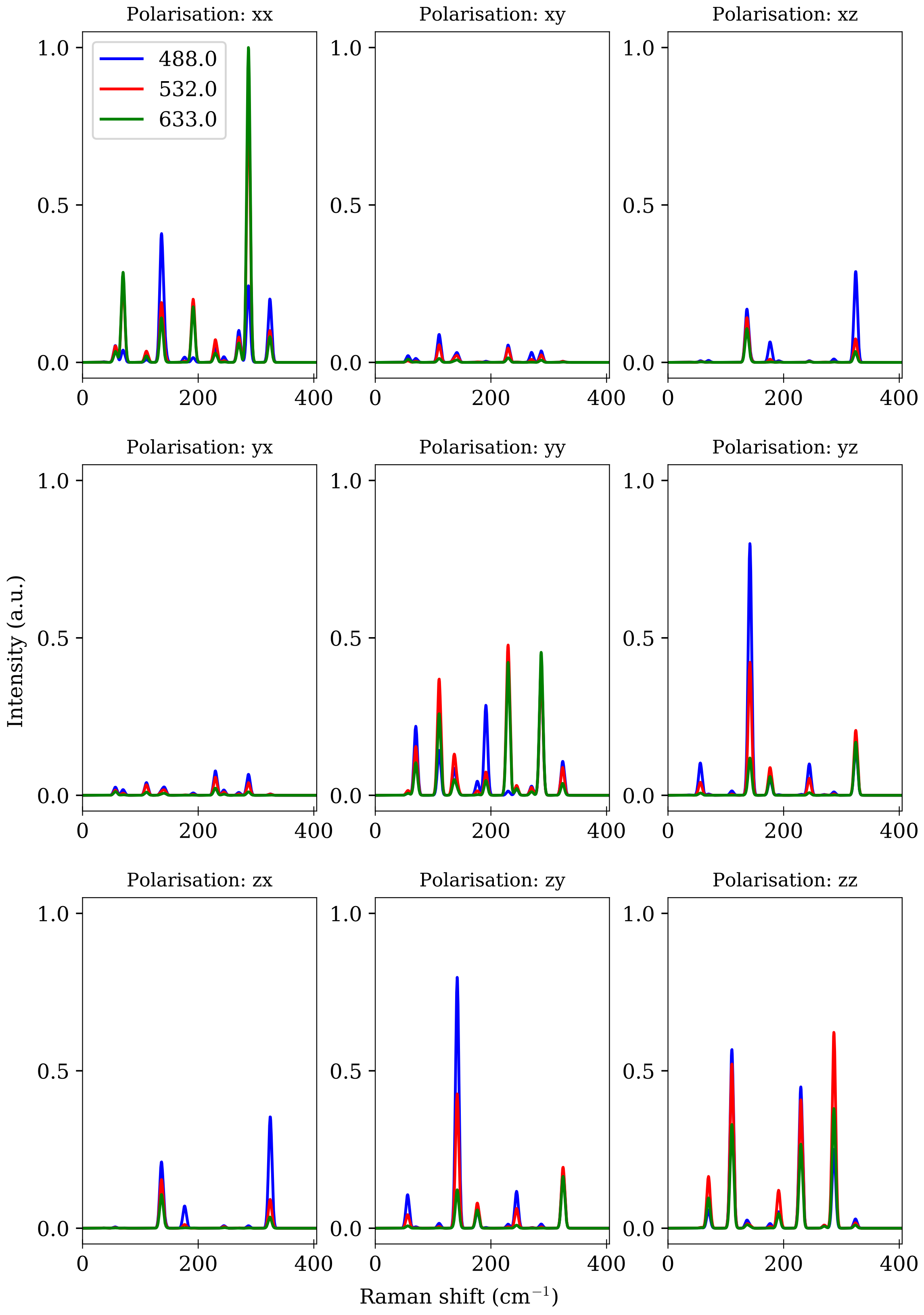

Supplementary Figure 513: Raman spectrum of $Sc_2I_2S_2$ with space group 59 and Wyckoff positions a-b-0.

$Sc_2I_2Se_2$, 59, a-b-0

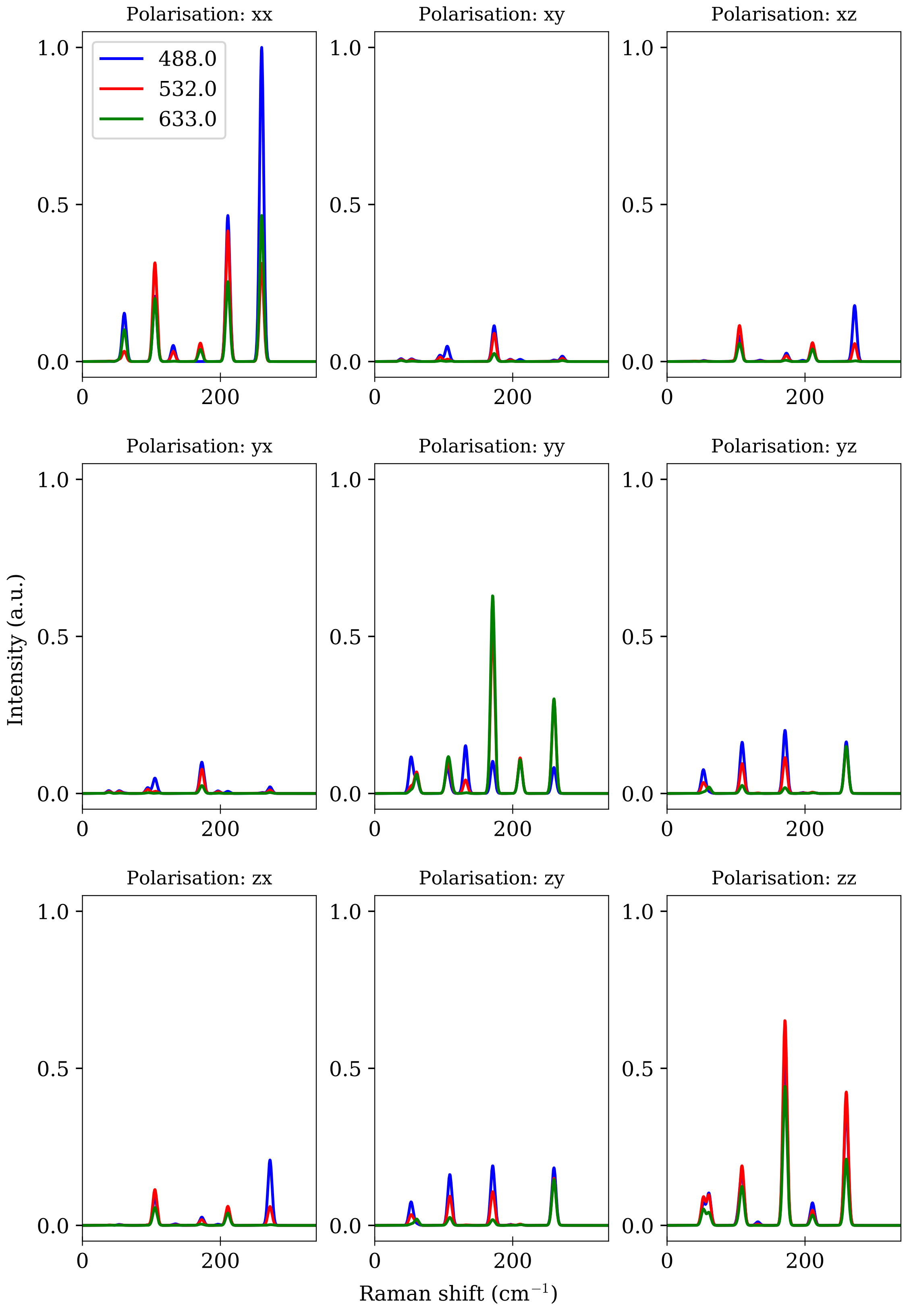

Supplementary Figure 514: Raman spectrum of $Sc_2I_2Se_2$ with space group 59 and Wyckoff positions a-b-0.

Sc_2NO_2, 164, b-d-0

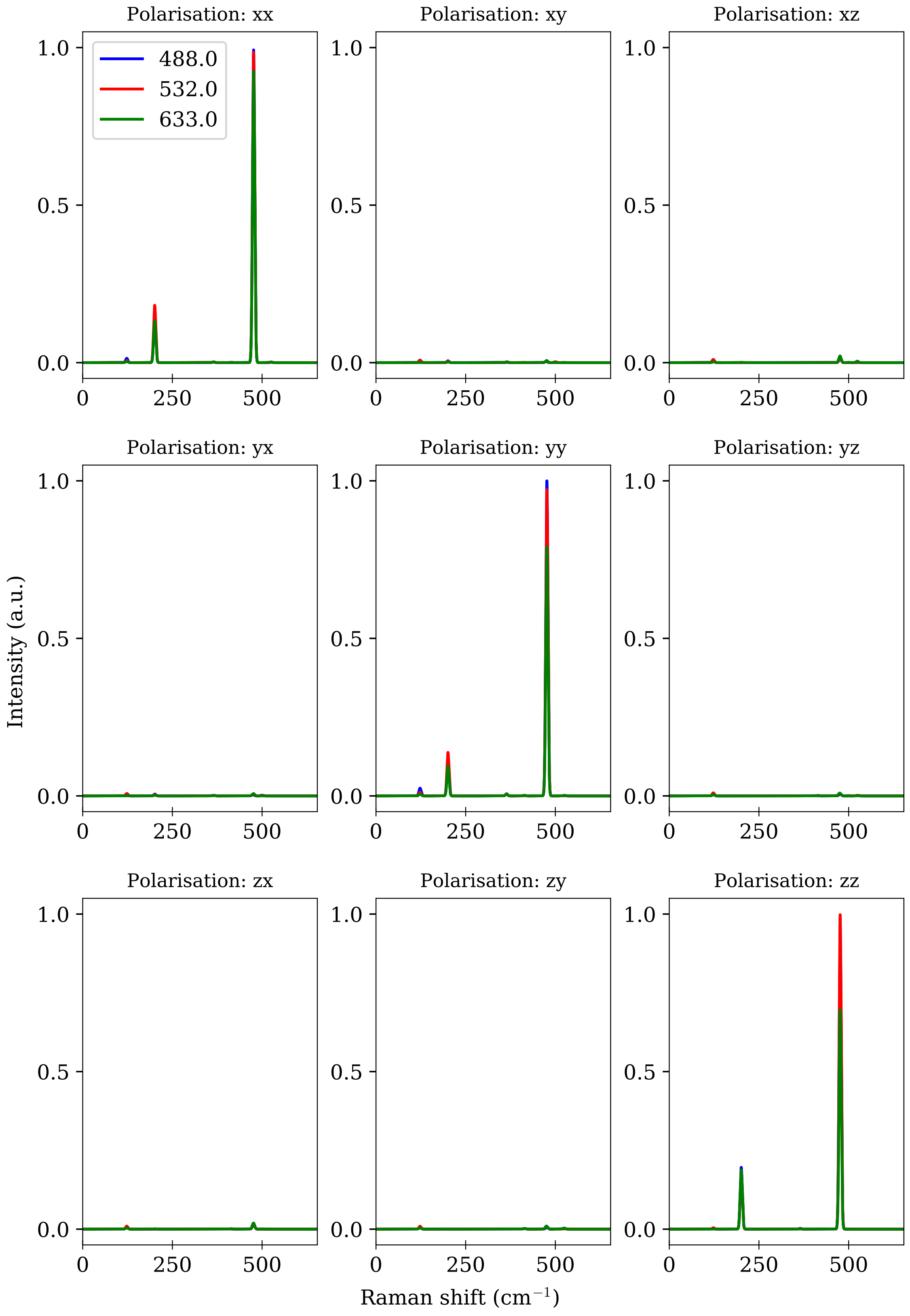

Supplementary Figure 515: Raman spectrum of Sc_2NO_2 with space group 164 and Wyckoff positions b-d-0.

Sc_2N, 164, b-d-0

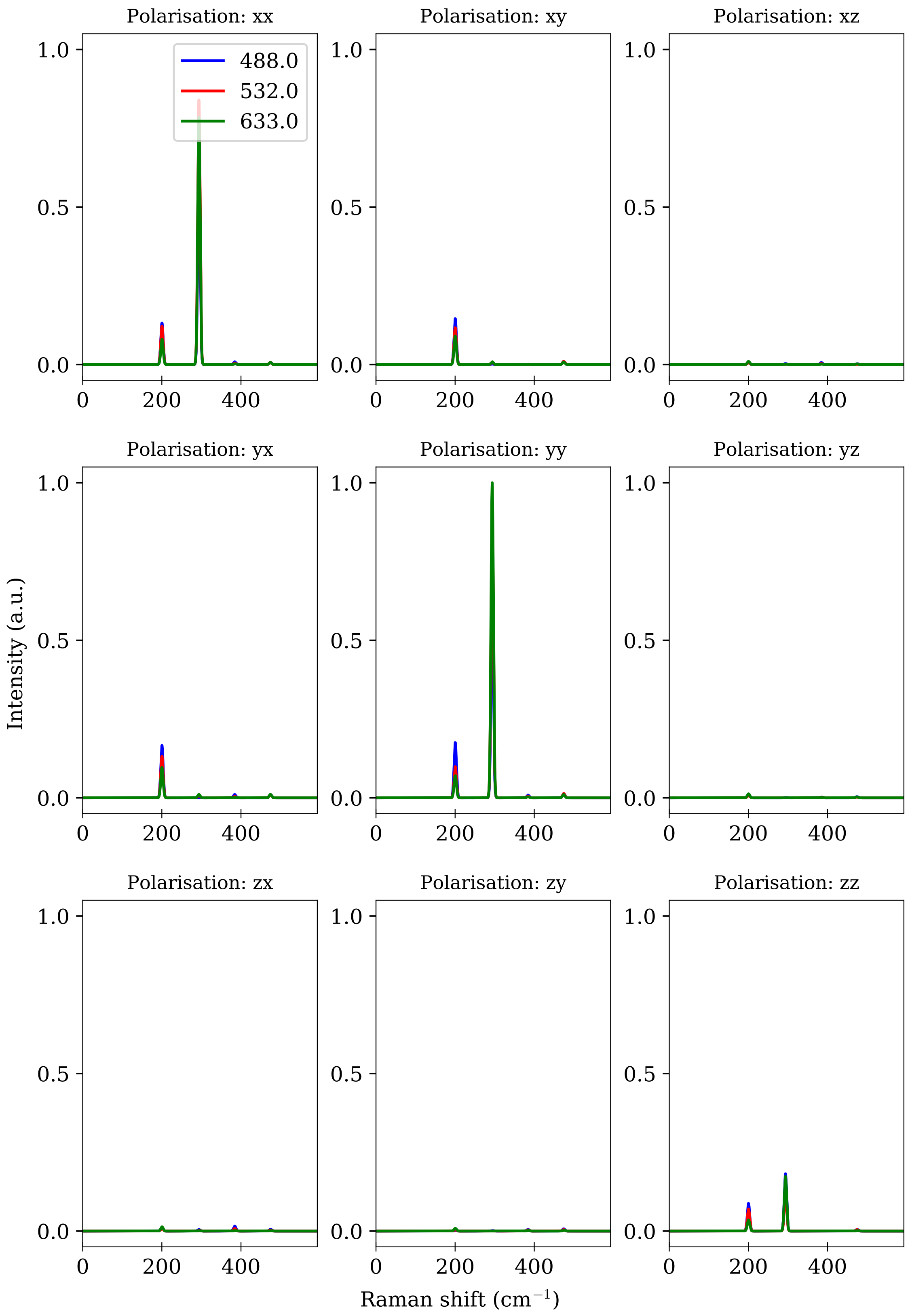

Supplementary Figure 516: Raman spectrum of Sc_2N with space group 164 and Wyckoff positions b-d-0.

$Sc_2P_2S_6$, 143, a-b-c-d-0

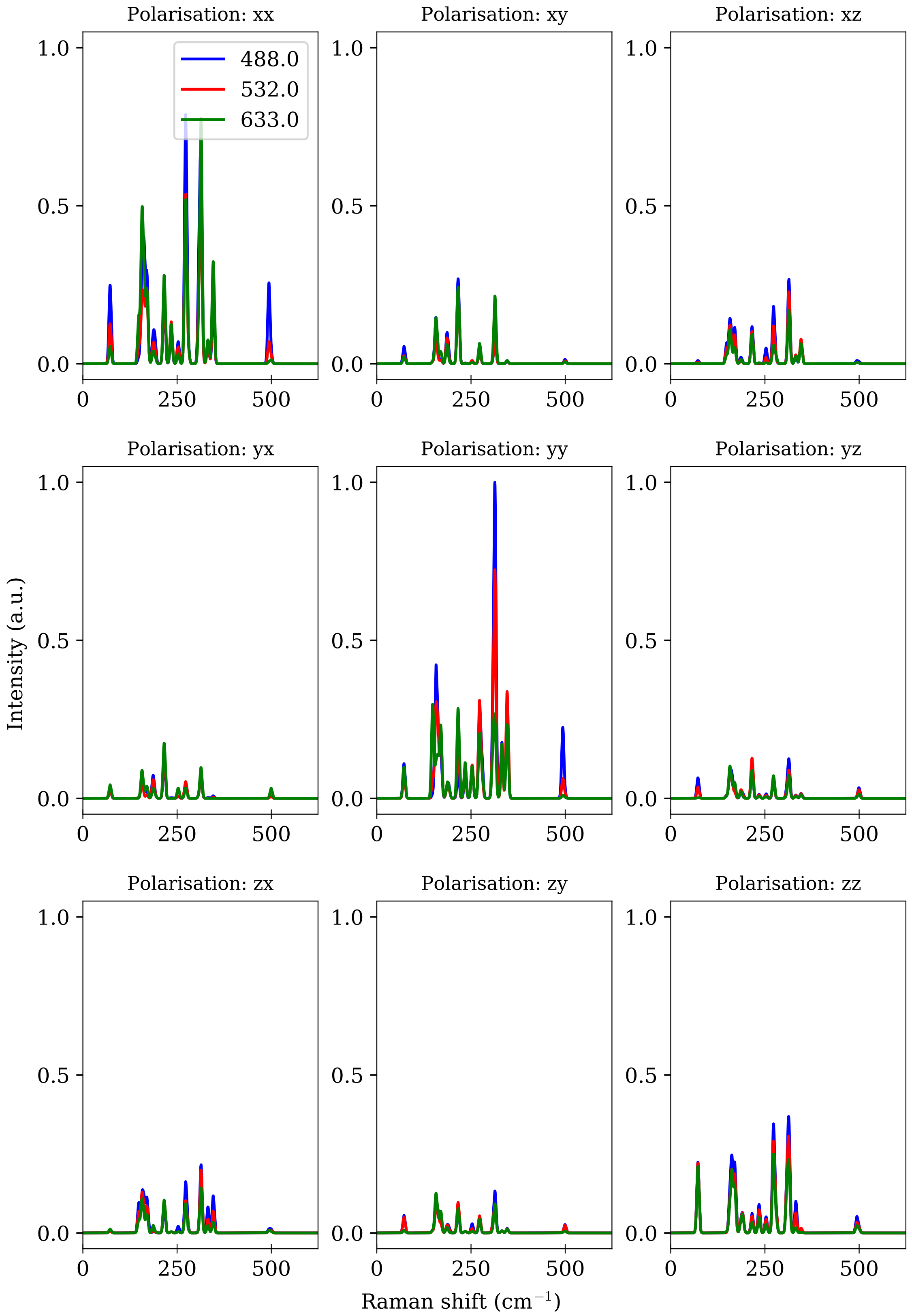

Supplementary Figure 517: Raman spectrum of $Sc_2P_2S_6$ with space group 143 and Wyckoff positions a-b-c-d-0.

$Sc_2P_2Se_6$, 143, a-b-c-d-0

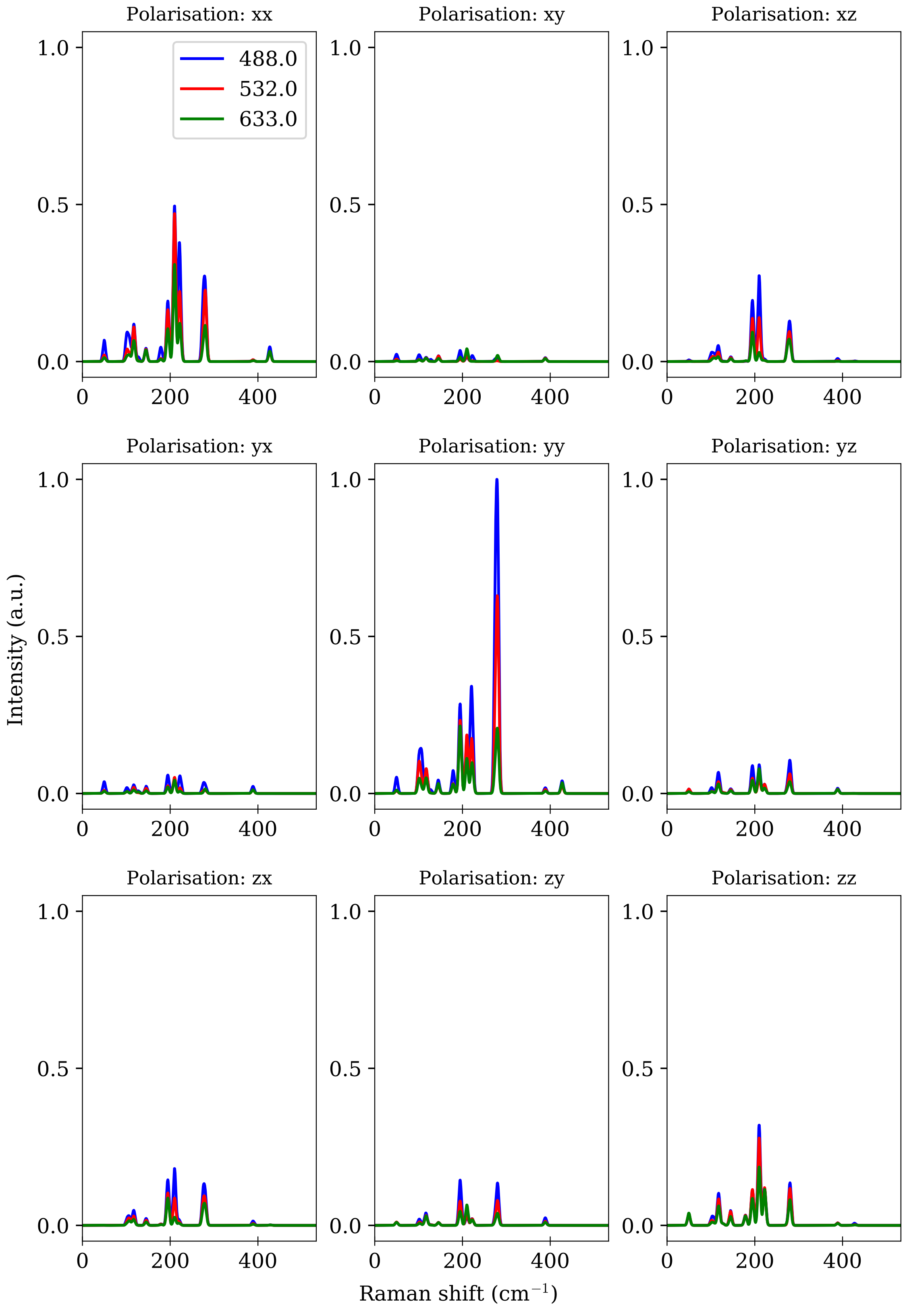

Supplementary Figure 518: Raman spectrum of $Sc_2P_2Se_6$ with space group 143 and Wyckoff positions a-b-c-d-0.

Sc_2S_2, 129, b-c-0

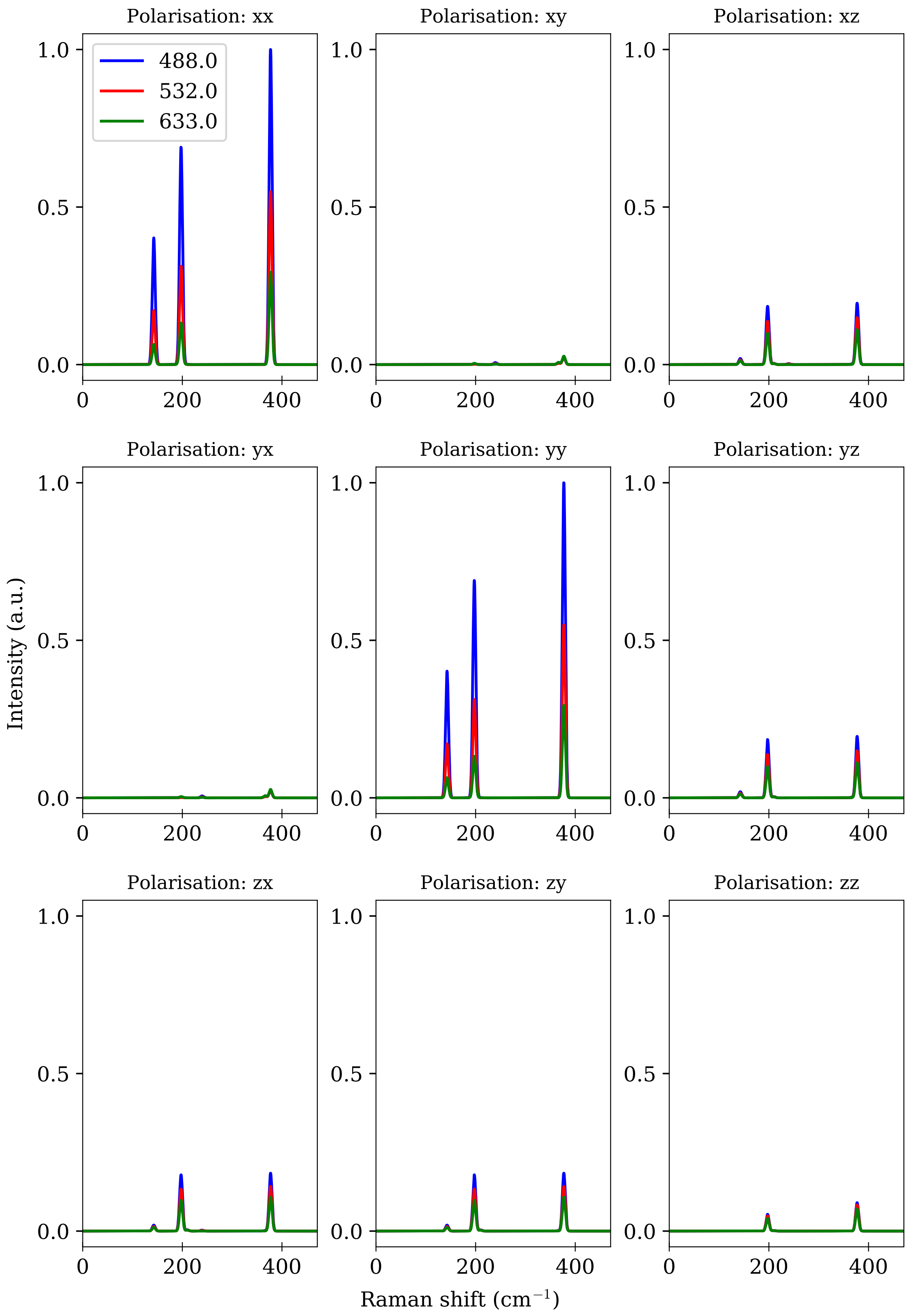

Supplementary Figure 519: Raman spectrum of Sc_2S_2 with space group 129 and Wyckoff positions b-c-0.

Sc_2S_2, 12, i-0

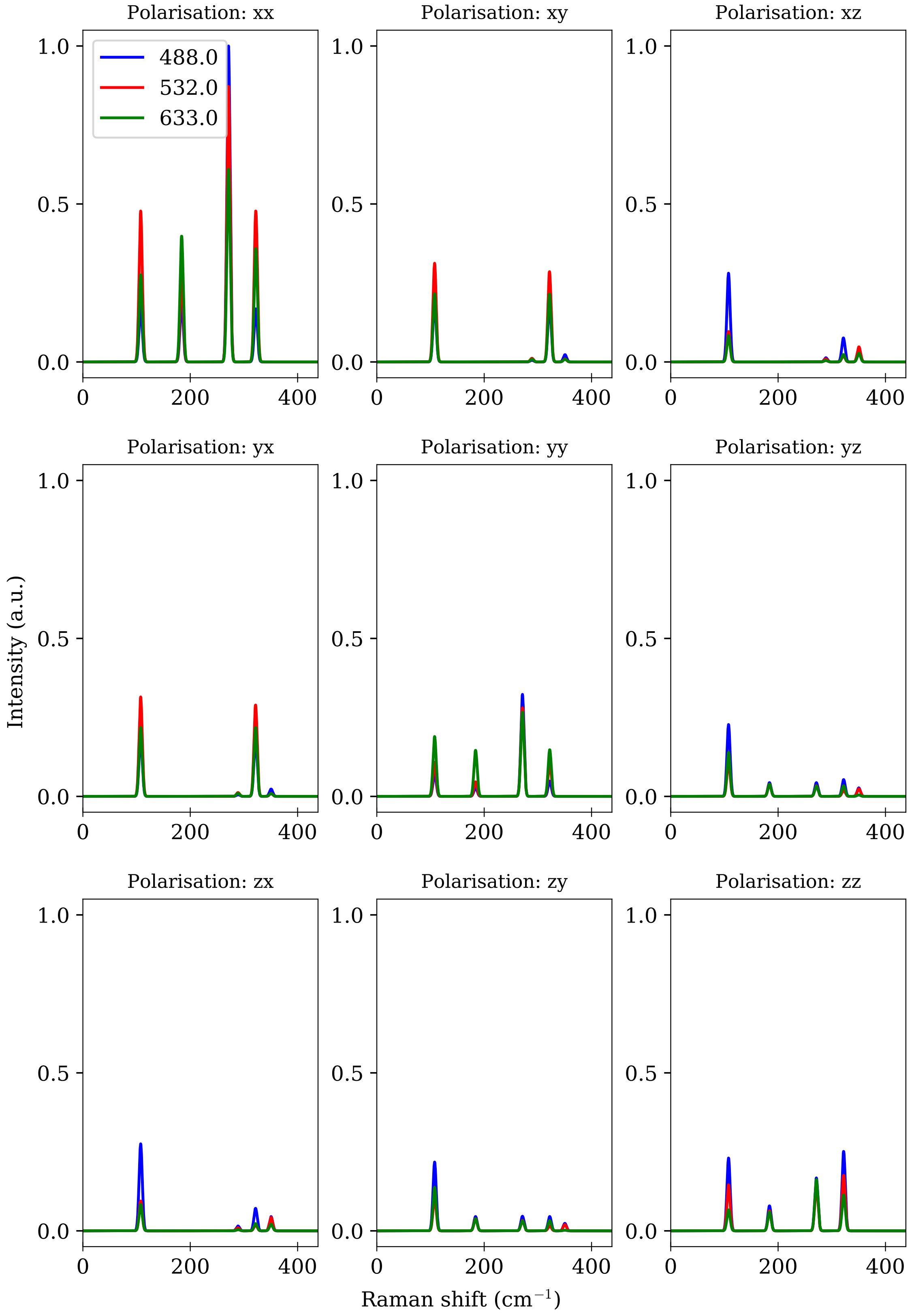

Supplementary Figure 520: Raman spectrum of Sc_2S_2 with space group 12 and Wyckoff positions i-0.

Sc_2Se_2, 129, b-c-0

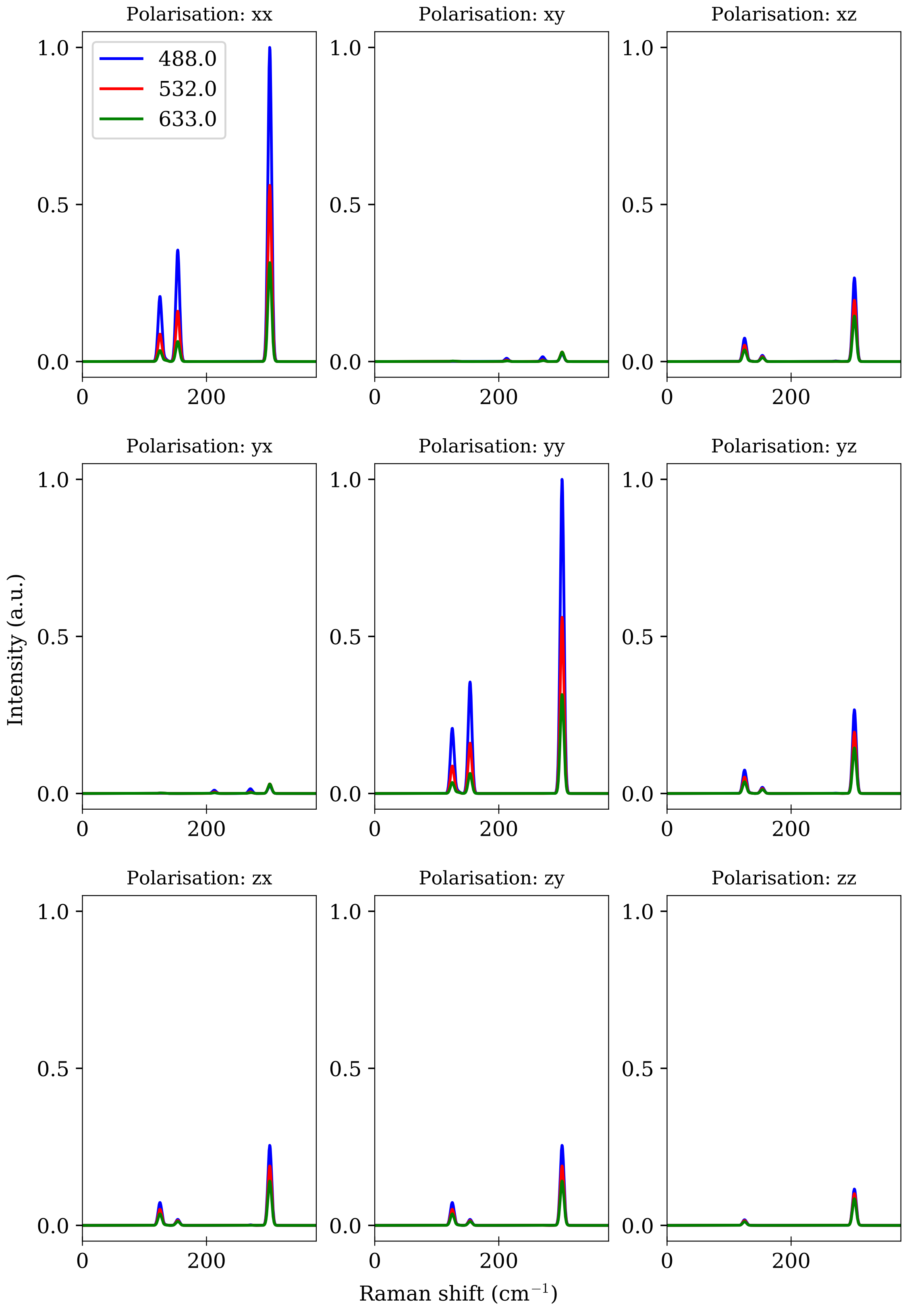

Supplementary Figure 521: Raman spectrum of Sc_2Se_2 with space group 129 and Wyckoff positions b-c-0.

Sc_2Te_2, 129, b-c-0

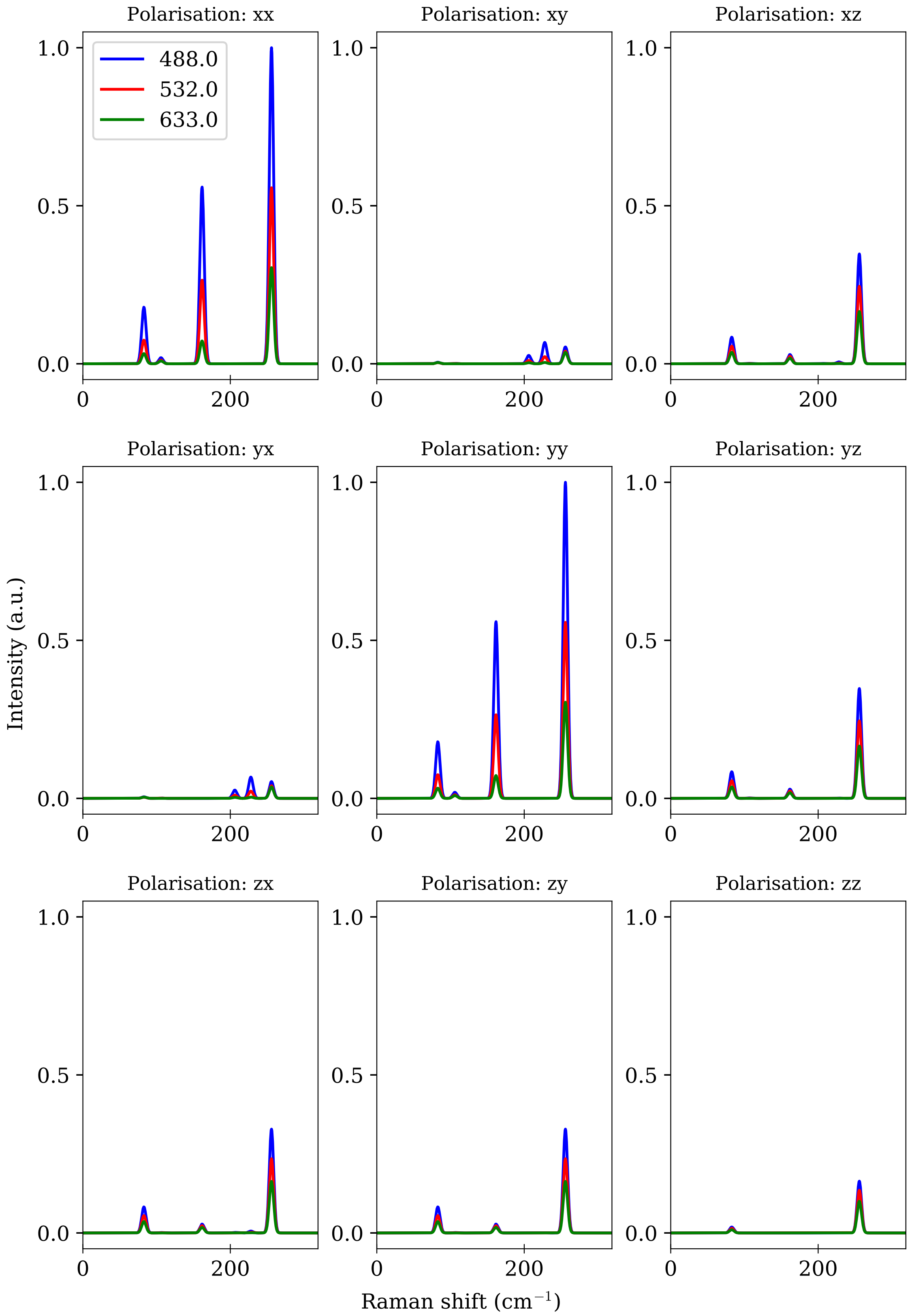

Supplementary Figure 522: Raman spectrum of Sc_2Te_2 with space group 129 and Wyckoff positions b-c-0.

$Sc_3F_2N_2$, 187, b-g-h-i-0

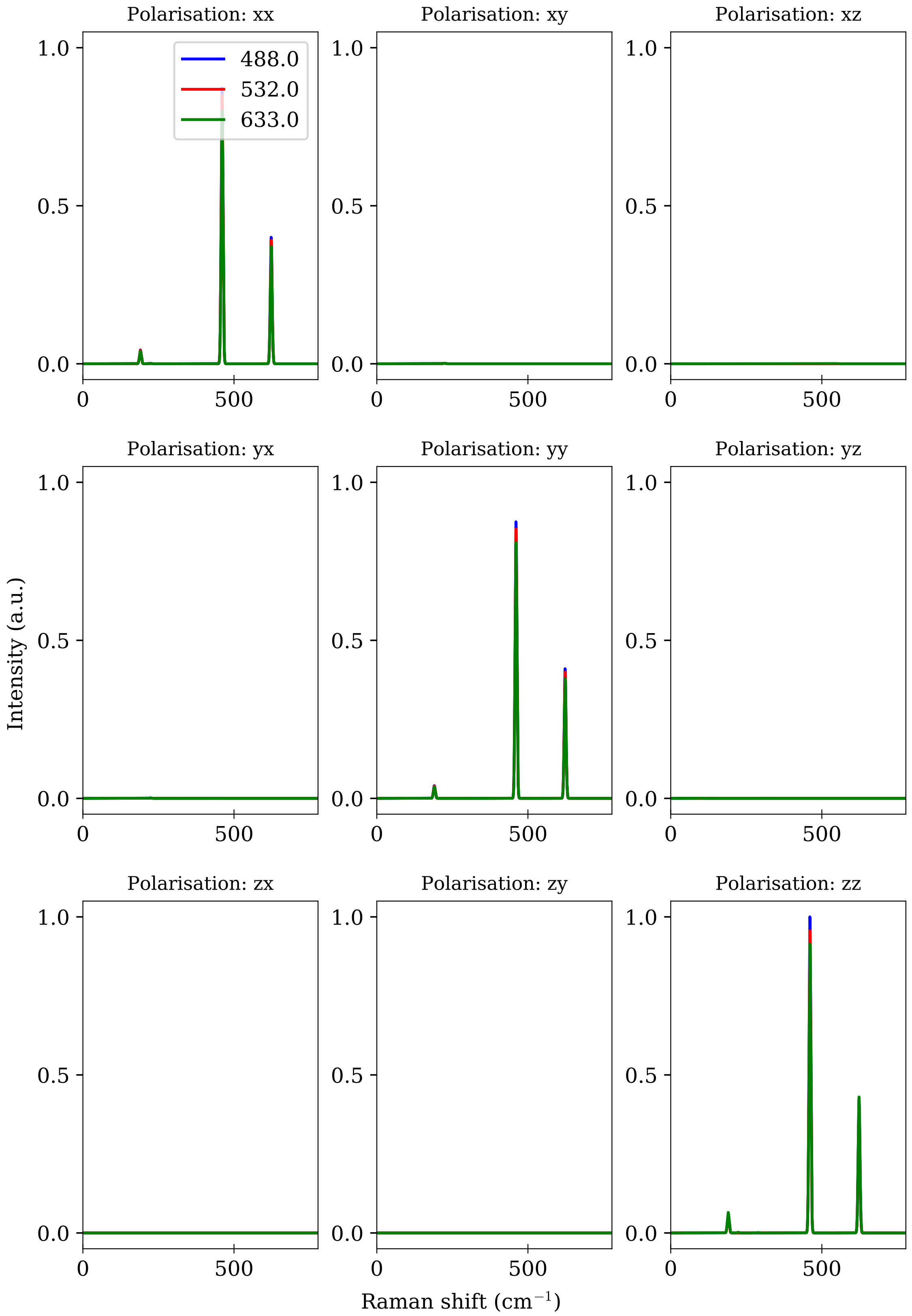

Supplementary Figure 523: Raman spectrum of $Sc_3F_2N_2$ with space group 187 and Wyckoff positions b-g-h-i-0.

Sc_3N_2, 187, b-h-i-0

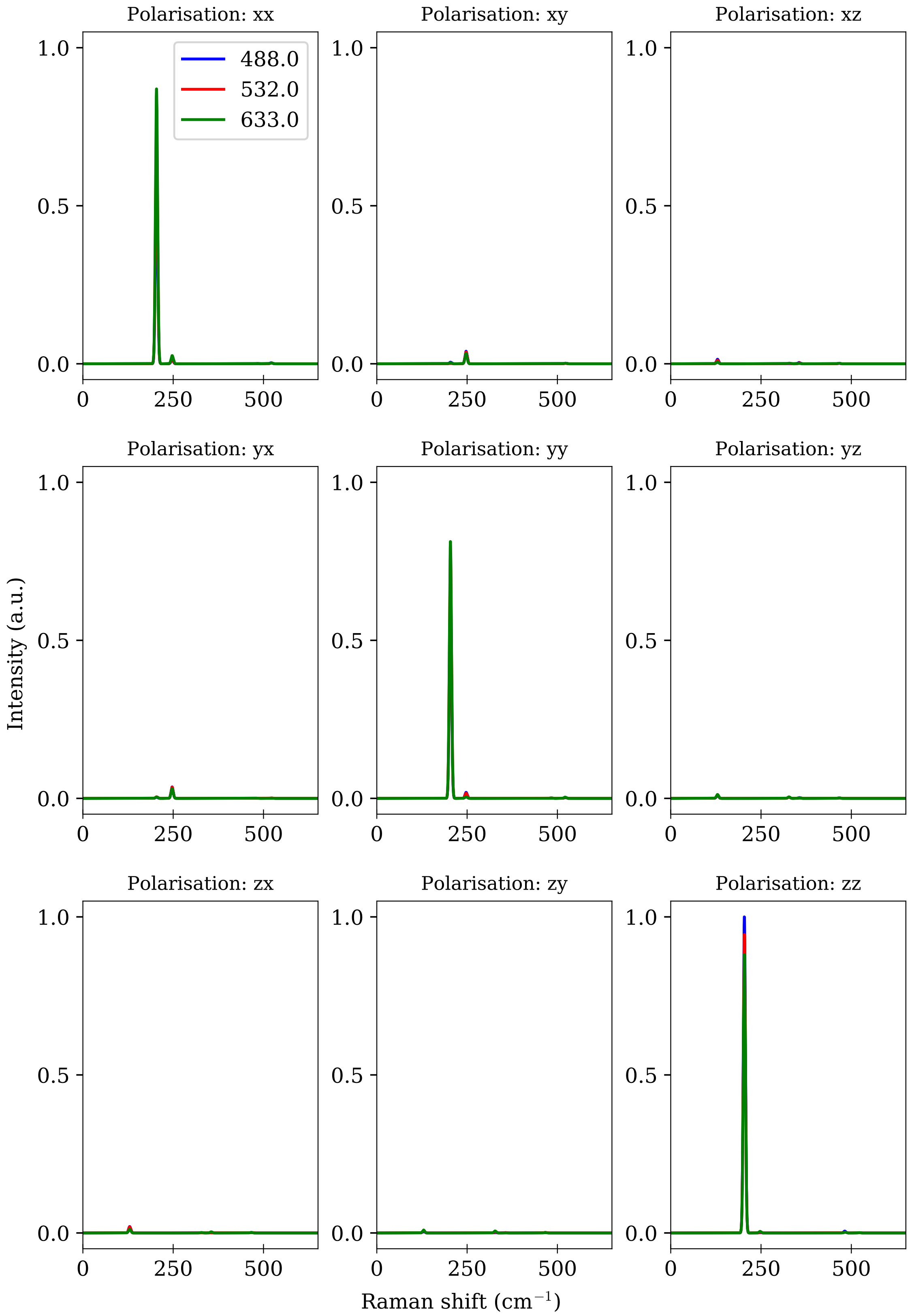

Supplementary Figure 524: Raman spectrum of Sc_3N_2 with space group 187 and Wyckoff positions b-h-i-0.

Sc_4C_3, 164, b-c-d-0

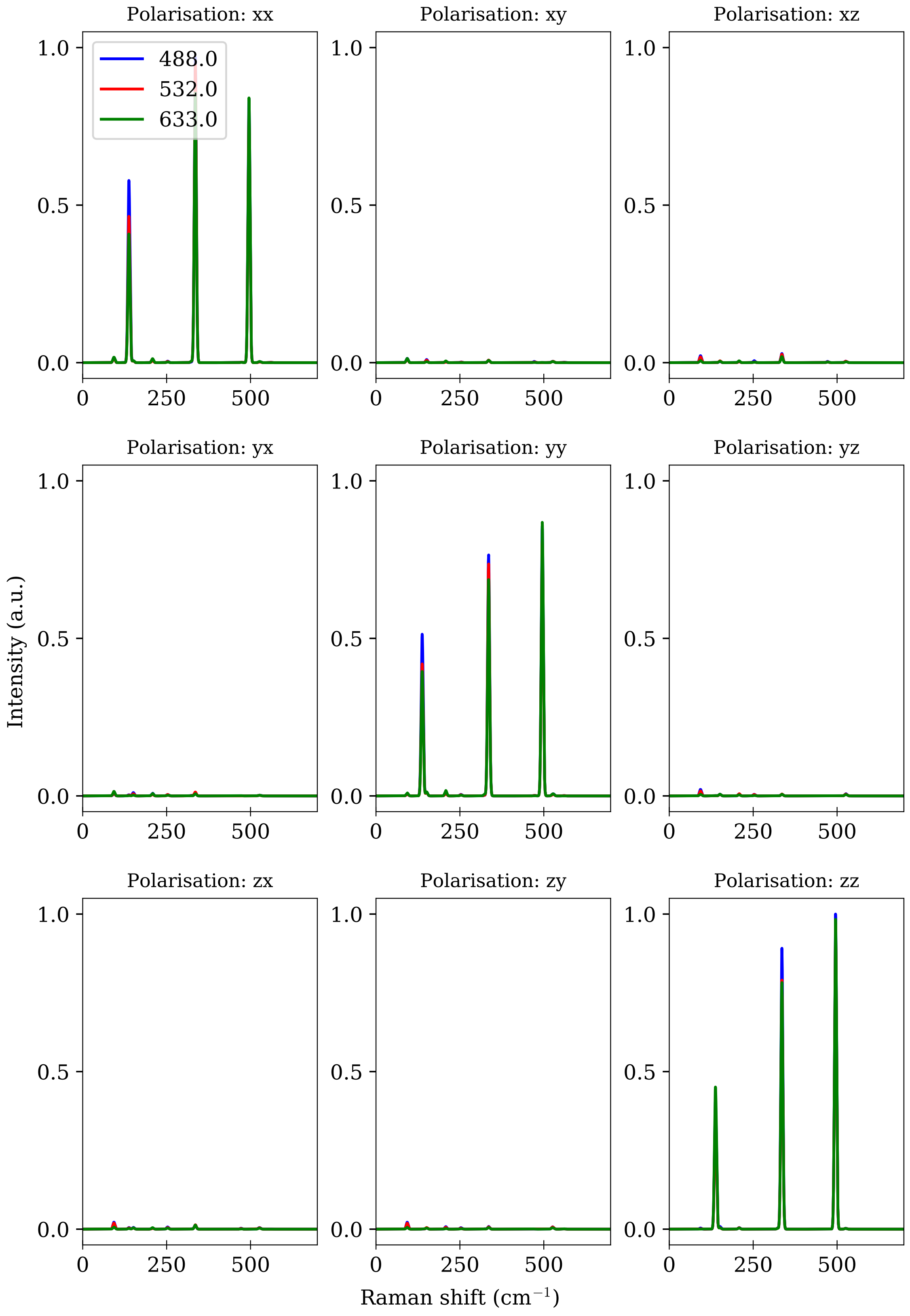

Supplementary Figure 525: Raman spectrum of Sc_4C_3 with space group 164 and Wyckoff positions b-c-d-0.

ScS_2, 164, b-d-0

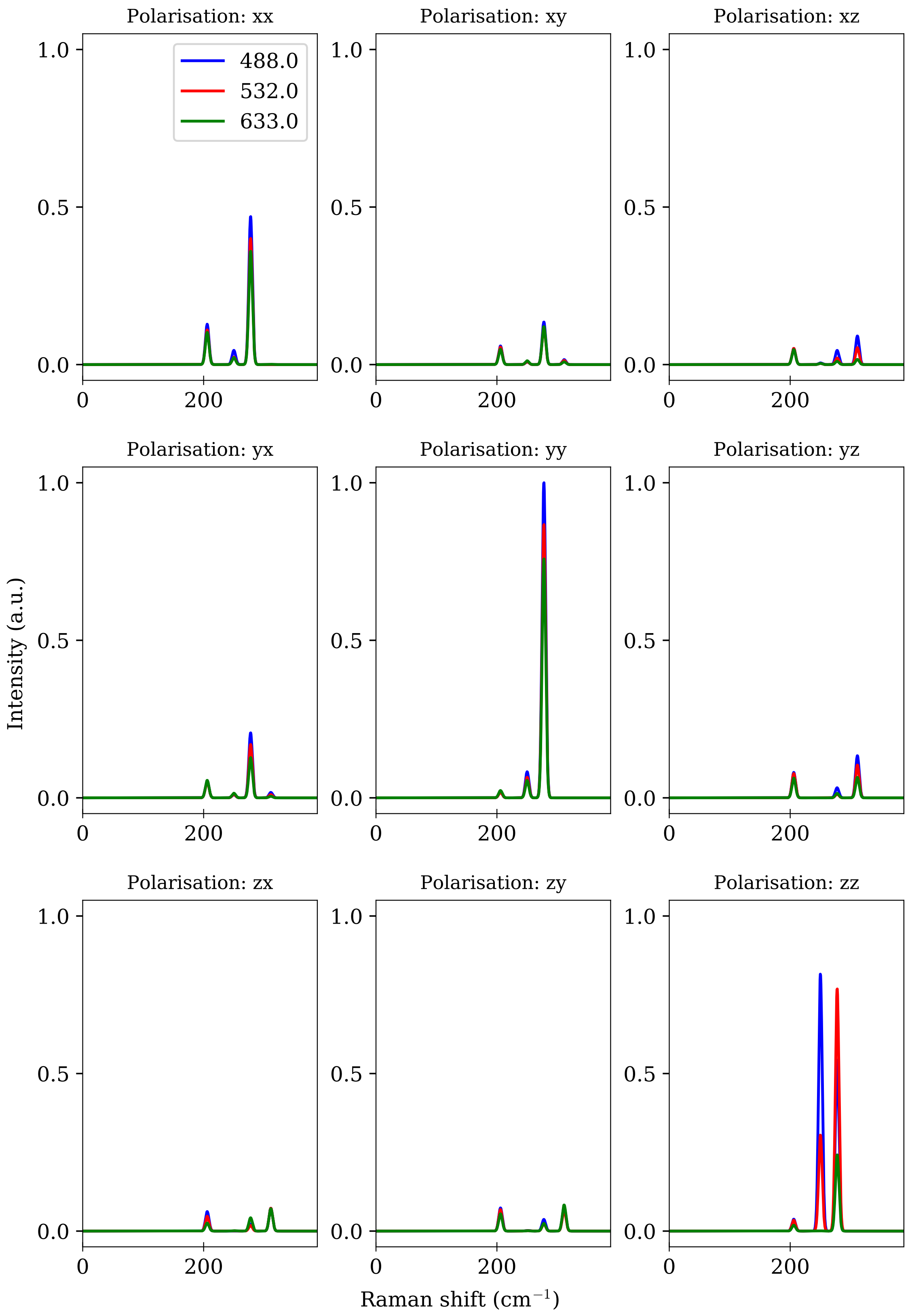

Supplementary Figure 526: Raman spectrum of ScS_2 with space group 164 and Wyckoff positions b-d-0.

$ScSe_2$, 164, b-d-0

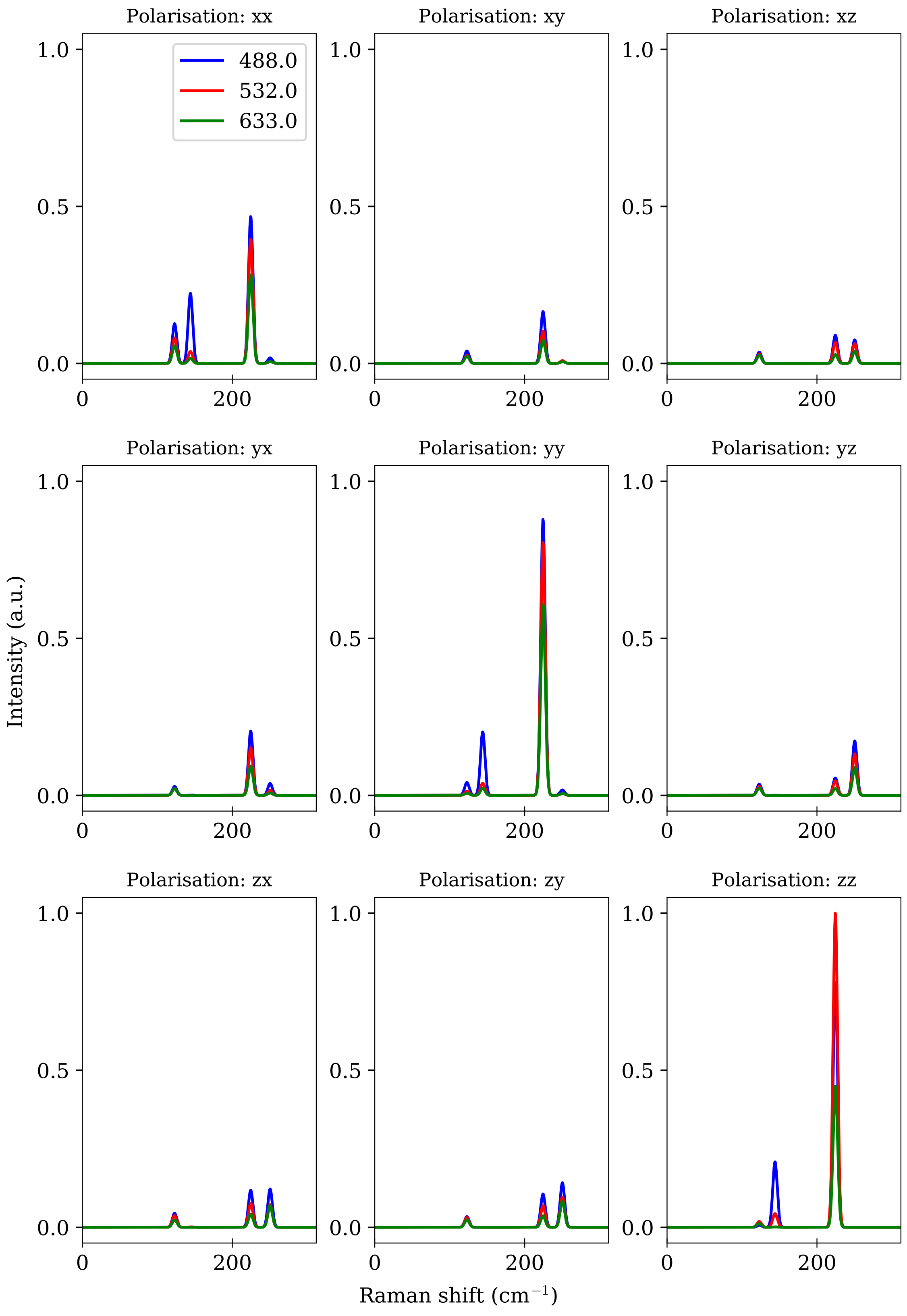

Supplementary Figure 527: Raman spectrum of $ScSe_2$ with space group 164 and Wyckoff positions b-d-0.

$ScTe_2$, 164, b-d-0

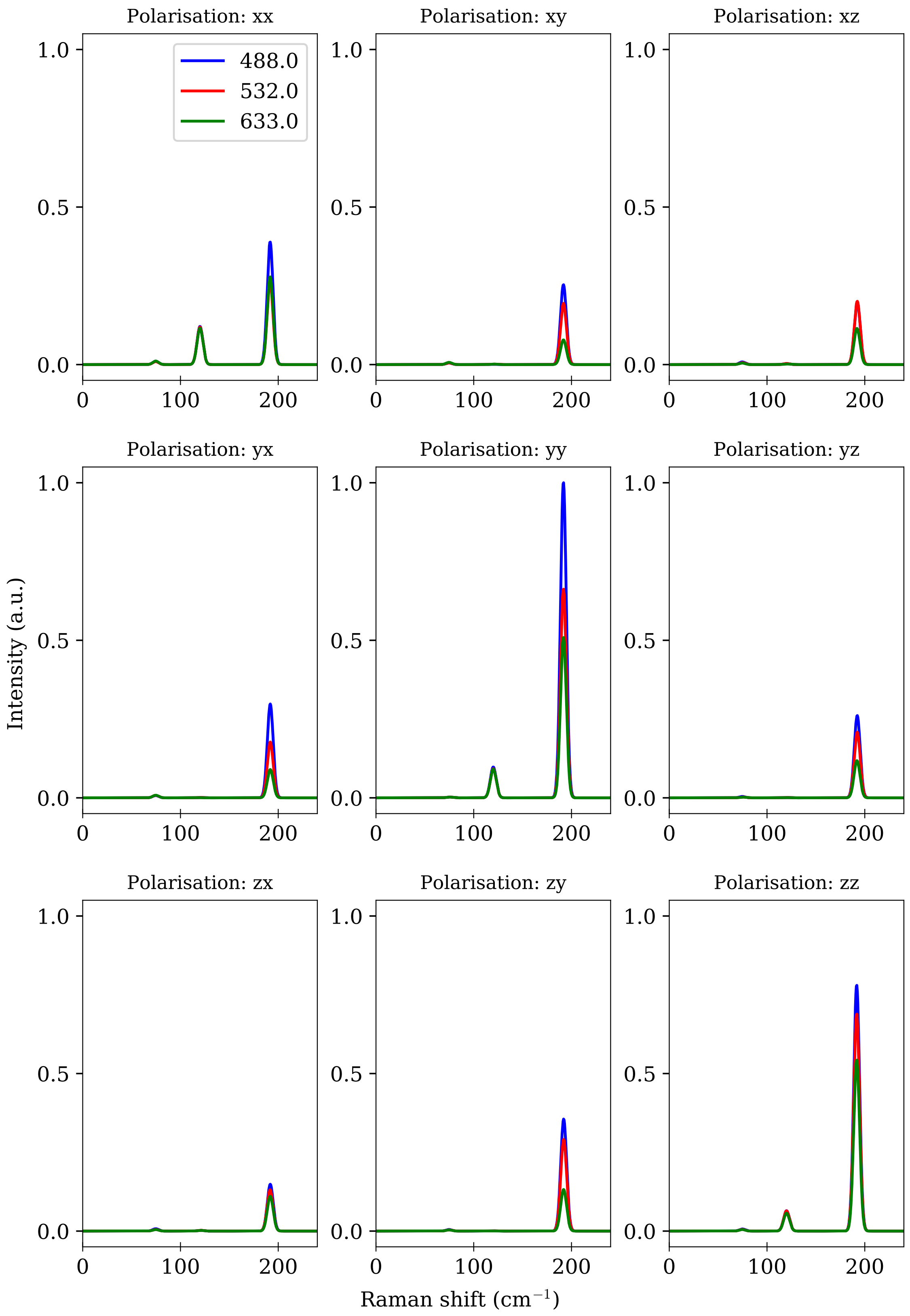

Supplementary Figure 528: Raman spectrum of $ScTe_2$ with space group 164 and Wyckoff positions b-d-0.

$ScTe_2$, 187, b-i-0

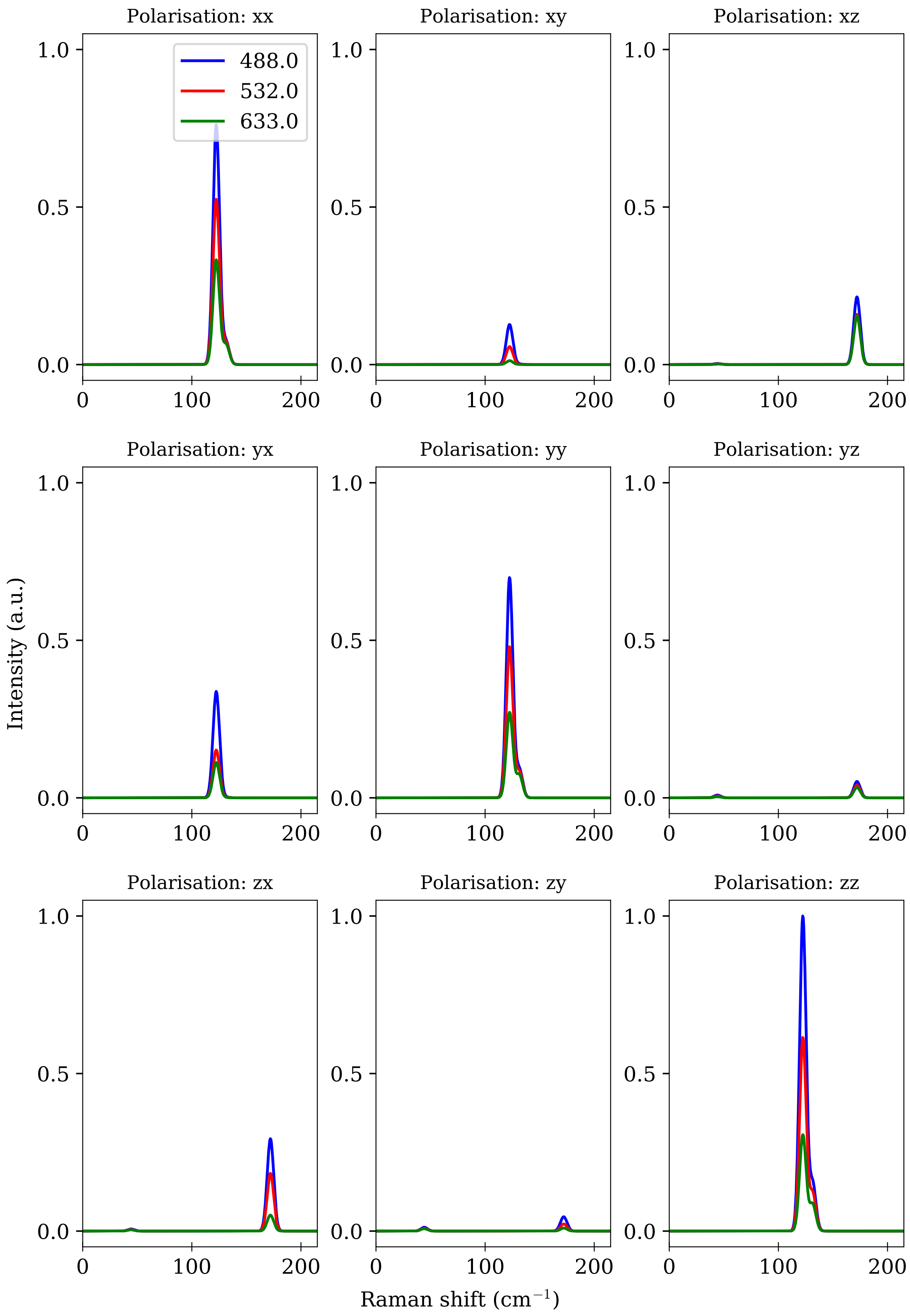

Supplementary Figure 529: Raman spectrum of $ScTe_2$ with space group 187 and Wyckoff positions b-i-0.

Si_2, 164, d-0

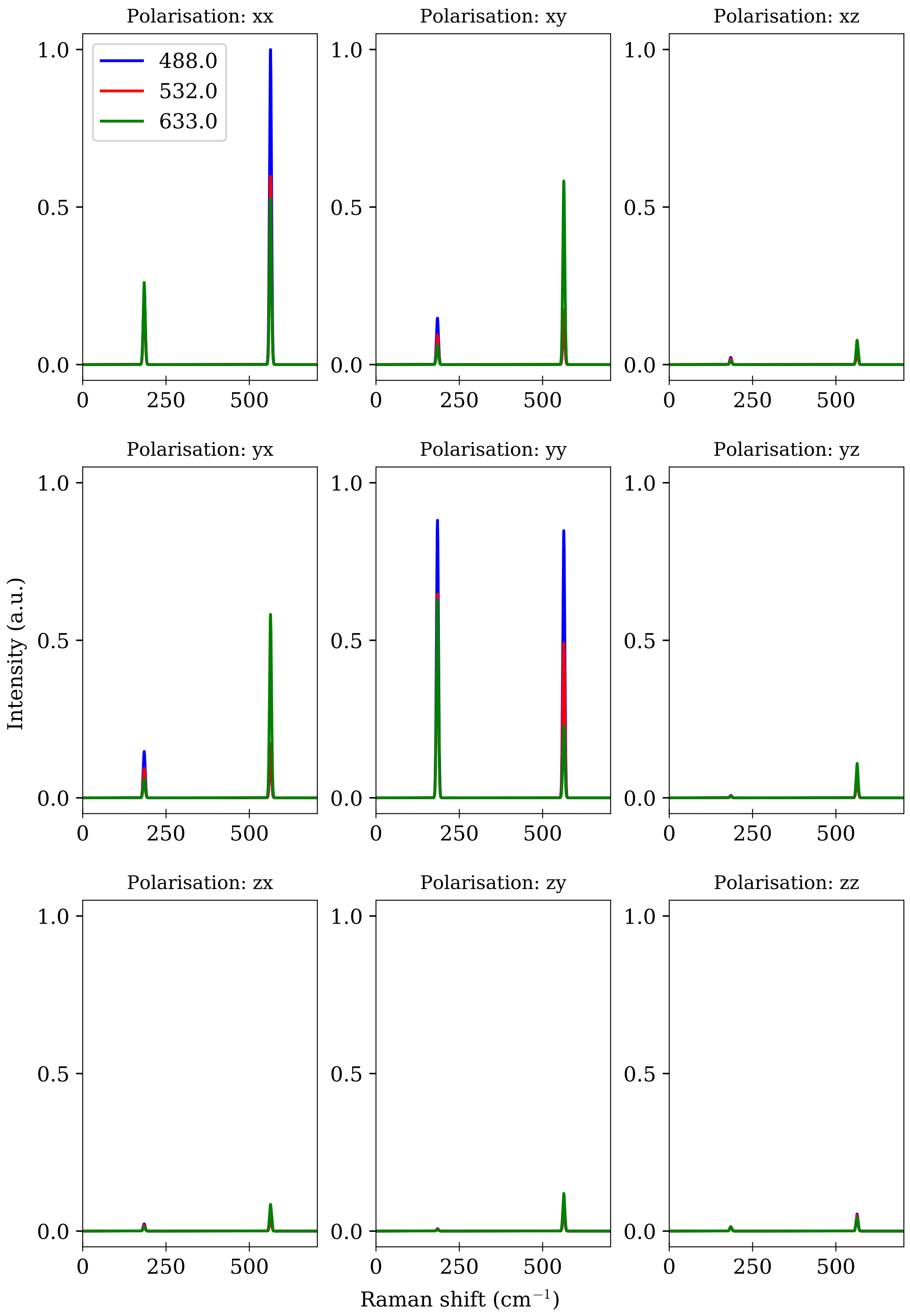

Supplementary Figure 530: Raman spectrum of Si_2 with space group 164 and Wyckoff positions d-0.

SnO_2, 164, b-d-0

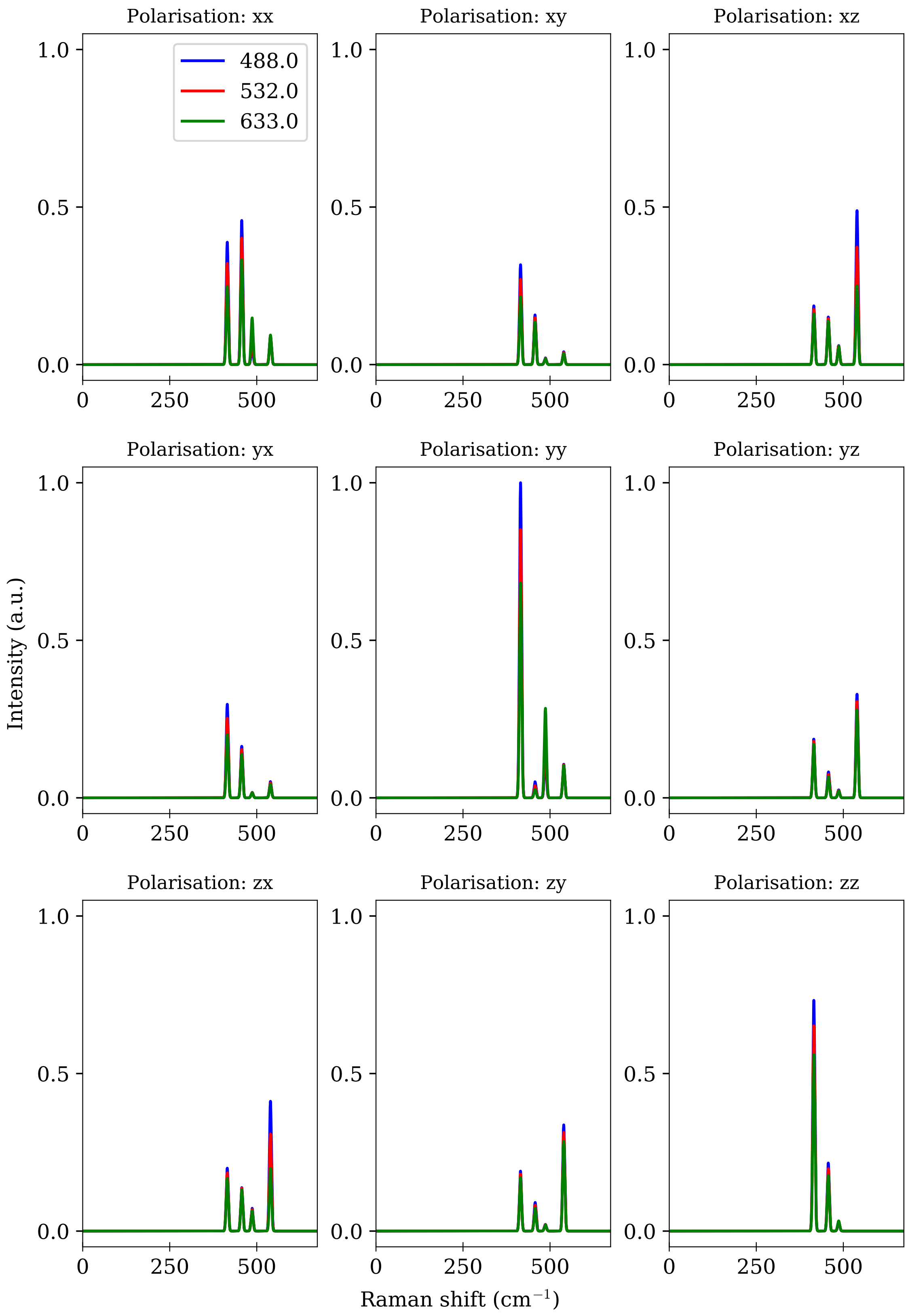

Supplementary Figure 531: Raman spectrum of SnO_2 with space group 164 and Wyckoff positions b-d-0.

SnO, 156, b-c-0

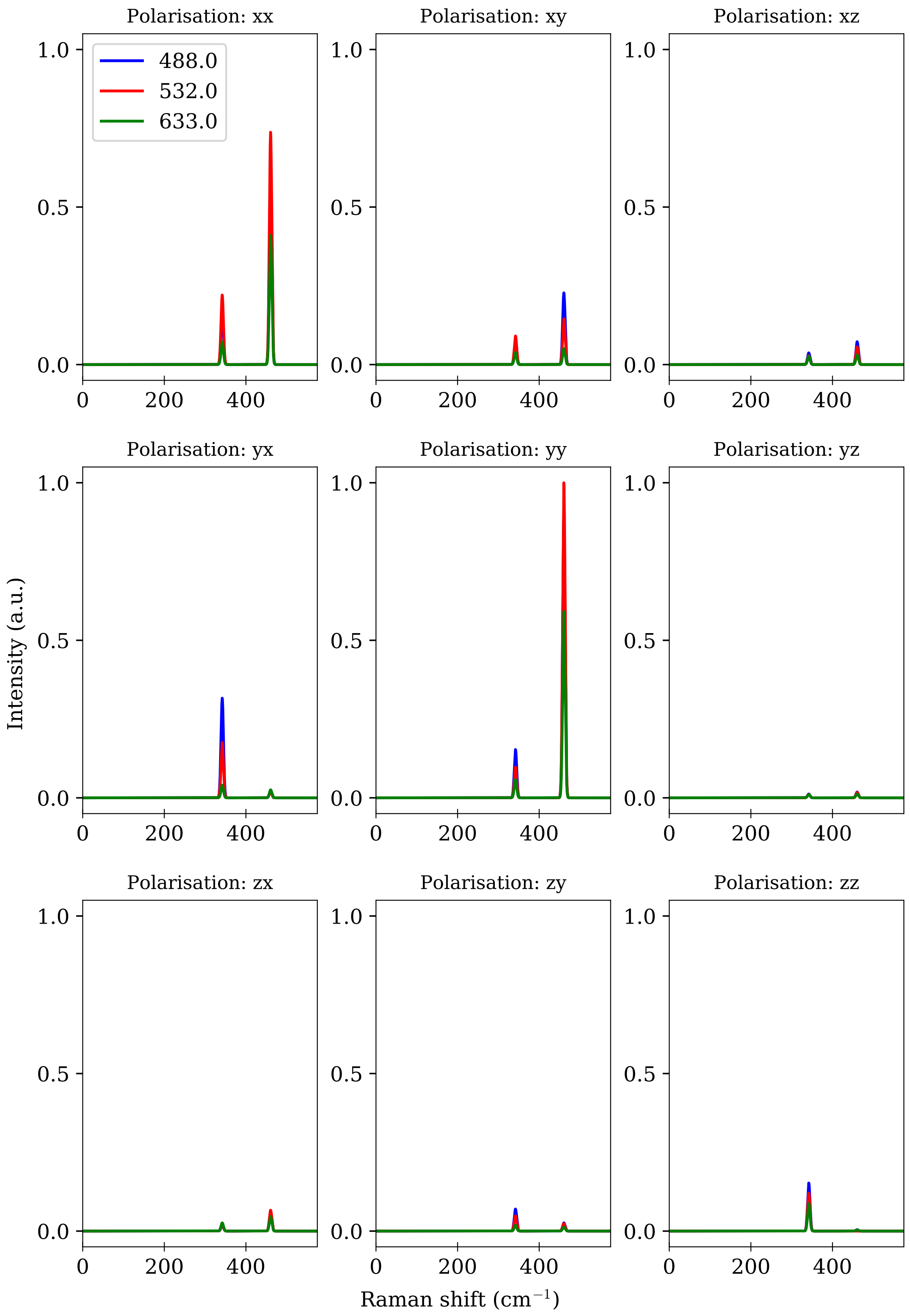

Supplementary Figure 532: Raman spectrum of SnO with space group 156 and Wyckoff positions b-c-0.

SnS_2, 115, d-g-0

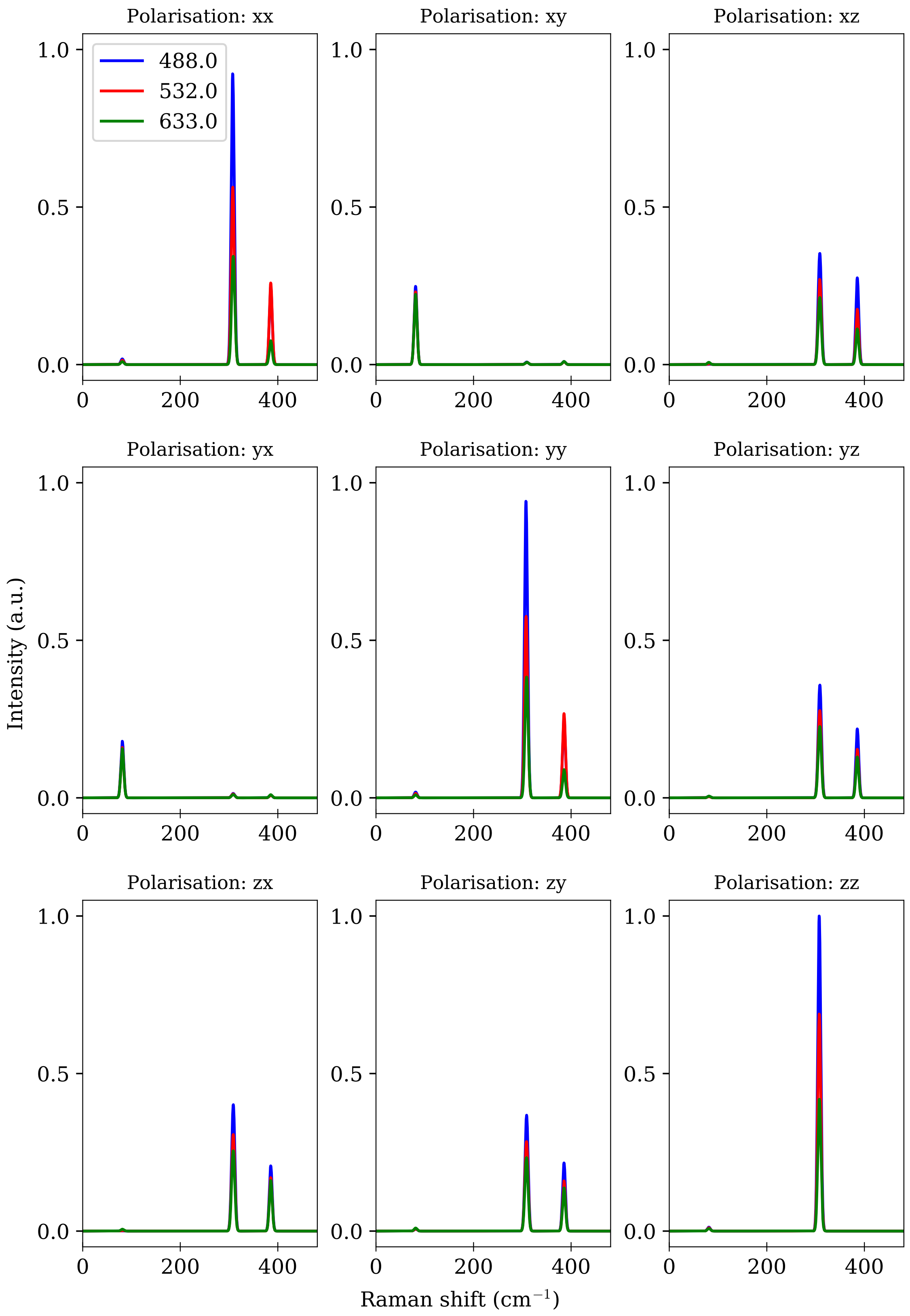

Supplementary Figure 533: Raman spectrum of SnS_2 with space group 115 and Wyckoff positions d-g-0.

SnS_2, 164, b-d-0

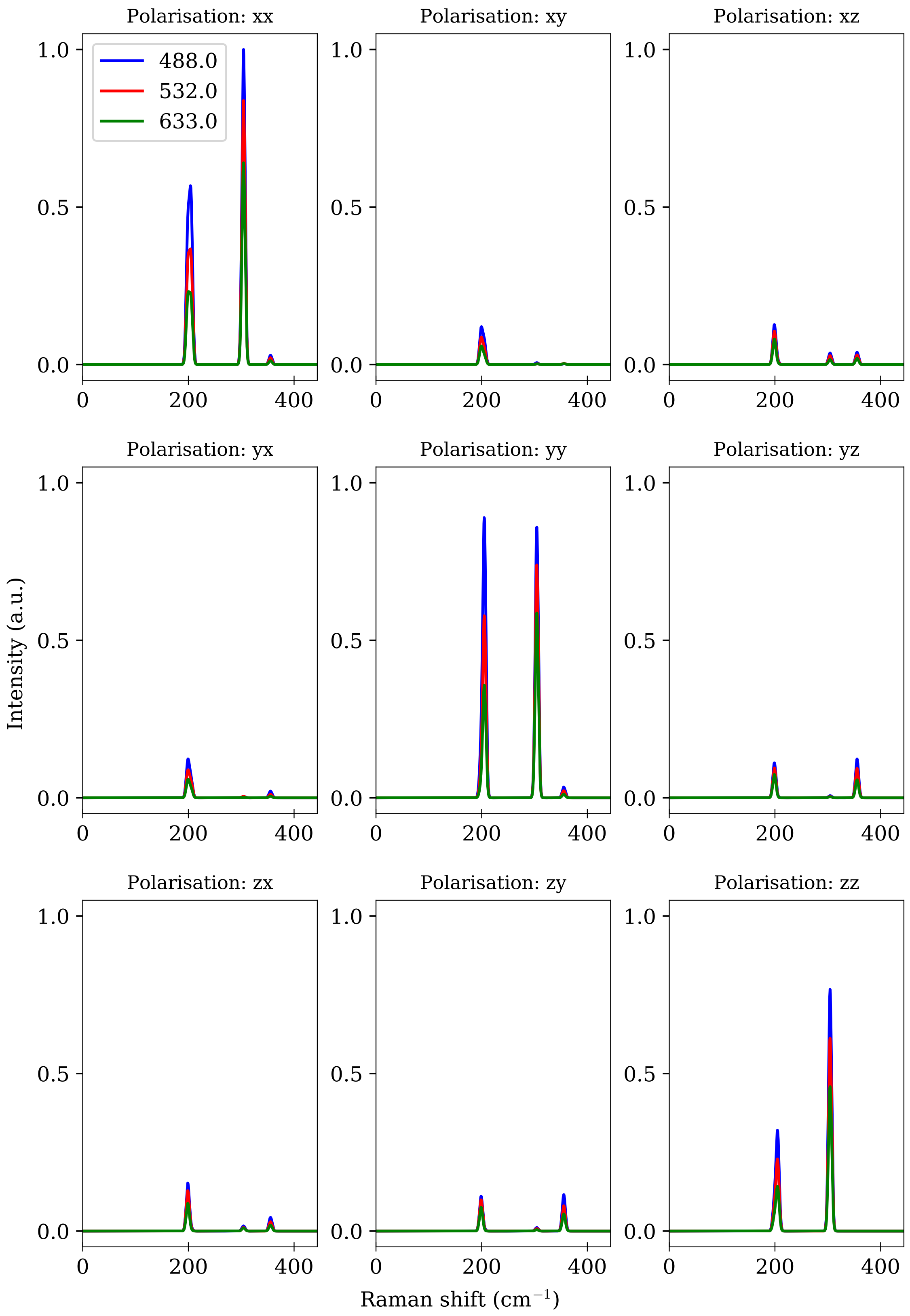

Supplementary Figure 534: Raman spectrum of SnS_2 with space group 164 and Wyckoff positions b-d-0.

$SnSe_2$, 164, b-d-0

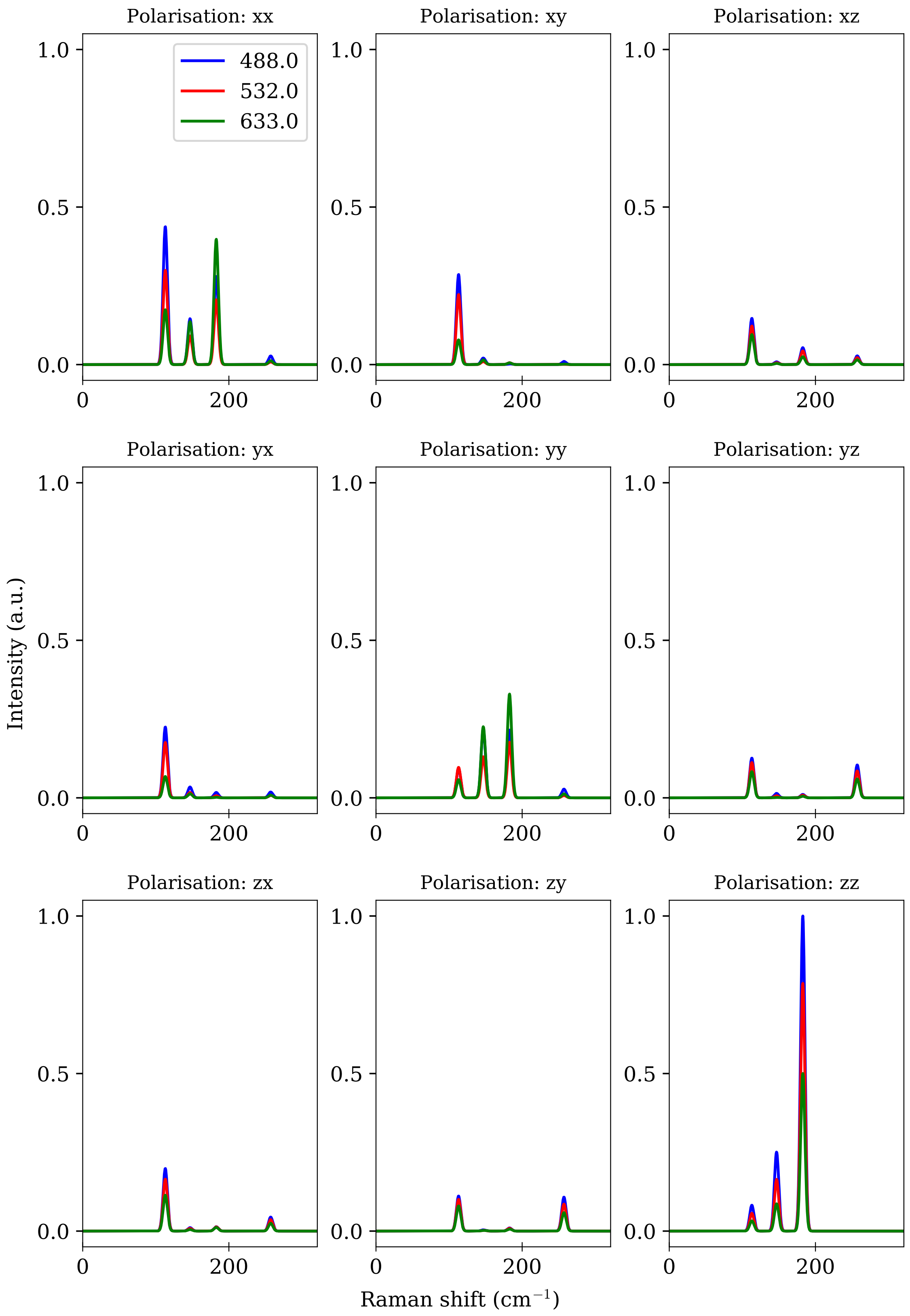

Supplementary Figure 535: Raman spectrum of $SnSe_2$ with space group 164 and Wyckoff positions b-d-0.

SnSe, 156, b-c-0

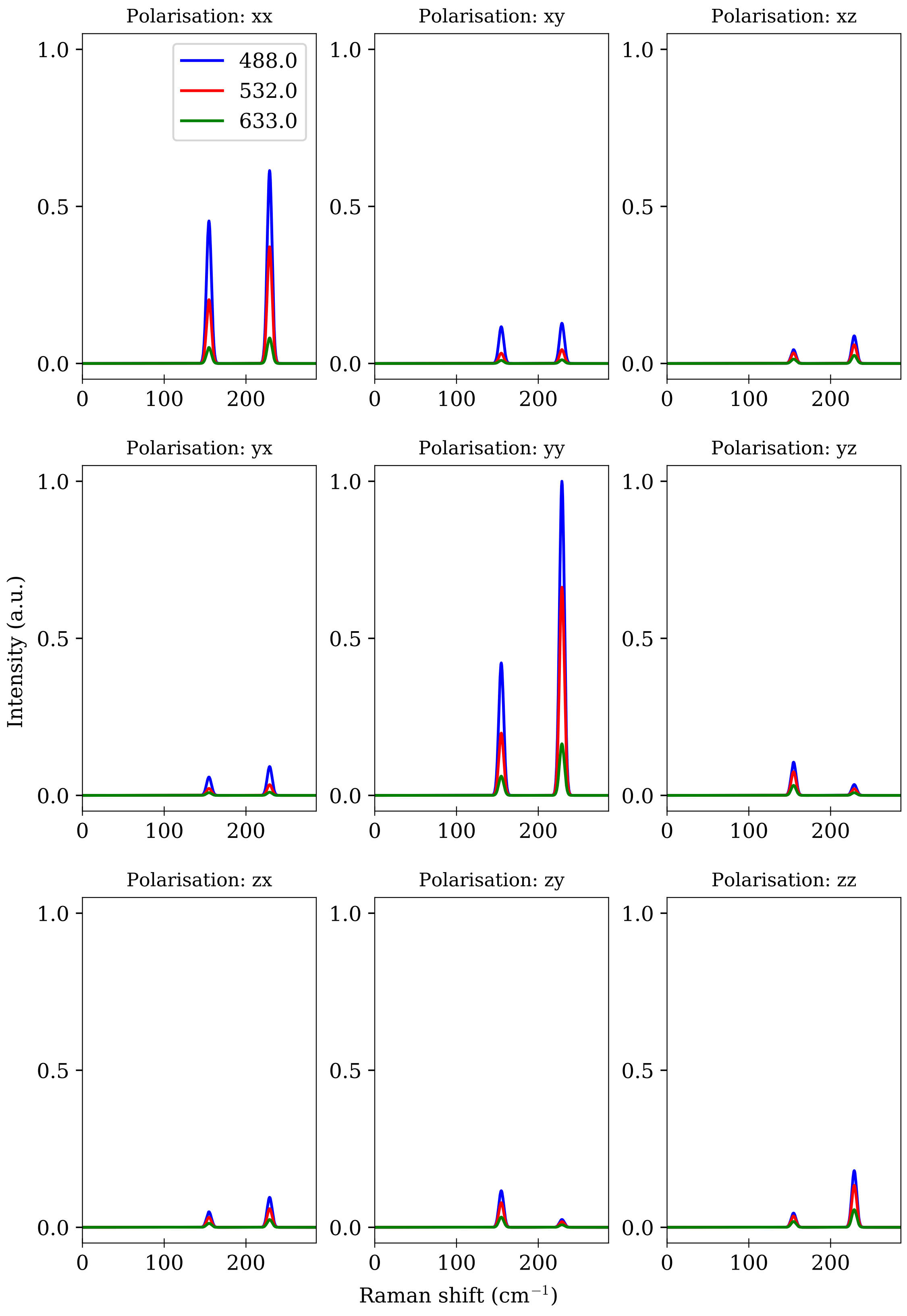

Supplementary Figure 536: Raman spectrum of SnSe with space group 156 and Wyckoff positions b-c-0.

SnS, 156, b-c-0

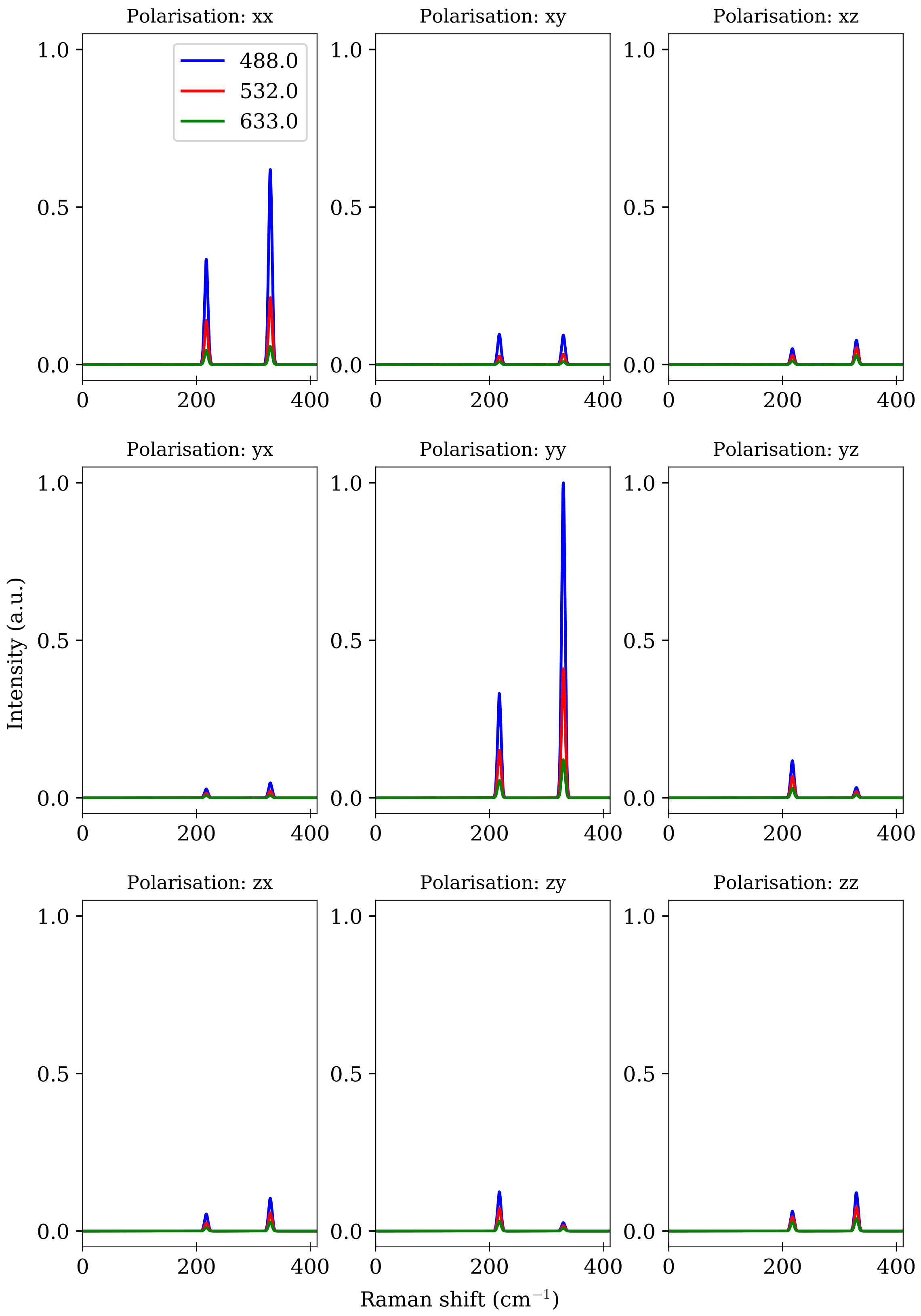

Supplementary Figure 537: Raman spectrum of SnS with space group 156 and Wyckoff positions b-c-0.

SnTe, 156, b-c-0

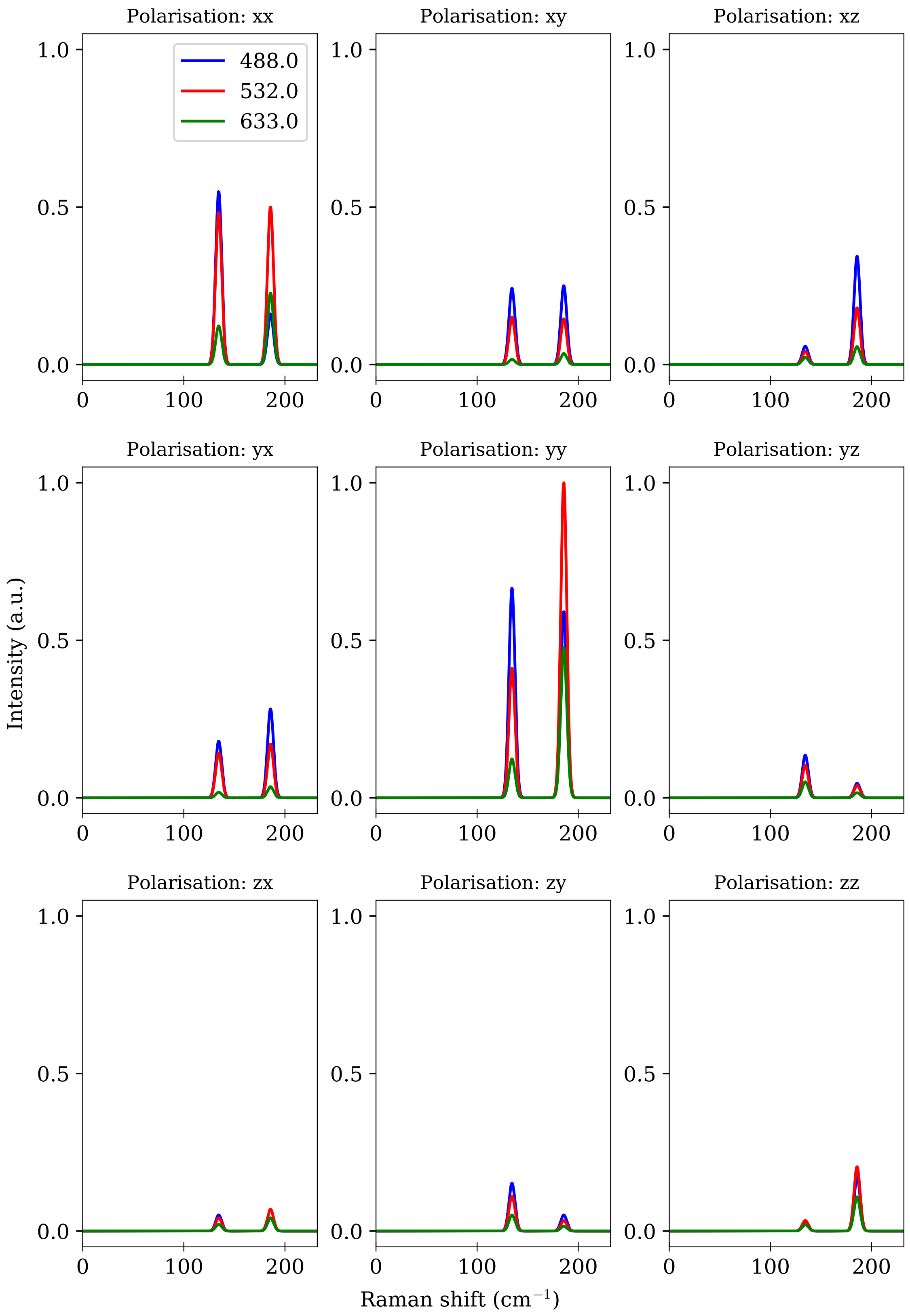

Supplementary Figure 538: Raman spectrum of SnTe with space group 156 and Wyckoff positions b-c-0.

$SrBr_2$, 164, b-d-0

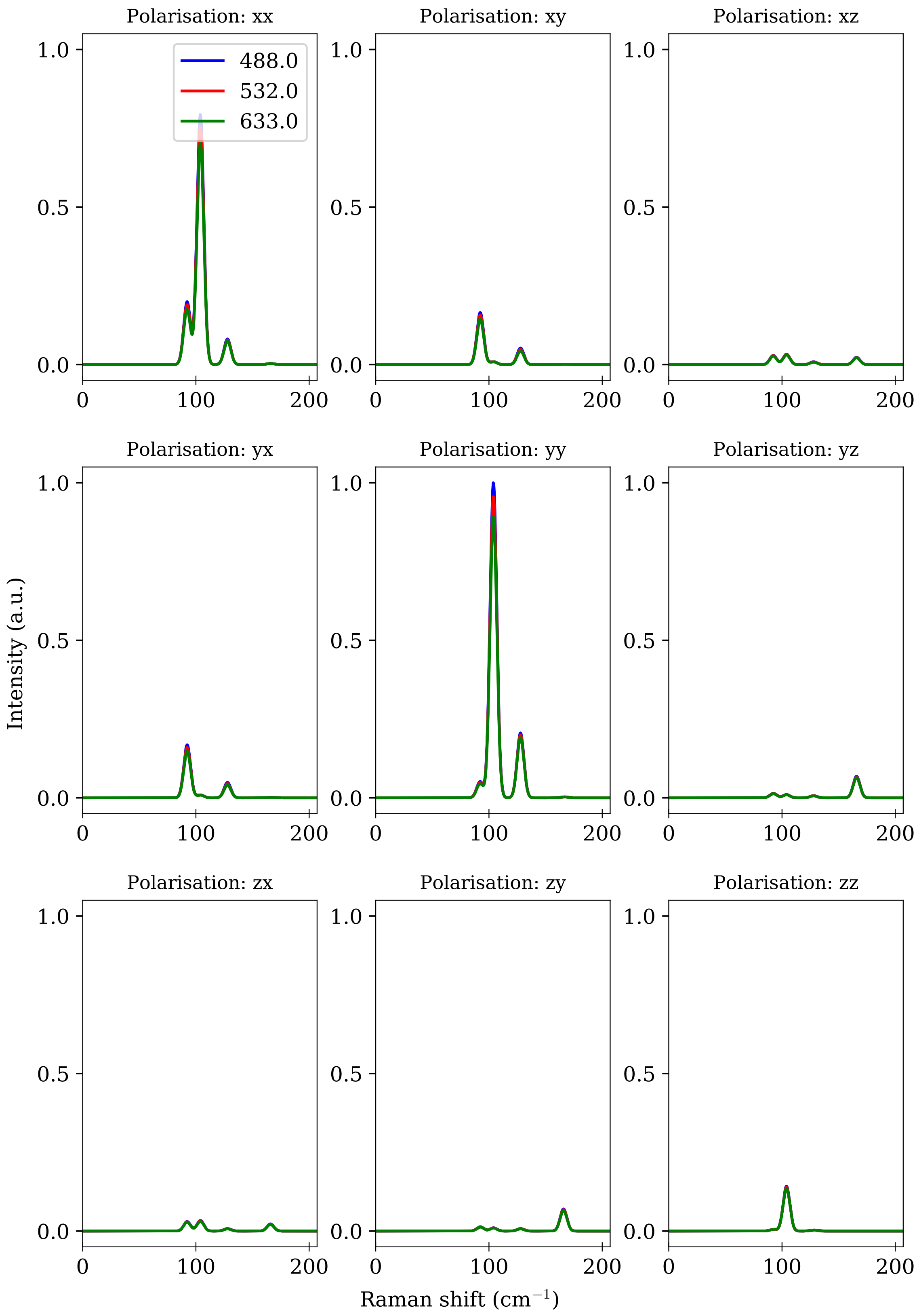

Supplementary Figure 539: Raman spectrum of $SrBr_2$ with space group 164 and Wyckoff positions b-d-0.

$SrCl_2$, 187, b-i-0

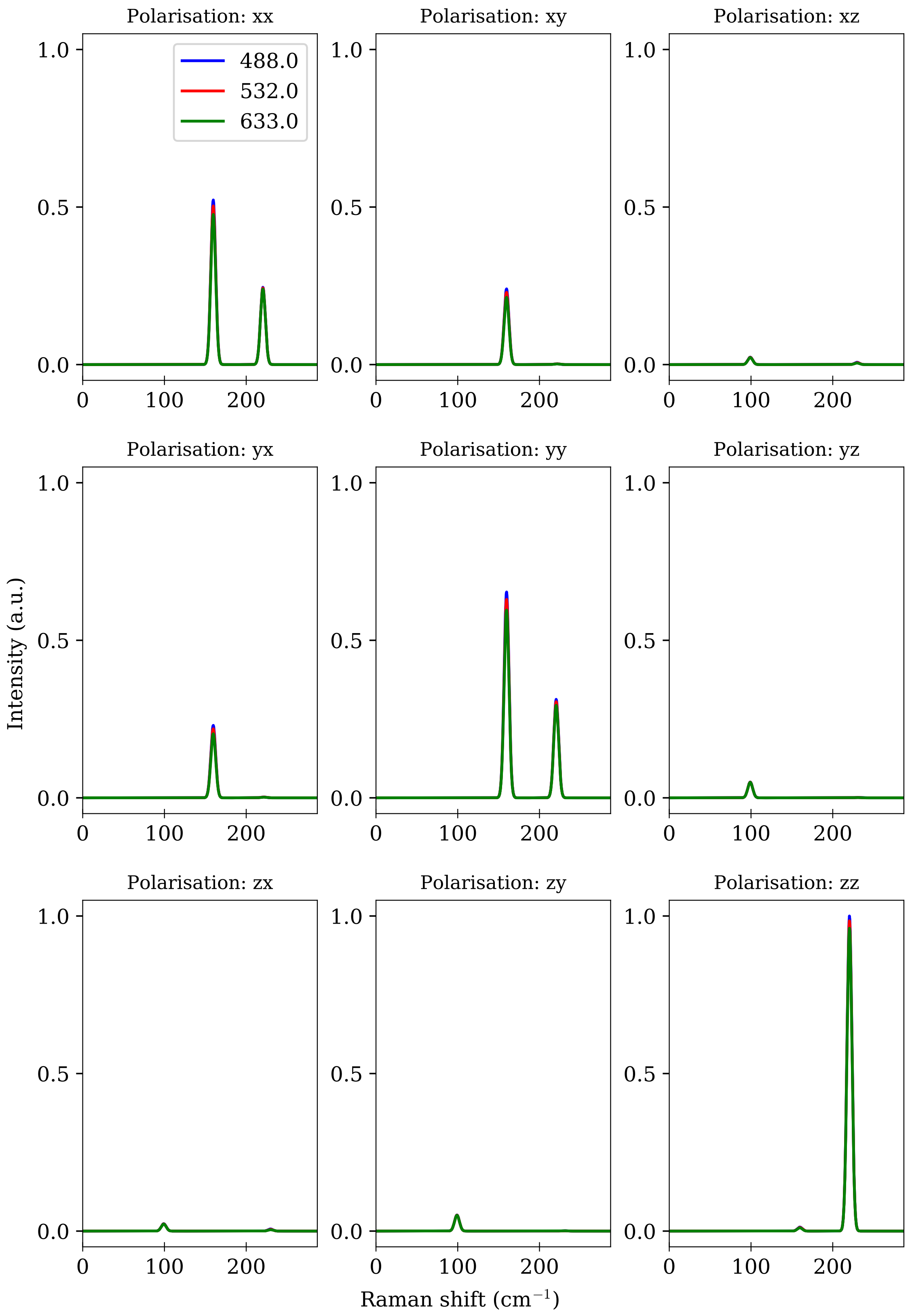

Supplementary Figure 540: Raman spectrum of $SrCl_2$ with space group 187 and Wyckoff positions b-i-0.

SrI_2, 164, b-d-0

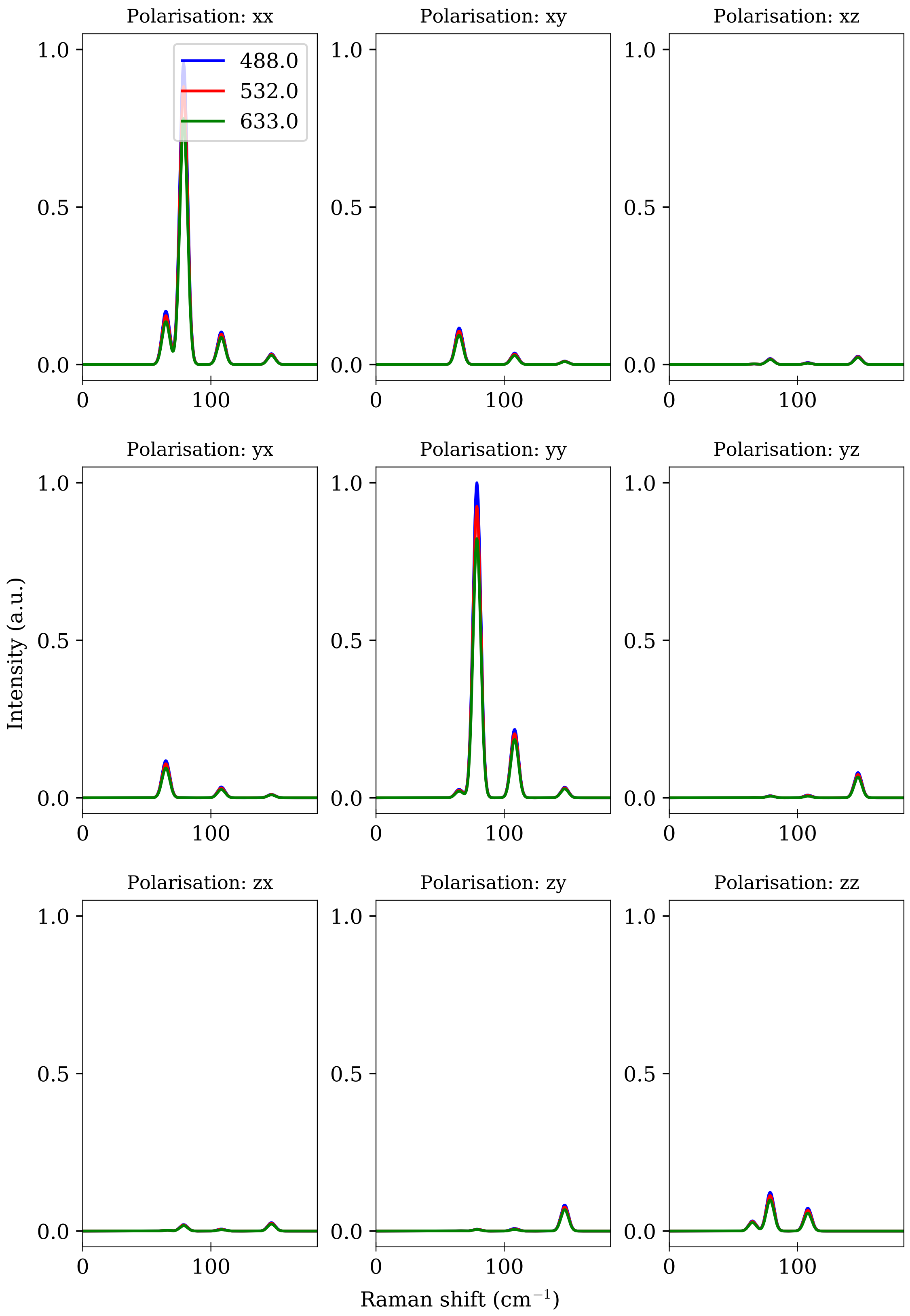

Supplementary Figure 541: Raman spectrum of SrI_2 with space group 164 and Wyckoff positions b-d-0.

SrI_2, 187, b-i-0

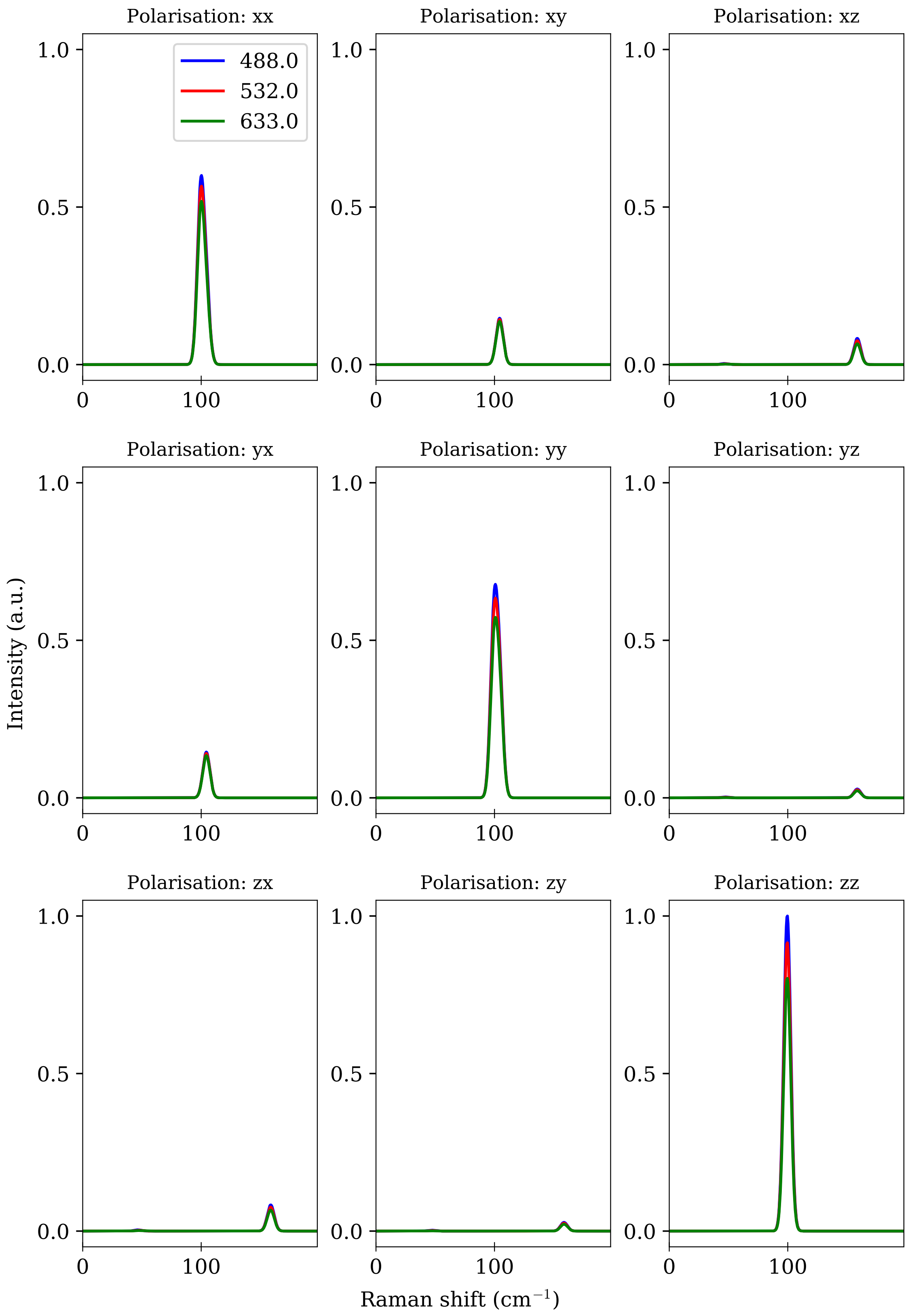

Supplementary Figure 542: Raman spectrum of SrI_2 with space group 187 and Wyckoff positions b-i-0.

Ta_2Br_4, 11, e-0

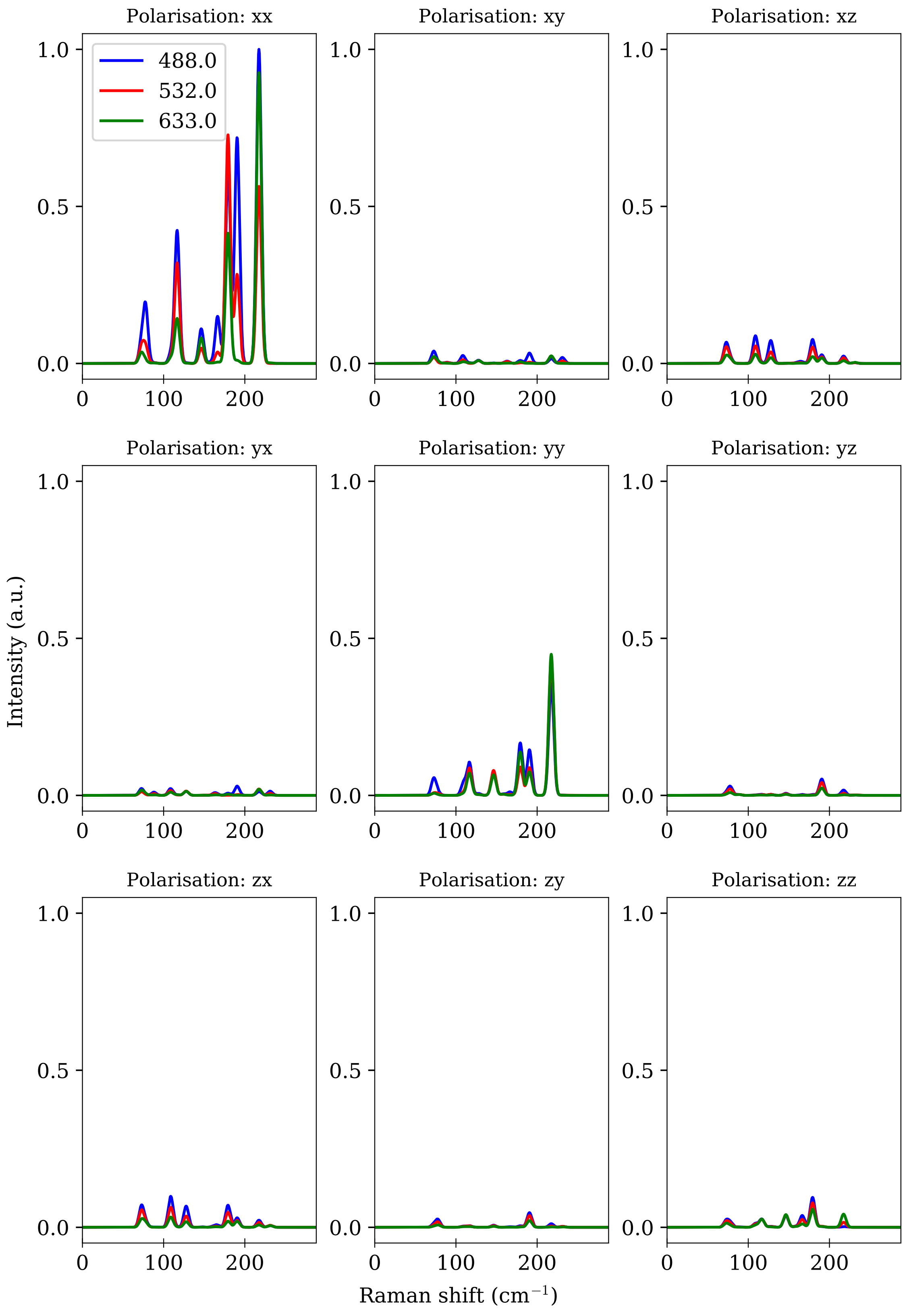

Supplementary Figure 543: Raman spectrum of Ta_2Br_4 with space group 11 and Wyckoff positions e-0.

Ta_2CO_2, 164, b-d-0

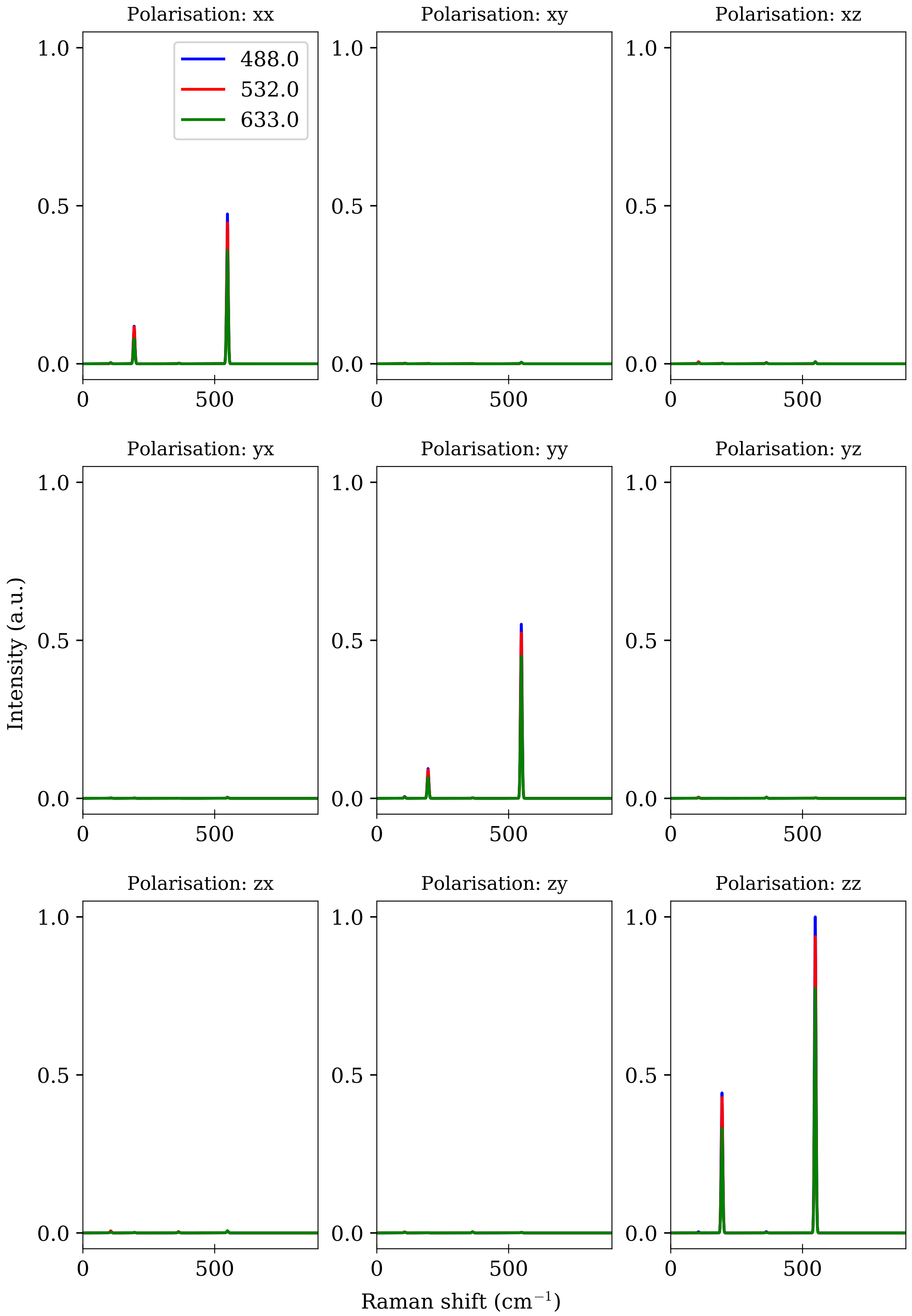

Supplementary Figure 544: Raman spectrum of Ta_2CO_2 with space group 164 and Wyckoff positions b-d-0.

Ta_2C, 164, b-d-0

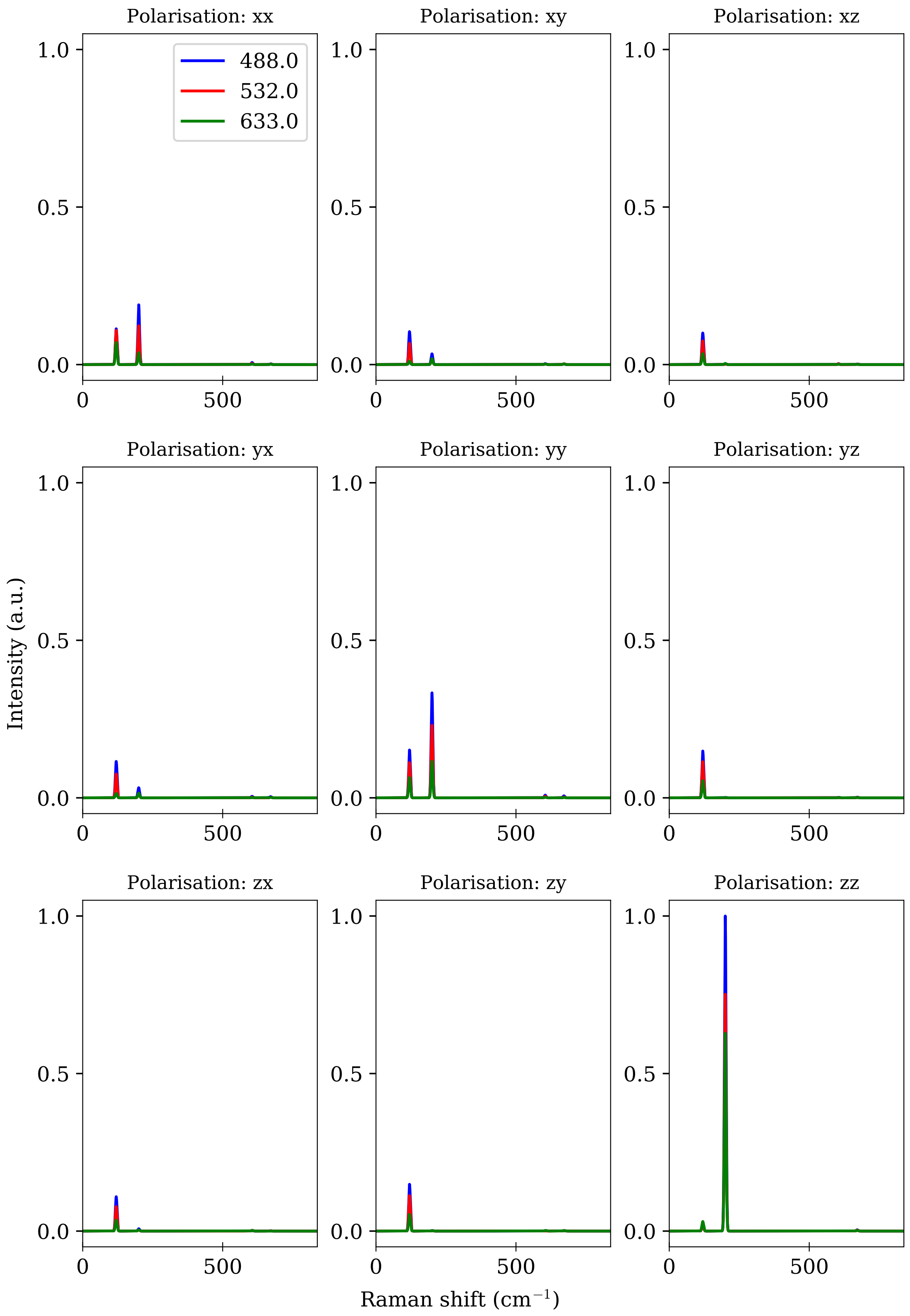

Supplementary Figure 545: Raman spectrum of Ta_2C with space group 164 and Wyckoff positions b-d-0.

Ta_2I_4, 11, e-0

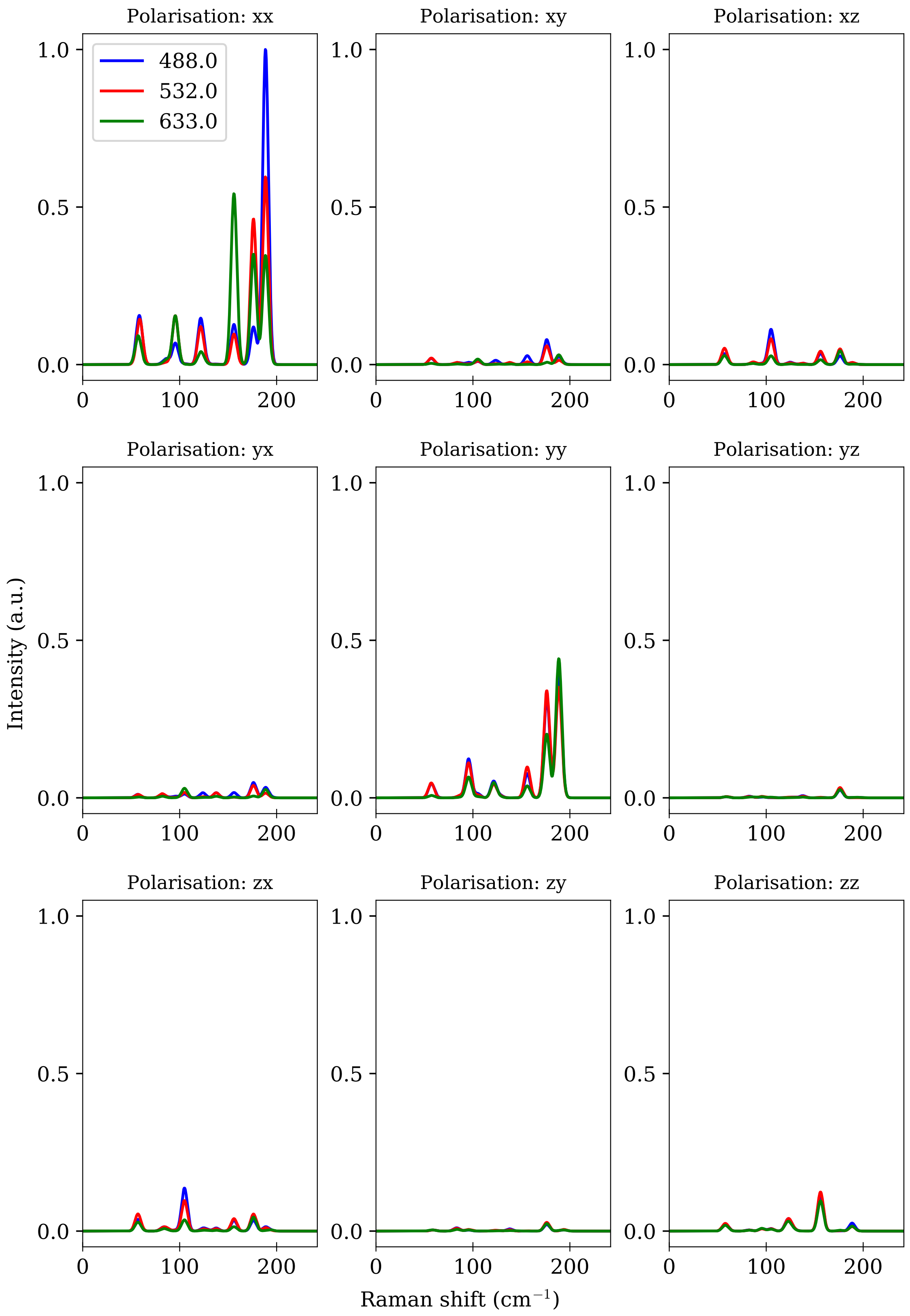

Supplementary Figure 546: Raman spectrum of Ta_2I_4 with space group 11 and Wyckoff positions e-0.

Ta_2N, 164, b-d-0

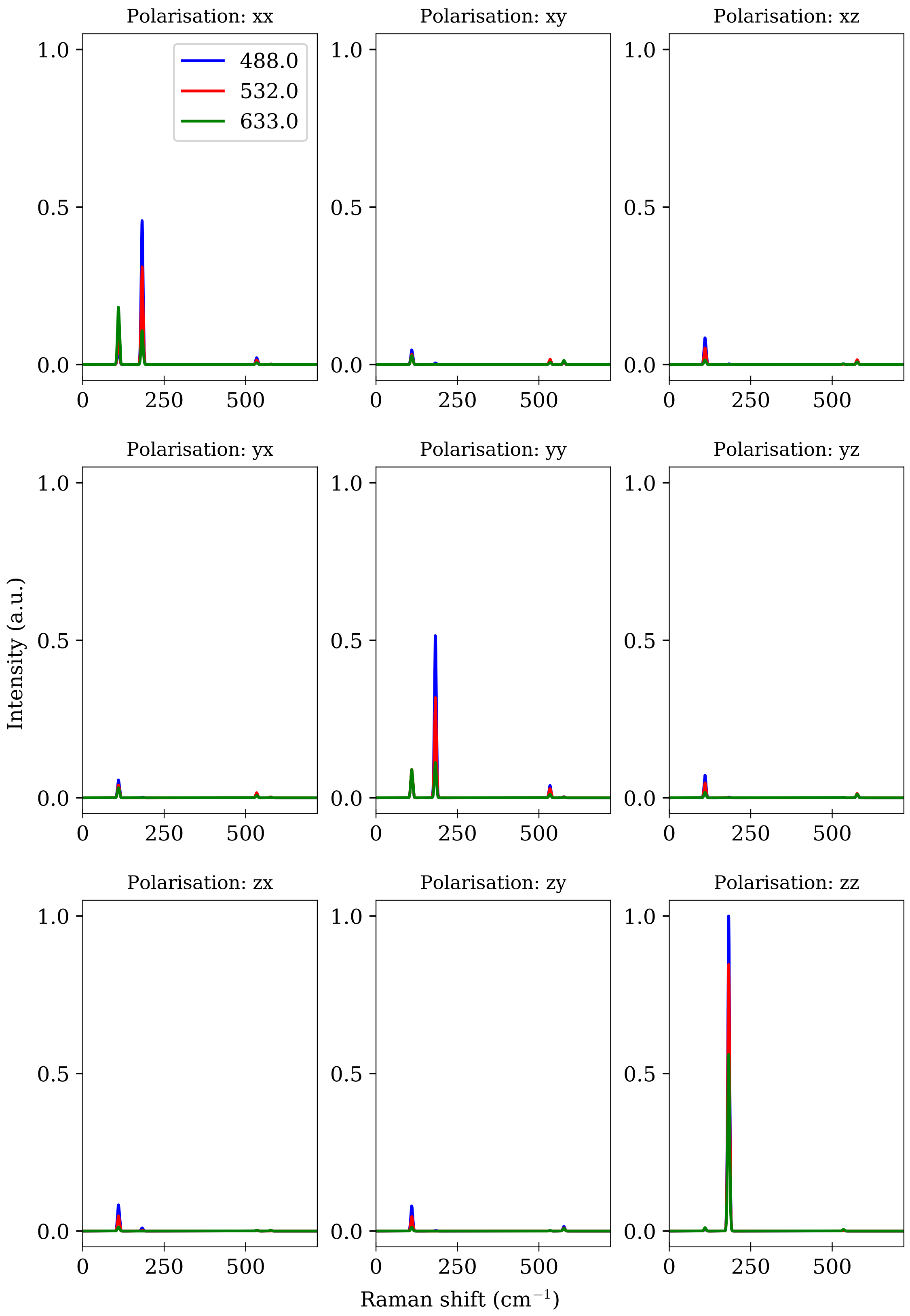

Supplementary Figure 547: Raman spectrum of Ta_2N with space group 164 and Wyckoff positions b-d-0.

Ta_2O_2, 6, a-b-0

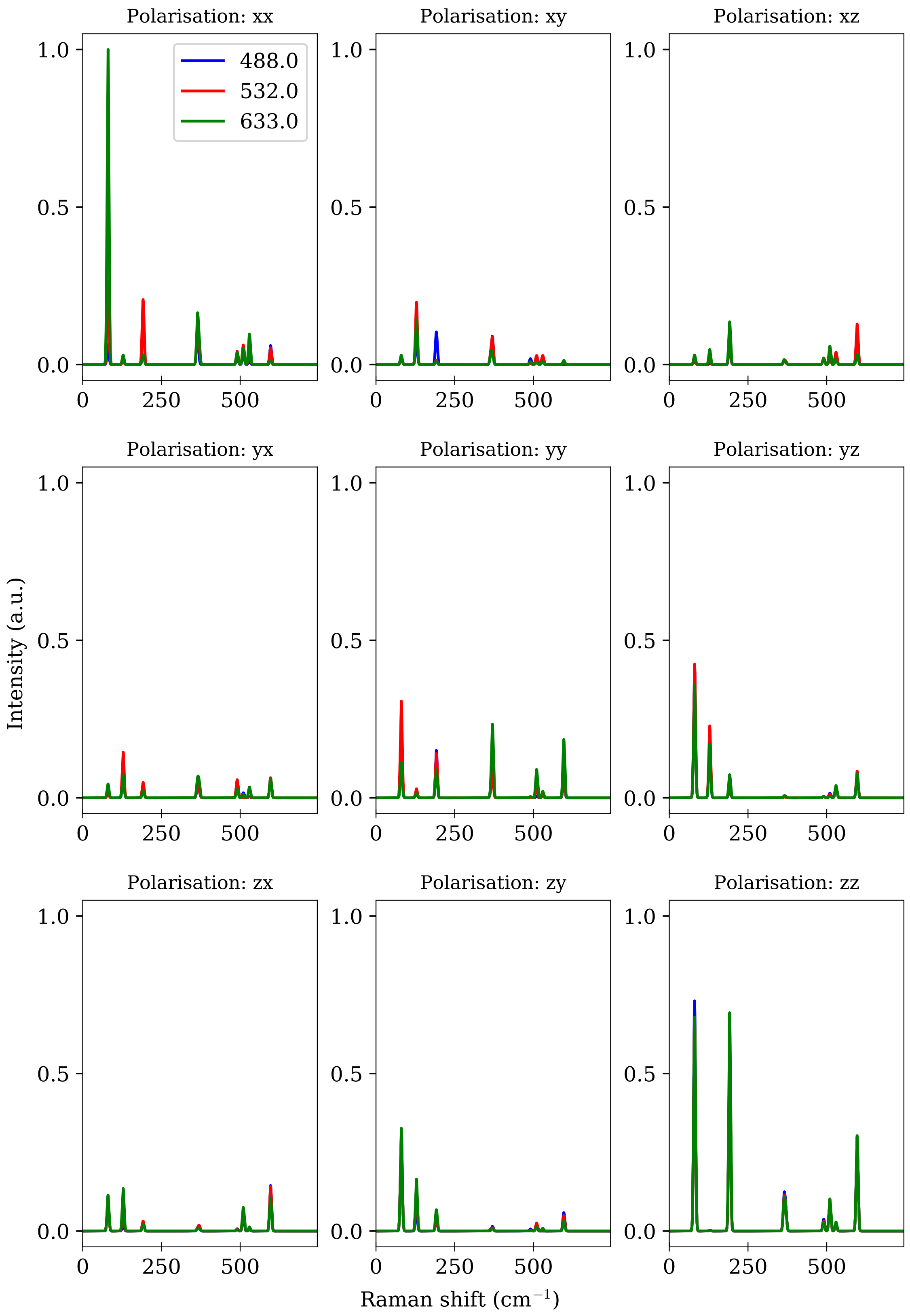

Supplementary Figure 548: Raman spectrum of Ta_2O_2 with space group 6 and Wyckoff positions a-b-0.

Ta_2S_6, 59, a-b-e-0

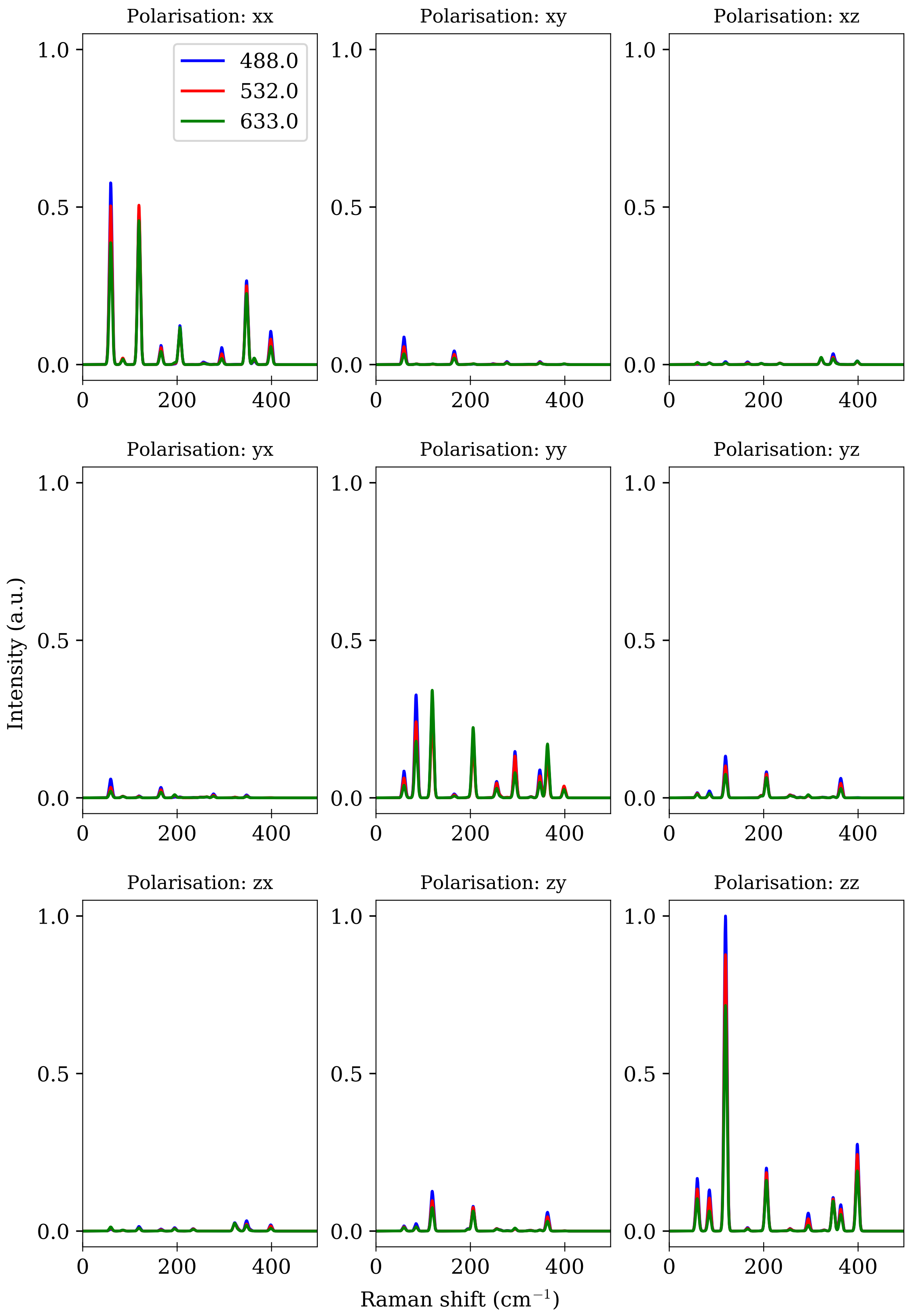

Supplementary Figure 549: Raman spectrum of Ta_2S_6 with space group 59 and Wyckoff positions a-b-e-0.

Ta_2Se_2, 164, d-0

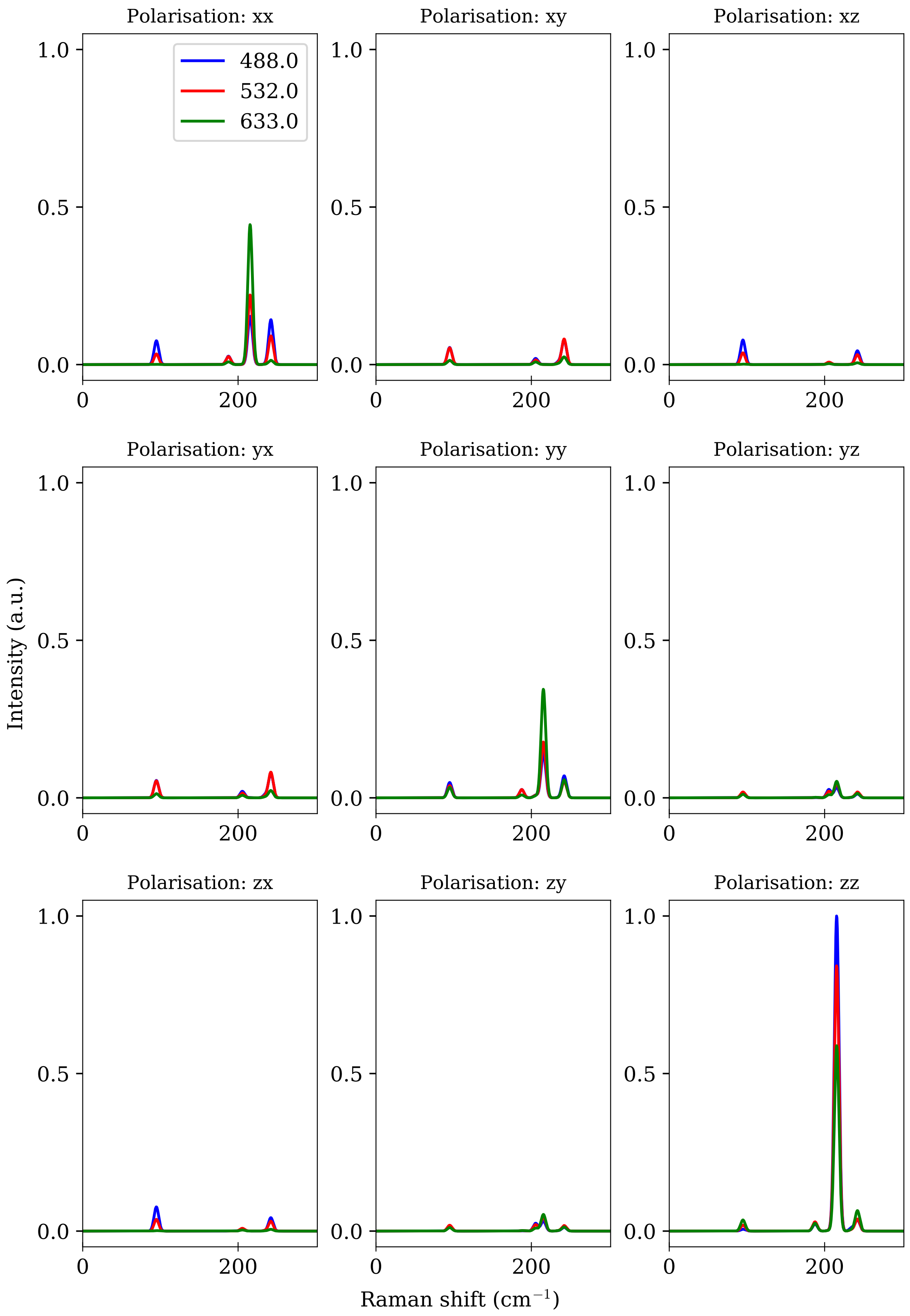

Supplementary Figure 550: Raman spectrum of Ta_2Se_2 with space group 164 and Wyckoff positions d-0.

Ta_2Se_6, 59, a-b-e-0

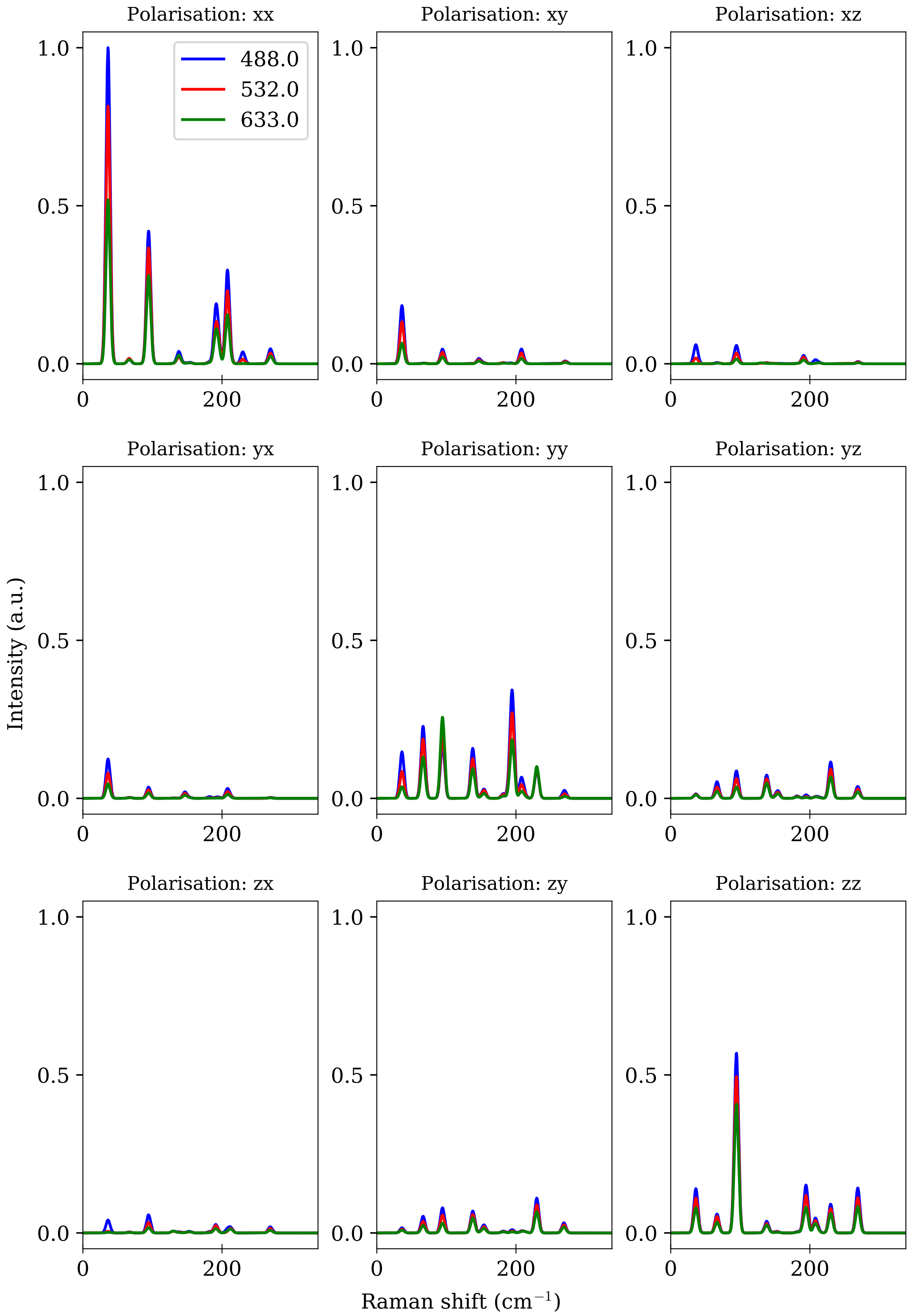

Supplementary Figure 551: Raman spectrum of Ta_2Se_6 with space group 59 and Wyckoff positions a-b-e-0.

Ta_2Te_2, 164, d-0

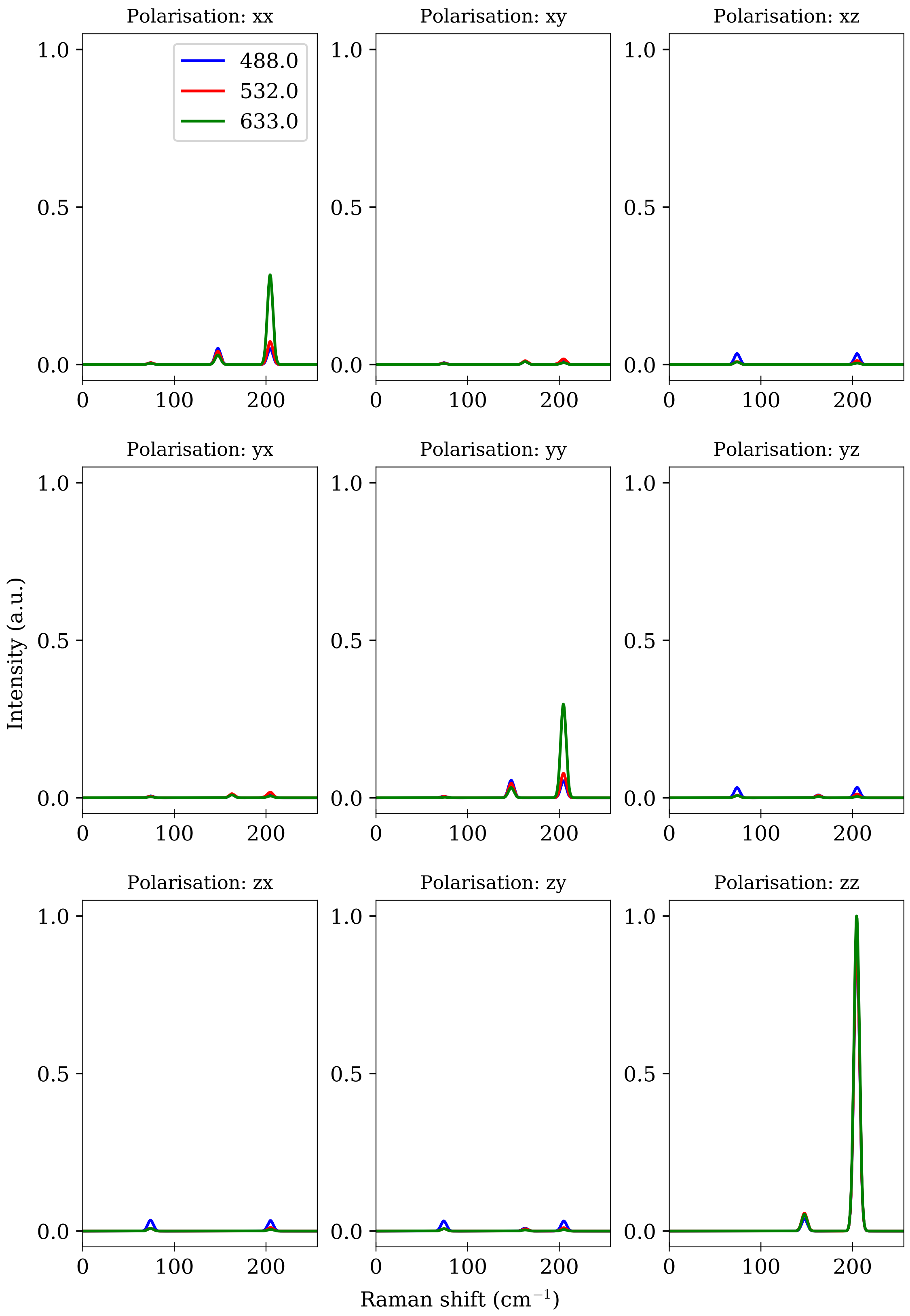

Supplementary Figure 552: Raman spectrum of Ta_2Te_2 with space group 164 and Wyckoff positions d-0.

$Ta_3C_2O_2$, 187, b-g-h-i-0

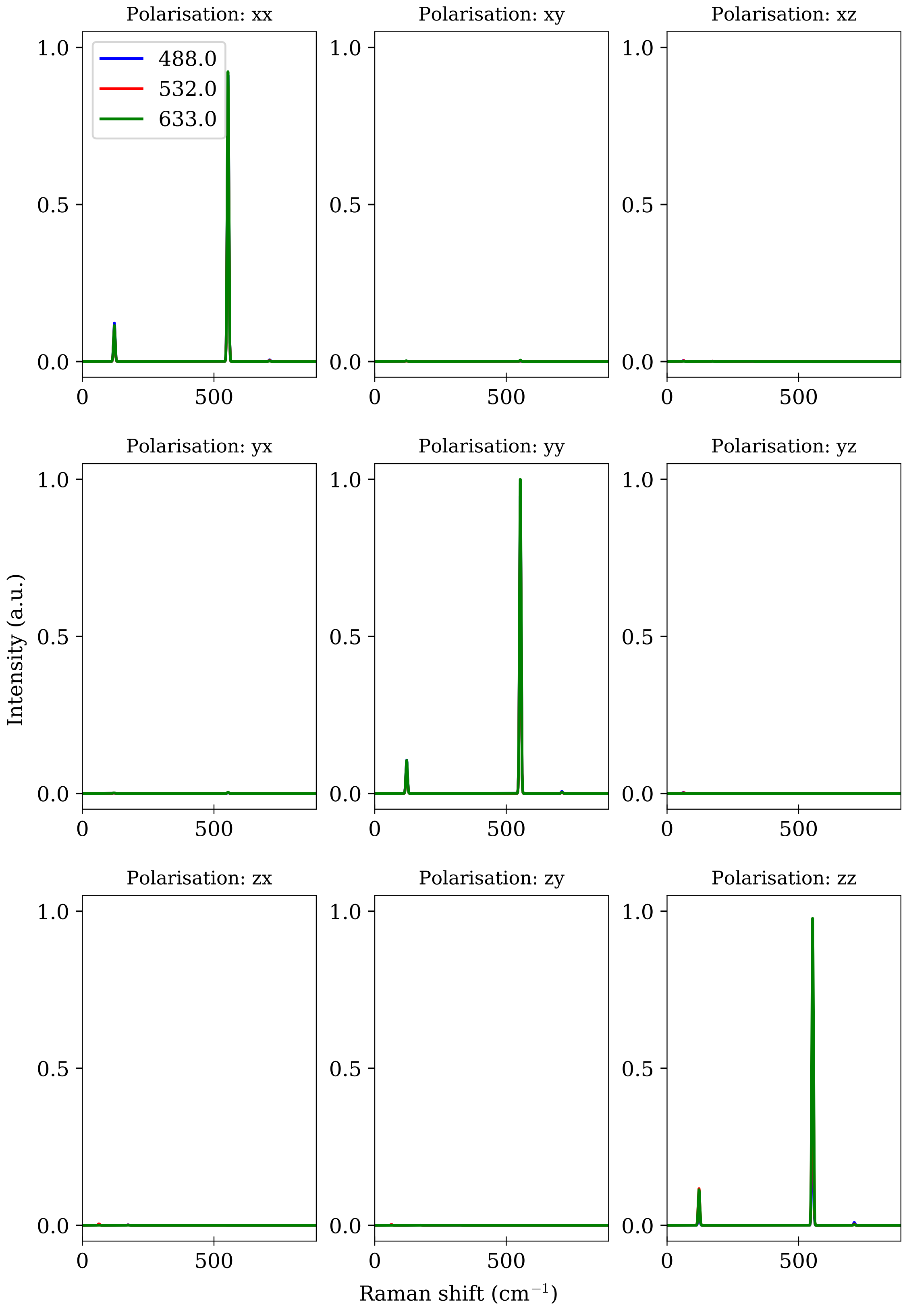

Supplementary Figure 553: Raman spectrum of $Ta_3C_2O_2$ with space group 187 and Wyckoff positions b-g-h-i-0.

Ta_3C_2, 187, b-h-i-0

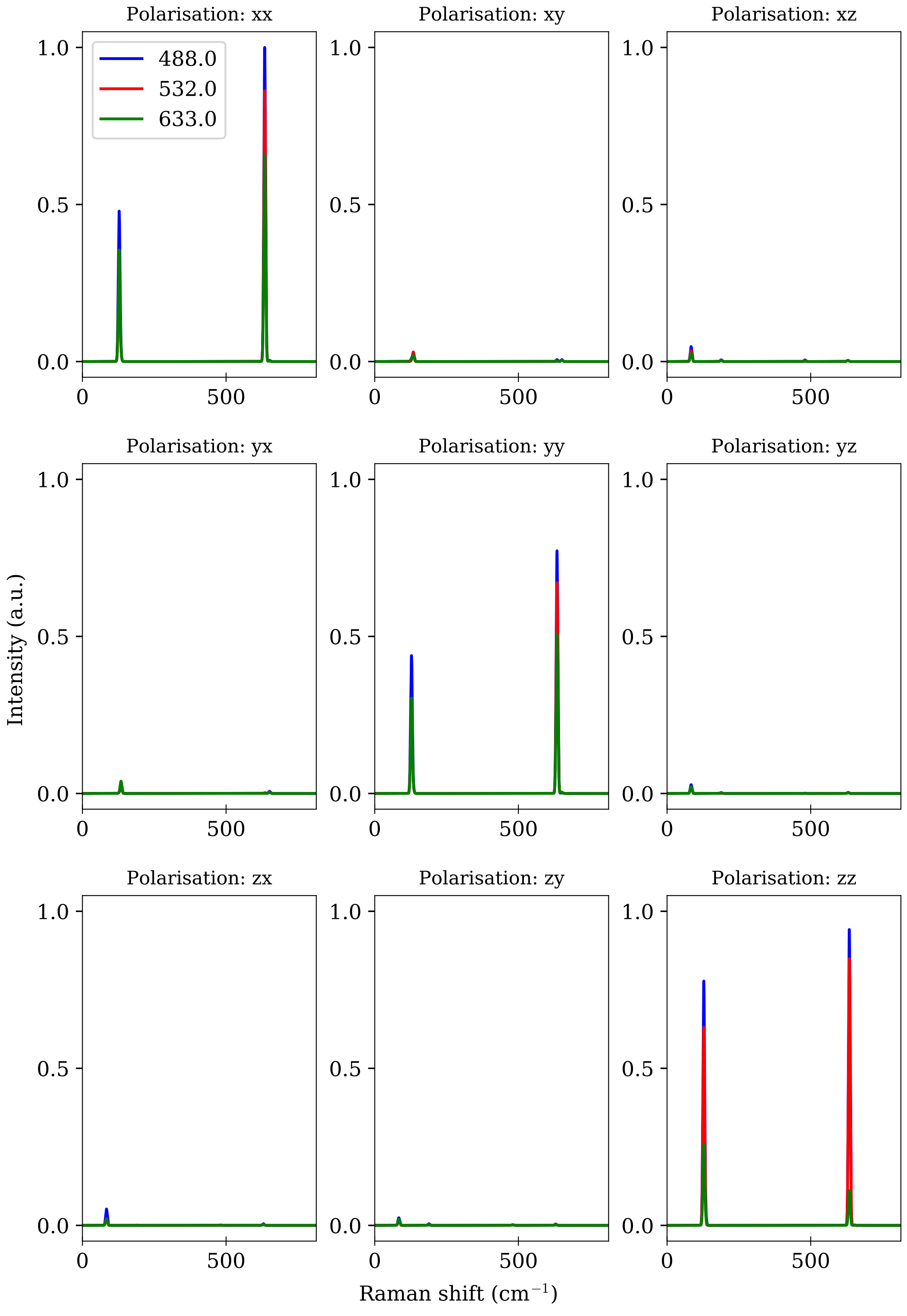

Supplementary Figure 554: Raman spectrum of Ta_3C_2 with space group 187 and Wyckoff positions b-h-i-0.

Ta_3N_2, 187, b-h-i-0

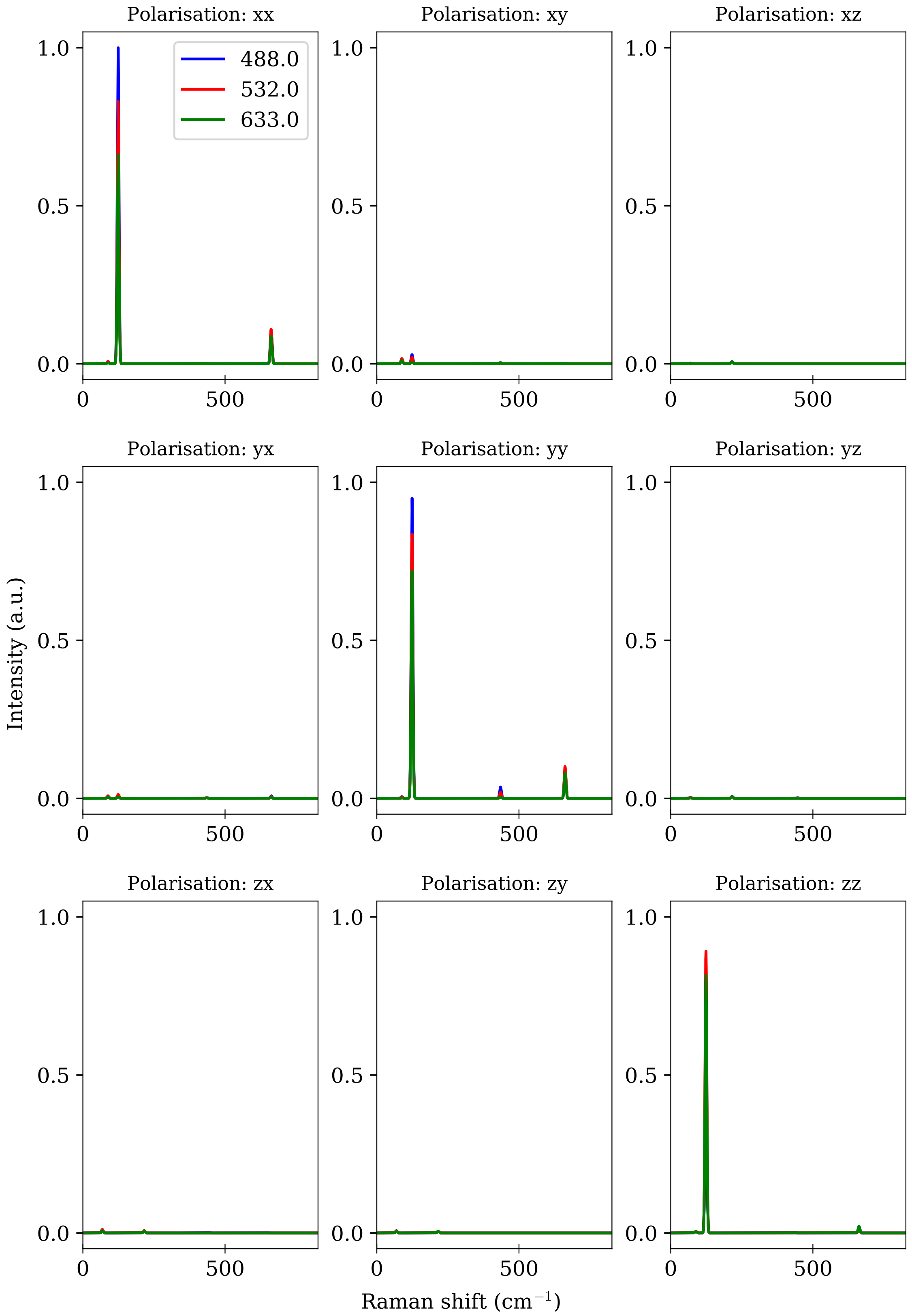

Supplementary Figure 555: Raman spectrum of Ta_3N_2 with space group 187 and Wyckoff positions b-h-i-0.

$TaAs_2$, 164, b-d-0

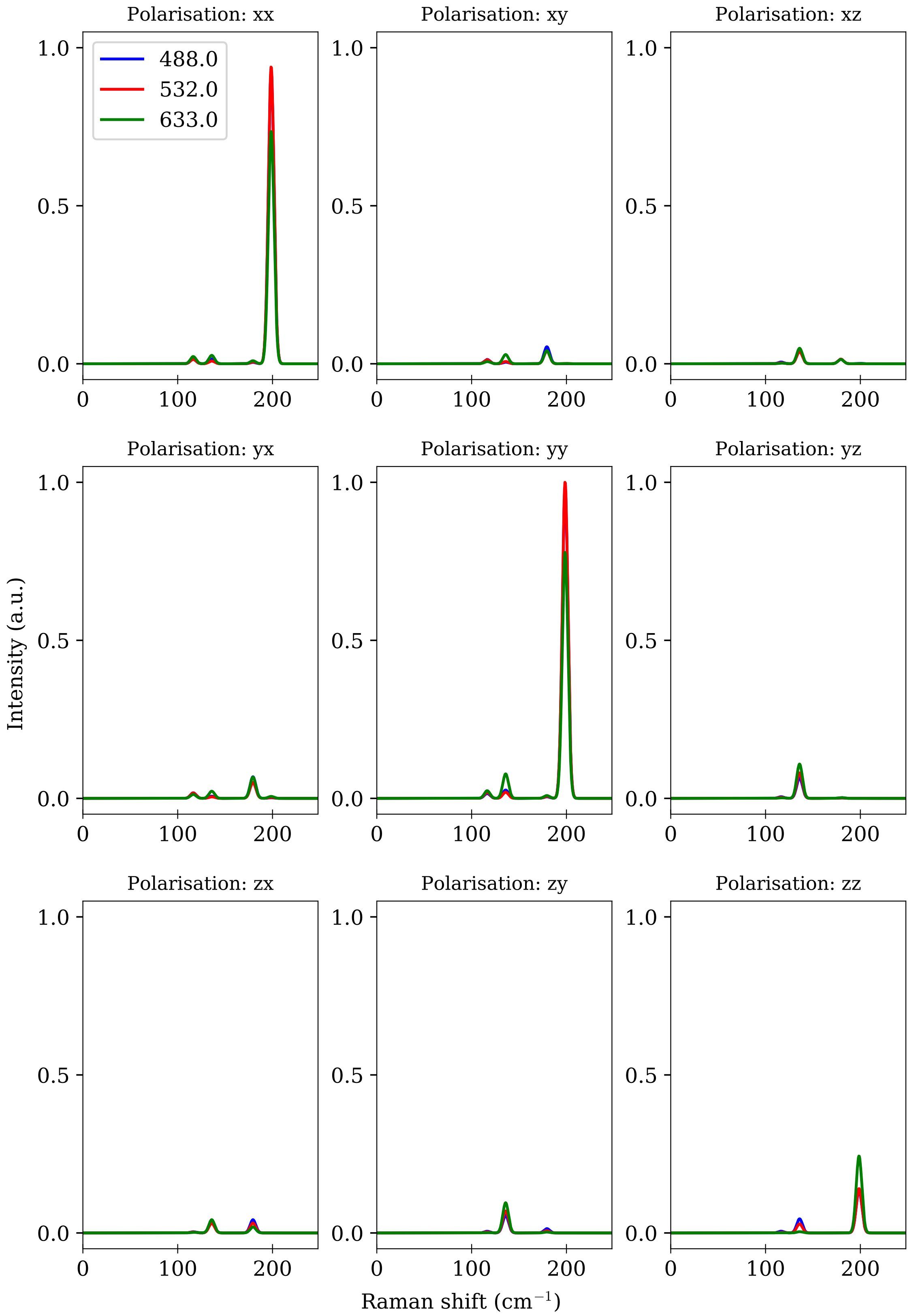

Supplementary Figure 556: Raman spectrum of $TaAs_2$ with space group 164 and Wyckoff positions b-d-0.

TaAsSb, 156, a-b-c-0

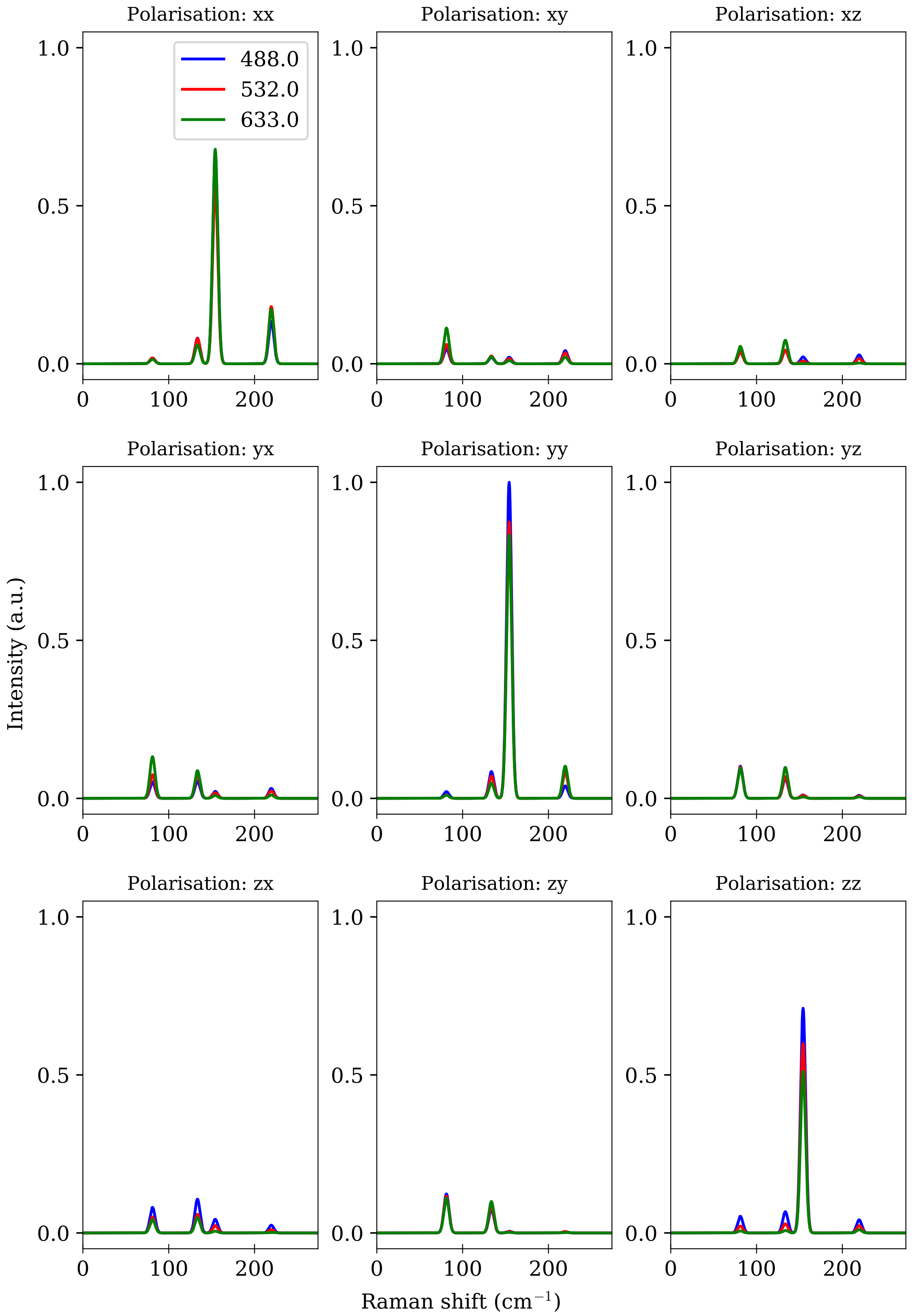

Supplementary Figure 557: Raman spectrum of TaAsSb with space group 156 and Wyckoff positions a-b-c-0.

TaAsSb, 156, a-c-0

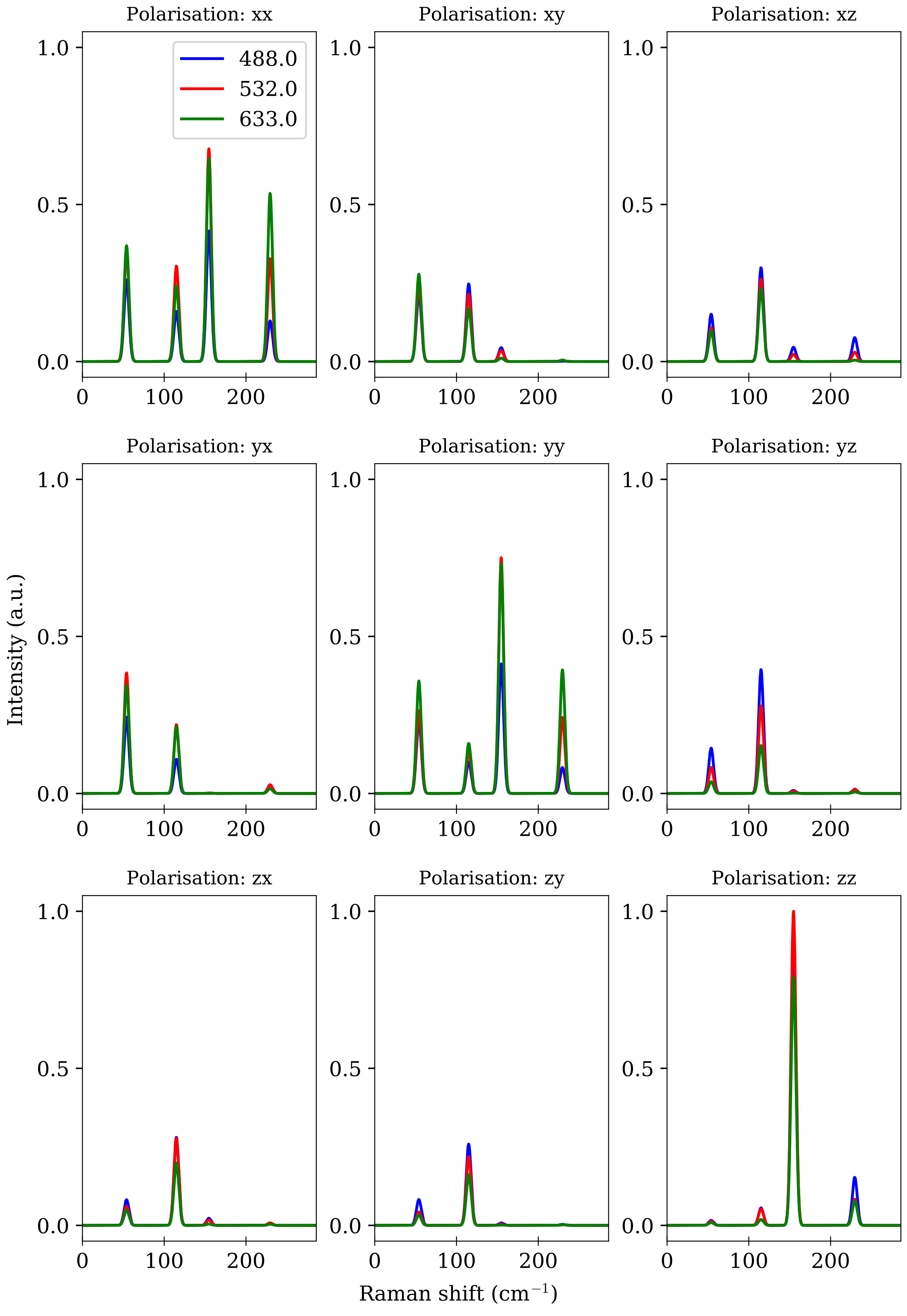

Supplementary Figure 558: Raman spectrum of TaAsSb with space group 156 and Wyckoff positions a-c-0.

$TaCl_2$, 164, b-d-0

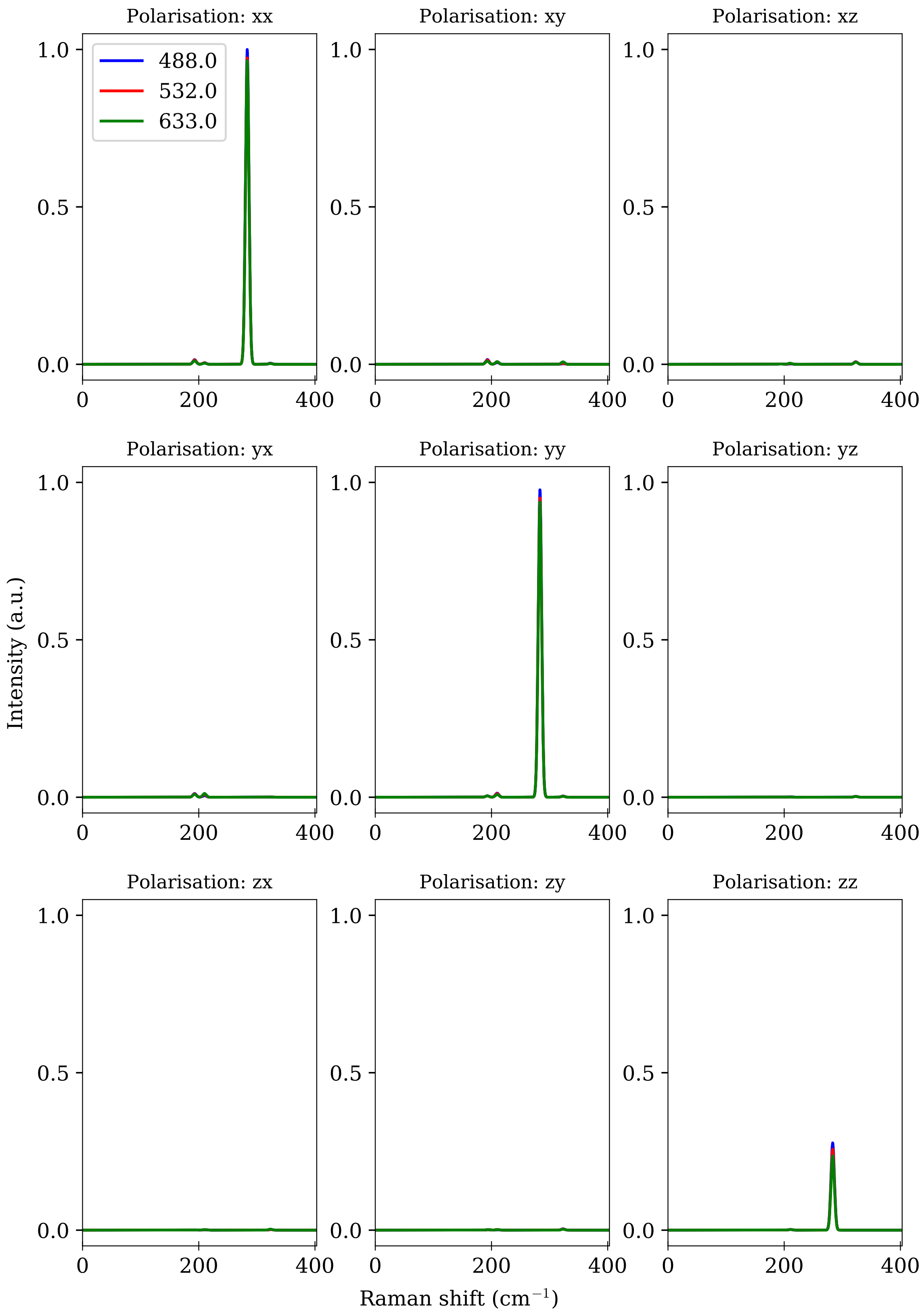

Supplementary Figure 559: Raman spectrum of $TaCl_2$ with space group 164 and Wyckoff positions b-d-0.

TaH_2, 187, a-i-0

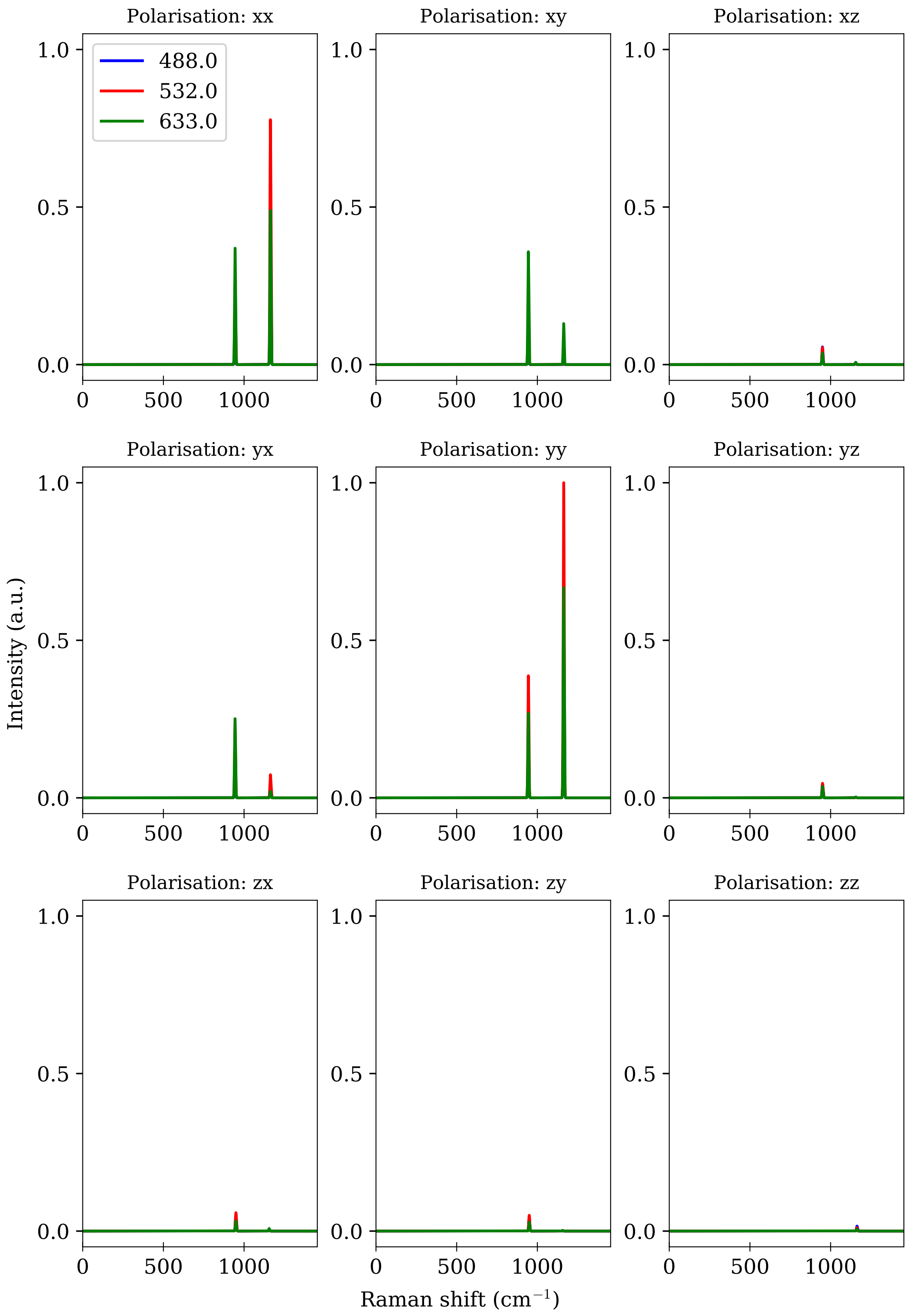

Supplementary Figure 560: Raman spectrum of TaH_2 with space group 187 and Wyckoff positions a-i-0.

TaS_2, 164, b-d-0

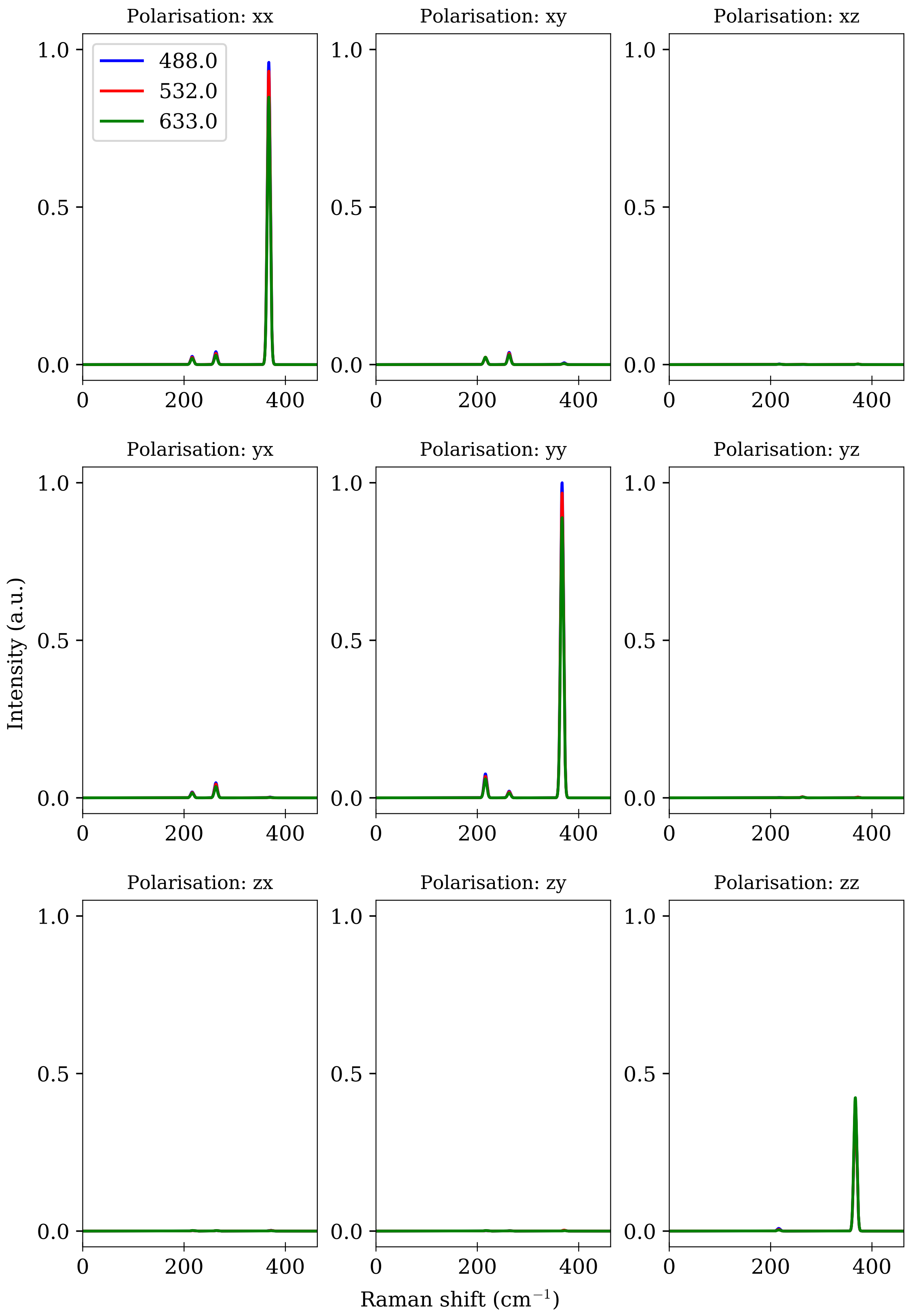

Supplementary Figure 561: Raman spectrum of TaS_2 with space group 164 and Wyckoff positions b-d-0.

$TaSb_2$, 164, b-d-0

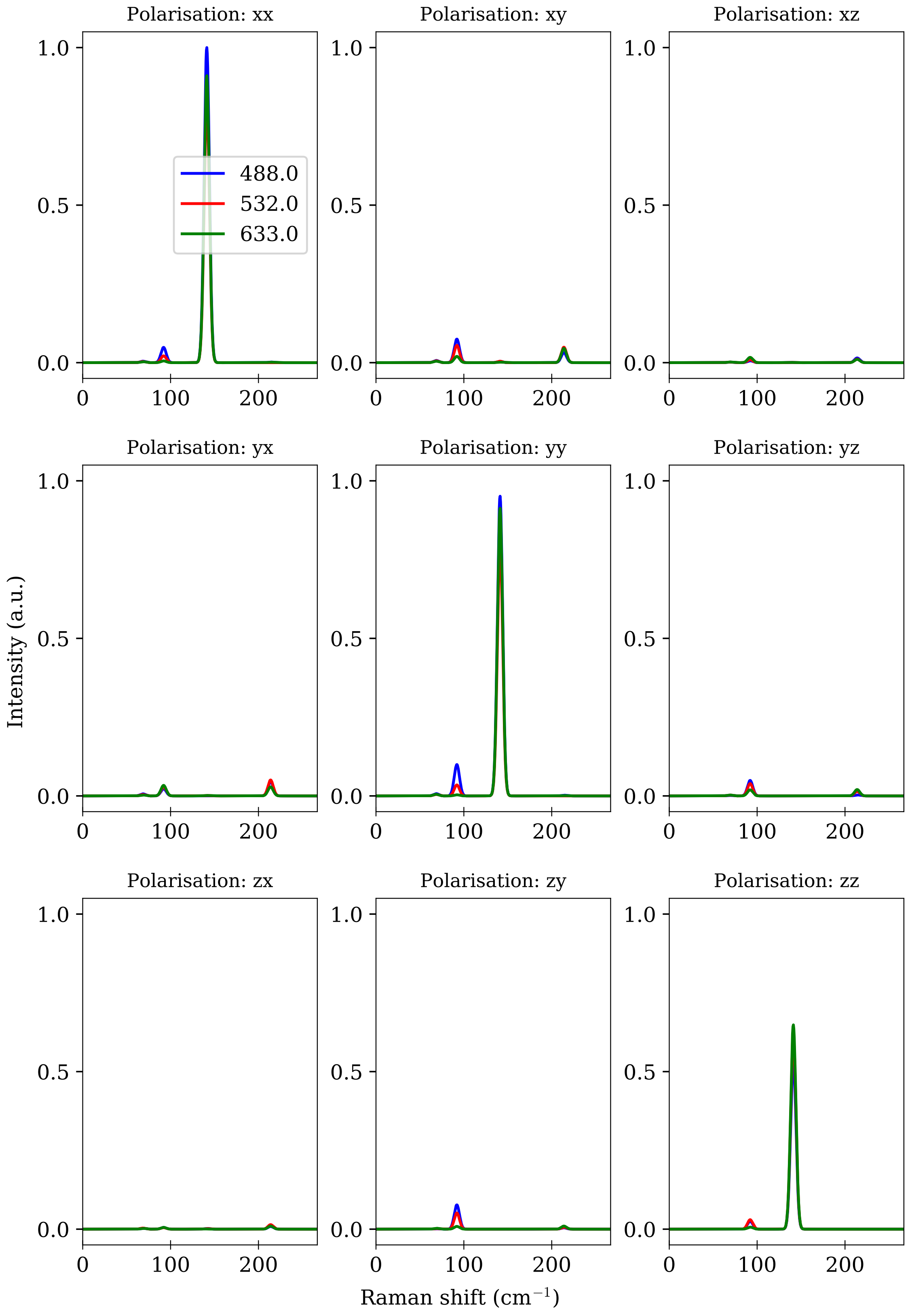

Supplementary Figure 562: Raman spectrum of $TaSb_2$ with space group 164 and Wyckoff positions b-d-0.

$TaSb_2$, 187, a-i-0

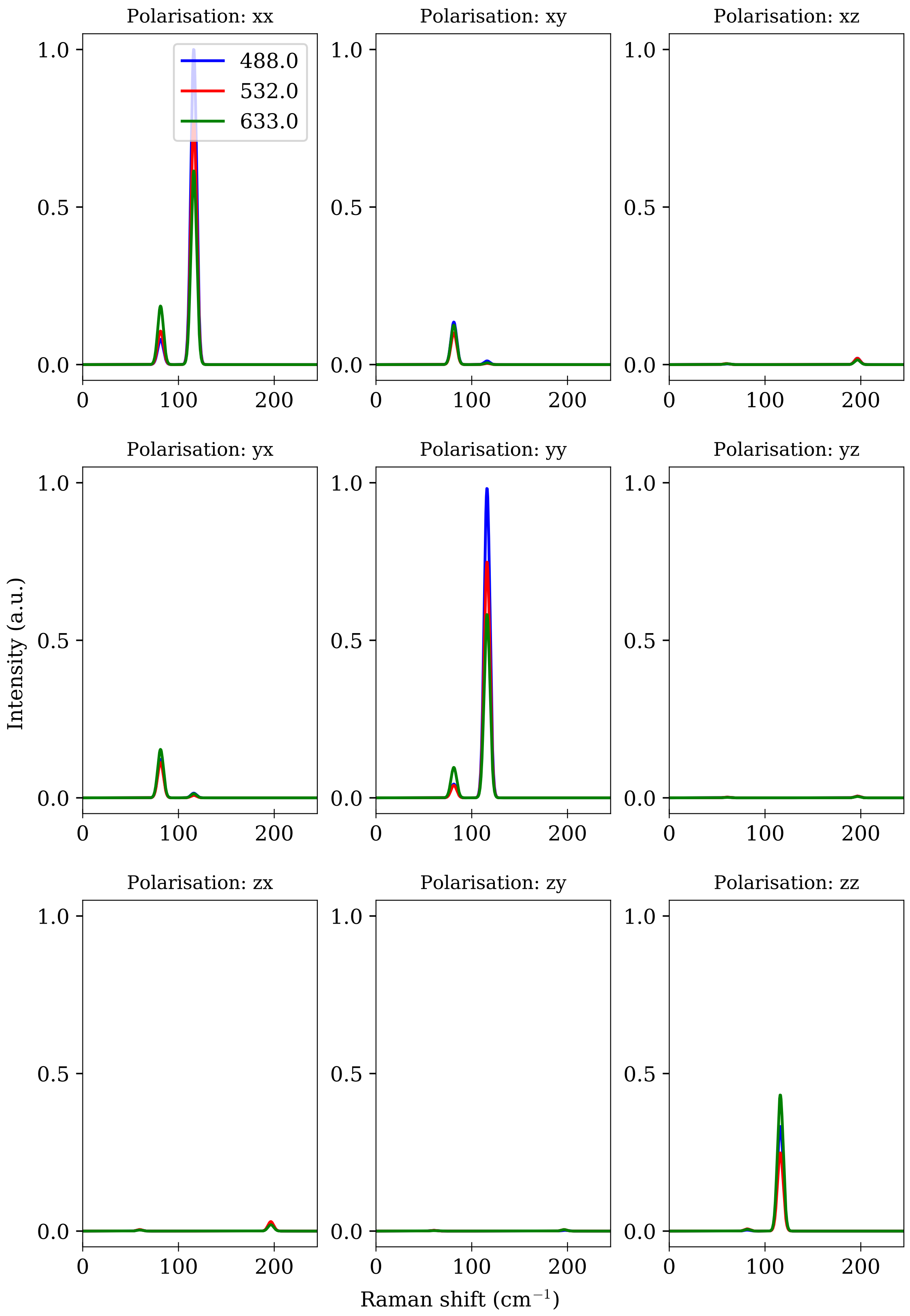

Supplementary Figure 563: Raman spectrum of $TaSb_2$ with space group 187 and Wyckoff positions a-i-0.

$TaSe_2$, 164, b-d-0

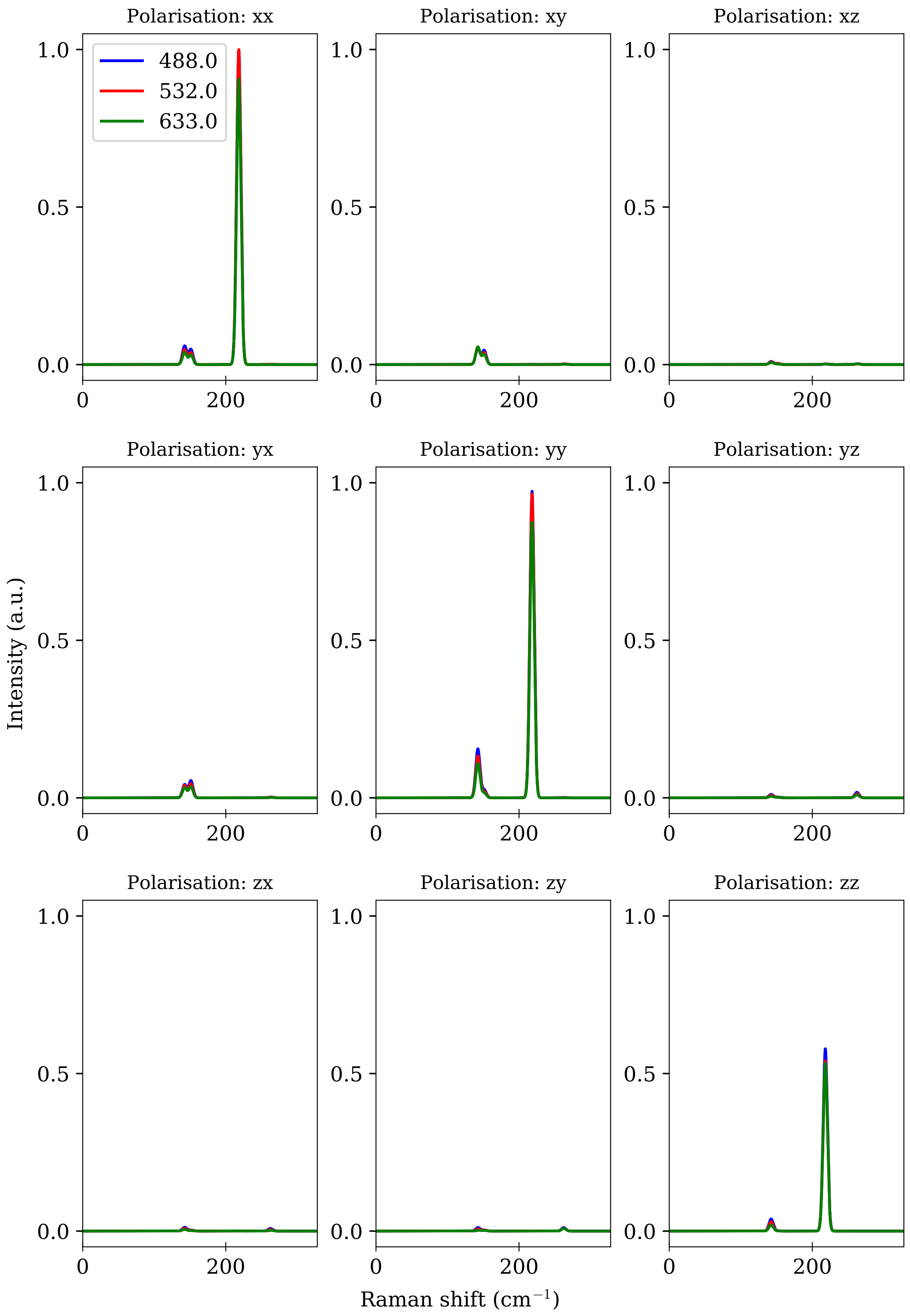

Supplementary Figure 564: Raman spectrum of $TaSe_2$ with space group 164 and Wyckoff positions b-d-0.

TaSeTe, 156, a-b-c-0

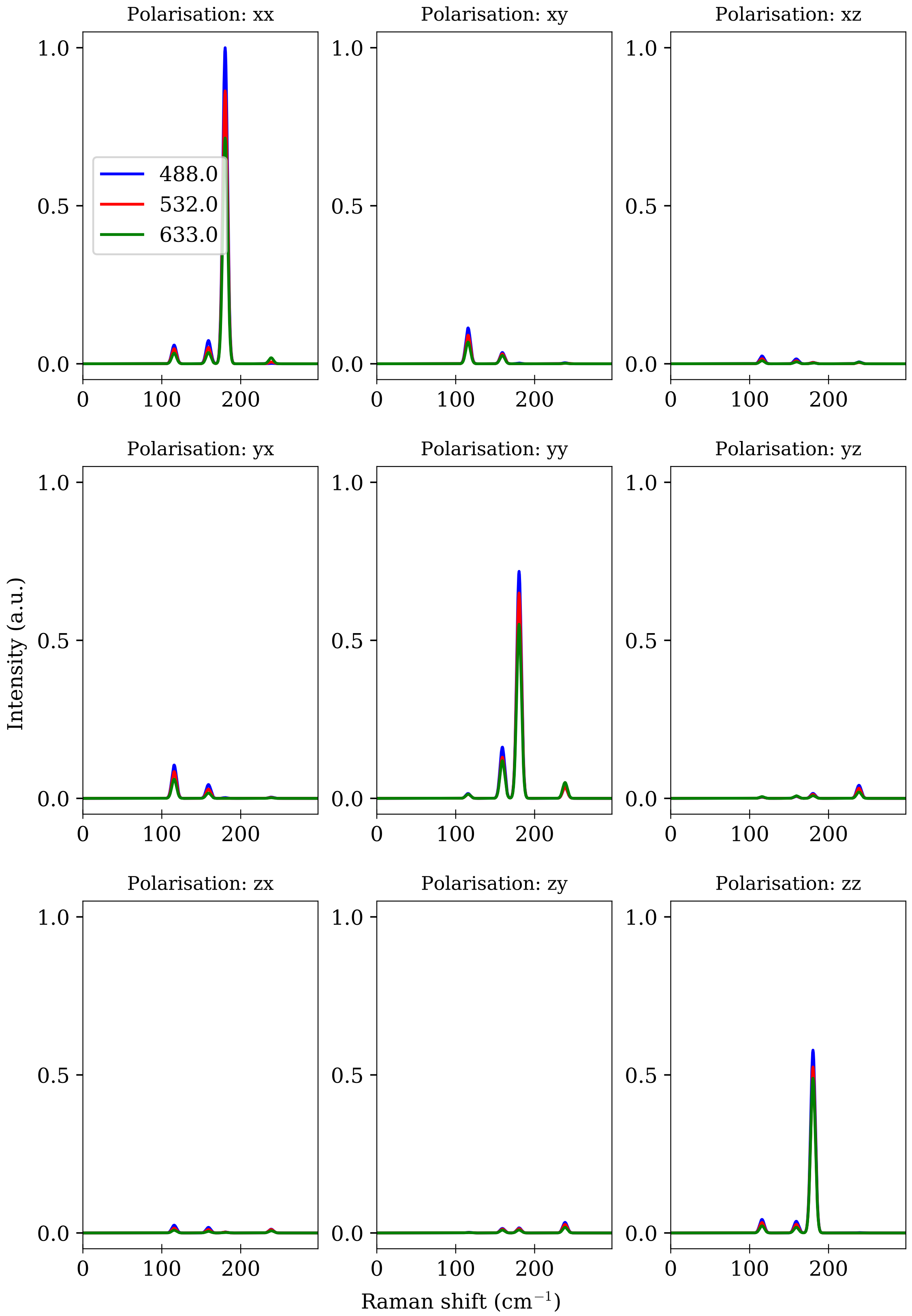

Supplementary Figure 565: Raman spectrum of TaSeTe with space group 156 and Wyckoff positions a-b-c-0.

TaSSe, 156, a-b-c-0

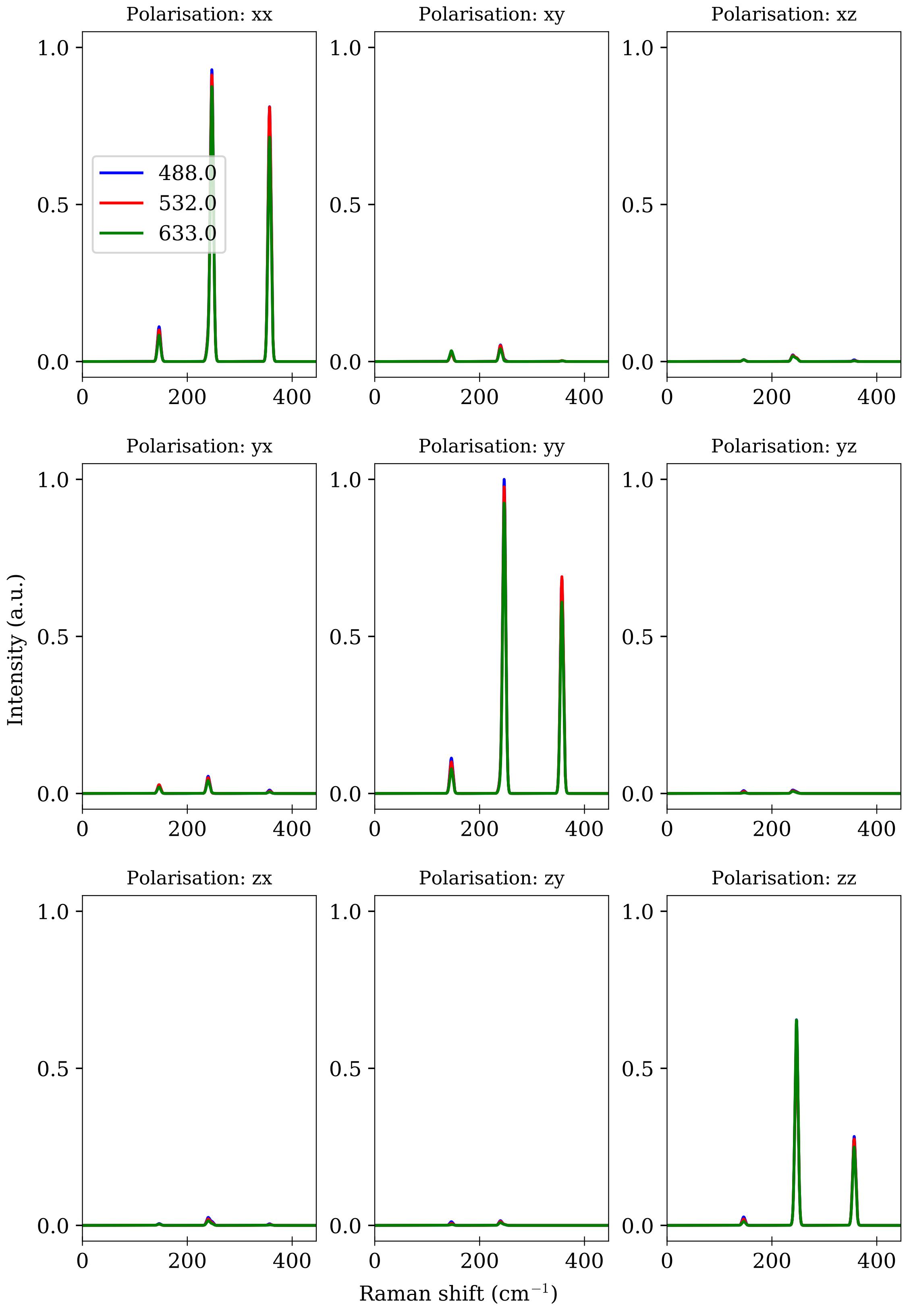

Supplementary Figure 566: Raman spectrum of TaSSe with space group 156 and Wyckoff positions a-b-c-0.

TaSTe, 156, a-b-c-0

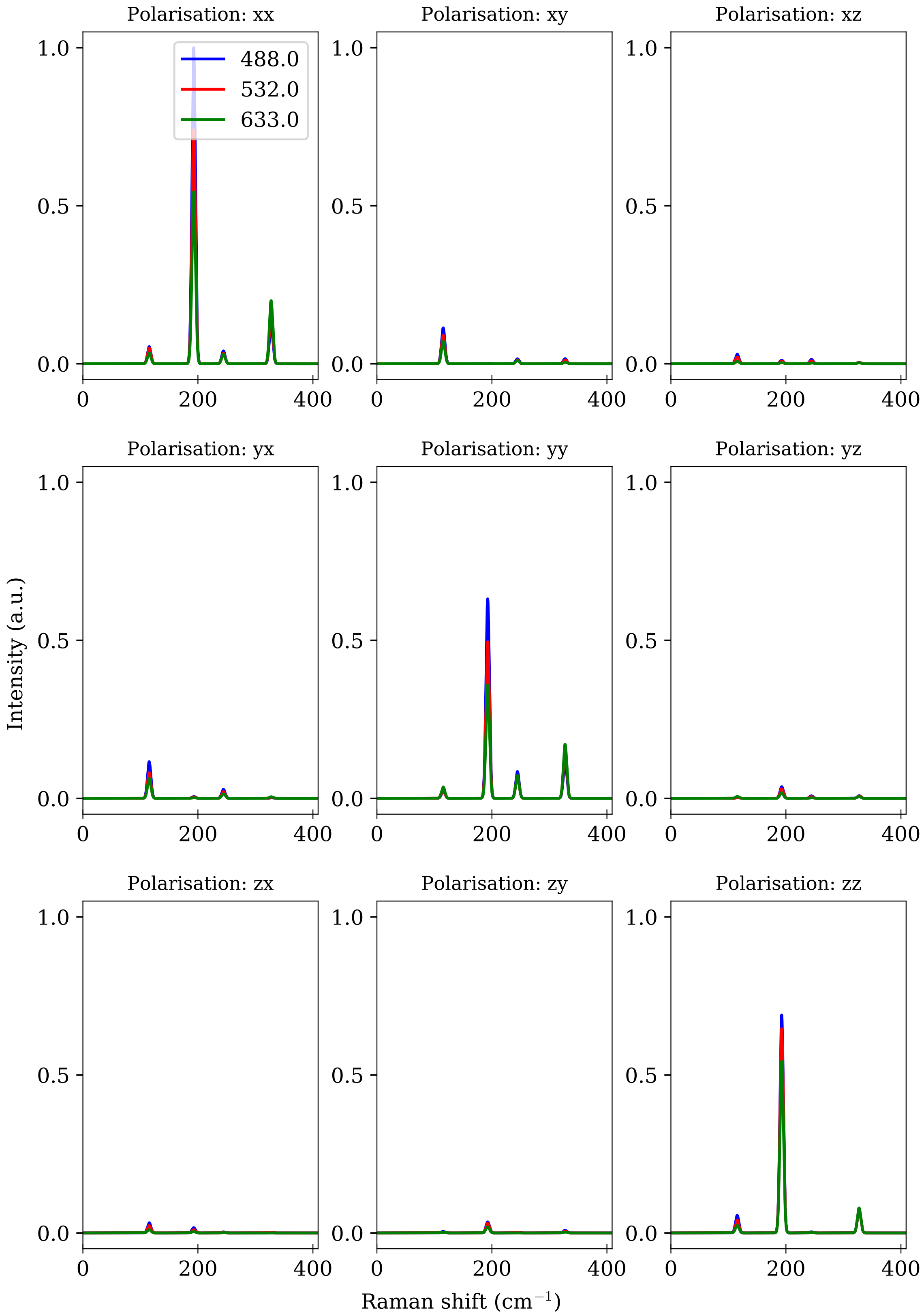

Supplementary Figure 567: Raman spectrum of TaSTe with space group 156 and Wyckoff positions a-b-c-0.

$TaTe_2$, 164, b-d-0

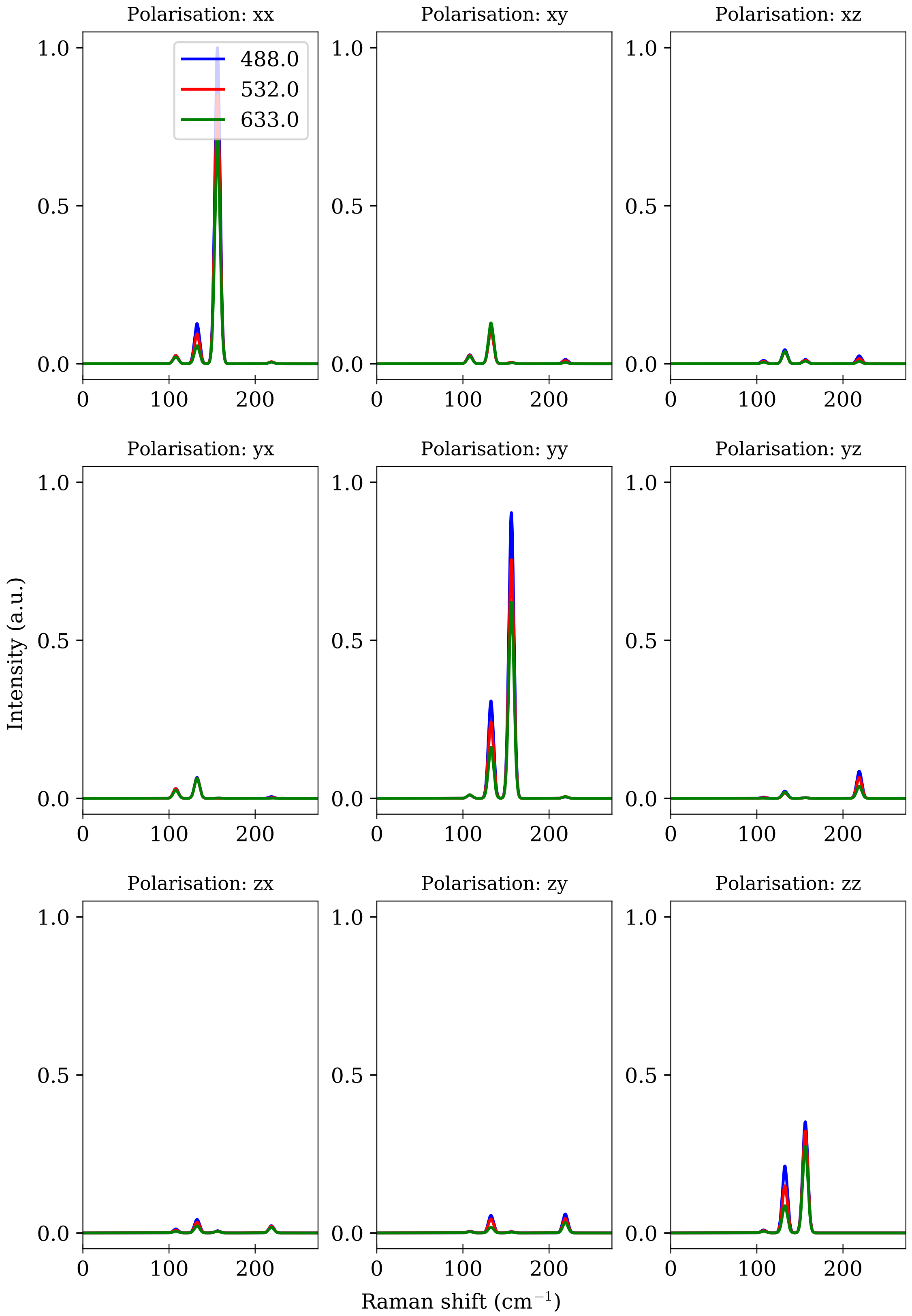

Supplementary Figure 568: Raman spectrum of $TaTe_2$ with space group 164 and Wyckoff positions b-d-0.

Ti_2Br_2, 129, b-c-0

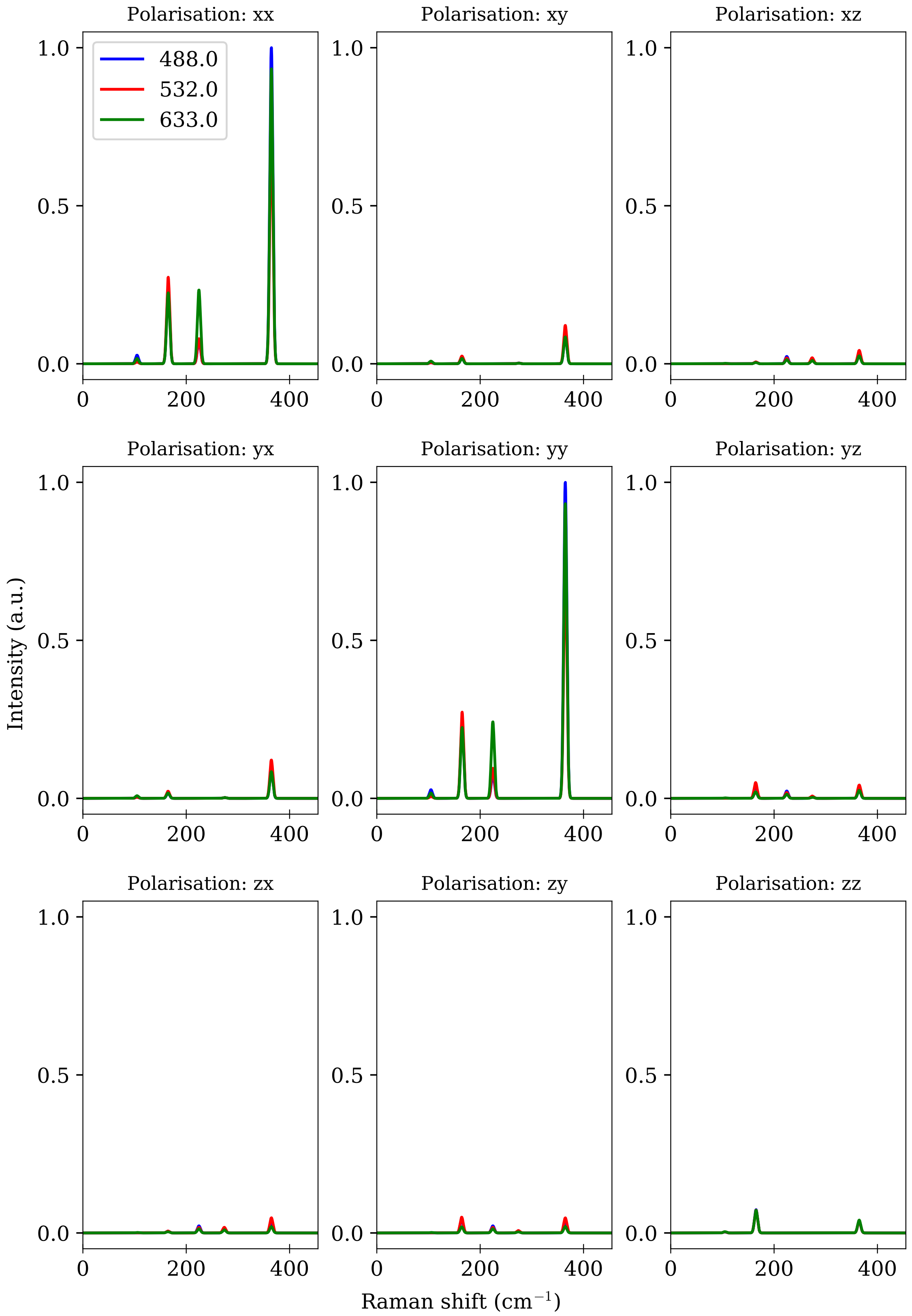

Supplementary Figure 569: Raman spectrum of Ti_2Br_2 with space group 129 and Wyckoff positions b-c-0.

Ti_2CF_2, 164, b-d-0

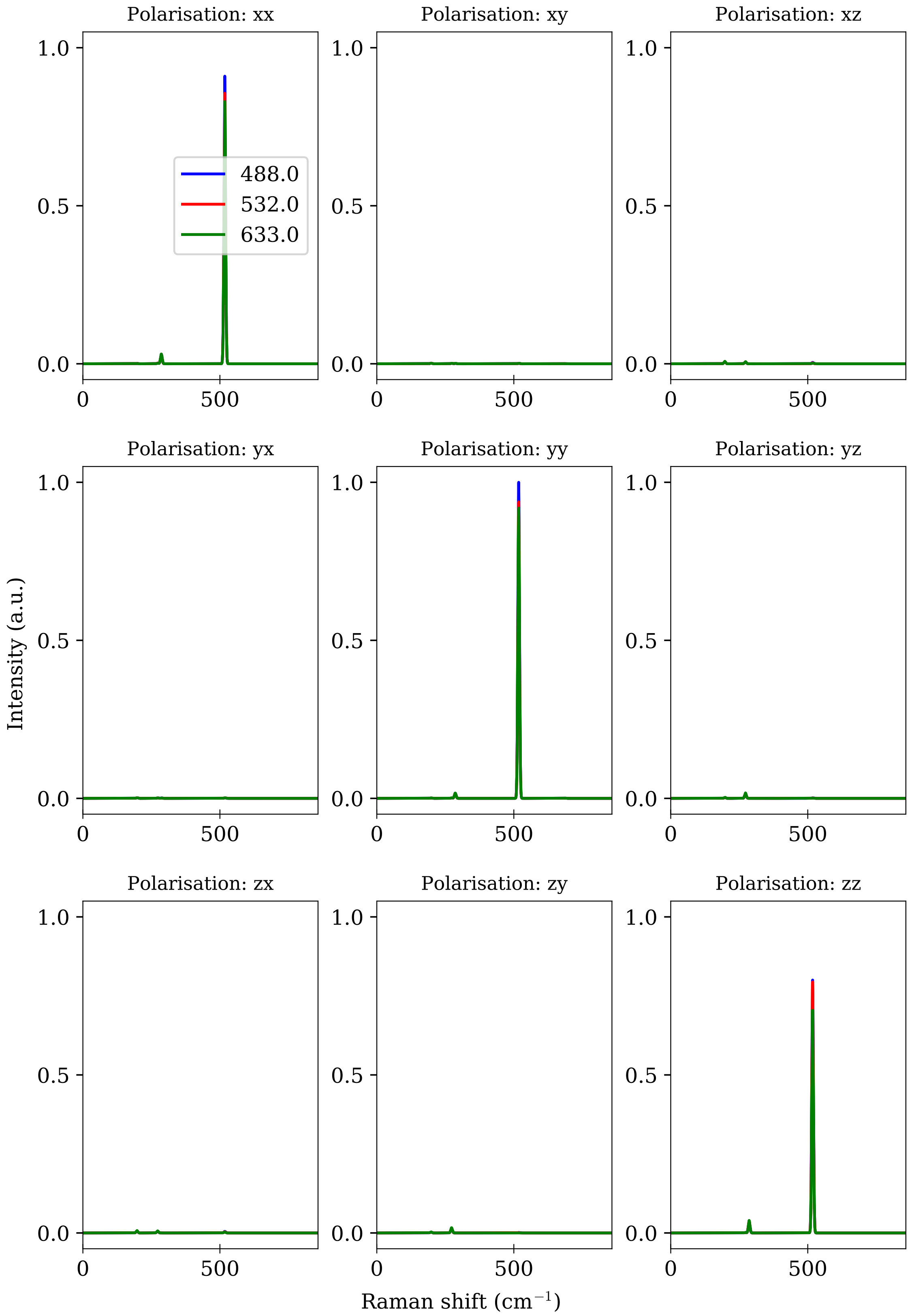

Supplementary Figure 570: Raman spectrum of Ti_2CF_2 with space group 164 and Wyckoff positions b-d-0.

$Ti_2CH_2O_2$, 164, b-d-0

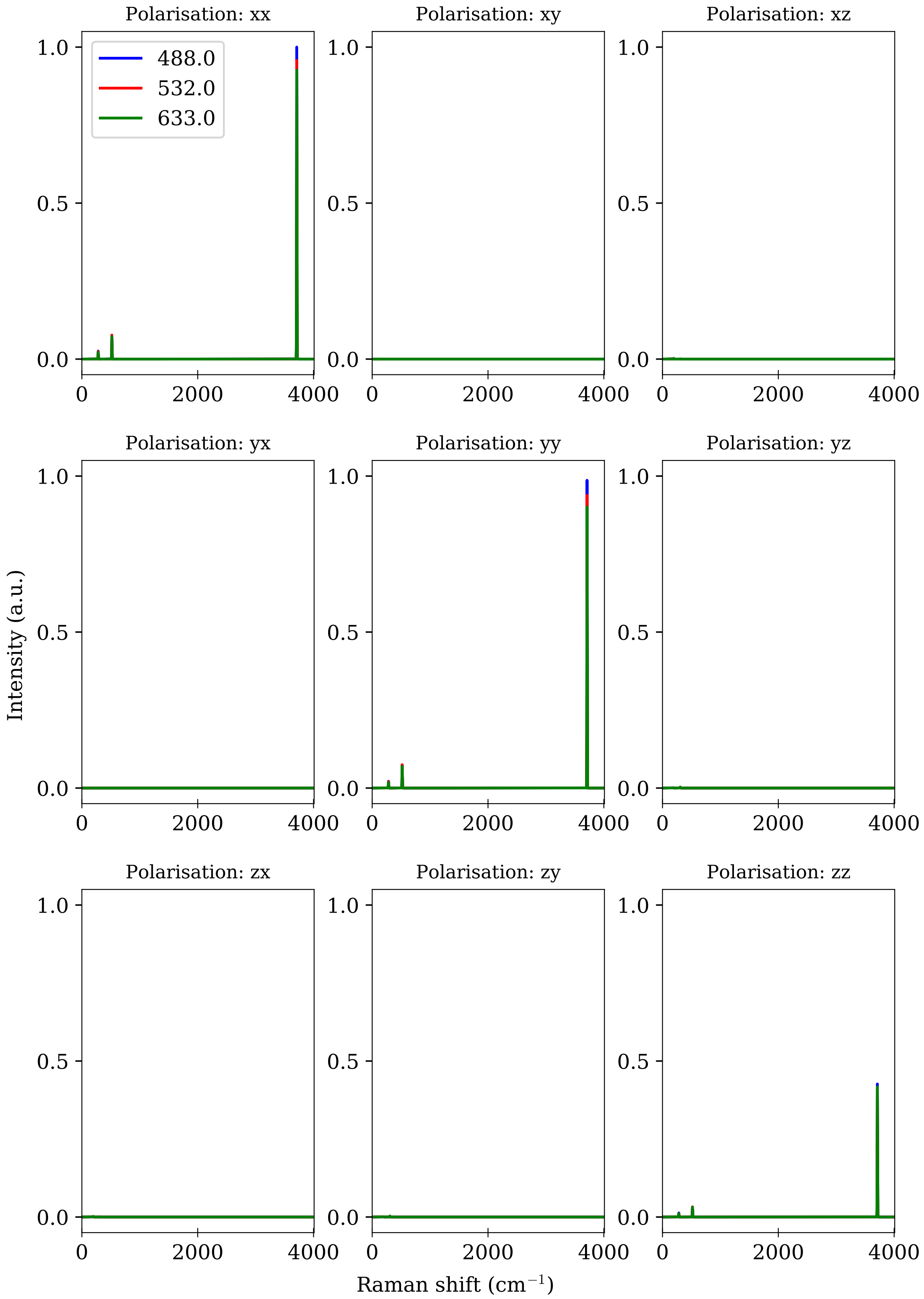

Supplementary Figure 571: Raman spectrum of $Ti_2CH_2O_2$ with space group 164 and Wyckoff positions b-d-0.

Ti_2Cl_4, 11, e-0

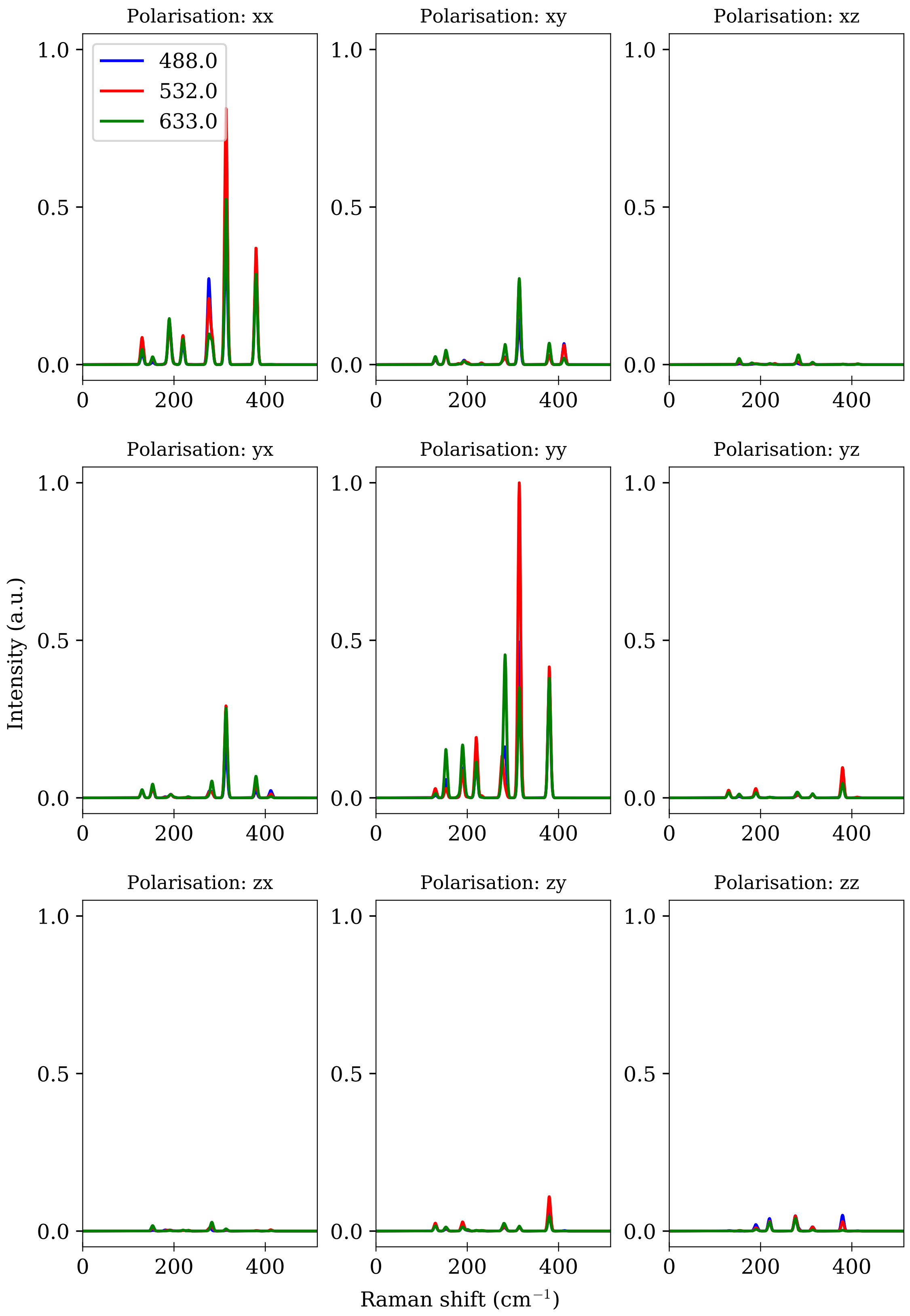

Supplementary Figure 572: Raman spectrum of Ti_2Cl_4 with space group 11 and Wyckoff positions e-0.

Ti_2CO_2, 164, b-d-0

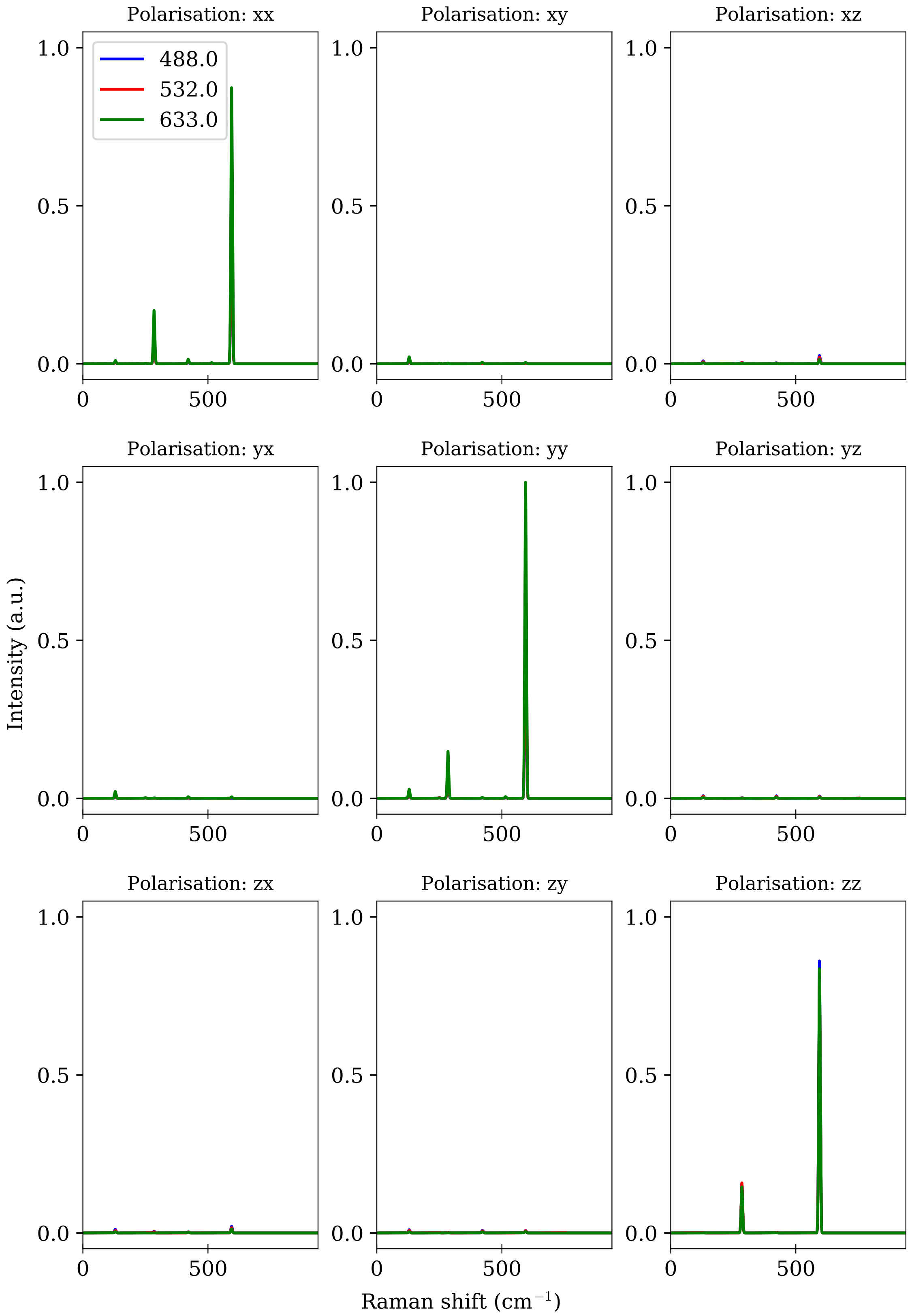

Supplementary Figure 573: Raman spectrum of Ti_2CO_2 with space group 164 and Wyckoff positions b-d-0.

Ti_2F_2N, 164, b-d-0

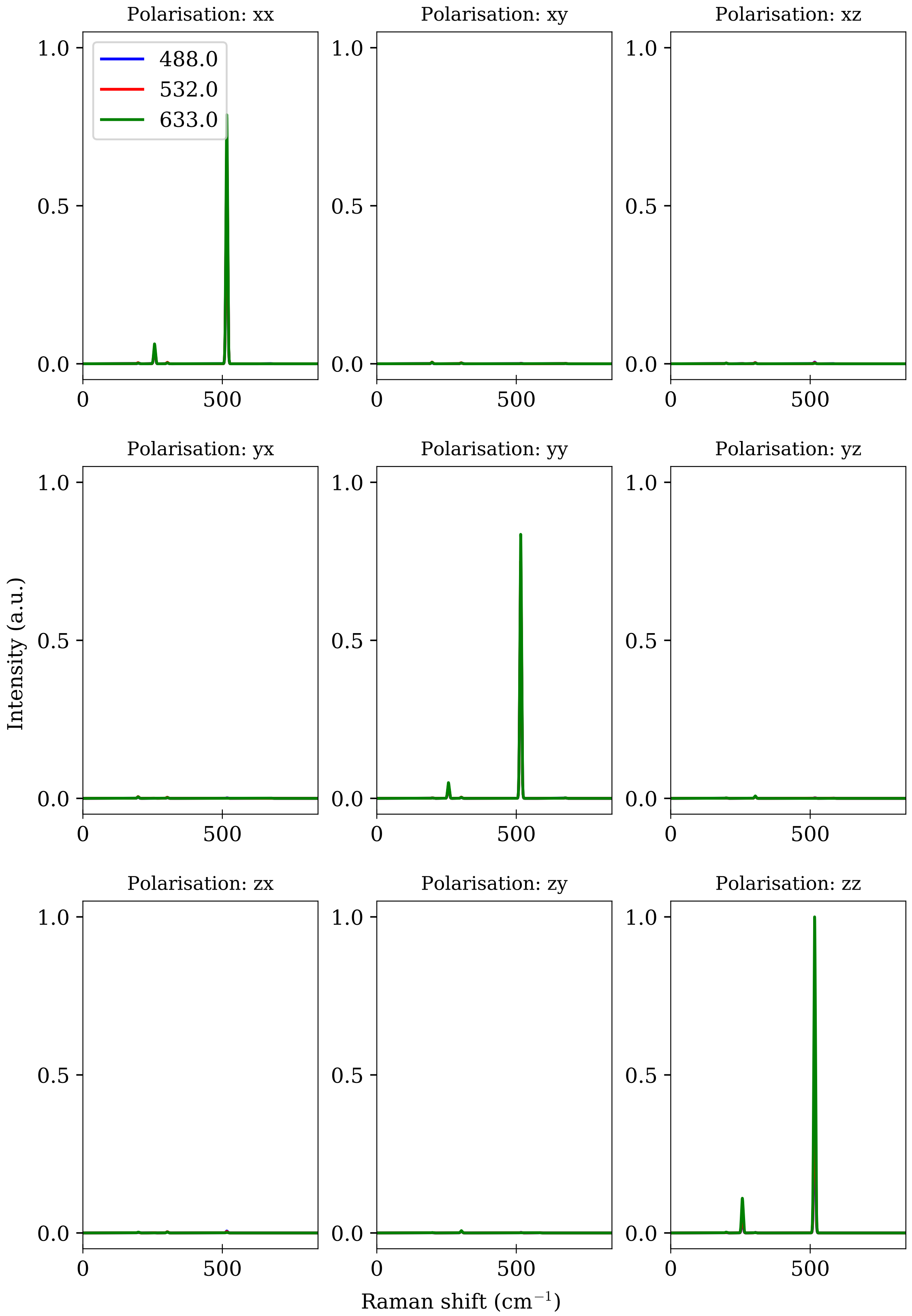

Supplementary Figure 574: Raman spectrum of Ti_2F_2N with space group 164 and Wyckoff positions b-d-0.

Ti_2F_2, 2, i-0

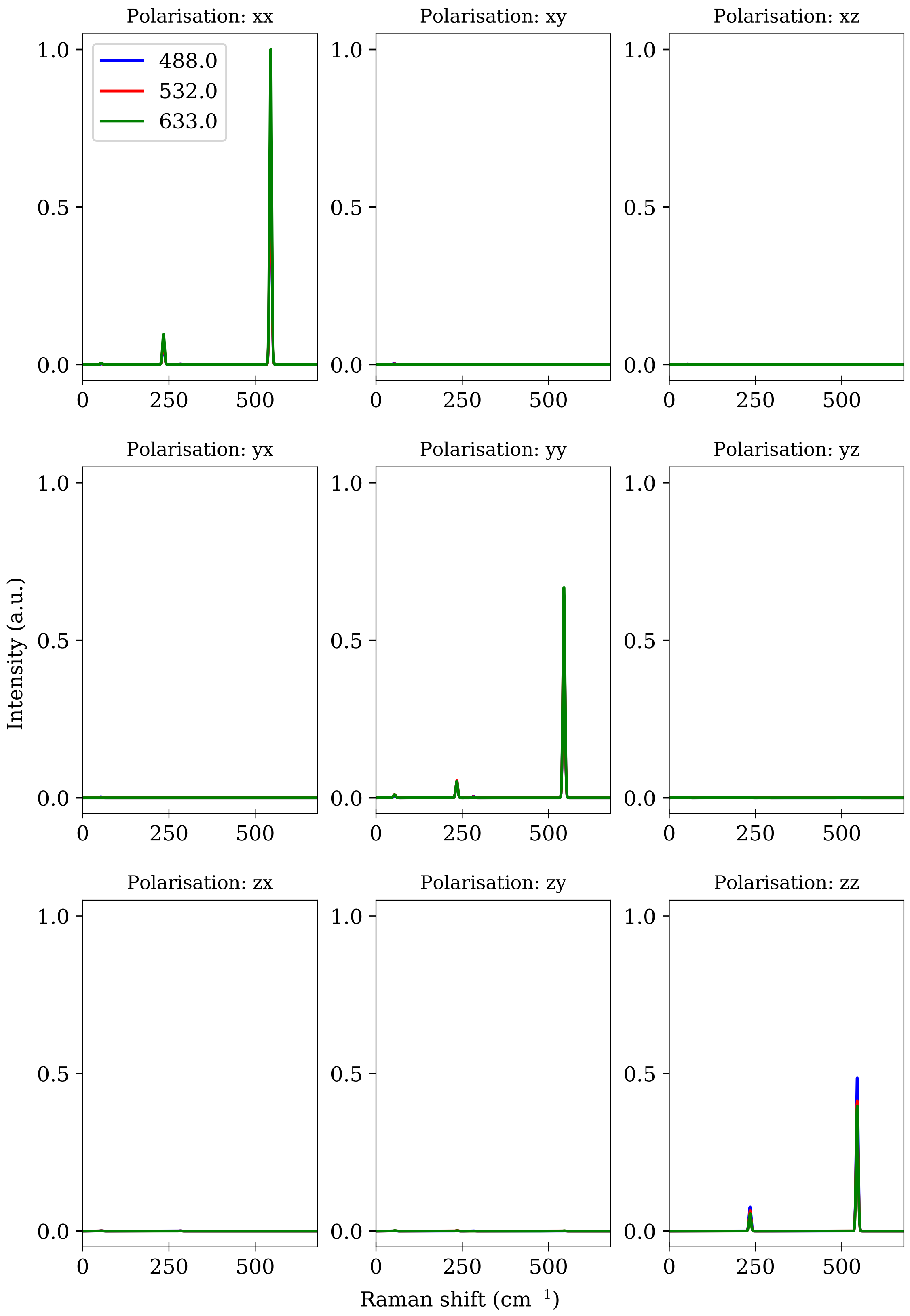

Supplementary Figure 575: Raman spectrum of Ti_2F_2 with space group 2 and Wyckoff positions i-0.

$Ti_2H_2NO_2$, 164, b-d-0

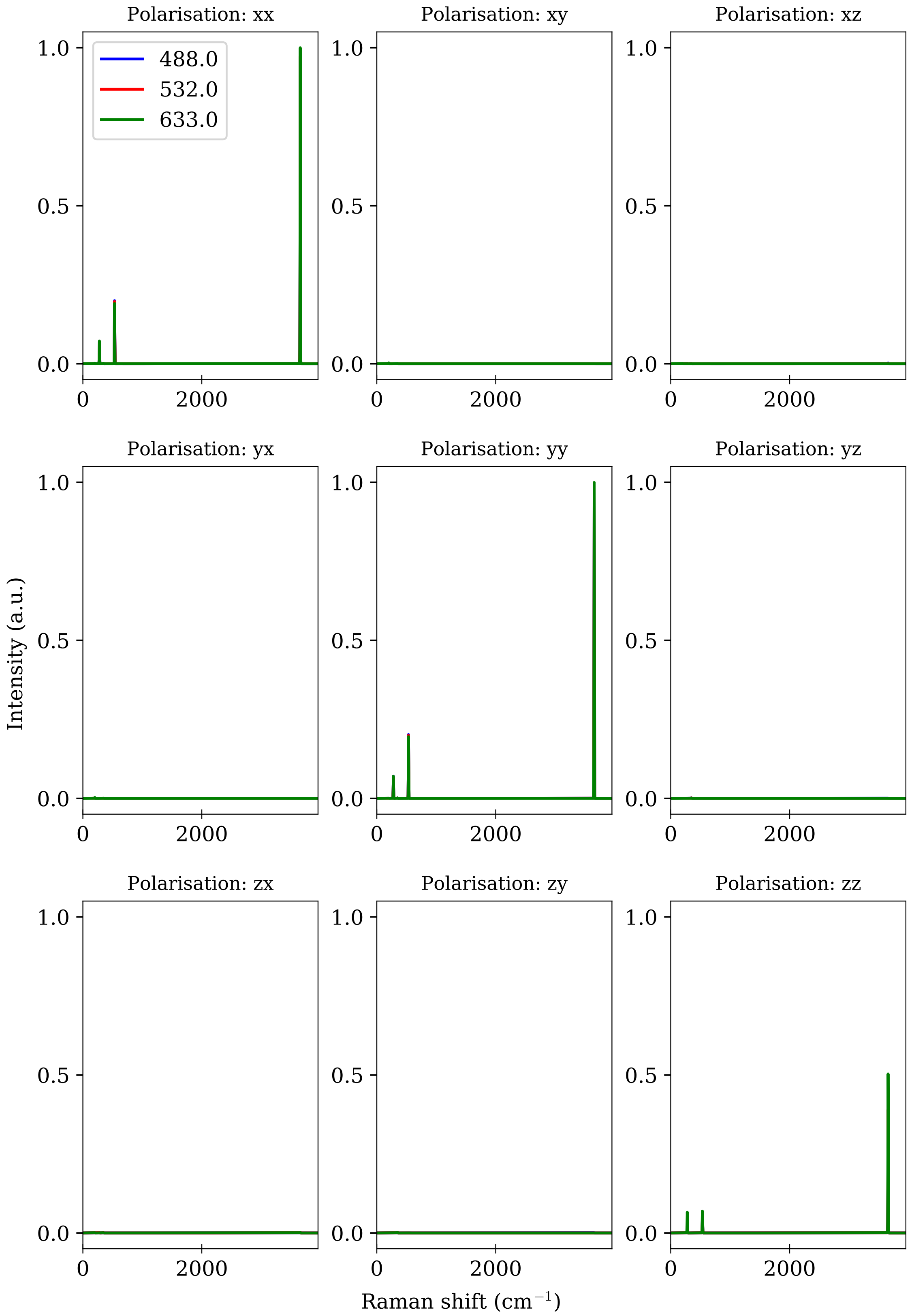

Supplementary Figure 576: Raman spectrum of $Ti_2H_2NO_2$ with space group 164 and Wyckoff positions b-d-0.

Ti_2I_2, 129, b-c-0

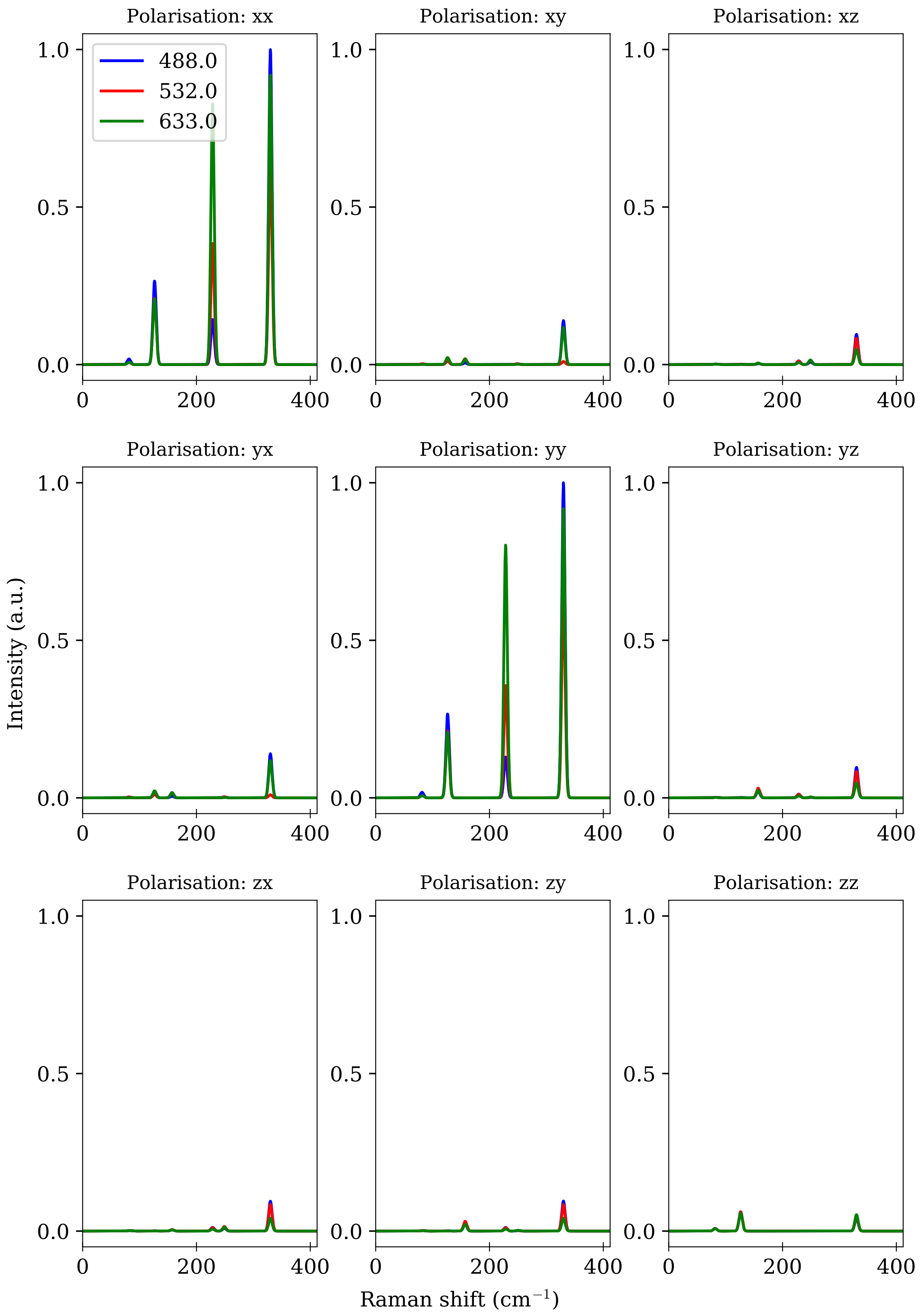

Supplementary Figure 577: Raman spectrum of Ti_2I_2 with space group 129 and Wyckoff positions b-c-0.

Ti_2O_2, 10, b-d-m-0

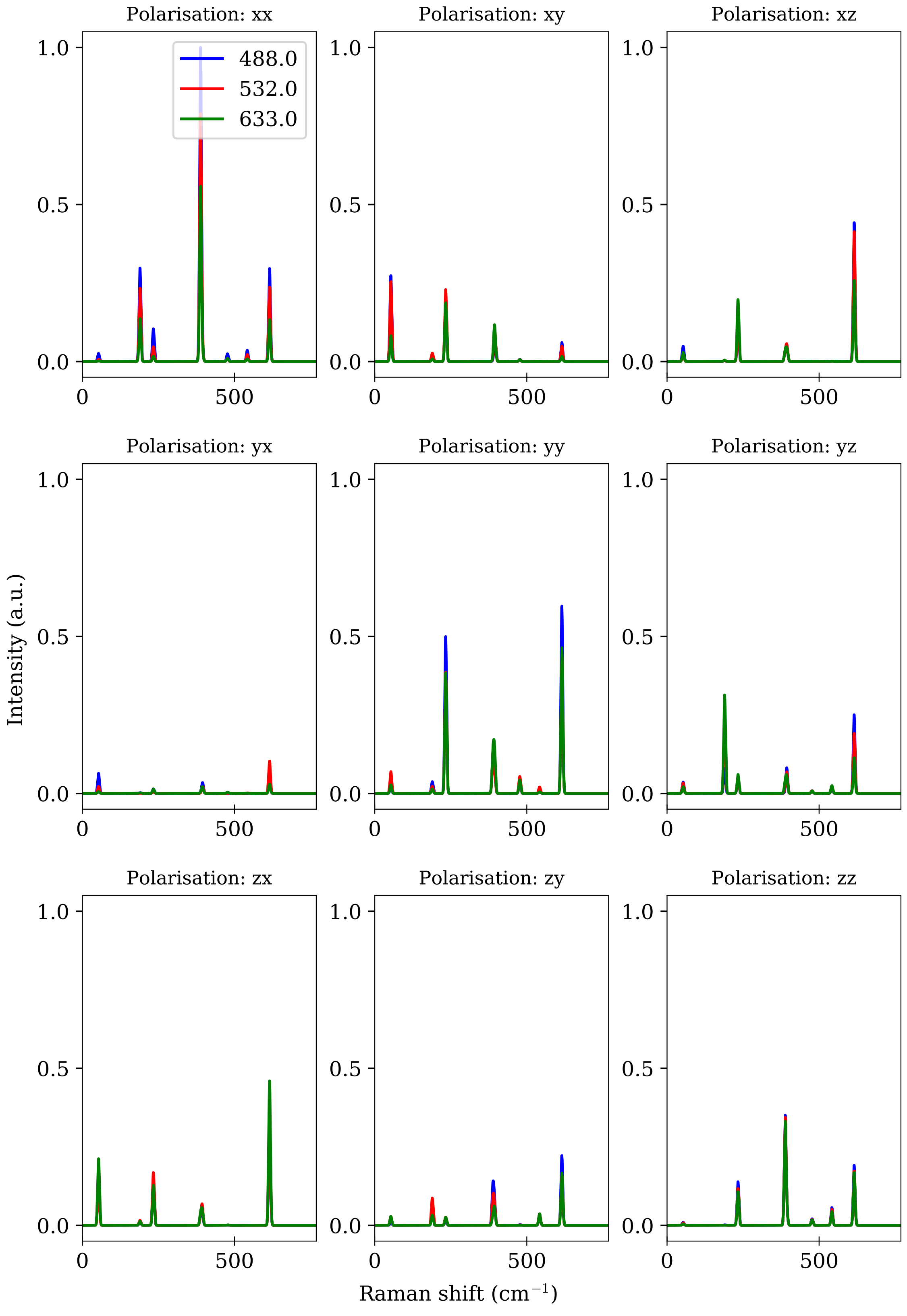

Supplementary Figure 578: Raman spectrum of Ti_2O_2 with space group 10 and Wyckoff positions b-d-m-0.

Ti_2O_2, 129, b-c-0

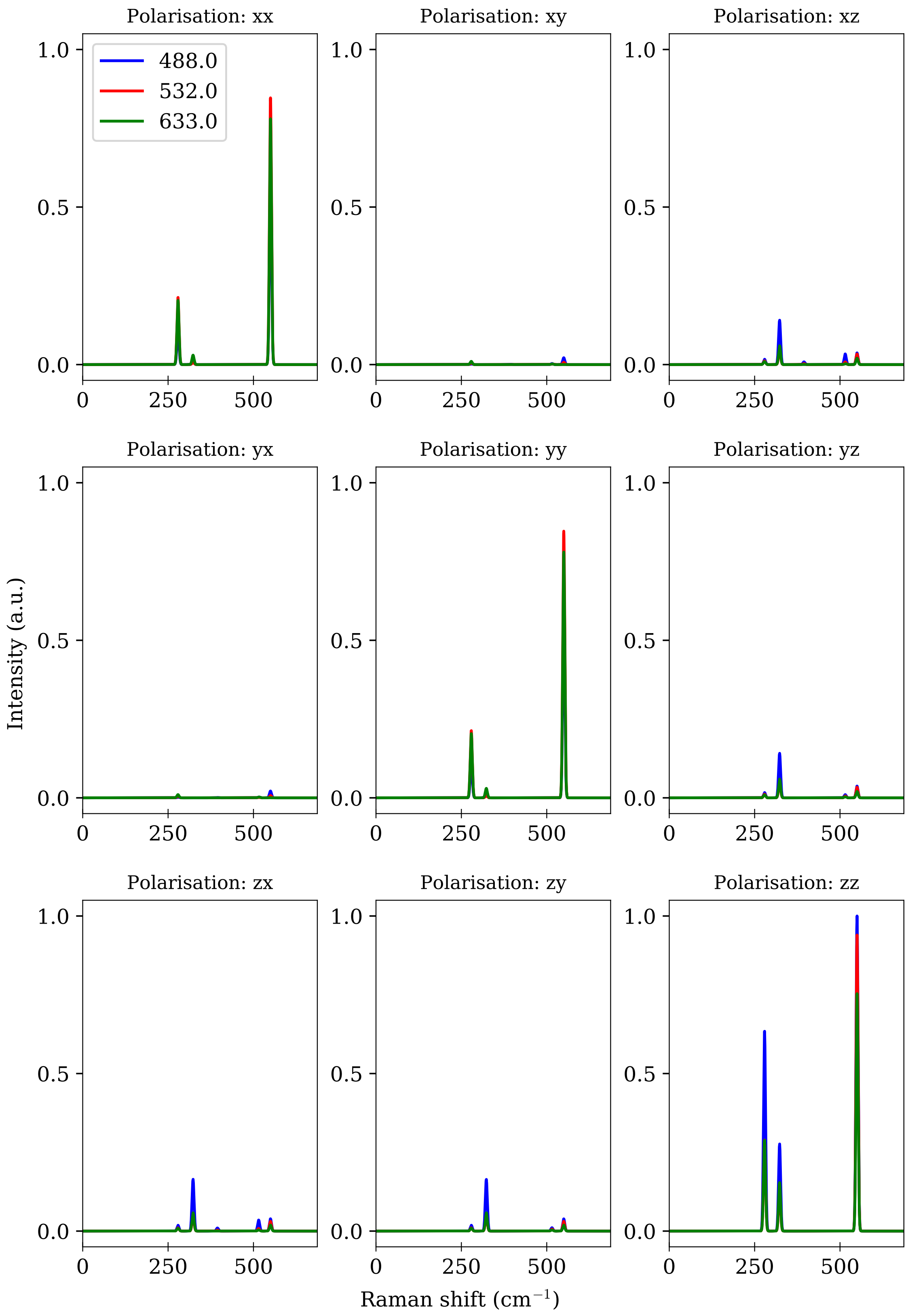

Supplementary Figure 579: Raman spectrum of Ti_2O_2 with space group 129 and Wyckoff positions b-c-0.

Ti_2O_4, 11, e-0

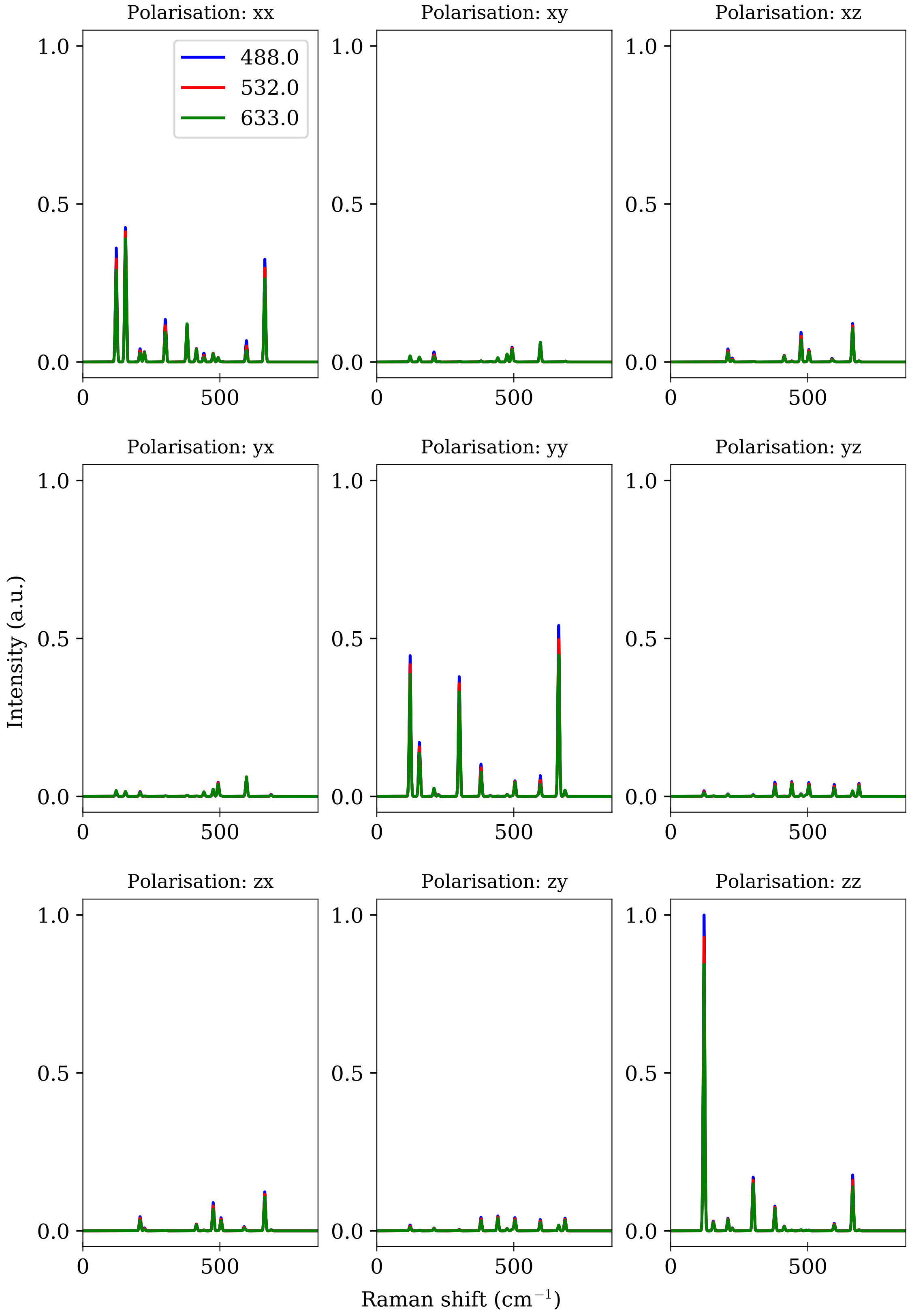

Supplementary Figure 580: Raman spectrum of Ti_2O_4 with space group 11 and Wyckoff positions e-0.

Ti_2O_6, 59, a-b-e-0

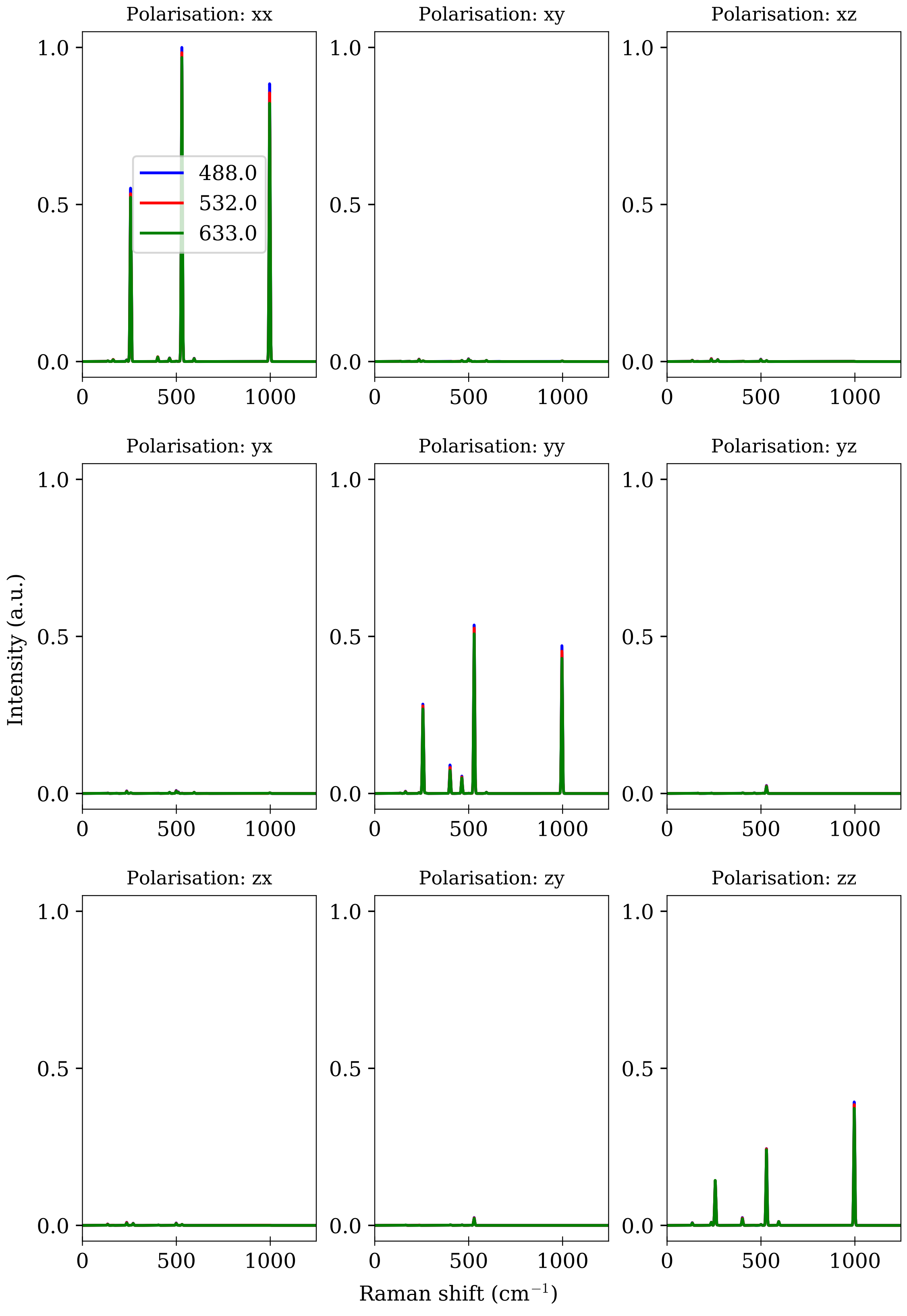

Supplementary Figure 581: Raman spectrum of Ti_2O_6 with space group 59 and Wyckoff positions a-b-e-0.

Ti_2S_2, 129, b-c-0

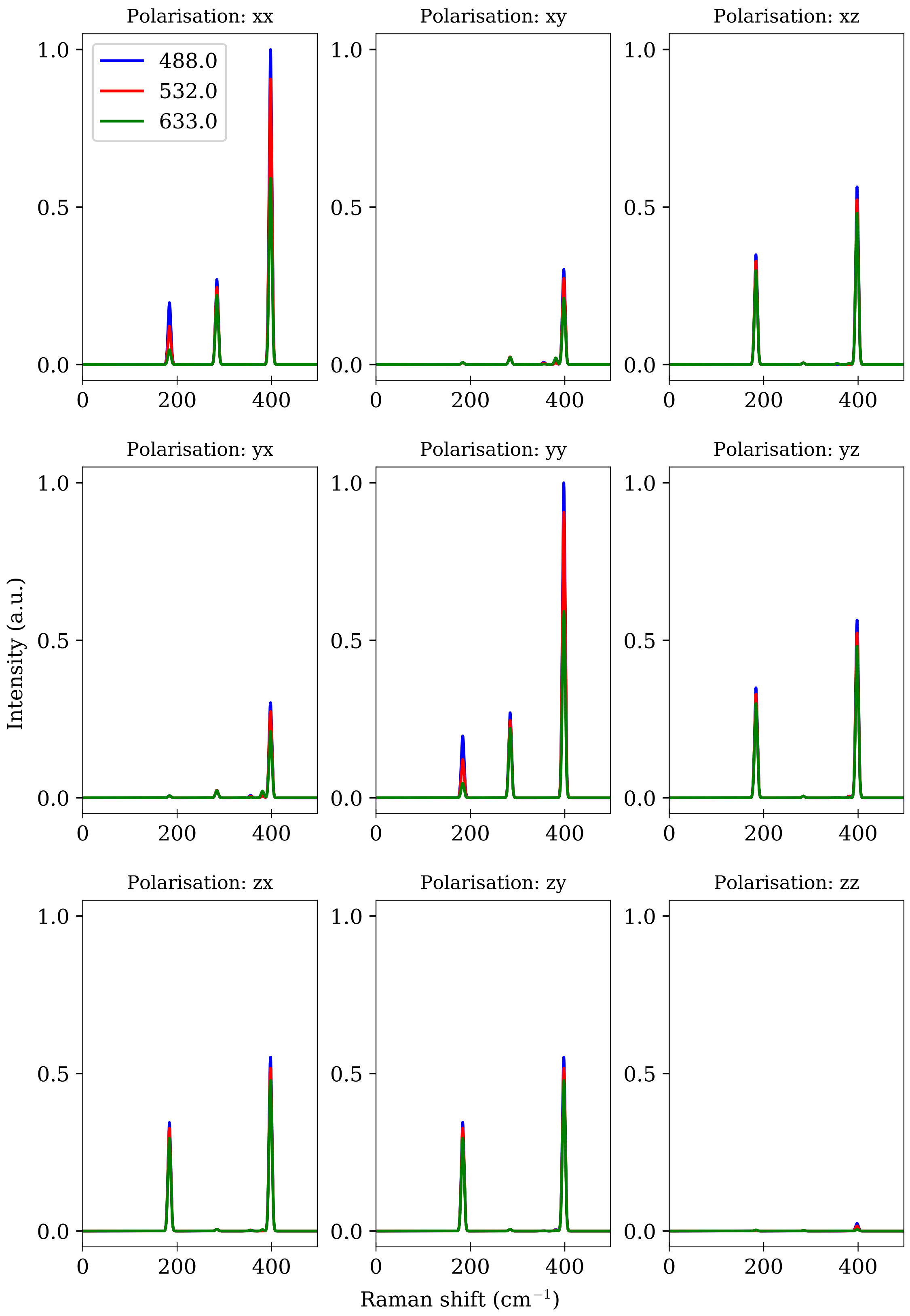

Supplementary Figure 582: Raman spectrum of Ti_2S_2 with space group 129 and Wyckoff positions b-c-0.

Ti_2S_2, 12, i-0

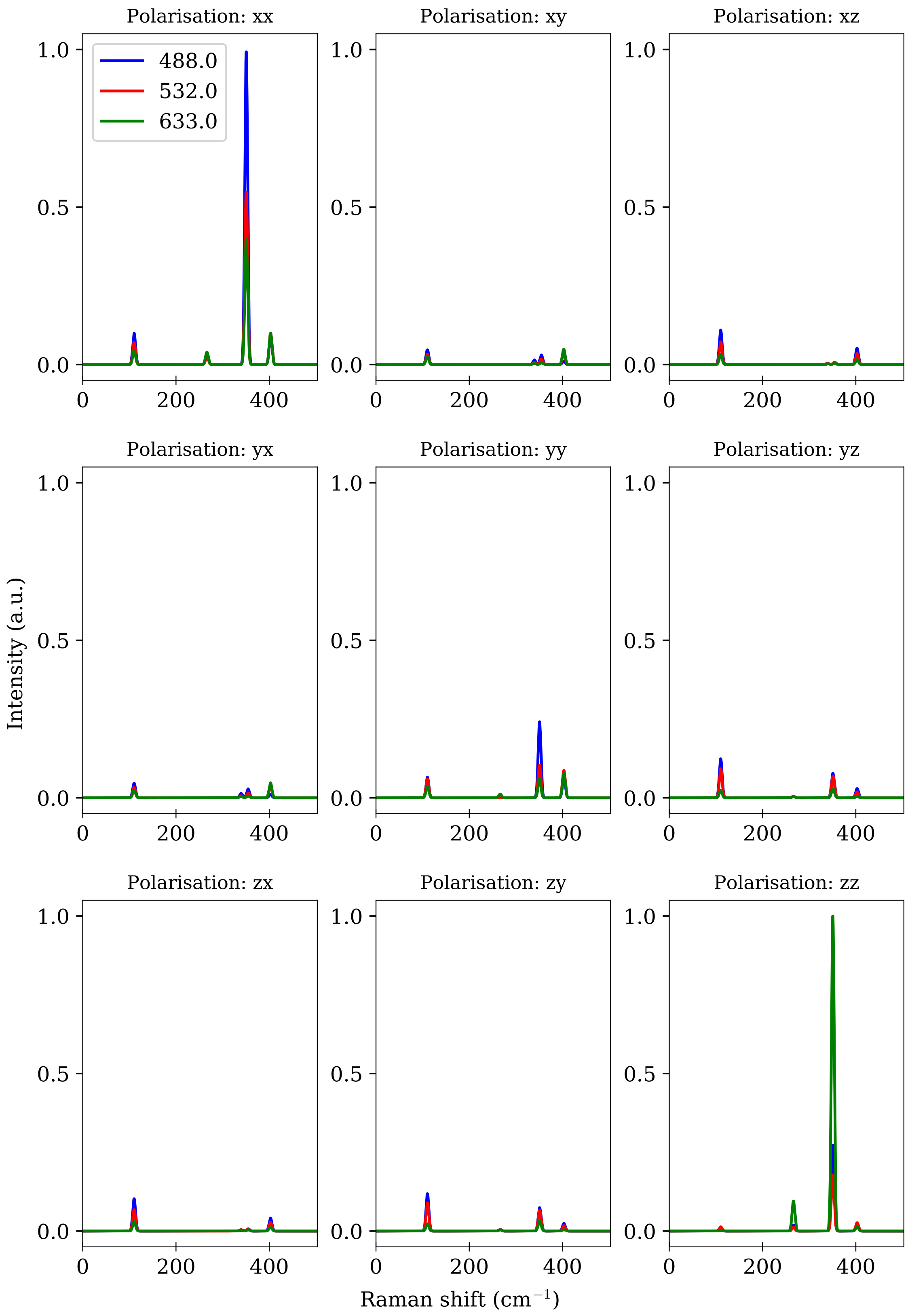

Supplementary Figure 583: Raman spectrum of Ti_2S_2 with space group 12 and Wyckoff positions i-0.

Ti_2Se_2, 12, i-0

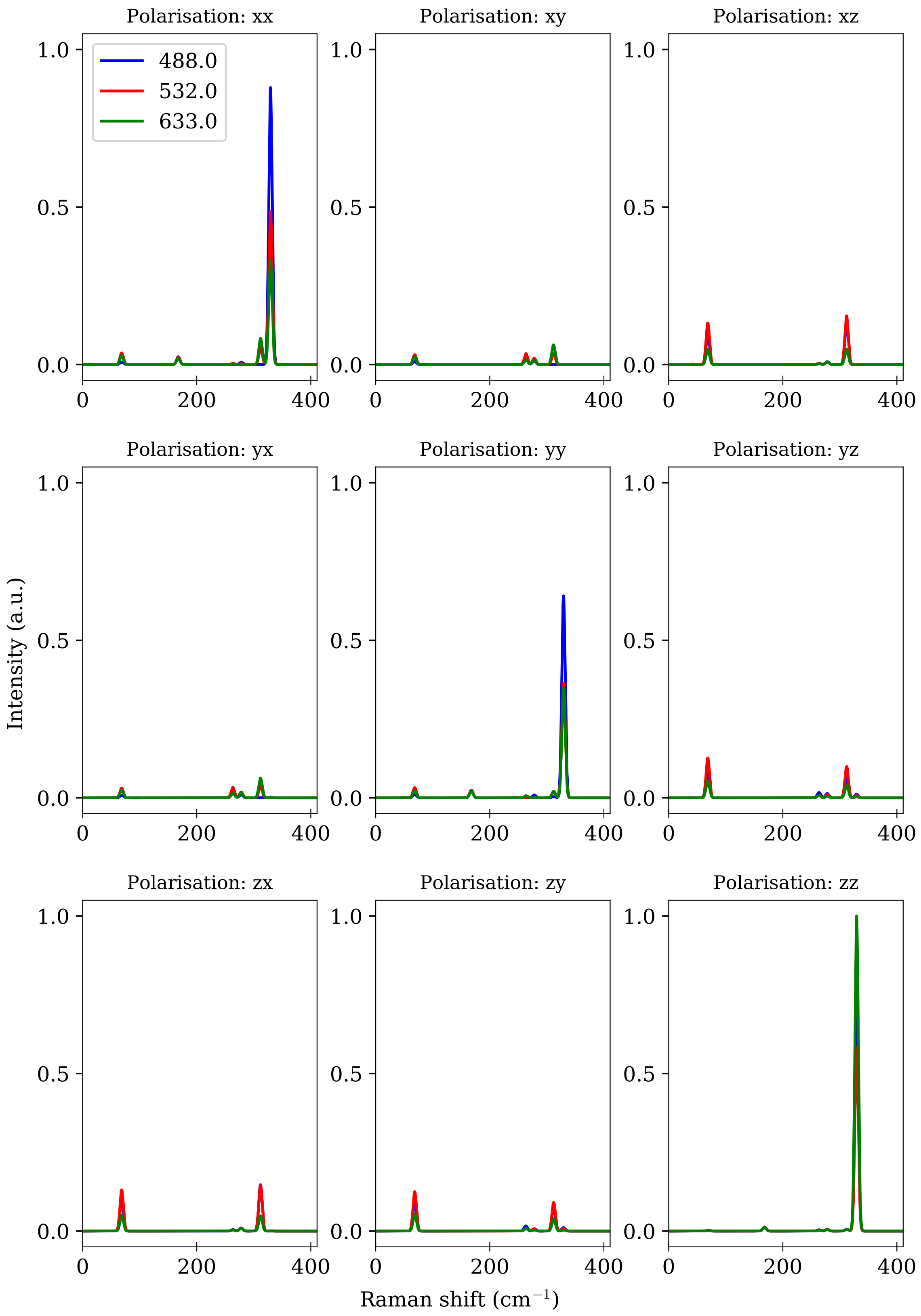

Supplementary Figure 584: Raman spectrum of Ti_2Se_2 with space group 12 and Wyckoff positions i-0.

Ti_2Se_2, 187, h-i-0

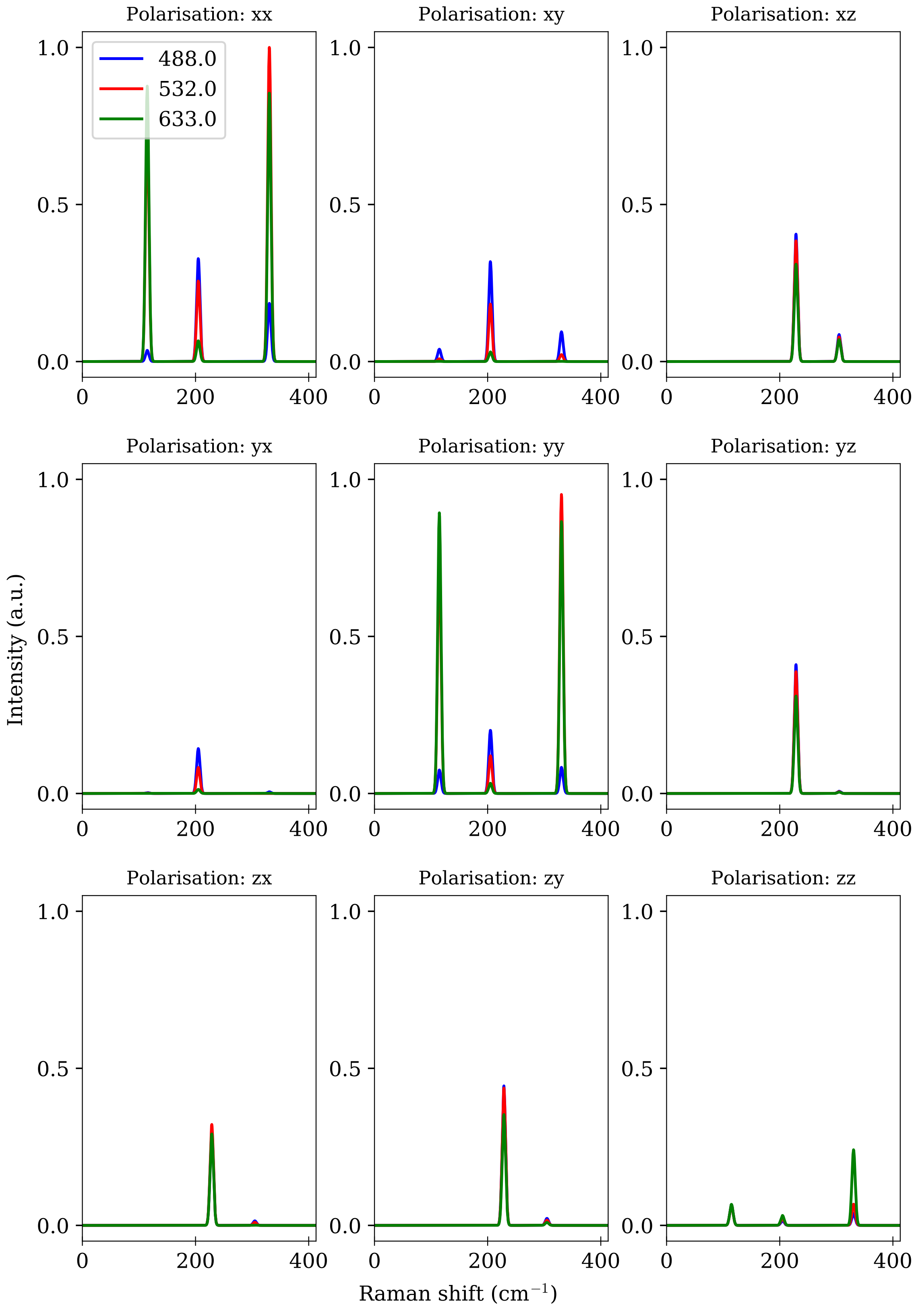

Supplementary Figure 585: Raman spectrum of Ti_2Se_2 with space group 187 and Wyckoff positions h-i-0.

Ti_2Te_2, 187, h-i-0

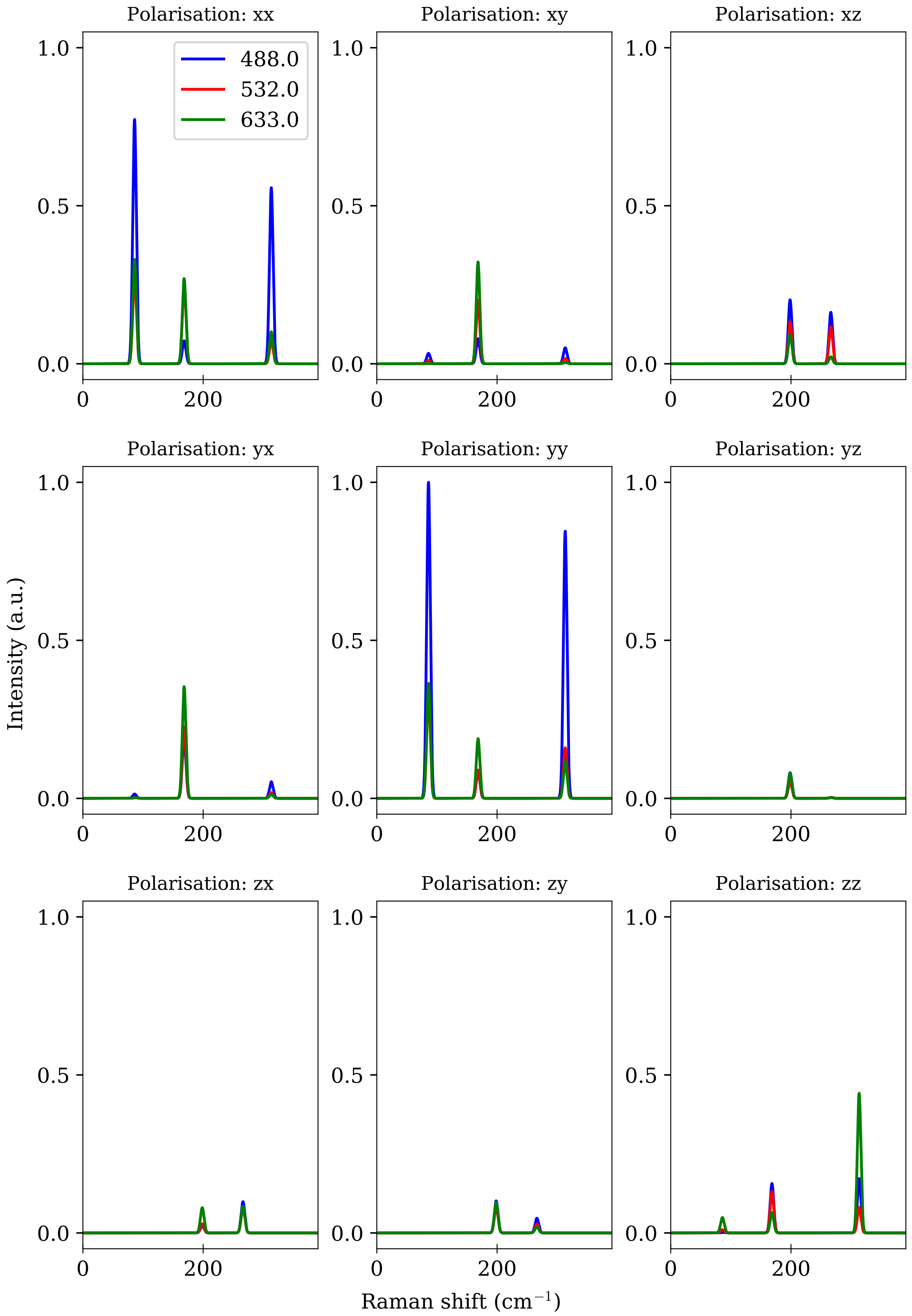

Supplementary Figure 586: Raman spectrum of Ti_2Te_2 with space group 187 and Wyckoff positions h-i-0.

Ti_2Te_2, 8, a-0

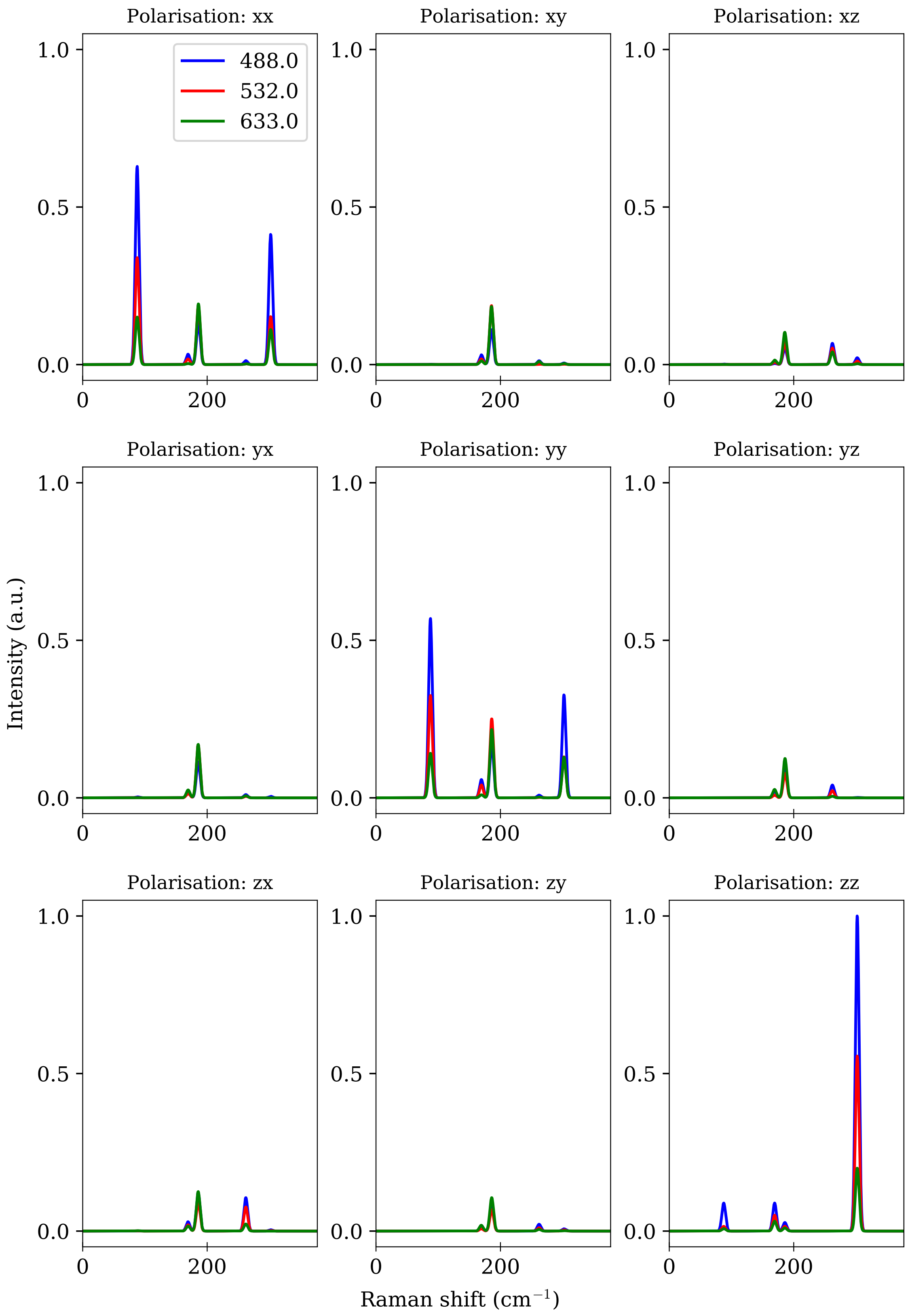

Supplementary Figure 587: Raman spectrum of Ti_2Te_2 with space group 8 and Wyckoff positions a-0.

$Ti_3C_2F_2$, 187, a-g-h-i-0

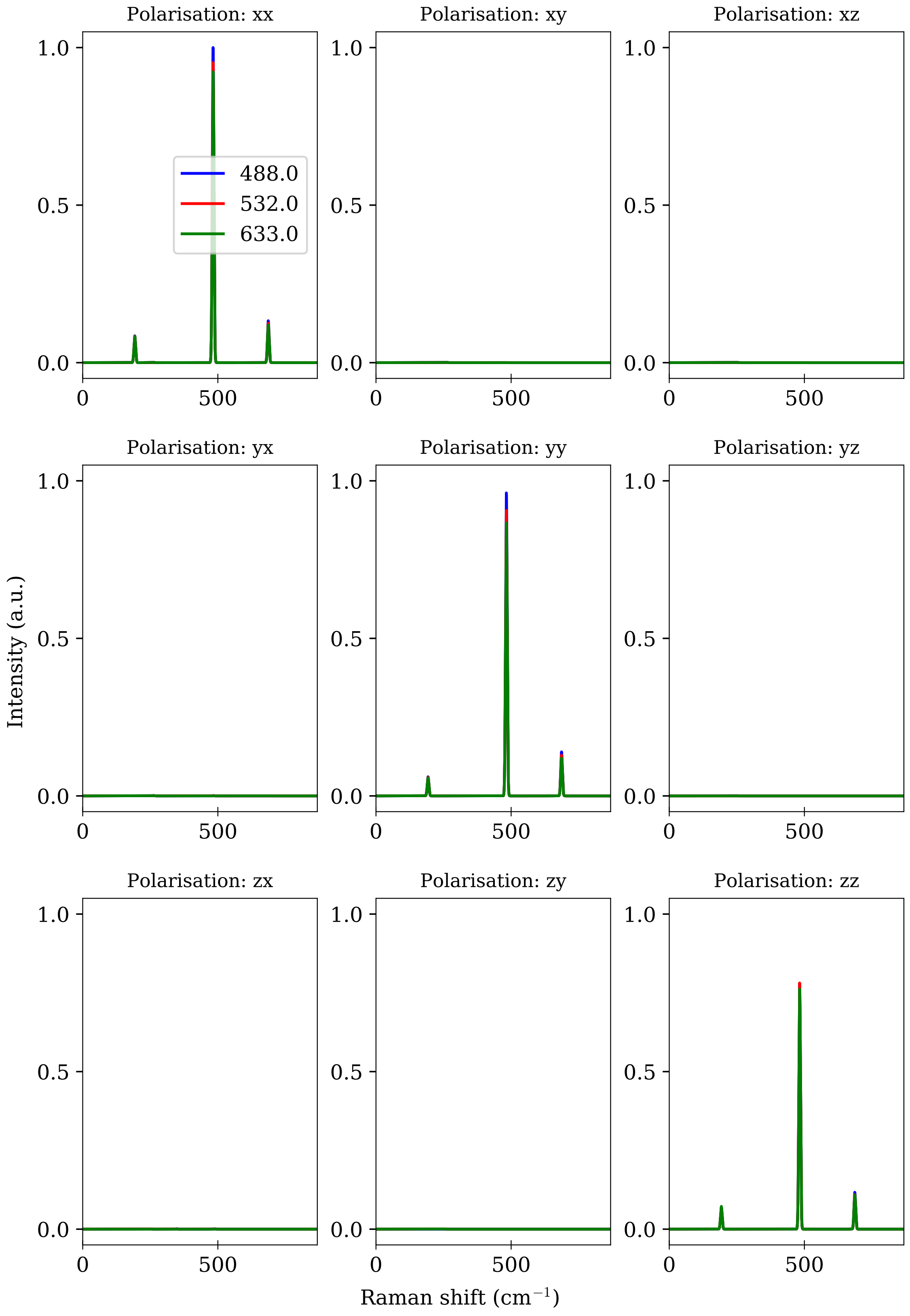

Supplementary Figure 588: Raman spectrum of $Ti_3C_2F_2$ with space group 187 and Wyckoff positions a-g-h-i-0.

$Ti_3C_2O_2$, 187, b-g-h-i-0

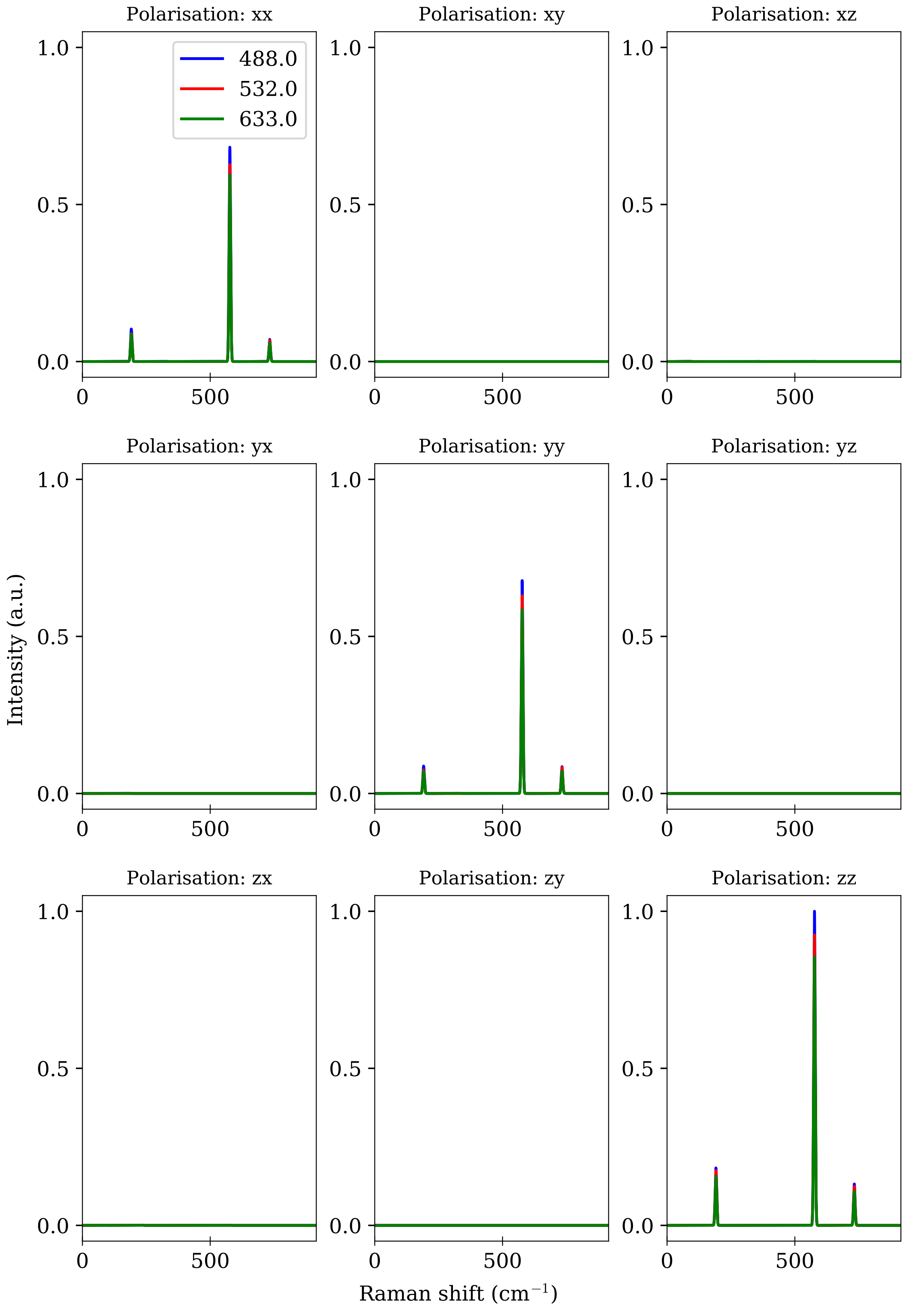

Supplementary Figure 589: Raman spectrum of $Ti_3C_2O_2$ with space group 187 and Wyckoff positions b-g-h-i-0.

$Ti_3N_2O_2$, 187, a-g-h-i-0

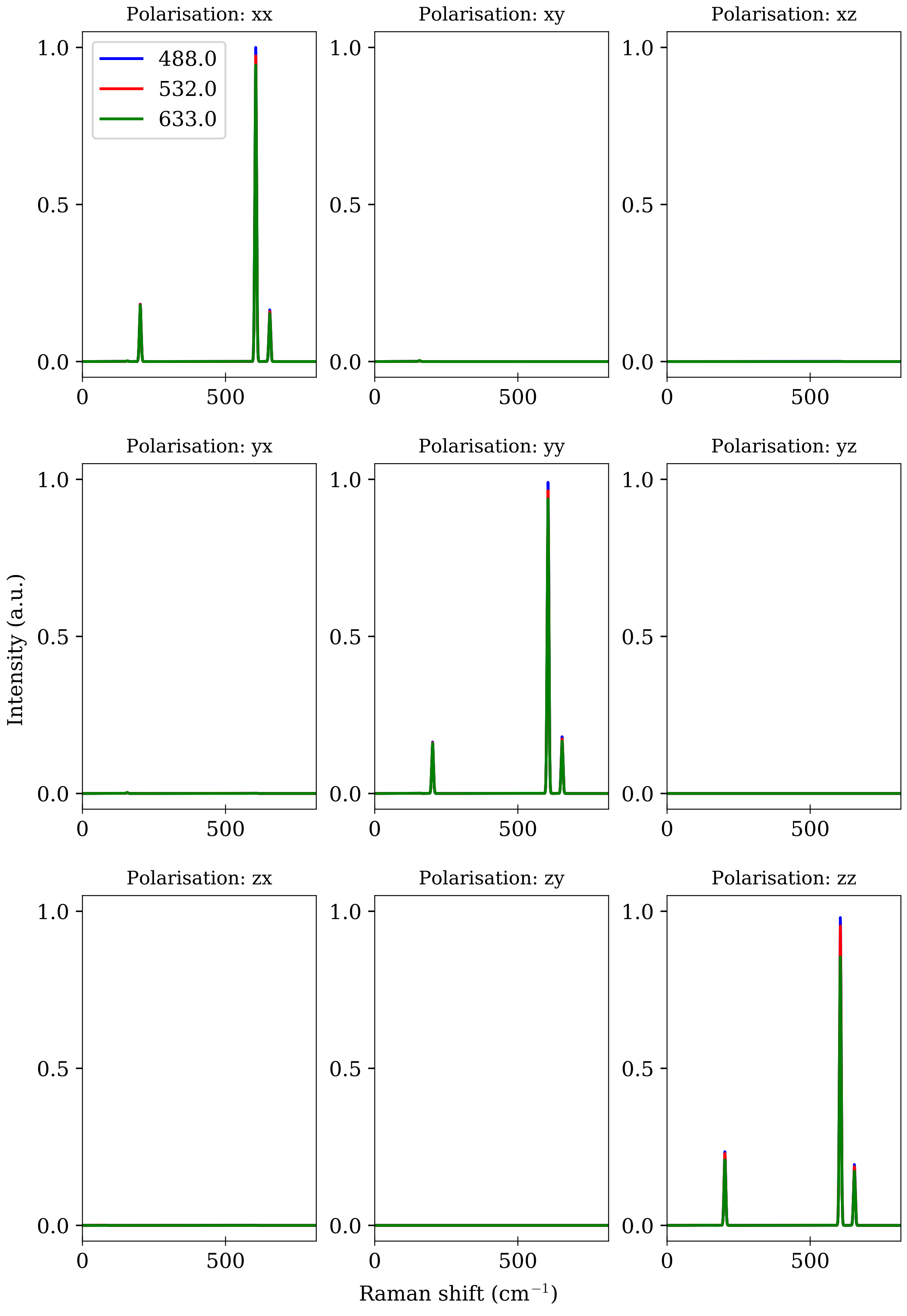

Supplementary Figure 590: Raman spectrum of $Ti_3N_2O_2$ with space group 187 and Wyckoff positions a-g-h-i-0.

Ti_3N_2, 187, b-h-i-0

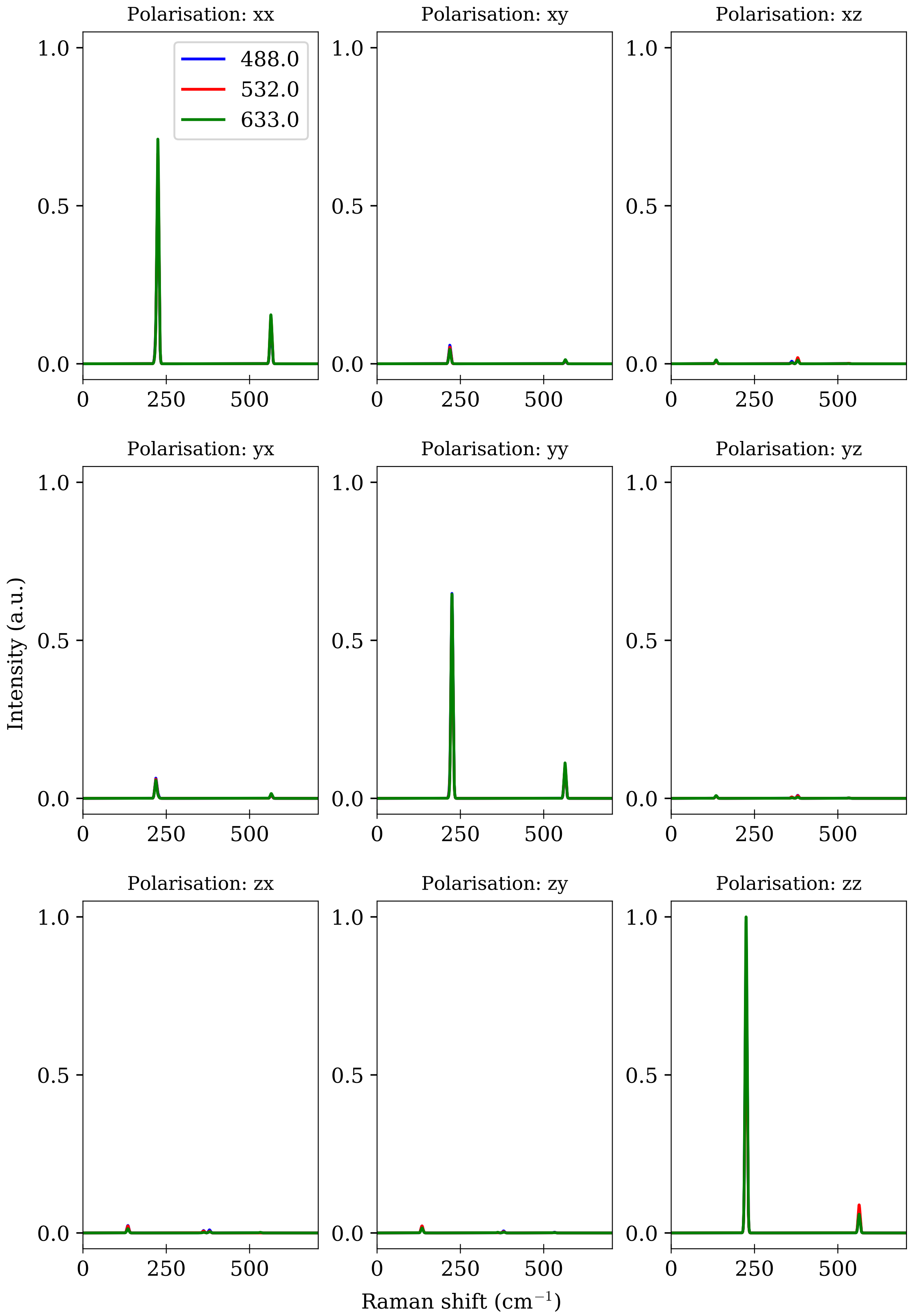

Supplementary Figure 591: Raman spectrum of Ti_3N_2 with space group 187 and Wyckoff positions b-h-i-0.

$TiAs_2$, 164, b-d-0

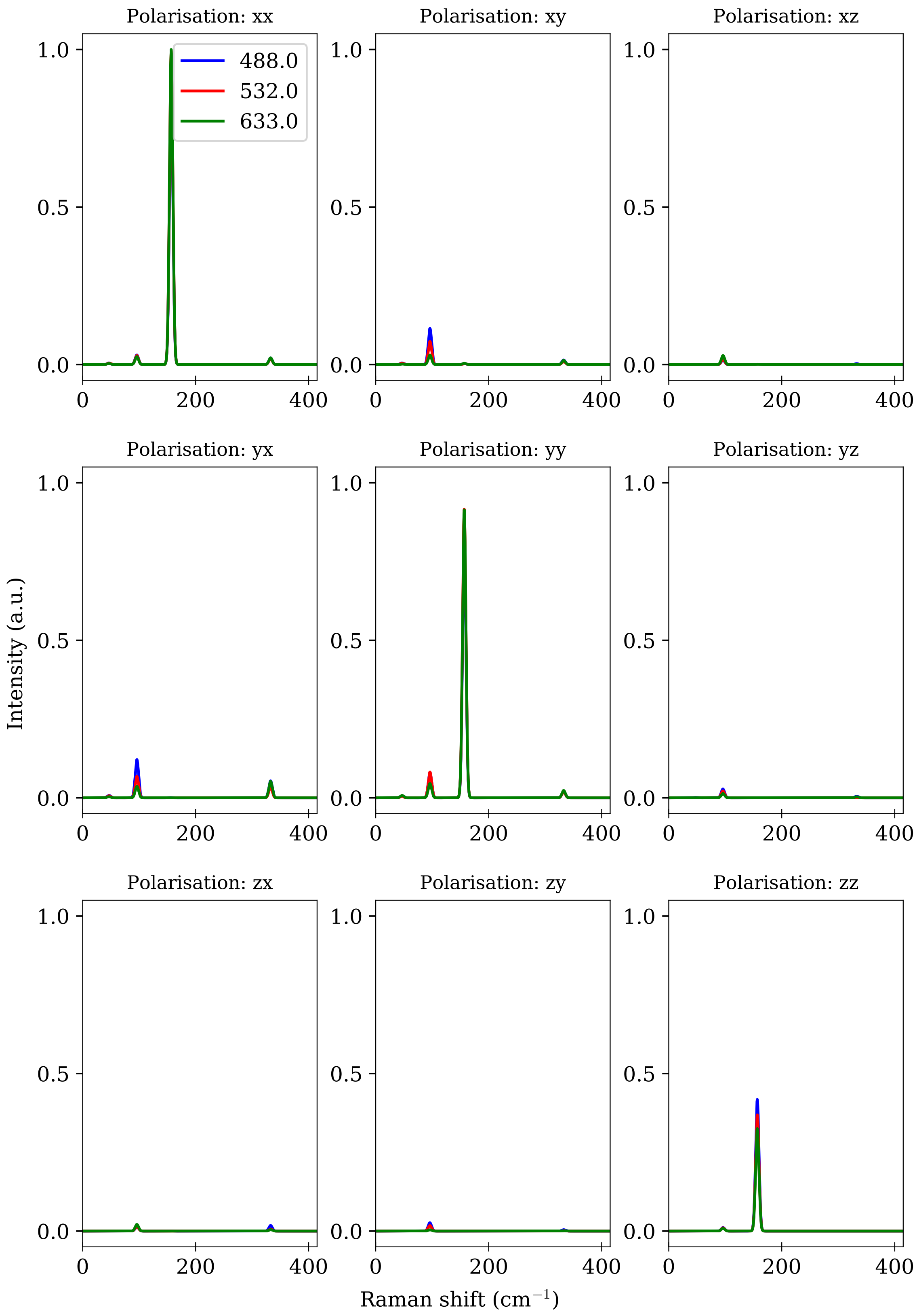

Supplementary Figure 592: Raman spectrum of $TiAs_2$ with space group 164 and Wyckoff positions b-d-0.

TiAs$_2$, 187, a-i-0

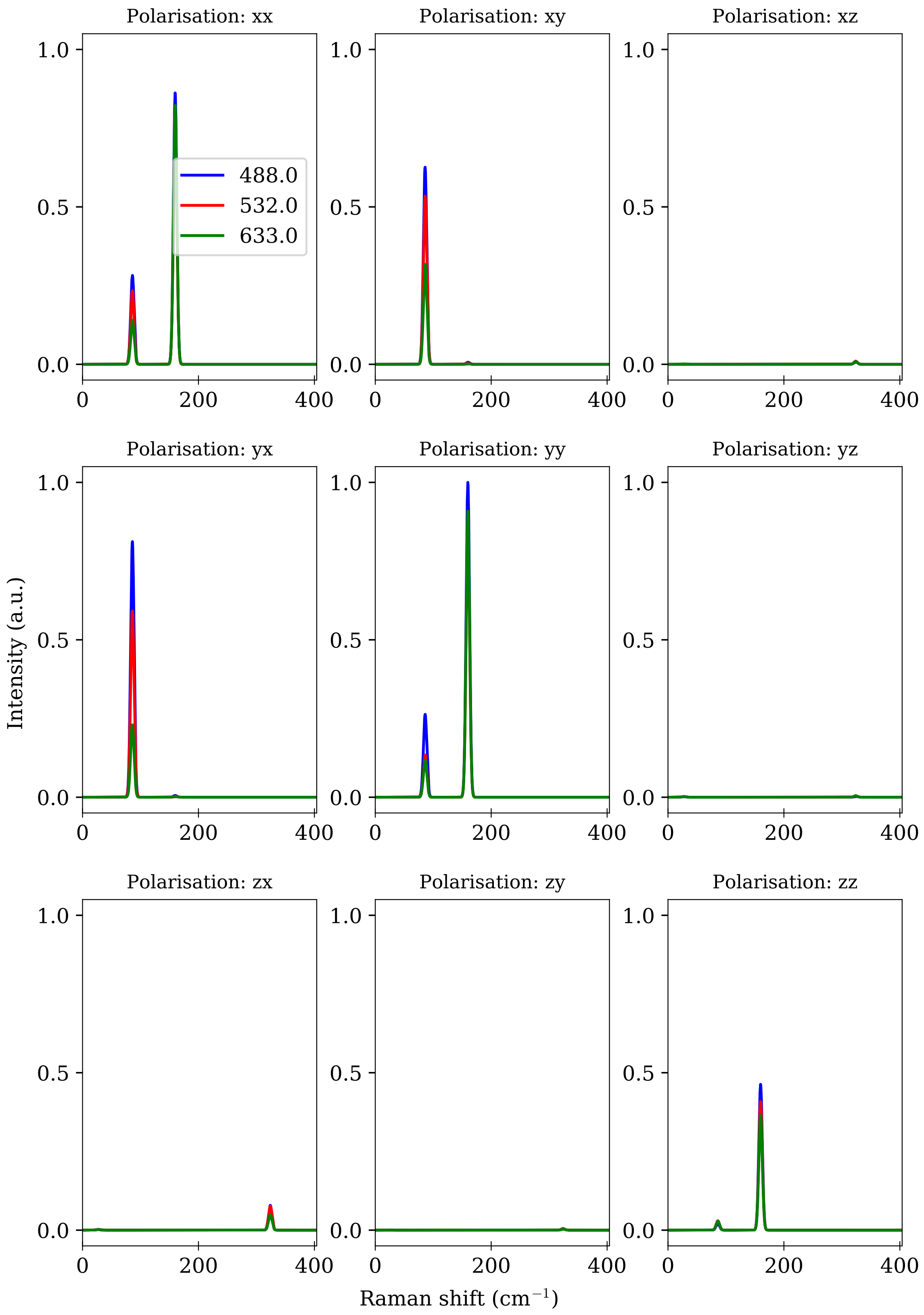

Supplementary Figure 593: Raman spectrum of TiAs$_2$ with space group 187 and Wyckoff positions a-i-0.

TiAsSb, 156, a-b-c-0

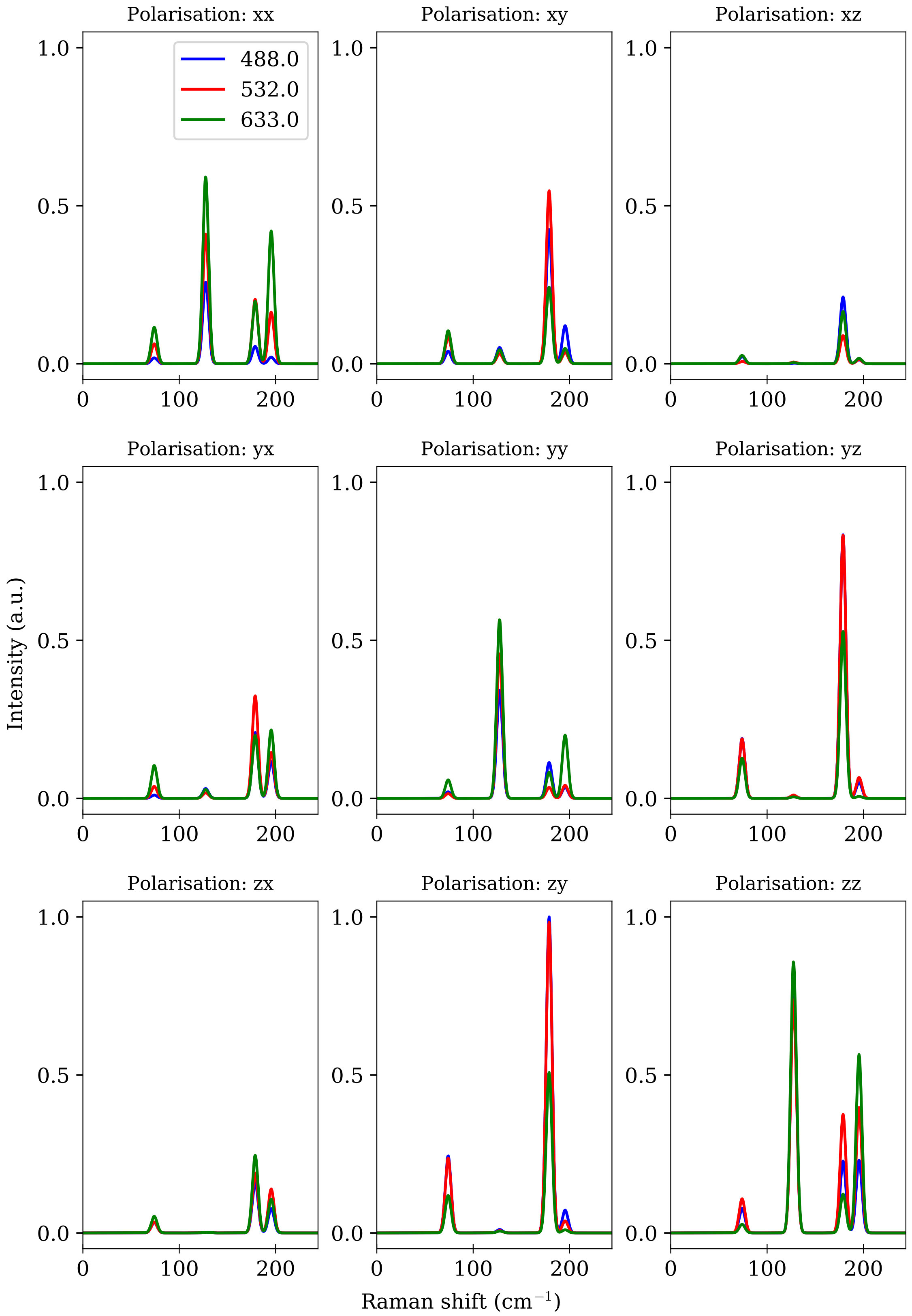

Supplementary Figure 594: Raman spectrum of TiAsSb with space group 156 and Wyckoff positions a-b-c-0.

$TiBr_2$, 187, b-i-0

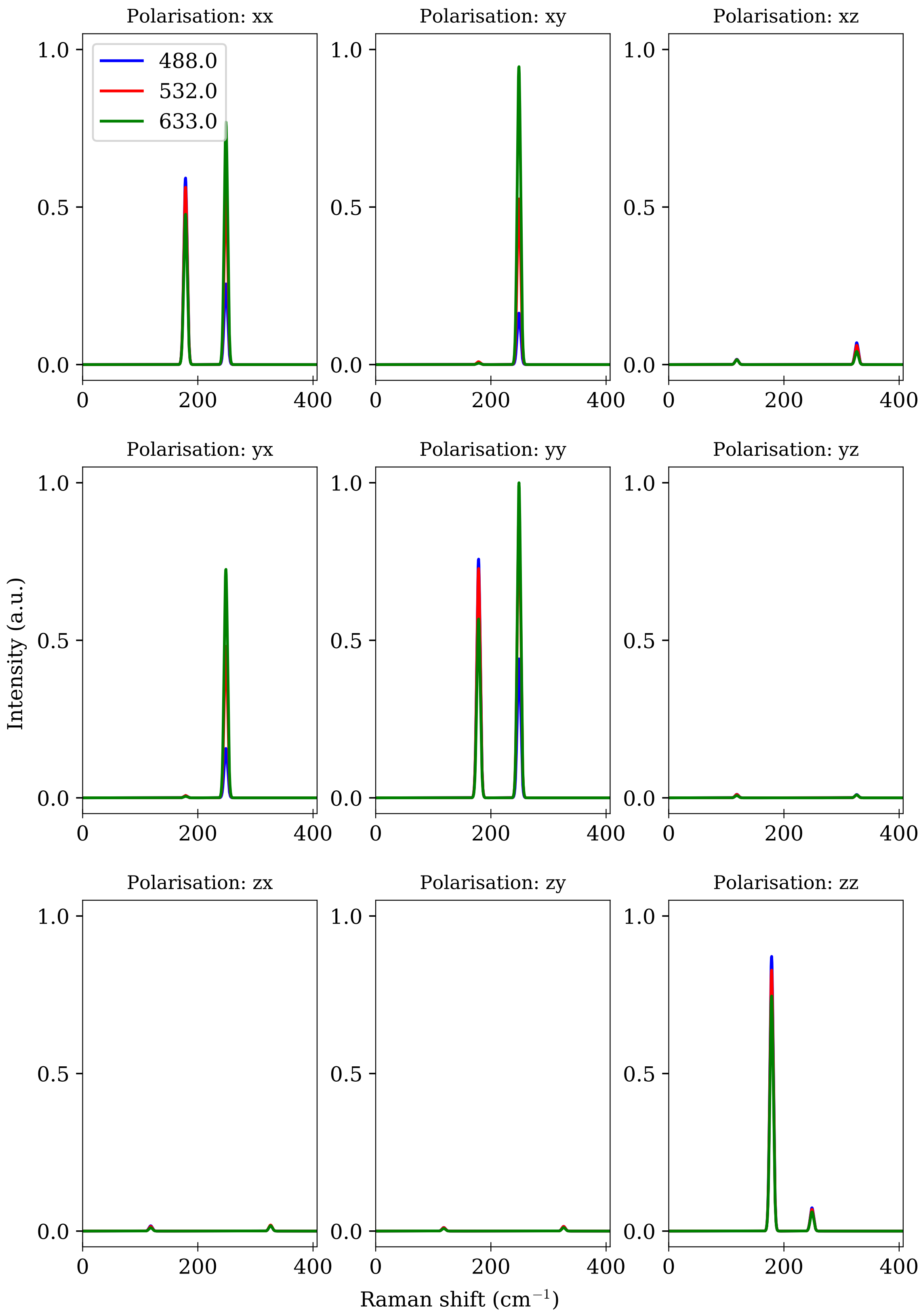

Supplementary Figure 595: Raman spectrum of $TiBr_2$ with space group 187 and Wyckoff positions b-i-0.

TiBrCl, 156, a-c-0

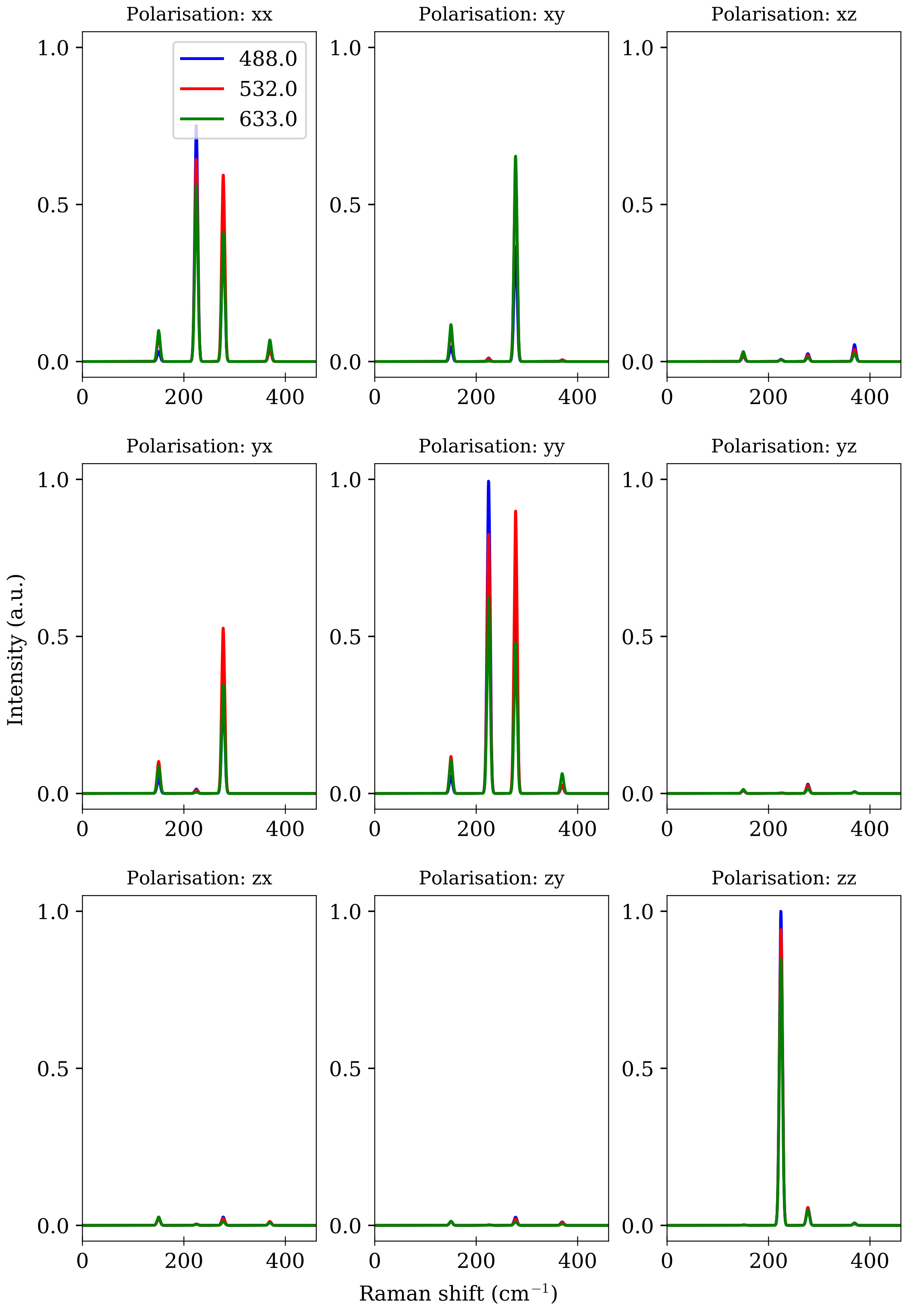

Supplementary Figure 596: Raman spectrum of TiBrCl with space group 156 and Wyckoff positions a-c-0.

TiBrI, 156, a-c-0

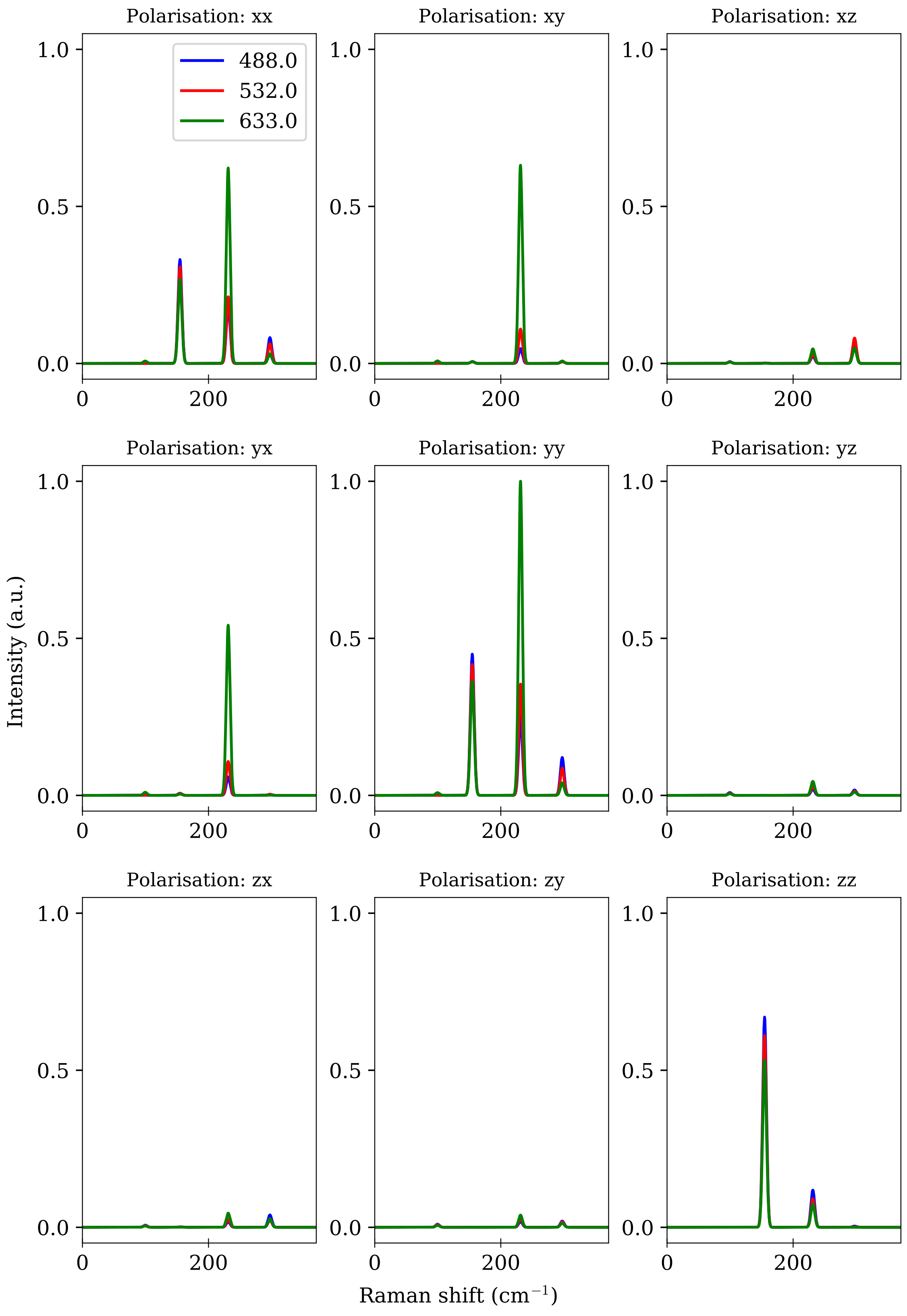

Supplementary Figure 597: Raman spectrum of TiBrI with space group 156 and Wyckoff positions a-c-0.

$TiCl_2$, 187, a-i-0

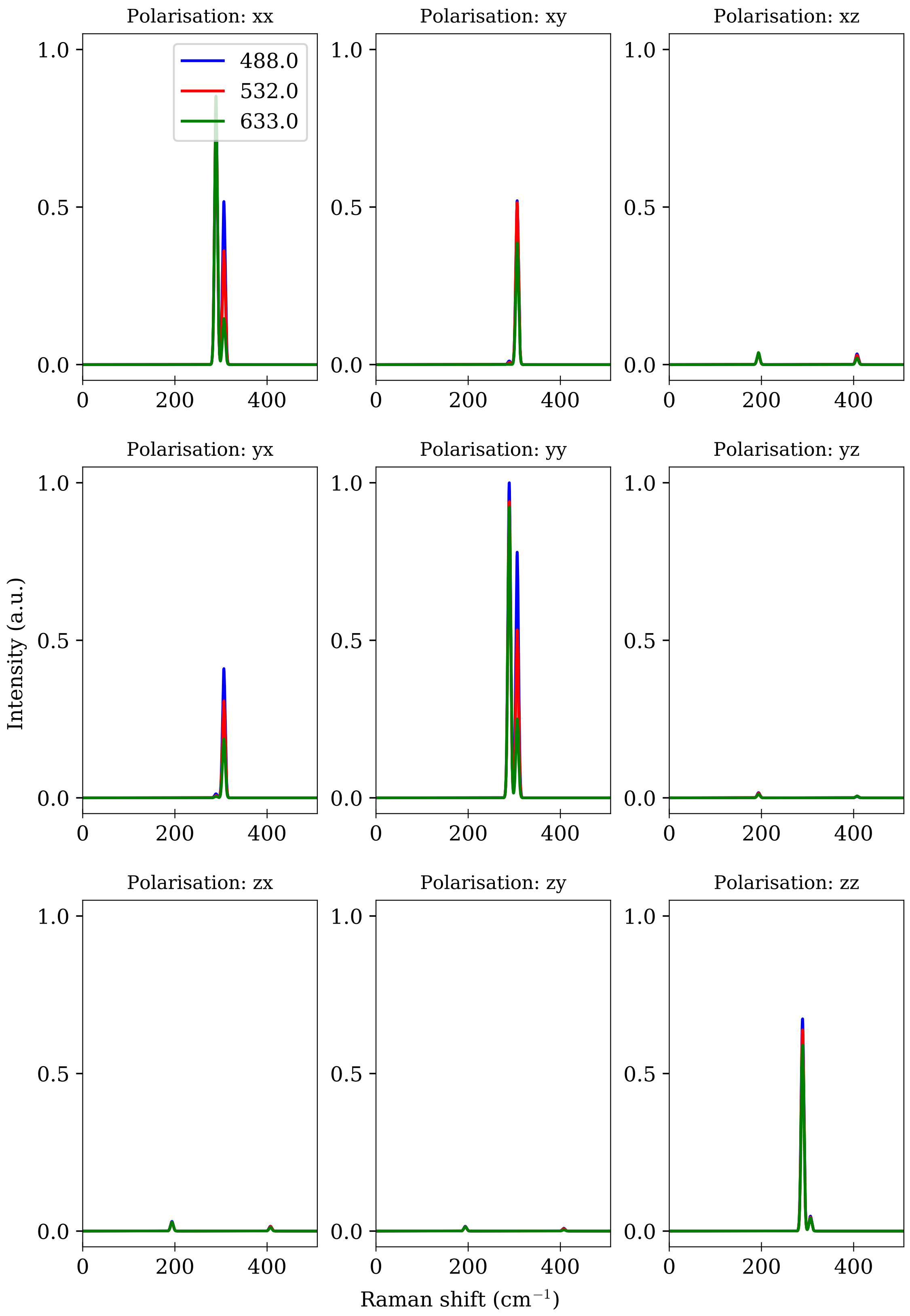

Supplementary Figure 598: Raman spectrum of $TiCl_2$ with space group 187 and Wyckoff positions a-i-0.

TiClI, 156, a-c-0

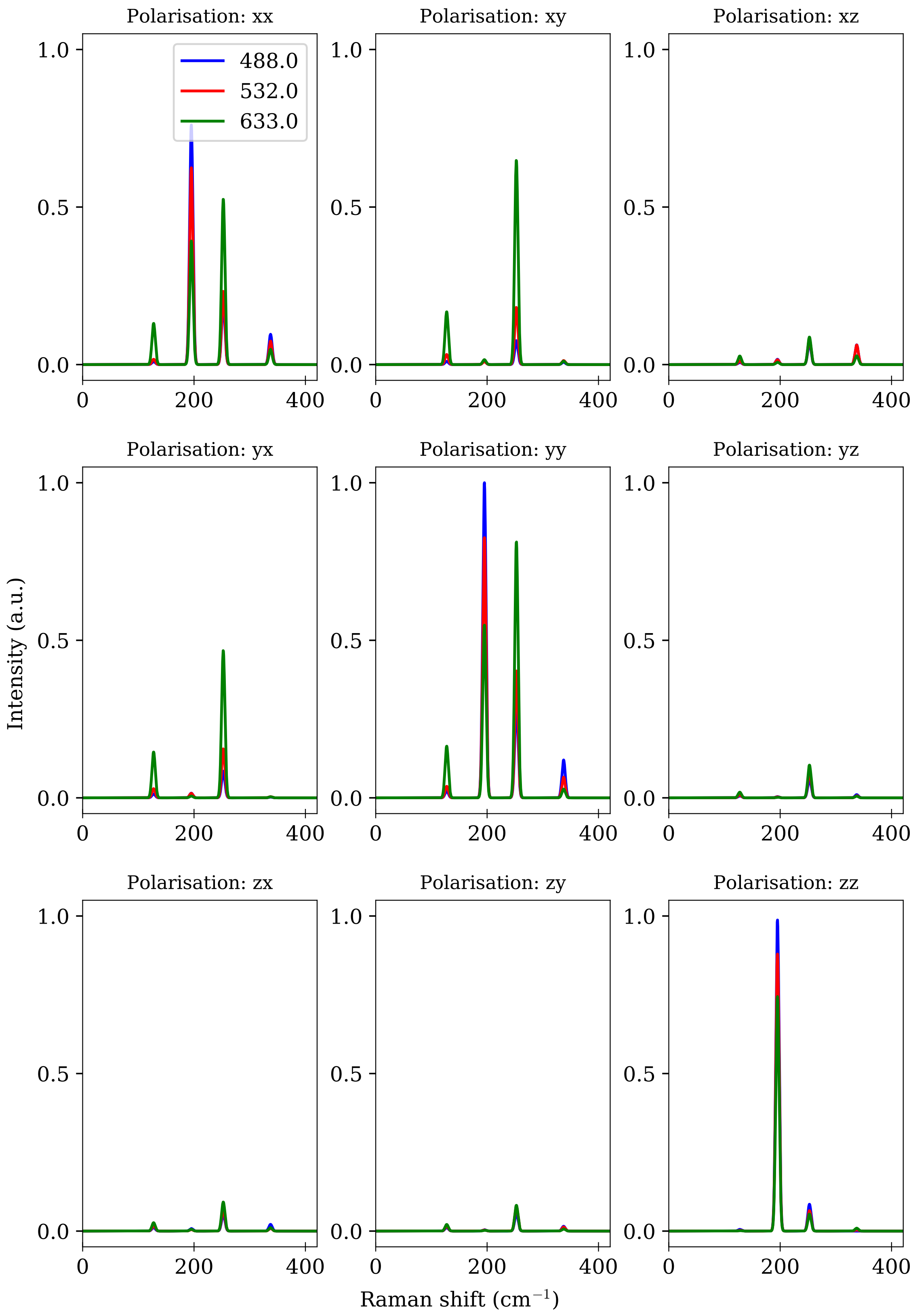

Supplementary Figure 599: Raman spectrum of TiClI with space group 156 and Wyckoff positions a-c-0.

TiH_2, 187, a-i-0

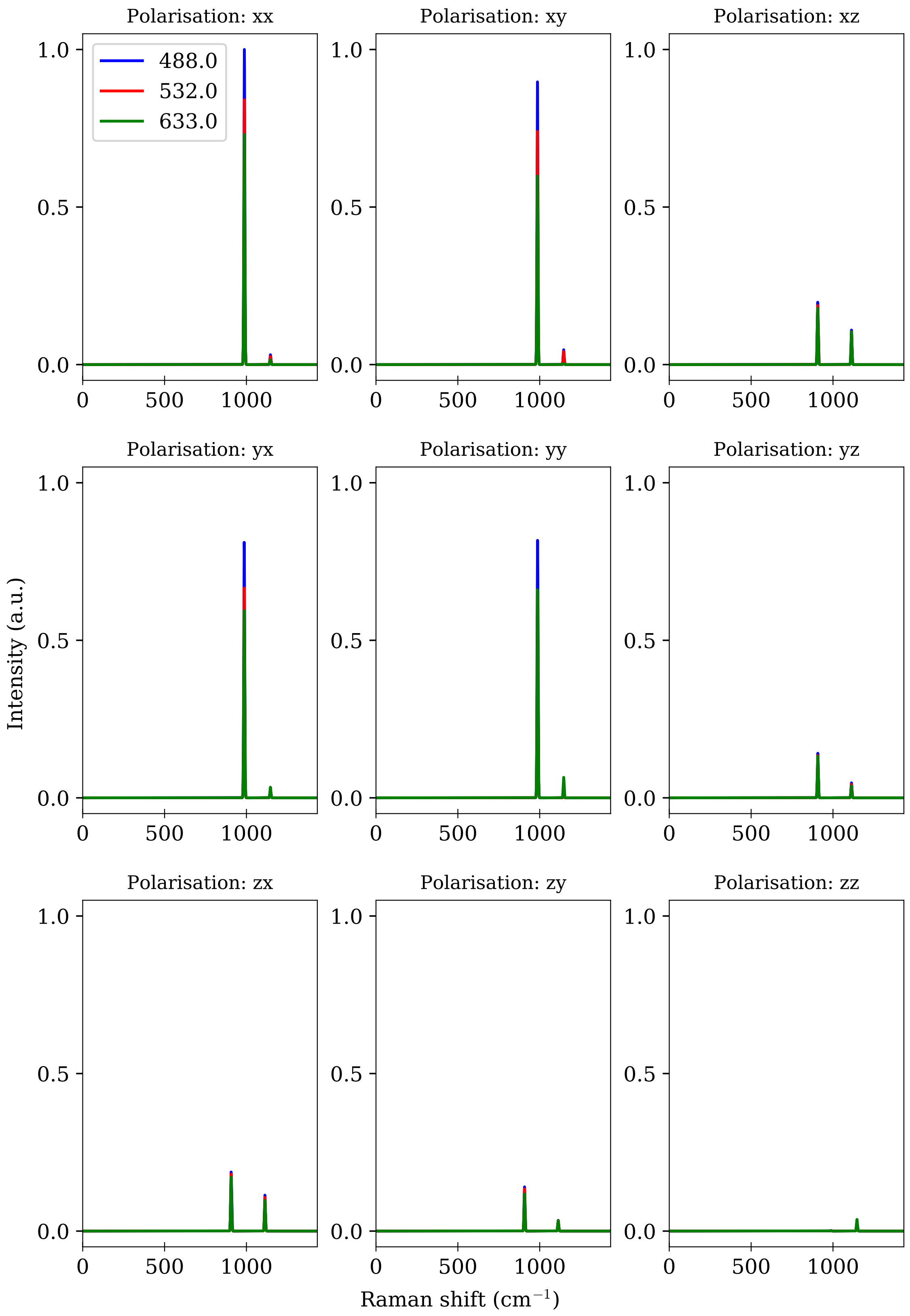

Supplementary Figure 600: Raman spectrum of TiH_2 with space group 187 and Wyckoff positions a-i-0.

TiI_2, 187, a-i-0

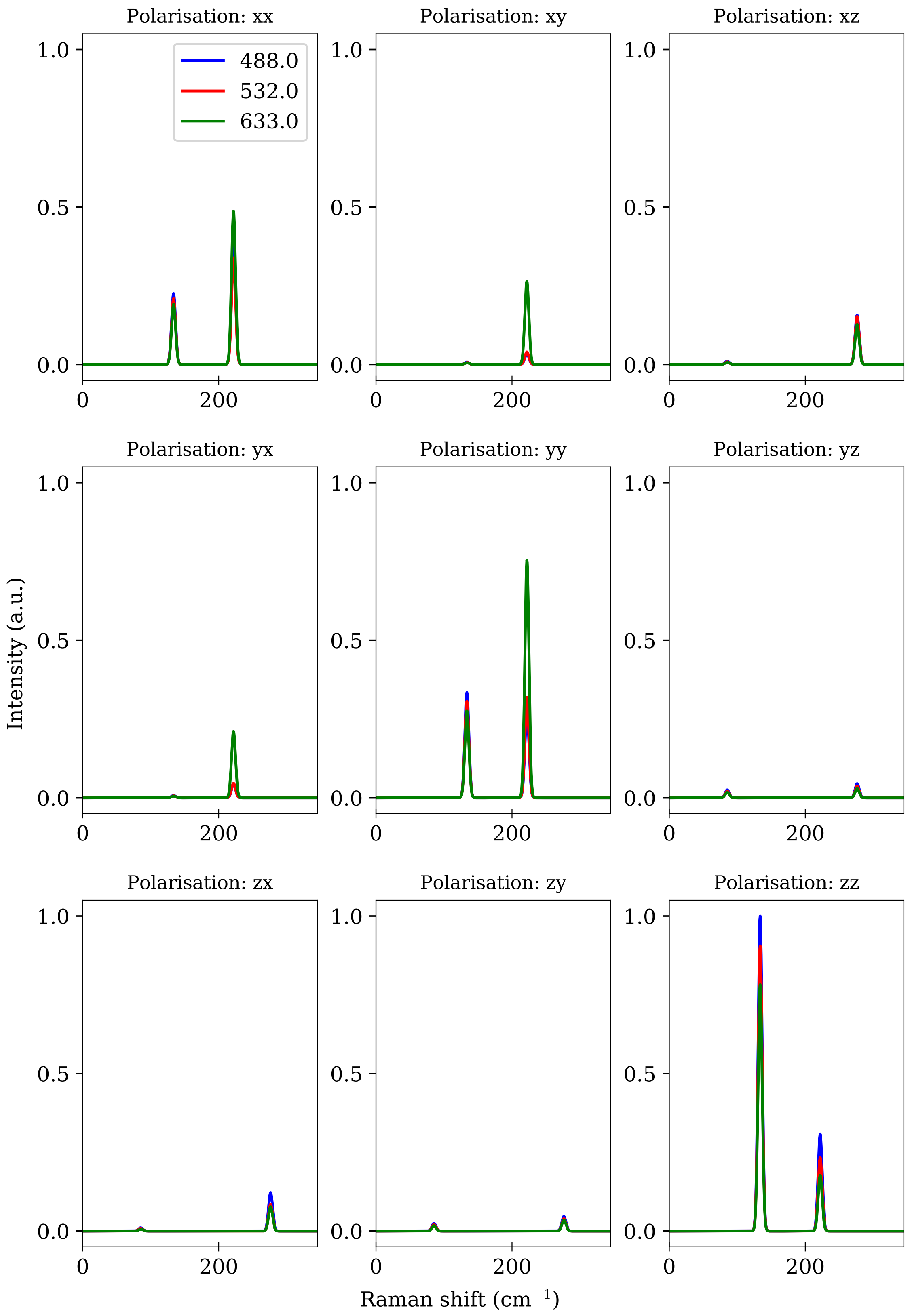

Supplementary Figure 601: Raman spectrum of TiI_2 with space group 187 and Wyckoff positions a-i-0.

TiO_2, 115, d-g-0

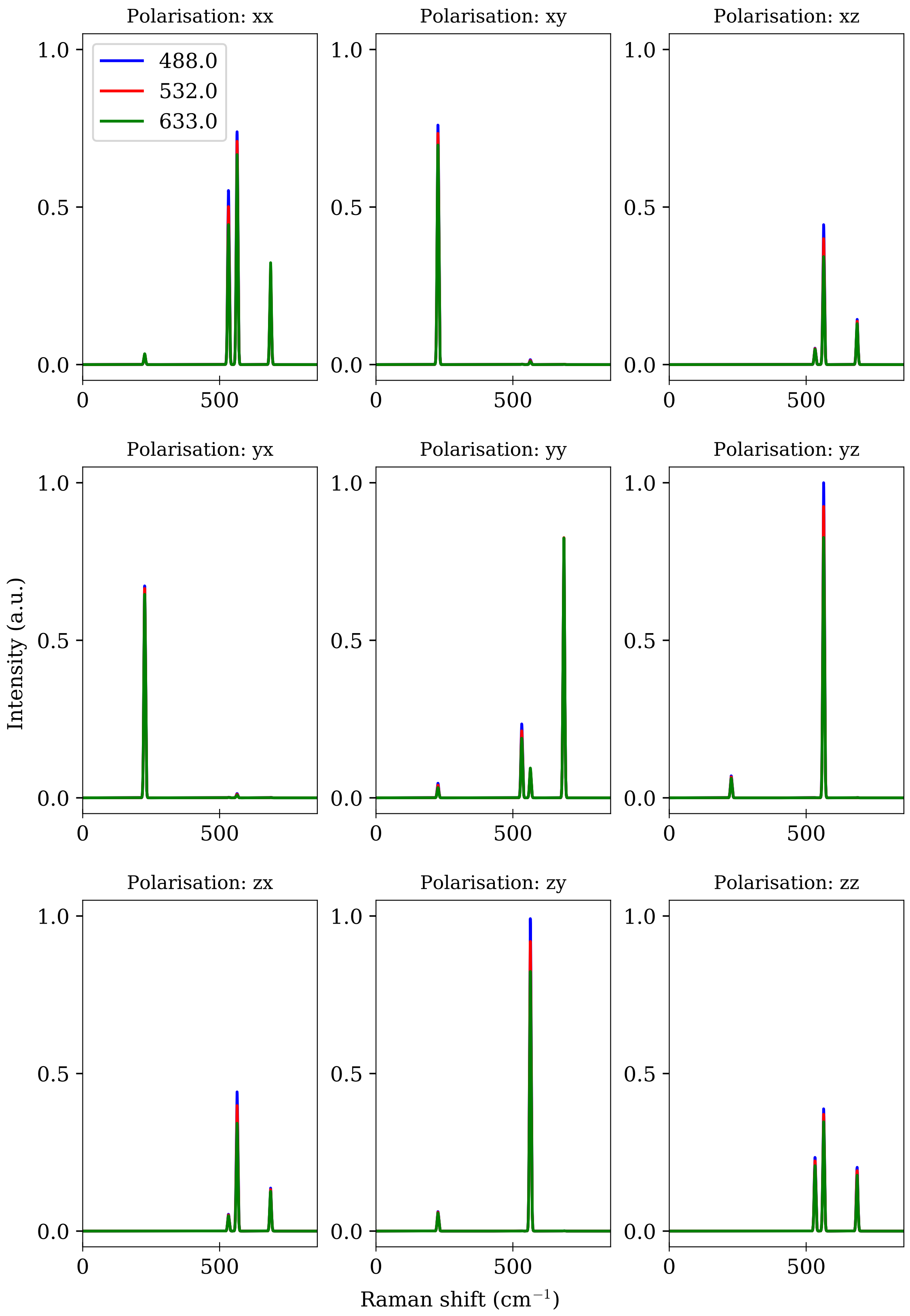

Supplementary Figure 602: Raman spectrum of TiO_2 with space group 115 and Wyckoff positions d-g-0.

TiS_2, 115, d-g-0

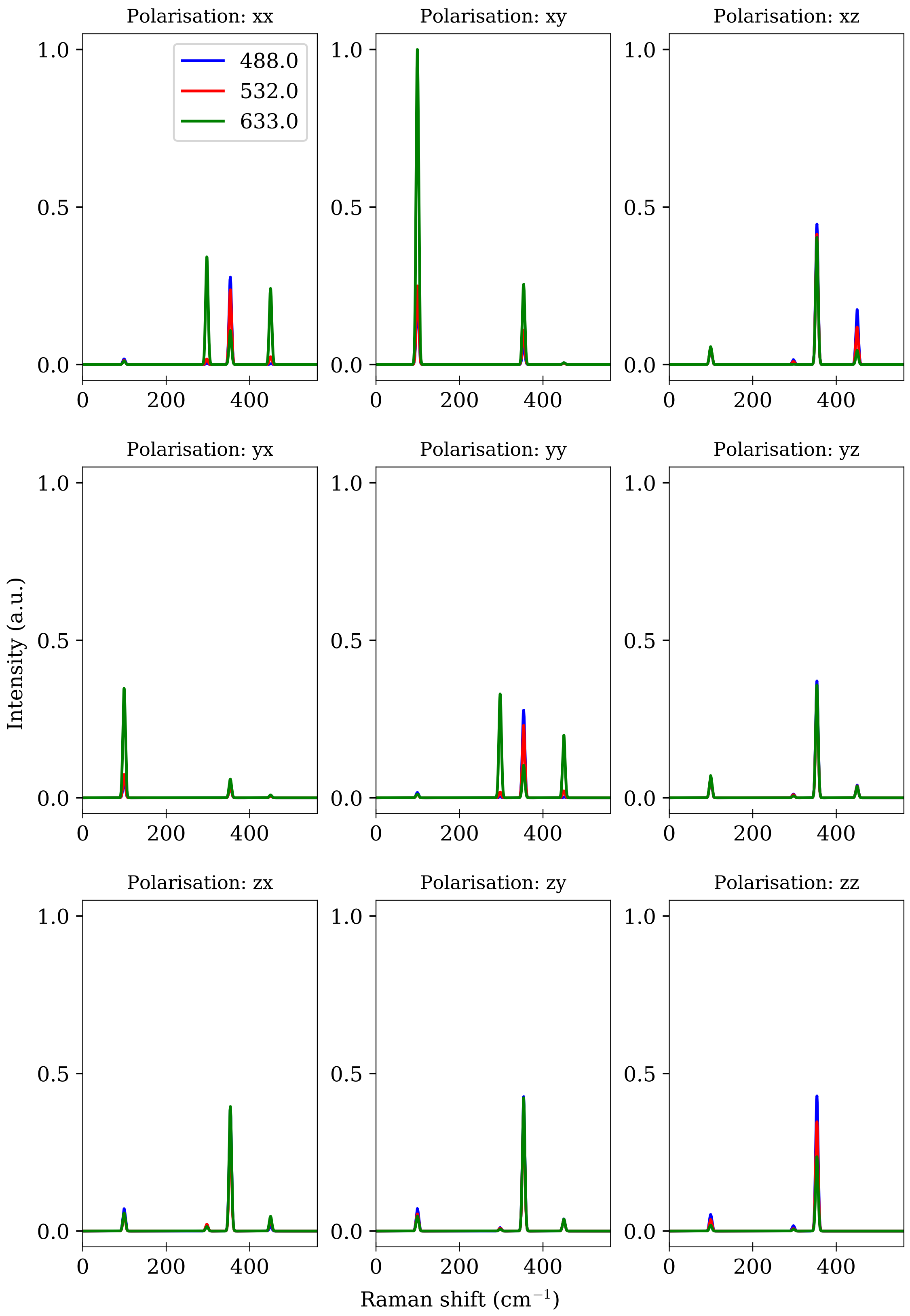

Supplementary Figure 603: Raman spectrum of TiS_2 with space group 115 and Wyckoff positions d-g-0.

$TiSb_2$, 164, b-d-0

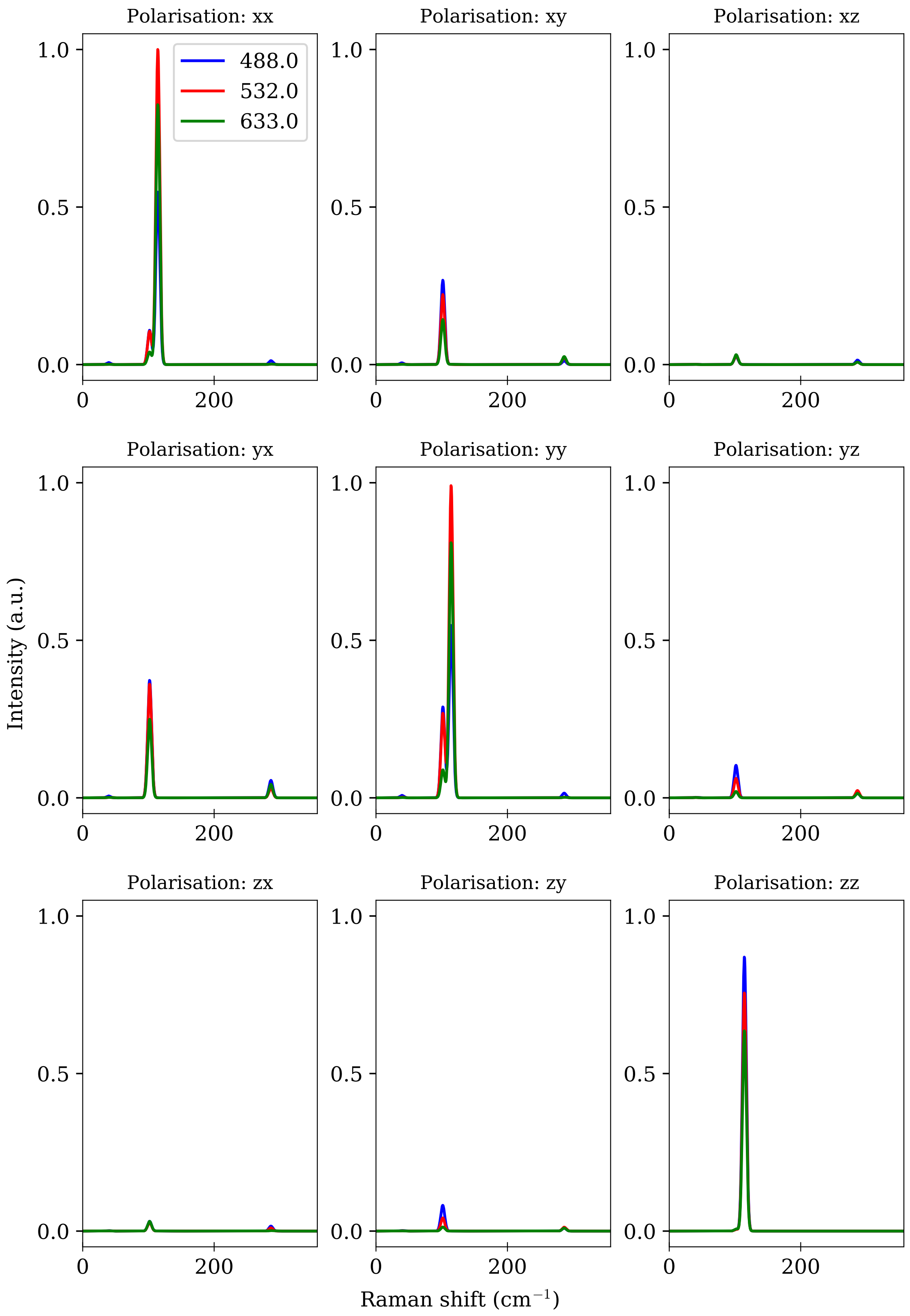

Supplementary Figure 604: Raman spectrum of $TiSb_2$ with space group 164 and Wyckoff positions b-d-0.

$TiSb_2$, 187, a-i-0

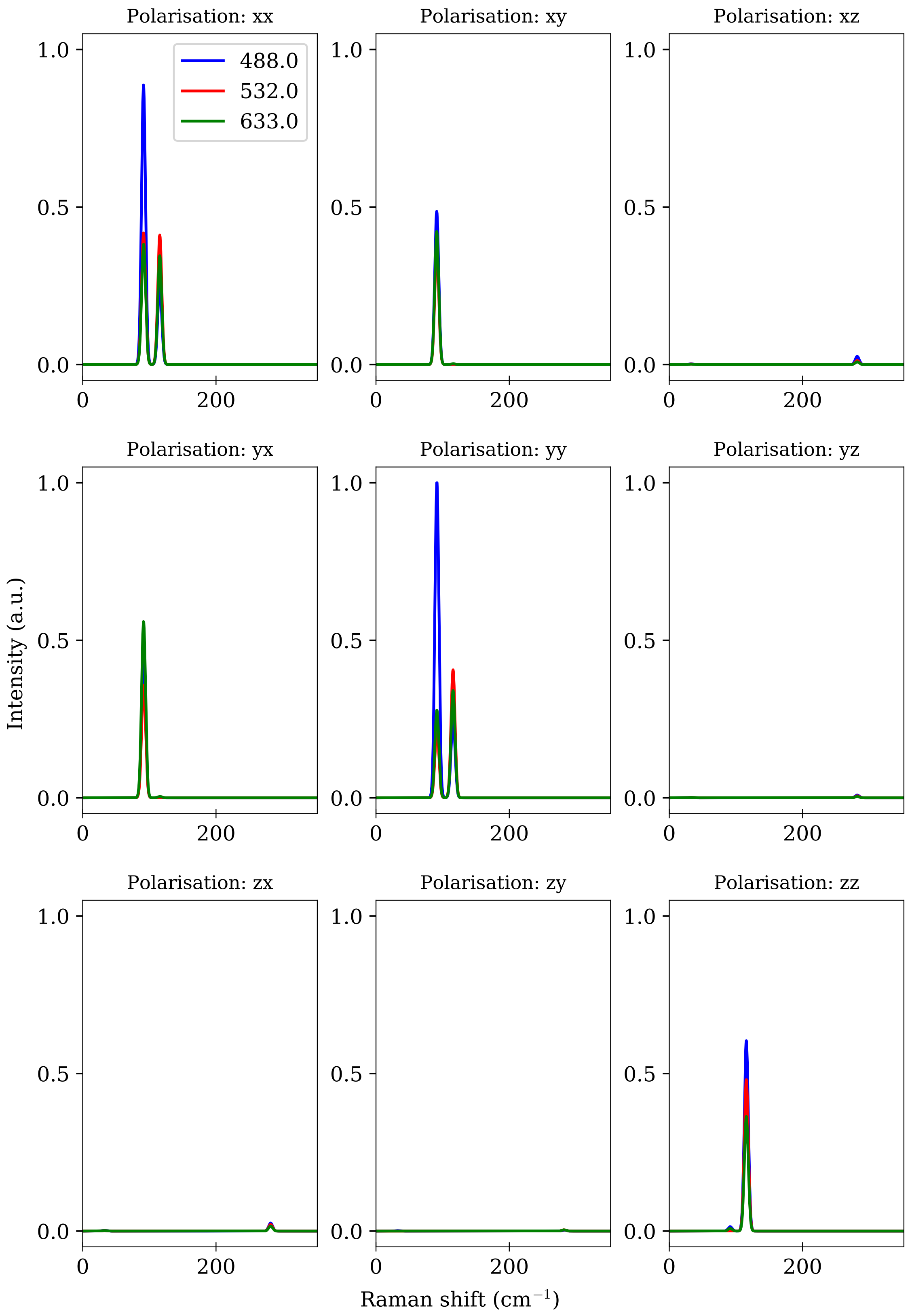

Supplementary Figure 605: Raman spectrum of $TiSb_2$ with space group 187 and Wyckoff positions a-i-0.

$TiSe_2$, 115, d-g-0

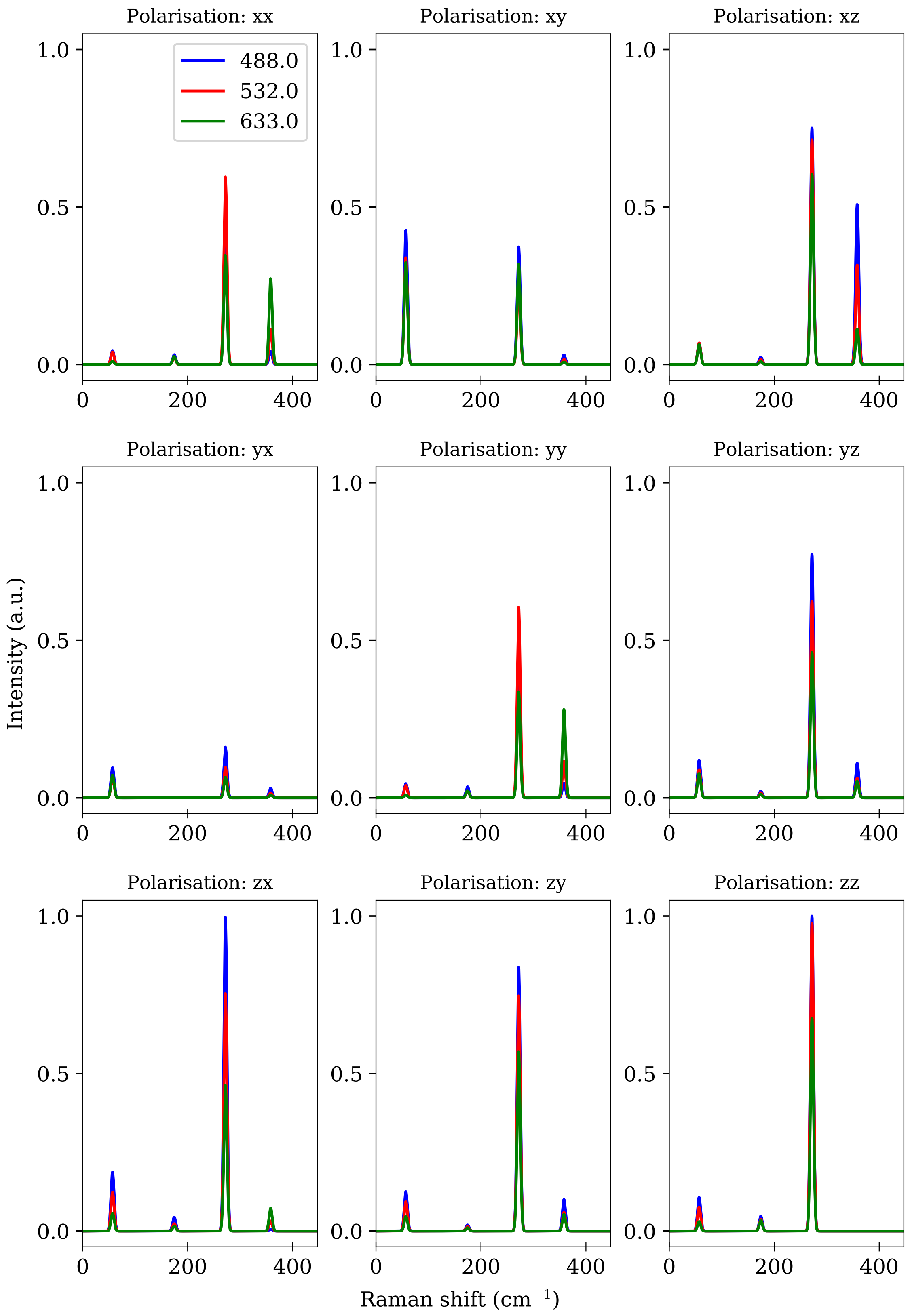

Supplementary Figure 606: Raman spectrum of $TiSe_2$ with space group 115 and Wyckoff positions d-g-0.

$TiSe_2$, 187, b-i-0

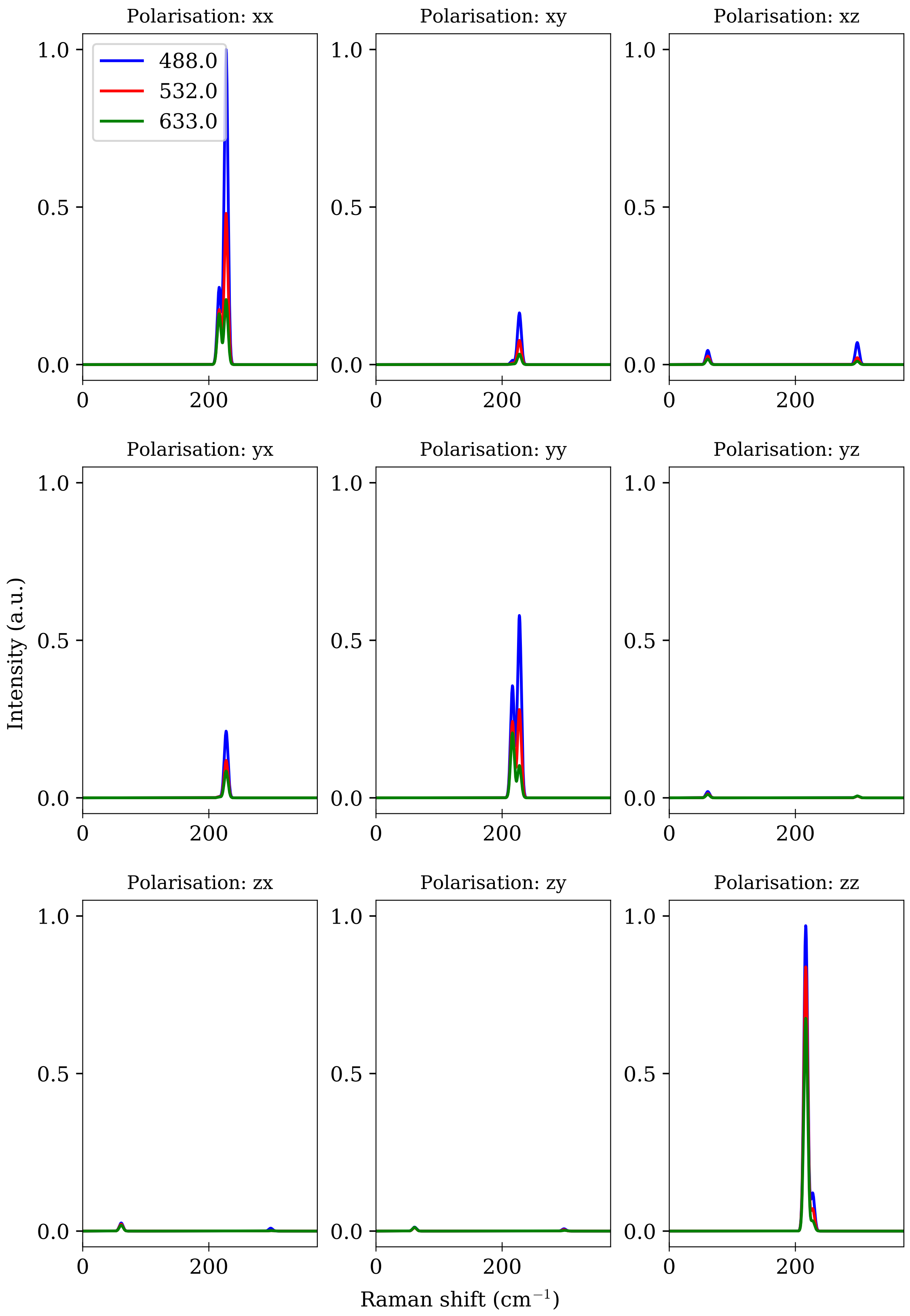

Supplementary Figure 607: Raman spectrum of $TiSe_2$ with space group 187 and Wyckoff positions b-i-0.

TiSeTe, 156, a-b-c-0

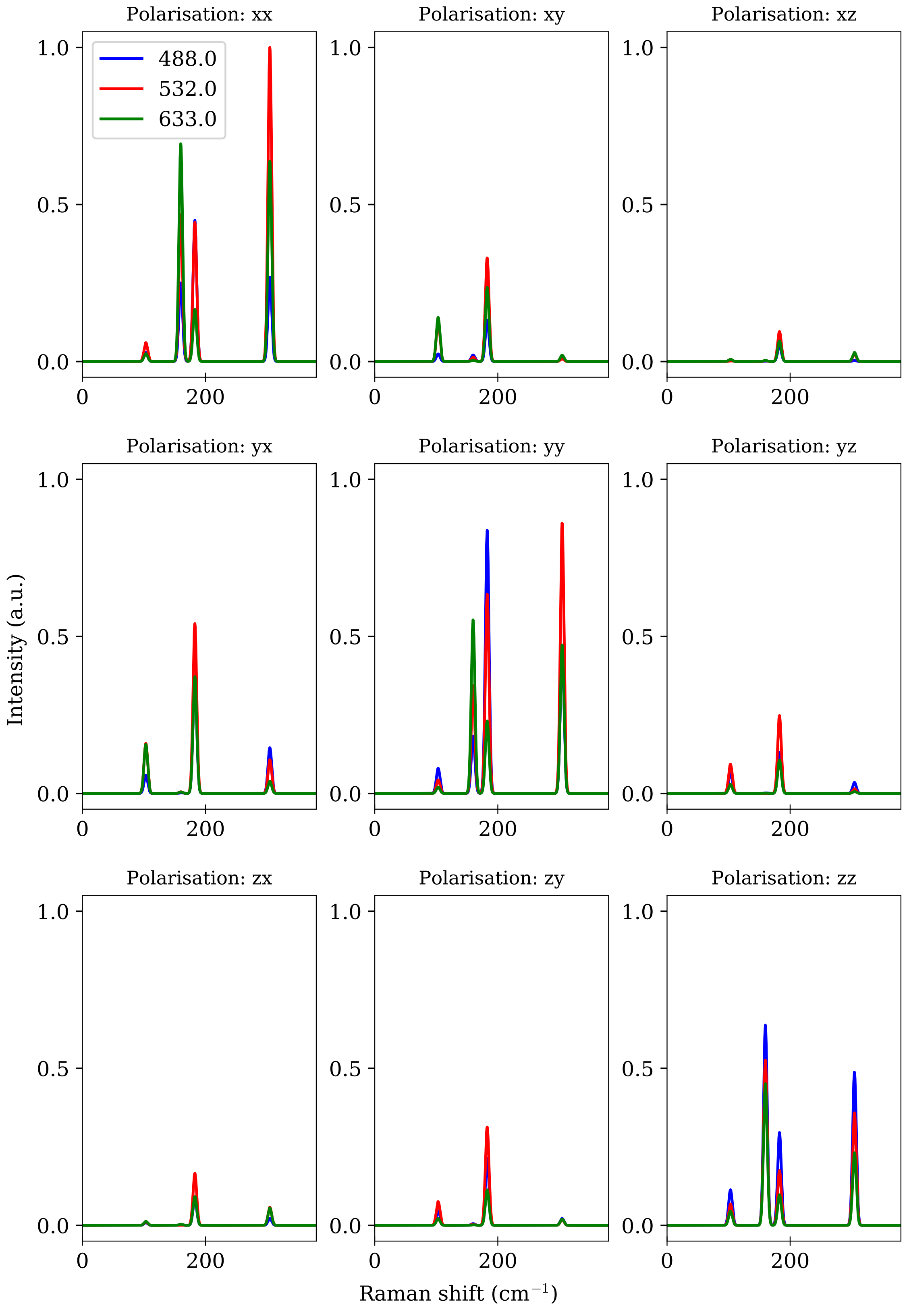

Supplementary Figure 608: Raman spectrum of TiSeTe with space group 156 and Wyckoff positions a-b-c-0.

TiSeTe, 156, a-c-0

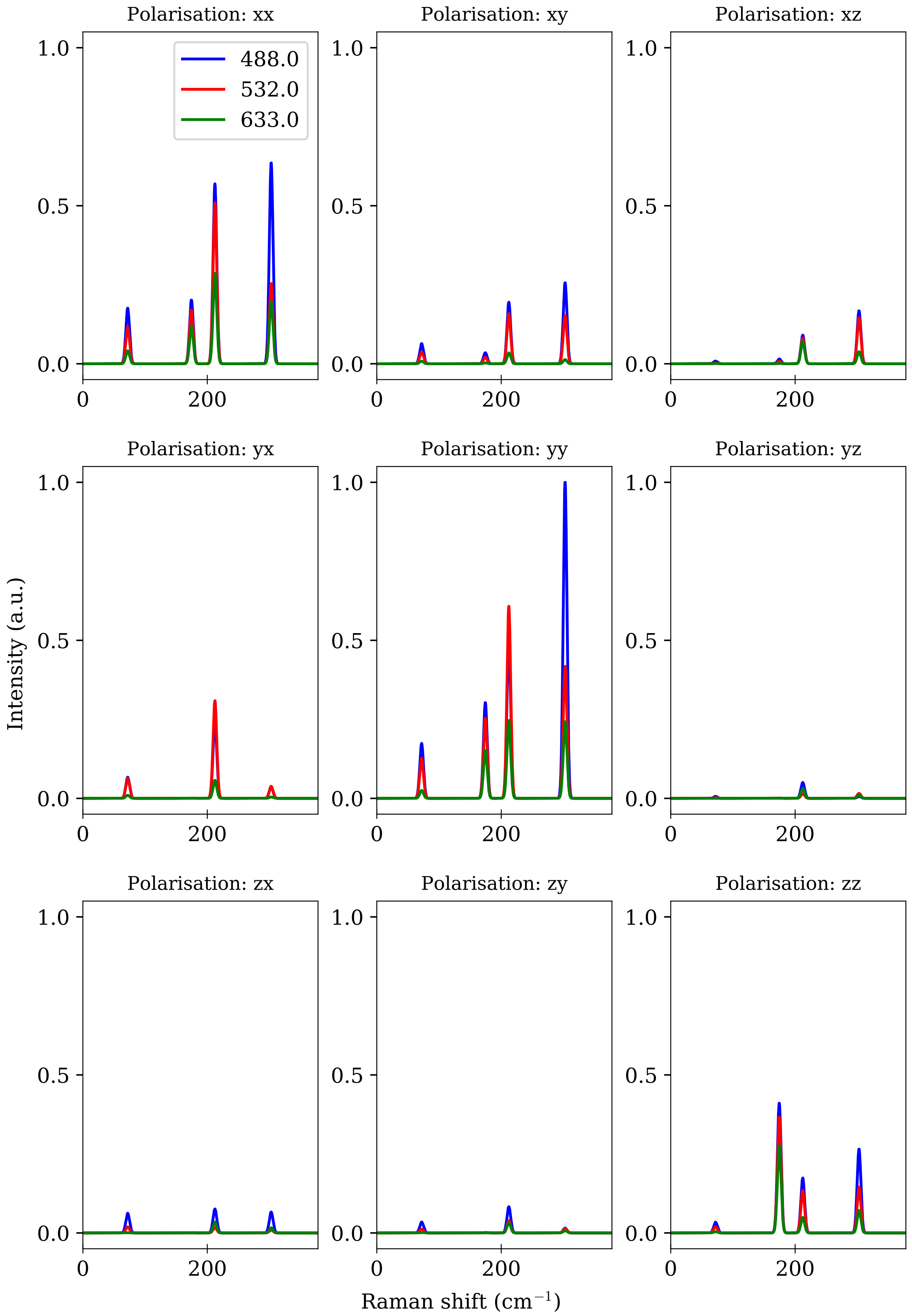

Supplementary Figure 609: Raman spectrum of TiSeTe with space group 156 and Wyckoff positions a-c-0.

TiSSe, 156, a-c-0

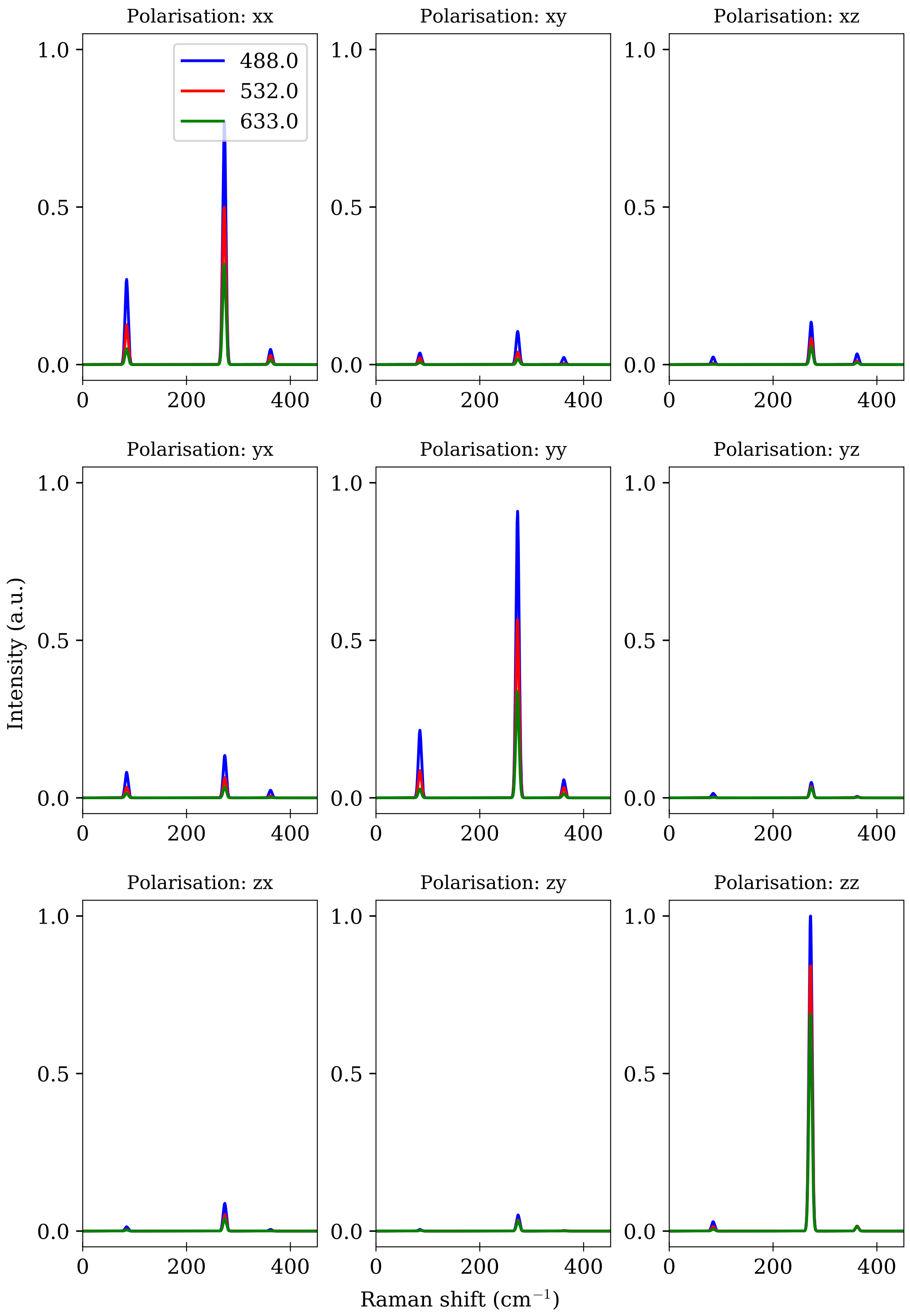

Supplementary Figure 610: Raman spectrum of TiSSe with space group 156 and Wyckoff positions a-c-0.

TiSTe, 156, a-b-c-0

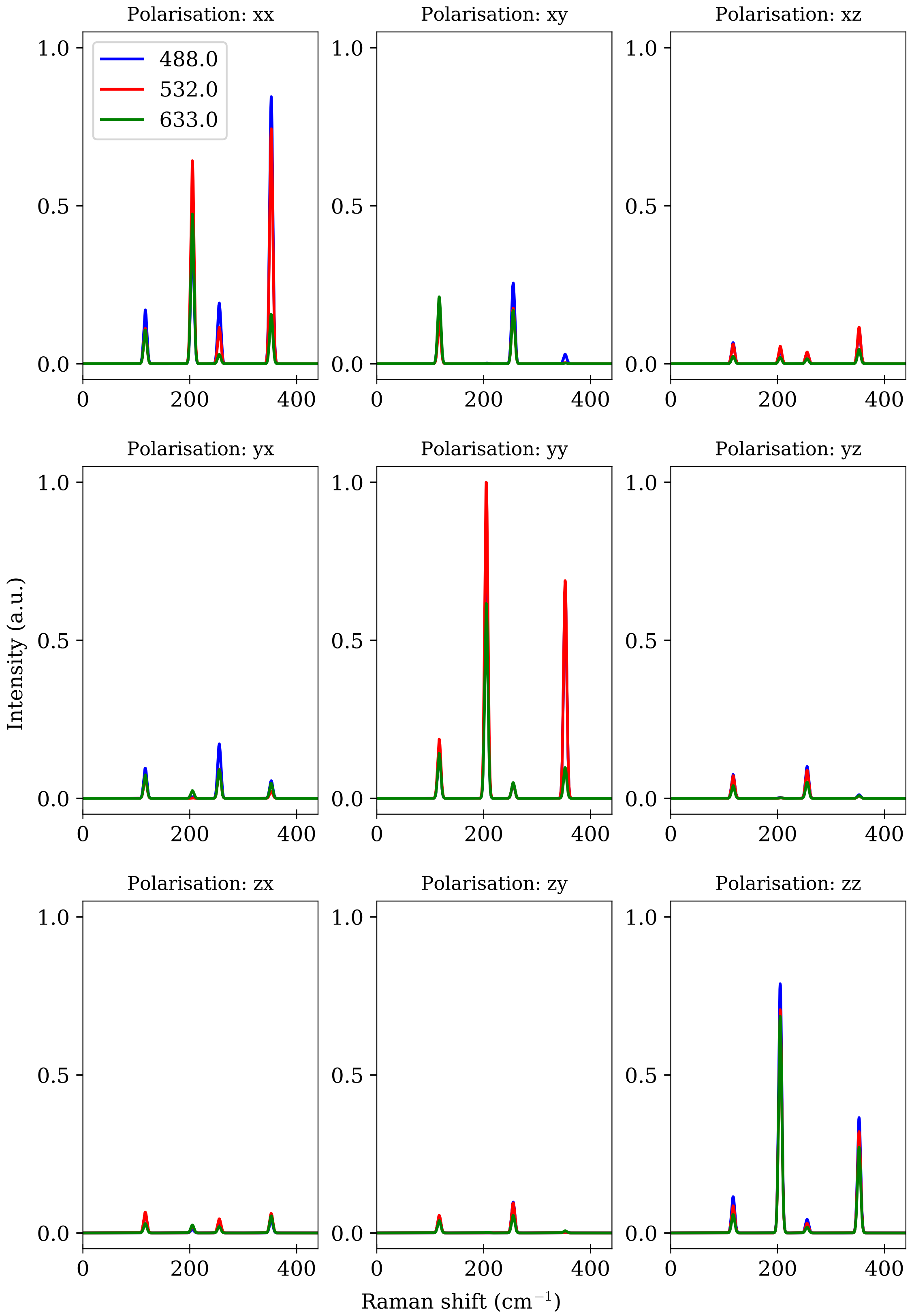

Supplementary Figure 611: Raman spectrum of TiSTe with space group 156 and Wyckoff positions a-b-c-0.

TiSTe, 156, a-c-0

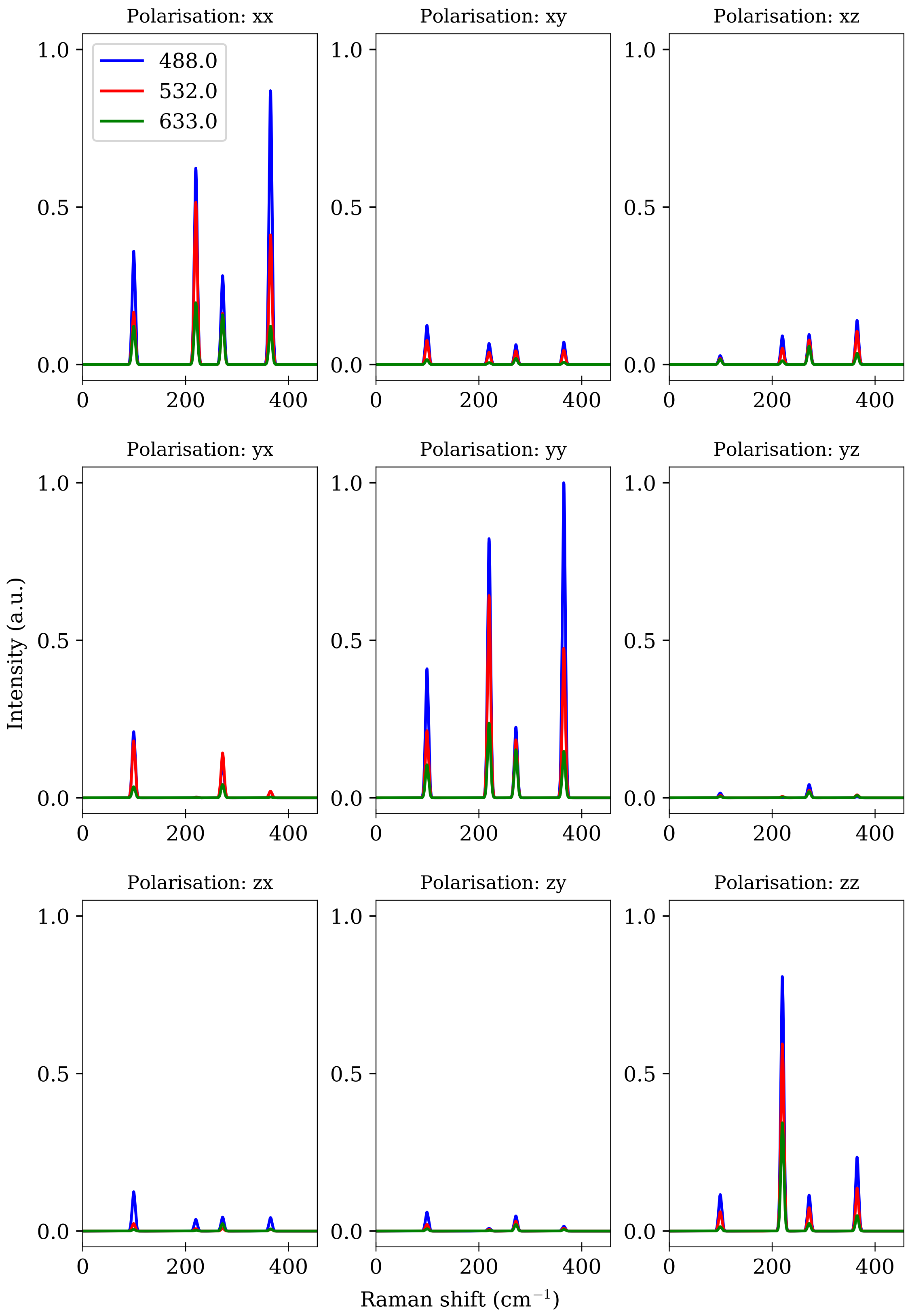

Supplementary Figure 612: Raman spectrum of TiSTe with space group 156 and Wyckoff positions a-c-0.

TiTe$_2$, 164, b-d-0

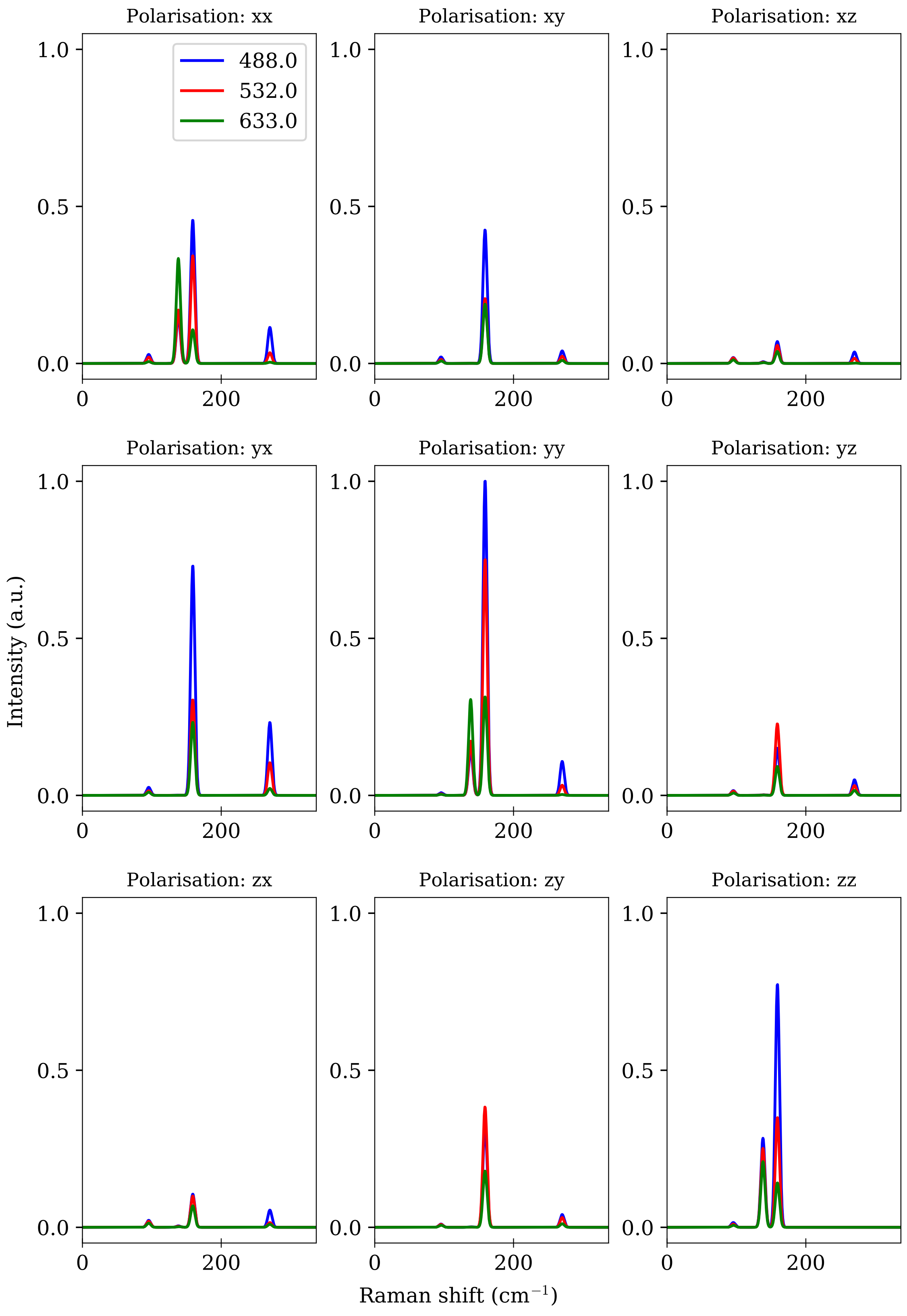

Supplementary Figure 613: Raman spectrum of TiTe$_2$ with space group 164 and Wyckoff positions b-d-0.

$TiTe_2$, 187, b-i-0

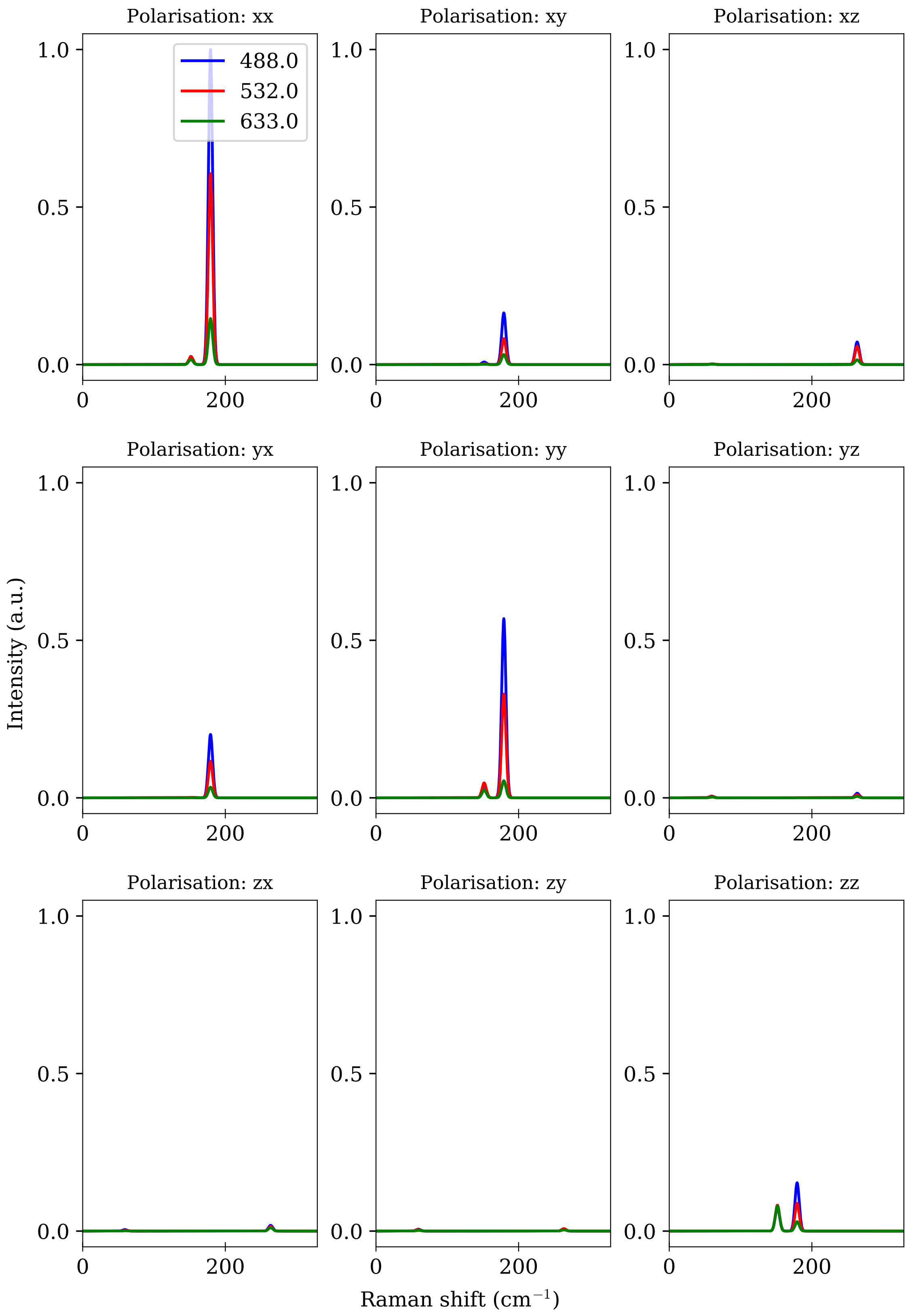

Supplementary Figure 614: Raman spectrum of $TiTe_2$ with space group 187 and Wyckoff positions b-i-0.

V_2CF_2, 164, b-d-0

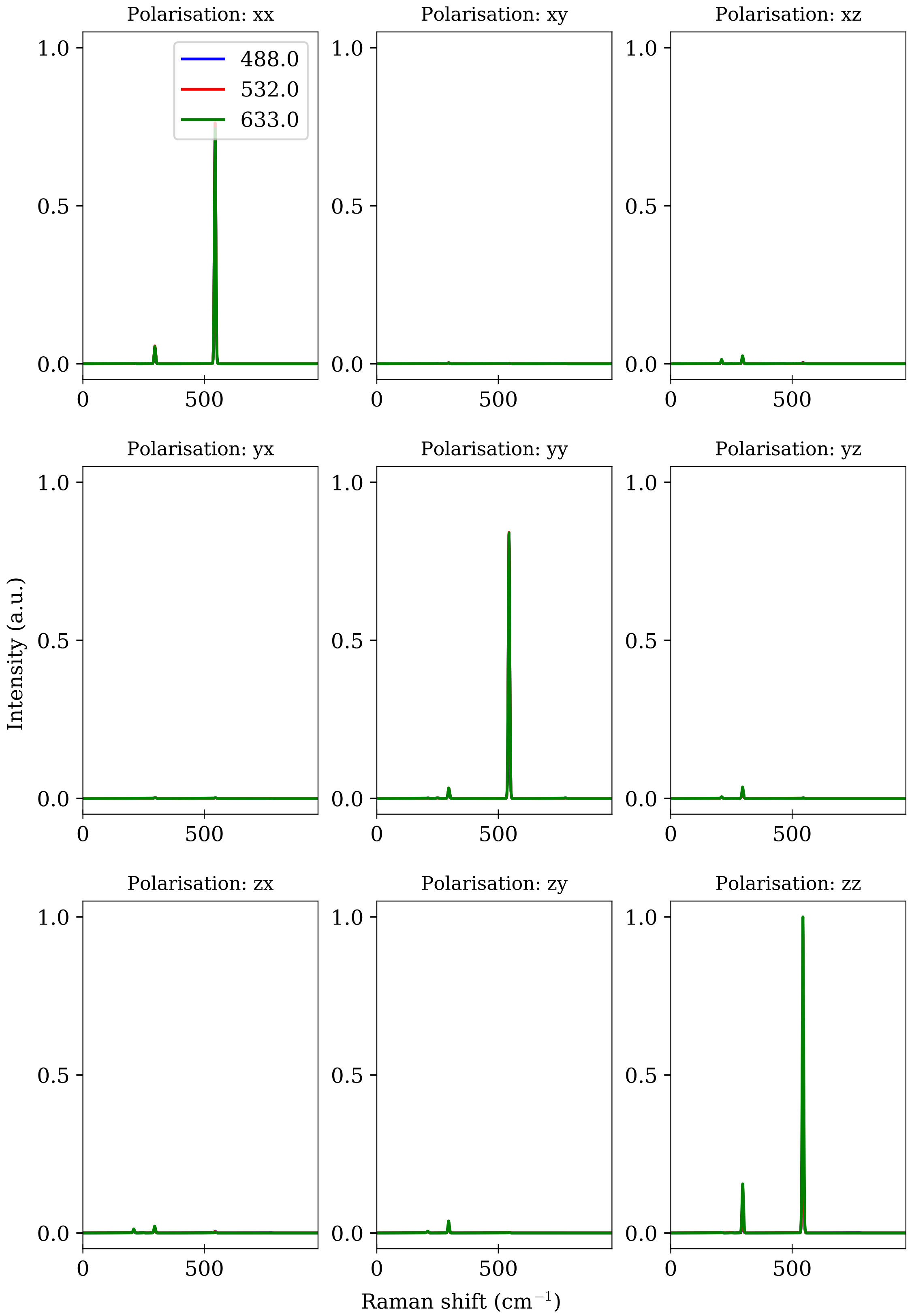

Supplementary Figure 615: Raman spectrum of V_2CF_2 with space group 164 and Wyckoff positions b-d-0.

$V_2CH_2O_2$, 164, b-d-0

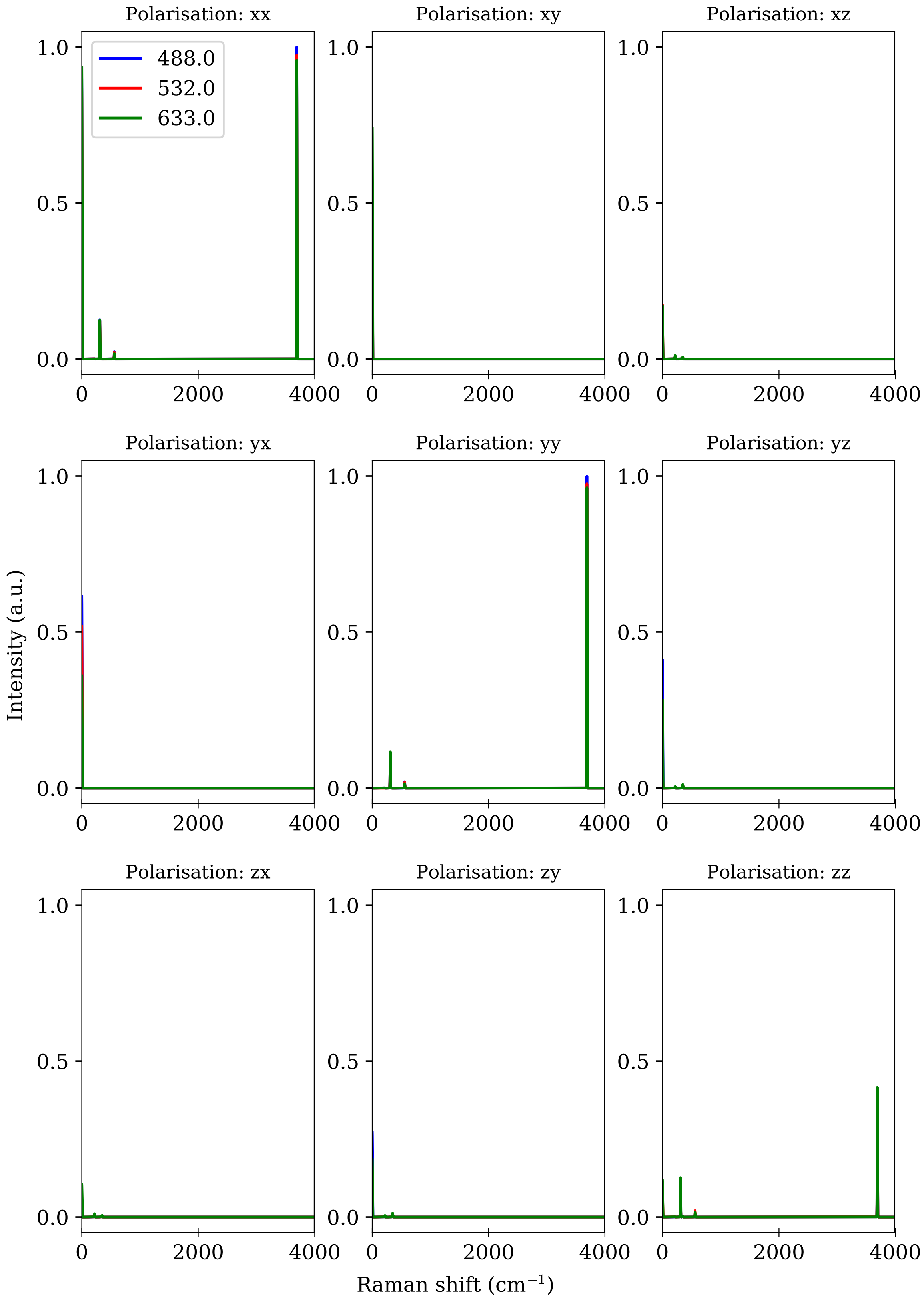

Supplementary Figure 616: Raman spectrum of $V_2CH_2O_2$ with space group 164 and Wyckoff positions b-d-0.

V_2CO_2, 164, b-d-0

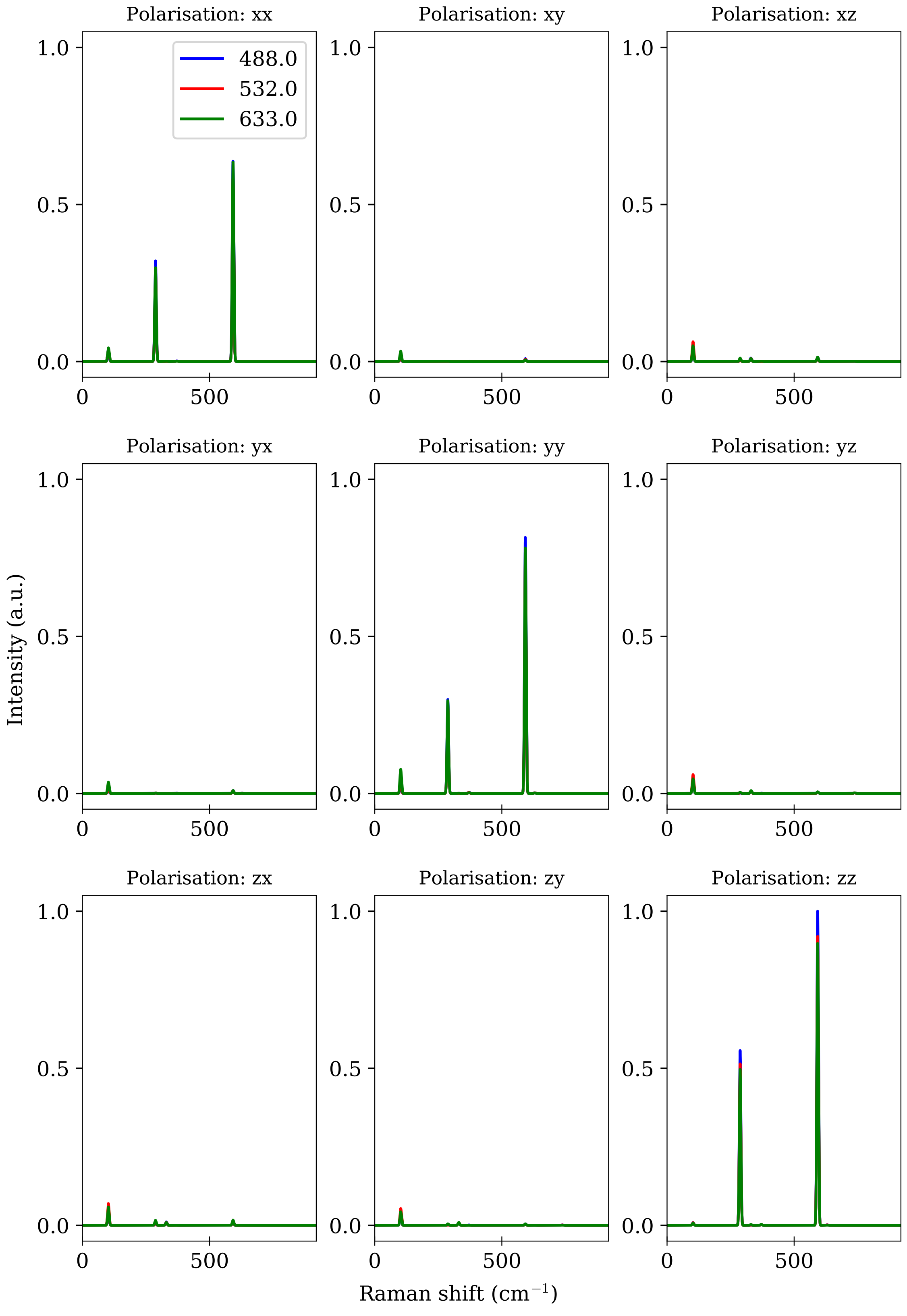

Supplementary Figure 617: Raman spectrum of V_2CO_2 with space group 164 and Wyckoff positions b-d-0.

V_2C, 164, b-d-0

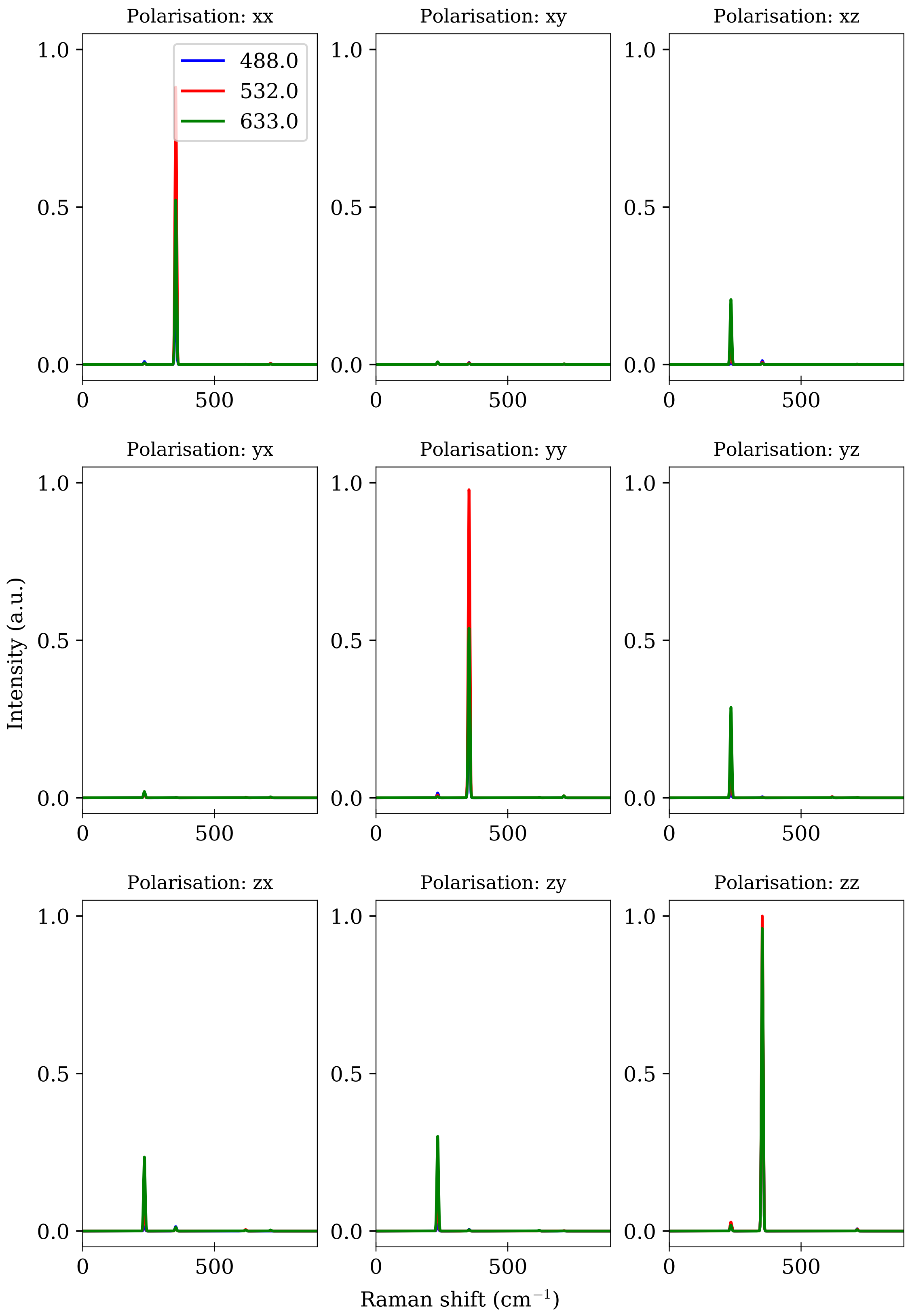

Supplementary Figure 618: Raman spectrum of V_2C with space group 164 and Wyckoff positions b-d-0.

V_2I_2, 129, b-c-0

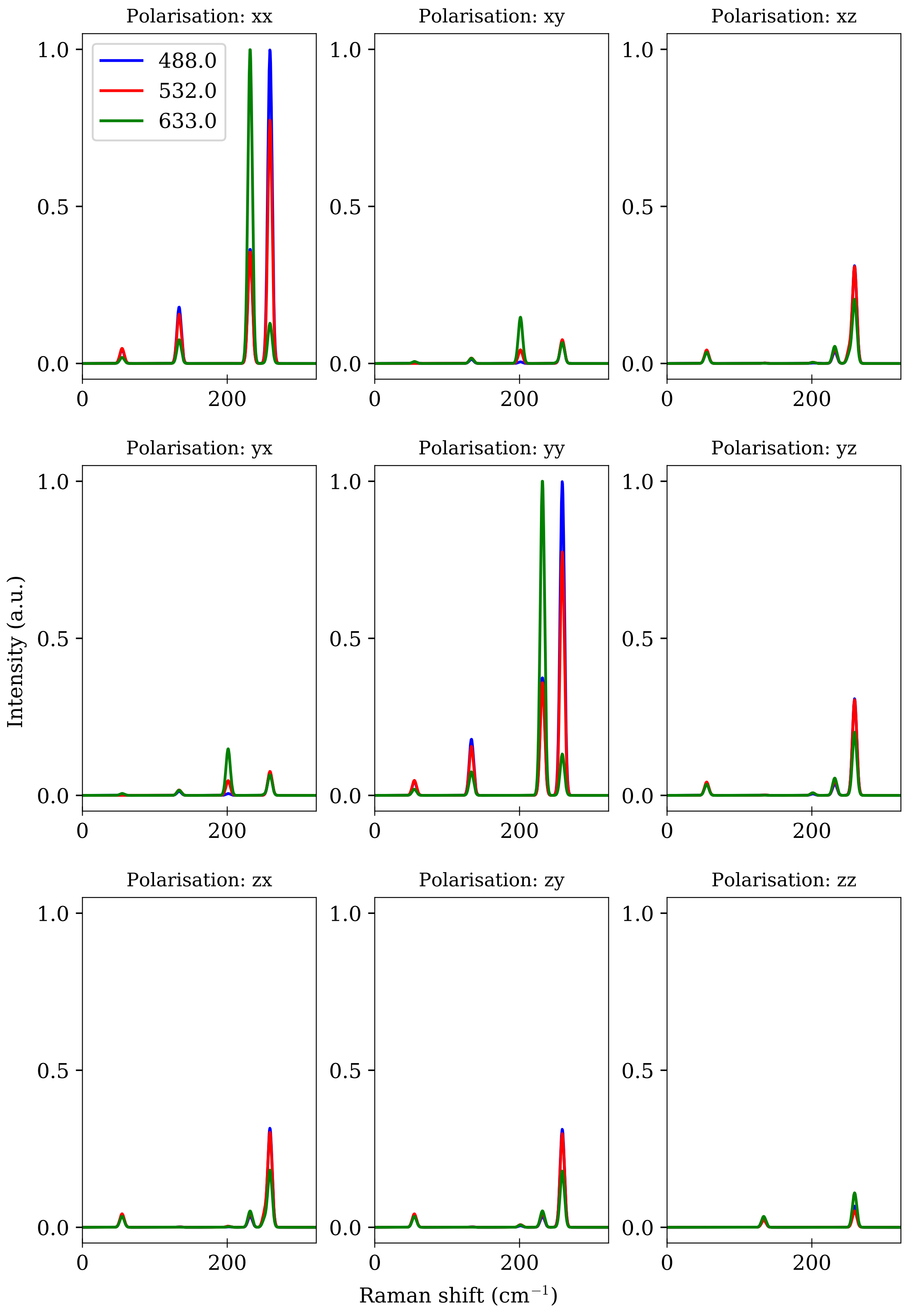

Supplementary Figure 619: Raman spectrum of V_2I_2 with space group 129 and Wyckoff positions b-c-0.

V_2N, 164, b-d-0

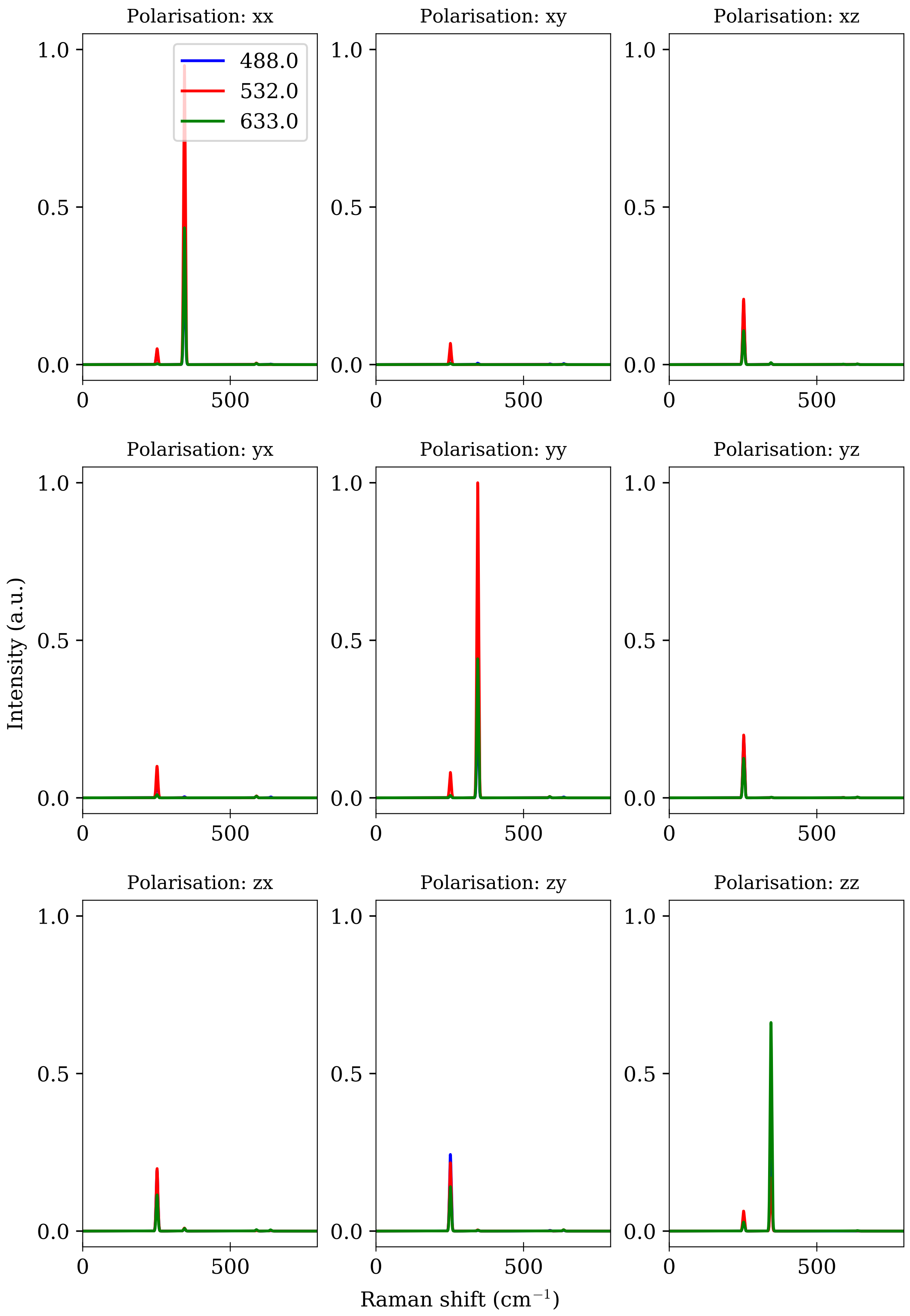

Supplementary Figure 620: Raman spectrum of V_2N with space group 164 and Wyckoff positions b-d-0.

V_2S_2, 164, d-0

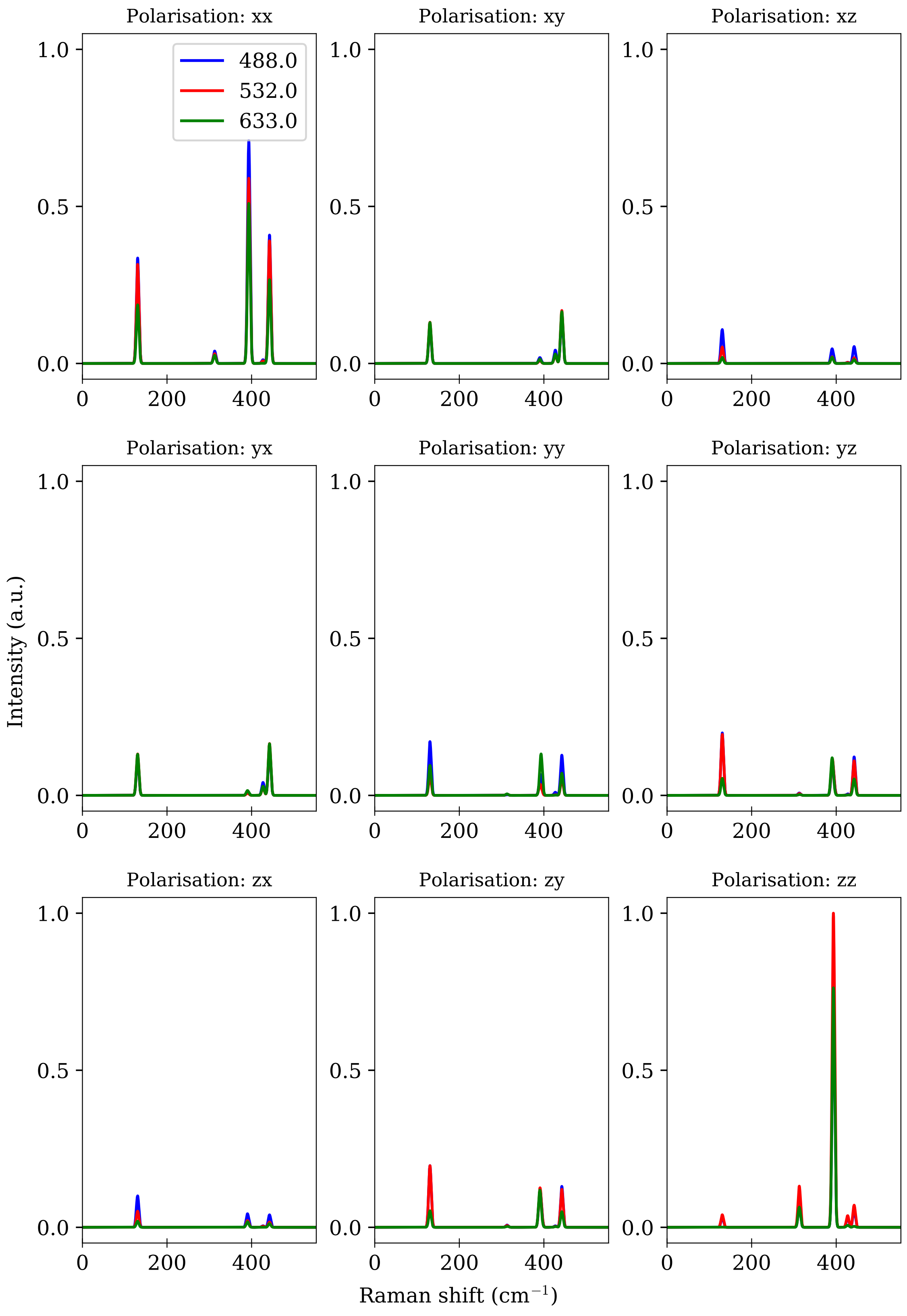

Supplementary Figure 621: Raman spectrum of V_2S_2 with space group 164 and Wyckoff positions d-0.

V_2Se_2, 164, d-0

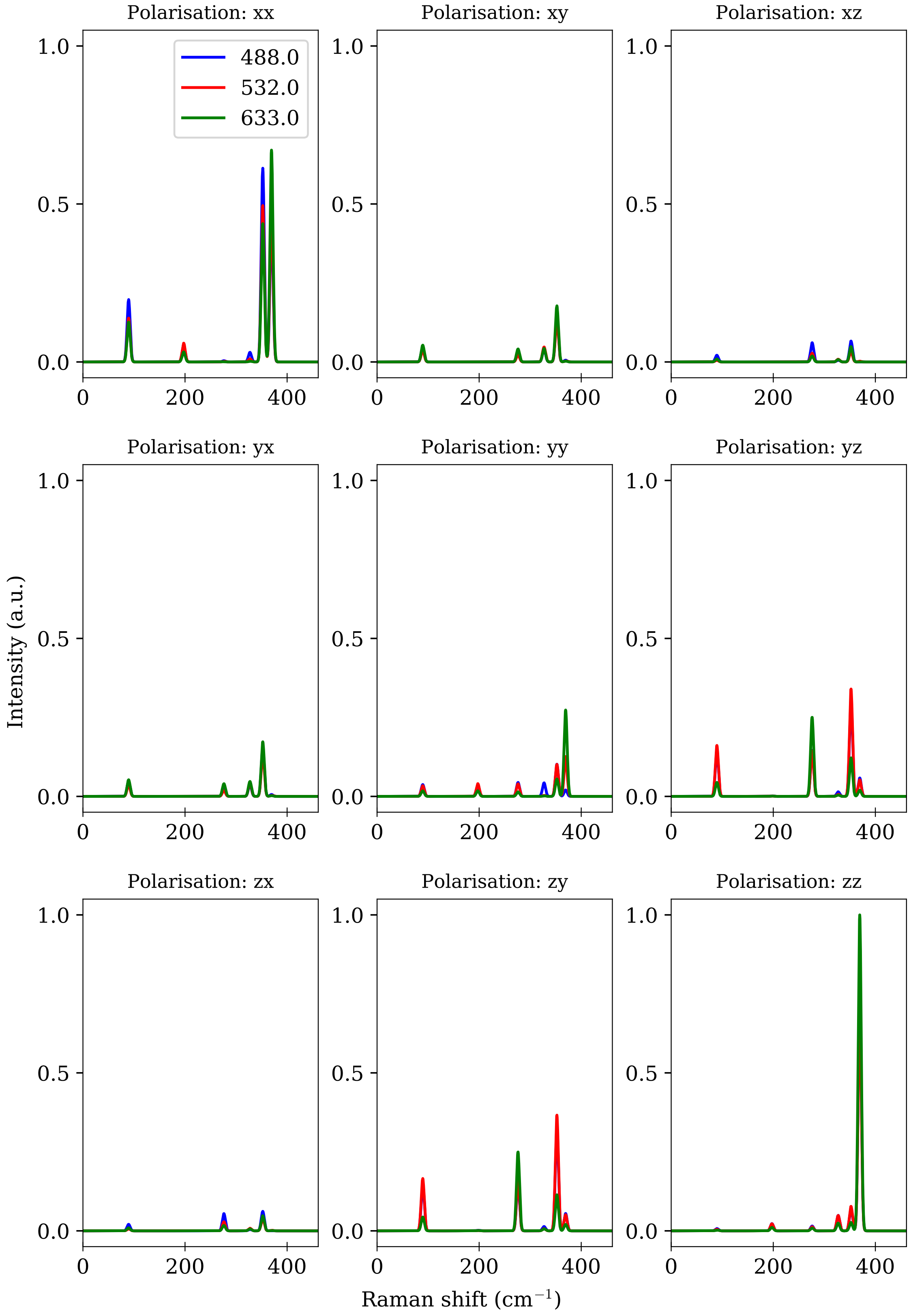

Supplementary Figure 622: Raman spectrum of V_2Se_2 with space group 164 and Wyckoff positions d-0.

V_2Te_2, 164, d-0

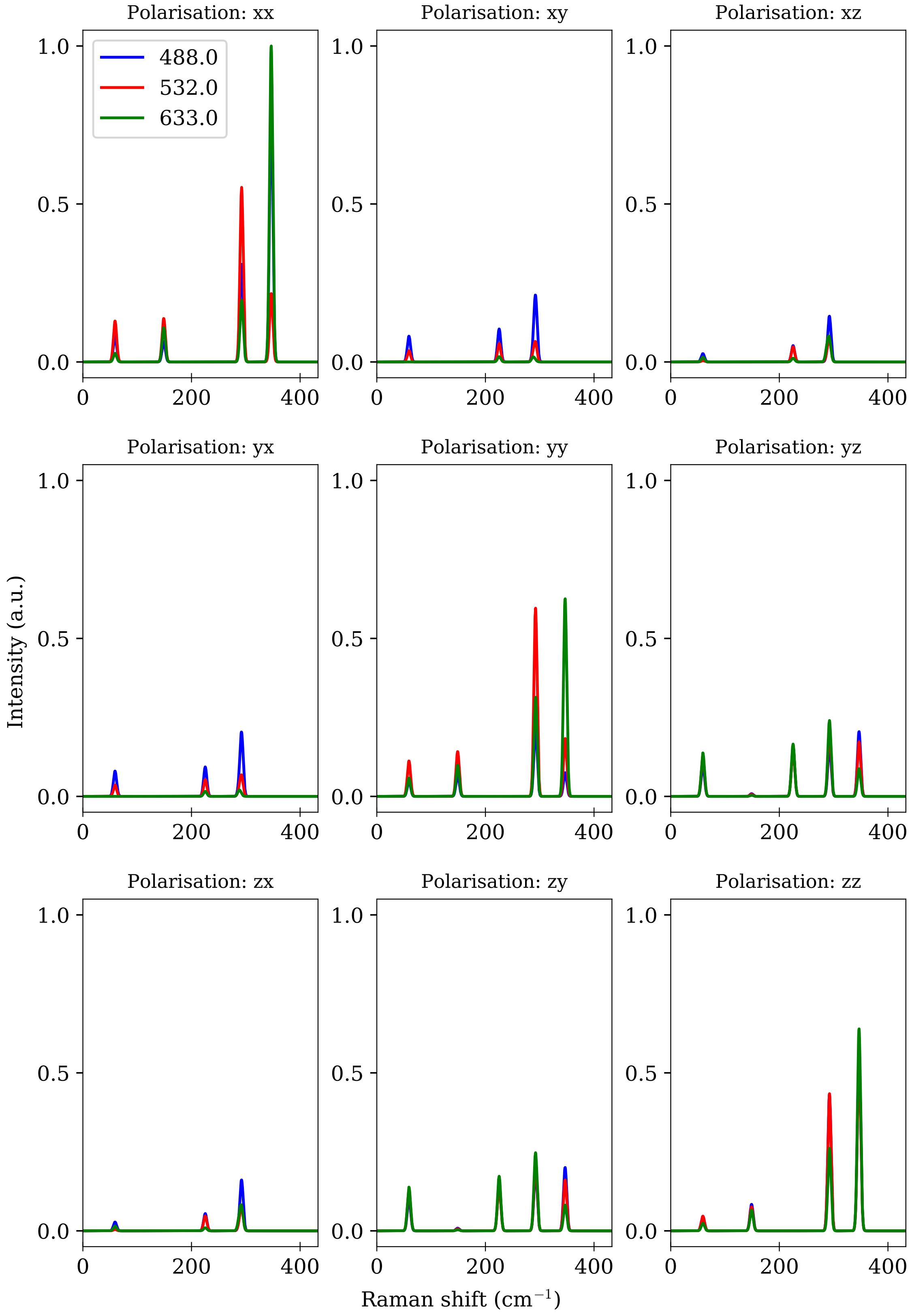

Supplementary Figure 623: Raman spectrum of V_2Te_2 with space group 164 and Wyckoff positions d-0.

V_3C_2, 187, b-h-i-0

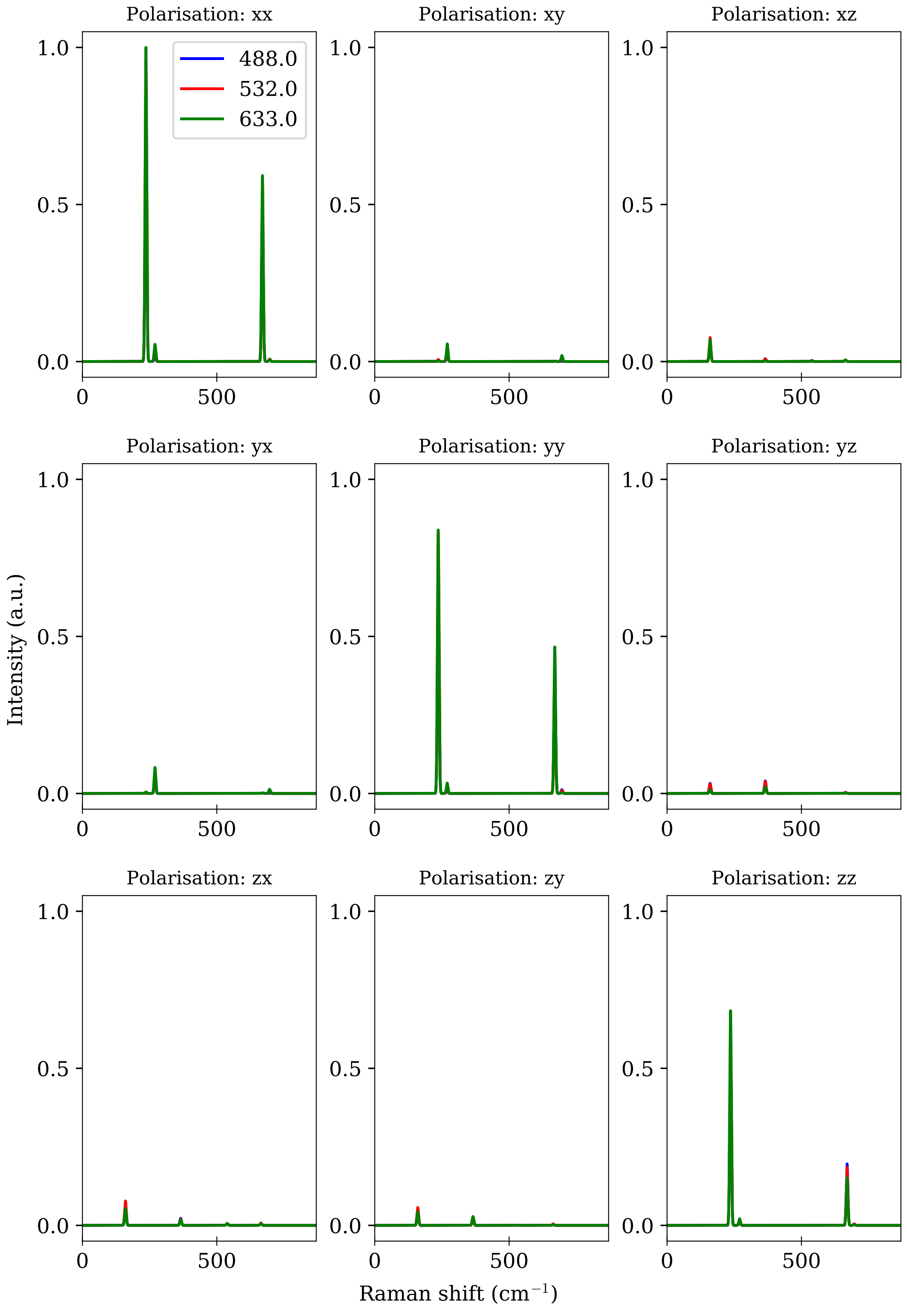

Supplementary Figure 624: Raman spectrum of V_3C_2 with space group 187 and Wyckoff positions b-h-i-0.

V_3N_2, 187, b-h-i-0

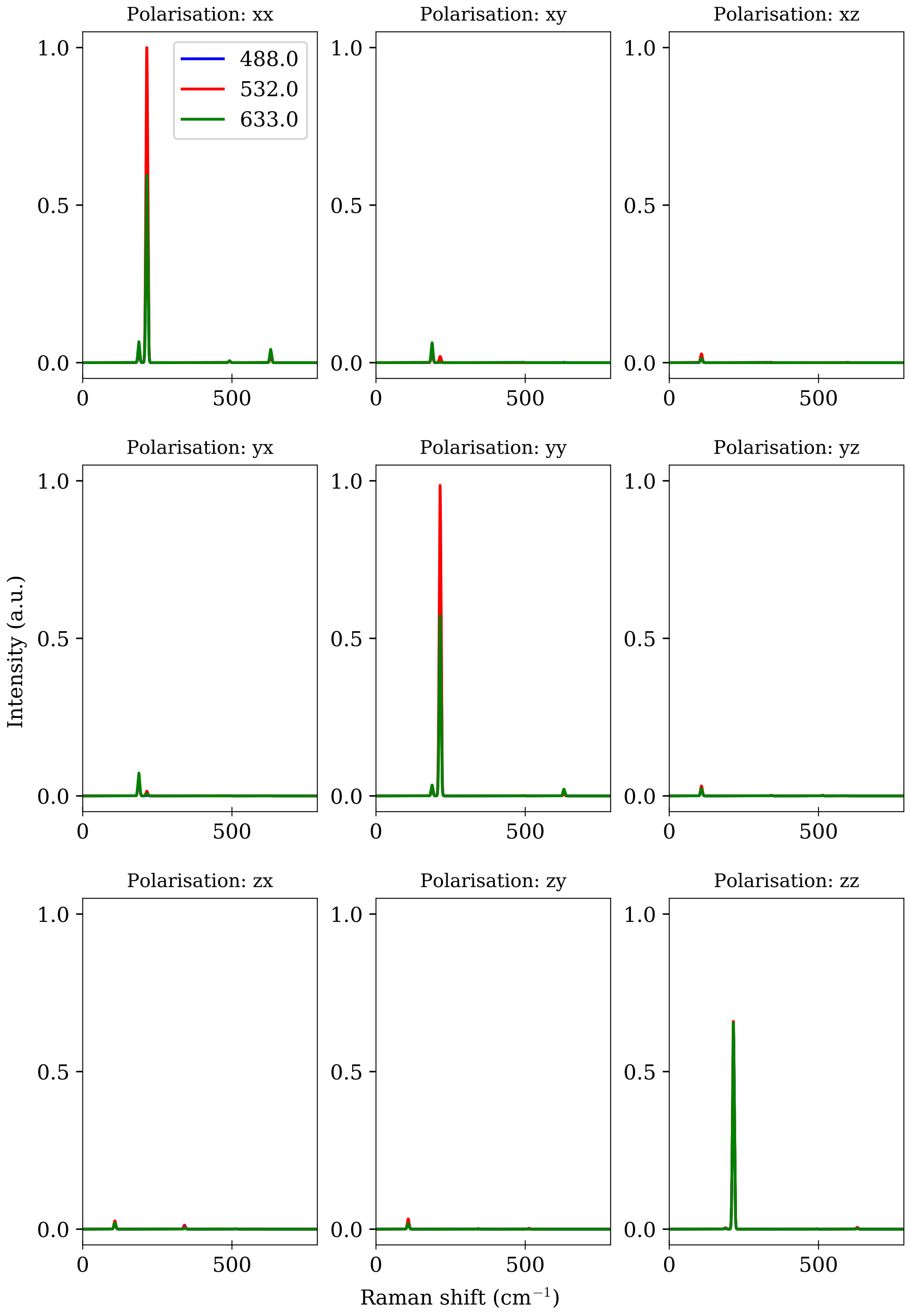

Supplementary Figure 625: Raman spectrum of V_3N_2 with space group 187 and Wyckoff positions b-h-i-0.

VAs_2, 164, b-d-0

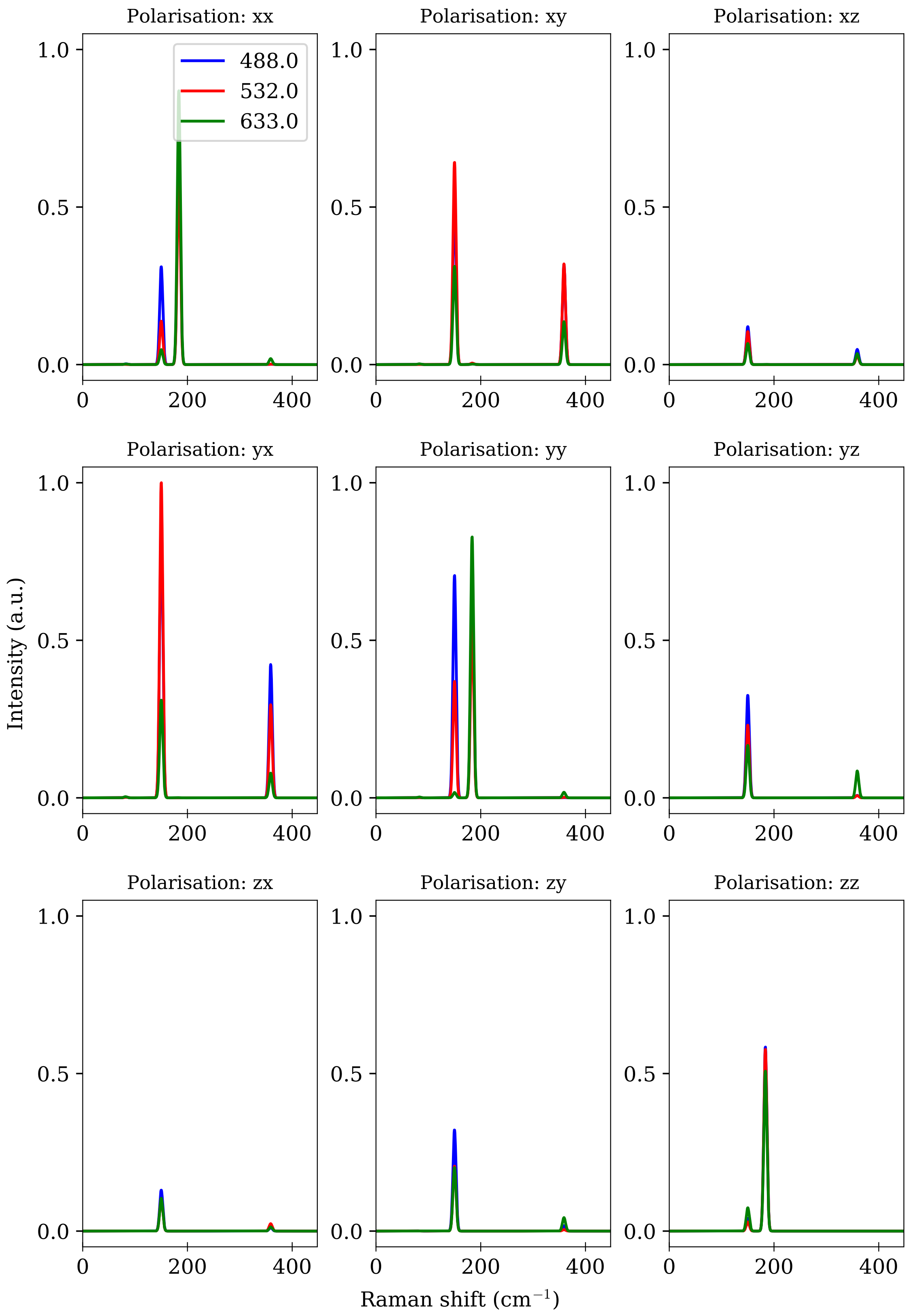

Supplementary Figure 626: Raman spectrum of VAs_2 with space group 164 and Wyckoff positions b-d-0.

VAs_2, 187, b-i-0

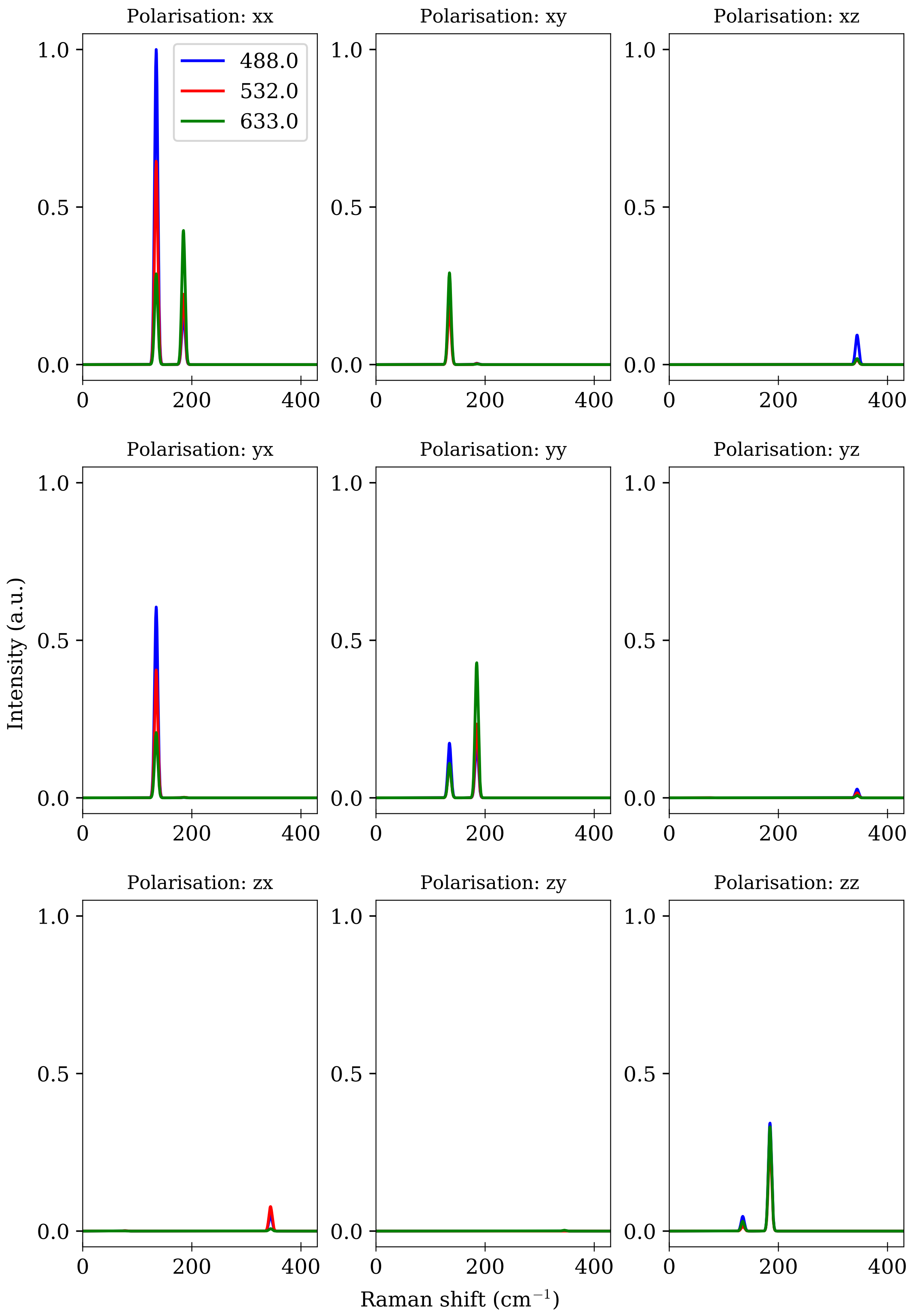

Supplementary Figure 627: Raman spectrum of VAs_2 with space group 187 and Wyckoff positions b-i-0.

VAsSb, 156, a-b-c-0

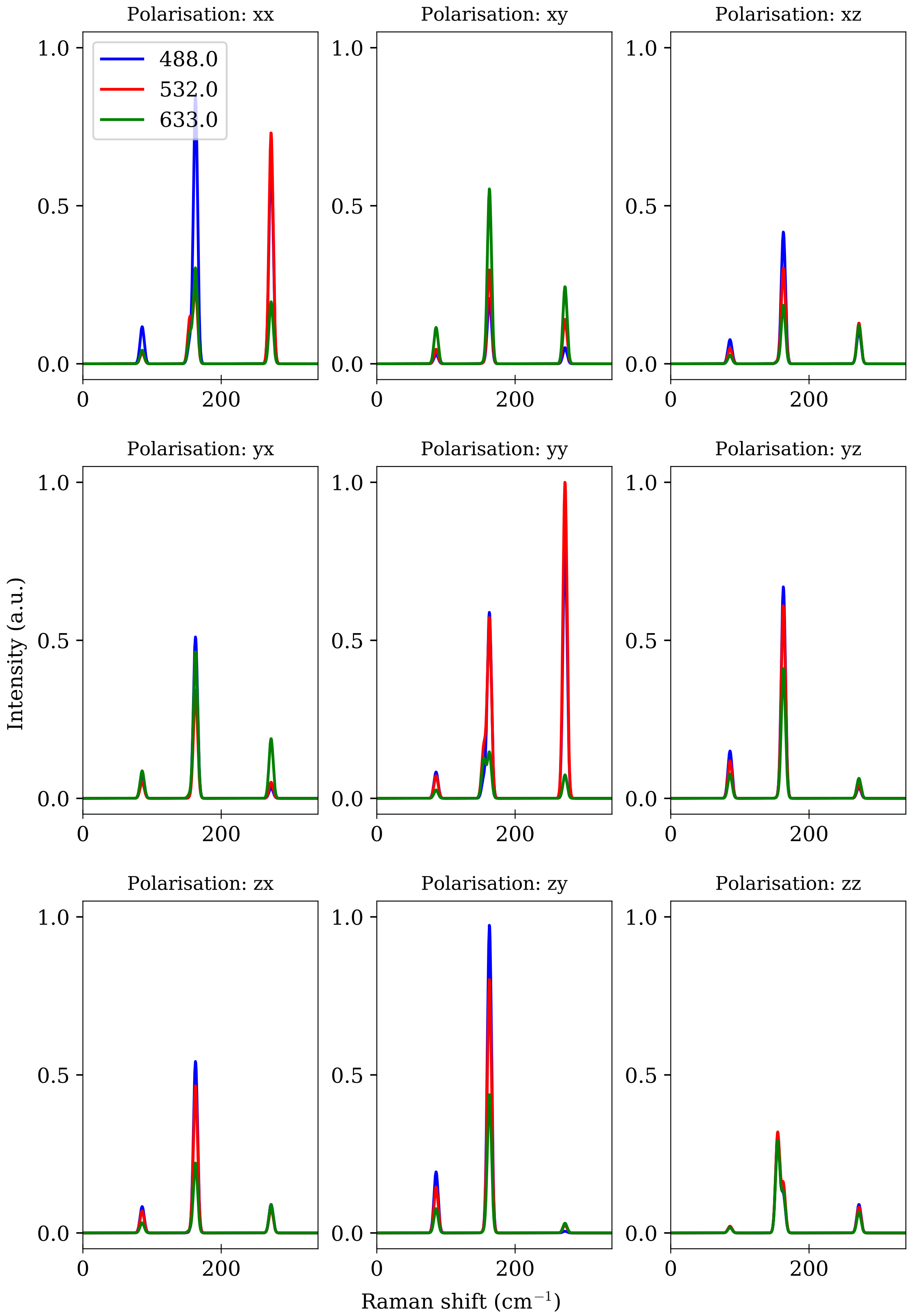

Supplementary Figure 628: Raman spectrum of VAsSb with space group 156 and Wyckoff positions a-b-c-0.

VAsSb, 156, a-c-0

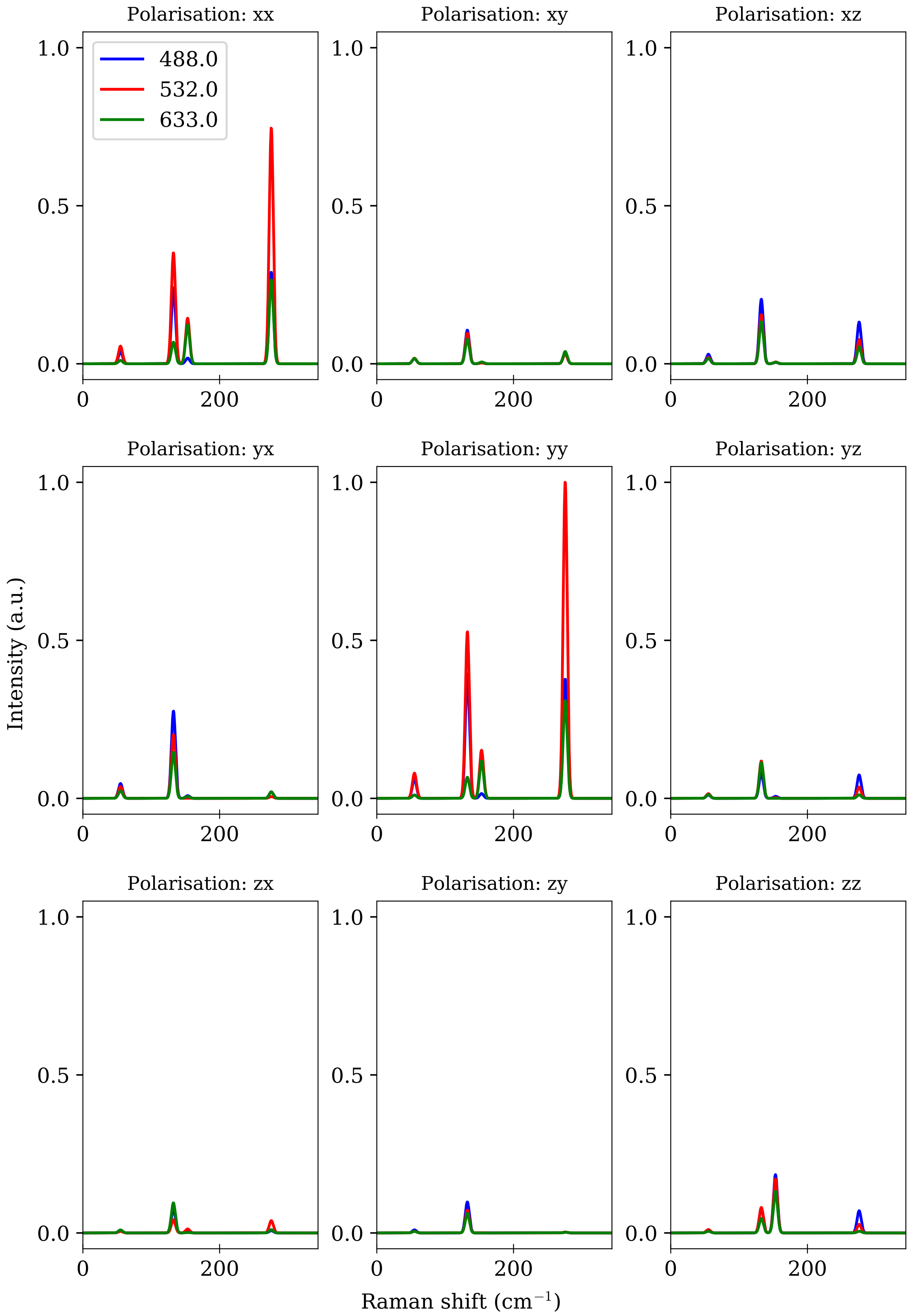

Supplementary Figure 629: Raman spectrum of VAsSb with space group 156 and Wyckoff positions a-c-0.

VH_2, 187, a-i-0

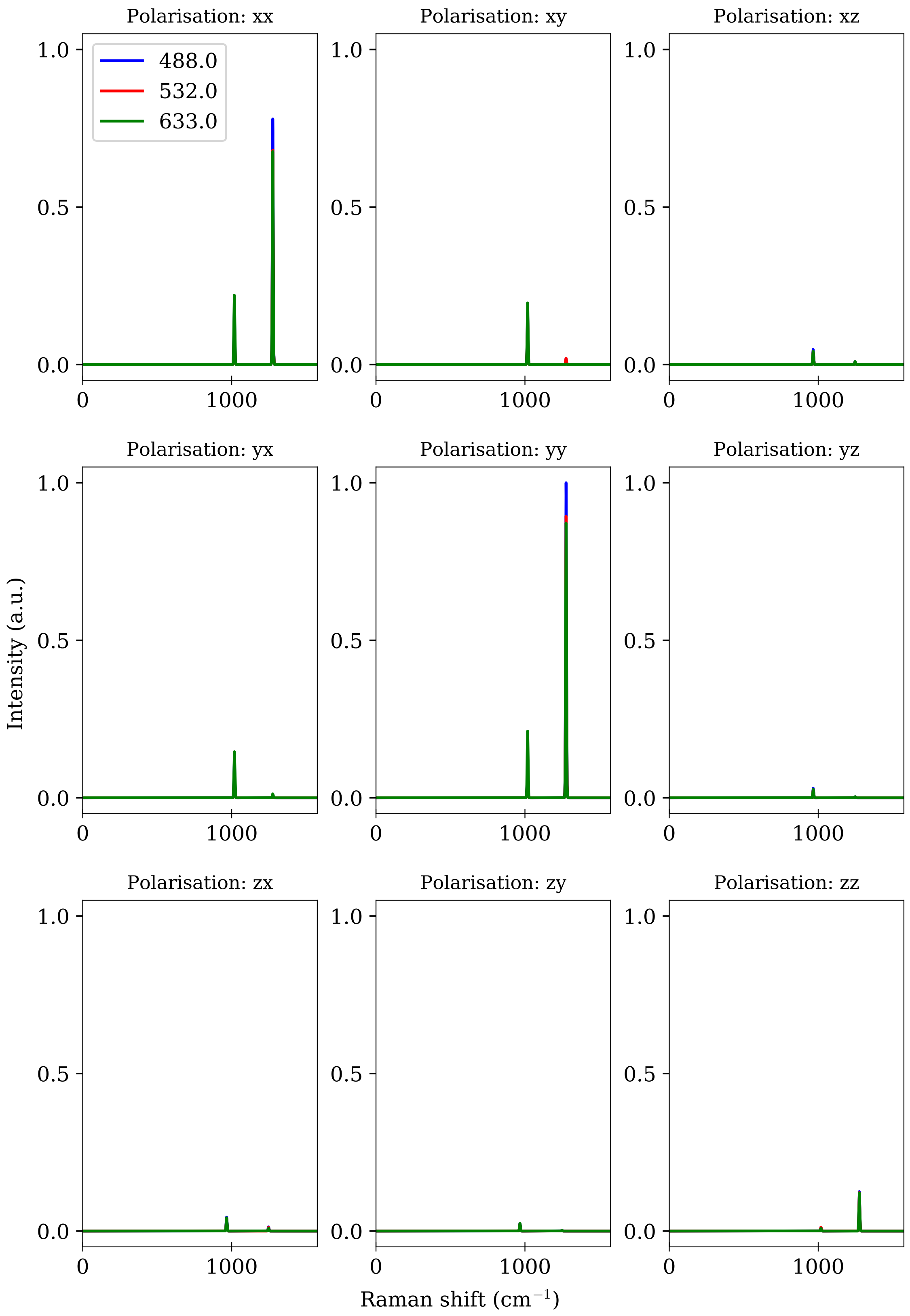

Supplementary Figure 630: Raman spectrum of VH_2 with space group 187 and Wyckoff positions a-i-0.

VS_2, 164, b-d-0

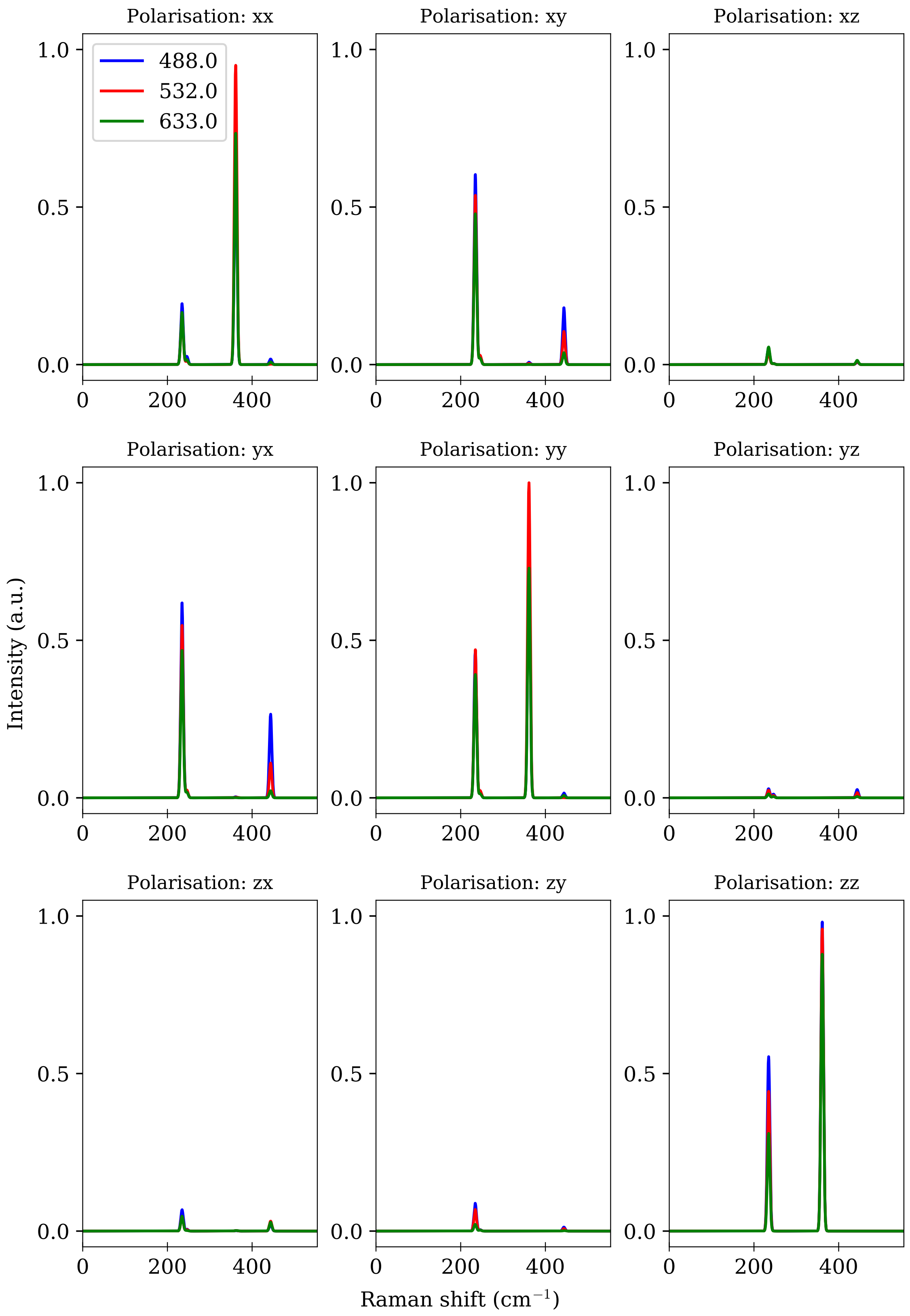

Supplementary Figure 631: Raman spectrum of VS_2 with space group 164 and Wyckoff positions b-d-0.

VSb_2, 164, b-d-0

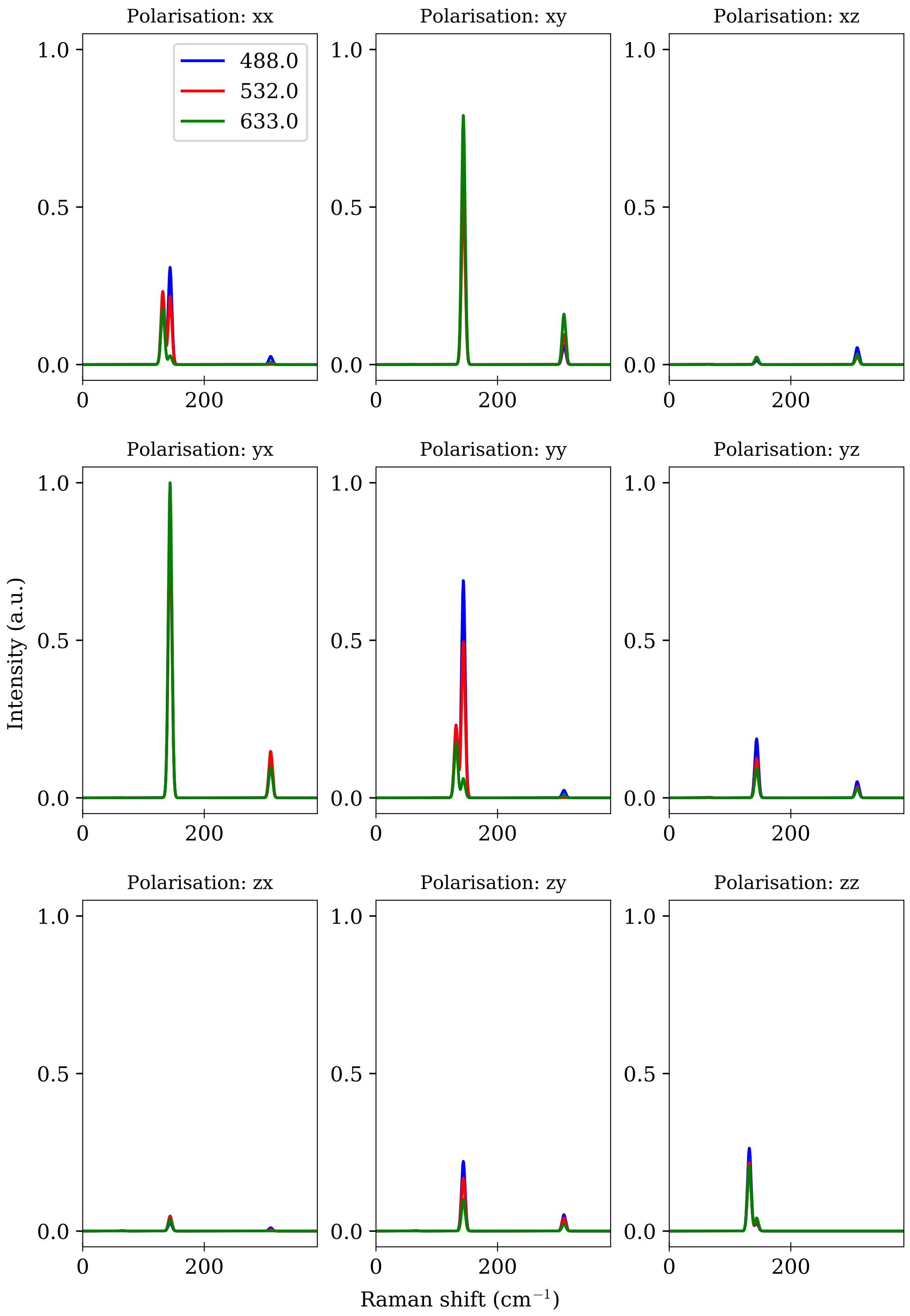

Supplementary Figure 632: Raman spectrum of VSb_2 with space group 164 and Wyckoff positions b-d-0.

VSb_2, 187, a-i-0

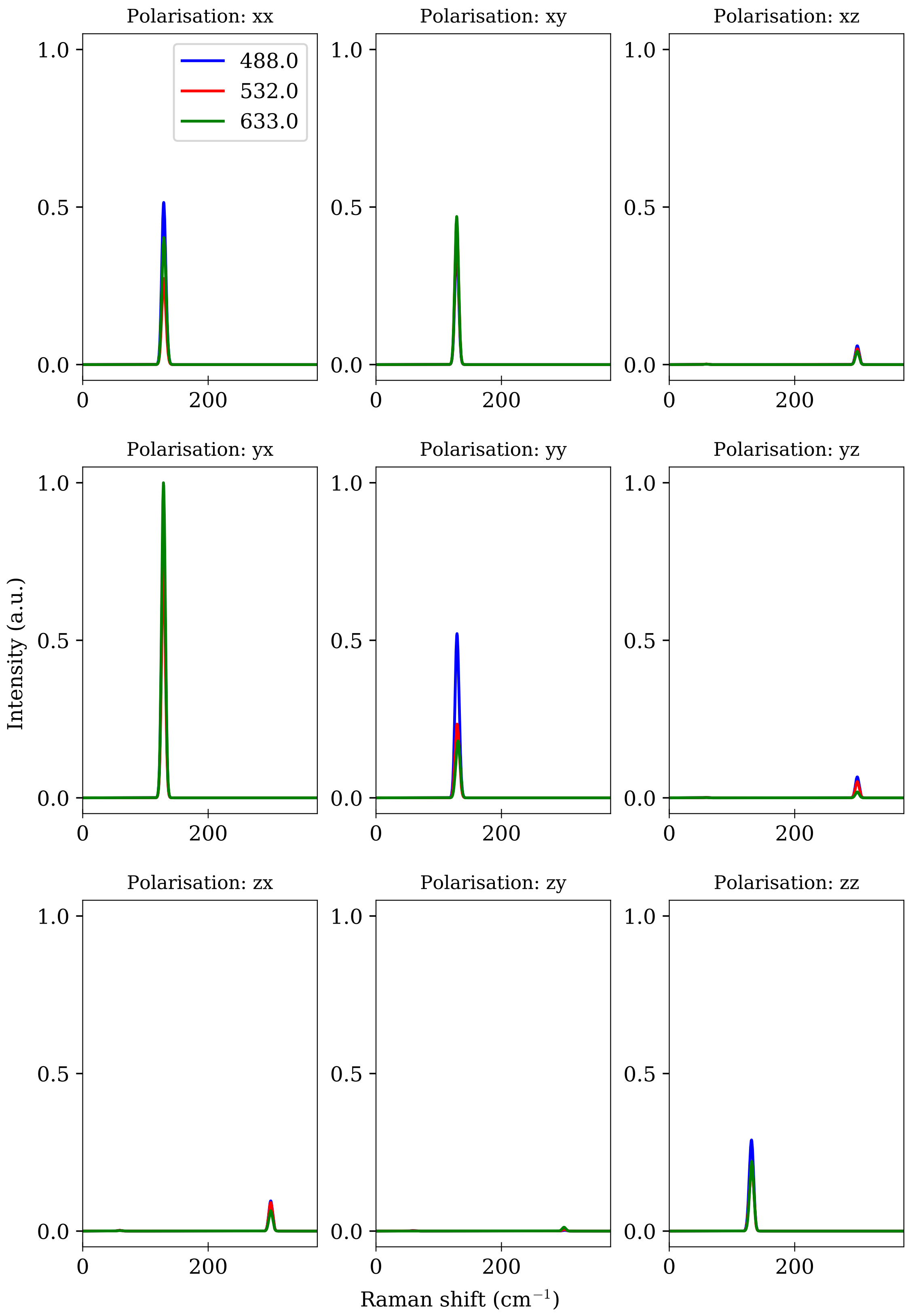

Supplementary Figure 633: Raman spectrum of VSb_2 with space group 187 and Wyckoff positions a-i-0.

VSe_2, 164, b-d-0

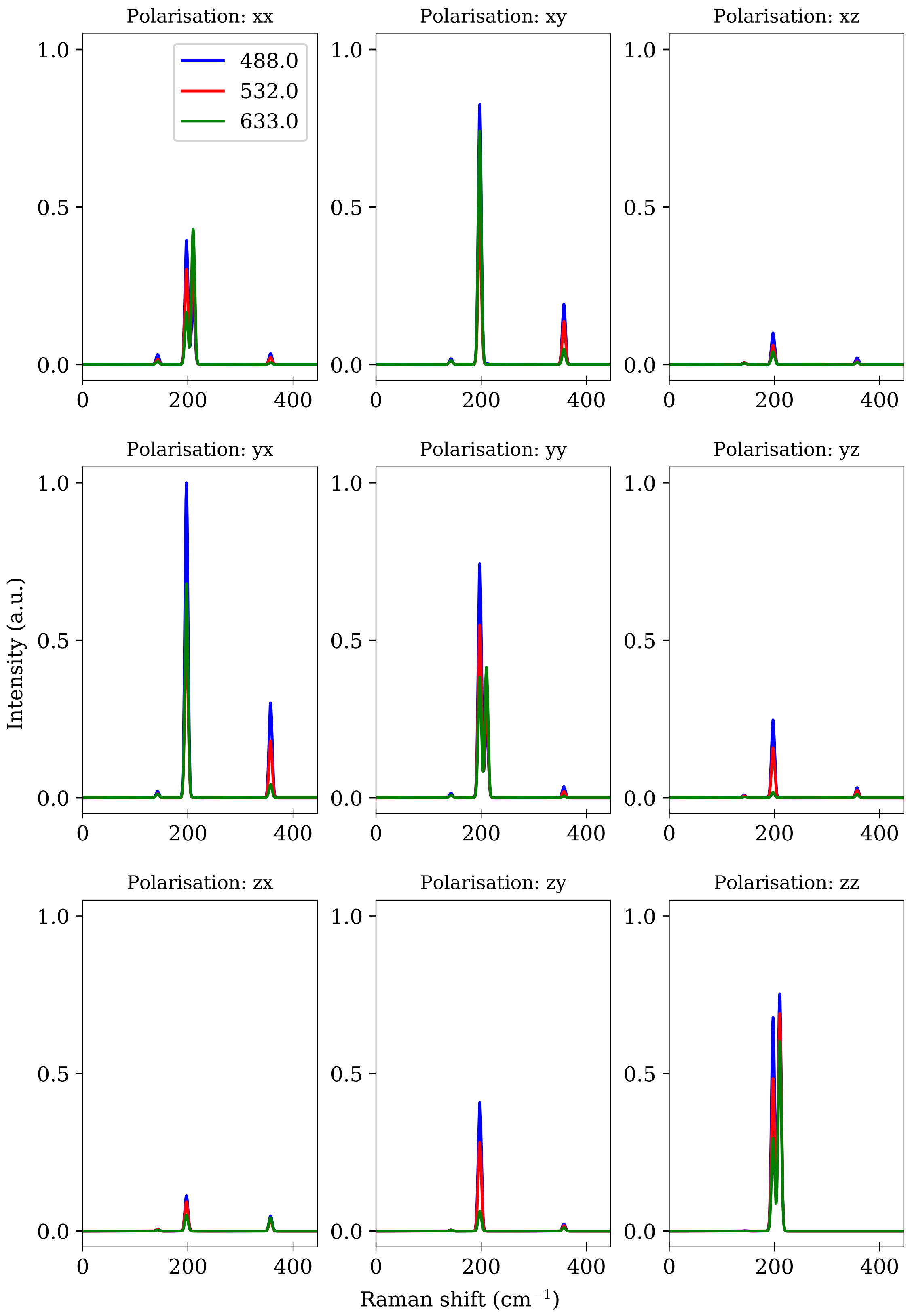

Supplementary Figure 634: Raman spectrum of VSe_2 with space group 164 and Wyckoff positions b-d-0.

VSSe, 156, a-b-c-0

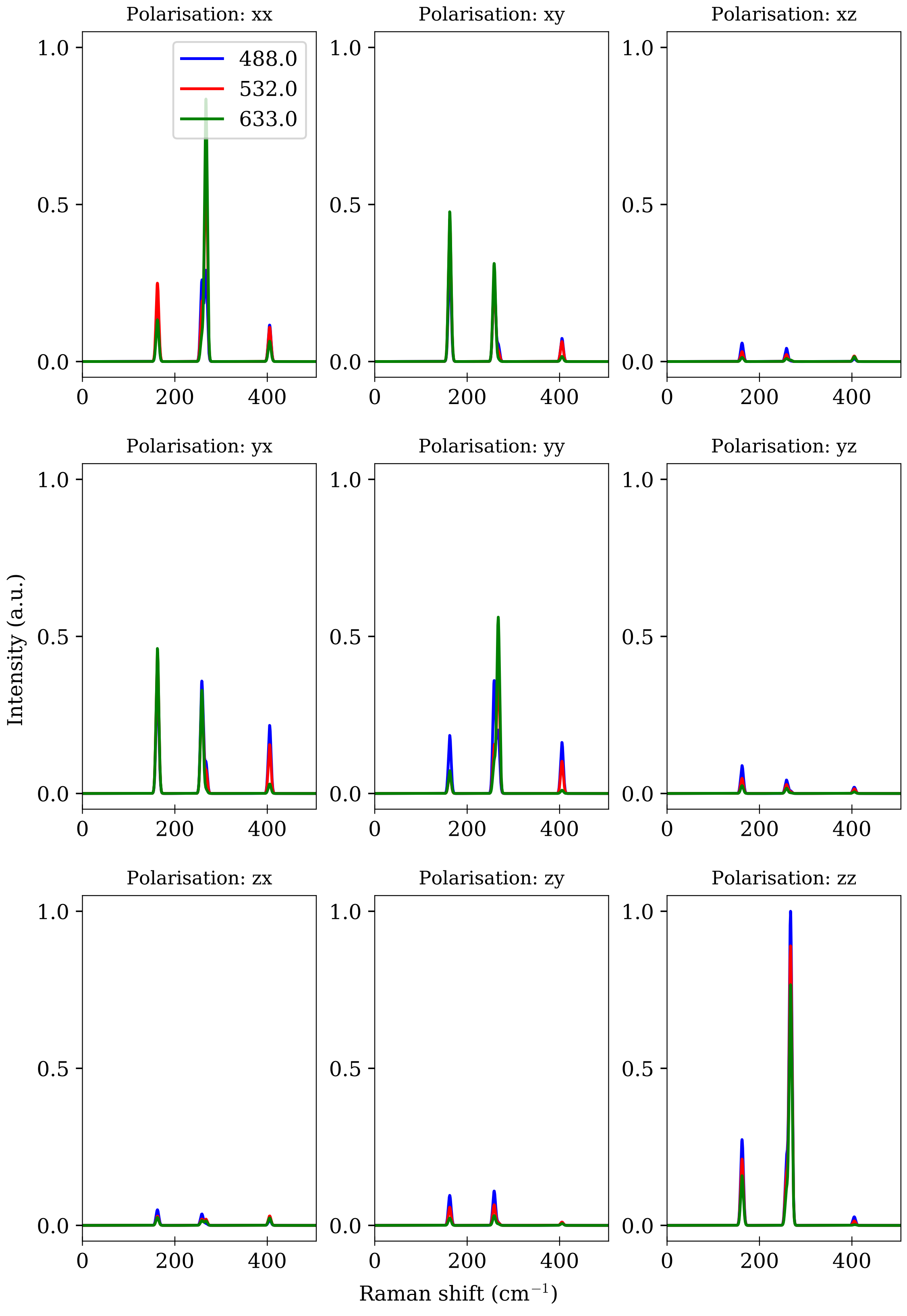

Supplementary Figure 635: Raman spectrum of VSSe with space group 156 and Wyckoff positions a-b-c-0.

W_2Cl_2, 12, i-0

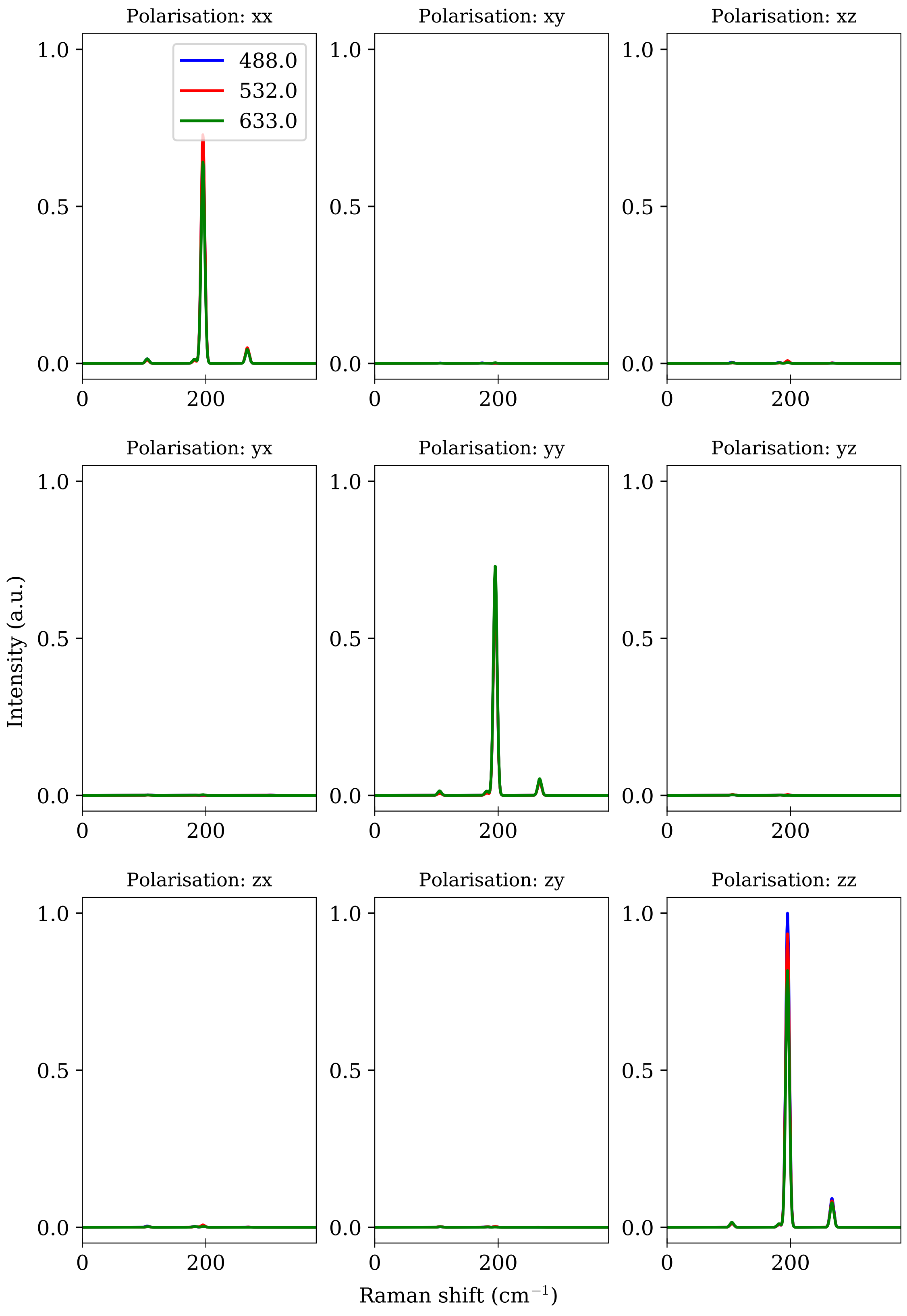

Supplementary Figure 636: Raman spectrum of W_2Cl_2 with space group 12 and Wyckoff positions i-0.

W_2Cl_6, 162, d-k-0

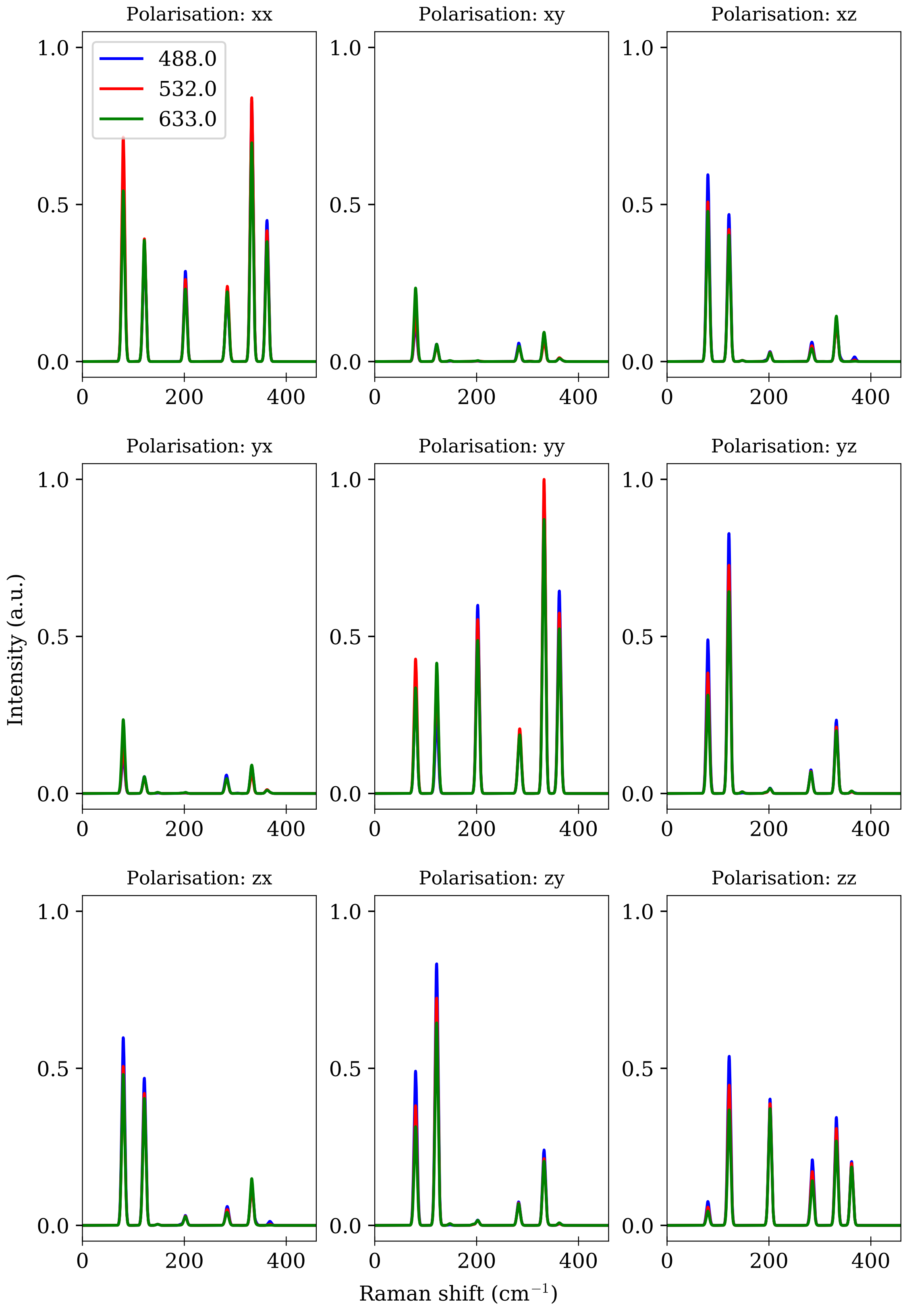

Supplementary Figure 637: Raman spectrum of W_2Cl_6 with space group 162 and Wyckoff positions d-k-0.

W_2C, 164, b-d-0

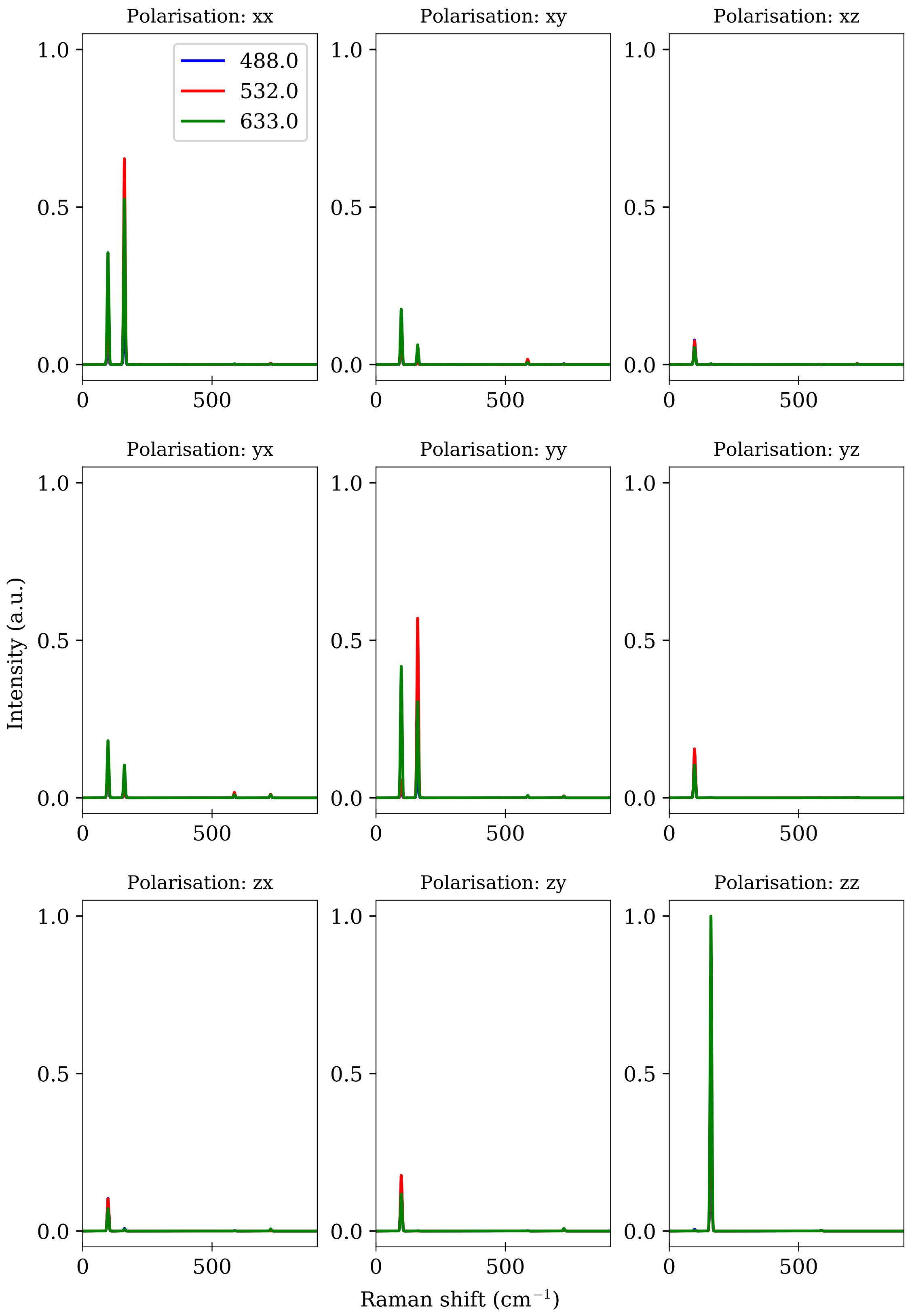

Supplementary Figure 638: Raman spectrum of W_2C with space group 164 and Wyckoff positions b-d-0.

W_2F_2, 12, i-0

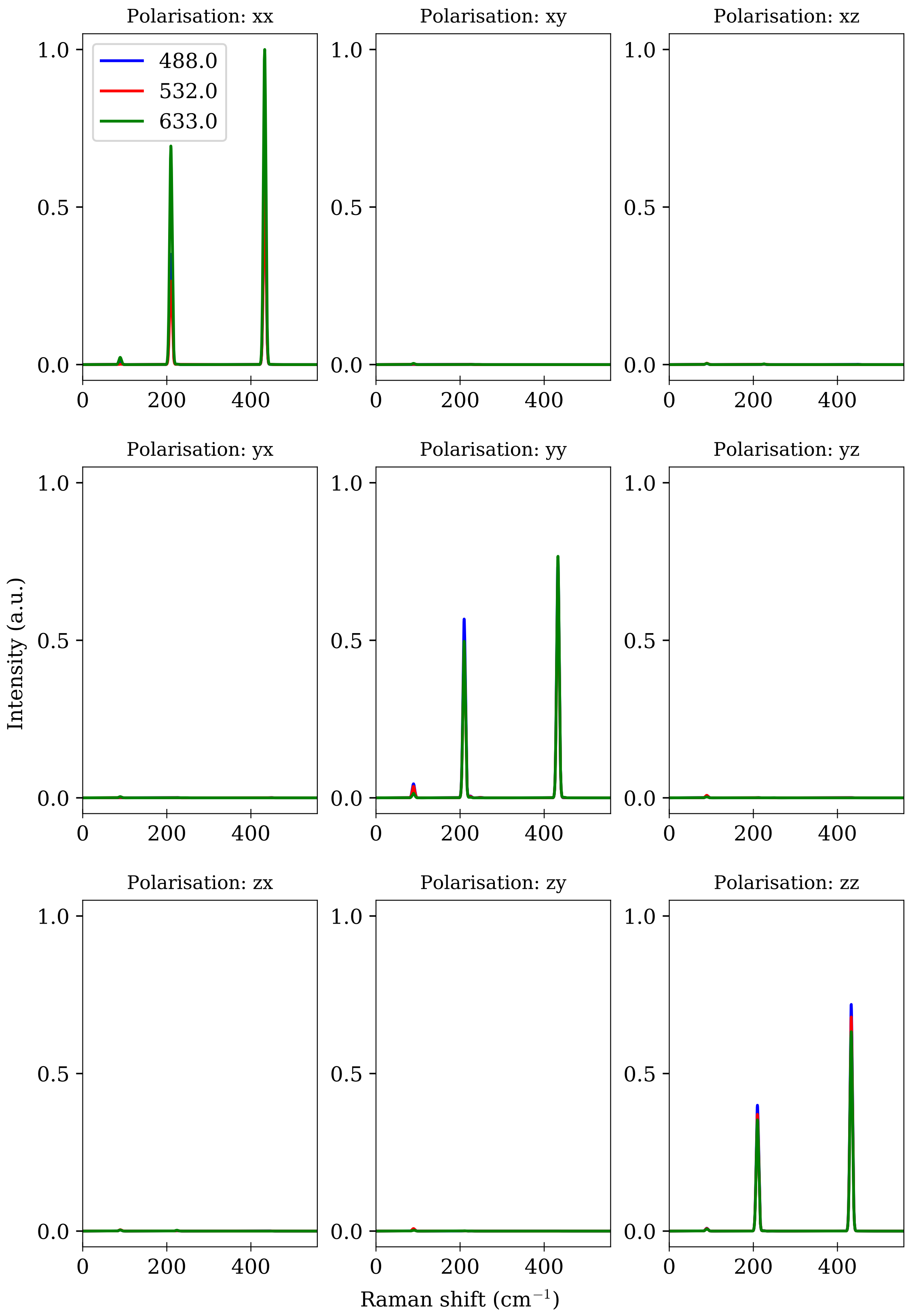

Supplementary Figure 639: Raman spectrum of W_2F_2 with space group 12 and Wyckoff positions i-0.

W_2O_4, 11, e-0

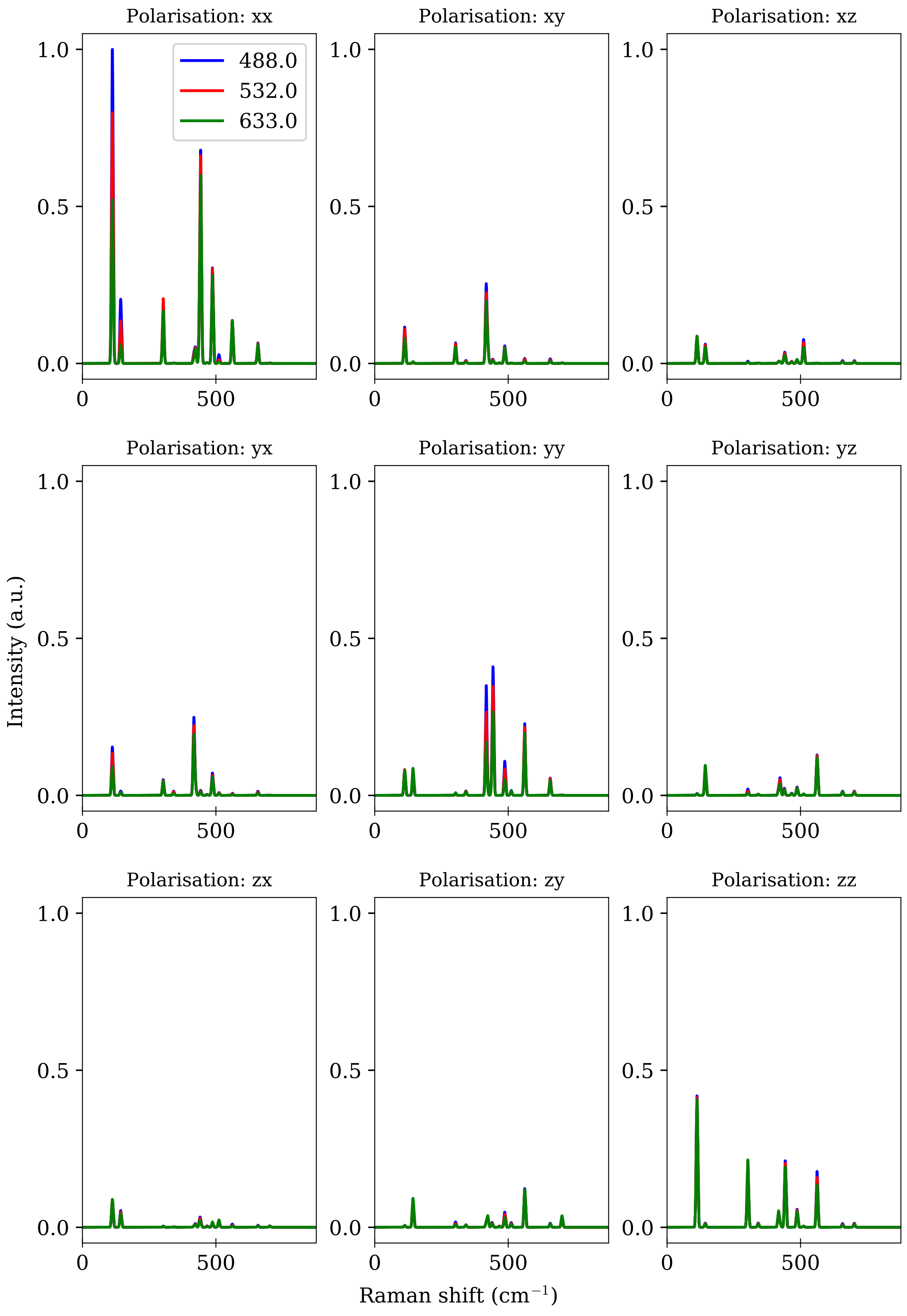

Supplementary Figure 640: Raman spectrum of W_2O_4 with space group 11 and Wyckoff positions e-0.

W_2S_2, 129, b-c-0

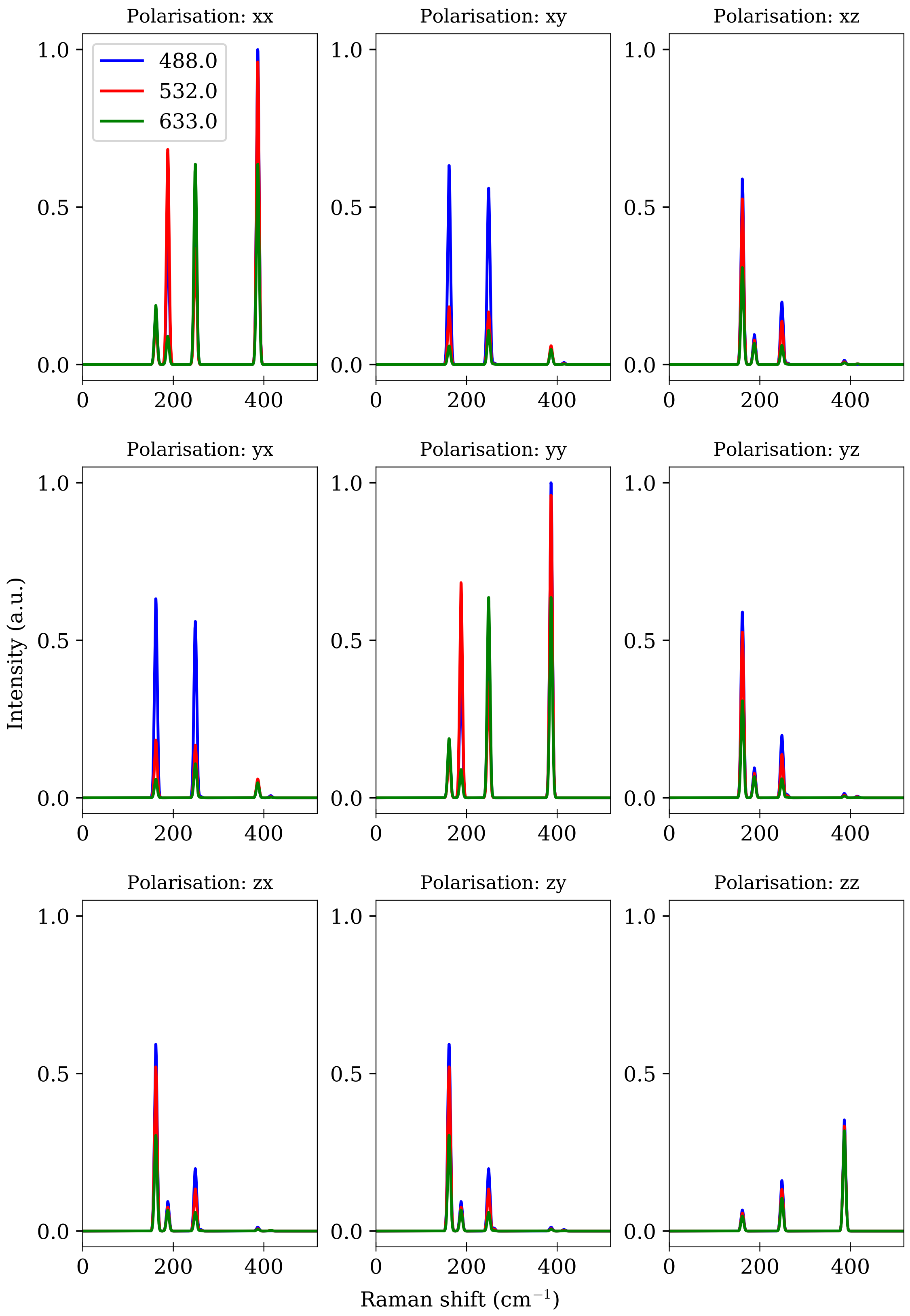

Supplementary Figure 641: Raman spectrum of W_2S_2 with space group 129 and Wyckoff positions b-c-0.

W_2S_4, 11, e-0

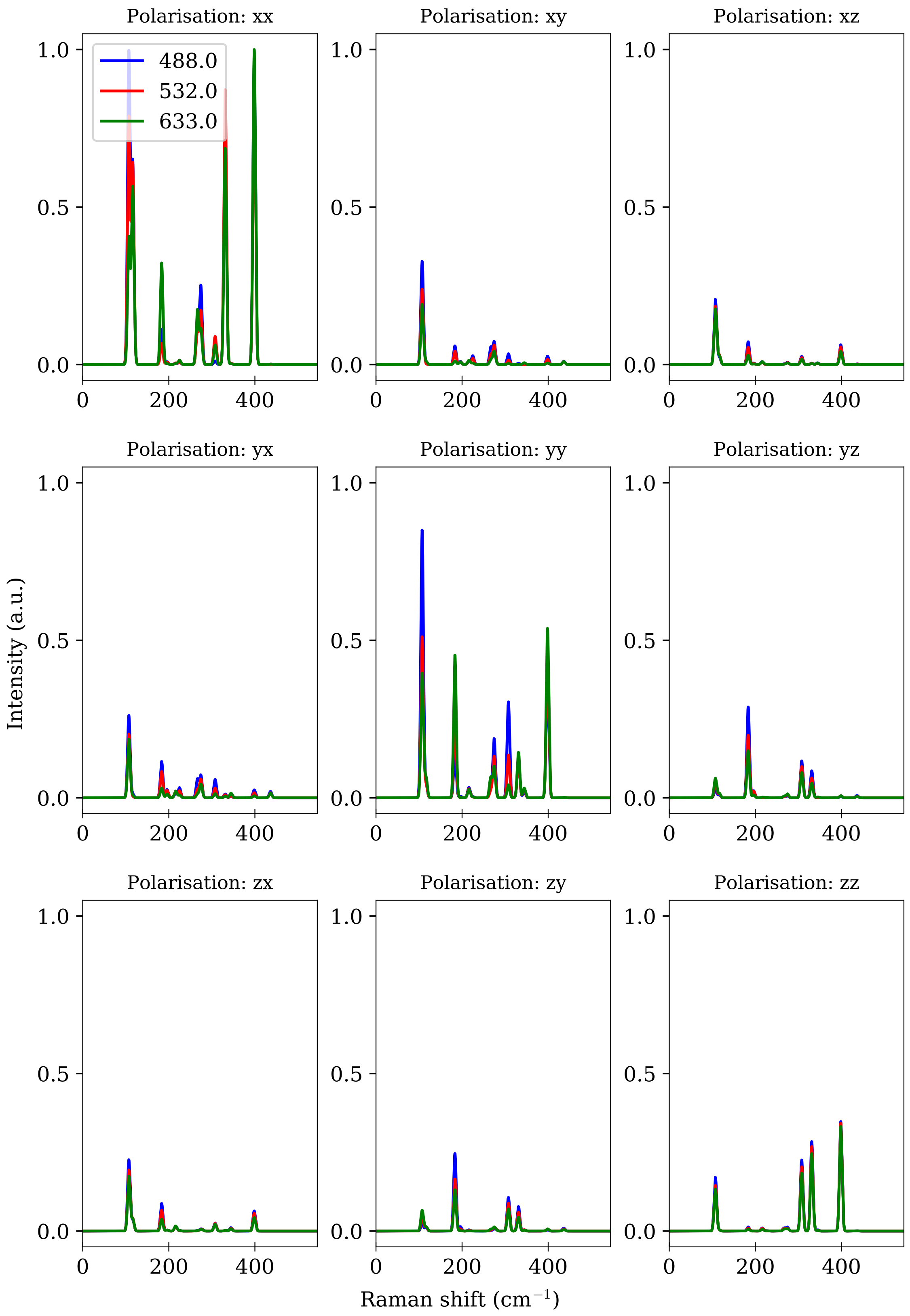

Supplementary Figure 642: Raman spectrum of W_2S_4 with space group 11 and Wyckoff positions e-0.

W_2Se_2, 2, i-0

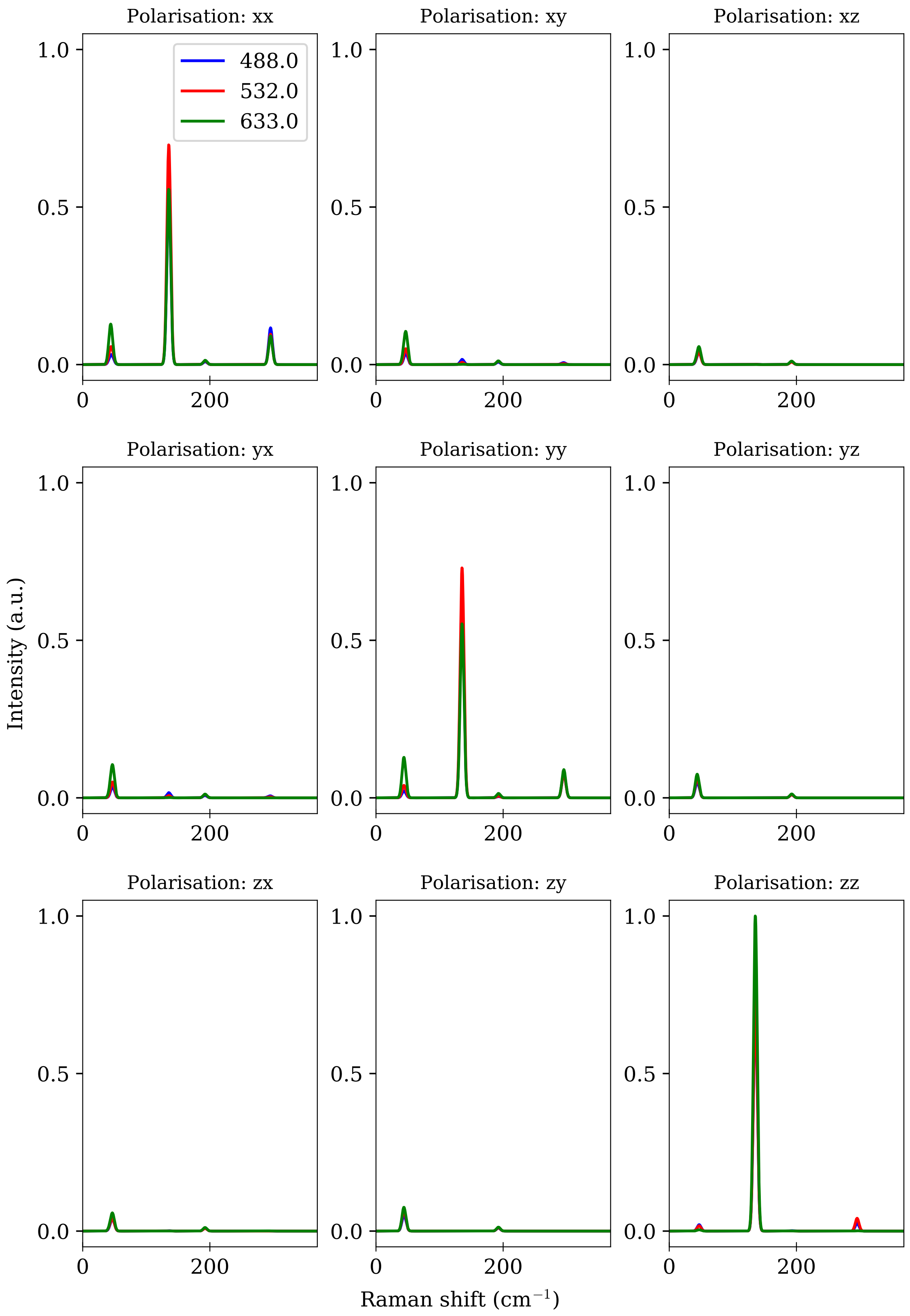

Supplementary Figure 643: Raman spectrum of W_2Se_2 with space group 2 and Wyckoff positions i-0.

W_2Se_4, 11, e-0

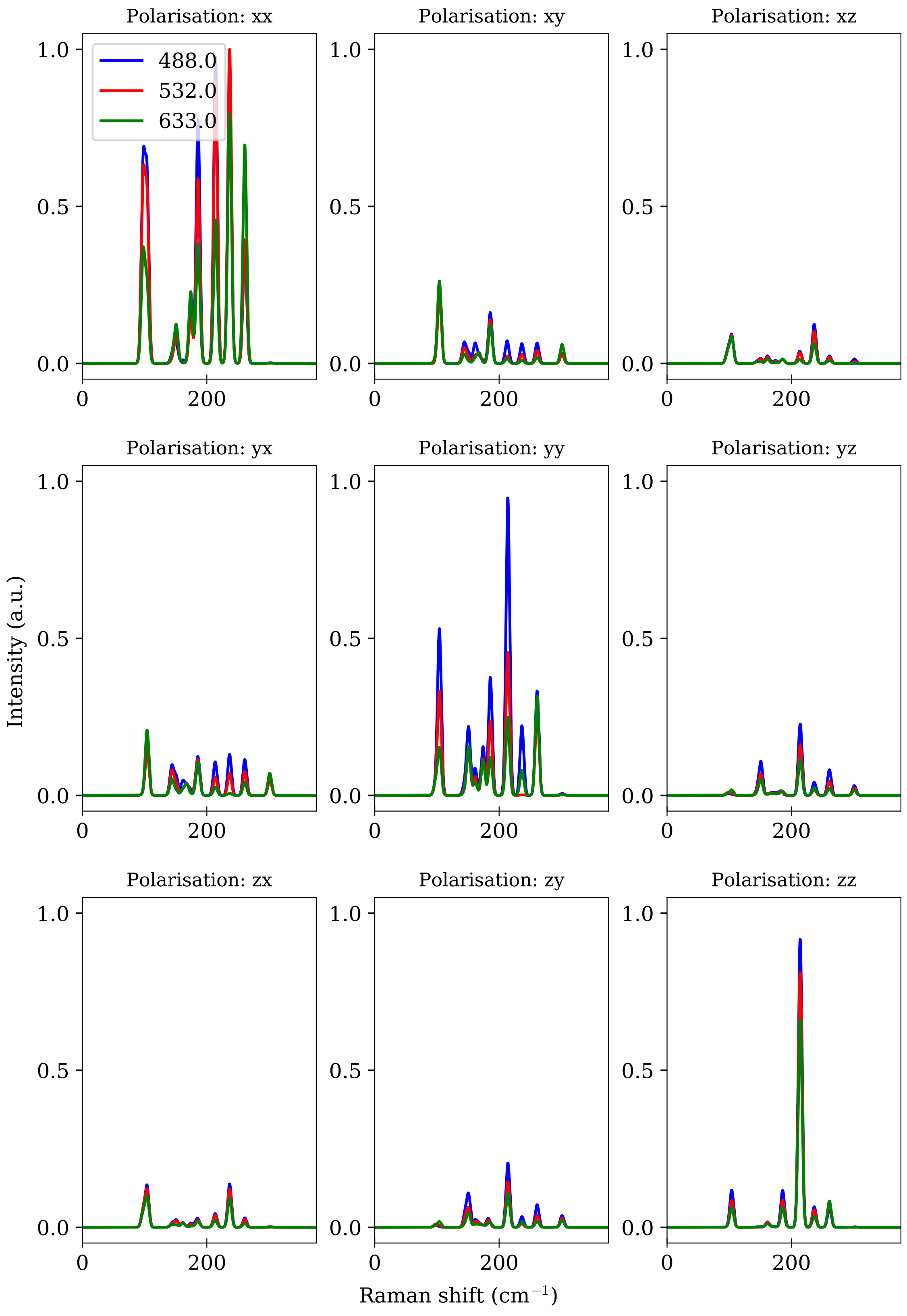

Supplementary Figure 644: Raman spectrum of W_2Se_4 with space group 11 and Wyckoff positions e-0.

W_2Te_4, 11, e-0

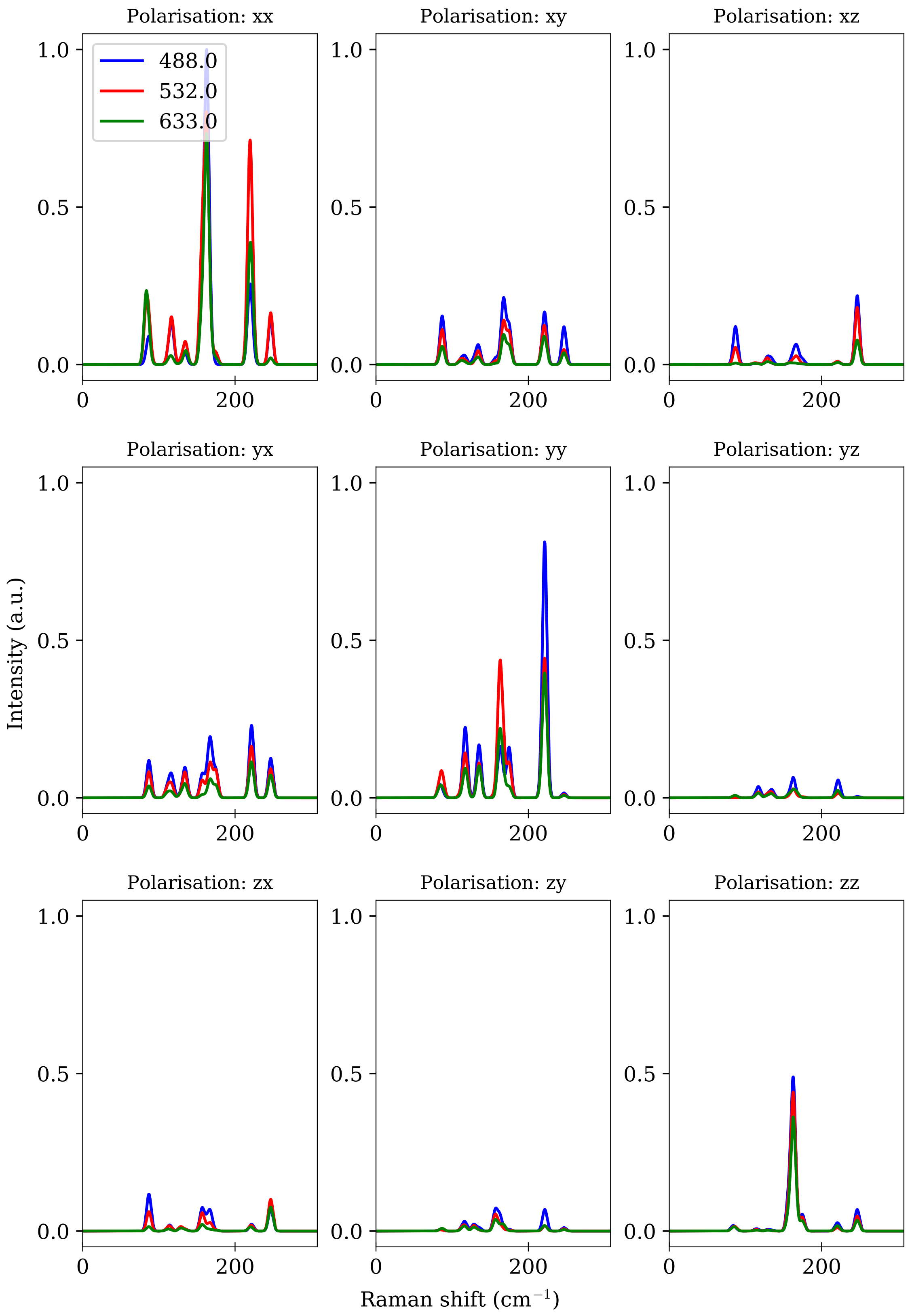

Supplementary Figure 645: Raman spectrum of W_2Te_4 with space group 11 and Wyckoff positions e-0.

W_3C_2, 187, a-h-i-0

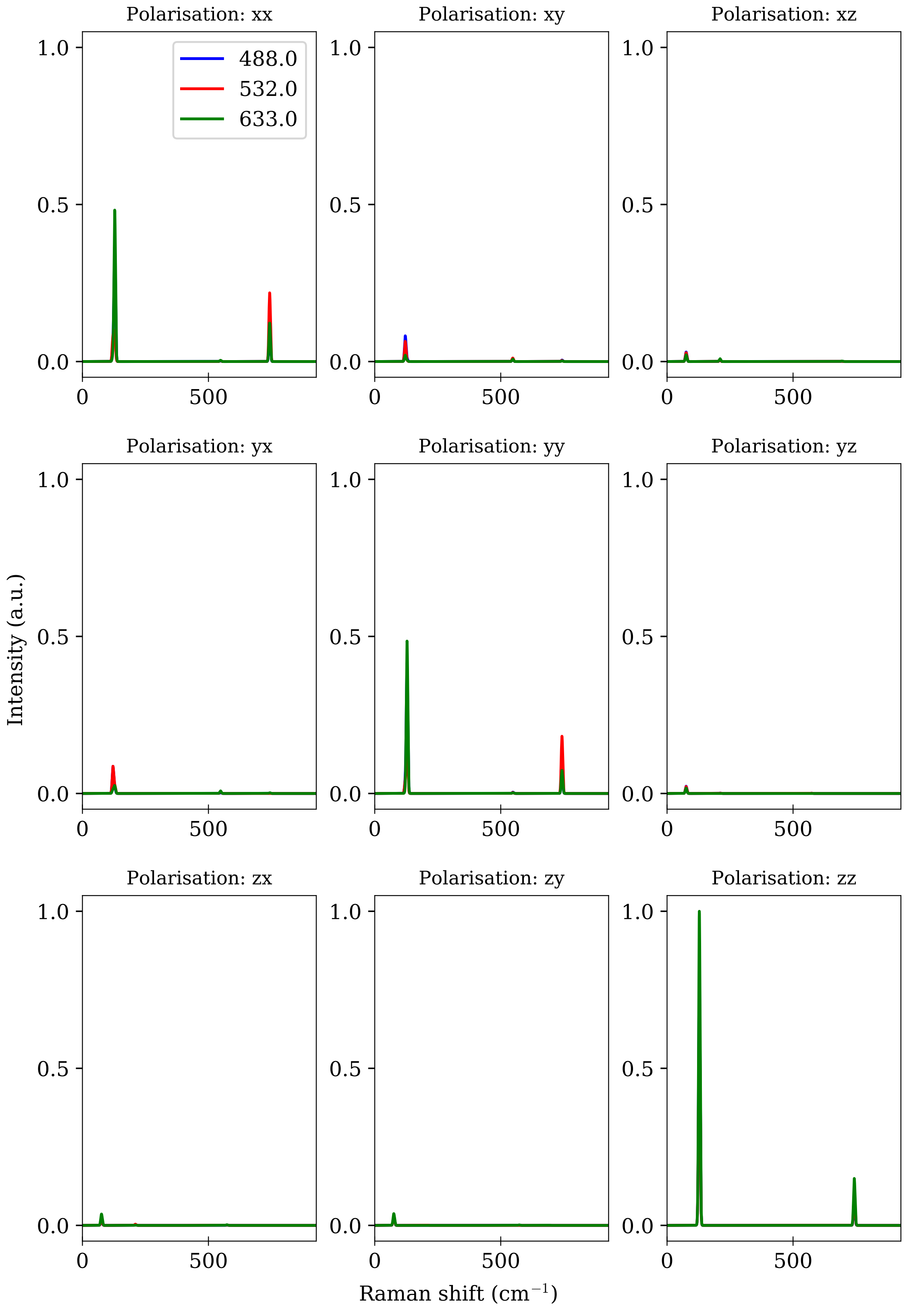

Supplementary Figure 646: Raman spectrum of W_3C_2 with space group 187 and Wyckoff positions a-h-i-0.

WAs_2, 164, b-d-0

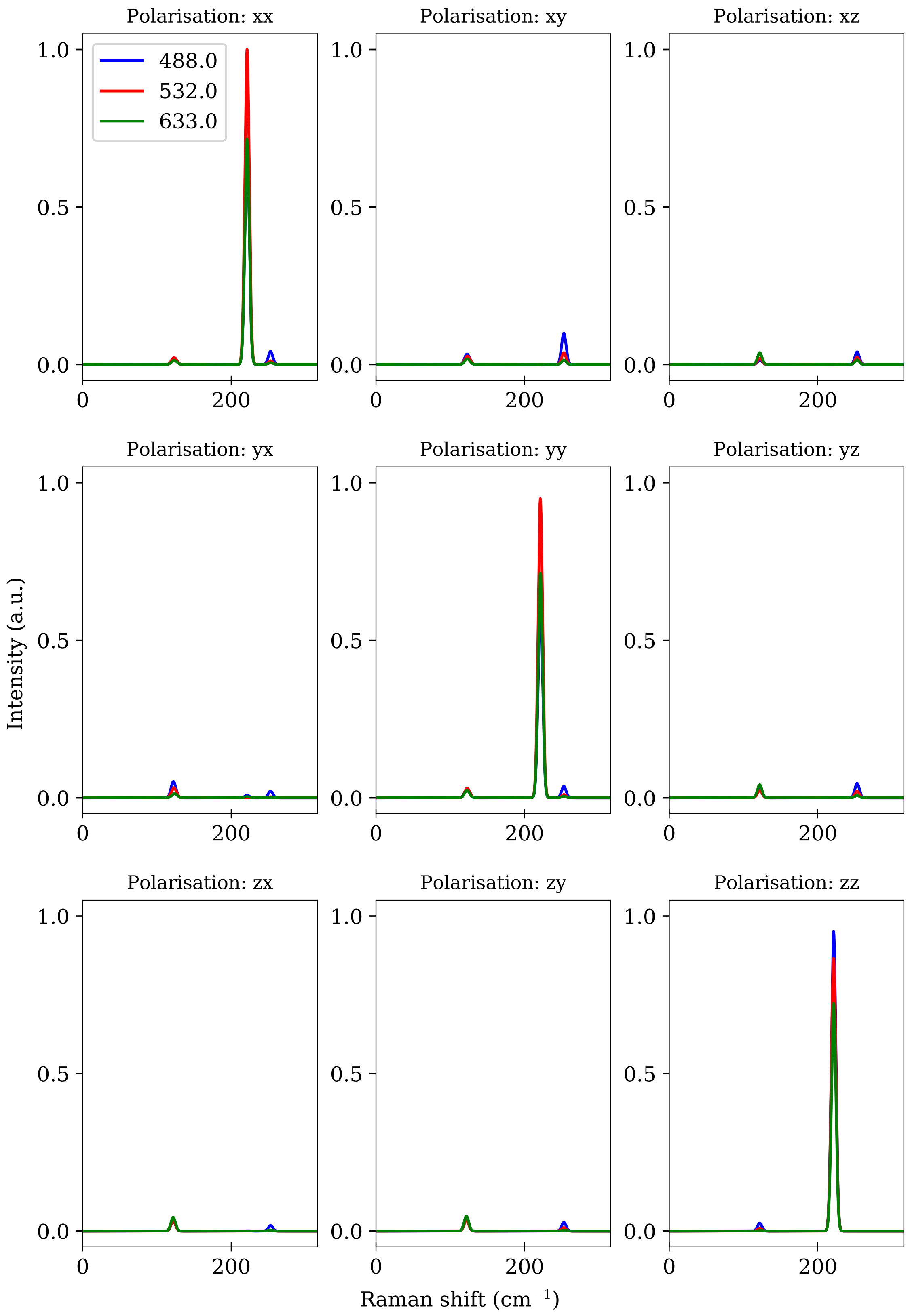

Supplementary Figure 647: Raman spectrum of WAs_2 with space group 164 and Wyckoff positions b-d-0.

WAs_2, 187, b-i-0

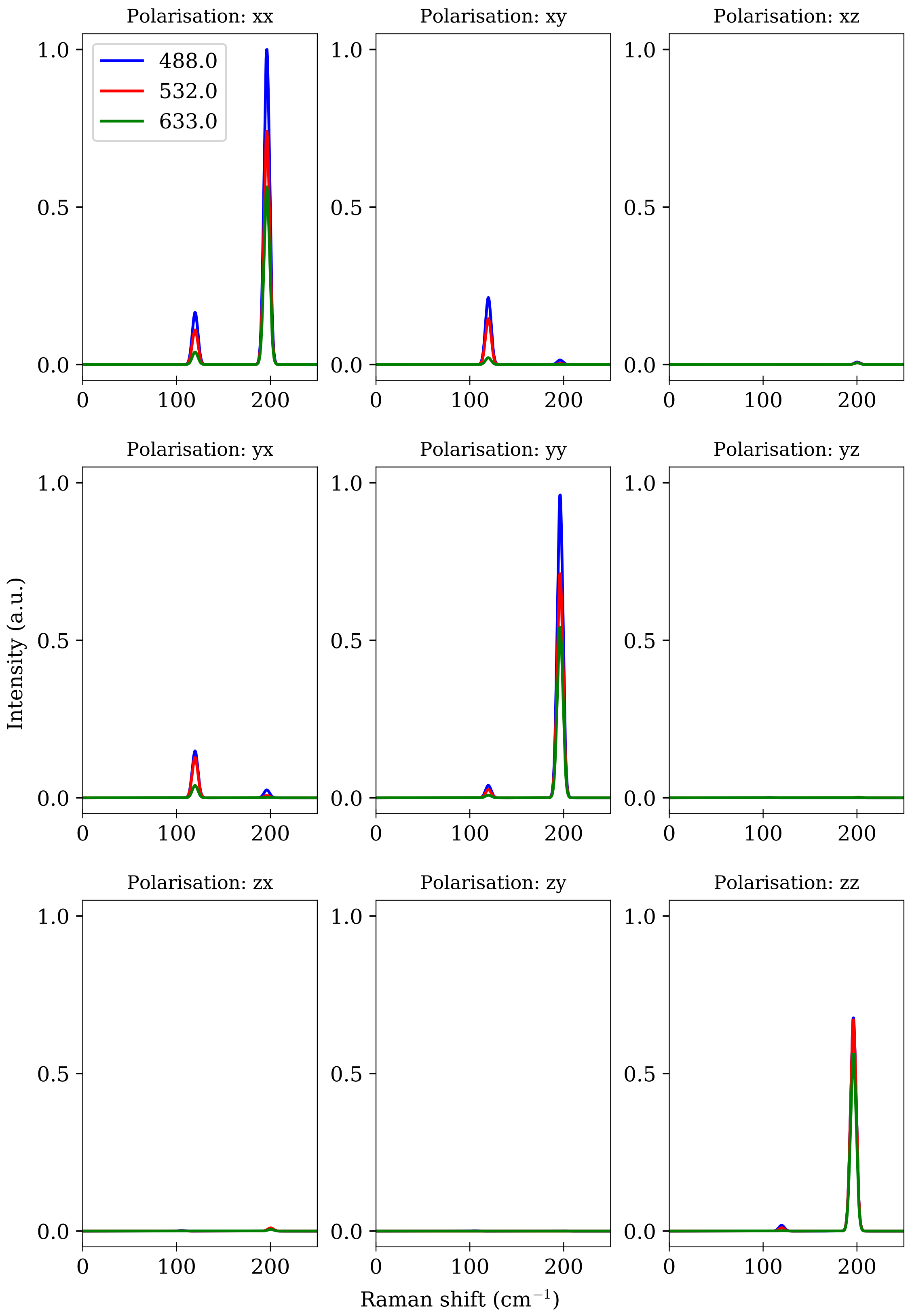

Supplementary Figure 648: Raman spectrum of WAs_2 with space group 187 and Wyckoff positions b-i-0.

WAsSb, 156, a-b-c-0

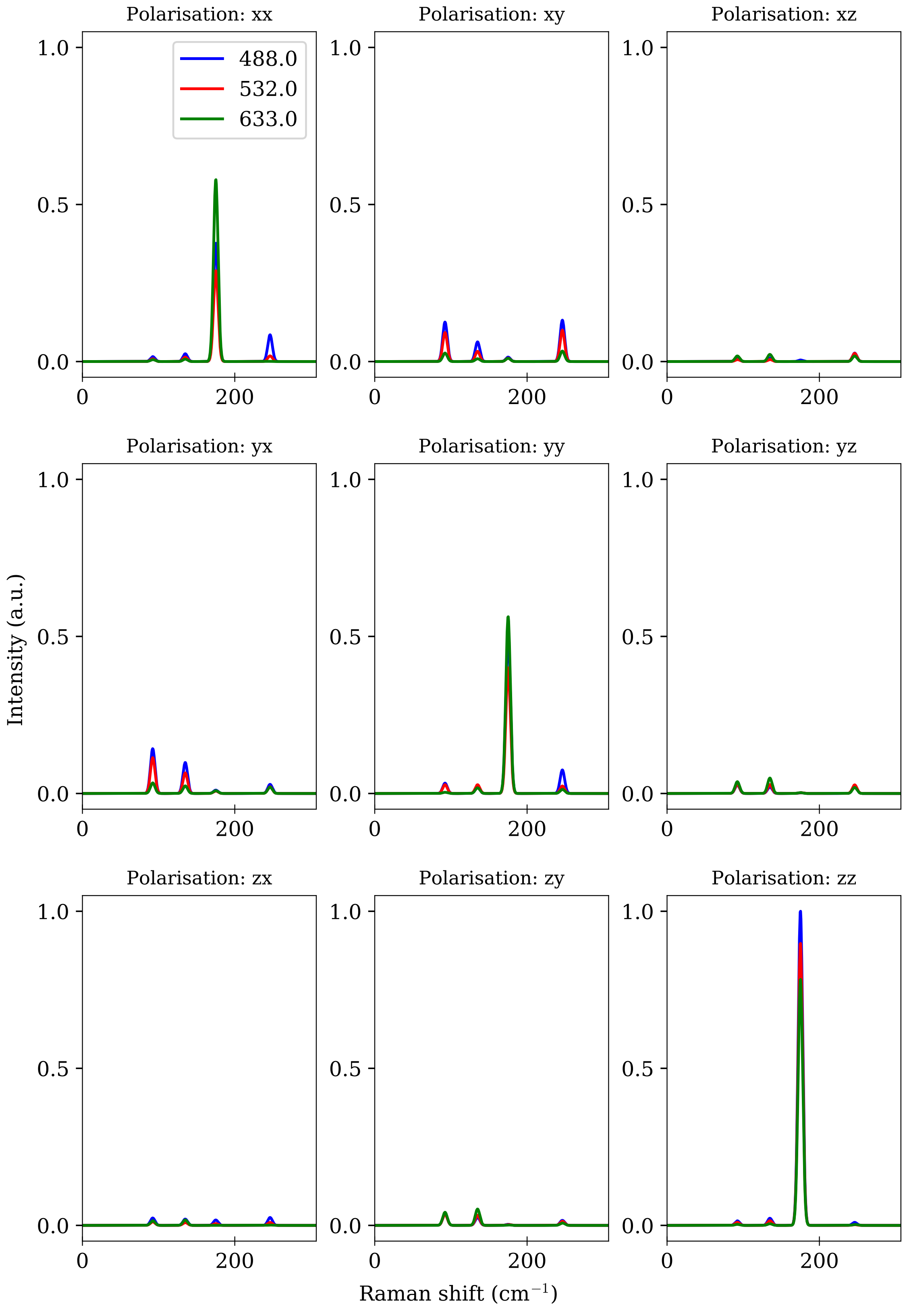

Supplementary Figure 649: Raman spectrum of WAsSb with space group 156 and Wyckoff positions a-b-c-0.

WH_2, 187, a-i-0

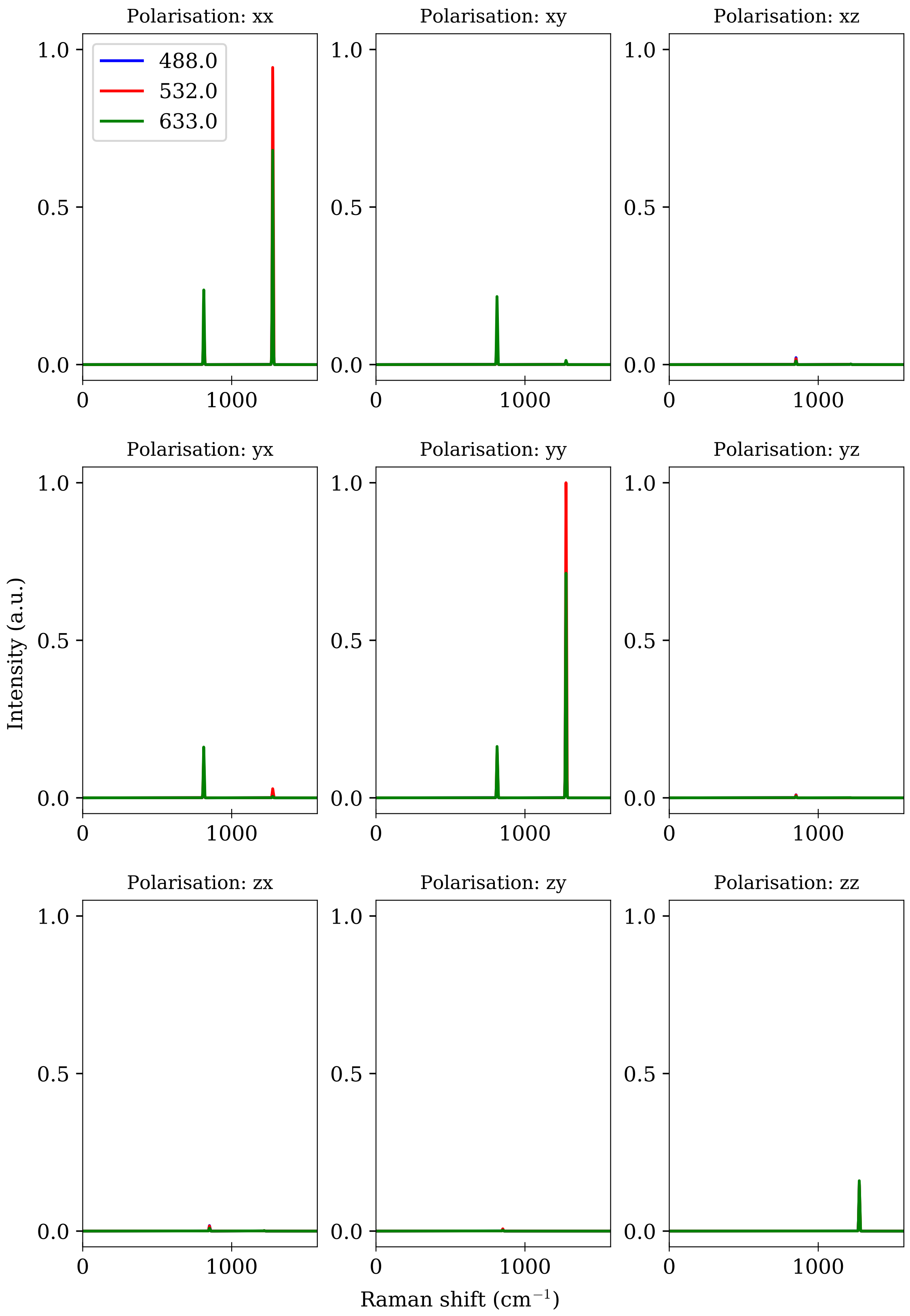

Supplementary Figure 650: Raman spectrum of WH_2 with space group 187 and Wyckoff positions a-i-0.

WO_2, 187, b-i-0

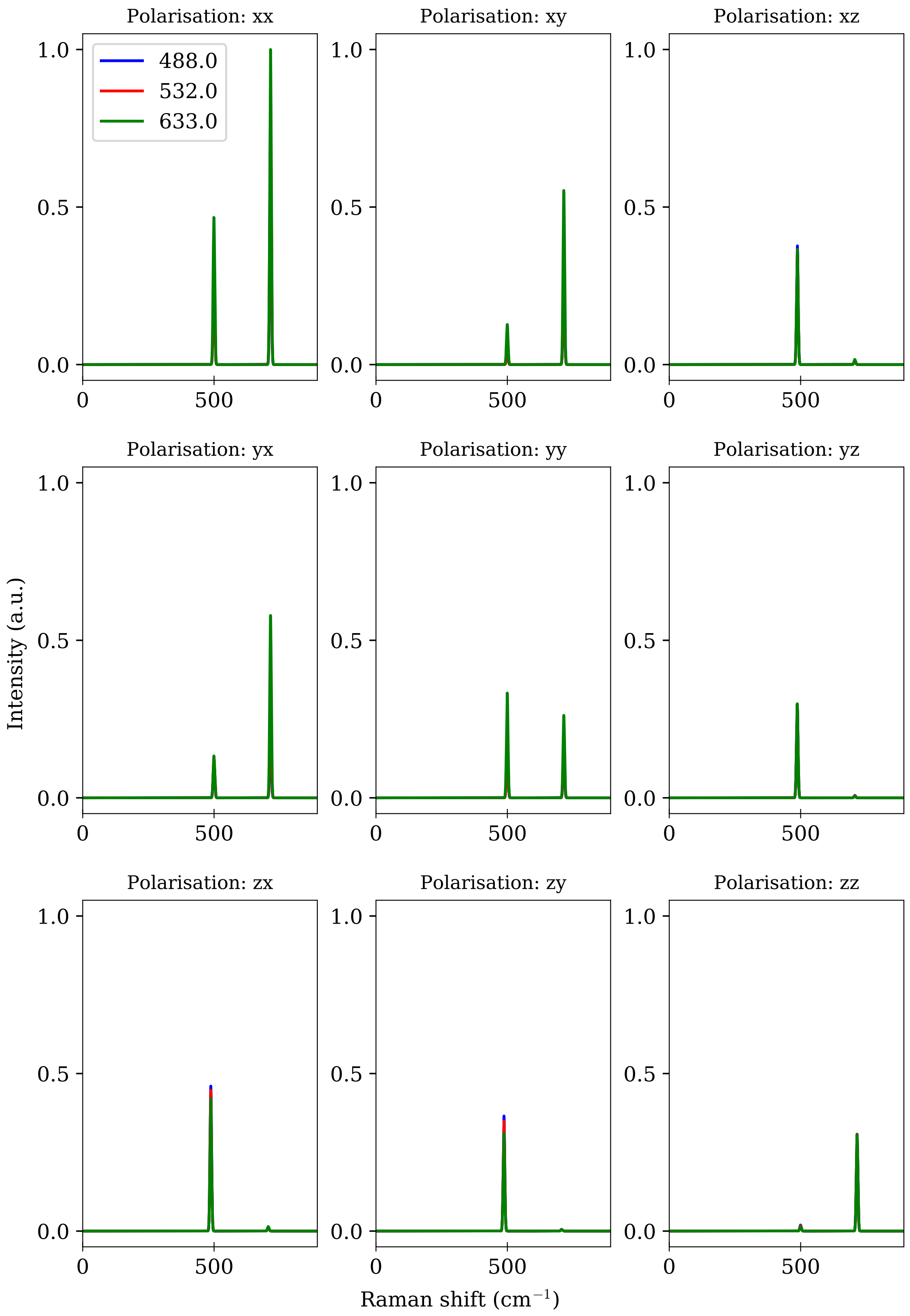

Supplementary Figure 651: Raman spectrum of WO_2 with space group 187 and Wyckoff positions b-i-0.

WS_2, 187, b-i-0

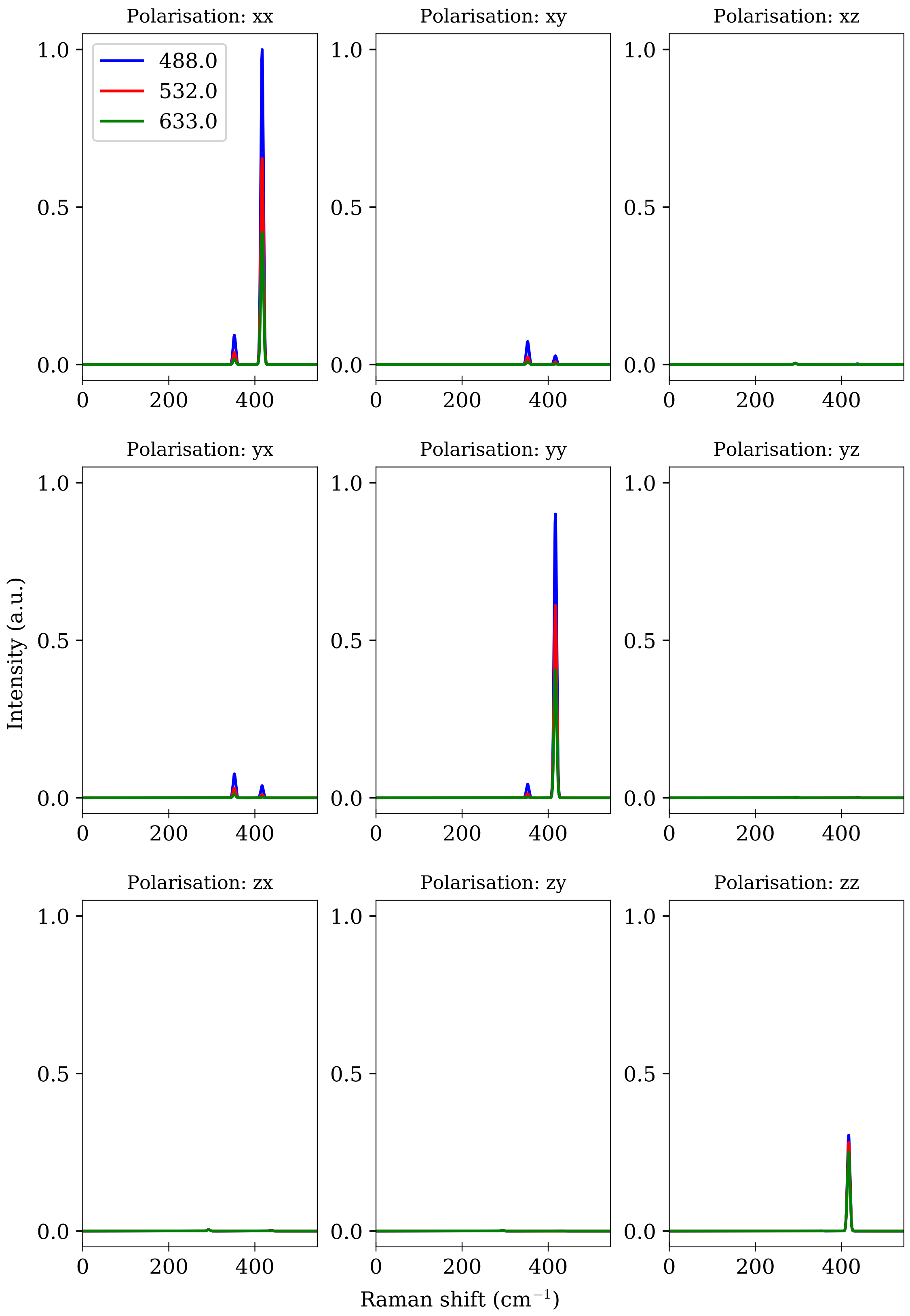

Supplementary Figure 652: Raman spectrum of WS_2 with space group 187 and Wyckoff positions b-i-0.

WSb_2, 164, b-d-0

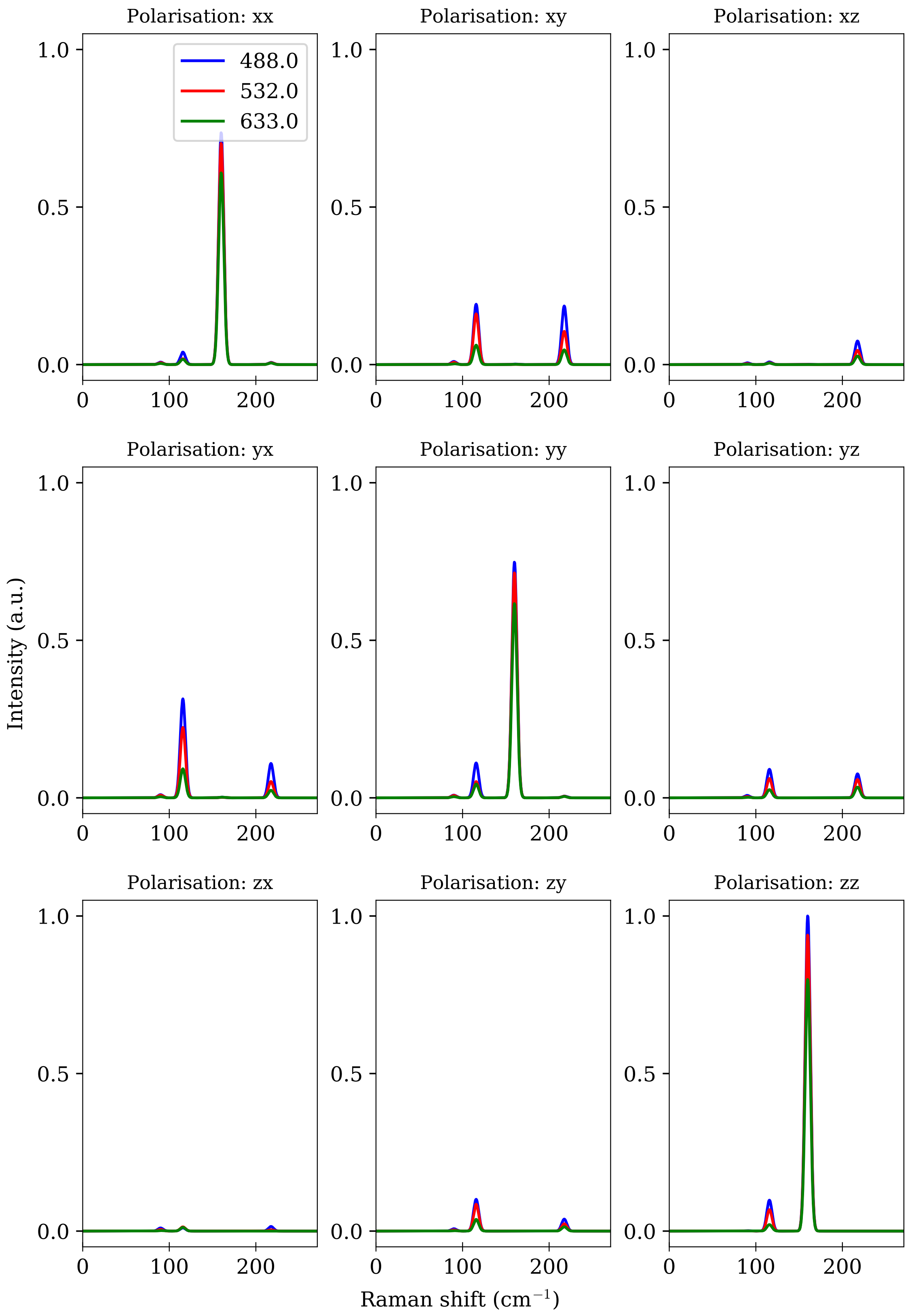

Supplementary Figure 653: Raman spectrum of WSb_2 with space group 164 and Wyckoff positions b-d-0.

WSb_2, 187, a-i-0

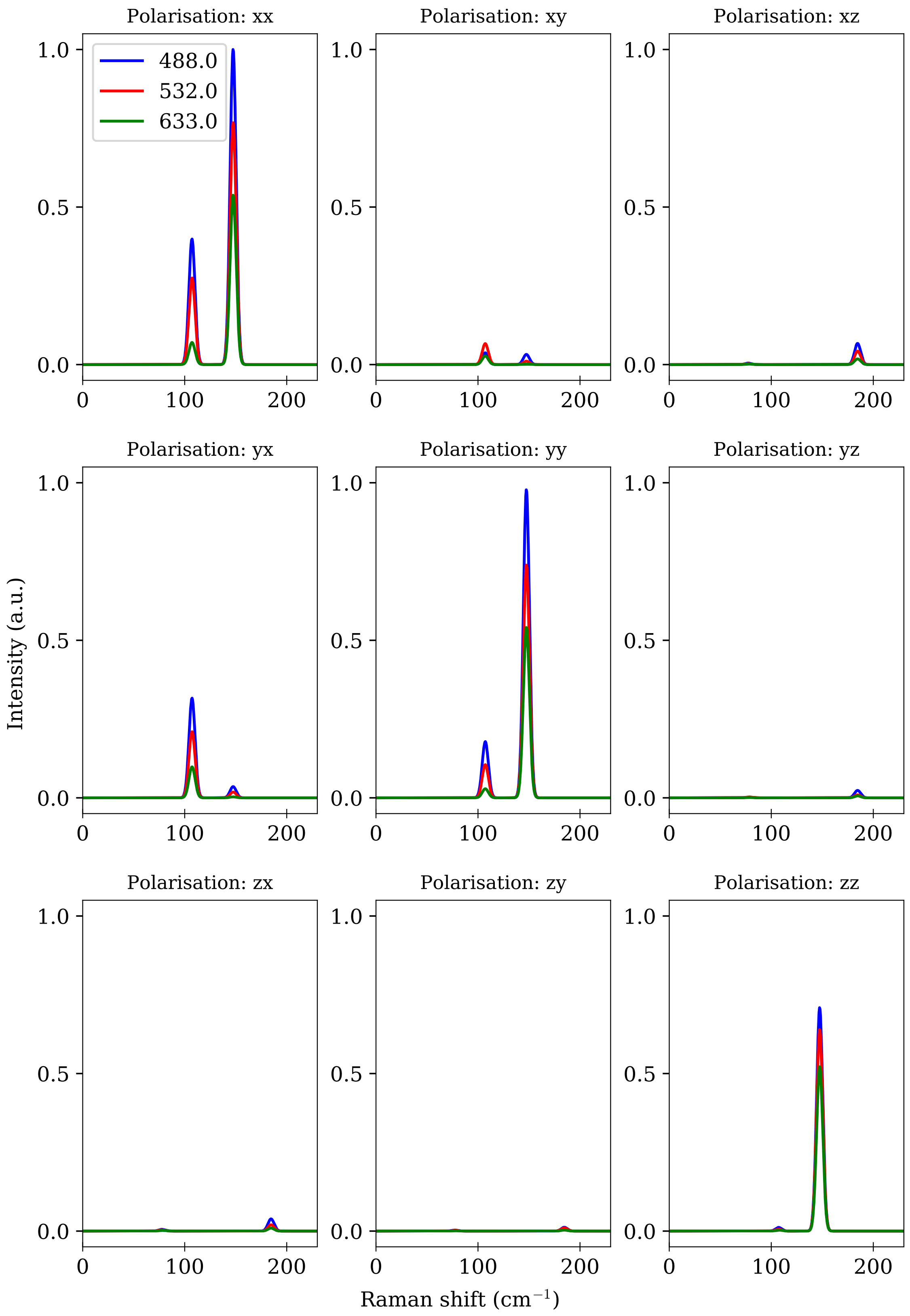

Supplementary Figure 654: Raman spectrum of WSb_2 with space group 187 and Wyckoff positions a-i-0.

WSe_2, 187, b-i-0

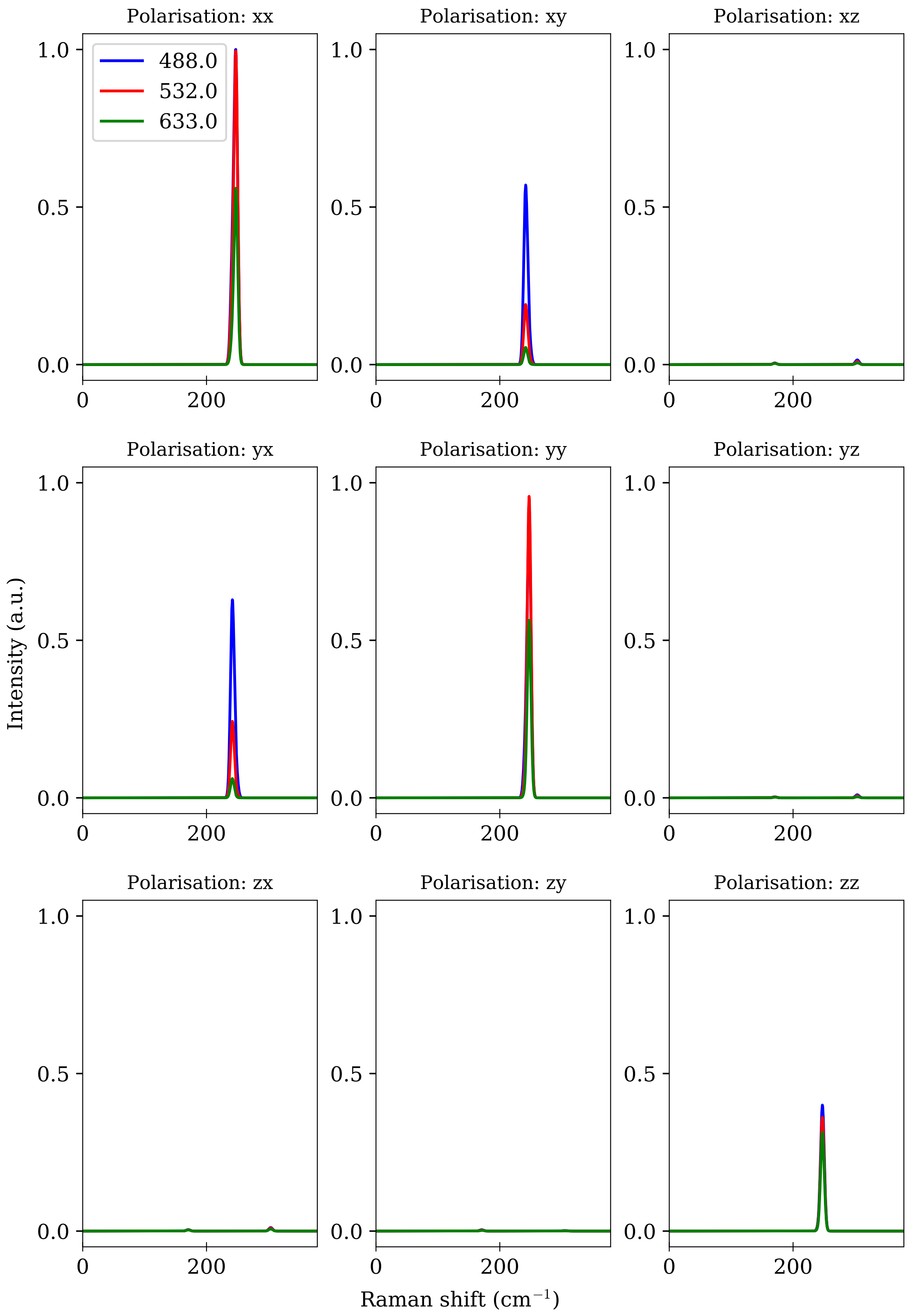

Supplementary Figure 655: Raman spectrum of WSe_2 with space group 187 and Wyckoff positions b-i-0.

WSeTe, 156, a-c-0

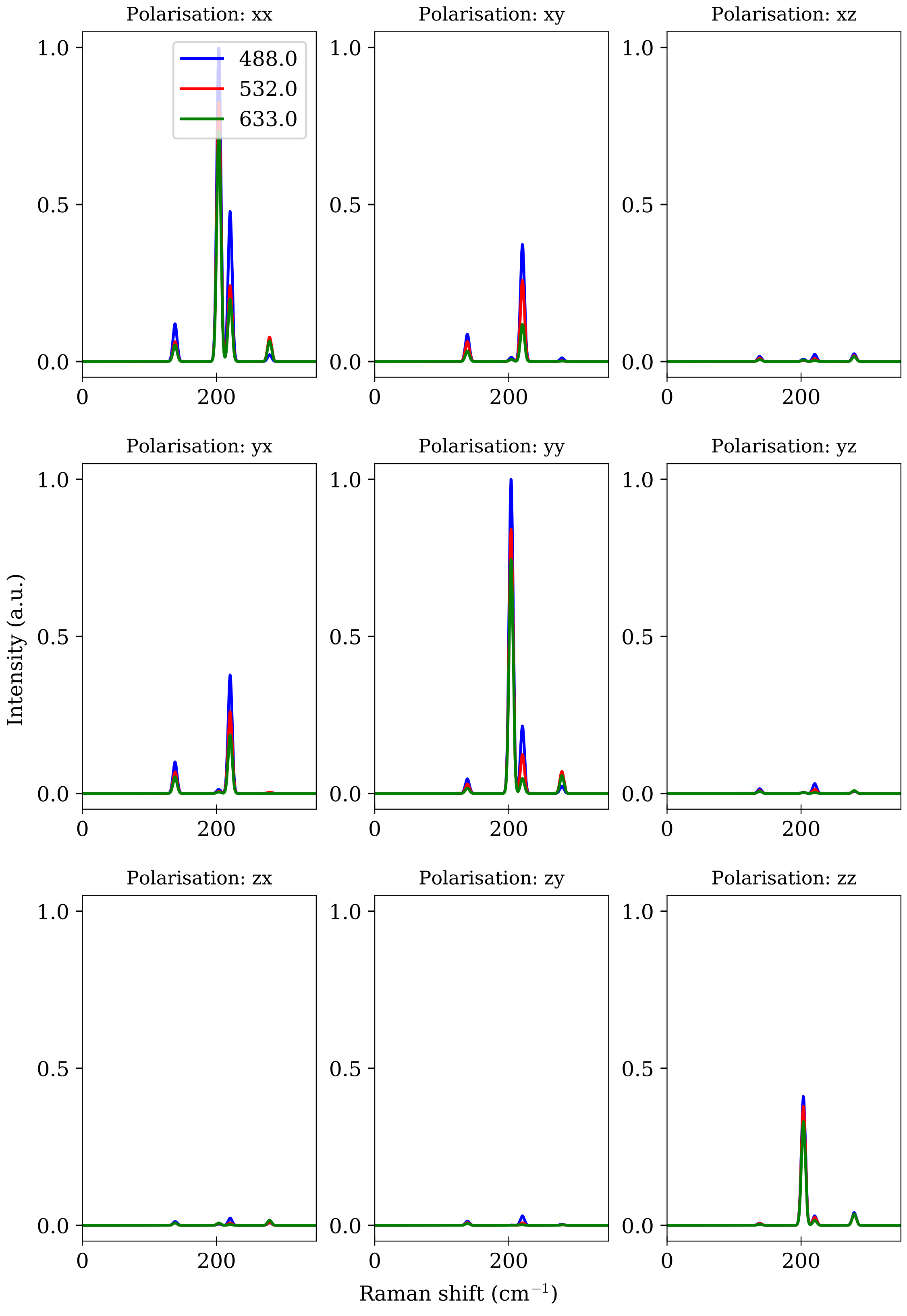

Supplementary Figure 656: Raman spectrum of WSeTe with space group 156 and Wyckoff positions a-c-0.

WSSe, 156, a-c-0

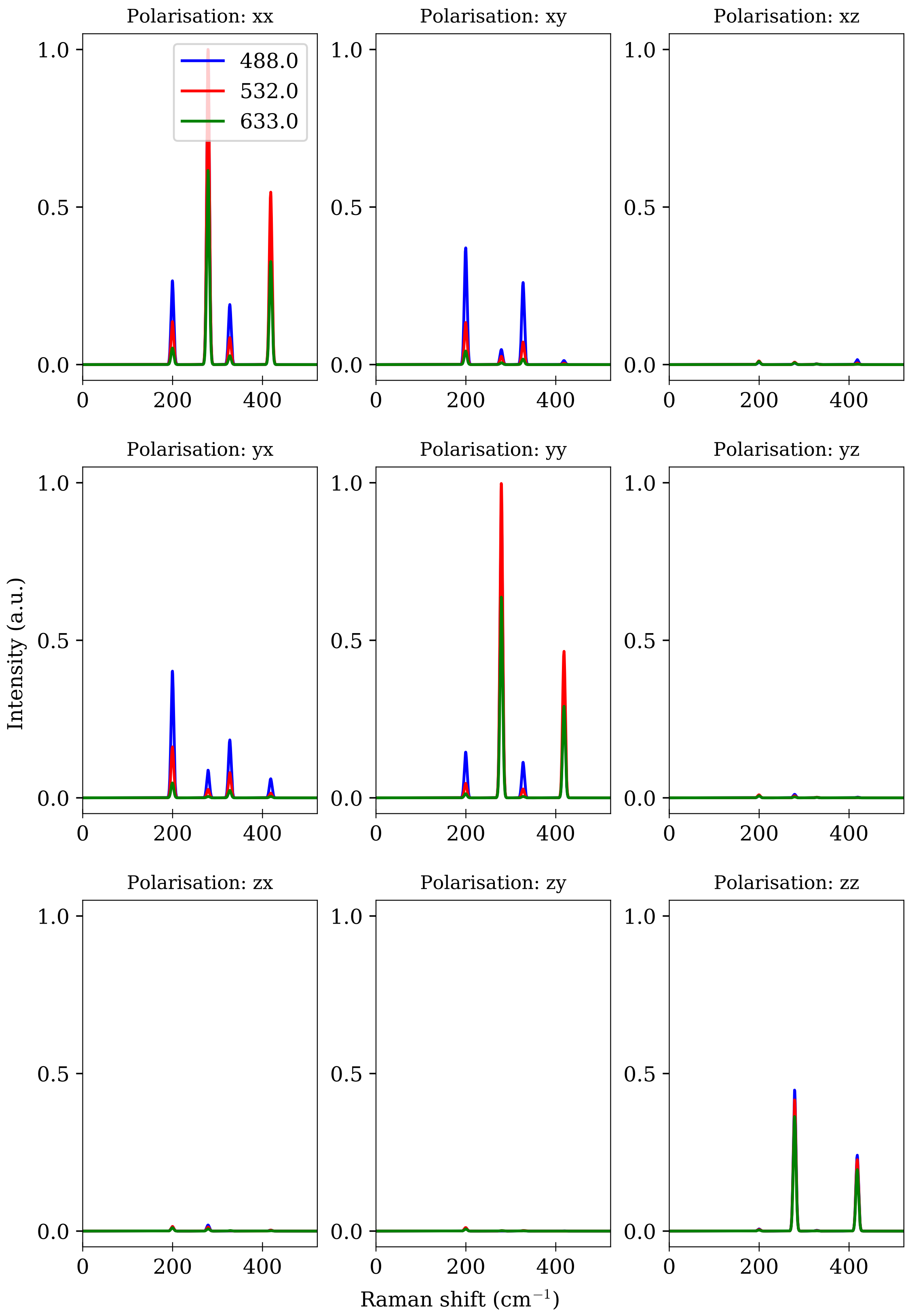

Supplementary Figure 657: Raman spectrum of WSSe with space group 156 and Wyckoff positions a-c-0.

WSTe, 156, a-c-0

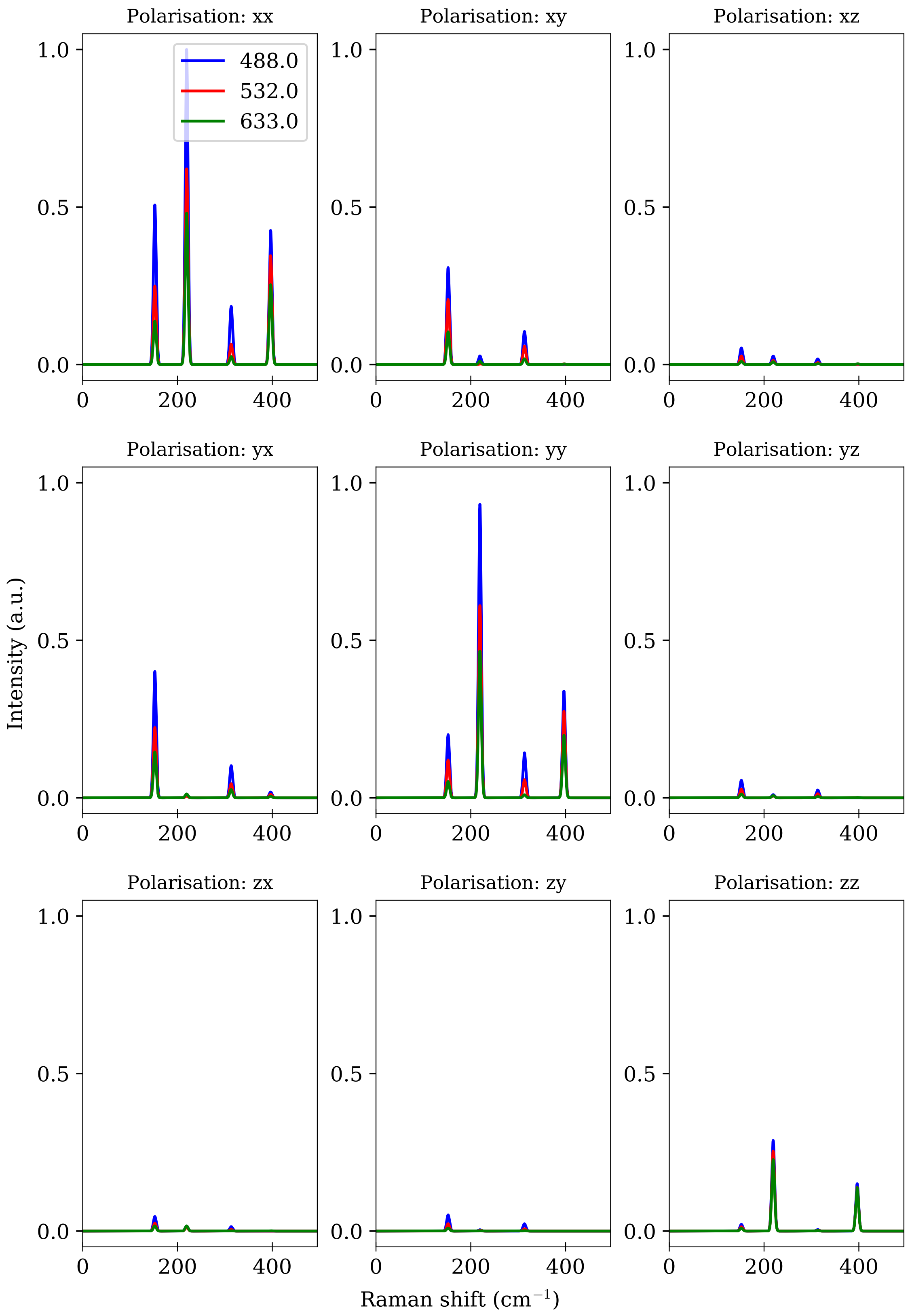

Supplementary Figure 658: Raman spectrum of WSTe with space group 156 and Wyckoff positions a-c-0.

WTe_2, 187, b-i-0

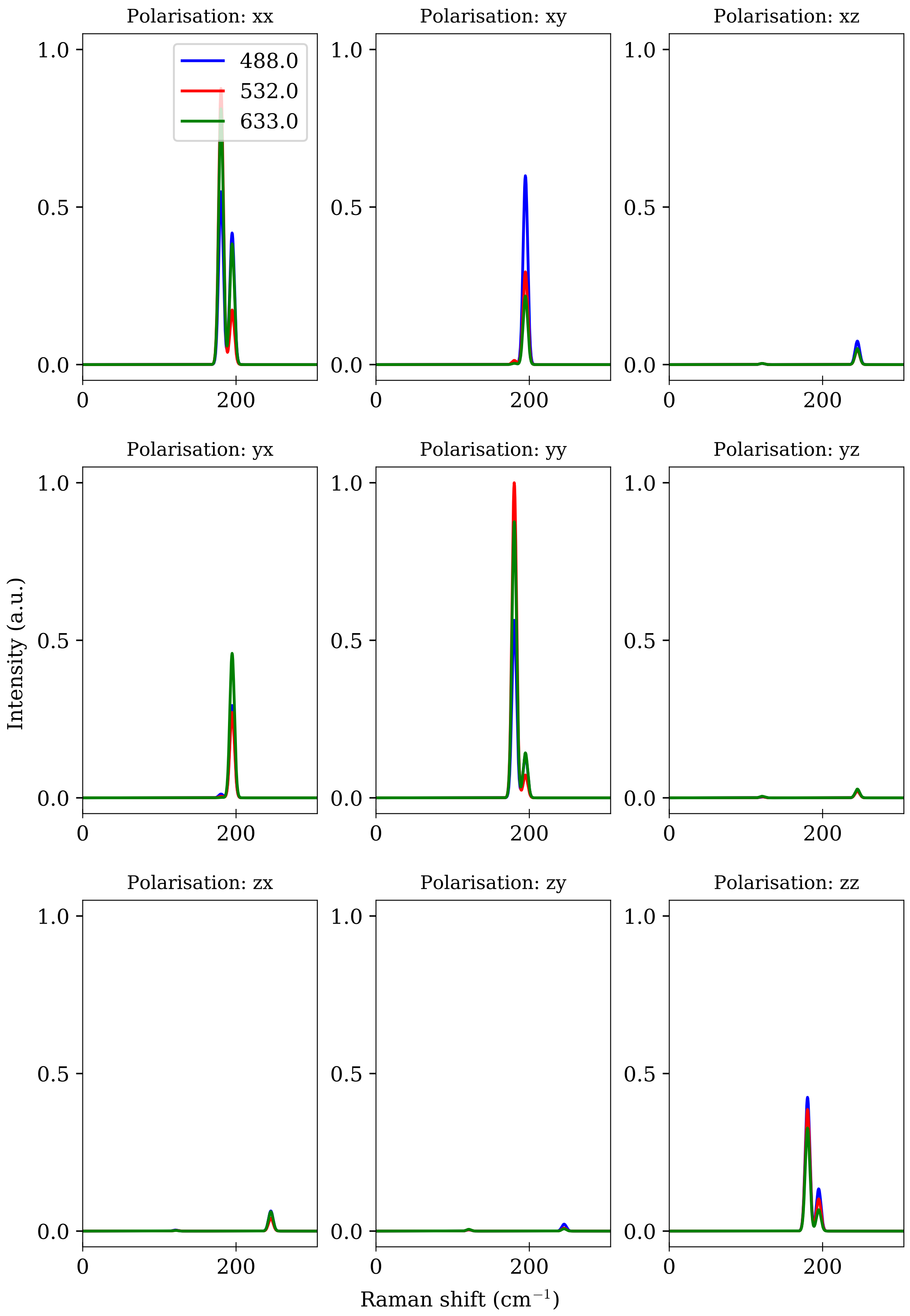

Supplementary Figure 659: Raman spectrum of WTe_2 with space group 187 and Wyckoff positions b-i-0.

Y_2CF_2, 164, b-d-0

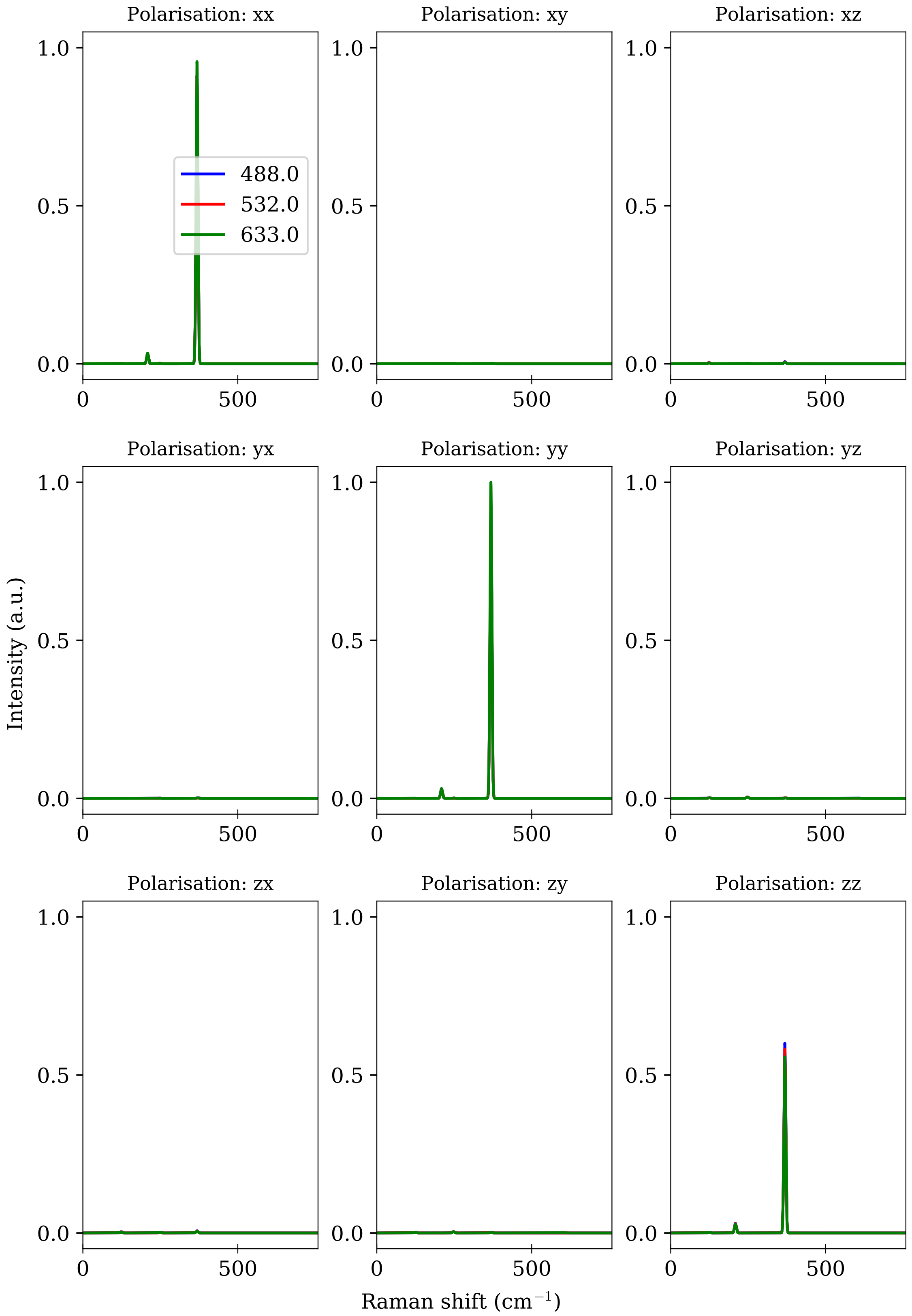

Supplementary Figure 660: Raman spectrum of Y_2CF_2 with space group 164 and Wyckoff positions b-d-0.

$Y_2CH_2O_2$, 164, b-d-0

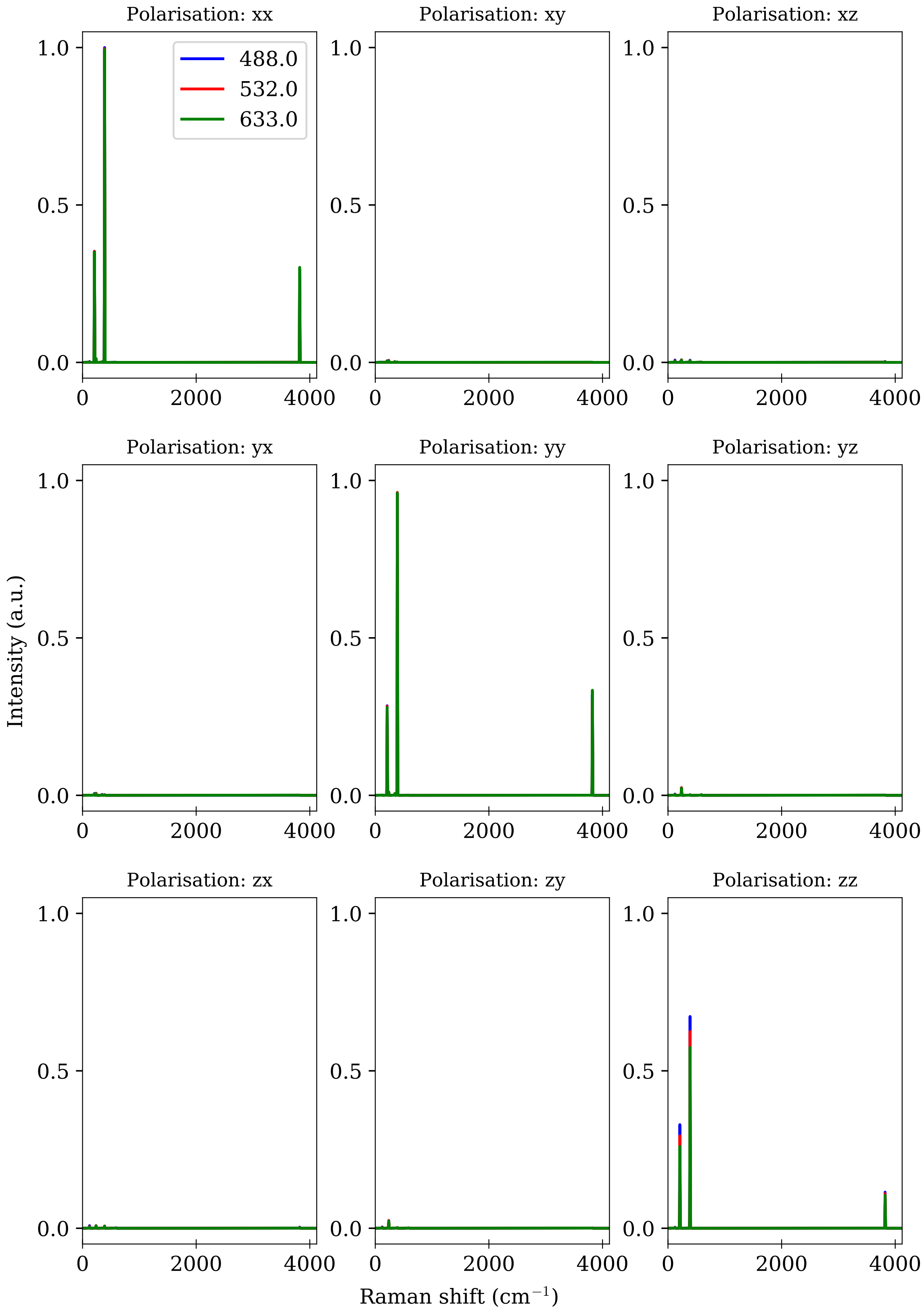

Supplementary Figure 661: Raman spectrum of $Y_2CH_2O_2$ with space group 164 and Wyckoff positions b-d-0.

Y_2C, 164, b-d-0

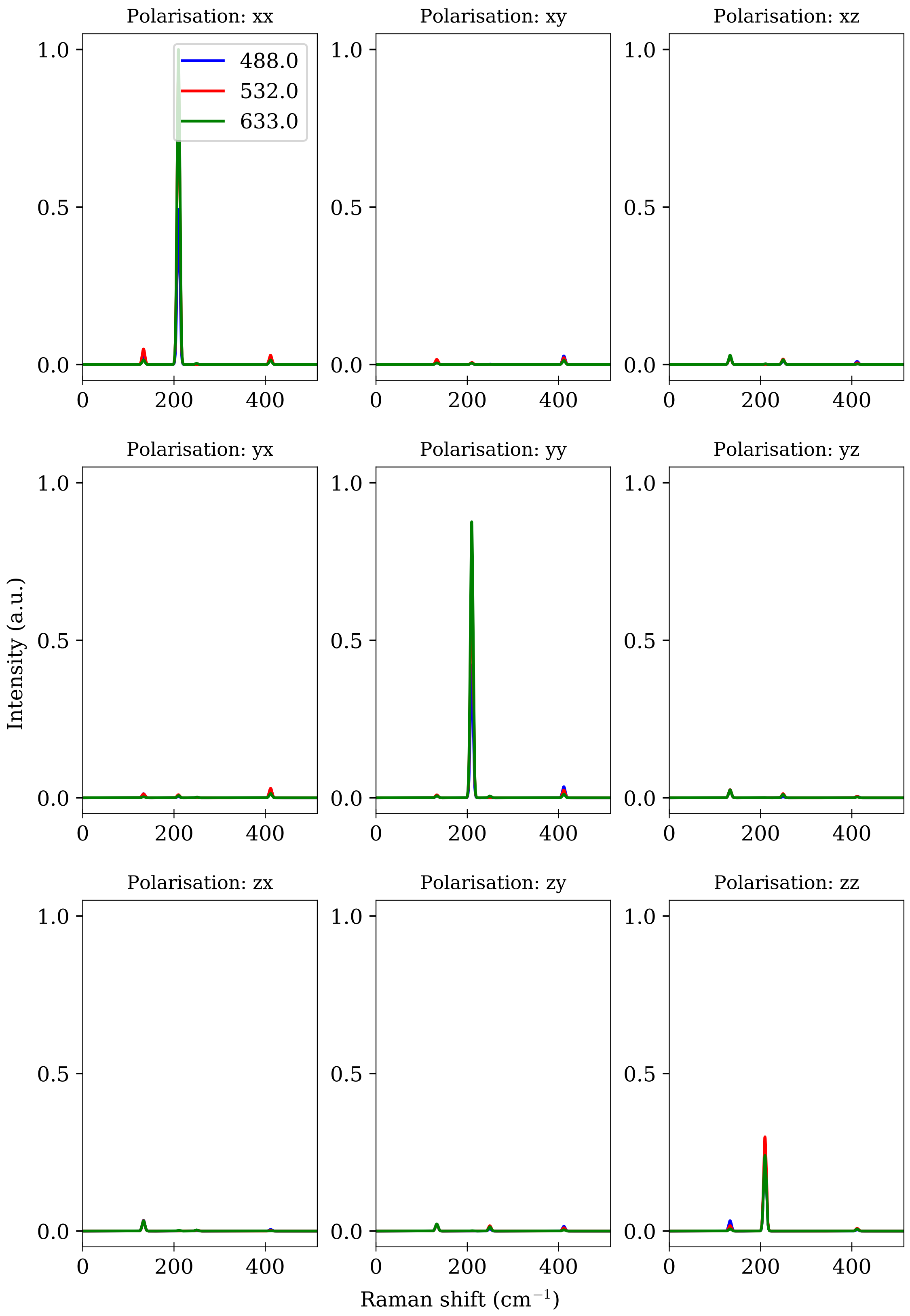

Supplementary Figure 662: Raman spectrum of Y_2C with space group 164 and Wyckoff positions b-d-0.

Y_2F_2N, 164, b-d-0

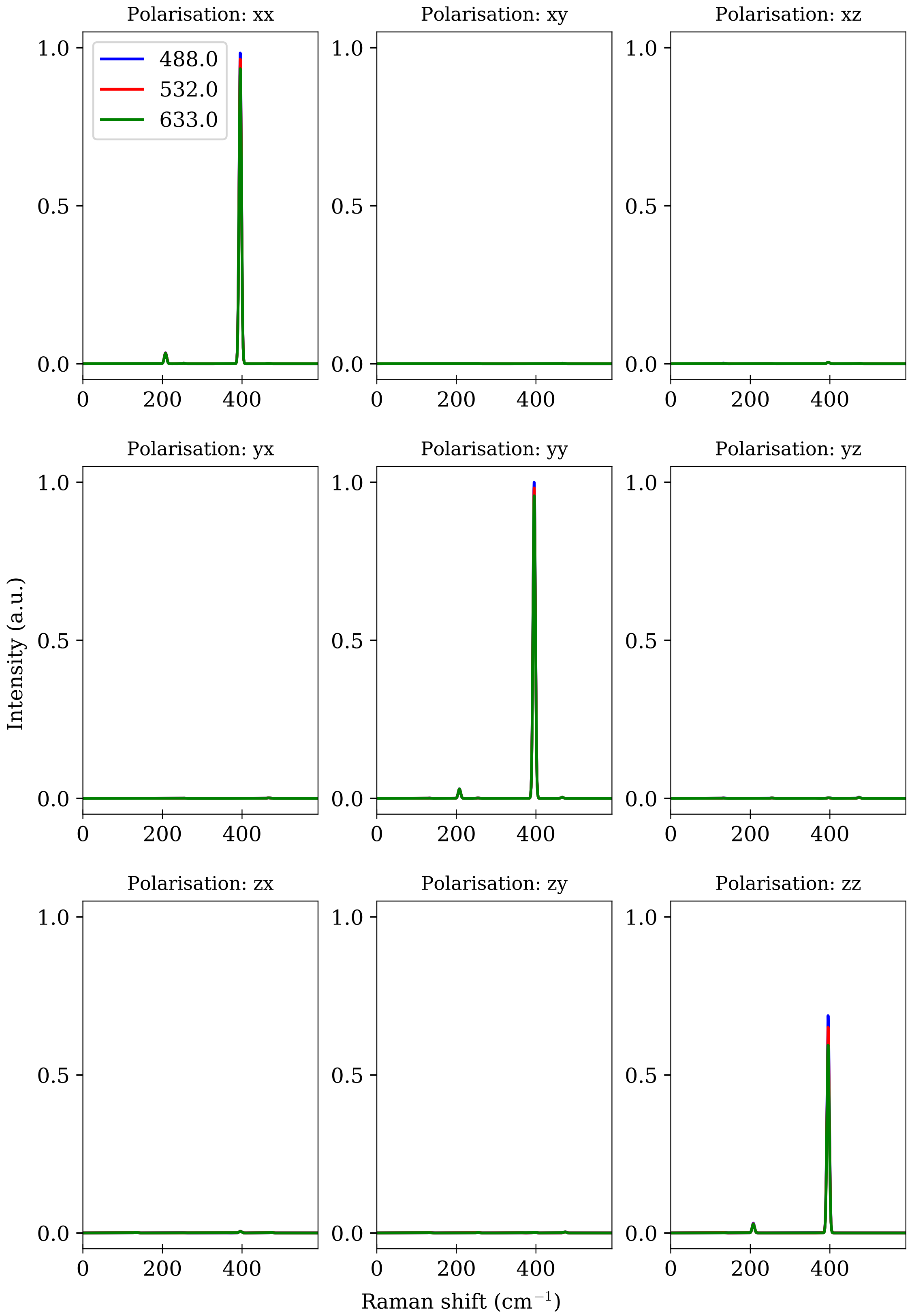

Supplementary Figure 663: Raman spectrum of Y_2F_2N with space group 164 and Wyckoff positions b-d-0.

$Y_2H_2NO_2$, 164, b-d-0

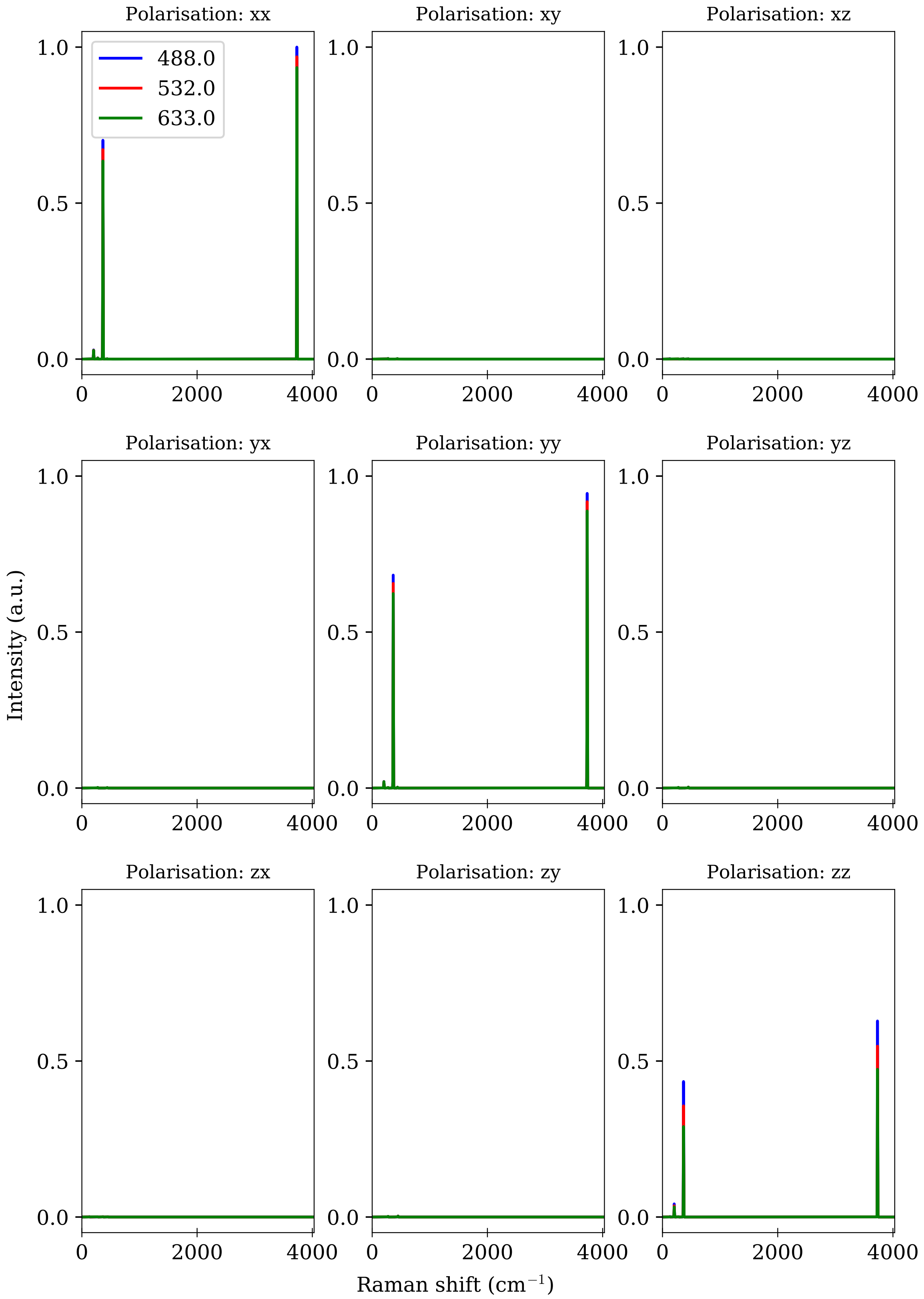

Supplementary Figure 664: Raman spectrum of $Y_2H_2NO_2$ with space group 164 and Wyckoff positions b-d-0.

Y_2N, 164, b-d-0

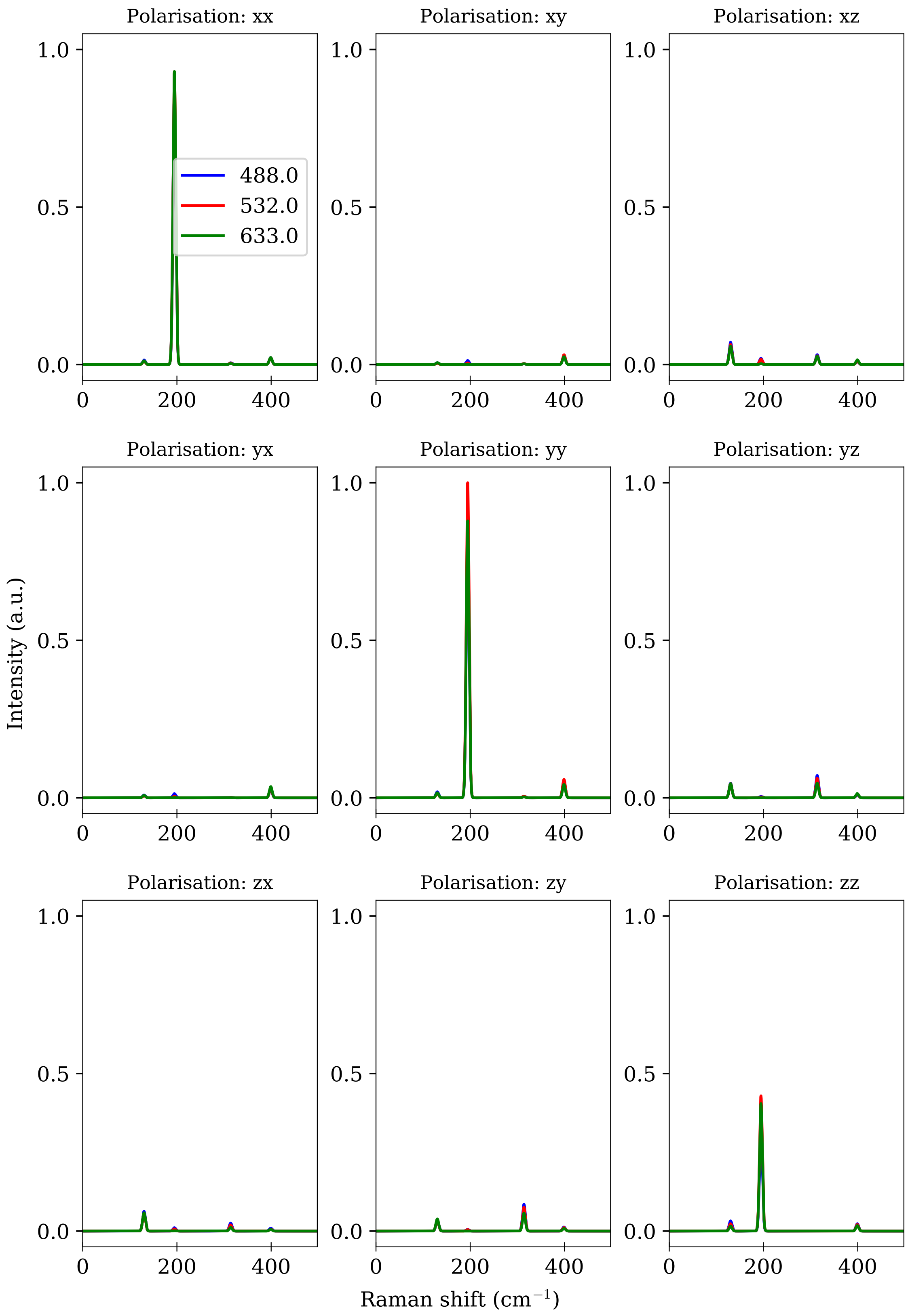

Supplementary Figure 665: Raman spectrum of Y_2N with space group 164 and Wyckoff positions b-d-0.

$Y_3C_2H_2O_2$, 187, a-g-h-i-0

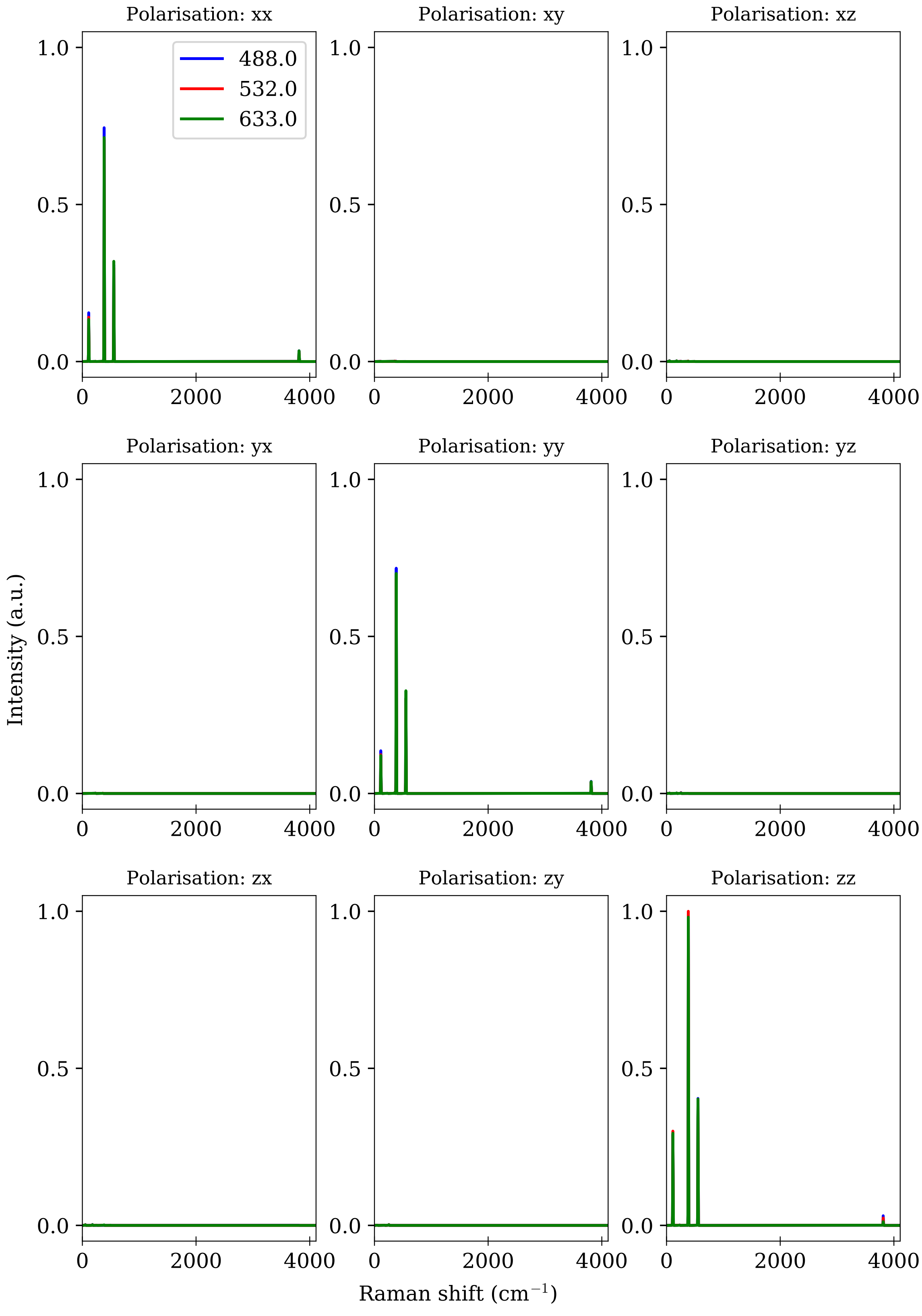

Supplementary Figure 666: Raman spectrum of $Y_3C_2H_2O_2$ with space group 187 and Wyckoff positions a-g-h-i-0.

$Y_3C_2O_2$, 187, b-g-h-i-0

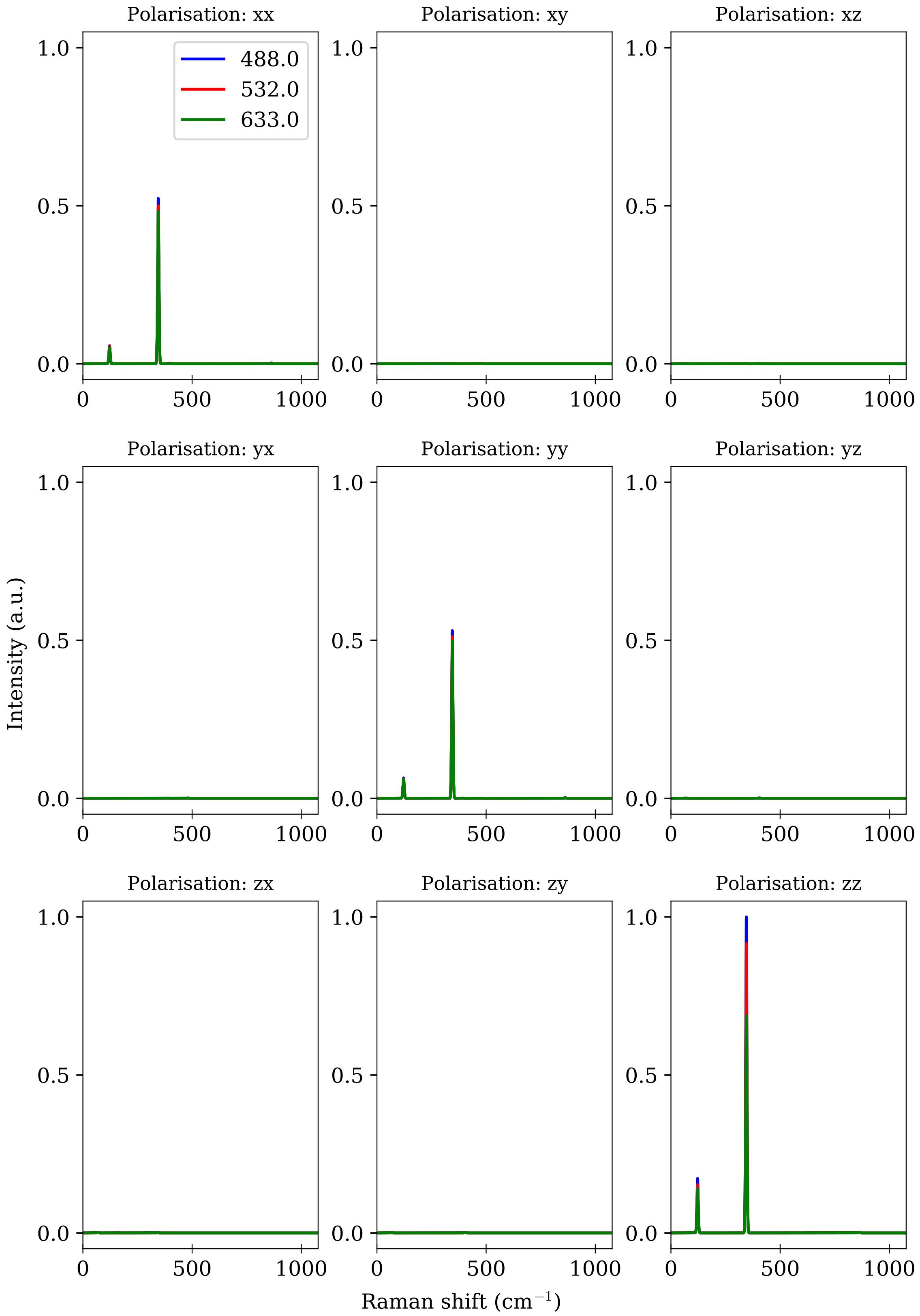

Supplementary Figure 667: Raman spectrum of $Y_3C_2O_2$ with space group 187 and Wyckoff positions b-g-h-i-0.

Y_3C_2, 187, b-h-i-0

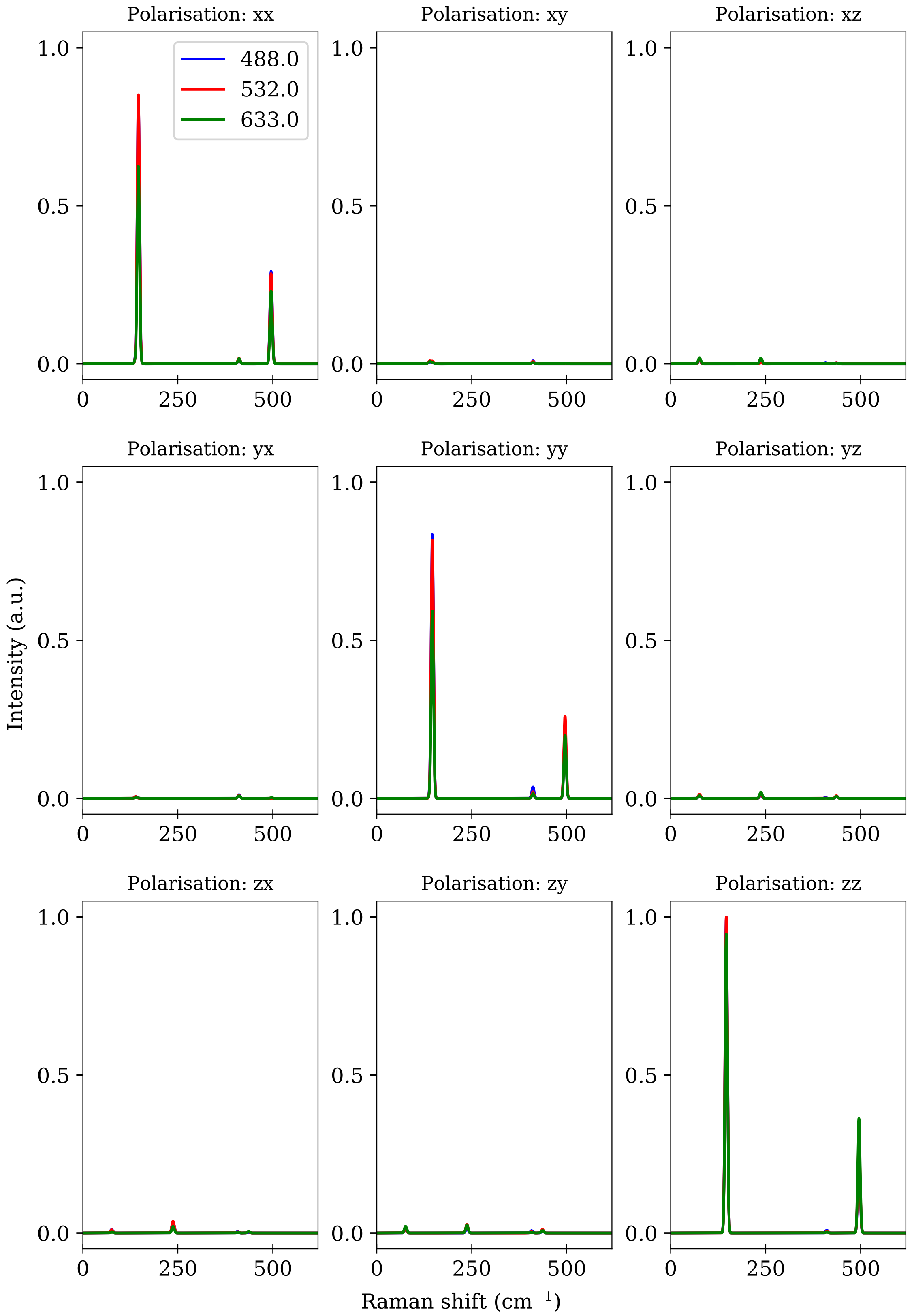

Supplementary Figure 668: Raman spectrum of Y_3C_2 with space group 187 and Wyckoff positions b-h-i-0.

$Y_3F_2N_2$, 187, b-g-h-i-0

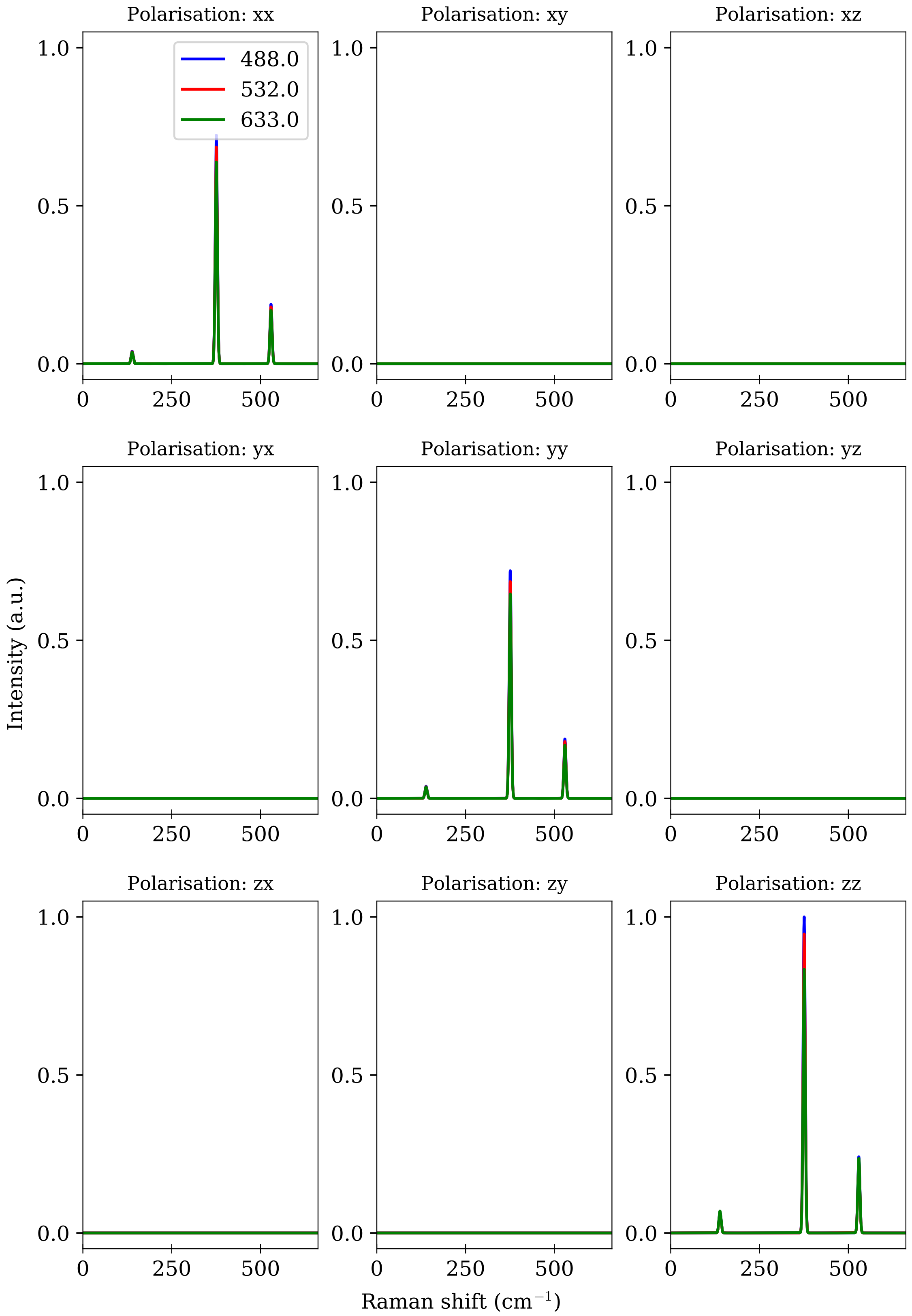

Supplementary Figure 669: Raman spectrum of $Y_3F_2N_2$ with space group 187 and Wyckoff positions b-g-h-i-0.

$Y_3H_2N_2O_2$, 187, b-g-h-i-0

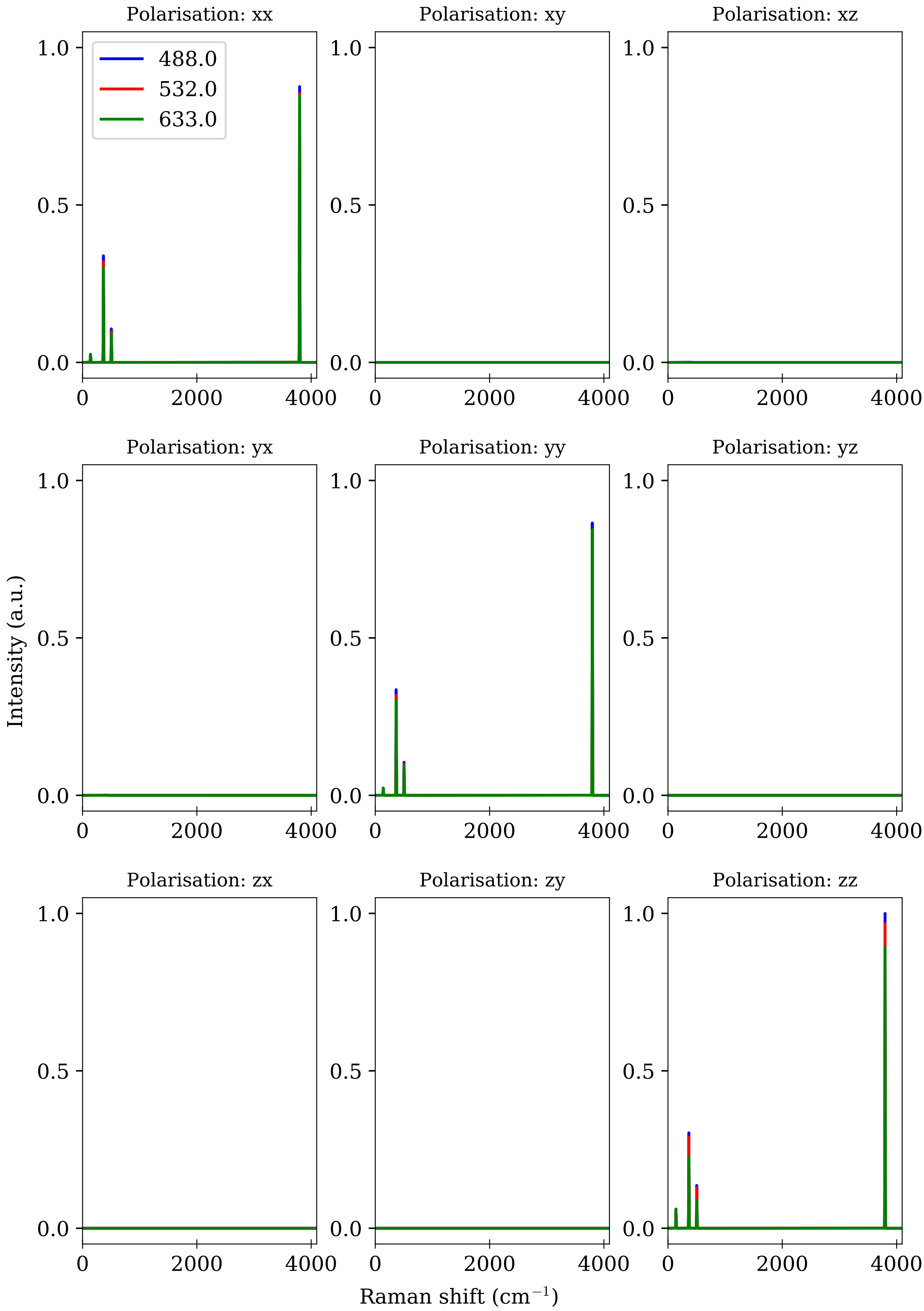

Supplementary Figure 670: Raman spectrum of $Y_3H_2N_2O_2$ with space group 187 and Wyckoff positions b-g-h-i-0.

Y_3N_2, 187, b-h-i-0

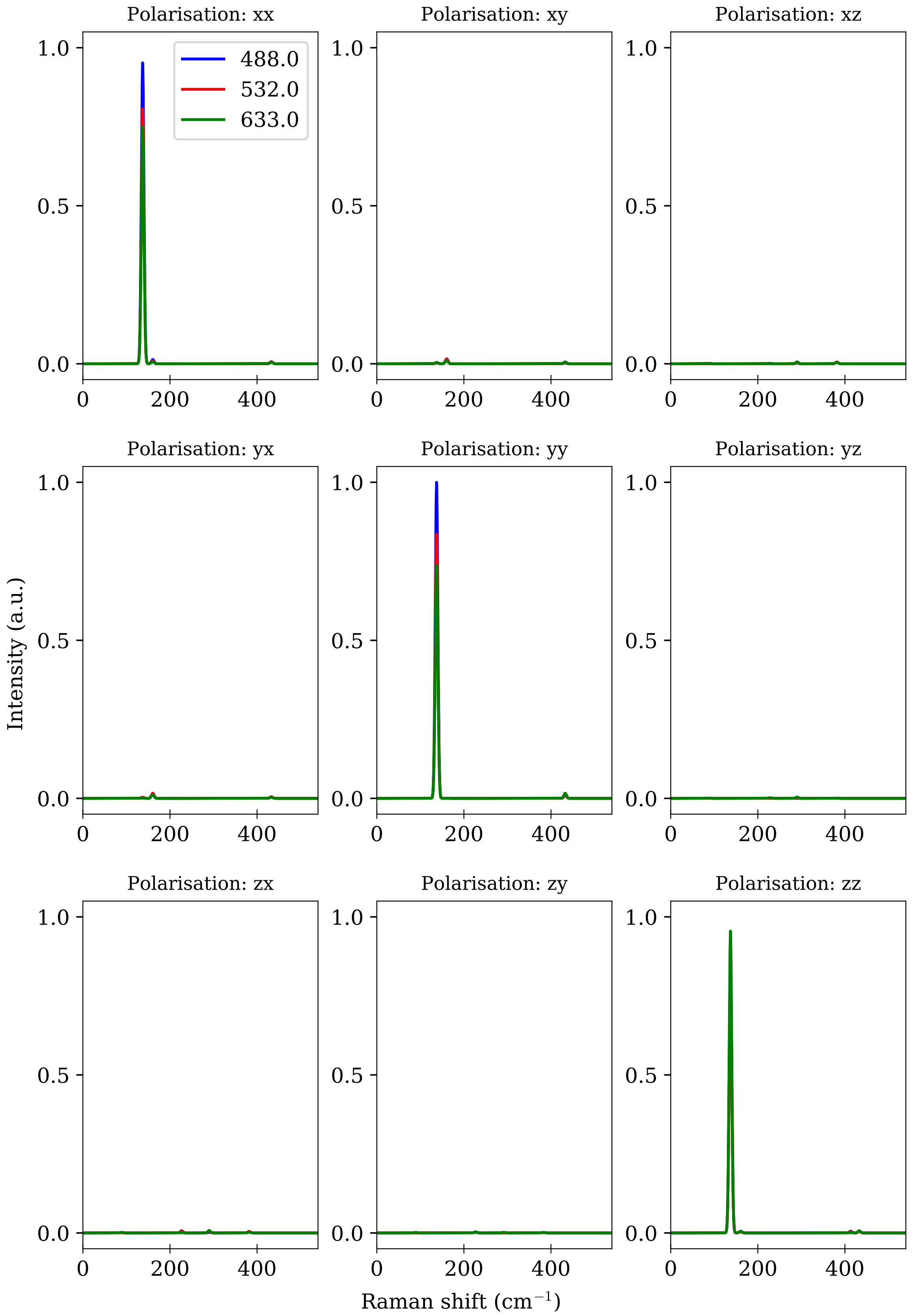

Supplementary Figure 671: Raman spectrum of Y_3N_2 with space group 187 and Wyckoff positions b-h-i-0.

Y_4C_3, 164, b-c-d-0

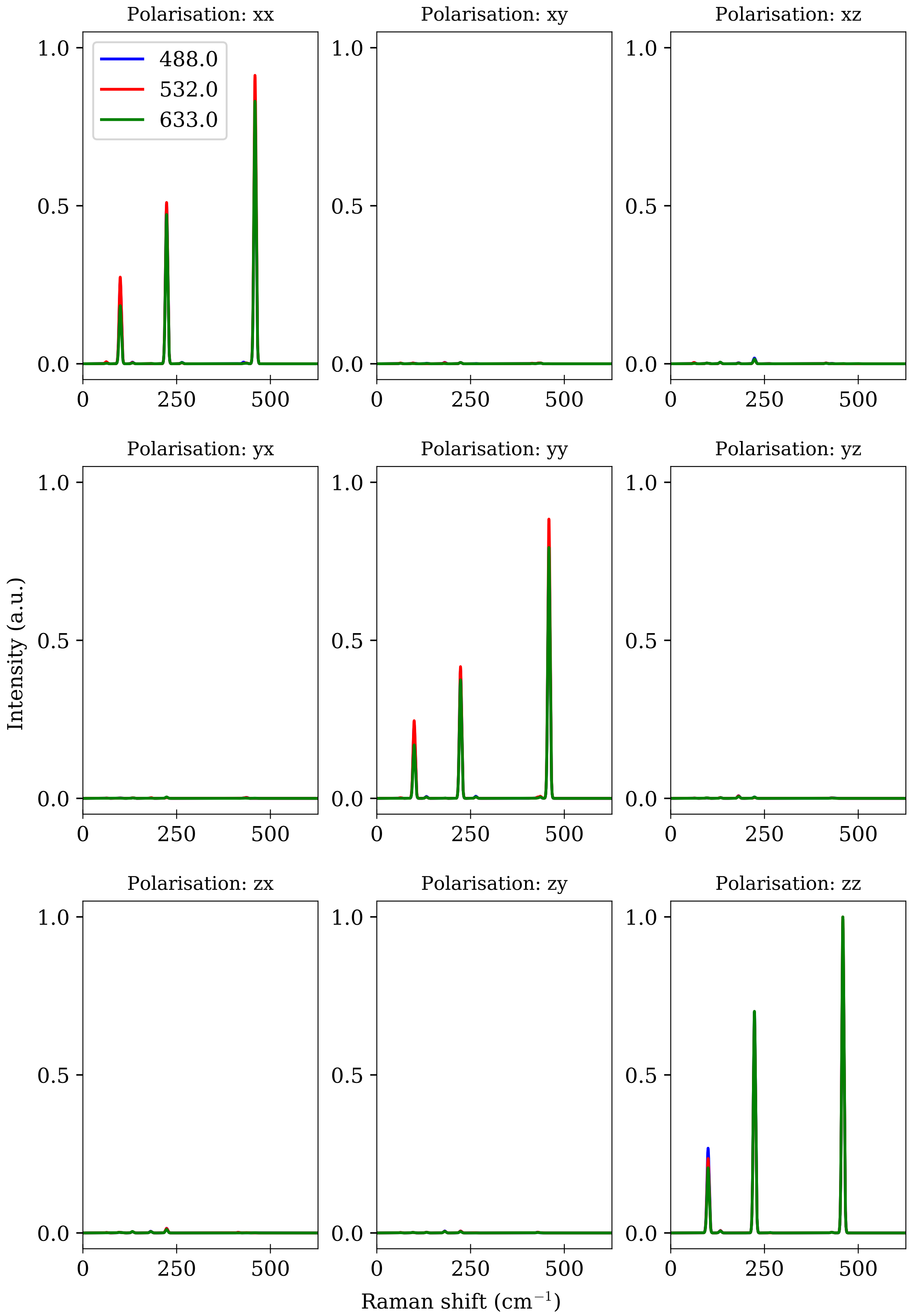

Supplementary Figure 672: Raman spectrum of Y_4C_3 with space group 164 and Wyckoff positions b-c-d-0.

Y_4N_3, 164, b-c-d-0

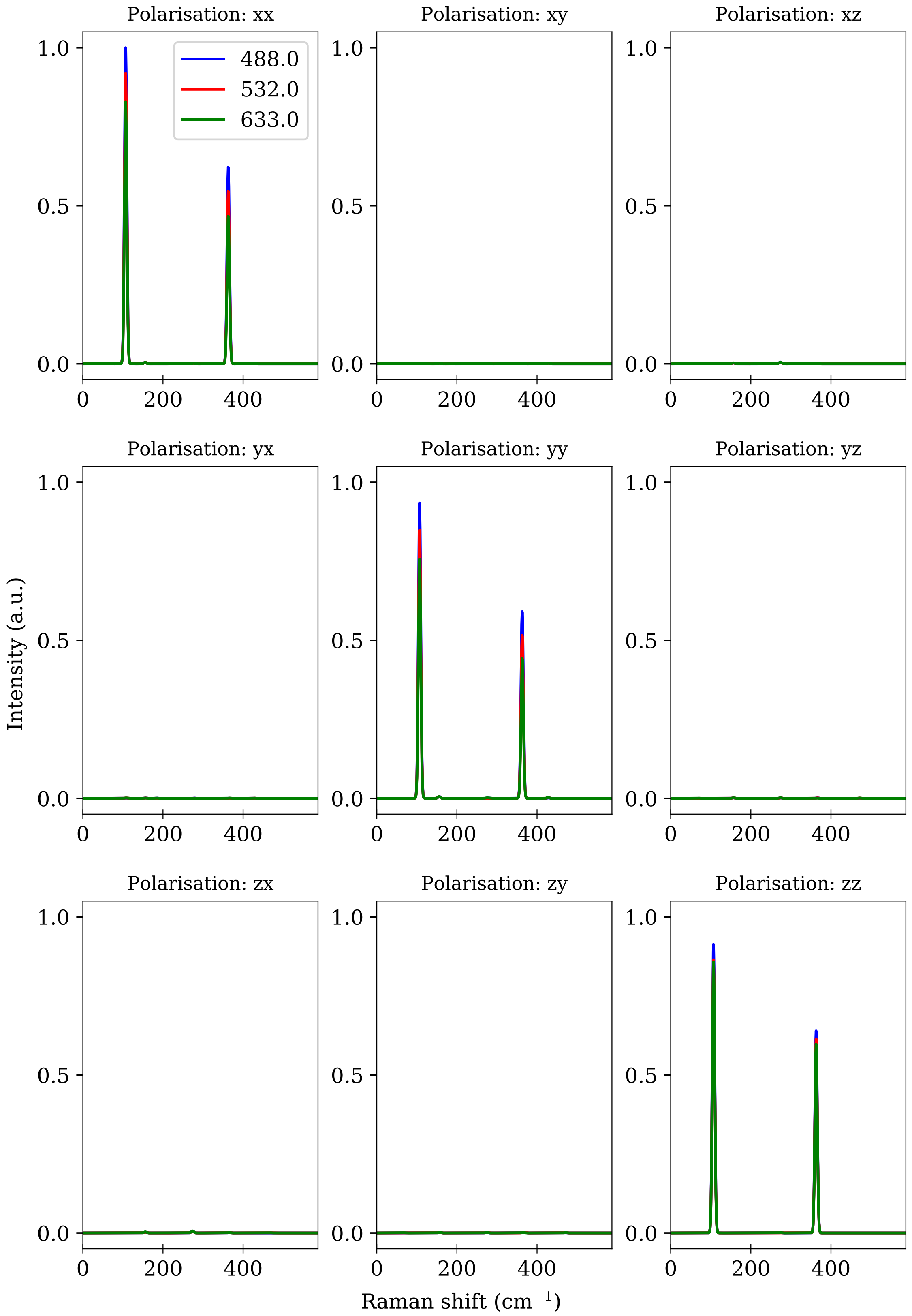

Supplementary Figure 673: Raman spectrum of Y_4N_3 with space group 164 and Wyckoff positions b-c-d-0.

$Zn_2P_2S_6$, 2, i-0

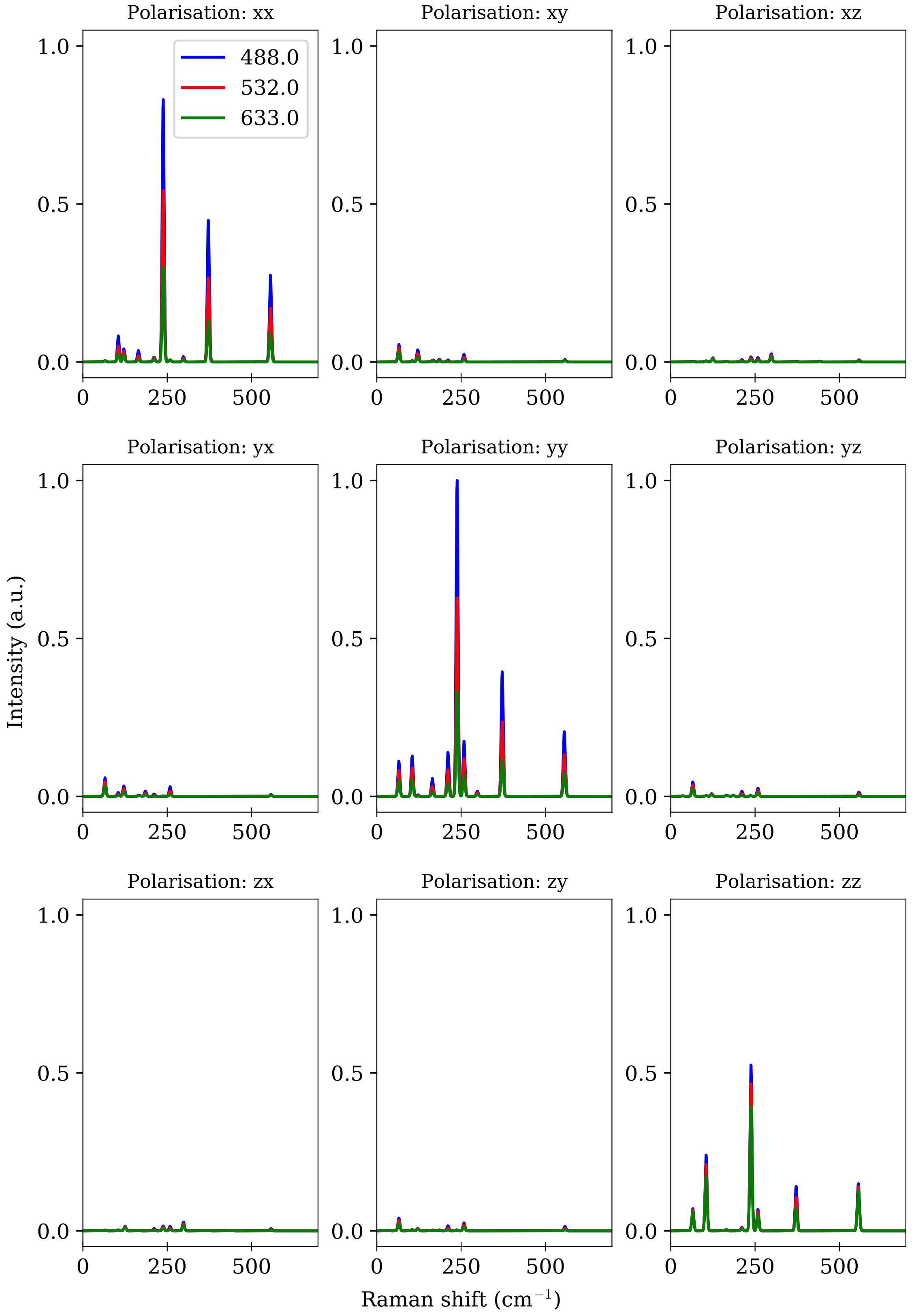

Supplementary Figure 674: Raman spectrum of $Zn_2P_2S_6$ with space group 2 and Wyckoff positions i-0.

Zn_2S_2, 164, d-0

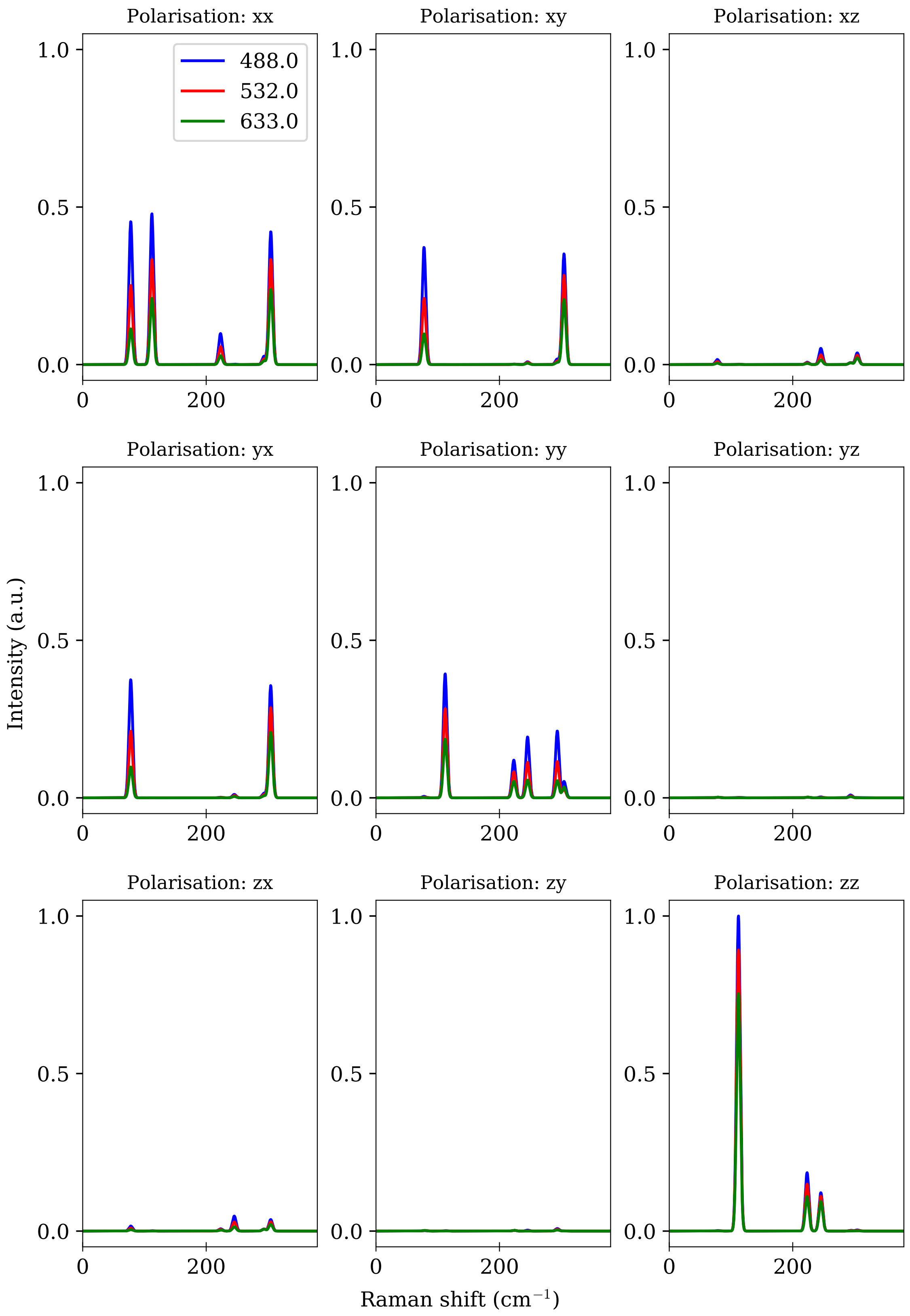

Supplementary Figure 675: Raman spectrum of Zn_2S_2 with space group 164 and Wyckoff positions d-0.

Zn_2S_4, 14, c-e-0

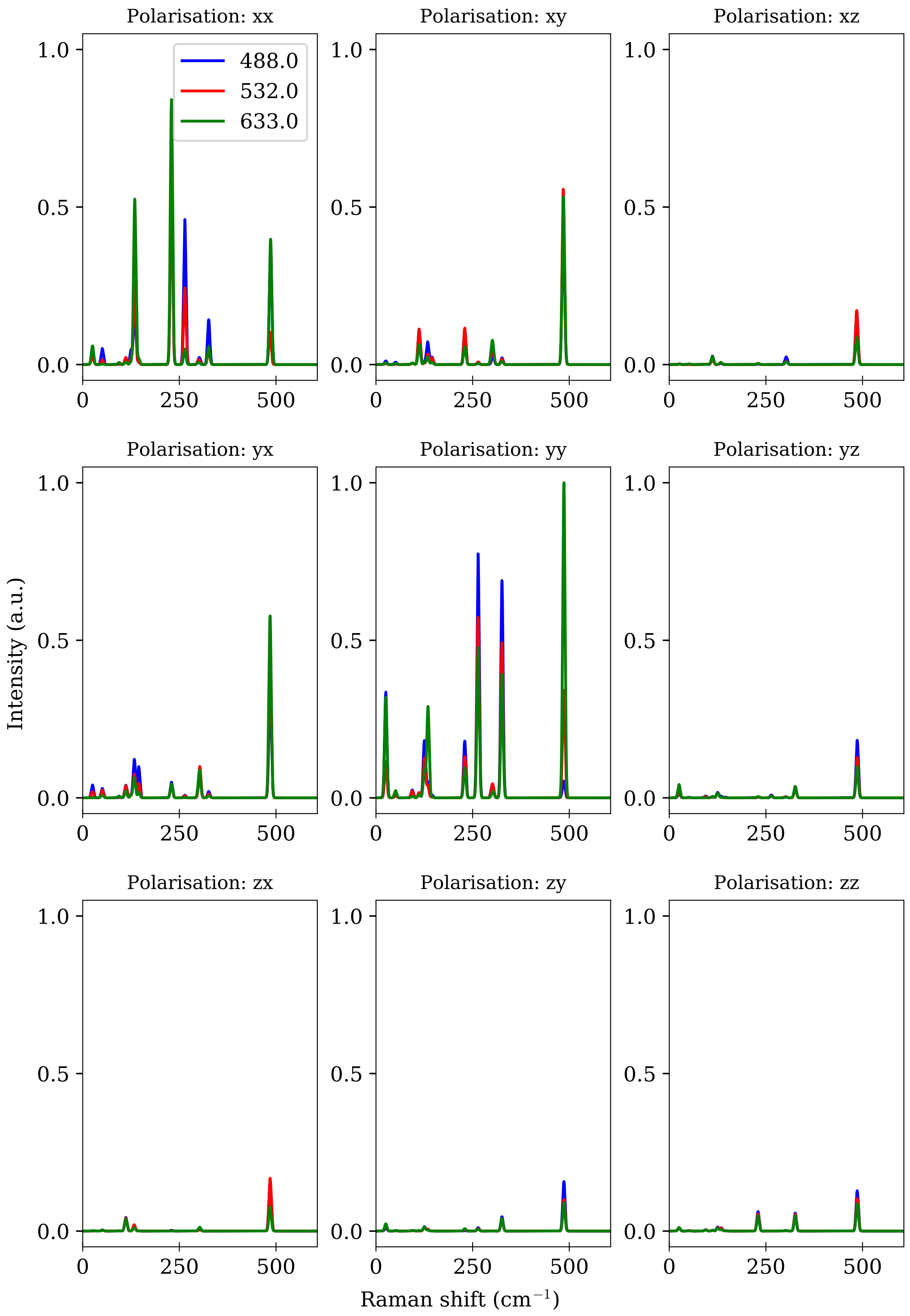

Supplementary Figure 676: Raman spectrum of Zn_2S_4 with space group 14 and Wyckoff positions c-e-0.

Zn_2Se_2, 164, d-0

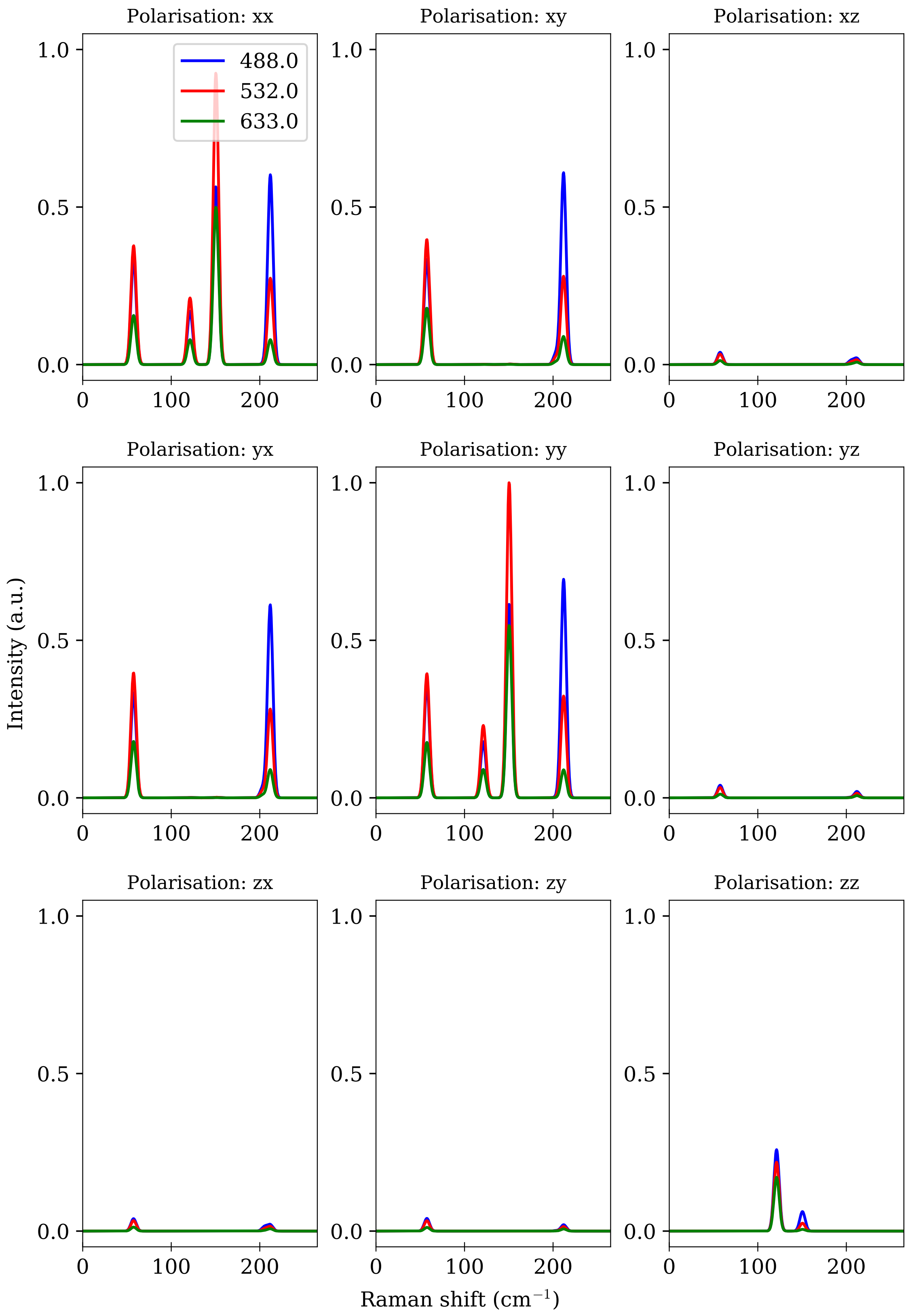

Supplementary Figure 677: Raman spectrum of Zn_2Se_2 with space group 164 and Wyckoff positions d-0.

Zn_2Se_4, 14, b-e-0

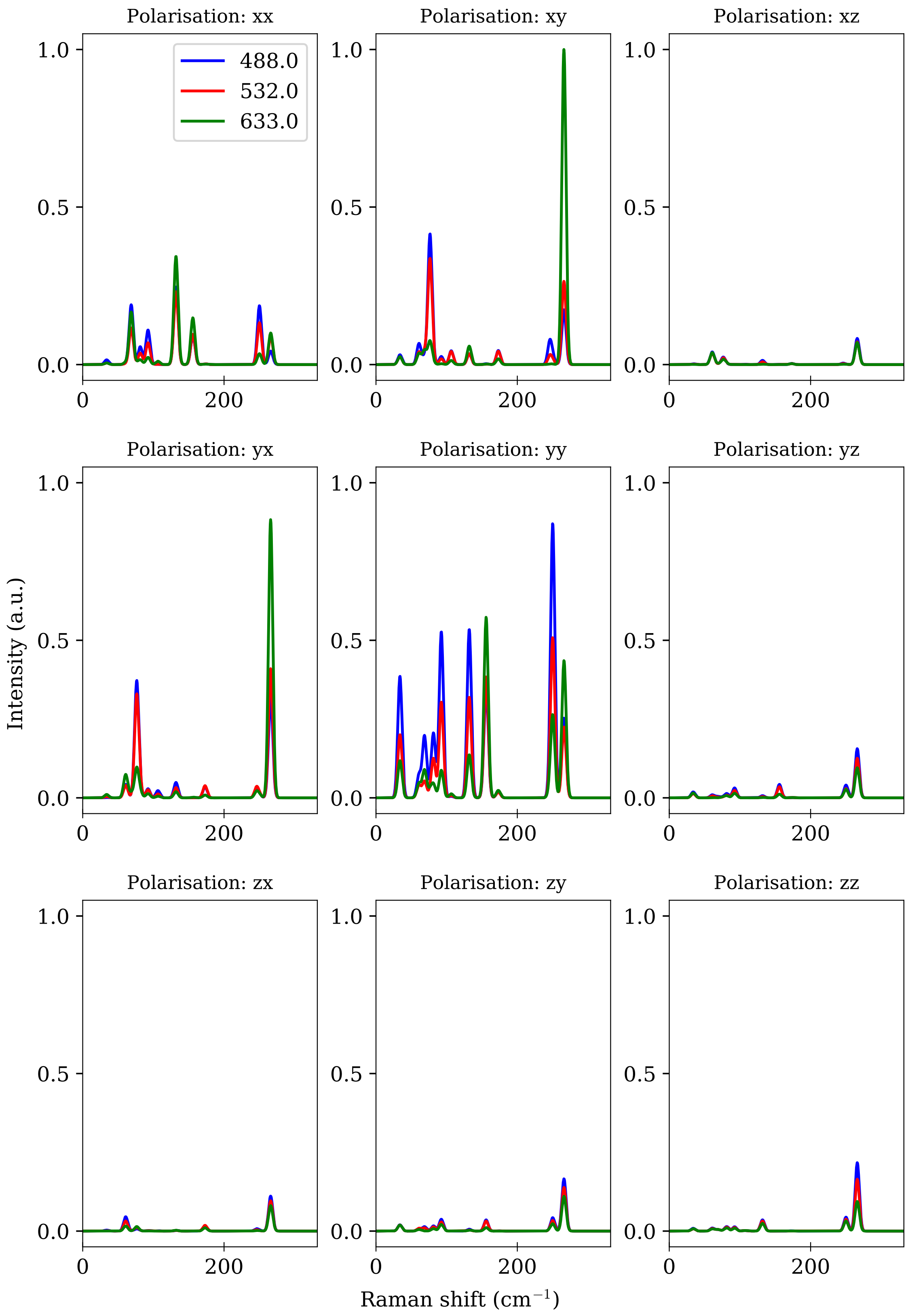

Supplementary Figure 678: Raman spectrum of Zn_2Se_4 with space group 14 and Wyckoff positions b-e-0.

Zn_2Te_2, 164, d-0

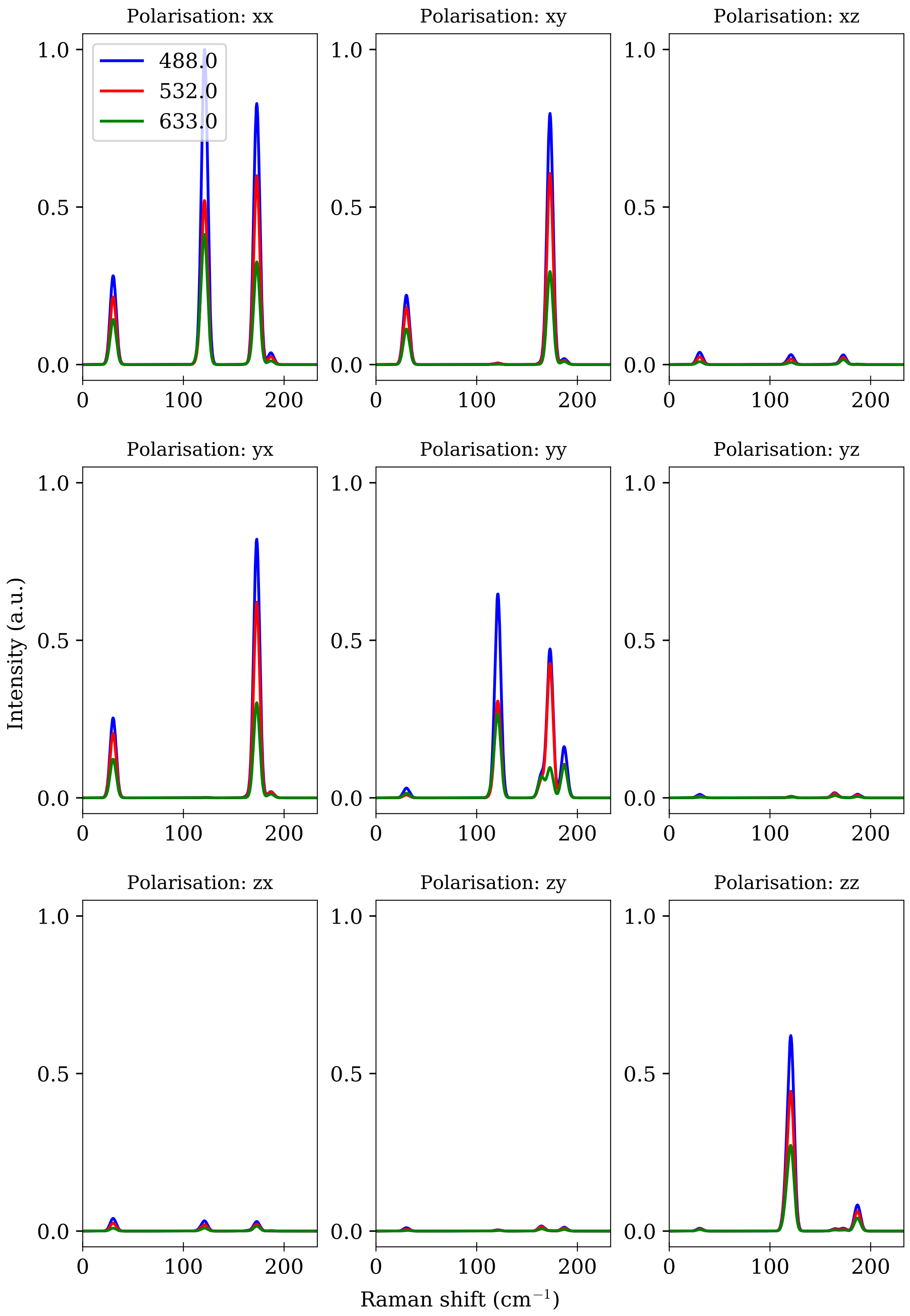

Supplementary Figure 679: Raman spectrum of Zn_2Te_2 with space group 164 and Wyckoff positions d-0.

$ZnBr_2$, 115, d-g-0

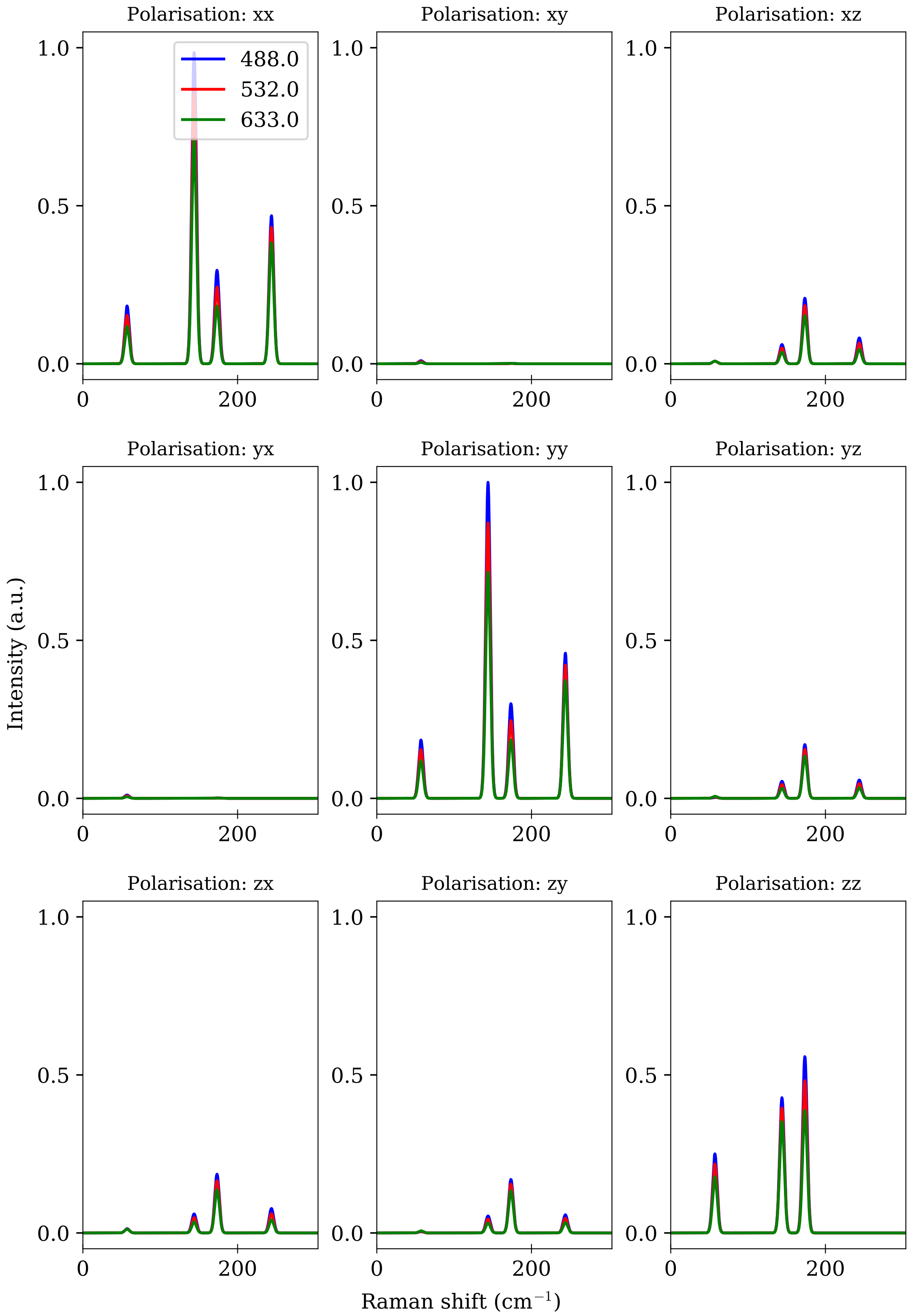

Supplementary Figure 680: Raman spectrum of $ZnBr_2$ with space group 115 and Wyckoff positions d-g-0.

$ZnBr_2$, 164, b-d-0

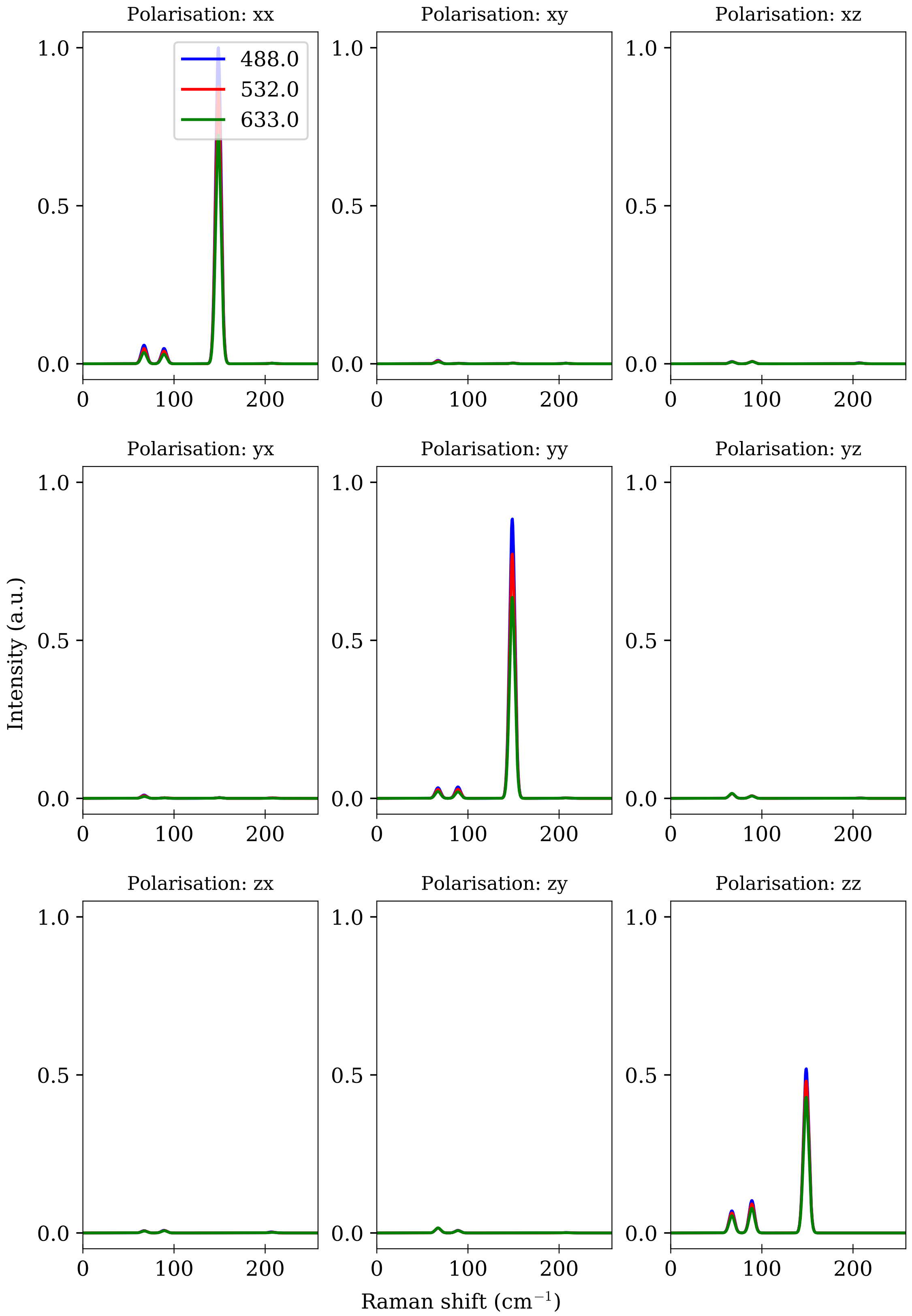

Supplementary Figure 681: Raman spectrum of $ZnBr_2$ with space group 164 and Wyckoff positions b-d-0.

$ZnCl_2$, 115, d-g-0

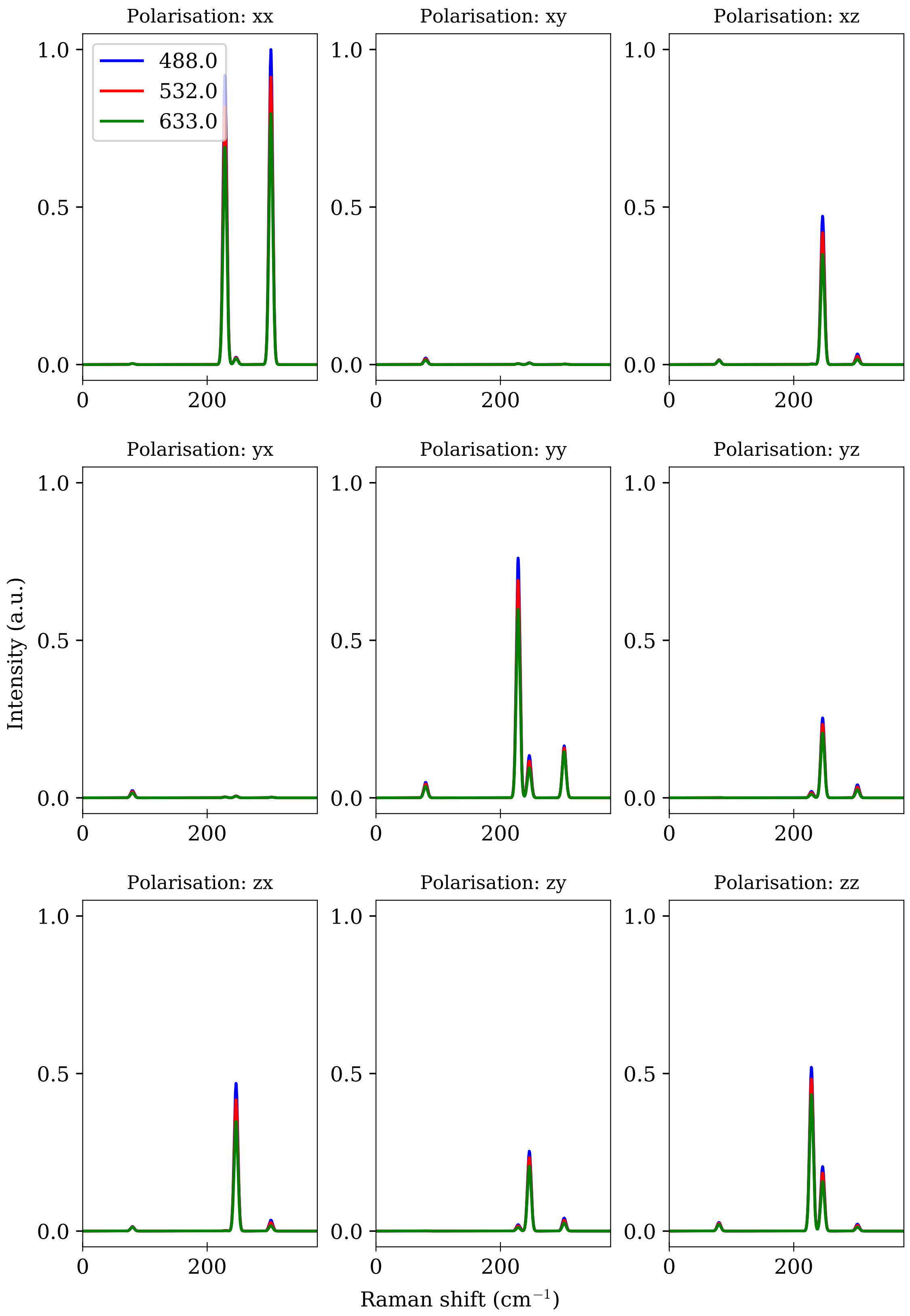

Supplementary Figure 682: Raman spectrum of $ZnCl_2$ with space group 115 and Wyckoff positions d-g-0.

$ZnCl_2$, 164, b-d-0

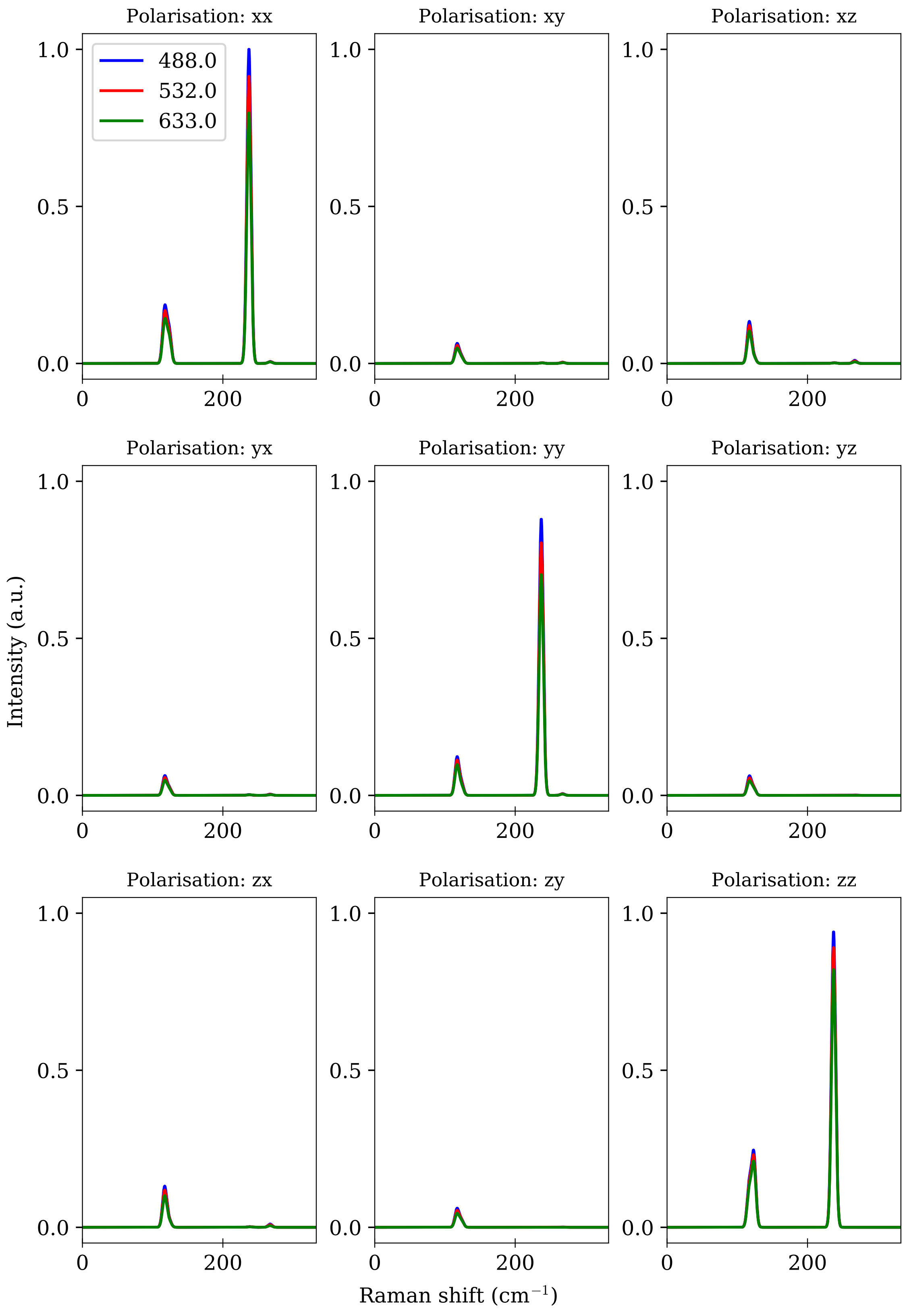

Supplementary Figure 683: Raman spectrum of $ZnCl_2$ with space group 164 and Wyckoff positions b-d-0.

ZnF_2, 115, d-g-0

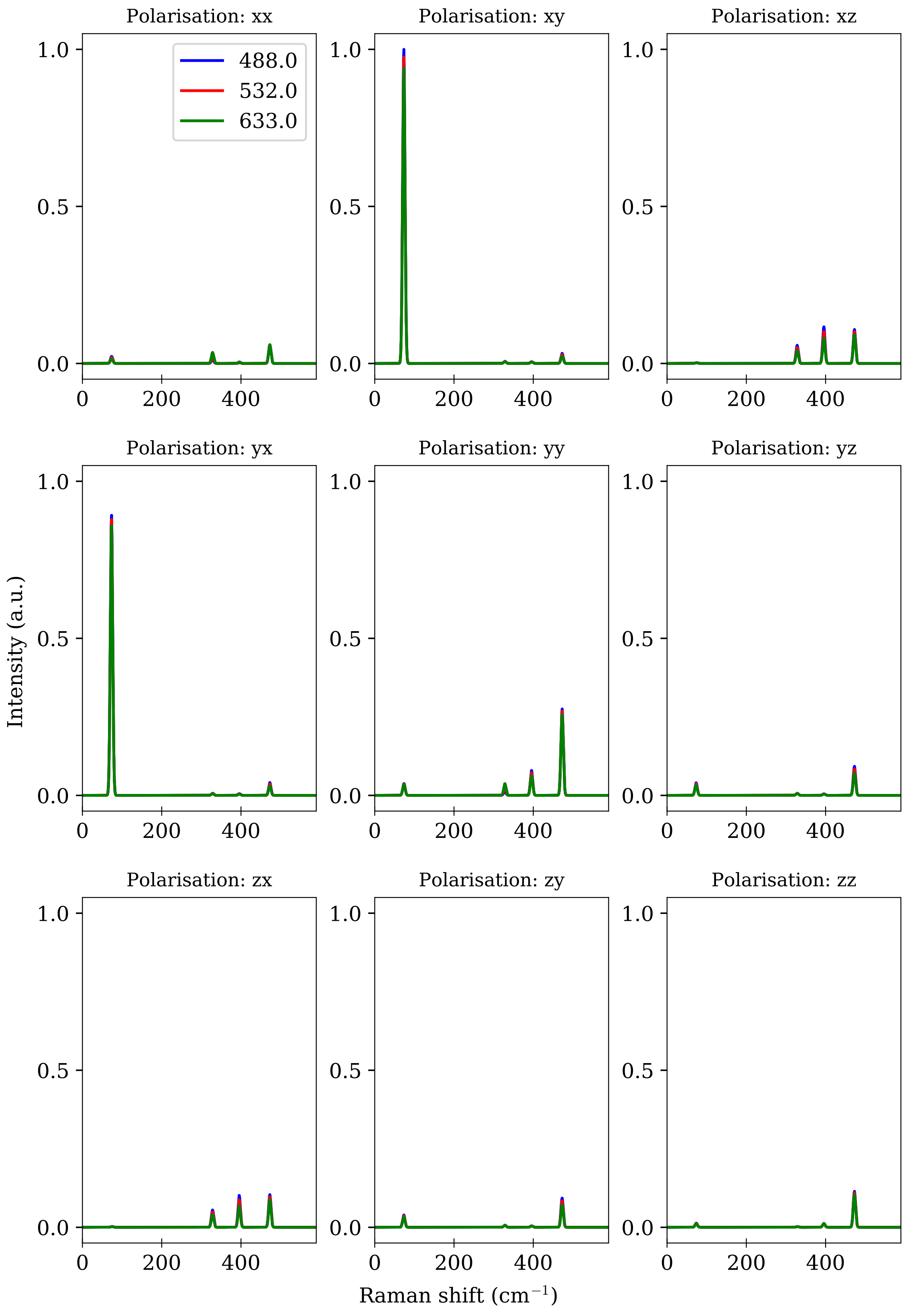

Supplementary Figure 684: Raman spectrum of ZnF_2 with space group 115 and Wyckoff positions d-g-0.

ZnI_2, 115, d-g-0

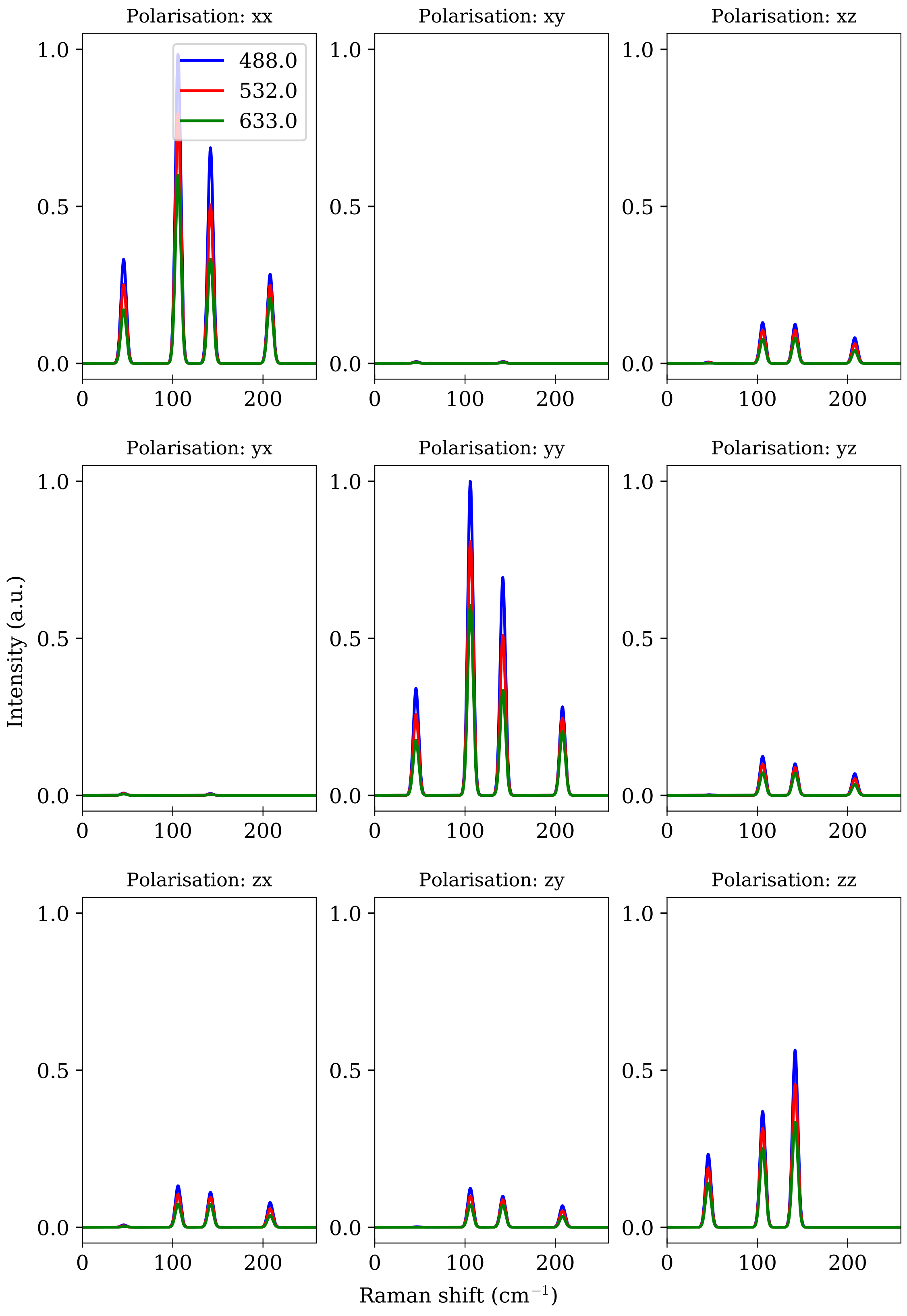

Supplementary Figure 685: Raman spectrum of ZnI_2 with space group 115 and Wyckoff positions d-g-0.

ZnI_2, 164, b-d-0

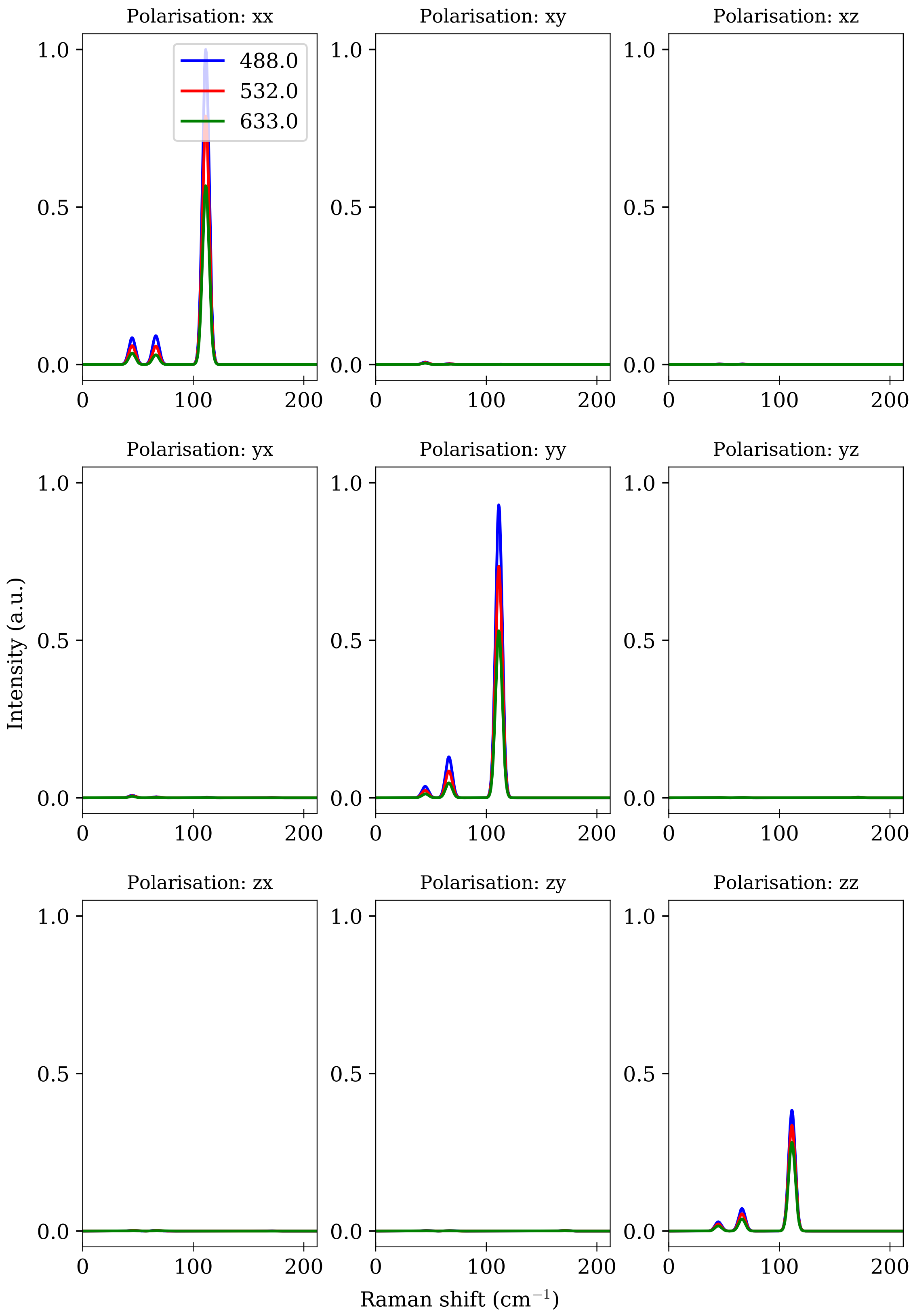

Supplementary Figure 686: Raman spectrum of ZnI_2 with space group 164 and Wyckoff positions b-d-0.

ZnS_2, 115, d-g-0

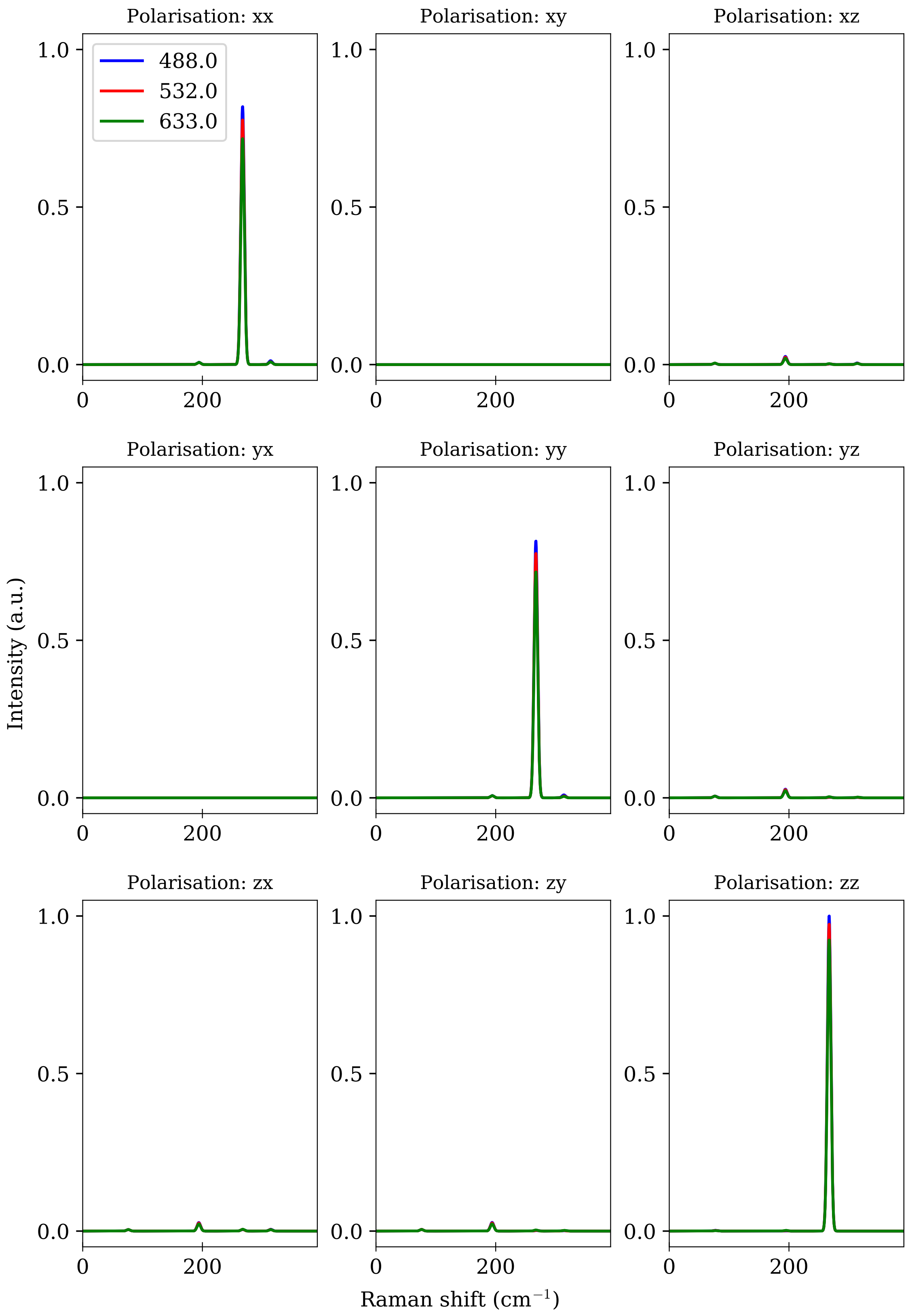

Supplementary Figure 687: Raman spectrum of ZnS_2 with space group 115 and Wyckoff positions d-g-0.

$ZnSe_2$, 115, d-g-0

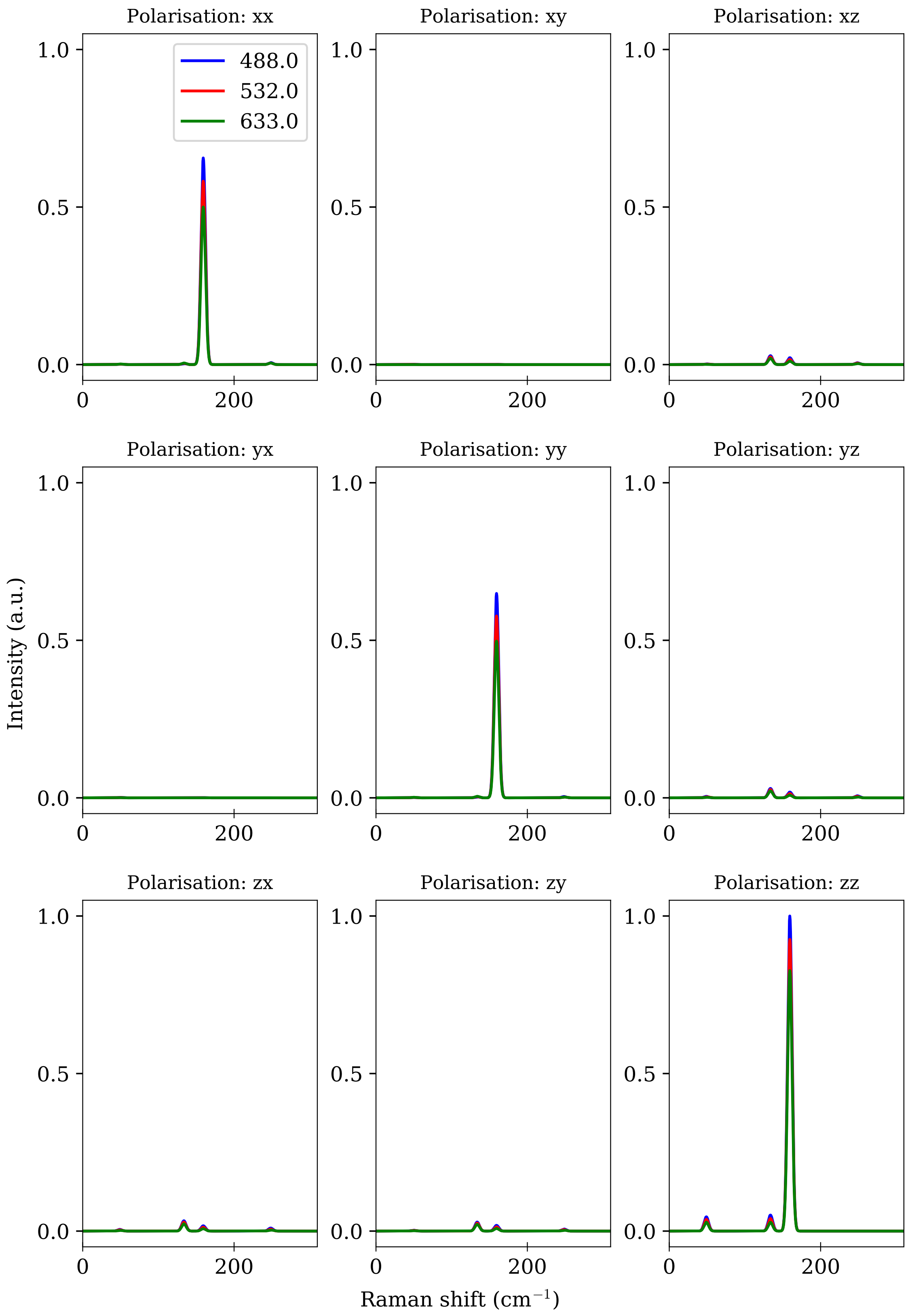

Supplementary Figure 688: Raman spectrum of $ZnSe_2$ with space group 115 and Wyckoff positions d-g-0.

$ZnTe_2$, 115, a-g-0

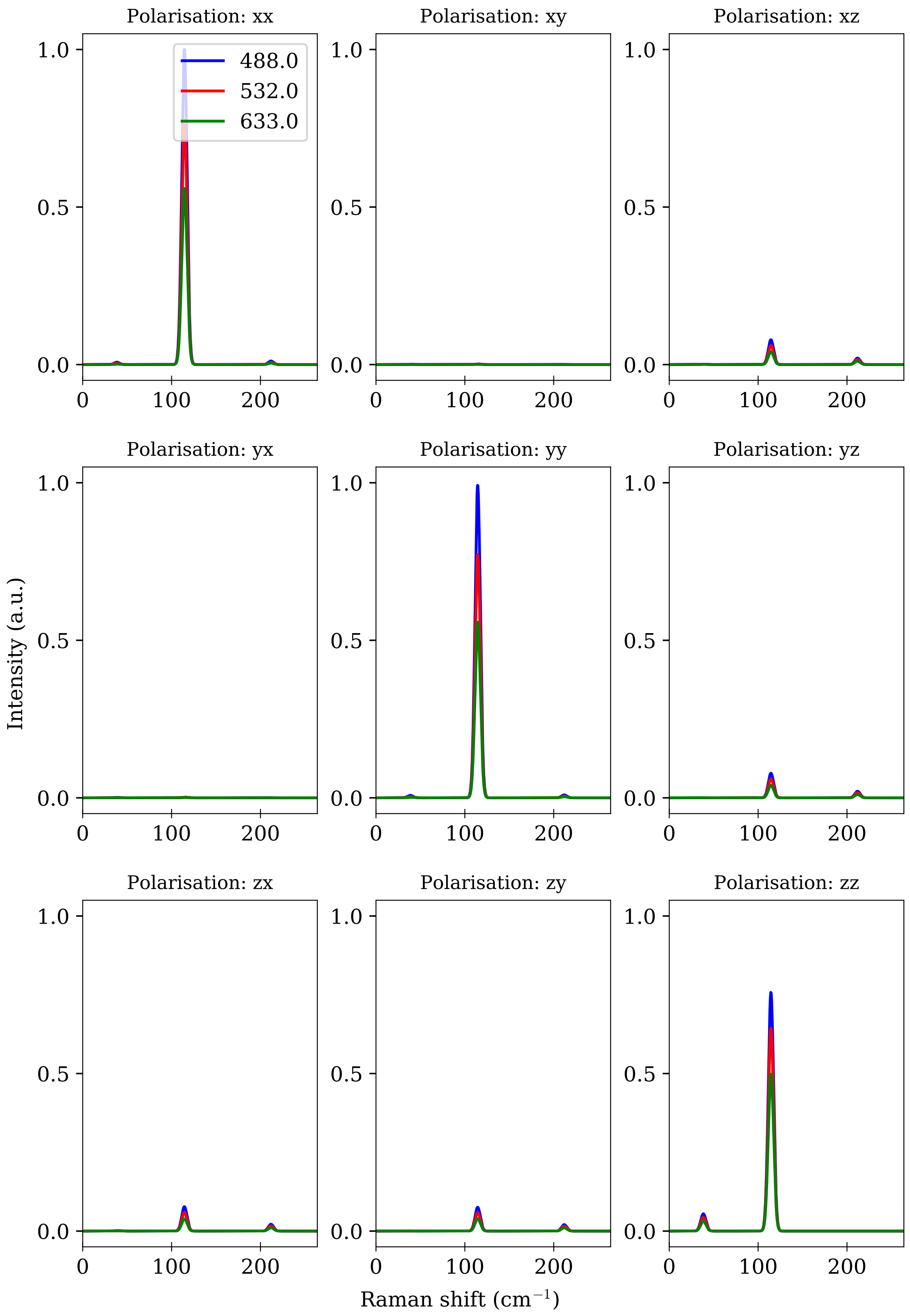

Supplementary Figure 689: Raman spectrum of $ZnTe_2$ with space group 115 and Wyckoff positions a-g-0.

Zr_2Br_4, 11, e-0

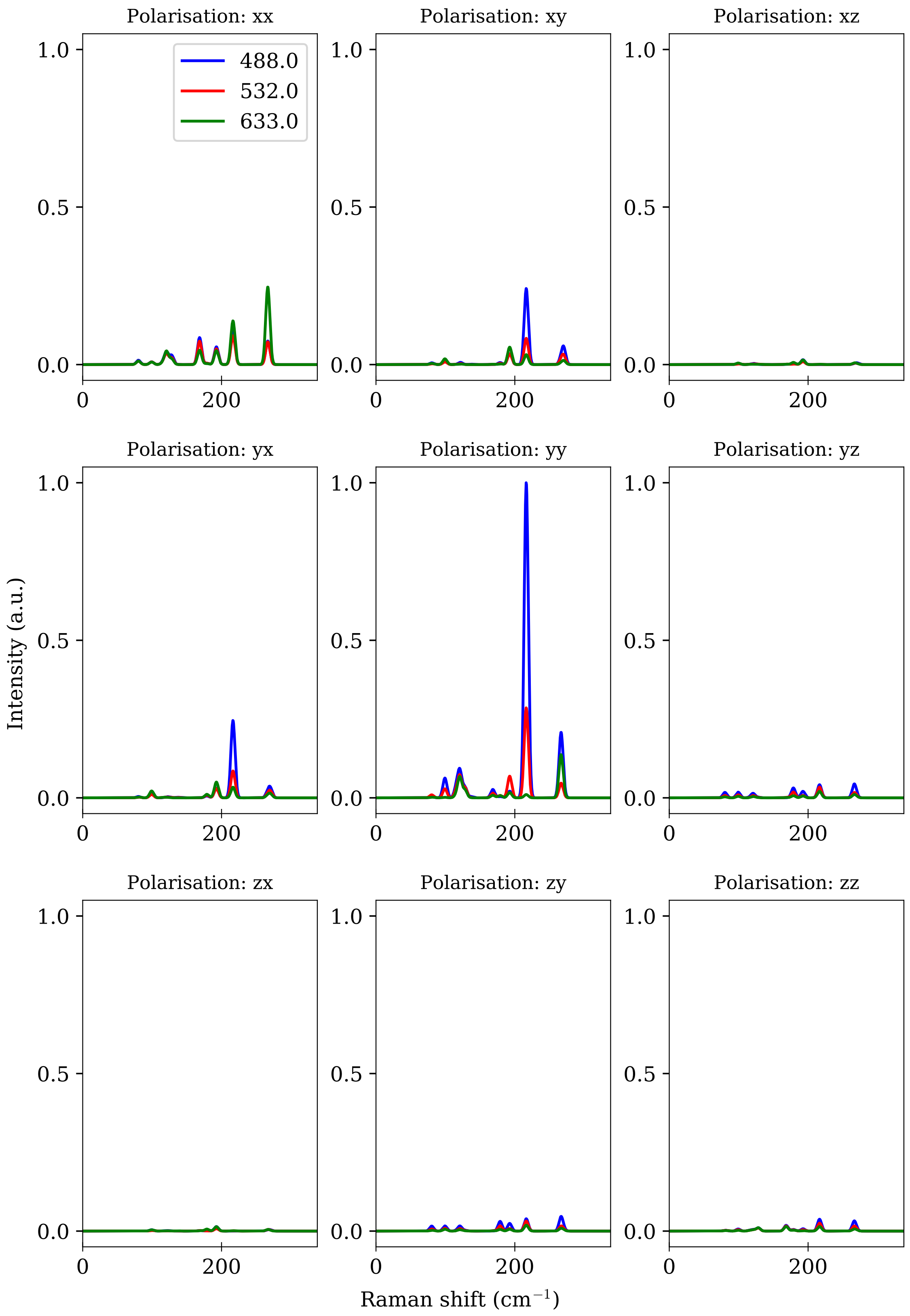

Supplementary Figure 690: Raman spectrum of Zr_2Br_4 with space group 11 and Wyckoff positions e-0.

Zr_2CF_2, 164, b-d-0

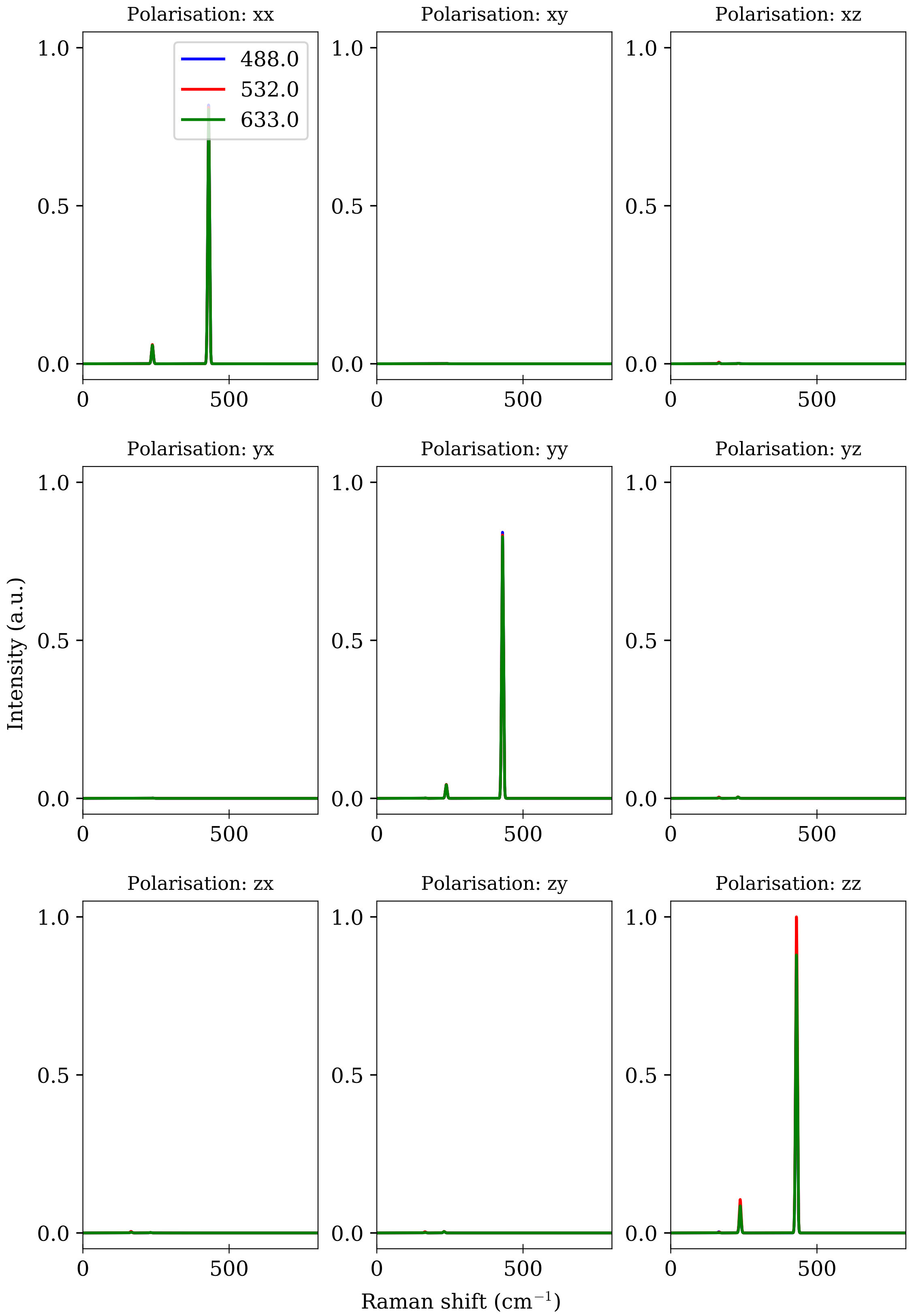

Supplementary Figure 691: Raman spectrum of Zr_2CF_2 with space group 164 and Wyckoff positions b-d-0.

$Zr_2CH_2O_2$, 164, b-d-0

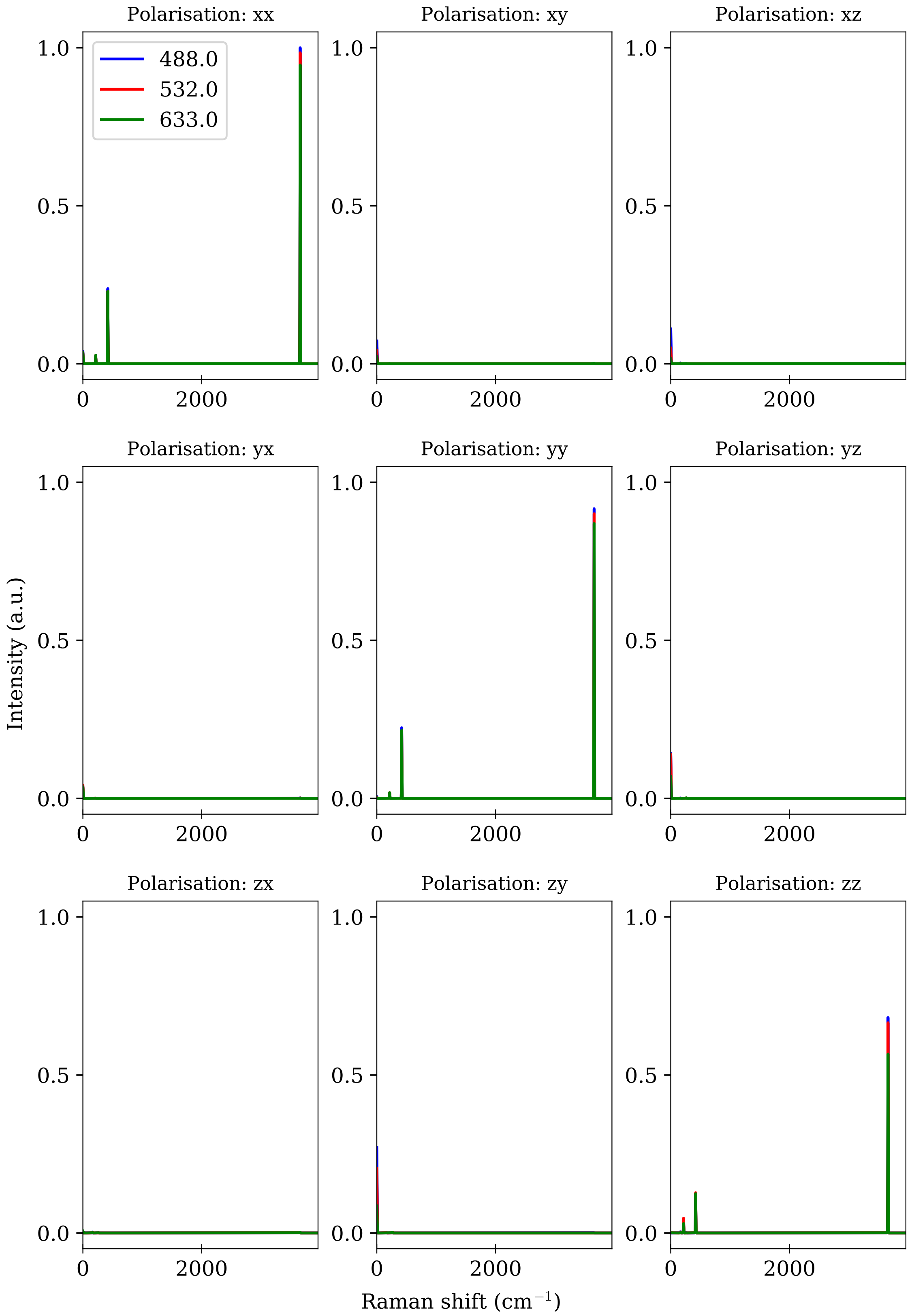

Supplementary Figure 692: Raman spectrum of $Zr_2CH_2O_2$ with space group 164 and Wyckoff positions b-d-0.

$Zr_2Cl_2S_2$, 59, a-b-0

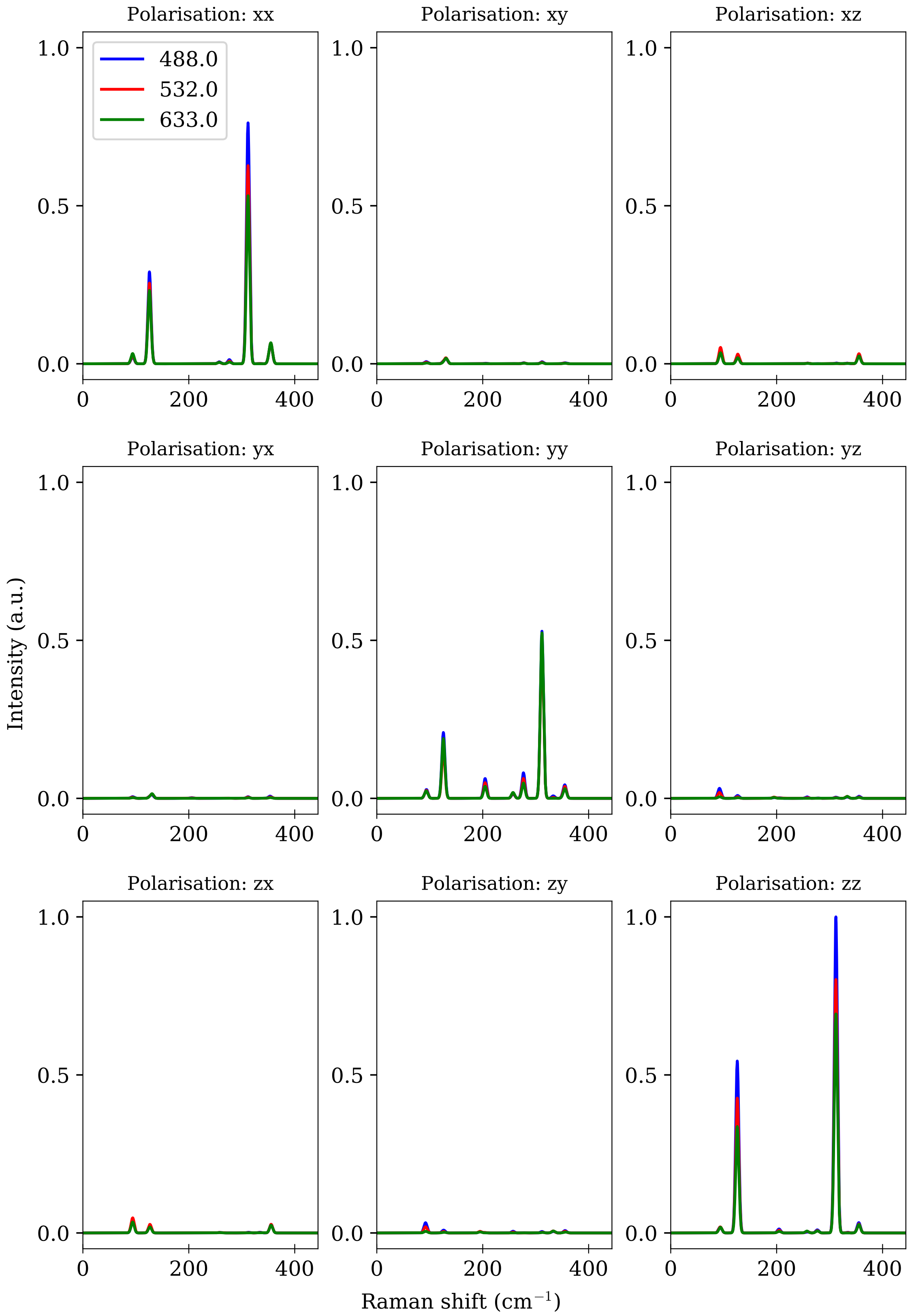

Supplementary Figure 693: Raman spectrum of $Zr_2Cl_2S_2$ with space group 59 and Wyckoff positions a-b-0.

Zr_2Cl_4, 11, e-0

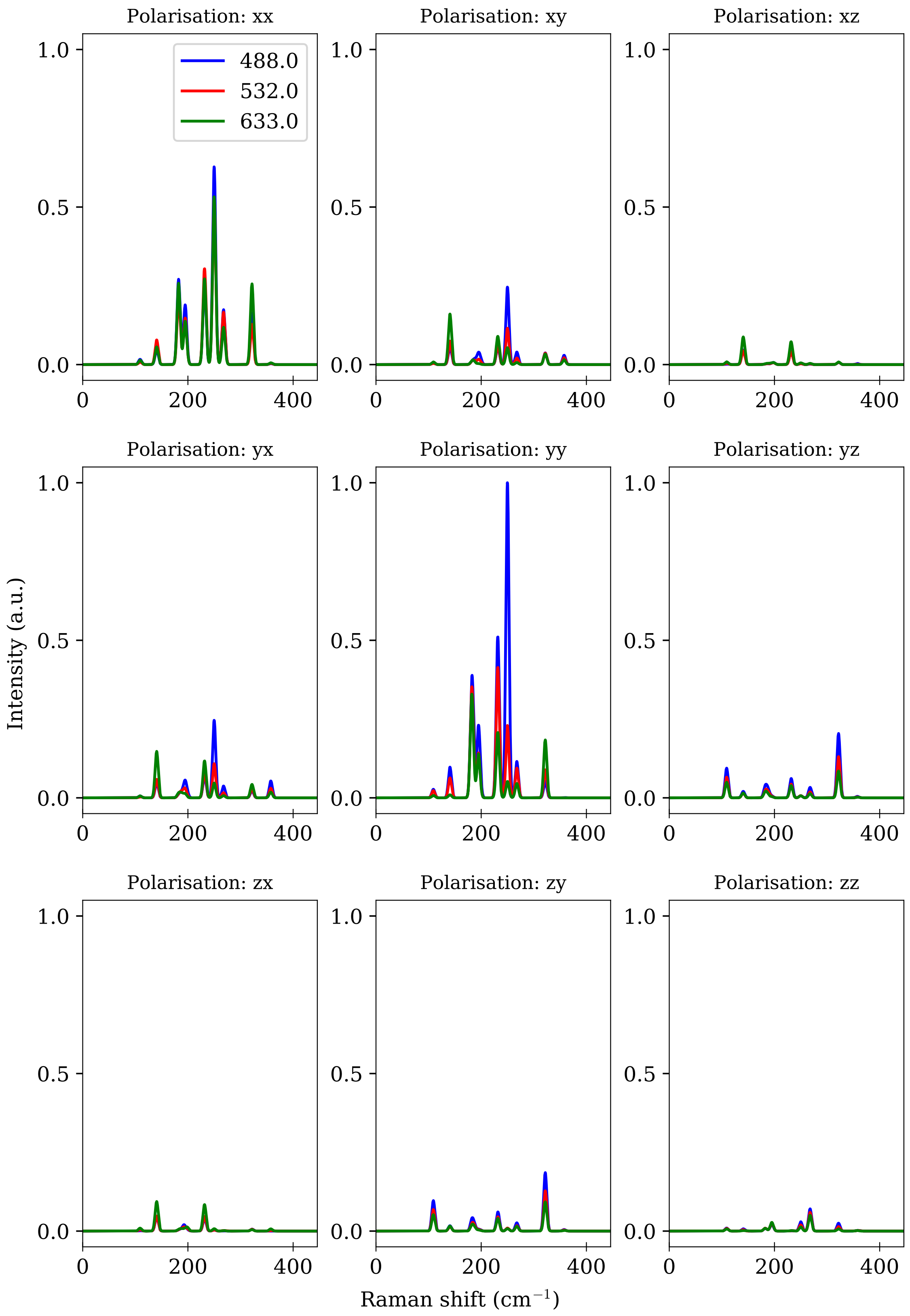

Supplementary Figure 694: Raman spectrum of Zr_2Cl_4 with space group 11 and Wyckoff positions e-0.

Zr_2CO_2, 164, b-d-0

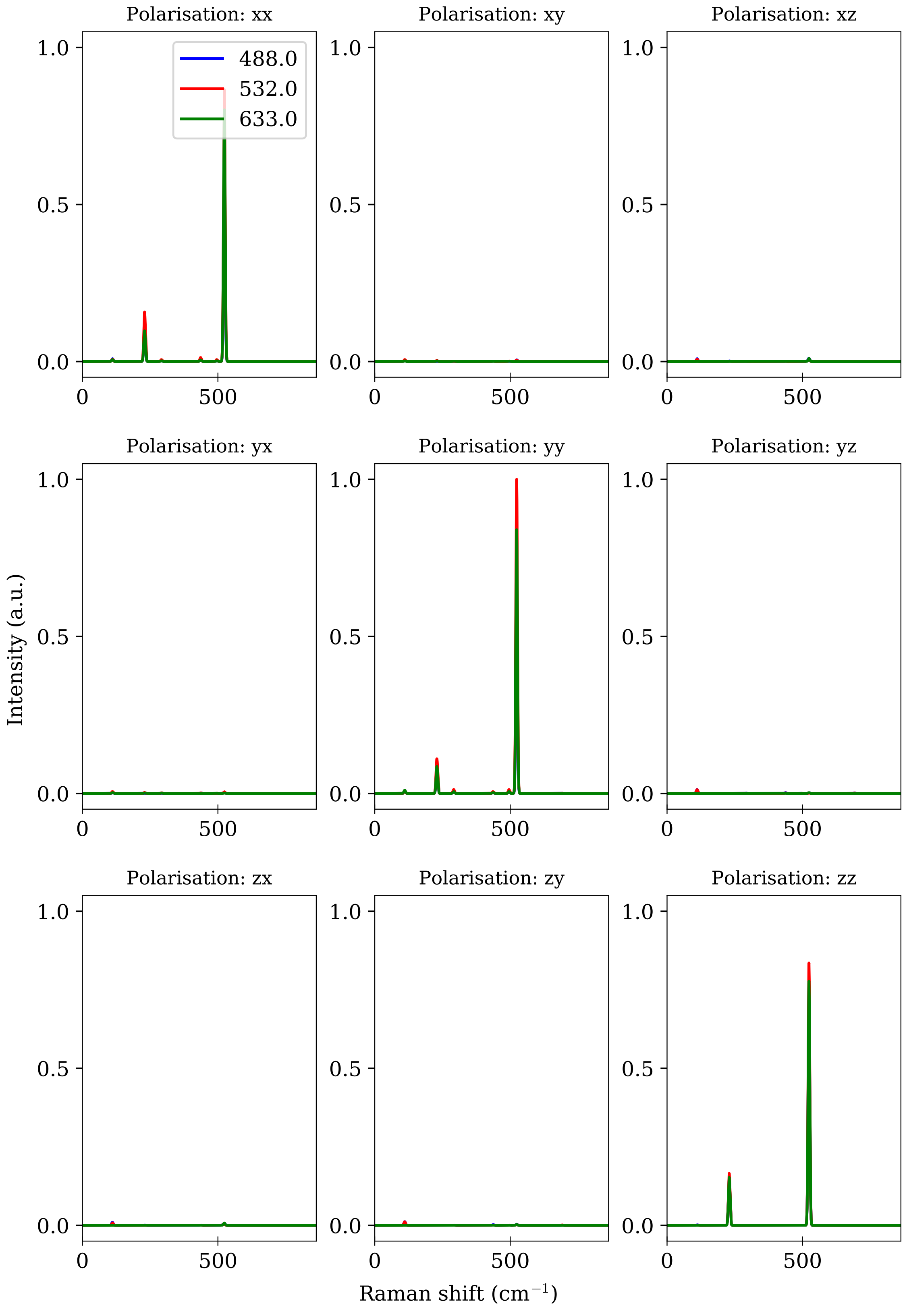

Supplementary Figure 695: Raman spectrum of Zr_2CO_2 with space group 164 and Wyckoff positions b-d-0.

Zr_2F_2N, 164, b-d-0

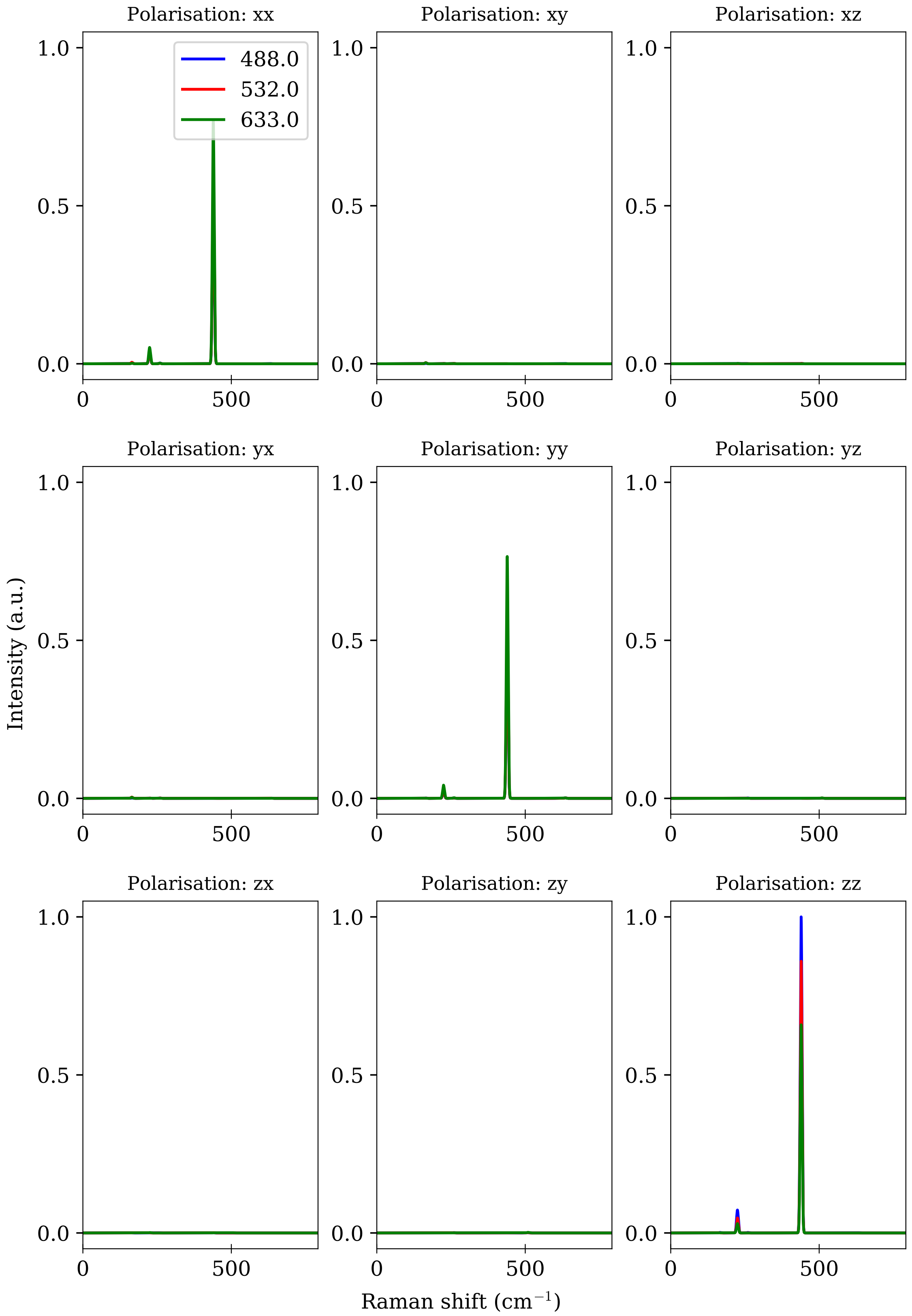

Supplementary Figure 696: Raman spectrum of Zr_2F_2N with space group 164 and Wyckoff positions b-d-0.

Zr_2F_2, 12, i-0

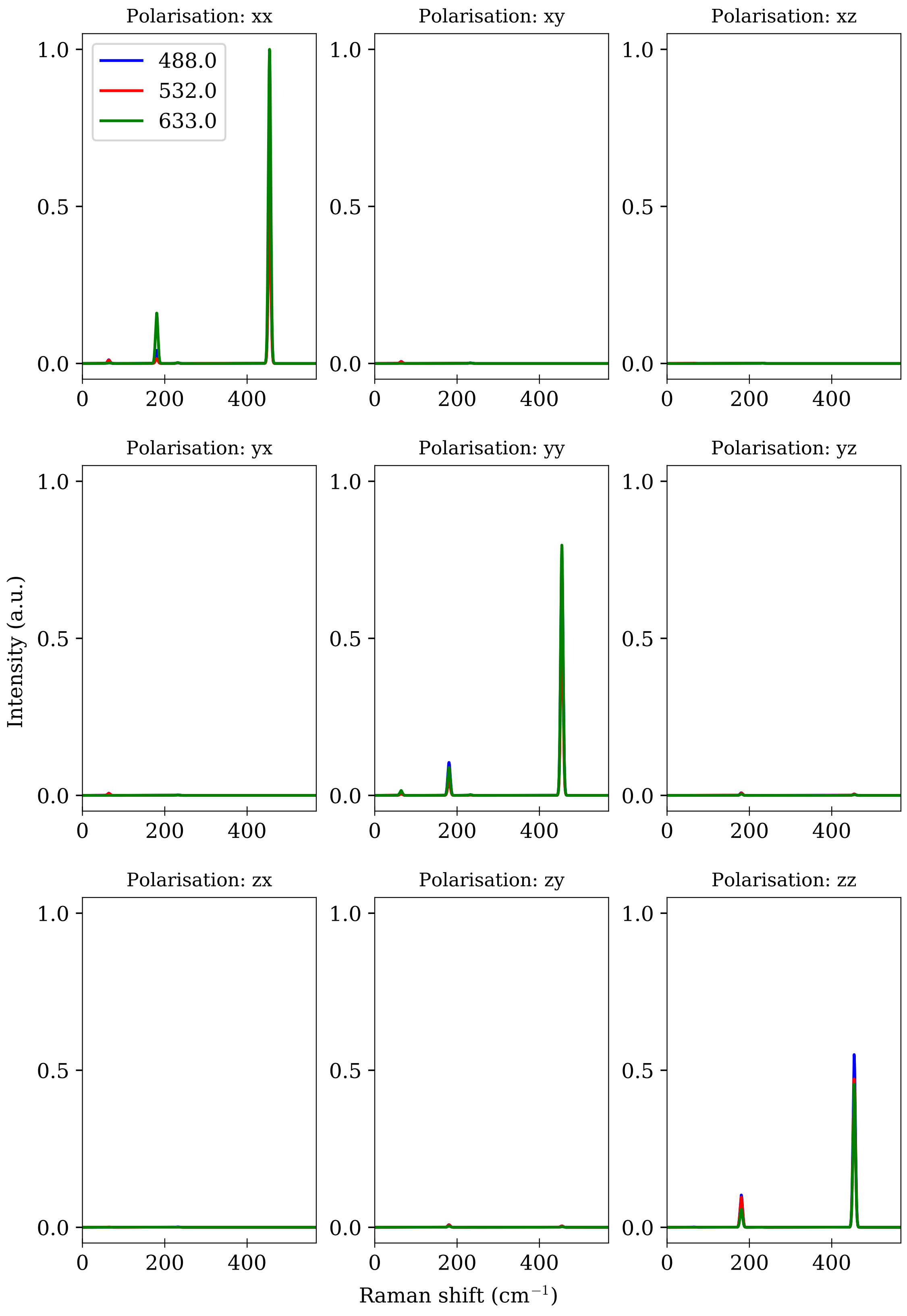

Supplementary Figure 697: Raman spectrum of Zr_2F_2 with space group 12 and Wyckoff positions i-0.

$Zr_2H_2NO_2$, 164, b-d-0

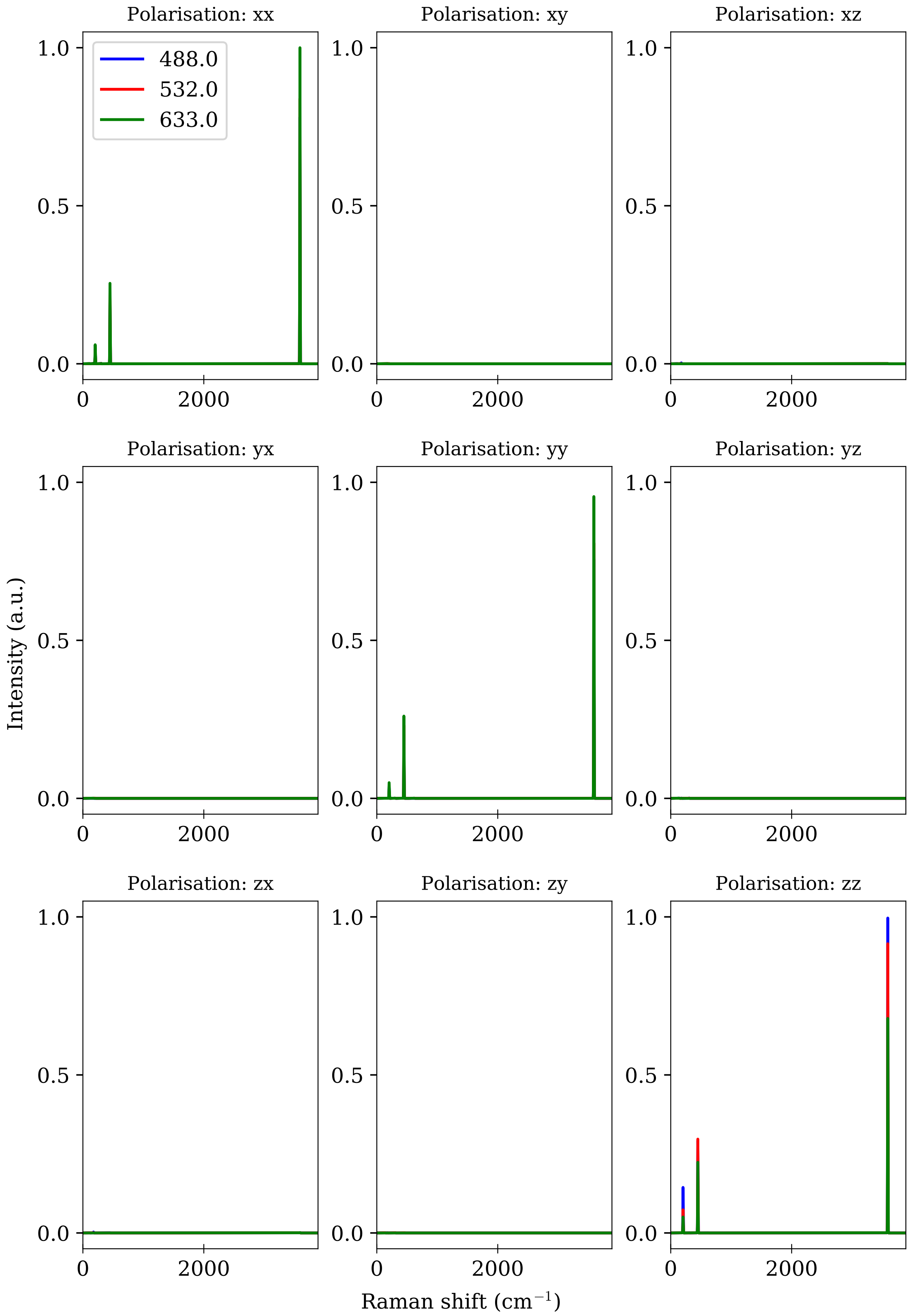

Supplementary Figure 698: Raman spectrum of $Zr_2H_2NO_2$ with space group 164 and Wyckoff positions b-d-0.

$Zr_2I_2S_2$, 59, a-b-0

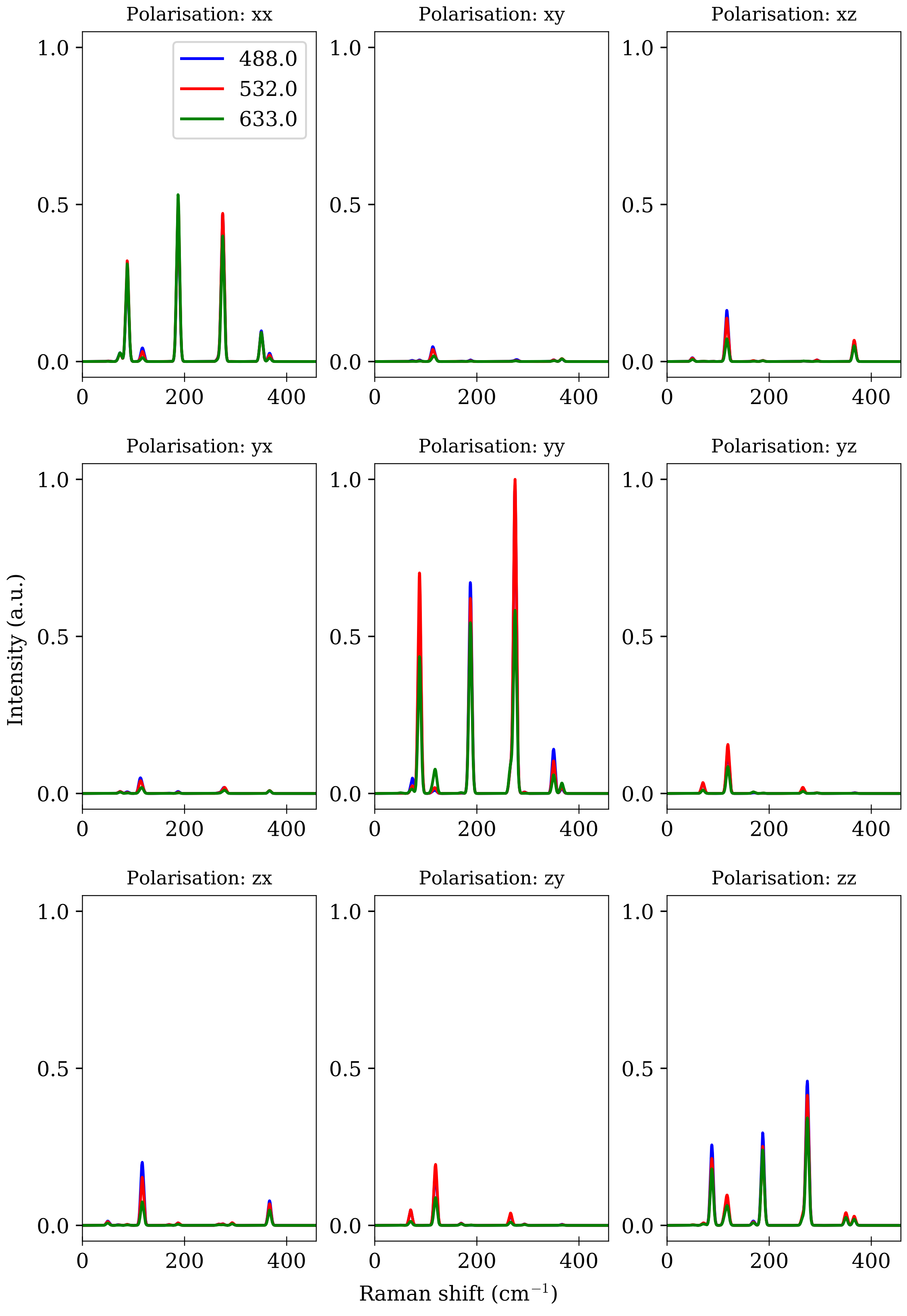

Supplementary Figure 699: Raman spectrum of $Zr_2I_2S_2$ with space group 59 and Wyckoff positions a-b-0.

Zr_2I_2, 129, b-c-0

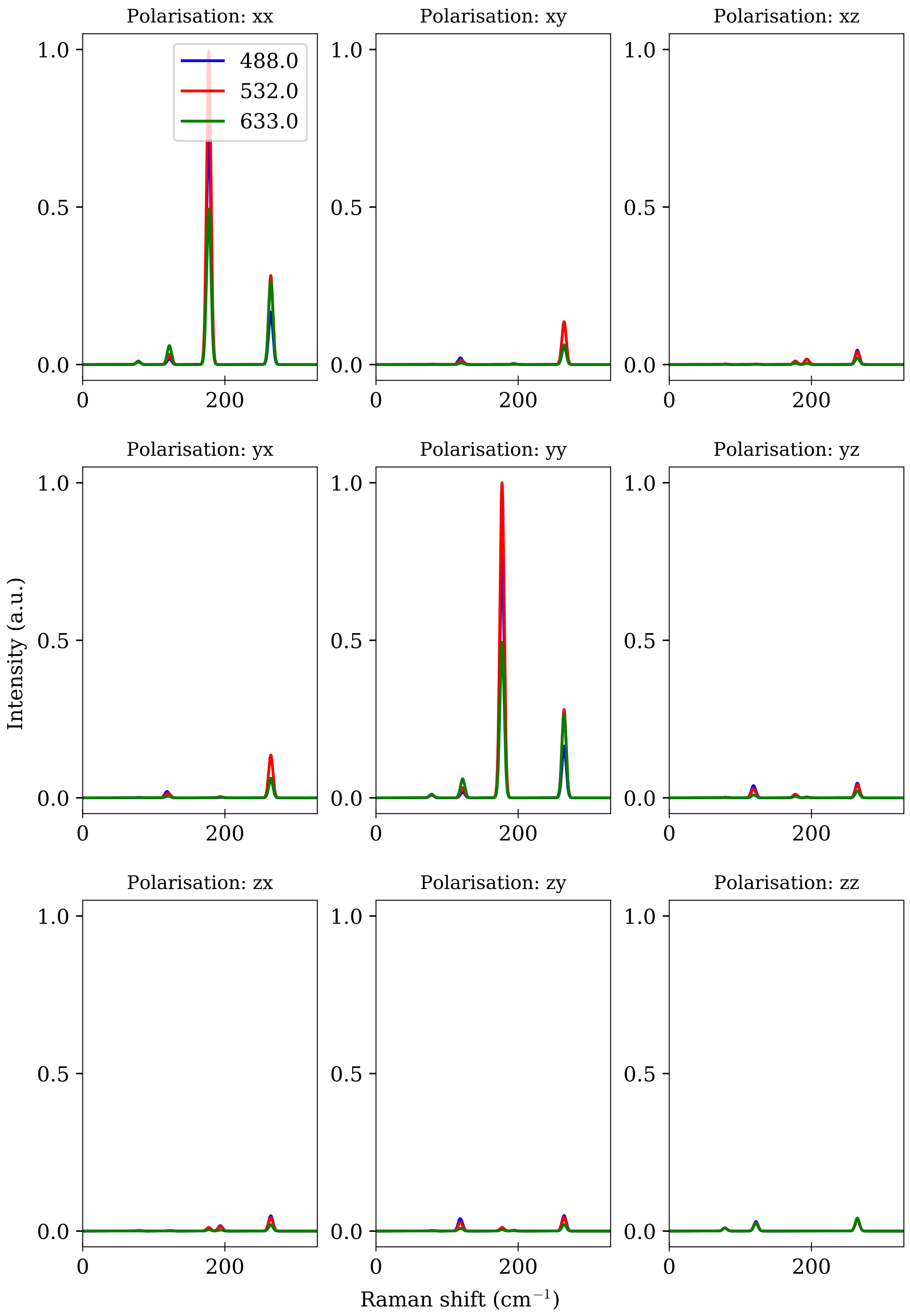

Supplementary Figure 700: Raman spectrum of Zr_2I_2 with space group 129 and Wyckoff positions b-c-0.

Zr_2NO_2, 164, b-d-0

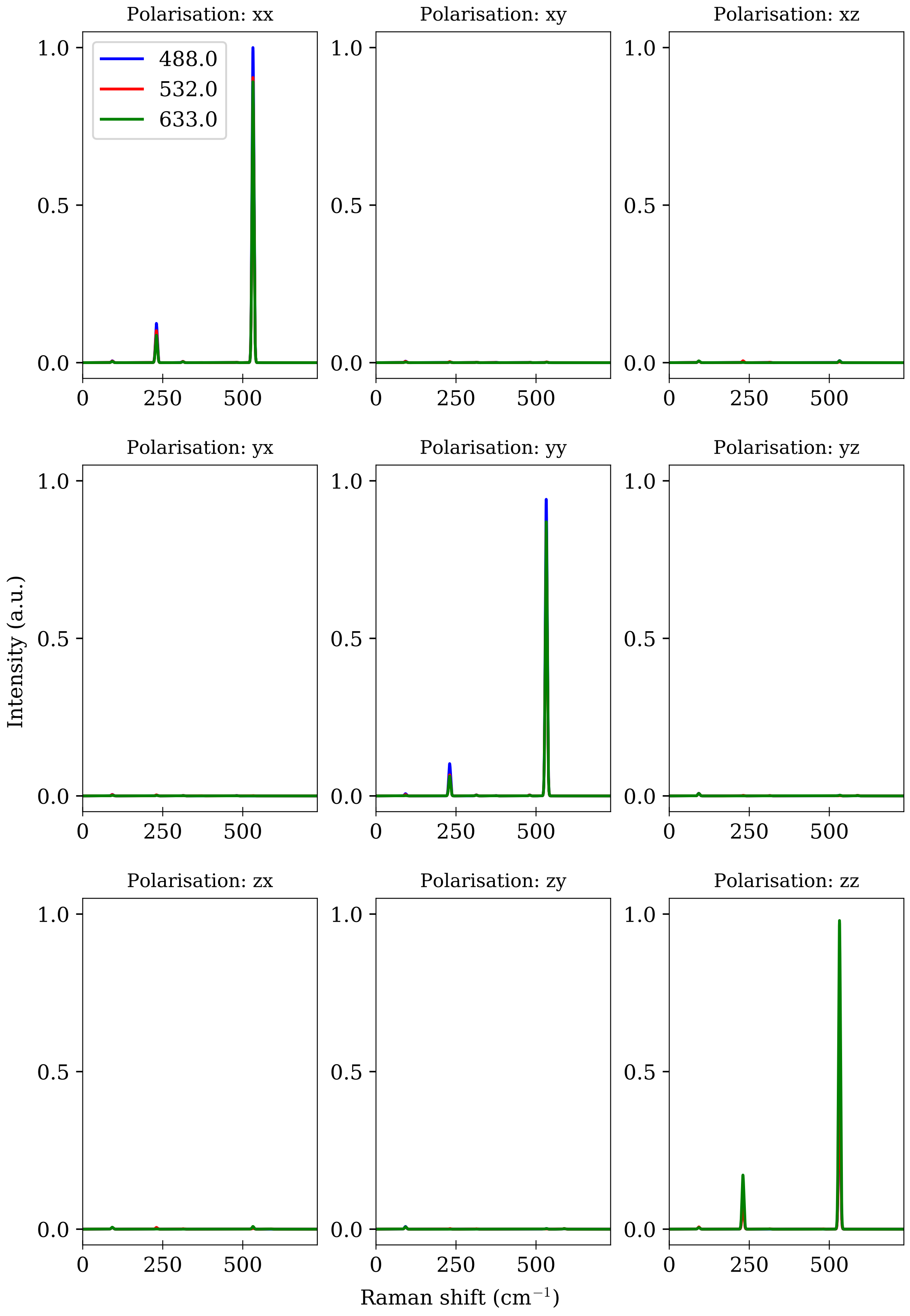

Supplementary Figure 701: Raman spectrum of Zr_2NO_2 with space group 164 and Wyckoff positions b-d-0.

Zr_2N, 164, b-d-0

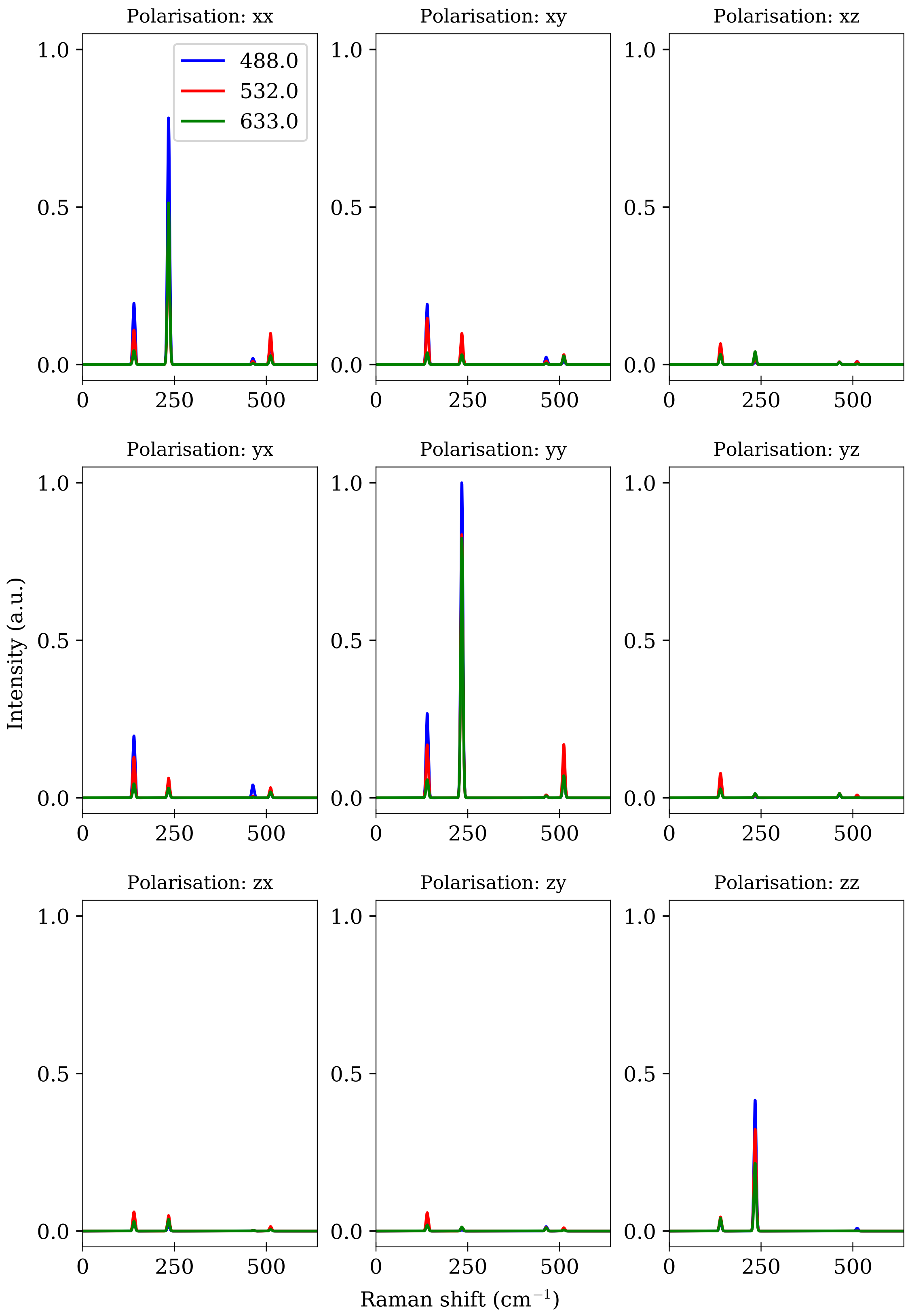

Supplementary Figure 702: Raman spectrum of Zr_2N with space group 164 and Wyckoff positions b-d-0.

Zr_2O_2, 10, b-d-m-0

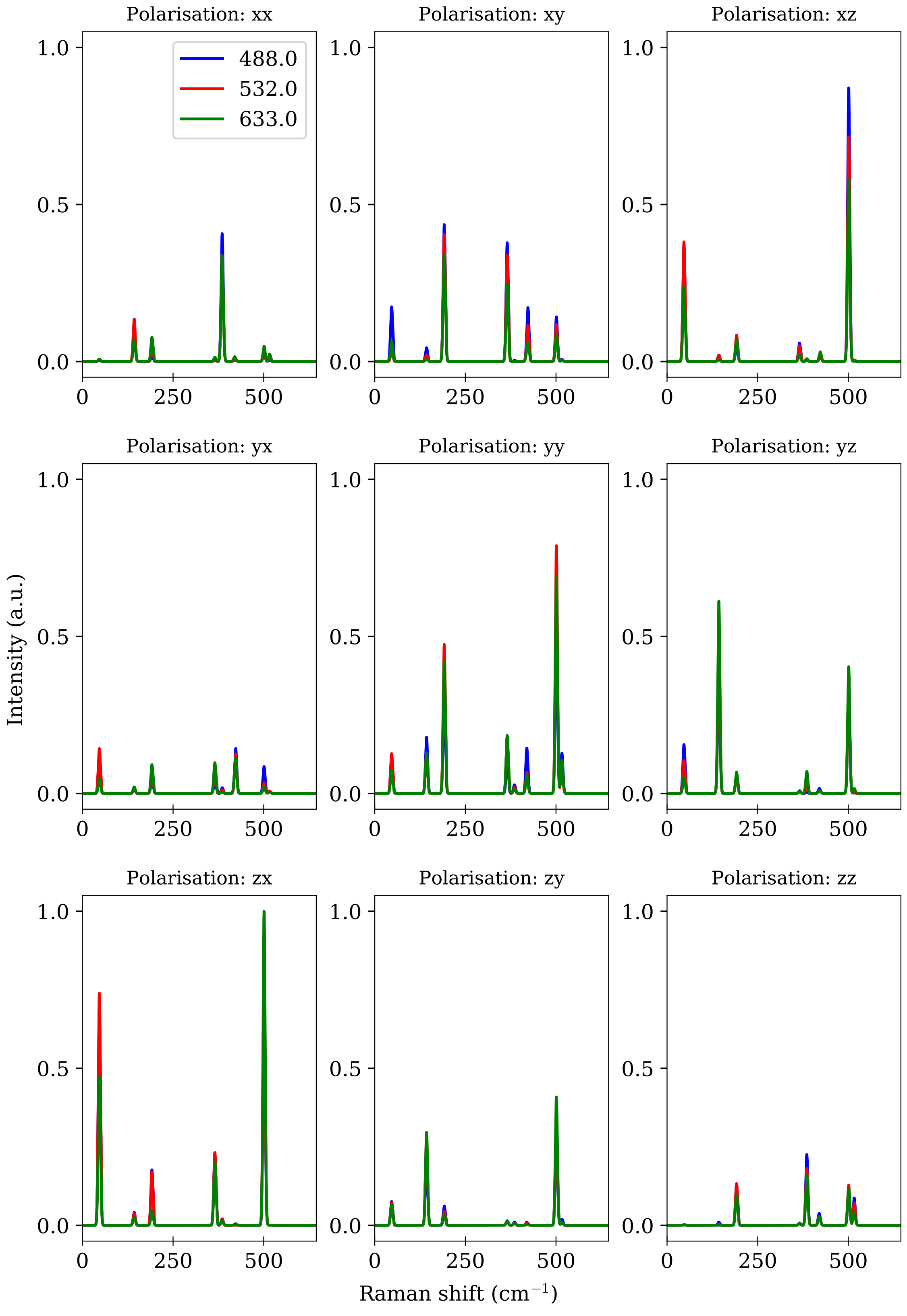

Supplementary Figure 703: Raman spectrum of Zr_2O_2 with space group 10 and Wyckoff positions b-d-m-0.

Zr_2O_6, 59, a-b-e-0

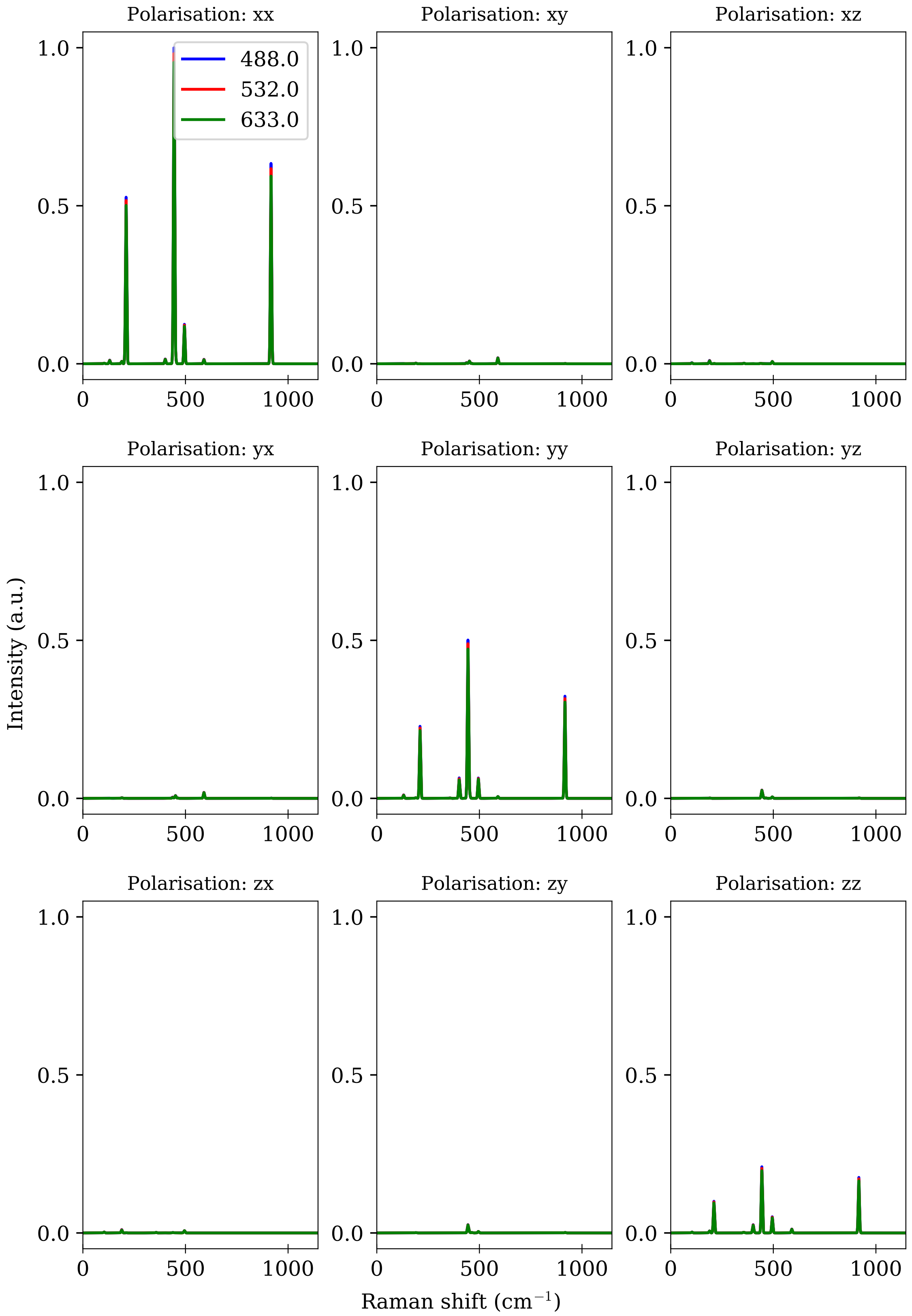

Supplementary Figure 704: Raman spectrum of Zr_2O_6 with space group 59 and Wyckoff positions a-b-e-0.

Zr_2S_2, 129, b-c-0

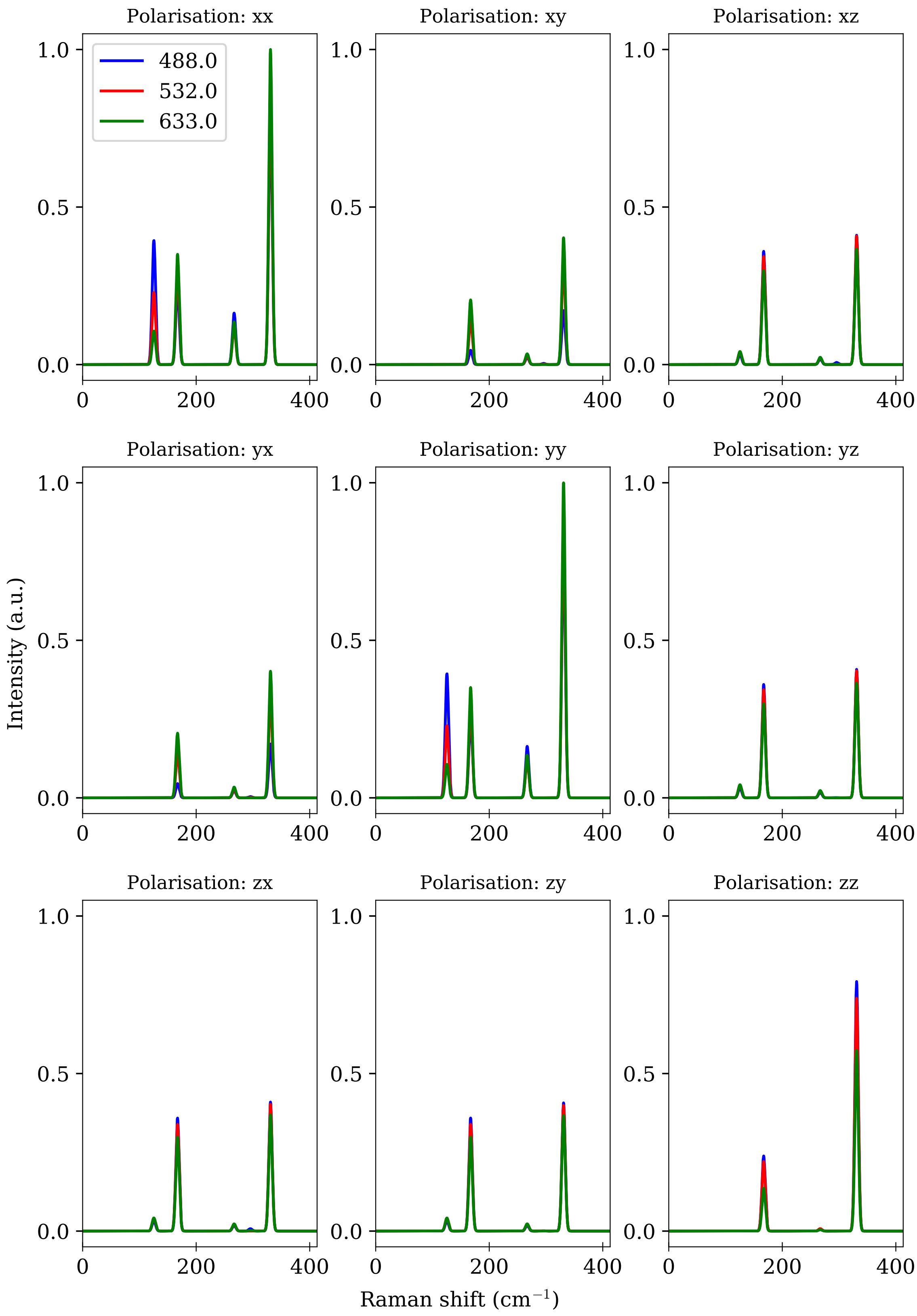

Supplementary Figure 705: Raman spectrum of Zr_2S_2 with space group 129 and Wyckoff positions b-c-0.

Zr_2S_2, 12, i-0

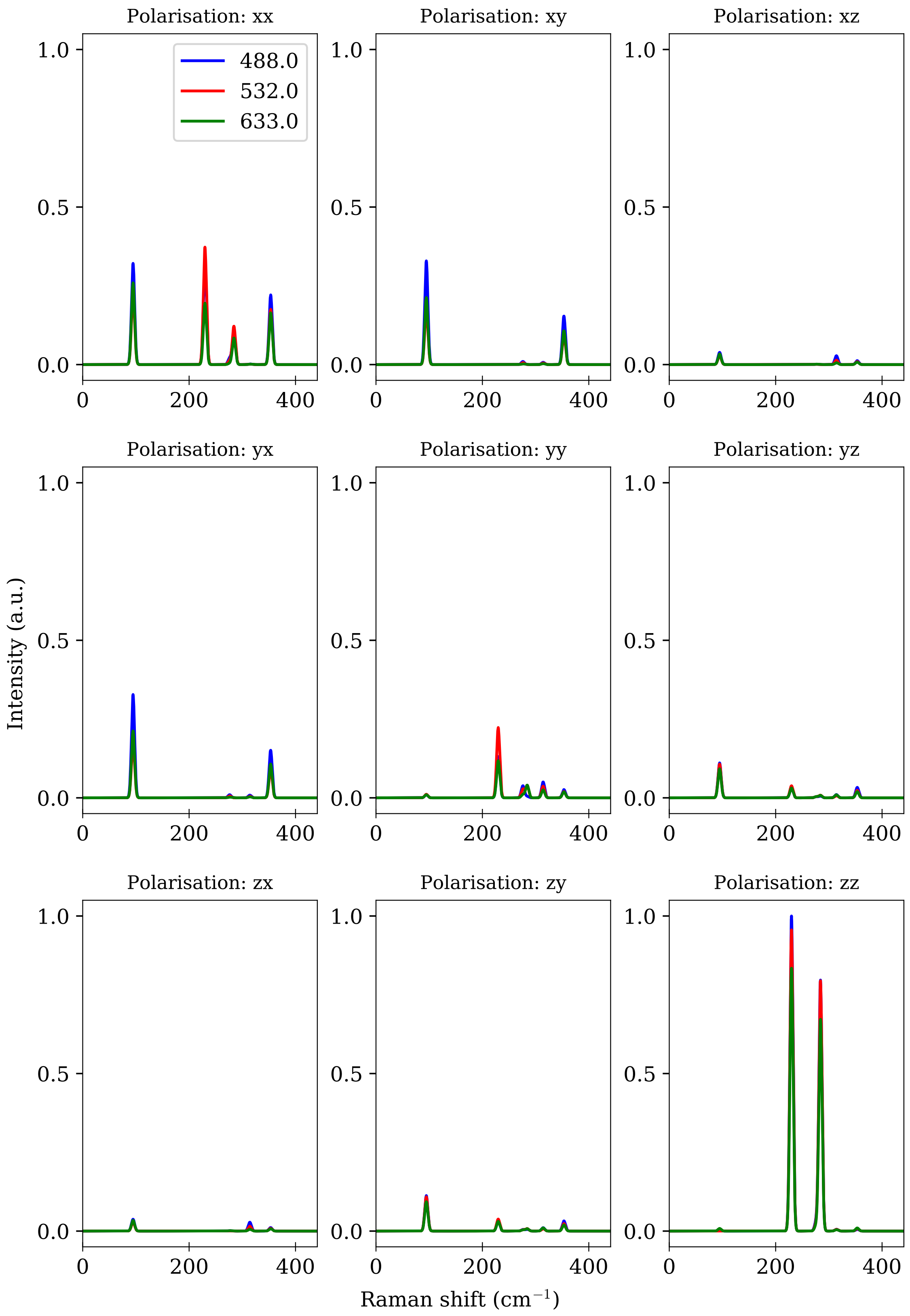

Supplementary Figure 706: Raman spectrum of Zr_2S_2 with space group 12 and Wyckoff positions i-0.

Zr_2Se_2, 129, b-c-0

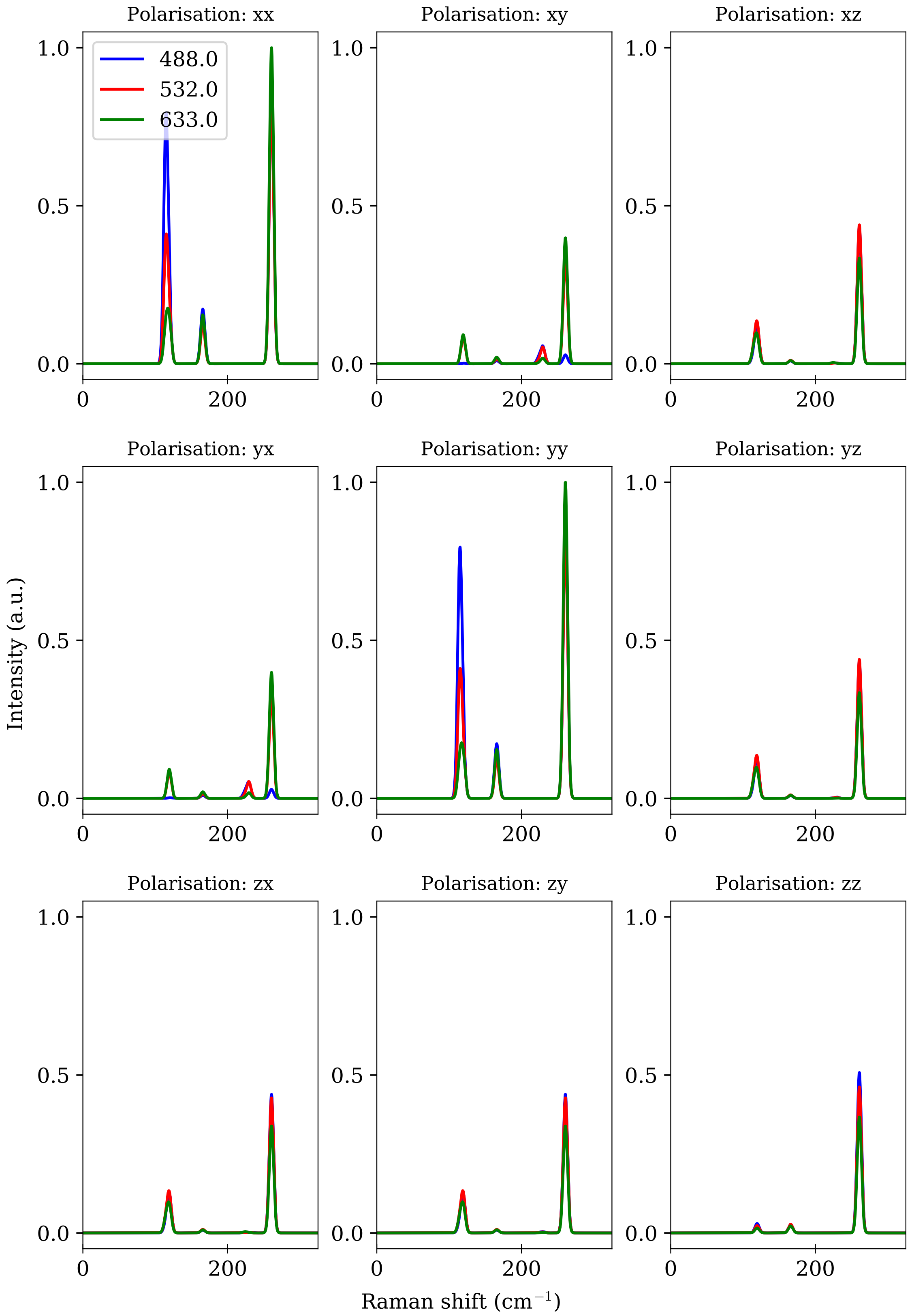

Supplementary Figure 707: Raman spectrum of Zr_2Se_2 with space group 129 and Wyckoff positions b-c-0.

Zr_2Se_2, 12, i-0

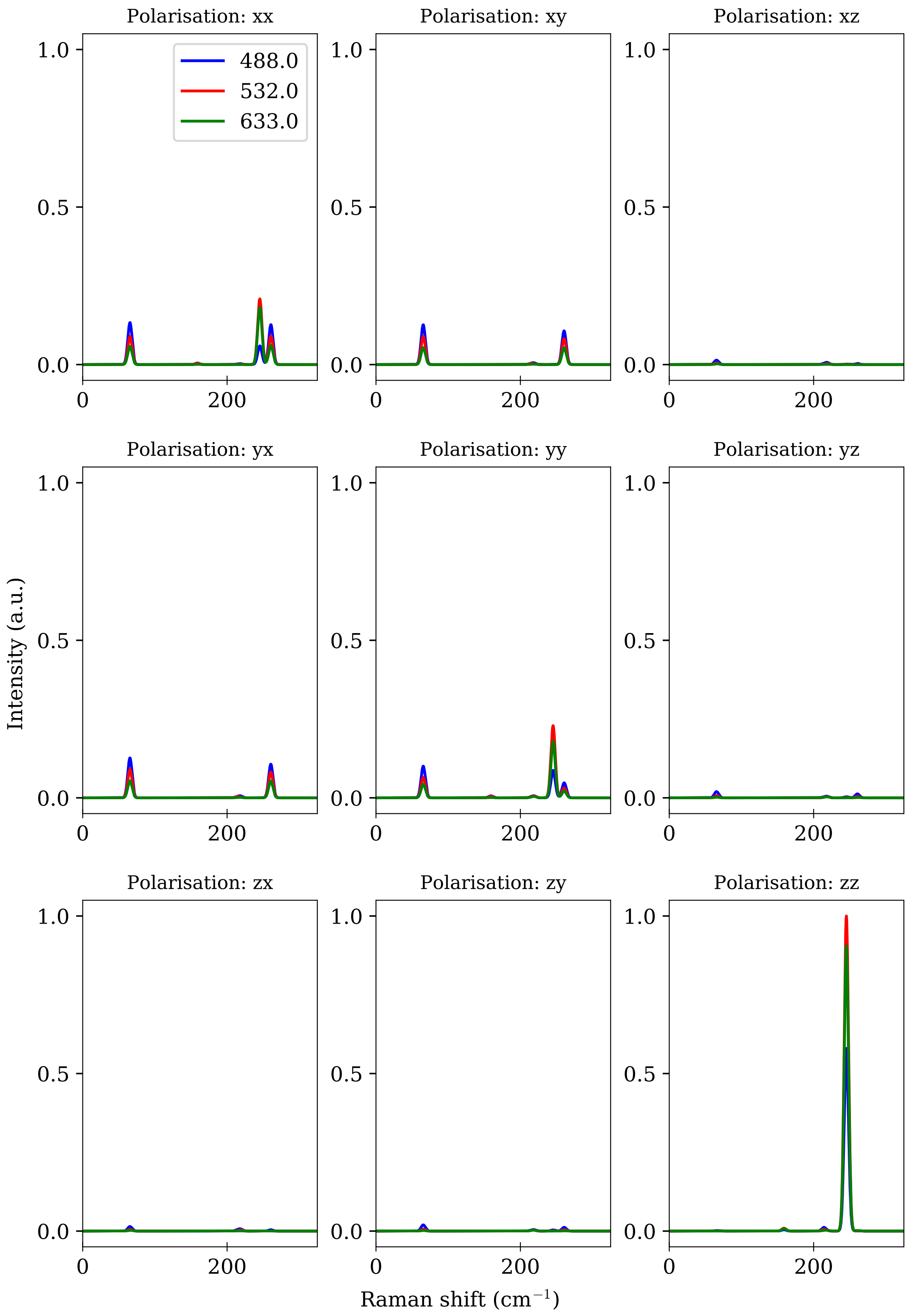

Supplementary Figure 708: Raman spectrum of Zr_2Se_2 with space group 12 and Wyckoff positions i-0.

Zr_2Se_2, 187, h-i-0

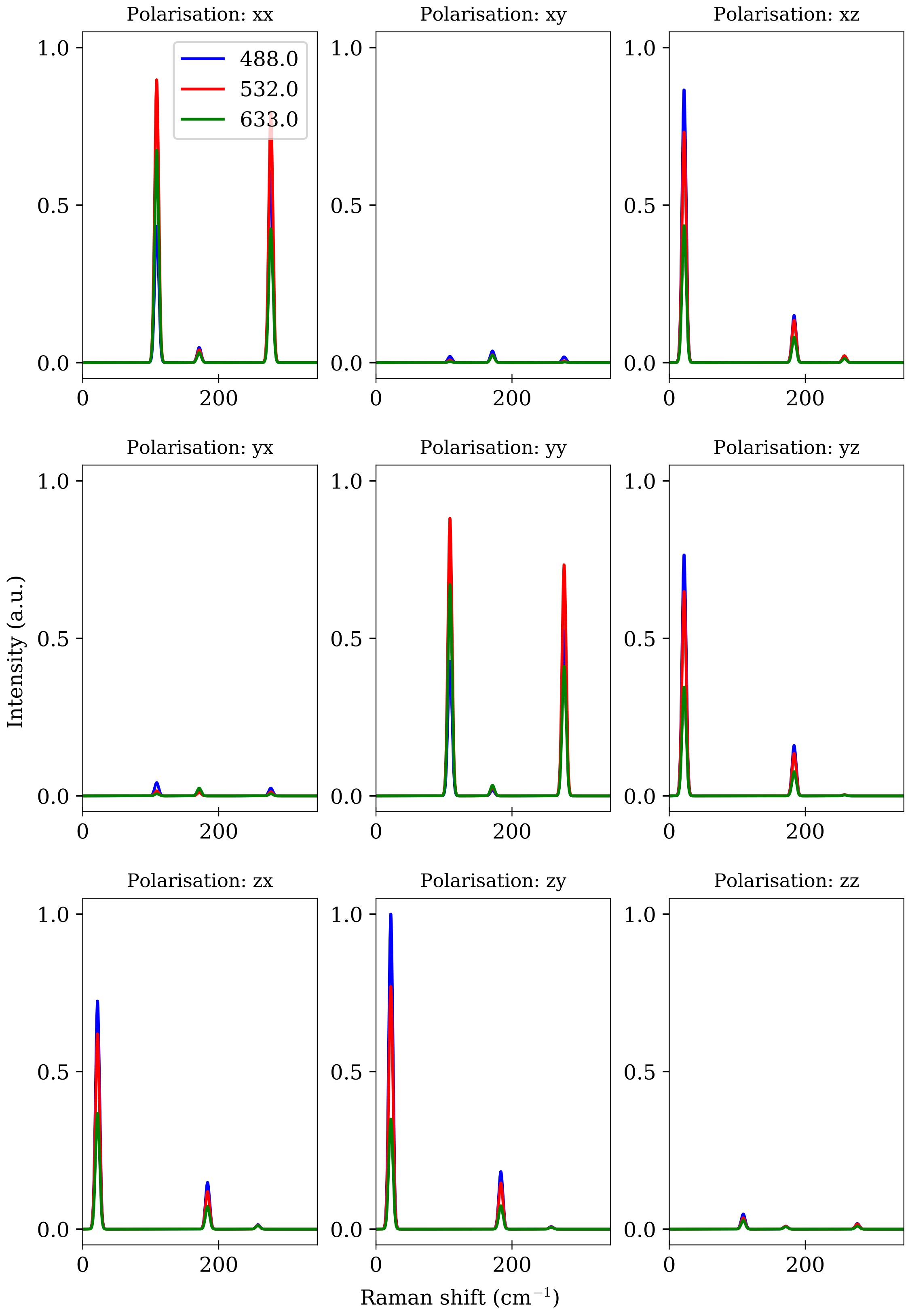

Supplementary Figure 709: Raman spectrum of Zr_2Se_2 with space group 187 and Wyckoff positions h-i-0.

Zr_2Te_2, 12, i-0

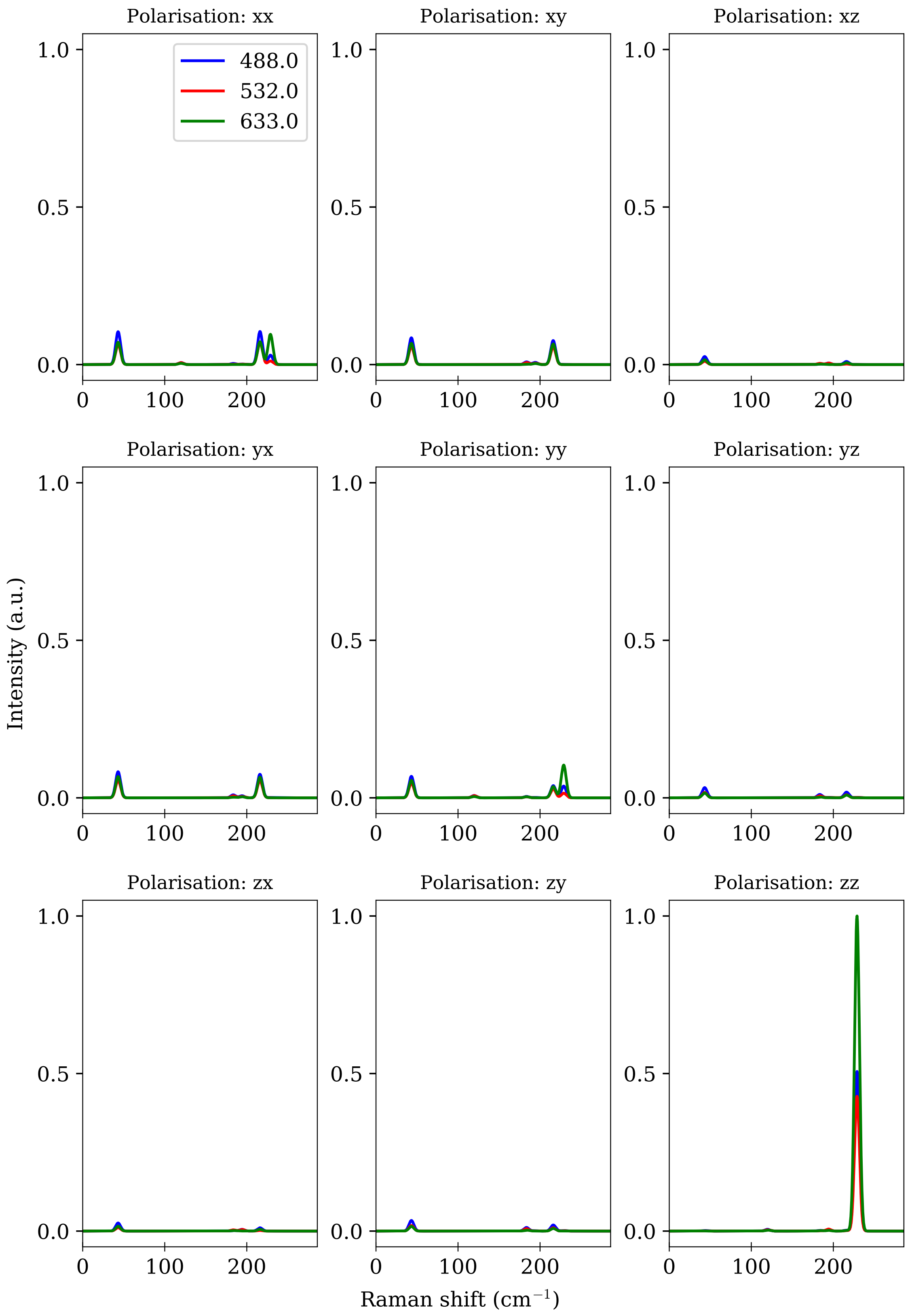

Supplementary Figure 710: Raman spectrum of Zr_2Te_2 with space group 12 and Wyckoff positions i-0.

$Zr_3C_2F_2$, 187, b-g-h-i-0

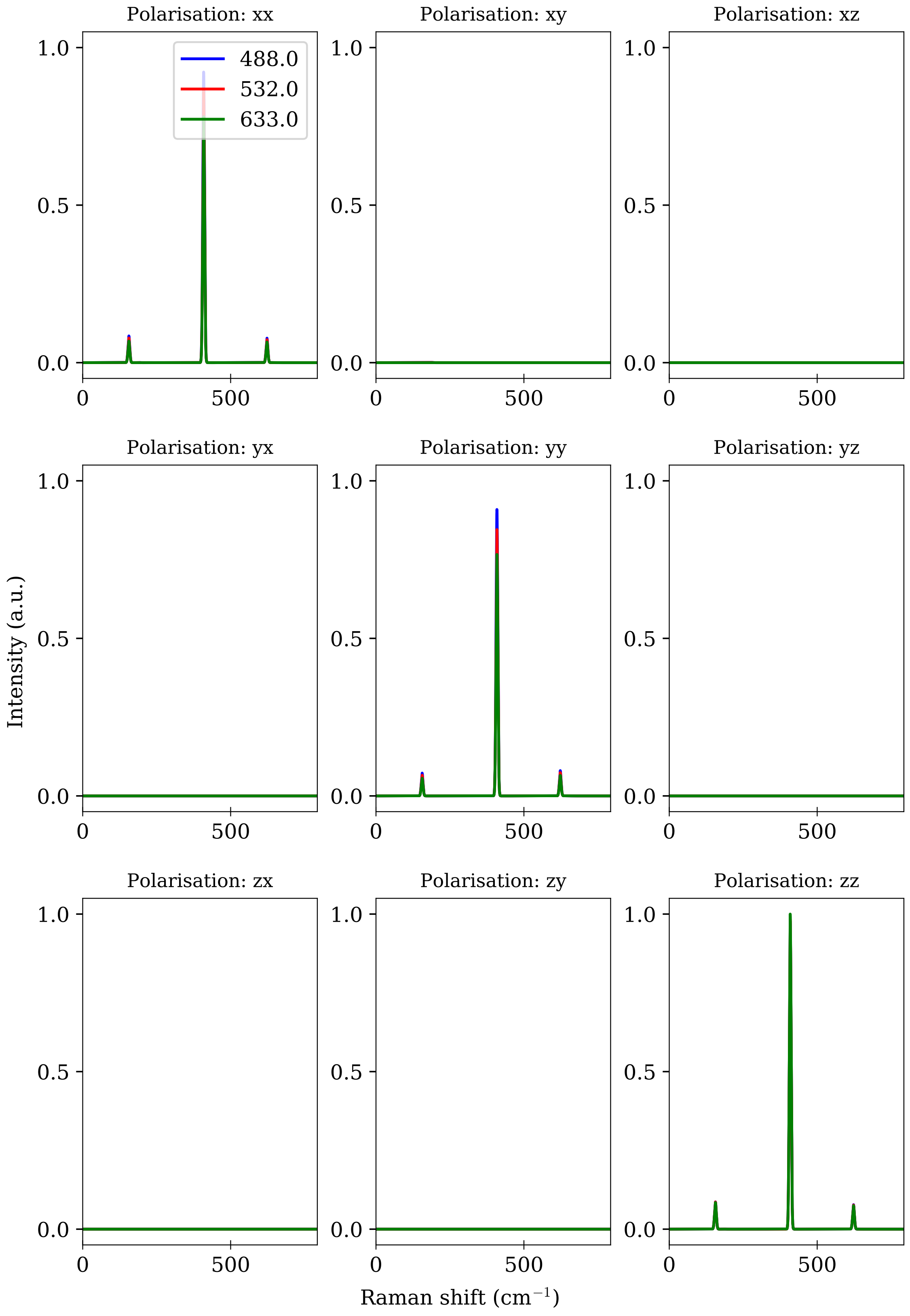

Supplementary Figure 711: Raman spectrum of $Zr_3C_2F_2$ with space group 187 and Wyckoff positions b-g-h-i-0.

$Zr_3C_2O_2$, 187, b-g-h-i-0

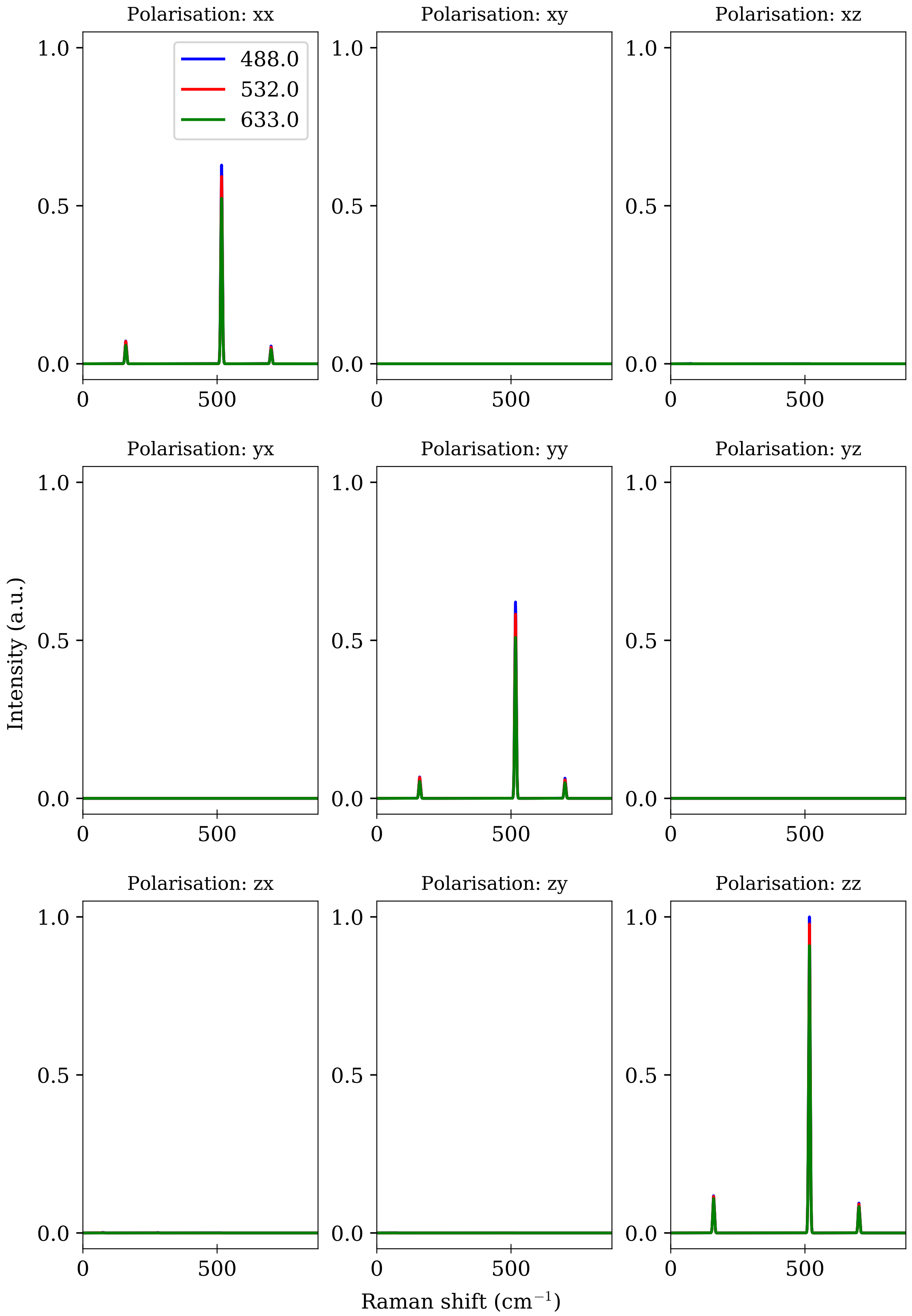

Supplementary Figure 712: Raman spectrum of $Zr_3C_2O_2$ with space group 187 and Wyckoff positions b-g-h-i-0.

$Zr_3N_2O_2$, 187, b-g-h-i-0

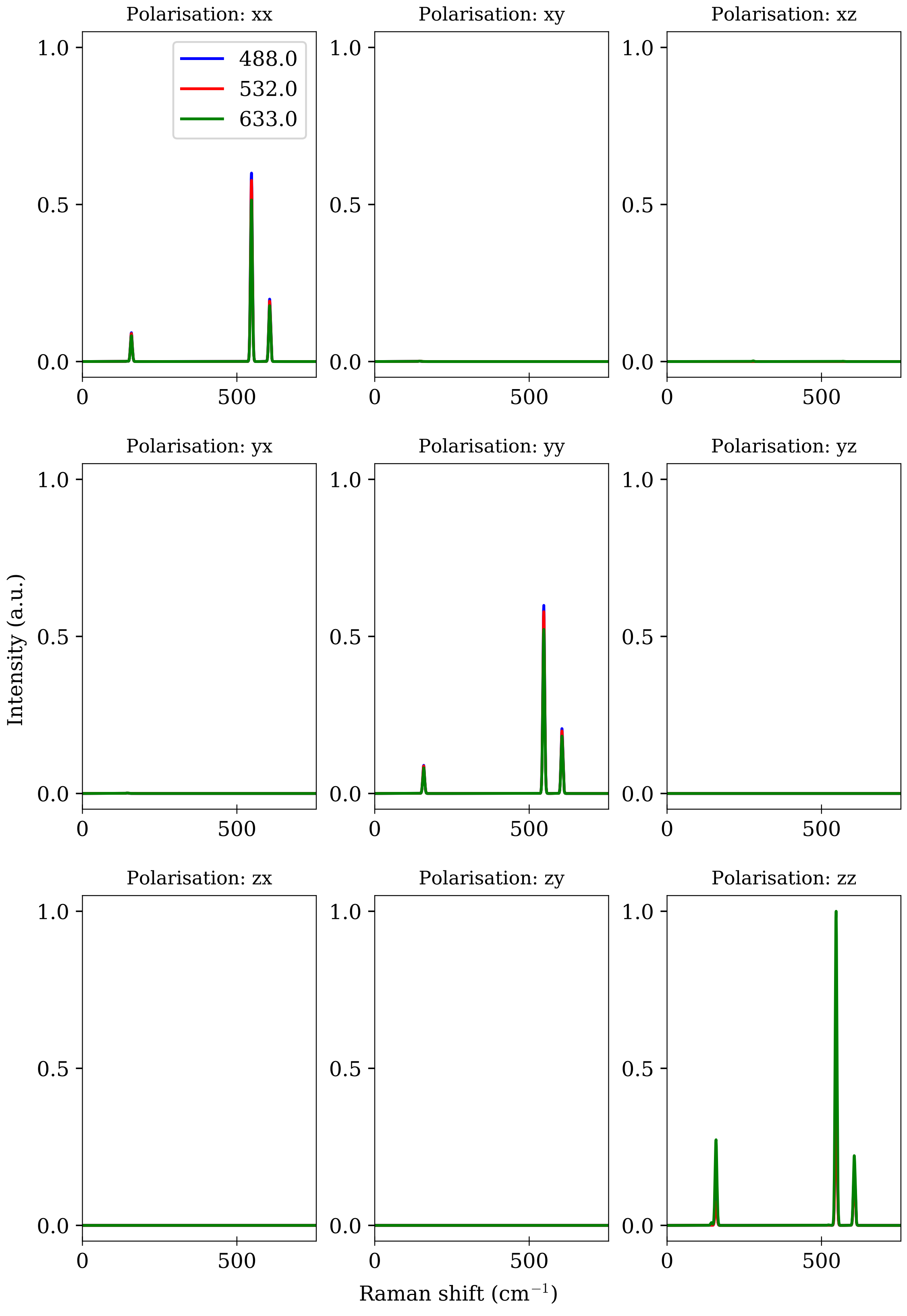

Supplementary Figure 713: Raman spectrum of $Zr_3N_2O_2$ with space group 187 and Wyckoff positions b-g-h-i-0.

Zr_3N_2, 187, a-h-i-0

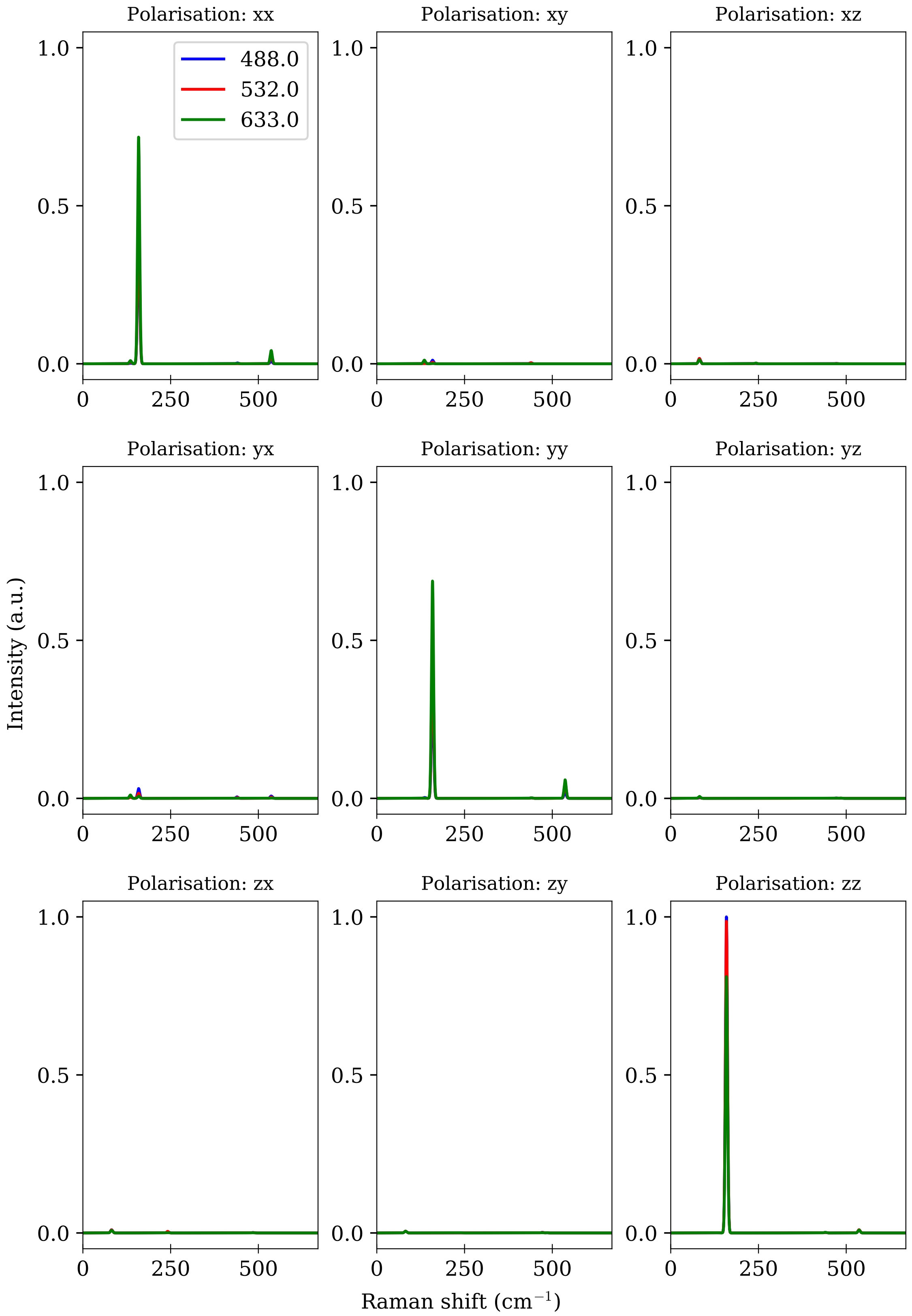

Supplementary Figure 714: Raman spectrum of Zr_3N_2 with space group 187 and Wyckoff positions a-h-i-0.

Zr_4N_3, 164, b-c-d-0

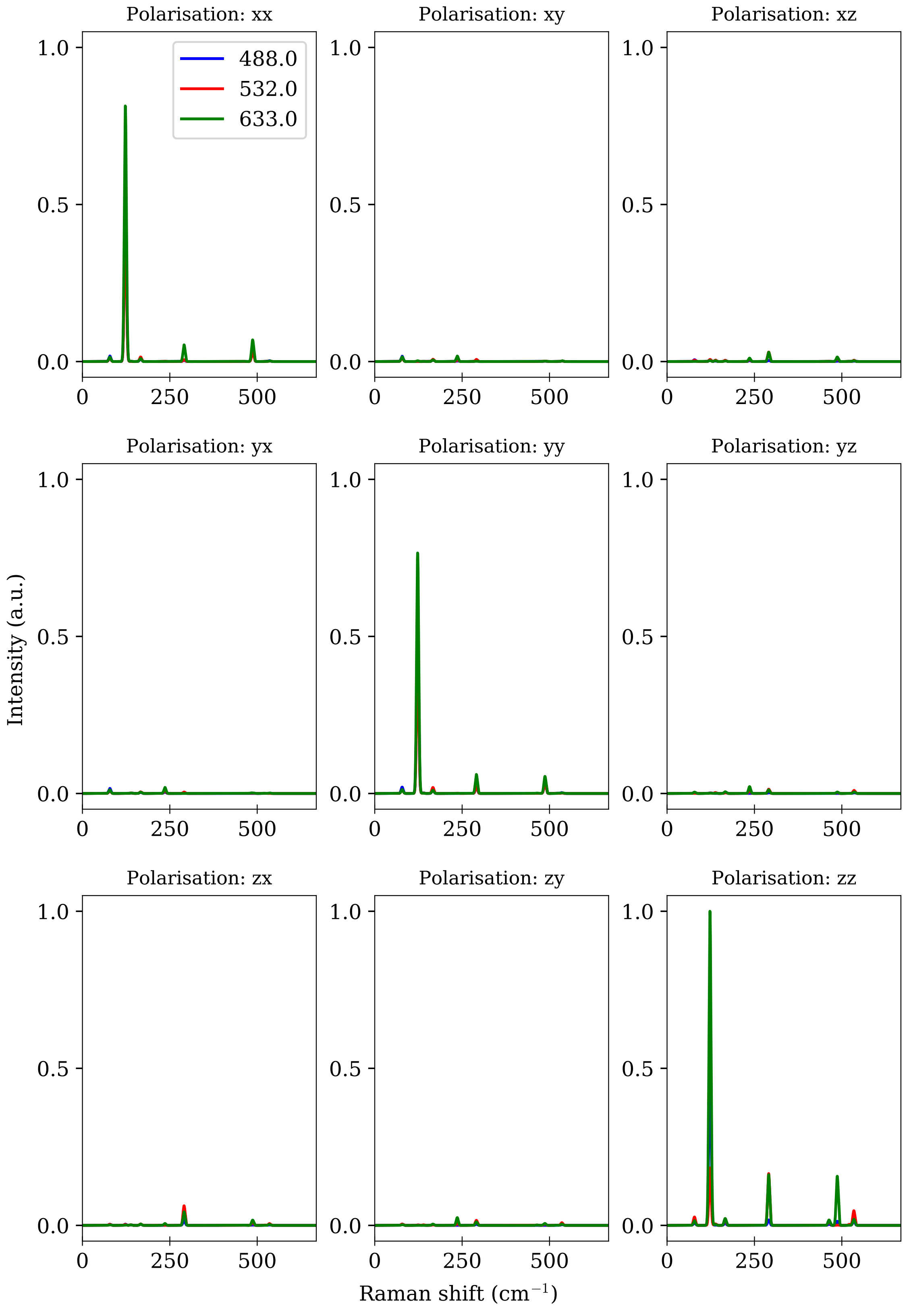

Supplementary Figure 715: Raman spectrum of Zr_4N_3 with space group 164 and Wyckoff positions b-c-d-0.

$ZrBr_2$, 187, b-i-0

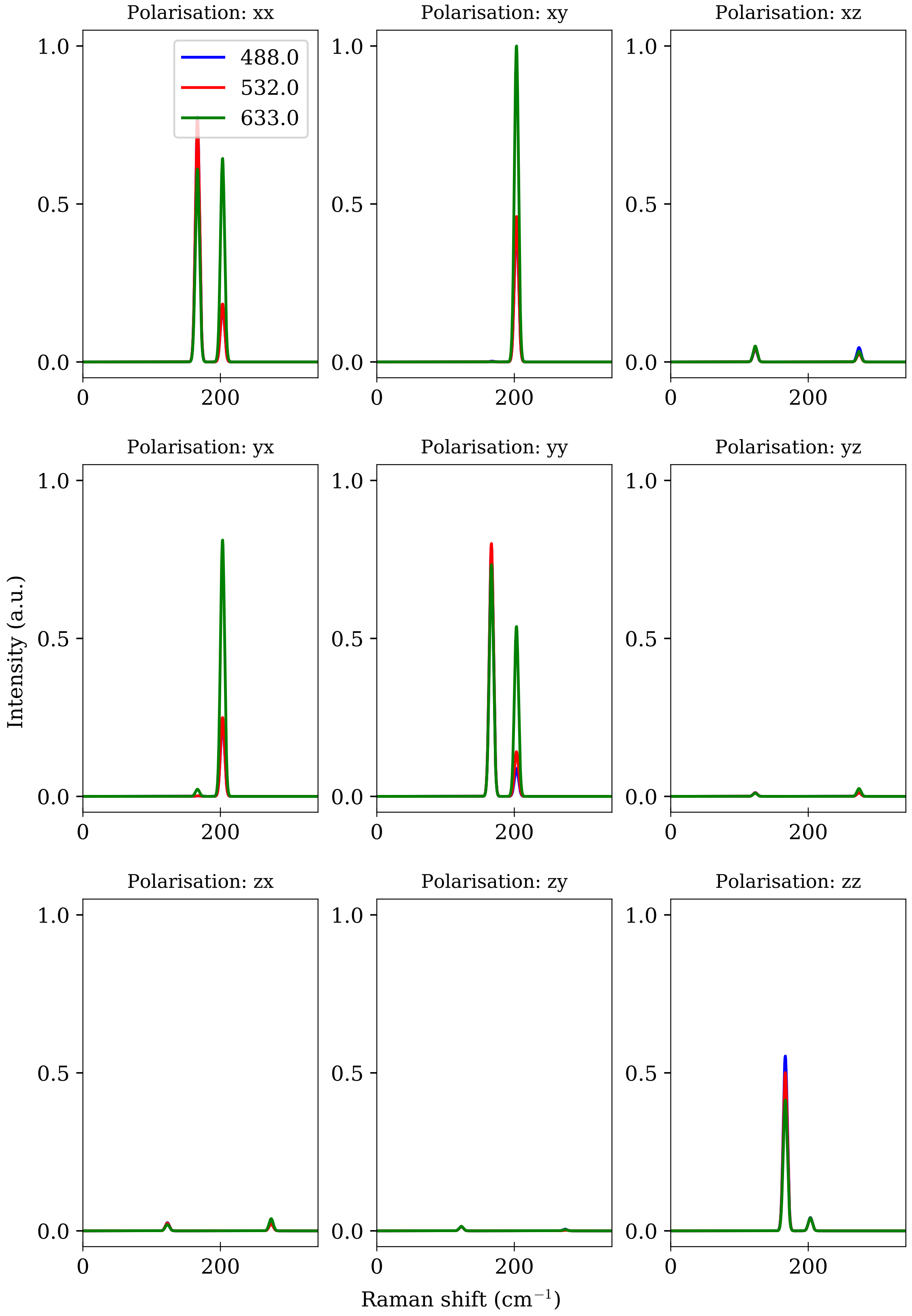

Supplementary Figure 716: Raman spectrum of $ZrBr_2$ with space group 187 and Wyckoff positions b-i-0.

ZrBrCl, 156, a-c-0

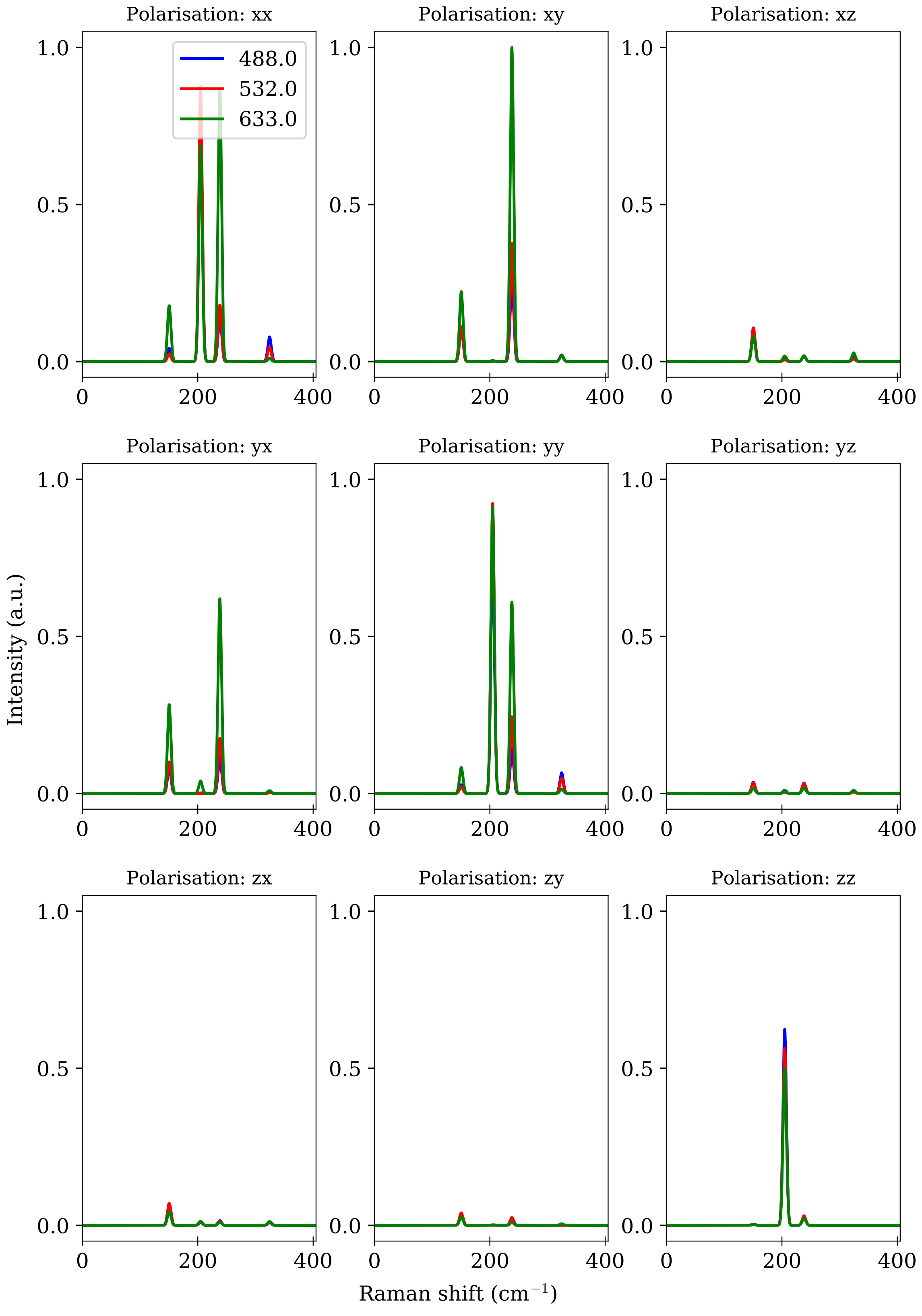

Supplementary Figure 717: Raman spectrum of ZrBrCl with space group 156 and Wyckoff positions a-c-0.

ZrBrI, 156, a-c-0

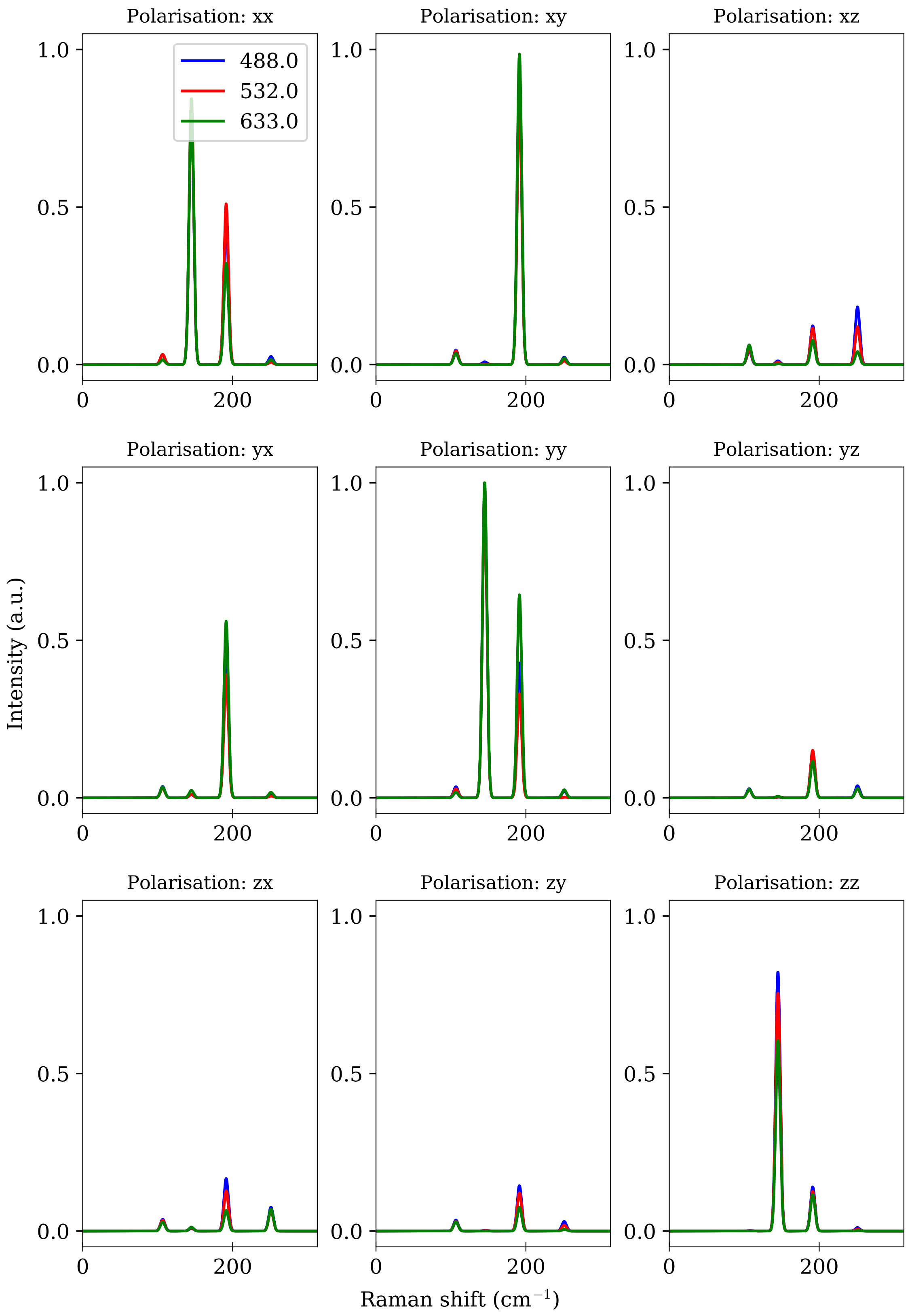

Supplementary Figure 718: Raman spectrum of ZrBrI with space group 156 and Wyckoff positions a-c-0.

$ZrCl_2$, 187, b-i-0

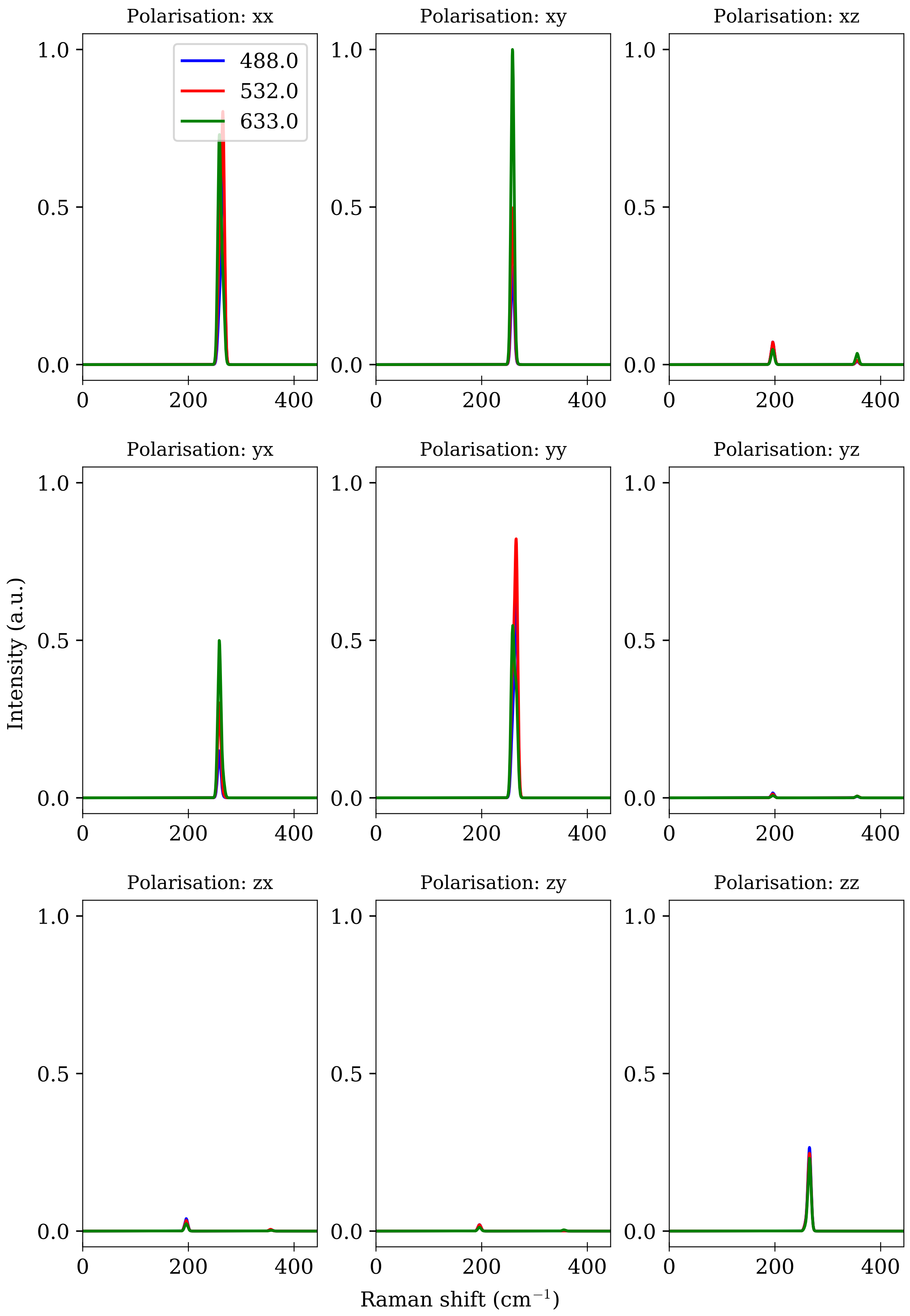

Supplementary Figure 719: Raman spectrum of $ZrCl_2$ with space group 187 and Wyckoff positions b-i-0.

ZrClI, 156, a-c-0

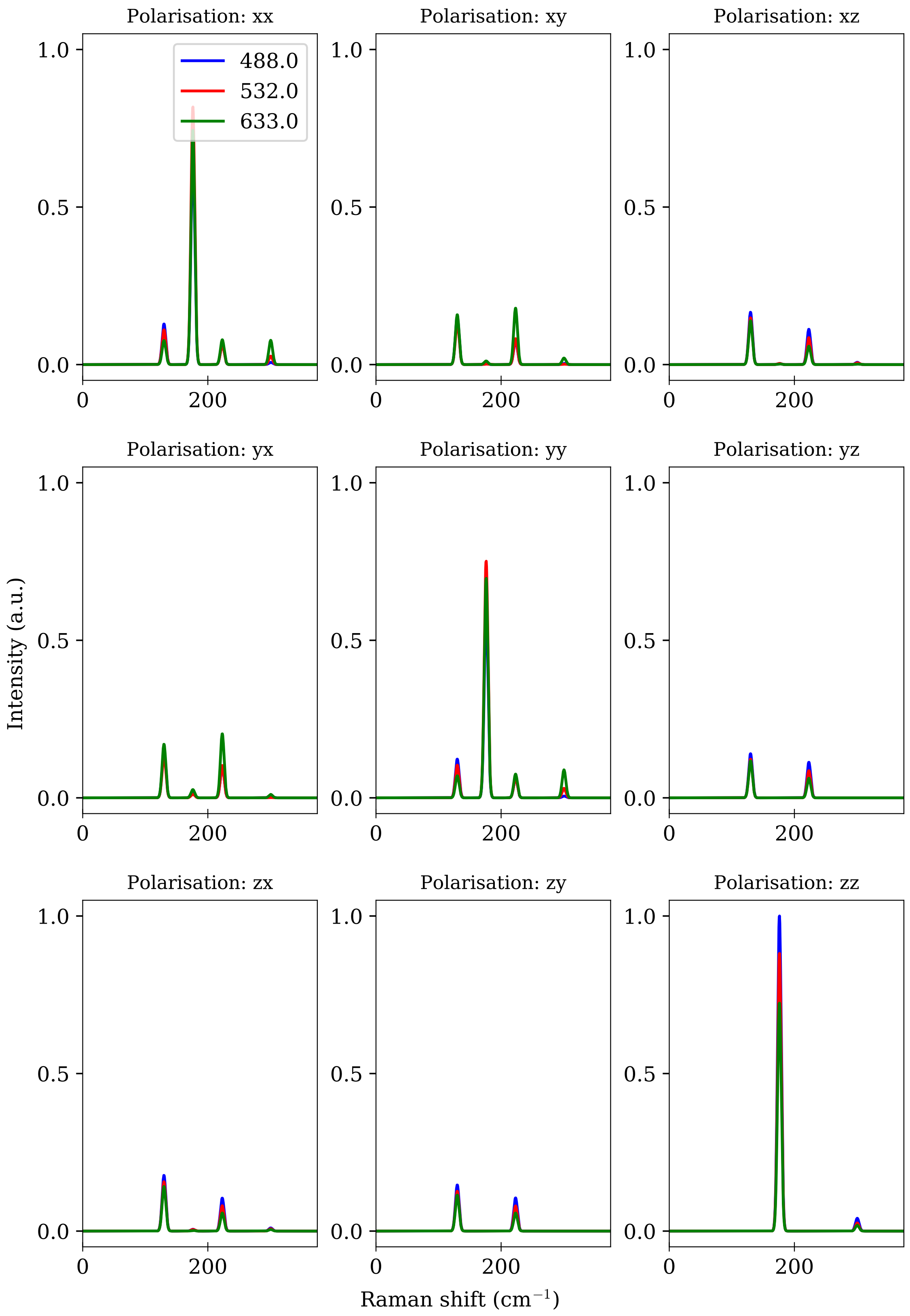

Supplementary Figure 720: Raman spectrum of ZrClI with space group 156 and Wyckoff positions a-c-0.

ZrH_2, 187, b-i-0

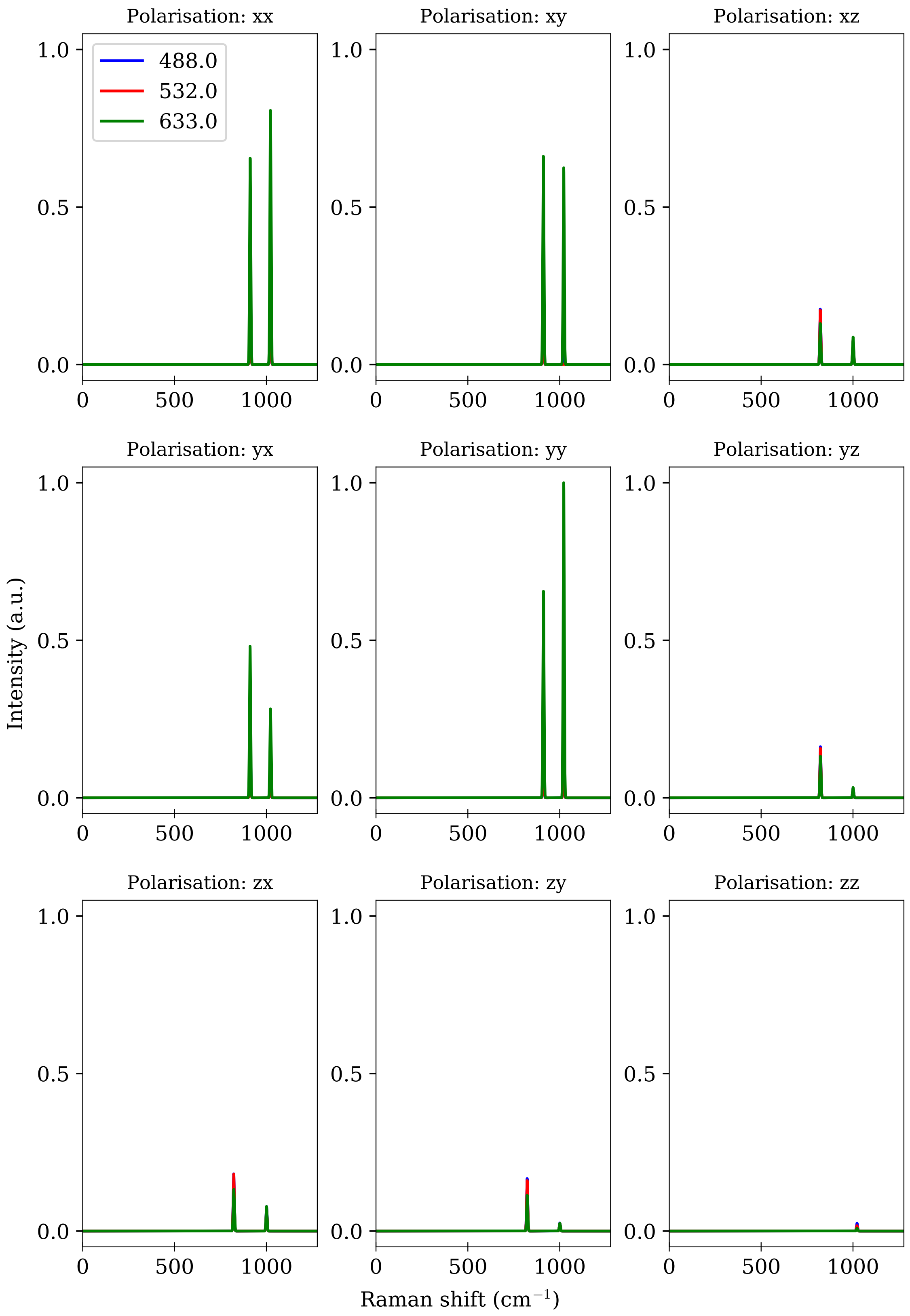

Supplementary Figure 721: Raman spectrum of ZrH_2 with space group 187 and Wyckoff positions b-i-0.

ZrI_2, 187, b-i-0

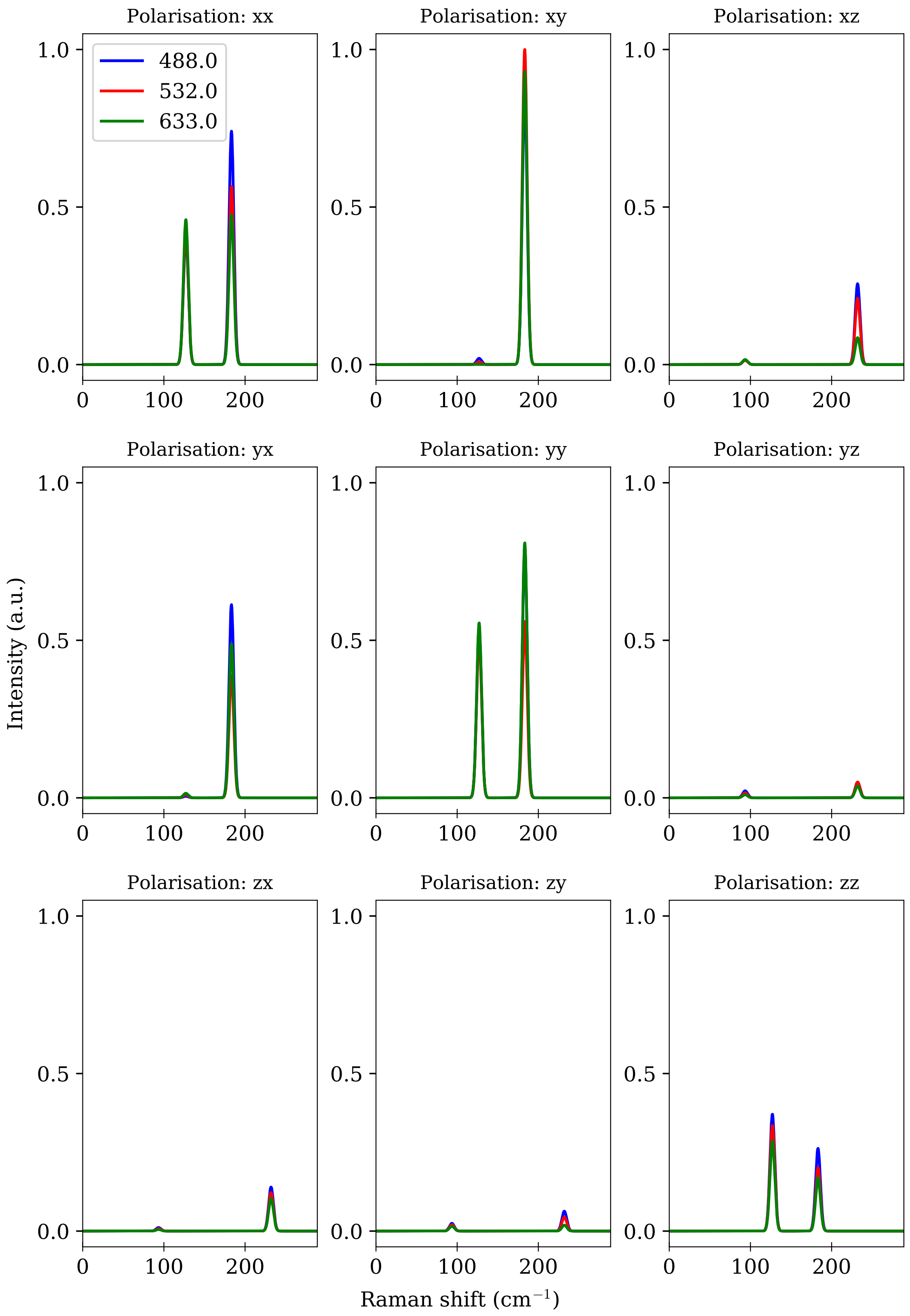

Supplementary Figure 722: Raman spectrum of ZrI_2 with space group 187 and Wyckoff positions b-i-0.

ZrO_2, 115, a-g-0

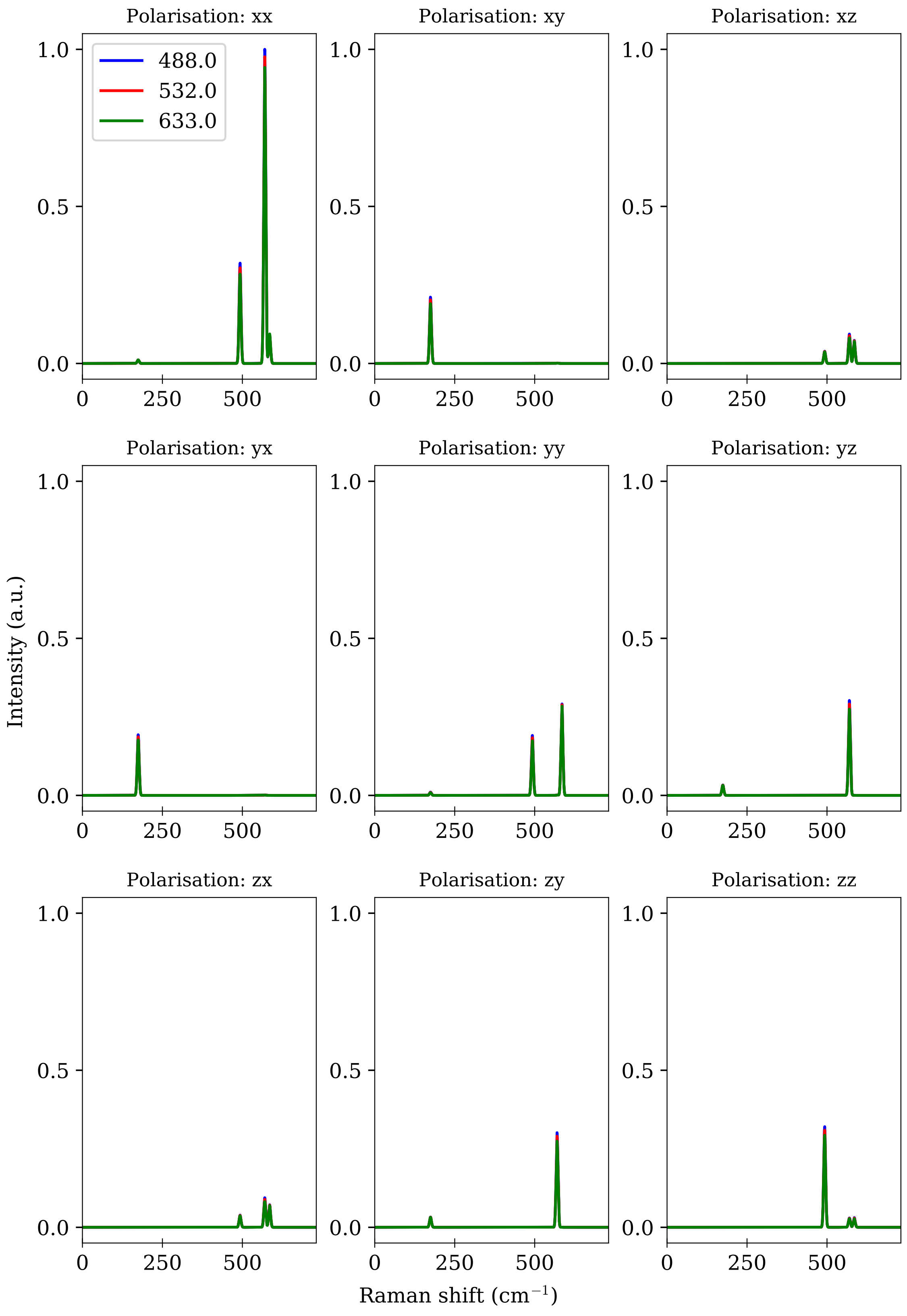

Supplementary Figure 723: Raman spectrum of ZrO_2 with space group 115 and Wyckoff positions a-g-0.

ZrO_2, 164, b-d-0

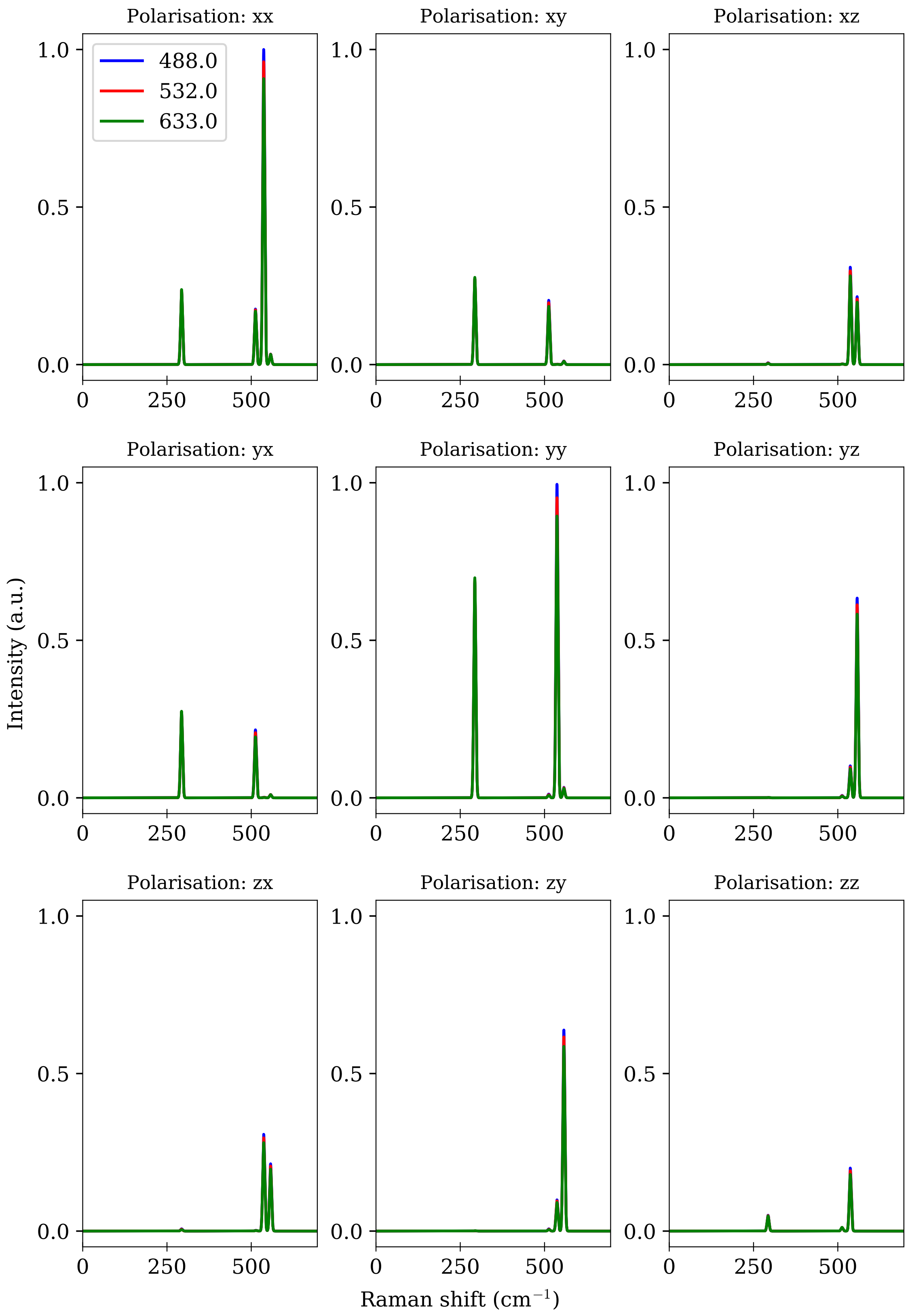

Supplementary Figure 724: Raman spectrum of ZrO_2 with space group 164 and Wyckoff positions b-d-0.

ZrS_2, 164, b-d-0

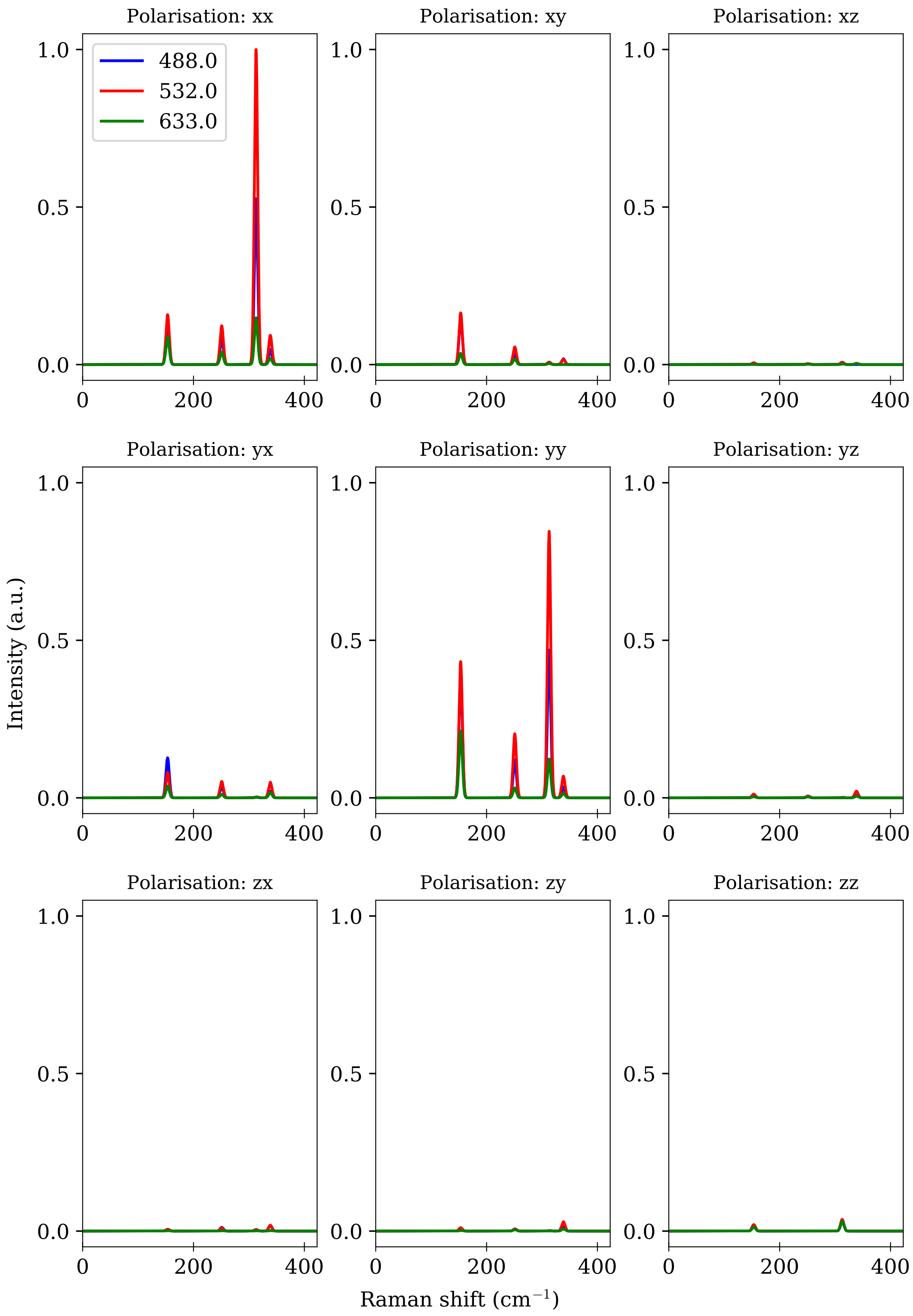

Supplementary Figure 725: Raman spectrum of ZrS_2 with space group 164 and Wyckoff positions b-d-0.

$ZrSb_2$, 164, b-d-0

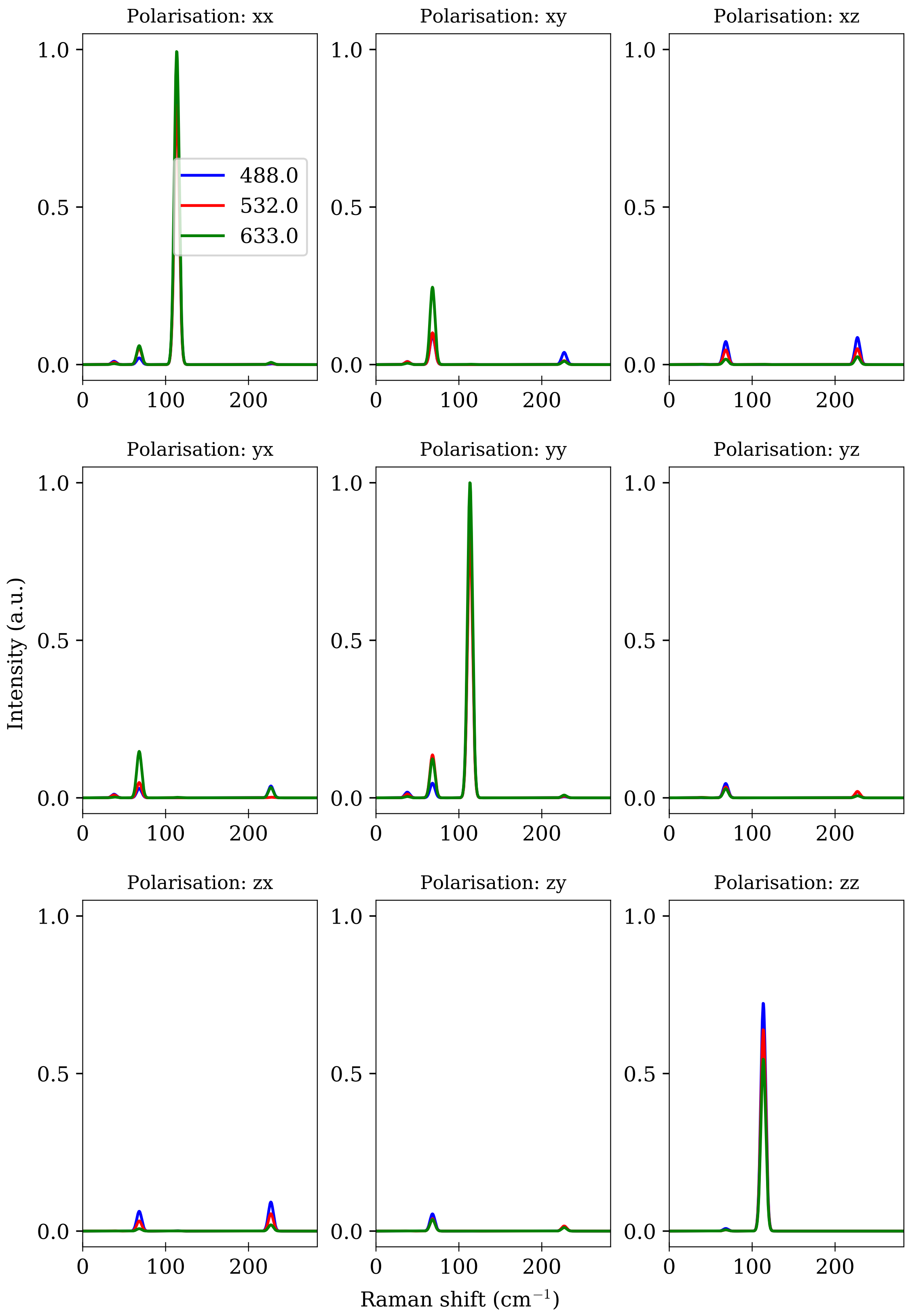

Supplementary Figure 726: Raman spectrum of $ZrSb_2$ with space group 164 and Wyckoff positions b-d-0.

$ZrSb_2$, 187, b-i-0

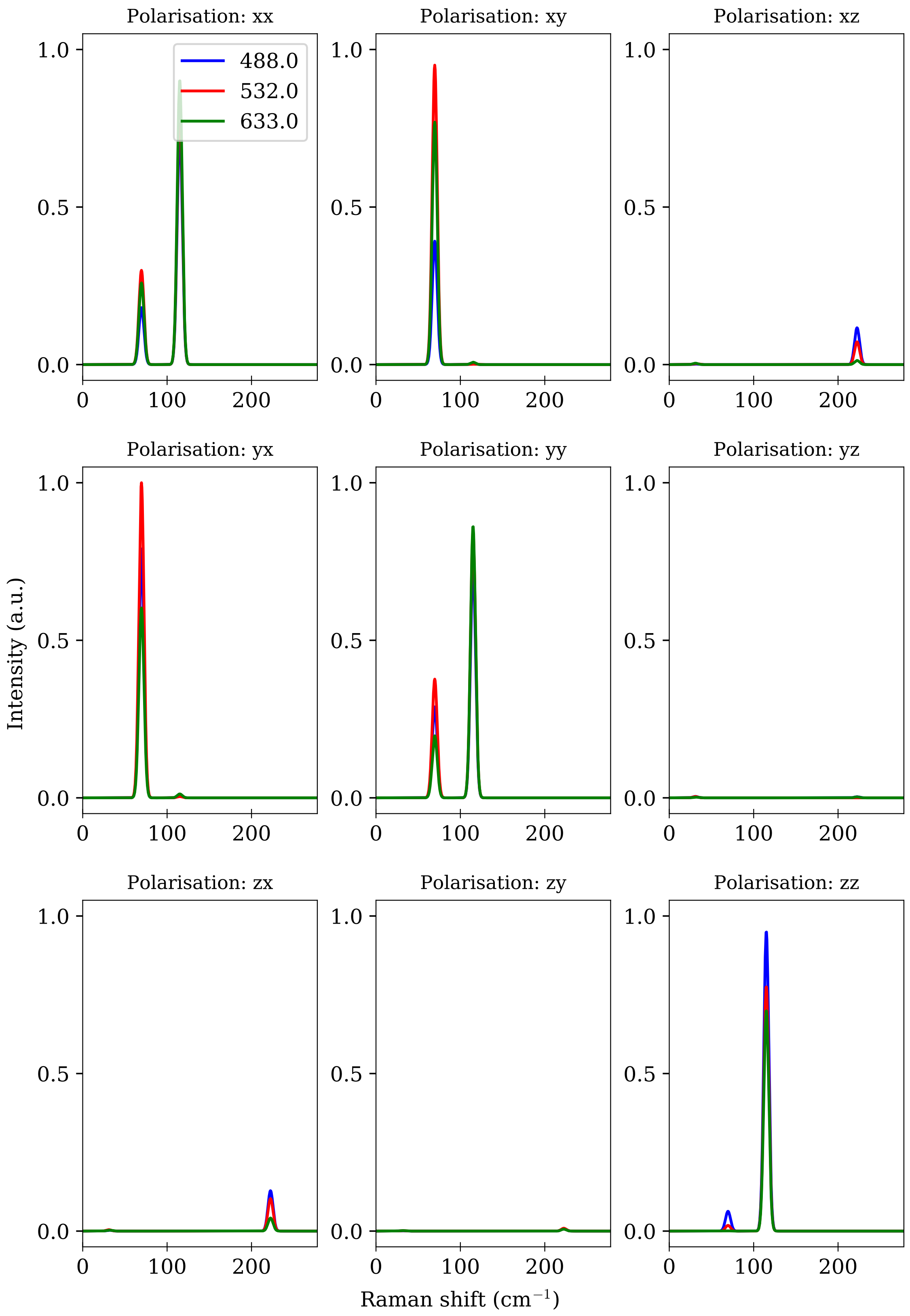

Supplementary Figure 727: Raman spectrum of $ZrSb_2$ with space group 187 and Wyckoff positions b-i-0.

$ZrSe_2$, 164, b-d-0

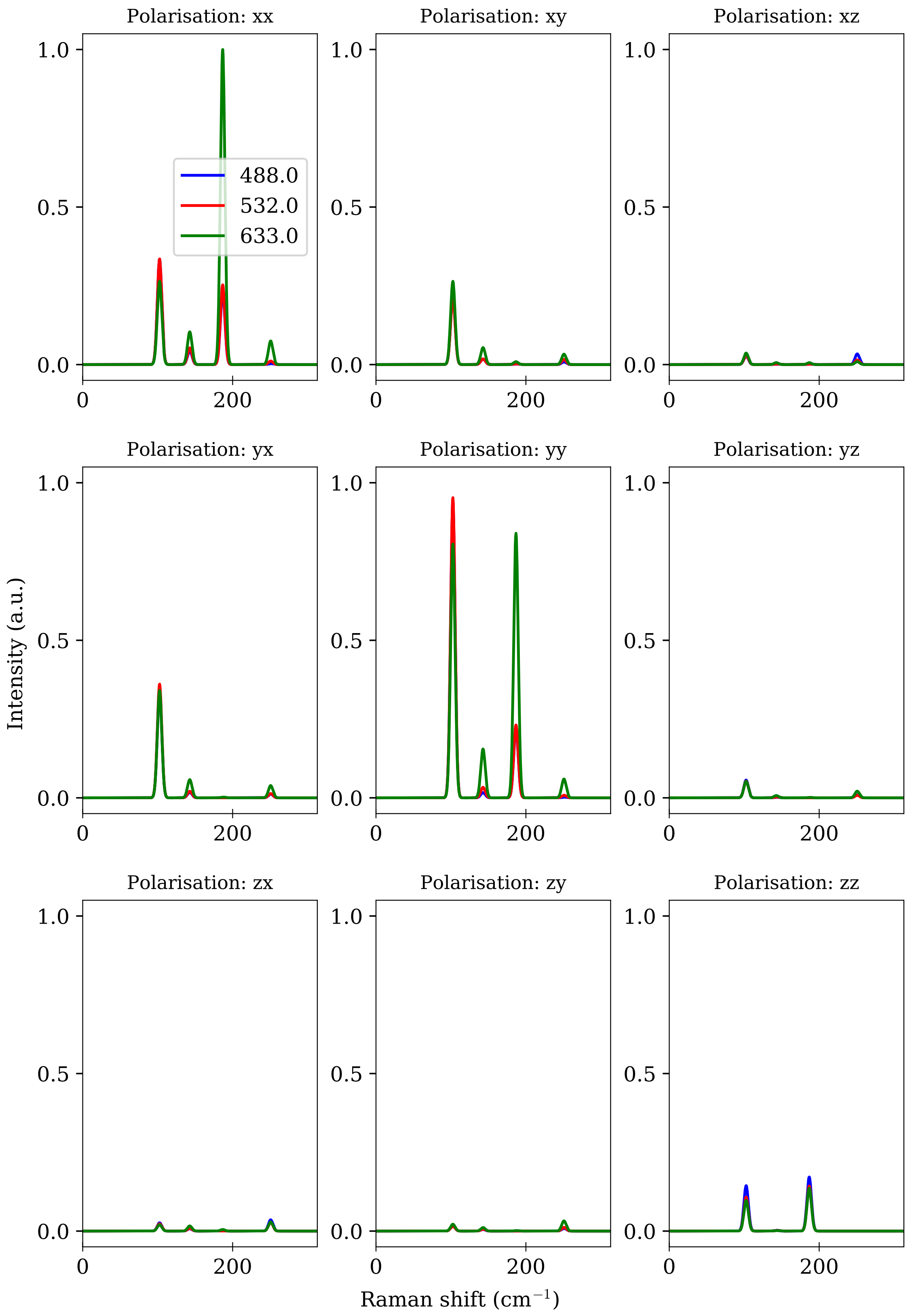

Supplementary Figure 728: Raman spectrum of $ZrSe_2$ with space group 164 and Wyckoff positions b-d-0.

ZrSeTe, 156, a-b-c-0

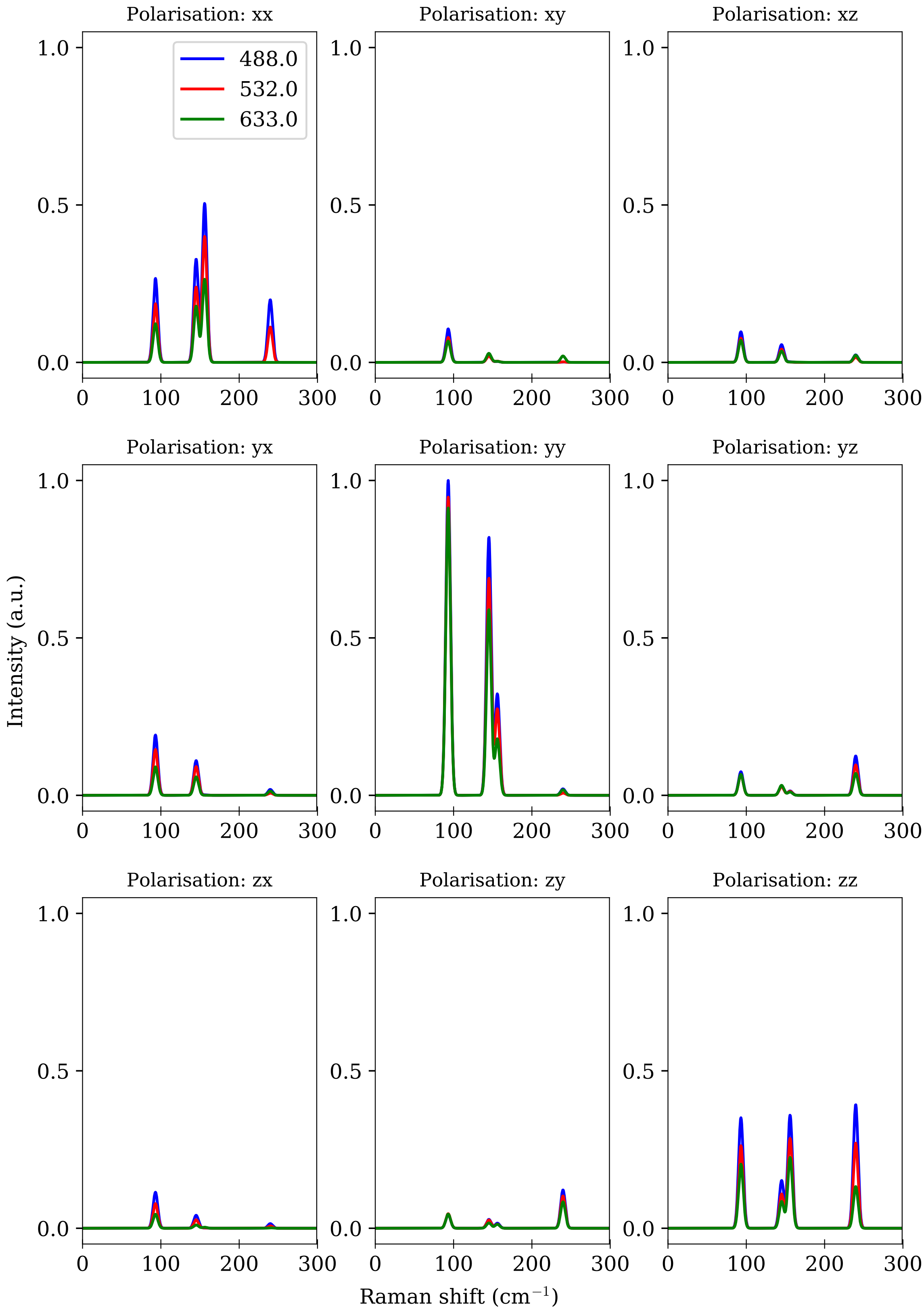

Supplementary Figure 729: Raman spectrum of ZrSeTe with space group 156 and Wyckoff positions a-b-c-0.

ZrSeTe, 156, a-c-0

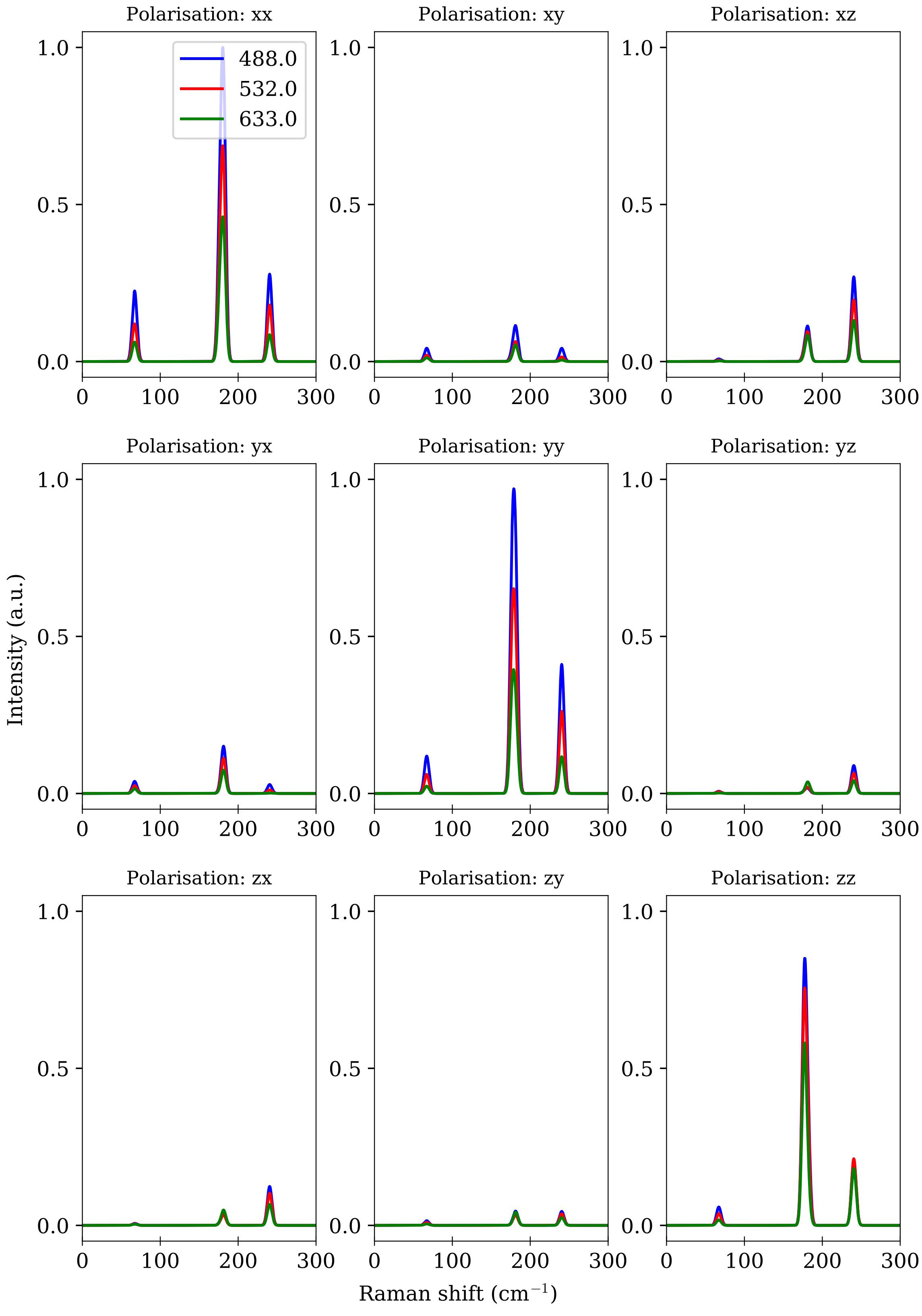

Supplementary Figure 730: Raman spectrum of ZrSeTe with space group 156 and Wyckoff positions a-c-0.

ZrSSe, 156, a-b-c-0

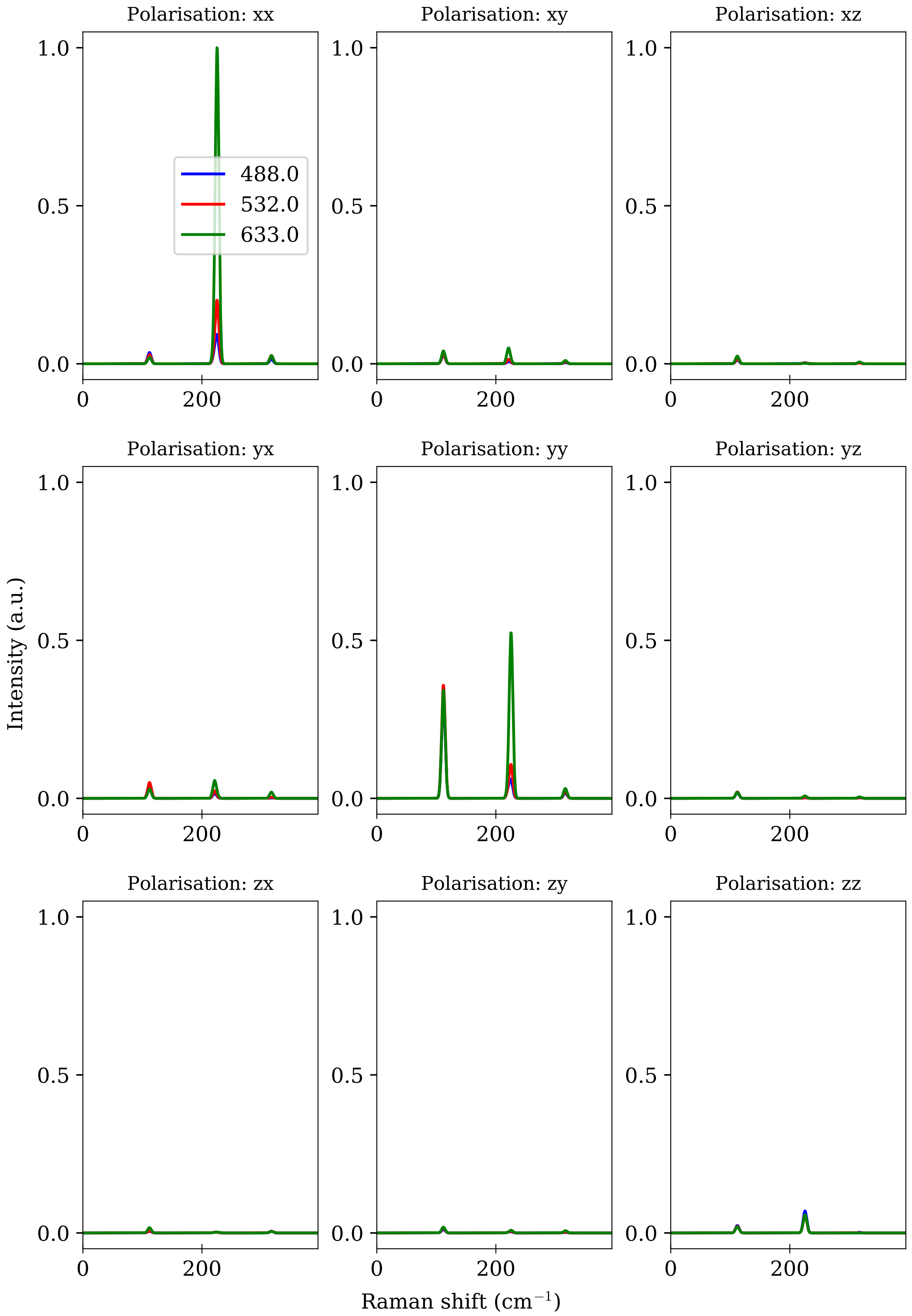

Supplementary Figure 731: Raman spectrum of ZrSSe with space group 156 and Wyckoff positions a-b-c-0.

ZrSTe, 156, a-b-c-0

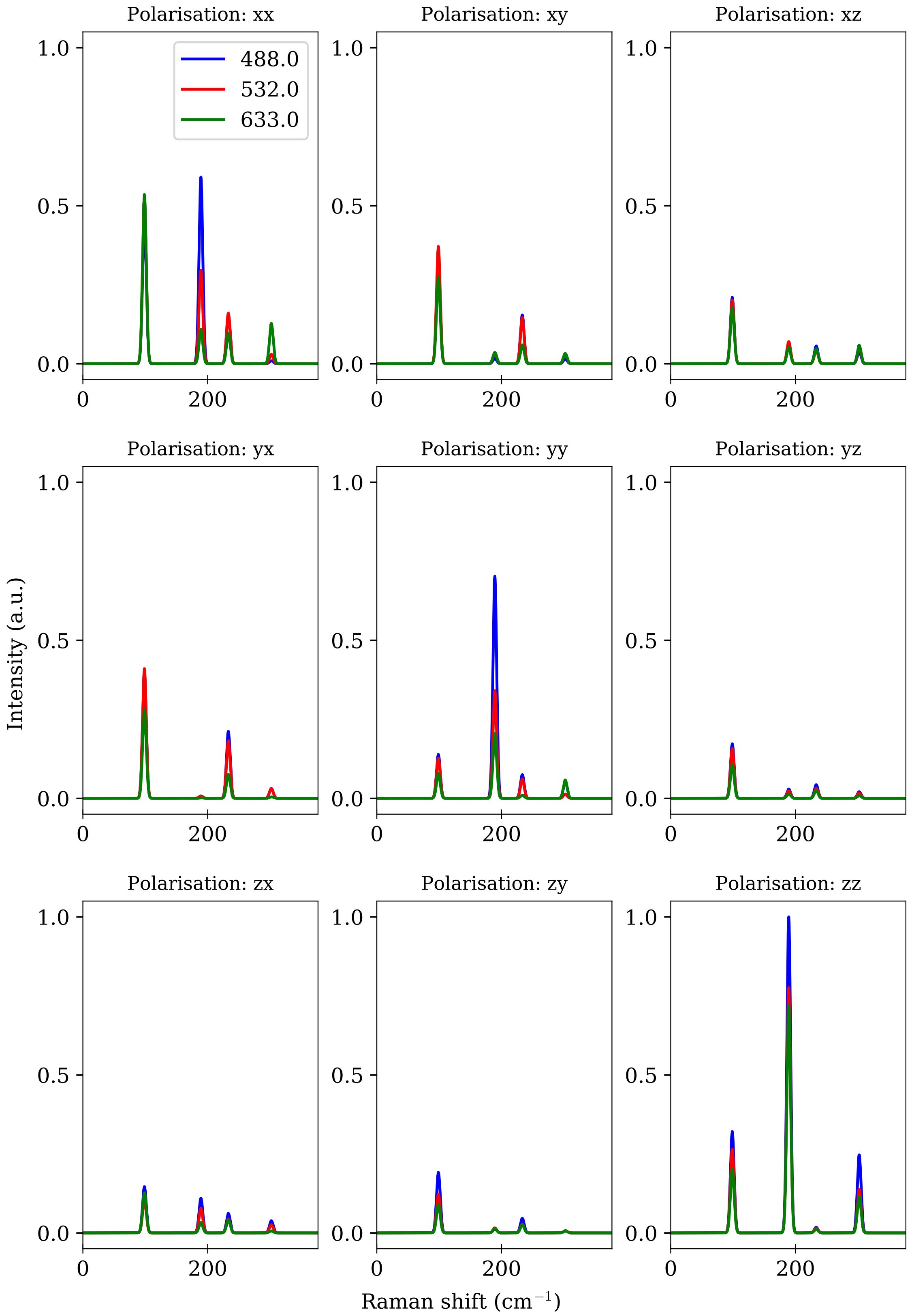

Supplementary Figure 732: Raman spectrum of ZrSTe with space group 156 and Wyckoff positions a-b-c-0.

ZrSTe, 156, a-c-0

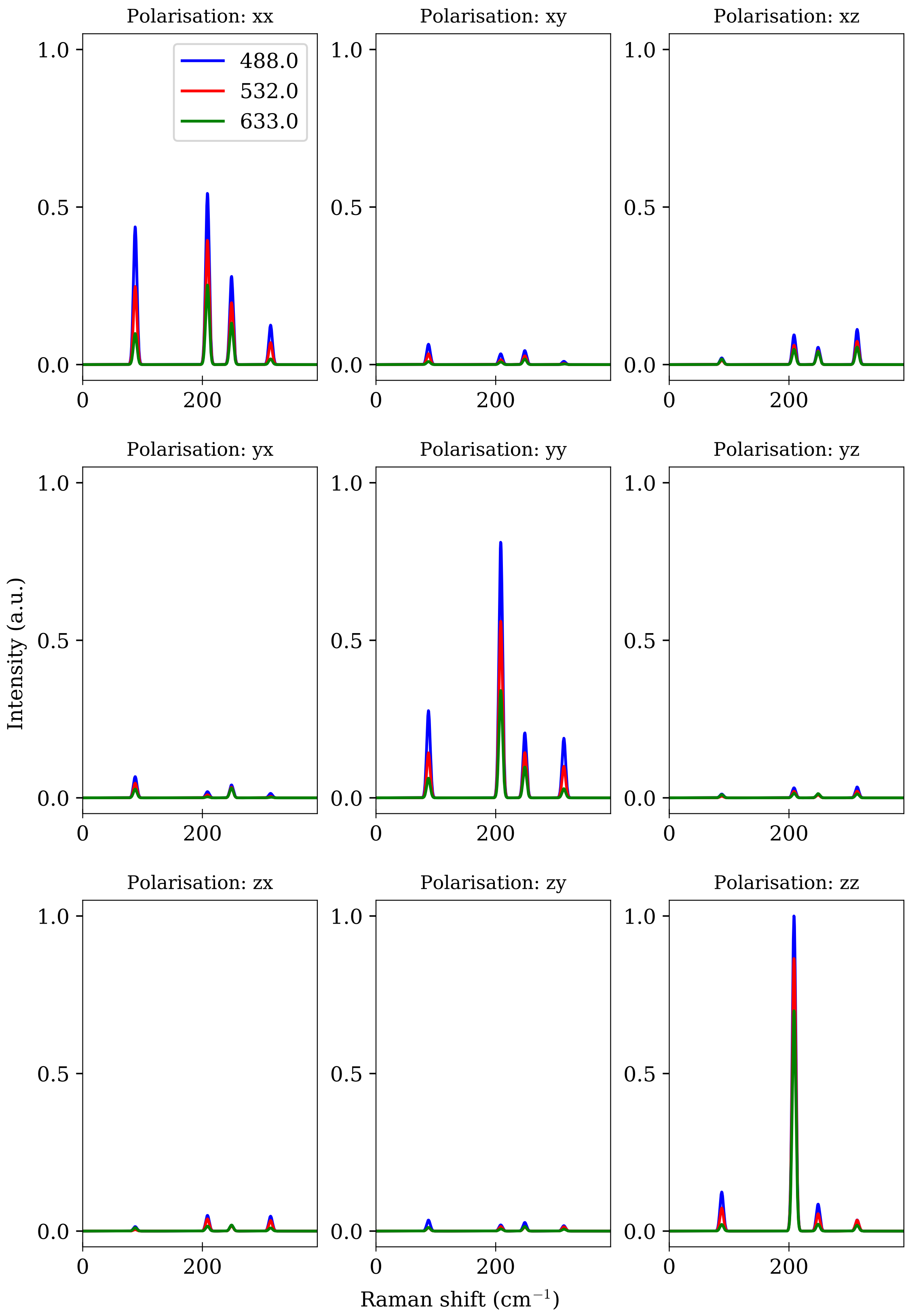

Supplementary Figure 733: Raman spectrum of ZrSTe with space group 156 and Wyckoff positions a-c-0.

$ZrTe_2$, 187, b-i-0

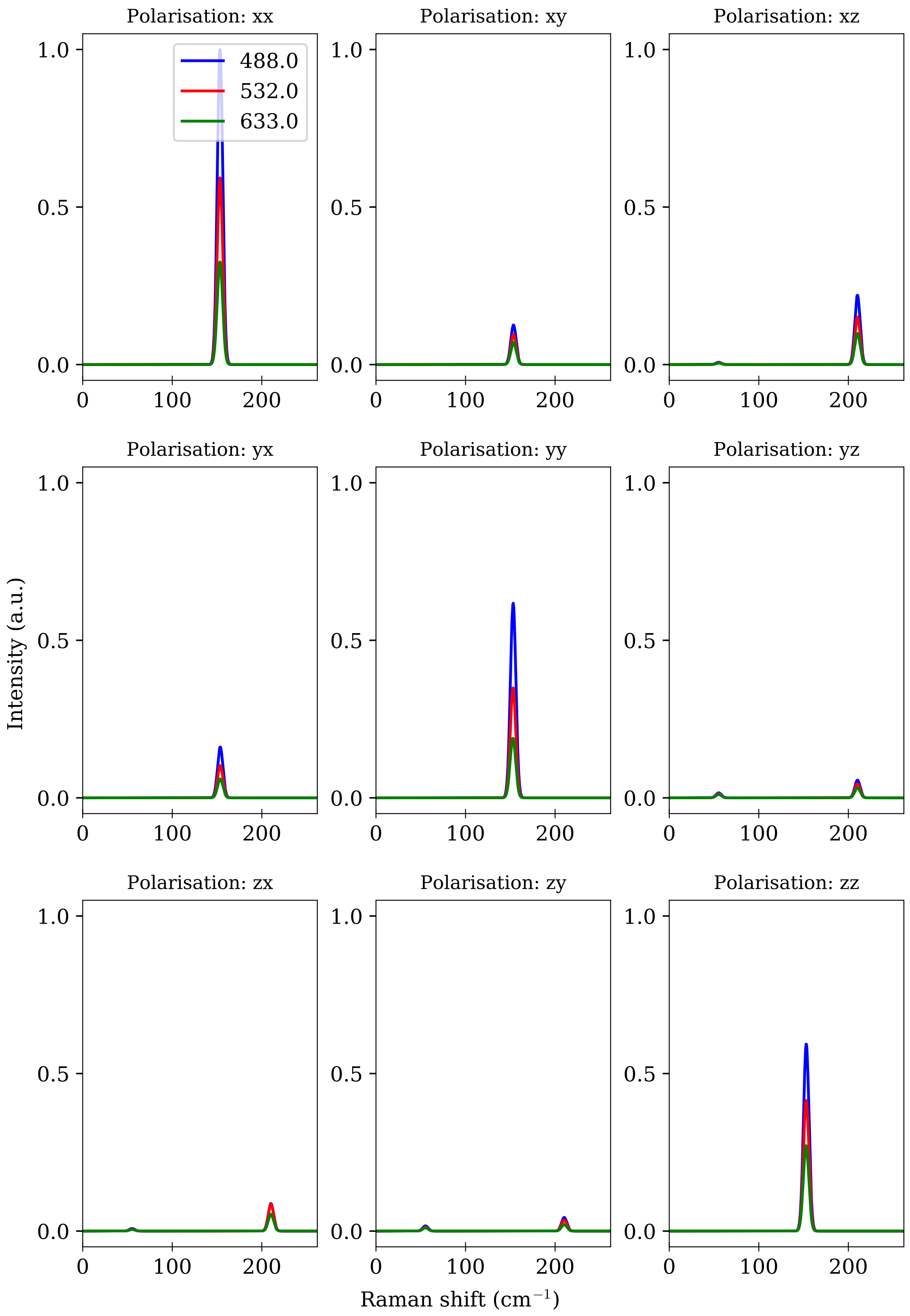

Supplementary Figure 734: Raman spectrum of $ZrTe_2$ with space group 187 and Wyckoff positions b-i-0.

Supplementary References

[1] R. Loudon, "The Raman effect in crystals", Adv. Phys. **13**, 423–482 (1964).

[2] J.-B. Wu, M.-L. Lin, X. Cong, H.-N. Liu, and P.-H. Tan, "Raman spectroscopy of graphene-based materials and its applications in related devices", Chem. Soc. Rev **47**, 1822–1873 (2018).

[3] Q. Cai, D. Scullion, A. Falin, K. Watanabe, T. Taniguchi, Y. Chen, E. J. G. Santos, and L. H. Li, "Raman signature and phonon dispersion of atomically thin boron nitride", Nanoscale **9**, 3059–3067 (2017).

[4] H.-L. Liu, T. Yang, Y. Tatsumi, Y. Zhang, B. Dong, H. Guo, Z. Zhang, Y. Kumamoto, M.-Y. Li, L.-J. Li, R. Saito, and S. Kawata, "Deep-ultraviolet Raman scattering spectroscopy of monolayer WS_2", Sci. Rep. **8**, 11398 (2018).

[5] F. Xia, H. Wang, and Y. Jia, "Rediscovering black phosphorus as an anisotropic layered material for optoelectronics and electronics", Nat. Commun. **5**, 4458 (2014).

[6] U. Gupta, B. S. Naidu, U. Maitra, A. Singh, S. N. Shirodkar, U. V. Waghmare, and C. N. R. Rao, "Characterization of few-layer 1T-$MoSe_2$ and its superior performance in the visible-light induced hydrogen evolution reaction", APL Mater. **2**, 092802 (2014).

[7] C. Ruppert, O. B. Aslan, and T. F. Heinz, "Optical properties and band gap of single- and few-layer $MoTe_2$ crystals", Nano Lett. **14**, 6231–6236 (2014).

[8] M. Yan, E. Wang, X. Zhou, G. Zhang, H. Zhang, K. Zhang, W. Yao, N. Lu, S. Yang, S. Wu, T. Yoshikawa, K. Miyamoto, T. Okuda, Y. Wu, P. Yu, W. Duan, and S. Zhou, "High quality atomically thin $PtSe_2$ films grown by molecular beam epitaxy", 2D Mater. **4**, 045015 (2017).

[9] Y. Zhao, J. Qiao, P. Yu, Z. Hu, Z. Lin, S. P. Lau, Z. Liu, W. Ji, and Y. Chai, "Extraordinarily strong interlayer interaction in 2D layered PtS_2", Adv. Mater. **28**, 2399–2407 (2016).

[10] M. Yang, X. Cheng, Y. Li, Y. Ren, M. Liu, and Z. Qi, "Anharmonicity of monolayer MoS_2, $MoSe_2$, and WSe_2: a Raman study under high pressure and elevated temperature", Appl. Phys. Lett. **110**, 093108 (2017).

[11] K. Chen, Z. Chen, X. Wan, Z. Zheng, F. Xie, W. Chen, X. Gui, H. Chen, W. Xie, and J. Xu, "A simple method for synthesis of high-quality millimeter-scale 1T′ transition-metal telluride and near-field nanooptical properties", Adv. Mater. **29**, 1700704 (2017).

[12] J. Yu, X. Kuang, Y. Gao, Y. Wang, K. Chen, Z. Ding, J. Liu, C. Cong, J. He, Z. Liu, and Y. Liu, "Direct observation of the linear dichroism transition in two-dimensional palladium diselenide", Nano Lett. **20**, 1172–1182 (2020).
